# Supplementary material for: The accuracy of Fiber-Optic Raman Spectroscopy in the detection and diagnosis of head and neck neoplasm in vivo: a systematic review and meta-analysis
Source: PeerJ. 2023 Dec 11;11:e16536. doi: 10.7717/peerj.16536 (PMC10720414; doi:10.7717/peerj.16536)
Supplement: Data S1 [file peerj-11-16536-s001.zip › article/10.1021@acs.analchem.9b00173.pdf]

analytical chemistry

Article

Cite This: *Anal. Chem.* 2019, 91, 8101−8108

pubs.acs.org/ac

Fiber-Optic Raman Spectroscopy with Nature-Inspired Genetic Algorithms Enhances Real-Time in Vivo Detection and Diagnosis of Nasopharyngeal Carcinoma

Petar Žuvela,[†,§] Kan Lin,[†,§] Chi Shu,[†,§] Wei Zheng,[†,§] Chwee Ming Lim,[‡] and Zhiwei Huang*[,†]

[†]Optical Bioimaging Laboratory, Department of Biomedical Engineering, Faculty of Engineering, National University of Singapore, 9 Engineering Drive 1, Singapore 117576

[‡]Department of Otolaryngology, Head and Neck Surgery, National University of Singapore and National University Health System, Singapore 119074

Supporting Information

ABSTRACT: Raman spectroscopy is an optical vibrational spectroscopic technique capable of probing specific biochemical structures and conformation of tissue and cells in biomedical systems. This work aims to assess the clinical utility of a fiber-optic Raman spectroscopy with nature-inspired genetic algorithms for enhancing in vivo detection and diagnosis of nasopharyngeal carcinoma (NPC) patients. The Raman diagnostic platform is developed based on simultaneous fingerprint (FP) and high-wavenumber (HW) fiber-optic Raman endoscopy associated with genetic algorithms-partial least-squares-linear discriminant analysis (GA-PLS-LDA). A total of 2126 in vivo FP/HW Raman spectra (598 NPC, 1528 normal) acquired from 113 tissue sites of 14 NPC patients and 48 healthy subjects during nasopharyngeal endoscopic examinations. Distinct Raman peaks have been identified (853 cm^{-1} - proteins, 1209 cm^{-1} - phenylalanine, 1265 cm^{-1} - proteins, 1335 cm^{-1} - proteins and nucleic acids, 1554 cm^{-1} - tryptophan, porphyrin, 2885 cm^{-1} - lipids, 2940 cm^{-1} - proteins, 3009 cm^{-1} - lipids, and 3250 cm^{-1} - water) that are related to the significant biochemical changes ($p < 1 \times 10^{-5}$) in NPC compared to normal tissue. Raman diagnostic performance is evaluated through the leave-one-object (tissue site)-out cross-validation (LOOCV) method. A statistically significant GA-PLS-LDA model ($p < 1 \times 10^{-5}$) on FP/HW Raman yields a CV diagnostic accuracy of 98.23% (111/113), sensitivity of 93.33% (28/30), and specificity of 100% (83/83) for NPC classification. This work demonstrates that the fiber-optic FP/HW Raman diagnostic platform developed has great promise for improving real-time in vivo detection and diagnosis of NPC at the molecular level during clinical nasopharyngeal endoscopy.

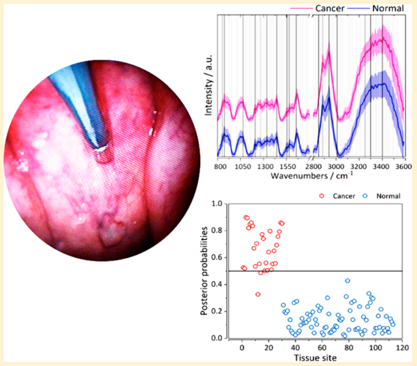

Nasopharyngeal carcinoma (NPC) is one of the most common malignancies in the head and neck in Southern China and Southeast Asia, including Singapore.[1,2] The incidence rates of NPC are particularly prevalent in China and East Asia as high as 21.4 and in Singapore as high as 31.5 per 100,000 persons per year.[2] Early detection and localization with immediate removal and treatment (e.g., radiochemotherapy) is crucial to improving survival rates of patients with NPC.[3] The current gold standard for early diagnosis of NPCs is based on H&E-stained histopathology of biopsies obtained during white-light reflectance (WLR)-guided endoscopy.[3] NPC biopsies obtained under WLR guidance greatly depend on visualization of morphological changes in nasopharyngeal tissue. Cancerous tissues are typically located in the silent anatomic regions, and poor detection rates arise from difficulty in discerning early NPC lesions from surrounding normal or benign tissue even for experienced clinicians. Consequently, more than 70% of NPC patients are diagnosed in the late stages, resulting in high mortalities.[4] Evidently, there is a clinically unmet need for developing innovative but objective diagnostic techniques for improving in vivo early NPC detection in the head and neck.

Raman spectroscopy is an inelastic light scattering technique with vibrational specificity to the Raman-active modes of molecules in the samples.[5] Hence, it has the ability to reveal biochemical and biomolecular structures of tissue and cells, providing an unique opportunity to distinguish among different pathologic tissue types at the molecular level.[6−25] In the fingerprint (FP) range (i.e., 800−1800 cm^{-1}), Raman spectroscopy has advantages of identifying specific diagnostic information about backbone structures of proteins, lipids, and nucleic acid assemblies in cells and tissue.[6,7,18−25] However, FP Raman can be compromised due to weak tissue Raman signals with overwhelming tissue autofluorescence (AF) background interference.[11,26] High-wavenumber (HW) (i.e., 2800−3600 cm^{-1}) Raman containing biochemical information (e.g., CH_2-/CH_3-stretching of lipids/proteins, and OH band) exhibits a relatively stronger tissue Raman signal with less AF interference.[14,15] The combined FP and HW Raman with

Received: January 10, 2019
Accepted: May 28, 2019
Published: May 28, 2019

ACS Publications © 2019 American Chemical Society

DOI: 10.1021/acs.analchem.9b00173
Anal. Chem. 2019, 91, 8101−8108

Downloaded via KEAN UNIV on July 24, 2019 at 07:21:10 (UTC).
See https://pubs.acs.org/sharingguidelines for options on how to legitimately share published articles.

complementary biochemical/biomolecular information thus has advantages for improving biomedical tissue characterization and diagnosis.[14,15,17] Preliminary ex vivo Raman studies on nasopharyngeal tissue samples have shown encouraging NPC diagnostic accuracies (85−95%).[7,25] With the development of a rapid fiber-optic Raman spectroscopy system capable of simultaneously acquiring both FP and HW tissue Raman spectra,[16,17] in vivo Raman diagnosis of NPC becomes possible at endoscopy. To date, in vivo Raman studies detailing FP/HW Raman spectroscopy for NPC diagnosis are still very limited,[11,22−24] and most Raman diagnostic algorithm development overlooked feature selections widely recognized as a de facto crucial step in regression/classification.[27] Spectral feature selection would not only improve diagnostic model performance but also provide valuable insights into tissue biochemical changes at the molecular level. Nature-inspired techniques (e.g., genetic algorithms (GA)[28,29]) have been shown to be effective for addressing the above purpose.[30−35] In Raman-based cancer diagnostics, GA has been used on a few occasions for feature selection. For instance, Duraipandian et al.[31] used GA to select informative Raman spectral bands for cervical cancer diagnosis. They identified seven diagnostically significant ($p < 1 \times 10^{-3}$) Raman bands related to proteins, nucleic acids, and lipids in tissue. The developed model yielded a diagnostic accuracy of 82.9%, sensitivity of 72.5%, and specificity of 89.2% (AUC 0.853). Li et al.[32] applied GA for selection of (surface-enhanced) Raman spectra for bladder cancer diagnosis from serum. Six diagnostically significant Raman bands were identified related to proteins, nucleic acids, and lipids. Sensitivity of 90.9% and perfect specificity were achieved. Song et al.[33] also used GA for selecting informative Raman bands to diagnose cancer in Barrett's esophagus and developed a diagnostic model with 86% sensitivity, 88% specificity, and 89% accuracy.

In this work, we report for the first time (to the best of our knowledge) the application of GA for spectral feature selection in the development of fiber-optic Raman diagnostic algorithms for NPC endoscopic diagnosis. The diagnostic platform is developed based on the simultaneous FP/HW Raman endoscopy and nature-inspired genetic algorithms coupled with probabilistic partial least-squares-linear discriminant analysis (GA-PLS-LDA).[31,34] The Raman diagnostic platform is utilized for FP/HW Raman spectral feature selection as well as robust discrimination between normal and NPC tissue. We compare the diagnostic performance of FP, HW, and the combined FP/HW Raman techniques with GA-PLS-LDA for NPC diagnosis. Besides cross-validation, resampling with replacement[35] is also used to evaluate the robustness (through confidence intervals of performance metrics) of the developed diagnostic algorithms. The receiver operating characteristic (ROC) curves[36] are also generated to assess the clinical utility of the combined FP/HW Raman diagnostic platform for improving in vivo diagnosis of NPC at endoscopy.

■ MATERIALS AND METHODS

Clinical Instrumentation. The innovative fiber-optic Raman spectroscopic system developed for in vivo tissue Raman measurements at endoscopy has been described elsewhere.[11,16] Briefly, the nasopharyngeal tissue was excited using a 785 nm near-infrared (NIR) diode laser (maximum output: 300 mW, B&W TEK). The radiation power on the tissue site was ∼1.5 W/cm^2 which is within the maximum exposure limits set by the American National Standards Institute (ANSI).[37] A specially designed and fabricated 1.9 m long fiber-optic Raman probe (∼1.8 mm in outer diameter)[8] is used for both laser excitation light delivery and in vivo epithelial tissue Raman signal collection at endoscopy. The compact fiber-optic Raman endoscopic probe comprises 18 × 200 μm beveled collection fibers (low-OH fused silica, numerical aperture (NA) = 0.22) surrounding the central light delivery fiber (low-OH fused silica, 200 μm in diameter, NA = 0.22). A 1.0 mm sapphire ball lens is coupled to the fiber tip of the probe to tightly focus the excitation light onto the tissue subsurface, enabling the effective Raman spectrum collection from the epithelial lining.[11,17] The depth-selective capability of the fiber-optic Raman spectroscopy technique ensures the shallower tissue interrogation (<200 μm) with tissue probing volume of <0.02 mm^3, thereby reducing the interferences and signal dilution from deeper stromal tissue while selectively interrogating the epithelium associated with neoplastic onset and progression.[8] Raman spectral information in 800−3600 cm^{-1} (∼5 cm^{-1} resolution) was acquired using a high-throughput reflective imaging spectrograph (Acton LS-785 f/2, Princeton Instruments) equipped with a customized gold-coated 830 gr/mm grating and an NIR-optimized charge-coupled device (CCD) camera (PIXIS: 400BR-eXcelon, Princeton Instruments). Raman spectra were calibrated using a mercury−argon lamp (HG-1, AR-1, Ocean Optics) and 4-acetamidophenol (ASTM E1840) for FP and HW regions, respectively.[13,14] All wavelength-calibrated spectra are corrected for the wavelength dependence of the system, using a tungsten calibration lamp (RS-10, EG&G Gamma Scientific, San Diego, CA). Measurement variations caused by intrinsic organ motions were minimized by acquiring spectra in ∼15−20 replicates within 0.5 s. FP/HW Raman spectral information was fed into a nature-inspired endoscopic diagnostic platform to provide real-time Raman spectral acquisition, processing, and diagnostic assessment of nasopharyngeal tissue during endoscopic examination.

Clinical Trial Protocol. Ethical protocol of this study was approved by the Institutional Review Board (IRB) of the National Healthcare Group (NHG) of Singapore (NHG DSRB ref No. 2014/00323). Patients with new suspected diagnosis of cancer in the nasopharynx without having dementia, cognitive impairment, or pregnancy were eligible for this study. All the patients involved in the clinical Raman measurements have signed consent forms allowing for in vivo Raman spectroscopic measurements during nasopharyngeal endoscopy. Endoscopic procedures were performed in the head and neck clinic at the National University Health System (NUHS), Singapore. High-quality in vivo FP/HW Raman spectra were acquired from nasopharyngeal tissue with the fiber-optic Raman probe being passed down to the instrument channel of a medical rhinolaryngoscope under WLR imaging guidance. The tip of the probe was visible ∼2−5 mm in front of the endoscope camera and placed in mild contact with the surface of the nasopharyngeal mucosa during Raman measurements. Position of the Raman probe against the various tissue sites was determined by the clinician guided by the images shown on the computer monitor while performing the endoscopies. Large areas of NPC tissue were scanned because the system can record multiple Raman spectra (∼15−20) within 0.5 s for tissue site. Out of 62 recruited subjects, 48 subjects were healthy volunteers, while 14 were patients with NPC. Nineteen (6 NPC, 13 normal) were women, and 43

DOI: 10.1021/acs.analchem.9b00173
Anal. Chem. 2019, 91, 8101−8108

were men (8 NPC, 35 normal) with mean ages of 46.4 ± 11.3 and 53.8 ± 16.9, respectively.

Immediately after performing the Raman measurements, biopsies were taken from the suspicious lesion sites measured, then fixed in formalin, sectioned, and stained with H&E for histopathology examinations by the three pathologists who were blinded to the Raman-based diagnosis. To avoid unnecessary invasive biopsies in full compliance with the approved ethical protocol, healthy volunteers were assumed as normal based on the observation of well-trained otorhinolaryngologists. Agreement among the opinions of the three independent pathologists was evaluated using Cohen's Kappa statistics ($\kappa > 0.9$). Diagnostic performance of the developed nature-inspired FP/HW Raman platform was examined based on histopathological assessments for which all the examiners had reached a consensus.

Spectral Processing and NPC Diagnostic Platform. In vivo FP/HW Raman spectra in the 800–3600 cm^{-1} range were acquired from nasopharyngeal tissue during endoscopy. Raw tissue spectra consisted of Raman, AF background, and noise. First, the noise was reduced through smoothing with a third-order Savitzsky-Golay filter (5-pixel window width corresponding to resolution of ~5 cm^{-1}). Subsequently, iterative polynomial fitting was used to separate the Raman signal from the AF background.[18,26] Polynomials of the fifth- and first-order were found to be optimal for AF removal in the FP (800–1800 cm^{-1}) and HW (2800–3600 cm^{-1}), respectively. Spectra were further normalized to the area under the FP and HW regions for spectra analysis. Significance of these characteristic spectral differences was evaluated using an unpaired two-sided Student's *t*-test at the significance level of 1×10^{-5}. The so-called silent region (1800–2800 cm^{-1}) with no distinct Raman peaks was excluded from analysis. Such processed spectra were fed to the developed diagnostic platform for tissue classification. The GA-PLS-DA platform developed is allowed for automatic spectral feature selection and robust discrimination between normal and NPC tissue. Selections of wavenumbers within one GA unit are performed in *n* equal and one unequal spectral window (Supporting Information in Figure S1 for details). The optimal window sizes are the average resolutions in FP, HW, and FP/HW ranges. Prior to feature selection, optimal number of PLS latent variables (LVs) was determined using leave-one-object (tissue site)-out cross validation (LOOCV). LOOCV was employed for diagnostic performance evaluation. CV diagnostic accuracy was maximized, while final diagnostic performance was obtained through majority voting for each tissue site.[11] All the models were statistically validated using cross-validation analysis of variance (CV-ANOVA).[38] To evaluate the robustness of the developed NPC diagnostic algorithms, resampling with replacement[35] (with 99%/1% training/testing separation equivalent to leave-one-tissue site-out CV) was used. Confidence intervals of all the performance metrics (accuracy, sensitivity, specificity, AUC) were also calculated.

For direct and unbiased comparison between FP, HW, and FP/HW PLS-LDA and GA-PLS-LDA models, we employed ROC curves and the sum of ranking differences (SRD) validated by comparison to normally distributed random numbers.[39] All the data analysis were performed in MATLAB (MathWorks, Natick, MA, US) using in-house written routines integrated into the fiber-optic Raman endoscope system.[11,13]

■ RESULTS

During endoscopic examinations, a total of 2126 high-quality in vivo FP/HW Raman spectra (598 carcinoma, 1528 normal) were acquired from 113 tissue sites (30 carcinoma and 83 normal) of 62 subjects as shown in Figure 1A (the corresponding raw spectra were depicted in Figure S2 in the

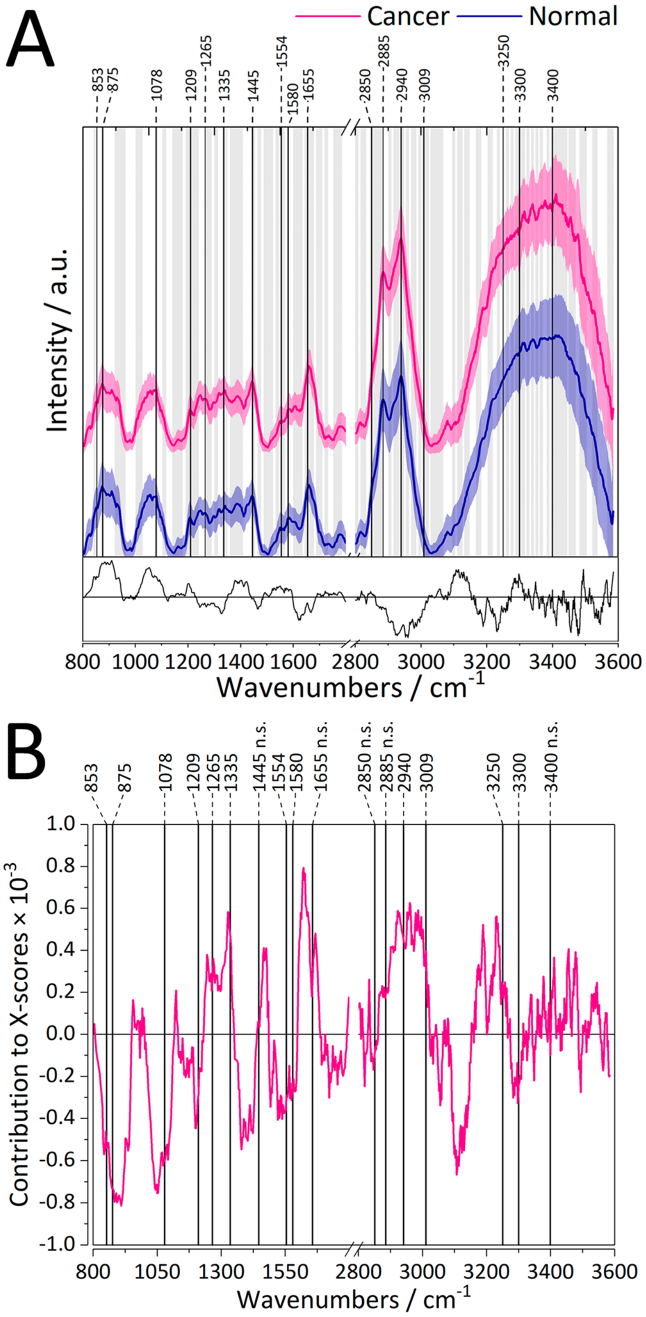

Figure 1. A) In vivo FP/HW spectra acquired from 113 tissue sites (normal = 83; NPC = 30) of 63 patients at clinical endoscopy with the selected wavenumbers using GA-PLS-LDA and cancer-normal difference spectra. Pink and blue lines/areas represent mean spectra/±1 standard deviation acquired for cancer and normal tissue, respectively. B) Contributions of differences between normal and cancer spectra to X-scores of the six extracted LVs for the PLS-LDA Raman model are also indicated. Tentatively assigned Raman peaks are denoted with black vertical lines, while nonsignificantly different (at confidence level of 1×10^{-5}) wavenumbers are denoted with n.s.

DOI: 10.1021/acs.analchem.9b00173
Anal. Chem. 2019, 91, 8101–8108

Table 1. Selected Wavenumbers by GA with Tentative Assignments of FP/HW Raman Peaks in Nasopharyngeal Tissue[a]

wavenumber/cm^{-1}	vibrational mode	biochemical components	p value	selected by GA
853	ν(C−C) stretching	proteins	5.94×10^{-23}*	yes
875		hydroxyproline	1.97×10^{-41}*	no
1078		lipids	4.25×10^{-28}*	no
1209		phenylalanine	1.52×10^{-8}*	yes
1265	amide III ν(C−N), δ(N−H)	proteins	2.55×10^{-6}*	yes
1335	$\gamma(CH_3{-}CH_2)$ twisting	proteins, nucleic acids	1.52×10^{-8}*	yes
1445	$\delta(CH_2)$ deformation	proteins, lipids	0.4788 n.s.	no
1554	scissoring NH_2	tryptophan, porphyrin	1.42×10^{-7}*	yes
1580	δ(C═C)	phenylalanine	1.228×10^{-6}*	no
1655	amide I ν(C═O)	proteins	0.0012 n.s.	no
2850	$\nu(CH_2)$ symmetric stretching	lipids	0.1183 n.s.	yes
2885	CH_2 stretching	lipids	0.0004 n.s.	yes
2940	$\nu(CH_3)$ stretching	proteins and lipids	3.08×10^{-17}*	yes
3009	ν(═C−H) asymmetric stretching	lipids	1.12×10^{-10}*	yes
~3300	amide A ν(C−N)	proteins	1.34×10^{-5}*	no
~3250	ν(OH) stretching	water	1.81×10^{-5}*	yes
~3400			0.074 n.s.	yes

[a]Probability (p) values obtained with unpaired two-sided Student's t-test denote the significant (*)/nonsignificant (n.s.) differences between NPC and normal tissue (significance level 1×10^{-5}). ν − stretching vibrational mode, δ − bending/deformation vibrational mode, γ − twisting/wagging vibrational mode. All other abbreviations are explained in the main text.

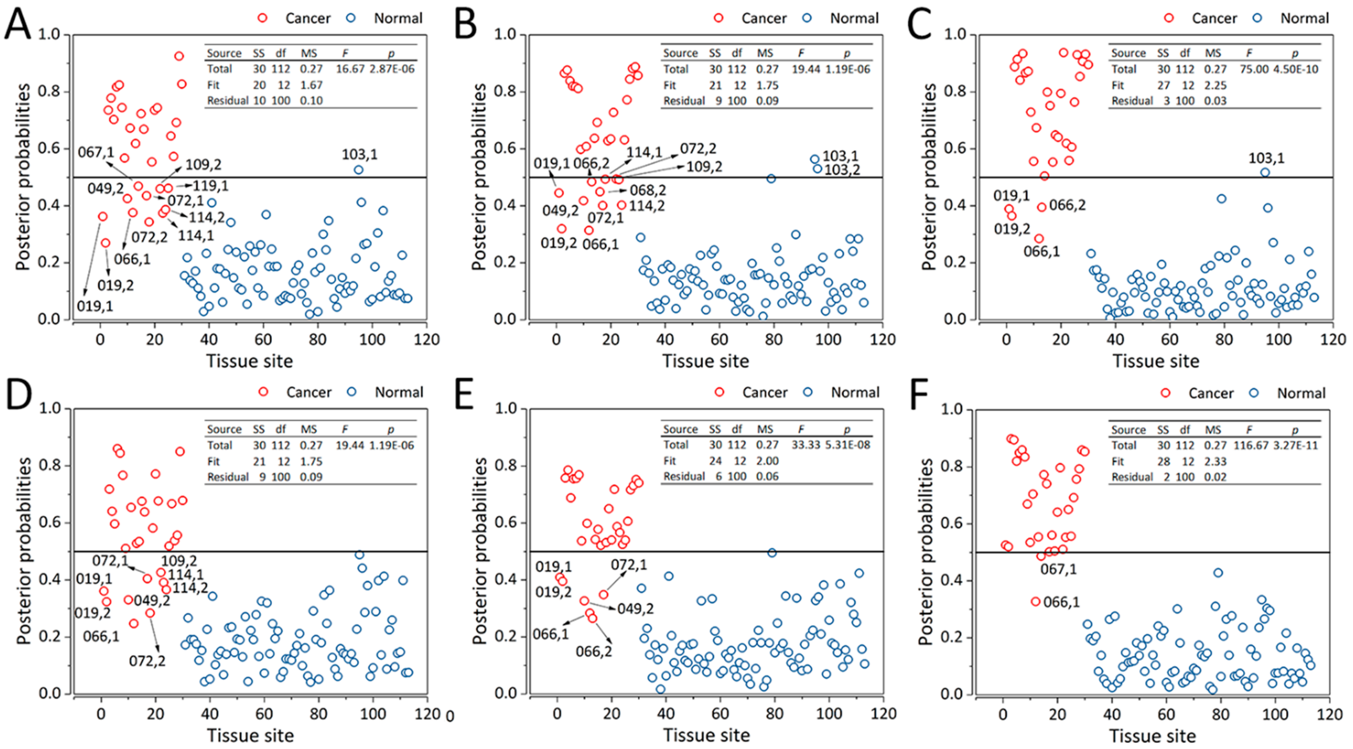

Figure 2. Posterior probabilities of PLS-LDA models for Raman in A) FP, B) HW, and C) FP/HW regions, as well as GA-PLS-LDA (best run out of three) for Raman in D) FP, E) HW, and F) FP/HW regions. CV-ANOVA tables are displayed in the insets. Abbreviations: SS − sum of squares, df − degrees of freedom, MS − mean squares, F − Fisher's F-statistic, p − probability value.

Supporting Information). Table 1 lists the tentative Raman peak assignments of nasopharyngeal tissue. Specifically, in the FP range, ν(C−C) stretching of proteins (853 cm^{-1}), hydroxyproline (875 cm^{-1}), lipids (1078 cm^{-1}), tryptophan and phenylalanine (1209 cm^{-1}), amide III ν(C−N) and δ(N−H) Raman bands of proteins (1265 cm^{-1}), CH_3CH_2 twisting of proteins and nucleic acids (1335 cm^{-1}), CH_2 deformation of proteins and lipids (1445 cm^{-1}), scissoring NH_2 vibration of tryptophan and porphyrin (1554 cm^{-1}), δ(C═C) of phenylalanine (1580 cm^{-1}), and amide I ν(C═O) (1655 cm^{-1}) of proteins are observed in nasopharyngeal tissue.[6−8,13,17,18,21,22,25] All the Raman peaks except 1445 and 1655 cm^{-1} exhibit significant differences between NPC and normal tissue ($p < 1 \times 10^{-5}$, unpaired two-sided Student's t-test). In the HW range, asymmetric CH_2 stretching of lipids at 2850 cm^{-1}, CH_2 stretching of lipids at 2885 cm^{-1}, CH_3 stretching of proteins at 2940 cm^{-1}, asymmetric ═CH stretching of lipids at 3009 cm^{-1}, and amide A−NH stretching

DOI: 10.1021/acs.analchem.9b00173
Anal. Chem. 2019, 91, 8101−8108

of proteins at 3300 cm^{-1}, as well as OH stretching vibrations at around 3250 and 3400 cm^{-1} corresponding to the broad Raman bands of water, are also observed in nasopharyngeal tissue.[9,11,14−17,19,24] We note that the optimal number of LVs is six for the PLS-LDA model determined using LOOCV. The six LVs explained >80% of Raman spectral variance in **X**- and **Y**-spaces. Scores of all six LVs exhibited significant differences ($p < 1 \times 10^{-5}$) between normal and cancerous tissue. Contribution plots of PLS-LDA were also constructed for averages of **X**-scores to identify which wavenumbers are most responsible for those differences (depicted in Figure 1B). The magnitude of the calculated contributions mostly concurred with the significantly different ($p < 1 \times 10^{-5}$) Raman peaks, confirming the superior ability of the extracted LVs to discern between normal and NPC tissue. Therefore, the scores of all the significant LVs extracted were fed to the LDA model for probabilistic diagnosis.

NPC diagnostic accuracy of PLS-LDA without GA-based feature selection for the combined FP/HW Raman spectra was 95.58% (108/113) with a sensitivity of 86.67% (26/30) and specificity of 98.80% (82/83). The combined FP/HW modality considerably outperforms the FP (accuracy of 89.38% (101/113), sensitivity of 63.33% (19/30), and specificity of 98.80% (82/83)) and HW (accuracy of 88.50% (100/113), sensitivity of 63.33% (19/30), and specificity of 97.59% (81/83)) methods. Robustness of the diagnostic PLS-LDA model based on FP/HW Raman spectra was evaluated through resampling with replacement. One hundred resampling iterations with 99%/1% training/testing separation equivalent to leave-one-tissue site-out have yielded accuracy, sensitivity, specificity, and AUC values of 94.43 (+2.04; −1.50)%, 82.93 (+3.73; −6.27)%, 98.57 (+1.42; −2.19)%, and 0.996 (+0.003; −0.032), respectively (Figure S3). To gain a deep understanding into the distribution of correctly classified and misclassified tissue sites, posterior probability plots were also constructed as displayed in Figure 2A−C. Diagnostic performance of the nature-inspired diagnostic platform was evaluated in an unbiased manner in three GA replicates. Table 2 outlines the diagnostic results of the best GA-PLS-LDA run among FP, HW, and the combined FP/HW techniques. For FP Raman, the average diagnostic performance of all the GA-PLS-LDA models has an average diagnostic accuracy of 91.15%, sensitivity of 67.78%, and specificity of 99.60%. For HW Raman, the average accuracy of 93.87%, sensitivity of 78.89%, and specificity of 99.60% are obtained. The combined FP/HW with the GA-PLS-LDA model yields an average accuracy of 96.76%, sensitivity of 87.78%, and specificity of 100.00%, which further affirmed the superior diagnostic performance compared to FP and HW Raman alone. Similar to the PLS-LDA model, the GA-PLS-LDA model robustness was tested through 100 resampling iterations (with 99%/1% training/testing separation equivalent to leave-one-tissue site-out) with replacement. As such, they have yielded accuracy, sensitivity, specificity, and AUC values of 97.44 (+1.67; −3.64)%, 90.40 (+13.73; −6.27)%, 99.99 (+1.19; −0.01), and 0.999 (+0.001; −0.024), respectively (Figure S4). For both PLS-LDA and GA-PLS-LDA models, the confidence intervals of the performance metrics are narrow. Therefore, the developed diagnostic algorithms can be considered robust.

For GA-PLS-LDA models, Figure 2 depicts CV-ANOVA tables, showing the statistical significance of the developed diagnostic models, and the distribution of tissue site misclassifications was also analyzed using posterior probability plots (Figure 2D−F). The diagnostic performance is also evaluated using ROCs and the respective AUCs. Figure 3A shows the ROC curves for PLS-LDA models, whereas Figure 3B depicts the ROC curves for GA-PLS-LDA models.

Table 2. Comparison of the Diagnostic Performance of the Three Raman Modalities (FP, HW, and FP/HW) Associated with GA-PLS-LDA Models (Best Run out of Three)[a]

Raman modality	acc/%	sens/%	spec/%	selected spectra	nLVs
FP	92.04 (104/113)	70.00 (21/30)	100.00 (83/83)	161/309	6
HW	94.69 (107/113)	80.00 (24/30)	100.00 (83/83)	218/470	6
FP/HW	98.23 (111/113)	93.33 (28/30)	100.00 (83/83)	407/779	6

[a]All the latent variables (LV) exhibit significant differences between normal and cancerous tissue ($p < 1 \times 10^{-5}$). acc − accuracy, sens − sensitivity, spec − specificity, nLV s − number of latent variables. All other abbreviations are explained in the main text.

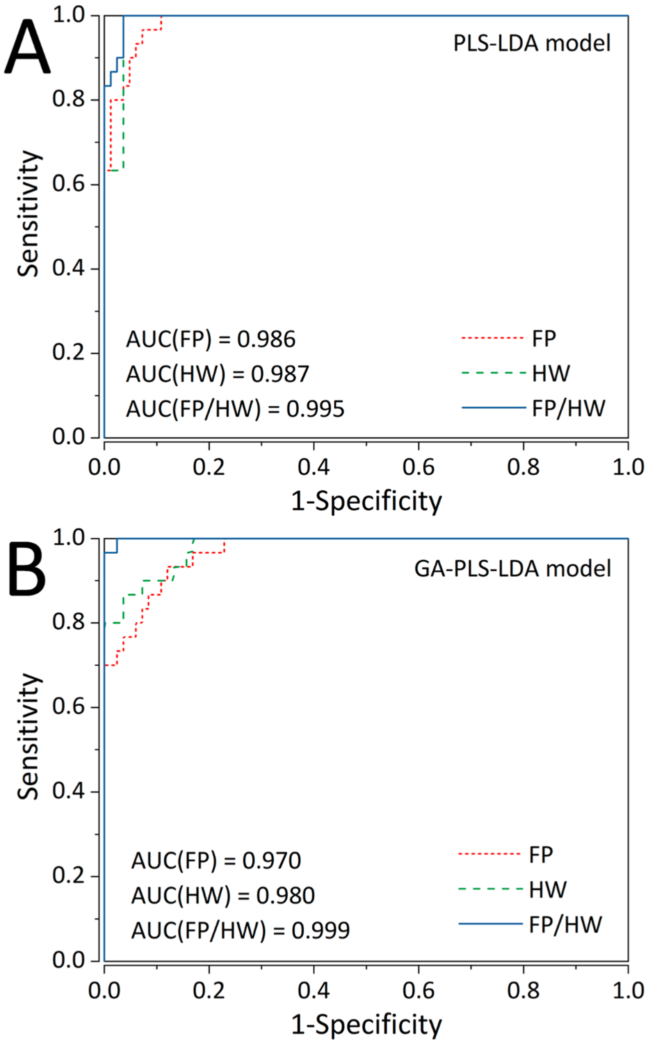

Figure 3. Receiver operating characteristic (ROC) curves of A) PLS-LDA and B) GA-PLS-LDA models for different Raman modalities (FP, HW, and FP/HW).

DOI: 10.1021/acs.analchem.9b00173
Anal. Chem. 2019, 91, 8101−8108

SRD analysis was employed for ranking and evaluation of differences between the developed PLS-LDA/GA-PLS-LDA models with the results being displayed in Figure 4.

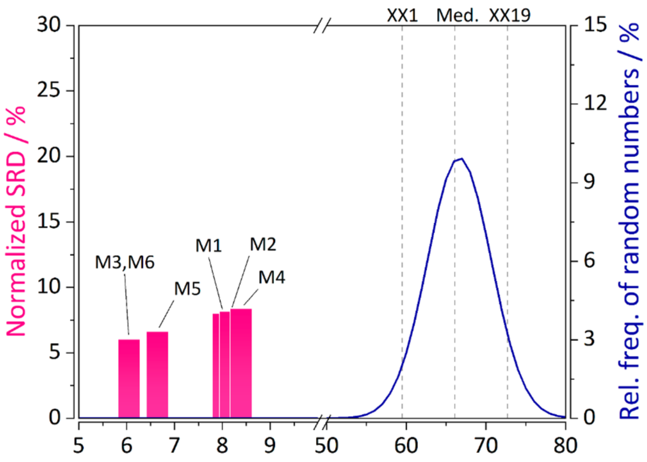

Figure 4. Comparative SRD analysis for PLS-LDA and GA-PLS-LDA models validated through comparison of ranks by random numbers. Statistical parameters of the Gaussian curve are first icosaile (XX1) of 59.5, median of 66.1, and the last icosaile (XX19) of 72.7%. Model labels: M1 – PLS-LDA (FP Raman), M2 – PLS-LDA (HW Raman), M3 – PLS-LDA (FP/HW Raman), M4 – GA-PLS-LDA (FP Raman), M5 – GA-PLS-LDA (HW Raman), M6 – GA-PLS-LDA (FP/HW Raman).

■ DISCUSSION

In this work, the effectiveness and clinical applicability of the nature-inspired fiber-optic Raman diagnostic platform developed was evaluated for the first time for enhancing in vivo diagnosis of NPC patients during endoscopy. The combined FP/HW Raman spectra abundant in both diagnostically and biochemically relevant information are fully utilized for diagnosis of NPC patients. Differences between NPC and normal tissue are discerned using PLS-LDA models. Since the combined FP/HW Raman spectra may still contain noise and uninformative features, PLS-LDA was coupled with GA for further diagnosis. Such automated selection of the most relevant Raman spectral information allows for a more reliable analysis of biochemical and biomolecular changes occurring in epithelial tissue observed through the in vivo fiber-optic Raman spectroscopy technique.[11]

Several Raman peak assignments have been made to analyze the significantly different molecular changes associated with the onset and progression of NPC (Table 1). PLS-LDA contribution plots were employed to gain information about significant wavenumber regions corresponding to the strongest biochemical differences. Figure 1B shows a significant reduction of Raman intensities at 853 (ν(C–C)-proteins), 875 (ν(C–C)-hydroxyproline), and 1078 cm^{-1} (ν(C–C)-lipids) related to the reduction in collagen and lipid contents in NPC with respect to normal tissue.[6,7,9,11,18] These decreased Raman peak intensities can be attributed to the thickening and bulging nasopharyngeal mucosa typically located near the invasive malignancies.[22,24] The thickened or bulging tissue can result in attenuation of the laser light excitation and interfere with Raman scattering emitted from the tissue sublayers.[11,12,18]

Strong positive contributions have been observed at 1209 (ν(C–C)-phenylalanine), 1265 (amide III ν(C–N)) and δ(N–H) of proteins), and 1335 cm^{-1} ($\gamma(CH_3–CH_2)$ twisting, nucleic acids), whereas the peak at 1445 cm^{-1} (CH_2 vibration of proteins and lipids) has exhibited virtually no contribution to **X**-scores. This agrees well with the information listed in Table 1 with the p-value of this peak >0.05. The stronger Raman intensity at 1335 cm^{-1} can be attributed to an increase in the DNA content of nasopharyngeal tissue associated with NPC tumorigenesis. In a recent large-scale study involved in a cohort of 20,174 participants, it was reported that NPC patients exhibit significantly higher amounts of NPC-associated Epstein–Barr virus DNA.[40] Meanwhile, the increased protein-related Raman peaks (e.g., 1209, 1265, and 1445 cm^{-1}) agree with the multitude of proteins reported as overexpressed in NPC tissue.[41,42]

Despite the high specificity, the biochemically relevant Raman peaks in the FP range are quite weak.[7,9] Raman peaks with considerably greater intensity can be observed in the HW range (Figure 1A) (the average relative intensity increases by ~230% compared to FP Raman signal determined based on the area under the curve). Significant differences between HW Raman spectra of NPC and normal tissue (Table 1) can be observed at 2940 ($\nu(CH_3)$ protein stretching), 3009 (ν(=C–H) asymmetric stretching of proteins), 3250 (ν(OH) stretching of water), and 3300 cm^{-1} (amide A ν(C–N) stretching).[13−17] Indeed, strong positive PLS contributions (Figure 1B) can be observed at 2940 and 3009 cm^{-1}, directly related to overexpression of proteins linked to growth of epithelial cells during NPC tumorigenesis.[42] On the other hand, the emergence of highly intensive water peaks at 3250 and 3400 cm^{-1} is typically attributed to local tissue conformation and interactions of OH- bonds within intracellular and extracellular matrices of epithelial tissue. The Raman water peak at ~3250 cm^{-1} exhibited strong and moderate positive PLS contributions. In NPC tissue, an increased intensity of Raman water band associated with OH stretching reflects a rearrangement of hydrogen-bond networks within the epithelium.[14−17] However, the water peak at ~3400 cm^{-1} did not exhibit a significant increase between NPC and normal tissue ($p > 1 \times 10^{-5}$) (Table 1), which may be related to the multivariate nature of PLS contributions, as the unpaired t-test is univariate which does not fully account for interactions between the mutually correlated Raman wavenumbers.

In-depth differences between the diagnostic performances of the developed PLS-LDA models can be observed from Figure 2A–C. The PLS-LDA models built from Raman spectra in the FP (Figure 2A) and HW ranges (Figure 2B) incorrectly predicted 12 (11 NPC, 1 normal) and 13 (11 NPC, 2 normal) tissue sites, respectively. The combined FP/HW Raman shows a considerable diagnostic improvement (Figure 2C) with only 5 tissue sites being incorrectly predicted (4 NPC, 1 normal). Overall, all the PLS-LDA models used are significant with $p < 1 \times 10^{-5}$, with the FP/HW PLS-LDA model being even more significant ($p < 1 \times 10^{-09}$). The increased diagnostic accuracy of the combined FP/HW technique over the FP and HW alone is also evident from the AUC values (0.995, 0.986, and 0.987) for the PLS-LDA models as shown in Figure 3A.

Performance of the best GA-PLS-LDA run is summarized in Table 2. For the combined FP/HW Raman spectra, there is a considerable improvement in diagnostic accuracy over PLS-LDA without feature selection. Since the automated polynomial fitting method[38] may leave artifacts in the preprocessed spectra, GA-based feature selection effectively

DOI: 10.1021/acs.analchem.9b00173
Anal. Chem. 2019, 91, 8101–8108

removes the affected wavenumbers, increasing the values of the diagnostic performance indicators.

Wavenumbers selected through GA-based feature selection mostly agree with the tentatively assigned Raman peaks, except for a few discrepancies (Table 1: 875, 1078, 1580, and ~3300 cm^{-1}), whereby the wavenumbers with significant NPC vs normal tissue differences were not selected. However, from Figure 1A, it is found that the wavenumbers in the immediate vicinity are indeed selected. Therefore, automating the selection of Raman wavenumbers through the developed nature-inspired diagnostic platform removes the bias that might arise from *a priori* peak assignments.

Similar to the case of PLS-LDA modeling, the posterior probability plots for GA-PLS-LDA models were constructed (Figure 2). From Figure 2D−F, it can be observed that the GA-PLS-LDA for the FP, HW, and the combined FP/HW techniques misclassified 9, 6, and 2 tissue sites (all NPC), respectively. FP/HW associated with GA-PLS-LDA models can predict most of the tissue sites correctly with the tissue site 067,1 being close to the threshold (Figure 2F), which is related to a more intricate diagnostic structure due to cancer staging. All the GA-PLS-LDA models are also significant as evident from p-values $<1 \times 10^{-9}$ with the FP/HW-based GA-PLS-LDA model being more significant ($p < 1 \times 10^{-10}$). The FP/HW-based GA-PLS-LDA model also exhibited a considerably higher AUC of 0.999 as compared to 0.970 and 0.980 for the corresponding FP and HW Raman techniques (Figure 3B).

Finally, SRD analysis has provided an objective ranking of the developed models (Figure 4). All the models have SRD values significantly different from those of normally distributed random numbers (i.e., all are located far from the first icosaile). Resulting ranking and comparison of diagnostic performance between the models are thereby clear and unbiased. The combined FP/HW Raman-based (GA-) PLS-LDA models were ranked the first two. The GA-PLS-LDA model for HW Raman was ranked third, whereas the FP Raman-based PLS-LDA model was ranked fourth. The PLS-LDA model for HW Raman was ranked fifth, while the FP Raman-based GA-PLS-LDA model was ranked last. Results of the SRD analysis affirm the diagnostic superiority of the nature-inspired diagnostic platform associated with the combined FP/HW Raman technique.

Therefore, the fully automated selection of the diagnostically significant Raman spectral information in the FP and its complementary HW range with a state-of-the-art nature-inspired Raman diagnostic platform considerably improve in vivo NPC diagnostics at endoscopy. Regardless of the outstanding diagnostic performance, a few limitations in this study should be noted. NPC patients are not randomized, which may give rise to a selection bias. Normal volunteers are not subjected to biopsies and diagnosed solely at the discretion of the clinicians. One may argue that the obtained perfect specificities may not entirely account for potential false negative diagnosis. Patients diagnosed with different grades of NPC were treated as one cancer group in the current study. Accounting for more intricate diagnostic structures should be used for further improving the sensitivity of the nature-inspired Raman-based diagnostic platform. Within the scope of future clinical trials, we aim to resolve these drawbacks by further increasing the cohort of patients to truly assess the clinical merits of fiber-optic FP/HW Raman spectroscopy with nature-inspired genetic algorithms for enhancing endoscopic diagnostics in the head and neck.

CONCLUSIONS

In conclusion, a fiber-optic Raman diagnostic platform with nature-inspired genetic algorithms has been developed and comprehensively evaluated for enhancing in vivo diagnosis of NPC patients at endoscopy. A plethora of diagnostically significant biochemical information are extracted through automated feature selection of in vivo FP/HW spectra of nasopharyngeal tissue. Significant Raman differences between normal and NPC tissue are elucidated using the robust GA-based diagnostic platform. FP/HW Raman-based GA-PLS-DA diagnostic models exhibit outstanding diagnostic performance in differentiating NPC from normal tissue. The promising result in this work unfolds the great potential to establish the nature-inspired fiber-optic Raman spectroscopy as a clinical tool for enhancing real-time in vivo detection and diagnosis of NPC patients at the molecular level during clinical endoscopic examination.

ASSOCIATED CONTENT

Supporting Information

The Supporting Information is available free of charge on the ACS Publications website at DOI: 10.1021/acs.analchem.9b00173.

Figure S1, depiction of GA unit for binary spectral feature selections exemplified on Raman spectra in FP region; Figure S2, in vivo raw FP/HW and difference spectra acquired from 113 tissue sites (normal = 83, NPC = 30) of 63 patents at clinical endoscopy; Figure S3, distribution of performance metrics: A), accuracy, B) sensitivity, C) specificity, and D) AUC, for PLS-LDA model calculated using resampling with replacement; Figure S4, distribution of performance metrics: A), accuracy, B) sensitivity, C) specificity, and D) AUC, for GA-PLS-LDA model calculated using resampling with replacement (PDF)

AUTHOR INFORMATION

Corresponding Author

*Phone: +65- 6516-8856. Fax: +65- 6872-3069. E-mail: biehzw@nus.edu.sg.

ORCID

Petar Žuvela: 0000-0001-6481-2241

Zhiwei Huang: 0000-0002-0104-9135

Author Contributions

§P.Ž., K.L., C.S., and W.Z. contributed equally to the work.

Notes

The authors declare no competing financial interest.

ACKNOWLEDGMENTS

This work was supported by the Academic Research Fund (AcRF)-Tier 1 and Tier 2 from the Ministry of Education (MOE) (MOE2014-T2-1-010) and the National Medical Research Council (NMRC) (NMRC/TCR/016-NNI/2016), Singapore.

REFERENCES

(1) Chua, M. L. K.; Wee, J. T. S.; Hui, E. P.; Chan, A. T. C. *Lancet* **2016**, *387* (10022), 1012−1024.

(2) Jemal, A.; Bray, F.; Center, M. M.; Ferlay, J.; Ward, E.; Forman, D. *Ca-Cancer J. Clin.* **2011**, *61* (2), 69−90.

(3) Stoker, S. D.; van Diessen, J. N. A.; de Boer, J. P.; Karakullukcu, B.; Leemans, C. R.; Tan, I. B. *Curr. Treat. Options Oncol.* **2013**, *14* (4), 475−491.
(4) Tang, L.-Q.; Li, C.-F.; Li, J.; Chen, W.-H.; Chen, Q.-Y.; Yuan, L.-X.; Lai, X.-P.; He, Y.; Xu, Y.-X.-X.; Hu, D.-P.; et al. *J. Natl. Cancer Inst.* **2016**, *108* (1), No. djv291.
(5) Raman, C. V.; Krishnan, K. S. *Nature* **1928**, *121* (3048), 501−502.
(6) Teh, S. K.; Zheng, W.; Lau, D. P. C.; Huang, Z. *Analyst* **2009**, *134* (6), 1232.
(7) Lau, D. P.; Huang, Z.; Lui, H.; Man, C. S.; Berean, K.; Morrison, M. D.; Zeng, H. *Lasers Surg. Med.* **2003**, *32* (3), 210−214.
(8) Wang, J.; Bergholt, M. S.; Zheng, W.; Huang, Z. *Opt. Lett.* **2013**, *38* (13), 2321−2323.
(9) Huang, W.; Wu, S.; Chen, M.; Sun, L.; Li, Y.; Huang, M.; Huang, S.; Xu, Z.; Chen, R.; Zeng, H. *J. Raman Spectrosc.* **2015**, *46* (6), 537−544.
(10) Lau, D. P.; Huang, Z.; Lui, H.; Anderson, D. W.; Berean, K.; Morrison, M. D.; Shen, L.; Zeng, H. *Lasers Surg. Med.* **2005**, *37* (3), 192−200.
(11) Lin, K.; Zheng, W.; Lim, C. M.; Huang, Z. *Theranostics* **2017**, *7* (14), 3517−3526.
(12) Bergholt, M. S.; Zheng, W.; Ho, K. Y.; Teh, M.; Yeoh, K. G.; Yan So, J. B.; Shabbir, A.; Huang, Z. *Gastroenterology* **2014**, *146* (1), 27−32.
(13) Bergholt, M. S.; Zheng, W.; Lin, K.; Wang, J.; Xu, H.; Ren, J.; Ho, K. Y.; Teh, M.; Yeoh, K. G.; Huang, Z. *Anal. Chem.* **2015**, *87* (2), 960−966.
(14) Mo, J.; Zheng, W.; Low, J. J. H.; Ng, J.; Ilancheran, A.; Huang, Z. *Anal. Chem.* **2009**, *81* (21), 8908−8915.
(15) Lin, K.; Lau, D. P. C.; Huang, Z. *Biosens. Bioelectron.* **2012**, *35* (1), 213−217.
(16) Wang, J.; Lin, K.; Zheng, W.; Yu Ho, K.; Teh, M.; Guan Yeoh, K.; Huang, Z. *Sci. Rep.* **2015**, *5* (1), 12957.
(17) Bergholt, M. S.; Lin, K.; Wang, J.; Zheng, W.; Xu, H.; Huang, Q.; Ren, J.; Ho, K. Y.; Teh, M.; Srivastava, S.; et al. *J. Biophotonics* **2016**, *9* (4), 333−342.
(18) Huang, Z.; McWilliams, A.; Lui, H.; McLean, D. I.; Lam, S.; Zeng, H. *Int. J. Cancer* **2003**, *107* (6), 1047−1052.
(19) Draga, R. O. P.; Grimbergen, M. C. M.; Vijverberg, P. L. M.; van Swol, C. F. P.; Jonges, T. G. N.; Kummer, J. A.; Ruud Bosch, J. L. H. *Anal. Chem.* **2010**, *82* (14), 5993−5999.
(20) Huang, Z.; Bergholt, M. S.; Zheng, W.; Lin, K.; Ho, K. Y.; Teh, M.; Yeoh, K. G. *J. Biomed. Opt.* **2010**, *15* (3), 037017.
(21) Duraipandian, S.; Bergholt, M. S.; Zheng, W.; Ho, K. Y.; Teh, M.; Yeoh, K. G.; Yan So, J. B.; Huang, Z. *J. Biomed. Opt.* **2012**, *17* (8), 081418.
(22) Lin, D.; Qiu, S.; Huang, W.; Pan, J.; Xu, Z.; Chen, R.; Feng, S.; Chen, G.; Li, Y.; Short, M.; et al. *J. Biophotonics* **2018**, *11* (4), No. e201700251.
(23) Bergholt, M. S.; Lin, K.; Zheng, W.; Lau, D. P. C.; Huang, Z. *J. Biomed. Opt.* **2012**, *17* (7), 0770021.
(24) Lim, C. M.; Nagaraja, R.; Loh, T.; Zheng, W.; Wang, J.; Lin, K.; Huang, Z. *Oncotarget* **2017**, *8* (30), 49443−49450.
(25) Stone, N.; Kendall, C. A.; Smith, J.; Crow, P.; Barr, H. *Faraday Discuss.* **2004**, *126*, 141−157.
(26) Lieber, C. A.; Mahadevan-Jansen, A. *Appl. Spectrosc.* **2003**, *57* (11), 1363−1367.
(27) Bolón-Canedo, V.; Sánchez-Maroño, N.; Alonso-Betanzos, A.; Benítez, J. M.; Herrera, F. *Inf. Sci. (N. Y.)* **2014**, *282*, 111−135.
(28) Forrest, S. *Science (Washington, DC, U. S.)* **1993**, *261* (5123), 872−878.
(29) Holland, J. H. *Adaptation in Natural and Artificial Systems: An Introductory Analysis with Applications to Biology, Control and Artificial Intelligence*; MIT Press: Cambridge, MA, USA, 1992.
(30) Žuvela, P.; Liu, J. J.; Macur, K.; Bączek, T. *Anal. Chem.* **2015**, *87* (19), 9876−9883.
(31) Duraipandian, S.; Mo, J.; Zheng, W.; Huang, Z. *Analyst* **2014**, *139* (21), 5379−5386.
(32) Li, S.; Li, L.; Zeng, Q.; Zhang, Y.; Guo, Z.; Liu, Z.; Jin, M.; Su, C.; Lin, L.; Xu, J.; et al. *Sci. Rep.* **2015**, *5* (1), 9582.
(33) Song, L. M. W. K.; Molckovsky, A.; Wang, K. K.; Burgart, L. J.; Dolenko, B.; Somorjai, R. L.; Wilson, B. C. *Proc. SPIE* **2005**, *5692*, 140−146.
(34) Barker, M.; Rayens, W. *J. Chemom.* **2003**, *17* (3), 166−173.
(35) Efron, B. *Biometrika* **1981**, *68* (3), 589−599.
(36) Zweig, M. H.; Campbell, G. *Clin. Chem.* **1993**, *39* (4), 561−577.
(37) American National Standards Institute. *American National Standard for the Safe Use of Lasers*; New York, 1986.
(38) Eriksson, L.; Trygg, J.; Wold, S. *J. Chemom.* **2008**, *22* (11−12), 594−600.
(39) Héberger, K. *TrAC, Trends Anal. Chem.* **2010**, *29* (1), 101−109.
(40) Lam, W. K. J.; Jiang, P.; Chan, K. C. A.; Cheng, S. H.; Zhang, H.; Peng, W.; Tse, O. Y. O.; Tong, Y. K.; Gai, W.; Zee, B. C. Y.; et al. *Proc. Natl. Acad. Sci. U. S. A.* **2018**, *115* (22), E5115−E5124.
(41) Branco, A.; Todorovic Fabro, A.; Gonçalves, T. M.; Garcia Martins, R. H. *Otolaryngol.–Head Neck Surg.* **2015**, *152* (2), 302−307.
(42) Lu, H.; Liang, D.; Zhu, Y.; Xu, W.; Zhou, K.; Liu, L.; Liu, S.; Yang, W. *Oncotarget* **2017**, *8* (56), 96359−96372.

DOI: 10.1021/acs.analchem.9b00173
Anal. Chem. 2019, 91, 8101−8108
