# Supplementary material for: The accuracy of Fiber-Optic Raman Spectroscopy in the detection and diagnosis of head and neck neoplasm in vivo: a systematic review and meta-analysis
Source: PeerJ. 2023 Dec 11;11:e16536. doi: 10.7717/peerj.16536 (PMC10720414; doi:10.7717/peerj.16536)
Supplement: Data S1 [file peerj-11-16536-s001.zip › article/Head Neck - 2017 - Malik - In vivo Raman spectroscopy assisted early identification of potential second primary.pdf]

Received: 1 December 2016 | Revised: 21 April 2017 | Accepted: 15 June 2017
DOI: 10.1002/hed.24884

ORIGINAL ARTICLE

WILEY

In vivo Raman spectroscopy–assisted early identification of potential second primary/recurrences in oral cancers: An exploratory study

Akshat Malik, MS[1] | Aditi Sahu, PhD[2] | S. P. Singh, PhD[2] | Atul Deshmukh, MDS[2] | Pankaj Chaturvedi, MS[1] | Deepa Nair, MS[1] | Sudhir Nair, M Ch[1] | C. Murali Krishna, PhD[2]

[1] Head and Neck Surgical Oncology, Tata Memorial Center, Mumbai, India
[2] Chilakapati Laboratory, Advanced Center for Training, Research, and Education in Cancer (ACTREC), Tata Memorial Center, Navi Mumbai, India

Correspondence
Chilakapati Murali Krishna, Chilakapati Laboratory, Advanced Center for Training, Research, and Education in Cancer (ACTREC), TMC, Kharghar, Sector "22," Navi Mumbai, 410210, India.
Email: mchilakapati@actrec.gov.in, pittu1043@gmail.com

Abstract

Background: Higher rates of local recurrences and second primaries, ascribable to field cancerization, are known problem in oral cancers. The present study explored utility of identification of potential recurrences by Raman spectroscopy, which has been shown to identify oral precancers, cancers, and field cancerization in humans and micro-sized mechanical irritation-induced tumors in animals.

Methods: Raman spectra were acquired from tumor and contralateral normal mucosa in 99 patients with oral cancer who were then followed up for appearance of clinically apparent cancerous lesions. Misclassifications observed in subsequent multivariate statistical analysis between contralateral normal and tumor spectra were correlated with appearance of new malignant lesions.

Results: The patients with mismatched spectra had 1.5 times higher chances of developing local recurrence. The sensitivity of Raman spectroscopy in predicting the recurrences was 80% and the specificity was 29.7%.

Conclusion: Findings provide proof-of-concept for Raman spectroscopy-based identification of sites that have higher propensity to progress to carcinomas before becoming clinically apparent. Prospective validation of Raman spectroscopy by including additional oral cavity subsites and use of multifiber bundles may improve rate of identification of recurrence-prone subjects.

KEYWORDS
cancer field effects, in vivo, oral cancer, principal component linear discriminant analysis (PC-LDA), Raman spectroscopy, recurrence

1 | INTRODUCTION

Oral cancers are among the most common cancers in Southeast Asia.[1,2] These are mostly associated with tobacco and alcohol use.[3,4] These carcinogens affect the entire upper aerodigestive tract mucosa resulting in molecular changes, which can progress further and result into carcinomas. Such changes occurring over the exposed mucosa are referred to as field cancerization.[5] Because of such changes, even after successful treatment of primary cancer, subjects stay at a risk of developing recurrences or second primaries. The risk of developing a second primary in patients with oral cancer is

 © 2017 Wiley Periodicals, Inc.

up to 7%.[6] Most of the time, such cancer field effects are not clinically apparent. Detecting such field changes requires tissue sample and molecular analysis, which may not be feasible in a clinical setting.

Raman spectroscopy, a vibrational spectroscopy method based on inelastic scattering of light, provides a molecular fingerprint of the sample under consideration.[7,8] Applications of Raman spectroscopy in cancers are well documented.[9–12] After exploratory studies in animal models with oral cancer,[13,14] ex vivo studies on human oral cancer and normal tissues demonstrated the potential of Raman spectroscopy in identification and grade-wise differentiation of oral cancers.[15–17] Less invasive samples, like saliva, blood plasma, and serum, have also been explored for oral cancer diagnoses.[18–21] Serum studies on approximately 350 subjects have even demonstrated potential of Raman spectroscopy in oral cancer screening.[22] Several in vivo Raman spectroscopy studies have demonstrated its utility in classification of healthy, oral precancerous and cancerous lesions in human subjects in the buccal mucosa subsite.[23–25] For studies involving multiple subsites, successful classification of normal tissues from malignant and potentially malignant tissues have been reported using anatomically matched algorithms[26,27]; whereas 2 recent studies with high sensitivity rates for oral cancer diagnosis have highlighted that anatomy-based algorithms may be unnecessary for oral cancer screening and diagnosis.[28,29] The potential of Raman spectroscopy in surgical demarcations has also been successfully explored in recent years.[30–32] Further, another study has identified cancer field effects/malignancy-associated changes in the normal mucosa of patients with oral cancer,[33] these are the earliest events in oral carcinogenesis. Hamster buccal pouch (animal model) is very similar to human buccal mucosa and mimics oral carcinogenesis. Studies on this model have shown significant incidence of misclassifications between spectra from 7,12-Dimethylbenz(a)anthracene (DMBA)-treated and control buccal pouches suggesting a role of either heterogeneity in treated pouches or presence of some changes in the control pouches.[34] Definitive histopathological and biochemical changes, including severe dysplasia, were detected in these control sections, thus, all such misclassifications should be given due consideration.[35] In view of this, the present Raman spectroscopy study was carried out in patients diagnosed with oral squamous cell carcinoma (SCC) to screen the mucosa adjacent to the cancer lesion (contralateral normal) for detecting such misclassifications, as these misclassifications may be indicative of underlying carcinogenic events. These patients were then followed up clinically for appearance of any clinically apparent cancerous lesion in the oral cavity. Correlation between the misclassifications seen in the spectra between the contralateral normal mucosa and tumor and appearance of any new malignant lesion in the oral cavity was carried out.

2 | MATERIALS AND METHODS

This was a prospective study conducted at a tertiary care referral cancer center. Institutional review board approval was taken before conducting the study. This study included diagnosed cases of oral SCC. The patients were selected randomly from the outpatient department depending upon their willingness to be part of the study. A total of 99 patients were recruited for the study between 2010 and 2012. Written informed consent was taken from all patients. After recruitment, patients were asked to rinse their mouth with water and were seated in front of the portable Raman instrument High Efficiency-785 (Jobin-Yvon-Horiba, France; Figure 1). This system consists of a diode laser (Process Instruments, Bakersfield, CA) of 785-nm wavelength as the excitation source, a high-efficiency spectrograph with fixed 950-gr/mm grating coupled with a CCD (Synapse). The instrument has no movable parts and the spectral resolution, as specified by the manufacturer, is $4\,cm^{-1}$. The commercially obtained fiber probe (Inphotonics, Norwood, MA) consisting of a 105-μm excitation fiber and a 200-μm collection fiber (NA-0.40) was used to couple the excitation source and the detection system. The estimated spot size and depth of the field, as per the manufacturer's specifications, are 105 μm and 1 mm, respectively. A detachable spacer of length (5 mm) was attached at the tip of the probe to maintain constant working distance during all measurements. Before each measurement, these spacers were disinfected by CIDEX (Johnson & Johnson, Mumbai, India) solution to avoid intersubject contamination. Spectral acquisition parameters were: λ_{ex} (785 nm), laser power -80 mW, spectra were integrated for 3 seconds and averaged over 3 accumulations. The spectra were acquired by a trained medical professional at designated subsites. The spectra recording of the patients were done in 2 stages. In the initial stage of the study, the spectra were recorded from the tumor site and from the contralateral buccal mucosa (approximately 4 spectra per subject). Contralateral buccal mucosa was selected for recording the spectra as it was farthest from the site of primary lesion. Spectra were acquired from mucosal surfaces opposing canine, first premolar, second premolar, first molar, and second molar to ensure uniformity in spectral acquisition. In the second stage of the study, spectra were acquired from the site of tumor as well as from 6 different contralateral subsites, namely, buccal mucosa, lip, tongue, hard palate, floor of mouth, and retromolar trigone (approximately 14 spectra per subject). Spectra were acquired from specific sites; from the buccal mucosa, spectra were acquired from mucosa opposing teeth positions of the second premolar, first molar, and second molar. In case of lip subsite, labial mucosa opposing the 2 central incisors was selected for spectral acquisition. For the tongue, spectra were acquired from

10970347, 2017, 11, Downloaded from https://onlinelibrary.wiley.com/doi/10.1002/hed.24884 by Sichuan University, Wiley Online Library on [27/10/2023]. See the Terms and Conditions (https://onlinelibrary.wiley.com/terms-and-conditions) on Wiley Online Library for rules of use; OA articles are governed by the applicable Creative Commons License

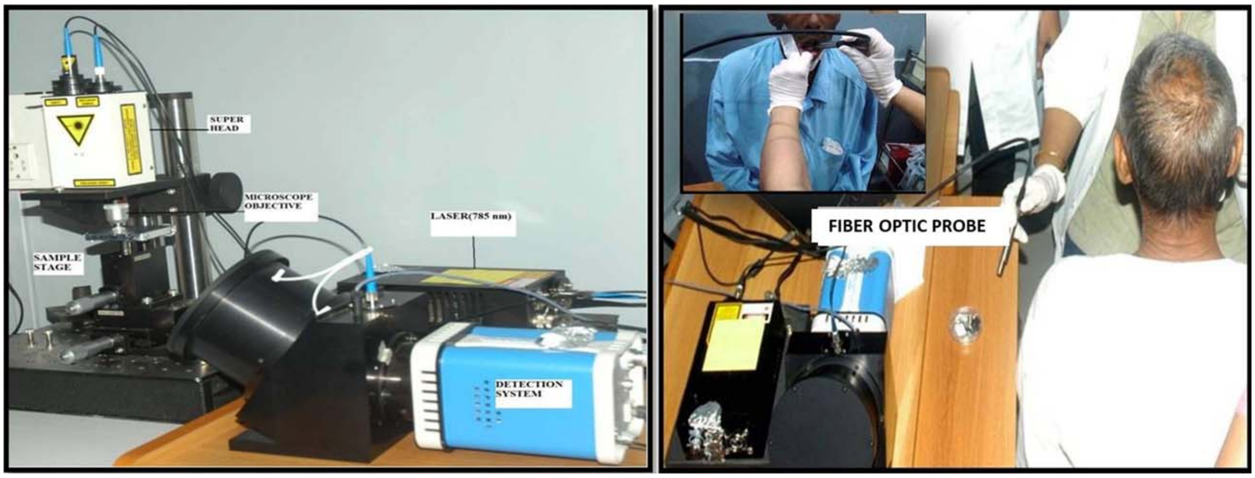

FIGURE 1 Fiber-optic Raman spectroscope for clinical applications [Color figure can be viewed at wileyonlinelibrary.com]

lateral border in the anterior part of the tongue. In cases of the hard palate, the point anterior to the hard palate-soft junction was used for spectra recording. Floor of the mouth points included those adjacent to the first and second premolars. For the retromolar trigone, the mucosal area posterior to the last molar was used for spectral acquisition. Spectra were analyzed using principal component linear discriminant analysis (PC-LDA) followed by the leave-one-out cross validation method using MATLAB-based in-house codes.[36]

All these patients underwent surgery followed by appropriate adjuvant therapy. Their clinical, histopathological, and follow-up details were noted from the electronic medical records. Only patients whose follow-up details of at least 2 years were available were included in the analysis. Note was made of appearance of any malignant lesion in the oral cavity during the period of follow-up. These lesions were included in the analysis only if their histopathological report of malignancy was available. Subsequently, presence of misclassifications seen between the normal mucosa and tumor and appearance of any new malignant lesion in the oral cavity were correlated. Sensitivity and specificity of Raman spectroscopy in predicting recurrences was calculated.

3 | RESULTS

This study involved 99 patients with oral SCCs. The first stage of the study involved 42 patients. Of these, 14 had early disease (stages I/II) and 28 had advanced disease (stages III/IV). Classifying based upon the site of primary lesion, 13 patients had primary lesions of the tongue, 16 had lesions over the buccal mucosa, 9 had lower alveolus lesions, and there was 1 patient each with retromolar trigone, floor of mouth, upper alveolus, and lip lesions. Mean Raman spectra of contralateral and tumor buccal mucosas are shown in Figure 2A. The spectral features indicated the presence of higher DNA and proteins in tumors compared to contralateral normal, thus corroborating earlier studies.[13–16,20,21] The spectra recorded from the contralateral buccal mucosa and tumor site were analyzed using PC-LDA followed by leave-one-out cross validation, (Figure 3A,B). As shown in Figure 3A, few misclassifications were noticed between the contralateral buccal mucosa and the tumor spectra. Such misclassifications were noted in 13 patients. Clinical data were evaluated to see if any of these patients developed local recurrences over follow-up. There were no local recurrences seen in the

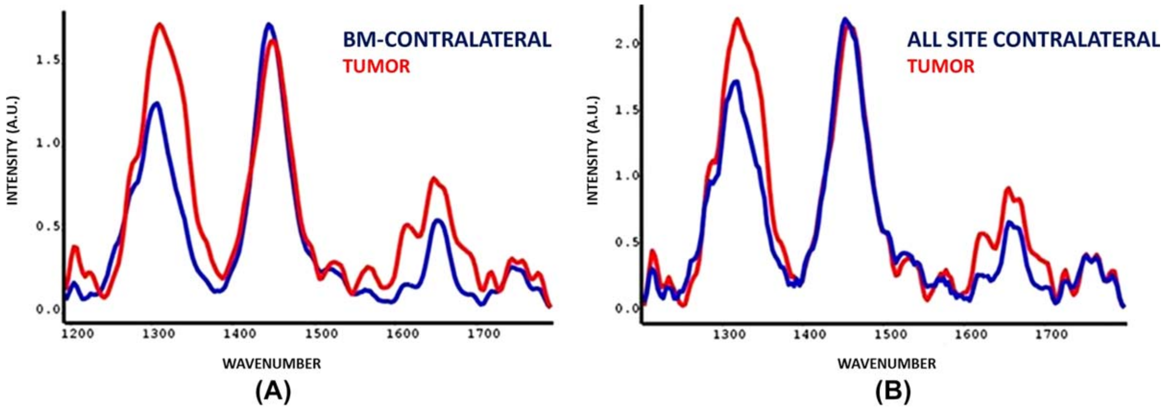

FIGURE 2 Average spectra for contralateral (blue) and tumor (red) for (A) buccal mucosa and (B) all subsites [Color figure can be viewed at wileyonlinelibrary.com]

10970347, 2017, 11, Downloaded from https://onlinelibrary.wiley.com/doi/10.1002/hed.24884 by Sichuan University, Wiley Online Library on [27/10/2023]. See the Terms and Conditions (https://onlinelibrary.wiley.com/terms-and-conditions) on Wiley Online Library for rules of use; OA articles are governed by the applicable Creative Commons License

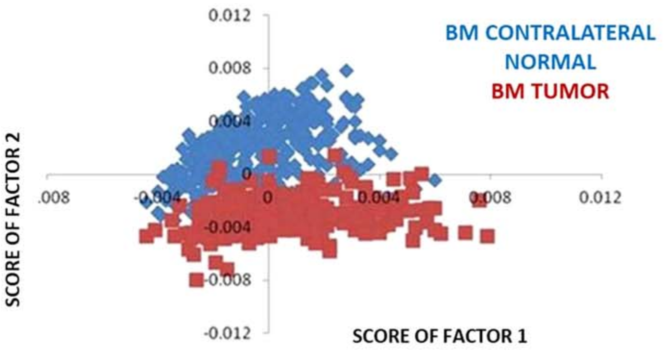

(A)

PC-LDA

	Contralateral	Tumor	Total
Contralateral	325	33	358
Tumor	7	238	245

LOOCV

	Contralateral	Tumor	Total
Contralateral	317	41	358
Tumor	12	233	245

(B)

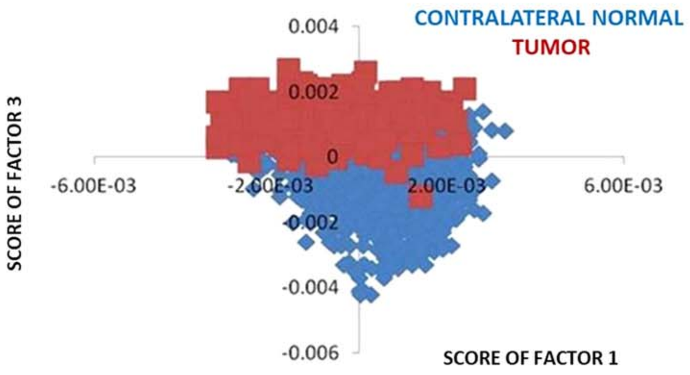

(C)

PC-LDA

	Contralateral	Tumor	Total
Contralateral	900	225	1125
Tumor	53	224	277

LOOCV

	Contralateral	Tumor	Total
Contralateral	897	228	1125
Tumor	61	216	277

(D)

FIGURE 3 Principal component linear discriminant analysis (PC-LDA) analysis for contralateral normal sites and tumor A, B, buccal mucosa (BM) site; C, D, all subsites). (A) PC-LDA scatter plot for BM contralateral and tumor. (B) PC-LDA and leave-one-out cross-validation method (LOOCV) confusion matrix for buccal mucosa contralateral and tumor. (C) PC-LDA scatter plot for all contralateral normal sites and tumors. (D) PC-LDA and LOOCV confusion matrix for all contralateral normal sites and tumors [Color figure can be viewed at wileyonlinelibrary.com]

mismatched group. In the matched group (no misclassifications of contralateral with tumor spectra), 6 patients developed local recurrence and 2 had second primary within the oral cavity. However, it should be noted that, in this case, spectra were recorded from only the buccal mucosa subsite. Therefore, a more thorough evaluation of the rest of the oral cavity was merited to rule out changes associated with field cancerization and to have a proper clinical correlation.

Hence, in the second stage, spectra were acquired from at least 6 different subsites, because of practical consideration of spectral acquisition time and subject compliance, apart from the site of the primary tumor. Fifty-seven patients were included in this stage. Early-stage disease (stages I/II) was seen in 14 patients and 43 patients had advanced-stage disease (stages III/IV). Twenty patients had primary lesions in the tongue, 22 had buccal mucosa primaries, 9 had lower alveolus primaries, 3 patients had primary over retromolar trigone, 2 had lesions over the hard palate, and 1 had floor of the mouth primary. Mean Raman spectra of contralateral and tumors pooled from different subsites are shown in Figure 2B. In this case, also higher presence of DNA and protein spectral features were noticed in tumor spectra with respect to contralateral normal.[13–16,20,21] As shown in Figure 3C, 2 slightly overlapping clusters were observed for normal and tumor groups. In the leave-one-out cross validation table (Figure 3D) of the 57 patients, 41 were noted to have misclassifications of apparently normal mucosal spectra with the spectra from the tumor site. In this case, among those whose spectra matched (no misclassification with tumor), 2 patients developed local recurrences (2/16; 12.5%). For the group whose spectra showed mismatch with tumor spectra, 8 patients had local recurrences (8/41; 19.5%). Thus, the patients with mismatched spectra had 1.5 times higher chances of developing local recurrence. Additionally, 8 of 10 patients who developed recurrence were correctly identified, whereas only 14 of 47 subjects who were less prone to development of recurrences (based on no misclassification with tumor) were correctly identified. Therefore, the sensitivity of Raman spectroscopy in predicting the recurrences was found to be 80%, whereas the specificity was 29.7%.

10970347, 2017, 11, Downloaded from https://onlinelibrary.wiley.com/doi/10.1002/hed.24884 by Sichuan University, Wiley Online Library on [27/10/2023]. See the Terms and Conditions (https://onlinelibrary.wiley.com/terms-and-conditions) on Wiley Online Library for rules of use; OA articles are governed by the applicable Creative Commons License

4 | DISCUSSION

In 1928, the Raman Effect was discovered by Sir C. V. Raman[7] after seminal experiments on light scattering. The Raman Effect is a fundamental 2-photon process in which energy is exchanged between light and matter, this exchange of energy is measured in the form of intensity measurement of inelastically scattered radiation. Raman applications in cancer diagnosis and screening have been one of the very active areas of research in recent times and a large number of studies on the variety of cancers have already been reported. There have also been several topical reviews covering different aspects of Raman applications in cancers.[9–11] Raman spectroscopy has shown potential in detection of both oral and cutaneous SCCs, along with melanoma and basal cell carcinomas. Although 1 study in 2008 demonstrated 100% sensitivity and 91% specificity in differentiating healthy and malignant SCC and basal cell carcinoma tissues, the most comprehensive in vivo study on skin cancers showed a sensitivity of 99% but lower specificity for malignant and benign lesion differentiation.[37,38] Several in vivo Raman spectroscopy studies in oral cancers have successfully demonstrated its utility in classification of normal, premalignant, and malignant lesions in the oral cavity[23–25] and even detecting very early cancer field effect and malignancy-associated changes.[33] Although the oral cancer diagnosis study by Singh et al[25] (2012) reported classification efficiencies in the range of 72%-96% for differentiating normal, premalignant, and malignant conditions, Krishna et al[27] reported sensitivity and specificity as 96% and 99% (normal vs malignant) and 99% and 98% (normal vs potentially malignant). Age-related physiological changes in buccal mucosa were also observed, but these did not have a bearing on the diagnostic ability of the technique.[39] Another study has reported site-wise (tongue, buccal, and lip) spectral variations in healthy volunteers. These variations were lost with the severity of the pathology (premalignant or malignant lesions). This suggested that no subsite-wise algorithms are required for screening purposes and the pooled subsite model gave a sensitivity of 100% and specificity of 98% in identifying normal against all pathological conditions (contralateral normal, premalignant, and malignant).[28] In a recent pilot study for differentiating normal and benign from premalignant and malignant conditions, premalignant and malignant lesions could be predicted with 100% sensitivity and 77% specificity.[29] Even the surgical demarcation studies could successfully differentiate tumor and nontumor tissue with an accuracy of either 86% or in the range of 75%-100%.[30–32] The majority of the oral cancer studies have shown high accuracy, sensitivity, and specificity values for oral cancer diagnosis, or specifically identifying normal, benign, and premalignant from malignant conditions. Several of these studies have also reported misclassifications between contralateral normal mucosa (clinically normal appearing areas in subjects with tumor) and tumor site. Misclassifications indicate similarities between contralateral sites and tumors. As these were contralateral spectra, due to ethical and practical considerations (for requirement of biopsy for histopathological examination), no definitive confirmatory diagnostic tests were carried out. These misclassifications were putatively attributed to either the inherent heterogeneity of tumors, especially islands of normal cells within tumors, or could be possibly attributed to probing of advancing front of tumors (at the tumor periphery). On the other hand, experimental carcinogenesis studies on hamster buccal pouch (a well-known animal model that mimics human buccal mucosa and oral carcinogenesis) could detect microsized tumors in controls, which were attributable to mechanical irritation.[34,35] In these studies, repeated misclassifications were reported between control and treated conditions. In this case, unlike in human healthy volunteer studies, in depth histopathology and immunohistochemistry analysis were carried out. These analyses demonstrated pathological changes in control buccal pouches. These studies indicate the potential clinical relevance of mismatches of control conditions (nontumor mucosa) with tumors. Further, it is known that in subjects exposed to carcinogens like tobacco and alcohol, the entire upper aerodigestive tract mucosa undergoes changes. The insult caused by these carcinogens results in cancer field effects or precancerous changes. Areas within such insulted mucosa may undergo progression and result in cancer. Significant numbers of local recurrences and second primaries observed in oral cancer subjects can be attributed to these field mucosal alterations. Hence, the present studies were taken up to assess the clinical importance of these misclassifications and decipher any possible relationship with development of second primary tumors/recurrences.

In the first stage of this study, spectra from the tumor and from the contralateral buccal mucosa were acquired and analyzed. In a few cases, spectra from the clinically normal mucosa were misclassified with that of the tumor. However, no correlation between these changes and local recurrences could be observed. As animal experiments had suggested otherwise, and in order to assess field cancerization more comprehensively, spectra were recorded from other subsites of oral cavity in the second stage of the study. In this part of the study, spectra were recorded from the primary tumor site as well as 6 other subsites in the oral cavity. These spectra were also analyzed as earlier using PC-LDA followed by leave-one-out cross validation. Note was made of patients whose spectra from clinically normal looking mucosa misclassified with tumor mucosa. Disease status on follow-up was noted for both the group whose spectra matched that of

10970347, 2017, 11, Downloaded from https://onlinelibrary.wiley.com/doi/10.1002/hed.24884 by Sichuan University, Wiley Online Library on [27/10/2023]. See the Terms and Conditions (https://onlinelibrary.wiley.com/terms-and-conditions) on Wiley Online Library for rules of use; OA articles are governed by the applicable Creative Commons License

the normal mucosa and for the group whose spectra mismatched with the tumor spectra. It was seen that, in patients whose spectra mismatched with that of the tumor, there was a 1.5-times higher chance of developing local recurrence. Thus, findings of the present exploratory study could be quite significant from the point of view of feasibility of early identification of recurrences and second primary lesions. This further provides the basic evidence that, although molecular changes of field cancerization may not be clinically apparent, these can be detected by Raman spectroscopy. These spectral mismatches with tumor might be indicative of molecular changes that have occurred in the normal looking mucosa and may suggest the possibility of progression to frank carcinoma in due course. Studies have shown that the head and neck cancers often arise from a preexisting field of genetically altered cells, this biological phenomenon is referred to as field cancerization.[5,40] These changes are initially present only at a molecular level and no clinically apparent lesion may be present. These can give rise to new tumors post curative treatment of the primary lesion.[41,42] Although recurrences are a significant problem in the management of oral cancers, there are no widely used techniques to detect field cancerization and to predict recurrences. The commonly used follow-up protocol regimen to detect recurrence in patients with oral cancer after primary curative treatment includes 3-month assessments for the initial 2 years, biannual assessment for the next 2 years, followed by a subsequent annual examination by visual inspection and palpation. Biopsy and imaging modalities are used only for clinically suspicious cases. In this context, it is pertinent to explore the noninvasive methods, like Raman spectroscopy for identification of field changes that can progress to tumors. Real-time in vivo Raman spectroscopy can provide rapid screening of the oral mucosa during follow-up and serve as a useful clinical adjunct to detect field changes. The present exploratory study provides a proof of concept for the same where Raman spectroscopy has been used to screen patients of oral cancer who may have a higher likelihood of developing local recurrences. These patients can then be followed up rigorously to detect possible recurrences in a timely manner. As a screening tool, the test is expected to have a good sensitivity and acceptable specificity. The sensitivity of Raman spectroscopy in predicting the recurrences was found to be 80% but the specificity was lower at 29.7%. In the context of high sensitivity and specificity (>90%), demonstrated by Raman spectroscopy in classifying cancer and noncancerous conditions for oral, cervical, and some cutaneous SCCs, the values obtained in the present study, especially specificity, is comparatively lower. However, high sensitivity and lower specificity rates may be acceptable for such preliminary screening tools, as therapeutic clinical decisions will only be based after standard confirmatory tests carried out subsequent to positive findings in the preliminary test. Further, the specificity could probably be improved upon by increasing the sample size (number of spectra recorded and the number of patients in the analysis) and including more subsites. In the present study, approximately 14 spectra from 6 different subsites with probing areas of 200 μm each were recorded. Better sampling from extensive mucosal areas can lead to better correlation, as is evident from this study, as the first stage involving analysis of only single control site led to poor/no correlation, whereas the second stage, which included more sites (and more number of spectra) yielded better correlation between recurrence and mismatched spectra. Further prospective studies should focus on probing larger areas, preferably the entire oral cavity using the multifiber-based bundles. Inclusion of more subsites may help improve the sensitivity and specificity rates observed in this study. Additionally, each spectrum was acquired for 3 seconds and averaged over 3 accumulations, thus, each spectra was recorded for approximately 10 seconds. In this study, the total acquisition time for the 6 different subsites (including device repositioning at each subsequent anatomic location) in each patient would take anywhere between 15 and 20 minutes. Studies have reported acquisition time for real-time in vivo Raman spectroscopy as ranging from minutes to less than 1 second. These studies involve use of specialized fiber-optic probes with multifiber bundles leading to large spot size (3.5 mm), which enable enhanced signal collection and reducing acquisition time.[38,43] Implementing similar instrumentation design can help in faster and more comprehensive Raman spectra acquisition in the entire oral cavity and facilitate detection of clinically occult field changes.

5 | CONCLUSION

This study provides proof of concept of using Raman spectroscopy for detecting field changes and changes at the molecular level, which can subsequently progress to tumor formation. This may allow detection of areas that have higher propensity to progress to carcinomas even before there is a clinically apparent lesion. As this was an exploratory study, further studies are required in which larger areas in the oral cavity may be probed and association of field changes detected with subsequent progression to malignancy established.

ACKNOWLEDGMENTS

The authors would like to acknowledge all the participants in the study. The work was carried out under DBT project BT/PRI11282/MED/32/83/2008, entitled “Development of in vivo laser Raman spectroscopy methods for diagnosis of

10970347, 2017, 11, Downloaded from https://onlinelibrary.wiley.com/doi/10.1002/hed.24884 by Sichuan University, Wiley Online Library on [27/10/2023]. See the Terms and Conditions (https://onlinelibrary.wiley.com/terms-and-conditions) on Wiley Online Library for rules of use; OA articles are governed by the applicable Creative Commons License

oral precancerous and cancerous conditions," Department of Biotechnology, Government of India.

REFERENCES

[1] Ferlay J, Soerjomataram I, Dikshit R, et al. Cancer incidence and mortality worldwide: sources, methods and major patterns in GLOBOCAN 2012. *Int J Cancer*. 2015;136(5):E359-E386.

[2] Dikshit R, Gupta PC, Ramasundarahettige C, et al. Cancer mortality in India: a nationally representative survey. *Lancet*. 2012; 379(9828):1807-1816.

[3] Hashibe M, Brennan P, Benhamou S, et al. Alcohol drinking in never users of tobacco, cigarette smoking in never drinkers, and the risk of head and neck cancer: pooled analysis in the International Head and Neck Cancer Epidemiology Consortium. *J Natl Cancer Inst*. 2007;99(10):777-789.

[4] Boffetta P, Hecht S, Gray N, Gupta P, Straif K. Smokeless tobacco and cancer. *Lancet Oncol*. 2008;9(7):667-675.

[5] Slaughter DP, Southwick HW, Smejkal W. Field cancerization in oral stratified squamous epithelium; clinical implications of multicentric origin. *Cancer*. 1953;6(5):963-968.

[6] van der Waal I, de Bree R. Second primary tumours in oral cancer. *Oral Oncol*. 2010;46(6):426-428.

[7] Raman CV, Krishnan KS. A new type of secondary radiation. *Nature*. 1928;121(3048):501-502.

[8] Ellis DI, Cowcher DP, Ashton L, O'Hagan S, Goodacre R. Illuminating disease and enlightening biomedicine: Raman spectroscopy as a diagnostic tool. *Analyst*. 2013;138(14):3871-3884.

[9] Hanlon EB, Manoharan R, Koo TW, et al. Prospects for in vivo Raman spectroscopy. *Phys Med Biol*. 2000;45(2):R1-R59.

[10] Fenn MB, Xanthopoulos P, Pyrgiotakis G, Grobmyer SR, Pardalos PM, Hench LL. Raman spectroscopy for clinical oncology. *Adv. Optic. Tech.* 2011;Abstract 213783.

[11] Kong K, Kendall C, Stone N, Notingher I. Raman spectroscopy for medical diagnostics—from in-vitro biofluid assays to in-vivo cancer detection. *Adv Drug Deliv Rev*. 2015;89:121-134.

[12] Austin LA, Osseiran S, Evans CL. Raman technologies in cancer diagnostics. *Analyst*. 2016;141(2):476-503.

[13] Bakker Schut T, Witjes M, Sterenborg H, et al. In vivo detection of dysplastic tissue by Raman spectroscopy. *Anal Chem*. 2000; 72(24):6010-6018.

[14] Oliveira AP, Bitar RA, Silveira L, Zângaro RA, Martin AA. Near-infrared Raman spectroscopy for oral carcinoma diagnosis. *Photomed Laser Surg*. 2006;24(3):348-353.

[15] Venkatakrishna K, Kurien J, Pai KM, et al. Optical pathology of oral tissue: a Raman spectroscopy diagnostic method. *Curr Sci*. 2001;80(5):665-669.

[16] Malini R, Venkatakrishna K, Kurien J, et al. Discrimination of normal, inflammatory, premalignant, and malignant oral tissue: a Raman spectroscopy study. *Biopolymers*. 2006;81(3):179-193.

[17] Shyam Sunder N, Rao NN, Kartha VB, Ullas G, Kurien J. Laser raman spectroscopy: a novel diagnostic tool for oral cancer. *J Orofac Sci*. 2011;3(2):15-19.

[18] Kho KW, Malini O, Shen ZX, Soo KC. Surface enhanced Raman spectroscopic (SERS) study of saliva in the early detection of oral cancer. Presented at the 2013 IEEE 4th International Conference on Photonics. 2005:84-91.

[19] Rekha P, Aruna P, Daniel A, et al. Raman spectroscopic characterization of blood plasma of oral cancer. Presented at the 2013 IEEE 4th International Conference on Photonics. 2013:135-137.

[20] Sahu A, Sawant S, Mamgain H, Krishna CM. Raman spectroscopy of serum: an exploratory study for detection of oral cancers. *Analyst*. 2013;138(14):4161-4174.

[21] Sahu A, Sawant S, Talathi-Desai S, Murali Krishna C. Raman spectroscopy of serum: a study on oral cancers. *Biomed Spectrosc Imaging*. 2015;4(2):171-187.

[22] Sahu AK, Dhoot S, Singh A, et al. Oral cancer screening: serum Raman spectroscopic approach. *J Biomed Opt*. 2015;20(11): 115006.

[23] Singh SP, Deshmukh A, Chaturvedi P, Krishna CM. Raman spectroscopy in head and neck cancers: toward oncological applications. *J Cancer Res Ther*. 2012;8 Suppl 1:S126-S132.

[24] Singh SP, Deshmukh A, Chaturvedi P, Krishna CM. In vivo Raman spectroscopy for oral cancers diagnosis. Presented at the Proc. SPIE 8219, Biomedical Vibrational Spectroscopy V: Advances in Research and Industry (February 9, 2012). International Society for Optics and Photonics. 2012:Abstract 82190K.

[25] Singh SP, Deshmukh A, Chaturvedi P, Murali Krishna C. In vivo Raman spectroscopic identification of premalignant lesions in oral buccal mucosa. *J Biomed Opt*. 2012;17(10):105002.

[26] Krishna H, Majumder SK, Chaturvedi P, Gupta PK. Anatomical variability of in vivo Raman spectra of normal oral cavity and its effect on oral tissue classification. *Biomed Spectrosc Imaging*. 2013;2(3):199-217.

[27] Krishna H, Majumder SK, Chaturvedi P, Sidramesh M, Gupta PK. In vivo Raman spectroscopy for detection of oral neoplasia: a pilot clinical study. *J Biophotonics*. 2014;7(9):690-702.

[28] Sahu A, Deshmukh A, Hole AR, Chaturvedi P, Krishna CM. In vivo subsite classification and diagnosis of oral cancers using Raman spectroscopy. *J Innov Opt Health Sci*. 2016;9(5): 1650017.

[29] Guze K, Pawluk HC, Short M, et al. Pilot study: Raman spectroscopy in differentiating premalignant and malignant oral lesions from normal mucosa and benign lesions in humans. *Head Neck*. 2015;37(4):511-517.

[30] Barroso EM, Smits RW, Bakker Schut TC, et al. Discrimination between oral cancer and healthy tissue based on water content determined by Raman spectroscopy. *Anal Chem*. 2015;87(4): 2419-2426.

[31] Barroso EM, Smits RW, van Lanschot CG, et al. Water concentration analysis by Raman spectroscopy to determine the location of the tumor border in oral cancer surgery. *Cancer Res*. 2016;76 (20):5945-5953.

[32] Cals FL, Bakker Schut TC, Hardillo JA, Baatenburg de Jong RJ, Koljenović S, Puppels GJ. Investigation of the potential of Raman spectroscopy for oral cancer detection in surgical margins. *Lab Invest*. 2015;95(10):1186-1196.

[33] Singh SP, Sahu A, Deshmukh A, Chaturvedi P, Krishna CM. In vivo Raman spectroscopy of oral buccal mucosa: a study on

10970347, 2017, 11, Downloaded from https://onlinelibrary.wiley.com/doi/10.1002/hed.24884 by Sichuan University, Wiley Online Library on [27/10/2023]. See the Terms and Conditions (https://onlinelibrary.wiley.com/terms-and-conditions) on Wiley Online Library for rules of use; OA articles are governed by the applicable Creative Commons License

malignancy associated changes (MAC)/cancer field effects (CFE). *Analyst*. 2013;138(14):4175-4182.

[34] Kumar P, Bhattacharjee T, Ingle A, Maru G, Krishna CM. Raman spectroscopy of experimental oral carcinogenesis: study on sequential cancer progression in hamster buccal pouch model. *Technol Cancer Res Treat*. 2016;15(5):NP60-NP72.

[35] Kumar P, Bhattacharjee T, Pandey M, Hole A, Ingle A, Murali Krishna C. Raman spectroscopy in experimental oral carcinogenesis: investigation of abnormal changes in control tissues. *J Raman Spectrosc*. 2016;47(11):1318-1326.

[36] Ghanate AD, Kothiwale S, Singh SP, Bertrand D, Krishna CM. Comparative evaluation of spectroscopic models using different multivariate statistical tools in a multicancer scenario. *J Biomed Opt*. 2011;16(2):025003.

[37] Lieber CA, Majumder SK, Ellis DL, Billheimer DD, Mahadevan-Jansen A. In vivo nonmelanoma skin cancer diagnosis using Raman microspectroscopy. *Lasers Surg Med*. 2008;40(7):461-467.

[38] Lui H, Zhao J, McLean D, Zeng H. Real-time Raman spectroscopy for in vivo skin cancer diagnosis. *Cancer Res*. 2012;72(10):2491-2500.

[39] Sahu A, Deshmukh A, Ghanate AD, Singh SP, Chaturvedi P, Krishna CM. Raman spectroscopy of oral buccal mucosa: a study on age-related physiological changes and tobacco-related pathological changes. *Technol Cancer Res Treat*. 2012;11(6):529-541.

[40] van Oijen MG, Slootweg PJ. Oral field cancerization: carcinogen-induced independent events or micrometastatic deposits? *Cancer Epidemiol Biomarkers Prev*. 2000;9(3):249-256.

[41] Tabor MP, Brakenhoff RH, van Houten VM, et al. Persistence of genetically altered fields in head and neck cancer patients biological and clinical implications. *Clin Cancer Res*. 2001;7(6):1523-1532.

[42] Braakhuis BJ, Brakenhoff RH, Leemans CR. Treatment choice for locally advanced head and neck cancers on the basis of risk factors: biological risk factors. *Ann Oncol*. 2012;23 Suppl 10:x173-x177.

[43] Zhao J, Lui H, McLean DI, Zeng H. Integrated real-time Raman system for clinical in vivo skin analysis. *Skin Res Technol*. 2008;14(4):484-492.

How to cite this article: Malik A, Sahu A, Singh SP, et al. In vivo Raman spectroscopy–assisted early identification of potential second primary/recurrences in oral cancers: An exploratory study. *Head & Neck*. 2017;39:2216–2223. https://doi.org/10.1002/hed.24884

10970347, 2017, 11, Downloaded from https://onlinelibrary.wiley.com/doi/10.1002/hed.24884 by Sichuan University, Wiley Online Library on [27/10/2023]. See the Terms and Conditions (https://onlinelibrary.wiley.com/terms-and-conditions) on Wiley Online Library for rules of use; OA articles are governed by the applicable Creative Commons License
