# Supplementary material for: The accuracy of Fiber-Optic Raman Spectroscopy in the detection and diagnosis of head and neck neoplasm in vivo: a systematic review and meta-analysis
Source: PeerJ. 2023 Dec 11;11:e16536. doi: 10.7717/peerj.16536 (PMC10720414; doi:10.7717/peerj.16536)
Supplement: Data S1 [file peerj-11-16536-s001.zip › article/krishna2013.pdf]

Early View publication on
www.wileyonlinelibrary.com
(issue and page numbers not yet assigned;
citable using Digital Object Identifier – **DOI**)

J. Biophotonics 1–13 (2013) / **DOI** 10.1002/jbio.201300030

Journal of
BIOPHOTONICS

FULL ARTICLE

In vivo Raman spectroscopy for detection of oral neoplasia: A pilot clinical study

Hemant Krishna[1], *Shovan Kumar Majumder*[*,1], *Pankaj Chaturvedi*[2], *Muttagi Sidramesh*[2], *and Pradeep Kumar Gupta*[1]

[1] Laser Biomedical Applications and Instrumentation Division, R & D Block-D, Raja Ramanna Centre for Advanced Technology, Indore-452013, India
[2] Department of Head and Neck Surgery, Tata Memorial Hospital, Mumbai-400012, India

Received 20 February 2013, revised 10 June 2013, accepted 11 June 2013
Published online 4 July 2013

Key words: *In vivo* Raman spectroscopy, oral lesions, probability based non-linear diagnostic algorithm, multi-class classification, maximum representation and discrimination feature (MRDF) and sparse multinomial logistic regression (SMLR)

We report a pilot study carried out to evaluate the applicability of *in vivo* Raman spectroscopy for differential diagnosis of malignant and potentially malignant lesions of human oral cavity in a clinical setting. The study involved 28 healthy volunteers and 171 patients having various lesions of oral cavity. The Raman spectra, measured from multiple sites of normal oral mucosa and of lesions belonging to three histopathological categories, viz. oral squamous cell carcinoma (OSCC), oral submucous fibrosis (OSMF) and leukoplakia (OLK), were subjected to a probability based multivariate statistical algorithm capable of direct multi-class classification. With respect to histology as the gold standard, the diagnostic algorithm was found to provide an accuracy of 85%, 89%, 85% and 82% in classifying the oral tissue spectra into the four tissue categories based on leave-one-subject-out cross validation. When employed for binary classification, the algorithm resulted in a sensitivity and specificity of 94% in discriminating normal from the rest of the abnormal spectra of OSCC, OSMF and OLK tissue sites pooled together.

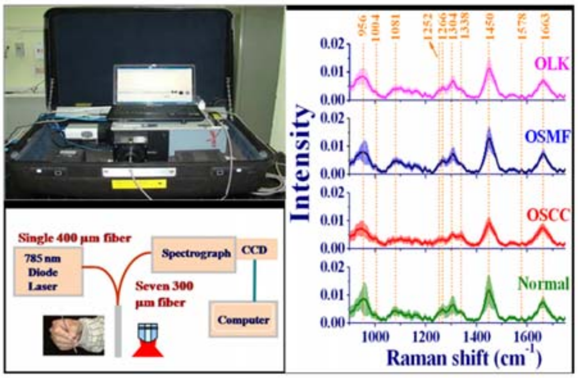

1. Introduction

Oral cancer is a major health problem in India and other South-Asian countries [1–2]. India tops in the prevalence of oral cancer in the world, and use of tobacco is known to account for this large incidence of oral cancer [3]. More than 34% of Indians (48% males and 20% females) above 15 either smoke or use tobacco in any form and nearly 0.9 million tobacco-related deaths occur in India annually as com-

* Corresponding author: e-mail: shkm@rrcat.gov.in, shovan.k.majumder@gmail.com, Phone: 91-731-2488437, Fax: 91-731-2488425

© 2013 by WILEY-VCH Verlag GmbH & Co. KGaA, Weinheim

pared to 5.5 million worldwide [4]. Although the oral cavity is easily accessible to inspection, patients with oral cancer most often present themselves at an advanced stage when treatment is less successful thereby leading to high morbidity and mortality [5–6]. Early detection remains the best way to ensure patient survival and quality of life [5–7].

The current gold standard for clinical diagnosis of oral lesions is biopsy and subsequent histopathologic correlation [7]. The process is both invasive and time-consuming. A real-time diagnostic method to enable non-invasive monitoring of oral cavity in individuals with suspicious oral lesions is thus an urgent current need. It is now well recognized that techniques based on optical spectroscopy can play a very important role towards this end. While fluorescence spectroscopy [8–12], diffuse reflectance spectroscopy [13–14], or a combination of these [15–17], has provided very encouraging results for *in vivo* applications, the use of Raman spectroscopy was primarily limited to either *ex-vivo* studies [18–24] or *in vivo* studies on animal models [25–26]. Perhaps the necessity of long data collection time owing to the weaker Raman signals precluded its use for *in vivo* studies on human oral cavity. With continued improvements in detectors and spectroscopic systems, it has now become possible to acquire good quality tissue Raman spectrum in a clinically acceptable data collection time (<5 sec) [27] and publications reporting *in vivo* studies have just started to appear [28–31]. The technique is ideal for *in vivo* diagnosis, as it is nondestructive, does not require external dyes, and can be applied via fiber-based instrumentation to provide rapid, *in situ*, objective and near real-time tissue evaluation with a high degree of accuracy.

While Guze et al. [28] and Bergholt et al. [29] were the first to apply Raman spectroscopy *in vivo* on human oral cavity, the common objective of both these studies was to characterize the normal human oral cavity in healthy volunteers using the *in vivo* Raman spectra acquired in higher wave number (1800–3000 cm^{-1}) region or conventional fingerprint region (800–1800 cm^{-1}) region. The first *in vivo* application of Raman spectrocopy for differential diagnosis of oral lesions was by Singh et al. [30] who used a commercial Raman spectrometer to measure *in vivo* Raman spectra from patients already identified of having maliganancy of oral buccal mucosa and showed that Raman spectroscopy in combination with a PCA-LDA based diagnostic algorithm could discriminate malignant from the uninvolved nomal tissue sites as well as the premalignat lesions appearing in the contralateral buccal mucosa of the same set of patients with an accuracy of up to 87%. In a concurrent study [31] they investigated the influence of aging related physiological changes on the differential detection of malignant, premalignant and normal buccal mucosa and showed that though there were aging related changes in the Raman spectra but that did not have any influence on the classification of lesions [31]. While these existing reports demonstrate the potential of Raman spectroscopy for *in vivo* discrimination of lesions in only a part of the oral cavity (i.e. buccal mucosa), there is no published report, thus far, of a full-scale clinical study on the comprehensive evaluation of the efficacy of *in vivo* Raman spectroscopy for differential detection of oral lesions in the whole of the oral cavity. This is important because availability of a spectral database of the whole oral cavity may facilitate rapid screening of a large population (e.g. in a community setting) for any abnormality in the oral cavity with the help of an appropriately trained diagnostic algorithm. Another limitation of the earlier studies [30] is that the patient population included therein was not a true representative of the tissue types interrogated, since it involved only patients already identified of having malignancy of the buccal mucosa.

We report, in this paper, the results of an *in vivo* clinical study carried out to address these issues. The study involved near-infrared (NIR) Raman spectroscopy of normal oral tissues and oral lesions from across the whole of the oral cavity. The lesions belonged to one of the three pathologic categories, oral squamous cell carcinoma (OSCC), oral leukoplakia (OLK) and oral submucosal fibrosis (OSMF). None of the patients had multiple types of lesions present simultaneously in their oral cavity. The normal oral tissue sites investigated were from the oral cavity of healthy volunteers without the history of any disease of the oral cavity. NIR Raman spectra measured from multiple sites across a given lesion were correlated with the corresponding histopathologic diagnosis. A probability based multivariate statistical algorithm capable of simultaneously classifying spectral data into multiple different classes (i.e. direct multiclass classification) was found to distinguish neoplastic from normal oral tissue sites, based on their Raman spectra, with a predictive accuracy of more than 90% with respect to histology as the gold standard.

2. Materials and methods

2.1 Study design

The *in vivo* study was conducted at the Tata Memorial Hospital (TMH), Mumbai with the approval of the TMH Ethical Committee. All the patients undergoing routine medical examination of the oral cavity at the Out Patient Department (OPD) of TMH were recruited for this *in vivo* study. The patients were recruited regardless of gender or race. The eligibility

© 2013 by WILEY-VCH Verlag GmbH & Co. KGaA, Weinheim

of each patient was determined by the participating doctor based on the medical condition of the patient such that patient care was not compromised.

The study involved 28 healthy volunteers with no history of the disease of the oral cavity and 171 patients enrolled for medical examination of the oral cavity at TMH. Informed consent was obtained from each patient as well as the healthy volunteers who participated in this study. Age, sex, and details of smoking habit (if any) were also recorded for all subjects included in the study. The age variations for OSCC, OSMF, OLK and healthy volunteers were 35 ± 11, 51 ± 13, 53 ± 14 and 44 ± 10 years respectively. The overall ratio of male to female population was ~6 : 1. As far as tobacco habits are concerned, 98% of the patients and 71% of the normal volunteers had habits of either smoking or chewing tobacco, and the remaining candidates had no history of tobacco consumption.

The patients included in this study had no history of malignancy or dysplasia, and were suspected by the examining physician of having either cancer or leukoplakia or submucosal fibrosis on visual examination of the oral cavity. Patients having gone through any prior treatment like surgery, chemotherapy or radio therapy for earlier cancers or with recurrences were excluded from the study. Biopsies were taken subsequent to acquisition of spectra from the oral cavity sites suspected of being malignant or potentially malignant. However, as per the terms of the approval from the Ethical Committee of the hospital, no biopsies were available from the investigated sites of the patients with oral submucous fibrosis (OSMF) and the diagnosis of this condition was based on clinical findings only. Similarly, no biopsies were allowed from the tissue sites of healthy volunteers. The biopsy samples were fixed in formalin and were examined later by an experienced pathologist who was blinded to the results of the optical spectra. Histopathology was taken as the "gold standard". All the Raman spectra from across the whole of the oral cavity were categorized in accordance to their histological identities and grouped into OSCC, OLK, OSMF, or normal oral tissue.

2.2 Instrumentation

In vivo Raman spectra were measured using a compact and portable Raman spectroscopic system assembled in-house. A photograph and a schematic of the system are shown in Figures 1a and b respectively. The portable clinical system has all its sub-systems (diode laser, fiber-optic probe, spectrograph and CCD camera) accommodated into a 32″ suitacase. A 785 nm diode laser (Crysta Laser, Reno, NV) is used to deliver excitation light (~80 mW) to the target tissue using a fiber-optic probe (Visionex Inc., Warner Robins, GA) consisting of a central 400-μm-core-diameter fused-silica excitation fiber surrounded by seven 300-μm fused-silica beam-steered collection fibers. While the distal ends of the collection fibers have in-line notch filters for rejection of the excitation light, a band-pass filter sitting at the tip of the excitation fiber blocks the signals generated in the fiber itself and allows only the laser line to pass through. The collection fibers are aligned linearly and imaged on to a 200 μm entrance slit of an imaging spectrograph (Andor Shamrock SR-303i, Belfast, Northern Ireland) coupled with a thermoelectrically cooled (−70 °C), back-illuminated, deep-depletion charge-coupled-device (CCD) camera (Andor DU420A-BR-DD, Belfast, Northern Ireland). The system is able to acquire good quality tissue Raman spectra with signal to noise ratio 50 : 1 for an integration time of less than 5 seconds. The overall resolution of the system is ~20 cm^{-1}.

2.3 Clinical measurements

All the spectral measurements were performed by the participating head and neck surgeon using a protocol which was maintained for all individuals in this study. Briefly, prior to recording spectra from an individual, the fiber-optic probe was disinfected with CIDEX (Johnson and Johnson, India), washed with PBS and cleaned dry with a piece of sterilized cotton. The mucosal surface was wiped with sterile gauge to remove any saliva, blood or betel quid incrustations accumulated at the tissue surface. The probe tip was also wiped dry between consecutive measurements from different tissue sites in an individual. For recording the *in vivo* Raman spectra, the tip of the fiber-optic probe was placed in gentle contact with the tissue surface and it was ensured that no subject complained of the probe being painful. The overhead room lights in the OPD room were turned off temporarily during spectral acquisition to minimize the contribution of the ambient light in the acquired spectra.

The good quality (signal-to-noise ratio ≥ 10) *in vivo* Raman spectra were recorded from a total of 515 tissue sites of 171 patients with an integration time of 5 s. Out of these, 94 sites were identified as OSMF by the examining doctor and from these no biopsies were taken. Of the remaining tissue sites, 316 were histopathologically characterized as OSCC, and 105 as OLK. Spectra were also recorded from 287 sites from healthy squamous tissue of 28 normal volunteers. The details of the histopathological distribution of the tissue sites included in the study are summarized in the Table 1. Each site was treated separately and classified via the diagnostic algorithm developed.

© 2013 by WILEY-VCH Verlag GmbH & Co. KGaA, Weinheim

Table 1 Histopathological distribution of tissue sites included in the clinical pilot study.

No. of Individuals	No. of Sites	Histopathological Diagnosis
113	316	Oral Squamous Cell Carcinoma (OSCC)
25	94	Oral Submucous Fibrosis (OSMF)
33	105	Oral Leukoplakia (OLK)
28	287	Normal

2.4 Pre-processing of data

Prior to Raman spectral measurements from a subject, the wavenumber axis was calibrated with the excitation laser line, acetaminophen, and naphthalene standards. For each measured Raman spectrum, the signal from the CCD was binned along the vertical axis to create a single spectrum per measurement. Prior to any signal processing, the spectrum was truncated to only include the region from about (900 cm^{-1}) to 1750 cm^{-1}. A sequence of pre-processing steps was then executed on this binned, truncated spectrum following the procedure described by Motz et al. [32]. First, the spectrum was corrected for the system spectral response by using a NIST traceable calibration lamp (LS-1, Ocean optics, Inc., Dunedin, FL) after removal of the dark signal. The next step was to remove the artifacts introduced in the measured tissue spectrum as a result of laser-induced artifacts generated in the fiber-optic probe. This was done by recording the spectrum of backscattered light from a roughened aluminium block and then iteratively subtracting this spectrum scaled by a range of different intensities till the optimal ratio for background removal is reached. The spectrum that results in the lowest standard deviation of the residual between the data and the model fit was used for fiber background removal. Following removal of fiber artifacts, the spectrum was noise smoothened using a second-order Savitzky–Golay filter and then background subtracted using the range-independent background subtraction algorithm (RIA) [33] to retrieve the weak tissue Raman spectrum. The underlying basis of RIA is iterative smoothing of the measured raw Raman spectrum. The method uses a model based on modified iterative smoothing of the measured Raman spectrum in such a manner that the high-frequency Raman peaks are gradually eliminated finally leaving the underlying broad baseline which can be subtracted from the raw spectrum to yield the true Raman signal. Each background-subtracted tissue Raman spectrum was normalized with respect to its mean spectral intensity across all the Raman bands.

2.5 Data analysis

A probability based multivariate statistical algorithm capable of simultaneously classifying spectral data into multiple (more than two) different classes was developed to analyze the diagnostic content of the spectra measured from the different oral tissue sites. The algorithm development consisted of two steps: (i) extraction of diagnostically relevant spectral information through maximum representation and discrimination feature (MRDF) and (ii) probabilistic classification via sparse multinomial logistic regression (SMLR). These techniques have been described in detail elsewhere [34–36]. In brief, MRDF [35] is a feature extraction procedure that aims to find a set of nonlinear transformations on the input data that optimally discriminate between the different classes in a reduced dimensionality space. SMLR [36] separates a set of labeled input data into its constituent classes by predicting the posterior probabilities of their class membership.

For each of the classification tasks, the input spectral data were first normalized using to the scheme described earlier [34, 35] and then subjected to feature extraction by the MRDF prior to classification by the SMLR. The MRDF dimensionally reduced the high-dimensional ($D = 243$) spectral data and resulted in a set of few non-linear output features that contained the maximum class discriminatory information. The optimal number of these features was decided by employing a cross-validation procedure with varying number of output nonlinear features and minimizing the misclassification error. It was found that optimal number of features varied from 15 to 20 depending on the classification task. This optimal number of output nonlinear features was used as input to the SMLR for subsequent classification. The full range of spectral data was never directly fed to the SMLR.

In order to have a statistically unbiased estimate of the generalized classification ability of the MRDF-SMLR algorithm (i.e. how correctly the algorithm can classify previously unseen data), it was necessary to ensure that the validation set data should neither contain any of the pieces of the training set data with which the algorithm was trained and optimized, nor any part of the data of a subject whose

© 2013 by WILEY-VCH Verlag GmbH & Co. KGaA, Weinheim

remaining part remained in the training set. The generalized classification ability of the algorithm was evaluated, for each classification task, by adopting the method of leave-one-subject-out cross validation. In this method, for N total number subjects (patients and healthy volunteer) for a given classification task, spectra of N-1 of them were used to train the algorithm and the set of spectra of the remaining subject was left excluded (and therefore not used by the algorithm during training) as the validation set. This was repeated N times (until the spectra of all the withheld subjects were classified) each time excluding a different subject for the purpose of validation and retraining the algorithm using spectra of the rest of the subjects. Since the training set data remained completely independent of the test data in each of the N loops (as the set of spectra from a subject was never a part of both the training and the validation sets simultaneously), the validation was statistically unbiased. Further, since the spectral data of each patient in the validation set (irrespective of the anatomical location) had proper representation in the training set which contained the full set of spectral data from all possible anatomical locations from across the oral cavity of patients and healthy volunteers, the information necessary for classifying the spectra of the test subject was appropriately learnt by the algorithm (from the training set data) and thus the prediction made by it was balanced and unbiased.

For a given classification task, MRDF-SMLR algorithm computed the posterior probabilities of the different interrogated tissue sites of belonging to various tissue classes. Subsequently, the class was assigned to a given tissue spectrum whose posterior probability of belonging to that particular class was the highest according to the Bayesian rule of classification [36]. The predictive accuracies of the diagnostic algorithm used for different classification tasks were calculated with respect to histopathology as the gold standard of reference.

Comparison of the algorithms' performance for the different classification tasks was challenging in the context of multiclass classification since a generic analytic treatment of the problem was not available and appropriate extension of binary formulations was required for defining a suitable multi-class performance measure. Although a variety of such measures like volume under the surface (VUS) [37], global performance Index [38], Matthews correlation coefficient (MCC) [39], confusion entropy (CEN) [40] etc. exist in literature, the Hand and Till measure (HTM) [41] is the most widely used and well known measure of performance for multi-class classification as it has the appealing property of being objective, requiring no subjective input from the user. HTM is based on multi-class Receiver Operating Characteristic (ROC) approximation [41] and extends the Area Under the Curve (AUC), one of the most popular measures for binary classifiers to multiclass tasks. Given 'c' number of pathology classes, overall performance of a multi-class diagnostic algorithm is taken as the average of pairwise area under the ROC curves between $c\,(c-1)/2$ pairs of classes and given by Hand and Till measure (HTM) [41] as:

$$\mathrm{HTM} = \frac{2}{c(c-1)} \sum_{i<j} \mathrm{AUC}\,(i,j) \tag{1}$$

Where, AUC is the area under the two-class ROC curve involving classes 'i' and 'j'. The summation is calculated over all pairs of distinct classes, irrespective of order. As is the case for two-class, the closer the HTM equals to 1, the more accurate the corresponding diagnostic algorithm is.

Prior to applying the MRDF-SMLR diagnostic algorithm, which mathematically transforms the spectra into a new feature-space thereby making it impossible to relate the diagnostically relevant features with the original wavenumber space, an initial analysis was performed to qualitatively determine the statistically significant differences between the tissue spectra. Though these results were not used in the diagnostic algorithms, they allowed exploration of the responsible mechanisms for differential diagnosis. To identify the region of the spectral differences between the pathologic and normal tissue spectra a statistical analysis was performed based on standard error (SE) confidence intervals. The var-

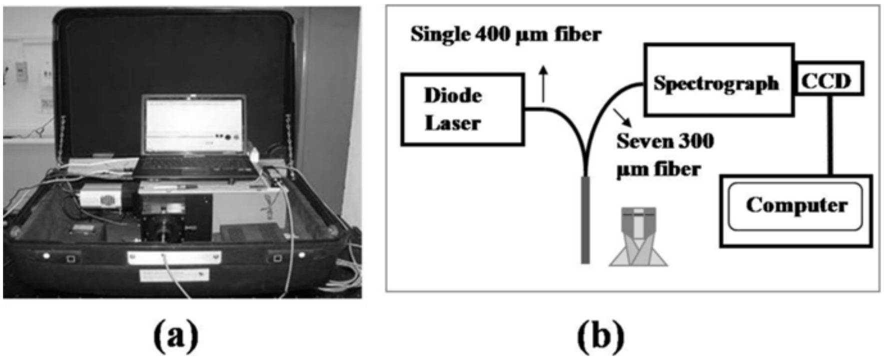

Figure 1 (**a**) A photograph and (**b**) a schematic of the portable clinical Raman spectroscopy system for *in vivo* Raman measurements.

© 2013 by WILEY-VCH Verlag GmbH & Co. KGaA, Weinheim

iance of the intensity at each wavenumber was first calculated for tissue spectra belonging to each pathology class. The composite variance (σ^2) of the spectra at each wavenumber was calculated as:

$$\sigma^2_{\text{lesions}}(\lambda) = \frac{\sum_i \sigma(\lambda)_i^2 \, (\mathrm{d}f)_i}{\sum_i (\mathrm{d}f)_i} \tag{2}$$

where, σ^2 is the variance of the intensity at each wavenumber λ for each lesion type 'i' and df corresponds to the degrees of freedom for each pathology (= number of tissue specimens-1). The SE of the pooled lesion spectra and normal oral tissue spectra was then calculated at each wavenumber as:

$$\mathrm{SE}(\lambda) = \sqrt{\frac{\sigma^2_{\text{normal}}(\lambda)}{n_{\text{normal}}} + \frac{\sigma^2_{\text{lesions}}(\lambda)}{n_{\text{lesions}}}} \tag{3}$$

where, n is the number of tissue spectra included in the particular tissue type. The SE was then multiplied by the appropriate t-values based on the total degrees of freedom and a predefined confidence level to produce a confidence interval. Difference spectra for the lesion with respect to the normal were overlaid on these confidence intervals to qualitatively identify statistically significant spectral differences.

3. Results

Figure 2a shows the average normalized Raman spectra for OSCC (316), OLK (105), OSMF (94), and normal squamous tissue sites (287) of the oral cavity, with the error bars representing the spectral standard deviations. From the figures it is evident that the variation in the measured spectral intensity is comparable for all the tissue types investigated. The percentage variation ($\sigma/\bar{x}$) in the spectral intensities from the different measurement sites was observed to lie in the range of ~15–35% over the respective number of tissue sites included in the four histopathological categories for all the measured spectra. Here, $\bar{x}$ is the mean intensity value from different measurement sites of one category and σ is one standard deviation.

For comparison of spectral differences among the different tissue types, the average Raman spectra are plotted without error bars in Figure 2b. The spectra are similar to those reported in Raman spectroscopic studies of human oral cavity [18–26] by others, with major peaks located at ~956, 1004, 1081, 1252, 1266, 1304, 1338, 1450, and 1663 cm^{-1} corresponding to various Raman-active biomolecules, notably collagen, elastin, keratin, lipids, and minerals known to be present in oral tissues [18–26, 29–31, 42–43]. Although the peak shapes and locations are consistent across all the pathology classes, subtle but significant differences are observed in peak intensities between the different pathology categories indicating biochemical differences inherent in the tissue types of different pathologies.

Figure 3 shows the mean difference spectra obtained by subtracting the mean tissue spectrum of each lesion from the mean spectrum of normal oral tissue. The grey bands show the confidence intervals calculated by multiplying SE with a t-value corresponding to 90% confidence intervals and the degrees of freedom equal to number of spectral measurements (corresponding to the pathology minus the number of pathologic categories). A number of significantly different Raman bands are observed for each lesion with respect to normal. The portion of the difference spectra outside the confidence interval represents the region of statistically significant spectral differences ($p < 0.1$) [44].

Table 2 shows the four-class (normal, OSCC, OSMF and OLK) classification results in the form of a confusion matrix displaying comparisons of the pathological diagnosis with that of the MRDF-

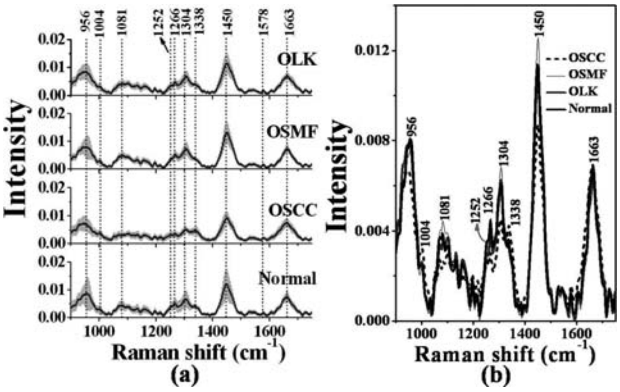

Figure 2 Mean, normalized Raman spectra of OSCC ($n = 316$), OSMF ($n = 94$), OLK ($n = 105$), and normal ($n = 287$) oral tissue sites. The error bars (gray) represent ±1 standard deviation.

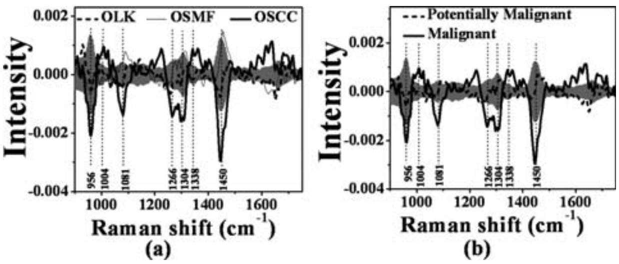

Figure 3 Mean difference spectra showing statistical differences between pathologic (OSCC: bold line, OSMF: fine line, and OLK: dashed line) and normal oral tissue spectra. Gray bands indicate the 90% confidence intervals of the difference determined by standard error confidence intervals.

© 2013 by WILEY-VCH Verlag GmbH & Co. KGaA, Weinheim

Table 2 Confusion matrix displaying classification of the Raman spectra of oral tissue sites into four classes using the MRDF-SMLR based diagnostic algorithm. The classification results represent leave-one-subject-out cross validation.

Pathology Diagnosis	Raman Diagnosis			
	Normal	OSCC	OSMF	OLK
Normal ($n = 287$)	85%	8%	4%	3%
OSCC ($n = 316$)	8%	89%	0%	3%
OSMF ($n = 94$)	14%	0%	85%	1%
OLK ($n = 105$)	13%	5%	0%	82%

'n' represents size of the spectral data in the corresponding tissue category.

SMLR based spectroscopic diagnostic algorithm. The classification results were obtained based on leave-one-subject-out cross validation of the MRDF-SMLR algorithm on the entire data set. One can see that the algorithm provided an overall classification accuracy of 86% (690 out of 802). It proved most adept at classifying OSCC tissues with a classification accuracy of 89%, though it fared worse in classifying other tissue types, and errors were spread among the various classes. Normal tissue spectra were correctly classified in 85% of the sites, while OLK and OSMF spectra were classified correctly in 85% and 82% of the sites.

Table 3 shows the confusion matrix depicting the results of classification with the MRDF-SMLR algorithm validated, in leave-one-subject-out cross-validation fashion, on the whole set of spectra separated into three categories (instead of four), normal, malignant (OSCC) and potentially malignant where the OSMF and OLK spectra were put together to form the last category. Although the overall discrimination accuracy is seen to be reduced marginally by 3% (from 86% to 83%), the accuracy with which the OSMF and OLK spectra together (belonging to the potentially malignant category) can be discriminated is found to improve to 88% with 176 out of 199 spectra of this category being classified correctly. However, the algorithm fared worse in classifying the normal squamous tissue spectra where the classification accuracy is found to be only 77%.

Table 4 shows the sensitivities and specificities yielded by the algorithm in discriminating spectra of

Table 3 Confusion matrix displaying results of classification of the Raman spectra of oral tissue sites into three classes using the MRDF-SMLR based diagnostic algorithm. Here the spectra belonging to OSCC are referred to as "Malignant" and those belonging to OSMF and OLK pooled together are referred to as "Potentially Malignant". The classification results represent leave-one-subject-out cross validation.

Pathology Diagnosis	Raman Diagnosis		
	Normal	Potentially Malignant (OSMF + OLK)	Malignant (OSCC)
Normal ($n = 287$)	77%	13%	10%
Potentially Malignant ($n = 199$)	9%	88%	3%
Malignant ($n = 316$)	11%	5%	84%

'n' represents size of the spectral data in the corresponding tissue category.

Table 4 The classification results of the Raman spectra of oral tissue sites into two classes using the MRDF-SMLR based diagnostic algorithm. Here the spectra belonging to OSCC are referred to as "Malignant", those belonging to OSMF and OLK pooled together are referred to as "Potentially Malignant", and the spectra belonging to OSCC, OSMF and OLK pooled together are referred to as "Abnormal". The classification results represent leave-one-subject-out cross validation.

Pathology Diagnosis	Raman Diagnosis	
	Sensitivity	Specificity
Normal ($n = 287$) vs. Malignant ($n = 316$)	96%	99%
Normal ($n = 287$) vs. Potentially Malignant ($n = 199$)	99%	98%
Normal ($n = 287$) vs. Abnormal ($n = 515$)	94%	94%

'n' represents size of the spectral data in the corresponding tissue category.

© 2013 by WILEY-VCH Verlag GmbH & Co. KGaA, Weinheim

Journal of
BIOPHOTONICS

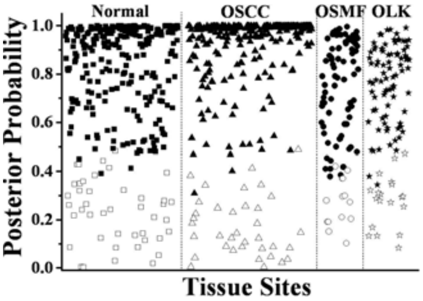

Figure 4 Posterior probabilities for being classified as normal, OSCC, OSMF, and OLK for the Raman spectra of the oral tissue sites interrogated. The closed symbols represent probabilities of correct class-membership and the open symbols represent the probabilities for the misclassified tissue sites.

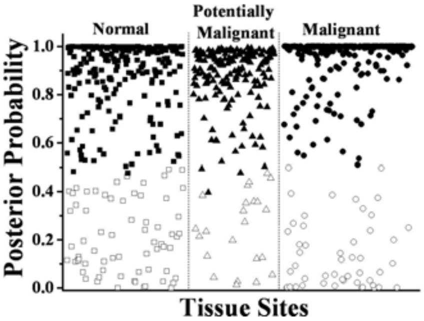

Figure 5 Posterior probabilities for being classified as normal, potentially malignant (OSMF & OLK grouped together), and malignant (OSCC) for the Raman spectra of the oral tissue sites interrogated. The closed symbols represent probabilities of correct class-membership and the open symbols represent the probabilities for the misclassified tissue sites.

the healthy volunteers (i.e. the normal category) from three separate categories: malignant, potentially malignant and a category termed "abnormal" which includes spectra of tissue sites belonging to OSCC, OSMF and OLK all pooled together. One may note that the algorithm could discriminate the spectra of normal from that of all three categories with mean sensitivity and specificity of ~96% and ~97% respectively. Further it is also noted that the performance of the algorithm is relatively superior for binary classification as compared to the 3-class and 4-class classification.

In addition to assigning class labels, the diagnostic algorithm also yielded posterior probabilities of the measured tissue sites belonging to each class of oral tissue. Figures 4–6 illustrate these posterior probabilities computed by the algorithm for the measured tissue spectra of each tissue class of belonging to that particular class for three different classification modes: 4-class, 3-class and binary. While the closed symbols in the figures represent probabilities of correct class-membership, the open symbols denote the probabilities for the misclassified tissue sites. One may note that while for the two-class case, the probability of belonging to the correct class is always greater than 50%, for classification involving more than two classes the corresponding probability of correct class-membership has fallen below 50%, for a few spectra, despite its being the highest.

The ROC analyses of the classification results provided a quantitative evaluation of the overall performance of the diagnostic algorithm for different classification scenarios. Table 5 lists the AUC value for binary classification and the HTM values obtained for 3- and 4-class classifications. While the estimated HTM values of the algorithm for the 4-class and 3-class classifications are 0.95 and 0.93 respectively, for the binary classifications the AUC value is found to be 0.99 for all three cases. It is important to mention here that the HTM (as well as the AUC) value is a quantitative measure of the gross performance of an algorithm and the HTM (and the AUC) for an ideal diagnostic algorithm will have a value of 1.

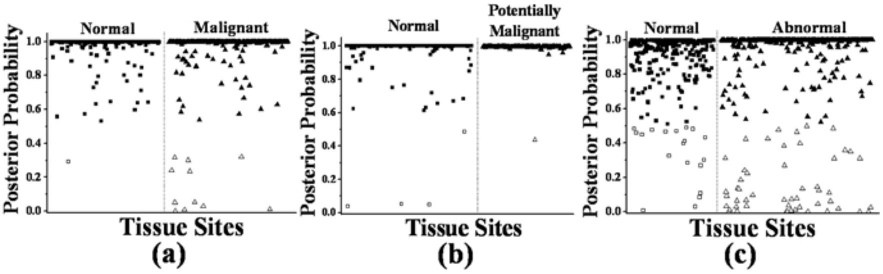

Figure 6 Posterior probabilities for being classified as (**a**) normal and malignant (OSCC), (**b**) normal and potentially malignant (OSMF and OLK grouped together), and (**c**) normal and abnormal (OSCC, OSMF and OLK grouped together) for the Raman spectra of the oral tissue sites interrogated. The closed symbols represent probabilities of correct class-membership and the open symbols represent the probabilities for the misclassified tissue sites.

© 2013 by WILEY-VCH Verlag GmbH & Co. KGaA, Weinheim

Table 5 The results of ROC analyses for binary, three-class and four-class classifications using the MRDF-SMLR based diagnostic algorithm. While AUC corresponds to the area under the ROC curve and is a measure of performance of the algorithm for binary classification, HTM is the performance measure for multi-class classification and corresponds to the average of pair-wise area under the ROC curves between $c(c-1)/2$ pairs of classes, c being the number of classes. Here "Malignant" refers to the spectra belonging to OSCC, "Potentially Malignant" to those belonging to OSMF and OLK pooled together, and "Abnormal" to the spectra belonging to OSCC, OSMF and OLK pooled together.

Binary Classification			Multiclass Classification	
AUC	AUC	AUC	HTM	HTM
Normal vs. OSCC	Normal vs. Potentially Malignant	Normal vs. Abnormal	Normal vs. Malignant vs. Potentially Malignant	Normal vs. OSCC vs. OSMF vs. OPK
0.99	0.99	0.98	0.93	0.95

4. Discussions

The primary basis for Raman spectroscopic detection is the array of biochemical changes that take place as tissue undergoes neoplastic transformations. A close examination of the observed differences in the Raman bands (Figure 3) appearing in the spectra of the different oral tissue types reveal that the pathologic spectra can largely be separated based on protein and lipid related Raman features. For example, the increased intensities of the characteristic protein Raman peaks (1004 cm^{-1}, and 1338 cm^{-1}) in the OSCC spectra as compared to the spectra of other categories can be correlated to an over-expression of protein, believed to take place in OSCC [18–19, 21–26, 29–31, 42–43]. On the other hand, a decreased concentration of the phospholipids and fatty acid as tissue changes from normal to OSCC [45] is the plausible explanation for the reduced intensities of the lipid-specific bands (1081, 1266, 1304, and 1450 cm^{-1}) [19, 29, 43] observed in the Raman spectra of OSCC and likely contributor to the differences in the regions of 1057–1100, 1248–1318, 1430–1474 cm^{-1} (Figure 3). In contrast, the differences in the Raman spectra of OLK and OSMF with respect to normal are seen to be rather subtler (Figure 3).

For the purpose of diagnosis, it is more relevant to explore the significance of the aforementioned spectral differences (observed between the different oral tissue types) towards pathological classification. A critical evaluation of the diagnostic results listed in Tables 2–4 reveals the following points that are worth noting. In the four-class classification case, the OSCC spectra are classified with the highest accuracy (89%) whereas the OSMF and OLK spectra are classified correctly in 85% and 82% of the sites. This is quite expected given the observation (see Figure 3) that the spectral differences between the OSCC and the normal squamous tissue are more conspicuous as compared to that between normal and OSMF or OLK. In the three-class mode, the classification accuracy of the OSMF and OLK spectra together (belonging to the potentially malignant category) is found to improve to 88%, though for the normal and the OSCC spectra the accuracies are found to reduce to 77% and 84% respectively. In general, the overall accuracy for the three-class classification is observed to be poorer as compared to the four-class case (Tables 2 and 3). This observation is quantitatively supported by the results of the multi-class ROC analysis (listed in Table 5) which shows an HTM value of 0.95 for the 4-class as compared to 0.93 for the 3-class classification. The reason for this relatively poorer performance of the algorithm in the three-class mode is most likely due to the fact that compared with the spectral difference between normal mucosa and the individual OSCC or OLK or OSMF, the spectra of normal and pooled OSMF and OLK (potentially malignant lesions) show relatively small differences (Figure 3) thereby resulting in a larger misclassification of normal tissue sites. The situation, however, drastically improves with a substantial increase in the accuracy of discrimination of the normal oral tissue sites (Table 4) when the algorithm switches to binary classification mode. One may see that the normal oral tissue sites are consistently classified correctly with a specificity of over 94% and the improvement in specificity in going from normal vs. abnormal (OSCC, OSML and OLK put together) through normal vs. potentially malignant (OSMF and OLK) to normal vs. malignant (OSCC) classifications is only incremental. The sensitivities in all three cases of binary classification are also found to be significantly improved (as compared to the multi-class cases) with accuracies lying in the range between 94% and 99%. These observations are further supported by the two-class ROC analyses that resulted in AUC values of 0.99. The improvement in classification performance for the binary as

© 2013 by WILEY-VCH Verlag GmbH & Co. KGaA, Weinheim

compared to multi-class classification is not quite unexpected. This is because in binary classification an algorithm can carve out the appropriate decision boundary for only one of the classes, the other class is simply the complement. In contrast, the multiple class classification is intrinsically harder because here the classification algorithm has to learn to construct a greater number of separation boundaries or relations [46]. Since each of the multiple classes needs to be explicitly predicted, misclassification errors can occur in the construction of any one of the many decision boundaries. Further, the errors increase when there is significant overlap among the class members as in the present case.

It is pertinent to mention here, although our observations, prima facie, are seen to be grossly consistent with those recently reported by Singh et al. [30] (for example, in both the cases the overall accuracy of leave-one-out-cross-validation is found to be ~83% in classifying the tissue Raman spectra into malignant, pre-malignant and normal), there exist a few differences between the two studies due to which a direct comparison is not possible. For example, in their case, "normal" corresponded to the normal appearing mucosa in the oral cavity of a patient having malignant lesions at the contralateral side and "premalignant" referred to patches of lesions scattered in the same visually normal region. In contrast, we had chosen "normal", as the normal oral mucosa of healthy volunteers with no history of any oral diseases and "potentially malignant" as either oral submucosal fibrosis or leukoplakia in the oral cavity of patients who did not have any known oral malignancy. The reason underlying our choice is the fact that the normal appearing region surrounding the malignant tumor of a patient might have some subvisual malignant signatures due to the field effect of malignancy and associated biochemical changes [47] and this effect, although, expected to be more pronounced at the advanced stage of the disease, cannot be completely ruled out even at early stages [48]. In the present context, it means that spectral data from many of the uninvolved tissue sites of patients assumed to be normal might not be truly normal due to the field effect of malignancy [47]. Due to the similar reason, there is also a possibility that many of the spectra that were measured from the tissue sites of lesions sitting in the contralateral uninvolved region and were assumed to be premalignant might not be truly premalignant. In contrast, this possibility does not exist for the squamous tissue sites from healthy volunteers who have no history of any disease of oral cavity.

The second major difference lies in the method of data analysis. It is important to mention here that the nonlinear diagnostic algorithm used in our study was selected for its ability to both compress the large amount of data obtained with each Raman spectrum as well as retain only the diagnostically relevant portions of the spectra in this compression. In contrast, the algorithm used by Singh et al [30] employs principal components analysis (PCA) [49] that creates only a single model for a dataset and compresses the data in decreasing degrees of shared variance. Since the diagnostically relevant features in the Raman spectra are very small in comparison with the shared spectral content, it is imperative that these features be retained. Further, the conventional diagnostic algorithms based on PCA or linear discriminant analysis (LDA) being linear in nature cannot perform well on non-symmetric data that may have multiple clusters per class and also suffer from the limitation of extracting information from only the second order correlation in the data [34, 48, 50]. In contrast, the nonlinear MRDF-SMLR uses higher order correlations and therefore can provide improved discrimination [34] because of its built-in capability to separate classes which are not linearly separable in the original input data space.

In a clinical scenario, depending on the requirement, the emphasis may be on either screening or screening and diagnosis both. While the task of screening requires discrimination of abnormalities without going for further evaluation, the diagnosis seeks to know the pahological status of the detected abnormality. The capability of our algorithm to operate in both binary as well as multi-class classification platforms certainly adds to the utility of the technique in a clinical situation where it can be opted for either screening and/or diagnosis. Another important feature of the algorithm is that being based on a Bayesian framework, it is able to predict the posterior probability of class-membership of the investigated tissue sites. In contrast, the PCA-LDA algorithm generally results in a numeric-valued output score to represent the degree to which a given observation belongs to a particular class. There are many advantages of having probability estimates. First, the availibility of probability estimates allows one to follow a systematic approach for selecting the appropriate threshold for classification. Therefore, instead of requiring to choose the threshold in an ad-hoc manner as in the PCA-LDA algorithm, the underlying Bayesian risk model in SMLR takes into account the relative cost of misclassifying normal tissue sites into abnormal and vice-versa. Second, the probability estimates provide a more robust approach for developing an outlier detection framework than methods based on aggregating the relative rankings of outlier scores [51] yielded by PCA-LDA like algorithms. Finally, the probability estimates are useful to determine the uncertainties in outlier prediction. The availability of this quantitative information during tissue discrimination would allow the clinician to reassess those sites that are classified with higher relative uncertainty. For example, one may note in Fig-

© 2013 by WILEY-VCH Verlag GmbH & Co. KGaA, Weinheim

ures 6c that most of the normal tissue sites have been classified with a posterior probability of greater than 80%. However, a few normal tissue sites are seen to show a very low posterior probability of being classified as normal. Here, the probabilistic approach can offer an important advantage for optimizing the discrimination goals. Compared to a non-probabilistic classification scheme like PCA-LDA [18, 23, 26, 28–31] where sites having a diagnostic score below a certain threshold would be classified as normal, in the probabilistic scheme the sites showing lower probability than that for "absolute normal" may be further interrogated if the objective is to not to miss any abnormal sites, as may be required for accurate screening of the oral cavity.

It is pertinent to mention here that the numeric scores generated by the PCA-LDA algorithms can also be converted to the posterior probability estimates by using various methods (e.g. using a sigmoid function) [52]. For example, in two independent recent studies, Yongzeng Li et al. [53] and Kan Lin et al. [54] used the posterior probability estimates obtaind from the numeric scores generated by the PCA-LDA based algorithms for successfully discriminating malignant nasopharyngeal and laryngeal tissues respectively from their corresponding normal based on the measured Raman spectra. However, the current probability mapping methods are limited only to two-class problems [52]. For the multiclass classification case where the mapping needs to be from ($k-1$)-dimensional space to another ($k-1$)-dimensional space, k being the number of classes, it is not clear which functional shape should be imposed to the mapping function. In such situation, for obtaining multiclass probability estimates from the scores generated by PCA-LDA like non-probabilistic classifiers, the multiclass problem is first decomposed into a series of binary problems. A classifier is then learnt for each one of binary problems with the scores from each classifier calibrated and combined to obtain multiclass classification probabilities. In contrast, the SMLR classifier in our algorithm is based on a principled Baysian framework which directly outputs multi-class probability distributions by incorporating probabilities in the learning optimization [34, 36].

The objective of the present study was a comprehensive evaluation of the efficacy of *in vivo* Raman spectroscopy for differential detection of oral lesions in the whole of the oral cavity. The intrinsic variability of the tissue Raman spectra that might result due to various factors like variations in the anatomical locations within the oral cavity being interrogated, influence of tobacco usage, age, gender etc. were not taken into account in the present study. It should be noted that these factors might also affect the algorithm's performance, although an in-depth analysis of the variability of the tissue Raman spectra due to these factors and investigating their effect on the diagnostic efficacy of *in vivo* Raman spectroscopy has not yet been demonstrated. Detailed clinical studies, which are required to address these issues, have been undertaken by us and their results will be presented in our future communications.

It is also pertinent to mention here that the diagnostic algorithms developed in this study were based on spectra from a limited number of patients assumed to be representative of the entire patient population. The patient selection criteria as well as the limited number of spectra in each pathologic category might influence the classification results obtained in this study. Therefore, further clinical studies in a larger patient population, which are already in progress, will be used to validate the classification estimates presented here.

5. Conclusions

To conclude, a pilot study was carried out to investigate the clinical applicability of *in vivo* Raman spectroscopy for discriminating normal from neoplastic lesions of human oral cavity. The *in vivo* Raman spectra were measured from multiple sites of normal oral mucosa and of lesions belonging to three other histopathological categories, viz. oral squamous cell carcinoma (OSCC), oral submucous fibrosis (OSMF) and leukoplakia (OLK). In order to test the ability of the measured Raman spectra to predict pathological designation of the interrogated tissue sites, a probability based multi-class diagnostic algorithm was applied on the set of Raman spectra corresponding to the different oral tissue sites. With respect to histology as the gold standard, the diagnostic algorithm was found to provide a leave-one-subject-out cross validation accuracy of up to ~89% in classifying the oral tissue spectra into the different tissue categories. When employed for binary classification, the algorithm resulted in a sensitivity of 94.2% and a specificity of 94.4% in discriminating the normal from all the abnormal oral tissue spectra belonging to OSCC, OSMF and OLK pooled together. The results add to the growing body of evidence that Raman spectroscopy along with an appropriate diagnostic algorithm has strong potential to provide real-time, noninvasive diagnosis of malignant and potentially malignant lesions of oral cavity in a clinical situation.

Acknowledgements The authors would like to thank the nursing staff of the Head and Neck Surgery Department, Tata Memorial Hospital, Mumbai for their active help and cooperation.

Author biographies Please see Supporting Information online.

© 2013 by WILEY-VCH Verlag GmbH & Co. KGaA, Weinheim

References

[1] P. N. Notani, Curr. Sci. **81**(5), 465 (2001).
[2] T. Rastogi, S. Devesa, P. Mangtani, A. Mathew, N. Cooper, R. Kao, and R. Sinha, Int. J. Epidemiol. **37**, 147 (2008).
[3] M. K. Nair and R. Sankaranarayanan, Cancer Causes Control. **2**(4), 263–265 (1991).
[4] Global Adult Tobacco Survey, Ministry of Health and Family Welfare, Government of India India 2009–2010.
[5] S. El-Mofty, Egypt J. Oral Maxillofac. Surg. **1**, 25 (2010).
[6] P. Garg and F. Karjodkar, Int. J. Prev. Med. **3**(10), 737 (2012).
[7] J. B. Epstein, L. Zhang, and M. Rosin, J. Can. Dent. Assoc. **68**(10), 617 (2002).
[8] D. C. G. De Veld, M. J. H. Witjes, H. J. C. M. Sterenborg, and J. L. N. Roodenburg, Oral Oncol. **41**, 117 (2005).
[9] I. Pavlova, M. Williams, A. El-Naggar, R. Richards-Kortum, and A. Gillenwater, Clin. Cancer Res. **14**(8), 2396 (2008).
[10] D. C. G. De Veld, M. Skurichina, M. J. H. Witjes, R. P. W. Duin RP, H. J. C. M. Sterenborg, and J. L. N. Roodenburg, J. Biomed. Opt. **9**(5), 940 (2004).
[11] C. F. Poh, L. Zhang, D. W. Anderson, J. S. Durham, P. M. Williams, R. W. Priddy, K. W. Berean, S. Ng, O. L. Tseng, C. MacAulay, and M. P. Rosin, Clin. Cancer Res. **12**(22), 6716 (2006).
[12] K. H. Awana, P. R. Morgan, and S. Warnakulasuriya, Oral Oncol. **47**(4), 274 (2011).
[13] N. Subhash, J. R. Mallia, S. S. Thomas, A. Mathews, P. Sebastain, and J. Madhavan, J. Biomed. Opt. **11**(1), 014018 (2006).
[14] L. T. Nieman, C. W. Kan, A. Gillenwater, M. K. Markey, and K. Sokolov, J. Biomed. Opt. **13**(2), 024011 (2008).
[15] R. Richards-Kortum and E. Sevick-Muraca, Ann. Rev. Phys. Chem. **47**, 555 (1996).
[16] D. C. G. de Veld, M. Skurichina, M. J. H. Witjes, R. P. W. Duin, H. J. C. M. Sterenborg, and J. L. N. Roodenburg, Lasers Surg. Med. **36**, 356 (2005).
[17] R.A. Schwarz, W. Gao, D. Daye, M. D. Williams, R. Richards-Kortum, and A. M. Gillenwater, Appl. Opt. **47**(6), 825 (2008).
[18] R. Malini, K. Venkatakrishna, J. Kurien, K. M. Pai, L. Rao, V. B. Kartha, and C. M. Krishna, Biopolymers **81**, 179 (2006).
[19] Y. Li, Z. N. Wen, L. J. Li, Meng-Long Li, N. Gao, and Y. Z. Guob, J. Raman Spectrosc. **41**, 142 (2010).
[20] N. S. Sunder, N. N. Rao, V. B. Kartha, G. Ullas, and J. Kurien, Orofac. Sci. **3**(2), 15 (2011).
[21] L. Su, Y. F. Sun, Y. Chen, P. Chen, A. G. Shen, X. H. Wang, J. Jia, Y. F. Zhao, X. D. Zhou, and J. M. Hu, Laser Phys. **22**(1), 311 (2012).
[22] K. Guze, M. Short, H. Zeng, M. Lerman, and S. Sonis, J. Raman Spectrosc. **42**, 1232 (2011).
[23] A. Deshmukh, S. P. Singh, P. Chaturvedi, and C. M. Krishna, J. Biomed. Opt. **16**(12), 127004 (2011).
[24] S. Devpura, J. S. Thakur, S. Dethi, V. M. Naik, and R. Naik, J. Raman Spectrosc. **43**, 490 (2012).
[25] T. C. B. Schut, M. J. H. Witjes, H. J. C. M. Sterenborg, O. C. Speelman, J. L. N. Roodenburg, E. T. Marple, H. A. Bruining, and G. J. Puppels, Anal. Chem. **72**(24), 6010 (2000).
[26] A. P. Oliveira, R. A. Bitar, L. Silveria, R. A. Zangaro, and A. A. Martin, Photomed. Laser Surg. **24**(3), 348 (2006).
[27] A. Mahadevan-Jansen, in: T. Vo-Dinh (ed.), Biomedical Photonics Handbook (CRC Press, Washington DC, 2003) Chapter 30.
[28] K. Guze, M. Short, S. Sonis, N. Karimbux, J. Chan, and H. Zeng, J. Biomed. Opt. **14**(1), 014016 (2009).
[29] M. S. Bergholt, W. Zheng, and Z. Huang, J. Raman Spectrosc. **43**, 255 (2012).
[30] S. P. Singh, A. Deshmukh,, P. Chaturvedi, and C. M. Krishna, J. Biomed. Opt. **17**(10), 105002 (2012).
[31] A. Sahu, A. Deshmukh, A. D. Ghanate, S. P. Singh, P. Chaturvedi, and C. M. Krishna, Technol. Cancer Res. Treat. **11**(6), 529 (2012).
[32] J. T. Motz, S. J. Gandhi, O. R. Scepanovic, A. S. Haka, J. R. Kramer, R. R. Dasari, and M. S. Feld, J. Biomed. Opt. **10**(3), 031113 (2005).
[33] H. Krishna, S. K. Majumder, and P. K. Gupta, J. Raman Spectrosc. **43**, 1884 (2012).
[34] S. K. Majumder, S. C. Gebhart, M. D. Johnson, R. Thompson, W. C. Lin, and A. Mahadevan-Jansen, Appl. Spectrosc. **61**(5), 548 (2007).
[35] A. Talukder, Ph.D. thesis, Carnegie Mellon University, Pennsylvania (1999).
[36] B. Krishnapuram, L. Cari, M. A. T. Figueiredo, and A. J Hartemink, IEEE Trans. Pattern Anal. Machine Intell. **27**(6), 957 (2005).
[37] C. Ferri, J. Hernández-Orallo, and M. A. Salido, in: Proceedings of the 14th European Conference on Machine Learning, Cavtat-Dubrovnik, Croatia, September 2003, pp. 108–120.
[38] G. Jurman, S. Riccadonna, and C. Furlanello, PLoS ONE **7**(8), e41882 (2012).
[39] J. Gorodkin, Comput. Biol. Chem. **28**, 367 (2004).
[40] J. M. Wei, X. J. Yuan, Q. H. Hu, and S. Q. Wang, Expert Syst. Appl. **37**(5), 3799 (2010).
[41] D. J. Hand and R. J. Till, Mach. Learn. **45**, 171 (2001).
[42] J. D. Gelder, K. D. Gussem, P. Vandenabeele, and L. Moens, J. Raman Spectrosc. **38**, 1133 (2007).
[43] F. M. Lyng, E. O. Faoláin, J. Conroy, A. D Meade, P. Knief, B. Duffy, M. B. Hunter, J. M. Byrne, P. Kelehan, and H. J. Byrne, Exp. Mol. Patho. **82**, 121 (2007).
[44] C. A. Lieber, S. K. Majumder, D. L. Ellis, D. D. Billheimer, and A. Mahadevan-Jansen, Lasers Surg. Med. **40**, 461 (2008).
[45] K. Kolanjiappan, C. R. Ramachandran, and S. Manoharan, Clin. Biochem. **36**, 61 (2003).
[46] R. Rifkin, S. Mukherjee, P. Tamayo, S. Ramaswamy, C. H. Yeang, M. Angelo, M. Reich, T. Poggio, E. S. Lander, T. R. Golub, and J. P. Mesirov, SIAM Rev. **45**(4), 706 (2003).

© 2013 by WILEY-VCH Verlag GmbH & Co. KGaA, Weinheim

FULL ARTICLE

[47] C. K. Brookner, U. Utzinger, G. Staerkel, R. Richards-Kortum, and M. F. Mitchell, Lasers Surg. Med. **24**, 29 (1999).
[48] S. K. Majumder, N. Ghosh, and P. K. Gupta, Lasers Surg. Med. **36**, 323 (2005).
[49] I. T. Jolliffe, Principal component analysis (Springer Series in Statistics, 2nd ed., Springer, New York, 2002).
[50] S. K. Majumder, N. Ghosh, and P. K. Gupta, J. Biomed. Opt. **10**(2), 024034 (2005).
[51] A. Lazarevic and V. Kumar, in: KDD '05: Proceeding of the eleventh ACM SIGKDD international conference on Knowledge discovery in data mining, New York, NY, USA, ACM Press 2005, pp. 157–166.
[52] B. Zadrozny and C. Elkan, in: Proceedings of the 8th International Conference on Knowledge Discovery and Data Mining, Edmonton, ACM Press, 2002, pp. 694–699.
[53] Y. Li, J. Pan, G. Chen, C. Li, S. Lin, Y. Shao, S. Feng, Z. Huang, S. Xie, H. Zeng, and R. Chena, J. Biomed. Opt. **18**(2), 027003 (2013).
[54] K. Lin, D. L. P. Cheng, and Z. Huang, Biosens. Bioelectron. **35**, 213, (2012).

© 2013 by WILEY-VCH Verlag GmbH & Co. KGaA, Weinheim
