# Supplementary material for: The accuracy of Fiber-Optic Raman Spectroscopy in the detection and diagnosis of head and neck neoplasm in vivo: a systematic review and meta-analysis
Source: PeerJ. 2023 Dec 11;11:e16536. doi: 10.7717/peerj.16536 (PMC10720414; doi:10.7717/peerj.16536)
Supplement: Data S1 [file peerj-11-16536-s001.zip › article/lin2012.pdf]

Biosensors and Bioelectronics 35 (2012) 213–217

Contents lists available at SciVerse ScienceDirect

Biosensors and Bioelectronics

journal homepage: www.elsevier.com/locate/bios

Optical diagnosis of laryngeal cancer using high wavenumber Raman spectroscopy

Kan Lin[a], David Lau Pang Cheng[b], Zhiwei Huang[a,*]

[a] Optical Bioimaging Laboratory, Department of Bioengineering, Faculty of Engineering, National University of Singapore, Singapore 117576, Singapore
[b] Department of Otolaryngology, Singapore General Hospital, Singapore 169608, Singapore

ARTICLE INFO

Article history:
Received 26 December 2011
Received in revised form 21 February 2012
Accepted 23 February 2012
Available online 15 March 2012

Keywords:
High wavenumber Raman spectroscopy
Laryngeal cancer
Optical diagnosis

ABSTRACT

We report the implementation of the transnasal image-guided high wavenumber (HW) Raman spectroscopy to differentiate tumor from normal laryngeal tissue at endoscopy. A rapid-acquisition Raman spectroscopy system coupled with a miniaturized fiber-optic Raman probe was utilized to realize real-time HW Raman (2800–3020 cm^{-1}) measurements in the larynx. A total of 94 HW Raman spectra (22 normal sites, 72 tumor sites) were acquired from 39 patients who underwent laryngoscopic screening. Significant differences in Raman intensities of prominent Raman bands at 2845, 2880 and 2920 cm^{-1} (CH_2 stretching of lipids), and 2940 cm^{-1} (CH_3 stretching of proteins) were observed between normal and cancer laryngeal tissue. The diagnostic algorithms based on principal components analysis (PCA) and linear discriminant analysis (LDA) together with the leave-one subject-out, cross-validation method on HW Raman spectra yielded a diagnostic sensitivity of 90.3% (65/72) and specificity of 90.9% (20/22) for laryngeal cancer identification. This study demonstrates that HW Raman spectroscopy has the potential for the noninvasive, real-time diagnosis and detection of laryngeal cancer at the molecular level.
© 2012 Elsevier B.V. All rights reserved.

1. Introduction

Laryngeal cancer is one of the most common malignancies in humans worldwide due to its high incidence rate and mortality (Chu and Kim, 2008). For instance, in Southeast Asia, the rates of incidence and mortality of laryngeal cancer are significantly higher than other areas of the world (Cao et al., 2007; Döbróssy, 2005). Early cancer diagnosis in the larynx with effective treatment (e.g., surgery, radiotherapy or chemotherapy alone or in combination) is crucial to improving the 5-year survival rate (Chu and Kim, 2008; Lee et al., 2008). Positive endoscopic biopsy currently is the gold standard for cancer diagnosis, but it is invasive and impractical for screening high risk patients, which might affect the quality of the voice due to multiple biopsies (Lee et al., 2008). Fiber-optic laryngoscopy is the primary physical examination tool for now (Chu and Kim, 2008), which relies on white-light illumination while requires highly experienced skills of recognization and locating pathologic tissues (Beser et al., 2009). Raman spectroscopy is a unique vibrational technique capable of probing biomolecular changes in tissue, which has shown great promise for early diagnosis and detection of precancer and cancer diagnosis in a variety of organs (e.g., skin, cervix, lung, esophagus, stomach, colon, kidney, bladder, breast, nasopharynx and the larynx) (Nijssen et al., 2002; Stone et al., 2000; Lau et al., 2003, 2005; Almond et al., 2011; Bergholt et al., 2011a,b; Draga et al., 2010; Gniadecka et al., 2004; Haka et al., 2006; Hale et al., 2009; Huang et al., 2003, 2005; Mo et al., 2009; Kanter et al., 2009; Magee et al., 2009; Short et al., 2006; Teh et al., 2008, 2009a; Widjaja et al., 2008; Mahadevan-Jansen and Richards-Kortum, 1996; Wong Kee Song et al., 2005; Shim et al., 2000). Current Raman research in diagnosing laryngeal cancer is mostly focused on the so-called fingerprint region (i.e., 800–1800 cm^{-1}) that contains rich biochemical information about the tissue (Stone et al., 2000; Mahadevan-Jansen and Richards-Kortum, 1996; Lau et al., 2003, 2005; Teh et al., 2009b). However, the strong fluorescence background and Raman signals attributed to the silica fiber severely interfere with the detection of the inherently weak tissue Raman signal, leading to a complex fiber probe filtering design as well as signal analysis in the fingerprint region. On the other hand, the high wavenumber (HW) (2800–3800 cm^{-1}) Raman spectroscopy can also provide complementary biochemical information for tissue diagnosis and characterization with much stronger tissue Raman signals but reduced tissue/fiber fluorescence background (Koljenovic et al., 2005; Nijssen et al., 2007; Mo et al., 2009), as compared to the fingerprint Raman spectroscopy. To date, HW Raman spectroscopy for laryngeal tissue diagnosis and characterization has yet been reported in literature. In this work, we study the implementation of the transnasal image-guided HW Raman spectroscopy developed to differentiate tumor from normal

* Corresponding author at: Optical Bioimaging Laboratory, Department of Bioengineering, Faculty of Engineering, National University of Singapore, 9 Engineering Drive 1, Singapore 117576, Singapore. Tel.: +65 6516 8856; fax: +65 6872 3069.
E-mail address: biehzw@nus.edu.sg (Z. Huang).

0956-5663/$ – see front matter © 2012 Elsevier B.V. All rights reserved.
doi:10.1016/j.bios.2012.02.050

laryngeal tissue. A rapid-acquisition Raman spectroscopy system coupled with a miniaturized fiber-optic Raman probe was utilized to realize real-time HW Raman (2800–3020 cm^{-1}) measurements in the larynx. Multivariate statistical techniques, including principal components analysis (PCA) and linear discriminant analysis (LDA), are utilized to develop diagnostic algorithms for differentiation between normal and cancerous laryngeal tissue. The receiver operating characteristic (ROC) curve is also conducted to further evaluate the performance of PCA–LDA algorithms on HW Raman spectroscopy for laryngeal cancer diagnosis.

2. Materials and methods

2.1. Raman endoscopic instrumentation

Fig. 1 shows the schematic diagram of the image-guided Raman spectroscopy developed for tissue measurements and characterization (Huang et al., 2009). Briefly, the Raman spectroscopy system consists of a spectrum stabilized 785 nm diode laser (maximum output: 300 mW, B&W TEK Inc., Newark, DE), a transmissive imaging spectrograph (Holospec f/1.8, Kaiser Optical Systems) equipped with a liquid nitrogen cooled (−120 °C), NIR-optimized, back-illuminated and deep depletion charge-coupled device (CCD) camera (1340 × 400 pixels at 20 μm × 20 μm per pixel; Spec-10: 400BR/LN, Princeton Instruments). We have constructed a 1.8 mm fiber-optic Raman endoscopic probe with dual coatings on the fiber tip for optimizing both the tissue excitation and Raman collections (Huang et al., 2009). The 785 nm laser is coupled into the central delivery fiber (200 μm, NA = 0.22) of the Raman probe for tissue excitation, while the backscattered tissue Raman photons from the laryngeal tissue are collected by the surrounding fibers (32 μm × 200 μm, NA = 0.22). The Raman fiber probe fits into the instrument channel of laryngoscope and can be safely targeted to different locations in the larynx under the multimodal wide-field imaging (i.e., white-light reflectance (WLR) and narrow-band imaging (NBI)) guidance. The system acquires HW Raman spectra over the range of 2800–3020 cm^{-1}, and each raw spectrum is acquired within 1 s with light irradiance of 1.5 W/cm^2. The spectral resolution of the system is about 9 cm^{-1}, and all wavelength-calibrated HW Raman spectra are also corrected for the wavelength-dependence of the system using a standard lamp (RS-10, EG&G Gamma Scientific, San Diego, CA). HW Raman spectra are then extracted from the raw tissue spectra using established pre-processing methods including smoothing, baseline subtraction, etc. (Bergholt et al., 2011a). All the spectral pre-processing is completed on-line, and the Raman spectra and the outcome of decision algorithms can be displayed in real-time in a comprehensible graphical user interface (GUI) during clinical transnasal Raman endoscopy.

2.2. Subjects

This study was approved by the SingHealth Centralized Institutional Review Board (IRB), Singapore. A total of 39 different patients with a mean age of 60 who underwent surgical resection due to laryngeal malignancies were recruited for this study. All patients preoperatively signed an informed consent permitting Raman measurements on laryngeal tissue. HW Raman spectra were directly acquired from the suspicious lesion sites for each patient through gently placing the fiber-optic Raman probe on the tissue with signal acquisition time of <1 s. HW Raman spectra were also measured from the surrounding normal sites that appear completely normal in the laryngoscopist's opinion (i.e., normal tissue does not exhibit colored patterned changes that only accompany precursor lesions) (Arens et al., 2004), but no biopsies were taken from normal appearing tissue. Only highly abnormal sites measured were biopsied and then submitted for histopathologic examination. A total of 94 Raman spectra (22 normal, 72 tumor as confirmed by histopathology) from different tissue sites were collected. For the assessment of diagnostic sensitivity and specificity of Raman endoscopy for tissue classification, histopathological results served as the gold standard.

2.3. Data preprocessing

All raw spectral data were processed on-line with software developed in the Matlab environment (The MathWorks, Inc., Natick, MA) (Bergholt et al., 2011a). Raw spectra are first pre-processed by a first-order Savitsky–Golay filter to reduce background noise (Savitzky and Golay, 1964). A first-order polynomial was used to fit tissue autofluorescence background and then subtracted from the raw spectra to obtain the pure HW Raman spectra. The HW Raman spectra are then normalized over the integrated area under the curve from 2800 to 3020 cm^{-1} to allow a better comparison of the spectral shapes and relative Raman band intensities among different subjects/tissue sites (Huang et al., 2003).

2.4. Multivariate statistical analysis

Principal components analysis (PCA) was used to reduce dimensionality of the Raman data (each Raman spectrum ranging from 2800 to 3020 cm^{-1} with set of 255 intensities), retaining the most diagnostically significant information for effective tissue classification. The spectra were first standardized to ensure that mean of the spectra was zero and the standard deviation (SD) was one, eliminating the influence of inter- and/or intra-subject spectral variability on PCA. Mean centering ensured that the principal components (PCs) form an orthogonal basis (Devore, 2009; Lachenbruch and Mickey, 1968). Thus, PCA were employed to extract a set of orthogonal PCs comprising loadings and scores that accounted for most of the total variance in original spectra. Each loading vector is related to the original spectrum by a variable called the PC score, which represents the weight of that particular component against the basis spectrum. The most diagnostically significant PCs ($p < 0.05$) were determined by Student's t-test (Devore, 2009) and then selected as input for the development of linear discriminant analysis algorithms for classification. LDA determines the discriminant function that maximizes the variances in the dataset between groups while minimizing the variances between members of the same group. The performance of the diagnostic algorithms rendered by the PCA–LDA models for correctly predicting the tissue groups (e.g., normal vs. cancer) was estimated in an unbiased manner using the leave-one subject-out, cross-validation method (Dillon and Goldstein, 1984; Lachenbruch and Mickey, 1968) on all model spectra. In this method, the spectra from each same patient were held out from the data set and the PCA–LDA modeling was redeveloped using the remaining HW Raman spectra. The redeveloped PCA–LDA diagnostic algorithm was then used to classify the withheld spectra. This process was repeated until all withheld spectra were classified. Receiver operating characteristic (ROC) curves were generated by successively changing the thresholds to determine correct and incorrect classifications for all tissues. Multivariate statistical analysis was performed online using in-house written scripts in the Matlab (Mathworks Inc., Natick, MA) programming environment (Bergholt et al., 2011a).

3. Results

Fig. 2A shows the comparison of mean HW Raman spectra ±1 SD of normal ($n = 22$) and cancer ($n = 72$) laryngeal tissue. Prominent Raman bands such as 2845, 2880, and 2920 cm^{-1} (CH_2 stretching of lipids), and 2940 cm^{-1} (CH_3 stretching of proteins) (Eikje et al., 2005; Mo et al., 2009; Santos et al., 2005) are found in both

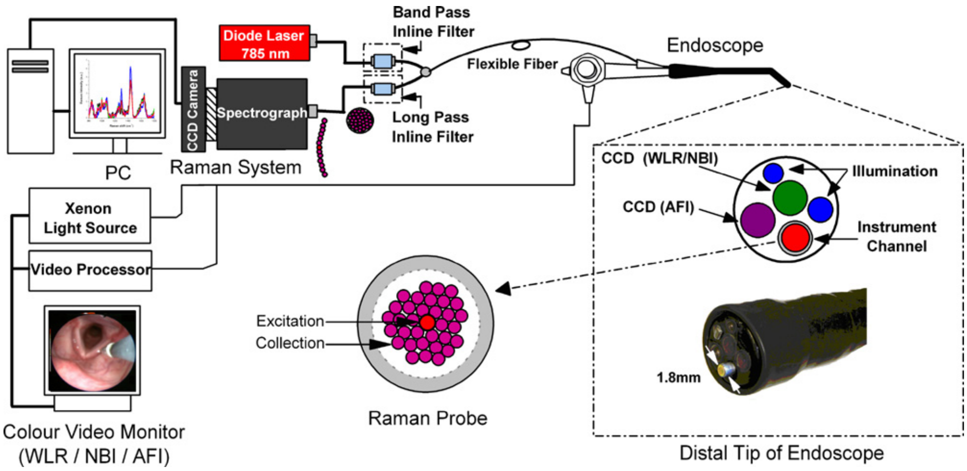

Fig. 1. Schematic of the integrated Raman spectroscopy and trimodal endoscopic imaging system developed for *in vivo* tissue Raman measurements.

normal and tumor laryngeal tissues. As shown in the difference spectrum (Fig. 2B), the intensities of Raman band between 2810 and 2900 cm^{-1} in cancer tissue is obviously greater than normal tissue, while the Raman band between 2900 and 3020 cm^{-1} the normal is higher. This suggests that there is an increase or decrease in particular types of biomolecules relative to the total Raman-active biomolecules in cancer tissue as compared to normal tissue, demonstrating the potential role of HW Raman spectroscopy for cancer diagnosis in the larynx.

To determine the most significant Raman features for tissue analysis and classification, the multivariate statistical technique (e.g., PCA–LDA) coupled with Student's *t*-test are performed by incorporating the entire HW Raman spectra. Fig. 3 shows the first five dominant principal components (PCs) accounting for about 99.2% (PC1: 89.1%; PC2: 7.41%; PC3: 1.52%; PC4: 1.08%; PC5: 0.07%) of the total variance calculated from HW Raman spectra of laryngeal tissues. Overall, the PC features among different PCs are different, but some PC features roughly correspond to HW Raman spectra, with peaks and troughs at positions (e.g., CH_2 stretch band (lipids) near 2845 cm^{-1}, 2880 cm^{-1}, 2920 cm^{-1} and CH_3 stretch band (proteins) near 2940 cm^{-1}) similar to those of tissue HW Raman spectra. The first PC accounts for the largest variance within the spectral data sets (e.g., 89.1%), whereas successive PCs describe features that contribute progressively smaller variances. Unpaired two-sided

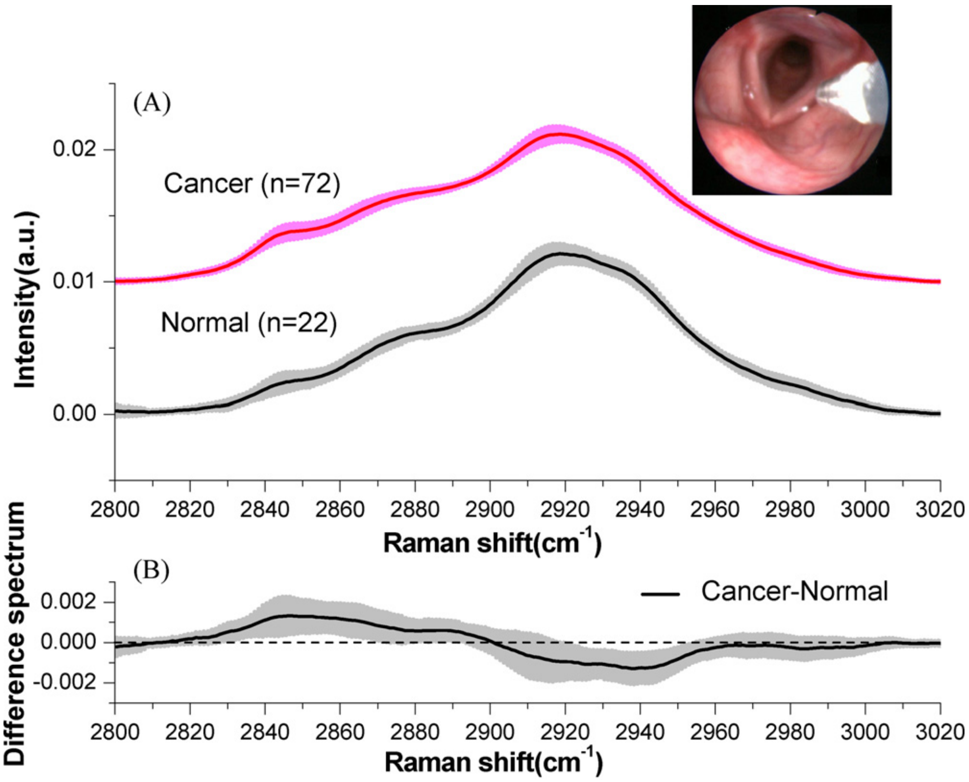

Fig. 2. (A) Comparison of the mean HW Raman spectra ±1 standard deviations (SD) of normal (n = 22) and cancer (n = 72) laryngeal tissue. (B) Difference spectrum ±1 SD between cancer (n = 72) and normal laryngeal tissue (n = 22). Note that the mean normalized HW Raman spectrum of normal tissue was shifted vertically for better visualization (panel A); the shaded areas indicate the respective standard deviations. The picture shown is the Raman acquisitions from the larynx using endoscopic fiber-optic Raman probe.

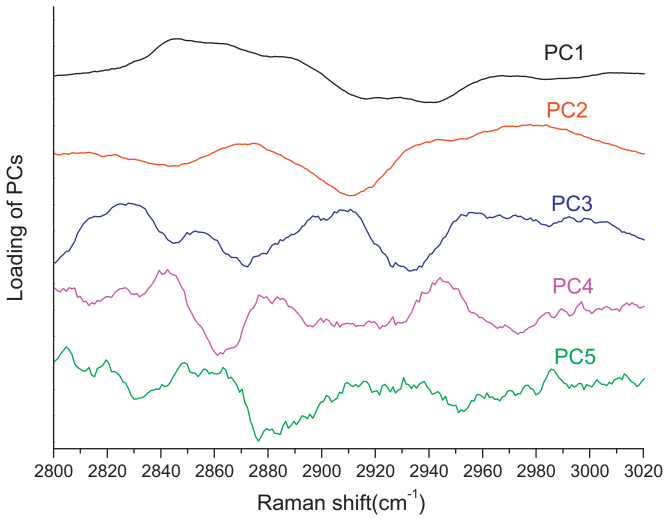

Fig. 3. The first five principal components (PCs) accounting for about 99.2% of the total variance calculated from HW Raman spectra of laryngeal tissue (PC1 = 89.1%; PC2 = 7.41%; PC3 = 1.52%; PC4 = 1.08%; PC5 = 0.07%).

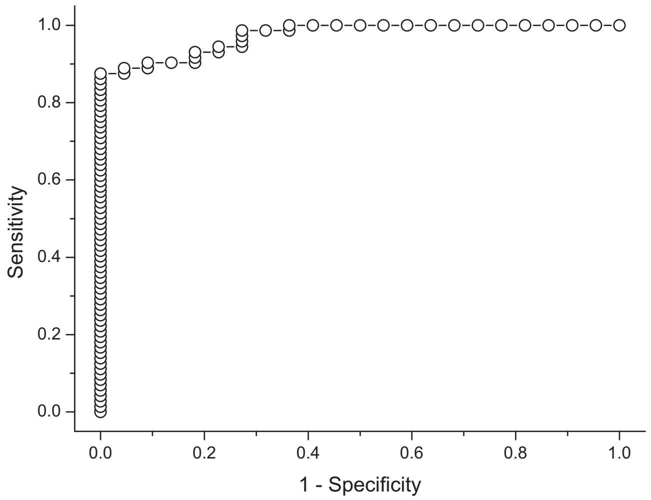

Fig. 5. ROC curve of discrimination results for HW Raman spectra utilizing the PCA–LDA-based spectral classification with leave-one subject-out, cross validation. The integration area under the ROC curves is 0.97 for PCA–LDA-based diagnostic algorithm.

Student's t-tests on the first five PCs show that only three PCs (PC1, PC2 and PC3, $p < 0.05$) are diagnostically significant.

To develop effective diagnostic algorithms for tissue classification, the three diagnostically significant PCs are fed into the LDA model together with leave-one subject-out, cross-validation technique for tissue classification. PCA–LDA algorithms on the HW tissue Raman data provide the diagnostic sensitivity of 90.3% (65/72) and specificity of 90.9% (20/22) for laryngeal cancer identification (Fig. 4). In addition, the ROC curve (Fig. 5) was also generated from the posterior probability plot in Fig. 4 at different threshold levels, displaying the performance of PCA–LDA-based diagnostic algorithms derived for laryngeal cancer detection. The integration area under the ROC curve is 0.97, further confirming that HW Raman technique coupled with PCA–LDA-based diagnostic algorithms is robust for laryngeal cancer diagnosis.

4. Discussion

Raman spectroscopy holds a great promise for clinical application, as it can be used as a non-invasive technique for early

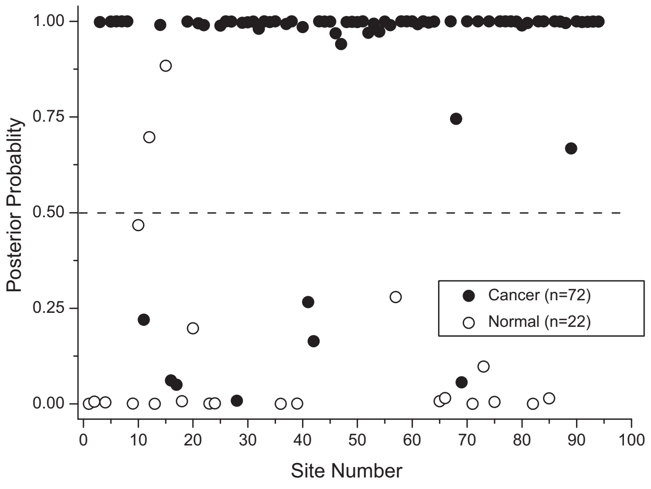

Fig. 4. Scatter plot of the posterior probability belonging to normal and cancer categories using the PCA–LDA method together with leave-one subject-out, cross-validation method. The algorithm yields a diagnostic sensitivity of 90.3% and specificity of 90.9% for differentiation between normal and tumor tissues.

detection of biomolecular changes associated with tissue pathology. Our study demonstrates that HW Raman technique is capable of generating spectral differences between normal and cancer tissue in the larynx as shown in Fig. 2. The empirical analysis based on the intensity ratio measurements, which relate to the changes in protein-to-lipid contents, has already been reported in literature (Huang et al., 2003; Short et al., 2005). Student's t-test ($p < 0.0001$) is used to test the significant difference of Raman intensity ratios of normal and cancer tissues. Intensity at 2920 cm^{-1} and 2940 cm^{-1} is higher in normal tissues, while at 2845 and 2890 cm^{-1} are higher in cancer tissues. The three significant Raman peak intensity ratios of I_{2940}/I_{2845}, I_{2940}/I_{2890} and I_{2940}/I_{2920} correlated with their histopathologic findings were also evaluated, and the decision lines (i.e., diagnostic algorithms) separated cancer from normal tissues with a sensitivity of 72.2% (52/72), 45.8% (33/72) and 44.4% (32/72), while a sensitivity of 81.8% (18/22), 72.7% (16/22) and 63.6% (14/22), respectively, for laryngeal cancer identification. These indicate that different ratios of Raman band intensities only give a certain levels of accuracy for tissue classification. The Raman intensity ratios of I_{2940}/I_{2845} and I_{2940}/I_{2890} are both lower for cancer tissue, this may be due to the decrease in content of collagen. In cancer progression, from genetic mutation to invasive cancer in the laryngeal, the epithelium turns thicker and thus obstructs the collagen Raman emission from deep collagen basal membrane, thereby decreases the overall ratio of I_{2940}/I_{2845} in laryngeal cancer tissue (Teh et al., 2008). Besides, the contribution of collagen in cancer tissue should be reduced due to proliferation of cancerous cells and express as a class of metalloportease, which in turn decrease the content of collagen level. Raman band at 2845 cm^{-1} is tentatively ascribed to CH_2 lipids and it seems to correspond to Raman peak at 1450 cm^{-1} in the fingerprint region, which is assigned to CH_2 protein/lipids. CH_2 protein/lipids are found to be higher in cancer than in normal laryngeal tissues, which can be explained by the increase of mitotic activity in the nucleus (Stone et al., 2000; Haka et al., 2006; Mo et al., 2009; Teh et al., 2008, 2009b). The distinctive differences in HW Raman spectra between normal and cancer laryngeal tissues reinforce that HW Raman spectroscopy can be used to reveal molecular changes associated with carcinogenesis progression.

Considering the complexity of biological tissue, multivariate statistical analysis (PCA–LDA) which incorporates the entire Raman spectra data for analysis is more robust and rigorous to differentiate spectra that represent either normal or cancer tissue. Compared

with intensity ratio approach, there is a great improvement in diagnostic sensitivity (∼25%) and specificity (∼11%) of PCA–LDA algorithms (Fig. 4). The ROC curve (Fig. 5) of PCA–LDA modeling (AUC = 0.97) further verifies a better diagnostic efficacy of HW Raman spectroscopy integrated with PCA–LDA algorithm as compared to the intensity ratio diagnostic algorithms. One note that PCA is primarily for data reduction rather than identification of biochemical or biomolecular components of tissue, it is difficult to interpret the physical meanings from the component spectra. This could be tackled by using more powerful diagnostic algorithms such as genetic algorithms (Mountford et al., 2001), the distinctive spectral regions that are optimal for tissue differentiation may be indentified and related to particular biochemical and biomolecular changes (e.g., proteins, lipids and nucleic acid) associated with neoplastic changes. It is also crucial to further understanding the relationship between the neoplastic-related morphologic/biochemical changes and tissue HW Raman spectra for laryngeal precancer/cancer diagnosis (Stone et al., 2000; Bergholt et al., 2011a; Teh et al., 2008). We are currently working on this direction by recruiting more patients for developing more powerful HW Raman genetic diagnostic algorithms for real-time laryngeal tissue diagnosis and characterization.

In summary, this work demonstrates that transnasal image-guided real-time HW Raman spectroscopic technique integrated with an endoscope-based fiber-optic Raman probe can be used to acquire HW Raman spectra from laryngeal tissue in the range 2800–3020 cm^{-1} during clinical endoscopic examination. The significant differences in HW Raman spectra are observed in normal and cancer laryngeal tissue. The PCA–LDA modeling on HW Raman spectra provides good tissue classification, illustrating the potential of HW Raman spectroscopy for real-time laryngeal cancer detection during clinical endoscopic examination.

Acknowledgement

This research was supported by the Biomedical Research Council, and the National Medical Research Council, Singapore.

References

Almond, L.M., Hutchings, J., Shepherd, N., Barr, H., Stone, N., Kendall, C., 2011. J. Biophotonics 4, 685–695.

Arens, C., Dreyer, T., Glanz, H., Malzahn, K., 2004. Eur. Arch. Otorhinolaryngol. 261, 71–76.

Bergholt, M.S., Zheng, W., Lin, K., Ho, K.Y., Teh, M., Yeoh, K.G., So, J.B.Y., Huang, Z., 2011a. Technol. Cancer Res. Treat. 10, 103–112.

Bergholt, M.S., Zheng, W., Lin, K., Ho, K.Y., Teh, M., Yeoh, K.G., Yan So, J.B., Huang, Z., 2011b. Int. J. Cancer 128 (11), 2673–2680.

Beser, M., Gultekin, E., Yener, M., Zeybek, M., Öner, B., Topçu, V., 2009. Eur. Arch. Otorhinolaryngol. 266, 1953–1958.

Cao, W.F., Zhang, L.Y., Liu, M.B., Tang, P.Z., Liu, Z.H., Sun, B.C., 2007. Hum. Pathol. 38, 747–752.

Chu, E.A., Kim, Y.J., 2008. Otolaryngol. Clin. North Am. 41, 673–695.

Devore, J.L., 2009. Probability and Statistics for Engineering and the Sciences. Thomson/Brooks/Cole, Belmont, CA.

Dillon, W.R., Goldstein, M., 1984. Nueva York. EUA: Wiley.

Döbróssy, L., 2005. Cancer Metastasis Rev. 24, 9–17.

Draga, R.O.P., Grimbergen, M.C.M., Vijverberg, P.L.M., Swol, C.F.P.v., Jonges, T.G.N., Kummer, J.A., Ruud Bosch, J.L.H., 2010. Anal. Chem. 82, 5993–5999.

Eikje, N.S., Ozaki, Y., Aizawa, K., Arase, S., 2005. J. Biomed. Opt. 10, 014013.

Gniadecka, M., Philipsen, P.A., Sigurdsson, S., Wessel, S., Nielsen, O.F., Christensen, D.H., Hercogova, J., Rossen, K., Thomsen, H.K., Gniadecki, R., Hansen, L.K., Wulf, H.C., 2004. J. Invest. Dermatol. 122, 443–449.

Haka, A.S., Volynskaya, Z., Gardecki, J.A., Nazemi, J., Lyons, J., Hicks, D., Fitzmaurice, M., Dasari, R.R., Crowe, J.P., Feld, M.S., 2006. Cancer Res. 66, 3317–3322.

Hale, W., Rachel, K., Cory, S., Brian, S., Raja, R., Janet, P., Abhilash, P., Greg, A., Michael, D.K., 2009. J. Pediatr. Surg. 44, 1152–1158.

Huang, Z., McWilliams, A., Lui, H., McLean, D.I., Lam, S., Zeng, H., 2003. Int. J. Cancer 107, 1047–1052.

Huang, Z., Lui, H., McLean, D.I., Korbelik, M., Zeng, H., 2005. Photochem. Photobiol. 81, 1219–1226.

Huang, Z., Teh, S.K., Zheng, W., Mo, J., Lin, K., Shao, X., Ho, K.Y., Teh, M., Yeoh, K.G., 2009. Opt. Lett. 34, 758–760.

Kanter, E.M., Vargis, E., Majumder, S., Keller, M.D., Woeste, E., Rao, G.G., Mahadevan-Jansen, A., 2009. J. Biophotonics 2, 81–90.

Koljenovic, S., Schut, T.C.B., Wolthuis, R., de Jong, B., Santos, L., Caspers, P.J., Kros, J.M., Puppels, G.J., 2005. J. Biomed. Opt. 10, 031111–031116.

Lachenbruch, P.A., Mickey, M.R., 1968. Technometrics 10, 1–11.

Lau, D.P., Huang, Z., Lui, H., Man, C.S., Berean, K., Morrison, M.D., Zeng, H., 2003. Lasers Surg. Med. 32 (3), 210–214.

Lau, D.P., Huang, Z., Lui, H., Anderson, D.W., Berean, K., Morrison, M.D., Shen, L., Zeng, H., 2005. Lasers Surg. Med. 37 (3), 192–200.

Lee, L.-A., Cheng, A.-J., Fang, T.-J., Huang, C.-G., Liao, C.-T., Chang, J.T.-C., Li, H.-Y., 2008. Laryngoscope 118, 50–55.

Magee, N.D., Villaumie, J.S., Marple, E.T., Ennis, M., Elborn, J.S., McGarvey, J.J., 2009. J. Phys. Chem. B 113, 8137–8141.

Mahadevan-Jansen, A., Richards-Kortum, R., 1996. J. Biomed. Opt. 1, 31–70.

Mo, J., Zheng, W., Low, J.J.H., Ng, J., Ilancheran, A., Huang, Z., 2009. Anal. Chem. 81, 8908–8915.

Mountford, C.E., Somorjai, R.L., Malycha, P., Gluch, L., Lean, C., Russell, P., Barraclough, B., Gillett, D., Himmelreich, U., Dolenko, B., Nikulin, A.E., Smith, I.C.P., 2001. Br. J. Surg. 88, 1234–1240.

Nijssen, A., Bakker Schut, T.C., Heule, F., Caspers, P.J., Hayes, D.P., Neumann, M.H.A., Puppels, G.J., 2002. J. Invest. Dermatol. 119, 64–69.

Nijssen, A., Maquelin, K., Santos, L.F., Caspers, P.J., Schut, T.C.B., den Hollander, J.C., Neumann, M.H.A., Puppels, G.J., 2007. J. Biomed. Opt. 12, 034004–034007.

Savitzky, A., Golay, M.J.E., 1964. Anal. Chem. 36, 1627–1639.

Santos, L.F., Wolthuis, R., Koljenović, S., Almeida, R.M., Puppels, G.J., 2005. Anal. Chem. 77, 6747–6752.

Shim, M.G., Wong Kee Song, L.M., Marcon, N.E., Wilson, B.C., 2000. Photochem. Photobiol. 72, 146–150.

Short, K.W., Carpenter, S., Freyer, J.P., Mourant, J.R., 2005. Biophys. J. 88, 4274–4288.

Short, M.A., Lui, H., McLean, D., Zeng, H., Alajlan, A., Chen, X.K., 2006. J. Biomed. Opt. 11, 034004–034009.

Stone, N., Stavroulaki, P., Kendall, C., Birchall, M., Barr, H., 2000. Laryngoscope 110, 1756–1763.

Teh, S.K., Zheng, W., Ho, K.Y., Teh, M., Yeoh, K.G., Huang, Z., 2008. Br. J. Cancer 98, 457–465.

Teh, S.K., Zheng, W., Lau, D.P., Huang, Z., 2009a. Analyst 134, 1232–1239.

Teh, S.K., Zheng, W., Ho, K.Y., Teh, M., Yeoh, K.G., Huang, Z., 2009b. J. Raman Spectrosc. 40, 908–914.

Widjaja, E., Zheng, W., Huang, Z., 2008. Int. J. Oncol. 32, 653–662.

Wong Kee Song, L.M., Molckovsky, A., Wang, K.K., Burgart, L.J., Dolenko, B., Somorjai, R.L., Wilson, B.C., 2005. Proc. SPIE 5692, 140–146.
