# Supplementary material for: The accuracy of Fiber-Optic Raman Spectroscopy in the detection and diagnosis of head and neck neoplasm in vivo: a systematic review and meta-analysis
Source: PeerJ. 2023 Dec 11;11:e16536. doi: 10.7717/peerj.16536 (PMC10720414; doi:10.7717/peerj.16536)
Supplement: Data S1 [file peerj-11-16536-s001.zip › article/lin2017 (1).pdf]

Accepted Article

Autofluorescence and white light imaging guided endoscopic Raman and diffuse reflectance spectroscopy for *in vivo* nasopharyngeal cancer detection

* Corresponding author: e-mail: hzeng@bccrc.ca, Phone: +1 604 675 8083, Fax: +1 604 675 8099
** Corresponding author: e-mail: chenr@fjnu.edu.cn, Phone: +86 591 83489919, Fax: +86 591 83465373

Duo Lin [†,1,2] , *Sufang Qiu* [†,3], *Wei Huang* [†,4], *Jianji Pan* [3], *Zhihong Xu* [1], *Rong Chen* [**,1], *Shangyuan Feng* [1], *Guannan Chen* [1], *Yongzeng Li* [1], *Michael Short* [5], *Jianhua Zhao* [5], *Yasser Fawzy* [5], *and Haishan Zeng* [*,1,5]

[1] Key Laboratory of OptoElectronic Science and Technology for Medicine, Ministry of Education, Fujian Provincial Key Laboratory for Photonics Technology, Fujian Normal University, Fuzhou 350007, China
[2] College of Integrated Traditional Chinese and Western Medicine, Fujian University of Traditional Chinese Medicine, Fuzhou, Fujian, 350122, China
[3] Department of Radiation Oncology, Fujian Provincial Cancer Hospital; Fujian Medical University Cancer Hospital; Fujian Provincial Key Laboratory of Translational Cancer Medicine, Fuzhou, 350014, China
[4] Fujian Metrology Institute, Fuzhou 350003, China
[5] Imaging Unit - Integrative Oncology Department, BC Cancer Agency Research Centre, Vancouver, BC V5Z 1L3, Canada

Key words: Autofluorescence imaging, Diffuse reflectance spectroscopy, Raman spectroscopy, Nasopharyngeal cancer detection

Nasopharyngeal cancer (NPC) is an endemic with high incidence in Southern China and Southeast Asia countries. Screening for NPC under conventional white light imaging (WLI) nasopharyngoscope examination remains a great clinical challenge due to its poor sensitivity. Here, we developed an integrated four-modality endoscopy system combining WLI, autofluorescence imaging (AFI), diffuse reflectance spectroscopy (DRS), and Raman spectroscopy (RS) technologies for *in vivo* endoscopic cancer detection for the first time. A pilot clinical test of the system for NPC detection was conducted, in which 283 in vivo Raman and diffuse reflectance spectral data sets from 30 NPC patients and 30 healthy subjects were acquired under the guidance of AFI and WLI. Both high diagnostic sensitivity (98.6%) and high specificity (95.1%) for differentiating cancer from normal tissue sites were achieved using this system combined with PCA-LDA (principal component analysis-linear discriminant analysis) diagnostic algorithm, demonstrating great potential for improving real-time *in vivo* diagnosis of NPC at endoscopy.

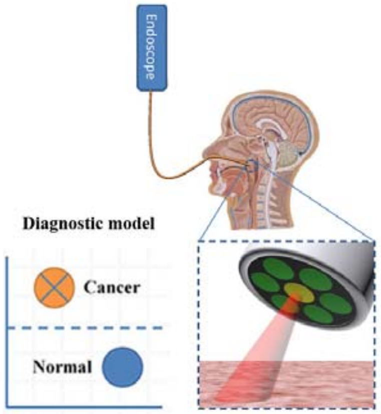

An integrated endoscopy system provides white light imaging, autofluorescence imaging, Raman spectroscopy and diffuse reflectance spectroscopy for *in vivo* nasopharyngeal cancer detection.

1. Introduction

This article has been accepted for publication and undergone full peer review but has not been through the copyediting, typesetting, pagination and proofreading process, which may lead to differences between this version and the Version of Record. Please cite this article as doi: 10.1002/jbio.201700251

This article is protected by copyright. All rights reserved.

Accepted Article

Nasopharyngeal cancer (NPC) is one of the head and neck malignancies arising from the nasopharyngeal epithelial lining. According to the Global Cancer Statistics, there were estimated 86,700 new cases and 50,800 deaths worldwide in 2012, most of which occurred in South-Eastern Asia including Malaysia, Indonesia, Singapore and Southern China regions, presenting a unique geographic and ethnic distribution [1, 2]. Unfortunately, approximately 75%-90% patients with NPC were diagnosed in advanced stages leading to poor treatment outcome in clinical practice [3]. Hence, accurate screening and diagnostics coupled with timely and appropriate treatment is critical to improving the survival of NPC patients. Immunoserology is an attractive early screening strategy for NPC. It is a convenient blood test that uses IgA antibody against EBV-related antigen, such as VCA-IgA, EA-IgA and EBNA1-IgA. However, this serological test may bring 2-18% false-positive rate in practical screening process [4]. White light nasopharyngoscopy examination is still the most commonly used method in clinical NPC screening. However it mainly relies on subjective visual identification of tissue morphological features, inevitably leading to missed diagnosis. This endoscopy guided biopsy followed by histopathological examination is regarded as the gold standard method for NPC diagnosis, but still suffers from the disadvantages of invasive procedure, time-consuming, and inapplicability for mass screening [5]. Therefore, development of a rapid, accurate, minimally invasive, real-time and *in vivo* diagnostic technology would be of imperative clinical value for improving NPC screening.

Currently, there exist numerous novel optical detection techniques that rely on sensing unique optical properties from biological tissues, with many being ideally suited for cancer screening and diagnosis. For instance, autofluorescence imaging (AFI) is capable of detecting tissue changes from the fluorescence profile of endogenous fluorophores associated with cancer transformation, making it possible to distinguish normal from malignant lesions [6, 7]. Our group previously applied AFI method for early lung cancer detection, and found that the green fluorescence intensity of cancerous tissue was significantly lower than that of normal tissue, suggesting great potential to improve diagnostic sensitivity [8]. To the best of our knowledge, there is no clinical report on AFI for NPC detection yet. Nevertheless, AFI suffers from the limitation of lower specificity while set for high sensitivity operation, resulting in high false positive biopsies. Chhajed et. al reported a sensitivity of 96% and specificity of down to 23% in lung cancer detection using lung imaging fluorescence endoscopy [9].

The drawback of AFI can be improved by introducing spectroscopic technologies with higher diagnostic specificity, such as diffuse reflectance spectroscopy (DRS) and Raman spectroscopy (RS). DRS can provide distinct quantitative information of tissue in morphological and functional aspects. A recent clinical study on tissue detection using DRS during *in vivo* lung biopsy, showed clear differences in absorption between tumor tissue and surrounding lung tissue, demonstrating great potential of DRS for biopsy guidance [10]. Besides, an integrated endoscopy system with AFI in conjunction with DRS was tested in clinical practice for lung cancer detection, presenting great potential to improve both diagnostic sensitivity and specificity at the same time [8, 11].

Raman spectroscopy (RS) based on inelastic light scattering is another powerful cancer diagnostic tool since it is capable of capturing ‘fingerprints’ information of biomolecular structures and conformations, providing a unique opportunity to detect subtle changes associated with malignant transformation [12-14]. Notably, RS is a rapid, non-invasive, reproducible measurement, and can be easily performed with fiber-optic probe, thereby particularly suitable for *in vivo* cancer detection [15]. An integrated real-time Raman spectroscopy system was developed by our group for *in vivo* skin cancer diagnosis, and it was found that malignant and benign skin lesions could be differentiated with good diagnostic accuracy comparable with expert clinical examination [14]. Furthermore, we found that the number of false-positive biopsies could be significantly reduced when RS was combined with AFI for lung cancer detection at endoscope [16, 17]. In addition, more applications of RS for *in vivo* cancer diagnosis have also been investigated by other groups for different types of cancers, such as laryngeal, esophageal, gastric, colorectal cervical and breast tumor, demonstrating great potential of RS to be a powerful clinical diagnostic tool [18, 19].

In this work, an integrated four-modality endoscopy system combining white light imaging, autofluorescence imaging, diffuse reflectance

This article is protected by copyright. All rights reserved.

Accepted Article

spectroscopy and Raman spectroscopy technologies, was developed for the first time. Relying on high diagnostic sensitivity of imaging technology (AFI) and high specificity of spectroscopic technologies (DRS and RS), this specially designed endoscopy system may offer a unique opportunity for improving *in vivo* NPC diagnostics in clinical practices.

2. Experimental

2.1. Patients

This study was performed in accordance with relevant guidelines and regulations, and approved by the ethical committee at our institution (Fujian Provincial Cancer Hospital, Fujian, China). Informed consents were obtained from all participating subjects. A total of 283 *in vivo* tissue Raman spectra and 283 diffuse reflectance spectra (normal: n=142; cancer: n=141) were successfully acquired from 30 healthy volunteers and 30 nasopharyngeal patients. Immediately after the spectroscopic measurement, each abnormal tissue site was biopsied and sent for histopathological examination, which is the gold standard for determining the diagnostic performance of RS and DRS techniques for differentiating NPC from normal nasopharyngeal tissues. Table 1 shows more detailed information on these patients.

Table 1 Clinical information on nasopharyngeal cancer (NPC) patients (EBV + undifferentiated type) and healthy volunteers.

	**Patients with NPC (n = 30)**	**Healthy controls (n = 30)**
**Age**		
Mean	55	52
Median	55.5	53
**Gender**		
Male	20	19
Female	10	11
**TNM stage** (seventh AJCC)		
II	5	N/A
III	10	N/A
IV	15	N/A

2.2. Instrumentation and measurements

This article is protected by copyright. All rights reserved.

Accepted Article

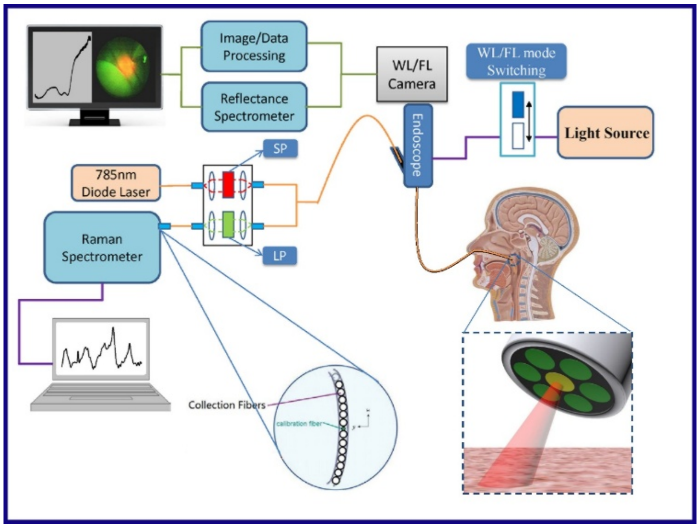

Figure 1 Schematic of the integrated endoscopic imaging and spectroscopy system including while light imaging, autofluorescence imaging, Raman spectroscopy, and diffuse reflectance spectroscopy, developed for *in vivo* tissue measurements at endoscopy. (WL – white light, FL – fluorescence, LP – long pass, SP – short pass)

Figure 1 shows the schematic of the integrated four modality endoscopy imaging and spectroscopy system. It consists of a fiber-optic catheter Raman system integrated with a special endoscopy system capable of performing white light imaging, autofluorescence imaging, and catheter free diffuse reflectance spectroscopy. The integrated system was packed onto a mobile cart for *in vivo* clinical measurements.

In brief, the special endoscopy system [8] (at the top of Fig. 1) consists of an endoscope (BF-40D, Olympus America), a light source (Xenon arc lamp, 100 W), a miniature spectrometer (USB2000, Ocean Optics) for DRS measurements, a specially designed 3 chip CCD camera, and a PC. The light source provides both broadband visible light (400-700 nm) for white light imaging/reflectance spectroscopy and strong blue light (400-460 nm, 78 mW) plus weak near-infrared (NIR) light (720-800 nm, 4 mW) for autofluorescence imaging. Imaging mode switching is realized by changing between two filters inside the light source. Inside the camera, a 45 degree mirror is placed at an interim image plane to facilitate reflectance spectral measurements, which is achieved by an optical fiber mounted at the center of the mirror surface to collect and transmit the reflected white light from the tissue surface to the spectrometer for spectral analysis. Compared to the original system setup [8], the long-pass filter (470–700 nm) at the spectrometer entrance was removed in order to acquire full range (400–700 nm) reflectance spectra. The integration time for reflectance spectral acquisition was set at 200 ms. To facilitate Raman spectral measurements, a short pass filter (cut off at 800 nm) was installed inside the light source to eliminate any residual illumination above 800 nm that interferes with the Raman wavelengths. The Raman spectroscopy system (at the bottom of Fig. 1) consists of a customized wavelength stabilized diode laser (PD-LD Inc., New Jersey), a NIR spectrograph (Acton LS-785, Princeton Instruments), an NIR-optimized, back-illuminated, deep-depletion CCD camera (1340×400 pixels, pixel size: 20 μm ×20 μm; Model PIXIS 400BR, Princeton Instruments), and a specially designed fiber optic Raman catheter.

This article is protected by copyright. All rights reserved.

Accepted Article

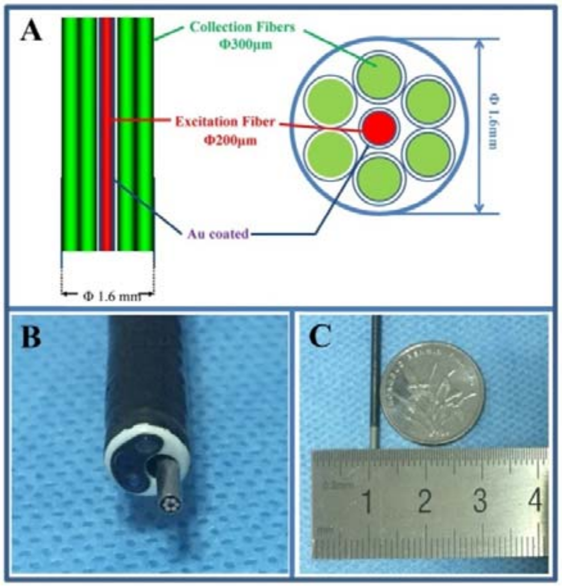

Figure 2 Details of the Raman probe. (A) Schematic of the Raman endoscopic probe; (B) Close-up view of the Raman catheter inside the instrument channel (2.2 mm diameter) of the endoscope; (C) Photo of the Raman probe (1.6 mm diameter).

The fiber-optic catheter was used for distal Raman measurement of the nasopharyngeal cavity through the instrument channel (2.2mm diameter) of the endoscope (Fig. 2B). The schematic of the Raman endoscopic probe is shown in Fig. 2A. It consists of a central excitation fiber (200 μm core diameter) and six surrounding collection fibers (300 μm core diameter) packed into a stainless steel monocoil tubing with polymer sleeve for distal detection and ensuring flexibility. The catheter is 75 cm long with a 1.6 mm outer diameter (Fig. 2C). The distal end of the catheter is coated with two different types of filters (Fig. 2A). The end of the central excitation fiber is coated with a short pass filter, allowing transmitting of 680-790 nm wavelength range for the 785 nm laser light to pass through. The surrounding 6 collection fibers are coated with long-pass filters with cut off wavelength at 805 nm, passing Raman signals in the 815-950 nm range. The short pass filter blocks the fluorescence and Raman emissions generated inside the fiber when the laser light passes through, which could interfere with NIR Raman signal detection. The long-pass filter at the end of the collection fibers blocks the reflected excitation laser light from the tissue, while allowing the tissue Raman signal to propagate to the spectrometer. The catheter was attached at its proximal end to a second set of optical filters with similar transmission characteristics, but better blocking power, to further reduce the unwanted emissions generated inside the laser conducting fiber and block residual reflected laser wavelengths collected by the collection fibers. The central excitation fiber also has a gold jacket to prevent cross-talking between the excitation and collection fibers. The collected Raman signals were coupled into the spectrograph by a special fiber bundle, of which the fibers at the spectrograph's entrance were spread out into an arc linear array to correct the spectrograph image aberrations. This unique design was reported in our previous paper [20], which could improve the resolution and signal-to-noise ratio of the Raman spectrum. The Raman system has a spectral resolution of 8 cm^{-1} and covers a spectral range of 800-1800 cm^{-1}.

Both the spectroscopy unit and the imaging unit were calibrated before each clinical measurement. The endoscope was slowly inserted into the nasal cavity by the physician with the guide of white light imaging (WLI). In current standard clinical practice, WLI is used to locate suspicious lesions, but often missed many of them due to WLI's low diagnostic sensitivity. In this study, following the WLI, the imaging unit was switched to the autofluorescence imaging (AFI) mode to search for more suspicious sites utilizing its high diagnostic sensitivity. In the AFI mode, the green region is representative of normal tissue, while the dark red area indicates diseased tissue. After that, the DRS measurement was performed under WLI image, while the RS spectra were obtained by inserting the Raman probe into the instrument channel of the endoscope until near the surface of the suspicious tissue sites. The RS spectrum was acquired with 150 mW excitation power and 1 second exposure time. The catheter was held at about 10 mm above the tissue surface based on our previous study that optimized the measurement geometries [21]. With that the laser spot size on the tissue surface is about 3 mm diameter and the calculated laser power density is within the ANSI maximum permissible exposure limit for the skin [22]. The whole procedure for each patient is less than 10 min.

2.3. Data processing and multivariate statistical analysis

In the first step, a multi-polynomial fitting algorithm developed by our group [23] was used to remove the autofluorescence background of the raw Raman data,

This article is protected by copyright. All rights reserved.

yielding the tissue Raman signals alone. Then all background-subtracted Raman spectra were normalized to the integrated area under the curve in the 400–1800 cm^{-1} wavenumber range for spectral analysis and subsequent statistical analysis. Multivariate statistical analysis based on principal component analysis (PCA) combined with linear discriminant analysis (LDA) was employed to analyze and differentiate between cancer and normal groups using data from DRS, RS, and DRS combined with RS (DRS+RS), respectively. Finally, receiver operating characteristic (ROC) curves were plotted to evaluate the diagnostic performances of DRS, RS and DRS+RS methods for NPC detection. The SPSS software package (SPSS Inc., Chicago) was used for the multivariate statistical analysis.

3. Results and discussion

3.1. While light and autofluorescence imaging

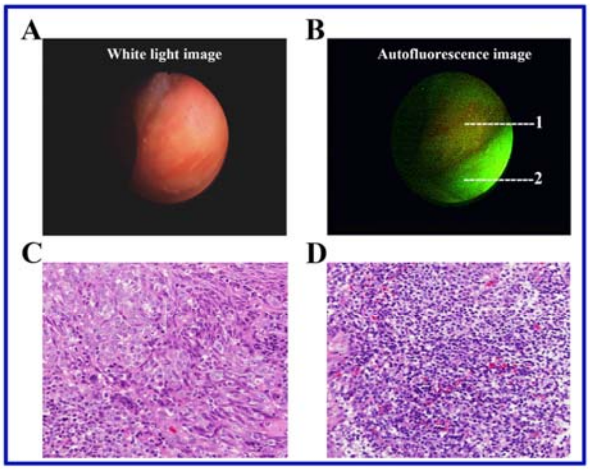

Figure 3 Example images acquired from nasopharyngeal tissue using the integrated system during endoscopic examination. (A) White light image and (B) autofluorescence image of the same location. The dark red region indicated as B1 is representative of suspicious tissue which is subsequently confirmed as stage IVA NPC by histopathological examination of biopsy from this site (C), and the green region indicated as B2 is representative of normal tissue, also confirmed by histopathological examination (D).

As the simplest imaging technology, WLI imaging is the most commonly used method for cancer screening, such as for nasopharyngeal and gastrointestinal cancers. In this study, the endoscope was inserted into the nasal cavity of a patient with the guide of WLI. Fig. 3A is a suspicious lesion determined based on the visual examination of the clinician under WLI. In routine clinical practice, part of this lesion will be randomly taken for biopsy. However, this procedure based on WLI suffers from poor sensitivity for detection of superficial cancers or precancerous lesions. Besides, it is also difficult to accurately discriminate the suspicious area from surrounding normal tissue by WLI, probably leading to unacceptably high false negative rate [24, 25]. Here, AFI method was used to further objectively locate the diseased lesion. Fig. 3B shows the fluorescence image of the same lesion presented in Fig. 3A. The green region is representative of normal tissue, while the dark red area indicates diseased tissue. Both sites were confirmed by subsequent histopathological examination (Figs. 3C and D). In contrast to reflectance WLI imaging, AFI is capable of providing important information on tissue biochemical composition and metabolic state, due to the fact that most endogenous fluorophores are related to the tissue matrix and cellular metabolic processes. When the epithelium changes from normal to cancer, there is a progressive decrease in green autofluorescence but proportionately less decrease in red fluorescence intensity, which could be attributed to the following factors: (1) a decrease in extracellular matrix in cancer; (2) high absorption of the excitation light and re-absorption of the fluorescence light by the thickened epithelium in cancer; (3) increased absorption of the blue excitation light by an increased concentration and distribution of hemoglobin; (4) a reduction in the amount of fluorophores involved in cellular metabolism, such as nicotinamide adenine dinucleotide and flavins [26]. With the use of AFI, more accurate localization of suspicious lesion could be achieved, enabling a better guidance for DRS and RS detection, and subsequent targeted biopsy. Although the diagnostic sensitivity can be significantly improved by AFI especially for the carcinoma in situ, the detection specificity of AFI in conjunction with WLI is lower compared with that of WLI alone, resulting in many false positive biopsies [8]. Therefore, spectroscopic detection methods including RS and DRS were integrated in this system for increasing the diagnostic specificity.

Accepted Article

This article is protected by copyright. All rights reserved.

Accepted Article

3.2. Raman spectroscopy and diffuse reflectance spectroscopy

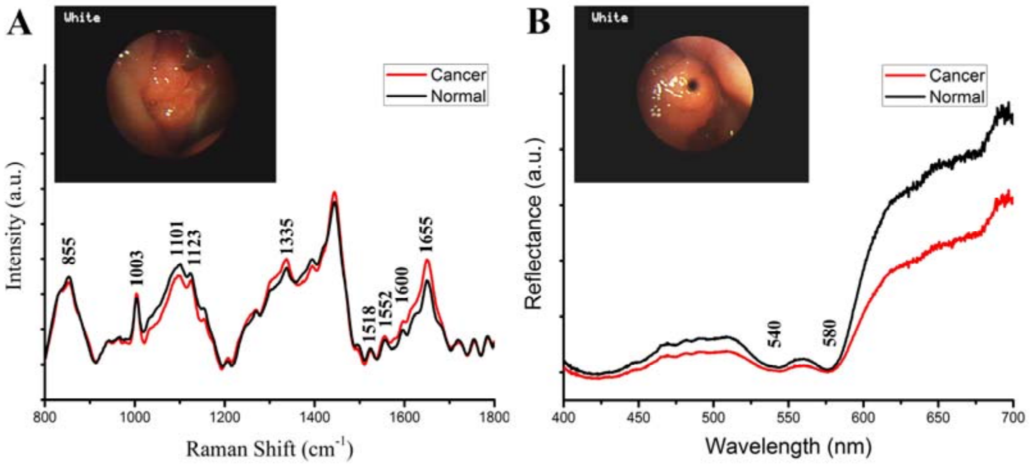

Figure 4. Comparison of the mean Raman spectrum (A) and diffuse reflectance spectrum (B) from normal and cancer tissue sites. The insert pictures show the views of Raman spectra detection and diffuse reflectance spectra detection under white light imaging, respectively.

High quality Raman spectra of tissue sites were obtained using specially-designed Raman probe under *in vivo* endoscopic examination. In the range of 800-1800 cm^{-1}, Raman spectral features and intensity are dominated by a variety of biomolecules, such as nucleic acids, proteins and lipids, making it possible to explore the biomolecular and structural changes in tissue associated with NPC transformation. Figure 4A shows a comparison of the mean Raman spectra from normal and NPC tissue sites. The prominent Raman peaks located at around 855, 1003, 1101, 1123, 1335, 1450, 1518, 1552, 1600 and 1655 cm^{-1} were consistently observed in both normal and cancer groups, though the peak intensity differences between them were still remarkable. For example, the relative peak intensities at 1101 cm^{-1} attributed to phospholipids showed lower percentage signals for cancer tissue compared to the normal, indicating a decreased concentration of phospholipids relative to the total Raman-active components in cancer sites. Similar changes in phospholipids has been previously reported in gastric precancer tissue *in vivo* using Raman spectroscopy and in *ex vivo* oral cancer tissue by chemical extraction method [27, 28]. The alteration in phospholipids is considered to be associated with cancer transformation due to the fact that cellular responses to external stimulations are mediated by membrane lipids. A decreased concentration of phospholipids is probably due to the increased phospholipid degradation and the decreased concentration of free fatty acids in tumor tissues [27]. In contrary to the decrease of the 1101 cm^{-1} peak, the Raman signals at 1600 cm^{-1} and 1655 cm^{-1} were found to be higher in cancer tissue, suggesting an increase in the percentage of phenylalanine and proteins in the a-helix conformation, respectively. This is in agreement with studies on gastric precancerous and lung cancer tissues [28, 29]. In addition, there was also a relative intensity increase of nucleic acids band (1335 cm^{-1}) in the cancer group, and this was probably due to the apoptosis and necrosis of cancer cells in the tumor microenvironment, leading to the abnormal metabolism of DNA or RNA bases in cancer tissue [30]. This observation is consistent with the *ex vivo* NPC tissue analysis using label-free Raman imaging technology [31]. Other peaks, such as 855, 1003, 1123, 1518 and 1552 cm^{-1} can be assigned to proline, phenylalanine, proteins, carotenoid and tryptophan, respectively [29].

Figure 4B shows the mean reflectance spectra from normal and NPC tissues. Although there was a close consistency between the spectral patterns of these two groups, spectral intensities changes were detectable. In the whole spectral range of 400-700 nm, the normal group showed higher intensity than the cancer group, and this difference became lager for wavelengths above 600 nm. Besides, compared with the cancer group, the hemoglobin absorption valleys at around 540 nm and 580 nm are larger and more obvious in normal spectrum,

This article is protected by copyright. All rights reserved.

Accepted Article

which indicates hypoxia in cancer sites (less oxygen in blood) and is in agreement with our previous study on lung cancer [11]. These results based on RS and DRS indicate that there are significant changes in the percentage of biomolecules between cancer and normal tissue, suggesting a diagnostic potential of RS and DRS for NPC detection.

3.3. Diagnostic statistical analysis

It should be noted that the simplistic peak intensity analysis above only utilized limited spectral information, and there were significant variations and overlapping intensities of Raman/reflectance spectra of normal and cancer tissues among subjects. Thus, a multivariate statistical analysis based on PCA-LDA [32] was employed to incorporate the entire spectrum and automatically determine the most diagnostically significant features for improving the diagnostic efficiency for NPC. In this work, PCA was used first to reduce the about 1000 intensity variables within the raw tissue Raman spectrum to a few PCs, which were linear combination of the original variables and retained the most diagnostically significant information for tissue differentiation. T-test analysis showed that three PCs (PC1, PC3 and PC6) of the variances were the most diagnostically significant ($p < 0.05$), and then the collected PC scores were retained and fed into an LDA model for optimally differentiating the cancer group from normal group. Similarly, PC1, PC2 and PC3 were the most diagnostically significant variances for tissue discrimination using DRS spectra. Figure 5A-C displayed the posterior probabilities belonging to the normal and cancer groups as calculated in the LDA model using DRS, RS and DRS+RS methods, respectively. Using a discrimination threshold of 0.5, the diagnostic sensitivity for detecting NPC was 81.6% (115/141), 89.4% (126/141) and 98.6 % (139/141) for DRS, RS and DRS+RS, respectively. And the corresponding diagnostic specificities for each method were 73.2% (104/142), 92.3% (131/142) and 95.1% (135/142), respectively. These classification results were summarized in Table 2.

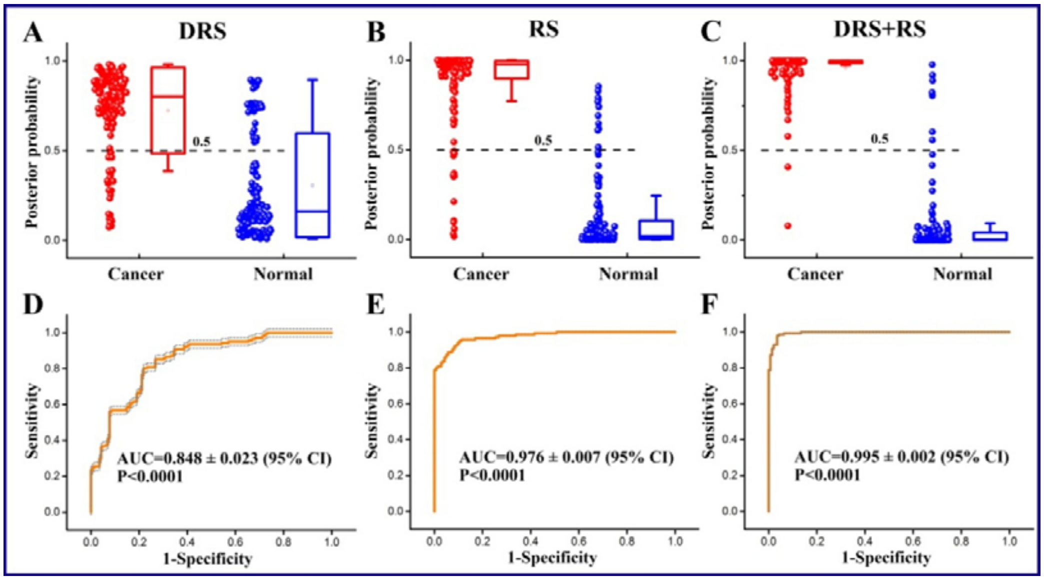

Figure 5 (A-C) Scatter plots of the posterior probabilities belonging to the normal and cancer groups derived from PCA-LDA algorithms based on DRS, RS and DRS+RS spectral data, respectively. Box represents the range of 25%-75%; Whisker represents the range of minimum to maximum. The diagnostic threshold (dotted lines) is 0.5.; (D-F) Corresponding receiver operating characteristic (ROC) curves of classification results for NPC diagnosis. AUC: the integrated areas under the ROC curves. CI: confidence interval.

This article is protected by copyright. All rights reserved.

Accepted Article

Table 2 Prediction Results for differentiating cancer from normal tissue sites using PCA-LDA diagnostic algorithms derived from DRS, RS and DRS+RS spectral data, respectively.

Method	Predicted parameters		
	Sensitivity	Specificity	Accuracy
Diffuse reflectance spectroscopy (DRS)	81.6% (115/141)	73.2% (104/142)	77.4% (219/283)
Raman spectroscopy (RS)	89.4% (126/141)	92.3% (131/142)	90.8% (257/283)
Diffuse reflectance combined with Raman spectroscopy (DRS+RS)	98.6% (139/141)	95.1% (135/142)	96.8% (274/283)

It is found that a diagnostic accuracy of 90.8% for differentiating NPC from normal tissue can be achieved using the RS method, an almost 13% improvement compared to the DRS method. Moreover, the diagnostic accuracy can be improved further to 96.8% for the combination of DRS and DS data. The integrated area under the ROC curves (AUC) is 0.995 ± 0.002 for DRS+RS method, which is higher than that for DRS (0.848 ± 0.023) and RS (0.976 ± 0.007) alone (Figs. 5D-F). These ROC results confirm that DRS+RS leads to better diagnostic performance for differentiation of NPC cancer from normal tissue sites. In contrast to some previous studies only using single spectroscopic method [10-12], the novel dual spectroscopic detection method developed here shows powerful diagnostic performance for *in vivo* endoscopic cancer detection, due to the fact that more biochemical and morphological information associated with cancer transformation can be comprehensively explored within one endoscopic examination.

4. Conclusion

An integrated four-modality endoscopy system combining white light imaging, autofluorescence imaging, diffuse reflectance spectroscopy, and Raman spectroscopy technologies was developed for *in vivo* endoscopic cancer detection for the first time. A pilot clinical test of the system for NPC detection was conducted. Spectral data were collected from 30 NPC patients and 30 healthy volunteers. Both high diagnostic sensitivity (98.6%) and high specificity (95.1%) for differentiating cancer from normal tissue sites were achieved using this system combined with PCA-LDA diagnostic algorithm, due to its ability of comprehensively capturing diagnostic information from morphological changes and biomolecular changes, such as epithelial layer thickening, endogenous fluorophores, hemoglobin, phospholipids, proteins and nucleic acid. The preliminary results from this study demonstrated great potential of this endoscopy system combined with PCA-LDA technique to be a rapid, objective and accurate optical biopsy for nasopharyngeal cancer screening. In next step, we will conduct a larger clinical trial to verify the reliability of this system. And more optical technologies, such as surface enhanced Raman spectroscopy will also be integrated with this system to further improve its efficiency in cancer detection.

Conflicts of interest

The authors declare no competing financial interest.

This article is protected by copyright. All rights reserved.

Accepted Article

Acknowledgements

This work was supported by the National Natural Science Foundation of China (No. U1605253, 61210016, 61405036, 61575043), the Innovation Team Development Plan by the Ministry of Education of China (No. IRT_15R10), the Natural Science Foundation of Fujian, China (No. 2017J01499, 2014J01227, 2015J01436), the Key Clinical Specialty Discipline Construction Program of Fujian, China, the National Clinical Key Specialty Construction Program of China, and the Canadian Institutes of Health Research (grant # 201603PJT-364146-PJT-CAAA-60404).

References

[1] L.A. Torre, F. Bray, R.L. Siegel, J. Ferlay, J. Lortet-Tieulent, A. Jemal, CA Cancer J Clin, **2015,** 65, 87.
[2] J.P. Bruce, K. Yip, S.V. Bratman, E. Ito, F.-F. Liu, J Clin Oncol, **2015,** 33, 3346.
[3] K. Chan, E.C. Hung, J.K. Woo, P.K. Chan, S.F. Leung, F. Lai, et al., Cancer, **2013,** 119, 1838.
[4] M.L. Chua, J.T. Wee, E.P. Hui, A.T. Chan, Lancet, **2016,** 387,
[5] D. Lin, J. Pan, H. Huang, G. Chen, S. Qiu, H. Shi, et al., Sci Rep, **2014,** 4, 4751.
[6] S. Lam, T. Kennedy, M. Unger, Y.E. Miller, D. Gelmont, V. Rusch, et al., Chest, **1998,** 113, 696.
[7] T.D. Wang, D.J. Van, J.M. Crawford, E.A. Preisinger, Y. Wang, M.S. Feld, Gastroenterology, **1996,** 111, 1182.
[8] H. Zeng, M. Petek, M.T. Zorman, A. McWilliams, B. Palcic, S. Lam, Opt Lett, **2004,** 29, 587.
[9] P.N. Chhajed, K. Shibuya, H. Hoshino, M. Chiyo, K. Yasufuku, K. Hiroshima, et al., European Respiratory Journal, **2005,** 25,
[10] J.W. Spliethoff, W. Prevoo, M.A. Meier, J. de Jong, D.J. Evers, H.J. Sterenborg, et al., Clin Cancer Res, **2016,** 22, 357.
[11] Y.S. Fawzy, M. Petek, M. Tercelj, H. Zeng, J Biomed Opt, **2006,** 11, 044003.
[12] Z. Huang, S.K. Teh, W. Zheng, K. Lin, K.Y. Ho, M. Teh, et al., Biosens Bioelectron, **2010,** 26, 383.
[13] S. Feng, R. Chen, J. Lin, J. Pan, G. Chen, Y. Li, et al., Biosens Bioelectron, **2010,** 25, 2414.
[14] H. Lui, J. Zhao, D. McLean, H. Zeng, Cancer res, **2012,** 72, 2491.
[15] C. Kallaway, L.M. Almond, H. Barr, J. Wood, J. Hutchings, C. Kendall, et al., Photodiagn Photodyn Ther, **2013,** 10, 207.
[16] M.A. Short, S. Lam, A.M. McWilliams, D.N. Ionescu, H. Zeng, J Thorac Oncol, **2011,** 6, 1206.
[17] H.C. McGregor, M.A. Short, A. McWilliams, T. Shaipanich, D.N. Ionescu, J. Zhao, et al., J Biophotonics, **2017,** 10, 98.
[18] M.S. Bergholt, W. Zheng, K. Lin, K.Y. Ho, M. Teh, K.G. Yeoh, et al., Biosens Bioelectron, **2011,** 26, 4104.
[19] K. Lin, W. Zheng, C.M. Lim, Z. Huang, Biomed Opt Express, **2016,** 7, 3705.
[20] Z. Huang, H. Zeng, I. Hamzavi, D.I. McLean, H. Lui, Opt Lett, **2001,** 26, 1782.
[21] M.A. Short, S. Lam, A. Mcwilliams, J. Zhao, H. Lui, H. Zeng, Opt Lett, **2008,** 33, 711.
[22] ANSI, American national standard for the safe use of lasers, American National Standards Institute ANSI Standard Z1361-2014 Washington, DC: American National Standards Institute 2014.
[23] J. Zhao, H. Lui, D.I. McLean, H. Zeng, Appl Spectrosc, **2007,** 61, 1225.
[24] S.J. Chung, D. Kim, J.H. Song, H.Y. Kang, G.E. Chung, J. Choi, et al., Gut, **2013,** 0, 1.
[25] C. Lee, C. Chang, Y. Lee, C. Tai, W. Wang, P.-H. Tseng, et al., Endoscopy, **2010,** 42, 613.
[26] H. Zeng, A. McWilliams, S. Lam, Photodiagn Photodyn Ther, **2004,** 1, 111.
[27] K. Kolanjiappan, C. Ramachandran, S. Manoharan, Clin Biochem, **2003,** 36, 61.
[28] S. Teh, W. Zheng, K. Ho, M. Teh, K. Yeoh, Z. Huang, Br J Cancer, **2008,** 98, 457.
[29] Z. Huang, A. McWilliams, H. Lui, D.I. McLean, S. Lam, H. Zeng, Int J Cancer, **2003,** 107, 1047.
[30] H. Schwarzenbach, D.S. Hoon, K. Pantel, Nat Rev Cancer, **2011,** 11, 426.
[31] S. Feng, J. Lin, M. Cheng, Y.-Z. Li, G. Chen, Z. Huang, et al., Appl Spectrosc, **2009,** 63, 1089.
[32] A. Sheini, H. Khajehsharifi, M. Shahbazy, M. Kompany-Zareh, Sens Actuators, B, **2017,** 242, 288.

This article is protected by copyright. All rights reserved.
