# Supplementary material for: The accuracy of Fiber-Optic Raman Spectroscopy in the detection and diagnosis of head and neck neoplasm in vivo: a systematic review and meta-analysis
Source: PeerJ. 2023 Dec 11;11:e16536. doi: 10.7717/peerj.16536 (PMC10720414; doi:10.7717/peerj.16536)
Supplement: Data S1 [file peerj-11-16536-s001.zip › article/lin2017.pdf]

IVYSPRING INTERNATIONAL PUBLISHER

Theranostics

2017; 7(14): 3517-3526. doi: 10.7150/thno.16359

Research Paper

Real-time *In vivo* Diagnosis of Nasopharyngeal Carcinoma Using Rapid Fiber-Optic Raman Spectroscopy

Kan Lin[1,2], Wei Zheng[1,2], Chwee Ming Lim[3], and Zhiwei Huang[1]✉

1. Optical Bioimaging Laboratory, Department of Biomedical Engineering, Faculty of Engineering, National University of Singapore, Singapore 117576;
2. Department of Medicine, Yong Loo Lin School of Medicine, National University of Singapore and National University Health System, Singapore 119260;
3. Department of Otolaryngology, Head and Neck Surgery, National University of Singapore and National University Health System, Singapore 119074.

✉ Corresponding author: Dr. Zhiwei Huang, Optical Bioimaging Laboratory, Department of Biomedical Engineering, Faculty of Engineering, National University of Singapore, 9 Engineering Drive 1, Singapore 117576, Phone: +65 6516 8856; Fax: +65 6872 3069; E-mail: biehzw@nus.edu.sg

© Ivyspring International Publisher. This is an open access article distributed under the terms of the Creative Commons Attribution (CC BY-NC) license (https://creativecommons.org/licenses/by-nc/4.0/). See http://ivyspring.com/terms for full terms and conditions.

Received: 2016.06.02; Accepted: 2017.07.23; Published: 2017.08.18

Abstract

We report the utility of a simultaneous fingerprint (FP) (i.e., 800–1800 cm^{-1}) and high-wavenumber (HW) (i.e., 2800–3600 cm^{-1}) fiber-optic Raman spectroscopy developed for real-time *in vivo* diagnosis of nasopharyngeal carcinoma (NPC) at endoscopy. A total of 3731 high-quality *in vivo* FP/HW Raman spectra (normal=1765; cancer=1966) were acquired in real-time from 204 tissue sites (normal=95; cancer=109) of 95 subjects (normal=57; cancer=38) undergoing endoscopic examination. FP/HW Raman spectra differ significantly between normal and cancerous nasopharyngeal tissues that could be attributed to changes of proteins, lipids, nucleic acids, and the bound water content in NPC. Principal components analysis (PCA) and linear discriminant analysis (LDA) together with leave-one subject-out, cross-validation (LOO-CV) were implemented to develop robust Raman diagnostic models. The simultaneous FP/HW Raman spectroscopy technique together with PCA-LDA and LOO-CV modeling provides a diagnostic accuracy of 93.1% (sensitivity of 93.6%; specificity of 92.6%) for nasopharyngeal cancer identification, which is superior to using either FP (accuracy of 89.2%; sensitivity of 89.9%; specificity of 88.4%) or HW (accuracy of 89.7%; sensitivity of 89.0%; specificity of 90.5%) Raman technique alone. Further receiver operating characteristic (ROC) analysis reconfirms the best performance of the simultaneous FP/HW Raman technique for *in vivo* diagnosis of NPC. This work demonstrates for the first time that simultaneous FP/HW fiber-optic Raman spectroscopy technique has great promise for enhancing real-time *in vivo* cancer diagnosis in the nasopharynx during endoscopic examination.

Key words: Fiber-optic Raman spectroscopy, *In vivo* diagnosis, Nasopharyngeal carcinoma

Introduction

Nasopharyngeal carcinoma (NPC) arising from the epithelial lining of nasopharynx is one of the most common malignancies in Southeast Asia and Southern China [1-3]. The major etiological factors related to NPC pathogenesis include genetic susceptibility, environment factors and Epstein-Barr virus (EBV) infection [4]. The incidence rates of NPC are more prevalent in Southeast Asia, which can be as high as 21.4 (China and East Asia) and 31.5 per 100,000 (Singapore) persons per year [5]. Early detection and localization with immediate removal and treatment (e.g., chemo-radiotherapy) is crucial to improving survival rates of patients with NPC [6, 7]. The current routine NPC diagnosis based on conventional white-light reflectance (WLR) endoscopy is clinically challenging. This is due to the fact that WLR endoscopy relies heavily on visual identification of gross morphologic changes, but WL

endoscopic presentations (i.e., structural and morphological details) of early NPC lesions may be indistinguishable from surrounding normal or benign tissue, resulting in low detection accuracy [8]. Most patients with NPC are diagnosed at advanced stages due to non-specific presenting symptoms observed under clinical nasopharynx examination [3, 9]. Well-performed but randomized endoscopic biopsies followed by formalin fix, tissue cryostat sectioning and hematoxylin and eosin (H&E) staining for microscopic examination by the pathologists remain the current gold standard for NPC diagnosis [8, 10]. However, clinical manifestations (e.g., suspicious nasopharyngeal mass, epistaxis and neck mass, etc.) in the nasopharynx are common features, excessively random biopsies have not been proved to significantly improve diagnostic accuracy of early cancer and cancer in the nasopharynx [11]. Hence, there is profound clinical need to develop a rapid, objective diagnostic technique for targeted biopsies to reduce the random sampling errors, and ultimately to improve the detection accuracy of NPC *in vivo* with molecular specificity.

Raman spectroscopy is an optical vibrational technique based on the fundamental principle of inelastic light scattering [12]. When incident laser light induces a polarization change of molecules, a small proportion of incident photons (~1 in 10^8) is inelastically scattered with the frequency shifts corresponding to the specific Raman-active vibrational modes of molecules in the samples [12]. Raman spectroscopy hence is capable of revealing biochemical and biomolecular structures and conformation of tissue, providing the unique opportunity to distinguish different pathological tissue types at the molecular level. In the so-called fingerprint (FP) spectral range (i.e., 800–1800 cm^{-1}), the unrivaled advantage of the FP Raman spectroscopy technique stems from its capability of identifying specific information about backbone structures of proteins, lipids and nucleic acid assemblies in cells and tissue [13-27]. The efficiency of the FP Raman spectroscopy technique could be compromised owing to extremely weak tissue Raman signals but overwhelming tissue autofluorescence (AF) background. Recent attentions have been directed towards the use of high-wavenumber (HW) (i.e., 2800–3600 cm^{-1}) regime, as the HW Raman exhibits stronger tissue Raman signals with less AF interference [28-35]. Thus, clinical rationales for combining FP and HW Raman spectroscopy for improving NPC diagnosis are manifold: (i) for nasopharyngeal tissue that exhibits intense AF overwhelming the weak tissue FP Raman signal, the HW Raman spectroscopy could still provide stronger tissue HW Raman peaks for NPC diagnosis; (ii) by offering complementary biochemical/biomolecular information, the simultaneous FP and HW Raman spectroscopy could have advantages for improving tissue characterization and diagnosis [32-35]. To date, simultaneous FP and HW Raman spectroscopy for *in vivo* NPC diagnosis has not yet been reported. Some preliminary *ex vivo* Raman studies on nasopharyngeal tissue samples using bulky commercial micro-Raman spectrograph systems have shown encouraging diagnostic accuracies of 85~90% for NPC classifications [27, 36, 37]. In this work, with our successful development of a rapid fiber-optic Raman spectroscopy system capable of simultaneously acquiring both the fingerprint (FP) (i.e., 800–1800 cm^{-1}) and high-wavenumber (HW) (i.e., 2800–3600 cm^{-1}) tissue Raman spectra *in vivo* at endoscopy [32-35], we aim to investigate the clinical utility of applying this unique simultaneous FP and HW fiber-optic Raman endoscopic technique for *in vivo* nasopharyngeal tissue Raman measurements for targeted biopsies, and ultimately for enhancing real-time *in vivo* diagnosis of NPC during clinical endoscopic examinations. We employ principal components analysis (PCA) and linear discriminant analysis (LDA) techniques to develop robust diagnostic algorithms for nasopharyngeal cancer diagnosis and classification. We have also examined the diagnostic performance of FP Raman, HW Raman, and the integrated FP/HW Raman techniques for NPC detection. The receiver operating characteristic (ROC) curve is further generated to assess the clinical performance of simultaneous FP/HW Raman spectroscopy for improving *in vivo* diagnosis of NPC at endoscopy.

Materials and Methods

Clinical fiber-optic Raman endoscopy instrumentation

We have developed a simultaneous FP and HW fiber-optic Raman spectroscopy technique for *in vivo* tissue Raman measurements at endoscopy [32-35]. Briefly, the fiber-optic Raman spectroscopic system consists of a near-infrared (NIR) diode laser (λ_{ex}=785 nm; maximum output: 300 mW, B&W TEK Inc.), a high-throughput reflective imaging spectrograph (Acton LS-785 f/2, Princeton Instruments Inc.) equipped with a customized gold-coated 830 gr/mm grating, an NIR-optimized charge-coupled device (CCD) camera (PIXIS: 400BR-eXcelon, Princeton Instruments Inc.) and a specially designed fiber-optic Raman probe. The system acquires tissue Raman spectra in the spectral range from 400-3600 cm^{-1} with a resolution of ~8 cm^{-1}. The specially designed and

fabricated 1.9 m long fiber-optic Raman probe (~1.8 mm in outer diameter) is used for both laser light delivery and *in vivo* epithelial tissue Raman signal collection at endoscopy. The compact fiber-optic Raman endoscopic probe comprises 18 × 200 μm beveled collection fibers (low-OH fused silica, numerical aperture (NA)=0.22) surrounding the central light delivery fiber (low-OH fused silica, 200 μm in diameter, NA=0.22). A 1.0 mm sapphire ball lens is coupled to the fiber tip of the probe to tightly focus the excitation light onto the tissue subsurface, enabling the effective Raman spectrum collection from the epithelial lining [35]. The depth-selective capability of the fiber-optic Raman spectroscopy technique ensures the shallower tissue interrogation (<200 μm) with tissue probing volume of <0.02 mm^3, thereby reducing the interferences and signal dilution from deeper stromal tissues while selectively interrogating the epithelium associated with neoplastic onset and progression [38]. The 785-nm laser power incident on the tissue surface is ~12 mW (equivalent to ~1.5 W/cm^2 within the spot size of ~300 μm) permissible by the American National Standards Institute (ANSI) [39]. At the proximal end of the Raman probe, the excitation and emission fibers were coupled into two separate in-line filter modules: one integrated with a narrow band-pass filter (LL01-785, Semrock, Inc.) for suppressing laser noise, and the other integrated with an edge long-pass filter (LP02-785RU, Semrock, Inc.) for further reduction of the scattered laser light while permitting the scattered-tissue Raman signals to transmit into the Raman spectrograph. The atomic emission lines of mercury−argon spectral calibration lamps (HG-1 and AR-1, Ocean Optics, Inc., Dunedin, FL) are used for wavelength calibration in the FP range, and 4-acetamidophenol that exhibits strong well-defined peaks at 2931 cm^{-1} and 3064 cm^{-1} (ASTM E1840 standard) is used for the HW Raman calibration [34]. All wavelength-calibrated spectra were corrected for the wavelength dependence of the system using a calibration lamp (RS-10, EG&G Gamma Scientific, San Diego, CA). The entire FP/HW fiber-optic Raman endoscopic system is controlled using a foot pedal in an intuitive software framework with auditory probabilistic feedback to the clinicians in real-time [25].

Clinical trial protocol

The ethical protocol of the present study was approved by the Institutional Review Board (IRB) of the National Healthcare Group (NHG) of Singapore (NHG DSRB Ref No: 2014/00323). Prior to Raman measurements, all patients signed individual informed consent permitting the *in vivo* Raman spectroscopic measurements during nasopharyngeal endoscope examinations conducted in the head and neck clinic at the National University Health System (NUHS), Singapore. A total of 95 subjects (68 men and 27 women with mean ages of ~52; healthy volunteers=57; NPC patients=38) were recruited in the Raman endoscopic examinations. The Raman probe passed down to the instrument channel of a medical rhinolaryngoscope under the WLR imaging guidance. The Raman probe tip was visible approximately 5 mm in front of the endoscope camera. During endoscopic examination of suspicious lesions, the Raman probe was placed in gentle contact with the nasopharyngeal mucosa surface, and the positioning of the Raman probe against the tissue sites was verified on the computer monitor by the endoscopists in charge at endoscopy. Multiple Raman spectra (~15 to 20) for each tissue site were measured within 0.5 s, allowing a rapid survey of large tissue areas. In this work, the healthy subjects measured by FP/HW Raman spectroscopy do not undergo invasive biopsies but are assumed to be normal based on otolaryngologists' observations; Only the suspicious tissue sites from NPC patients measured by Raman spectroscopy are biopsied, and then fixed in formalin, sectioned and stained with H&E for histopathology examinations by three independent pathologists who are blinded to the Raman results. One notes that the suction markings have been made on the tissue sites measured during *in vivo* Raman measurements to ensure that the tissue biopsies are taken exactly from the tissue sites measured for histopathology examination. The inter-agreement among pathologists is assessed using Cohen's Kappa statistics (k>0.9) [40]. Only consensus histopathology assessments on the biopsied tissues are included, serving as the standard to determine the diagnostic ability of FP/HW fiber-optic Raman technique for identifying cancer from normal tissue in the nasopharynx.

Data preprocessing and statistical analysis

The measured nasopharyngeal tissue spectra represented a combination of weak tissue Raman scattering, intense AF background and the noise. The spectra were pre-processed by a third-order Savitzky-Golay smoothing filter (a window width of 5 pixels) to reduce the noise. Due to the simplicity and effectiveness, the modified multi-polynomial fitting method was adopted to remove AF background in the preprocessed spectra [25, 41]. The iterative fifth-order polynomial fitting was found to be optimal for fitting the AF background in the noise-smoothed spectrum over the range of 800-1800 cm^{-1}, and this polynomial was then subtracted from the measured FP spectrum

to yield the FP tissue Raman spectrum alone [21, 25]. While in the HW range (2800-3600 cm^{-1}), a first-order polynomial fit was used for removing AF background. The tissue FP/HW Raman spectrum was finally normalized to the integrated area of the entire FP and HW range to compare the differences in spectral shapes and relative Raman band intensities between normal and cancerous nasopharyngeal tissue. As there are no obvious tissue Raman peaks observed in the 1800-2800 cm^{-1} range, this Raman silent region is therefore excluded for data analysis. All the preprocessing is completed within 30 ms, and the processed Raman spectra and diagnostic outcomes can be displayed on the computer screen in real-time, facilitating clinical Raman endoscopic examinations [25].

Principal components analysis (PCA) and linear discriminant analysis (LDA) were implemented to develop robust diagnostic algorithms for the differentiation between normal and cancerous nasopharyngeal tissues. Leave-one subject-out, cross-validation (LOOCV) was utilized to evaluate the diagnostic models developed in an unbiased manner [42, 43]. To minimize the influence of measurement variations due to patient's breathing and organ motions as well as fiber probe handlings during *in vivo* clinical Raman measurements, multiple Raman spectra (~15 to 20) are acquired from each tissue site within 0.5 s, and the majority voting strategy is used for final tissue classification for each tissue site measured. Receiver operating characteristic (ROC) curves were also generated by successively changing the thresholds to determine the correct and incorrect classifications for normal and NPC tissues. All the spectra preprocessing and multivariate statistical analysis were performed in real-time using in-house written scripts in the MATLAB-programming environment [25].

Results

A total of 3731 high-quality *in vivo* FP/HW Raman spectra (normal=1765; cancer=1966) were successfully acquired from 204 nasopharyngeal tissue sites (normal=95; cancer=109) of the 95 subjects recruited (normal=57; NPC=38) in real-time during endoscopic examination. Fig. 1A shows the mean *in vivo* FP/HW Raman spectra ± 1 standard deviations (SD) (shaded area) of normal and cancerous nasopharyngeal tissue. The corresponding images of the simultaneous FP/HW Raman procedures under white-light reflectance rhinolaryngoscopy guidance are also shown in Fig. 1. Prominent nasopharyngeal tissue Raman peaks can be observed in the FP range, i.e., 853 cm^{-1} (ν(C-C) proteins), 875 cm^{-1} (v(C-C) stretching of hydroxyproline), 1078 cm^{-1} (ν(C-C) of lipids), 1209 cm^{-1} (tryptophan and phenylalanine),1265 cm^{-1} (amide III ν(C-N) and δ(N-H) of proteins), 1335 cm^{-1} (CH_3CH_2 twisting of proteins and nucleic acids), 1445 cm^{-1} ($\delta(CH_2)$ deformation of proteins and lipids), 1554 cm^{-1} (ν(C=C) of amide III), 1580 cm^{-1} (δ(C=C) of phenylalanine) and 1655 cm^{-1} (amide I ν(C=O) of lipids) [13-24, 44]. Meanwhile, intense tissue Raman peaks are also observed in the HW range, i.e., 2850 and 2885 cm^{-1} (symmetric and asymmetric CH_2 stretching of lipids), 2940 cm^{-1} (CH_3 stretching of proteins), 3009 cm^{-1} (asymmetric =CH stretching of lipid), ~3300 cm^{-1} (amide A (NH stretching of proteins)) and the broad Raman bands of water (OH stretching vibrations peaking at ~3250 and ~3400 cm^{-1}) that are related to the local conformation and interactions of OH-bonds in the intracellular and extracellular space of nasopharyngeal tissue [28-31, 34, 35]. Fig. 1B shows the difference Raman spectra (i.e., cancer - normal) ± 1SD (shaded area), reflecting different Raman-active components' contribution associated with nasopharyngeal carcinogenesis *in vivo*, confirming the potential of FP/HW Raman spectroscopy for early diagnosis of NPC tissue at endoscopy. Figure 2 shows the representative H&E-stained histopathologic slide of NPC from nasopharyngeal tissue biopsied, delineating the syncytial sheet of undifferentiated cancer cells with oval or round vesicular nuclei and prominent nucleoli as observed in nasopharyngeal carcinoma tissue (magnification: x200).

To gain deeper insights into the underlying molecular biomarkers accompanied with nasopharyngeal neoplastic transformation, PCA-LDA together with Student's *t*-test are implemented on the *in vivo* tissue Raman spectra acquired (Fig. 1) to evaluate the elusive differences observed in the Raman spectra of different tissue types. The diagnostically significant PCs (unpaired two-sided Student's *t*-test, p<0.01) as shown in Fig. 3 account for 64.2% (PC1), 18.9% (PC2) and 7.5% (PC3) of Raman spectral variations, respectively. Some distinct features (e.g., peaks, troughs, and spectral shapes) of different significant PCs in Fig. 3 are similar to those of tissue Raman spectral patterns in Fig. 1. The first significant PC accounts for the largest variance within the spectral data sets (i.e., 64.2%), whereas successive PCs describe features that contribute progressively smaller variances. To further develop sophisticated multivariate spectral diagnostic algorithms, all the three diagnostically significant PCs are fed into the LDA model together with leave-one subject-out, cross-validation (LOOCV) technique for tissue classification. In each loop of validation, Raman spectra from one subject were excluded as the

validation dataset and the remaining spectral dataset were fed into algorithms as the training dataset. Fig. 4 shows the scattered plots of posterior probability of Raman predictions (i.e., normal and NPC) using PCA-LDA/LOOCV modeling and majority voting for (a) integrated FP/HW, (b) FP, and (c) HW, respectively. The diagnostic accuracy with simultaneous FP/HW Raman spectroscopy for NPC identification is 93.1% (sensitivity of 93.6%; specificity of 92.6%), which is superior to using either FP 89.2% (sensitivity of 89.9%; specificity of 88.4%) or HW 89.7% (sensitivity of 89.0%; specificity of 90.5%) Raman technique alone. The receiver operating characteristic (ROC) curves are also generated (Fig. 5), with the integration areas under the ROC curves of being 0.983, 0.961 and 0.960, respectively, for the simultaneous FP/HW, FP and HW Raman techniques.

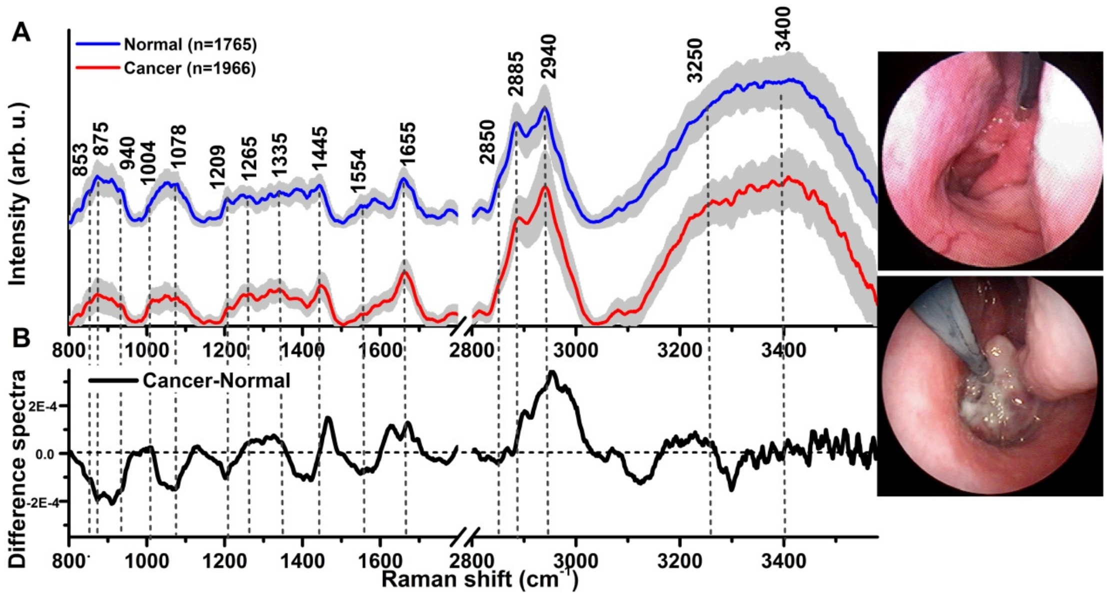

Fig 1. (A) The mean *in vivo* FP/HW Raman spectra ±1 standard deviations (SD) of normal (n=1765) and NPC (n=1966) acquired from 204 tissue sites (normal=95; carcinoma=109) of 95 subjects during clinical endoscopy. (B) Difference Raman spectra (i.e., carcinoma - normal) ± 1 SD resolving the distinct spectral features of NPC. The corresponding images of the simultaneous FP/HW Raman procedures under white-light reflectance endoscopic imaging guidance are also shown.

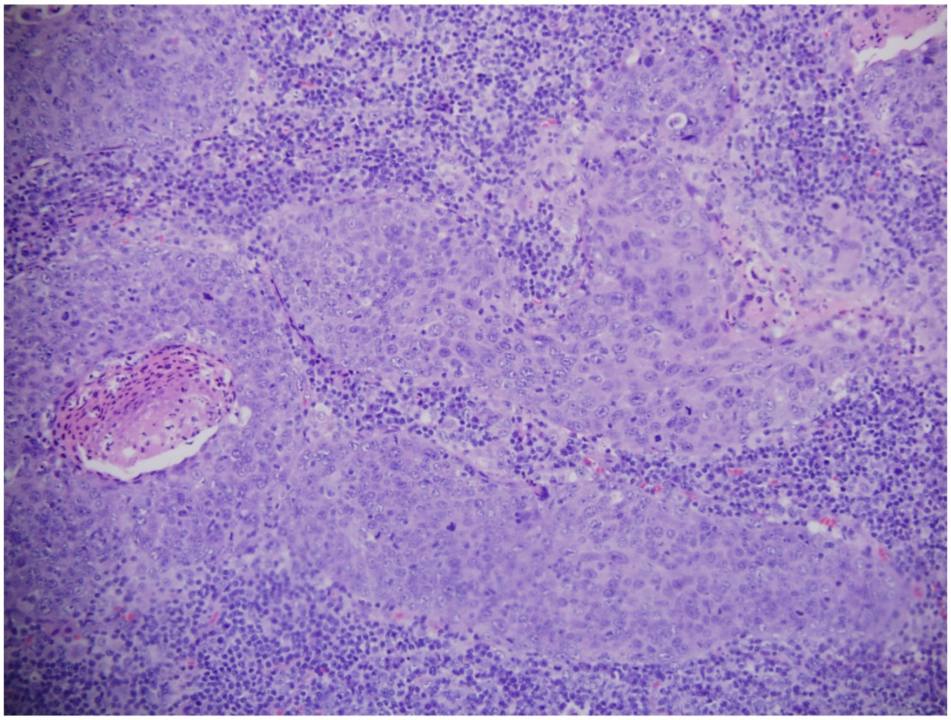

Fig. 2. Representative hematoxylin and eosin (H&E)-stained histopathologic slide of undifferentiated carcinoma of NPC tissue (magnification: ×200).

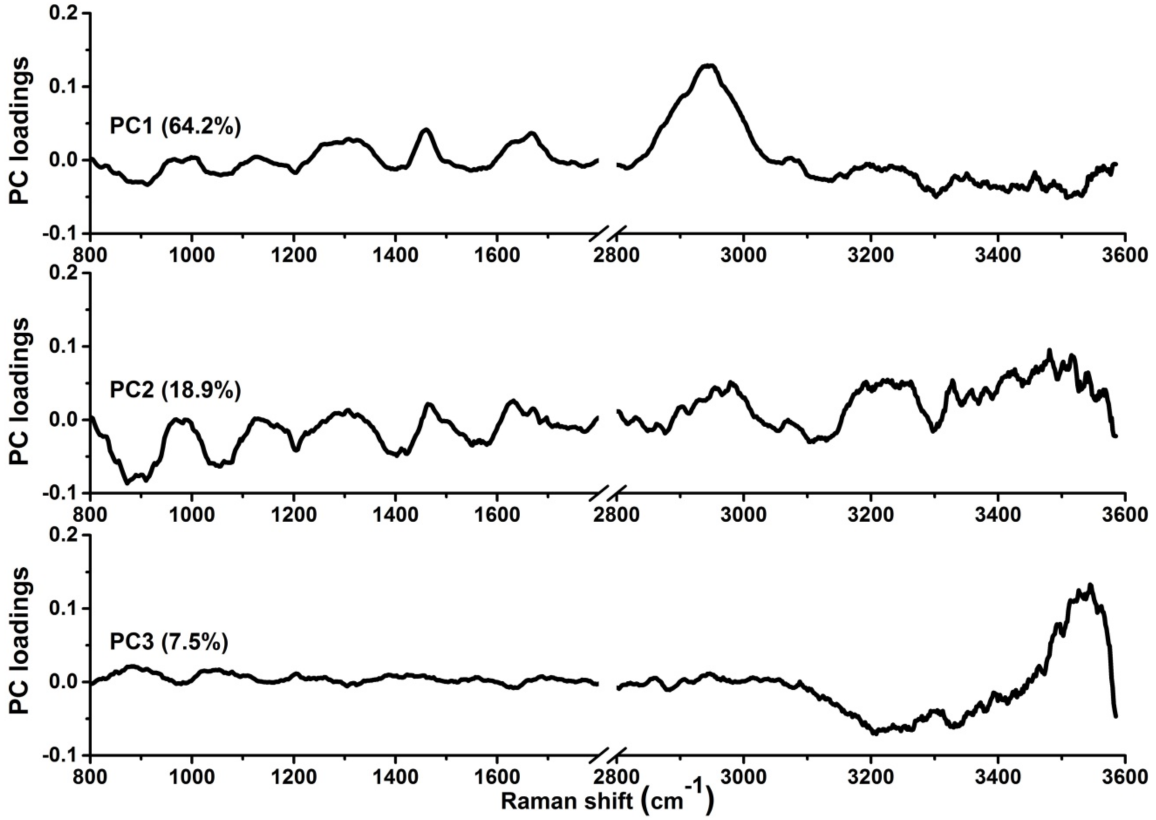

Fig 3. The first three diagnostically significant principal components (PCs) accounting for ~90% of the total variance calculated from integrated FP/HW Raman spectra of normal and NPC tissues (PC1 = 64.2%; PC2 = 18.9%; PC3 = 7.5%;).

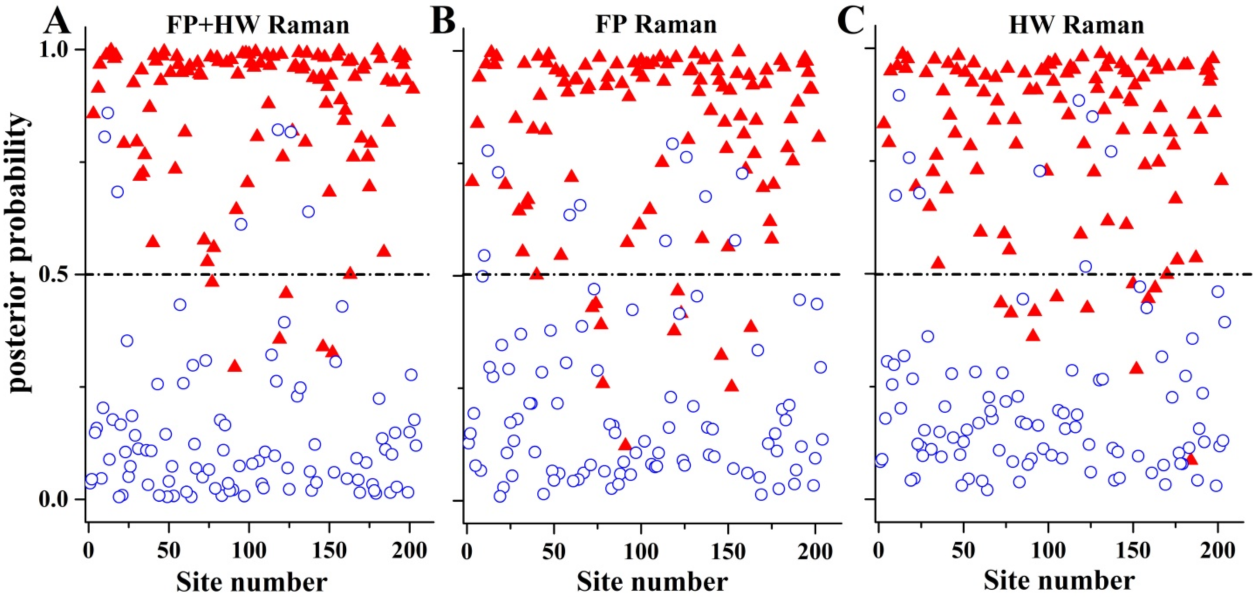

Fig 4. The posterior probabilities of nasopharyngeal tissue sites belonging to NPC and normal tissue based on the integrated FP/HW, FP and HW Raman techniques, respectively, together with PCA-LDA and leave-one tissue site-out, cross-validation methods. (○) normal; and (▲) carcinoma.

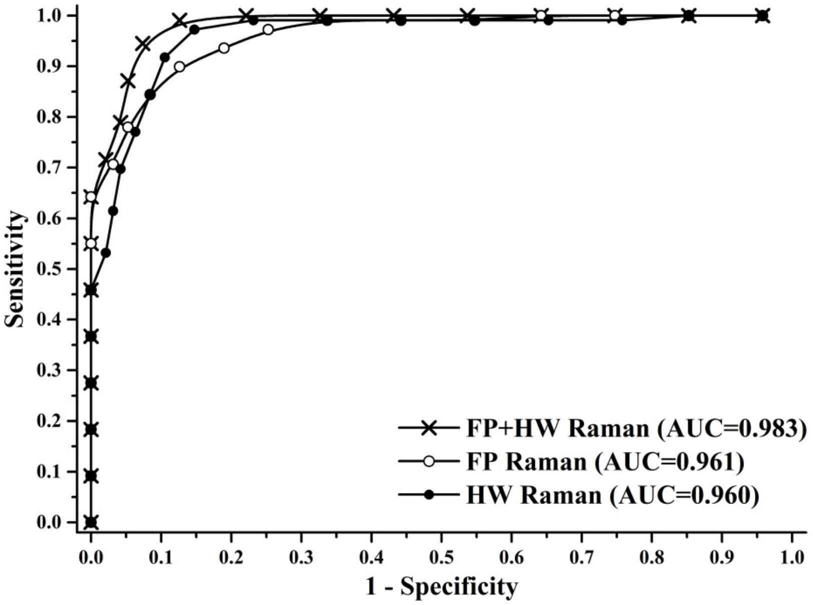

Fig. 5. Receiver operating characteristic (ROC) curves of the classification results for separating NPC from normal tissue using the integrated FP/HW Raman, FP, and HW Raman techniques together with PCA-LDA algorithms with leave-one subject-out, cross-validation methods. The areas under the ROC curves (AUC) are 0.983, 0.961 and 0.960, respectively, by using the integrated FP/HW, FP, and HW Raman techniques.

Discussion

In this study, we explored the feasibility of applying the unique simultaneous FP and HW fiber-optic Raman spectroscopy technique developed for real-time *in vivo* diagnosis of NPC during endoscopic examination. We demonstrate that high quality FP and HW tissue Raman spectra can be acquired *in vivo* from nasopharyngeal tissue, which could be used to identify the specific biochemical compositions of the nasopharynx for providing an objective characterization of normal and cancerous nasopharyngeal tissue *in vivo*. While the histopathology characterization identifies cellular features of syncytial pattern in cancerous lesions (Fig. 2), FP/HW Raman endoscopy uncovers the biochemical and biomolecular (e.g., proteins, lipids, DNA and water) changes occurring in epithelial tissues (Fig. 1B). Significant FP and HW Raman spectral differences were observed between normal and cancerous nasopharyngeal tissue, confirming the diagnostic potential of the integrated FP and HW Raman spectroscopy for enhancing *in vivo* diagnosis of NPC at endoscopy. Significant different sub-regions of FP Raman bands (e.g., 800-955, 1000-1120, 1180-1230, 1235-1265, 1400-1470, 1500-1580 and 1590-1683 cm^{-1}) were also identified (Fig. 1B), uncovering the relative changes of diagnostically significant Raman intensities associated with nasopharyngeal carcinoma development. For instance, NPC is accompanied with a significantly reduced Raman peak intensity at 853 cm^{-1} (v(C-C) proteins) and 875 cm^{-1} (v(C-C) stretching of hydroxyproline), indicating a relative reduction in collagen content of NPC. We also found that the Raman peak at 1078 cm^{-1} assigned to the lipids is significantly decreased associated with NPC. As NPC is characterized by bulging and thickened nasopharynx mucosa that is usually adjacent to an invasive malignancy [45], the decreased Raman peaking at 853 and 1078 cm^{-1} can be explained by the nasopharyngeal mucosa thickenings which may result in attenuating the laser light excitation as well as obscuring Raman emission from nasopharyngeal sub-layers. Besides, NPC is associated with significantly stronger Raman intensity at 1335 cm^{-1} (CH_3CH_2 twisting of proteins and nucleic acids), which is probably due to the increased DNA content associated with carcinoma transformation in the nasopharynx [46, 47]. NPC is also characterized by the increased Raman peaks related to proteins (i.e., 1265 (amide III v(C-N) and δ(N-H) of proteins), 1335 (CH_3CH_2 twisting of proteins and nucleic acids), 1445 ($\delta(CH_2)$ deformation of proteins and lipids)). This observation is in agreement with the molecular biology study whereby an over-expression of various proteins associated with NPC has been reported [48, 49]. The FP Raman spectra show specific but relatively weak Raman peaks that are predominantly related to backbone structures of proteins, lipids and DNA [13-24, 44]. The HW Raman spectra, on the other hand, offer exclusive new insights into the CH_2 and CH_3 stretching information and structure as well as the interactions of intra- and inter-cellular water *in situ*. The intense broad Raman band of water above

3000 cm^{-1} has also been observed in other soft tissues (e.g., brain, gastric, larynx and the oral cavity) [50-52]. Specifically, significant HW Raman spectra differences between NPC and normal nasopharyngeal tissue were found in the regions with the bands at 2850, 2885 cm^{-1} (symmetric and asymmetric CH_2 stretching of lipids) and 2940 cm^{-1} (CH_3 stretching of proteins), which were also likely due to the increase of proliferation activity for the epithelial cells during the development of nasopharyngeal cancer states [48, 49]. The increased water content observed at ~3250 cm^{-1} for the nasopharyngeal cancer tissue (Fig. 1B) reflects the re-arrangement of hydrogen-bonded networks in the epithelium layer. In addition, the Raman peak intensity ratios of I_{2940}/I_{2850} (normal: 2.21 ± 0.39, NPC: 2.87 ± 0.35) and I_{2940} /I_{2885} (normal: 1.02 ± 0.14, NPC: 1.19 ± 0.11) were calcuated, revealing a significantly increased in protein content while decreased in lipid content associated with NPC (unpaired two-side Student's t-test, p<0.01). The changes of lipid and protein contents asscociated with NPC we observed in this study are similar to other tissue types using Raman spectroscopy [14, 17, 20, 21, 23, 29-31, 32-35, 44, 52-54]. We also evluated the efficiency of the protein/lipid ratios for the classification of normal and NPC. We found the Raman peak intensity ratios of I_{2940} /I_{2850} and I_{2940} /I_{2885} could separate normal nasopharynx and NPC with sensitivities of 70.6% (77/109) and 65.1% (71/109), and specificities of 76.8% (73/95) and 71.6% (68/93), respectively. Hence, one of the major advantages of the FP/HW Raman system over prior Raman techniques is its ability to acquire both the FP Raman spectra and its complementary HW Raman spectra simultaneously for improving *in vivo* cancer diagnostics in the nasopharynx during endoscopic examination.

To further improve the diagnostic performance of the fiber-optic FP/HW Raman technique, we fully utilize all the diagnostically significant information contained in tissue FP/HW Raman spectra by implementing PCA-LDA and LOOCV technique for diagnostic model development. PCA-LDA analysis demonstrates the best diagnostic performances of the simultaneous FP and HW Raman spectroscopy for *in vivo* detection of nasopharyngeal carcinoma (Figs. 4-5). The enhanced tissue diagnosis using the simultaneous FP/HW Raman spectroscopy can partially be explained by back-tracing the incorrect predictions of each Raman modality. The FP and HW Raman modalities made incorrect predictions on 22 (normal: 11, and NPC: 11) and 21 (normal: 9, and NPC: 12) tissue sites, respectively, of the total 95 subjects recruited. Combining the FP and HW Raman modalities reduced the number of incorrect predictions to 14 (normal: 7, and NPC: 7). Further investigations reveal that the Raman signals of misclassified FP Raman spectra are extremely weak but with relatively higher AF level; while the addition of the HW modality containing the more intense Raman peaks (e.g., 2885, 2940, 3250 and 3400 cm^{-1}) but comparably diminutive AF background could enhance the overall signal-to-noise ratio of the integrated FP/HW Raman spectra. The encouraging results above suggest the great potential of fiber-optic FP/HW Raman technique to be a robust diagnostic tool for enhancing *in vivo* NPC detection.

Under NIR laser light excitation, the generated inherently weak tissue Raman emission is always accompanied with a relatively strong NIR AF background which could arise from endogenous fluorophores such as collagen, elastin, nicotinamide adenine dinucleotide (NADH), hematoporphyrin, and flavin adenine dinucleotide (FAD) in human tissues [55]. Hence, one of the major challenges to apply Raman spectroscopy for real-time *in vivo* biomedical applications is the implementation of effective methods for extracting extremely weak tissue Raman signals from concomitant strong tissue AF background emission. For example, computational methods (e.g., polynomial fitting, Fourier transformation) [7, 14, 21, 22, 25, 56-61] or hardware modifications (e.g., time gating, shifted excitation,) [62, 63] have been employed to eliminate the AF interference for deriving tissue Raman signals. In this study, we have applied the computation method based on the modified multi-polynomial fitting method [25, 41] to achieve effective and rapid (<20 ms) tissue AF elimination for real-time *in vivo* esophageal tissue Raman measurements without the need of hardware modifications.

On the other hand, some limitations of the current exploratory *in vivo* nasopharyngeal Raman studies should be pointed out. For instance, under the approved ethical protocol design, WLR endoscopically normal looking tissue sites from healthy subjects were referred to as normal for FP/HW Raman measurements, but no biopsies were taken from these nasopharyngeal tissue sites measured for histopathology examination. Hence, the normal tissue baselines simply determined by otolaryngologists' WLR visualization may not be fully corrected, which could result in errors in tissue Raman classification. Another, the NPC patients recruited are not randomized that may have selection bias (i.e., non-respondent bias); And the patient numbers measured are still very limited in this study, thus PCA-LDA together with leave-one subject-out cross-validation on the limited *in vivo* Raman dataset has to be implemented to avoid biased diagnosis. To push the frontier of real-time fiber-optic FP/HW

Raman spectroscopy into routine clinical endoscopic diagnostics, we are currently conducting clinical trials on a larger series of head and neck patients. With a larger tissue Raman database to be built, the independent training and testing of *in vivo* Raman datasets will be performed for further assessing true clinically diagnostic value of the FP/HW Raman spectroscopy technique for early cancer diagnosis in the head and neck during endoscopic screening.

Conclusions

We demonstrate for the first time that the simultaneous fingerprint (FP) and high-wavenumber (HW) fiber-optic Raman spectroscopy can be performed in real-time during endoscopic screening of NPC patients. A wealth of biochemical information contained in FP/HW tissue Raman spectra can be acquired nondestructively from nasopharynx *in vivo*. Significant FP and HW Raman spectral differences between normal and NPC tissue are observed *in vivo*, affirming that the rapid FP/HW fiber-optic Raman spectroscopy has potential for enhancing real-time, *in vivo* cancer diagnosis in the nasopharynx at the molecular level during endoscopic examination.

Acknowledgements

This work was supported by the National Medical Research Council (NMRC) (CIRG/1331/ 2012; NMRC/BnB/0012b/2014; NMRC/BnB/0012c /2014; NMRC/TCR/016-NNI/2016), and the Academic Research Fund (AcRF)-Tier 2 from Ministry of Education (MOE) (MOE2014-T2-1-010), Singapore.

Competing Interests

The authors have declared that no competing interest exists.

References

1. Ferlay J, Soerjomataram I, Dikshit R, Eser S, Mathers C, Rebelo M, et al. Cancer incidence and mortality worldwide: Sources, methods and major patterns in GLOBOCAN 2012. Int J Cancer. 2015; 136: E359-E86.
2. Jemal A, Bray F, Center MM, Ferlay J, Ward E, Forman D. Global cancer statistics. CA Cancer J Clin. 2011; 61: 69-90.
3. Chan KCA, Hung ECW, Woo JKS, Chan PKS, Leung SF, Lai FPT, et al. Early detection of nasopharyngeal carcinoma by plasma Epstein-Barr virus DNA analysis in a surveillance program. Cancer. 2013; 119: 1838-44.
4. Tsao SW, Yip YL, Tsang CM, Pang PS, Lau VMY, Zhang G, et al. Etiological factors of nasopharyngeal carcinoma. Oral Oncol. 2014; 50: 330-8.
5. Strazzulla A, Barreca GS, Giancotti A, Pisani V, Costa C, Zicca E, et al. Nasopharyngeal carcinoma: review of the literature with a focus on therapeutical implications. Infez Med. 2015; 23: 224-9.
6. Zhang L, Chen Q-Y, Liu H, Tang LQ, Mai HQ. Emerging treatment options for nasopharyngeal carcinoma. Drug Des Devel Ther. 2013; 7: 37-52.
7. Bergholt MS, Lin K, Zheng W, Lau DP, Huang Z. *In vivo*, real-time, transnasal, image-guided Raman endoscopy: defining spectral properties in the nasopharynx and larynx. J Biomed Opt. 2012; 17: 077002.
8. Chen Y, Zhao W, Lin L, Xiao X, Zhou X, Ming H, et al. Nasopharyngeal Epstein-Barr Virus Load: An Efficient Supplementary Method for Population-Based Nasopharyngeal Carcinoma Screening. PloS one. 2015; 10: e0132669.
9. Tabuchi K, Nakayama M, Nishimura B, Hayashi K, Hara A. Early Detection of Nasopharyngeal Carcinoma. Int J Otolaryngol. 2011; 2011.
10. Stoker SD, van Diessen JNA, de Boer JP, Karakullukcu B, Leemans CR, Tan IB. Current Treatment Options for Local Residual Nasopharyngeal Carcinoma. Curr Treat Options Oncol. 2013; 14: 475-91.
11. Hsieh CC, Wang WH, Lin YC, Weng HH, Lee KF. A large-scale study of the association between biopsy results and clinical manifestations in patients with suspicion of nasopharyngeal carcinoma. The Laryngoscope. 2012; 122: 1988-93.
12. Raman CV, Krishnan KS. A New Type of Secondary Radiation. Nature. 1928; 121: 501-2.
13. Patil CA, Pence IJ, Lieber CA, Mahadevan-Jansen A. 1064 nm dispersive Raman spectroscopy of tissues with strong near-infrared autofluorescence. Opt Lett. 2014; 39: 303-6.
14. Teh S, Zheng W, Ho K, Teh M, Yeoh K, Huang Z. Diagnostic potential of near-infrared Raman spectroscopy in the stomach: differentiating dysplasia from normal tissue. Br J Cancer. 2008; 98: 457-65.
15. Stone N, Kendall C, Smith J, Crow P, Barr H. Raman spectroscopy for identification of epithelial cancers. Faraday Discuss. 2004; 126: 141-57; discussion 69-83.
16. Short MA, Lam S, McWilliams A, Zhao J, Lui H, Zeng H. Development and preliminary results of an endoscopic Raman probe for potential *in vivo* diagnosis of lung cancers. Opt Lett. 2008; 33: 711-3.
17. Shim MG, Wilson BC, Marple E, Wach M. Study of Fiber-Optic Probes for *in vivo* Medical Raman Spectroscopy. Appl Spectrosc. 1999; 53: 619-27.
18. Motz JT, Hunter M, Galindo LH, Gardecki JA, Kramer JR, Dasari RR, et al. Optical fiber probe for biomedical Raman spectroscopy. Appl Opt. 2004; 43: 542-54.
19. Komachi Y, Sato H, Aizawa K, Tashiro H. Micro-optical fiber probe for use in an intravascular Raman endoscope. Appl Opt. 2005; 44: 4722-32.
20. Huang Z, Teh SK, Zheng W, Mo J, Lin K, Shao X, et al. Integrated Raman spectroscopy and trimodal wide-field imaging techniques for real-time *in vivo* tissue Raman measurements at endoscopy. Opt Lett. 2009; 34: 758-60.
21. Huang Z, McWilliams A, Lui H, McLean DI, Lam S, Zeng H. Near-infrared Raman spectroscopy for optical diagnosis of lung cancer. Int J Cancer. 2003; 107: 1047-52.
22. Huang Z, Bergholt MS, Zheng W, Lin K, Ho KY, Teh M, et al. *In vivo* early diagnosis of gastric dysplasia using narrow-band image-guided Raman endoscopy. J Biomed Opt. 2010; 15: 037017.
23. Draga RO, Grimbergen MC, Vijverberg PL, van Swol CF, Jonges TG, Kummer JA, Ruud Bosch JL. *In vivo* Bladder Cancer Diagnosis by High-Volume Raman Spectroscopy. Anal Chem. 2010; 82: 5993-9.
24. Day JCC, Bennett R, Smith B, Kendall C, Hutchings J, Meaden GM, et al. A miniature confocal Raman probe for endoscopic use. Phys Med Biol. 2009; 54: 7077.
25. Duraipandian S, Bergholt M, Zheng W, Ho KY, Teh M, Yeoh KG, Huang Z. Real-time Raman spectroscopy for *in vivo*, online gastric cancer diagnosis during clinical endoscopic examination. J Biomed Opt. 2012; 17: 081418.
26. Lau DP, Huang Z, Lui H, Anderson DW, Berean K, Morrison MD, et al. Raman spectroscopy for optical diagnosis in the larynx: preliminary findings. Lasers Surg Med. 2005; 37: 192-200.
27. Lau DP, Huang Z, Lui H, Man CS, Berean K, Morrison MD, et al. Raman spectroscopy for optical diagnosis in normal and cancerous tissue of the nasopharynx—preliminary findings. Lasers Surg Med. 2003; 32: 210-4.
28. Koljenovic S, Bakker Schut TC, Wolthuis R, de Jong B, Santos L, Caspers PJ, et al. Tissue characterization using high wave number Raman spectroscopy. J Biomed Opt. 2005; 10: 031116.
29. Santos LF, Wolthuis R, Koljenovic S, Almeida RM, Puppels GJ. Fiber-optic probes for *in vivo* Raman spectroscopy in the high-wavenumber region. Anal Chem. 2005; 77: 6747-52.
30. Mo J, Zheng W, Low JJH, Ng J, Ilancheran A, Huang Z. High Wavenumber Raman Spectroscopy for *in vivo* Detection of Cervical Dysplasia. Anal Chem. 2009; 81: 8908-15.
31. Lin K, Lau DP, Huang Z. Optical diagnosis of laryngeal cancer using high wavenumber Raman spectroscopy. Biosens Bioelectron. 2012; 35: 213-7.
32. Lin K, Wang J, Zheng W, Ho KY, Teh M, Yeoh KG, Huang Z. Rapid fiber-optic Raman spectroscopy for real-time *in vivo* detection of gastric intestinal metaplasia during clinical gastroscopy. Cancer Prevention Research. 2016; 9:476-83.
33. Wang J, Lin K, Zheng W, Ho KY, Teh M, Yeoh KG, Huang Z. Real-time fiber-optic Raman spectroscopy for *in vivo* diagnosis of gastric dysplasia. Faraday Discussions. 2016; 187: 377-92.
34. Wang J, Lin K, Zheng W, Ho KY, Teh M, Yeoh KG, Huang Z. Simultaneous fingerprint and high-wavenumber fiber-optic Raman spectroscopy improves *in vivo* diagnosis of esophageal squamous cell carcinoma at endoscopy. Sci Rep. 2015; 5: 12957.
35. Bergholt MS, Lin K, Wang J, Zheng W, Xu H, Huang Q, et al. Simultaneous fingerprint and high-wavenumber fiber-optic Raman spectroscopy enhances real-time *in vivo* diagnosis of adenomatous polyps during colonoscopy. J Biophotonics. 2016; 9: 333-42.
36. Huang W, Wu S, Chen M, Sun L, Li Y, Huang M, et al. Study of both fingerprint and high wavenumber Raman spectroscopy of pathological nasopharyngeal tissues. J Raman Spectrosc. 2015; 46: 537-44.
37. Li Y, Pan J, Chen G, Li C, Lin S, Shao Y, et al. Micro-Raman spectroscopy study of cancerous and normal nasopharyngeal tissues, J Biomed Opt. 2013; 18: 027003.

38. Wang J, Bergholt MS, Zheng W, Huang Z. Development of a beveled fiber-optic confocal Raman probe for enhancing *in vivo* epithelial tissue Raman measurements at endoscopy. Opt Lett. 2013; 38: 2321-3.
39. American National Standard for the safe use of lasers. Rev. ed. ed. New York: ANSI; 1986.
40. Carletta J. Assessing agreement on classification tasks: the kappa statistic. Comput Linguist. 1996; 22: 249-54.
41. Lieber CA, Mahadevan-Jansen A. Automated method for subtraction of fluorescence from biological Raman spectra. Appl Spectrosc. 2003; 57: 1363-7.
42. Lachenbruch PA, Mickey MR. Estimation of Error Rates in Discriminant Analysis. Technometrics. 1968; 10: 1-11.
43. Ahrens H. Dillon, W. R. and M. Goldstein: Multivariate Analysis–Methods and Applications. Wiley, New York 1984, 587 S. Biometrical Journal. 1987; 29: 755-6.
44. Almond LM, Hutchings J, Shepherd N, Barr H, Stone N, Kendall C. Raman spectroscopy: a potential tool for early objective diagnosis of neoplasia in the oesophagus. J Biophotonics. 2011; 4: 685-95.
45. Sakr WA, Gale N, Gnepp DR, Crissman JD. Chapter 1 - Squamous Intraepithelial Neoplasia of the Upper Aerodigestive Tract. Diagnostic Surgical Pathology of the Head and Neck (2nd ed). Philadelphia: W.B. Saunders; 2009: 1-44.
46. Lo KW, Huang DP. Genetic and epigenetic changes in nasopharyngeal carcinoma. Semin Cancer Biol. 2002; 12: 451-62.
47. Tong JH, Tsang RK, Lo KW, Woo JK, Kwong J, Chan MW, et al. Quantitative Epstein-Barr virus DNA analysis and detection of gene promoter hypermethylation in nasopharyngeal (NP) brushing samples from patients with NP carcinoma. Clin Cancer Res. 2002; 8: 2612-9.
48. Chou J, Lin YC, Kim J, You L, Xu Z, He B, et al. Nasopharyngeal carcinoma—Review of the molecular mechanisms of tumorigenesis. Head Neck. 2008; 30: 946-63.
49. Ouyang J, Wu M, Huang C, Cao L, Li G. Overexpression of oxidored-nitro domain containing protein 1 inhibits human nasopharyngeal carcinoma and cervical cancer cell proliferation and induces apoptosis: Involvement of mitochondrial apoptotic pathways. Oncol Rep. 2013; 29: 79-86.
50. Wolthuis R, van Aken M, Fountas K, Robinson JS, Jr., Bruining HA, Puppels GJ. Determination of water concentration in brain tissue by Raman spectroscopy. Anal Chem. 2001; 73: 3915-20.
51. Barroso EM, Smits RW, Bakker Schut TC, ten Hove I, Hardillo JA, Wolvius EB, et al. Discrimination between oral cancer and healthy tissue based on water content determined by Raman spectroscopy. Anal Chem. 2015; 87: 2419-26.
52. Lin K, Zheng W, Lim CM, Huang Z, Real-time *in vivo* diagnosis of laryngeal carcinoma with rapid fiber-optic Raman endoscopy, Biomed Opt Express. 2016;7: 3705-15.
53. Bergholt MS, Zheng W, Ho KY, Teh M, Yeoh KG, Yan So JB, Shabbir A, Huang Z. Fiberoptic confocal raman spectroscopy for real-time *in vivo* diagnosis of dysplasia in Barrett's esophagus. Gastroenterology. 2014; 146: 27-32.
54. Bergholt MS, Zheng W, Lin K, Ho KY, Teh M, Yeoh KG, Huang Z. *In vivo* diagnosis of esophageal cancer using image-guided Raman endoscopy and biomolecular modeling. Technol Cancer Res Treat. 2011; 10: 103-12.
55. Shao X, Zheng W, Huang Z. *In vivo* diagnosis of colonic precancer and cancer using near-infrared autofluorescence spectroscopy and biochemical modeling, J Biomed Opt. 2011; 16: 067005.
56. Vickers TJ, Wambles RE, Mann CK. Curve Fitting and Linearity: Data Processing in Raman Spectroscopy. Appl Spectrosc. 2001; 55: 389-93.
57. Knorr F, Smith ZJ, Wachsmann-Hogiu S. Development of a time-gated system for Raman spectroscopy of biological samples. Opt Express. 2010; 18: 20049-58.
58. Philipsen PA, Knudsen L, Gniadecka M, Ravnbak MH, Wulf HC. Diagnosis of malignant melanoma and basal cell carcinoma by *in vivo* NIR-FT Raman spectroscopy is independent of skin pigmentation. Photochem Photobiol Sci. 2013; 12: 770-6.
59. Christian K, Johanna M, Werner A, Kathrin B, Tesfay GM, Robert H, et al. Raman difference spectroscopy: a non-invasive method for identification of oral squamous cell carcinoma. Biomed Opt Express. 2014; 5: 3252-65.
60. Schulze G, Jirasek A, Yu MM, Lim A, Turner RF, Blades MW. Investigation of selected baseline removal techniques as candidates for automated implementation. Appl Spectrosc. 2005; 59:545-74.
61. Atakan AK, Blass WE, Jennings DE. Elimination of Baseline Variations from a Recorded Spectrum by Ultra-low Frequency Filtering. Appl Spectrosc. 1980; 34: 369-72.
62. Morris MD, Matousek P, Towrie M, Parker AW, Goodship AE, Draper ER. Kerr-gated time-resolved Raman spectroscopy of equine cortical bone tissue. J Biomed Opt. 2005; 10:14014.
63. Baraga JJ, Feld MS, Rava RP. Rapid near-Infrared Raman spectroscopy of human tissue with a spectrograph and CCD detector. Appl Spectrosc.1992; 46: 187-90.
