# Supplementary material for: The accuracy of Fiber-Optic Raman Spectroscopy in the detection and diagnosis of head and neck neoplasm in vivo: a systematic review and meta-analysis
Source: PeerJ. 2023 Dec 11;11:e16536. doi: 10.7717/peerj.16536 (PMC10720414; doi:10.7717/peerj.16536)
Supplement: Data S1 [file peerj-11-16536-s001.zip › article/Real-time in vivo diagnosis of laryngeal__carcinoma with rapid fiber-optic Raman__spectroscopy.pdf]

Research Article | Vol. 7, No. 9 | 1 Sep 2016 | BIOMEDICAL OPTICS EXPRESS 3705
Biomedical Optics EXPRESS

Real-time *in vivo* diagnosis of laryngeal carcinoma with rapid fiber-optic Raman spectroscopy

Kan Lin,[1,2] Wei Zheng,[1,2] Chwee Ming Lim,[3] and Zhiwei Huang[1,*]

[1]*Optical Bioimaging Laboratory, Department of Biomedical Engineering, Faculty of Engineering, National University of Singapore, 117576 Singapore*
[2]*Department of Medicine, Yong Loo Lin School of Medicine, National University of Singapore and National University Health System, 119260 Singapore*
[3]*Department of Otolaryngology, Head and Neck Surgery, National University of Singapore and National University Health System, 119074 Singapore*
[*]*biehzw@nus.edu.sg*

Abstract: We assess the clinical utility of a unique simultaneous fingerprint (FP) (i.e., 800-1800 cm^{-1}) and high-wavenumber (HW) (i.e., 2800-3600 cm^{-1}) fiber-optic Raman spectroscopy for *in vivo* diagnosis of laryngeal cancer at endoscopy. A total of 2124 high-quality *in vivo* FP/HW Raman spectra (normal = 1321; cancer = 581) were acquired from 101 tissue sites (normal = 71; cancer = 30) of 60 patients (normal = 44; cancer = 16) undergoing routine endoscopic examination. FP/HW Raman spectra differ significantly between normal and cancerous laryngeal tissue that could be attributed to changes of proteins, lipids, nucleic acids, and the bound water content in the larynx. Partial least squares-discriminant analysis and leave-one tissue site-out, cross-validation were employed on the in vivo FP/HW tissue Raman spectra acquired, yielding a diagnostic accuracy of 91.1% (sensitivity: 93.3% (28/30); specificity: 90.1% (64/71)) for laryngeal cancer identification, which is superior to using either FP (accuracy: 86.1%; sensitivity: 86.7% (26/30); specificity: 85.9% (61/71)) or HW (accuracy: 84.2%; sensitivity: 76.7% (23/30); specificity: 87.3% (62/71)) Raman technique alone. Further receiver operating characteristic analysis reconfirms the best performance of the simultaneous FP/HW Raman technique for laryngeal cancer diagnosis. We demonstrate for the first time that the simultaneous FP/HW Raman spectroscopy technique can be used for improving real-time *in vivo* diagnosis of laryngeal carcinoma during endoscopic examination.

©2016 Optical Society of America

OCIS codes: (170.5660) Raman spectroscopy; (170.6510) Spectroscopy, tissue diagnostics; (120.3890) Medical optics instrumentation.

References and links

1. K. Markou, A. Christoforidou, I. Karasmanis, G. Tsiropoulos, S. Triaridis, I. Constantinidis, V. Vital, and A. Nikolaou, "Laryngeal cancer: epidemiological data from Northern Greece and review of the literature," Hippokratia **17**(4), 313–318 (2013).
2. J. S. Williamson, T. C. Biggs, and D. Ingrams, "Laryngeal cancer: an overview," Trends in Urology Men's Health **3**(6), 14–17 (2012).
3. L.-A. Lee, A.-J. Cheng, T.-J. Fang, C.-G. Huang, C.-T. Liao, J. T.-C. Chang, and H.-Y. Li, "High Incidence of Malignant Transformation of Laryngeal Papilloma in Taiwan," Laryngoscope **118**(1), 50–55 (2008).
4. E. A. Chu and Y. J. Kim, "Laryngeal Cancer: Diagnosis and Preoperative Work-up," Otolaryngol. Clin. North Am. **41**(4), 673–695 (2008).
5. N. Stone, P. Stavroulaki, C. Kendall, M. Birchall, and H. Barr, "Raman spectroscopy for early detection of laryngeal malignancy: preliminary results," Laryngoscope **110**(10), 1756–1763 (2000).
6. D. P. Lau, Z. Huang, H. Lui, C. S. Man, K. Berean, M. D. Morrison, and H. Zeng, "Raman spectroscopy for optical diagnosis in normal and cancerous tissue of the nasopharynx-preliminary findings," Lasers Surg. Med. **32**(3), 210–214 (2003).
7. D. P. Lau, Z. Huang, H. Lui, D. W. Anderson, K. Berean, M. D. Morrison, L. Shen, and H. Zeng, "Raman spectroscopy for optical diagnosis in the larynx: preliminary findings," Lasers Surg. Med. **37**(3), 192–200 (2005).
8. S. K. Teh, W. Zheng, D. P. Lau, and Z. Huang, "Spectroscopic diagnosis of laryngeal carcinoma using near-infrared Raman spectroscopy and random recursive partitioning ensemble techniques," Analyst (Lond.) **134**(6),

#268821 http://dx.doi.org/10.1364/BOE.7.003705
Journal © 2016 Received 8 Jul 2016; revised 23 Aug 2016; accepted 23 Aug 2016; published 26 Aug 2016

1232–1239 (2009).
9. K. Lin, D. L. P. Cheng, and Z. Huang, "Optical diagnosis of laryngeal cancer using high wavenumber Raman spectroscopy," Biosens. Bioelectron. **35**(1), 213–217 (2012).
10. M. S. Bergholt, W. Zheng, and Z. Huang, "Characterizing variability in in vivo Raman spectroscopic properties of different anatomical sites of normal tissue in the oral cavity," J. Raman Spectrosc. **43**(2), 255–262 (2012).
11. E. M. Barroso, R. W. H. Smits, T. C. Bakker Schut, I. ten Hove, J. A. Hardillo, E. B. Wolvius, R. J. Baatenburg de Jong, S. Koljenović, and G. J. Puppels, "Discrimination between oral cancer and healthy tissue based on water content determined by Raman spectroscopy," Anal. Chem. **87**(4), 2419–2426 (2015).
12. Z. Huang, S. K. Teh, W. Zheng, J. Mo, K. Lin, X. Shao, K. Y. Ho, M. Teh, and K. G. Yeoh, "Integrated Raman spectroscopy and trimodal wide-field imaging techniques for real-time in vivo tissue Raman measurements at endoscopy," Opt. Lett. **34**(6), 758–760 (2009).
13. O. R. Hughes, N. Stone, M. Kraft, C. Arens, and M. A. Birchall, "Optical and molecular techniques to identify tumor margins within the larynx," Head Neck **32**(11), 1544–1553 (2010).
14. M. G. Shim, B. C. Wilson, E. Marple, and M. Wach, "Study of Fiber-Optic Probes for in Vivo Medical Raman Spectroscopy," Appl. Spectrosc. **53**(6), 619–627 (1999).
15. Z. Huang, A. McWilliams, H. Lui, D. I. McLean, S. Lam, and H. Zeng, "Near-infrared Raman spectroscopy for optical diagnosis of lung cancer," Int. J. Cancer **107**(6), 1047–1052 (2003).
16. N. Stone, C. Kendall, J. Smith, P. Crow, and H. Barr, "Raman spectroscopy for identification of epithelial cancers," Faraday Discuss. **126**, 141–157, discussion 169–183 (2004).
17. S. K. Teh, W. Zheng, K. Y. Ho, M. Teh, K. G. Yeoh, and Z. Huang, "Diagnostic potential of near-infrared Raman spectroscopy in the stomach: differentiating dysplasia from normal tissue," Br. J. Cancer **98**(2), 457–465 (2008).
18. M. A. Short, S. Lam, A. McWilliams, J. Zhao, H. Lui, and H. Zeng, "Development and preliminary results of an endoscopic Raman probe for potential in vivo diagnosis of lung cancers," Opt. Lett. **33**(7), 711–713 (2008).
19. J. T. Motz, M. Hunter, L. H. Galindo, J. A. Gardecki, J. R. Kramer, R. R. Dasari, and M. S. Feld, "Optical fiber probe for biomedical Raman spectroscopy," Appl. Opt. **43**(3), 542–554 (2004).
20. C. A. Patil, I. J. Pence, C. A. Lieber, and A. Mahadevan-Jansen, "1064 nm dispersive Raman spectroscopy of tissues with strong near-infrared autofluorescence," Opt. Lett. **39**(2), 303–306 (2014).
21. Y. Komachi, H. Sato, K. Aizawa, and H. Tashiro, "Micro-optical fiber probe for use in an intravascular Raman endoscope," Appl. Opt. **44**(22), 4722–4732 (2005).
22. Z. Huang, M. S. Bergholt, W. Zheng, K. Lin, K. Y. Ho, M. Teh, and K. G. Yeoh, "In vivo early diagnosis of gastric dysplasia using narrow-band image-guided Raman endoscopy," J. Biomed. Opt. **15**(3), 037017 (2010).
23. R. O. P. Draga, M. C. M. Grimbergen, P. L. M. Vijverberg, C. F. van Swol, T. G. N. Jonges, J. A. Kummer, and J. L. H. Ruud Bosch, "In Vivo Bladder Cancer Diagnosis by High-Volume Raman Spectroscopy," Anal. Chem. **82**(14), 5993–5999 (2010).
24. J. C. C. Day, R. Bennett, B. Smith, C. Kendall, J. Hutchings, G. M. Meaden, C. Born, S. Yu, and N. Stone, "A miniature confocal Raman probe for endoscopic use," Phys. Med. Biol. **54**(23), 7077–7087 (2009).
25. S. Duraipandian, M. Sylvest Bergholt, W. Zheng, K. Yu Ho, M. Teh, K. Guan Yeoh, J. Bok Yan So, A. Shabbir, and Z. Huang, "Real-time Raman spectroscopy for in vivo, online gastric cancer diagnosis during clinical endoscopic examination," J. Biomed. Opt. **17**(8), 081418 (2012).
26. S. Koljenović, T. C. Bakker Schut, R. Wolthuis, B. de Jong, L. Santos, P. J. Caspers, J. M. Kros, and G. J. Puppels, "Tissue characterization using high wave number Raman spectroscopy," J. Biomed. Opt. **10**(3), 031116 (2005).
27. L. F. Santos, R. Wolthuis, S. Koljenović, R. M. Almeida, and G. J. Puppels, "Fiber-optic probes for in vivo Raman spectroscopy in the high-wavenumber region," Anal. Chem. **77**(20), 6747–6752 (2005).
28. J. Mo, W. Zheng, J. J. H. Low, J. Ng, A. Ilancheran, and Z. Huang, "High Wavenumber Raman Spectroscopy for in Vivo Detection of Cervical Dysplasia," Anal. Chem. **81**(21), 8908–8915 (2009).
29. J. Wang, K. Lin, W. Zheng, K. Y. Ho, M. Teh, K. G. Yeoh, and Z. Huang, "Fiber-optic Raman spectroscopy for in vivo diagnosis of gastric dysplasia," Faraday Discuss. **187**, 377–392 (2016).
30. J. Wang, K. Lin, W. Zheng, K. Y. Ho, M. Teh, K. G. Yeoh, and Z. Huang, "Simultaneous fingerprint and high-wavenumber fiber-optic Raman spectroscopy improves in vivo diagnosis of esophageal squamous cell carcinoma at endoscopy," Sci. Rep. **5**, 12957 (2015).
31. M. S. Bergholt, K. Lin, J. Wang, W. Zheng, H. Xu, Q. Huang, J. L. Ren, K. Y. Ho, M. Teh, S. Srivastava, B. Wong, K. G. Yeoh, and Z. Huang, "Simultaneous fingerprint and high-wavenumber fiber-optic Raman spectroscopy enhances real-time in vivo diagnosis of adenomatous polyps during colonoscopy," J. Biophotonics **9**(4), 333–342 (2016).
32. J. Wang, M. S. Bergholt, W. Zheng, and Z. Huang, "Development of a beveled fiber-optic confocal Raman probe for enhancing in vivo epithelial tissue Raman measurements at endoscopy," Opt. Lett. **38**(13), 2321–2323 (2013).
33. M. S. Bergholt, W. Zheng, K. Lin, K. Y. Ho, M. Teh, K. G. Yeoh, J. B. So, and Z. Huang, "Raman endoscopy for in vivo differentiation between benign and malignant ulcers in the stomach," Analyst (Lond.) **135**(12), 3162–3168 (2010).
34. L. M. Almond, J. Hutchings, N. Shepherd, H. Barr, N. Stone, and C. Kendall, "Raman spectroscopy: a potential tool for early objective diagnosis of neoplasia in the oesophagus," J. Biophotonics **4**(10), 685–695 (2011).
35. R. Wolthuis, M. van Aken, K. Fountas, J. S. Robinson, Jr., H. A. Bruining, and G. J. Puppels, "Determination of

Biomedical Optics EXPRESS

water concentration in brain tissue by Raman spectroscopy," Anal. Chem. **73**(16), 3915–3920 (2001).
36. M. Gniadecka, H. C. Wulf, O. F. Nielsen, D. H. Christensen, and J. Hercogova, "Distinctive molecular abnormalities in benign and malignant skin lesions: studies by Raman spectroscopy," Photochem. Photobiol. **66**(4), 418–423 (1997).
37. Z. Huang, H. Lui, D. I. McLean, M. Korbelik, and H. Zeng, "Raman spectroscopy in combination with background near-infrared autofluorescence enhances the in vivo assessment of malignant tissues," Photochem. Photobiol. **81**(5), 1219–1226 (2005).
38. K. Badizadegan, V. Backman, C. W. Boone, C. P. Crum, R. R. Dasari, I. Georgakoudi, K. Keefe, K. Munger, S. M. Shapshay, E. E. Sheetse, and M. S. Feld, "Spectroscopic diagnosis and imaging of invisible pre-cancer," Faraday Discussions **126**, 265–311 (2004).
39. K. W. Short, S. Carpenter, J. P. Freyer, and J. R. Mourant, "Raman Spectroscopy Detects Biochemical Changes Due to Proliferation in Mammalian Cell Cultures," Biophys. J. **88**(6), 4274–4288 (2005).
40. M. S. Bergholt, W. Zheng, K. Y. Ho, M. Teh, K. G. Yeoh, J. B. Yan So, A. Shabbir, and Z. Huang, "Fiberoptic confocal raman spectroscopy for real-time in vivo diagnosis of dysplasia in Barrett's esophagus," Gastroenterology **146**(1), 27–32 (2014).
41. A. Kumar Myakalwar, N. Spegazzini, C. Zhang, S. Kumar Anubham, R. R. Dasari, I. Barman, and M. Kumar Gundawar, "Less is more: Avoiding the LIBS dimensionality curse through judicious feature selection for explosive detection," Sci. Rep. **5**, 13169 (2015).
42. C. M. Andersen and R. Bro, "Variable selection in regression- a tutorial," J. Chemometr. **24**(11-12), 728–737 (2010).

1. Introduction

Laryngeal cancer is one of the most common malignancies in the head and neck, accounting for 30% to 40% of head and neck cancer [1]. Smoking tobacco and excess alcohol consumption could be one of the major risk factors for developing laryngeal cancer [2, 3]. Early detection and diagnosis of carcinoma in the larynx together with effective treatments (e.g., surgery, radiotherapy and thermotherapy) can significantly improve the 5-year survival rates of the patients [3, 4]. Currently, well-performed but randomized endoscopic biopsies followed by hematoxylin and eosin staining and microscopic viewing by the pathologists remains the gold standard for cancer diagnosis, but it is invasive and impractical for screening high risk patients with multiple suspicious lesions, which might affect the quality of life due to multiple biopsies [3, 4]. Conventional white-light reflectance (WLR) endoscopy suffers from limited diagnostic accuracy due to the lack of obvious morphological changes of early neoplastic lesions on the tissue surface. There is of great clinical needs to develop rapid, objective and minimally invasive or non-invasive diagnostic techniques for real-time diagnosis and characterization of laryngeal cancer with biomolecular specificity.

Raman spectroscopy is a vibrational spectroscopic technique capable of probing biochemical and biomolecular structures and conformations of tissue, and has received great interest for optical diagnosis and characterization of human tissue in the head and neck (e.g., oral cavity, nasopharynx and larynx) [5–11]. The diagnostic sensitivities and specificities of ~70-95% have been achieved for differentiation between different pathologic types (e.g., normal, dysplasia and carcinoma) of head and neck tissue *in vitro* using Raman spectroscopy [5, 7, 8]. However, in vivo Raman spectroscopic studies on the larynx are still very limited due to technical challenges in making miniaturized flexible fiber-optic Raman probes with high collection efficiencies while effective elimination of interference from fluorescence and silica Raman signals [12]. Additionally, most Raman studies for identification of different pathological types are focused on the so-called fingerprint (FP) range (i.e., 800-1800cm^{-1}) [5, 7, 8, 13]. The unrivaled advantage of the FP Raman spectroscopy technique stems from its capability to reveal specific information about backbone structures of proteins, lipids and nucleic acid assemblies in cells and tissue [6, 12, 14–25]. The efficiency of the FP Raman spectroscopy technique is compromised owing to its extremely weak tissue Raman signal but overwhelming tissue autofluorescence (AF) background. Whereas, high-wavenumber range (HW) (i.e., 2800-3600 cm^{-1}) Raman spectroscopy exhibits stronger tissue Raman signals with much reduced tissue AF interference, providing complementary biochemical information such as water content for tissue diagnosis [9, 26–30]. Hence, the integrated FP/HW Raman

spectroscopy with complementary biochemical and biomolecular information could have advantages for improving tissue characterization and diagnosis.

With our successful development of a rapid fiber-optic Raman spectroscopy system capable of simultaneously acquiring both the fingerprint (FP) (i.e., 800-1800 cm^{-1}) and high-wavenumber (HW) (i.e., 2800-3600 cm^{-1}) tissue Raman spectra *in vivo* at endoscopy [30, 31], in this work, we aim to assess the clinical utility of the rapid FP and HW Raman spectroscopy system for real-time *in vivo* diagnosis of cancerous tissue in the larynx. Unpaired two-sided Student's t-test is employed to extract the statistically different Raman spectral features between normal and cancer laryngeal tissue. Partial least squares-discriminant analysis (PLS-DA) and leave-one site-out, cross-validation (LOO-CV) are employed to develop robust Raman diagnostic algorithms for tissue classification. We compare the diagnostic performance of FP Raman, HW Raman, and the integrated FP/HW Raman techniques based on PLS-DA and LOO-CV modeling for laryngeal cancer identification. The receiver operating characteristic (ROC) curve is further generated to examine the clinical performance of simultaneous FP/HW Raman spectroscopy for improving *in vivo* diagnosis of laryngeal carcinoma at endoscopy.

2. Material and methods

2.1 Clinical fiber-optic Raman instrumentation

We have developed a simultaneous fingerprint (FP) and high wavenumber (HW) fiber-optic Raman spectroscopy technique for real-time *in vivo* tissue Raman measurements at endoscopy [30, 31]. Briefly, the fiber-optic Raman spectroscopic system consists of a near-infrared (NIR) diode laser (λ_{ex} = 785 nm) (maximum output: 300 mW, B&W TEK Inc.), a specially designed fiber-optic Raman probe [32], a high-throughput reflective imaging spectrograph (Acton LS-785 f/2, Princeton Instruments Inc.) equipped with a gold-coated 830 gr/mm grating and a thermo electric-cooled, NIR-optimized charge-coupled device (CCD) camera (PIXIS: 400BR-eXcelon, Princeton Instruments Inc.). We have developed a 1.9 m long fiber-optic Raman probe (~1.8 mm in outer diameter) for both laser light delivery and *in vivo* epithelial tissue Raman signal collection at endoscopy [31, 32]. The compact fiber-optic Raman endoscopic probe designed for endoscopy comprises 18 × 200 µm beveled collection fibers (low-OH fused silica, numerical aperture (NA) = 0.22) surrounding the central light delivery fiber (low-OH fused silica, 200 µm in diameter, NA = 0.22). A 1.0 mm sapphire ball lens (NA = 1.78) is coupled to the fiber tip of the probe to tightly focus the excitation light onto the tissue subsurface, enabling the effective Raman spectrum collection from the epithelial lining [31]. The depth-selective capability of the fiber-optic Raman spectroscopy technique ensures the shallower tissue interrogation (<200 µm) with tissue probing volume of <0.02 mm^3, thereby reducing the interferences and signal dilution from deeper bulky tissues, while selectively interrogating the epithelium associated with neoplastic onset and progression [32]. At the proximal ends of the Raman probe, the excitation and emission fibers were coupled into two separate in-line filter modules: one integrated with a narrow band-pass filter (LL01-785, Semrock, Inc.) for suppressing laser noise, and the other integrated with an edge long-pass filter (LP02-785RU, Semrock, Inc.) for further reduction of the scattered laser light while permitting the emitted tissue Raman photons to transmit into the Raman spectrograph. The atomic emission lines of mercury–argon spectral calibration lamps (HG-1 and AR-1, Ocean Optics, Inc., Dunedin, FL) are used for wavelength calibration. All wavelength-calibrated spectra were corrected for the wavelength dependence of the system using a tungsten calibration lamp (RS-10, EG&G Gamma Scientific, San Diego, CA). The system acquires tissue Raman spectra in the spectral range from 400 to 3600 cm^{-1} with a resolution of ~8 cm^{-1}. The entire FP/HW fiber-optic Raman endoscopic system is controlled using a foot pedal in an intuitive software framework with auditory probabilistic feedback to the clinician in

Biomedical Optics EXPRESS

real-time, pushing the frontier of Raman spectroscopy into routine clinical endoscopic diagnostics [25].

2.2 Clinical trial protocol

The ethical protocol of the present study was approved by the Institutional Review Board (IRB) of the National Healthcare Group (NHG) of Singapore. Prior to Raman measurements, all patients signed individual informed consent permitting the *in vivo* Raman spectroscopic measurements during endoscopic examination conducted in the head and neck clinic at the National University Health System (NUHS), Singapore. A total of 60 patients (47 men and 13 women with mean ages of 51; normal = 46 and cancer = 14) were enrolled in the Raman endoscopic examinations. The Raman probe passes down to the instrument channel of flexible rhino-laryngo fiberscope under the white-light imaging guidance. The Raman probe tip was visible approximately 0.2 cm in front of the endoscope camera. During endoscopic examination of suspicious lesions, the Raman probe was placed in gentle contact with the laryngeal mucosa surface, and the positioning of the Raman probe against the tissue sites was verified on the computer monitor by the endoscopists in-charge at endoscopy. FP/HW Raman spectrum was acquired from laryngeal tissue sites in vivo within 0.2 s, which permitted a rapid surveillance of large tissue areas in the larynx. Multiple spectra (15-20) for each site were measured and fed into PLS-DA algorithm modeling together with leave-one tissue site-out, cross-validation technique for tissue diagnosis and classification. Immediately after the tissue Raman acquisitions, each suspicious tissue site measured was biopsied and sent for histopathological examination. The consented histopathology assessments on the biopsied tissues serve as the gold standard to determine the diagnostic ability of the simultaneous FP/HW fiber-optic Raman technique for identifying laryngeal carcinoma from normal tissue in the larynx.

2.3 Data preprocessing and statistical analysis

The measured laryngeal tissue spectra represented a combination of weak tissue Raman scattering, intense AF and the noise. Therefore, the spectra were preprocessed by a third-order Savitzky-Golay smoothing filter (a window width of 5 pixels) to reduce the noise. A fifth-order polynomial was found to be optimal for fitting the AF background in the noise-smoothed spectrum over the range of 800-1800 cm^{-1}, and this polynomial was then subtracted from the measured FP spectrum to yield the FP tissue Raman spectrum alone. While in the HW range (2800-3600 cm^{-1}), a first-order polynomial fit was used for removing the AF background. The tissue FP/HW Raman spectrum was finally normalized to the integrated area of the entire FP and HW range to compare the differences in spectral shapes and relative Raman band intensities between cancerous and normal laryngeal tissue. There are no obvious tissue Raman peaks observed in the 1800-2800 cm^{-1} range, this silent range is therefore excluded for data analysis. All the preprocessing is completed within 30 ms, and the processed Raman spectra and diagnostic outcomes can be displayed on the computer screen in real-time during clinical endoscopic examinations [25].

Prior to multivariate statistical analysis, mean-centering was performed to remove common variance from the data set of *in vivo* laryngeal tissue Raman spectra. The unpaired two-sided Student's t-test was used to evaluate Raman spectral differences between normal and cancer laryngeal tissue. PLS-DA was further applied for tissue diagnosis model development. PLS-DA employs the fundamental principle of principal components analysis (PCA) but further rotates the components (latent variables (LVs)) by maximizing the covariance between the spectral variation and group affinity, so that the LVs explain the diagnostic relevant variations rather than the most prominent variations in the spectral data set. Using LOO-CV, the number of latent variables (LVs) with a minimum root mean squared error of cross-validation (RMSECV) for tissue prediction was selected for PLS-DA modeling [33]. One notes that multiple Raman spectra (15~20) were acquired from each tissue site, and

Biomedical Optics EXPRESS

the majority voting strategy was applied for final classification on each laryngeal tissue. The overall discriminatory accuracy of the FP, HW, and integrated FP/HW diagnostic models were further evaluated through the use of the ROC analysis. All the above spectra preprocessing and multivariate statistical analysis were performed online using in-house written scripts in MATLAB-programming environment [25].

3. Results and discussion

Figure 1(a) shows the mean *in vivo* FP/HW Raman spectra ± 1 standard deviation (SD) (shaded area) of normal (n = 1321) and cancerous (squamous cell carcinoma, n = 581) laryngeal tissues acquired from 101 (normal = 71 and cancer = 30) tissue sites of the 60 patients recruited (normal = 44; cancer = 14). The corresponding WLR image of the simultaneous FP/HW Raman procedures under WLR endoscopy guidance is also shown in Fig. 1. Prominent tissue FP/HW Raman peaks can be observed in both normal and cancerous laryngeal tissue. For instance, in the FP range, distinct Raman peaks are found at around 875 cm^{-1} (ν(C-C) stretching of hydroxyproline), 940 cm^{-1} (proline and valine), 1078 cm^{-1} (ν(C-C) of lipids), 1209 cm^{-1} (tryptophan and phenylalanine), 1265 cm^{-1} (amide III ν(C-N) and δ(N-H) of proteins), 1335 cm^{-1} (CH_3CH_2 twisting of proteins and nucleic acids), 1445 cm^{-1} ($\delta(CH_2)$ deformation of proteins and lipids), 1554 (ν(C = C) of amide III), 1580 cm^{-1} (δ(C = C) of phenylalanine) and 1655 cm^{-1} (amide I ν(C = O) of lipids)) [12, 14–24, 34]. Intense Raman peaks in the HW range are also observed; that is: 2850 and 2885 cm^{-1} (symmetric and asymmetric CH_2 stretching of lipids), 2940 cm^{-1} (CH_3 stretching of proteins), 3009 cm^{-1} (asymmetric = CH stretching of lipids), ~3300 cm^{-1} (amide A (NH stretching of proteins)), and water Raman band (OH stretching vibrations peaking at ~3250 and ~3400 cm^{-1}) that are related to the local conformation and interactions of OH-bonds in the intracellular and extracellular space of laryngeal tissue [9, 26–31]. The intense broad Raman band of water above 3000 cm^{-1} has also been observed in other soft tissues (e.g., brain and oral cavity) [11, 35]. Figure 1(b) shows the difference Raman spectra (i.e., cancer - normal) ± 1SD (shaded area), reflecting the different Raman-active component contributions (e.g., peak intensity) associated with cancerous progression in the larynx, confirming the potential of FP/HW Raman spectroscopy for early diagnosis of laryngeal cancer *in vivo* at endoscopy.

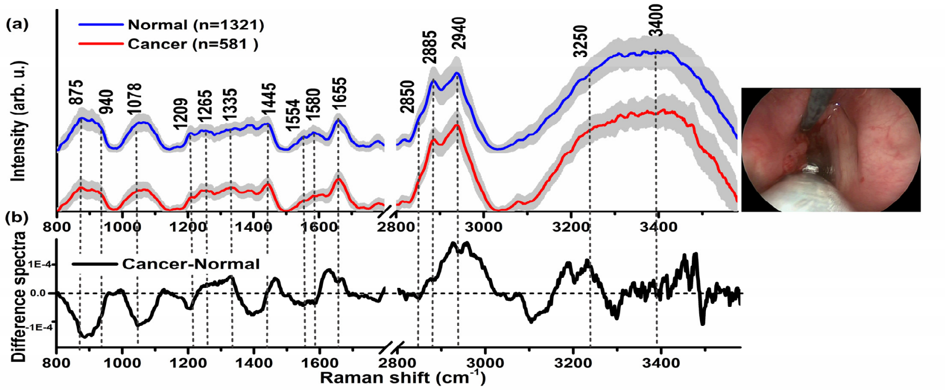

Fig. 1. (a) The mean *in vivo* FP/HW Raman spectra ± 1 standard deviations (SD) of normal (n = 1321) and laryngeal carcinoma (n = 581) acquired from 101 tissue sites (normal = 71 and carcinoma = 30) of 60 patients during clinical endoscopy. (b) Difference spectra (i.e., carcinoma - normal) ± 1 SD resolving the distinct spectral features of laryngeal carcinoma. The representative image of the simultaneous FP/HW Raman procedures under WLR imaging guidance is also shown.

To elucidate the diagnostically important Raman-active components, Fig. 2(a) shows a logarithmic plot of the calculated p-values (unpaired two-sided Student’s t-test) for each of

the Raman intensities in the entire FP and HW spectral range (i.e., 800-1800 cm^{-1} and 2800-3600 cm^{-1}) for laryngeal cancer diagnosis. Six spectral sub-regions with statistically significant differences ($p < 0.001$) between normal and laryngeal carcinoma are found at both FP and HW ranges; that is: 830-930 cm^{-1}, 1030-1095 cm^{-1}, 1510-1580 cm^{-1}, 1615-1670 cm^{-1}, 2880-3000 cm^{-1} and 3170-3260 cm^{-1}, which could be related to biochemical constituents (e.g., proteins, lipids, nucleic acids and water) in laryngeal tissue. Figure 2(b) shows a histogram of the most diagnostically significant Raman peak intensities (mean ± SD) in the FP range, e.g., 875 cm^{-1}, 1078 cm^{-1}, 1554 cm^{-1}, 1655 cm^{-1} and HW range at 2885 cm^{-1}, 2940 cm^{-1} and 3250 cm^{-1}, reconfirming the capability of simultaneous FP and HW Raman spectroscopy for in vivo diagnosis of laryngeal carcinoma at the molecular level.

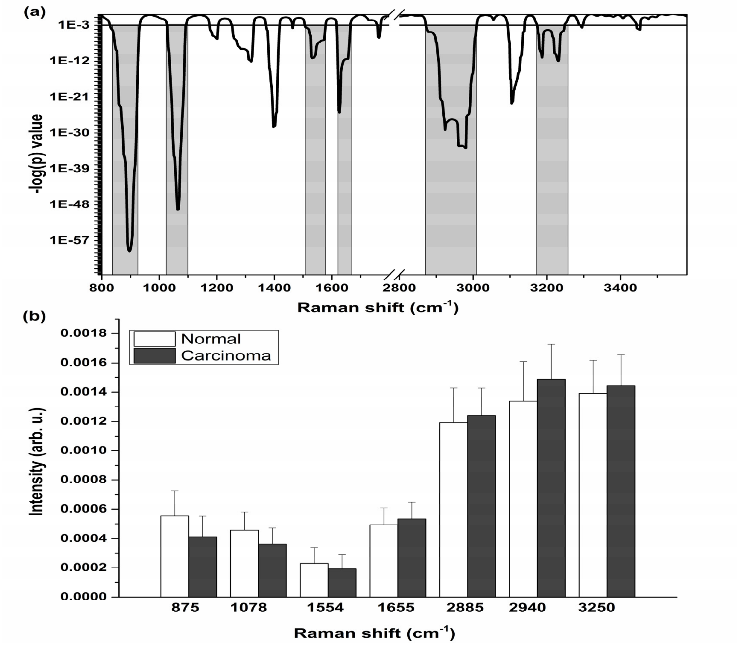

Fig. 2. (a) Unpaired two-sided Student's t-test on Raman peak intensities over the entire spectral range (i.e., 800–1800 cm^{-1} and 2800–3600 cm^{-1}) for laryngeal cancer diagnosis. (b) Histogram of the most diagnostically different ($p < 0.001$) Raman peak intensities (mean ± SD) in the FP (e.g., 875 cm^{-1}, 1078 cm^{-1}, 1554 cm^{-1}, 1655 cm^{-1}, and HW at 2885 cm^{-1}, 2940 cm^{-1} and 3250 cm^{-1} for tissue differentiation between normal and cancer larynx.

PLS-DA and LOO-CV are implemented on the *in vivo* tissue FP and HW Raman spectra acquired to develop robust diagnostic model to evaluate the elusive differences observed in Raman spectra of different laryngeal tissue types for enhancing *in vivo* cancer diagnosis in the larynx. The PLS-DA approach assigns orthogonal latent variables (LVs) on the basis of the spectral variance that correlate with the gold standard of histopathology (i.e., normal and carcinoma). Using a leave-one site-out-cross-validation, the first two latent variables (LV) [Fig. 3] providing the minimum root mean squared error of cross-validation (RMSECV) for tissue prediction are selected for PLS-DA/LOO-CV modeling. The molecular features associated with laryngeal carcinogenesis can be further extracted and visualized through the LV loadings [Fig. 4]. The first 2 loadings of LVs largely captured specific Raman features arising from different biochemicals in tissues, such as 875 cm^{-1} (ν(C-C) stretching of hydroxyproline), 940 cm^{-1} (proline and valine), 1004 cm^{-1} ((ν(C-C)), 1078 cm^{-1} (ν(C-C) of lipids), 1265 cm^{-1} (amide III ν(C-N) and δ(N-H) of proteins), 1335 cm^{-1} (CH_3CH_2 twisting of proteins and nucleic acids), 1445 cm^{-1} ($\delta(CH_2)$ deformation of proteins and lipids), and 1655

cm^{-1} (amide I v(C = O) of lipids)), and also HW Raman peaks at 2850 and 2885 cm^{-1} (symmetric and asymmetric CH_2 stretching of lipids), 2940 cm^{-1} (CH_3 stretching of proteins), 3250 cm^{-1} (symmetric and asymmetric OH vibrations), and 3329 cm^{-1} (amide A (N–H stretching)) in laryngeal tissue.

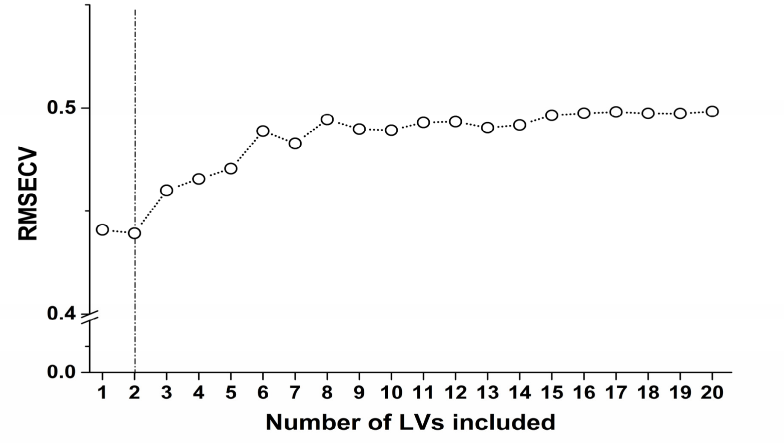

Fig. 3. The relationship between the number of PLS factors-latent variables (LVs) and the root mean square error of cross validation (RMSECV) for PLS-DA modeling.

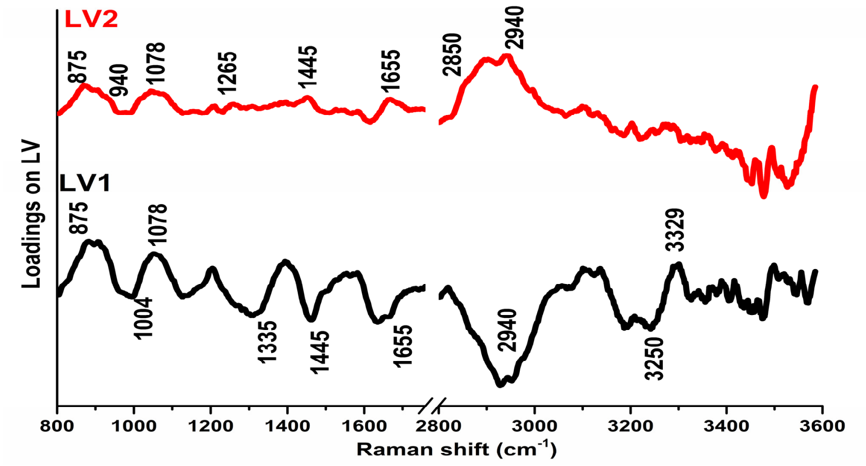

Fig. 4. The first two latent variables (LV1 and LV2) loadings with Raman spectral features for PLS-DA/LOO-CV algorithms development for laryngeal tissue classification (i.e., normal and carcinoma).

Figure 5 shows the scattered plots of posterior probability of Raman predictions for normal and carcinomatous larynx using PLS-DA/LOO-CV modeling for (a) integrated FP/HW, (b) FP, and (c) HW, respectively. The diagnostic accuracy with integrated FP/HW Raman spectroscopy is 91.1% (sensitivity of 93.3% (28/30) and specificity of 90.1% (64/71)), which is superior to using either FP (accuracy: 86.1%; sensitivity: 86.7% (26/30); specificity: 85.9% (61/71)) or HW (accuracy: 84.2%; sensitivity: 76.7% (23/30); specificity: 87.3% (62/71)) Raman technique alone. The receiver operating characteristic (ROC) curves are also generated [Fig. 6], with the integration areas under the ROC curves of being 0.968, 0.936 and 0.907, respectively, for the integrated FP/HW, FP and HW Raman techniques. The above

results confirm that the simultaneous FP and HW Raman technique yields the enhanced diagnostic performance for detecting laryngeal cancer *in vivo* during endoscopic examination.

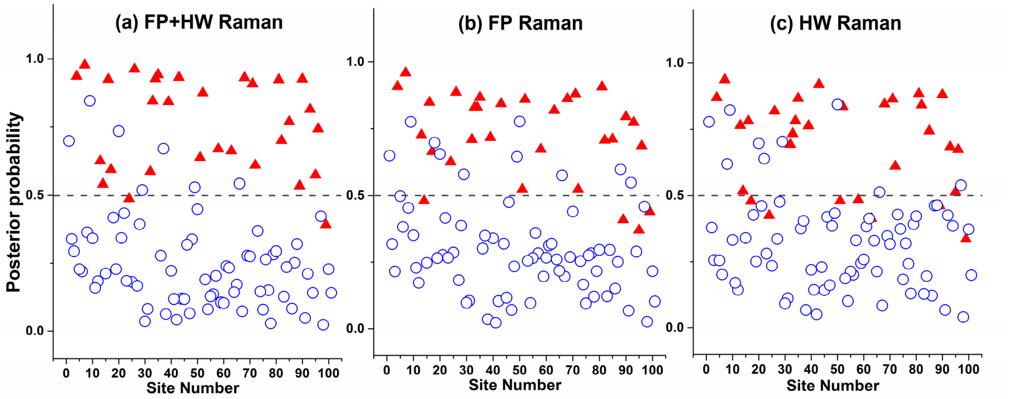

Fig. 5. Scattered plots of posterior probability of Raman predictions for normal and carcinomatous larynx using PLS-DA/LOO-CVmodeling for (a) integrated FP/HW, (b) FP, and (c) HW, respectively. (○) normal; and (▲) carcinoma.

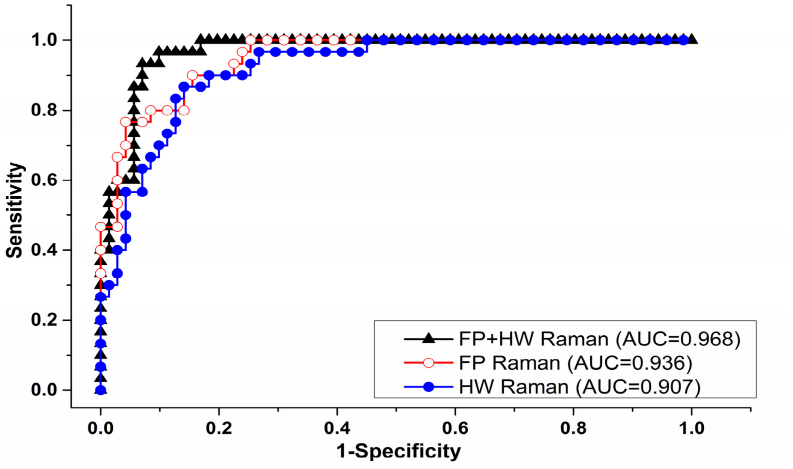

Fig. 6. Receiver operating characteristic (ROC) curves of the classification results for separating laryngeal cancer from normal tissue using the integrated FP/HW Raman, FP, and HW Raman techniques together with PLS-DA/LOO-CV methods. The areas under the ROC curves (AUC) are 0.968, 0.936 and 0.907, respectively, by using the integrated FP and HW, FP and HW Raman techniques.

In this work, high quality simultaneous FP and HW Raman spectra from normal and cancerous laryngeal tissues were successfully acquired *in vivo* in real-time during endoscopic examination. Significant Raman spectral differences in both the FP and HW Raman regions were observed between normal and cancerous laryngeal tissue, reconfirming the diagnostic potential of the integrated FP and HW Raman spectroscopy for improving *in vivo* diagnosis of neoplastic laryngeal lesions. For example, statistically different sub-regions of FP Raman bands (e.g., 800-955, 1000-1120, 1180-1230, 1235-1265, 1400-1470, 1500-1580 and 1590-1683 cm^{-1}) were identified [Fig. 2], uncovering the relative biochemical changes of diagnostically significant Raman bands associated with cancer development in the larynx. The Raman intensities at 875 cm^{-1} (ν(C-C) stretching of hydroxyproline) that are found to be significantly lower for cancer, indicating a reduction in the percentage of collagen contents

Biomedical Optics EXPRESS

relative to the total Raman-active components in the stroma layer of cancer tissue [8, 36]. We also found that the Raman peak at 940 cm^{-1} (proline and valine), 1078 cm^{-1} (lipids) is significantly decreased which may be due to thickening of the epithelium associated with cancerous progression that obscures the collagen Raman emission from the deeper tissue layers [37, 38]. In contrast, a significant increase of Raman intensity at 1335 cm^{-1} (CH_3CH_2 twisting of proteins and nucleic acids) is noted, which is probably related to the increased DNA content associated with carcinoma transformation in the larynx. These findings are in agreement with the molecular biology study findings of the increase of nucleic acids to lipids ratios in cancerous tissues [5, 39]. We also note that the bandwidth of the Raman peak at 1655 cm^{-1} (amide I v(C = O) of proteins) is broader for cancer tissue compared to normal laryngeal tissues; and this observation is in agreement with findings of our previous Raman study [8]. On the other hand, the HW Raman spectra [Fig. 2] offer exclusive new insights into the CH_2 and CH_3 stretching information and structure as well as the interactions of intra- and inter-cellular water *in situ*. Specifically, significant HW Raman spectra differences between normal and cancer laryngeal tissue were found at around 2850, 2885 cm^{-1} (symmetric and asymmetric CH_2 stretching of lipids) and 2940 cm^{-1} (CH_3 stretching of proteins). The higher Raman peak intensity ratio of I_{2940}/I_{2850} and I_{2940}/I_{2885} in laryngeal cancerous tissue is found, revealing a significant increase in protein contents while relatively decrease in lipid contents associated with the development of laryngeal cancer. The increased water content observed at ~3250 cm^{-1} for laryngeal cancer tissue reflects the re-arrangement of hydrogen-bonded networks in the epithelium layer. The changes of lipids, proteins and water contents asscociated with laryngeal carcinoma observed in this study are similar to other tissue types using Raman spectroscopy [9, 15, 17, 23, 28, 30, 31, 34, 40].

Further, we compare the diagnostic performance among the integrated FP/HW, FP, and HW Raman techniques for laryngeal cancer diagnosis using PLS-DA and LOO-CV methods. The PLD-DA and ROC analysis demonstrates the best diagnostic accuracy (91.1%) (sensitivity of 93.3% (28/30); specificity of 90.1% (64/71)) of the integrated FP and HW Raman spectroscopy for in vivo detection of laryngeal carcinoma. In addition, based on the current in vivo laryngeal tissue Raman data sets, we have also investigated the wavelength or wavenumber selection-based analysis in conjunction with nonlinear support vector regression (SVR) [41] for laryngeal cancer diagnosis. Wavenumber selection is performed using windows of wavenumber and leave-one tissue site-out cross-validated stepwise regression [42]. The best diagnostic performance is also confirmed with integrated FP and HW Raman technique using wavenumber selection and SVR methods, but an improved diagnostic accuracy (95.6%) is noted as compared to PLS full spectrum analysis. This indicates that the combination of feature selections with nonlinear calibration scheme can lead to a minimum allowable spectral subset required for establishing a robust but accurate model for rapid tissue Raman diagnosis at endoscopy. On the other hand, the enhanced tissue diagnosis using the simultaneous FP/HW Raman spectroscopy can partially be explained by back-tracing the incorrect predictions of each Raman modality. The FP Raman and HW Raman misclassified 14 (normal: 10, and cancer: 4) and 16 (normal: 9, and cancer: 7) tissue sites, respectively, of the total 60 patients recruited. The simultaneous FP and HW Raman modality reduced the number of misclassified predictions to 10 (normal: 8, and cancer: 2). The complementary biochemical/biomolecular properties of the integrated FP and HW Raman technique could greatly improve the *in vivo* diagnosis of laryngeal carcinoma at endoscopy. Further investigations reveal that the Raman signals of misclassified FP Raman spectra are extremely weak but with relatively higher AF level; while the addition of the HW modality containing more intense Raman peaks (e.g., 2885, 2940, 3250 and 3400 cm^{-1}) but comparably diminutive AF background could enhance the overall signal/noise ratio of the integrated FP/HW Raman spectra, and therefore improves the performance of the tissue classification with simultaneous FP/HW Raman spectroscopy. Therefore, clinically diagnostic rationales for combining FP and HW Raman spectroscopy for enhancing laryngeal carcinoma detection at endoscopy are

Biomedical Optics EXPRESS

manifold: for laryngeal tissues exhibiting intense AF overwhelming the tissue FP Raman signals, the HW Raman spectroscopy could still contain reasonably intense tissue HW Raman peaks associated with laryngeal tissue; further, the FP and HW Raman spectra provide complementary biochemical/biomolecular information, and the integrated FP/HW Raman spectroscopy could have significant advantages for improving in vivo tissue characterization and diagnosis.

4. Conclusions

We demonstrate for the first time that the simultaneous fingerprint (FP) and high-wavenumber (HW) fiber-optic Raman spectroscopy can be performed *in vivo* in real-time in the larynx at endoscopy. Significant FP/HW Raman spectral differences between normal and cancerous laryngeal tissue are observed. This study shows that the simultaneous FP/HW tissue Raman spectroscopy provides the best diagnostic performance for laryngeal cancer identification as compared to FP or HW Raman alone, illustrating the great potential of simultaneous FP/HW Raman technique for improving real-time *in vivo* detection and diagnosis of laryngeal cancer during endoscopic examination. Currently, clinical Raman studies on a larger series of patients using simultaneous FP/HW Raman spectroscopy technique are in progress at NUHS to further assess its clinical merits for early cancer detection and intraoperative tumor margins in the head and neck.

Acknowledgments

This work was supported by National Medical Research Council (NMRC) (CIRG/1331/2012; NMRC/BnB/0012b/2014; NMRC/BnB/0012c/2014), and Academic Research Fund (AcRF)-Tier 2 from Ministry of Education (MOE) (MOE2014-T2-1-010), Singapore.
