# Supplementary material for: The accuracy of Fiber-Optic Raman Spectroscopy in the detection and diagnosis of head and neck neoplasm in vivo: a systematic review and meta-analysis
Source: PeerJ. 2023 Dec 11;11:e16536. doi: 10.7717/peerj.16536 (PMC10720414; doi:10.7717/peerj.16536)
Supplement: Data S1 [file peerj-11-16536-s001.zip › article/sahu2015.pdf]

Journal of Innovative Optical Health Sciences
Vol. 9, No. 5 (2016) 1650017 (14 pages)
© The Author(s)
DOI: 10.1142/S1793545816500176

World Scientific
www.worldscientific.com

J. Innov. Opt. Health Sci. Downloaded from www.worldscientific.com
by 189.219.167.56 on 06/22/16. For personal use only.

In vivo subsite classification and diagnosis of oral cancers using Raman spectroscopy

Aditi Sahu*, Atul Deshmukh*, Arti R. Hole*, Pankaj Chaturvedi† and C. Murali Krishna*,‡

*Chilakapati Laboratory, ACTREC, TMC
Kharghar, Navi Mumbai 410210, India

†Head and Neck Surgical Oncology,
Tata Memorial Hospital, Dr. E. Borges Road,
Parel, Mumbai, Maharashtra- 400012, India
‡mchilakapati@actrec.gov.in; pittu1043@gmail.com

Received 21 August 2015
Accepted 10 November 2015
Published 12 January 2016

Oral cancers suffer from poor disease-free survival rates due to delayed diagnosis. Noninvasive, rapid, objective approaches as adjuncts to visual inspection can help in better management of oral cancers. Raman spectroscopy (RS) has shown potential in identification of oral premalignant and malignant conditions and also in the detection of early cancer changes like cancer-field-effects (CFE) at buccal mucosa subsite. Anatomic differences between different oral subsites have also been reported using RS. In this study, anatomical differences between subsites and their possible influence on healthy vs pathological classification were evaluated on 85 oral cancer and 72 healthy subjects. Spectra were acquired from buccal mucosa, lip and tongue in healthy, contralateral (internal healthy control), premalignant and cancer conditions using fiber-optic Raman spectrometer. Mean spectra indicate predominance of lipids in healthy buccal mucosa, contribution of both lipids and proteins in lip while major dominance of protein in tongue spectra. From healthy to tumor, changes in protein secondary-structure, DNA and heme-related features were observed. Principal component linear discriminant analysis (PC-LDA) followed by leave-one-out-cross-validation (LOOCV) was used for data analysis. Findings indicate buccal mucosa and tongue are distinct entities, while lip misclassifies with both these subsites. Additionally, the diagnostic algorithm for individual subsites gave improved classification efficiencies with respect to the pooled subsites model. However, as the pooled subsites model yielded 98% specificity and 100% sensitivity, this model may be more useful for preliminary screening applications. Large-scale validation studies are a pre-requisite before envisaging future clinical applications.

Keywords: Oral cancer; screening; *in vivo*; Raman spectroscopy; PC-LDA.

‡Corresponding author.

This is an Open Access article published by World Scientific Publishing Company. It is distributed under the terms of the Creative Commons Attribution 4.0 (CC-BY) License. Further distribution of this work is permitted, provided the original work is properly cited.

J. Innov. Opt. Health Sci. Downloaded from www.worldscientific.com
by 189.219.167.56 on 06/22/16. For personal use only.

1. Introduction

Oral cancer is the 16th most common cancer worldwide, with about 300,000 cases reported annually.[1] It is a major problem for South Asian countries like India, where it accounts for over 30% of all cancers.[2] In India, it is the most common cancer and the major cause of death in men, while being the third most common cancer in women.[3] Tobacco and alcohol abuse are major etiological factors.[4] Oral cancers suffer from a low five year disease free survival rate, attributed mainly to factors like delayed diagnosis and recurrence.[5] Lack of awareness regarding tobacco abuse, inadequate access to trained providers and limited health services pose difficulty in management of oral cancers.[6] These cancers can arise in one of the many subsites of the oral cavity. In Western countries, tongue (27.5%), floor of mouth (24.5%) and lip (19%) account for $\sim$70% of all cancers while in the Indian sub-continent, buccal mucosa and tongue along with lip are the most commonly affected subsites.[7,8] The anatomical location of cancer occurrence may also serve as a prognostic indicator. In spite of its amenability and accessibility to visual inspection, cancers in these oral subsites are frequently diagnosed at later stages, especially in developing countries where >60% cases are detected in advanced stages. This results in low treatment outcomes and considerable costs to patients who cannot afford healthcare.[9] Early diagnosis can lead to improved cure rates, lower cost of treatments and morbidity associated with oral cancers.[10,11]

Current diagnosis includes visual inspection followed by biopsy and histopathology of suspicious lesions. This procedure suffers from major disadvantages like patient noncompliance, time-consuming, subjectivity, errors like fatigue, sampling errors and inconsistency in interpretation especially in early/premalignant stages.[12,13] Thus, there is a need for alternative approaches to enable early, rapid, objective and noninvasive diagnosis of oral cancer and pre-cancers. During recent times, approaches like cytological methods, salivary based diagnostics and *in vivo* imaging and spectroscopy have been evaluated for early oral cancer diagnosis.[14] *In vivo* spectroscopic methods including optical coherence tomography (OCT),[15] high-resolution micro endoscopy (HRME),[16] elastic scattering spectroscopy (ESS),[17] fluorescence[18] and Raman spectroscopy (RS)[19,20] have shown potential in classifying healthy and malignant tissues. Additionally, RS,[21] fluorescence spectroscopy[22,23] and OCT[24] have shown potential in discriminating normal, premalignant and malignant conditions. RS has also shown to detect age-related physiological conditions and even subtle cancer-field effects (CFE).[25,26] However, these two studies undertaken in the present laboratory established the potential of RS for only buccal mucosa subsite. Additionally, two recent studies have also investigated possibility of surgical demarcation using tumor and normal biopsies.[27,28]

Development of diagnostic algorithms for oral cancer needs to take into consideration the anatomical differences between subsites. It is known that subsites in the oral cavity have distinct origins, and consequently distinct anatomical and molecular features.[29,30] Previous fluorescence[31] and reflectance spectroscopy[32,33] studies have demonstrated definite spectral contrasts between different subsites in healthy populations. Three major RS studies in the finger-print and high wavenumber region have also demonstrated differences arising due to epithelial and sub-epithelial structures, submucosa and degree of keratinization in subsites of healthy subjects and suggested clustering of sites based on anatomical and spectral similarities.[34–36] While the first *in vivo* study by Guze *et al.* suggested that spectra for different oral sites within the same ethnic group are significantly different and clearly separable, the consequent study by Bergholt *et al.* divided major subsites into three different clusters based on their histological and spectroscopic characteristics. The three groups included — (a) buccal mucosa, inner lip and soft palate, (b) dorsal, ventral tongue and floor of mouth, (c) gingiva and hard palate. Krishna *et al.* proposed division of subsites into four major anatomical clusters — (a) outer lip, and lip vermillion, (b) buccal mucosa (c) hard palate (d) dorsal, lateral and ventral tongue and soft palate. Further, the authors also suggest the use of anatomy-matched algorithms to increase discrimination between healthy and abnormal conditions.[37] There exist ambiguities in the anatomical classification of the buccal mucosa, tongue and lip subsites. Thus, in the present study, spectral contrast between the anatomical sites buccal mucosa, lip and tongue were investigated first in healthy as well different pathological conditions. Thereafter, the effect of this spectral contrast on efficiency of diagnostic

algorithms was explored. Findings are presented in the manuscript.

2. Materials and Methods

2.1. *Sample details*

Oral cancer patients visiting the outpatient department (OPD) of Tata Memorial Center (TMC), Mumbai were recruited for this study. Eighty-five subjects with tobacco-associated pathologically verified squamous cell carcinoma (SCC) lesions in the oral cavity were recruited. The average age for oral cancer patients was found to be 49 years and the male: female ratio for these patients was 5:1. From these patients, spectra were acquired from contralateral normal regions and tumors at buccal mucosa, lip and tongue in the oral cavity. Premalignant patches like leukoplakia and condition like oral submucous fibrosis were observed in buccal mucosa, lip and tongue sites of some patients. Spectra were acquired from these premalignant areas on buccal mucosa, lip and tongue. Multiple spectra (~ 3) were recorded from each contralateral normal, premalignant or malignant site. Information about tobacco usage, age, sex and tumor grade of all subjects was obtained from the electronic medical record (EMR) system of Tata Memorial Hospital, Mumbai, India. Most of the tumors were moderately-differentiated SCCs followed by poorly- and well-differentiated carcinomas. Oral cancer patients mostly belonged to late stages-T3 or T4.

A total of 72 subjects from Advanced Center for Treatment, Research, Education in Cancer (ACTREC), Mumbai were recruited as healthy volunteers (HV). Subjects recruited for the study comprised of both genders between the age group 21–60 years. The mean age of healthy subjects and the male:female ratio was 36 years and 3:1, respectively. Like patients, spectra were acquired from three sites — buccal mucosa, lip, tongue. Subjects with no current/past tobacco or alcohol habits and no history of malignancy were considered as HV. To avoid any differences because of the mouth environment, subjects were required to wash their mouth with distilled water before spectral acquisitions. Photographs and schematics representing the spectra acquisition scheme are shown in Fig. 1. All subjects were recruited only after obtaining an informed and written consent. The study was approved by the institutional review board (IRB). A summary of subject accrual and demographics is presented in Table 1.

2.2. *Raman spectroscopy*

Spectra were recorded with an HE-785 commercial Raman spectrometer (Jobin-Yvon-Horiba, France).[25] This system consists of a diode laser (Process Instruments, USA) of 785 nm wavelength as the excitation source, a high efficiency (HE) spectrograph with fixed 950 gr/mm grating coupled with a CCD (Synapse). The instrument has no movable parts and the spectral resolution as specified by the manufacturer is $4\,\text{cm}^{-1}$. The commercially available Inphotonics (Inphotonics Inc, Downy St. USA) probe consisting of a $105\,\mu$m excitation fiber and a $200\,\mu$m collection fiber (NA-0.40) was used to couple the excitation source and the detection system. The estimated spot size and depth of the field as per the manufacturer's specifications are $105\,\mu$m and 1 mm, respectively. The working distance of the probe is 5 mm and therefore, a detachable spacer of length 5 mm was attached at the tip of the probe to maintain focus during all measurements. Prior to each measurement, these spacers were disinfected by CIDEX (Johnson and Johnson, Mumbai, India) solution to avoid inter-subject contamination. Spectral acquisition parameters were: λ_{ex} — 785 nm, laser power — 80 mW, spectra were integrated for 3 s and averaged over three accumulations. The schematic of a fiber-optic Raman instrument is presented in Fig. 2.

As spectra were recorded directly on patients, certain important logistics were considered before the study was initiated. Disinfection of the stainless steel spacers was meticulously carried out to ensure patient safety. Acquisition time was patient and clinician friendly to obviate movements during spectral acquisition and obtain good quality spectra. The laser power at the sampling point employed in the present study (80 mW) was well within the maximum permissible exposure allowed based on the American National Standards Institute (ANSI) regulations. To prevent variability from physician to physician in terms of probe placement and effect of varying pressures applied to probe during acquisition, spectral acquisition was carried out at pre-designated sites and spectra was acquired by a single clinician for all subjects. Spectra were acquired from specific sites; from the buccal mucosa, spectra were acquired from mucosa

J. Innov. Opt. Health Sci. Downloaded from www.worldscientific.com by 189.219.167.56 on 06/22/16. For personal use only.

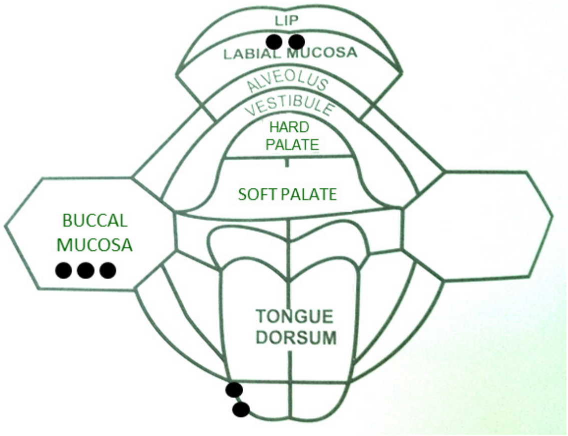

(a)

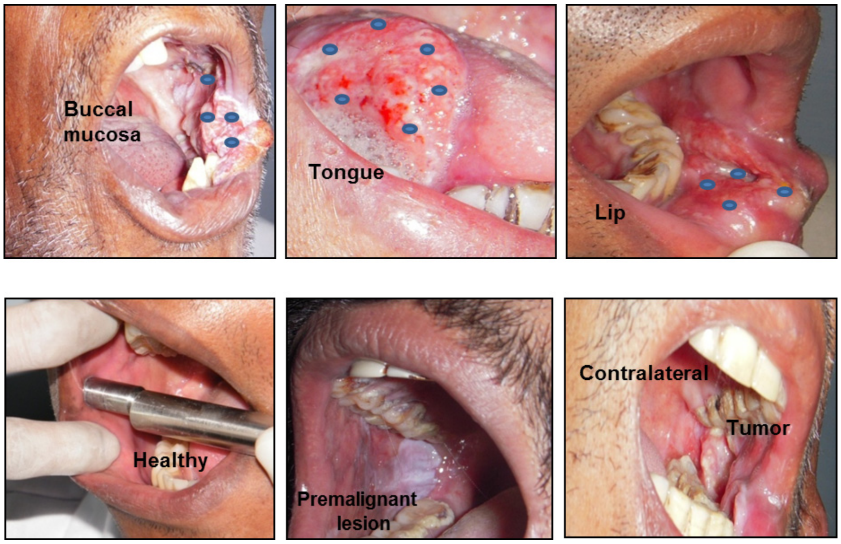

(b)

Fig. 1. (a) Schematic representation of spectra acquisition sites at buccal mucosa, lip and tongue in healthy and contralateral subsites, (b) Photographical representation of spectral acquisition scheme for spectral acquisition from tumors on buccal mucosa, tongue and lip in the first panel and spectral acquisition scheme from healthy, premalignant, contralateral and tumor sites for a particular subsite (buccal mucosa) in the second panel.

J. Innov. Opt. Health Sci. Downloaded from www.worldscientific.com by 189.219.167.56 on 06/22/16. For personal use only.

opposing teeth positions of second pre-molar, first molar and second molar. In case of lip, labial mucosa opposing the two central incisors was selected for spectral acquisition. For tongue, spectra were acquired from lateral borders in the anterior part of the tongue adjacent to the premolar and molar teeth.

2.3. *Spectral pre-processing and data analysis*

Spectra were corrected for CCD response with a National Institute of Science and Technology (NIST) certified standard reference material 2241 (SRM 2241) followed by the subtraction of background signals from optical elements and substrate. To remove interference of the slow moving background, first derivatives of spectra (Savitzky–Golay method and window size 3) were computed.[38,39] Spectra were interpolated in 1200–1800 cm^{1}, vector-normalized and used as input for multivariate analysis. Multivariate tool principal component linear discriminant analysis (PC-LDA) followed by leave-one-out-cross-validation (LOOCV) was used for data analysis. LDA can be used in conjunction with PCA (PC-LDA) to increase the efficiency of classification. The advantage of doing this is to

Table 1. Summary of subject accrual and demographics.

Sr. No.	Category	No. of subjects	Age range/ median age (years)	Gender ratio (male: female)	Tobacco habits	Spectra acquired
1.	Healthy	72	21–60 years/36 years	3:1	No	666
	-Buccal mucosa					−353
	-Lip					−137
	-Tongue					−176
2.	Oral cancer	85	27–82 years/49 years	5:1	Yes	
	• Tumor	85				251
	-Buccal mucosa					−118
	-Lip					−11
	-Tongue					−122
	• Contralateral	85				580
	-Buccal mucosa					−233
	-Lip					−164
	-Tongue					−184
	• Premalignant	40				106
	-Buccal mucosa					−82
	-Lip					−7
	-Tongue					−17

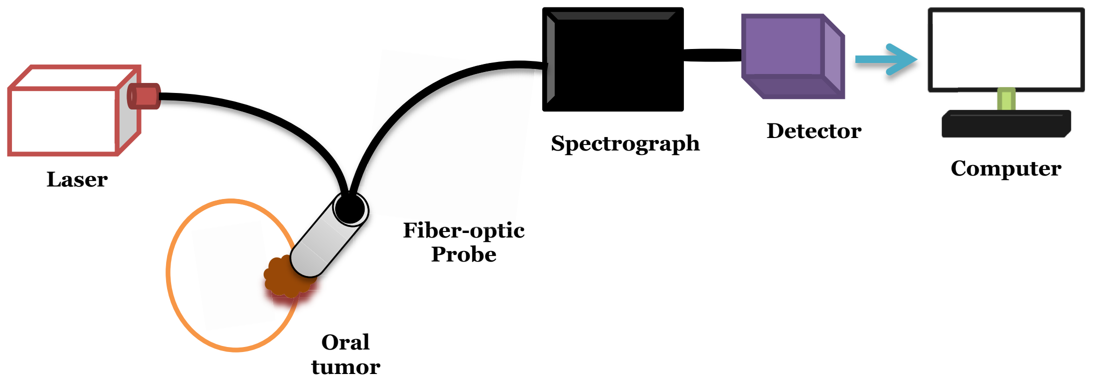

Fig. 2. Schematic representation of fiber-optic Raman spectrometer.

J. Innov. Opt. Health Sci. Downloaded from www.worldscientific.com by 189.219.167.56 on 06/22/16. For personal use only.

remove or minimize noise from the data and concentrate on variables important for classification. In our analysis, significant principal components ($p < 0.05$) were selected as input for LDA. In order to avoid over-fitting of the data, as a thumb rule, total number of factors selected for analysis were less than half the number of the spectra in the smallest group.[40–42] During PC-LDA, use of less than 10 PC factors for LDA is the best compromise on the information being included and data noise being excluded.[43] During PC-LDA, several factors were explored for classification and only factors with minimum over-fitting and maximum classification efficiency were finally selected for analysis. PC-LDA models were validated by LOOCV. Algorithms for these analyses were implemented in MATLAB (Mathworks Inc., USA) based in-house software.[44]

Average spectra were also computed for spectral comparisons across the groups. Background-corrected spectra were baseline corrected prior to derivatization by fitting a fifth-order polynomial function. These baseline-corrected, smoothed (Savitzky–Golay, 3) and normalized spectra were used for spectral comparisons.

3. Results

3.1. *Spectral analysis*

Mean spectra for healthy, contralateral normal, premalignant and tumor for buccal mucosa, lip and

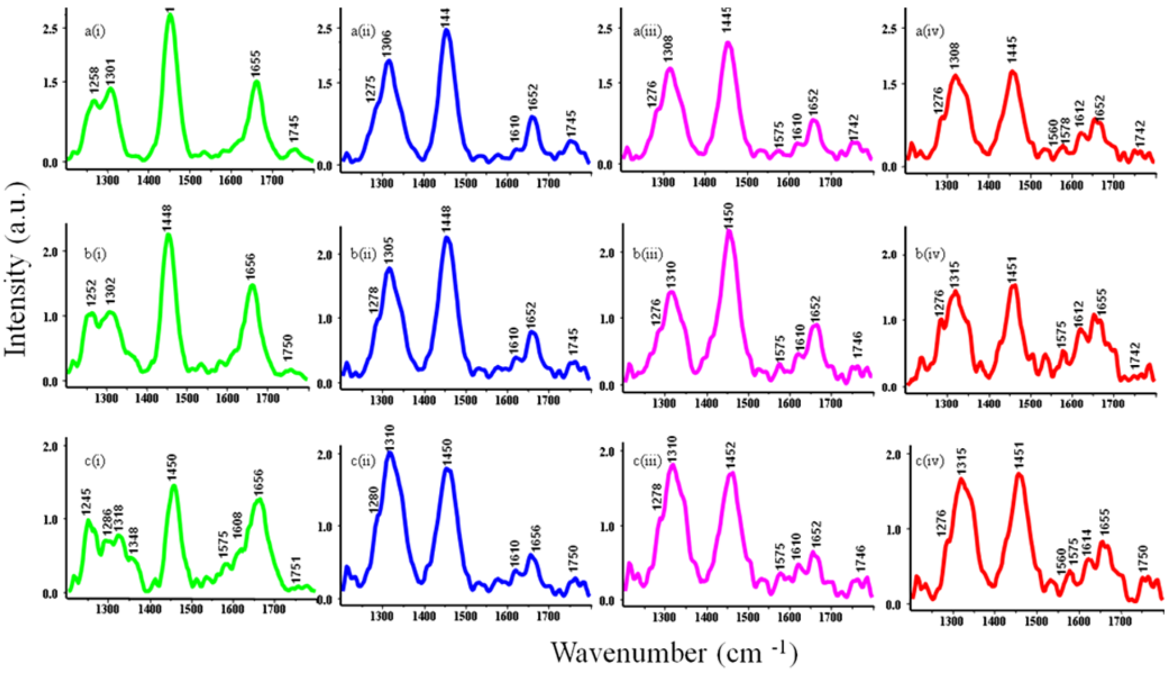

Fig. 3. Average spectra for subsites (a) Buccal mucosa, (b) Lip, (c) Tongue in (i) healthy (green), (ii) contralateral (blue), (iii) premalignant (pink) and (iv) tumor (red) conditions.

tongue have been shown in Figs. 3(a)–3(c). Spectral features of healthy buccal mucosa (Fig. 3(a)(i)) demonstrate two sharp features in the amide III region, strong δCH_2 bend and sharp peak in the amide I region and an ester band at around 1742 cm^{-1}. Thus, important contributions from lipids are observed in the healthy spectra. Similar features were observed for healthy lip spectra (Fig. 3(b)(i)), along with broader amide III and amide I regions, indicating protein contributions in the spectra. In case of tongue (Fig. 3(c)(i)), features like broad amide III, amide I along with CH deformation and CH_2, CH_3 wagging (collagen assignment) at 1343 cm^{-1} was prominently observed.

With increase in severity of pathological condition, an increase in protein-related features like broadening of amide III and amide I, shifted amide III and shifted δCH_2 bend were observed for all groups (Figs. 3(a)(ii)–(iv), 3(b)(ii)–(iv) and 3(c)(ii)–(iv)). DNA and heme-related features were also observed at 1340 cm^{-1}, 1480 cm^{-1} and 1560 cm^{-1} in these groups. Thus, spectra from contralateral, premalignant and tumor had increased contribution from proteins, DNA and heme. These findings corroborate our previous *ex vivo* and *in vivo* findings.[19–21,25,26] The tentative assignments were made as per available literature.[45,46]

3.2. *Exploring anatomical differences in healthy, contralateral, premalignant and tumor conditions*

The anatomical differences between the three subsites — buccal mucosa, lip and tongue were explored in healthy, contralateral, premalignant and malignant conditions.

3.2.1. *Anatomical differences in healthy subsites*

To investigate spectroscopically apparent anatomical differences, 666 spectra were acquired from buccal mucosa (353 spectra), lip (137 spectra) and tongue (176 spectra) of 72 HV. Spectral features for healthy buccal mucosa, lip and tongue shown in Figs. 3(a)(i), 3(b)(i) and 3(c)(i) indicate two sharp features in the amide III region, strong δCH_2 bend and sharp peak in the amide I region, ester band at around 1742 cm^{-1} for buccal mucosa; broad features in amide III, amide I and δCH_2 deformation in the lip spectra; and features around 1240 cm^{-1}, broad amide III and amide I regions, CH_2, CH_3 wagging (collagen assignment) at 1343 cm^{-1} indicate a predominantly protein-dominated spectrum in tongue.

J. Innov. Opt. Health Sci. Downloaded from www.worldscientific.com by 189.219.167.56 on 06/22/16. For personal use only.

Table 2. PC-LDA for identifying anatomical differences at buccal mucosa, lip and tongue subsites in oral cavity of healthy subjects.

	Buccal mucosa	Lip	Tongue	Total
Buccal mucosa	298	52	3	353
Lip	31	62	44	137
Tongue	1	32	143	176

PC-LDA was carried out in the next step using 3 factors accounting for ~72% variance. LOOCV findings shown in Table 2 indicate that of the 353 buccal mucosa spectra, 298 were correctly identified while 52 and 3 misclassified with lip and tongue, respectively. For lip, 62/137 spectra (45.2%) were correctly classified, while 31 and 44 misclassified with buccal mucosa and tongue, respectively. In case of tongue, 143/176 spectra were correctly classified, 32 misclassified as lip. Thus, buccal mucosa and tongue classified as almost distinct subsites, and major overlap of both was observed with the intermediate subsite lip.

3.2.2. *Anatomical differences in contralateral subsites*

Five-hundred eighty one spectra were acquired from buccal mucosa (233 spectra), lip (164 spectra) and tongue (184 spectra) subsites to investigate differences in contralateral subsites. The spectral differences are shown in Figs. 3(a)(ii), 3(b)(ii) and 3(c)(ii) indicate sharp features at $1306\,cm^{-1}$, $1445\,cm^{-1}$, $1655\,cm^{-1}$ and $1744\,cm^{-1}$ for buccal mucosa; broader features at amide III, amide I and δCH_2 deformation for lip; while for tongue, broad amide III, collagen features at 1343, features at $1440\,cm^{-1}$ and $1450\,cm^{-1}$, and $1648\,cm^{-1}$ and $1660\,cm^{-1}$ were observed.

These spectra were then subjected to PC-LDA using two factors accounting for 66% variance. Out of 233 buccal mucosa spectra, 161 were correctly classified, 54 and 18 misclassified as lip and tongue, respectively. 73/164 lip spectra were correctly classified (~44%), while 41 and 50 misclassified as buccal mucosa and tongue. For tongue, 149/184 spectra were correctly predicted as tongue, 33 and 2 misclassified as lip and buccal mucosa (Table 3). Thus, like healthy subsites, contralateral buccal mucosa and tongue are almost distinct subsites, and overlap of both is majorly observed with lip.

Table 3. PC-LDA for identifying anatomical differences at buccal mucosa, lip and tongue subsites in contralateral conditions.

	Buccal mucosa	Lip	Tongue	Total
Buccal mucosa	161	54	18	233
Lip	41	73	50	164
Tongue	2	33	149	184

3.2.3. *Anatomical differences in premalignant subsites*

Premalignant conditions like leukoplakia and oral sub-mucous fibrosis on buccal mucosa and leukoplakia on lip and tongue were used for spectral acquisition and 106 spectra were acquired- 82 spectral buccal mucosa, 7 spectra from lip and 17 spectra from tongue. Spectra from premalignant lesions at buccal mucosa, lip and tongue shown in Figs. 3(a)(iii), 3(b)(iii) and 3(c)(iii) indicate similar spectral profiles, with major features at $1306\,cm^{-1}$, $1448\,cm^{-1}$, $1650\,cm^{-1}$ and $1750\,cm^{-1}$. Minor spectral shifts were observed for lip and tongue at amide III and only tongue in CH_2 deformation region.

PC-LDA was carried out using 3 factors covering ~77% classification efficiency. As shown in LOOCV findings in Table 4, 58/82 (70%) buccal mucosa spectra were correctly identified while 9 and 15 misclassified as lip and tongue. The seven spectra for lip mostly misclassified with buccal mucosa (4) and tongue (1), Two were correctly predicted as lip. For tongue, 15/17 were correctly identified as tongue leukoplakia. In the premalignant lesion/condition found on these subsites, buccal mucosa and tongue could be distinctly identified.

3.2.4. *Anatomical differences in malignant subsites*

To explore spectroscopic differences between tumors at different subsites, 251 spectra were acquired from SCC at buccal mucosa (118 spectra), lip

Table 4. PC-LDA for identifying anatomical differences at buccal mucosa, lip and tongue subsites in premalignant conditions.

	Buccal mucosa	Lip	Tongue	Total
Buccal mucosa	58	9	15	82
Lip	4	2	1	7
Tongue	1	1	15	17

J. Innov. Opt. Health Sci. Downloaded from www.worldscientific.com by 189.219.167.56 on 06/22/16. For personal use only.

Table 5. PC-LDA for identifying anatomical differences at buccal mucosa, lip and tongue subsites in tumor conditions.

	Buccal mucosa	Lip	Tongue	Total
Buccal mucosa	49	21	48	118
Lip	5	2	4	11
Tongue	38	4	80	122

(11 spectra) and tongue (122 spectra). As shown in Figs. 3(a)(iv), 3(b)(iv) and 3(c)(iv), major spectral features observed in all spectra were 1310 cm^{-1}, 1450 cm^{-1}, 1615 cm^{-1}, 1648 cm^{-1}, representing amide III, CH_2 deformation, C=C in protein and amide I. Almost same spectral profiles with minor intensity variations were observed for all subsites.

PC-LDA was carried out using two factors accounting for ~52% correct classifications. Of the 118 buccal mucosa tumor spectra, only 49 were correctly predicted. For 11 lip tumor spectra, two were correctly identified while for 122 tongue tumor spectra, 80 were correctly classified as tongue (Table 5).

3.3. *RS-based diagnostics for oral cancers at different subsites*

To enable accurate tissue diagnosis and characterization, the influence of the anatomical variations on normal vs abnormal classification using RS was confirmed. RS of healthy and pathological-contralateral normal, premalignant and malignant was undertaken for both, individual subsites and pooled subsites. In the first step, efficiency of classification for — healthy, contralateral normal, premalignant and malignant condition at individual subsites-buccal mucosa, lip and tongue was explored. In the final step, spectra from all subsites (buccal mucosa, lip and tongue) were clustered and efficiency of RS in classifying healthy vs pathology was explored.

3.3.1. *Efficiency of RS in discriminating healthy from pathology at individual subsites: Buccal mucosa*

To explore efficiency of diagnostic algorithm at buccal mucosa subsite, 353 healthy, 233 contralateral normal, 82 premalignant and 118 malignant spectra were acquired. Spectral features from healthy, contralateral normal, premalignant and malignant conditions shown in Figs. 3(a)(i)–(iv) demonstrate sequential decrease in lipid and increase in protein features. Heme-related features that signify increased presence of blood were observed in tumor spectra.

These spectra were then subjected to PC-LDA using five factors (~82% classification efficiency). As shown in Table 6, of the 353 healthy spectra, 349 were correctly classified, 162/233 spectra contralateral were correctly classified, 46/82 and 85/118 tumor were correctly predicted. Less misclassification between the pathological groups — contralateral normal, premalignant and malignant was observed. Overall classification efficiency for healthy, contralateral normal, premalignant and malignant was found to be 99%, 70%, 56% and 72%.

3.3.2. *Efficiency of RS in discriminating healthy from pathology at individual subsites: Lip*

A total of 137 healthy, 164 contralateral normal, 7 premalignant and 11 malignant spectra were acquired from lip. Spectral features shown in Figs. 3(b)(i)–(iv) demonstrate decrease in lipid-like features in pathological conditions. Spectra from contralateral and premalignant sites show very similar features while broad amide III, amide I and heme-related features were observed in tumor spectra.

PC-LDA was carried out using two factors accounting for 79% efficiency. LOOCV findings shown in Table 7 indicate 131/137 healthy, 106/164 contralateral, 2/7 premalignant and 5/11 tumor

Table 6. PC-LDA for efficiency of RS in identifying healthy, contralateral, premalignant and tumor conditions at buccal mucosa subsite.

	Healthy	Contralateral	Premalignant	Tumor	Total
Healthy	349	3	0	1	353
Contralateral	0	162	64	7	233
Premalignant	0	21	46	15	82
Tumor	0	2	31	85	118

J. Innov. Opt. Health Sci. Downloaded from www.worldscientific.com by 189.219.167.56 on 06/22/16. For personal use only.

Table 7. PC-LDA for efficiency of RS in identifying healthy, contralateral, premalignant and tumor conditions at lip subsite.

	Healthy	Contralateral	Premalignant	Tumor	Total
Healthy	131	0	0	6	137
Contralateral	0	106	52	6	164
Premalignant	0	4	2	1	7
Tumor	0	0	6	5	11

spectra were correctly predicted. Overall classification efficiency for healthy, contralateral normal, premalignant and malignant was found to be 96%, 64%, 29% and 45%. The findings will have to be validated after inclusion of additional lip tumor and lip leukoplakia cases.

3.3.3. *Efficiency of RS in discriminating healthy from pathology at individual subsites: Tongue*

Spectra were acquired from healthy (176 spectra), contralateral normal (184 spectra), premalignant (17 spectra) and malignant (122 spectra) conditions on tongue. Spectra shown in Figs. 3(c)(i)–(iv) indicate changes in protein and lipid content in the healthy and pathological conditions. Spectral shifts and intensity-variations were observed at amide III, I and CH_2 deformation.

These spectra were subjected to PC-LDA using two factors accounting for ~74% correct classifications. 173/176 healthy spectra, 119/184 contralateral, 2/17 premalignant and 79/122 tumor spectra were correctly classified (Table 8). Thus, overall classification rates were identified at 98%, 65%, 11% and 65% for healthy, contralateral normal, premalignant and malignant, respectively.

3.3.4. *Efficiency of RS in discriminating healthy from pathology at pooled subsites: BM, lip and tongue*

Spectra from buccal mucosa, lip and tongue were grouped together as per their pathological status: healthy, contralateral normal, premalignant and malignant and PC-LDA was carried out using 10 factors (~76% efficiency of classification). LOOCV findings shown in Table 9 indicate that out of 666 healthy buccal, lip and tongue spectra, 657 were correctly predicted, 1 and 8 spectra misclassified with contralateral and tumor. For contralateral, 331/580 spectra were correctly classified, 167 and 82 misclassified as premalignant and tumor. 47/106 premalignant were correctly predicted, 43 and 16 misclassified with contralateral and tumor. Out of 251 tumor spectra, 174 correctly classified while 39

Table 8. PC-LDA for efficiency of RS in identifying healthy, contralateral, premalignant and tumor conditions at tongue subsite.

	Healthy	Contralateral	Premalignant	Tumor	Total
Healthy	173	1	1	1	176
Contralateral	0	119	31	34	184
Premalignant	0	7	2	8	17
Tumor	0	29	14	79	122

Table 9. PC-LDA for efficiency of RS in identifying healthy, contralateral, premalignant and tumor conditions at pooled subsites.

	Healthy	Contralateral	Premalignant	Tumor	Total
Healthy	657	1	0	8	666
Contralateral	0	331	167	82	580
Premalignant	0	43	47	16	106
Tumor	0	39	38	174	251

J. Innov. Opt. Health Sci. Downloaded from www.worldscientific.com by 189.219.167.56 on 06/22/16. For personal use only.

and 38 were misclassified as contralateral and premalignant. Thus, overall classification rates were identified at 97.8%, 60.6%, 30.1% and 76.4% for healthy, contralateral normal, premalignant and malignant, respectively. No misclassifications of pathological conditions were observed with healthy. Therefore, sensitivity and specificity using the pooled subsite model was 100% and 98%, respectively.

4. Discussion

The development of new, rapid, noninvasive and objective techniques for oral cancer diagnosis can improve patient compliance rates and enable early diagnosis. Optical spectroscopy like OCT, Raman and Fluorescence have shown potential in distinctly identifying normal, premalignant and malignant conditions.[15,18,20,21,24] Most of these studies have focused on a single oral subsite while others have pooled subsites into matching clusters and demonstrated their respective efficacies. Previous studies on finger-print and high wavenumber region have demonstrated that buccal and labial mucosa, keratinized or masticatory mucosa and specialized mucosa on the tongue can be classified as distinct clusters of subsites.[34–36] While some studies indicate that these subsite-anatomical differences confound the healthy and pathological discrimination,[37] other studies have stated that the inherent anatomical differences may not hinder healthy vs pathological classification.[47] In the present study, Raman spectroscopic differences between the three common subsites of oral cancer development in the Indian subcontinent-buccal mucosa, movable mucosa of the lip and tongue were evaluated. In the first step, differences between subsites-buccal mucosa, lip and tongue in healthy, contralateral normal, premalignant and malignant subjects were explored. In the next step, effect of anatomical differences in the normal vs abnormal classification was explored. To achieve this, healthy vs pathological classification was first explored at individual subsites, followed by evaluating efficiency of the algorithm in a combined model (buccal mucosa, lip and tongue). PC-LDA followed by LOOCV was employed to explore classification.

Spectral analysis across all groups was carried out. Major spectral differences between buccal mucosa, lip and tongue were found to be with respect to lipid and protein content, especially in the healthy and contralateral conditions. While buccal mucosa had high lipid features, tongue had a protein dominated spectrum while lipid had features from both lipids and proteins. As buccal mucosa has a characteristic fatty submucosa and tongue being highly muscular in nature, these findings can be explained. No major differences were observed in spectra from premalignant and malignant conditions. Findings demonstrate higher lipid content in most healthy conditions while higher DNA, heme and protein-related features in severe pathological conditions. The change from lipid to protein and DNA can be explained on the basis of changes in tissue architecture and morphology, increased angiogenesis and cellular proliferation in premalignant and malignant conditions.

In the analysis for investigating spectral contrasts at the three anatomical sites, it was observed that RS-based discrimination between buccal mucosa, lip and tongue reduced sequentially from healthy to the most severe pathological condition (tumor), with total loss of anatomical influence in tumor conditions. It is known that OSCC at different sites are histopathologically a SCC, and therefore the site of cancer cannot be detected based on histopathology of the tumor specimens.[48] Due to loss of information from the underlying architectural arrangement and supposedly seeming similarity in SCC irrespective of subsite, tumor subsites could not be classified. The similarity of lip subsite with both buccal mucosa and tongue could be explained due to the fundamental anatomical characteristics of this subsite. Although both buccal mucosa and lip are covered with the same lining mucosa, the underlying connective tissue and submucosa are different for these subsites. The similarity in buccal and lip could be preliminary attributed to the presence of a thick, stratified, squamous, nonkeratinizing mucosa at both sites. This could be the basis of what has been previously reported by Bergholt *et al.* and Guze *et al.* However, below the labial lining mucosa and dense fibrous connective tissues and the submucosa consisting of collagen and elastin fibers interspersed with fat and small mixed glands, orbicularis oris muscle is located. In case of buccal mucosa, beyond the thick nonkeratinized epithelium, dense fibro-elastic tissue from lamina propria penetrate the fatty elastic submucosa consisting prominently of loose connective tissue, large blood vessels, nerves, salivary glands and adipose tissue. In contrast, tongue is lined by a specialized mucosa below which thin, papillated lamina propria connect the underlying

J. Innov. Opt. Health Sci. Downloaded from www.worldscientific.com by 189.219.167.56 on 06/22/16. For personal use only.

J. Innov. Opt. Health Sci. Downloaded from www.worldscientific.com
by 189.219.167.56 on 06/22/16. For personal use only.

compact masses of skeletal muscle fibers. Thus, due to the overlying epithelium and small similarities in lamina propria and submucosa, buccal mucosa and lip misclassify with each other. Due to presence of underlying skeletal muscle fibers of orbicularis oris, lip misclassifies with tongue, majorly composed of skeletal muscle fibers.[49–51] These anatomical attributes give lip an intermediate position in subsite classification. However, in lieu of observed similarities and disparities, this inter-anatomical variability should have to be verified for Raman based oral cancer diagnostics.

In the next step, effect of this apparent anatomical variability on healthy vs pathological classification was assessed in clustered and independent sites. It was observed that PC-LDA of individual subsites yielded enhanced overall outcomes. Misclassifications observed between the pathological conditions contralateral normal, premalignant and malignant could be explained due to the inherent heterogeneity of the premalignant and tumor lesions. Furthermore, for all tumor spectral acquisitions, the advancing front of the tumor was also probed along with the center of the malignancy. Several normal sites probed during the advancing front measurements cannot be ruled out.[47] Early malignancy associated changes (MAC)/CFE changes in the contralateral mucosa of the patients because of chronic tobacco exposure may also explain the misclassification between contralateral and tumor. In case of tongue, increased misclassification between contralateral and tumor were observed. Contralateral tongue sites may not be an ideal normal for comparison with tumors. Internal infiltration of tumor to the contralateral site without obvious external clinical presentation may be a putative reason for these increased misclassifications. The misclassifications of contralateral with tumor were observed mostly for advanced stages of disease, when the tumor reaches or crosses tongue midline.

As premalignant lesions were found in the oral cavity of the oral cancer patients, misclassifications between the contralateral normal and premalignant conditions can be understood. Further, like malignant sites, premalignant sites may also be characterized by dysplastic regions. Thus, misclassifications between these sites can also be understood.

It is noteworthy that in spite of the slightly higher classification rate observed for the individual subsites, the pooled subsites analysis also yielded satisfactory classification rates of 97.8%, 60.6%, 30.1% and 76.4% for healthy, contralateral normal, premalignant and malignant, respectively. Thus, healthy could be predicted as healthy with 98% efficiency. Further as no abnormal/pathological spectra misclassified with the healthy group, abnormal group could be identified with a sensitivity of 100%. Therefore, the pooled subsites standard model differentiated between healthy and abnormal conditions with 100% sensitivity and 98% specificity. Such a standard model inclusive of all subsites may be a more desirable and practical approach for preliminary screening applications as their main aim is to distinguish between normal and all other pathological conditions. After screening using this model, sites predicted as abnormal/pathological can be confirmed using other confirmatory procedures like biopsy followed by histopathological evaluation. The high misclassification between the pathological conditions in the "pooled-subsites" model could possibly be attributed to the inherent anatomical disparities. It is possible that detection of premalignant and early cancer states may benefit from sub-classification of sites. In the present study, very few numbers of premalignant and malignant subjects could be recruited, especially for the lip and tongue subsites. Due to these reasons, a conclusive statement on the efficacy of the diagnostic algorithm at these subsites cannot be made. Future studies using higher number of subjects with premalignant and malignant lesions at lip and tongue will have to be conducted to verify the actual potential of the pooled subsites model in preliminary oral cancer screening and the need for a subsite specific diagnostic algorithm for early pre-cancer and cancer diagnosis.

Competing Financial Interests

The authors declare that they have no competing interests.

Acknowledgment

The Raman spectrometer employed in the study was procured from DBT project BT/PRI11282/MED/32/83/2008, entitled "Development of *in vivo* laser RS methods for diagnosis of oral precancerous and cancerous conditions", Department of Biotechnology, Government of India. The authors would like to acknowledge all HV, patients who participated in

the study and Mr. Pramod Tawde for technical assistance.

References

1. J. Ferlay, I. Soerjomataram, R. Dikshit, S. Eser, C. Mathers, M. Rebelo *et al.*, "Cancer incidence and mortality worldwide: Sources, methods and major patterns in GLOBOCAN 2012," *Int. J. Cancer* **136**, E359–E386 (2015).
2. Z. U. Khan, "An overview of oral cancer in Indian subcontinent and recommendations to decrease its incidence," *WebmedCentral CANCER* **3**(8), WMC003626 (2012).
3. R. Sankaranarayanan, K. Ramadas, G. Thomas, R. Muwonge, S. Thara, B. Mathew *et al.*, "Effect of screening on oral cancer mortality in Kerala, India: A cluster-randomised controlled trial," *Lancet* **365**, 1927–1933 (2005).
4. M. Hashibe, P. Brennan, S. Benhamou, X. Castellsague, C. Chen, M. P. Curado *et al.*, "Alcohol drinking in never users of tobacco, cigarette smoking in never drinkers, and the risk of head and neck cancer: Pooled analysis in the International Head and Neck Cancer Epidemiology Consortium," *J. Nat. Cancer Inst.* **99**, 777–789 (2007).
5. S. Khandekar, P. Bagdey, R. Tiwari, "Oral cancer and some epidemiological factors: A hospital based study," *Indian J. Commun. Med.* **31**, 157–159 (2006).
6. S. Kumar, R. Heller, U. Pandey, V. Tewari, N. Bala, K. Oanh, "Delay in presentation of oral cancer: A multifactor analytical study," *Nat. Med. J. India* **14**, 13–17 (2001).
7. R. E. Pollock, J. H. Doroshow, *UICC Manual of Clinical Oncology*, Wiley-liss, New York (2004).
8. S. Carnelio, G. Rodrigues, "Oral cancer at a glance," *Internet J. Dent. Sci.* **1**, 1–12 (2004).
9. S. Warnakulasuriya, "Global epidemiology of oral and oropharyngeal cancer," *Oral Oncol.* **45**, 309–316 (2009).
10. S. S. Napier, P. M. Speight, "Natural history of potentially malignant oral lesions and conditions: An overview of the literature," *J. Oral Pathol. Med.* **37**, 1–10 (2008).
11. I. van der Waal, "Potentially malignant disorders of the oral and oropharyngeal mucosa; present concepts of management," *Oral Oncol.* **46**, 423–425 (2010).
12. C. F. Poh, S. Ng, K. W. Berean, P. M. Williams, M. P. Rosin, L. Zhang, "Biopsy and histopathologic diagnosis of oral premalignant and malignant lesions," *J. Canad. Dent. Assoc.* **74**, 283–288 (2008).
13. A. Karabulut, J. Reibel, M. H. Therkildsen, F. Prætorius, H. Nielsen, E. Dabelsteen, "Observer variability in the histologic assessment of oral premalignant lesions," *J. Oral Pathol. Med.* **24**, 198–200 (1995).
14. D. V. Messadi, "Diagnostic aids for detection of oral precancerous conditions," *Int. J. Oral Sci.* **5**, 59–65 (2013).
15. M. DeCoro, P. Wilder-Smith, "Potential of optical coherence tomography for early diagnosis of oral malignancies," *Expert Review of Anticancer Therapy* **10**(3), 321–329 (2010).
16. T. J. Muldoon, D. Roblyer, M. D. Williams, V. M. Stepanek, R. Richards-Kortum, A. M. Gillenwater, "Noninvasive imaging of oral neoplasia with a high-resolution fiber-optic microendoscope," *Head Neck* **34**, 305–312 (2012).
17. A. Sharwani, W. Jerjes, V. Salih, B. Swinson, I. Bigio, M. El-Maaytah *et al.*, "Assessment of oral premalignancy using elastic scattering spectroscopy," *Oral Oncol.* **42**, 343–349 (2006).
18. P. Chaturvedi, S. K. Majumder, H. Krishna, S. Muttagi, P. K. Gupta, "Fluorescence spectroscopy for noninvasive early diagnosis of oral mucosal malignant and potentially malignant lesions," *J. Cancer Res. Therapeut.* **6**, 497 (2010).
19. R. Malini, K. Venkatakrishna, J. Kurien, K. M Pai, L. Rao, V. Kartha *et al.*, "Discrimination of normal, inflammatory, premalignant, and malignant oral tissue: A Raman spectroscopy study," *Biopolymers* **81**, 179–193 (2006).
20. S. Singh, A. Deshmukh, P. Chaturvedi *et al.*, "*In vivo* Raman spectroscopy for oral cancers diagnosis," *SPIE BiOS*, Vol. 8219, International Society for Optics and Photonics, 2012, p. 82190K-82190K-82196.
21. S. Singh, A. Deshmukh, P. Chaturvedi, C. M. Krishna, "*In vivo* Raman spectroscopic identification of premalignant lesions in oral buccal mucosa," *J. Biomed. Opt.* **17**, 1050021–1050029 (2012).
22. S. N. Shaiju, S. Ariya, R. Asish, P. S. Haris, B. Anita, G. A. Kumar *et al.*, "Habits with killer instincts: *In vivo* analysis on the severity of oral mucosal alterations using autofluorescence spectroscopy," *J. Biomed. Opt.* **16**, 087006-087006-13 (2011).
23. A. Gillenwater, R. Jacob, R. Ganeshappa, B. Kemp, A. K. El-Naggar, J. L. Palmer *et al.*, "Noninvasive diagnosis of oral neoplasia based on fluorescence spectroscopy and native tissue autofluorescence," *Arch. Otolaryngol.–Head Neck Surg.* **124**, 1251–1258 (1998).
24. C.-K. Lee, T.-T. Chi, C.-T. Wu, M.-T. Tsai, C.-P. Chiang, C.-C. C. Yang, "Diagnosis of oral precancer with optical coherence tomography," *Biomed. Opt. Express* **3**, 1632–1646 (2012).
25. S. Singh, A. Sahu, A. Deshmukh, P. Chaturvedi, C. M. Krishna, "*In vivo* Raman spectroscopy of oral buccal mucosa: A study on malignancy associated

J. Innov. Opt. Health Sci. Downloaded from www.worldscientific.com by 189.219.167.56 on 06/22/16. For personal use only.

changes (MAC)/cancer field effects (CFE)," *Analyst* **138**, 4175–4182 (2013).
26. A. Sahu, A. Deshmukh, A. Ghanate, S. Singh, P. Chaturvedi, C. M. Krishna, "Raman spectroscopy of oral buccal mucosa: A study on age-related physiological changes and tobacco-related pathological changes," *Technol. Cancer Res. Treat.* **11**, 529–541 (2012).
27. E. M. Barroso, R. W. H. Smits, T. Bakker Schut, I. ten Hove, J. A. Hardillo, E. B. Wolvius *et al.*, "Discrimination between oral cancer and healthy tissue based on water content determined by Raman spectroscopy," *Anal. Chem.* **87**, 2419–2426 (2015).
28. F. L. Cals, T. C. B. Schut, J. A. Hardillo, R. J. B. de Jong, S. Koljenović, G. J. Puppels, "Investigation of the potential of Raman spectroscopy for oral cancer detection in surgical margins," *Lab. Invest.* **95**(10), 1186–1196 (2015).
29. R. Z. German, J. B. Palmer, "Anatomy and development of oral cavity and pharynx," *GI Motility Online* (2006).
30. T. A. Winning, G. C. Townsend, "Oral mucosal embryology and histology," *Clin. Dermatol.* **18**, 499–511 (2000).
31. D. C. de Veld, M. Skurichina, M. J. Witjes, R. P. Duin, D. J. Sterenborg, W. M. Star *et al.*, "Autofluorescence characteristics of healthy oral mucosa at different anatomical sites," *Lasers Surg. Med.* **32**, 367–376 (2003).
32. S. McGee, V. Mardirossian, A. Elackattu, J. Mirkovic, R. Pistey, G. Gallagher *et al.*, "Anatomy-based algorithms for detecting oral cancer using reflectance and fluorescence spectroscopy," *Ann. Otol. Rhinol. Laryngol.* **119**, 817–826 (2010).
33. S. McGee, J. Mirkovic, V. Mardirossian, A. Elackattu, C.-C. Yu, S. Kabani *et al.*, "Model-based spectroscopic analysis of the oral cavity: Impact of anatomy," *J. Biomed. Opt.* **13**, 064034-064034-15 (2008).
34. K. Guze, M. Short, S. Sonis, N. Karimbux, J. Chan, H. Zeng, "Parameters defining the potential applicability of Raman spectroscopy as a diagnostic tool for oral disease," *J. Biomed. Opt.* **14**, 014016-014016-9 (2009).
35. M. S. Bergholt, W. Zheng, Z. Huang, "Characterizing variability in *in vivo* Raman spectroscopic properties of different anatomical sites of normal tissue in the oral cavity," *J. Raman Spectrosc.* **43**, 255–262 (2012).
36. H. Krishna, S. K. Majumder, P. Chaturvedi, P. K. Gupta, "Anatomical variability of *in vivo* Raman spectra of normal oral cavity and its effect on oral tissue classification," *Biomed. Spectrosc. Imag.* **2**, 199–217 (2013).
37. H. Krishna, S. K. Majumder, P. Chaturvedi, M. Sidramesh, P. K. Gupta, "*In vivo* Raman spectroscopy for detection of oral neoplasia: A pilot clinical study," *J. Biophoton.* **7**, 690–702 (2014).
38. A. Nijssen, K. Maquelin, L. F. Santos, P. J. Caspers, T. C. B. Schut, J. C. den Hollander *et al.*, "Discriminating basal cell carcinoma from perilesional skin using high wave-number Raman spectroscopy," *J. Biomed. Opt.* **12**, 034004-034004-7 (2007).
39. S. Koljenović, T. C. B. Schut, J. M. Kros, H. J. van den Berge, G. J. Puppels, "Discriminating vital tumor from necrotic tissue in human glioblastoma tissue samples by Raman spectroscopy," *Lab. Invest.* **82**, 1265–1277 (2002).
40. P. Crow, B. Barrass, C. Kendall, M. Hart-Prieto, M. Wright, R. Persad *et al.*, "The use of Raman spectroscopy to differentiate between different prostatic adenocarcinoma cell lines," *Br. J. Cancer* **92**, 2166–2170 (2005).
41. T. Harvey, E. Gazi, A. Henderson, R. Snook, N. Clarke, M. Brown *et al.*, "Factors influencing the discrimination and classification of prostate cancer cell lines by FTIR microspectroscopy," *Analyst* **134**, 1083–1091 (2009).
42. T. C. Bakker Schut, R. Wolthuis, P. J. Caspers, G. J. Puppels, "Real-time tissue characterization on the basis of *in vivo* Raman spectra," *J. Raman Spectrosc.* **33**, 580–585 (2002).
43. J. G. Kelly, J. Trevisan, A. D. Scott, P. L. Carmichael, H. M. Pollock, P. L. Martin-Hirsch *et al.*, "Biospectroscopy to metabolically profile biomolecular structure: A multistage approach linking computational analysis with biomarkers," *J. Proteome Res.* **10**, 1437–1448 (2011).
44. A. Ghanate, S. Kothiwale, S. Singh, D. Bertrand, C. M. Krishna, "Comparative evaluation of spectroscopic models using different multivariate statistical tools in a multicancer scenario," *J. Biomed. Opt.* **16**, 025003-025003-9 (2011).
45. F. S. Parker, *Applications of Infrared, Raman, and Resonance Raman Spectroscopy in Biochemistry*, Springer Science & Business Media, New York and London (1983).
46. Z. Movasaghi, S. Rehman, I. U. Rehman, "Raman spectroscopy of biological tissues," *Appl. Spectrosc. Rev.* **42**, 493–541 (2007).
47. K. Guze, H. C. Pawluk, M. Short, H. Zeng, J. Lorch, C. Norris *et al.*, "Pilot study: Raman spectroscopy in differentiating premalignant and malignant oral lesions from normal mucosa and benign lesions in humans," *Head Neck* **37**, 511–517 (2015).
48. K. M. Baldwin, *Wheater's Review of Histology and Basic Pathology*, Churchill Livingstone (2010).

J. Innov. Opt. Health Sci. Downloaded from www.worldscientific.com by 189.219.167.56 on 06/22/16. For personal use only.

49. G. Kumar, *Orban's Oral Histology & Embryology*, Elsevier Health Sciences, New York (2014).
50. A. Nanci, *Ten Cate's Oral Histology-Pageburst on VitalSource: Development, Structure, and Function*, Elsevier Health Sciences, New York (2007).
51. C. Squier, M. Finkelstein, "Oral mucosa," *Ten Cate's Oral Histology: Development, Structure, and Function*, 6th Edition. Mosby, St. Louis (2003), pp. 369–372.

J. Innov. Opt. Health Sci. Downloaded from www.worldscientific.com by 189.219.167.56 on 06/22/16. For personal use only.
