# Supplementary material for: The accuracy of Fiber-Optic Raman Spectroscopy in the detection and diagnosis of head and neck neoplasm in vivo: a systematic review and meta-analysis
Source: PeerJ. 2023 Dec 11;11:e16536. doi: 10.7717/peerj.16536 (PMC10720414; doi:10.7717/peerj.16536)
Supplement: Data S1 [file peerj-11-16536-s001.zip › article/singh2013.pdf]

Analyst

RSC Publishing

PAPER

View Article Online
View Journal | View Issue

Published on 25 January 2013. Downloaded by Yale University Library on 26/08/2013 01:56:54.

Cite this: *Analyst*, 2013, **138**, 4175

In vivo Raman spectroscopy of oral buccal mucosa: a study on malignancy associated changes (MAC)/cancer field effects (CFE)

S. P. Singh,[a] Aditi Sahu,[a] Atul Deshmukh,[a] Pankaj Chaturvedi[b] and C. Murali Krishna*[a]

Occurrence of metachronous and synchronous secondary tumors in oral cavities has been associated with poor prognosis and decreased 5-year disease-free survival rates. The origin of secondary tumors in the oral cavity has been primarily attributed to cancer field effects (CFE) or malignancy-associated changes (MAC) in uninvolved areas. Classification of normal, cancerous and pre-cancerous oral lesions by *in vivo* Raman spectroscopy (RS) has already been demonstrated. In the present study, MAC/CFE in oral buccal mucosa were explored. *In vivo* Raman spectra from 84 subjects (722 spectra) under five categories – cancer and contralateral normal (opposite side of tumor), healthy controls (no tobacco habit, no cancer), habitués healthy controls (tobacco habit, no cancer) and non-habitués contralateral normal (no tobacco habit with cancer) were acquired. Mean and difference spectra suggest that loss of lipids and additional features representing proteins and DNA are characteristics of all pathological conditions, with respect to healthy controls. Spectral data were analyzed by PC-LDA followed by leave-one-out cross-validation. Results suggest that Raman characteristics of mucosa of healthy controls are exclusive, while those of habitués healthy controls are similar to those of contralateral normal mucosa. It was observed that the cluster of non-habitués contralateral normal mucosa is different from habitués healthy controls, suggesting that malignancy associated changes can be identified and also indicating that transformation of uninvolved oral mucosa due to tobacco habit or malignancy is different. The findings of the study demonstrate the potential of RS in identifying early transformation changes in oral mucosa and the efficacy of this approach in oral cancer applications.

Received 27th November 2012
Accepted 24th January 2013

DOI: 10.1039/c3an36761d

www.rsc.org/analyst

Introduction

Oral cancer, the sixth most common cancer, has 5-year disease-free survival rates of ~50% in developed and ~30% in developing countries.[1] One-fifth of the world's oral cancer subjects are from India and other South Asian countries.[2] Tobacco habits, including use of smokeless tobacco, are well known causes of oral cancer.[3–5] Oral carcinogenesis is a highly complex multifocal process that is believed to arise from a series of stepwise genetic changes induced by carcinogens, leading to clinical and microscopic changes summating to form an invasive neoplasm.[6,7] Appearance of a clinically visibly pre-cancerous lesion or condition in oral cancers is often preceded by micro-architectural changes in the oral cavity. Occurrence of these clinically or histologically unrecognizable subtle perturbations are attributed to 'malignancy-associated-changes (MACs) or cancer-field-effects (CFEs)', terms often used interchangeably.[8–11] The origin of these effects can be either due to diffusion of tumor cells in surrounding normal areas or generalized exposure of mouth cavity to carcinogens.[7] Slaughter *et al.* in 1953 have shown that these effects are associated with independent transformation leading to development of second primary tumors (SPTs) in the oral cavity.[12] Studies have suggested that the onset of SPTs decreases the 5-year survival rates by 18–30% as compared to those with only a single tumor.[13–18] Subjects suffering from SPT pose serious challenges in clinical evaluation, planning of treatment regimen and post-treatment quality of patient's life in terms of associated morbidity. Most often such cases are considered as inoperable and subjected to palliative therapy. Hence early detection of potentially malignant patches remains the best option. However, clinical evaluation often fails to detect these changes and as a result preventive onco-surgery procedures for removal of such patches are not initiated.[19,20] Identification of CFEs or MACs can serve as a novel screening tool which can reduce the morbidity and mortality associated with multiple potentially malignant transforming fields.

The efficacy of optical spectroscopic techniques such as fluorescence, Raman and Fourier-Transform Infrared (FTIR) spectroscopy in classifying normal and pathological conditions

[a]*Chilakapati lab, ACTREC, Tata Memorial Center, Kharghar, Navi Mumbai 410210, India. E-mail: mchilakapati@actrec.gov.in; pittu1043@gmail.com; Fax: +91-22-2740 5095; Tel: +91-22-2740 5039/5544*

[b]*Department of Surgical Oncology, Tata Memorial Hospital, Mumbai, 400012, India*

This journal is © The Royal Society of Chemistry 2013

Published on 25 January 2013. Downloaded by Yale University Library on 26/08/2013 01:56:54.

has already been demonstrated.[21–23] Reflectance and fluorescence spectroscopic studies have demonstrated the feasibility of differential diagnosis of malignant and potentially malignant lesions, including oral sub-mucous fibrosis in oral cancer.[24,25] Raman spectroscopic methods, based on inelastic scattering of light, with attributes such as negligible water interference, sharp spectral signatures and abundance of Raman active molecules in biological samples, are ideal for *in vivo* applications. Our recent studies have shown that healthy, malignant and premalignant lesions can be classified by *in vivo* Raman spectroscopy (RS).[26–28]

As mentioned earlier, oral malignancy is preceded by early transformation such as CFEs or MACs, and identifying such changes is the aim of optical spectroscopic methods. Backman *et al.* have shown the feasibility of identifying field changes in colon cancer using elastic light scattering spectroscopy[8–10] A few *ex vivo* Raman spectroscopic studies using 'culture raft models' in skin and cervical cancer have demonstrated the feasibility of identifying MACs or CFEs.[11,29] In the present study, we have explored the feasibility of *in vivo* RS in detecting early changes that are indicative of neoplastic transformation. We have analyzed *in vivo* spectra acquired from tumor adjacent mucosa (TAM) of subjects with and without tobacco associated cancerous lesion and from age-matched healthy controls with and without tobacco habits. Findings are discussed in the manuscript.

Materials and methods

Sample details

Eighty-four (84) subjects approved by the Institutional Ethics Committee (Tata Memorial Hospital-Institutional Review Board) were recruited for this study, after obtaining informed written consent. Information about tobacco usage, age, sex and tumor grade of all subjects was obtained from the Electronic Medical Record (EMR) system of Tata Memorial Hospital, Mumbai, India. Forty (40) subjects with tobacco associated pathologically verified squamous cell carcinoma (SCC) lesions of buccal mucosa were recruited. From this group, 170 spectra of contralateral normal (opposite side of tumor), referred to as contralateral and 192 spectra from cancerous lesion (tumor) were recorded. Fifteen (15) each of age-matched healthy controls with long-term tobacco habit (habitués healthy controls) and without any history of tobacco use (healthy controls) were also recruited. In this case, 150 spectra were recorded from each group. The screening criterion of tobacco habit for more than 10 years was applied for the recruitment of habitués healthy subjects. The average time of tobacco habit of these subjects was ~14.5 years. Sixty (60) spectra from the contralateral normal side of 14 subjects with buccal mucosa SCC lesion and without any history of tobacco use were also recorded (non-habitués contralateral). On an average ~9 to 10 spectra were acquired from each individual. For cancer patients, 5 spectra were recorded from the contralateral normal and tumor site, respectively to obtain a total of 10 spectra. For healthy tobacco and non-tobacco users, 10 spectra were recorded from the left and right buccal mucosa. In order to ensure reproducibility, spectra were recorded as per teeth positions at different points on buccal mucosa. Buccal surfaces opposing teeth were considered as the reference point. Spectra were recorded from opposing buccal surfaces of canine, first premolar, second premolar, first molar and second molar on both sides. To avoid any differences because of the mouth environment, subjects were required to wash their mouth with distilled water before spectral acquisitions. Median age of tumor, habitués healthy control; non-habitués contralateral and healthy control subjects were 44, 50, 45 and 50 years, respectively. Subject accrual is summarized in Table 1.

Raman spectroscopy

Spectra were recorded with an HE-785 commercial Raman spectrometer (LabRam, Jobin-Yvon-Horiba, France). Briefly, this system consists of a diode laser (Process Instruments) of 785 nm wavelength as the excitation source, a high efficiency (HE) spectrograph with fixed 950 gr mm^{-1} grating coupled with a CCD (Synapse). The instrument has no movable parts and the spectral resolution as specified by the manufacturer is ~4 cm^{-1}. The commercially available In Photonics (Inc, Downy St. USA) probe consisting of a 105 μm excitation fiber and a 200 μm collection fiber (NA-0.40) was used to couple the excitation source and the detection system. The estimated spot size and depth of the field as per the manufacturer's specifications are 105 μm and 1 mm, respectively. The working distance of the probe is 5 mm and therefore, a detachable spacer of length 5 mm was attached at the tip of the probe to maintain focus during all measurements. Prior to each measurement, these spacers were disinfected by CIDEX (Johnson and Johnson, Mumbai, India) solution to avoid inter-subject contamination. Spectral acquisition parameters were: λ_{ex} – 785 nm, laser power – 80 mW, spectra were integrated for 3 seconds and averaged over 3 accumulations.

Table 1 Subject accrual details

Category	Site of spectra recording	Age-range (median age)	Total no. of spectra/subjects (sex)	Tumor	Tobacco habits
Healthy control	Buccal mucosa	(40–59 years) 50 years	150/15 (13 M, 2 F)	No	No
Contralateral normal	Buccal mucosa	(24–67 years) 44 years	170/40 (40 M)	Yes	Yes
Non-habitués contralateral	Buccal mucosa	(26–81 years) 45 years	60/14 (8 M, 6 F)	Yes	No
Habitués healthy controls	Buccal mucosa	(25–60 years) 50 years	150/15 (14 M, 1 F)	No	Yes
Tumor	Buccal mucosa	(24–67 years) 44 years	192/40 (40 M)	Yes	Yes

This journal is © The Royal Society of Chemistry 2013

Published on 25 January 2013. Downloaded by Yale University Library on 26/08/2013 01:56:54.

Data analysis

In vivo Raman spectra were corrected for CCD response with a NIST certified SRM 2241 material followed by subtraction of background signals of optical elements. To remove interference of the slow moving background, first derivatives of spectra (Savitzky-Golay method and window size 3) were computed. Our previous studies have demonstrated the utility of the 1200–1800 cm^{-1} region in classifying normal, malignant and premalignant conditions of oral cancers, the same region was therefore employed in this study.[26–28] Another advantage of using this region is minimal interference of fiber Raman signals.† First derivative and vector normalized spectra were subjected to multivariate Principal Component-Linear Discriminant Analysis (PC-LDA).[26–28] Principal component analysis (PCA) is routinely used for data compression and visualization. It describes data variance by identifying a new set of orthogonal features, called principal components (PCs) or factors. For visual discrimination, we project each of the spectra in the newly formed co-ordinate space of selected PCs. While PCA aims to identify features that represent variance among the complete data, LDA provides data classification based on an optimized criterion which is aimed for better class separability. In LDA, the classification criterion is identified using the scatter measure of within class and between class variance. LDA can be used together with PCA to increase the efficiency of classification. For this, PCA scores obtained using a set of significant PCs with maximum variance amongst data are used as input data for LDA based classification. The advantage of doing this is to filter out noise and employ optimum variables for classification. In order to avoid over-fitting of the data, as a thumb rule, the total number of factors selected for analysis were less than half the number of the spectra in the smallest group.[26–28,30,31] LDA models were validated by leave-one-spectrum-out cross-validation (LOOCV). Algorithms for these analyses were implemented in MATLAB (Mathworks Inc.) based in-house software.[32]

Average spectra were computed from the background subtracted spectra (without derivatization) for each class by averaging *Y*-axis variations keeping the *X*-axis constant. The baseline corrections were performed by fitting a fifth order polynomial function. These spectra were used as a representative for spectral comparisons as well as to compute difference spectra.

† Principal components or factors are calculated by identifying eigenvectors for the covariance matrix of mean-centered data. Because of their orthogonal characteristics, first few PCs are often sufficient to represent maximum data variance. LDA is a method of choice when input data have higher within class variance that could lead to the development of PCs which are inappropriate for visual discrimination. LDA transformations are identified as an eigenvector matrix of this classification criterion. With the help of this LDA transform matrix, any test spectra can be classified into a class by iteratively calculating the Euclidean/RMS or Mahalanobis distance of transformed test spectra and the mean of transformed input dataset. In this study we have employed Mahalanobis distance for class prediction, since it handles nonlinearity well (S. Balakrishnama, A. Ganapathiraju, "Linear Discriminant Analysis – A Brief Tutorial", Institute for Signal and Information Processing, Mississippi State university, 1998). The results of PC-LDA can be depicted in the form of a confusion matrix, where all diagonal elements are true positive predictions and ex-diagonal elements are false positive predictions. The confusion matrix is generated to understand separation between the groups obtained by taking into account the contribution of all factors selected for analysis. These results can also be depicted in the form of scatter plots, generated by plotting combinations of scores of factors. Plotting different combinations of factor scores gives visual understanding of the classification pattern in the data.

Results and discussion

Average spectra of (a) healthy control, (b) contralateral normal, (c) non-habitués contralateral, (d) habitués healthy controls and (e) tumor were computed and are shown in Fig. 1. Spectral features of healthy controls are dominated by the strong δCH_2 band, two sharp features in the amide III region, the sharp peak around amide I and the ester band around 1744 cm^{-1} suggesting a high lipid content. Features in the amide III region, the broad and shifted δCH_2 band and broad amide I in the mean tumor spectrum indicate protein dominance. In healthy conditions, epithelium and lamina propria contain reticulin and collagen fibers while submucosa is rich in adipose tissue. In the case of tumors or other pathological conditions there is loss in the architectural arrangement of different layers indicating loss of lipid features as contents of different layers are mixed.[33–35] In addition to this, cells of pathological conditions have large amounts of surface and receptor proteins, enzymes, antigens, and antibodies which may give rise to a protein-dominated spectrum. These findings further corroborate earlier reports of *ex vivo* and *in vivo* RS studies.[26–28,33–38] Spectra of contralateral normal and habitués healthy controls are also dominated by lipid bands but exhibit minor differences, with respect to healthy controls, such as minor shift in amide III and δCH_2 band as well as broadening of the amide I region which suggests changes in protein secondary structures. Mean spectra of non-habitués contralateral subjects show a similar spectral pattern to that of healthy controls with minor differences like sharp amide I and additional bands in the ester region.

In order to bring out spectral differences between different groups, difference spectra were computed and are shown in Fig. 2A–D. Subtraction of mean spectra is one of the conventional ways of looking at spectral differences which can provide differences over the selected spectral range and understanding of the moieties that are modified. Difference spectra of pathological conditions *i.e.* tumor (i), contralateral normal (ii), non-habitués contralateral normal (iii) and habitués healthy controls (iv) were computed by subtracting them from healthy controls. As can be seen from Fig. 2A(i–iv), all positive peaks correspond to healthy controls and negative bands are from various pathological conditions. The difference tumor spectrum (Fig. 2A(i)) exhibits loss of lipids (1440, 1300, 1743 cm^{-1}) and the presence of DNA (1340, 1480 cm^{-1}) and hemoglobin ($\sim$1560 cm^{-1}), which could be attributed to the increase in number of dividing nuclei and angiogenesis, respectively, which are known hallmarks of tumorigenesis. Negative bands suggesting the presence of DNA (1340, 1480 cm^{-1}) and loss of lipids (1300, 1440, 1743 cm^{-1}) were also seen in the difference spectrum of other pathological conditions (Fig. 2A(ii–iv)). Long term tobacco exposure is known to cause increase in proliferation of epithelial cells in the upper aero-digestive tract of

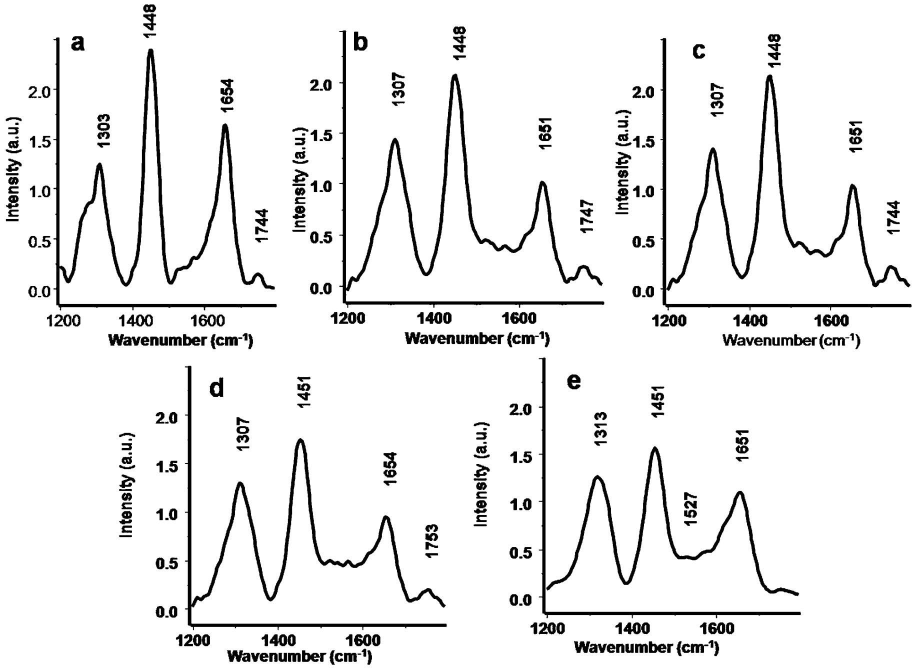

Fig. 1 Mean spectra from healthy control (a), contralateral normal (b), non-habitués contralateral (c), habitués healthy controls (d), and tumor (e).

tobacco users and can be considered as the preliminary event for genetic changes culminating in the development of oral SCC.[39] To understand the influence of long-term tobacco exposure, spectra of tumor, contralateral normal and non-habitués contralateral were subtracted from the mean habitués healthy controls spectrum [Fig. 2B(i–iii)]. In this case positive

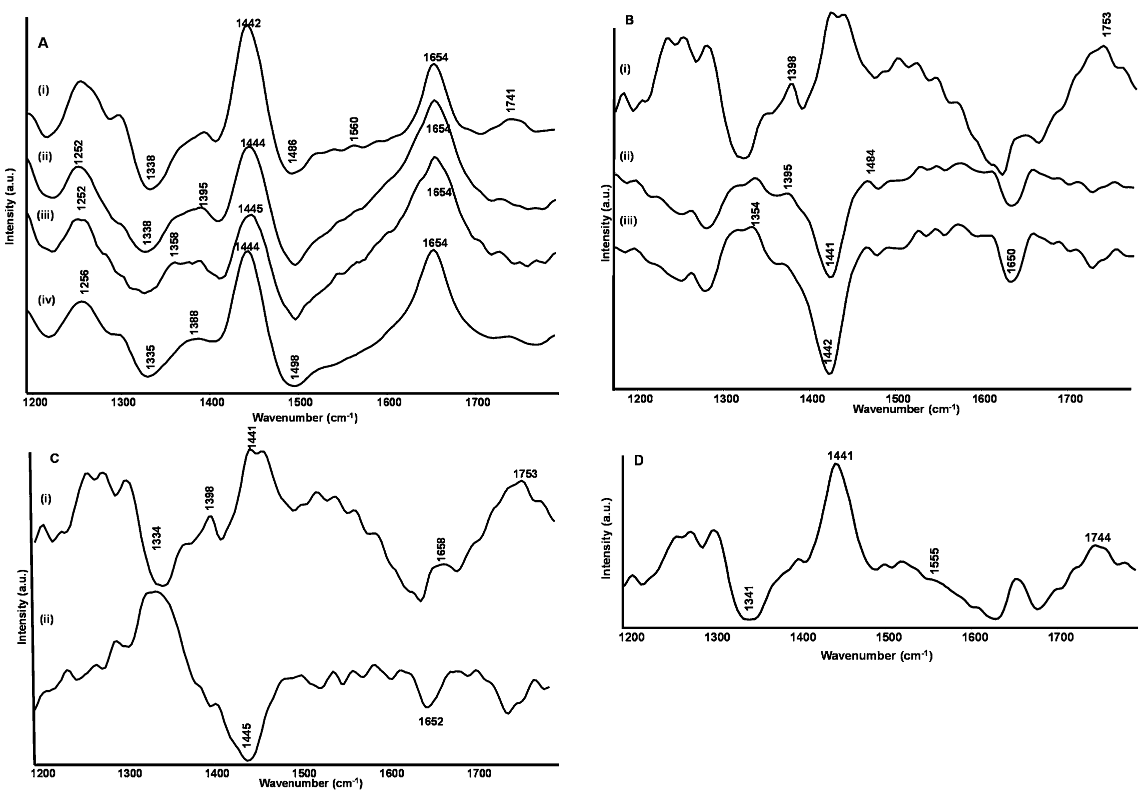

Fig. 2 Comparison of difference spectra across different groups. A(i) healthy control – tumor; A(ii) healthy control – contralateral normal; A(iii) healthy control – non-habitués contralateral normal; A(iv) healthy control – habitués healthy control; B(i) habitués healthy control – tumor; B(ii) habitués healthy control – contralateral normal; B(iii) habitués healthy control – non-habitués contralateral normal, C(i) contralateral normal – tumor; C(ii) contralateral normal – non-habitués contralateral normal, (D) non-habitués contralateral normal – tumor.

This journal is © The Royal Society of Chemistry 2013

Published on 25 January 2013. Downloaded by Yale University Library on 26/08/2013 01:56:54.

Published on 25 January 2013. Downloaded by Yale University Library on 26/08/2013 01:56:54.

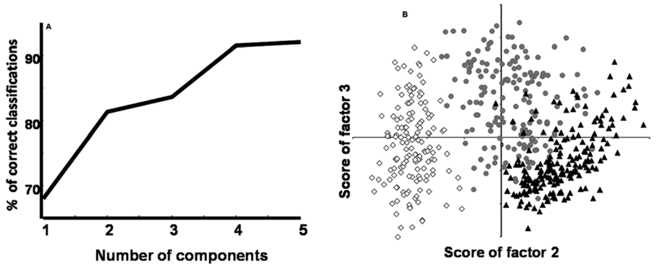

Fig. 3 (A) Cumulative percent variance contribution of PCA factors used for LDA. (B) LDA results of comparison of healthy control (◇), contralateral normal (●) and tumor (△).

bands correspond to habitués healthy controls and negative bands to pathological conditions. Once again, tumor spectra show loss of lipids (1300, 1440, 1743 cm^{-1}), predominant protein features (broad 1660 cm^{-1}) and prominent DNA bands (1340, 1480 cm^{-1}) (Fig. 2B(i)). Positive DNA bands along with loss of lipid features (1300, 1440, 1650, 1743 cm^{-1}) were observed in the difference spectrum of non-habitués contralateral, which could be attributed to tobacco induced hypercellularity (Fig. 2B(iii)). These features were further corroborated by computing difference spectra of contralateral normal as shown in Fig. 2B(ii). No major differences except the minor shift in the δCH_2 band were observed. The difference spectrum of tumor and non-habitués contralateral were computed by subtracting contralateral normal spectra [Fig. 2C(i–ii)]. Positive peaks correspond to contralateral normal and negative peaks to tumor and non-habitués contralateral normal. Similar to earlier observations loss of lipids and predominant protein features suggested by negative amide I (~1658 cm^{-1}) and amide III were seen in the difference spectrum of tumors [Fig. 2C(i)]. Difference spectra of non-habitués contralateral normal suggest differences in the lipid content (negative bands 1440, 1743 cm^{-1}); positive DNA bands (1340, 1480 cm^{-1}) were also observed which could be attributed to tobacco induced hypercellularity [Fig. 2C(ii)]. The difference tumor spectrum was computed by subtracting the mean

Table 2 Summary of classification between (A) healthy control, contralateral normal and tumor; (B) healthy control, contralateral normal, habitués healthy controls and tumor; (C) healthy control, contralateral normal, habitués healthy control and non-habitués contralateral normal subjects (diagonal elements are true positive predictions and ex-diagonal elements are false positive predictions)

A	Healthy control	Contralateral normal	Tumor	% Class eff.
Healthy control	148/150	2	0	98.66
Contralateral normal	4	143/170	23	84.11
Tumor	0	12	180/192	93.75
**Leave-one-out cross validation**				
Healthy control	148/150	2	0	98.66
Contralateral normal	4	143/170	23	84.11
Tumor	0	14	178/192	92.70

B	Healthy control	Contralateral normal	Habitués healthy controls	Tumor	% Class eff.
Healthy control	146/150	3	1	0	97.33
Contralateral normal	0	119/170	30	21	70
Habitués healthy controls	5	41	104/150	0	69.33
Tumor	0	7	17	168/192	87.5
**Leave-one-out cross validation**					
Healthy control	144/150	4	2	0	96
Contralateral normal	0	118/170	31	21	69.41
Habitués healthy controls	5	42	103/150	0	68.66
Tumor	0	7	19	166/192	86.45

C	Healthy control	Contralateral normal	Habitués healthy controls	Nonhabitués contralateral normal	% Class eff.
Healthy control	148/150	0	2	0	98.66
Contralateral normal	1	131/170	38	0	77.05
Habitués healthy controls	4	22	123/150	1	82
Nonhabitués contralateral normal	0	2	4	54/60	90
**Leave-one-out cross validation**					
Healthy control	148/150	0	2	0	98.66
Contralateral normal	1	128/170	41	0	75.29
Habitués healthy controls	4	30	115/150	1	76.66
Nonhabitués contralateral normal	0	5	3	52/60	86.66

View Article Online

Published on 25 January 2013. Downloaded by Yale University Library on 26/08/2013 01:56:54.

spectrum of non-habitués contralateral normal and spectral features corroborate earlier observations, *i.e.* loss of lipids (1300, 1440, 1743 cm^{-1}) and presence of DNA bands (1340, 1480 cm^{-1}), as shown in Fig. 2D. Overall, major spectral variability was observed in DNA (1340, 1480 cm^{-1}), proteins (amide I, III, δCH_2) and lipids (δCH_2 deformation, twisting and ester). Loss of lipids seems to be a common feature for most of the pathological conditions in comparison to healthy controls. The spectral assignments were based as reported in the literature.[34,38,40,41]

Pattern recognition tool PC-LDA followed by LOOCV was used to identify discrimination patterns among different groups. In the first step, the efficacy of RS in correctly identifying negative controls *i.e.* healthy controls was evaluated. Spectral data of healthy controls, contralateral normal and tumor sites were subjected to PC-LDA using 5 factors which accounted for ~92% variance. The cluster belonging to healthy control is exclusive, while contralateral normal and tumor show minimal overlap (Fig. 3). In LOOCV (Table 2A) only 2 out of 150 (1%) spectra from healthy control were wrongly classified as contralateral normal. 14 tumor spectra were misclassified as contralateral normal, while 23 contralateral normal spectra were wrongly classified as tumor. Corroborating our earlier studies,[26–28] minor misclassifications of tumor spectra as contralateral normal and *vice versa* were observed, which can be attributed to tumor heterogeneity. As spectra in the present study were recorded at different points, the possibility of acquiring spectra from analogues normal or dysplastic site in the tumor region cannot be ruled out. As already mentioned, transformation of buccal mucosa often precedes premalignant lesions eventually leading to malignancy. The non-overlap of healthy controls and overlap among contralateral and tumor could be attributed to the presence of transformation zones in visibly normal mucosa and heterogeneous nature of tumors.

In order to explore the feasibility of identifying changes due to tobacco exposure, spectral data of habitués healthy controls along with healthy controls, contralateral normal and tumor sites were analyzed by PC-LDA. In this case, 8 factors accounting for ~82% variance were used for analysis (Fig. 4). Once again, an exclusive cluster of healthy controls and two minimally overlapping clusters of contralateral normal and tumor sites of habitués oral cancer subjects were obtained (Fig. 4). Major overlap was observed between contralateral normal mucosa and habitués healthy controls. The findings of LOOCV, shown in Table 2B, indicate that only 6 spectra from healthy controls were misclassified (4 as contralateral normal and 2 as habitués healthy control) and 52 out of 170 spectra of contralateral normal were wrongly classified (21 as tumor and 31 as habitués healthy controls). For habitués healthy controls, 47 misclassifications (42 were contralateral and 5 with healthy controls) were noted. This could be attributed to tobacco induced CFE suggesting that early changes due to carcinogen exposure can be identified. In the case of tumor, 26 spectra (19 as habitués healthy and 7 as contralateral normal) were wrongly classified, which could be due to the presence of regions of inflammation as a result of tobacco exposure within tumor (tumor heterogeneity) and transformation zones in visibly normal mucosa.

Although tobacco is a known major etiological factor for oral cancers, there is a sizeable occurrence of oral cancers in subjects without tobacco habits. To study the transformation in uninvolved mucosa of such cases, analysis was carried out on non-habitués contralateral subjects along with habitués contralateral normal, habitués and non-habitués healthy controls by PC-LDA using 7 factors, which cover ~85% spectral variance (Fig. 5). Similar to earlier observations, an exclusive cluster of healthy controls and overlapping clusters of contralateral normal and habitués healthy controls were obtained. The contralateral normal side of non-habitués cancer subjects also yielded a separate cluster (Fig. 5). Fifty-two (52) out of 60 spectra of non-habitués contralateral normal were correctly classified by LOOCV (Table 2C). The minor misclassifications were with contralateral (5 spectra) and habitués healthy controls (3 spectra). Similar to analysis described above, overlap between contralateral normal and habitués healthy controls were observed. Of the total 42 misclassifications of contralateral normal, 41 were habitués healthy controls and 1 was healthy control. In the case of habitués healthy controls, of the 35 misclassifications, 30 were contralateral normal, 4 being

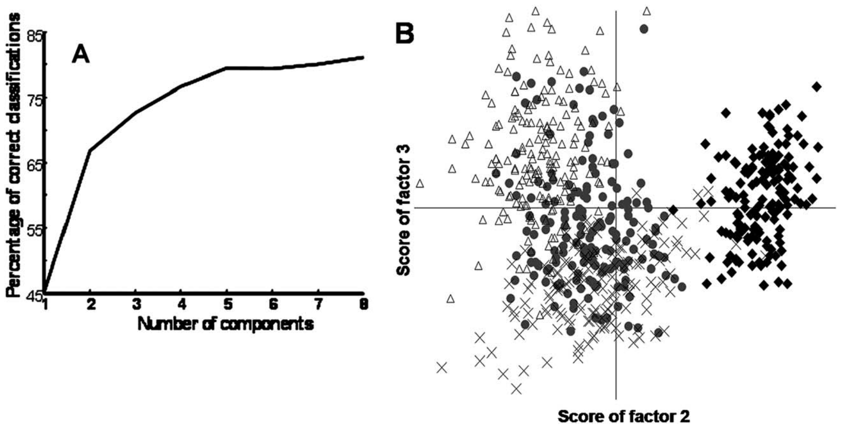

Fig. 4 (A) Cumulative percent variance contribution of PCA factors used for LDA. (B) LDA results of comparison of healthy control (◇), contralateral normal (●), habitués healthy control subjects (X) and tumor (△).

 This journal is © The Royal Society of Chemistry 2013

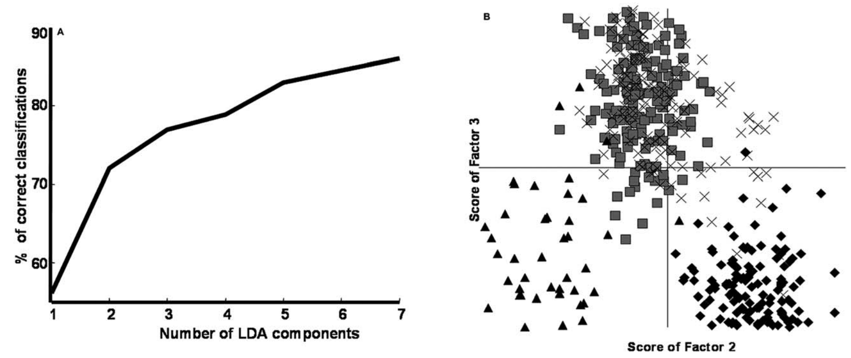

Fig. 5 (A) Cumulative percent variance contribution of PCA factors used for LDA. (B) LDA results of comparison of healthy control (◆), contralateral normal (□), non-habitués contralateral normal (▲) and habitués healthy control (X).

Published on 25 January 2013. Downloaded by Yale University Library on 26/08/2013 01:56:54.

healthy controls and 1 was non-habitués contralateral. In this case, both contralateral normal mucosa and non-habitués contralateral mucosa showed no overlap with healthy controls, suggesting the presence of CFE/MAC. No overlap between contralateral normal mucosa and non-habitués contralateral mucosa was also observed which suggests that early transformation changes in both groups may be different.

Conclusions

Oral cancer subjects with multiple primary tumors have poor prognosis and a lower disease free survival rate. Identification of uninvolved, transformation prone regions may allow estimation of risk before occurrence of a disease. Histopathological diagnoses, current gold standard, may not be an ideal tool to detect early changes such as CFE/MAC. Molecular diagnosis methods have been shown to be feasible for identifying such changes. In fact, the study by Brennan *et al.* has shown that out of 25 subjects having completely negative diagnosis by histopathology, 13 were found to be positive by molecular analysis, of which five have shown recurrence within 2 years.[42] In colon cancers, molecular markers like the colonocyte proliferation rate, apoptosis assays and others have provided evidence that transformation prone non-neoplastic rectal mucosa can be identified.[8] However, assaying these cellular and genetic markers may be imprecise, has inter-observer discrepancies, involves invasive procedures, is cumbersome and therefore impractical for clinical utilization or routine screening. Identification of cellular micro-architectural changes with an objective and sensitive technique may provide an insight into the genetic/environmental milieu during early carcinogenesis.[8–11,29] In oral cancer, screening of early lesions and identification of transformation prone areas in even chronic tobacco users can help in reducing the incidence as well as occurrence of multiple primary neoplasias. Since invasive screening methods like biopsy or blood collection are not practical, novel diagnostic methods which are preferably non-invasive and sensitive to minor biochemical variations are always desirable for screening of large population. Optical diagnostic methods are being explored as a novel, non-invasive method of diagnosis for cancers. The amenability of optical data to multivariate analysis tools provides objectivity to the method. The feasibility of identifying normal, cancerous and pre-cancerous lesion in the oral cavity with *in vivo* RS has already been demonstrated.[26–28] Therefore it is pertinent to explore this technique in identifying changes like CFE/MAC in uninvolved normal mucosa which have been implicated in the development of multiple primary tumors of the oral cavity.

The findings of the study suggest that subtle changes because of tobacco abuse/unknown etiological factors, which may be indicative of early neoplastic transformation in clinically normal appearing contralateral mucosa *i.e.* CFE/MAC, may be detected by Raman spectroscopy. The non-invasiveness and use of a harmless excitation wavelength impart this method several advantages, and thus prospectively RS has potential to become an ideal mass screening tool in public health programs.

Abbreviations

OSCC	Oral squamous cell carcinoma
FTIR	Fourier transformed infrared
RS	Raman spectroscopy
LDA	Linear discriminant analysis
LOO	Leave one out.

Acknowledgements

This work was carried out under project no: BT/PRI11282/MED/32/83/2008, Department of Biotechnology, Government of India. Authors would like to acknowledge all the subjects who participated in the study. Dr A. G. Ramchandani is also acknowledged for the editorial makeover of the manuscript.

References

1 D. M. Parkin, F. Bray, J. Fertay and P. Pisani, *Ca-Cancer J. Clin.*, 2005, **55**, 74–108.
2 D. M. Parkin, S. L. Whelan, J. Ferlay, L. Teppo and D. B. Thomas, *Cancer incidence in five continents Vol. VIII*, IARC Press, Lyon, 2002, pp. 232–249.
3 P. Boffetta, S. Hecht, N. Gray, P. Gupta and K. Straif, *Lancet Oncol.*, 2008, **9**, 667–675.

Published on 25 January 2013. Downloaded by Yale University Library on 26/08/2013 01:56:54.

4 J. H. Jeng, M. C. Chang and L. J. Hahn, *Oral Oncol.*, 2001, **37**, 477–492.
5 M. Hashibe, P. Brennan, S. Benhamou, X. Castellsague, C. Chen and M. P. Curado, *J. Natl. Cancer Inst.*, 2007, **99**, 777–789.
6 T. Tanaka, M. Tanaka and T. Tanaka, *Pathol. Res. Int.*, 2011, **2011**, 10.
7 M. G. C. T. V. Oijen and P. J. Slootweg, *Cancer Epidemiol., Biomarkers Prev.*, 2000, **9**, 249–256.
8 H. K. Roy, A. Gomes, V. Turzhitsky, M. J. Goldberg, J. Rogers, S. Ruderman, K. L. Young, A. Kromine, R. E. Brand, M. Jameel, P. Vakil, N. Hasabou and V. Backman, *Gastroenterology*, 2008, **135**, 1069–1078.
9 H. K. Roy, Y. L. Kim, R. K. Wali, Y. Liu, J. Koetsier, D. P. Kunte, M. J. Goldberg and V. Backman, *Cancer Epidemiol., Biomarkers Prev.*, 2005, **14**, 1639–1645.
10 H. K. Roy, Y. Liu, R. K. Wali, Y. L. Kim, A. K. Kromine, M. J. Goldberg and V. Backman, *Gastroenterology*, 2004, **126**, 1071–1081.
11 A. Robichaux Viehoever, D. Anderson, D. Jansen and A. Mahadevan-Jansen, *Photochem. Photobiol.*, 2003, **78**, 517–524.
12 D. P. Slaughter, H. W. Southwick and W. Smejkal, *Cancer*, 1953, **6**, 963–968.
13 J. A. Woolgar, S. Rogers, C. R. West, R. D. Errington, J. S. Brown and E. D. Vaughan, *Oral Oncol.*, 1999, **35**, 257–265.
14 F. Cianfrigkia, D. A. Di Gregorio and A. Manieri, *Oral Oncol.*, 1999, **35**, 157–163.
15 I. J. Dhooge, M. De Vos and P. B. Van Cauwenberge, *Laryngoscope*, 1998, **108**, 250–256.
16 A. H. Shikhani, G. M. Matanoski, M. M. Jones, H. K. Kashima and M. E. Johns, *Arch. Otolaryngol., Head Neck Surg.*, 1986, **112**, 1172–1179.
17 G. L. Day and W. J. Blot, *Cancer*, 1992, **70**, 14–19.
18 B. S. Tepperman and P. J. Fitzpatrick, *Lancet*, 1981, **12**, 547–549.
19 A. Nancy, *Tencate's Oral Histology: Development, Structure and Function*, Mosby, Inc., an affiliate of Elsevier Inc; 2006.
20 *Devita, Hellman & Rosenberg's Cancer: Principles & Practice of Oncology*, ed. V. T. Devita, T. S. Lawrence and S. A. Rosenberg, Lippincott Williams and Wilkins, Philadelphia, 8th edn, 2008.
21 D. C. G. De Veld, M. J. H. Witjes, H. J. C. M. Sterenborg and J. L. M. Roodenburg, *Oral Oncol.*, 2005, **41**, 117–31.
22 C. Kendall, M. Isabelle, F. Bazant-Hegemark, J. Hutchings, L. Orr, J. Babrah, R. Baker and N. Stone, *Analyst*, 2009, **134**, 1029–1045.
23 A. Nijssen, S. Koljenovic, T. C. B. Schut, P. J. Caspers and G. J. Pupples, *J. Biophoton*, 2009, **2**, 29–36.
24 S. Nazeer Shaiju, S. Ariya, R. Asish, H. P. Salim, B. Anita, G. Arun Kumar and R. S. Jayasree, *J. Biomed. Opt.*, 2011, **16**, 087006.
25 P. Chaturvedi, S. K. Majumder, H. Krishna, S. Muttagi and P. K. Gupta, *J. Cancer Res. Ther.*, 2011, **6**, 497–502.
26 S. P. Singh, A. Deshmukh, P. Chaturvedi and C. M. Krishna, *J. Cancer Res. Ther.*, 2012, **8**, S126–S132.
27 S. P. Singh, A. Deshmukh, P. Chaturvedi and C. M. Krishna, *Proc. SPIE*, 2012, **8219**, 82190K1–K6.
28 S. P. Singh, A. Deshmukh, P. Chaturvedi and C. M. Krishna, *J. Biomed. Opt.*, 2012, **17**, 105002.
29 C. A. Lieber, H. E. Nethercott and M. H. Kabeer, *Biomed. Opt. Express*, 2010, **1**, 975–982.
30 P. Crow, B. Barrass, C. Kendall, M. Hart-Prieto, M. Wright, R. Persad and N. Stone, *Br. J. Cancer*, 2005, **92**, 2166–2170.
31 T. J. Harvey, E. Gazi, A. Henderson, R. D. Snook, N. W. Clarke, M. Brown and P. Gardner, *Analyst*, 2009, **134**, 1083–1091.
32 A. D. Ghanate, S. Kothiwale, S. P. Singh, D. Bertrand and C. M. Krishna, *J. Biomed. Opt.*, 2011, **16**, 025003.
33 A. Deshmukh, S. P. Singh, P. Chaturvedi and C. M. Krishna, *J. Biomed. Opt.*, 2011, **16**, 127004.
34 K. Venkatakrishna, J. Kurien, K. M. Pai, C. M. Krishna, G. Ullas and V. B. Kartha, *Curr. Sci.*, 2001, **80**, 101–105.
35 R. Malini, K. Venkatakrishna, J. Kurien, K. M. Pai, L. Rao, V. B. Kartha and C. M. Krishna, *Biopolymers*, 2006, **81**, 179–193.
36 K. Guze, M. Short, S. Sonis, N. Karimbux, J. Chan and H. Zeng, *J. Biomed. Opt.*, 2009, **14**, 014016.
37 Z. Huang, S. K. Teh, W. Zheng, J. Mo, K. Lin, X. Shao, K. Y. Ho, M. Teh and K. G. Yeoh, *Opt. Lett.*, 2009, **34**, 758–760.
38 A. T. Harris, A. Rennie, H. Waqar-Uddin, S. R. Wheatley, S. K. Ghosh, D. P. Martin-Hirsch, S. E. Fisher, A. S. High, J. Kirkham and T. Upile, *Head Neck Oncol.*, 2010, **2**, 1–6.
39 D. M. Shin, N. Voravud, J. Y. Ro, J. S. Lee, W. K. Hong and W. N. Hittelman, *J. Natl. Cancer Inst.*, 1993, **12**, 971–978.
40 F. S. Parker, *Applications of Infrared, Raman and Resonance Raman Spectroscopy in Biochemistry*, Plenum Press, Newyork and London, 1983.
41 M. Zanyar, R. Shazza and I. U. Rehman, *Appl. Spectrosc. Rev.*, 2007, **42**, 493–541.
42 J. A. Brennan, L. Mao, R. H. Hruban, J. O. Boyle, Y. J. Eby, W. M. Koch, S. N. Goodman and D. Sidransky, *N. Engl. J. Med.*, 1995, **15**, 3–10.

This journal is © The Royal Society of Chemistry 2013
