# Supplementary material for: The accuracy of Fiber-Optic Raman Spectroscopy in the detection and diagnosis of head and neck neoplasm in vivo: a systematic review and meta-analysis
Source: PeerJ. 2023 Dec 11;11:e16536. doi: 10.7717/peerj.16536 (PMC10720414; doi:10.7717/peerj.16536)
Supplement: Data S1 [file peerj-11-16536-s001.zip › Table 2.docx]

Table 2. General information^10^ of the studies included in the article

| Year | Author | Country | Disease | Number of people | Mean age | Male: female | Site of disease | Sample type | Diagnostic algorithm | Spectrum range | TP | FN | TN | FP | Acquisition time |
| --- | --- | --- | --- | --- | --- | --- | --- | --- | --- | --- | --- | --- | --- | --- | --- |
| 2016 | Lin, K. a^11^ | Singapore | Laryngeal carcinoma | 60 | 51 | 47：13 | Larynx | In vivo | PLS-DA+LOOCV | FP+HW | 28 | 2 | 64 | 7 | ＜0.2 s |
| 2016 | Lin, K. b^11^ | Singapore | Laryngeal carcinoma | 60 | 51 | 47：13 | Larynx | In vivo | PLS-DA+LOOCV | FP | 26 | 4 | 61 | 10 | ＜0.2 s |
| 2016 | Lin, K. c^11^ | Singapore | Laryngeal carcinoma | 60 | 51 | 47：13 | Larynx | In vivo | PLS-DA+LOOCV | HW | 23 | 7 | 62 | 9 | ＜0.2 s |
| 2012 | Lin ,K.^15^ | Singapore | Laryngeal carcinoma | 39 | 60 | -* | Larynx | In vivo | PCA+LDA+LOOCV | HW | 65 | 7 | 20 | 2 | ＜1 s |
| 2018 | Lin, D.^19^ | China | Nasopharyngeal carcinoma | 60 | 53.8 | 39：21 | Nasopharynx | In vivo | PCA+LDA | FP | 126 | 15 | 131 | 11 | 1 s |
| 2019 | Zuvela, P. a^10^ | Singapore | Nasopharyngeal carcinoma | 62 | Male 53.8±16.9, female 46.4±11.3 | 43：19 | Nasopharynx | In vivo | GA-PLS-LDA, LOOCV | FP+HW | 28 | 2 | 83 | 0 | ＜0.5 s |
| 2019 | Zuvela, P. b^10^ | Singapore | Nasopharyngeal carcinoma | 62 | Male 53.8±16.9, female 46.4±11.3 | 43：19 | Nasopharynx | In vivo | GA-PLS-LDA, LOOCV | FP | 21 | 9 | 83 | 0 | ＜0.5 s |
| 2019 | Zuvela, P. c^10^ | Singapore | Nasopharyngeal carcinoma | 62 | Male 53.8±16.9, female 46.4±11.3 | 43：19 | Nasopharynx | In vivo | GA-PLS-LDA, LOOCV | HW | 24 | 6 | 83 | 0 | ＜0.5 s |
| 2017 | Lin, K. a^12^ | Singapore | Nasopharyngeal carcinoma | 95 | 52 | 68：27 | Nasopharynx | In vivo | PCA, LDA, LOOCV | FP+HW | 102 | 7 | 88 | 7 | ＜0.5 s |
| 2017 | Lin, K. b^12^ | Singapore | Nasopharyngeal carcinoma | 95 | 52 | 68：27 | Nasopharynx | In vivo | PCA, LDA, LOOCV | FP | 98 | 11 | 84 | 11 | ＜0.5 s |
| 2017 | Lin, K. c^12^ | Singapore | Nasopharyngeal carcinoma | 95 | 52 | 68：27 | Nasopharynx | In vivo | PCA, LDA, LOOCV | HW | 97 | 12 | 86 | 9 | ＜0.5 s |
| 2017 | Ming, L.C.^18^ | Singapore | Nasopharyngeal carcinoma | 79 | -* | 56：23 | Nasopharynx | In vivo | PLS | FP+HW | 20 | 2 | 40 | 2 | 0.1 s-0.5 s |
| 2013 | Singh, S.P.^16^ | India | Oral carcinoma | 84 | 46.3 | 75：9 | Buccal | in vivo | PC-LDA, PCA, LDA, LOOCV | FP | 166 | 26 | 449 | 21 | 3s |
| 2014 | Krishna, H.^14^ | India | Oral carcinoma | 199 | 41.2 | 6：1 | Oral | In vivo | SMLR, LOOCV | FP | 281 | 35 | 458 | 28 | 5s |
| 2016 | Sahu, A.^17^ | India | Oral carcinoma | 157 | 43 | 125:32 | Oral | In vivo | PC-LDA, LOOCV, LDA | FP | 174 | 77 | 1246 | 106 | 3s |
| 2017 | Malik, A.^13^ | India | Oral carcinoma | 42 | -* | -* | Buccal | In vivo | PC-LDA, LOOCV | FP | 233 | 12 | 317 | 41 | 3s |

(*:“-” in this table indicates that no relative data were found in the article. Partial Least Squares-Discrimination Analysis (PLS-DA), Leave-one-out cross-validation (LOOCV), Principal component analysis + Linear discriminant analysis (PCA+LDA), Genetic algorithm- Partial Least Squares- Linear discriminant analysis (GA-PLS-LDA) and Stepwise analysis of multiple linear regression (SMLR) in Table 2 refer to different diagnostic algorithms of Raman spectra. Data in articles can be used to construct a fourfold table including true positives (TPs), true negatives (TNs), false positives (FPs) and false negatives (FNs).)
