# Supplementary material for: The accuracy of Fiber-Optic Raman Spectroscopy in the detection and diagnosis of head and neck neoplasm in vivo: a systematic review and meta-analysis
Source: PeerJ. 2023 Dec 11;11:e16536. doi: 10.7717/peerj.16536 (PMC10720414; doi:10.7717/peerj.16536)
Supplement: Supplemental Information 2 [file peerj-11-16536-s002.doc]

Database: Embase <1974 to 2023 March 17>

Search Strategy:

--------------------------------------------------------------------------------

1 (head and neck neoplasms).mp. [mp=title, abstract, heading word, drug trade name, original title, device manufacturer, drug manufacturer, device trade name, keyword heading word, floating subheading word, candidate term word] (4215)

2 Facial Neoplasms.mp. or face tumor/ (3403)

3 Eyelid Neoplasms.mp. or eyelid tumor/ (3017)

4 Mouth Neoplasms.mp. or mouth tumor/ (15555)

5 Gingival Neoplasms.mp. or gingiva tumor/ (1961)

6 Leukoplakia, Oral.mp. or oral leukoplakia/ (347)

7 Leukoplakia, Hairy.mp. or hairy leukoplakia/ (260)

8 Lip Neoplasms.mp. or lip tumor/ (1656)

9 Palatal Neoplasms.mp. or jaw tumor/ (4051)

10 Salivary Gland Neoplasms.mp. or salivary gland tumor/ (8685)

11 Parotid Neoplasms.mp. or parotid gland tumor/ (6638)

12 Sublingual Gland Neoplasms.mp. or salivary gland tumor/ (7929)

13 Submandibular Gland Neoplasms.mp. or salivary gland tumor/ (7944)

14 Tongue Neoplasms.mp. or tongue tumor/ (4599)

15 Otorhinolaryngologic Neoplasms.mp. or "head and neck tumor"/ (17295)

16 Laryngeal Neoplasms.mp. or larynx tumor/ (10462)

17 Nose Neoplasms.mp. or nose tumor/ (5257)

18 Paranasal Sinus Neoplasms.mp. or paranasal sinus tumor/ (3678)

19 Maxillary Sinus Neoplasms.mp. or maxillary sinus tumor/ (122)

20 Pharyngeal Neoplasms.mp. or pharynx cancer/ (5206)

21 Hypopharyngeal Neoplasms.mp. or hypopharynx tumor/ (1475)

22 Nasopharyngeal Neoplasms.mp. or nasopharynx tumor/ (6000)

23 Nasopharyngeal Carcinoma.mp. or nasopharynx carcinoma/ (24623)

24 Oropharyngeal Neoplasms.mp. or oropharynx tumor/ (2569)

25 Tonsillar Neoplasms.mp. or tonsil tumor/ (954)

26 Parathyroid Neoplasms.mp. or parathyroid tumor/ (3062)

27 (Squamous Cell Carcinoma of Head and Neck).mp. [mp=title, abstract, heading word, drug trade name, original title, device manufacturer, drug manufacturer, device trade name, keyword heading word, floating subheading word, candidate term word] (955)

28 Thyroid Neoplasms.mp. or thyroid tumor/ (18693)

29 Thyroid Cancer, Papillary.mp. or thyroid papillary carcinoma/ (19069)

30 Thyroid Nodule.mp. or thyroid nodule/ (20027)

31 Tracheal Neoplasms.mp. or trachea tumor/ (2366)

32 (Neoplasms, Head and Neck).mp. [mp=title, abstract, heading word, drug trade name, original title, device manufacturer, drug manufacturer, device trade name, keyword heading word, floating subheading word, candidate term word] (18)

33 Head, Neck Neoplasms.mp. (25)

34 (Cancer of Head and Neck).mp. [mp=title, abstract, heading word, drug trade name, original title, device manufacturer, drug manufacturer, device trade name, keyword heading word, floating subheading word, candidate term word] (2062)

35 (Head and Neck Cancer).mp. [mp=title, abstract, heading word, drug trade name, original title, device manufacturer, drug manufacturer, device trade name, keyword heading word, floating subheading word, candidate term word] (74000)

36 (Cancer of the Head and Neck).mp. [mp=title, abstract, heading word, drug trade name, original title, device manufacturer, drug manufacturer, device trade name, keyword heading word, floating subheading word, candidate term word] (4669)

37 Head Neoplasms.mp. or head cancer/ (1889)

38 Neoplasms, Head.mp. (22)

39 Neck Neoplasms.mp. (4242)

40 Neoplasms, Neck.mp. (3)

41 Cancer of Head.mp. (2162)

42 Head Cancer.mp. or head cancer/ (2688)

43 Cancer of the Head.mp. (5132)

44 Cancer of Neck.mp. (230)

45 Neck Cancer.mp. or neck cancer/ (74619)

46 Cancer of the Neck.mp. (1302)

47 ((nasopharyn$ or oropharyn$ or laryn$ or glotti$ or tonsil$ or epiglotti$ or oral cavity or oral or tongue or gingiva$ or bucca$ or lip or palat$ or gum or mouth floor or floor of mouth or lingual or (head and neck) or HN) adj4 (cancer$ or tumor$ or tumor$ or neoplasm$ or carcinoma$ or squamous cell carcinoma or SCC)).mp. [mp=title, abstract, heading word, drug trade name, original title, device manufacturer, drug manufacturer, device trade name, keyword heading word, floating subheading word, candidate term word] (253682)

48 ((cancer$ or tumor$ or tumor$ or neoplasm$ or carcinoma$ or squamous cell carcinoma or SCC) adj4 (nasopharyn$ or oropharyn$ or laryn$ or glotti$ or tonsil$ or epiglotti$ or oral cavity or oral or tongue or gingiva$ or bucca$ or lip or palat$ or gum or mouth floor or floor of mouth or lingual or (head and neck) or HN)).mp. [mp=title, abstract, heading word, drug trade name, original title, device manufacturer, drug manufacturer, device trade name, keyword heading word, floating subheading word, candidate term word] (253682)

49 (HNSCC or SCCHN or HNC or OSCC or OCSCC or OPSCC or LSCC or NPC).mp. [mp=title, abstract, heading word, drug trade name, original title, device manufacturer, drug manufacturer, device trade name, keyword heading word, floating subheading word, candidate term word] (65874)

50 spectrum analysis, raman.mp. or Raman spectrometry/ (49196)

51 Raman Spectrum Analysis.mp. or Raman spectrometry/ (49187)

52 Raman Spectroscopy.mp. or Raman spectrometry/ (56785)

53 Spectroscopy, Raman.mp. (874)

54 Analysis, Raman Spectrum.mp. (1)

55 Raman Optical Activity Spectroscopy.mp. (20)

56 Raman Scattering.mp. (10842)

57 Scattering, Raman.mp. (59)

58 1 or 2 or 3 or 4 or 5 or 6 or 7 or 8 or 9 or 10 or 11 or 12 or 13 or 14 or 15 or 16 or 17 or 18 or 19 or 20 or 21 or 22 or 23 or 24 or 25 or 26 or 27 or 28 or 29 or 30 or 31 or 32 or 33 or 34 or 35 or 36 or 37 or 38 or 39 or 40 or 41 or 42 or 43 or 44 or 45 or 46 or 47 or 48 or 49 (357309)

59 50 or 51 or 52 or 53 or 54 or 55 or 56 or 57 (59079)

60 58 and 59 (383)

***************************

1.

CuO decorated vacancy-rich CeO2 nanopencils for highly efficient catalytic NO reduction by CO at low temperature.

Wang F., Yu Z., Zhai S., Li Y., Xu Y., Ye Y., Wei X., Xu J., Xue B.

Environmental science and pollution research international. 30(11) (pp 31895-31904), 2023. Date of Publication: 01 Mar 2023.

AN: 639685290

With the rapid development of transportation and vehicles, the elimination of NOx and CO has highly attracted public attention. In this work, vacancy-rich CeO2 nanopencil supported CuO catalysts (CuO/CeO2-NPC) were successfully prepared for NO reduction by CO. Importantly, CeO2 with nanopencil-like shape (CeO2-NPC) have been synthesis by solvothermal method for the first time. The physicochemical properties of all samples were studied in detail by combining the means of X-ray diffraction (XRD), Raman spectroscopy, electron paramagnetic resonance (EPR), X-ray photoelectron spectroscopy (XPS), H2-temperature-programmed reduction (H2-TPR), transmission electron microscopy (TEM), scanning electron microscopy (SEM), N2 physisorption (Brunauer-Emmett-Teller), and NO and CO temperature-programmed desorption (NO-TPD and CO-TPD) techniques. Compared with CeO2 nanorods and nanoparticles supported CuO catalysts (CuO/CeO2-NR and CuO/CeO2-NP), the CuO/CeO2-NPC catalysts showed the highest catalytic activity, affording more than 90% NO conversion at 69 degreeC as well as excellent H2O tolerance at 150 degreeC, which is superior to catalysts previously reported. Characterization results indicated that the synergistic effect between the well-dispersed CuO and the CeO2 nanopencil support enables a favorable electron transfer between these components and enhances the density of surface oxygen vacancies and Cu+ species, which consequently accelerating the redox cycle. The results indicated that the morphology control of CeO2 support could be an efficient way to evidently enhance the catalytic performance for NO+CO reaction.

Copyright © 2022. The Author(s), under exclusive licence to Springer-Verlag GmbH Germany, part of Springer Nature.

PMID

36459322 [<https://www.ncbi.nlm.nih.gov/pubmed/?term=36459322>]

Author NameID

Wang, Fei; ORCID: <https://orcid.org/0000-0003-3475-1163>

Institution

(Wang, Yu, Zhai, Li, Xu, Ye, Xu, Xue) Advanced Catalysis and Green Manufacturing Collaborative Innovation Center, School of Petrochemical and Engineering, Changzhou University, Changzhou 213164, China (Wei) School of Chemical Engineering and Materials, Changzhou Institute of Technology, Changzhou 213032, China

Publisher

NLM (Medline)

Emtree Heading

chemistry; cold; temperature; *cerium; copper; cupric oxide.

Drug Index Terms

*cerium; copper; cupric oxide.

Other Index Terms

chemistry; cold; temperature.

Link to the Ovid Full Text or citation:

[Click here for full text options](https://ovidsp.ovid.com/ovidweb.cgi?T=JS&CSC=Y&NEWS=N&PAGE=fulltext&D=emexb&AN=639685290)

Link to the External Link Resolver:

[SFX](https://sfx-86scu.hosted.exlibrisgroup.com.cn/86scu?sid=OVID:embase&id=pmid:36459322&id=doi:10.1007%2Fs11356-022-24508-1&issn=16147499&isbn=&volume=30&issue=11&spage=31895&pages=31895-31904&date=2023&title=Environmental+science+and+pollution+research+international&atitle=CuO+decorated+vacancy-rich+CeO2+nanopencils+for+highly+efficient+catalytic+NO+reduction+by+CO+at+low+temperature&aulast=Wang&pid=<author>Wang+F.%3BYu+Z.%3BZhai+S.%3BLi+Y.%3BXu+Y.%3BYe+Y.%3BWei+X.%3BXu+J.%3BXue+B.<%2Fauthor><AN>639685290<%2FAN><DT>Article<%2FDT>)

2.

Mobile multi-configuration clinical translational Raman system for oral cancer application.

Maryam S., Konugolu Venkata Sekar S., Ghauri M.D., Fahy E., Nogueira M.S., Lu H., Beffara F., Humbert G., Ni Riordain R., Sheahan P., Burke R., Wei Kho K., Gautam R., Andersson-Engels S.

The Analyst. (no pagination), 2023. Date of Publication: 10 Mar 2023.

AN: 640543061

Early diagnosis of oral cancer is critical to improve the survival rate of patients. Raman spectroscopy, a non-invasive spectroscopic technique, has shown potential in identifying early-stage oral cancer biomarkers in the oral cavity environment. However, inherently weak signals necessitate highly sensitive detectors, which restricts widespread usage due to high setup costs. In this research, the fabrication and assembly of a customised Raman system that can adapt three different configurations for the in vivo and ex vivo analysis is reported. This novel design will help in reducing the cost required to have multiple Raman instruments specific for a given application. First, we demonstrated the capability of a customized microscope for acquiring Raman signals from a single cell with high signal-to-noise ratio. Generally, when working with liquid samples with low concentration of analytes (such as saliva) under a microscope, excitation light interacts with a small sample volume, which may not be representative of whole sample. To address this issue, we have designed a novel long-path transmission set-up, which was found to be sensitive towards low concentration of analytes in aqueous solution. We further demonstrated that the same Raman system can be incorporated with the multimodal fibre optical probe to collect in vivo data from oral tissues. In summary, this flexible, portable, multi-configuration Raman system has the potential to provide a cost-effective solution for complete screening of precancer oral lesions.

PMID

36896767 [<https://www.ncbi.nlm.nih.gov/pubmed/?term=36896767>]

Author NameID

Maryam, Siddra; ORCID: <https://orcid.org/0000-0003-2644-2088> Gautam, Rekha; ORCID: <https://orcid.org/0000-0002-1176-8491>

Andersson-Engels, Stefan; ORCID: <https://orcid.org/0000-0001-5640-3122>

Institution

(Maryam) Tyndall National Institute, University College Cork, Cork, Ireland (Konugolu Venkata Sekar, Ghauri, Nogueira, Lu, Burke, Wei Kho, Gautam, Andersson-Engels) Tyndall National Institute, University College Cork, Cork, Ireland

(Fahy, Ni Riordain) Cork University Dental School and Hospital, Wilton, Cork, Ireland

(Beffara, Humbert) XLIM Research Institute, UMR 7252 CNRS/Limoges University, Limoges, France

(Ni Riordain) ENTO Research Institute, University College Cork, Cork, Ireland

(Sheahan) South Infirmary Victoria University Hospital, Cork, Ireland

Publisher

NLM (Medline)

Emtree Heading

animal experiment; animal model; animal tissue; aqueous solution; article; controlled study; ex vivo study; excitation; human; in vivo study; microscope; *mouth cancer; mouth lesion; mouth tissue; nonhuman; precancer; saliva; signal noise ratio.

Other Index Terms

animal experiment [m]; animal model [m]; animal tissue [m]; aqueous solution [m]; article [m]; controlled study [m]; ex vivo study [m]; excitation [m]; human [m]; in vivo study [m]; microscope [m]; *mouth cancer [m]; mouth lesion [m]; mouth tissue [m]; nonhuman [m]; precancer [m]; saliva [m]; signal noise ratio [m].

Link to the Ovid Full Text or citation:

[Click here for full text options](https://ovidsp.ovid.com/ovidweb.cgi?T=JS&CSC=Y&NEWS=N&PAGE=fulltext&D=emexb&AN=640543061)

Link to the External Link Resolver:

[SFX](https://sfx-86scu.hosted.exlibrisgroup.com.cn/86scu?sid=OVID:embase&id=pmid:36896767&id=doi:10.1039%2Fd2an01921c&issn=13645528&isbn=&volume=&issue=&spage=&pages=&date=2023&title=The+Analyst&atitle=Mobile+multi-configuration+clinical+translational+Raman+system+for+oral+cancer+application&aulast=Maryam&pid=<author>Maryam+S.%3BKonugolu+Venkata+Sekar+S.%3BGhauri+M.D.%3BFahy+E.%3BNogueira+M.S.%3BLu+H.%3BBeffara+F.%3BHumbert+G.%3BNi+Riordain+R.%3BSheahan+P.%3BBurke+R.%3BWei+Kho+K.%3BGautam+R.%3BAndersson-Engels+S.<%2Fauthor><AN>640543061<%2FAN><DT>Article<%2FDT>)

3.

High-Precision Detection of Cellular Drug Response Based on SERS Spectrum and Multivariate Statistical Analysis.

Wu F., Wu Z., Wang X., Liu Y., Ye Q.

Biosensors. 13(2) (no pagination), 2023. Article Number: 241. Date of Publication: February 2023.

AN: 2021795073

The rapid development of personalized medicine places high demands on the control of drug dose and cellular drug response to provide patients with better curative effects and low side effects. To solve the problem of low detection accuracies of the cell-counting kit-8 (CCK8) method, a detection method based on surface-enhanced Raman spectroscopy (SERS) of cell-secreted proteins was adopted to evaluate the concentration of the anticancer drug cisplatin and the cellular drug response of nasopharyngeal carcinoma. CNE1 and NP69 cell lines were used to evaluate cisplatin response. The results showed that the combination of the SERS spectrum with principal component analysis-linear discriminant analysis could detect the difference in the response of cisplatin with a concentration difference of 1 mug/mL, which considerably exceeded that of CCK8. In addition, the SERS spectral peak intensity of the cell-secreted proteins strongly correlated with the cisplatin concentration. Furthermore, the mass spectrum of the secreted proteins of the nasopharyngeal carcinoma cells was analyzed to verify the results obtained using the SERS spectrum. The results demonstrated that SERS of secreted proteins has great potential for high-precision detection of chemotherapeutic drug response.

Copyright © 2023 by the authors.

PMID

36832007 [<https://www.ncbi.nlm.nih.gov/pubmed/?term=36832007>]

Author NameID

Wu, Fengfang; ORCID: <https://orcid.org/0000-0002-4415-8171>

Institution

(Wu, Wang, Liu, Ye) Shengli Clinical Medical College of Fujian Medical University, Fuzhou 350001, China (Wu) Department of Otolaryngology, Head and Neck Surgery, Quanzhou First Hospital Affiliated to Fujian Medical University, Quanzhou 362000, China

(Wu, Ye) Department of Otolaryngology, Head and Neck Surgery, Fujian Provincial Hospital, Fuzhou 350001, China

Publisher

MDPI

Emtree Heading

article; cell viability assay kit; CNE-1 cell line; controlled study; discriminant analysis; *drug response; human; human cell; mass spectrometry; *nasopharynx carcinoma; NP69SV40T cell line; principal component analysis; *surface enhanced Raman spectroscopy; antiinfective agent; *cisplatin.

Drug Index Terms

antiinfective agent [m]; *cisplatin [m].

Other Index Terms

article [m]; cell viability assay kit [m]; CNE-1 cell line [m]; controlled study [m]; discriminant analysis [m]; *drug response [m]; human [m]; human cell [m]; mass spectrometry [m]; *nasopharynx carcinoma [m]; NP69SV40T cell line [m]; principal component analysis [m]; *surface enhanced Raman spectroscopy [m].

Link to the Ovid Full Text or citation:

[Click here for full text options](https://ovidsp.ovid.com/ovidweb.cgi?T=JS&CSC=Y&NEWS=N&PAGE=fulltext&D=emexb&AN=2021795073)

Link to the External Link Resolver:

[SFX](https://sfx-86scu.hosted.exlibrisgroup.com.cn/86scu?sid=OVID:embase&id=pmid:36832007&id=doi:10.3390%2Fbios13020241&issn=20796374&isbn=&volume=13&issue=2&spage=241&pages=&date=2023&title=Biosensors&atitle=High-Precision+Detection+of+Cellular+Drug+Response+Based+on+SERS+Spectrum+and+Multivariate+Statistical+Analysis&aulast=Wu&pid=<author>Wu+F.%3BWu+Z.%3BWang+X.%3BLiu+Y.%3BYe+Q.<%2Fauthor><AN>2021795073<%2FAN><DT>Article<%2FDT>)

4.

Mannose-modified erythrocyte membrane-encapsulated chitovanic nanoparticles as a DNA vaccine carrier against reticuloendothelial tissue hyperplasia virus.

Feng Y., Tang F., Li S., Wu D., Liu Q., Li H., Zhang X., Liu Z., Zhang L., Feng H.

Frontiers in Immunology. 13 (no pagination), 2023. Article Number: 1066268. Date of Publication: 04 Jan 2023.

AN: 2021093526

Introduction: The erythrocyte membranes used in nanovaccines include high membrane stability, long circulation life, adaptability and extremely good bio compatibility. Nanoparticles encapsulated by erythrocyte membranes are widely used as ideal drug delivery vehicles because of their high drug loading, long circulation time, and excellent biocompatibility. The mannose modification of delivery materials can help target mannose receptors (MRs) to deliver antigens to antigen-presenting cells (APCs).

Method(s): In this study, the antigen gene gp90 of avian reticuloendotheliosis virus (REV) was encapsulated with carboxymethyl chitosan (CS) to obtain CSgp90 nanoparticles, which were coated with mannose-modied fowl erythrocyte membranes to yield CS-gp90@M-M nanoparticles. The physicochemical characterization and immune response of the CS-gp90@M-M nanoparticles were investigated in vitro and in vivo.

Result(s): CS-gp90@M-M nanoparticles were rapidly phagocytized in vitro by macrophages to induce the production of cytokines and nitric oxide. In vivo, CS-gp90@M-M nanoparticles increased cytokine levels, the CD4+/8+ ratio, REV-specific antibodies in the peripheral blood of chicks, and the mRNA levels of immune-related genes in the spleen and bursa of immunized chicks. CS-gp90@M-M nanoparticles could be targeted to lymphoid organs to prolong the retention time of the nanoparticles at the injection site and lymphatic organs, leading to a strong, sustained immune response. Moreover, the CS-gp90@M-M nano-vaccine showed a lasting immunoprotective effect and improved the body weight of chicks after the challenge.

Conclusion(s): Overall, CS-gp90@M-M nanoparticles can be used in vaccine designs as an effective delivery carrier with immune response-enhancing effects.

Copyright © 2023 Feng, Tang, Li, Wu, Liu, Li, Zhang, Liu, Zhang and Feng.

PMID

36776397 [<https://www.ncbi.nlm.nih.gov/pubmed/?term=36776397>]

Institution

(Feng, Tang, Li, Wu, Liu, Li, Zhang, Liu, Zhang, Feng) College of Animal Husbandry and Veterinary Medicine, Southwest Minzu University, Chengdu, China (Feng, Tang, Li, Wu, Liu, Li, Zhang, Liu, Zhang, Feng) Key Laboratory of Ministry of Education and Sichuan Province for Qinghai-Tibetan Plateau Animal Genetic Resource Reservation and Utilization, Southwest-Minzu University, Chengdu, China

Publisher

Frontiers Media S.A.

Emtree Heading

animal experiment; animal model; animal tissue; antigen presenting cell; article; biocompatibility; CD4+ T lymphocyte; CD8+ T lymphocyte; confocal laser scanning microscopy; controlled study; cytotoxicity; dispersity; *drug delivery system; enzyme linked immunosorbent assay; *erythrocyte membrane; flow cytometer; Fourier transform infrared spectroscopy; immune response; immunization; LSCC-HD11 cell line; MTT assay; nonhuman; particle size; Raman spectrometry; real time polymerase chain reaction; *reticuloendotheliosis/dt [Drug Therapy]; *Reticuloendotheliosis virus; RNA extraction; T lymphocyte; transmission electron microscopy; zeta potential; *chitosan/an [Drug Analysis]; *chitosan/dv [Drug Development]; *chitosan/dt [Drug Therapy]; *DNA vaccine; *drug carrier; inducible nitric oxide synthase; interferon induced helicase C domain containing protein 1; interferon regulatory factor 7; interleukin 4; liposome; *mannose; *nanoparticle/an [Drug Analysis]; *nanoparticle/dv [Drug Development]; nitric oxide; STAT1 protein; toll like receptor 7; confocal laser scanning microscope; ELISA kit; microplate reader; polymerase chain reaction system; spectrophotometer; transmission electron microscope; 8400S; iMark; LabRAM; Tecnai G2 F20.

Candidate Terms

8400S [device term] iMark [device term]

LabRAM [device term]

Tecnai G2 F20 [device term]

Device Index Terms

confocal laser scanning microscope; ELISA kit; flow cytometer; microplate reader; polymerase chain reaction system; spectrophotometer; transmission electron microscope.

Drug Index Terms

*chitosan / *drug analysis / *drug development / *drug therapy; *DNA vaccine; *drug carrier; inducible nitric oxide synthase; interferon induced helicase C domain containing protein 1; interferon regulatory factor 7; interleukin 4; liposome; *mannose; *nanoparticle / *drug analysis / *drug development; nitric oxide; STAT1 protein; toll like receptor 7.

Other Index Terms

animal experiment; animal model; animal tissue; antigen presenting cell; Article; biocompatibility; CD4+ T lymphocyte; CD8+ T lymphocyte; confocal laser scanning microscopy; controlled study; cytotoxicity; dispersity; *drug delivery system; enzyme linked immunosorbent assay; *erythrocyte membrane; flow cytometer; Fourier transform infrared spectroscopy; immune response; immunization; LSCC-HD11 cell line; MTT assay; nonhuman; particle size; Raman spectrometry; real time polymerase chain reaction; *reticuloendotheliosis / *drug therapy; *Reticuloendotheliosis virus; RNA extraction; T lymphocyte; transmission electron microscopy; zeta potential.

Link to the Ovid Full Text or citation:

[Click here for full text options](https://ovidsp.ovid.com/ovidweb.cgi?T=JS&CSC=Y&NEWS=N&PAGE=fulltext&D=emexb&AN=2021093526)

Link to the External Link Resolver:

[SFX](https://sfx-86scu.hosted.exlibrisgroup.com.cn/86scu?sid=OVID:embase&id=pmid:36776397&id=doi:10.3389%2Ffimmu.2022.1066268&issn=16643224&isbn=&volume=13&issue=&spage=1066268&pages=&date=2023&title=Frontiers+in+Immunology&atitle=Mannose-modified+erythrocyte+membrane-encapsulated+chitovanic+nanoparticles+as+a+DNA+vaccine+carrier+against+reticuloendothelial+tissue+hyperplasia+virus&aulast=Feng&pid=<author>Feng+Y.%3BTang+F.%3BLi+S.%3BWu+D.%3BLiu+Q.%3BLi+H.%3BZhang+X.%3BLiu+Z.%3BZhang+L.%3BFeng+H.<%2Fauthor><AN>2021093526<%2FAN><DT>Article<%2FDT>)

5.

Shifted-excitation Raman difference spectroscopy for improving in vivo detection of nasopharyngeal carcinoma.

Lin J., Lin D., Qiu S., Huang Z., Liu F., Huang W., Xu Y., Zhang X., Feng S.

Talanta. 257 (pp 124330), 2023. Date of Publication: 08 Feb 2023.

AN: 640304244

A strong fluorescence background is one of the common interference factors of Raman spectroscopic analysis in biological tissue. This study developed an endoscopic shifted-excitation Raman difference spectroscopy (SERDS) system for real-time in vivo detection of nasopharyngeal carcinoma (NPC) for the first time. Owing to the use of the SERDS method, the high-quality Raman signals of nasopharyngeal tissue could be well extracted and characterized from the complex raw spectra by removing the fluorescence interference signals. Significant spectral differences relating to proteins, phospholipids, glucose, and DNA were found between 42 NPC and 42 normal tissue sites. Using linear discriminant analysis, the diagnostic accuracy of SERDS for NPC detection was 100%, which was much higher than that of raw Raman spectroscopy (75.0%), showing the great potential of SERDS for improving the accurate in vivo detection of NPC.

Copyright © 2023. Published by Elsevier B.V.

PMID

36773510 [<https://www.ncbi.nlm.nih.gov/pubmed/?term=36773510>]

Institution

(Lin) Clinical Oncology School of Fujian Medical University, Fujian Cancer Hospital, Fuzhou, 350014, China; Key Laboratory of OptoElectronic Science and Technology for Medicine, Ministry of Education, Fujian Provincial Key Laboratory for Photonics Technology, Fujian Normal University, Fuzhou, 350007, China (Lin, Huang, Zhang, Feng) Key Laboratory of OptoElectronic Science and Technology for Medicine, Ministry of Education, Fujian Provincial Key Laboratory for Photonics Technology, Fujian Normal University, Fuzhou 350007, China

(Qiu, Xu) Clinical Oncology School of Fujian Medical University, Fujian Cancer Hospital, Fuzhou 350014, China

(Liu) Simple & Smart Instrument (Beijing) Co., Ltd, China

(Huang) Department of Forensic Science, Fujian Police College, Fuzhou 350007, China

Publisher

NLM (Medline)

Emtree Heading

animal experiment; animal model; animal tissue; article; controlled study; diagnostic accuracy; diagnostic test accuracy study; discriminant analysis; *endoscopy; *excitation; fluorescence; human; *in vivo study; *nasopharynx carcinoma; nonhuman; *Raman spectrometry; glucose; phospholipid.

Drug Index Terms

glucose [m]; phospholipid [m].

Other Index Terms

animal experiment [m]; animal model [m]; animal tissue [m]; article [m]; controlled study [m]; diagnostic accuracy [m]; diagnostic test accuracy study [m]; discriminant analysis [m]; *endoscopy [m]; *excitation [m]; fluorescence [m]; human [m]; *in vivo study [m]; *nasopharynx carcinoma [m]; nonhuman [m]; *Raman spectrometry [m].

Link to the Ovid Full Text or citation:

[Click here for full text options](https://ovidsp.ovid.com/ovidweb.cgi?T=JS&CSC=Y&NEWS=N&PAGE=fulltext&D=emexb&AN=640304244)

Link to the External Link Resolver:

[SFX](https://sfx-86scu.hosted.exlibrisgroup.com.cn/86scu?sid=OVID:embase&id=pmid:36773510&id=doi:10.1016%2Fj.talanta.2023.124330&issn=18733573&isbn=&volume=257&issue=&spage=124330&pages=124330&date=2023&title=Talanta&atitle=Shifted-excitation+Raman+difference+spectroscopy+for+improving+in+vivo+detection+of+nasopharyngeal+carcinoma&aulast=Lin&pid=<author>Lin+J.%3BLin+D.%3BQiu+S.%3BHuang+Z.%3BLiu+F.%3BHuang+W.%3BXu+Y.%3BZhang+X.%3BFeng+S.<%2Fauthor><AN>640304244<%2FAN><DT>Article<%2FDT>)

6.

Diagnostic Aids and Techniques of Oral Cancer-An Updated Review.

Deivanayagi M., Afreen Fathima I.

International Journal of Research in Pharmaceutical Sciences. 14(1) (pp 15-20), 2023. Date of Publication: 05 Jan 2023.

AN: 2018967535

Oral cancer is the most prevalent and lethal type with a four in every 3000 incidence rate worldwide and a 3% survival rate. Oral squamous cell car-cinoma (OSCC), which is multifactorial, is brought on by genetic and epigenetic instability. Oral potentially malignant disorders (OPMDs) are precursor lesions that often precede oral cancer, and their early diagnosis is advanta-geous for patients since it may lengthen their productive longevity. Currently, oral cancer screening, early identification, and its pre-invasive intraepithelial phases are still largely focused on visual inspection of the mouth. These methods are subjective and early lesions can be easily missed. Due to a lack of early identification, oral cancer's five-year survival rate is still low. Recently, the World Health Organization (WHO) and the International Agency for Research on Cancer (IARC) emphasised that by developing efficient cancer control and screening strategies, we can prevent a third of the 15 million cancer cases that are expected to occur in the future and better manage a second third. Oral cancer screening aids and techniques have witnessed a lot of advancements recently. This article reviews the current diagnostic methods and instruments for detecting oral cancer, which include the oral CDx brush, Velscope, Chemi-luminescence, DNA ploidy, microarray technology, colposcopy, and oral scan. It also adds on the molecular (genetic and epigenetic alterations) & decoding of the oral carcinogenesis genomics data.

Copyright © International Journal of Research in Pharmaceutical Sciences.

Institution

(Deivanayagi) Department of Oral Medicine and Radiology, Adhiparasakthi Dental College and Hospital, Melmaruvathur, Tamil Nadu, Chengalpattu 603319, India (Afreen Fathima) Ragas Dental College and Hospital, Uthandi, Tamil Nadu, Chennai 600119, India

Publisher

J. K. Welfare and Pharmascope Foundation

Emtree Heading

brush biopsy; cancer screening; carcinogenesis; chemoluminescence; colposcopy; cytology; diagnostic procedure; DNA microarray; early diagnosis; elastography; epigenetics; epithelial mesenchymal transition; fluorescence imaging; gene rearrangement; genomics; high throughput sequencing; human; microarray analysis; *mouth cancer/di [Diagnosis]; optical coherence tomography; oral potentially malignant disorder; ploidy; positron emission tomography; proteomics; reverse transcription polymerase chain reaction; review; sensitivity analysis; squamous cell carcinoma; surface enhanced Raman spectroscopy; survival rate; transcriptomics; tumor invasion; World Health Organization; amylase/ec [Endogenous Compound]; epidermal growth factor receptor/ec [Endogenous Compound]; interleukin 8/ec [Endogenous Compound]; biopsy brush; cancer prognostic test kit; nanochip; ultraviolet detector; reflective tissue fluorescence; tissue fluorescence imaging; Oral CDx brush.

Candidate Terms

reflective tissue fluorescence [other term] tissue fluorescence imaging [other term]

Oral CDx brush [device term]

Device Index Terms

biopsy brush; cancer prognostic test kit; nanochip; ultraviolet detector.

Drug Index Terms

amylase / endogenous compound; epidermal growth factor receptor / endogenous compound; interleukin 8 / endogenous compound.

Other Index Terms

brush biopsy; cancer screening; carcinogenesis; chemoluminescence; colposcopy; cytology; diagnostic procedure; DNA microarray; early diagnosis; elastography; epigenetics; epithelial mesenchymal transition; fluorescence imaging; gene rearrangement; genomics; high throughput sequencing; human; microarray analysis; *mouth cancer / *diagnosis; optical coherence tomography; oral potentially malignant disorder; ploidy; positron emission tomography; proteomics; reverse transcription polymerase chain reaction; Review; sensitivity analysis; squamous cell carcinoma; surface enhanced Raman spectroscopy; survival rate; transcriptomics; tumor invasion; World Health Organization.

Link to the Ovid Full Text or citation:

[Click here for full text options](https://ovidsp.ovid.com/ovidweb.cgi?T=JS&CSC=Y&NEWS=N&PAGE=fulltext&D=emexb&AN=2018967535)

Link to the External Link Resolver:

[SFX](https://sfx-86scu.hosted.exlibrisgroup.com.cn/86scu?sid=OVID:embase&id=pmid:&id=doi:10.26452%2Fijrps.v14i1.4177&issn=09757538&isbn=&volume=14&issue=1&spage=15&pages=15-20&date=2023&title=International+Journal+of+Research+in+Pharmaceutical+Sciences&atitle=Diagnostic+Aids+and+Techniques+of+Oral+Cancer-An+Updated+Review&aulast=Deivanayagi&pid=<author>Deivanayagi+M.%3BAfreen+Fathima+I.<%2Fauthor><AN>2018967535<%2FAN><DT>Review<%2FDT>)

7.

Rapid adsorption of triclosan and p-chloro-m-xylenol by nitrogen-doped magnetic porous carbon.

Li Q., Huang L., Zhu P., Zhong M., Xu S.

Environmental science and pollution research international. 30(1) (pp 1640-1655), 2023. Date of Publication: 01 Jan 2023.

AN: 638660255

Contamination of water resources with organic substances like phenolic fungicides is undesirable due to the improvement of living standards, the huge production of chemicals, the heavy consumption of daily chemical products, and the growth of the population. In this study, Co-based zeolitic imidazole framework-67 (ZIF-67(Co)) was synthesized using the "one-pot method," and the best Co-based N-doped magnetic porous carbon (Co-NPC) was prepared by ZIF-67(Co) carbonization in an atmosphere of N2. The materials were tested using an X-ray diffractometer (XRD), scanning electron microscope (SEM), infrared spectroscopy (IR), Raman spectroscopy, X-ray photoelectron spectroscopy (XPS), N2 adsorption-desorption, and magnetization analysis. These characterizations indicated that the Co-NPC was successfully prepared. With the original morphology of ZIF-67(Co) crystals, the Co-NPC also has good porosity, magnetic properties, and a large specific surface area. In water, Co-NPC-800 has a good adsorption capacity for triclosan (TCS) and p-chloro-m-xylenol (PCMX), which are kinds of aromatic fungicides. The adsorption of Co-NPC-800 on both reached equilibrium within 3 min, which is in accordance with the quasi-second-order kinetic model. At 298 K, the maximum adsorption capacity of Co-NPC-800 for TCS and PCMX was 163 and 39 mg.g-1, respectively. The adsorption of TCS and PCMX by Co-NPC-800 is a spontaneous endothermic process with reduced entropy. The combination of Co-NPC-800 and phenols come from multiple actions of electrostatic, pi-pi, and hydrogen bond effects. Moreover, Co-NPC-800 can be regenerated through simple washing and can be reused at least three times by a magnet. The Co-NPC-800 has good porosity, large specific surface area, comparable adsorption capacity, rapid adsorption time, so it could be broadly used in sewage treatments and other environmental fields.

Copyright © 2022. The Author(s), under exclusive licence to Springer-Verlag GmbH Germany, part of Springer Nature.

PMID

35921007 [<https://www.ncbi.nlm.nih.gov/pubmed/?term=35921007>]

Author NameID

Xu, Shuxia; ORCID: <https://orcid.org/0000-0002-4235-7847>

Institution

(Li) College of Earth Science, Chengdu University of Technology, Chengdu, Sichuan 610059, China (Huang, Zhu, Zhong, Xu) College of Ecology and Environment, Chengdu University of Technology, Chengdu, Sichuan 610059, China

(Zhong, Xu) State Environmental Protection Key Laboratory of Synergetic Control and Joint Remediation for Soil & Water Pollution, Chengdu University of Technology, Chengdu 610059, China

Publisher

NLM (Medline)

Emtree Heading

adsorption; chemistry; kinetics; magnetism; porosity; *water pollutant; carbon; chloroxylenol; *fungicide; nitrogen; *triclosan.

Drug Index Terms

carbon; chloroxylenol; *fungicide; nitrogen; *triclosan.

Other Index Terms

adsorption; chemistry; kinetics; magnetism; porosity; *water pollutant.

Link to the Ovid Full Text or citation:

[Click here for full text options](https://ovidsp.ovid.com/ovidweb.cgi?T=JS&CSC=Y&NEWS=N&PAGE=fulltext&D=emexb&AN=638660255)

Link to the External Link Resolver:

[SFX](https://sfx-86scu.hosted.exlibrisgroup.com.cn/86scu?sid=OVID:embase&id=pmid:35921007&id=doi:10.1007%2Fs11356-022-22084-y&issn=16147499&isbn=&volume=30&issue=1&spage=1640&pages=1640-1655&date=2023&title=Environmental+science+and+pollution+research+international&atitle=Rapid+adsorption+of+triclosan+and+p-chloro-m-xylenol+by+nitrogen-doped+magnetic+porous+carbon&aulast=Li&pid=<author>Li+Q.%3BHuang+L.%3BZhu+P.%3BZhong+M.%3BXu+S.<%2Fauthor><AN>638660255<%2FAN><DT>Article<%2FDT>)

8.

Analysis of urine using electronic tongue towards non-invasive cancer diagnosis.

Zniber M., Vahdatiyekta P., Huynh T.-P.

Biosensors and Bioelectronics. 219 (no pagination), 2023. Article Number: 114810. Date of Publication: 01 Jan 2023.

AN: 2020777988

Electronic tongues (e-tongues) have been broadly employed in monitoring the quality of food, beverage, cosmetics, and pharmaceutical products, and in diagnosis of diseases, as the e-tongues can discriminate samples of high complexity, reduce interference of the matrix, offer rapid response. Compared to other analytical approaches using expensive and complex instrumentation as well as required sample preparation, the e-tongue is non-destructive, miniaturizable and on-site method with little or no preparation of samples. Even though e-tongues are successfully commercialized, their application in cancer diagnosis from urine samples is underestimated. In this review, we would like to highlight the various analytical techniques such as Raman spectroscopy, infrared spectroscopy, fluorescence spectroscopy, and electrochemical methods (potentiometry and voltammetry) used as e-tongues for urine analysis towards non-invasive cancer diagnosis. Besides, different machine learning approaches, for instance, supervised and unsupervised learning algorithms are introduced to analyze extracted chemical data. Finally, capabilities of e-tongues in distinguishing between patients diagnosed with cancer and healthy controls are highlighted.

Copyright © 2022 The Authors

PMID

36272349 [<https://www.ncbi.nlm.nih.gov/pubmed/?term=36272349>]

Author NameID

Zniber, Mohammed; ORCID: <https://orcid.org/0000-0001-9617-5099> Huynh, Tan-Phat; ORCID: <https://orcid.org/0000-0002-7408-7669>

Institution

(Zniber, Vahdatiyekta, Huynh) Laboratory of Molecular Science and Engineering, Abo Akademi University, 20500, Turku, Finland

Publisher

Elsevier Ltd

Emtree Heading

back propagation neural network; breast cancer/di [Diagnosis]; cancer diagnosis; cyclic voltammetry; discriminant analysis; electrochemistry; human; infrared spectroscopy; machine learning; *malignant neoplasm/di [Diagnosis]; *non invasive procedure; principal component analysis; prostate cancer/di [Diagnosis]; Raman spectrometry; review; spectrofluorometry; *urinalysis; voltammetry; biosensor; *medical device; *electronic tongue.

Candidate Terms

*electronic tongue [device term]

Device Index Terms

biosensor; *medical device.

Other Index Terms

back propagation neural network; breast cancer / diagnosis; cancer diagnosis; cyclic voltammetry; discriminant analysis; electrochemistry; human; infrared spectroscopy; machine learning; *malignant neoplasm / *diagnosis; *non invasive procedure; principal component analysis; prostate cancer / diagnosis; Raman spectrometry; Review; spectrofluorometry; *urinalysis; voltammetry.

Link to the Ovid Full Text or citation:

[Click here for full text options](https://ovidsp.ovid.com/ovidweb.cgi?T=JS&CSC=Y&NEWS=N&PAGE=fulltext&D=emexa&AN=2020777988)

Link to the External Link Resolver:

[SFX](https://sfx-86scu.hosted.exlibrisgroup.com.cn/86scu?sid=OVID:embase&id=pmid:36272349&id=doi:10.1016%2Fj.bios.2022.114810&issn=09565663&isbn=&volume=219&issue=&spage=114810&pages=&date=2023&title=Biosensors+and+Bioelectronics&atitle=Analysis+of+urine+using+electronic+tongue+towards+non-invasive+cancer+diagnosis&aulast=Zniber&pid=<author>Zniber+M.%3BVahdatiyekta+P.%3BHuynh+T.-P.<%2Fauthor><AN>2020777988<%2FAN><DT>Review<%2FDT>)

9.

Gefitinib-resveratrol Cocrystal with Optimized Performance in Dissolution and Stability.

Zhai L., Zhang Z., Guo L., Dong H., Yu J., Zhang G.

Journal of Pharmaceutical Sciences. 111(12) (pp 3224-3231), 2022. Date of Publication: December 2022.

AN: 2020773144

Gefitinib (GEF) is an anti-tumor oral solid formulation with a superior advantage for lung tumors. However, it has poor aqueous solubility which limits its utility in vivo. Herein, a novel cocrystal (GEF-RES) assembled by GEF and RES (Resveratrol) has been successfully prepared and comprehensively characterized by differential scanning calorimetry, thermogravimetric analysis, Raman spectroscopy and powder X-ray diffraction. A single-crystal structure of the GEF-RES cocrystal was solved and illustrated in detail. In aqueous hydrochloric acid, the GEF-RES cocrystal showed that the maximum concentration of GEF was slightly higher than that of raw GEF. Furthermore, the thermal and physical stability of the GEF-RES cocrystal were also evaluated in this paper. The enhanced solubility and excellent solid-state stability results may provide new potential to the application of key GEF in clinical.

Copyright © 2022

PMID

36202251 [<https://www.ncbi.nlm.nih.gov/pubmed/?term=36202251>]

Author NameID

Zhang, Guimin; ORCID: <https://orcid.org/0000-0002-3431-8442>

Institution

(Zhai, Zhang, Guo, Dong, Yu, Zhang) Lunan Pharmaceutical Group Co., Ltd, Linyi 273400, China (Zhai, Zhang, Guo, Dong, Zhang) National Engineering and Technology Research Centre of Chiral Pharmaceutical, Linyi 273400, China

Publisher

Elsevier B.V.

Emtree Heading

article; biocompatibility; chemical structure; crystal structure; crystallization; differential scanning calorimetry; *dissolution; drug coating; drug delivery system; drug mixture; drug penetration; drug release; *drug solubility; *drug stability; flow rate; high performance liquid chromatography; hydrogen bond; in vitro study; infrared spectroscopy; maximum concentration; melting point; molecular dynamics; molecular weight; particle size; Raman spectrometry; scanning electron microscopy; size exclusion chromatography; *solid state; surface property; terahertz spectroscopy; thermal analysis; thermogravimetry; thermostability; transmission electron microscopy; X ray diffraction; flavonoid; *gefitinib/an [Drug Analysis]; *gefitinib/pr [Pharmaceutics]; *gefitinib/pk [Pharmacokinetics]; *gefitinib/pd [Pharmacology]; indium; polyphenol; *resveratrol/an [Drug Analysis]; *resveratrol/pr [Pharmaceutics]; *resveratrol/pk [Pharmacokinetics]; *resveratrol/pd [Pharmacology]; high performance liquid chromatograph; scanning electron microscope; thermometer; X ray detector; Sigma 500; THMS 600.

Candidate Terms

Sigma 500 [device term] THMS 600 [device term]

Device Index Terms

high performance liquid chromatograph; scanning electron microscope; thermometer; X ray detector.

Drug Index Terms

flavonoid; *gefitinib / *drug analysis / *pharmaceutics / *pharmacokinetics / *pharmacology; indium; polyphenol; *resveratrol / *drug analysis / *pharmaceutics / *pharmacokinetics / *pharmacology.

Other Index Terms

Article; biocompatibility; chemical structure; crystal structure; crystallization; differential scanning calorimetry; *dissolution; drug coating; drug delivery system; drug mixture; drug penetration; drug release; *drug solubility; *drug stability; flow rate; high performance liquid chromatography; hydrogen bond; in vitro study; infrared spectroscopy; maximum concentration; melting point; molecular dynamics; molecular weight; particle size; Raman spectrometry; scanning electron microscopy; size exclusion chromatography; *solid state; surface property; terahertz spectroscopy; thermal analysis; thermogravimetry; thermostability; transmission electron microscopy; X ray diffraction.

Link to the Ovid Full Text or citation:

[Click here for full text options](https://ovidsp.ovid.com/ovidweb.cgi?T=JS&CSC=Y&NEWS=N&PAGE=fulltext&D=emexb&AN=2020773144)

Link to the External Link Resolver:

[SFX](https://sfx-86scu.hosted.exlibrisgroup.com.cn/86scu?sid=OVID:embase&id=pmid:36202251&id=doi:10.1016%2Fj.xphs.2022.09.031&issn=00223549&isbn=&volume=111&issue=12&spage=3224&pages=3224-3231&date=2022&title=Journal+of+Pharmaceutical+Sciences&atitle=Gefitinib-resveratrol+Cocrystal+with+Optimized+Performance+in+Dissolution+and+Stability&aulast=Zhai&pid=<author>Zhai+L.%3BZhang+Z.%3BGuo+L.%3BDong+H.%3BYu+J.%3BZhang+G.<%2Fauthor><AN>2020773144<%2FAN><DT>Article<%2FDT>)

10.

A narrative review on machine learning in diagnosis and prognosis prediction for tongue squamous cell carcinoma.

Lin Y., Tang M., Liu Y., Jiang M., He S., Zeng D., Cui M.-Y.

Translational Cancer Research. 11(12) (pp 4409-4415), 2022. Date of Publication: December 2022.

AN: 2022596776

Background: Tongue squamous cell carcinoma (TSCC) is the most common subtype of oral cavity squamous cell carcinoma (OCSCC), and it also has the worst prognosis. It is crucial to find an effective way to solve the challenges in diagnosis and prognosis prediction for TSCC. Machine learning (ML) has been widely used in medical research and has shown good performance. It can be used for feature extraction, feature selection, model construction, etc. Radiomics and deep learning (DL), the new components of ML, have also been utilized to explore the relationship between image features and diseases. The current study aimed to highlight the importance of ML as a potential method for addressing the challenges in diagnosis and prognosis prediction of TSCC by reviewing studies on ML in TSCC.

Method(s): The studies on ML in TSCC in PubMed, Scopus, Web of Science, and China National Knowledge Infrastructure published between the dates of inception of these databases and April 30, 2022, were reviewed. Key Content and Findings: ML (including radiomics and DL) which was used in diagnosis and prognosis prediction for TSCC, has shown promising performance.

Conclusion(s): Despite its limitations, ML is still a potential approach that can help to deal with the challenges in diagnosis and prognosis prediction for TSCC. Nevertheless, more efforts are needed to enhance the usefulness of ML in this field.

Copyright © 2022 AME Publishing Company. All rights reserved.

Author NameID

Lin, Yingyu; ORCID: <https://orcid.org/0000-0003-4043-5968> Cui, Min-Yi; ORCID: <https://orcid.org/0000-0002-8900-2461>

Institution

(Lin, Tang) Department of Radiology, The First Affiliated Hospital, Sun Yat-sen University, Guangzhou, China (Liu, Jiang, He, Zeng, Cui) Department of Radiology, Hospital of Stomatology, Sun Yat-sen University, Guangdong Provincial Key Laboratory of Stomatology, Guangzhou, China

Publisher

AME Publishing Company

Emtree Heading

artificial neural network; autofluorescence imaging; *cancer prognosis; computer assisted tomography; deep learning; depth of invasion; diagnostic procedure; Doppler flowmetry; early intervention; feature extraction; feature selection; hyperspectral imaging; *machine learning; mouth cavity; neutrophil lymphocyte ratio; nuclear magnetic resonance; overall survival; Raman spectrometry; reoperation; review; sensitivity and specificity; support vector machine; surgical margin; T2 weighted imaging; *tongue carcinoma/di [Diagnosis].

Other Index Terms

artificial neural network; autofluorescence imaging; *cancer prognosis; computer assisted tomography; deep learning; depth of invasion; diagnostic procedure; Doppler flowmetry; early intervention; feature extraction; feature selection; hyperspectral imaging; *machine learning; mouth cavity; neutrophil lymphocyte ratio; nuclear magnetic resonance; overall survival; Raman spectrometry; reoperation; Review; sensitivity and specificity; support vector machine; surgical margin; T2 weighted imaging; *tongue carcinoma / *diagnosis.

Link to the Ovid Full Text or citation:

[Click here for full text options](https://ovidsp.ovid.com/ovidweb.cgi?T=JS&CSC=Y&NEWS=N&PAGE=fulltext&D=emexb&AN=2022596776)

Link to the External Link Resolver:

[SFX](https://sfx-86scu.hosted.exlibrisgroup.com.cn/86scu?sid=OVID:embase&id=pmid:&id=doi:10.21037%2Ftcr-22-1669&issn=2218676X&isbn=&volume=11&issue=12&spage=4409&pages=4409-4415&date=2022&title=Translational+Cancer+Research&atitle=A+narrative+review+on+machine+learning+in+diagnosis+and+prognosis+prediction+for+tongue+squamous+cell+carcinoma&aulast=Lin&pid=<author>Lin+Y.%3BTang+M.%3BLiu+Y.%3BJiang+M.%3BHe+S.%3BZeng+D.%3BCui+M.-Y.<%2Fauthor><AN>2022596776<%2FAN><DT>Review<%2FDT>)

11.

Gold Nanoparticles in Diagnosis & Management of Oral Cancer & Pre-Cancerous Lesions.

Balmuchu I., Bhagawati B.T., Kumar N., Sharanamma B., Jyotsana

European Journal of Molecular and Clinical Medicine. 9(7) (pp 9263-9267), 2022. Date of Publication: June 2022.

AN: 2022161473

Gold is one of the first metals to have been discovered; the history of its study and application spans at least several thousand years. In particular, the optical properties of GNP are determined by their plasmon resonance, which is associated with the collective excitation of conduction electrons and localized in the broad region. The application of nanotechnology for the treatment of cancer is mostly based on early tumor detection and diagnosis by nanodevices capable of selective targeting and delivery of chemotherapeutic drugs to the specific tumor site. The nanotechnology-based detection and diagnostic methods used are Magnetic resonance imaging (MRI), Optical coherence tomography (OCT), Photoacoustic imaging, Surface plasmon resonance scattering, Surface-enhanced Raman spectroscopy, Diffusion reflection imaging, Quantum dots imaging, and Nano-based ultrasensitive biomarker detection. Due to the remarkable properties of gold nanoparticles, they have long been considered a potential tool for the diagnosis of various cancers and for drug delivery applications. Recent advances in nanomedicine make it auspicious for cancer diagnosis and treatment. The attractive properties of gold nanomaterials, particularly, antiangiogenic properties, are highly useful in a variety of cancer studies. In addition, they can bind many proteins and drugs and can be actively targeted to cancer cells by over-expressing cell surface receptors and they are biocompatible in nature with a high atomic number, which directs to greater absorption of kilovoltage X-rays and provides greater contrast than standard agents. Nano-based contrast agents for MRI, OCT, and photoacoustic imaging have lower toxicity, prolonged blood circulation half-life, and the ability to target unique cell surface molecules. The nano agents exhibit better image contrast properties and improved penetration depth than routine contrast agents. They can provide molecular-targeted imaging, analyze biomarkers at the nano-scale, enable intraoperative identification of surgical resection margins, and monitor oral cancer prognosis after treatment. Using nano-based techniques can help clinicians to detect and better monitor diseases during different phases of oral malignancy. This review will focus on such current modalities in the diagnosis & management of oral cancer and pre-cancerous lesions using gold nanoparticles.

Copyright © 2022 Authors. All rights reserved.

Institution

(Balmuchu, Kumar) Department of Oral Medicine and Radiology, Surendera Dental College & Research Institute, Rajasthan, Sriganganagar, India (Bhagawati) Department of Oral Medicine and Radiology, Dashmesh Institute of Research and Dental Sciences, Punjab, Faridkot, India

(Sharanamma) Department of Periodontology and Implantology, Hazaribagh Dental College, Jharkhand, Hazaribagh, India

(Jyotsana) Department of Pedodontics and Preventive Dentistry, Surendera Dental College & Research Institute, Rajasthan, India

Publisher

EJMCM, International House

Emtree Heading

article; *cancer cell; cancer diagnosis; cancer prognosis; cancer surgery; cell surface; circulation; diagnosis; diagnostic procedure; diffusion; drug delivery system; electron; excision; excitation; gene overexpression; half life time; human; *mouth cancer; nanodevice; nanomedicine; nanotechnology; nuclear magnetic resonance imaging; optical coherence tomography; photoacoustics; *precancer; surface enhanced Raman spectroscopy; surface plasmon resonance; surgery; X ray; antiinfective agent; biological marker; cell surface receptor; contrast medium; *gold nanoparticle; nanochain; quantum dot.

Drug Index Terms

antiinfective agent [m]; biological marker [m]; cell surface receptor [m]; contrast medium [m]; *gold nanoparticle [m]; nanochain [m]; quantum dot [m].

Other Index Terms

article [m]; *cancer cell [m]; cancer diagnosis [m]; cancer prognosis [m]; cancer surgery [m]; cell surface [m]; circulation [m]; diagnosis [m]; diagnostic procedure [m]; diffusion [m]; drug delivery system [m]; electron [m]; excision [m]; excitation [m]; gene overexpression [m]; half life time [m]; human [m]; *mouth cancer [m]; nanodevice [m]; nanomedicine [m]; nanotechnology [m]; nuclear magnetic resonance imaging [m]; optical coherence tomography [m]; photoacoustics [m]; *precancer [m]; surface enhanced Raman spectroscopy [m]; surface plasmon resonance [m]; surgery [m]; X ray [m].

Link to the Ovid Full Text or citation:

[Click here for full text options](https://ovidsp.ovid.com/ovidweb.cgi?T=JS&CSC=Y&NEWS=N&PAGE=fulltext&D=emexb&AN=2022161473)

Link to the External Link Resolver:

[SFX](https://sfx-86scu.hosted.exlibrisgroup.com.cn/86scu?sid=OVID:embase&id=pmid:&id=doi:&issn=25158260&isbn=&volume=9&issue=7&spage=9263&pages=9263-9267&date=2022&title=European+Journal+of+Molecular+and+Clinical+Medicine&atitle=Gold+Nanoparticles+in+Diagnosis+%26+Management+of+Oral+Cancer+%26+Pre-Cancerous+Lesions&aulast=Balmuchu&pid=<author>Balmuchu+I.%3BBhagawati+B.T.%3BKumar+N.%3BSharanamma+B.%3BJyotsana<%2Fauthor><AN>2022161473<%2FAN><DT>Article<%2FDT>)

12.

Profiling of Tumor Cell-Delivered Exosome by Surface Enhanced Raman Spectroscopy-Based Biosensor for Evaluation of Nasopharyngeal Cancer Radioresistance.

Wu Q., Ding Q., Lin W., Weng Y., Feng S., Chen R., Chen C., Qiu S., Lin D.

Advanced Healthcare Materials. (no pagination), 2022. Date of Publication: 2022.

AN: 2020843469

Although the advancement of radiotherapy significantly improves the survival of nasopharyngeal cancer (NPC), radioresistance associated with recurrence and poor outcomes still remains a daunting challenge in the clinical scenario. Currently, effective biomarkers and convenient detection methods for predicting radioresistance have not been well established. Here, the surface-enhanced Raman spectroscopy combined with proteomics is used to firstly profile the characteristic spectral patterns of exosomes secreted from self-established NPC radioresistance cells, and reveals specific variations of proteins expression during radioresistance formation, including collagen alpha-2 (I) chain (COL1A2) that is associated with a favorable prognosis in NPC and is negatively associated with DNA repair scores and DNA repair-related genes via bioinformatic analysis. Furthermore, deep learning model-based diagnostic model is generated to accurately identify the exosomes from radioresistance group. This work demonstrates the promising potential of exosomes as a novel biomarker for predicting the radioresistance and develops a rapid and sensitive liquid biopsy method that will provide a personalized and precise strategy for clinical NPC treatment.

Copyright © 2022 Wiley-VCH GmbH.

PMID

36528342 [<https://www.ncbi.nlm.nih.gov/pubmed/?term=36528342>]

Author NameID

Lin, Duo; ORCID: <https://orcid.org/0000-0001-6959-5995>

Institution

(Wu, Feng, Chen, Lin) Key Laboratory of OptoElectronic Science and Technology for Medicine, Ministry of Education, Fujian Provincial Key Laboratory for Photonics Technology, Fujian Normal University, Fujian, Fuzhou 350001, China (Wu) College of Physics and Electronic Information Engineering, Minjiang University, Fujian, Fuzhou 350001, China

(Ding, Weng, Chen, Qiu) Department of Radiation Oncology, Clinical Oncology School of Fujian Medical University, Fujian Cancer Hospital, Fujian, Fuzhou 350001, China

(Lin) Department of Radiation Oncology, Shanghai Proton and Heavy Ion Center, Fudan University Cancer Hospital, Shanghai 201321, China

Publisher

John Wiley and Sons Inc

Emtree Heading

article; *biosensor; cancer therapy; controlled study; deep learning; *exosome; human; human cell; liquid biopsy; *nasopharynx cancer; *protein fingerprinting; *radiosensitivity; radiotherapy; *surface enhanced Raman spectroscopy; *tumor cell; *biological marker.

Drug Index Terms

*biological marker [m].

Other Index Terms

article [m]; *biosensor [m]; cancer therapy [m]; controlled study [m]; deep learning [m]; *exosome [m]; human [m]; human cell [m]; liquid biopsy [m]; *nasopharynx cancer [m]; *protein fingerprinting [m]; *radiosensitivity [m]; radiotherapy [m]; *surface enhanced Raman spectroscopy [m]; *tumor cell [m].

Link to the Ovid Full Text or citation:

[Click here for full text options](https://ovidsp.ovid.com/ovidweb.cgi?T=JS&CSC=Y&NEWS=N&PAGE=fulltext&D=emexb&AN=2020843469)

Link to the External Link Resolver:

[SFX](https://sfx-86scu.hosted.exlibrisgroup.com.cn/86scu?sid=OVID:embase&id=pmid:36528342&id=doi:10.1002%2Fadhm.202202482&issn=21922640&isbn=&volume=&issue=&spage=&pages=&date=2022&title=Advanced+Healthcare+Materials&atitle=Profiling+of+Tumor+Cell-Delivered+Exosome+by+Surface+Enhanced+Raman+Spectroscopy-Based+Biosensor+for+Evaluation+of+Nasopharyngeal+Cancer+Radioresistance&aulast=Wu&pid=<author>Wu+Q.%3BDing+Q.%3BLin+W.%3BWeng+Y.%3BFeng+S.%3BChen+R.%3BChen+C.%3BQiu+S.%3BLin+D.<%2Fauthor><AN>2020843469<%2FAN><DT>Article<%2FDT>)

13.

Pump-free microfluidic chip based laryngeal squamous cell carcinoma-related microRNAs detection through the combination of surface-enhanced Raman scattering techniques and catalytic hairpin assembly amplification.

Ge S., Li G., Zhou X., Mao Y., Li Z., Gu Y., Cao X.

Talanta. 245 (pp 123478), 2022. Date of Publication: 12 Apr 2022.

AN: 637816171

MicroRNA (miRNA), as one of the ideal target biomarker analytes, plays an essential role in biological processes; thus, the development of rapidly sensitive detection methods is imperative. Herein, we proposed a pump-free surface-enhanced Raman scatting (SERS) microfluidic chip for the rapid and ultrasensitive detection of miR-106b and miR-196b, laryngeal squamous cell carcinoma (LSCC)-related miRNAs. Ag-Au core-shell nanorods (Ag-AuNRs) were applied to prepare SERS tags by modifying Raman reporters and hairpin DNAs. The capture probes were synthesized by labeling hairpin DNAs onto the magnetic beads (MBs) surface. In the presence of targets, the catalytic hairpin assembly (CHA) reactions between SERS tags and capture probes could be triggered, causing the aggregation of Ag-AuNRs. The tiny magnets installed under the rectangular chamber could magnetically gather the CHA products, leading to the further aggregation of Ag-AuNRs. Thus, this strategy could achieve the double aggregation of Ag-AuNRs, resulting in the significant amplification of the SERS signal. The proposed strategy achieved simultaneous and sensitive detection of miR-106b and miR-196b, with limits of detection low to aM level. The whole detection process could be completed within 5 min. Moreover, this microfluidic chip exhibited excellent reproducibility, stability, and specificity. The high accuracy of this SERS microfluidic chip was proved by practical analysis in LSCC patients' serum. The results demonstrated that SERS could be a promising alternative clinical diagnosis tool and exhibited potential application for the dynamic monitoring of cancer staging.

Copyright © 2022 Elsevier B.V. All rights reserved.

PMID

35436733 [<https://www.ncbi.nlm.nih.gov/pubmed/?term=35436733>]

Institution

(Ge, Zhou, Mao, Gu, Gu) Institute of Translational Medicine, Medical College, Yangzhou University, Yangzhou, 225001, PR China; Jiangsu Key Laboratory of Integrated Traditional Chinese and Western Medicine for Prevention and Treatment of Senile Diseases, Yangzhou University, Yangzhou, PR China; Jiangsu Key Laboratory of Experimental & Translational Noncoding RNA Research, Medical College, Yangzhou University, Yangzhou, China (Li) Department of Otorhinolaryngology-Head and Neck Surgery, Affiliated Hospital of Yangzhou University, Yangzhou University, Yangzhou 225001, China

(Li) First Clinical College, Dalian Medical University, Dalian, China

(Cao) Institute of Translational Medicine, Medical College, Yangzhou University, Yangzhou, 225001, PR China; Jiangsu Key Laboratory of Integrated Traditional Chinese and Western Medicine for Prevention and Treatment of Senile Diseases, Yangzhou University, Yangzhou, PR China; Jiangsu Key Laboratory of Experimental & Translational Noncoding RNA Research, Medical College, Yangzhou University, Yangzhou, China. Electronic address: cxw19861121@163.com

Publisher

NLM (Medline)

Emtree Heading

adult; article; cancer patient; cancer staging; *catalysis; controlled study; *gene amplification; human; human tissue; *lab on a chip; *larynx squamous cell carcinoma; limit of detection; magnet; reproducibility; *surface enhanced Raman spectroscopy; endogenous compound; gold; *microRNA.

Drug Index Terms

endogenous compound [m]; gold [m]; *microRNA [m].

Other Index Terms

adult [m]; article [m]; cancer patient [m]; cancer staging [m]; *catalysis [m]; controlled study [m]; *gene amplification [m]; human [m]; human tissue [m]; *lab on a chip [m]; *larynx squamous cell carcinoma [m]; limit of detection [m]; magnet [m]; reproducibility [m]; *surface enhanced Raman spectroscopy [m].

Link to the Ovid Full Text or citation:

[Click here for full text options](https://ovidsp.ovid.com/ovidweb.cgi?T=JS&CSC=Y&NEWS=N&PAGE=fulltext&D=emexb&AN=637816171)

Link to the External Link Resolver:

[SFX](https://sfx-86scu.hosted.exlibrisgroup.com.cn/86scu?sid=OVID:embase&id=pmid:35436733&id=doi:10.1016%2Fj.talanta.2022.123478&issn=18733573&isbn=&volume=245&issue=&spage=123478&pages=123478&date=2022&title=Talanta&atitle=Pump-free+microfluidic+chip+based+laryngeal+squamous+cell+carcinoma-related+microRNAs+detection+through+the+combination+of+surface-enhanced+Raman+scattering+techniques+and+catalytic+hairpin+assembly+amplification&aulast=Ge&pid=<author>Ge+S.%3BLi+G.%3BZhou+X.%3BMao+Y.%3BLi+Z.%3BGu+Y.%3BCao+X.<%2Fauthor><AN>637816171<%2FAN><DT>Article<%2FDT>)

14.

Label-Free Optical Spectroscopy for Early Detection of Oral Cancer.

Maryam S., Nogueira M.S., Gautam R., Krishnamoorthy S., Venkata Sekar S.K., Kho K.W., Lu H., Ni Riordain R., Feeley L., Sheahan P., Burke R., Andersson-Engels S.

Diagnostics. 12(12) (no pagination), 2022. Article Number: 2896. Date of Publication: December 2022.

AN: 2020779646

Oral cancer is the 16th most common cancer worldwide. It commonly arises from painless white or red plaques within the oral cavity. Clinical outcome is highly related to the stage when diagnosed. However, early diagnosis is complex owing to the impracticality of biopsying every potentially premalignant intraoral lesion. Therefore, there is a need to develop a non-invasive cost-effective diagnostic technique to differentiate non-malignant and early-stage malignant lesions. Optical spectroscopy may provide an appropriate solution to facilitate early detection of these lesions. It has many advantages over traditional approaches including cost, speed, objectivity, sensitivity, painlessness, and ease-of use in clinical setting for real-time diagnosis. This review consists of a comprehensive overview of optical spectroscopy for oral cancer diagnosis, epidemiology, and recent improvements in this field for diagnostic purposes. It summarizes major developments in label-free optical spectroscopy, including Raman, fluorescence, and diffuse reflectance spectroscopy during recent years. Among the wide range of optical techniques available, we chose these three for this review because they have the ability to provide biochemical information and show great potential for real-time deep-tissue point-based in vivo analysis. This review also highlights the importance of saliva-based potential biomarkers for non-invasive early-stage diagnosis. It concludes with the discussion on the scope of development and future demands from a clinical point of view.

Copyright © 2022 by the authors.

Author NameID

Nogueira, Marcelo Saito; ORCID: <https://orcid.org/0000-0002-5611-9620> Gautam, Rekha; ORCID: <https://orcid.org/0000-0002-1176-8491>

Venkata Sekar, Sanathana Konugolu; ORCID: <https://orcid.org/0000-0003-0912-1282>

Lu, Huihui; ORCID: <https://orcid.org/0000-0002-7038-202X>

Andersson-Engels, Stefan; ORCID: <https://orcid.org/0000-0001-5640-3122>

Ni Riordain, Richeal; ORCID: <https://orcid.org/0000-0002-1937-8105>

Institution

(Maryam, Nogueira, Gautam, Krishnamoorthy, Venkata Sekar, Kho, Lu, Burke, Andersson-Engels) Tyndall National Institute, University College Cork, Cork T12 R229, Ireland (Ni Riordain, Feeley, Sheahan) ENTO Research Institute, University College Cork, Cork T12 R229, Ireland

(Ni Riordain) Cork University Dental School and Hospital, Wilton, Cork T12 E8YV, Ireland

(Feeley) Cork University Hospital, Cork T12 DC4A, Ireland

(Sheahan) South Infirmary, Victoria University Hospital, Cork T12 X23H, Ireland

Publisher

MDPI

Emtree Heading

cancer diagnosis; cancer staging; clinical outcome; diagnosis; diagnostic procedure; *diffuse reflectance spectroscopy; early diagnosis; human; in vivo study; *mouth cancer; mouth cavity; mouth lesion; *optical spectroscopy; outcome assessment; *Raman spectrometry; review; *saliva analysis; *spectrofluorometry; velocity; *biological marker.

Drug Index Terms

*biological marker [m].

Other Index Terms

cancer diagnosis [m]; cancer staging [m]; clinical outcome [m]; diagnosis [m]; diagnostic procedure [m]; *diffuse reflectance spectroscopy [m]; early diagnosis [m]; human [m]; in vivo study [m]; *mouth cancer [m]; mouth cavity [m]; mouth lesion [m]; *optical spectroscopy [m]; outcome assessment [m]; *Raman spectrometry [m]; review [m]; *saliva analysis [m]; *spectrofluorometry [m]; velocity [m].

Link to the Ovid Full Text or citation:

[Click here for full text options](https://ovidsp.ovid.com/ovidweb.cgi?T=JS&CSC=Y&NEWS=N&PAGE=fulltext&D=emexa&AN=2020779646)

Link to the External Link Resolver:

[SFX](https://sfx-86scu.hosted.exlibrisgroup.com.cn/86scu?sid=OVID:embase&id=pmid:&id=doi:10.3390%2Fdiagnostics12122896&issn=20754418&isbn=&volume=12&issue=12&spage=2896&pages=&date=2022&title=Diagnostics&atitle=Label-Free+Optical+Spectroscopy+for+Early+Detection+of+Oral+Cancer&aulast=Maryam&pid=<author>Maryam+S.%3BNogueira+M.S.%3BGautam+R.%3BKrishnamoorthy+S.%3BVenkata+Sekar+S.K.%3BKho+K.W.%3BLu+H.%3BNi+Riordain+R.%3BFeeley+L.%3BSheahan+P.%3BBurke+R.%3BAndersson-Engels+S.<%2Fauthor><AN>2020779646<%2FAN><DT>Review<%2FDT>)

15.

Machine Learning of Raman Spectroscopy Data for Classifying Cancers: A Review of the Recent Literature.

Blake N., Gaifulina R., Griffin L.D., Bell I.M., Thomas G.M.H.

Diagnostics. 12(6) (no pagination), 2022. Article Number: 1491. Date of Publication: June 2022.

AN: 2017218051

Raman Spectroscopy has long been anticipated to augment clinical decision making, such as classifying oncological samples. Unfortunately, the complexity of Raman data has thus far inhibited their routine use in clinical settings. Traditional machine learning models have been used to help exploit this information, but recent advances in deep learning have the potential to improve the field. However, there are a number of potential pitfalls with both traditional and deep learning models. We conduct a literature review to ascertain the recent machine learning methods used to classify cancers using Raman spectral data. We find that while deep learning models are popular, and ostensibly outperform traditional learning models, there are many methodological considerations which may be leading to an over-estimation of performance; primarily, small sample sizes which compound suboptimal choices regarding sampling and validation strategies. Amongst several recommendations is a call to collate large benchmark Raman datasets, similar to those that have helped transform digital pathology, which researchers can use to develop and refine deep learning models.

Copyright © 2022 by the authors. Licensee MDPI, Basel, Switzerland.

Institution

(Blake, Gaifulina, Thomas) Department of Cell and Developmental Biology, University College London, London WC1E 6BT, United Kingdom (Griffin) Department of Computer Science, University College London, London WC1E 6BT, United Kingdom

(Bell) Spectroscopy Products Division, Renishaw plc, Wotton-under-Edge GL12 8JR, United Kingdom

Publisher

MDPI

Emtree Heading

brain cancer/di [Diagnosis]; breast cancer/di [Diagnosis]; *cancer classification; cancer diagnosis; convolutional neural network; cross validation; deep learning; digital pathology; female genital tract cancer/di [Diagnosis]; gastrointestinal cancer/di [Diagnosis]; human; lung cancer/di [Diagnosis]; *machine learning; *malignant neoplasm/di [Diagnosis]; mouth cancer/di [Diagnosis]; nasopharynx cancer/di [Diagnosis]; prostate cancer/di [Diagnosis]; *Raman spectrometry; review; sample size; skin cancer/di [Diagnosis]; validation process.

Other Index Terms

brain cancer / diagnosis; breast cancer / diagnosis; *cancer classification; cancer diagnosis; convolutional neural network; cross validation; deep learning; digital pathology; female genital tract cancer / diagnosis; gastrointestinal cancer / diagnosis; human; lung cancer / diagnosis; *machine learning; *malignant neoplasm / *diagnosis; mouth cancer / diagnosis; nasopharynx cancer / diagnosis; prostate cancer / diagnosis; *Raman spectrometry; Review; sample size; skin cancer / diagnosis; validation process.

Link to the Ovid Full Text or citation:

[Click here for full text options](https://ovidsp.ovid.com/ovidweb.cgi?T=JS&CSC=Y&NEWS=N&PAGE=fulltext&D=emexa&AN=2017218051)

Link to the External Link Resolver:

[SFX](https://sfx-86scu.hosted.exlibrisgroup.com.cn/86scu?sid=OVID:embase&id=pmid:&id=doi:10.3390%2Fdiagnostics12061491&issn=20754418&isbn=&volume=12&issue=6&spage=1491&pages=&date=2022&title=Diagnostics&atitle=Machine+Learning+of+Raman+Spectroscopy+Data+for+Classifying+Cancers%3A+A+Review+of+the+Recent+Literature&aulast=Blake&pid=<author>Blake+N.%3BGaifulina+R.%3BGriffin+L.D.%3BBell+I.M.%3BThomas+G.M.H.<%2Fauthor><AN>2017218051<%2FAN><DT>Review<%2FDT>)

16.

Multi-Modal Optical Imaging and Combined Phototherapy of Nasopharyngeal Carcinoma Based on a Nanoplatform.

Lin Y., Qiu T., Lan Y., Li Z., Wang X., Zhou M., Li Q., Li Y., Liang J., Zhang J.

International Journal of Nanomedicine. 17 (pp 2435-2446), 2022. Date of Publication: 2022.

AN: 2018803470

Nasopharyngeal carcinoma (NPC) is a common malignant tumor of the head and neck with a high incidence rate worldwide, especially in southern China. Phototheranostics in combination with nanoparticles is an integrated strategy for enabling simultaneous diagnosis, real-time monitoring, and administration of precision therapy for nasopharyngeal carcinoma (NPC). It has shown great potential in the field of cancer diagnosis and treatment owing to its unique noninvasive advantages. Many Chinese and international research teams have applied nano-targeted drugs to optical diagnosis and treatment technology to conduct multimodal imaging and collaborative treatment of NPC, which has become a hot research topic. In this review, we aimed to introduce the recent developments in phototheranostics of NPC based on a nanoplatform. This study aimed to elaborate on the applications of nanoplat-form-based optical imaging strategies and treatment modalities, including fluorescence imaging, photoacoustic imaging, Raman spectroscopy imaging, photodynamic therapy, and photothermal therapy. This study is expected to provide a scientific basis for further research and development of NPC diagnosis and treatment.

Copyright © 2022 Lin et al.

PMID

35656166 [<https://www.ncbi.nlm.nih.gov/pubmed/?term=35656166>]

Institution

(Lin, Li, Li, Li, Liang) Department of Radiology, DongGuan Tungwah Hospital, Guangdong, DongGuan 523000, China (Qiu) Department of Radiology, Zhuhai People's Hospital (Zhuhai Hospital Affiliated with Jinan University), Guangdong, Zhuhai 519000, China

(Lan, Zhou, Zhang) Department of Biomedical Engineering, School of Basic Medical Sciences, Guangzhou Medical University, Guangdong, Guangzhou 511436, China

(Wang, Zhang) Department of Oncology, The Sixth Affiliated Hospital of Guangzhou Medical University, Qingyuan People's Hospital, Guangdong, Qingyuan 511500, China

Publisher

Dove Medical Press Ltd

Emtree Heading

cancer diagnosis; diagnosis; *fluorescence imaging; human; multimodal imaging; *nasopharynx carcinoma; photoacoustics; photodynamic therapy; *phototherapy; photothermal therapy; Raman spectrometry; review; *nanochain.

Drug Index Terms

*nanochain [m].

Other Index Terms

cancer diagnosis [m]; diagnosis [m]; *fluorescence imaging [m]; human [m]; multimodal imaging [m]; *nasopharynx carcinoma [m]; photoacoustics [m]; photodynamic therapy [m]; *phototherapy [m]; photothermal therapy [m]; Raman spectrometry [m]; review [m].

Link to the Ovid Full Text or citation:

[Click here for full text options](https://ovidsp.ovid.com/ovidweb.cgi?T=JS&CSC=Y&NEWS=N&PAGE=fulltext&D=emexa&AN=2018803470)

Link to the External Link Resolver:

[SFX](https://sfx-86scu.hosted.exlibrisgroup.com.cn/86scu?sid=OVID:embase&id=pmid:35656166&id=doi:10.2147%2FIJN.S357493&issn=11769114&isbn=&volume=17&issue=&spage=2435&pages=2435-2446&date=2022&title=International+Journal+of+Nanomedicine&atitle=Multi-Modal+Optical+Imaging+and+Combined+Phototherapy+of+Nasopharyngeal+Carcinoma+Based+on+a+Nanoplatform&aulast=Lin&pid=<author>Lin+Y.%3BQiu+T.%3BLan+Y.%3BLi+Z.%3BWang+X.%3BZhou+M.%3BLi+Q.%3BLi+Y.%3BLiang+J.%3BZhang+J.<%2Fauthor><AN>2018803470<%2FAN><DT>Review<%2FDT>)

17.

Bimetallic Au-Pd nanoparticles/RGO as theranostic nanoplatform for photothermal therapy of throat cancer - an in vitro approach.

Henglei R., Chunli G., Min L., Liang Z.

Journal of Radiation Research and Applied Sciences. 15(4) (no pagination), 2022. Article Number: 100473. Date of Publication: December 2022.

AN: 2021213560

The current work showed the fabrication of bimetallic Au-Pd-NPs on reduced graphene oxide (RGO) sheets functionalized with plant polyphenols of Ziziphus ziziphus leaf extract for enhanced aqueous dispersibility using a simple green method. The synthesized Au-Pd/RGO nanocomposite was effectively employed for the in-vitro photothermal killing of HNE-1 cells. Applying the 808 nm laser (1 W cm-2) and maintaining the concentration of Au-Pd/RGO at 75 mug mL-1, the solution temperature was brought to 58 degreeC, which is adequate for the eradication of cancer cells. Later, the solution temperature was brought to 80 degreeC by increasing the concentrations to 90 mug mL-1. Ultimately, the near total photoablation (=97%) of HNE-1 cells was obtained by maintaining the concentration of Au-Pd-RGO nanocomposites at =20 mug mL-1, a laser power of 1 W cm-1 and a LC50 value of 7 mug mL-1. This exceptional efficiency was attributed mostly to the synergistic effect of Au-Pd NPs, and RGO. The current study might lead the way for the synthesis of advanced materials that could be used as prospective photothermal candidates for the ablation of various unregulated cells.

Copyright © 2022

Institution

(Henglei) Eye and Ent Hospital, China (Chunli, Min, Liang) Fudan University, China

Publisher

Egyptian Society of Radiation Science and Applications (ESRSA)

Emtree Heading

article; cancer cell; cancer therapy; controlled study; cytotoxicity assay; energy dispersive X ray spectroscopy; glioblastoma; HNE-1 cell line; human; human cell; hypoxia; LC50; microvascular thrombosis; MTS assay; MTT assay; *pharynx cancer; *photothermal therapy; Raman spectrometry; transmission electron microscopy; ultraviolet spectrophotometry; X ray diffraction; *gold nanoparticle/an [Drug Analysis]; *gold nanoparticle/cb [Drug Combination]; graphene oxide; nanocomposite; *palladium nanoparticle/an [Drug Analysis]; *palladium nanoparticle/cb [Drug Combination]; plant extract; polyphenol; laser.

Device Index Terms

laser.

Drug Index Terms

*gold nanoparticle / *drug analysis / *drug combination; graphene oxide; nanocomposite; *palladium nanoparticle / *drug analysis / *drug combination; plant extract; polyphenol.

Other Index Terms

Article; cancer cell; cancer therapy; controlled study; cytotoxicity assay; energy dispersive X ray spectroscopy; glioblastoma; HNE-1 cell line; human; human cell; hypoxia; LC50; microvascular thrombosis; MTS assay; MTT assay; *pharynx cancer; *photothermal therapy; Raman spectrometry; transmission electron microscopy; ultraviolet spectrophotometry; X ray diffraction.

Link to the Ovid Full Text or citation:

[Click here for full text options](https://ovidsp.ovid.com/ovidweb.cgi?T=JS&CSC=Y&NEWS=N&PAGE=fulltext&D=emexa&AN=2021213560)

Link to the External Link Resolver:

[SFX](https://sfx-86scu.hosted.exlibrisgroup.com.cn/86scu?sid=OVID:embase&id=pmid:&id=doi:10.1016%2Fj.jrras.2022.100473&issn=16878507&isbn=&volume=15&issue=4&spage=100473&pages=&date=2022&title=Journal+of+Radiation+Research+and+Applied+Sciences&atitle=Bimetallic+Au-Pd+nanoparticles%2FRGO+as+theranostic+nanoplatform+for+photothermal+therapy+of+throat+cancer+-+an+in+vitro+approach&aulast=Henglei&pid=<author>Henglei+R.%3BChunli+G.%3BMin+L.%3BLiang+Z.<%2Fauthor><AN>2021213560<%2FAN><DT>Article<%2FDT>)

18.

Clinical Use of Raman Spectroscopy Improves Diagnostic Accuracy for Indeterminate Thyroid Nodules.

Palermo A., Sodo A., Naciu A.M., Di Gioacchino M., Paolucci A., di Masi A., Maggi D., Crucitti P., Longo F., Perrella E., Taffon C., Verri M., Ricci M.A., Crescenzi A.

The Journal of clinical endocrinology and metabolism. 107(12) (pp 3309-3319), 2022. Date of Publication: 25 Nov 2022.

AN: 639027650

BACKGROUND AND OBJECTIVE: Molecular analysis of thyroid fine-needle aspiration (FNA) specimens is believed to improve the management of indeterminate nodules. Raman spectroscopy (RS) can differentiate benign and malignant thyroid lesions in surgically removed tissues, generating distinctive structural profiles. Herein, the diagnostic performance of RS was tested on FNA biopsies of thyroid gland. DESIGN: Prospective, blinded, and single-center study.

METHOD(S): We enrolled 123 patients with indeterminate or more ominous cytologic diagnoses (TIR3A-low-risk indeterminate lesion, TIR3B-high-risk indeterminate lesion, TIR4-suspicious of malignancy, TIR5-malignant). All subjects were surgical candidates (defined by international guidelines) and submitted to FNA procedures for RS analysis. We compared RS data, cytologic findings, and final histologic assessments (as reference standard) using various statistical techniques.

RESULT(S): The distribution of our study population was as follows: TIR3A:37, TIR3B:32, TIR4:16, and TIR5:38. In 30.9% of patients, histologic diagnoses were benign. For predicting thyroid malignancy in FNA samples, the overall specificity of RS was 86.8%, with 86.5% specificity in indeterminate cytologic categories. In patients with high-risk ultrasound categories, the specificity of RS increased to 87.5% for TIR3A, reaching 100% for TIR3B. Benign histologic diagnoses accounted for 72.9% of patients classified as TIR3A and 31.3% of those classified as TIR3B. Based on positive RS testing, unnecessary surgery was reduced to 7.4% overall (TIR3A-33.3%, TIR3B-6.7%).

CONCLUSION(S): This premier use of RS for thyroid cytology confirms its role as a valuable diagnostic tool and a valid alternative to molecular studies, capable of improving the management of indeterminate nodules and reducing unnecessary surgery.

Copyright © The Author(s) 2022. Published by Oxford University Press on behalf of the Endocrine Society. All rights reserved. For permissions, please e-mail: journals.permissions@oup.com.

PMID

36103268 [<https://www.ncbi.nlm.nih.gov/pubmed/?term=36103268>]

Author NameID

Naciu, Anda Mihaela; ORCID: <https://orcid.org/0000-0002-4576-3381> Palermo, Andrea; ORCID: <https://orcid.org/0000-0002-1143-4926>

Crescenzi, Anna; ORCID: <https://orcid.org/0000-0002-8156-5753>

Institution

(Palermo, Naciu) Unit of Metabolic Bone and Thyroid Disorders, Fondazione Policlinico Universitario Campus Bio-Medico ,Via Alvaro del Portillo, 200 - 00128 Roma, Italy (Palermo) Unit of Endocrinology and Diabetes, Department of Medicine and Surgery, Universita Campus Bio-Medico di Roma, Via Alvaro del Portillo, 21 - 00128 Roma, Italy

(Sodo, Di Gioacchino, Paolucci, di Masi, Ricci) Dipartimento di Scienze, Universita Roma Tre, Rome, Italy

(Maggi) Unit of Endocrinology and Diabetes, Fondazione Policlinico Universitario Campus Bio-Medico, Rome, Italy

(Crucitti, Longo) Unit of Thoracic Surgery, Fondazione Policlinico Universitario Campus Bio-Medico, Rome, Italy

(Perrella, Taffon, Verri, Crescenzi) Unit of Pathology, Fondazione Policlinico Universitario Campus Bio-Medico, Rome, Italy

Publisher

NLM (Medline)

Emtree Heading

fine needle aspiration biopsy; human; pathology; prospective study; Raman spectrometry; retrospective study; *thyroid nodule/di [Diagnosis]; *thyroid nodule/su [Surgery]; *thyroid tumor/di [Diagnosis]; *thyroid tumor/su [Surgery].

Other Index Terms

fine needle aspiration biopsy; human; pathology; prospective study; Raman spectrometry; retrospective study; *thyroid nodule / *diagnosis / *surgery; *thyroid tumor / *diagnosis / *surgery.

Link to the Ovid Full Text or citation:

[Click here for full text options](https://ovidsp.ovid.com/ovidweb.cgi?T=JS&CSC=Y&NEWS=N&PAGE=fulltext&D=emexa&AN=639027650)

Link to the External Link Resolver:

[SFX](https://sfx-86scu.hosted.exlibrisgroup.com.cn/86scu?sid=OVID:embase&id=pmid:36103268&id=doi:10.1210%2Fclinem%2Fdgac537&issn=19457197&isbn=&volume=107&issue=12&spage=3309&pages=3309-3319&date=2022&title=The+Journal+of+clinical+endocrinology+and+metabolism&atitle=Clinical+Use+of+Raman+Spectroscopy+Improves+Diagnostic+Accuracy+for+Indeterminate+Thyroid+Nodules&aulast=Palermo&pid=<author>Palermo+A.%3BSodo+A.%3BNaciu+A.M.%3BDi+Gioacchino+M.%3BPaolucci+A.%3Bdi+Masi+A.%3BMaggi+D.%3BCrucitti+P.%3BLongo+F.%3BPerrella+E.%3BTaffon+C.%3BVerri+M.%3BRicci+M.A.%3BCrescenzi+A.<%2Fauthor><AN>639027650<%2FAN><DT>Article<%2FDT>)

19.

New frontiers in microfluidics devices for miRNA analysis.

Khashayar P., Al-Madhagi S., Azimzadeh M., Scognamiglio V., Arduini F.

TrAC - Trends in Analytical Chemistry. 156 (no pagination), 2022. Article Number: 116706. Date of Publication: November 2022.

AN: 2018922280

miRNA detection using microfluidics-based devices is one of the most important innovations in biology and modern medicine. miRNAs are small regulatory molecules, whose varied concentrations can indicate diseases or pathologic conditions, boosting their use as reliable modern biomarkers for advanced medical diagnostics. Due to their small size and low concentration (from femtomolar to picomolar), miRNA measurement is quite challenging. Being rapid and affordable analytical devices for high-throughput quantification of miRNAs, microfluidics-based biosensors are promising. Beside the small volumes of required sample, simple handling, and manipulation of the sample, these tools enable amplification and detection of target miRNA/miRNAs in a single device, overcoming common limitations of conventional methodologies. Herein, we review the recent advancements in microfluidic devices applied for miRNA sample handling, manipulation, and measurement. We also discuss their advantages and limitations as well as future trends in this field.

Copyright © 2022

Institution

(Khashayar, Al-Madhagi) Center for Microsystems Technology, Imec and Ghent University, Ghent 9050, Belgium (Azimzadeh) Department of Medical Biotechnology, School of Medicine, Shahid Sadoughi University of Medical Sciences, Yazd 8915173143, Iran, Islamic Republic of

(Azimzadeh) Medical Nanotechnology & Tissue Engineering Research Center, Yazd Reproductive Sciences Institute, Shahid Sadoughi University of Medical Sciences, Yazd 89195-999, Iran, Islamic Republic of

(Azimzadeh) Stem Cell Biology Research Center, Yazd Reproductive Sciences Institute, Shahid Sadoughi University of Medical Sciences, Yazd 89195-999, Iran, Islamic Republic of

(Scognamiglio, Arduini) Institute of Crystallography, National Research Council, Department of Chemical Sciences and Materials Technologies, Via Salaria km 29.300, Monterotondo, Rome 00015, Italy

(Arduini) University of Rome "Tor Vergata", Department of Chemical Science and Technologies, Via della Ricerca Scientifica, Rome 00133, Italy

(Arduini) SENSE4MED, Via della Ricerca Scientifica, Rome 00133, Italy

Publisher

Elsevier B.V.

Emtree Heading

brain cancer; cardiovascular disease/di [Diagnosis]; chemoluminescence; CRISPR Cas system; droplet digital polymerase chain reaction; electrochemical detection; electrospray mass spectrometry; exosome; fluorescence; human; lung cancer; mass spectrometry; MCF-7 cell line; MDA-MB-231 cell line; *microfluidics; oral cancer cell line; pancreas cancer/di [Diagnosis]; paratuberculosis; polymerase chain reaction; real time reverse transcription polymerase chain reaction; review; sandwich ELISA; size exclusion chromatography; surface enhanced Raman spectroscopy; surface plasmon resonance; dimeticone; exonuclease/ec [Endogenous Compound]; glucose oxidase/ec [Endogenous Compound]; glypican 1; gold nanoparticle; hydrogel; *microRNA/ec [Endogenous Compound]; oligonucleotide/ec [Endogenous Compound]; peptide nucleic acid/ec [Endogenous Compound]; tetramethylammonium hydroxide; tumor marker; biosensor; field effect transistor; fluorescence microscope; lab on a chip; nucleic acid isolation kit; ultrasound transducer; voltage assisted liquid desorption electrospray ionization tandem mass spectrometry; exoEasy Maxi Kit.

Candidate Terms

voltage assisted liquid desorption electrospray ionization tandem mass spectrometry [other term] exoEasy Maxi Kit [device term]

Device Index Terms

biosensor; field effect transistor; fluorescence microscope; lab on a chip; nucleic acid isolation kit; ultrasound transducer.

Drug Index Terms

dimeticone; exonuclease / endogenous compound; glucose oxidase / endogenous compound; glypican 1; gold nanoparticle; hydrogel; *microRNA / *endogenous compound; oligonucleotide / endogenous compound; peptide nucleic acid / endogenous compound; tetramethylammonium hydroxide; tumor marker.

Other Index Terms

brain cancer; cardiovascular disease / diagnosis; chemoluminescence; CRISPR Cas system; droplet digital polymerase chain reaction; electrochemical detection; electrospray mass spectrometry; exosome; fluorescence; human; lung cancer; mass spectrometry; MCF-7 cell line; MDA-MB-231 cell line; *microfluidics; oral cancer cell line; pancreas cancer / diagnosis; paratuberculosis; polymerase chain reaction; real time reverse transcription polymerase chain reaction; Review; sandwich ELISA; size exclusion chromatography; surface enhanced Raman spectroscopy; surface plasmon resonance.

Link to the Ovid Full Text or citation:

[Click here for full text options](https://ovidsp.ovid.com/ovidweb.cgi?T=JS&CSC=Y&NEWS=N&PAGE=fulltext&D=emexa&AN=2018922280)

Link to the External Link Resolver:

[SFX](https://sfx-86scu.hosted.exlibrisgroup.com.cn/86scu?sid=OVID:embase&id=pmid:&id=doi:10.1016%2Fj.trac.2022.116706&issn=01659936&isbn=&volume=156&issue=&spage=116706&pages=&date=2022&title=TrAC+-+Trends+in+Analytical+Chemistry&atitle=New+frontiers+in+microfluidics+devices+for+miRNA+analysis&aulast=Khashayar&pid=<author>Khashayar+P.%3BAl-Madhagi+S.%3BAzimzadeh+M.%3BScognamiglio+V.%3BArduini+F.<%2Fauthor><AN>2018922280<%2FAN><DT>Review<%2FDT>)

20.

Effect of Paclitaxel Stereochemistry on X-ray-Triggered Release of Paclitaxel from CaWO4/Paclitaxel-Coloaded PEG-PLA Nanoparticles.

Sarkar K., Torregrossa-Allen S.E., Elzey B.D., Narayanan S., Langer M.P., Durm G.A., Won Y.-Y.

Molecular Pharmaceutics. 19(8) (pp 2776-2794), 2022. Date of Publication: 01 Aug 2022.

AN: 2019622171

For many locally advanced tumors, the chemotherapy-radiotherapy (CT-RT) combination ("chemoradiation") is currently the standard of care. Intratumoral (IT) CT-based chemoradiation has the potential to overcome the limitations of conventional systemic CT-RT (side effects). For maximizing the benefits of IT CT-RT, our laboratory has previously developed a radiation-controlled drug release formulation, in which anticancer drug paclitaxel (PTX) and radioluminescent CaWO4 (CWO) nanoparticles (NPs) are co-encapsulated with poly(ethylene glycol)-poly(lactic acid) (PEG-PLA) block copolymers ("PEG-PLA/CWO/PTX NPs"). These PEG-PLA/CWO/PTX NPs enable radiation-controlled release of PTX and are capable of producing sustained therapeutic effects lasting for at least one month following a single IT injection. The present article focuses on discussing our recent finding about the effect of the stereochemical structure of PTX on the efficacy of this PEG-PLA/CWO/PTX NP formulation. Stereochemical differences in two different PTX compounds ("PTX-S"from Samyang Biopharmaceuticals and "PTX-B"from Biotang) were characterized by 2D heteronuclear/homonuclear NMR, Raman spectroscopy, and circular dichroism measurements. The difference in PTX stereochemistry was found to significantly influence their water solubility (WS); PTX-S (WS = 4.69 mug/mL) is about 19 times more water soluble than PTX-B (WS = 0.25 mug/mL). The two PTX compounds showed similar cancer cell-killing performances in vitro when used as free drugs. However, the subtle stereochemical difference significantly influenced their X-ray-triggered release kinetics from the PEG-PLA/CWO/PTX NPs; the more water-soluble PTX-S was released faster than the less water-soluble PTX-B. This difference was manifested in the IT pharmacokinetics and eventually in the survival percentages of test animals (mice) treated with PEG-PLA/CWO/PTX NPs + X-rays in an in vivo human tumor xenograft study; at short times (<1 month), concurrent PEG-PLA/CWO/PTX-S NPs produced a greater tumor-suppression effect, whereas PEG-PLA/CWO/PTX-B NPs had a longer-lasting radio-sensitizing effect. This study demonstrates the importance of the stereochemistry of a drug in a therapy based on a controlled release formulation.

Copyright © 2022 American Chemical Society.

PMID

35834797 [<https://www.ncbi.nlm.nih.gov/pubmed/?term=35834797>]

Author NameID

Won, You-Yeon; ORCID: <https://orcid.org/0000-0002-8347-6375>

Institution

(Sarkar, Won) Davidson School of Chemical Engineering, Purdue University, West Lafayette, IN 47907, United States (Torregrossa-Allen, Elzey, Won) Purdue University Center of Cancer Research, West Lafayette, IN 47907, United States

(Elzey, Narayanan) Department of Comparative Pathobiology, Purdue University, West Lafayette, IN 47907, United States

(Langer) Department of Radiation Oncology, Indiana University School of Medicine, Indianapolis, IN 46202, United States

(Durm) Department of Medicine, Indiana University School of Medicine, Indianapolis, IN 46202, United States

Publisher

American Chemical Society

Emtree Heading

animal experiment; animal model; animal tissue; antineoplastic activity; article; cancer cell; cancer inhibition; cancer survival; carbon nuclear magnetic resonance; cell killing; chemoluminescence; chemoradiotherapy; circular dichroism; *controlled drug release; controlled release formulation; controlled study; drug blood level; drug cytotoxicity; drug degradation; drug efficacy; drug elimination; drug solubility; drug tumor level; female; gel permeation chromatography; head and neck cancer cell line; head and neck squamous cell carcinoma/dt [Drug Therapy]; head and neck squamous cell carcinoma/rt [Radiotherapy]; heteronuclear multiple quantum coherence; human; human cell; in vitro study; in vivo study; kinetics; mouse; nanoencapsulation; nonhuman; nuclear Overhauser effect; physical chemistry; proton nuclear magnetic resonance; radiosensitization; Raman spectrometry; *stereochemistry; transmission electron microscopy; tumor xenograft; water solubility; *X ray; *calcium derivative; copolymer; *macrogol; *paclitaxel/an [Drug Analysis]; *paclitaxel/cr [Drug Concentration]; *paclitaxel/dt [Drug Therapy]; *paclitaxel/tu [Intratumoral Drug Administration]; *paclitaxel/pr [Pharmaceutics]; *paclitaxel/pk [Pharmacokinetics]; *paclitaxel/pd [Pharmacology]; *polyglactin; *polymer nanoparticle; unclassified drug; *calcium tungstate.

Candidate Terms

*calcium tungstate [drug term]

Drug Index Terms

*calcium derivative; copolymer; *macrogol; *paclitaxel / *drug analysis / *drug concentration / *drug therapy / *intratumoral drug administration / *pharmaceutics / *pharmacokinetics / *pharmacology; *polyglactin; *polymer nanoparticle; unclassified drug.

Other Index Terms

animal experiment; animal model; animal tissue; antineoplastic activity; Article; cancer cell; cancer inhibition; cancer survival; carbon nuclear magnetic resonance; cell killing; chemoluminescence; chemoradiotherapy; circular dichroism; *controlled drug release; controlled release formulation; controlled study; drug blood level; drug cytotoxicity; drug degradation; drug efficacy; drug elimination; drug solubility; drug tumor level; female; gel permeation chromatography; head and neck cancer cell line; head and neck squamous cell carcinoma / drug therapy / radiotherapy; heteronuclear multiple quantum coherence; human; human cell; in vitro study; in vivo study; kinetics; mouse; nanoencapsulation; nonhuman; nuclear Overhauser effect; physical chemistry; proton nuclear magnetic resonance; radiosensitization; Raman spectrometry; *stereochemistry; transmission electron microscopy; tumor xenograft; water solubility; *X ray.

Link to the Ovid Full Text or citation:

[Click here for full text options](https://ovidsp.ovid.com/ovidweb.cgi?T=JS&CSC=Y&NEWS=N&PAGE=fulltext&D=emexa&AN=2019622171)

Link to the External Link Resolver:

[SFX](https://sfx-86scu.hosted.exlibrisgroup.com.cn/86scu?sid=OVID:embase&id=pmid:35834797&id=doi:10.1021%2Facs.molpharmaceut.2c00148&issn=15438384&isbn=&volume=19&issue=8&spage=2776&pages=2776-2794&date=2022&title=Molecular+Pharmaceutics&atitle=Effect+of+Paclitaxel+Stereochemistry+on+X-ray-Triggered+Release+of+Paclitaxel+from+CaWO4%2FPaclitaxel-Coloaded+PEG-PLA+Nanoparticles&aulast=Sarkar&pid=<author>Sarkar+K.%3BTorregrossa-Allen+S.E.%3BElzey+B.D.%3BNarayanan+S.%3BLanger+M.P.%3BDurm+G.A.%3BWon+Y.-Y.<%2Fauthor><AN>2019622171<%2FAN><DT>Article<%2FDT>)

21.

Sensitive SERS detection of oral squamous cell carcinoma-related miRNAs in saliva via a gold nanohexagon array coupled with hybridization chain reaction amplification.

Wang Y., Zhang Y., Du Q., Cao D., Lu X., Meng Z.

Analytical methods : advancing methods and applications. 14(44) (pp 4563-4575), 2022. Date of Publication: 18 Nov 2022.

AN: 639407185

In this work, a highly specific and sensitive method for the detection of dual miRNAs was successfully developed by a hybridization chain reaction (HCR) amplification coupled with surface-enhanced Raman scattering (SERS) on Au-Ag hollow nanoparticles (Au-Ag HNPs) and a gold nanohexagon (AuNH) array. Two Raman reporter-labelled and hairpin DNA-modified Au-Ag HNPs acted as SERS probes (Au-Ag HNPs@4-MBA@HP1-1, Au-Ag HNPs@4-MBA@HP2-1, Au-Ag HNPs@DTNB@HP1-2, and Au-Ag HNPs@DTNB@HP2-2), and the hairpin DNA-modified AuNH array acted as the capture substrate. The HCR process could be triggered by the presence of target miRNAs, and long DNA hybridization chains on the substrate were formed by self-assembly rapidly, causing significant signal enhancement. Using the mentioned strategy, a low detection limit (LOD) of 6.51 aM for miR-31 and 6.52 aM for miR-21 in human saliva were obtained, showing the biosensor's remarkable sensitivity. The proposed biosensor also displays a significant specificity in detecting target miRNAs by introducing different interfering factors. This method has been successfully applied to detect and identify miR-21 and miR-31 in saliva from oral squamous cell carcinoma (OSCC) patients and healthy subjects. The results were consistent with those of the traditional test method in detecting target miRNAs, which confirmed the good accuracy of our method. Hence, the new assay method has great potential to be a valuable platform for detecting miRNAs in the early diagnosis of OSCC.

PMID

36317581 [<https://www.ncbi.nlm.nih.gov/pubmed/?term=36317581>]

Author NameID

Meng, Zhibing; ORCID: <https://orcid.org/0000-0003-2402-6056>

Institution

(Wang, Du, Cao) Department of Neurosurgery, Affiliated Hospital of Yangzhou University, Yangzhou, Jiangsu 225000, China (Zhang) Graduate School of Dalian Medical University, Dalian, Liaoning 116011, China

(Lu) Department of Oncology, Affiliated Hospital of Yangzhou University, Yangzhou, Jiangsu 225000, China

(Meng) Department of Oral and Maxillofacial Surgery, Affiliated Hospital of Yangzhou University, Yangzhou, Jiangsu 225000, China

Publisher

NLM (Medline)

Emtree Heading

genetics; *head and neck tumor; human; *mouth tumor/di [Diagnosis]; saliva; *squamous cell carcinoma/di [Diagnosis]; 5,5' dithiobis(2 nitrobenzoic acid); DNA; gold; *microRNA; silver; 4-mercaptobutyramidine.

Candidate Terms

4-mercaptobutyramidine [drug term]

Drug Index Terms

5,5' dithiobis(2 nitrobenzoic acid); DNA; gold; *microRNA; silver.

Other Index Terms

genetics; *head and neck tumor; human; *mouth tumor / *diagnosis; saliva; *squamous cell carcinoma / *diagnosis.

Link to the Ovid Full Text or citation:

[Click here for full text options](https://ovidsp.ovid.com/ovidweb.cgi?T=JS&CSC=Y&NEWS=N&PAGE=fulltext&D=emexa&AN=639407185)

Link to the External Link Resolver:

[SFX](https://sfx-86scu.hosted.exlibrisgroup.com.cn/86scu?sid=OVID:embase&id=pmid:36317581&id=doi:10.1039%2Fd2ay01180h&issn=17599679&isbn=&volume=14&issue=44&spage=4563&pages=4563-4575&date=2022&title=Analytical+methods+%3A+advancing+methods+and+applications&atitle=Sensitive+SERS+detection+of+oral+squamous+cell+carcinoma-related+miRNAs+in+saliva+via+a+gold+nanohexagon+array+coupled+with+hybridization+chain+reaction+amplification&aulast=Wang&pid=<author>Wang+Y.%3BZhang+Y.%3BDu+Q.%3BCao+D.%3BLu+X.%3BMeng+Z.<%2Fauthor><AN>639407185<%2FAN><DT>Article<%2FDT>)

22.

Machine-learning-assisted spontaneous Raman spectroscopy classification and feature extraction for the diagnosis of human laryngeal cancer.

Li Z., Chen Q., Zhang J., Dunham M.E., McWhorter A.J., Feng J.-M., Li Y., Yao S., Xu J.

Computers in Biology and Medicine. 146 (no pagination), 2022. Article Number: 105617. Date of Publication: July 2022.

AN: 2018260768

The early detection of laryngeal cancer significantly increases the survival rates, permits more conservative larynx sparing treatments, and reduces healthcare costs. A non-invasive optical form of biopsy for laryngeal carcinoma can increase the early detection rate, allow for more accurate monitoring of its recurrence, and improve intraoperative margin control. In this study, we evaluated a Raman spectroscopy system for the rapid intraoperative detection of human laryngeal carcinoma. The spectral analysis methods included principal component analysis (PCA), random forest (RF), and one-dimensional (1D) convolutional neural network (CNN) methods. We measured the Raman spectra from 207 normal and 500 tumor sites collected from 10 human laryngeal cancer surgical specimens. Random Forest analysis yielded an overall accuracy of 90.5%, sensitivity of 88.2%, and specificity of 92.8% on average over 10 trials. The 1D CNN demonstrated the highest performance with an accuracy of 96.1%, sensitivity of 95.2%, and specificity of 96.9% on average over 50 trials. In predicting the first three principal components (PCs) of normal and tumor data, both RF and CNN demonstrated high performances, except for the tumor PC2. This is the first study in which CNN-assisted Raman spectroscopy was used to identify human laryngeal cancer tissue with extracted feature weights. The proposed Raman spectroscopy feature extraction approach has not been previously applied to human cancer diagnosis. Raman spectroscopy, as assisted by machine learning (ML) methods, has the potential to serve as an intraoperative, non-invasive tool for the rapid diagnosis of laryngeal cancer and margin detection.

Copyright © 2022 Elsevier Ltd

PMID

35605486 [<https://www.ncbi.nlm.nih.gov/pubmed/?term=35605486>]

Author NameID

Li, Zheng; ORCID: <https://orcid.org/0000-0001-9511-0701>

Institution

(Li, Li, Xu) Division of Electrical and Computer Engineering, College of Engineering, Louisiana State University, Baton Rouge, LA 70803, United States (Chen, Zhang) Division of Computer Science & Engineering, College of Engineering, Louisiana State University, Baton Rouge, LA 70803, United States

(Dunham, McWhorter) Department of Otolaryngology, School of Medicine, Louisiana State University Health Science Center, New Orleans, LA 70112, United States

(Feng, Yao) Department of Comparative Biomedical Science, School of Veterinary Medicine, Louisiana State University, Baton Rouge, LA 70803, United States

(Li) School of Environment and Sustainability, University of Saskatchewan, Saskatoon, SK S7N 5C9, Canada

Publisher

Elsevier Ltd

Emtree Heading

area under the curve; article; *cancer diagnosis; clinical article; clinical evaluation; controlled study; *convolutional neural network; diagnostic accuracy; diagnostic test accuracy study; *feature extraction; human; human tissue; *larynx cancer/di [Diagnosis]; larynx carcinoma; principal component analysis; *Raman spectrometry; random forest; receiver operating characteristic; sensitivity and specificity; survival rate; computer; data analysis software; diode laser; operating room; spectrometer.

Device Index Terms

computer; data analysis software; diode laser; operating room; spectrometer.

Other Index Terms

area under the curve; Article; *cancer diagnosis; clinical article; clinical evaluation; controlled study; *convolutional neural network; diagnostic accuracy; diagnostic test accuracy study; *feature extraction; human; human tissue; *larynx cancer / *diagnosis; larynx carcinoma; principal component analysis; *Raman spectrometry; random forest; receiver operating characteristic; sensitivity and specificity; survival rate.

Link to the Ovid Full Text or citation:

[Click here for full text options](https://ovidsp.ovid.com/ovidweb.cgi?T=JS&CSC=Y&NEWS=N&PAGE=fulltext&D=emexa&AN=2018260768)

Link to the External Link Resolver:

[SFX](https://sfx-86scu.hosted.exlibrisgroup.com.cn/86scu?sid=OVID:embase&id=pmid:35605486&id=doi:10.1016%2Fj.compbiomed.2022.105617&issn=00104825&isbn=&volume=146&issue=&spage=105617&pages=&date=2022&title=Computers+in+Biology+and+Medicine&atitle=Machine-learning-assisted+spontaneous+Raman+spectroscopy+classification+and+feature+extraction+for+the+diagnosis+of+human+laryngeal+cancer&aulast=Li&pid=<author>Li+Z.%3BChen+Q.%3BZhang+J.%3BDunham+M.E.%3BMcWhorter+A.J.%3BFeng+J.-M.%3BLi+Y.%3BYao+S.%3BXu+J.<%2Fauthor><AN>2018260768<%2FAN><DT>Article<%2FDT>)

23.

Fused Raman spectroscopic analysis of blood and saliva delivers high accuracy for head and neck cancer diagnostics.

Koster H.J., Guillen-Perez A., Gomez-Diaz J.S., Navas-Moreno M., Birkeland A.C., Carney R.P.

Scientific reports. 12(1) (pp 18464), 2022. Date of Publication: 02 Nov 2022.

AN: 639432121

As a rapid, label-free, non-destructive analytical measurement requiring little to no sample preparation, Raman spectroscopy shows great promise for liquid biopsy cancer detection and diagnosis. We carried out Raman analysis and mass spectrometry of plasma and saliva from more than 50 subjects in a cohort of head and neck cancer patients and benign controls (e.g., patients with benign oral masses). Unsupervised data models were built to assess diagnostic performance. Raman spectra collected from either biofluid provided moderate performance to discriminate cancer samples. However, by fusing together the Raman spectra of plasma and saliva for each patient, subsequent analytical models delivered an impressive sensitivity, specificity, and accuracy of 96.3%, 85.7%, and 91.7%, respectively. We further confirmed that the metabolites driving the differences in Raman spectra for our models are among the same ones that drive mass spectrometry models, unifying the two techniques and validating the underlying ability of Raman to assess metabolite composition. This study bolsters the relevance of Raman to provide additive value by probing the unique chemical compositions across biofluid sources. Ultimately, we show that a simple data augmentation routine of fusing plasma and saliva spectra provided significantly higher clinical value than either biofluid alone, pushing forward the potential of clinical translation of Raman spectroscopy for liquid biopsy cancer diagnostics.

Copyright © 2022. The Author(s).

PMID

36323705 [<https://www.ncbi.nlm.nih.gov/pubmed/?term=36323705>]

Institution

(Koster, Carney) Biomedical Engineering, University of California, Davis, CA, United States (Guillen-Perez, Gomez-Diaz) Electrical and Computer Engineering, University of California, Davis, CA, United States

(Navas-Moreno) BroomfieldCOUnited States

(Birkeland) Department of Otolaryngology, University of California, Davis, CA, United States

Publisher

NLM (Medline)

Emtree Heading

*head and neck tumor/di [Diagnosis]; human; procedures; *Raman spectrometry; saliva; specimen handling.

Other Index Terms

*head and neck tumor / *diagnosis; human; procedures; *Raman spectrometry; saliva; specimen handling.

Link to the Ovid Full Text or citation:

[Click here for full text options](https://ovidsp.ovid.com/ovidweb.cgi?T=JS&CSC=Y&NEWS=N&PAGE=fulltext&D=emexa&AN=639432121)

Link to the External Link Resolver:

[SFX](https://sfx-86scu.hosted.exlibrisgroup.com.cn/86scu?sid=OVID:embase&id=pmid:36323705&id=doi:10.1038%2Fs41598-022-22197-x&issn=20452322&isbn=&volume=12&issue=1&spage=18464&pages=18464&date=2022&title=Scientific+reports&atitle=Fused+Raman+spectroscopic+analysis+of+blood+and+saliva+delivers+high+accuracy+for+head+and+neck+cancer+diagnostics&aulast=Koster&pid=<author>Koster+H.J.%3BGuillen-Perez+A.%3BGomez-Diaz+J.S.%3BNavas-Moreno+M.%3BBirkeland+A.C.%3BCarney+R.P.<%2Fauthor><AN>639432121<%2FAN><DT>Article<%2FDT>)

24.

Raman spectroscopy in oral cavity and oropharyngeal cancer: a systematic review.

Faur C.I., Falamas A., Chirila M., Roman R.C., Rotaru H., Moldovan M.A., Albu S., Baciut M., Robu I., Hedesiu M.

International Journal of Oral and Maxillofacial Surgery. 51(11) (pp 1373-1381), 2022. Date of Publication: November 2022.

AN: 2017212618

Due to the high incidence of head and neck cancer and under-diagnosis in the early stages, non-invasive and highly accurate diagnostic tests are required for cancer detection. Recent advances in Raman spectroscopy techniques have yielded promising sensitivity and specificity results in the evaluation of cancer. The aim of this study was to investigate the potential value of Raman spectroscopy in oral cavity and oropharyngeal cancer diagnosis based on currently available scientific papers. A search of the PubMed database was performed using a specific strategy and according to the PRISMA guidelines. Raman spectroscopy achieved a maximum accuracy of 98% in cancer detection, while accuracy was 97.24% for tumour grading evaluation, 95% for cancer treatment assessment, and 77% for the detection of cancer recurrence. Moreover, early-stage cancer can be identified by Raman spectroscopy investigation of liquid biopsy samples. An in vivo technique with direct mucosa examination by fibre-optic Raman spectroscopy obtained a maximum accuracy of 94% in cancer diagnosis. The most prominent markers of the presence of malignancy were an increase in Raman signal intensity for proteins, nucleic acids, and water and a decrease for lipids. These cancer discriminants were detected in both fingerprint and high wavenumber regions. In conclusion, Raman spectroscopy is a promising tool for oral cavity and oropharyngeal cancer screening.

Copyright © 2022 International Association of Oral and Maxillofacial Surgeons

PMID

35282942 [<https://www.ncbi.nlm.nih.gov/pubmed/?term=35282942>]

Author NameID

Faur C.I.; ORCID: <https://orcid.org/0000-0001-9808-5580> Chirila M.; ORCID: <https://orcid.org/0000-0002-3771-6614>

Rotaru H.; ORCID: <https://orcid.org/0000-0003-2138-3322>

Institution

(Faur, Hedesiu) Department of Oral Radiology, Iuliu Hatieganu University of Medicine and Pharmacy, Cluj-Napoca, Romania (Falamas) Department of Molecular and Biomolecular Physics, National Institute for Research and Development of Isotopic and Molecular Technologies, Cluj-Napoca, Romania

(Chirila) Department of Otorhinolaryngology, Iuliu Hatieganu University of Medicine and Pharmacy, Cluj-Napoca, Romania

(Roman, Rotaru, Moldovan, Albu) Department of Oral and Craniomaxillofacial Surgery, Iuliu Hatieganu University of Medicine and Pharmacy, Cluj-Napoca, Romania

(Baciut) Department of Maxillofacial Surgery and Implantology, Iuliu Hatieganu University of Medicine and Pharmacy Cluj-Napoca, Romania

(Robu) Doctoral School, Iuliu Hatieganu University of Medicine and Pharmacy, Cluj-Napoca, Romania

Publisher

Churchill Livingstone

Emtree Heading

biochemical analysis; *cancer diagnosis; cancer grading; cancer recurrence; cancer therapy; diagnostic accuracy; diagnostic test accuracy study; early cancer diagnosis; human; liquid biopsy; *mouth cancer/di [Diagnosis]; oral biopsy; *oropharynx cancer/di [Diagnosis]; practice guideline; *Raman spectrometry; review; systematic review; carotenoid/ec [Endogenous Compound]; lipid/ec [Endogenous Compound]; nucleic acid/ec [Endogenous Compound]; protein/ec [Endogenous Compound].

Drug Index Terms

carotenoid / endogenous compound; lipid / endogenous compound; nucleic acid / endogenous compound; protein / endogenous compound.

Other Index Terms

biochemical analysis; *cancer diagnosis; cancer grading; cancer recurrence; cancer therapy; diagnostic accuracy; diagnostic test accuracy study; early cancer diagnosis; human; liquid biopsy; *mouth cancer / *diagnosis; oral biopsy; *oropharynx cancer / *diagnosis; practice guideline; *Raman spectrometry; Review; systematic review.

Link to the Ovid Full Text or citation:

[Click here for full text options](https://ovidsp.ovid.com/ovidweb.cgi?T=JS&CSC=Y&NEWS=N&PAGE=fulltext&D=emexa&AN=2017212618)

Link to the External Link Resolver:

[SFX](https://sfx-86scu.hosted.exlibrisgroup.com.cn/86scu?sid=OVID:embase&id=pmid:35282942&id=doi:10.1016%2Fj.ijom.2022.02.015&issn=09015027&isbn=&volume=51&issue=11&spage=1373&pages=1373-1381&date=2022&title=International+Journal+of+Oral+and+Maxillofacial+Surgery&atitle=Raman+spectroscopy+in+oral+cavity+and+oropharyngeal+cancer%3A+a+systematic+review&aulast=Faur&pid=<author>Faur+C.I.%3BFalamas+A.%3BChirila+M.%3BRoman+R.C.%3BRotaru+H.%3BMoldovan+M.A.%3BAlbu+S.%3BBaciut+M.%3BRobu+I.%3BHedesiu+M.<%2Fauthor><AN>2017212618<%2FAN><DT>Review<%2FDT>)

25.

Simulated fine-needle aspiration diagnosis of follicular thyroid nodules by hyperspectral Raman microscopy and chemometric analysis.

Soares de Oliveira M.A., Campbell M., Afify A.M., Huang E.C., Chan J.W.

Journal of biomedical optics. 27(9) (no pagination), 2022. Date of Publication: 01 Sep 2022.

AN: 638967989

SIGNIFICANCE: Follicular thyroid carcinoma carries a substantially poor prognosis due to its unique biological behavior and less favorable outcomes. In particular, fine-needle aspiration (FNA) biopsies, which play a key role in screening thyroid nodules, cannot differentiate benign from malignant follicular neoplasm. AIM: We report on the use of hyperspectral Raman microscopy in combination with chemometric analysis for identifying and classifying single cells obtained from clinical samples of human follicular thyroid neoplasms. APPROACH: We used a method intended to simulate the FNA procedure to obtain single cells from thyroid nodules. A total of 392 hyperspectral Raman images of single cells from follicular thyroid neoplasms were collected.

RESULT(S): Malignant cells were identified based on their intrinsic Raman spectral signatures with an overall diagnostic accuracy of up to 83.7%.

CONCLUSION(S): Our findings indicate that hyperspectral Raman microscopy can potentially be developed into an ancillary test for analyzing single cells from thyroid FNA biopsies to better stratify "indeterminate" nodules and other cytologically challenging cases.

PMID

36071559 [<https://www.ncbi.nlm.nih.gov/pubmed/?term=36071559>]

Institution

(Soares de Oliveira, Afify, Chan) University of California Davis, Department of Pathology and Laboratory Medicine, Sacramento, CA, United States (Campbell) University of California Davis, Department of Surgery, Sacramento, CA, United States

(Huang) University of Washington, Department of Laboratory Medicine and Pathology, Seattle, WA, United States

Publisher

NLM (Medline)

Emtree Heading

chemometrics; diagnostic imaging; fine needle aspiration biopsy; human; microscopy; pathology; *thyroid nodule; *thyroid tumor.

Other Index Terms

chemometrics; diagnostic imaging; fine needle aspiration biopsy; human; microscopy; pathology; *thyroid nodule; *thyroid tumor.

Link to the Ovid Full Text or citation:

[Click here for full text options](https://ovidsp.ovid.com/ovidweb.cgi?T=JS&CSC=Y&NEWS=N&PAGE=fulltext&D=emed23&AN=638967989)

Link to the External Link Resolver:

[SFX](https://sfx-86scu.hosted.exlibrisgroup.com.cn/86scu?sid=OVID:embase&id=pmid:36071559&id=doi:10.1117%2F1.JBO.27.9.095001&issn=15602281&isbn=&volume=27&issue=9&spage=&pages=&date=2022&title=Journal+of+biomedical+optics&atitle=Simulated+fine-needle+aspiration+diagnosis+of+follicular+thyroid+nodules+by+hyperspectral+Raman+microscopy+and+chemometric+analysis&aulast=Soares+de+Oliveira&pid=<author>Soares+de+Oliveira+M.A.%3BCampbell+M.%3BAfify+A.M.%3BHuang+E.C.%3BChan+J.W.<%2Fauthor><AN>638967989<%2FAN><DT>Article<%2FDT>)

26.

Raman Spectroscopy: A Potential Diagnostic Tool for Oral Diseases.

Zhang Y., Ren L., Wang Q., Wen Z., Liu C., Ding Y.

Frontiers in cellular and infection microbiology. 12 (pp 775236), 2022. Date of Publication: 2022.

AN: 637310878

Oral diseases impose a major health burden worldwide and have a profound effect on general health. Dental caries, periodontal diseases, and oral cancers are the most common oral health conditions. Their occurrence and development are related to oral microbes, and effective measures for their prevention and the promotion of oral health are urgently needed. Raman spectroscopy detects molecular vibration information by collecting inelastic scattering light, allowing a "fingerprint" of a sample to be acquired. It provides the advantages of rapid, sensitive, accurate, and minimally invasive detection as well as minimal interference from water in the "fingerprint region." Owing to these characteristics, Raman spectroscopy has been used in medical detection in various fields to assist diagnosis and evaluate prognosis, such as detecting and differentiating between bacteria or between neoplastic and normal brain tissues. Many oral diseases are related to oral microbial dysbiosis, and their lesions differ from normal tissues in essential components. The colonization of keystone pathogens, such as Porphyromonas gingivalis, resulting in microbial dysbiosis in subgingival plaque, is the main cause of periodontitis. Moreover, the components in gingival crevicular fluid, such as infiltrating inflammatory cells and tissue degradation products, are markedly different between individuals with and without periodontitis. Regarding dental caries, the compositions of decayed teeth are transformed, accompanied by an increase in acid-producing bacteria. In oral cancers, the compositions and structures of lesions and normal tissues are different. Thus, the changes in bacteria and the components of saliva and tissue can be used in examinations as special markers for these oral diseases, and Raman spectroscopy has been acknowledged as a promising measure for detecting these markers. This review summarizes and discusses key research and remaining problems in this area. Based on this, suggestions for further study are proposed.

Copyright © 2022 Zhang, Ren, Wang, Wen, Liu and Ding.

PMID

35186787 [<https://www.ncbi.nlm.nih.gov/pubmed/?term=35186787>]

Institution

(Zhang, Ren, Liu, Ding) State Key Laboratory of Oral Diseases, National Clinical Research Center for Oral Diseases, Department of Periodontics, West China Hospital of Stomatology, Sichuan University, Chengdu, China (Wang) State Key Laboratory of Oral Diseases, National Clinical Research Center for Oral Diseases, Department of Prosthodontics, West China Hospital of Stomatology, Sichuan University, Chengdu, China

(Wen) College of Chemistry, Sichuan University, Chengdu, China

Publisher

NLM (Medline)

Emtree Heading

*dental caries/di [Diagnosis]; dysbiosis; human; microbiology; *periodontitis; Porphyromonas gingivalis; Raman spectrometry.

Other Index Terms

*dental caries / *diagnosis; dysbiosis; human; microbiology; *periodontitis; Porphyromonas gingivalis; Raman spectrometry.

Link to the Ovid Full Text or citation:

[Click here for full text options](https://ovidsp.ovid.com/ovidweb.cgi?T=JS&CSC=Y&NEWS=N&PAGE=fulltext&D=emed23&AN=637310878)

Link to the External Link Resolver:

[SFX](https://sfx-86scu.hosted.exlibrisgroup.com.cn/86scu?sid=OVID:embase&id=pmid:35186787&id=doi:10.3389%2Ffcimb.2022.775236&issn=22352988&isbn=&volume=12&issue=&spage=775236&pages=775236&date=2022&title=Frontiers+in+cellular+and+infection+microbiology&atitle=Raman+Spectroscopy%3A+A+Potential+Diagnostic+Tool+for+Oral+Diseases&aulast=Zhang&pid=<author>Zhang+Y.%3BRen+L.%3BWang+Q.%3BWen+Z.%3BLiu+C.%3BDing+Y.<%2Fauthor><AN>637310878<%2FAN><DT>Review<%2FDT>)

27.

Utilization of Raman spectroscopy in biochemical fingerprint analysis for oral cancer screening and diagnosis.

Sundramoorthy A.K., Atchudan R., Arya S.

Oral Oncology. 135 (no pagination), 2022. Article Number: 106192. Date of Publication: December 2022.

AN: 2020762616

PMID

36270203 [<https://www.ncbi.nlm.nih.gov/pubmed/?term=36270203>]

Institution

(Sundramoorthy) Centre for Nano-Biosensors, Department of Prosthodontics, Saveetha Dental College and Hospitals, Saveetha Institute of Medical and Technical Sciences, Tamil Nadu, Chennai 600077, India (Atchudan) School of Chemical Engineering, Yeungnam University, Gyeongsan 38541, South Korea

(Arya) Department of Physics, University of Jammu, Jammu and Kashmir, Jammu 180006, India

Publisher

Elsevier Ltd

Emtree Heading

bandwidth; *biochemical analysis; *cancer cell; *cancer screening; connective tissue; dentin; enamel; gingiva; gingivitis; human; letter; light; *mouth cancer/di [Diagnosis]; mouth epithelium; optical coherence tomography; *Raman spectrometry; soft tissue; tooth tissue; laser; spectrometer.

Device Index Terms

laser; spectrometer.

Other Index Terms

bandwidth; *biochemical analysis; *cancer cell; *cancer screening; connective tissue; dentin; enamel; gingiva; gingivitis; human; Letter; light; *mouth cancer / *diagnosis; mouth epithelium; optical coherence tomography; *Raman spectrometry; soft tissue; tooth tissue.

Link to the Ovid Full Text or citation:

[Click here for full text options](https://ovidsp.ovid.com/ovidweb.cgi?T=JS&CSC=Y&NEWS=N&PAGE=fulltext&D=emed23&AN=2020762616)

Link to the External Link Resolver:

[SFX](https://sfx-86scu.hosted.exlibrisgroup.com.cn/86scu?sid=OVID:embase&id=pmid:36270203&id=doi:10.1016%2Fj.oraloncology.2022.106192&issn=13688375&isbn=&volume=135&issue=&spage=106192&pages=&date=2022&title=Oral+Oncology&atitle=Utilization+of+Raman+spectroscopy+in+biochemical+fingerprint+analysis+for+oral+cancer+screening+and+diagnosis&aulast=Sundramoorthy&pid=<author>Sundramoorthy+A.K.%3BAtchudan+R.%3BArya+S.<%2Fauthor><AN>2020762616<%2FAN><DT>Letter<%2FDT>)

28.

Diagnostic accuracy of Raman spectroscopy in oral squamous cell carcinoma.

Han R., Lin N., Huang J., Ma X.

Frontiers in Oncology. 12 (no pagination), 2022. Article Number: 925032. Date of Publication: 05 Aug 2022.

AN: 2018703719

Background: Raman spectroscopy (RS) has shown great potential in the diagnosis of oral squamous cell carcinoma (OSCC). Although many single-central original studies have been carried out, it is difficult to use RS in real clinical settings based on the current limited evidence. Herein, we conducted this meta-analysis of diagnostic studies to evaluate the overall performance of RS in OSCC diagnosis.

Method(s): We systematically searched databases including Medline, Embase, and Web of Science for studies up to March 2022 with no start date limited. Data of true positives, true negatives, false positives, and false negatives were extracted from the included studies to calculate the pooled sensitivity, specificity, accuracy, positive and negative likelihood ratios (LRs), and diagnostic odds ratio (DOR) with 95% confidence intervals, then we plotted the summary receiver operating characteristic (SROC) curve and the area under the curve (AUC) to evaluate the overall performance of RS. Quality assessments and publication bias were evaluated by Quality Assessment of Diagnostic Accuracy Studies 2 (QUADAS-2) checklist in Review Manager 5.3. The statistical parameters were calculated with StataSE version 12 and MetaDiSc 1.4.

Result(s): In total, 13 studies were included in our meta-analysis. The pooled diagnostic sensitivity and specificity of RS in OSCC were 0.89 (95% CI, 0.85-0.92) and 0.84 (95% CI, 0.78-0.89). The AUC of SROC curve was 0.93 (95% CI, 0.91-0.95).

Conclusion(s): RS is a non-invasive diagnostic technology with high specificity and sensitivity for detecting OSCC and has the potential to be applied clinically.

Copyright © 2022 Han, Lin, Huang and Ma.

Institution

(Han) State Key Laboratory of Oral Diseases, National Clinical Research Center for Oral Diseases, Sichuan University, Chengdu, China (Lin, Ma) Department of Biotherapy, West China Hospital and State Key Laboratory of Biotherapy, Sichuan University, Chengdu, China

(Huang) Department of Hematology, Sichuan Academy of Medical Sciences, Sichuan Provincial People's Hospital, University of Electronic Science and Technology of China, Chengdu, China

Publisher

Frontiers Media S.A.

Emtree Heading

area under the curve; article; controlled study; *diagnostic accuracy; diagnostic test accuracy study; human; meta analysis; *mouth squamous cell carcinoma; *Raman spectrometry; receiver operating characteristic; sensitivity and specificity.

Other Index Terms

area under the curve; Article; controlled study; *diagnostic accuracy; diagnostic test accuracy study; human; meta analysis; *mouth squamous cell carcinoma; *Raman spectrometry; receiver operating characteristic; sensitivity and specificity.

Link to the Ovid Full Text or citation:

[Click here for full text options](https://ovidsp.ovid.com/ovidweb.cgi?T=JS&CSC=Y&NEWS=N&PAGE=fulltext&D=emed23&AN=2018703719)

Link to the External Link Resolver:

[SFX](https://sfx-86scu.hosted.exlibrisgroup.com.cn/86scu?sid=OVID:embase&id=pmid:&id=doi:10.3389%2Ffonc.2022.925032&issn=2234943X&isbn=&volume=12&issue=&spage=925032&pages=&date=2022&title=Frontiers+in+Oncology&atitle=Diagnostic+accuracy+of+Raman+spectroscopy+in+oral+squamous+cell+carcinoma&aulast=Han&pid=<author>Han+R.%3BLin+N.%3BHuang+J.%3BMa+X.<%2Fauthor><AN>2018703719<%2FAN><DT>Article<%2FDT>)

29.

The Year in Surgical Thyroidology: Recent Technological Developments and Future Challenges.

Tufano R.P., Mohamed Ali K.

Thyroid. 32(1) (pp 14-18), 2022. Date of Publication: 01 Jan 2022.

AN: 637070816

Background: The field of surgical and interventional thyroidology is rapidly evolving. In the past few years, we have seen the introduction and establishment of many novel surgical adjuncts, techniques, and disruptive ablative technologies that have impacted the field.

Method(s): We identified the most influential articles on technological developments in surgical and interventional thyroidology that were published from September 1, 2020, to August 1, 2021. We searched three electronic databases and consulted experts.

Result(s): Major findings are summarized. Continuous intraoperative nerve monitoring (cIONM) lowered the risk of early postoperative vocal cord palsy 1.8-fold and permanent palsy 29 . 4-fold compared with intermittent intraoperative nerve monitoring. Parathyroid autofluorescence yielded a diagnostic odds ratio (OR) of 228.9 for detection of parathyroid glands over visualization, with 96% sensitivity and 92% specificity. There was no significant difference in the incidence of major complications between the transoral endoscopic thyroidectomy vestibular approach (TOETVA) and transcervical thyroidectomy (1.5% vs. 2.1%, p = 0.75), and a higher body mass index did not lead to a significant increase in the odds of cumulative complication with TOETVA for the overweight (OR = 0.52 [95% confidence interval {CI} 0.17-1.58]) and obese groups (OR = 1.69 [CI 0.74-3.88]). Radiofrequency ablation (RFA) for benign thyroid nodules typically resulted in a 50-85% volume reduction with faster recovery times, less pain levels, and higher social and psychological well-being compared with conventional thyroidectomy at 15 months post-treatment, although physical well-being levels were higher in the conventional thyroidectomy group at this time. RFA for papillary thyroid microcarcinoma showed no significant difference in local tumor progression (1.8% vs. 3.3%, p = 0.209), lymph node metastasis (0.6% vs. 0.6%, p = 1.000), recurrence (1.2% vs. 2.4%, p = 0.244), and 4-year recurrence-free survival rates (98.2% vs. 97.0%, p = 0.223) when compared with transcervical lobectomy.

Conclusion(s): cIONM, parathyroid autofluorescence, transoral vestibular approach thyroid surgery, and RFA for benign and malignant thyroid nodules are some of the latest additions to the surgeon's and interventionalist's armamentarium to manage thyroid disease. These technological advancements demonstrate promise to improve outcomes, decrease complications, and enhance a patient's quality of life, but further rigorous studies are needed to define their utility and value.

© Copyright 2022, Mary Ann Liebert, Inc., publishers 2022.

PMID

34915767 [<https://www.ncbi.nlm.nih.gov/pubmed/?term=34915767>]

Author NameID

Mohamed Ali, Khalid; ORCID: <https://orcid.org/0000-0002-8160-3510>

Institution

(Tufano, Mohamed Ali) Department Of Otolaryngology-Head And Neck Surgery, Johns Hopkins School Of Medicine, Baltimore, MD, United States (Tufano) The Fpg Thyroid And Parathyroid Center, Department Of Otolaryngology-Head And Neck Surgery, Sarasota Memorial Health Care System, Sarasota, FL, United States

Publisher

Mary Ann Liebert Inc.

Emtree Heading

active surveillance; adult; article; autofluorescence; body mass; cancer localization; cancer recurrence; controlled study; diagnostic test accuracy study; endoscopic thyroidectomy; female; human; hypoparathyroidism; lymph node metastasis; male; Medline; obesity; open thyroidectomy; optical coherence tomography; parathyroid gland; physical well-being; postoperative care; predictive value; psychological well-being; radiofrequency ablation; Raman spectrometry; recurrence free survival; recurrent laryngeal nerve; retrospective study; surgical approach; systematic review; thyroid nodule/su [Surgery]; thyroid papillary carcinoma/su [Surgery]; *thyroid surgery; thyroidectomy; tumor growth; vocal cord paralysis; operating room; endoscopic thyroidectomy vestibular approach; transcervical thyroidectomy.

Candidate Terms

endoscopic thyroidectomy vestibular approach [other term] transcervical thyroidectomy [other term]

Device Index Terms

operating room.

Other Index Terms

active surveillance; adult; Article; autofluorescence; body mass; cancer localization; cancer recurrence; controlled study; diagnostic test accuracy study; endoscopic thyroidectomy; female; human; hypoparathyroidism; lymph node metastasis; male; Medline; obesity; open thyroidectomy; optical coherence tomography; parathyroid gland; physical well-being; postoperative care; predictive value; psychological well-being; radiofrequency ablation; Raman spectrometry; recurrence free survival; recurrent laryngeal nerve; retrospective study; surgical approach; systematic review; thyroid nodule / surgery; thyroid papillary carcinoma / surgery; *thyroid surgery; thyroidectomy; tumor growth; vocal cord paralysis.

Link to the Ovid Full Text or citation:

[Click here for full text options](https://ovidsp.ovid.com/ovidweb.cgi?T=JS&CSC=Y&NEWS=N&PAGE=fulltext&D=emed23&AN=637070816)

Link to the External Link Resolver:

[SFX](https://sfx-86scu.hosted.exlibrisgroup.com.cn/86scu?sid=OVID:embase&id=pmid:34915767&id=doi:10.1089%2Fthy.2021.0590&issn=10507256&isbn=&volume=32&issue=1&spage=14&pages=14-18&date=2022&title=Thyroid&atitle=The+Year+in+Surgical+Thyroidology%3A+Recent+Technological+Developments+and+Future+Challenges&aulast=Tufano&pid=<author>Tufano+R.P.%3BMohamed+Ali+K.<%2Fauthor><AN>637070816<%2FAN><DT>Article<%2FDT>)

30.

Simultaneous detection of circulating tumor DNAs using a SERS-based lateral flow assay biosensor for point-of-care diagnostics of head and neck cancer.

Li G., Ge S., Niu P., Zhang J., Mao Y., Wang Y., Sun A.

Biomedical Optics Express. 13(8) (pp 4102-4117), 2022. Date of Publication: 01 Aug 2022.

AN: 2019591869

Circulating tumor DNA (ctDNA) has recently emerged as an ideal target for biomarker analytes. Thus, the development of rapid and ultrasensitive ctDNA detection methods is essential. In this study, a high-throughput surface-enhanced Raman scattering (SERS)-based lateral flow assay (LFA) strip is proposed. The aim of this method is to achieve accurate quantification of TP53 and PIK3CA E545K, two types of ctDNAs associated with head and neck squamous cell carcinoma (HNSCC), particularly for point-of-care testing (POCT). Raman reporters and hairpin DNAs are used to functionalize the Pd-Au core-shell nanorods (Pd-AuNRs), which serve as the SERS probes. During the detection process, the existence of targets could open the hairpins on the surface of Pd-AuNRs and trigger the first step of catalytic hairpin assembly (CHA) amplification. The next stage of CHA amplification is initiated by the hairpins prefixed on the test lines, generating numerous "hot spots" to enhance the SERS signal significantly. By the combination of high-performing SERS probes and a target-specific signal amplification strategy, TP53 and PIK3CA E545K are directly quantified in the range of 100 aM-1 nM, with the respective limits of detection (LOD) calculated as 33.1 aM and 20.0 aM in the PBS buffer and 37.8 aM and 23.1 aM in human serum, which are significantly lower than for traditional colorimetric LFA methods. The entire detection process is completed within 45 min, and the multichannel design realizes the parallel detection of multiple groups of samples. Moreover, the analytical performance is validated, including reproducibility, uniformity, and specificity. Finally, the SERS-LFA biosensor is employed to analyze the expression levels of TP53 and PIK3CA E545K in the serum of patients with HNSCC. The results are verified as consistent with those of qRT-PCR. Thus, the SERS-LFA biosensor can be considered as a noninvasive liquid biopsy assay for clinical cancer diagnosis.

Copyright © 2022 Optica Publishing Group under the terms of the Optica Open Access Publishing Agreement.

Institution

(Li, Ge, Sun) Department of Otorhinolaryngology-Head and Neck Surgery, The Affiliated Hospital of Yangzhou University, Yangzhou University, Yangzhou 225001, China (Ge, Mao) Institute of Translational Medicine, Medical College, Yangzhou University, Yangzhou 225001, China

(Niu) Departments of Otolaryngology, Qingzhou People's Hospital, Qingzhou 262500, China

(Zhang) Department of Anesthesiology, The Affiliated Hospital of Yangzhou University, Yangzhou University, Yangzhou 225001, China

(Wang) Department of Neurosurgery, The Affiliated Hospital of Yangzhou University, Yangzhou University, Yangzhou 225001, China

Publisher

Optica Publishing Group (formerly OSA)

Emtree Heading

adult; article; clinical article; controlled study; female; gene amplification; *head and neck cancer/di [Diagnosis]; head and neck squamous cell carcinoma/di [Diagnosis]; human; human tissue; lateral flow immunochromatography; limit of detection; limit of quantitation; male; mathematical parameters; photon correlation spectroscopy; protein expression; quantitative analysis; Raman spectrometry; sensitivity and specificity; signal noise ratio; surface enhanced Raman spectroscopy; surface plasmon resonance; transmission electron microscopy; carcinoembryonic antigen/ec [Endogenous Compound]; cell penetrating peptide/ec [Endogenous Compound]; *circulating tumor DNA/ec [Endogenous Compound]; *biosensor; spectrometer; transmission electron microscope; S-4800II; UV-3600.

Candidate Terms

S-4800II [device term] UV-3600 [device term]

Device Index Terms

*biosensor; spectrometer; transmission electron microscope.

Drug Index Terms

carcinoembryonic antigen / endogenous compound; cell penetrating peptide / endogenous compound; *circulating tumor DNA / *endogenous compound.

Other Index Terms

adult; Article; clinical article; controlled study; female; gene amplification; *head and neck cancer / *diagnosis; head and neck squamous cell carcinoma / diagnosis; human; human tissue; lateral flow immunochromatography; limit of detection; limit of quantitation; male; mathematical parameters; photon correlation spectroscopy; protein expression; quantitative analysis; Raman spectrometry; sensitivity and specificity; signal noise ratio; surface enhanced Raman spectroscopy; surface plasmon resonance; transmission electron microscopy.

Link to the Ovid Full Text or citation:

[Click here for full text options](https://ovidsp.ovid.com/ovidweb.cgi?T=JS&CSC=Y&NEWS=N&PAGE=fulltext&D=emed23&AN=2019591869)

Link to the External Link Resolver:

[SFX](https://sfx-86scu.hosted.exlibrisgroup.com.cn/86scu?sid=OVID:embase&id=pmid:&id=doi:10.1364%2FBOE.463612&issn=21567085&isbn=&volume=13&issue=8&spage=4102&pages=4102-4117&date=2022&title=Biomedical+Optics+Express&atitle=Simultaneous+detection+of+circulating+tumor+DNAs+using+a+SERS-based+lateral+flow+assay+biosensor+for+point-of-care+diagnostics+of+head+and+neck+cancer&aulast=Li&pid=<author>Li+G.%3BGe+S.%3BNiu+P.%3BZhang+J.%3BMao+Y.%3BWang+Y.%3BSun+A.<%2Fauthor><AN>2019591869<%2FAN><DT>Article<%2FDT>)

31.

Evaluationof Gold Nanoparticles for the Detection of Oral Squamous Cell Carcinoma Using RamanSpectroscopy: In-Vitro Study.

Kamel S.S., Faid A.H., Hamdy O., Eltayeb E.A., Zaky A.A.

NeuroQuantology. 20(10) (pp 2284-2296), 2022. Date of Publication: 2022.

AN: 2017833979

Oral cancer is a common and aggressive cancer with high morbidity, mortality, and recurrence rate globally. Early detection is of utmost importance for cancer prevention and disease management. At present, the application of traditional noninvasive methodsis limited by insufficient sensitivity and specificity. Compared to current imaging contrast agents, nanoparticles are more biocompatible, easier to synthesize, and able to target specific surface molecules.Gold nanoparticles (GNPs) synthesized by using sodium citrate as a reducing agent, were used as surface-enhanced Raman scattering substrates to detect human oral squamous cell carcinoma cell lines. Our resultsreveal that the application of GNPs increased the rate of cancer detection in the normal and cancer cells compared to using Raman only. Moreover,there were no significant differences between the average nanoscale concentration ratio and its effect on the size of the cancerous or the normal cells in the null hypothesis. On the other hand, the alternative hypothesis shows significant differences between the average concentration of nanoparticles and their effect on the size of both the normal and cancerous cells.

Copyright © 2022, Anka Publishers. All rights reserved.

Institution

(Kamel, Eltayeb, Zaky) Cairo University, Department of Medical Application of Laser, National Institute of Laser Enhanced Sciences (NILES), Giza 12613, Egypt (Faid) Cairo University, Department of laser science and interaction, National Institute of Laser Enhanced Sciences (NILES), Giza 12613, Egypt

(Hamdy) Cairo University, Department of Engineering Applications of Lasers, National Institute of Laser Enhanced Sciences (NILES), Giza 12613, Egypt

Publisher

Anka Publishers

Emtree Heading

article; biocompatibility; cancer cell; cancer diagnosis; cancer prevention; cell proliferation; cell survival; cell viability; colorimetry; controlled study; cytotoxicity; cytotoxicity assay; cytotoxicity test; diagnostic test accuracy study; enzyme linked immunosorbent assay; Fourier transform infrared spectroscopy; human; human cell; in vitro study; infrared spectroscopy; morbidity; mouth cancer; *mouth squamous cell carcinoma; OECM-1 cell line; oral squamous cell carcinoma cell line; photon correlation spectroscopy; *Raman spectrometry; recurrence risk; sensitivity and specificity; surface enhanced Raman spectroscopy; surface plasmon resonance; transmission electron microscopy; zeta potential; citrate sodium; *gold nanoparticle; nanomaterial; nanoparticle; centrifuge; confocal microscope; electron microscope; ELISA reader; microplate reader; microscope; microwell plate; particle size analyzer; spectrometer; transmission electron microscope; ultraviolet spectrophotometer; Cary 5000; Neya 16R; Tecnai G20; Zetasizer Nano ZN.

Candidate Terms

Cary 5000 [device term] Neya 16R [device term]

Tecnai G20 [device term]

Zetasizer Nano ZN [device term]

Device Index Terms

centrifuge; confocal microscope; electron microscope; ELISA reader; microplate reader; microscope; microwell plate; particle size analyzer; spectrometer; transmission electron microscope; ultraviolet spectrophotometer.

Drug Index Terms

citrate sodium; *gold nanoparticle; nanomaterial; nanoparticle.

Other Index Terms

Article; biocompatibility; cancer cell; cancer diagnosis; cancer prevention; cell proliferation; cell survival; cell viability; colorimetry; controlled study; cytotoxicity; cytotoxicity assay; cytotoxicity test; diagnostic test accuracy study; enzyme linked immunosorbent assay; Fourier transform infrared spectroscopy; human; human cell; in vitro study; infrared spectroscopy; morbidity; mouth cancer; *mouth squamous cell carcinoma; OECM-1 cell line; oral squamous cell carcinoma cell line; photon correlation spectroscopy; *Raman spectrometry; recurrence risk; sensitivity and specificity; surface enhanced Raman spectroscopy; surface plasmon resonance; transmission electron microscopy; zeta potential.

Link to the Ovid Full Text or citation:

[Click here for full text options](https://ovidsp.ovid.com/ovidweb.cgi?T=JS&CSC=Y&NEWS=N&PAGE=fulltext&D=emed23&AN=2017833979)

Link to the External Link Resolver:

[SFX](https://sfx-86scu.hosted.exlibrisgroup.com.cn/86scu?sid=OVID:embase&id=pmid:&id=doi:10.14704%2Fnq.2022.20.10.NQ55197&issn=13035150&isbn=&volume=20&issue=10&spage=2284&pages=2284-2296&date=2022&title=NeuroQuantology&atitle=Evaluationof+Gold+Nanoparticles+for+the+Detection+of+Oral+Squamous+Cell+Carcinoma+Using+RamanSpectroscopy%3A+In-Vitro+Study&aulast=Kamel&pid=<author>Kamel+S.S.%3BFaid+A.H.%3BHamdy+O.%3BEltayeb+E.A.%3BZaky+A.A.<%2Fauthor><AN>2017833979<%2FAN><DT>Article<%2FDT>)

32.

Diagnostics and Therapeutic Applications of Nanotechnology and Nanomedicine in Oral Squamous Cell Carcinoma*.

Dhar R., Shivji G.G., Devi A.

Journal of Stem Cells. 16(4) (pp 207-221), 2022. Date of Publication: 2022.

AN: 2017769241

Oral squamous cell carcinoma (OSCC) is the most common cancer worldwide. Several factors influence OSCC, tobacco use having a high impact on OSCC development. The prevention of OSCC may be possible by reducing exposure to the risk factors of OSCC. OSCC is the sixth most deadly cancer globally, owing to the drawback of the traditional approach in OSCC treatment and failure of diagnosis at an early stage. The primary stage of detection and a sedentary lifestyle mainly contributes to the morbidity and mortality of OSCC patients. In this scenario, nanotechnology gives a new direction for the treatment and diagnosis of oral cancer. Nanomedicine shows a highly promising result with low toxicity. The toxicity of nanomaterial can be controlled by improving functionalization and systematically minimizing toxicity. As a result, nanomaterial and nanomedicine becomes more biologically compatible. Nanomedicine-based targeted drug delivery to the tumor site releases therapeutic agents most effectively, possibly increasing the efficacy of nanomedicine. This book chapter aims to cover the recent developments in Nanotechnology-based drug delivery systems, advance nanomedicines (Nano-capsules, Gold nanoparticles, Quantum dots, and Carbon nanotubes) and their diagnostics as well as therapeutic applications in OSCC.

Copyright © 2022 Nova Science Publishers, Inc.

Institution

(Dhar, Shivji, Devi) Cancer Biology and Stem Cell Biology Lab, Department of Genetic Engineering, SRM Institute of Science and Technology, Tamil Nadu, India

Publisher

Nova Science Publishers, Inc.

Emtree Heading

adult; *cancer diagnosis; cancer prevention; cancer risk; clinical trial (topic); controlled study; drug delivery system; human; morbidity; mortality; *mouth squamous cell carcinoma; *nanomedicine; *nanotechnology; note; patient safety; Raman spectrometry; risk factor; sedentary lifestyle; *treatment outcome; *carbon nanotube; *gold nanoparticle; hydrogel; lipid nanoparticle; nanocapsule; nanomaterial; polymer nanoparticle; *quantum dot.

Drug Index Terms

*carbon nanotube; *gold nanoparticle; hydrogel; lipid nanoparticle; nanocapsule; nanomaterial; polymer nanoparticle; *quantum dot.

Other Index Terms

adult; *cancer diagnosis; cancer prevention; cancer risk; clinical trial (topic); controlled study; drug delivery system; human; morbidity; mortality; *mouth squamous cell carcinoma; *nanomedicine; *nanotechnology; Note; patient safety; Raman spectrometry; risk factor; sedentary lifestyle; *treatment outcome.

Link to the Ovid Full Text or citation:

[Click here for full text options](https://ovidsp.ovid.com/ovidweb.cgi?T=JS&CSC=Y&NEWS=N&PAGE=fulltext&D=emed23&AN=2017769241)

Link to the External Link Resolver:

[SFX](https://sfx-86scu.hosted.exlibrisgroup.com.cn/86scu?sid=OVID:embase&id=pmid:&id=doi:&issn=15568539&isbn=&volume=16&issue=4&spage=207&pages=207-221&date=2022&title=Journal+of+Stem+Cells&atitle=Diagnostics+and+Therapeutic+Applications+of+Nanotechnology+and+Nanomedicine+in+Oral+Squamous+Cell+Carcinoma*&aulast=Dhar&pid=<author>Dhar+R.%3BShivji+G.G.%3BDevi+A.<%2Fauthor><AN>2017769241<%2FAN><DT>Note<%2FDT>)

33.

Stimulated Raman Histology for Rapid Intra-Operative Diagnosis of Sinonasal and Skull Base Tumors.

Fitzgerald C.W.R., Dogan S., Bou-Nassif R., Mclean T., Woods R., Cracchiolo J.R., Ganly I., Tabar V., Cohen M.A.

Laryngoscope. 132(11) (pp 2142-2147), 2022. Date of Publication: November 2022.

AN: 2017453336

Objective: Intra-operative stimulated Raman histology (SRH) is a novel technology that uses laser spectroscopy and color-matching algorithms to create images similar to the formalin-fixed paraffin-embedded (FFPE) section. We aim to assess the accuracy of SRH in a novel range of sinonasal and skull base tumors.

Method(s): Select patients undergoing sinonasal and skull base surgery using the Invenio ImagingTM NioTM Laser Imaging SRH system between June 2020 and September 2021 were assessed. The SRH images were reviewed for pathologic features similar to frozen section (FS) and FFPE. Time taken for results and diagnostic concordance was assessed.

Result(s): Sixty-seven SRH images from 7 tumor types in 12 patients were assessed. Pathologies included squamous cell carcinoma, rhabdomyosarcoma, inverted papilloma, adenoid cystic carcinoma, SMARCB1-deficient sinonasal carcinoma, mucosal melanoma, metastatic colonic adenocarcinoma, and meningioma. Tumor was identified in 100% of lesional specimens, with characteristic diagnostic features readily appreciable on SRH. Median time for diagnosis was significantly faster for SRH (4.3 min) versus FS (44.5 min; p = <.0001). Where SRH sample site matched precisely to FS (n = 32/67, 47.8%), the same diagnosis was confirmed in 93.8%. Sensitivity, specificity, precision, and overall accuracy of SRH were 93.3%, 94.1%, 93.8%, and 93.3%, respectively. Near-perfect concordance was seen between SRH and FS (Cohen's kappa [kappa] = 0.89).

Conclusion(s): Stimulated Raman histology can rapidly produce images similar to FFPE H&E in sinonasal and skull base tumors. This technology has the potential to act as an adjunct or alternative to standard FS.

Level of Evidence: 4 Laryngoscope, 132:2142-2147, 2022.

Copyright © 2022 The American Laryngological, Rhinological and Otological Society, Inc.

PMID

35634892 [<https://www.ncbi.nlm.nih.gov/pubmed/?term=35634892>]

Author NameID

Fitzgerald, Conall W.R.; ORCID: <https://orcid.org/0000-0003-4700-7261> Ganly, Ian; ORCID: <https://orcid.org/0000-0001-7636-5426>

Institution

(Fitzgerald, Mclean, Woods, Cracchiolo, Ganly, Cohen) Head and Neck Service, Department of Surgery, Memorial Sloan Kettering Cancer Center, New York, NY, United States (Dogan) Department of Pathology, Memorial Sloan Kettering Cancer Center, New York, NY, United States

(Bou-Nassif, Tabar) Department of Neurosurgery, Memorial Sloan Kettering Cancer Center, New York, NY, United States

Publisher

John Wiley and Sons Inc

Emtree Heading

accuracy; adenoid cystic carcinoma/di [Diagnosis]; adult; aged; algorithm; article; clinical article; controlled study; diagnosis time; diagnostic test accuracy study; *early diagnosis; ethmoid sinus; female; frontal sinus; frozen section; histopathology; human; human tissue; *intraoperative period; inverted papilloma/di [Diagnosis]; laser spectroscopy; male; maxillary sinus; meningioma/di [Diagnosis]; metastatic colon cancer; mucosal melanoma/di [Diagnosis]; nose cavity; nose surgery; *nose tumor; protein content; pterygopalatine fossa; *Raman spectrometry; rhabdomyosarcoma/di [Diagnosis]; sensitivity and specificity; *skull base tumor; skull surgery; sphenoid sinus; squamous cell carcinoma; tumor diagnosis; formaldehyde; lipid; SWI/SNF related matrix associated actin dependent regulator of chromatin subfamily B member 1; imaging system; Invenio Imaging; nio.

Candidate Terms

Invenio Imaging [device term] Nio [device term]

Device Index Terms

imaging system.

Drug Index Terms

formaldehyde; lipid; SWI/SNF related matrix associated actin dependent regulator of chromatin subfamily B member 1.

Other Index Terms

accuracy; adenoid cystic carcinoma / diagnosis; adult; aged; algorithm; Article; clinical article; controlled study; diagnosis time; diagnostic test accuracy study; *early diagnosis; ethmoid sinus; female; frontal sinus; frozen section; histopathology; human; human tissue; *intraoperative period; inverted papilloma / diagnosis; laser spectroscopy; male; maxillary sinus; meningioma / diagnosis; metastatic colon cancer; mucosal melanoma / diagnosis; nose cavity; nose surgery; *nose tumor; protein content; pterygopalatine fossa; *Raman spectrometry; rhabdomyosarcoma / diagnosis; sensitivity and specificity; *skull base tumor; skull surgery; sphenoid sinus; squamous cell carcinoma; tumor diagnosis.

Link to the Ovid Full Text or citation:

[Click here for full text options](https://ovidsp.ovid.com/ovidweb.cgi?T=JS&CSC=Y&NEWS=N&PAGE=fulltext&D=emed23&AN=2017453336)

Link to the External Link Resolver:

[SFX](https://sfx-86scu.hosted.exlibrisgroup.com.cn/86scu?sid=OVID:embase&id=pmid:35634892&id=doi:10.1002%2Flary.30233&issn=0023852X&isbn=&volume=132&issue=11&spage=2142&pages=2142-2147&date=2022&title=Laryngoscope&atitle=Stimulated+Raman+Histology+for+Rapid+Intra-Operative+Diagnosis+of+Sinonasal+and+Skull+Base+Tumors&aulast=Fitzgerald&pid=<author>Fitzgerald+C.W.R.%3BDogan+S.%3BBou-Nassif+R.%3BMclean+T.%3BWoods+R.%3BCracchiolo+J.R.%3BGanly+I.%3BTabar+V.%3BCohen+M.A.<%2Fauthor><AN>2017453336<%2FAN><DT>Article<%2FDT>)

34.

Histologically resolved multiomics enables precise molecular profiling of human intratumor heterogeneity.

Chen T., Cao C., Zhang J., Streets A., Li T., Huang Y.

PLoS Biology. 20(7) (no pagination), 2022. Article Number: e3001699. Date of Publication: July 2022.

AN: 2019399645

ABUoth: tPhleeacsoemcopnofsiritmiotnhaotfacllehlel taydpiensglaevnedlsthareeirresppraetsiaelndteisdtcriobrurteiocntlyin: a tissue play a critical role in cellular function, organ development, and disease progression. For example, intratumor heterogeneity and the distribution of transcriptional and genetic events in single cells drive the genesis and development of cancer. However, it can be challenging to fully characterize the molecular profile of cells in a tissue with high spatial resolution because microscopy has limited ability to extract comprehensive genomic information, and the spatial resolution of genomic techniques tends to be limited by dissection. There is a growing need for tools that can be used to explore the relationship between histological features, gene expression patterns, and spatially correlated genomic alterations in healthy and diseased tissue samples. Here, we present a technique that combines label-free histology with spatially resolved multiomics in unfixed and unstained tissue sections. This approach leverages stimulated Raman scattering microscopy to provide chemical contrast that reveals histological tissue architecture, allowing for high-resolution in situ laser microdissection of regions of interests. These microtissue samples are then processed for DNA and RNA sequencing to identify unique genetic profiles that correspond to distinct anatomical regions. We demonstrate the capabilities of this technique by mapping gene expression and copy number alterations to histologically defined regions in human oral squamous cell carcinoma (OSCC). Our approach provides complementary insights in tumorigenesis and offers an integrative tool for macroscale cancer tissues with spatial multiomics assessments.

Copyright © 2022 Chen et al. This is an open access article distributed under the terms of the Creative Commons Attribution License, which permits unrestricted use, distribution, and reproduction in any medium, provided the original author and source are credited.

PMID

35776767 [<https://www.ncbi.nlm.nih.gov/pubmed/?term=35776767>]

Institution

(Chen, Cao, Streets, Huang) Biomedical Pioneering Innovation Center (BIOPIC), School of Life Sciences, Peking University, Beijing, China (Chen, Huang) College of Engineering, Peking University, Beijing, China

(Zhang, Li) Department of Oral Pathology, Peking University, School and Hospital of Stomatology, National Center of Stomatology, National Clinical Research Center for Oral Diseases, National Engineering Research Center of Oral Biomaterials and Digital Medical Devices, Beijing, China

(Zhang, Li) Beijing Key Laboratory of Digital Stomatology, Chinese Academy of Medical Sciences (2019RU034), Beijing, China

(Li) Research Unit of Precision Pathologic Diagnosis in Tumors of the Oral and Maxillofacial Regions, Chinese Academy of Medical Sciences (2019RU034), Beijing, China

(Huang) Peking-Tsinghua Center for Life Sciences, Peking University, Beijing, China

(Huang) Beijing Advanced Innovation Center for Genomics (ICG), Peking University, Beijing, China

(Huang) College of Chemistry and Molecular Engineering, Peking University, Beijing, China

(Huang) Institute for Cell Analysis, Shenzhen Bay Laboratory, Guangdong, China

Publisher

Public Library of Science

Emtree Heading

accuracy; article; cancer survival; cancer tissue; case report; clinical article; controlled study; DNA sequencing; female; gene amplification; gene dosage; gene expression; genetic profile; human; human tissue; laser microdissection; male; microscopy; *molecular fingerprinting; mouth squamous cell carcinoma; *multiomics; overall survival; RNA sequencing; survival time; tissue section; transcriptomics.

Other Index Terms

accuracy; Article; cancer survival; cancer tissue; case report; clinical article; controlled study; DNA sequencing; female; gene amplification; gene dosage; gene expression; genetic profile; human; human tissue; laser microdissection; male; microscopy; *molecular fingerprinting; mouth squamous cell carcinoma; *multiomics; overall survival; RNA sequencing; survival time; tissue section; transcriptomics.

Link to the Ovid Full Text or citation:

[Click here for full text options](https://ovidsp.ovid.com/ovidweb.cgi?T=JS&CSC=Y&NEWS=N&PAGE=fulltext&D=emed23&AN=2019399645)

Link to the External Link Resolver:

[SFX](https://sfx-86scu.hosted.exlibrisgroup.com.cn/86scu?sid=OVID:embase&id=pmid:35776767&id=doi:10.1371%2Fjournal.pbio.3001699&issn=15449173&isbn=&volume=20&issue=7&spage=e3001699&pages=&date=2022&title=PLoS+Biology&atitle=Histologically+resolved+multiomics+enables+precise+molecular+profiling+of+human+intratumor+heterogeneity&aulast=Chen&pid=<author>Chen+T.%3BCao+C.%3BZhang+J.%3BStreets+A.%3BLi+T.%3BHuang+Y.<%2Fauthor><AN>2019399645<%2FAN><DT>Article<%2FDT>)

35.

One-step synthesis of core-shell CoP@ N, P co-doped porous carbon sheet + CNTs: Boosting high-rate/long-life lithium storage via triple-carbon synergistic effects.

Lan B., Zhang X., Lu J., Wei C., Wang Y., Wen G.

Colloids and Surfaces A: Physicochemical and Engineering Aspects. 651 (no pagination), 2022. Article Number: 129648. Date of Publication: 20 Oct 2022.

AN: 2019236065

A reasonable structural design is essential to suppress the volume expansion of transition metal phosphides and to enhance their electrochemical performance as anode materials for lithium-ion batteries. Herein, a three-dimensional architecture was formed by the carbon nanotubes (CNTs) connected with nitrogen, phosphorus co-doped porous carbon sheets (NPC), in which core-shell-like carbon-coated CoP nanoparticles were uniformly embedded, synthesized CoP@NPC+CNTs composites with a triple-carbon synergistic cross-linked network structure by a one-step self-templating method. The novel spatial structure effectively suppresses the volume change of CoP, ensures structural stability, and provides a multidimensional pathway for fast ion/electron transport. In addition, the N, P co-doping can synergistically promote the material conductivity and provides plenty of active sites. Profiting from the unique structure, the CoP@NPC+CNTs anode displays excellent lithium storage performance (1074.5 mAh g-1 at 0.1 A g-1 after 280 cycles), superior rate capacities and long cycle stability (701.7 mAh g-1 at 1.0 A g-1 after 1000 cycles). The simple synthesis method and special structure also provide new ideas for preparing other transition metal phosphides.

Copyright © 2022 Elsevier B.V.

Institution

(Lan, Zhang, Lu, Wei, Wang, Wen) School of Materials Science and Engineering, Shandong University of Technology, Zibo 255000, China (Zhang) Shandong Guiyuan Advanced Ceramic Corporation Law, Zibo 255086, China

(Wang) School of Materials Science and Engineering, Harbin Institute of Technology, Harbin 150001, China

Publisher

Elsevier B.V.

Emtree Heading

adsorption; article; carbonization; coating thickness; conductance; cross linking; crystal structure; current density; decomposition; desorption; diffusion; electric conductivity; electrochemical analysis; electron diffraction; hydrogen bond; ion transport; isotherm; lithiation; micromorphology; molecular stability; particle size; phosphorylation; physical phase; pore size distribution; porosity; precursor; pyrolysis; Raman spectrometry; scanning electron microscopy; *storage; surface area; surface property; *synergistic effect; *synthesis; thermogravimetry; volume; *carbon nanotube; *cobalt derivative; *lithium; *phosphorus derivative; anode electrode; electric battery.

Device Index Terms

anode electrode; electric battery.

Drug Index Terms

*carbon nanotube; *cobalt derivative; *lithium; *phosphorus derivative.

Other Index Terms

adsorption; Article; carbonization; coating thickness; conductance; cross linking; crystal structure; current density; decomposition; desorption; diffusion; electric conductivity; electrochemical analysis; electron diffraction; hydrogen bond; ion transport; isotherm; lithiation; micromorphology; molecular stability; particle size; phosphorylation; physical phase; pore size distribution; porosity; precursor; pyrolysis; Raman spectrometry; scanning electron microscopy; *storage; surface area; surface property; *synergistic effect; *synthesis; thermogravimetry; volume.

Link to the Ovid Full Text or citation:

[Click here for full text options](https://ovidsp.ovid.com/ovidweb.cgi?T=JS&CSC=Y&NEWS=N&PAGE=fulltext&D=emed23&AN=2019236065)

Link to the External Link Resolver:

[SFX](https://sfx-86scu.hosted.exlibrisgroup.com.cn/86scu?sid=OVID:embase&id=pmid:&id=doi:10.1016%2Fj.colsurfa.2022.129648&issn=09277757&isbn=&volume=651&issue=&spage=129648&pages=&date=2022&title=Colloids+and+Surfaces+A%3A+Physicochemical+and+Engineering+Aspects&atitle=One-step+synthesis+of+core-shell+CoP@+N%2C+P+co-doped+porous+carbon+sheet+%2B+CNTs%3A+Boosting+high-rate%2Flong-life+lithium+storage+via+triple-carbon+synergistic+effects&aulast=Lan&pid=<author>Lan+B.%3BZhang+X.%3BLu+J.%3BWei+C.%3BWang+Y.%3BWen+G.<%2Fauthor><AN>2019236065<%2FAN><DT>Article<%2FDT>)

36.

Catheter like U-shaped fiber as a probe for oral cancer.

Biswas R.

Biosensors and Bioelectronics: X. 11 (no pagination), 2022. Article Number: 100181. Date of Publication: September 2022.

AN: 2019010232

This short communication reports a unique proposition of a versatile sensing scheme-aimed at early detection of oral cancer. Based on intensity modulation, the probe can render both qualitative as well as quantitative assessments via saliva samples. It can further plausibly affirm the stage of oral cancer in the patient. With remarkable response time and superior specificity due to incorporation of biomarkers, this catheter like probe allows user to get rid of sample storage problem and other allied complications.

Copyright © 2022 The Author(s)

Author NameID

Biswas, Rajib; ORCID: <https://orcid.org/0000-0001-6246-2196>

Institution

(Biswas) Applied Optics and Photonics Lab, Department of Physics, Tezpur University, Tezpur 784028, India

Publisher

Elsevier Ltd

Emtree Heading

article; autofluorescence imaging; *cancer diagnosis; cancer staging; diffuse reflectance spectroscopy; early cancer diagnosis; fluorescence microscopy; human; *mouth cancer/di [Diagnosis]; optical coherence tomography; optical sensor; Raman spectrometry; reaction time; real time reverse transcription polymerase chain reaction; saliva analysis; biological marker/ec [Endogenous Compound]; *fiber optic biosensor.

Device Index Terms

*fiber optic biosensor.

Drug Index Terms

biological marker / endogenous compound.

Other Index Terms

Article; autofluorescence imaging; *cancer diagnosis; cancer staging; diffuse reflectance spectroscopy; early cancer diagnosis; fluorescence microscopy; human; *mouth cancer / *diagnosis; optical coherence tomography; optical sensor; Raman spectrometry; reaction time; real time reverse transcription polymerase chain reaction; saliva analysis.

Link to the Ovid Full Text or citation:

[Click here for full text options](https://ovidsp.ovid.com/ovidweb.cgi?T=JS&CSC=Y&NEWS=N&PAGE=fulltext&D=emed23&AN=2019010232)

Link to the External Link Resolver:

[SFX](https://sfx-86scu.hosted.exlibrisgroup.com.cn/86scu?sid=OVID:embase&id=pmid:&id=doi:10.1016%2Fj.biosx.2022.100181&issn=25901370&isbn=&volume=11&issue=&spage=100181&pages=&date=2022&title=Biosensors+and+Bioelectronics%3A+X&atitle=Catheter+like+U-shaped+fiber+as+a+probe+for+oral+cancer&aulast=Biswas&pid=<author>Biswas+R.<%2Fauthor><AN>2019010232<%2FAN><DT>Article<%2FDT>)

37.

Development of a SERS based cancer diagnosis approach employing cryosectioned thyroid tissue samples on PDMS.

Mert S., Sancak S., Aydin H., Fersahoglu A.T., Somay A., Ozkan F., Culha M.

Nanomedicine: Nanotechnology, Biology, and Medicine. 44 (no pagination), 2022. Article Number: 102577. Date of Publication: August 2022.

AN: 2018897696

An efficient SERS based novel analytical approach named Cryosectioned-PDMS was developed systematically and evaluated applying on 64 thyroid biopsy samples. To utilize thyroid biopsy samples, a 20-mul volume of h-AgNPs suspension was dropped on a 5-mum thick cryosectioned biopsy specimen placed on the PDMS coated glass slide. The SERS spectra from a 10 x 10 points array acquired by mapping 22.5 mum x 22.5 mum sized area from suspended dried droplets placed on the tissue surface. The probability of correctly predicted performance for diagnosis of malignant, benign and healthy tissues was resulted in the accuracy of 100 % for the spectral bands at 667, 724, 920, 960, 1052, 1096, 1315 and 1457 cm-1 using PCA-fed LDA machine learning. The Cryosectioned-PDMS biophotonic approach with PCA-LDA predictive model demonstrated that the vibrational signatures can accurately recognize the fingerprint of cancer pathology from a healthy one with a simple and fast sample preparation methodology.

Copyright © 2022 Elsevier Inc.

PMID

35716872 [<https://www.ncbi.nlm.nih.gov/pubmed/?term=35716872>]

Institution

(Mert) Department of Genetics and Bioengineering, Faculty of Engineering, Yeditepe University, Istanbul 34755, Turkey (Mert) Department of Genetics and Bioengineering, Faculty of Engineering, Istanbul Okan University, Istanbul 34959, Turkey

(Sancak) Department of Internal Medicine, Endocrinology and Metabolism Disorders, Fatih Sultan Mehmet Education and Research Hospital, University of Health Sciences, Istanbul 34752, Turkey

(Aydin) Department of Internal Medicine, Section of Endocrinology and Metabolism, Yeditepe University Hospital, Istanbul 34752, Turkey

(Fersahoglu) General Surgery Clinic, Fatih Sultan Mehmet Education and Research Hospital, University of Health Sciences, Istanbul 34752, Turkey

(Somay) Department of Pathology, Fatih Sultan Mehmet Education and Research Hospital, University of Health Sciences, Istanbul 34752, Turkey

(Ozkan) Department of Pathology, Yeditepe University Hospital, Istanbul 34752, Turkey

(Culha) The Knight Cancer Institute, Cancer Early Detection Advanced Research Center (CEDAR), Oregon Health and Science University, Portland, OR 97239, United States

(Culha) Sabanci University Nanotechnology Research and Application Center (SUNUM), Istanbul, Tuzla 34956, Turkey

(Culha) Department of Chemistry & Physics, Augusta University, Augusta, GA 30912, United States

Publisher

Elsevier Inc.

Emtree Heading

article; *cancer diagnosis; classification algorithm; colloid; controlled study; diagnostic accuracy; discriminant analysis; freeze thawing; frozen section; human; human tissue; leave one out cross validation; machine learning; measurement; predictive model; principal component analysis; reproducibility; sensitivity and specificity; *surface enhanced Raman spectroscopy; *thyroid cancer/di [Diagnosis]; *thyroid gland tissue; *thyroid tumor/di [Diagnosis]; *tumor biopsy; ultraviolet visible spectroscopy; *dimeticone; glass; silastic; silver nanoparticle; silver nitrate; unclassified drug; cryostat; hydroxylamine hydrochloride.

Candidate Terms

hydroxylamine hydrochloride [drug term]

Device Index Terms

cryostat.

Drug Index Terms

*dimeticone; glass; silastic; silver nanoparticle; silver nitrate; unclassified drug.

Other Index Terms

Article; *cancer diagnosis; classification algorithm; colloid; controlled study; diagnostic accuracy; discriminant analysis; freeze thawing; frozen section; human; human tissue; leave one out cross validation; machine learning; measurement; predictive model; principal component analysis; reproducibility; sensitivity and specificity; *surface enhanced Raman spectroscopy; *thyroid cancer / *diagnosis; *thyroid gland tissue; *thyroid tumor / *diagnosis; *tumor biopsy; ultraviolet visible spectroscopy.

Link to the Ovid Full Text or citation:

[Click here for full text options](https://ovidsp.ovid.com/ovidweb.cgi?T=JS&CSC=Y&NEWS=N&PAGE=fulltext&D=emed23&AN=2018897696)

Link to the External Link Resolver:

[SFX](https://sfx-86scu.hosted.exlibrisgroup.com.cn/86scu?sid=OVID:embase&id=pmid:35716872&id=doi:10.1016%2Fj.nano.2022.102577&issn=15499634&isbn=&volume=44&issue=&spage=102577&pages=&date=2022&title=Nanomedicine%3A+Nanotechnology%2C+Biology%2C+and+Medicine&atitle=Development+of+a+SERS+based+cancer+diagnosis+approach+employing+cryosectioned+thyroid+tissue+samples+on+PDMS&aulast=Mert&pid=<author>Mert+S.%3BSancak+S.%3BAydin+H.%3BFersahoglu+A.T.%3BSomay+A.%3BOzkan+F.%3BCulha+M.<%2Fauthor><AN>2018897696<%2FAN><DT>Article<%2FDT>)

38.

Aptamers used for molecular imaging and theranostics - recent developments.

Bohrmann L., Burghardt T., Haynes C., Saatchi K., Hafeli U.O.

Theranostics. 12(9) (pp 4010-4050), 2022. Date of Publication: 2022.

AN: 2018739201

Aptamers are single stranded oligonucleotides that fold into three dimensional structures and are able to recognize a variety of molecular targets. Due to the similarity to antibodies with regards to specificity and affinity and their chemical versatility, aptamers are increasingly used to create targeted probes for in vivo molecular imaging and therapy. Hence, aptamer-based probes have been utilized in practically all major imaging modalities such as nuclear imaging, magnetic resonance imaging, x-ray computed tomography, echography and fluorescence imaging, as well as newer modalities such as surface enhanced Raman spectroscopy. Aside from targeting, aptamers have been used for the creation of sensors that allow the localized detection of cellular markers such as ATP in vivo. This review focuses on in vivo studies of aptamer-based probes for imaging and theranostics since the comprehensive overview by Bouvier-Muller and Duconge in 2018.

Copyright © The author(s)

PMID

35673581 [<https://www.ncbi.nlm.nih.gov/pubmed/?term=35673581>]

Institution

(Bohrmann, Burghardt, Saatchi, Hafeli) Faculty of Pharmaceutical Sciences, University of British Columbia, Vancouver, BC, Canada (Bohrmann, Hafeli) Department of Pharmacy, Faculty of Health and Medical Sciences, University of Copenhagen, Copenhagen, Denmark

(Haynes) Michael Smith Laboratories, University of British Columbia, Vancouver, BC, Canada

Publisher

Ivyspring International Publisher

Emtree Heading

drug delivery system; echography; fluorescence imaging; human; in vivo study; liver cell carcinoma; *molecular imaging; nonhuman; nuclear magnetic resonance imaging; PEGylation; positron emission tomography; review; single photon emission computed tomography; surface enhanced Raman spectroscopy; *theranostic nanomedicine; thyroid papillary carcinoma; tumor microenvironment; x-ray computed tomography; adenosine triphosphate/ec [Endogenous Compound]; antibody drug conjugate; *aptamer; nanomaterial.

Drug Index Terms

adenosine triphosphate / endogenous compound; antibody drug conjugate; *aptamer; nanomaterial.

Other Index Terms

drug delivery system; echography; fluorescence imaging; human; in vivo study; liver cell carcinoma; *molecular imaging; nonhuman; nuclear magnetic resonance imaging; PEGylation; positron emission tomography; Review; single photon emission computed tomography; surface enhanced Raman spectroscopy; *theranostic nanomedicine; thyroid papillary carcinoma; tumor microenvironment; x-ray computed tomography.

Link to the Ovid Full Text or citation:

[Click here for full text options](https://ovidsp.ovid.com/ovidweb.cgi?T=JS&CSC=Y&NEWS=N&PAGE=fulltext&D=emed23&AN=2018739201)

Link to the External Link Resolver:

[SFX](https://sfx-86scu.hosted.exlibrisgroup.com.cn/86scu?sid=OVID:embase&id=pmid:35673581&id=doi:10.7150%2FTHNO.72949&issn=18387640&isbn=&volume=12&issue=9&spage=4010&pages=4010-4050&date=2022&title=Theranostics&atitle=Aptamers+used+for+molecular+imaging+and+theranostics+-+recent+developments&aulast=Bohrmann&pid=<author>Bohrmann+L.%3BBurghardt+T.%3BHaynes+C.%3BSaatchi+K.%3BHafeli+U.O.<%2Fauthor><AN>2018739201<%2FAN><DT>Review<%2FDT>)

39.

Encapsulation and controlled release of fragrances from MIL-101(Fe)-based recyclable magnetic nanoporous carbon.

Wu C.-J., Liu Y.-F., Zhang W.-F., Zhang C., Chai G.-B., Zhang Q.-D., Mao J., Ahmad I., Zhang S.-S., Xie J.-P.

Colloids and Surfaces A: Physicochemical and Engineering Aspects. 640 (no pagination), 2022. Article Number: 128453. Date of Publication: 05 May 2022.

AN: 2016765006

In this work, MIL-101(Fe), a biocompatible metal-organic framework (MOFs), was calcinated to obtain high-performance nanoporous carbon (Fe-MNPC). The carbonized product was selected as a promising candidate adsorbent for the controlled release of fragrances. Isobutyraldehyde (BA), ethyl acetate (EA), benzaldehyde (BAD), and methoxybenzene (MB) of high saturated vapor pressures were selected as the representative fragrances for inclusion and controlled release studies. The prepared Fe-MNPC featured a large specific surface area (SSA) of 121.46 m2 g-1, which provided feasible storage and controlled-release ability. The high stability and magnetic property of Fe-MNPC guaranteed fast collection and efficient recyclability. The effective loading amounts of BA are 0.22 and EA are 0.16 g g-1, respectively, while that of BAD and MB can reach 0.56 and 0.63 g g-1, respectively. Fe-MNPC exhibited a much longer release effect over BA and EA than BAD and MB, suggesting an excellent adsorption-release selectivity of Fe-MNPC to different fragrances. This study sets an example for the usability of MOF-derived nanoporous carbon (NPC) in fragrance release and enriches the application of NPC in volatile release.

Copyright © 2022 Elsevier B.V.

Institution

(Wu, Liu, Zhang) College of Chemistry, Zhengzhou University, Kexue Avenue 100, Zhengzhou, Henan 450001, China (Chai, Zhang, Mao, Xie) Zhengzhou Tobacco Research Institute of CNTC, Fengyang Road, Zhengzhou, Henan 450001, China

(Chai, Zhang, Mao, Xie) Henan University of Chinese Medicine, Zhengzhou 450008, China

(Wu, Chai, Zhang, Mao, Xie) Research Center of Flavor Science, Zhengzhou University, Kexue Avenue 100, Zhengzhou, Henan 450001, China

(Zhang, Ahmad, Zhang) Center of Advanced Analysis and Gene Sequencing, Key Laboratory of Molecular Sensing and Harmful Substances Detection Technology, Zhengzhou University, Kexue Avenue 100, Zhengzhou, Henan 450001, China

(Zhang, Ahmad, Zhang) Green Catalysis Center and College of Chemistry, Zhengzhou University, Zhengzhou 450001, China

Publisher

Elsevier B.V.

Emtree Heading

adsorption; article; biocompatibility; chemical composition; community structure; controlled study; crystal structure; drug delivery system; electrochemical analysis; elemental analysis; emulsion; *encapsulation; energy dispersive X ray spectroscopy; fungal gene; high performance liquid chromatography; human; hydrogen bond; infrared spectroscopy; isotherm; methanogenesis; particle size; photon correlation spectroscopy; pyrolysis; Raman spectrometry; scanning electron microscopy; scientific literature; solid phase microextraction; surface area; surface property; sustained release preparation; thermogravimetry; transmission electron microscopy; usability; vapor pressure; volatilization; acetic acid ethyl ester; adsorbent; anisole; benzaldehyde; *carbon; fragrance; iron; iron oxide; isobutyraldehyde; *magnetic nanoparticle; metabotropic receptor; metal organic framework; n,n dimethylformamide; nanocomposite; nanoparticle.

Drug Index Terms

acetic acid ethyl ester; adsorbent; anisole; benzaldehyde; *carbon; fragrance; iron; iron oxide; isobutyraldehyde; *magnetic nanoparticle; metabotropic receptor; metal organic framework; n,n dimethylformamide; nanocomposite; nanoparticle.

Other Index Terms

adsorption; Article; biocompatibility; chemical composition; community structure; controlled study; crystal structure; drug delivery system; electrochemical analysis; elemental analysis; emulsion; *encapsulation; energy dispersive X ray spectroscopy; fungal gene; high performance liquid chromatography; human; hydrogen bond; infrared spectroscopy; isotherm; methanogenesis; particle size; photon correlation spectroscopy; pyrolysis; Raman spectrometry; scanning electron microscopy; scientific literature; solid phase microextraction; surface area; surface property; sustained release preparation; thermogravimetry; transmission electron microscopy; usability; vapor pressure; volatilization.

Link to the Ovid Full Text or citation:

[Click here for full text options](https://ovidsp.ovid.com/ovidweb.cgi?T=JS&CSC=Y&NEWS=N&PAGE=fulltext&D=emed23&AN=2016765006)

Link to the External Link Resolver:

[SFX](https://sfx-86scu.hosted.exlibrisgroup.com.cn/86scu?sid=OVID:embase&id=pmid:&id=doi:10.1016%2Fj.colsurfa.2022.128453&issn=09277757&isbn=&volume=640&issue=&spage=128453&pages=&date=2022&title=Colloids+and+Surfaces+A%3A+Physicochemical+and+Engineering+Aspects&atitle=Encapsulation+and+controlled+release+of+fragrances+from+MIL-101(Fe)-based+recyclable+magnetic+nanoporous+carbon&aulast=Wu&pid=<author>Wu+C.-J.%3BLiu+Y.-F.%3BZhang+W.-F.%3BZhang+C.%3BChai+G.-B.%3BZhang+Q.-D.%3BMao+J.%3BAhmad+I.%3BZhang+S.-S.%3BXie+J.-P.<%2Fauthor><AN>2016765006<%2FAN><DT>Article<%2FDT>)

40.

Phase separation of FG-nucleoporins in nuclear pore complexes.

Nag N., Sasidharan S., Uversky V.N., Saudagar P., Tripathi T.

Biochimica et Biophysica Acta - Molecular Cell Research. 1869(4) (no pagination), 2022. Article Number: 119205. Date of Publication: April 2022.

AN: 2016359870

The nuclear envelope (NE) is a bilayer membrane that separates and physically isolates the genetic material from the cytoplasm. Nuclear pore complexes (NPCs) are cylindrical structures embedded in the NE and remain the sole channel of communication between the nucleus and the cytoplasm. The interior of NPCs contains densely packed intrinsically disordered FG-nucleoporins (FG-Nups), consequently forming a permeability barrier. This barrier facilitates the selection and specificity of the cargoes that are imported, exported, or shuttled through the NPCs. Recent studies have revealed that FG-Nups undergo the process of liquid-liquid phase separation into liquid droplets. Moreover, these liquid droplets mimic the permeability barrier observed in the interior of NPCs. This review highlights the phase separation of FG-Nups occurring inside the NPCs rooted in the NE. We discuss the phase separation of FG-Nups and compare the different aspects contributing to their phase separation. Furthermore, several diseases caused by the aberrant phase separation of the proteins are examined with respect to NEs. By understanding the fundamental process of phase separation at the nuclear membrane, the review seeks to explore the parameters influencing this phenomenon as well as its importance, ultimately paving the way for better research on the structure-function relationship of biomolecular condensates.

Copyright © 2022 Elsevier B.V.

PMID

34995711 [<https://www.ncbi.nlm.nih.gov/pubmed/?term=34995711>]

Institution

(Nag, Tripathi) Molecular and Structural Biophysics Laboratory, Department of Biochemistry, North-Eastern Hill University, Shillong 793022, India (Sasidharan, Saudagar) Department of Biotechnology, National Institute of Technology Warangal, Warangal 506004, India

(Uversky) Department of Molecular Medicine and Byrd Alzheimer's Research Institute, Morsani College of Medicine, University of South Florida, Tampa, FL 33620, United States

(Uversky) Center for Molecular Mechanisms of Aging and Age-Related Diseases, Moscow Institute of Physics and Technology, Institutskiy pereulok, 9, Dolgoprudny, Moscow Region 141700, Russian Federation

Publisher

Elsevier B.V.

Emtree Heading

acute myeloid leukemia; Alzheimer disease; amyotrophic lateral sclerosis; analytic method; article; artificial neural network; atomic force microscopy; carcinogenesis; cell nucleus membrane; chemical phenomena; chronic myeloid leukemia; circular dichroism; deep neural network; differential scanning calorimetry; Drosophila melanogaster; electron microscopy; electron spin resonance; fluorescence correlation spectroscopy; fluorescence microscopy; fluorescence recovery after photobleaching; fluorescence resonance energy transfer; frontotemporal dementia; gene mutation; gene sequence; human; Huntington chorea; infrared spectroscopy; mitosis; molecular dynamics; molecular weight; Monte Carlo method; neutron scattering; nonhuman; nuclear export signal; nuclear localization signal; nuclear magnetic resonance spectroscopy; *nuclear pore complex; nucleocytoplasmic transport; Parkinson disease; *phase separation; prion disease; protein structure; radiation scattering; Raman spectrometry; signal transduction; static electricity; stomach carcinoma; support vector machine; thyroid papillary carcinoma; ultracentrifugation; Xenopus laevis; alpha synuclein/ec [Endogenous Compound]; cyclic GMP dependent protein kinase/ec [Endogenous Compound]; heterogeneous nuclear ribonucleoprotein A1/ec [Endogenous Compound]; hydrogel; membrane protein/ec [Endogenous Compound]; *nucleoporin/ec [Endogenous Compound]; RNA binding protein FUS/ec [Endogenous Compound]; T lymphocyte receptor/ec [Endogenous Compound]; TAR DNA binding protein/ec [Endogenous Compound]; tau protein/ec [Endogenous Compound]; thioflavine; unclassified drug; liquid liquid phase separation; static light scattering; cGAS protein/ec [Endogenous Compound]; *fg nucleoporin/ec [Endogenous Compound]; ntrk1 protein/ec [Endogenous Compound].

Candidate Terms

liquid liquid phase separation [other term] static light scattering [other term]

cGAS protein / endogenous compound [drug term]

*fg nucleoporin / *endogenous compound [drug term]

NTrk1 protein / endogenous compound [drug term]

Drug Index Terms

alpha synuclein / endogenous compound; cyclic GMP dependent protein kinase / endogenous compound; heterogeneous nuclear ribonucleoprotein A1 / endogenous compound; hydrogel; membrane protein / endogenous compound; *nucleoporin / *endogenous compound; RNA binding protein FUS / endogenous compound; T lymphocyte receptor / endogenous compound; TAR DNA binding protein / endogenous compound; tau protein / endogenous compound; thioflavine; unclassified drug.

Other Index Terms

acute myeloid leukemia; Alzheimer disease; amyotrophic lateral sclerosis; analytic method; Article; artificial neural network; atomic force microscopy; carcinogenesis; cell nucleus membrane; chemical phenomena; chronic myeloid leukemia; circular dichroism; deep neural network; differential scanning calorimetry; Drosophila melanogaster; electron microscopy; electron spin resonance; fluorescence correlation spectroscopy; fluorescence microscopy; fluorescence recovery after photobleaching; fluorescence resonance energy transfer; frontotemporal dementia; gene mutation; gene sequence; human; Huntington chorea; infrared spectroscopy; mitosis; molecular dynamics; molecular weight; Monte Carlo method; neutron scattering; nonhuman; nuclear export signal; nuclear localization signal; nuclear magnetic resonance spectroscopy; *nuclear pore complex; nucleocytoplasmic transport; Parkinson disease; *phase separation; prion disease; protein structure; radiation scattering; Raman spectrometry; signal transduction; static electricity; stomach carcinoma; support vector machine; thyroid papillary carcinoma; ultracentrifugation; Xenopus laevis.

Link to the Ovid Full Text or citation:

[Click here for full text options](https://ovidsp.ovid.com/ovidweb.cgi?T=JS&CSC=Y&NEWS=N&PAGE=fulltext&D=emed23&AN=2016359870)

Link to the External Link Resolver:

[SFX](https://sfx-86scu.hosted.exlibrisgroup.com.cn/86scu?sid=OVID:embase&id=pmid:34995711&id=doi:10.1016%2Fj.bbamcr.2021.119205&issn=01674889&isbn=&volume=1869&issue=4&spage=119205&pages=&date=2022&title=Biochimica+et+Biophysica+Acta+-+Molecular+Cell+Research&atitle=Phase+separation+of+FG-nucleoporins+in+nuclear+pore+complexes&aulast=Nag&pid=<author>Nag+N.%3BSasidharan+S.%3BUversky+V.N.%3BSaudagar+P.%3BTripathi+T.<%2Fauthor><AN>2016359870<%2FAN><DT>Article<%2FDT>)

41.

Rapid identification of papillary thyroid carcinoma and papillary microcarcinoma based on serum Raman spectroscopy combined with machine learning models.

Song H., Dong C., Zhang X., Wu W., Ma B., Chen F., Chen C., Lv X.

Photodiagnosis and Photodynamic Therapy. 37 (no pagination), 2022. Article Number: 102647. Date of Publication: March 2022.

AN: 2015782587

Thyroid carcinoma is with the highest diagnosis rate in the endocrine system, and its main histological subtype is papillary thyroid carcinoma (PTC) accounting for 80% of thyroid malignancies. In recent years, the incidence of thyroid cancer has increased exponentially, and its substantial increase was closely related to the overdiagnosis of papillary microcarcinoma (PMC). Therefore, early and accurate identification of PTC and PMC can prevent patients from over treatment. This study aimed to identify PTC and PMC using Raman spectroscopy. We collected serum Raman spectra from 16 patients with PTC and 31 patients with PMC. Firstly, the collected imbalance data were preprocessed using the synthetic minority over-sampling technique (SMOTE). Then, the equalized data were dimensionality reduced by principal component analysis (PCA). Finally, the processed data were fed into the single decision tree (DT) classifier, as well as the random forest (RF) built on the idea of Boosting ensemble and the Adaptive Boosting (Adaboost) model built on the idea of Bagging ensemble for classification. The classification accuracy of the three models in the testing set were 75.38%, 81.54%, and 84.61%, respectively. Compared with the DT classifier, the accuracy of the models introducing the idea of ensemble learning was enhanced by 6.16% and 9.23%, respectively. The best model was the Adaboost. This result demonstrates that serum Raman spectroscopy combined with an ensemble learning algorithm was feasible in rapidly identifying PTC and PMC. At the same time, the method has great potential for application in the field of clinical diagnosis.

Copyright © 2021 Elsevier B.V.

PMID

34818598 [<https://www.ncbi.nlm.nih.gov/pubmed/?term=34818598>]

Institution

(Song, Dong, Ma) Department of Breast, Head and Neck Surgery, Xinjiang Medical University Affiliated Tumor Hospital, Urumqi, China (Wu, Chen, Lv) College of Software, Xinjiang University, Urumqi 830046, China

(Chen, Lv) Key Laboratory of Signal Detection and Processing, Xinjiang University, Urumqi 830046, China

(Zhang, Chen, Chen, Lv) College of Information Science and Engineering, Xinjiang University, Urumqi 830046, China

Publisher

Elsevier B.V.

Emtree Heading

adult; article; blood sampling; classifier; clinical article; controlled study; cross validation; decision tree; follow up; human; learning algorithm; *machine learning; performance indicator; prediction; principal component analysis; *Raman spectrometry; *serum; *thyroid papillary carcinoma/di [Diagnosis]; spectrometer; SR-510 Pro.

Candidate Terms

SR-510 Pro [device term]

Device Index Terms

spectrometer.

Other Index Terms

adult; Article; blood sampling; classifier; clinical article; controlled study; cross validation; decision tree; follow up; human; learning algorithm; *machine learning; performance indicator; prediction; principal component analysis; *Raman spectrometry; *serum; *thyroid papillary carcinoma / *diagnosis.

Link to the Ovid Full Text or citation:

[Click here for full text options](https://ovidsp.ovid.com/ovidweb.cgi?T=JS&CSC=Y&NEWS=N&PAGE=fulltext&D=emed23&AN=2015782587)

Link to the External Link Resolver:

[SFX](https://sfx-86scu.hosted.exlibrisgroup.com.cn/86scu?sid=OVID:embase&id=pmid:34818598&id=doi:10.1016%2Fj.pdpdt.2021.102647&issn=15721000&isbn=&volume=37&issue=&spage=102647&pages=&date=2022&title=Photodiagnosis+and+Photodynamic+Therapy&atitle=Rapid+identification+of+papillary+thyroid+carcinoma+and+papillary+microcarcinoma+based+on+serum+Raman+spectroscopy+combined+with+machine+learning+models&aulast=Song&pid=<author>Song+H.%3BDong+C.%3BZhang+X.%3BWu+W.%3BMa+B.%3BChen+F.%3BChen+C.%3BLv+X.<%2Fauthor><AN>2015782587<%2FAN><DT>Article<%2FDT>)

42.

Advanced CT and MR Imaging of the Posttreatment Head and Neck.

Lobo R., Turk S., Bapuraj J.R., Srinivasan A.

Neuroimaging Clinics of North America. 32(1) (pp 133-144), 2022. Date of Publication: February 2022.

AN: 2015669813

PMID

34809834 [<https://www.ncbi.nlm.nih.gov/pubmed/?term=34809834>]

Institution

(Lobo, Turk) Neuroradiology Division, Radiology, Michigan Medicine, 1500 E Medical Center Drive, Ann Arbor, MI 48109, United States (Bapuraj, Srinivasan) Neuroradiology Division, Radiology, Michigan Medicine, 1500 E Medical Center Drive, B2A209, Ann Arbor, MI 48109, United States

Publisher

W.B. Saunders

Emtree Heading

apparent diffusion coefficient; arterial spin labeling; BOLD signal; cancer free survival; cancer prognosis; cancer recurrence; cancer regression; cancer staging; capillary permeability; chemoradiotherapy; *computer assisted tomography; diagnostic accuracy; diffusion weighted imaging; dynamic contrast-enhanced magnetic resonance imaging; gross tumor volume; *head and neck squamous cell carcinoma/di [Diagnosis]; *head and neck squamous cell carcinoma/dt [Drug Therapy]; *head and neck squamous cell carcinoma/rt [Radiotherapy]; human; image analysis; lymph node metastasis/di [Diagnosis]; magnetic field; *nuclear magnetic resonance imaging; overall survival; predictive value; progression free survival; proton nuclear magnetic resonance; radiation dose; Raman spectrometry; receiver operating characteristic; review; sensitivity and specificity; treatment response; tumor blood flow; antineoplastic agent/dt [Drug Therapy]; antineoplastic agent/tm [Unexpected Outcome of Drug Treatment]; gadolinium.

Drug Index Terms

antineoplastic agent / drug therapy / unexpected outcome of drug treatment; gadolinium.

Other Index Terms

apparent diffusion coefficient; arterial spin labeling; BOLD signal; cancer free survival; cancer prognosis; cancer recurrence; cancer regression; cancer staging; capillary permeability; chemoradiotherapy; *computer assisted tomography; diagnostic accuracy; diffusion weighted imaging; dynamic contrast-enhanced magnetic resonance imaging; gross tumor volume; *head and neck squamous cell carcinoma / *diagnosis / *drug therapy / *radiotherapy; human; image analysis; lymph node metastasis / diagnosis; magnetic field; *nuclear magnetic resonance imaging; overall survival; predictive value; progression free survival; proton nuclear magnetic resonance; radiation dose; Raman spectrometry; receiver operating characteristic; Review; sensitivity and specificity; treatment response; tumor blood flow.

Link to the Ovid Full Text or citation:

[Click here for full text options](https://ovidsp.ovid.com/ovidweb.cgi?T=JS&CSC=Y&NEWS=N&PAGE=fulltext&D=emed23&AN=2015669813)

Link to the External Link Resolver:

[SFX](https://sfx-86scu.hosted.exlibrisgroup.com.cn/86scu?sid=OVID:embase&id=pmid:34809834&id=doi:10.1016%2Fj.nic.2021.08.013&issn=10525149&isbn=&volume=32&issue=1&spage=133&pages=133-144&date=2022&title=Neuroimaging+Clinics+of+North+America&atitle=Advanced+CT+and+MR+Imaging+of+the+Posttreatment+Head+and+Neck&aulast=Lobo&pid=<author>Lobo+R.%3BTurk+S.%3BBapuraj+J.R.%3BSrinivasan+A.<%2Fauthor><AN>2015669813<%2FAN><DT>Review<%2FDT>)

43.

Deep learning-based image processing in optical microscopy.

Melanthota S.K., Gopal D., Chakrabarti S., Kashyap A.A., Radhakrishnan R., Mazumder N.

Biophysical Reviews. 14(2) (pp 463-481), 2022. Date of Publication: April 2022.

AN: 2015559123

Optical microscopy has emerged as a key driver of fundamental research since it provides the ability to probe into imperceptible structures in the biomedical world. For the detailed investigation of samples, a high-resolution image with enhanced contrast and minimal damage is preferred. To achieve this, an automated image analysis method is preferable over manual analysis in terms of both speed of acquisition and reduced error accumulation. In this regard, deep learning (DL)-based image processing can be highly beneficial. The review summarises and critiques the use of DL in image processing for the data collected using various optical microscopic techniques. In tandem with optical microscopy, DL has already found applications in various problems related to image classification and segmentation. It has also performed well in enhancing image resolution in smartphone-based microscopy, which in turn enablse crucial medical assistance in remote places. Graphical abstract: [Figure not available: see fulltext.].

Copyright © 2022, The Author(s).

Author NameID

Mazumder, Nirmal; ORCID: <https://orcid.org/0000-0001-8068-6484>

Institution

(Melanthota, Mazumder) Department of Biophysics, Manipal School of Life Sciences, Manipal Academy of Higher Education, Karnataka, Manipal 576104, India (Gopal, Chakrabarti) Department of Bioinformatics, Manipal School of Life Sciences, Manipal Academy of Higher Education, Karnataka, Manipal 576104, India

(Kashyap) Computer Science and Engineering, Manipal Institute of Technology, Manipal Academy of Higher Education, Karnataka, Manipal 576104, India

(Radhakrishnan) Department of Oral Pathology, Manipal College of Dental Sciences, Manipal, Manipal Academy of Higher Education, Manipal 576104, India

Publisher

Springer Science and Business Media Deutschland GmbH

Emtree Heading

adenocarcinoma; artificial neural network; bright field microscopy; cell nucleus; cell vacuole; convolutional neural network; cytoplasm; *deep learning; diagnostic accuracy; endoplasmic reticulum; endosome; fluorescence imaging; fluorescence microscopy; Golgi complex; health care system; human; hyperkeratosis; image analysis; *image processing; image quality; keratosis; learning algorithm; light-sheet microscopy; machine learning; microfluidics; *microscopy; mitochondrion; mouth cancer; multiphoton microscopy; non small cell lung cancer; nucleolus; phase contrast microscopy; predictive value; Raman spectrometry; receiver operating characteristic; review; squamous cell carcinoma; actin; eosin; hematoxylin; microsphere; medical device; smartphone; quantitative phase microscopy; Raman microscopy; oral cancer screening device.

Candidate Terms

quantitative phase microscopy [other term] raman microscopy [other term]

oral cancer screening device [device term]

Device Index Terms

medical device; smartphone.

Drug Index Terms

actin; eosin; hematoxylin; microsphere.

Other Index Terms

adenocarcinoma; artificial neural network; bright field microscopy; cell nucleus; cell vacuole; convolutional neural network; cytoplasm; *deep learning; diagnostic accuracy; endoplasmic reticulum; endosome; fluorescence imaging; fluorescence microscopy; Golgi complex; health care system; human; hyperkeratosis; image analysis; *image processing; image quality; keratosis; learning algorithm; light-sheet microscopy; machine learning; microfluidics; *microscopy; mitochondrion; mouth cancer; multiphoton microscopy; non small cell lung cancer; nucleolus; phase contrast microscopy; predictive value; Raman spectrometry; receiver operating characteristic; Review; squamous cell carcinoma.

Link to the Ovid Full Text or citation:

[Click here for full text options](https://ovidsp.ovid.com/ovidweb.cgi?T=JS&CSC=Y&NEWS=N&PAGE=fulltext&D=emed23&AN=2015559123)

Link to the External Link Resolver:

[SFX](https://sfx-86scu.hosted.exlibrisgroup.com.cn/86scu?sid=OVID:embase&id=pmid:&id=doi:10.1007%2Fs12551-022-00949-3&issn=18672450&isbn=&volume=14&issue=2&spage=463&pages=463-481&date=2022&title=Biophysical+Reviews&atitle=Deep+learning-based+image+processing+in+optical+microscopy&aulast=Melanthota&pid=<author>Melanthota+S.K.%3BGopal+D.%3BChakrabarti+S.%3BKashyap+A.A.%3BRadhakrishnan+R.%3BMazumder+N.<%2Fauthor><AN>2015559123<%2FAN><DT>Review<%2FDT>)

44.

Surgical tumour margins and their significance in oral squamous cell carcinoma.

Brennan P.A., Dylgjeri F., Coletta R.D., Arakeri G., Goodson A.M.

Journal of Oral Pathology and Medicine. 51(4) (pp 311-314), 2022. Date of Publication: April 2022.

AN: 2015046814

There are many prognostic indicators used to predict tumour recurrence and overall prognosis in oral squamous cell carcinoma (OSCC). Most of these biological factors cannot be directly influenced by clinicians managing these heterogeneous group of tumours. Excision margins can potentially be increased at the time of surgery by including more normal tissue than the commonly accepted 1 cm resection distance from the macroscopic tumour edge. However, this can lead to poorer quality of life for patients and does not necessarily address microscopic extensions or dicohesive patterns of tumour growth. Surgical margins can be affected by tissue shrinkage immediately following resection and the choice of instrument used for surgery. Currently, most regard a clear resection margin as being >5 mm, a close margin as more than 1 mm but <5 mm and an involved margin <1 mm. In this article, we provide a brief overview of tumour margins in OSCC, including several recently published large meta-analyses. Based upon these and other studies, there is still conflicting data in the literature about the ideal margin for OSCC. There is a growing body of evidence that suggests a clearance of 1 mm might be adequate for some cancers. However, adequacy of resection should be considered along with the many other prognostic indicators of OSCC when the multi-disciplinary team considers further treatment for these patients.

Copyright © 2022 John Wiley & Sons A/S. Published by John Wiley & Sons Ltd.

PMID

35080080 [<https://www.ncbi.nlm.nih.gov/pubmed/?term=35080080>]

Institution

(Brennan, Dylgjeri, Goodson) Department of Oral and Maxillofacial Surgery, Queen Alexandra Hospital, Portsmouth, United Kingdom (Coletta) Graduate Program in Oral Biology, School of Dentistry, University of Campinas, Sao Paulo, Piracicaba, Brazil

(Coletta) Department of Oral Diagnosis, School of Dentistry, University of Campinas, Sao Paulo, Piracicaba, Brazil

(Arakeri) Department of Head and Neck Oncology, Centre for Academic Research, HCG Cancer Center, Karnataka, Bengaluru, India

(Arakeri) Department of Oral and Maxillofacial Surgery, Navodaya Dental College and Hospital, Karnataka, Raichur, India

Publisher

John Wiley and Sons Inc

Emtree Heading

adjuvant therapy; bone resection; cancer prognosis; *cancer surgery; confocal microscopy; depth of invasion; disease specific survival; female; free tissue graft; human; male; meta analysis; *mouth squamous cell carcinoma/di [Diagnosis]; *mouth squamous cell carcinoma/su [Surgery]; multidisciplinary team; optical coherence tomography; overall survival; quality of life; Raman spectrometry; review; *surgical margin; systematic review; tumor growth; tumor recurrence.

Other Index Terms

adjuvant therapy; bone resection; cancer prognosis; *cancer surgery; confocal microscopy; depth of invasion; disease specific survival; female; free tissue graft; human; male; meta analysis; *mouth squamous cell carcinoma / *diagnosis / *surgery; multidisciplinary team; optical coherence tomography; overall survival; quality of life; Raman spectrometry; Review; *surgical margin; systematic review; tumor growth; tumor recurrence.

Link to the Ovid Full Text or citation:

[Click here for full text options](https://ovidsp.ovid.com/ovidweb.cgi?T=JS&CSC=Y&NEWS=N&PAGE=fulltext&D=emed23&AN=2015046814)

Link to the External Link Resolver:

[SFX](https://sfx-86scu.hosted.exlibrisgroup.com.cn/86scu?sid=OVID:embase&id=pmid:35080080&id=doi:10.1111%2Fjop.13276&issn=09042512&isbn=&volume=51&issue=4&spage=311&pages=311-314&date=2022&title=Journal+of+Oral+Pathology+and+Medicine&atitle=Surgical+tumour+margins+and+their+significance+in+oral+squamous+cell+carcinoma&aulast=Brennan&pid=<author>Brennan+P.A.%3BDylgjeri+F.%3BColetta+R.D.%3BArakeri+G.%3BGoodson+A.M.<%2Fauthor><AN>2015046814<%2FAN><DT>Review<%2FDT>)

45.

Deep reinforced neural network model for cyto-spectroscopic analysis of epigenetic markers for automated oral cancer risk prediction.

Ghosh A., Chaudhuri D., Adhikary S., Chatterjee K., Roychowdhury A., Das A.K., Barui A.

Chemometrics and Intelligent Laboratory Systems. 224 (no pagination), 2022. Article Number: 104548. Date of Publication: 15 May 2022.

AN: 2017425894

Understanding epigenetic changes can provide vital information for early stage oral cancer diagnosis. Vibrational spectroscopy methods like Raman spectroscopy (RS) and Fourier Transform Infrared Spectroscopy (FTIR) can provide several advantages over conventional molecular biology methods, by incorporating information from fingerprint regions. Moreover, application of advanced spectral analysis tools like deep learning (DL) techniques can be efficiently applied for analyzing the large spectral dataset and extracting the vital features. Epigenetic changes are identifies in oral epithelial cells of healthy individuals, oral leucoplakia and squamous cell carcinoma patients through analysis of Raman (400-1800 cm-1) and FTIR data (700-2000 cm-1). Deep reinforced neural network (DRNN) model is employed to classify the epigenetic changes identified from the Raman and FTIR spectra. Feature extraction layer of DL model uses peak detection layer and reinforced learning layer to identify significant epigenetic features. Classification layer is made up of N numbers of back propagated Artificial Neural Network (ANN) layers. DL model developed is fully automated and overcome the wave shift problem of spectroscopic data. Testing accuracy of the proposed DRNN model is 83.33%. Class wise accuracies for NRML, OLPK and OSCC are 83.3%, 87% and 95.24%, respectively. Proposed DRNN model attains an overall ROC of 0.88 Present study employs combination of two complementary vibrational spectroscopy methods for oral cancer detection and chemometric analysis of the spectral features with DRNN mode. Identification of the epigenetic changes and utilization of the knowledge in cancer prediction will enable the proposed study to develop smart point-of-care diagnostic system.

Copyright © 2022 Elsevier B.V.

Author NameID

Barui, Ananya; ORCID: <https://orcid.org/0000-0001-9710-9448>

Institution

(Ghosh, Chaudhuri, Adhikary, Barui) Centre for Healthcare Science and Technology, Indian Institute of Engineering, Science and Technology, Shibpur, West-Bengal, Howrah 711103, India (Chatterjee) Department of Oral and Maxillofacial Pathology, Buddha Institute of Dental Sciences, West TV Tower, Gandhinagar, Kankarabagh, Bihar, Patna 800020, India

(Roychowdhury) Department of Applied Mechanics, Indian Institute of Engineering, Science and Technology, Shibpur, West-Bengal, Howrah 711103, India

(Das) Department of Computer Science and Technology, Indian Institute of Engineering, Science and Technology, Shibpur, West-Bengal, Howrah 711103, India

Publisher

Elsevier B.V.

Emtree Heading

article; *cancer risk; cancer staging; chemometrics; clinical article; controlled study; *cytophotometry; deep learning; *deep neural network; diagnostic accuracy; diagnostic test accuracy study; discriminant analysis; *epigenetics; epithelium cell; Fourier transform infrared spectroscopy; histopathology; human; human tissue; *mouth cancer/di [Diagnosis]; mouth squamous cell carcinoma/di [Diagnosis]; oral leukoplakia/di [Diagnosis]; predictive value; Raman spectrometry; receiver operating characteristic; *risk assessment; sensitivity and specificity; signal noise ratio; validation process; vibrational spectroscopy.

Other Index Terms

Article; *cancer risk; cancer staging; chemometrics; clinical article; controlled study; *cytophotometry; deep learning; *deep neural network; diagnostic accuracy; diagnostic test accuracy study; discriminant analysis; *epigenetics; epithelium cell; Fourier transform infrared spectroscopy; histopathology; human; human tissue; *mouth cancer / *diagnosis; mouth squamous cell carcinoma / diagnosis; oral leukoplakia / diagnosis; predictive value; Raman spectrometry; receiver operating characteristic; *risk assessment; sensitivity and specificity; signal noise ratio; validation process; vibrational spectroscopy.

Link to the Ovid Full Text or citation:

[Click here for full text options](https://ovidsp.ovid.com/ovidweb.cgi?T=JS&CSC=Y&NEWS=N&PAGE=fulltext&D=emed23&AN=2017425894)

Link to the External Link Resolver:

[SFX](https://sfx-86scu.hosted.exlibrisgroup.com.cn/86scu?sid=OVID:embase&id=pmid:&id=doi:10.1016%2Fj.chemolab.2022.104548&issn=01697439&isbn=&volume=224&issue=&spage=104548&pages=&date=2022&title=Chemometrics+and+Intelligent+Laboratory+Systems&atitle=Deep+reinforced+neural+network+model+for+cyto-spectroscopic+analysis+of+epigenetic+markers+for+automated+oral+cancer+risk+prediction&aulast=Ghosh&pid=<author>Ghosh+A.%3BChaudhuri+D.%3BAdhikary+S.%3BChatterjee+K.%3BRoychowdhury+A.%3BDas+A.K.%3BBarui+A.<%2Fauthor><AN>2017425894<%2FAN><DT>Article<%2FDT>)

46.

Unmet Needs and Perspectives in Oral Cancer Prevention.

Bouaoud J., Bossi P., Elkabets M., Schmitz S., van Kempen L.C., Martinez P., Jagadeeshan S., Breuskin I., Puppels G.J., Hoffmann C., Hunter K.D., Simon C., Machiels J.-P., Gregoire V., Bertolus C., Brakenhoff R.H., Koljenovic S., Saintigny P.

Cancers. 14(7) (no pagination), 2022. Article Number: 1815. Date of Publication: April-1 2022.

AN: 2016116004

Oral potentially malignant disorders (OPMD) may precede oral squamous cell carcinoma (OSCC). Reported rates of malignant transformation of OPMD range from 3 to 50%. While some clinical, histological, and molecular factors have been associated with a high-risk OPMD, they are, to date, insufficiently accurate for treatment decision-making. Moreover, this range highlights differences in the clinical definition of OPMD, variation in follow-up periods, and molecular and biological heterogeneity of OPMD. Finally, while treatment of OPMD may improve outcome, standard therapy has been shown to be ineffective to prevent OSCC development in patients with OPMD. In this perspective paper, several experts discuss the main challenges in oral cancer prevention, in particular the need to (i) to define an OPMD classification system by integrating new pathological and molecular characteristics, aiming (ii) to better identify OPMD at high risk of malignant transformation, and (iii) to develop treatment strategies to eradicate OPMD or prevent malignant transformation.

Copyright © 2022 by the authors. Licensee MDPI, Basel, Switzerland.

Institution

(Bouaoud, Martinez, Saintigny) Centre de Recherche en Cancerologie de Lyon, Centre Leon Berard, CNRS 5286, INSERM 1052, Universite Claude Bernard Lyon 1, University Lyon, Lyon F-69008, France (Bouaoud, Martinez, Gregoire, Bertolus, Saintigny) Department of Translational Research and Innovation, Centre Leon Berard, Universite Claude Bernard Lyon 1, University Lyon, Lyon F-69008, France

(Bouaoud, Bertolus) Department of Maxillo-Facial Surgery, Assistance Publique des Hopitaux de Paris, Sorbonne Universite, Hopital Pitie-Salpetriere, Paris F-75013, France

(Bossi) Medical Oncology, ASST Spedali Civili Brescia, Brescia I-25064, Italy

(Bossi) Department of Medical and Surgical Specialties, Radiological Sciences and Public Health, University of Brescia, Brescia I-25123, Italy

(Elkabets, Jagadeeshan) The Shraga Segal Department of Microbiology, Immunology and Genetics, Ben-Gurion University of the Negev, Beer-Sheva 8410501, Israel

(Elkabets, Jagadeeshan) Faculty of Health Sciences, Ben-Gurion University of the Negev, Beer-Sheva 8410501, Israel

(Schmitz, Machiels) Department of Medical Oncology and Head and Neck Surgery, Institut Roi Albert II, Cliniques Universitaires Saint-Luc and Institut de Recherche Clinique et Experimentale (Pole MIRO), UCLouvain, Brussels 1200, Belgium

(van Kempen) Department of Pathology and Medical Biology, University Medical Center Groningen, University of Groningen, Groningen 9712 CP, Netherlands

(Breuskin) Department of Head and Neck Oncology, Gustave Roussy Cancer Campus, Villejuif F-94805, France

(Puppels) Department of Dermatology, Erasmus MC, University Medical Center Rotterdam, Room Ee-1691, P.O. Box 2040, CA Rotterdam 3000, Netherlands

(Hoffmann) INSERM U932 Research Unit, Department of Surgery, Institut Curie, PSL Research University, Paris F-75006, France

(Hunter) Unit of Oral and Maxillofacial Pathology, School of Clinical Dentistry, University of Sheffield, Sheffield S10 2TA, United Kingdom

(Simon) Department of Otolaryngology and Head and Neck Surgery, Lausanne University Hospital, Lausanne 1011, Switzerland

(Gregoire) Radiation Oncology Department, Centre Leon Berard, Universite Claude Bernard Lyon 1, University Lyon, Lyon F-69008, France

(Brakenhoff) Cancer Center Amsterdam, Section Head and Neck Cancer Biology & Immunology, Otolaryngology and Head and Neck Surgery, Vrije Universiteit Amsterdam, Amsterdam UMC, Amsterdam 1081 HV, Netherlands

(Koljenovic) Department of Pathology, Erasmus MC, University Medical Center Rotterdam, Rotterdam 3015 GD, Netherlands

(Saintigny) Department of Medical Oncology, Centre Leon Berard, Universite Claude Bernard Lyon 1, University Lyon, 28 Promenade Lea et Napoleon Bullukian, Lyon F-69008, France

Publisher

MDPI

Emtree Heading

artificial intelligence; autofluorescence imaging; cancer cell culture; cancer classification; *cancer prevention; cancer risk; clinical decision making; clinical feature; clinical outcome; early cancer diagnosis; endoscopy; fluorescence imaging; follow up; genetically engineered mouse strain; heterozygosity loss; high risk patient; histopathology; human; in vivo study; malignant transformation; mouse model; mouth cancer/di [Diagnosis]; mouth cancer/et [Etiology]; mouth cancer/pc [Prevention]; mouth flora; *mouth squamous cell carcinoma/di [Diagnosis]; *mouth squamous cell carcinoma/et [Etiology]; *mouth squamous cell carcinoma/pc [Prevention]; narrow band imaging; oral potentially malignant disorder/di [Diagnosis]; precancer; preclinical study; Raman spectrometry; review; risk factor; three dimensional cell culture; tissue culture; tumor microenvironment; two dimensional cell culture; *unmet medical need; carcinogen; high resolution microendoscopy.

Candidate Terms

high resolution microendoscopy [other term]

Drug Index Terms

carcinogen.

Other Index Terms

artificial intelligence; autofluorescence imaging; cancer cell culture; cancer classification; *cancer prevention; cancer risk; clinical decision making; clinical feature; clinical outcome; early cancer diagnosis; endoscopy; fluorescence imaging; follow up; genetically engineered mouse strain; heterozygosity loss; high risk patient; histopathology; human; in vivo study; malignant transformation; mouse model; mouth cancer / diagnosis / etiology / prevention; mouth flora; *mouth squamous cell carcinoma / *diagnosis / *etiology / *prevention; narrow band imaging; oral potentially malignant disorder / diagnosis; precancer; preclinical study; Raman spectrometry; Review; risk factor; three dimensional cell culture; tissue culture; tumor microenvironment; two dimensional cell culture; *unmet medical need.

Link to the Ovid Full Text or citation:

[Click here for full text options](https://ovidsp.ovid.com/ovidweb.cgi?T=JS&CSC=Y&NEWS=N&PAGE=fulltext&D=emed23&AN=2016116004)

Link to the External Link Resolver:

[SFX](https://sfx-86scu.hosted.exlibrisgroup.com.cn/86scu?sid=OVID:embase&id=pmid:&id=doi:10.3390%2Fcancers14071815&issn=20726694&isbn=&volume=14&issue=7&spage=1815&pages=&date=2022&title=Cancers&atitle=Unmet+Needs+and+Perspectives+in+Oral+Cancer+Prevention&aulast=Bouaoud&pid=<author>Bouaoud+J.%3BBossi+P.%3BElkabets+M.%3BSchmitz+S.%3Bvan+Kempen+L.C.%3BMartinez+P.%3BJagadeeshan+S.%3BBreuskin+I.%3BPuppels+G.J.%3BHoffmann+C.%3BHunter+K.D.%3BSimon+C.%3BMachiels+J.-P.%3BGregoire+V.%3BBertolus+C.%3BBrakenhoff+R.H.%3BKoljenovic+S.%3BSaintigny+P.<%2Fauthor><AN>2016116004<%2FAN><DT>Review<%2FDT>)

47.

Synthesis of Electron-Rich Porous Organic Polymers via Schiff-Base Chemistry for Efficient Iodine Capture.

Tian P., Ai Z., Hu H., Wang M., Li Y., Gao X., Qian J., Su X., Xiao S., Xu H., Lu F., Gao Y.

Molecules (Basel, Switzerland). 27(16) (no pagination), 2022. Date of Publication: 12 Aug 2022.

AN: 638847238

As one of the main nuclear wastes generated in the process of nuclear fission, radioactive iodine has attracted worldwide attention due to its harm to public safety and environmental pollution. Therefore, it is of crucial importance to develop materials that can rapidly and efficiently capture radioactive iodine. Herein, we report the construction of three electron-rich porous organic polymers (POPs), denoted as POP-E, POP-T and POP-P via Schiff base polycondensations reactions between Td-symmetric adamantane knot and four-branched "linkage" molecules. We demonstrated that all the three POPs showed high iodine adsorption capability, among which the adsorption capacity of POP-T for iodine vapor reached up to 3.94 g.g-1 and the removal rate of iodine in n-hexane solution was up to 99%. The efficient iodine capture mechanism of the POP-T was investigated through systematic comparison of Fourier transform infrared spectroscopy (FT-IR), Raman spectroscopy and X-ray photoelectron spectroscopy (XPS) before and after iodine adsorption. The unique pi-pi conjugated system between imine bonds linked aromatic rings with iodine result in charge-transfer complexes, which explains the exceptional iodine capture capacity. Additionally, the introduction of heteroatoms into the framework would also enhance the iodine adsorption capability of POPs. Good retention behavior and recycling capacity were also observed for the POPs.

PMID

36014397 [<https://www.ncbi.nlm.nih.gov/pubmed/?term=36014397>]

Author NameID

Gao, Yanan; ORCID: <https://orcid.org/0000-0002-5543-6296>

Institution

(Tian, Ai, Hu, Wang, Li, Gao, Qian, Su, Lu, Gao) Key Laboratory of Ministry of Education for Advanced Materials in Tropical Island Resources, Department of Chemistry and Chemical Engineering, Hainan University, No 58, Haikou 570228, China (Xiao) China Institute of Atomic Energy, Beijing 102413, China

(Xu) School of Science, Qiongtai Normal University, Haikou 571127, China

Publisher

NLM (Medline)

Emtree Heading

chemistry; electron; human; infrared spectroscopy; porosity; *thyroid tumor; iodide; *iodine; polymer; radioactive iodine; Schiff base.

Drug Index Terms

iodide; *iodine; polymer; radioactive iodine; Schiff base.

Other Index Terms

chemistry; electron; human; infrared spectroscopy; porosity; *thyroid tumor.

Link to the Ovid Full Text or citation:

[Click here for full text options](https://ovidsp.ovid.com/ovidweb.cgi?T=JS&CSC=Y&NEWS=N&PAGE=fulltext&D=emed23&AN=638847238)

Link to the External Link Resolver:

[SFX](https://sfx-86scu.hosted.exlibrisgroup.com.cn/86scu?sid=OVID:embase&id=pmid:36014397&id=doi:10.3390%2Fmolecules27165161&issn=14203049&isbn=&volume=27&issue=16&spage=5161&pages=&date=2022&title=Molecules+(Basel%2C+Switzerland)&atitle=Synthesis+of+Electron-Rich+Porous+Organic+Polymers+via+Schiff-Base+Chemistry+for+Efficient+Iodine+Capture&aulast=Tian&pid=<author>Tian+P.%3BAi+Z.%3BHu+H.%3BWang+M.%3BLi+Y.%3BGao+X.%3BQian+J.%3BSu+X.%3BXiao+S.%3BXu+H.%3BLu+F.%3BGao+Y.<%2Fauthor><AN>638847238<%2FAN><DT>Article<%2FDT>)

48.

Corrigendum to <Diverse spectral band-based deep residual network for tongue squamous cell carcinoma classification using fiber optic Raman spectroscopy> <[Photodiagnosis and Photodynamic Therapy, Volume 32, December 2020, 102048]> (Photodiagnosis and Photodynamic Therapy (2020) 32, (S1572100020304026), (10.1016/j.pdpdt.2020.102048)).

Ding J., Yu M., Zhu L., Zhang T., Xia J., Sun G.

Photodiagnosis and Photodynamic Therapy. 39 (no pagination), 2022. Article Number: 102981. Date of Publication: September 2022.

AN: 2019632389

The authors would like to inform the readers that the Funding support of this research is corrected to below: "This research was funded by R&D Program of Beijing Municipal Education Commission with grant number KM202011232007, Natural Science Foundation of Beijing - Program of Original Innovation Joint Foundation of Haidian District with grant number L182066, National Natural Science Foundation of China (NSFC) with grant number 11904026, and Beijing Natural Science Foundation with grant number 1194021.". The authors would like to apologise for any inconvenience caused.

Copyright © 2022

Institution

(Ding, Yu, Zhu, Xia, Sun) Key Laboratory of the Ministry of Education for Optoelectronic Measurement Technology and Instrument, Beijing Information Science and Technology University, Beijing 100192, China (Zhu, Xia) School of Instrument Science and Opto-electronics Engineering, Hefei University of Technology, Hefei 230009, China

(Zhang) Department of stomatology, Peking Union Medical College Hospital, No. 1 Shuaifuyuan Wangfujing, Dongcheng District, Beijing 100730, China

Publisher

Elsevier B.V.

Emtree Heading

*erratum.

Other Index Terms

*erratum.

Link to the Ovid Full Text or citation:

[Click here for full text options](https://ovidsp.ovid.com/ovidweb.cgi?T=JS&CSC=Y&NEWS=N&PAGE=fulltext&D=emed23&AN=2019632389)

Link to the External Link Resolver:

[SFX](https://sfx-86scu.hosted.exlibrisgroup.com.cn/86scu?sid=OVID:embase&id=pmid:&id=doi:10.1016%2Fj.pdpdt.2022.102981&issn=15721000&isbn=&volume=39&issue=&spage=102981&pages=&date=2022&title=Photodiagnosis+and+Photodynamic+Therapy&atitle=Corrigendum+to+<Diverse+spectral+band-based+deep+residual+network+for+tongue+squamous+cell+carcinoma+classification+using+fiber+optic+Raman+spectroscopy>+<%5BPhotodiagnosis+and+Photodynamic+Therapy%2C+Volume+32%2C+December+2020%2C+102048%5D>+(Photodiagnosis+and+Photodynamic+Therapy+(2020)+32%2C+(S1572100020304026)%2C+(10.1016%2Fj.pdpdt.2020.102048))&aulast=Ding&pid=<author>Ding+J.%3BYu+M.%3BZhu+L.%3BZhang+T.%3BXia+J.%3BSun+G.<%2Fauthor><AN>2019632389<%2FAN><DT>Erratum<%2FDT>)

49.

The effect of therapeutic radiation on dental enamel and dentin: A systematic review.

Douchy L., Gauthier R., Abouelleil-Sayed H., Colon P., Grosgogeat B., Bosco J.

Dental materials : official publication of the Academy of Dental Materials. 38(7) (pp e181-e201), 2022. Date of Publication: 01 Jul 2022.

AN: 638338753

OBJECTIVES: The conventional radiotherapy protocol to treat head-and-neck cancer is usually followed by tooth-decay onset. Radiation impact on mineralized tooth structures is not well-understood. This systematic review aimed to collect the recorded effects of therapeutic radiation on tooth chemical, structural and mechanical properties, in relation with their means of investigation. DATA: Systematic search (January 01 2012 - September 30 2021) terms were "Radiotherapy", "Radiation effects", "Dental enamel", "Dentin", "Human" and "Radiotherapy" NOT "Laser". SOURCES: PubMed, DOSS and Embase databases were searched. STUDY SELECTION: Selected studies compared dental enamel, coronal and root dentin properties before and after in vitro or in vivo irradiation up to 80 Gy.

RESULT(S): The systematic search identified 353 different articles, with 28 satisfying inclusion criteria. Their reference lists provided two more. Twenty-two studies evaluated dental enamel evolution, nine assessed coronal dentin and eight concerned root dentin. Coronal and root dentin results indicate a major impact of the radiation on their organic matrix. Dental enamel's chemical properties are less modified. Enamel and root dentin's hardness are decreased by therapeutic radiation, but no consensus arises for coronal dentin.

CONCLUSION(S): Our findings revealed some interesting information about enzymatic degradation mechanisms of dentin organic matrix and highlighted that dental hard-tissue characterization requires highly specific expertise in materials science. That scientific knowledge is necessary to design suitable protocols, adequately analyze the obtained data, and, thus, provide relevant conclusions. CLINICAL SIGNIFICANCE STATEMENT: Better knowledge and understanding of the mechanisms involved in the degradation of enamel and dentin would enable development of new preventive and therapeutic methods for improved medical care of patients undergoing radiotherapy.

Copyright © 2022 Elsevier Inc. All rights reserved.

PMID

35570008 [<https://www.ncbi.nlm.nih.gov/pubmed/?term=35570008>]

Institution

(Douchy, Colon) Universite de Lyon, Universite Lyon 1, Laboratoire des Multimateriaux et des Interfaces, UMR CNRS, 5615, Lyon, France; Universite de Paris, Faculte d'Odontologie, Paris, France; Assistance Publique-Hopitaux de Paris, Hopital Rothschild, Service Odontologie, Paris, France (Gauthier) Universite de Lyon, Laboratoire des Multimateriaux et des Interfaces, UMR CNRS, Universite Lyon 1, Lyon, France

(Abouelleil-Sayed) Universite de Lyon, Universite Lyon 1, Laboratoire des Multimateriaux et des Interfaces, UMR CNRS, 5615, Lyon, France; Universite de Lyon, Universite Lyon 1, Faculte d'Odontologie, Lyon, France

(Grosgogeat) Universite de Lyon, Universite Lyon 1, Laboratoire des Multimateriaux et des Interfaces, UMR CNRS, 5615, Lyon, France; Hospices Civils de Lyon, Service d'Odontologie, Lyon, France; Universite de Lyon, Universite Lyon 1, Faculte d'Odontologie, Lyon, France

(Bosco) Universite de Lyon, Universite Lyon 1, Laboratoire des Multimateriaux et des Interfaces, UMR CNRS, 5615, Lyon, France; Universite de Paris, Faculte d'Odontologie, Paris, France; Assistance Publique-Hopitaux de Paris, Hopital Pitie-Salpetriere, Service Odontologie, Paris, France

Publisher

NLM (Medline)

Emtree Heading

chemistry; *dental caries; dentin; enamel; human; *radiation injury; *tooth.

Other Index Terms

chemistry; *dental caries; dentin; enamel; human; *radiation injury; *tooth.

Link to the Ovid Full Text or citation:

[Click here for full text options](https://ovidsp.ovid.com/ovidweb.cgi?T=JS&CSC=Y&NEWS=N&PAGE=fulltext&D=emed23&AN=638338753)

Link to the External Link Resolver:

[SFX](https://sfx-86scu.hosted.exlibrisgroup.com.cn/86scu?sid=OVID:embase&id=pmid:35570008&id=doi:10.1016%2Fj.dental.2022.04.014&issn=18790097&isbn=&volume=38&issue=7&spage=e181&pages=e181-e201&date=2022&title=Dental+materials+%3A+official+publication+of+the+Academy+of+Dental+Materials&atitle=The+effect+of+therapeutic+radiation+on+dental+enamel+and+dentin%3A+A+systematic+review&aulast=Douchy&pid=<author>Douchy+L.%3BGauthier+R.%3BAbouelleil-Sayed+H.%3BColon+P.%3BGrosgogeat+B.%3BBosco+J.<%2Fauthor><AN>638338753<%2FAN><DT>Review<%2FDT>)

50.

Effect of paclitaxel stereochemistry on x-ray-triggered release of paclitaxel from CaWO4/paclitaxel-coloaded PEG-PLA nanoparticles.

Sarkar K., Torresgrossa-Allen S., Langer M.P., Durm G., Narayanan S., Elzey B.D., Won Y.-Y.

Cancer Research. Conference: American Association for Cancer Research Annual Meeting, ACCR 2020. New Orleans, LA United States. 82(12 Supplement) (no pagination), 2022. Date of Publication: June 2022.

AN: 638678709

Head and neck squamous cell carcinoma (HNSCC) is the 8th most common cancer in the United States predominantly affecting people over 65 years of age with an increasing rate of incidence across the world. Current therapies for HNSCC include surgical resection, chemotherapy (CT), and radiotherapy (RT). For locally advanced/unresectable HNSCC, the CT-RT combination ("chemoradiation") has been shown to be more effective than CT or RT alone, and is currently the standard of care. Intratumoral (IT) chemotherapy-based chemoradiation has the potential to overcome the limitations of conventional systemic CT-RT that severely affects a patient's quality of life. For realization of maximum benefits from IT CT-RT, our team has developed a radiation-controlled drug release nanoparticle formulation (paclitaxel (PTX) and CaWO4 nanoparticles (CWO NPs) co-encapsulated within a capsule formed by poly(ethylene glycol)-poly (lactic acid) (PEG-PLA), named "PEG-PLA/CWO/PTX NPs"). This unique formulation releases PTX only when it is exposed to X-ray irradiation. We have previously reported that IT-administered PEG-PLA/CWO/PTX NPs stay within the tumor for at least a month, producing significant therapeutic effects in terms of tumor suppression and survival in mouse models of HNSCC. This work demonstrates the effect of PTX stereochemistry on radiation-controlled release of the drug from a nano polymer matrix system (PEG-PLA/CWO/PTX NPs). The stereoisomertic characteristics of PTX products from two different manufacturers ("PTX-S", and "PTX-B") were analyzed by Raman spectroscopy, circular dichroism and 2D HMQC/NOESY NMR measurements. In their unencapsulated (free) state, PTX-S and PTX- B were comparable in their ability to kill cancer cells in vitro. However, they were found to be significantly different in water solubility; PTX-S (water solubility = 4.69 mug/mL) is about 19 times more water soluble than PTX-B (water solubility = 0.25 mug/mL). This difference in water solubility was found to cause a large difference in X-ray-triggered release kinetics of the PTX loaded within the PEG-PLA/CWO/PTX NPs in both in vitro and in vivo environments; PTX-S is released from PEG-PLA/CWO NPs significantly faster upon X-ray irradiation than PTX-B. This difference in release kinetics produced an interesting difference in their time-dependent therapeutic effects; at short times (< 1 month), concurrent PEG-PLA/CWO/PTX-S NPs produced a greater tumor suppression effect; on the other hand, PEG-PLA/CWO/PTX-B NPs had a longer lasting radio-sensitizing effect. In summary, the stereoisomers of PTX exhibit significantly different PK characteristics when used with controlled release carriers, even though they are pharmacologically indistinguishable in their unformulated form.

Publisher

American Association for Cancer Research Inc.

Emtree Heading

adult; animal experiment; animal model; cancer cell; *cancer combination chemotherapy; cancer inhibition; cancer survival; chemoradiotherapy; chemotherapy; circular dichroism; conference abstract; controlled drug release; controlled study; drug formulation; *drug solubility; head and neck squamous cell carcinoma; human; in vitro study; in vivo study; intratumoral drug administration; kinetics; male; microcapsule; mouse; mouse model; nonhuman; nuclear Overhauser effect; quality of life; radiotherapy; Raman spectrometry; *stereochemistry; stereoisomerism; therapy effect; water solubility; X irradiation; *X ray; lactic acid; *nanochain; *paclitaxel; polymer; water.

Drug Index Terms

lactic acid; *nanochain; *paclitaxel; polymer; water.

Other Index Terms

adult; animal experiment; animal model; cancer cell; *cancer combination chemotherapy; cancer inhibition; cancer survival; chemoradiotherapy; chemotherapy; circular dichroism; conference abstract; controlled drug release; controlled study; drug formulation; *drug solubility; head and neck squamous cell carcinoma; human; in vitro study; in vivo study; intratumoral drug administration; kinetics; male; microcapsule; mouse; mouse model; nonhuman; nuclear Overhauser effect; quality of life; radiotherapy; Raman spectrometry; *stereochemistry; stereoisomerism; therapy effect; water solubility; X irradiation; *X ray.

Link to the Ovid Full Text or citation:

[Click here for full text options](https://ovidsp.ovid.com/ovidweb.cgi?T=JS&CSC=Y&NEWS=N&PAGE=fulltext&D=emed23&AN=638678709)

Link to the External Link Resolver:

[SFX](https://sfx-86scu.hosted.exlibrisgroup.com.cn/86scu?sid=OVID:embase&id=pmid:&id=doi:10.1158%2F1538-7445.AM2022-304&issn=15387445&isbn=&volume=82&issue=12+Supplement&spage=304&pages=&date=2022&title=Cancer+Research&atitle=Effect+of+paclitaxel+stereochemistry+on+x-ray-triggered+release+of+paclitaxel+from+CaWO4%2Fpaclitaxel-coloaded+PEG-PLA+nanoparticles&aulast=Sarkar&pid=<author>Sarkar+K.%3BTorresgrossa-Allen+S.%3BLanger+M.P.%3BDurm+G.%3BNarayanan+S.%3BElzey+B.D.%3BWon+Y.-Y.<%2Fauthor><AN>638678709<%2FAN><DT>Conference+Abstract<%2FDT>)

51.

Establishment of a tissue processing workflow and analysis of the influence of formalin fixation and paraffin embedding on the spectral bioinformation of salivary gland tumors.

Till M., Elena G.-H., Mona S., Miriam B., Marc B., Rudolf H., Agmal S., Stephan H.

Laryngo- Rhino- Otologie. Conference: 93rd Annual Meeting of the German Society of Oto-Rhino-Laryngology, Head and Neck Surgery. Hanover Germany. 101(Supplement 2) (pp S196-S197), 2022. Date of Publication: May 2022.

AN: 638571801

Introduction The pre-, intra- and postoperative entity and dignity determination of salivary gland tumors (ST) solely based on histomorphological criteria is often associated with great uncertainties. The spectra of Raman spectroscopy (RS) and infrared spectroscopy (IS) contain information about the molecular composition of the investigated tissue. The aim of this study was the establishment of a tissue processing workflow and the analysis of the influence of formalin and paraffin fixation on the spectral bioinformation compared to cryofixation. In addition, an overview of the use of RS and IS in head and neck oncology is given. Material and methods 10 mum thick, consecutive sections from cryo-, formalin-, and paraffin-fixed cystadenolymphomas (n = 5) and pleomorphic adenomas (n = 4) were examined by the RS and IS and data were evaluated multivariately. The measurements were made in correlation to the histomorphology via a corresponding HE section both in the tumor tissue and in the healthy salivary gland tissue. Results The mean value spectrum analysis showed a clear paraffin signature, formalin fixation had no significant influence. This could be confirmed by the principal component analysis (PCA). A discrimination of tumor and non-tumor tissue by the PCA and coupled discriminant analysis was also possible with both spectroscopic methods, RS and IS, with a high sensitivity. Discussion For a translation of spectral processes, knowledge on factors influencing the spectral bioinformation of tissue preparation and fixation is essential. The additive integration of spectral methods into existing work processes is possible. The influence of formalin fixation on the spectral bioinformation is small. The bioinformatic analysis of the extensive data sets is challenging.

Institution

(Till, Rudolf, Agmal) Institutes Klinik und Poliklinik fur Hals-,Nasen- und Ohrenkrankheiten, plastische und asthetische Operationen, Universitatsklinikum Wurzburg, Wurzburg, Germany (Elena) Institut fur Pathologie, Wurzburg, Germany

(Mona, Miriam, Marc) Lehr- und Forschungszentrum Process Analysis and Technology PA and T, Hochschule Reutlingen Reutlingen, Germany

(Stephan) Klinik fur Hals-Nasen-Ohren-Heilkunde, Kopf- und Hals-Chirurgie, Uniklinik RWTH Aachen, Aachen, Germany

Publisher

Georg Thieme Verlag

Emtree Heading

clinical article; conference abstract; controlled study; discriminant analysis; histopathology; human; human tissue; infrared spectroscopy; male; neck; *paraffin embedding; pleomorphic adenoma; principal component analysis; Raman spectrometry; salivary gland; *salivary gland tumor; tissue preparation; *workflow; *formaldehyde; paraffin.

Drug Index Terms

*formaldehyde; paraffin.

Other Index Terms

clinical article; conference abstract; controlled study; discriminant analysis; histopathology; human; human tissue; infrared spectroscopy; male; neck; *paraffin embedding; pleomorphic adenoma; principal component analysis; Raman spectrometry; salivary gland; *salivary gland tumor; tissue preparation; *workflow.

Link to the Ovid Full Text or citation:

[Click here for full text options](https://ovidsp.ovid.com/ovidweb.cgi?T=JS&CSC=Y&NEWS=N&PAGE=fulltext&D=emed23&AN=638571801)

Link to the External Link Resolver:

[SFX](https://sfx-86scu.hosted.exlibrisgroup.com.cn/86scu?sid=OVID:embase&id=pmid:&id=doi:10.1055%2Fs-0042-1746596&issn=14388685&isbn=&volume=101&issue=Supplement+2&spage=S196&pages=S196-S197&date=2022&title=Laryngo-+Rhino-+Otologie&atitle=Establishment+of+a+tissue+processing+workflow+and+analysis+of+the+influence+of+formalin+fixation+and+paraffin+embedding+on+the+spectral+bioinformation+of+salivary+gland+tumors&aulast=Till&pid=<author>Till+M.%3BElena+G.-H.%3BMona+S.%3BMiriam+B.%3BMarc+B.%3BRudolf+H.%3BAgmal+S.%3BStephan+H.<%2Fauthor><AN>638571801<%2FAN><DT>Conference+Abstract<%2FDT>)

52.

Corrigendum: Diagnostic accuracy of Raman spectroscopy in oral squamous cell carcinoma(Front. Oncol., (2022), 12, (925032), 10.3389/fonc.2022.925032).

Han R., Lin N., Huang J., Ma X.

Frontiers in Oncology. 12 (no pagination), 2022. Article Number: 1030058. Date of Publication: 21 Sep 2022.

AN: 2019428814

In the published article, there was an error in affiliation 1. Instead of "State Key Laboratory of Oral Diseases, National Clinical Research Center for Oral Diseases, Sichuan University, Chengdu, China", it should be "Department of Biotherapy, West China Hospital and State Key Laboratory of Biotherapy, Sichuan University, Chengdu, China". In the published article, there was an error in affiliation 2. Instead of "Department of Biotherapy, West China Hospital and State Key Laboratory of Biotherapy, Sichuan University, Chengdu, China", it should be "State Key Laboratory of Oral Diseases, National Clinical Research Center for Oral Diseases, Sichuan University, Chengdu, China". In the published article, there was an error regarding the affiliation for Ruiying Han. As well as having affiliation 2, they should also have Department of Biotherapy, West China Hospital and State Key Laboratory of Biotherapy, Sichuan University, Chengdu, China. In the published article, there was an error. There was a mistake in the time range of the searched literature in Abstract. A correction has been made to Abstract, Methods, 1. This sentence previously stated: "We systematically searched databases including Medline, Embase, and Web of Science for studies up to March 2022 with no start date limited."The corrected sentence appears below: "We systematically searched databases including Medline, Embase, and Web of Science for studies from January 2000 to March 2022." The authors apologize for these errors and state that this does not change the scientific conclusions of the article in any way. The original article has been updated.

Copyright © 2022 Han, Lin, Huang and Ma.

Institution

(Han, Lin, Ma) Department of Biotherapy, West China Hospital and State Key Laboratory of Biotherapy, Sichuan University, Chengdu, China (Han) State Key Laboratory of Oral Diseases, National Clinical Research Center for Oral Diseases, Sichuan University, Chengdu, China

(Huang) Department of Hematology, Sichuan Academy of Medical Sciences, Sichuan Provincial People's Hospital, University of Electronic Science and Technology of China, Chengdu, China

Publisher

Frontiers Media S.A.

Emtree Heading

*erratum.

Other Index Terms

*erratum.

Link to the Ovid Full Text or citation:

[Click here for full text options](https://ovidsp.ovid.com/ovidweb.cgi?T=JS&CSC=Y&NEWS=N&PAGE=fulltext&D=emed23&AN=2019428814)

Link to the External Link Resolver:

[SFX](https://sfx-86scu.hosted.exlibrisgroup.com.cn/86scu?sid=OVID:embase&id=pmid:&id=doi:10.3389%2Ffonc.2022.1030058&issn=2234943X&isbn=&volume=12&issue=&spage=1030058&pages=&date=2022&title=Frontiers+in+Oncology&atitle=Corrigendum%3A+Diagnostic+accuracy+of+Raman+spectroscopy+in+oral+squamous+cell+carcinoma(Front.+Oncol.%2C+(2022)%2C+12%2C+(925032)%2C+10.3389%2Ffonc.2022.925032)&aulast=Han&pid=<author>Han+R.%3BLin+N.%3BHuang+J.%3BMa+X.<%2Fauthor><AN>2019428814<%2FAN><DT>Erratum<%2FDT>)

53.

Bioengineering the ameloblastoma tumour to study its effect on bone nodule formation.

Bakkalci D., Jay A., Rezaei A., Howard C.A., Haugen H.J., Pape J., Kishida S., Kishida M., Jell G., Arnett T.R., Fedele S., Cheema U.

Scientific reports. 11(1) (pp 24088), 2021. Date of Publication: 16 Dec 2021.

AN: 636916151

Ameloblastoma is a benign, epithelial cancer of the jawbone, which causes bone resorption and disfigurement to patients affected. The interaction of ameloblastoma with its tumour stroma drives invasion and progression. We used stiff collagen matrices to engineer active bone forming stroma, to probe the interaction of ameloblastoma with its native tumour bone microenvironment. This bone-stroma was assessed by nano-CT, transmission electron microscopy (TEM), Raman spectroscopy and gene analysis. Furthermore, we investigated gene correlation between bone forming 3D bone stroma and ameloblastoma introduced 3D bone stroma. Ameloblastoma cells increased expression of MMP-2 and -9 and RANK temporally in 3D compared to 2D. Our 3D biomimetic model formed bone nodules of an average surface area of 0.1 mm2 and average height of 92.37 [Formula: see text] 7.96 mum over 21 days. We demonstrate a woven bone phenotype with distinct mineral and matrix components and increased expression of bone formation genes in our engineered bone. Introducing ameloblastoma to the bone stroma, completely inhibited bone formation, in a spatially specific manner. Multivariate gene analysis showed that ameloblastoma cells downregulate bone formation genes such as RUNX2. Through the development of a comprehensive bone stroma, we show that an ameloblastoma tumour mass prevents osteoblasts from forming new bone nodules and severely restricted the growth of existing bone nodules. We have identified potential pathways for this inhibition. More critically, we present novel findings on the interaction of stromal osteoblasts with ameloblastoma.

Copyright © 2021. The Author(s).

PMID

34916549 [<https://www.ncbi.nlm.nih.gov/pubmed/?term=34916549>]

Institution

(Bakkalci, Pape, Cheema) UCL Centre of 3D Models Health and Disease, Division of Surgery and Interventional Sciences, University College London, Charles Bell House, London, United Kingdom (Jay) University College London Hospitals, London, United Kingdom

(Rezaei, Jell) Division of Surgery and Interventional Sciences, University College London, Royal Free Campus, London, United Kingdom

(Howard) Deparment of Physics & Astronomy, University College London, London, United Kingdom

(Haugen) Department of Biomaterial Institute for Clinical Dentistry, University of Oslo, Oslo, Norway

(Kishida, Kishida) Department of Biochemistry and Genetics, Graduate School of Medical and Dental Sciences, Kagoshima University, Kagoshima, Japan

(Arnett) Department of Cell and & Developmental Biology, University College London, London, United Kingdom

(Fedele) Eastman Dental Institute, University College London, London, United Kingdom

Publisher

NLM (Medline)

Emtree Heading

ameloblastoma/th [Therapy]; animal; *bone development; complication; etiology; gene expression; genetics; human; jaw tumor/th [Therapy]; metabolism; osteoblast; osteolysis; *pathophysiology; physiology; *procedures; rat; *stroma cell; tissue engineering; tumor cell culture; tumor invasion; tumor microenvironment; gelatinase A; osteoclast differentiation factor; transcription factor RUNX2; Runx2 protein, rat.

Candidate Terms

Runx2 protein, rat [drug term]

Drug Index Terms

gelatinase A; osteoclast differentiation factor; transcription factor RUNX2.

Other Index Terms

ameloblastoma / therapy; animal; *bone development; complication; etiology; gene expression; genetics; human; jaw tumor / therapy; metabolism; osteoblast; osteolysis; *pathophysiology; physiology; *procedures; rat; *stroma cell; tissue engineering; tumor cell culture; tumor invasion; tumor microenvironment.

Link to the Ovid Full Text or citation:

[Click here for full text options](https://ovidsp.ovid.com/ovidweb.cgi?T=JS&CSC=Y&NEWS=N&PAGE=fulltext&D=emed23&AN=636916151)

Link to the External Link Resolver:

[SFX](https://sfx-86scu.hosted.exlibrisgroup.com.cn/86scu?sid=OVID:embase&id=pmid:34916549&id=doi:10.1038%2Fs41598-021-03484-5&issn=20452322&isbn=&volume=11&issue=1&spage=24088&pages=24088&date=2021&title=Scientific+reports&atitle=Bioengineering+the+ameloblastoma+tumour+to+study+its+effect+on+bone+nodule+formation&aulast=Bakkalci&pid=<author>Bakkalci+D.%3BJay+A.%3BRezaei+A.%3BHoward+C.A.%3BHaugen+H.J.%3BPape+J.%3BKishida+S.%3BKishida+M.%3BJell+G.%3BArnett+T.R.%3BFedele+S.%3BCheema+U.<%2Fauthor><AN>636916151<%2FAN><DT>Article<%2FDT>)

54.

Tracking of the biochemical changes upon pleomorphic adenoma progression using vibrational microspectroscopy.

Paluszkiewicz C., Roman M., Piergies N., Pieta E., Wozniak M., Guidi M.C., Miskiewicz-Orczyk K., Markow M., Scierski W., Misiolek M., Drozdzowska B., Kwiatek W.M.

Scientific reports. 11(1) (pp 18010), 2021. Date of Publication: 09 Sep 2021.

AN: 636106069

Head and neck tumors can be very challenging to treat because of the risk of problems or complications after surgery. Therefore, prompt and accurate diagnosis is extremely important to drive appropriate treatment decisions, which may reduce the chance of recurrence. This paper presents the original research exploring the feasibility of Fourier transform infrared (FT-IR) and Raman spectroscopy (RS) methods to investigate biochemical alterations upon the development of the pleomorphic adenoma. Principal component analysis (PCA) was used for a detailed assessment of the observed changes and to determine the spectroscopic basis for salivary gland neoplastic pathogenesis. It is implied that within the healthy margin, as opposed to the tumoral tissue, there are parts that differ significantly in lipid content. This observation shed new light on the crucial role of lipids in tissue physiology and tumorigenesis. Thus, a novel approach that eliminates the influence of lipids on the elucidation of biochemical changes is proposed. The performed analysis suggests that the highly heterogeneous healthy margin contains more unsaturated triacylglycerols, while the tumoral section is rich in proteins. The difference in protein content was also observed for these two tissue types, i.e. the healthy tissue possesses more proteins in the anti-parallel beta-sheet conformation, whereas the tumoral tissue is dominated by proteins rich in unordered random coils. Furthermore, the pathogenic tissue shows a higher content of carbohydrates and reveals noticeable differences in nucleic acid content. Finally, FT-IR and Raman spectroscopy methods were proposed as very promising methods in the discrimination of tumoral and healthy tissues of the salivary gland.

Copyright © 2021. The Author(s).

PMID

34504182 [<https://www.ncbi.nlm.nih.gov/pubmed/?term=34504182>]

Author NameID

Paluszkiewicz, Czeslawa; ORCID: <https://orcid.org/0000-0003-0186-8090> Roman, Maciej; ORCID: <https://orcid.org/0000-0003-0921-426X>

Piergies, Natalia; ORCID: <https://orcid.org/0000-0003-4899-3534>

Pieta, Ewa; ORCID: <https://orcid.org/0000-0001-7071-0284>

Wozniak, Monika; ORCID: <https://orcid.org/0000-0002-3709-8318>

Kwiatek, Wojciech M.; ORCID: <https://orcid.org/0000-0002-2197-8572>

Guidi, Mariangela Cestelli; ORCID: <https://orcid.org/0000-0002-6884-3915>

Miskiewicz-Orczyk, Katarzyna; ORCID: <https://orcid.org/0000-0001-8088-3437>

Markow, Magdalena; ORCID: <https://orcid.org/0000-0002-1072-2125>

Scierski, Wojciech; ORCID: <https://orcid.org/0000-0003-3242-8047>

Misiolek, Maciej; ORCID: <https://orcid.org/0000-0002-8476-9153>

Drozdzowska, Bogna; ORCID: <https://orcid.org/0000-0002-2287-6842>

Institution

(Paluszkiewicz, Roman, Piergies, Pieta, Wozniak, Kwiatek) Institute of Nuclear Physics, Polish Academy of Sciences, Radzikowskiego 152, Krakow 31-342, Poland (Guidi) INFN-Laboratori Nazionali di Frascati, Via E. Fermi 40, Frascati 00044, Italy

(Miskiewicz-Orczyk, Markow, Scierski, Misiolek) Department of Otorhinolaryngology and Laryngological Oncology in Zabrze, Medical University of Silesia Katowice, Zabrze 41800, Poland

(Drozdzowska) Department of Pathomorphology Zabrze, Medical University of Silesia, Katowice, Poland

Publisher

NLM (Medline)

Emtree Heading

alpha helix; antibody specificity; beta sheet; carcinogenesis; chemistry; cytochemistry; female; human; information processing; infrared spectroscopy; male; metabolism; middle aged; pathology; pleomorphic adenoma/di [Diagnosis]; pleomorphic adenoma/su [Surgery]; principal component analysis; *procedures; Raman spectrometry; salivary gland tumor/di [Diagnosis]; salivary gland tumor/su [Surgery]; carbohydrate; eosin; hematoxylin; nucleic acid; triacylglycerol; tumor protein.

Drug Index Terms

carbohydrate; eosin; hematoxylin; nucleic acid; triacylglycerol; tumor protein.

Other Index Terms

alpha helix; antibody specificity; beta sheet; carcinogenesis; chemistry; cytochemistry; female; human; information processing; infrared spectroscopy; male; metabolism; middle aged; pathology; pleomorphic adenoma / diagnosis / surgery; principal component analysis; *procedures; Raman spectrometry; salivary gland tumor / diagnosis / surgery.

Link to the Ovid Full Text or citation:

[Click here for full text options](https://ovidsp.ovid.com/ovidweb.cgi?T=JS&CSC=Y&NEWS=N&PAGE=fulltext&D=emed23&AN=636106069)

Link to the External Link Resolver:

[SFX](https://sfx-86scu.hosted.exlibrisgroup.com.cn/86scu?sid=OVID:embase&id=pmid:34504182&id=doi:10.1038%2Fs41598-021-97377-2&issn=20452322&isbn=&volume=11&issue=1&spage=18010&pages=18010&date=2021&title=Scientific+reports&atitle=Tracking+of+the+biochemical+changes+upon+pleomorphic+adenoma+progression+using+vibrational+microspectroscopy&aulast=Paluszkiewicz&pid=<author>Paluszkiewicz+C.%3BRoman+M.%3BPiergies+N.%3BPieta+E.%3BWozniak+M.%3BGuidi+M.C.%3BMiskiewicz-Orczyk+K.%3BMarkow+M.%3BScierski+W.%3BMisiolek+M.%3BDrozdzowska+B.%3BKwiatek+W.M.<%2Fauthor><AN>636106069<%2FAN><DT>Article<%2FDT>)

55.

Geometrically encoded SERS nanobarcodes for the logical detection of nasopharyngeal carcinoma-related progression biomarkers.

Lin D., Hsieh C.-L., Hsu K.-C., Liao P.-H., Qiu S., Gong T., Yong K.-T., Feng S., Kong K.V.

Nature Communications. 12(1) (no pagination), 2021. Article Number: 3430. Date of Publication: 01 Dec 2021.

AN: 2012308289

The limited availability of nasopharyngeal carcinoma-related progression biomarker array kits that offer physicians comprehensive information is disadvantageous for monitoring cancer progression. To develop a biomarker array kit, systematic identification and differentiation of a large number of distinct molecular surface-enhanced Raman scattering (SERS) reporters with high spectral temporal resolution is a major challenge. To address this unmet need, we use the chemistry of metal carbonyls to construct a series of unique SERS reporters with the potential to provide logical and highly multiplex information during testing. In this study, we report that geometric control over metal carbonyls on nanotags can produce 14 distinct barcodes that can be decoded unambiguously using commercial Raman spectroscopy. These metal carbonyl nanobarcodes are tested on human blood samples and show strong sensitivity (0.07 ng/mL limit of detection, average CV of 6.1% and >92% degree of recovery) and multiplexing capabilities for MMPs.

Copyright © 2021, The Author(s).

PMID

34078895 [<https://www.ncbi.nlm.nih.gov/pubmed/?term=34078895>]

Author NameID

Lin, Duo; ORCID: <https://orcid.org/0000-0001-6959-5995> Gong, Tianxun; ORCID: <https://orcid.org/0000-0001-9383-3666>

Institution

(Lin, Feng) Key Laboratory of OptoElectronic Science and Technology for Medicine, Ministry of Education, Fujian Provincial Key Laboratory for Photonics Technology, Fujian Normal University, Fuzhou, Fujian, China (Hsieh, Hsu, Liao, Kong) Department of Chemistry, National Taiwan University, Taipei, Taiwan (Republic of China)

(Qiu) Fujian Medical University Cancer Hospital, Fujian Cancer Hospital, Fuzhou, Fujian, China

(Gong) State Key Laboratory of Electronic Thin Films and Integrated Devices, School of Electronic Science and Engineering (National Exemplary School of Microelectronics), University of Electronic Science and Technology of China, Chengdu, China

(Yong) School of Biomedical Engineering, The University of Sydney, Sydney, NSW, Australia

(Yong) The University of Sydney Nano Institute, The University of Sydney, Sydney, NSW, Australia

Publisher

Nature Research

Emtree Heading

article; blood sampling; cancer growth; electromagnetic radiation; Epstein Barr virus infection; genetic susceptibility; human; information science; limit of detection; metastasis; *nasopharynx carcinoma; polarization; protein degradation; protein expression; *surface enhanced Raman spectroscopy; synthesis; tumor invasion; avidin; biological marker/ec [Endogenous Compound]; biotin; *carbonyl derivative; collagen type 10/ec [Endogenous Compound]; collagen type 11/ec [Endogenous Compound]; collagen type 4/ec [Endogenous Compound]; collagen type 9/ec [Endogenous Compound]; cysteine/ec [Endogenous Compound]; gelatin; gelatinase A/ec [Endogenous Compound]; gelatinase B/ec [Endogenous Compound]; gold nanoparticle; interstitial collagenase/ec [Endogenous Compound]; iodine; matrilysin/ec [Endogenous Compound]; matrix metalloproteinase/ec [Endogenous Compound]; molybdenum; *nanogel; rhenium; ruthenium; stromelysin/ec [Endogenous Compound]; thiol group/ec [Endogenous Compound]; *transition element; tungsten.

Drug Index Terms

avidin; biological marker / endogenous compound; biotin; *carbonyl derivative; collagen type 10 / endogenous compound; collagen type 11 / endogenous compound; collagen type 4 / endogenous compound; collagen type 9 / endogenous compound; cysteine / endogenous compound; gelatin; gelatinase A / endogenous compound; gelatinase B / endogenous compound; gold nanoparticle; interstitial collagenase / endogenous compound; iodine; matrilysin / endogenous compound; matrix metalloproteinase / endogenous compound; molybdenum; *nanogel; rhenium; ruthenium; stromelysin / endogenous compound; thiol group / endogenous compound; *transition element; tungsten.

Other Index Terms

Article; blood sampling; cancer growth; electromagnetic radiation; Epstein Barr virus infection; genetic susceptibility; human; information science; limit of detection; metastasis; *nasopharynx carcinoma; polarization; protein degradation; protein expression; *surface enhanced Raman spectroscopy; synthesis; tumor invasion.

Link to the Ovid Full Text or citation:

[Click here for full text options](https://ovidsp.ovid.com/ovidweb.cgi?T=JS&CSC=Y&NEWS=N&PAGE=fulltext&D=emed23&AN=2012308289)

Link to the External Link Resolver:

[SFX](https://sfx-86scu.hosted.exlibrisgroup.com.cn/86scu?sid=OVID:embase&id=pmid:34078895&id=doi:10.1038%2Fs41467-021-23789-3&issn=20411723&isbn=&volume=12&issue=1&spage=3430&pages=&date=2021&title=Nature+Communications&atitle=Geometrically+encoded+SERS+nanobarcodes+for+the+logical+detection+of+nasopharyngeal+carcinoma-related+progression+biomarkers&aulast=Lin&pid=<author>Lin+D.%3BHsieh+C.-L.%3BHsu+K.-C.%3BLiao+P.-H.%3BQiu+S.%3BGong+T.%3BYong+K.-T.%3BFeng+S.%3BKong+K.V.<%2Fauthor><AN>2012308289<%2FAN><DT>Article<%2FDT>)

56.

Brachytherapy approach using177 Lu conjugated gold nanostars and evaluation of biodistribution, tumor retention, dosimetry and therapeutic efficacy in head and neck tumor model.

Lin M.-Y., Hsieh H.-H., Chen J.-C., Chen C.-L., Sheu N.-C., Huang W.-S., Ho S.-Y., Chen T.-W., Lee Y.-J., Wu C.-Y.

Pharmaceutics. 13(11) (no pagination), 2021. Article Number: 1903. Date of Publication: November 2021.

AN: 2014592910

Brachytherapy can provide sufficient doses to head and neck squamous cell carcinoma (HNSCC) with minimal damage to nearby normal tissues. In this study, the beta--emitter177 Lu was conjugated to DTPA-polyethylene glycol (PEG) decorated gold nanostars (177 Lu-DTPA-pAuNS) used in surface-enhanced Raman scattering and photothermal therapy (PTT). The accumulation and therapeutic efficacy of177 Lu-DTPA-pAuNS were compared with those of177 Lu-DTPA on an orthotopic HNSCC tumor model. The SPECT/CT imaging and biodistribution studies showed that177 Lu-DTPA-pAuNS can be accumulated in the tumor up to 15 days, but177 Lu-DTPA could not be detected at 24 h after injection. The tumor viability and growth were suppressed by injected177 Lu-DTPA-pAuNS but not nonconjugated177 Lu-DTPA, as evaluated by bioluminescent imaging. The radiation-absorbed dose of the normal organ was the highest in the liver (0.33 mSv/MBq) estimated in a 73 kg adult, but that of tumorsphere (0.5 g) was 3.55 mGy/MBq, while intravenous injection of177 Lu-DTPA-pAuNS resulted in 1.97 mSv/MBq and 0.13 mGy/MBq for liver and tumorsphere, respectively. We also observed further enhancement of tumor-suppressive effects by a combination of177 Lu-DTPA-pAuNS and PTT compared to177 Lu-DTPA-pAuNS alone. In conclusion,177 Lu-DTPA-pAuNS may be considered as a potential radiopharmaceutical agent for HNSCC brachytherapy.

Copyright © 2021 by the authors. Licensee MDPI, Basel, Switzerland.

Institution

(Lin, Hsieh, Chen, Chen, Sheu, Lee, Wu) Department of Biomedical Imaging and Radiological Sciences, National Yang Ming Chiao Tung University, Taipei Branch, Taipei 112, Taiwan (Republic of China) (Huang) Department of Nuclear Medicine, Taipei Medical University Hospital, Taipei 11031, Taiwan (Republic of China)

(Ho, Chen) Institute of Bioinformatics and Systems Biology, National Yang Ming Chiao Tung University, Hsinchu Branch, Hsinchu 30068, Taiwan (Republic of China)

(Chen) Department of Biological Science and Technology, National Yang Ming Chiao Tung University, Hsinchu Branch, Hsinchu 30068, Taiwan (Republic of China)

(Lee) Cancer Progression Research Center, National Yang Ming Chiao Tung University, Taipei Branch, Taipei 112, Taiwan (Republic of China)

Publisher

MDPI

Emtree Heading

animal experiment; animal model; animal tissue; article; *brachytherapy; *clinical effectiveness; concentration response; controlled study; *dosimetry; *drug distribution; *drug retention; *head and neck squamous cell carcinoma/dt [Drug Therapy]; *head and neck squamous cell carcinoma/th [Therapy]; human; human cell; mouse; nonhuman; photothermal therapy; radiation absorption; radiation dose distribution; single photon emission computed tomography-computed tomography; staining; surface enhanced Raman spectroscopy; theranostic nanomedicine; treatment outcome; treatment response; *gold nanoparticle; *lutetium 177/dt [Drug Therapy]; *lutetium 177/tu [Intratumoral Drug Administration]; *lutetium 177/iv [Intravenous Drug Administration]; *lutetium 177/pk [Pharmacokinetics]; macrogol; *nanostar; pentetic acid.

Drug Index Terms

*gold nanoparticle; *lutetium 177 / *drug therapy / *intratumoral drug administration / *intravenous drug administration / *pharmacokinetics; macrogol; *nanostar; pentetic acid.

Other Index Terms

animal experiment; animal model; animal tissue; Article; *brachytherapy; *clinical effectiveness; concentration response; controlled study; *dosimetry; *drug distribution; *drug retention; *head and neck squamous cell carcinoma / *drug therapy / *therapy; human; human cell; mouse; nonhuman; photothermal therapy; radiation absorption; radiation dose distribution; single photon emission computed tomography-computed tomography; staining; surface enhanced Raman spectroscopy; theranostic nanomedicine; treatment outcome; treatment response.

Link to the Ovid Full Text or citation:

[Click here for full text options](https://ovidsp.ovid.com/ovidweb.cgi?T=JS&CSC=Y&NEWS=N&PAGE=fulltext&D=emed23&AN=2014592910)

Link to the External Link Resolver:

[SFX](https://sfx-86scu.hosted.exlibrisgroup.com.cn/86scu?sid=OVID:embase&id=pmid:&id=doi:10.3390%2Fpharmaceutics13111903&issn=19994923&isbn=&volume=13&issue=11&spage=1903&pages=&date=2021&title=Pharmaceutics&atitle=Brachytherapy+approach+using177+Lu+conjugated+gold+nanostars+and+evaluation+of+biodistribution%2C+tumor+retention%2C+dosimetry+and+therapeutic+efficacy+in+head+and+neck+tumor+model&aulast=Lin&pid=<author>Lin+M.-Y.%3BHsieh+H.-H.%3BChen+J.-C.%3BChen+C.-L.%3BSheu+N.-C.%3BHuang+W.-S.%3BHo+S.-Y.%3BChen+T.-W.%3BLee+Y.-J.%3BWu+C.-Y.<%2Fauthor><AN>2014592910<%2FAN><DT>Article<%2FDT>)

57.

Pilot study on the value of Raman spectroscopy in the entity assignment of salivary gland tumors.

Meyer T.J., Gerhard-Hartmann E., Lodes N., Scherzad A., Hagen R., Steinke M., Hackenberg S.

PLoS ONE. 16(9 September) (no pagination), 2021. Article Number: e0257470. Date of Publication: September 2021.

AN: 2014640311

Background The entity assignment of salivary gland tumors (SGT) based on histomorphology can be challenging. Raman spectroscopy has been applied to analyze differences in the molecular composition of tissues. The aim of this study was to evaluate the suitability of RS for entity assignment in SGT. Methods Raman data were collected in deparaffinized sections of pleomorphic adenomas (PA) and adenoid cystic carcinomas (ACC). Multivariate data and chemometric analysis were completed using the Unscrambler software. Results The Raman spectra detected in ACC samples were mostly assigned to nucleic acids, lipids, and amides. In a principal component-based linear discriminant analysis (LDA) 18 of 20 tumor samples were classified correctly. Conclusion In this proof of concept study, we show that a reliable SGT diagnosis based on LDA algorithm appears possible, despite variations in the entity-specific mean spectra. However, a standardized workflow for tissue sample preparation, measurement setup, and chemometric algorithms is essential to get reliable results.

Copyright © 2021 Meyer et al. This is an open access article distributed under the terms of the Creative Commons Attribution License, which permits unrestricted use, distribution, and reproduction in any medium, provided the original author and source are credited.

PMID

34529739 [<https://www.ncbi.nlm.nih.gov/pubmed/?term=34529739>]

Institution

(Meyer, Scherzad, Hagen, Hackenberg) Department of Oto-Rhino-Laryngology, Plastic, Aesthetic and Reconstructive Head and Neck Surgery, University Hospital Wurzburg, Wurzburg, Germany (Gerhard-Hartmann) Institute of Pathology, University of Wurzburg, Wurzburg, Germany

(Lodes, Steinke) Chair of Tissue Engineering and Regenerative Medicine, University Hospital Wurzburg, Wurzburg, Germany

(Steinke) Fraunhofer Institute for Silicate Research ISC, Wurzburg, Germany

(Hackenberg) Department of Otorhinolaryngology - Head and Neck Surgery, RWTH Aachen University Hospital, Aachen, Germany

Publisher

Public Library of Science

Emtree Heading

adenoid cystic carcinoma; adult; aged; article; cell heterogeneity; cell infiltration; cell nucleus; chemometric analysis; cholangiocyte; clinical article; cytoplasm; discriminant analysis; extracellular matrix; female; fluorescence microscopy; histopathology; human; human tissue; male; mesenchymal stroma cell; myoepithelium cell; parotid gland slice; perineural invasion; pilot study; pleomorphic adenoma; predictive value; principal component analysis; *Raman spectrometry; *salivary gland tumor; tumor diagnosis; very elderly; amide/ec [Endogenous Compound]; carbohydrate/ec [Endogenous Compound]; collagen/ec [Endogenous Compound]; eosin; formaldehyde; glucose/ec [Endogenous Compound]; glycogen/ec [Endogenous Compound]; hematoxylin; lipid/ec [Endogenous Compound]; nucleic acid/ec [Endogenous Compound]; phenylalanine/ec [Endogenous Compound]; protein/ec [Endogenous Compound]; RNA/ec [Endogenous Compound]; confocal microscope; digital microscope; diode laser; fluorescence microscope; BioRam; BIOREVO BZ-9000.

Candidate Terms

BioRam [device term] BIOREVO BZ 9000 [device term]

Device Index Terms

confocal microscope; digital microscope; diode laser; fluorescence microscope.

Drug Index Terms

amide / endogenous compound; carbohydrate / endogenous compound; collagen / endogenous compound; eosin; formaldehyde; glucose / endogenous compound; glycogen / endogenous compound; hematoxylin; lipid / endogenous compound; nucleic acid / endogenous compound; phenylalanine / endogenous compound; protein / endogenous compound; RNA / endogenous compound.

Other Index Terms

adenoid cystic carcinoma; adult; aged; Article; cell heterogeneity; cell infiltration; cell nucleus; chemometric analysis; cholangiocyte; clinical article; cytoplasm; discriminant analysis; extracellular matrix; female; fluorescence microscopy; histopathology; human; human tissue; male; mesenchymal stroma cell; myoepithelium cell; parotid gland slice; perineural invasion; pilot study; pleomorphic adenoma; predictive value; principal component analysis; *Raman spectrometry; *salivary gland tumor; tumor diagnosis; very elderly.

Link to the Ovid Full Text or citation:

[Click here for full text options](https://ovidsp.ovid.com/ovidweb.cgi?T=JS&CSC=Y&NEWS=N&PAGE=fulltext&D=emed23&AN=2014640311)

Link to the External Link Resolver:

[SFX](https://sfx-86scu.hosted.exlibrisgroup.com.cn/86scu?sid=OVID:embase&id=pmid:34529739&id=doi:10.1371%2Fjournal.pone.0257470&issn=19326203&isbn=&volume=16&issue=9+September&spage=e0257470&pages=&date=2021&title=PLoS+ONE&atitle=Pilot+study+on+the+value+of+Raman+spectroscopy+in+the+entity+assignment+of+salivary+gland+tumors&aulast=Meyer&pid=<author>Meyer+T.J.%3BGerhard-Hartmann+E.%3BLodes+N.%3BScherzad+A.%3BHagen+R.%3BSteinke+M.%3BHackenberg+S.<%2Fauthor><AN>2014640311<%2FAN><DT>Article<%2FDT>)

58.

N-rich porous carbon catalysts with huge surface areas from bean curd activated by K2CO3.

Yi C., Zhang L., Xiang G., Chen X., Cheng N., Liu Z.

New Journal of Chemistry. 45(36) (pp 16469-16476), 2021. Date of Publication: 28 Sep 2021.

AN: 636014442

Nitrogen-doped carbon materials with hierarchical porous structures have attracted huge attention in the field of catalysis owing to their special structures and properties in past few decades. In this study, a series of metal-free and N-rich porous carbon catalysts (NPCs) were synthesized by a one-step activation-pyrolysis process. The catalytic performance of NPCs obtained from using bean curd as the carbon and nitrogen precursors and activatedviapyrolysis in a nitrogen atmosphere with different amounts of K2CO3was investigated for the selective oxidation of ethylbenzene. The catalysts were characterized using a series of techniques, such as N2adsorption-desorption isotherms, Raman spectroscopy, TEM, SEM, and XPS. The results presented that the as-prepared samples have specific surface areas as high as 1459 m2g-1. Furthermore, NPC-10 displayed the optimal catalytic performance among the catalysts. The ethylbenzene conversion of 96.2% over NPC-10 with a selectivity to acetophenone of 97.0% is even comparable to the results of some metal-doped catalysts, which may be attributed to the hierarchical porous structures and plenty of C-NXactive sites. This work provides a facile and novel strategy for the construction of metal-free and N-rich carbon materials with excellent catalytic activity for the selective oxidation of ethylbenzene.

Copyright © The Royal Society of Chemistry and the Centre National de la Recherche Scientifique 2021.

Institution

(Yi, Zhang, Xiang, Chen, Cheng, Liu) Advanced Catalytic Engineering Research Center of the Ministry of Education, College of Chemistry and Chemical Engineering, Hunan University, Hunan, Changsha 410082, China

Publisher

Royal Society of Chemistry

Emtree Heading

adsorption; article; Brunauer Emmett Teller method; catalysis; catalyst; desorption; oxidation; performance; pyrolysis; Raman spectrometry; surface area; *tofu; transmission electron microscopy; X ray diffraction; acetophenone; *carbon; ethylbenzene; nitrogen.

Drug Index Terms

acetophenone; *carbon; ethylbenzene; nitrogen.

Other Index Terms

adsorption; Article; Brunauer Emmett Teller method; catalysis; catalyst; desorption; oxidation; performance; pyrolysis; Raman spectrometry; surface area; *tofu; transmission electron microscopy; X ray diffraction.

Link to the Ovid Full Text or citation:

[Click here for full text options](https://ovidsp.ovid.com/ovidweb.cgi?T=JS&CSC=Y&NEWS=N&PAGE=fulltext&D=emed22&AN=636014442)

Link to the External Link Resolver:

[SFX](https://sfx-86scu.hosted.exlibrisgroup.com.cn/86scu?sid=OVID:embase&id=pmid:&id=doi:10.1039%2Fd1nj02548a&issn=11440546&isbn=&volume=45&issue=36&spage=16469&pages=16469-16476&date=2021&title=New+Journal+of+Chemistry&atitle=N-rich+porous+carbon+catalysts+with+huge+surface+areas+from+bean+curd+activated+by+K2CO3&aulast=Yi&pid=<author>Yi+C.%3BZhang+L.%3BXiang+G.%3BChen+X.%3BCheng+N.%3BLiu+Z.<%2Fauthor><AN>636014442<%2FAN><DT>Article<%2FDT>)

59.

Novel amorphous solid dispersion based on natural deep eutectic solvent for enhancing delivery of anti-tumor RA-XII by oral administration in rats.

Liu M., Lai Z., Zhu L., Ding X., Tong X., Wang Z., Bi Q., Tan N.

European Journal of Pharmaceutical Sciences. 166 (no pagination), 2021. Article Number: 105931. Date of Publication: 01 Nov 2021.

AN: 2014001927

At present, oral chemotherapy showing the advantages of non-invasiveness, convenience, and high patient compliance, is gradually replacing traditional intravenous chemotherapy to treat patients with cancer. RA-XII, a unique natural cyclopeptide, exhibits various biological activities, such as anti-tumor, anti-angiogenic, and anti-metastatic activities. Designing an orally available formulation of RA-XII is of great importance in the development of clinically useful anticancer agents. However, RA-XII shows low oral bioavailability in rats due to its poor solubility and low permeability. To overcome these limitations, in this work, a natural deep eutectic solvent (NADES) was designed to efficiently deliver RA-XII by oral administration. A novel NADES composed of betaine and mandelic acid in the molar ratio of 1:1 (Bet-Man NADES) was successfully prepared based on a binary phase diagram of Bet and Man. Acute toxicity studies indicated that Bet-Man NADES was well tolerated with acceptable toxicity. In Bet-Man NADES solutions, the solubility of RA-XII was increased by up to 17.54-fold, and the diffusion and permeability of RA-XII carried out in a Franz cell was also significantly improved 10.35 times. In terms of biopharmaceutical classification this is translated into a change for RA-XII from class IV to class II systems. More importantly, Bet-Man NADES was transferred into the solid formulation by the inclusion of a polymer, and amorphous solid dispersions based on Bet-Man NADES (PVP K30/NADES/RA-XII, ASDs) were successfully prepared to improve uniformity, apparent solubility, dissolution, and cytotoxicity in vitro. Consequently, the oral bioavailability of RA-XII in NADES solutions and ASDs was enhanced by approximately 11.58 and 7.56 times compared with that of pure RA-XII in 0.5% CMC[sbnd]Na. Thus, it can be seen that a natural deep eutectic solvent and its modified amorphous solid dispersions are appropriate novel strategies for improving dissolution rate and bioavailability of poor soluble natural products such as RA-XII.

Copyright © 2021

PMID

34256100 [<https://www.ncbi.nlm.nih.gov/pubmed/?term=34256100>]

Institution

(Liu, Lai, Zhu, Ding, Tong, Wang, Bi, Tan) Department of TCMs Pharmaceuticals, School of Traditional Chinese Pharmacy, China Pharmaceutical University, Nanjing 211198, China

Publisher

Elsevier B.V.

Emtree Heading

acute toxicity; animal experiment; animal tissue; *antineoplastic activity; apparent permeability; area under the curve; article; carbon nuclear magnetic resonance; controlled study; differential scanning calorimetry; *dispersion; drug bioavailability; drug cytotoxicity; *drug delivery system; drug diffusion; drug formulation; drug half life; drug penetration; drug solubility; enterohepatic circulation; eutectic point; female; Fourier transform infrared spectroscopy; human; human cell; in vitro study; in vivo study; male; maximum plasma concentration; mean residence time; mouse; nonhuman; oral absorption; plasma concentration-time curve; polarization microscopy; proton nuclear magnetic resonance; Raman spectrometry; rat; scanning electron microscopy; time to maximum plasma concentration; toxicity testing; water solubility; X ray powder diffraction; *antineoplastic agent/po [Oral Drug Administration]; *antineoplastic agent/pr [Pharmaceutics]; *antineoplastic agent/pk [Pharmacokinetics]; betaine; *deep eutectic solvent/to [Drug Toxicity]; mandelic acid; *natural product/pr [Pharmaceutics]; *natural product/pk [Pharmacokinetics]; polymer; solubilizer; unclassified drug; calorimeter; nuclear magnetic resonance spectrometer; polarization microscope; scanning electron microscope; spectrophotometer; X-ray diffractometer; *ra xii/po [Oral Drug Administration]; *ra xii/pr [Pharmaceutics]; *ra xii/pk [Pharmacokinetics].

Candidate Terms

*ra xii / *oral drug administration / *pharmaceutics / *pharmacokinetics [drug term]

Device Index Terms

calorimeter; nuclear magnetic resonance spectrometer; polarization microscope; scanning electron microscope; spectrophotometer; X-ray diffractometer.

Drug Index Terms

*antineoplastic agent / *oral drug administration / *pharmaceutics / *pharmacokinetics; betaine; *deep eutectic solvent / *drug toxicity; mandelic acid; *natural product / *pharmaceutics / *pharmacokinetics; polymer; solubilizer; unclassified drug.

Other Index Terms

acute toxicity; animal experiment; animal tissue; *antineoplastic activity; apparent permeability; area under the curve; Article; carbon nuclear magnetic resonance; controlled study; differential scanning calorimetry; *dispersion; drug bioavailability; drug cytotoxicity; *drug delivery system; drug diffusion; drug formulation; drug half life; drug penetration; drug solubility; enterohepatic circulation; eutectic point; female; Fourier transform infrared spectroscopy; human; human cell; in vitro study; in vivo study; male; maximum plasma concentration; mean residence time; mouse; nonhuman; oral absorption; plasma concentration-time curve; polarization microscopy; proton nuclear magnetic resonance; Raman spectrometry; rat; scanning electron microscopy; time to maximum plasma concentration; toxicity testing; water solubility; X ray powder diffraction.

Drug Trade Name

ra xii

Link to the Ovid Full Text or citation:

[Click here for full text options](https://ovidsp.ovid.com/ovidweb.cgi?T=JS&CSC=Y&NEWS=N&PAGE=fulltext&D=emed22&AN=2014001927)

Link to the External Link Resolver:

[SFX](https://sfx-86scu.hosted.exlibrisgroup.com.cn/86scu?sid=OVID:embase&id=pmid:34256100&id=doi:10.1016%2Fj.ejps.2021.105931&issn=09280987&isbn=&volume=166&issue=&spage=105931&pages=&date=2021&title=European+Journal+of+Pharmaceutical+Sciences&atitle=Novel+amorphous+solid+dispersion+based+on+natural+deep+eutectic+solvent+for+enhancing+delivery+of+anti-tumor+RA-XII+by+oral+administration+in+rats&aulast=Liu&pid=<author>Liu+M.%3BLai+Z.%3BZhu+L.%3BDing+X.%3BTong+X.%3BWang+Z.%3BBi+Q.%3BTan+N.<%2Fauthor><AN>2014001927<%2FAN><DT>Article<%2FDT>)

60.

Nanoparticles in dentistry: A comprehensive review.

Moraes G., Zambom C., Siqueira W.L.

Pharmaceuticals. 14(8) (no pagination), 2021. Article Number: 752. Date of Publication: August 2021.

AN: 2013305229

In recent years, nanoparticles (NPs) have been receiving more attention in dentistry. Their advantageous physicochemical and biological properties can improve the diagnosis, prevention, and treatment of numerous oral diseases, including dental caries, periodontal diseases, pulp and periapical lesions, oral candidiasis, denture stomatitis, hyposalivation, and head, neck, and oral cancer. NPs can also enhance the mechanical and microbiological properties of dental prostheses and implants and can be used to improve drug delivery through the oral mucosa. This paper reviewed studies from 2015 to 2020 and summarized the potential applications of different types of NPs in the many fields of dentistry.

Copyright © 2021 by the authors. Licensee MDPI, Basel, Switzerland.

Institution

(Moraes, Siqueira) College of Dentistry, University of Saskatchewan, Saskatoon, SK S7N 5E4, Canada (Moraes) Department of Dentistry, State University of Ponta Grossa, Ponta Grossa 84030-900, Brazil

(Zambom) Department of Biochemistry and Organic Chemistry, Institute of Chemistry, UNESP-Sao Paulo State University, Araraquara 14800-060, Brazil

Publisher

MDPI

Emtree Heading

bacterial colonization; bactericidal activity; biocompatibility; biodegradability; cell proliferation; cytotoxicity; *dental caries; *dentistry; early diagnosis; genotoxicity; head and neck cancer; human; hydrophilicity; hyposalivation; longevity; mineralization; mouth cancer; *nanotechnology; optical coherence tomography; osseointegration; periimplantitis/di [Diagnosis]; periodontal disease/di [Diagnosis]; physical chemistry; prevalence; review; stomatitis/di [Diagnosis]; surface enhanced Raman spectroscopy; tensile strength; thrush/di [Diagnosis]; calcium fluoride; *nanoparticle; poly(methyl methacrylate); titanium dioxide; zirconium oxide; immunosensor.

Device Index Terms

immunosensor.

Drug Index Terms

calcium fluoride; *nanoparticle; poly(methyl methacrylate); titanium dioxide; zirconium oxide.

Other Index Terms

bacterial colonization; bactericidal activity; biocompatibility; biodegradability; cell proliferation; cytotoxicity; *dental caries; *dentistry; early diagnosis; genotoxicity; head and neck cancer; human; hydrophilicity; hyposalivation; longevity; mineralization; mouth cancer; *nanotechnology; optical coherence tomography; osseointegration; periimplantitis / diagnosis; periodontal disease / diagnosis; physical chemistry; prevalence; Review; stomatitis / diagnosis; surface enhanced Raman spectroscopy; tensile strength; thrush / diagnosis.

Link to the Ovid Full Text or citation:

[Click here for full text options](https://ovidsp.ovid.com/ovidweb.cgi?T=JS&CSC=Y&NEWS=N&PAGE=fulltext&D=emed22&AN=2013305229)

Link to the External Link Resolver:

[SFX](https://sfx-86scu.hosted.exlibrisgroup.com.cn/86scu?sid=OVID:embase&id=pmid:&id=doi:10.3390%2Fph14080752&issn=14248247&isbn=&volume=14&issue=8&spage=752&pages=&date=2021&title=Pharmaceuticals&atitle=Nanoparticles+in+dentistry%3A+A+comprehensive+review&aulast=Moraes&pid=<author>Moraes+G.%3BZambom+C.%3BSiqueira+W.L.<%2Fauthor><AN>2013305229<%2FAN><DT>Review<%2FDT>)

61.

Raman spectral cytopathology for cancer diagnostic applications.

Traynor D., Behl I., O'Dea D., Bonnier F., Nicholson S., O'Connell F., Maguire A., Flint S., Galvin S., Healy C.M., Martin C.M., O'Leary J.J., Malkin A., Byrne H.J., Lyng F.M.

Nature Protocols. 16(7) (pp 3716-3735), 2021. Date of Publication: July 2021.

AN: 2012386363

Raman spectroscopy can provide a rapid, label-free, nondestructive measurement of the chemical fingerprint of a sample and has shown potential for cancer screening and diagnosis. Here we report a protocol for Raman microspectroscopic analysis of different exfoliative cytology samples (cervical, oral and lung), covering sample preparation, spectral acquisition, preprocessing and data analysis. The protocol takes 2 h 20 min for sample preparation, measurement and data preprocessing and up to 8 h for a complete analysis. A key feature of the protocol is that it uses the same sample preparation procedure as commonly used in diagnostic cytology laboratories (i.e., liquid-based cytology on glass slides), ensuring compatibility with clinical workflows. Our protocol also covers methods to correct for the spectral contribution of glass and sample pretreatment methods to remove contaminants (such as blood and mucus) that can obscure spectral features in the exfoliated cells and lead to variability. The protocol establishes a standardized clinical routine allowing the collection of highly reproducible data for Raman spectral cytopathology for cancer diagnostic applications for cervical and lung cancer and for monitoring suspicious lesions for oral cancer.

Copyright © 2021, The Author(s), under exclusive licence to Springer Nature Limited.

PMID

34117476 [<https://www.ncbi.nlm.nih.gov/pubmed/?term=34117476>]

Author NameID

Lyng, Fiona M.; ORCID: <https://orcid.org/0000-0002-9876-963X>

Institution

(Traynor, Behl, O'Dea, Lyng) Centre for Radiation and Environmental Science, FOCAS Research Institute, Technological University Dublin, Dublin, Ireland (Traynor, Behl, Lyng) School of Physics & Clinical & Optometric Sciences, Technological University Dublin, Dublin, Ireland

(O'Dea, Malkin) School of Biological and Health Sciences, Technological University Dublin, Dublin, Ireland

(Bonnier) EA 6295 Nanomedicaments et Nanosondes, Universite de Tours, Tours, France

(Nicholson, O'Connell, Maguire) St James's Hospital, Dublin, Ireland

(Flint, Galvin, Healy) Oral Medicine Unit, Dublin Dental University Hospital, Trinity College, Dublin, Ireland

(Martin, O'Leary) Discipline of Histopathology, University of Dublin Trinity College, Dublin, Ireland

(Martin, O'Leary) Emer Casey Molecular Pathology Research Laboratory, The Coombe Women and Infants University Hospital, Dublin, Ireland

(Martin, O'Leary, Lyng) CERVIVA Research Consortium, Dublin, Ireland

(Byrne) FOCAS Research Institute, Technological University Dublin, Dublin, Ireland

Publisher

Nature Research

Emtree Heading

bronchoscopy; cancer screening; cancer staging; cytology; *cytopathology; data analysis; discriminant analysis; dizziness; eye irritation; fine needle aspiration biopsy; human; lung cancer; *malignant neoplasm/di [Diagnosis]; microscopy; mouth cancer; mucus; principal component analysis; *Raman spectrometry; refraction index; review; signal noise ratio; skin irritation; tissue microarray; urine cytology; uterine cervix cancer; vagina flora; workflow; lactic acid/ec [Endogenous Compound]; optical tweezers.

Device Index Terms

optical tweezers.

Drug Index Terms

lactic acid / endogenous compound.

Other Index Terms

bronchoscopy; cancer screening; cancer staging; cytology; *cytopathology; data analysis; discriminant analysis; dizziness; eye irritation; fine needle aspiration biopsy; human; lung cancer; *malignant neoplasm / *diagnosis; microscopy; mouth cancer; mucus; principal component analysis; *Raman spectrometry; refraction index; Review; signal noise ratio; skin irritation; tissue microarray; urine cytology; uterine cervix cancer; vagina flora; workflow.

Link to the Ovid Full Text or citation:

[Click here for full text options](https://ovidsp.ovid.com/ovidweb.cgi?T=JS&CSC=Y&NEWS=N&PAGE=fulltext&D=emed22&AN=2012386363)

Link to the External Link Resolver:

[SFX](https://sfx-86scu.hosted.exlibrisgroup.com.cn/86scu?sid=OVID:embase&id=pmid:34117476&id=doi:10.1038%2Fs41596-021-00559-5&issn=17542189&isbn=&volume=16&issue=7&spage=3716&pages=3716-3735&date=2021&title=Nature+Protocols&atitle=Raman+spectral+cytopathology+for+cancer+diagnostic+applications&aulast=Traynor&pid=<author>Traynor+D.%3BBehl+I.%3BO'Dea+D.%3BBonnier+F.%3BNicholson+S.%3BO'Connell+F.%3BMaguire+A.%3BFlint+S.%3BGalvin+S.%3BHealy+C.M.%3BMartin+C.M.%3BO'Leary+J.J.%3BMalkin+A.%3BByrne+H.J.%3BLyng+F.M.<%2Fauthor><AN>2012386363<%2FAN><DT>Review<%2FDT>)

62.

Risk prediction by Raman spectroscopy for disease-free survival in oral cancers.

Bhattacharjee A., Hole A., Malik A., Sahu A., Singh S.P., Deshmukh A., Nair S., Chaturvedi P., Murali Krishna C.

Lasers in Medical Science. 36(8) (pp 1691-1700), 2021. Date of Publication: October 2021.

AN: 2010693163

In the present study, the potential of Raman spectroscopy (RS) in predicting disease-free survival (DFS) in oral cancer patients has been explored. Raman spectra were obtained from the tumor and contralateral regions of 94 oral squamous cell carcinoma patients. These patients were managed surgically and recommended for adjuvant therapy. The Cox proportional survival analysis was carried out to identify the spectral regions that can be correlated to DFS. The survival analysis was performed with 95% confidence intervals, hazard ratio, and p-values in the 1200-1800 cm-1 spectral region. Out of a total of 182 spectral points, 76 were found to be correlating with DFS, suggesting their utility to predict the patient outcome. The cut-off points of each correlating RS-point values were defined and tested towards predicting the DFS. The performance of predicting the power of spectral points was validated through Brier value, and it was found to be closer to the actual progression. The 76 spectral points identified from the tumors have the potential to accurately predict DFS in oral squamous cell carcinoma through a relatively simplistic prediction model in the absence of confounding factors.

Copyright © 2021, The Author(s), under exclusive licence to Springer-Verlag London Ltd. part of Springer Nature.

PMID

33661401 [<https://www.ncbi.nlm.nih.gov/pubmed/?term=33661401>]

Author NameID

Murali Krishna C.; ORCID: <https://orcid.org/0000-0002-4974-8533>

Institution

(Bhattacharjee, Chaturvedi) Section of Biostatistics, Centre for Cancer Epidemiology, Tata Memorial Centre, Mumbai, India (Bhattacharjee) Homi Bhabha National Institute, Mumbai, India

(Hole, Sahu, Singh, Deshmukh, Murali Krishna) Chilakapati Laboratory, Advanced Center for Training, Research, and Education in Cancer (ACTREC), Tata Memorial Center, Kharghar, Sector '22', Navi Mumbai 410210, India

(Malik, Nair, Chaturvedi, Murali Krishna) Head and Neck Surgical Oncology, Tata Memorial Center, Mumbai, India

(Malik, Nair, Chaturvedi) Department of Head Neck Surgery, Tata Memorial Centre, Mumbai, India

Publisher

Springer Science and Business Media Deutschland GmbH

Emtree Heading

article; cancer adjuvant therapy; cancer model; cancer patient; cancer surgery; confidence interval; confounding variable; *disease free survival; hazard ratio; human; major clinical study; *mouth squamous cell carcinoma; prediction; proportional hazards model; *Raman spectrometry; *risk assessment; survival analysis; spectrometer; HE-785; raman spectrometer.

Candidate Terms

HE-785 [device term] Raman spectrometer [device term]

Device Index Terms

spectrometer.

Other Index Terms

Article; cancer adjuvant therapy; cancer model; cancer patient; cancer surgery; confidence interval; confounding variable; *disease free survival; hazard ratio; human; major clinical study; *mouth squamous cell carcinoma; prediction; proportional hazards model; *Raman spectrometry; *risk assessment; survival analysis.

Link to the Ovid Full Text or citation:

[Click here for full text options](https://ovidsp.ovid.com/ovidweb.cgi?T=JS&CSC=Y&NEWS=N&PAGE=fulltext&D=emed22&AN=2010693163)

Link to the External Link Resolver:

[SFX](https://sfx-86scu.hosted.exlibrisgroup.com.cn/86scu?sid=OVID:embase&id=pmid:33661401&id=doi:10.1007%2Fs10103-021-03276-3&issn=02688921&isbn=&volume=36&issue=8&spage=1691&pages=1691-1700&date=2021&title=Lasers+in+Medical+Science&atitle=Risk+prediction+by+Raman+spectroscopy+for+disease-free+survival+in+oral+cancers&aulast=Bhattacharjee&pid=<author>Bhattacharjee+A.%3BHole+A.%3BMalik+A.%3BSahu+A.%3BSingh+S.P.%3BDeshmukh+A.%3BNair+S.%3BChaturvedi+P.%3BMurali+Krishna+C.<%2Fauthor><AN>2010693163<%2FAN><DT>Article<%2FDT>)

63.

Imaging of Oral SCC Cells by Raman Micro-Spectroscopy Technique.

Kinoshita H., Miyoshi N., Ogasawara T.

Molecules (Basel, Switzerland). 26(12) (no pagination), 2021. Date of Publication: 15 Jun 2021.

AN: 635510570

We used Raman micro-spectroscopy technique to analyze the molecular changes associated with oral squamous cell carcinoma (SCC) cells in the form of frozen tissue. Previously, Raman micro-spectroscopy technique on human tissue was mainly based on spectral analysis, but we worked on imaging of molecular structure. In this study, we evaluated the distribution of four components at the cell level (about 10 mum) to describe the changes in protein and molecular structures of protein belonging to malignant tissue. We analyzed ten oral SCC samples of five patients without special pretreatments of the use of formaldehyde. We obtained cell level images of the oral SCC cells at various components (peak at 935 cm-1: proline and valine, 1004 cm-1: phenylalanine, 1223 cm-1: nucleic acids, and 1650 cm-1: amide I). These mapping images of SCC cells showed the distribution of nucleic acids in the nuclear areas; meanwhile, proline and valine, phenylalanine, and amide I were detected in the cytoplasm areas of the SCC cells. Furthermore, the peak of amide I in the cancer area shifts to the higher wavenumber side, which indicates the alpha-helix component may decrease in its relative amounts of protein in the beta-sheet or random coil conformation. Imaging of SCC cells with Raman micro-spectroscopy technique indicated that such a new observation of cancer cells is useful for analyzing the detailed distribution of various molecular conformation within SCC cells.

PMID

34203597 [<https://www.ncbi.nlm.nih.gov/pubmed/?term=34203597>]

Institution

(Kinoshita, Ogasawara) Division of Dentistry and Oral Surgery, Fukui General Hospital, Egami, Fukui 910-8561, Japan (Kinoshita) Department of Rehabilitation Medicine, Fukui College of Health Sciences, Egami, Fukui 910-3190, Japan

(Miyoshi) Department of Gastroenterology, Faculty of Medicine, Tsukuba University, Tennoudai, Tsukuba 305-8575, Japan

Publisher

NLM (Medline)

Emtree Heading

cell nucleus; conformation; cytoplasm; diagnostic imaging; human; image processing; Japan; metabolism; mouth tumor; pathology; procedures; Raman spectrometry; squamous cell carcinoma.

Other Index Terms

cell nucleus; conformation; cytoplasm; diagnostic imaging; human; image processing; Japan; metabolism; mouth tumor; pathology; procedures; Raman spectrometry; squamous cell carcinoma.

Link to the Ovid Full Text or citation:

[Click here for full text options](https://ovidsp.ovid.com/ovidweb.cgi?T=JS&CSC=Y&NEWS=N&PAGE=fulltext&D=emed22&AN=635510570)

Link to the External Link Resolver:

[SFX](https://sfx-86scu.hosted.exlibrisgroup.com.cn/86scu?sid=OVID:embase&id=pmid:34203597&id=doi:10.3390%2Fmolecules26123640&issn=14203049&isbn=&volume=26&issue=12&spage=3640&pages=&date=2021&title=Molecules+(Basel%2C+Switzerland)&atitle=Imaging+of+Oral+SCC+Cells+by+Raman+Micro-Spectroscopy+Technique&aulast=Kinoshita&pid=<author>Kinoshita+H.%3BMiyoshi+N.%3BOgasawara+T.<%2Fauthor><AN>635510570<%2FAN><DT>Article<%2FDT>)

64.

Experimental study on needle insertion force to minimize tissue deformation in tongue tissue.

Aaboubout Y., Nunes Soares M.R., Barroso E.M., van der Sar L.C., Bocharnikov A., Usenov I., Artyushenko V., Caspers P.J., Koljenovic S., Bakker Schut T.C., van den Dobbelsteen J.J., Puppels G.J.

Medical Engineering and Physics. 97 (pp 40-46), 2021. Date of Publication: November 2021.

AN: 2015062758

This study reports on the effects of insertion velocity, needle tip geometry and needle diameter on tissue deformation and maximum insertion force. Moreover, the effect of multiple insertions with the same needle on the maximum insertion force is reported. The tissue deformation and maximum insertion force strongly depend on the insertion velocity and the tip geometry. No correlation was found between the outer diameter and the maximum insertion force for small needles (30G - 32G). The endurance experiments showed no remarkable difference in the maximum insertion force during 100 insertions.

Copyright © 2021

PMID

34756337 [<https://www.ncbi.nlm.nih.gov/pubmed/?term=34756337>]

Author NameID

Koljenovic S.; ORCID: <https://orcid.org/0000-0002-5185-3455> Caspers P.J.; ORCID: <https://orcid.org/0000-0002-6122-5049>

Puppels G.J.; ORCID: <https://orcid.org/0000-0001-8017-1923>

Institution

(Aaboubout, Nunes Soares, Barroso, van der Sar, Koljenovic) Department of Pathology, Erasmus MC, University Medical Center Rotterdam, Wytemaweg 80 CN, Rotterdam 3015, Netherlands (Aaboubout) Department of Otorhinolaryngology and Head and Neck Surgery, Erasmus MC, University Medical Center Rotterdam, Netherlands

(Barroso) Department of Oral and Maxillofacial Surgery, Erasmus MC, University Medical Center Rotterdam, Netherlands

(Barroso, Caspers, Bakker Schut, Puppels) Department of Dermatology, Erasmus MC, University Medical Center Rotterdam, Netherlands

(Bocharnikov, Usenov, Artyushenko) Art Photonics GmbH, Berlin, Germany

(van den Dobbelsteen) Department of Biomechanical Engineering, Delft University of Technology, Delft, Netherlands

Publisher

Elsevier Ltd

Emtree Heading

animal tissue; article; controlled study; equipment design; experimental study; *medical parameters; mouth cavity; *mouth tissue; nonhuman; *tissue and organ preparation and culture; gauge; needle; insertion velocity; maximum insertion force; needle diameter; *needle insertion force; needle tip geometry; *tissue deformation; *tongue tissue.

Candidate Terms

insertion velocity [other term] maximum insertion force [other term]

needle diameter [other term]

*needle insertion force [other term]

needle tip geometry [other term]

*tissue deformation [other term]

*tongue tissue [other term]

Device Index Terms

gauge; needle.

Other Index Terms

animal tissue; Article; controlled study; equipment design; experimental study; *medical parameters; mouth cavity; *mouth tissue; nonhuman; *tissue and organ preparation and culture.

Link to the Ovid Full Text or citation:

[Click here for full text options](https://ovidsp.ovid.com/ovidweb.cgi?T=JS&CSC=Y&NEWS=N&PAGE=fulltext&D=emed22&AN=2015062758)

Link to the External Link Resolver:

[SFX](https://sfx-86scu.hosted.exlibrisgroup.com.cn/86scu?sid=OVID:embase&id=pmid:34756337&id=doi:10.1016%2Fj.medengphy.2021.10.003&issn=13504533&isbn=&volume=97&issue=&spage=40&pages=40-46&date=2021&title=Medical+Engineering+and+Physics&atitle=Experimental+study+on+needle+insertion+force+to+minimize+tissue+deformation+in+tongue+tissue&aulast=Aaboubout&pid=<author>Aaboubout+Y.%3BNunes+Soares+M.R.%3BBarroso+E.M.%3Bvan+der+Sar+L.C.%3BBocharnikov+A.%3BUsenov+I.%3BArtyushenko+V.%3BCaspers+P.J.%3BKoljenovic+S.%3BBakker+Schut+T.C.%3Bvan+den+Dobbelsteen+J.J.%3BPuppels+G.J.<%2Fauthor><AN>2015062758<%2FAN><DT>Article<%2FDT>)

65.

RuCo alloy trifunctional electrocatalysts with ratio-dependent activity for Zn-air batteries and self-powered water splitting.

Pei Y., He W., Wang M., Sun T., Hu L., Zhu J., Tan Y., Wang J.

Chemical Communications. 57(12) (pp 1498-1501), 2021. Date of Publication: 11 Feb 2021.

AN: 634206138

Herein, we reported a RuCo alloy with nitrogen-doped porous carbon (RuCo/NPC) as efficient trifunctional electrocatalysts for Zn-air batteries and water splitting. The versatility and catalytic activity of this catalyst is achieved by adjusting the Ru/Co ratio. The as-assembled Zn-air battery and overall water splitting with RuCo/NPC present outstanding catalytic performances.

Copyright © The Royal Society of Chemistry 2021.

PMID

33443250 [<https://www.ncbi.nlm.nih.gov/pubmed/?term=33443250>]

Institution

(Pei, He, Wang) State Key Laboratory of High-Performance Ceramics and Superfine Microstructure, Shanghai Institute of Ceramics, Chinese Academy of Sciences, 585 Heshuo Road, Shanghai 201899, China (Pei, Wang) Center of Materials Science and Optoelectronics Engineering, University of Chinese Academy of Sciences, Beijing 100049, China

(Wang, Wang, Sun, Hu, Zhu, Tan) College of Chemistry and Chemical Engineering, Nantong University, Nantong 226019, China

Publisher

Royal Society of Chemistry

Emtree Heading

article; *catalyst; cost benefit analysis; electrochemical analysis; *electrochemistry; electrolysis; high resolution transmission electron microscopy; porosity; Raman spectrometry; surface property; X ray diffraction; *alloy; carbon; *carbon monoxide; metal nanoparticle; nitrogen; *ruthenium; water; *zinc.

Drug Index Terms

*alloy; carbon; *carbon monoxide; metal nanoparticle; nitrogen; *ruthenium; water; *zinc.

Other Index Terms

Article; *catalyst; cost benefit analysis; electrochemical analysis; *electrochemistry; electrolysis; high resolution transmission electron microscopy; porosity; Raman spectrometry; surface property; X ray diffraction.

Link to the Ovid Full Text or citation:

[Click here for full text options](https://ovidsp.ovid.com/ovidweb.cgi?T=JS&CSC=Y&NEWS=N&PAGE=fulltext&D=emed22&AN=634206138)

Link to the External Link Resolver:

[SFX](https://sfx-86scu.hosted.exlibrisgroup.com.cn/86scu?sid=OVID:embase&id=pmid:33443250&id=doi:10.1039%2Fd0cc07565e&issn=13597345&isbn=&volume=57&issue=12&spage=1498&pages=1498-1501&date=2021&title=Chemical+Communications&atitle=RuCo+alloy+trifunctional+electrocatalysts+with+ratio-dependent+activity+for+Zn-air+batteries+and+self-powered+water+splitting&aulast=Pei&pid=<author>Pei+Y.%3BHe+W.%3BWang+M.%3BSun+T.%3BHu+L.%3BZhu+J.%3BTan+Y.%3BWang+J.<%2Fauthor><AN>634206138<%2FAN><DT>Article<%2FDT>)

66.

Raman spectroscopy and surface-enhanced Raman spectroscopy (SERS) spectra of salivary glands carcinoma, tumor and healthy tissues and their homogenates analyzed by chemometry: Towards development of the novel tool for clinical diagnosis.

Czaplicka M., Kowalska A.A., Nowicka A.B., Kurzydlowski D., Gronkiewicz Z., Machulak A., Kukwa W., Kaminska A.

Analytica Chimica Acta. 1177 (no pagination), 2021. Article Number: 338784. Date of Publication: 08 Sep 2021.

AN: 2013287707

In this study, two approaches to salivary glands studies are presented: Raman imaging (RI) of tissue cross-section and surface-enhanced Raman spectroscopy (SERS) of tissue homogenates prepared according to elaborated protocol. Collected and analyzed data demonstrate the significant potential of SERS combined with multivariate analysis for distinguishing carcinoma or tumor from the normal salivary gland tissues as a rapid, label-free tool in cancer detection in oncological diagnostics. Raman imaging allows a detailed analysis of the cell wall's chemical composition; thus, the compound's distribution can be semi-quantitatively analyzed, while SERS of tissue homogenates allow for detailed analysis of all moieties forming these tissues. In this sense, SERS is more sensitive and reliable to study any changes in the area of infected tissues. Principal component analysis (PCA), as an unsupervised pattern recognition method, was used to identify the differences in the SERS salivary glands homogenates. The partial least squares-discriminant analysis (PLS-DA), the supervised pattern classification technique, was also used to strengthen further the computed model based on the latent variables in the SERS spectra. Moreover, the chemometric quantification of obtained data was analyzed using principal component regression (PCR) multivariate calibration. The presented data prove that the PCA algorithm allows for 91% in seven following components and the determination between healthy and tumor salivary gland homogenates. The PCR and PLS-DA methods predict 90% and 95% of the variance between the studied groups (in 6 components and 4 factors, respectively). Moreover, according to calculated RMSEC (RMSEP), R2C (R2P) values and correlation accuracy (based on the ROC curve), the PLS-DA model fits better for the studied data. Thus, SERS methods combined with PLS-DA analysis can be used to differentiate healthy, neoplastic, and mixed tissues as a competitive tool in relation to the commonly used method of histopathological staining of tumor tissue.

Copyright © 2021 Elsevier B.V.

PMID

34482902 [<https://www.ncbi.nlm.nih.gov/pubmed/?term=34482902>]

Institution

(Czaplicka, Kowalska, Nowicka, Kaminska) Institute of Physical Chemistry, Polish Academy of Sciences, Kasprzaka 44/52, Warsaw 01-224, Poland (Kurzydlowski) Cardinal Stefan Wyszynski University in Warsaw, Dewajtis 5, Warsaw 01-815, Poland

(Gronkiewicz, Machulak, Kukwa) Szpital Czerniakowski, Medical University of Warsaw, Zwirki I Wigury 61, Warsaw 02-091, Poland

Publisher

Elsevier B.V.

Emtree Heading

article; calibration; chemical composition; *chemometrics; clinical article; controlled study; discriminant analysis; human; human tissue; multivariate analysis; partial least squares regression; pattern recognition; principal component analysis; *Raman spectrometry; reliability; salivary gland; *salivary gland carcinoma/di [Diagnosis]; *salivary gland tumor/di [Diagnosis]; sensitivity analysis; structural equation modeling; *surface enhanced Raman spectroscopy; *tissue homogenate.

Other Index Terms

Article; calibration; chemical composition; *chemometrics; clinical article; controlled study; discriminant analysis; human; human tissue; multivariate analysis; partial least squares regression; pattern recognition; principal component analysis; *Raman spectrometry; reliability; salivary gland; *salivary gland carcinoma / *diagnosis; *salivary gland tumor / *diagnosis; sensitivity analysis; structural equation modeling; *surface enhanced Raman spectroscopy; *tissue homogenate.

Link to the Ovid Full Text or citation:

[Click here for full text options](https://ovidsp.ovid.com/ovidweb.cgi?T=JS&CSC=Y&NEWS=N&PAGE=fulltext&D=emed22&AN=2013287707)

Link to the External Link Resolver:

[SFX](https://sfx-86scu.hosted.exlibrisgroup.com.cn/86scu?sid=OVID:embase&id=pmid:34482902&id=doi:10.1016%2Fj.aca.2021.338784&issn=00032670&isbn=&volume=1177&issue=&spage=338784&pages=&date=2021&title=Analytica+Chimica+Acta&atitle=Raman+spectroscopy+and+surface-enhanced+Raman+spectroscopy+(SERS)+spectra+of+salivary+glands+carcinoma%2C+tumor+and+healthy+tissues+and+their+homogenates+analyzed+by+chemometry%3A+Towards+development+of+the+novel+tool+for+clinical+diagnosis&aulast=Czaplicka&pid=<author>Czaplicka+M.%3BKowalska+A.A.%3BNowicka+A.B.%3BKurzydlowski+D.%3BGronkiewicz+Z.%3BMachulak+A.%3BKukwa+W.%3BKaminska+A.<%2Fauthor><AN>2013287707<%2FAN><DT>Article<%2FDT>)

67.

Developing an algorithm for discriminating oral cancerous and normal tissues using raman spectroscopy.

Sharma M., Jeng M.-J., Young C.-K., Huang S.-F., Chang L.-B.

Journal of Personalized Medicine. 11(11) (no pagination), 2021. Article Number: 1165. Date of Publication: November 2021.

AN: 2014614150

The aim of this study was to investigate the clinical potential of Raman spectroscopy (RS) in detecting oral squamous cell carcinoma (OSCC) in tumor and healthy tissues in surgical resection specimens during surgery. Raman experiments were performed on cryopreserved specimens from patients with OSCC. Univariate and multivariate analysis was performed based on the fingerprint region (700-1800 cm-1) of the Raman spectra. One hundred thirty-one ex-vivo Raman experiments were performed on 131 surgical resection specimens obtained from 67 patients. The principal component analysis (PCA) and partial least square (PLS) methods with linear discriminant analysis (LDA) were applied on an independent validation dataset. Both models were able to differentiate between the tissue types, but PLS-LDA showed 100% accuracy, sensitivity, and specificity. In this study, Raman measurements of fresh resection tissue specimens demonstrated that OSCC had significantly higher nucleic acid, protein, and several amino acid contents than adjacent healthy tissues. The specific spectral information obtained in this study can be used to develop an in vivo Raman spectroscopic method for the tumor-free resection boundary during surgery.

Copyright © 2021 by the authors. Licensee MDPI, Basel, Switzerland.

Institution

(Sharma, Jeng, Chang) Department of Electronic Engineering, Chang Gung University, Taoyuan 333, Taiwan (Republic of China) (Jeng, Huang, Chang) Department of Otolaryngology-Head and Neck Surgery, Chang Gung Memorial Hospital, Linkou 244, Taiwan (Republic of China)

(Young) Department of Otolaryngology, Head and Neck Surgery, Chang Gung Memorial Hospital, Keelung Branch, Keelung 204, Taiwan (Republic of China)

(Huang) Department of Public Health, Chang Gung University, Taoyuan 333, Taiwan (Republic of China)

(Chang) Green Technology Research Center, Chang Gung University, Taoyuan 333, Taiwan (Republic of China)

Publisher

MDPI

Emtree Heading

*algorithm; area under the curve; article; classifier; controlled study; cryopreservation; data processing; diagnostic accuracy; diagnostic test accuracy study; discriminant analysis; human; human tissue; infrared spectroscopy; *least square analysis; machine learning; major clinical study; metabolomics; morphology; *mouth squamous cell carcinoma; nonhuman; overall survival; principal component analysis; qualitative research; *Raman spectrometry; receiver operating characteristic; sensitivity and specificity; signal noise ratio; support vector machine; training; validation process; validation study; amino acid; carotenoid; hydroxyproline; nucleic acid; protein; terpenoid; *tryptophan.

Drug Index Terms

amino acid; carotenoid; hydroxyproline; nucleic acid; protein; terpenoid; *tryptophan.

Other Index Terms

*algorithm; area under the curve; Article; classifier; controlled study; cryopreservation; data processing; diagnostic accuracy; diagnostic test accuracy study; discriminant analysis; human; human tissue; infrared spectroscopy; *least square analysis; machine learning; major clinical study; metabolomics; morphology; *mouth squamous cell carcinoma; nonhuman; overall survival; principal component analysis; qualitative research; *Raman spectrometry; receiver operating characteristic; sensitivity and specificity; signal noise ratio; support vector machine; training; validation process; validation study.

Link to the Ovid Full Text or citation:

[Click here for full text options](https://ovidsp.ovid.com/ovidweb.cgi?T=JS&CSC=Y&NEWS=N&PAGE=fulltext&D=emed22&AN=2014614150)

Link to the External Link Resolver:

[SFX](https://sfx-86scu.hosted.exlibrisgroup.com.cn/86scu?sid=OVID:embase&id=pmid:&id=doi:10.3390%2Fjpm11111165&issn=20754426&isbn=&volume=11&issue=11&spage=1165&pages=&date=2021&title=Journal+of+Personalized+Medicine&atitle=Developing+an+algorithm+for+discriminating+oral+cancerous+and+normal+tissues+using+raman+spectroscopy&aulast=Sharma&pid=<author>Sharma+M.%3BJeng+M.-J.%3BYoung+C.-K.%3BHuang+S.-F.%3BChang+L.-B.<%2Fauthor><AN>2014614150<%2FAN><DT>Article<%2FDT>)

68.

A new look into cancer-a review on the contribution of vibrational spectroscopy on early diagnosis and surgery guidance.

Mamede A.P., Santos I.P., Batista de Carvalho A.L.M., Figueiredo P., Silva M.C., Tavares M.V., Marques M.P.M., Batista de Carvalho L.A.E.

Cancers. 13(21) (no pagination), 2021. Article Number: 5336. Date of Publication: November-1 2021.

AN: 2014205476

In 2020, approximately 10 million people died of cancer, rendering this disease the second leading cause of death worldwide. Detecting cancer in its early stages is paramount for patients' prognosis and survival. Hence, the scientific and medical communities are engaged in improving both therapeutic strategies and diagnostic methodologies, beyond prevention. Optical vibrational spectroscopy has been shown to be an ideal diagnostic method for early cancer diagnosis and surgical margins assessment, as a complement to histopathological analysis. Being highly sensitive, non-invasive and capable of real-time molecular imaging, Raman and Fourier transform infrared (FTIR) spectroscopies give information on the biochemical profile of the tissue under analysis, detecting the metabolic differences between healthy and cancerous portions of the same sample. This constitutes tremendous progress in the field, since the cancer-prompted morphological alterations often occur after the biochemical imbalances in the oncogenic process. Therefore, the early cancer-associated metabolic changes are unnoticed by the histopathologist. Additionally, Raman and FTIR spectroscopies significantly reduce the subjectivity linked to cancer diagnosis. This review focuses on breast and head and neck cancers, their clinical needs and the progress made to date using vibrational spectroscopy as a diagnostic technique prior to surgical intervention and intraoperative margin assessment.

Copyright © 2021 by the authors. Licensee MDPI, Basel, Switzerland.

Institution

(Mamede, Santos, Batista de Carvalho, Tavares, Marques, Batista de Carvalho) "Unidade de I&D Quimica-Fisica Molecular" (QFM-UC), Departament of Chemistry, University of Coimbra, Coimbra 3004-504, Portugal (Figueiredo) Pathology Department, Portuguese Institute of Oncology Francisco Gentil (IPOFG), Coimbra 3000-075, Portugal

(Silva) Surgery Department, Portuguese Institute of Oncology Francisco Gentil (IPOFG), Coimbra 3000-075, Portugal

(Tavares) Gynaecology Department, Portuguese Institute of Oncology Francisco Gentil (IPOFG), Porto 4200-072, Portugal

(Marques) Department of Life Sciences, University of Coimbra, Coimbra 3000-456, Portugal

Publisher

MDPI

Emtree Heading

autofluorescence imaging; breast biopsy; breast cancer/di [Diagnosis]; breast cancer/rt [Radiotherapy]; breast cancer/su [Surgery]; breast ductal carcinoma/di [Diagnosis]; breast-conserving surgery; cancer prognosis; *cancer surgery; classification algorithm; diagnostic accuracy; *early cancer diagnosis; Fourier transform infrared spectroscopy; frozen section; glioblastoma; head and neck cancer/di [Diagnosis]; head and neck cancer/su [Surgery]; histopathology; human; luminal A breast cancer/di [Diagnosis]; lumpectomy; lymph node metastasis/di [Diagnosis]; mastectomy; mouth cancer/di [Diagnosis]; near infrared reflectance spectroscopy; nonhuman; papillomavirus infection; Raman spectrometry; review; sensitivity and specificity; surface enhanced Raman spectroscopy; surgical margin; tissue microarray; tongue carcinoma/su [Surgery]; tumor localization; *vibrational spectroscopy; epidermal growth factor receptor 2/ec [Endogenous Compound]; estrogen receptor/ec [Endogenous Compound]; Ki 67 antigen/ec [Endogenous Compound]; progesterone receptor/ec [Endogenous Compound].

Drug Index Terms

epidermal growth factor receptor 2 / endogenous compound; estrogen receptor / endogenous compound; Ki 67 antigen / endogenous compound; progesterone receptor / endogenous compound.

Other Index Terms

autofluorescence imaging; breast biopsy; breast cancer / diagnosis / radiotherapy / surgery; breast ductal carcinoma / diagnosis; breast-conserving surgery; cancer prognosis; *cancer surgery; classification algorithm; diagnostic accuracy; *early cancer diagnosis; Fourier transform infrared spectroscopy; frozen section; glioblastoma; head and neck cancer / diagnosis / surgery; histopathology; human; luminal A breast cancer / diagnosis; lumpectomy; lymph node metastasis / diagnosis; mastectomy; mouth cancer / diagnosis; near infrared reflectance spectroscopy; nonhuman; papillomavirus infection; Raman spectrometry; Review; sensitivity and specificity; surface enhanced Raman spectroscopy; surgical margin; tissue microarray; tongue carcinoma / surgery; tumor localization; *vibrational spectroscopy.

Link to the Ovid Full Text or citation:

[Click here for full text options](https://ovidsp.ovid.com/ovidweb.cgi?T=JS&CSC=Y&NEWS=N&PAGE=fulltext&D=emed22&AN=2014205476)

Link to the External Link Resolver:

[SFX](https://sfx-86scu.hosted.exlibrisgroup.com.cn/86scu?sid=OVID:embase&id=pmid:&id=doi:10.3390%2Fcancers13215336&issn=20726694&isbn=&volume=13&issue=21&spage=5336&pages=&date=2021&title=Cancers&atitle=A+new+look+into+cancer-a+review+on+the+contribution+of+vibrational+spectroscopy+on+early+diagnosis+and+surgery+guidance&aulast=Mamede&pid=<author>Mamede+A.P.%3BSantos+I.P.%3BBatista+de+Carvalho+A.L.M.%3BFigueiredo+P.%3BSilva+M.C.%3BTavares+M.V.%3BMarques+M.P.M.%3BBatista+de+Carvalho+L.A.E.<%2Fauthor><AN>2014205476<%2FAN><DT>Review<%2FDT>)

69.

Raman spectroscopy discloses altered molecular profile in thyroid adenomas.

Sodo A., Verri M., Palermo A., Naciu A.M., Sponziello M., Durante C., Di Gioacchino M., Paolucci A., Di Masi A., Longo F., Crucitti P., Taffon C., Ricci M.A., Crescenzi A.

Diagnostics. 11(1) (no pagination), 2021. Article Number: 43. Date of Publication: January 2021.

AN: 2007424451

Follicular patterned nodules are sometimes complex to be classified due to ambiguous nuclear features and/or questionable capsular or vascular invasion. In this setting, there is a poor inter-observer concordance even among expert pathologists. Raman spectroscopy was recently used to separate benign and malignant thyroid nodules based on their molecular fingerprint; anyway, some histologically proved follicular adenomas were clustered as having a characteristic profile of malignant lesions. In this study, we analyzed five follicular thyroid adenomas with a malignant spectroscopic profile compared to five follicular adenomas with a benign Raman spectrum in order to assess possible molecular differences between the two groups. Morphological, immunohistochem-ical, and molecular analyses evidenced expression of malignancy-associated proteins in four out of five malignant clustered adenomas. The remaining malignant clustered adenoma showed a TSHR mutation previously associated with autonomously functioning follicular carcinomas. In conclu-sion, thyroid follicular adenomas are a group of morphologically benign neoplasms that may have altered the mutational or expression profile; cases of adenomas with altered immunophenotype are recognized as showing a profile associated with malignancy by Raman spectroscopy. This correlation warrants a more extensive evaluation and suggests a potential predictive value of spectroscopic assessment in recognizing characteristics associated with tumor progression in follicular thyroid neoplasms.

Copyright © 2020 by the authors. Licensee MDPI, Basel, Switzerland.

Institution

(Sodo, Di Gioacchino, Paolucci, Di Masi, Ricci) Department of Sciences, University Roma Tre, Rome 00146, Italy (Verri, Taffon, Crescenzi) Pathology Unit, Campus Bio-Medico University Hospital, Rome 00128, Italy

(Palermo, Naciu) Unit of Endocrinology and Diabetes, Campus Bio-Medico University, Rome 00128, Italy

(Sponziello, Durante) Department of Translational and Precision Medicine, Sapienza University of Rome, Rome 00185, Italy

(Longo, Crucitti) Unit of Thoracic Surgery, Campus Bio-Medico University Hospital, Rome 00128, Italy

Publisher

MDPI

Emtree Heading

adult; aged; article; cancer diagnosis; carcinogenesis; clinical article; cluster analysis; comparative study; correlational study; digital polymerase chain reaction; female; follicular carcinoma/et [Etiology]; frozen section; gain of function mutation; gene; gene mutation; hierarchical clustering; high throughput sequencing; human; human tissue; immunohistochemistry; immunophenotyping; lobectomy; male; middle aged; *molecular fingerprinting; morphological trait; predictive value; protein expression; *Raman spectrometry; surgical margin; *thyroid adenoma/di [Diagnosis]; *thyroid adenoma/et [Etiology]; *thyroid adenoma/su [Surgery]; thyroid follicular cell; thyroid gland tissue; thyroid parafollicular cell; thyroidectomy; eosin; hematoxylin; microRNA/ec [Endogenous Compound]; molecular marker/ec [Endogenous Compound]; unclassified drug; agglomerative hierarchical clustering analysis; EIF1AX gene; *follicular thyroid adenoma/di [Diagnosis]; *follicular thyroid adenoma/et [Etiology]; *follicular thyroid adenoma/su [Surgery]; TSHR gene; microrna 146b/ec [Endogenous Compound].

Candidate Terms

agglomerative hierarchical clustering analysis [other term] eif1ax gene [other term]

*follicular thyroid adenoma / *diagnosis / *etiology / *surgery [other term]

tshr gene [other term]

microRNA 146b / endogenous compound [drug term]

Drug Index Terms

eosin; hematoxylin; microRNA / endogenous compound; molecular marker / endogenous compound; unclassified drug.

Other Index Terms

adult; aged; Article; cancer diagnosis; carcinogenesis; clinical article; cluster analysis; comparative study; correlational study; digital polymerase chain reaction; female; follicular carcinoma / etiology; frozen section; gain of function mutation; gene; gene mutation; hierarchical clustering; high throughput sequencing; human; human tissue; immunohistochemistry; immunophenotyping; lobectomy; male; middle aged; *molecular fingerprinting; morphological trait; predictive value; protein expression; *Raman spectrometry; surgical margin; *thyroid adenoma / *diagnosis / *etiology / *surgery; thyroid follicular cell; thyroid gland tissue; thyroid parafollicular cell; thyroidectomy.

Link to the Ovid Full Text or citation:

[Click here for full text options](https://ovidsp.ovid.com/ovidweb.cgi?T=JS&CSC=Y&NEWS=N&PAGE=fulltext&D=emed22&AN=2007424451)

Link to the External Link Resolver:

[SFX](https://sfx-86scu.hosted.exlibrisgroup.com.cn/86scu?sid=OVID:embase&id=pmid:&id=doi:10.3390%2Fdiagnostics11010043&issn=20754418&isbn=&volume=11&issue=1&spage=43&pages=&date=2021&title=Diagnostics&atitle=Raman+spectroscopy+discloses+altered+molecular+profile+in+thyroid+adenomas&aulast=Sodo&pid=<author>Sodo+A.%3BVerri+M.%3BPalermo+A.%3BNaciu+A.M.%3BSponziello+M.%3BDurante+C.%3BDi+Gioacchino+M.%3BPaolucci+A.%3BDi+Masi+A.%3BLongo+F.%3BCrucitti+P.%3BTaffon+C.%3BRicci+M.A.%3BCrescenzi+A.<%2Fauthor><AN>2007424451<%2FAN><DT>Article<%2FDT>)

70.

The potential of raman spectroscopy in the diagnosis of dysplastic and malignant oral lesions.

Ibrahim O., Toner M., Flint S., Byrne H.J., Lyng F.M.

Cancers. 13(4) (pp 1-14), 2021. Article Number: 619. Date of Publication: 02 Feb 2021.

AN: 2005945979

Early diagnosis, treatment and/or surveillance of oral premalignant lesions are important in preventing progression to oral squamous cell carcinoma (OSCC). The current gold standard is through histopathological diagnosis, which is limited by inter-and intra-observer errors and sampling errors. The objective of this work was to use Raman spectroscopy to discriminate between benign, mild, moderate and severe dysplasia and OSCC in formalin fixed paraffin preserved (FFPP) tissues. The study included 72 different pathologies from which 17 were benign lesions, 20 mildly dysplastic, 20 moderately dysplastic, 10 severely dysplastic and 5 invasive OSCC. The glass substrate and paraffin wax background were digitally removed and PLSDA with LOPO cross-validation was used to differentiate the pathologies. OSCC could be differentiated from the other pathologies with an accuracy of 70%, while the accuracy of the classifier for benign, moderate and severe dysplasia was ~60%. The accuracy of the classifier was lowest for mild dysplasia (~46%). The main discriminating features were increased nucleic acid contributions and decreased protein and lipid contributions in the epithelium and decreased collagen contributions in the connective tissue. Smoking and the presence of inflammation were found to significantly influence the Raman classification with respective accuracies of 76% and 94%.

Copyright © 2021 by the authors. Licensee MDPI, Basel, Switzerland.

Institution

(Ibrahim) School of Dental Science, Trinity College Dublin, Lincoln Place, Dublin 2 D02, Ireland (Toner) Central Pathology Laboratory, St. James Hospital, James Street, Dublin 8 D08, Ireland

(Flint) Oral Medicine Unit, Dublin Dental University Hospital, Trinity College Dublin, Lincoln Place, Dublin 2 D02, Ireland

(Byrne) FOCAS Research Institute, City Campus, Technological University Dublin, Kevin Street, Dublin 8 D08, Ireland

(Lyng) Radiation and Environmental Science Centre FOCAS Research Institute, City Campus, Technological University Dublin, Kevin Street, Dublin 8 D08, Ireland

(Lyng) School of Physics & Clinical & Optometric Sciences, City Campus, Technological University Dublin, Kevin Street, Dublin 8 D08, Ireland

Publisher

MDPI AG

Emtree Heading

adult; article; clinical feature; cohort analysis; connective tissue; controlled study; cross validation; diagnostic accuracy; disease severity; epithelium; female; human; human tissue; inflammation/di [Diagnosis]; major clinical study; male; *mouth cancer/di [Diagnosis]; *mouth disease/di [Diagnosis]; mouth epithelium; patient; precancer/di [Diagnosis]; *Raman spectrometry; sensitivity and specificity; smoking; tissue section; tumor invasion; collagen/ec [Endogenous Compound]; lipid/ec [Endogenous Compound]; nucleic acid; protein/ec [Endogenous Compound]; microscope; spectrophotometer; *oral dysplasia/di [Diagnosis]; Horiba Jobin Yvon LabRam HR 800; MPLAN N.

Candidate Terms

*oral dysplasia / *diagnosis [other term] Horiba Jobin Yvon LabRam HR 800 [device term]

MPLAN N [device term]

Device Index Terms

microscope; spectrophotometer.

Drug Index Terms

collagen / endogenous compound; lipid / endogenous compound; nucleic acid; protein / endogenous compound.

Other Index Terms

adult; Article; clinical feature; cohort analysis; connective tissue; controlled study; cross validation; diagnostic accuracy; disease severity; epithelium; female; human; human tissue; inflammation / diagnosis; major clinical study; male; *mouth cancer / *diagnosis; *mouth disease / *diagnosis; mouth epithelium; patient; precancer / diagnosis; *Raman spectrometry; sensitivity and specificity; smoking; tissue section; tumor invasion.

Link to the Ovid Full Text or citation:

[Click here for full text options](https://ovidsp.ovid.com/ovidweb.cgi?T=JS&CSC=Y&NEWS=N&PAGE=fulltext&D=emed22&AN=2005945979)

Link to the External Link Resolver:

[SFX](https://sfx-86scu.hosted.exlibrisgroup.com.cn/86scu?sid=OVID:embase&id=pmid:&id=doi:10.3390%2Fcancers13040619&issn=20726694&isbn=&volume=13&issue=4&spage=1&pages=1-14&date=2021&title=Cancers&atitle=The+potential+of+raman+spectroscopy+in+the+diagnosis+of+dysplastic+and+malignant+oral+lesions&aulast=Ibrahim&pid=<author>Ibrahim+O.%3BToner+M.%3BFlint+S.%3BByrne+H.J.%3BLyng+F.M.<%2Fauthor><AN>2005945979<%2FAN><DT>Article<%2FDT>)

71.

Incorporation of fixed oils into spearmint oil-loaded nanoemulsions and their influence on characteristic and cytotoxic properties against human oral cancer cells.

Tubtimsri S., Limmatvapirat C., Limsirichaikul S., Akkaramongkolporn P., Piriyaprasarth S., Patomchaiviwat V., Limmatvapirat S.

Journal of Drug Delivery Science and Technology. 63 (no pagination), 2021. Article Number: 102443. Date of Publication: June 2021.

AN: 2011194883

Spearmint oil (SMO) has been revealed to inhibit oral cancer cells. Its activity increases when it is incorporated into nanoemulsions. However, SMO alone cannot be prepared into stable nanoemulsions without an Ostwald ripening inhibitor. This study aimed to evaluate the effect of an Ostwald ripening inhibitor, i.e., fixed oil, on the formation and characteristic of anticancer nanoemulsions. Various fixed oils, namely, virgin coconut oil (VCO; C12), palm oil (PMO; C16), olive oil (OLO; C18:1), soybean oil (SBO; C18:2), and perilla oil (PRO; C18:3), were selected, and their abilities to stabilize nanoemulsions were compared. Results indicated that the formation and characteristic of nanoemulsions were dependent on the type of fixed oils and the ratio of SMO to fixed oils. Nevertheless, the stable nanoemulsions with nearly the same droplet size could be prepared at a ratio of 80:20 regardless of the type of fixed oils. The stable nanoemulsions might be a result of specific molecular interactions among surfactants, SMO, and fixed oils. Among the nanoemulsions with fixed oils, 80:20 SMO:PRO nanoemulsion showed the most potent cytotoxic effect against oral cancer cells and therefore was selected for further study. The influence of excipients on the properties, including droplet size, carvone content, and cytotoxic activity, of the formulated nanoemulsions were comparatively evaluated after storage at 40 degreeC for 6 months. Both nanoemulsions had excellent stability. An apoptotic property assay was employed to probe the cell death mechanism. 80:20 SMO:PRO nanoemulsion could induce cancer cell death via the intrinsic apoptosis pathway, which was the desirable pathway for candidate anticancer agents. In conclusion, nanoemulsions containing SMO with cytotoxic effects against oral cancer cells were successfully prepared by selecting specific amounts and types of surfactants and fixed oils. This research might serve as a basis for fabricating stable nanoemulsions containing other volatile oils.

Copyright © 2021 Elsevier B.V.

Author NameID

Limmatvapirat, Sontaya; ORCID: <https://orcid.org/0000-0002-3829-4062>

Institution

(Tubtimsri) Faculty of Pharmaceutical Science, Burapha University, Thailand (Limmatvapirat) Department of Pharmaceutical Chemistry, Faculty of Pharmacy, Silpakorn University, Thailand

(Limsirichaikul) Department of Biopharmacy, Faculty of Pharmacy, Silpakorn University, Thailand

(Tubtimsri, Akkaramongkolporn, Piriyaprasarth, Patomchaiviwat, Limmatvapirat) Department of Pharmaceutical Technology, Faculty of Pharmacy, Silpakorn University, Thailand

Publisher

Editions de Sante

Emtree Heading

*antineoplastic activity; apoptosis; apoptosis assay; apoptotic cell percentage; article; *cancer cell; cell death; cell viability; controlled study; down regulation; *drug cytotoxicity; flow cytometry; Fourier transform infrared spectroscopy; human; human cell; mitochondrion; molecular interaction; morphological trait; *mouth cancer; *nanoemulsion; nuclear magnetic resonance spectroscopy; nuclear Overhauser effect; Raman spectrometry; real time polymerase chain reaction; upregulation; viscosity; zeta potential; actin/ec [Endogenous Compound]; carvone; *coconut oil/an [Drug Analysis]; *coconut oil/pr [Pharmaceutics]; *coconut oil/pd [Pharmacology]; cytochrome c/ec [Endogenous Compound]; excipient; fluorouracil; glyceraldehyde 3 phosphate dehydrogenase/ec [Endogenous Compound]; macrogol; *olive oil/an [Drug Analysis]; *olive oil/pr [Pharmaceutics]; *olive oil/pd [Pharmacology]; *palm oil/an [Drug Analysis]; *palm oil/pr [Pharmaceutics]; *palm oil/pd [Pharmacology]; *perilla oil/an [Drug Analysis]; *perilla oil/pr [Pharmaceutics]; *perilla oil/pd [Pharmacology]; protein Bax/ec [Endogenous Compound]; protein bcl 2/ec [Endogenous Compound]; protein p53/ec [Endogenous Compound]; RNA 18S/ec [Endogenous Compound]; *soybean oil/an [Drug Analysis]; *soybean oil/pr [Pharmaceutics]; *soybean oil/pd [Pharmacology]; surfactant; unclassified drug; flow cytometer; microscope; *virgin coconut oil/an [Drug Analysis]; *virgin coconut oil/pr [Pharmaceutics]; *virgin coconut oil/pd [Pharmacology].

Candidate Terms

*virgin coconut oil / *drug analysis / *pharmaceutics / *pharmacology [drug term]

Device Index Terms

flow cytometer; microscope.

Drug Index Terms

actin / endogenous compound; carvone; *coconut oil / *drug analysis / *pharmaceutics / *pharmacology; cytochrome c / endogenous compound; excipient; fluorouracil; glyceraldehyde 3 phosphate dehydrogenase / endogenous compound; macrogol; *olive oil / *drug analysis / *pharmaceutics / *pharmacology; *palm oil / *drug analysis / *pharmaceutics / *pharmacology; *perilla oil / *drug analysis / *pharmaceutics / *pharmacology; protein Bax / endogenous compound; protein bcl 2 / endogenous compound; protein p53 / endogenous compound; RNA 18S / endogenous compound; *soybean oil / *drug analysis / *pharmaceutics / *pharmacology; surfactant; unclassified drug.

Other Index Terms

*antineoplastic activity; apoptosis; apoptosis assay; apoptotic cell percentage; Article; *cancer cell; cell death; cell viability; controlled study; down regulation; *drug cytotoxicity; flow cytometry; Fourier transform infrared spectroscopy; human; human cell; mitochondrion; molecular interaction; morphological trait; *mouth cancer; *nanoemulsion; nuclear magnetic resonance spectroscopy; nuclear Overhauser effect; Raman spectrometry; real time polymerase chain reaction; upregulation; viscosity; zeta potential.

Link to the Ovid Full Text or citation:

[Click here for full text options](https://ovidsp.ovid.com/ovidweb.cgi?T=JS&CSC=Y&NEWS=N&PAGE=fulltext&D=emed22&AN=2011194883)

Link to the External Link Resolver:

[SFX](https://sfx-86scu.hosted.exlibrisgroup.com.cn/86scu?sid=OVID:embase&id=pmid:&id=doi:10.1016%2Fj.jddst.2021.102443&issn=17732247&isbn=&volume=63&issue=&spage=102443&pages=&date=2021&title=Journal+of+Drug+Delivery+Science+and+Technology&atitle=Incorporation+of+fixed+oils+into+spearmint+oil-loaded+nanoemulsions+and+their+influence+on+characteristic+and+cytotoxic+properties+against+human+oral+cancer+cells&aulast=Tubtimsri&pid=<author>Tubtimsri+S.%3BLimmatvapirat+C.%3BLimsirichaikul+S.%3BAkkaramongkolporn+P.%3BPiriyaprasarth+S.%3BPatomchaiviwat+V.%3BLimmatvapirat+S.<%2Fauthor><AN>2011194883<%2FAN><DT>Article<%2FDT>)

72.

Current intraoperative imaging techniques to improve surgical resection of laryngeal cancer: A systematic review.

Lauwerends L.J., Galema H.A., Hardillo J.A.U., Sewnaik A., Monserez D., van Driel P.B.A.A., Verhoef C., Baatenburg de Jong R.J., Hilling D.E., Keereweer S.

Cancers. 13(8) (no pagination), 2021. Article Number: 1895. Date of Publication: 02 Apr 2021.

AN: 2006947117

Laryngeal cancer is a prevalent head and neck malignancy, with poor prognosis and low survival rates for patients with advanced disease. Treatment consists of unimodal therapy through surgery or radiotherapy in early staged tumors, while advanced stage tumors are generally treated with multimodal chemoradiotherapy or (total) laryngectomy followed by radiotherapy. Still, the recurrence rate for advanced laryngeal cancer is between 25 and 50%. In order to improve surgical resection of laryngeal cancer and reduce local recurrence rates, various intraoperative optical imaging techniques have been investigated. In this systematic review, we identify these technologies, evaluating the current state and future directions of optical imaging for this indication. Narrow-band imaging (NBI) and autofluorescence (AF) are established tools for early detection of laryngeal cancer. Nonetheless, their intraoperative utility is limited by an intrinsic inability to image beyond the (sub-)mucosa. Likewise, contact endoscopy (CE) and optical coherence tomography (OCT) are technically cumbersome and only useful for mucosal margin assessment. Research on fluorescence imaging (FLI) for this application is sparse, dealing solely with nonspecific fluorescent agents. Evidently, the imaging modalities that have been investigated thus far are generally unsuitable for deep margin assessment. We discuss two optical imaging techniques that can overcome these limitations and suggest how they can be used to achieve adequate margins in laryngeal cancer at all stages.

Copyright © 2021 by the authors. Licensee MDPI, Basel, Switzerland.

Institution

(Lauwerends, Galema, Hardillo, Sewnaik, Monserez, Baatenburg de Jong, Keereweer) Department of Otorhinolaryngology, Head and Neck Surgery, Erasmus MC Cancer Institute, Rotterdam 3015 GD, Netherlands (Galema, Verhoef, Hilling) Department of Surgical Oncology and Gastrointestinal Surgery, Erasmus MC Cancer Institute, Rotterdam 3015 GD, Netherlands

(van Driel) Department of Orthopedic Surgery, Isala Hospital, Zwolle 8025 AB, Netherlands

Publisher

MDPI AG

Emtree Heading

autofluorescence; cancer staging; *cancer surgery; early cancer diagnosis; *fluorescence imaging; human; *image analysis; *intraoperative period; intrinsic mode function; *larynx cancer/di [Diagnosis]; *larynx cancer/su [Surgery]; mucosa; narrow band imaging; optical coherence tomography; Raman spectrometry; review; surgical margin; systematic review; treatment indication; aminolevulinic acid; protoporphyrin; contact endoscopy.

Candidate Terms

contact endoscopy [other term]

Drug Index Terms

aminolevulinic acid; protoporphyrin.

Other Index Terms

autofluorescence; cancer staging; *cancer surgery; early cancer diagnosis; *fluorescence imaging; human; *image analysis; *intraoperative period; intrinsic mode function; *larynx cancer / *diagnosis / *surgery; mucosa; narrow band imaging; optical coherence tomography; Raman spectrometry; Review; surgical margin; systematic review; treatment indication.

Link to the Ovid Full Text or citation:

[Click here for full text options](https://ovidsp.ovid.com/ovidweb.cgi?T=JS&CSC=Y&NEWS=N&PAGE=fulltext&D=emed22&AN=2006947117)

Link to the External Link Resolver:

[SFX](https://sfx-86scu.hosted.exlibrisgroup.com.cn/86scu?sid=OVID:embase&id=pmid:&id=doi:10.3390%2Fcancers13081895&issn=20726694&isbn=&volume=13&issue=8&spage=1895&pages=&date=2021&title=Cancers&atitle=Current+intraoperative+imaging+techniques+to+improve+surgical+resection+of+laryngeal+cancer%3A+A+systematic+review&aulast=Lauwerends&pid=<author>Lauwerends+L.J.%3BGalema+H.A.%3BHardillo+J.A.U.%3BSewnaik+A.%3BMonserez+D.%3Bvan+Driel+P.B.A.A.%3BVerhoef+C.%3BBaatenburg+de+Jong+R.J.%3BHilling+D.E.%3BKeereweer+S.<%2Fauthor><AN>2006947117<%2FAN><DT>Review<%2FDT>)

73.

Identifying benign and malignant thyroid nodules based on blood serum surface-enhanced Raman spectroscopy.

Xia L., Lu J., Chen Z., Cui X., Chen S., Pei D.

Nanomedicine: Nanotechnology, Biology, and Medicine. 32 (no pagination), 2021. Article Number: 102328. Date of Publication: February 2021.

AN: 2010135205

The aim of this study is to evaluate the feasibility of using blood serum surface-enhanced Raman spectroscopy (SERS) to identify benign and malignant thyroid nodules. Blood serum samples collected from three different groups including healthy volunteers (n = 22), patients with benign nodules (n = 19) and malignant nodules (n = 22) were measured by SERS. The spectral analysis results demonstrate that biomolecules in serum, such as amino acids, adenine and nucleic acid bases, change differently due to the different progression of nodules. By further combining with partial least square analysis and linear discriminant analysis (PLS-LDA) method, diagnostic accuracies of 93.65% and 82.93%, sensitivities of 92.68% and 81.82% and specificities of 95.45% and 84.21% can be achieved for differentiating healthy versus thyroid nodular groups and benign versus malignant groups, respectively. The above results have suggested that the blood serum SERS technique is helpful for precise diagnosis and timely treatment for patients with thyroid nodules.

Copyright © 2020 Elsevier Inc.

PMID

33181274 [<https://www.ncbi.nlm.nih.gov/pubmed/?term=33181274>]

Institution

(Xia, Chen, Cui, Chen) College of Medicine and Biological Information Engineering, Northeastern University, Shenyang, China (Lu, Pei) Department of Health Management & Department of Family Medicine, Shengjing Hospital of China Medical University, Shenyang, China

(Cui, Chen) Key Laboratory of Intelligent Computing in Medical Image, Ministry of Education, China

Publisher

Elsevier Inc.

Emtree Heading

adult; amino acid blood level; article; cancer diagnosis; clinical article; controlled study; diagnostic accuracy; discriminant analysis; female; human; human tissue; least square analysis; male; sensitivity and specificity; *surface enhanced Raman spectroscopy; *thyroid cancer/di [Diagnosis]; *thyroid nodule/di [Diagnosis]; adenine/ec [Endogenous Compound]; nucleic acid base/ec [Endogenous Compound]; microscope; microscope slide; HR Evolution.

Candidate Terms

HR Evolution [device term]

Device Index Terms

microscope; microscope slide.

Drug Index Terms

adenine / endogenous compound; nucleic acid base / endogenous compound.

Other Index Terms

adult; amino acid blood level; Article; cancer diagnosis; clinical article; controlled study; diagnostic accuracy; discriminant analysis; female; human; human tissue; least square analysis; male; sensitivity and specificity; *surface enhanced Raman spectroscopy; *thyroid cancer / *diagnosis; *thyroid nodule / *diagnosis.

Link to the Ovid Full Text or citation:

[Click here for full text options](https://ovidsp.ovid.com/ovidweb.cgi?T=JS&CSC=Y&NEWS=N&PAGE=fulltext&D=emed22&AN=2010135205)

Link to the External Link Resolver:

[SFX](https://sfx-86scu.hosted.exlibrisgroup.com.cn/86scu?sid=OVID:embase&id=pmid:33181274&id=doi:10.1016%2Fj.nano.2020.102328&issn=15499634&isbn=&volume=32&issue=&spage=102328&pages=&date=2021&title=Nanomedicine%3A+Nanotechnology%2C+Biology%2C+and+Medicine&atitle=Identifying+benign+and+malignant+thyroid+nodules+based+on+blood+serum+surface-enhanced+Raman+spectroscopy&aulast=Xia&pid=<author>Xia+L.%3BLu+J.%3BChen+Z.%3BCui+X.%3BChen+S.%3BPei+D.<%2Fauthor><AN>2010135205<%2FAN><DT>Article<%2FDT>)

74.

Axon-Myelin Unit Blistering as Early Event in MS Normal Appearing White Matter.

Luchicchi A., Hart B., Frigerio I., van Dam A.-M., Perna L., Offerhaus H.L., Stys P.K., Schenk G.J., Geurts J.J.G.

Annals of Neurology. 89(4) (pp 711-725), 2021. Date of Publication: April 2021.

AN: 2010277030

Objective: Multiple sclerosis (MS) is a chronic neuroinflammatory and neurodegenerative disease of unknown etiology. Although the prevalent view regards a CD4+-lymphocyte autoimmune reaction against myelin at the root of the disease, recent studies propose autoimmunity as a secondary reaction to idiopathic brain damage. To gain knowledge about this possibility we investigated the presence of axonal and myelinic morphological alterations, which could implicate imbalance of axon-myelin units as primary event in MS pathogenesis.

Method(s): Using high resolution imaging histological brain specimens from patients with MS and non-neurological/non-MS controls, we explored molecular changes underpinning imbalanced interaction between axon and myelin in normal appearing white matter (NAWM), a region characterized by normal myelination and absent inflammatory activity.

Result(s): In MS brains, we detected blister-like swellings formed by myelin detachment from axons, which were substantially less frequently retrieved in non-neurological/non-MS controls. Swellings in MS NAWM presented altered glutamate receptor expression, myelin associated glycoprotein (MAG) distribution, and lipid biochemical composition of myelin sheaths. Changes in tethering protein expression, widening of nodes of Ranvier and altered distribution of sodium channels in nodal regions of otherwise normally myelinated axons were also present in MS NAWM. Finally, we demonstrate a significant increase, compared with controls, in citrullinated proteins in myelin of MS cases, pointing toward biochemical modifications that may amplify the immunogenicity of MS myelin.

Interpretation(s): Collectively, the impaired interaction of myelin and axons potentially leads to myelin disintegration. Conceptually, the ensuing release of (post-translationally modified) myelin antigens may elicit a subsequent immune attack in MS. ANN NEUROL 2021;89:711-725.

Copyright © 2021 The Authors. Annals of Neurology published by Wiley Periodicals LLC on behalf of American Neurological Association.

PMID

33410190 [<https://www.ncbi.nlm.nih.gov/pubmed/?term=33410190>]

Author NameID

Luchicchi, Antonio; ORCID: <https://orcid.org/0000-0002-0189-4347> Hart, Bert't; ORCID: <https://orcid.org/0000-0002-0036-5267>

van Dam, Anne-Marie; ORCID: <https://orcid.org/0000-0003-4191-3941>

Offerhaus, Herman L.; ORCID: <https://orcid.org/0000-0003-3697-5750>

Stys, Peter K.; ORCID: <https://orcid.org/0000-0003-0326-4194>

Institution

(Luchicchi, Hart, Frigerio, van Dam, Perna, Schenk, Geurts) Amsterdam UMC, Vrije Universiteit, Department of Anatomy and Neurosciences, Amsterdam Neuroscience, MS Center Amsterdam, Amsterdam, Netherlands (Hart) Department Biomedical Sciences of Cells and Systems, University Medical Center Groningen, Groningen, Netherlands

(Offerhaus) Faculty of Science and Technology, University of Twente, Enschede, Netherlands

(Stys) Cummings School of Medicine, University of Calgary, Calgary, AB, Canada

Publisher

John Wiley and Sons Inc

Emtree Heading

adult; aged; amino acid sequence; antigen retrieval; article; aspiration pneumonia; *axon; biochemical composition; cachexia; chronic obstructive lung disease; clinical article; coherent anti Stokes Raman spectroscopy; controlled study; corpus callosum; female; fluorescence microscopy; heart failure; human; immunohistochemistry; lung carcinoma; male; middle aged; molecular fingerprinting; *multiple sclerosis/et [Etiology]; multispectral imaging; myelin sheath; myelination; nerve fiber degeneration; ovary carcinoma; pancreas carcinoma; pathogenesis; pneumonia; priority journal; protein expression; respiratory failure; sepsis; tongue carcinoma; *white matter; young adult; glutamate receptor/ec [Endogenous Compound]; *myelin/ec [Endogenous Compound]; myelin associated glycoprotein/ec [Endogenous Compound]; n methyl dextro aspartic acid receptor/ec [Endogenous Compound]; sodium azide; sodium channel/ec [Endogenous Compound]; imaging system; microscope.

Device Index Terms

imaging system; microscope.

Drug Index Terms

glutamate receptor / endogenous compound; *myelin / *endogenous compound; myelin associated glycoprotein / endogenous compound; n methyl dextro aspartic acid receptor / endogenous compound; sodium azide; sodium channel / endogenous compound.

Other Index Terms

adult; aged; amino acid sequence; antigen retrieval; Article; aspiration pneumonia; *axon; biochemical composition; cachexia; chronic obstructive lung disease; clinical article; coherent anti Stokes Raman spectroscopy; controlled study; corpus callosum; female; fluorescence microscopy; heart failure; human; immunohistochemistry; lung carcinoma; male; middle aged; molecular fingerprinting; *multiple sclerosis / *etiology; multispectral imaging; myelin sheath; myelination; nerve fiber degeneration; ovary carcinoma; pancreas carcinoma; pathogenesis; pneumonia; priority journal; protein expression; respiratory failure; sepsis; tongue carcinoma; *white matter; young adult.

Link to the Ovid Full Text or citation:

[Click here for full text options](https://ovidsp.ovid.com/ovidweb.cgi?T=JS&CSC=Y&NEWS=N&PAGE=fulltext&D=emed22&AN=2010277030)

Link to the External Link Resolver:

[SFX](https://sfx-86scu.hosted.exlibrisgroup.com.cn/86scu?sid=OVID:embase&id=pmid:33410190&id=doi:10.1002%2Fana.26014&issn=03645134&isbn=&volume=89&issue=4&spage=711&pages=711-725&date=2021&title=Annals+of+Neurology&atitle=Axon-Myelin+Unit+Blistering+as+Early+Event+in+MS+Normal+Appearing+White+Matter&aulast=Luchicchi&pid=<author>Luchicchi+A.%3BHart+B.%3BFrigerio+I.%3Bvan+Dam+A.-M.%3BPerna+L.%3BOfferhaus+H.L.%3BStys+P.K.%3BSchenk+G.J.%3BGeurts+J.J.G.<%2Fauthor><AN>2010277030<%2FAN><DT>Article<%2FDT>)

75.

Optical biopsy of laryngeal lesions using femtosecond multiphoton microscopy.

Zhang H., Chen Y., Cao D., Li W., Jing Y., Zhong H., Liu H., Zhu X.

Biomedical Optics Express. 12(3) (pp 1308-1319), 2021. Date of Publication: 01 Mar 2021.

AN: 2011122079

Laryngeal squamous cell carcinoma (LSCC) is one of the most prevalent malignancy of the upper aerodigestive tract. Detection of early lesions in vivo could improve the survival rate significantly. In this study, we demonstrated that femtosecond multiphoton microscopy (MPM) is an effective tool to visualize the microscopic features within fixed laryngeal tissues, without sectioning, staining, or labeling. Accurate detection of lesions and determination of the tumor grading can be achieved, with excellent consistency with conventional histological examination. These results suggest that MPM may represent a powerful tool for in-vivo or fast ex-vivo diagnosis of laryngeal lesions at the point of care.

Copyright © 2021 Optical Society of America under the terms of the OSA Open Access Publishing Agreement.

Institution

(Zhang, Cao, Li, Jing, Liu) Department of Pathology, Beijing Tongren Hospital, Capital Medical University, Beijing Key Laboratory of Head and Neck Molecular Diagnostic Pathology, Beijing 100730, China (Chen, Zhong, Zhu) Femtosecond Research Center (Guangzhou), A616 80 Lanyue Road, Guangzhou 510663, China

Publisher

The Optical Society

Emtree Heading

angiography; article; confocal microscopy; controlled study; cornea epithelium; ex vivo study; fluorescence imaging; histology; human; human tissue; image analysis; image processing; image segmentation; immunohistochemistry; in vivo study; intestine metaplasia; keratinization; lamina propria; larynx cancer; *larynx injury; *multiphoton microscopy; neoplasm; Raman spectrometry; signal noise ratio; squamous cell carcinoma; survival rate; transmission electron microscopy.

Other Index Terms

angiography; Article; confocal microscopy; controlled study; cornea epithelium; ex vivo study; fluorescence imaging; histology; human; human tissue; image analysis; image processing; image segmentation; immunohistochemistry; in vivo study; intestine metaplasia; keratinization; lamina propria; larynx cancer; *larynx injury; *multiphoton microscopy; neoplasm; Raman spectrometry; signal noise ratio; squamous cell carcinoma; survival rate; transmission electron microscopy.

Link to the Ovid Full Text or citation:

[Click here for full text options](https://ovidsp.ovid.com/ovidweb.cgi?T=JS&CSC=Y&NEWS=N&PAGE=fulltext&D=emed22&AN=2011122079)

Link to the External Link Resolver:

[SFX](https://sfx-86scu.hosted.exlibrisgroup.com.cn/86scu?sid=OVID:embase&id=pmid:&id=doi:10.1364%2FBOE.414931&issn=21567085&isbn=&volume=12&issue=3&spage=1308&pages=1308-1319&date=2021&title=Biomedical+Optics+Express&atitle=Optical+biopsy+of+laryngeal+lesions+using+femtosecond+multiphoton+microscopy&aulast=Zhang&pid=<author>Zhang+H.%3BChen+Y.%3BCao+D.%3BLi+W.%3BJing+Y.%3BZhong+H.%3BLiu+H.%3BZhu+X.<%2Fauthor><AN>2011122079<%2FAN><DT>Article<%2FDT>)

76.

Optical diagnosis of oral cavity lesions by label-free Raman spectroscopy.

Matthies L., Gebrekidan M.T., Tegtmeyer J.F., Oetter N., Rohde M., Vollkommer T., Smeets R., Wilczak W., Stelzle F., Gosau M., Braeuer A.S., Knipfer C.

Biomedical Optics Express. 12(2) (pp 836-851), 2021. Date of Publication: 2021.

AN: 2010961879

Oral squamous cell carcinoma (OSCC) is one of the most prevalent cancers and frequently preceded by non-malignant lesions. Using Shifted-Excitation Raman Difference Spectroscopy (SERDS), principal component and linear discriminant analysis in native tissue specimens, 9500 raw Raman spectra of OSCC, 4300 of non-malignant lesions and 4200 of physiological mucosa were evaluated. Non-malignant lesions were distinguished from physiological mucosa with a classification accuracy of 95.3% (95.4% sensitivity, 95.2% specificity, area under the curve (AUC) 0.99). Discriminating OSCC from non-malignant lesions showed an accuracy of 88.4% (93.7% sensitivity, 76.7% specificity, AUC 0.93). OSCC was identified against physiological mucosa with an accuracy of 89.8% (93.7% sensitivity, 81.0% specificity, AUC 0.90). These findings underline the potential of SERDS for the diagnosis of oral cavity lesions.

Copyright © 2021 Optical Society of America.

Institution

(Matthies, Tegtmeyer, Vollkommer, Smeets, Gosau, Knipfer) University Medical Center Hamburg-Eppendorf (UKE), Department of Oral and Maxillofacial Surgery, Martinistrase 52, Hamburg D-20246, Germany (Gebrekidan, Oetter, Stelzle) Friedrich-Alexander-Universitat Erlangen-Nurnberg (FAU), Erlangen Graduate School in Advanced Optical Technologies (SAOT), Paul-Gordan-Strase 6, Erlangen D-91054, Germany

(Gebrekidan, Braeuer) Technische Universitat Bergakademie Freiberg (TUBAF), Institute of Thermal-Environmental- and Resources' Process Engineering (ITUN), Leipziger Strase 28, Freiberg D-09599, Germany

(Oetter, Rohde, Stelzle) Friedrich-Alexander-Universitat Erlangen-Nurnberg (FAU), Department of Oral and Maxillofacial Surgery, Gluckstrase 11, Erlangen D-91054, Germany

(Wilczak) University Medical Center Hamburg-Eppendorf (UKE), Institute of Pathology, Martinistrase 52, Hamburg D-20246, Germany

Publisher

The Optical Society

Emtree Heading

alveolar ridge; article; autofluorescence; biometry; clinical article; controlled study; diagnostic accuracy; diagnostic test accuracy study; follow up; Fourier transform; histopathology; human; human tissue; hyperkeratosis; infrared spectroscopy; leukoplakia; local field potential; molecular imaging; mouth cavity; *mouth squamous cell carcinoma/di [Diagnosis]; mucosa; optical coherence tomography; *Raman spectrometry; receiver operating characteristic; sensitivity and specificity; signal noise ratio; spectral sensitivity; spectroscopy; squamous cell carcinoma; uterus myoma; light emitting diode; sensor; *shifted excitation Raman difference spectroscopy.

Candidate Terms

*shifted excitation Raman difference spectroscopy [other term]

Device Index Terms

light emitting diode; sensor.

Other Index Terms

alveolar ridge; Article; autofluorescence; biometry; clinical article; controlled study; diagnostic accuracy; diagnostic test accuracy study; follow up; Fourier transform; histopathology; human; human tissue; hyperkeratosis; infrared spectroscopy; leukoplakia; local field potential; molecular imaging; mouth cavity; *mouth squamous cell carcinoma / *diagnosis; mucosa; optical coherence tomography; *Raman spectrometry; receiver operating characteristic; sensitivity and specificity; signal noise ratio; spectral sensitivity; spectroscopy; squamous cell carcinoma; uterus myoma.

Link to the Ovid Full Text or citation:

[Click here for full text options](https://ovidsp.ovid.com/ovidweb.cgi?T=JS&CSC=Y&NEWS=N&PAGE=fulltext&D=emed22&AN=2010961879)

Link to the External Link Resolver:

[SFX](https://sfx-86scu.hosted.exlibrisgroup.com.cn/86scu?sid=OVID:embase&id=pmid:&id=doi:10.1364%2FBOE.409456&issn=21567085&isbn=&volume=12&issue=2&spage=836&pages=836-851&date=2021&title=Biomedical+Optics+Express&atitle=Optical+diagnosis+of+oral+cavity+lesions+by+label-free+Raman+spectroscopy&aulast=Matthies&pid=<author>Matthies+L.%3BGebrekidan+M.T.%3BTegtmeyer+J.F.%3BOetter+N.%3BRohde+M.%3BVollkommer+T.%3BSmeets+R.%3BWilczak+W.%3BStelzle+F.%3BGosau+M.%3BBraeuer+A.S.%3BKnipfer+C.<%2Fauthor><AN>2010961879<%2FAN><DT>Article<%2FDT>)

77.

Chitosan modified nitrogen-doped porous carbon composite as a highly-efficient adsorbent for phenolic pollutants removal.

Liu Y., Li L., Duan Z., You Q., Liao G., Wang D.

Colloids and Surfaces A: Physicochemical and Engineering Aspects. 610 (no pagination), 2021. Article Number: 125728. Date of Publication: 05 Feb 2021.

AN: 2008378282

In this work, a chitosan modified nitrogen-doped porous carbon composite (CS-NPC) with abundant micropore and mesopore was prepared by the hydrothermal carbonization combined with chemical activation, and used as an adsorbent for phenolic pollutants removal. The CS-NPC composite were characterized by the field emission scanning electron microscopy (FE-SEM), N2 adsorption-desorption curves, X-ray photoelectron spectrum (XPS), X-ray diffraction (XRD) and Raman. It was found that the resulting carbon material owned large specific surface areas (2189.88 m2 g-1) and total pore volume (1.123 cm3 g-1), and its micropore and mesopore volume was 0.494 and 0.629 cm3 g-1, respectively. Batch adsorption experiments were carried out to study the impact of various factors like solution pH value, contact time and ionic intensity. The adsorption process can be described by the pseudo-second-order kinetic model and Langmuir adsorption isotherm model. And at 298 K, the adsorption capability of the CS-NPC composite for phenol, BPA and 2,4-DCP was 254.45, 675.68 and 892.86 mg g-1, respectively, which was much higher than that of previously reported carbon-based materials due to its own high specific surface areas, abundant functional groups ([sbnd] NH2, -OH) and porous structure. The adsorbent still remained excellent regeneration after five cycles. And the CS-NPC composite can be regarded as a broad-spectrum adsorbent toward other organic pollutants including methyl orange, methylene blue and tetracycline. This study developed a novel, low-cost and green porous carbon composite which was combined with the advantage of both phenolic resin and CS, and the CS-NPC would be an outstanding adsorbent for the removal of various organic contaminants from wastewater.

Copyright © 2020 Elsevier B.V.

Institution

(Liu, Duan, Liao) Engineering Research Center of Nano-Geomaterials of Ministry of Education, Faculty of Material Science and Chemistry, China University of Geosciences, Wuhan, Hubei 430074, China (Li, Wang) School of Environmental Studies, China University of Geosciences, Wuhan, Hubei 430074, China

(You) Key Laboratory of Optoelectronic Chemical Materials and Devices, Ministry of Education, School of Chemical and Environmental Engineering, Jianghan University, Wuhan, Hubei 430056, China

(Wang) State Key Laboratory of Environmental Aquatic Chemistry, Research Center for Co-Environmental Sciences, Chinese Academy of Sciences, Beijing 100085, China

Publisher

Elsevier B.V.

Emtree Heading

*adsorption; article; carbonization; chemical structure; contact time; controlled study; desorption; field emission scanning electron microscopy; ionic strength; isotherm; *organic pollution; *persistent organic pollutant; pH; physical chemistry; pore volume; porosity; priority journal; Raman spectrometry; surface area; *waste component removal; *waste water management; X ray photoemission spectroscopy; X ray powder diffraction; 2,4 dichlorophenol; 4,4' isopropylidenediphenol; *adsorbent; ammonia; *carbon; *chitosan; functional group; hydroxyl group; methyl orange; methylene blue; *nanocomposite; *nitrogen; phenol; *phenol derivative; resin; tetracycline; chemical analyzer; membrane filter; scanning electron microscope; X ray film; Langmuir adsorption isotherm model; F8 Focus Powder XRD; Sirion 200.

Candidate Terms

Langmuir adsorption isotherm model [other term] F8 Focus Powder XRD [device term]

Sirion 200 [device term]

Device Index Terms

chemical analyzer; membrane filter; scanning electron microscope; X ray film.

Drug Index Terms

2,4 dichlorophenol; 4,4' isopropylidenediphenol; *adsorbent; ammonia; *carbon; *chitosan; functional group; hydroxyl group; methyl orange; methylene blue; *nanocomposite; *nitrogen; phenol; *phenol derivative; resin; tetracycline.

Other Index Terms

*adsorption; Article; carbonization; chemical structure; contact time; controlled study; desorption; field emission scanning electron microscopy; ionic strength; isotherm; *organic pollution; *persistent organic pollutant; pH; physical chemistry; pore volume; porosity; priority journal; Raman spectrometry; surface area; *waste component removal; *waste water management; X ray photoemission spectroscopy; X ray powder diffraction.

Link to the Ovid Full Text or citation:

[Click here for full text options](https://ovidsp.ovid.com/ovidweb.cgi?T=JS&CSC=Y&NEWS=N&PAGE=fulltext&D=emed22&AN=2008378282)

Link to the External Link Resolver:

[SFX](https://sfx-86scu.hosted.exlibrisgroup.com.cn/86scu?sid=OVID:embase&id=pmid:&id=doi:10.1016%2Fj.colsurfa.2020.125728&issn=09277757&isbn=&volume=610&issue=&spage=125728&pages=&date=2021&title=Colloids+and+Surfaces+A%3A+Physicochemical+and+Engineering+Aspects&atitle=Chitosan+modified+nitrogen-doped+porous+carbon+composite+as+a+highly-efficient+adsorbent+for+phenolic+pollutants+removal&aulast=Liu&pid=<author>Liu+Y.%3BLi+L.%3BDuan+Z.%3BYou+Q.%3BLiao+G.%3BWang+D.<%2Fauthor><AN>2008378282<%2FAN><DT>Article<%2FDT>)

78.

Specimen-driven intraoperative assessment of resection margins should be standard of care for oral cancer patients.

Aaboubout Y., ten Hove I., Smits R.W.H., Hardillo J.A., Puppels G.J., Koljenovic S.

Oral Diseases. 27(1) (pp 111-116), 2021. Date of Publication: January 2021.

AN: 2006140277

With an incidence of 350.000 new cases per year, cancer of the oral cavity ranks among the 10 most common solid organ cancers. Most of these cancers are squamous cell carcinomas. Five-year survival is about 50%. It has been shown that clear resection margins (>5 mm healthy tissue surrounding the resected tumor) have a significant positive effect on locoregional control and survival. It is not uncommon that the resection margins of oral tumors are inadequate. However, when providing the surgeon with intraoperative feedback on the resection margin status, it is expected that obtaining adequate resection margins is improved. In this respect, it has been shown that specimen-driven intraoperative assessment of resection margins is superior to defect-driven intraoperative assessment of resection margins. In this concise report, it is described how a specimen-driven approach can increase the rate of adequate resections of oral cavity squamous cell carcinoma as well as that it is discussed how intraoperative assessment can be further improved with regard to the surgical treatment of oral cavity squamous cell carcinoma.

Copyright © 2020 The Authors. Oral Diseases published by John Wiley & Sons Ltd

PMID

32816373 [<https://www.ncbi.nlm.nih.gov/pubmed/?term=32816373>]

Author NameID

Koljenovic, Senada; ORCID: <https://orcid.org/0000-0002-5185-3455>

Institution

(Aaboubout, Koljenovic) Depatment of Pathology, Erasmus MC, University Medical Center Rotterdam, Rotterdam, Netherlands (Aaboubout, Smits, Hardillo) Department of Otorhinolaryngology and Head and Neck Surgery, Erasmus MC, University Medical Center Rotterdam, Rotterdam, Netherlands

(ten Hove) Department of Oral and Maxillofacial Surgery, Erasmus MC, University Medical Center Rotterdam, Rotterdam, Netherlands

(ten Hove) Department of Oral and Maxillofacial Surgery, LUMC, Leiden University Medical Center, Rotterdam, Netherlands

(Puppels) Department of Dermatology, Erasmus MC, University Medical Center Rotterdam, Rotterdam, Netherlands

Publisher

Blackwell Publishing Ltd

Emtree Heading

cancer patient; cancer surgery; cancer tissue; frozen section; *health care quality; human; *intraoperative period; *mouth squamous cell carcinoma/su [Surgery]; *oral surgery; priority journal; Raman spectrometry; review; *surgical margin.

Other Index Terms

cancer patient; cancer surgery; cancer tissue; frozen section; *health care quality; human; *intraoperative period; *mouth squamous cell carcinoma / *surgery; *oral surgery; priority journal; Raman spectrometry; Review; *surgical margin.

Link to the Ovid Full Text or citation:

[Click here for full text options](https://ovidsp.ovid.com/ovidweb.cgi?T=JS&CSC=Y&NEWS=N&PAGE=fulltext&D=emed22&AN=2006140277)

Link to the External Link Resolver:

[SFX](https://sfx-86scu.hosted.exlibrisgroup.com.cn/86scu?sid=OVID:embase&id=pmid:32816373&id=doi:10.1111%2Fodi.13619&issn=1354523X&isbn=&volume=27&issue=1&spage=111&pages=111-116&date=2021&title=Oral+Diseases&atitle=Specimen-driven+intraoperative+assessment+of+resection+margins+should+be+standard+of+care+for+oral+cancer+patients&aulast=Aaboubout&pid=<author>Aaboubout+Y.%3Bten+Hove+I.%3BSmits+R.W.H.%3BHardillo+J.A.%3BPuppels+G.J.%3BKoljenovic+S.<%2Fauthor><AN>2006140277<%2FAN><DT>Review<%2FDT>)

79.

Salivary Raman Spectroscopy: Standardization of Sampling Protocols and Stratification of Healthy and Oral Cancer Subjects.

Hole A., Tyagi G., Deshmukh A., Deshpande R., Gota V., Chaturvedi P., Krishna C.M.

Applied spectroscopy. 75(5) (pp 581-588), 2021. Date of Publication: 01 May 2021.

AN: 633278953

Minimally invasive cancer detection using bio-fluids has been actively pursued due to practical limitations, though there are better suited noninvasive and online in vivo methods. Saliva is one such clinically informative bio-fluid that offers the advantages of easy and multiple sample collection. Despite its potential in cancer diagnostics, saliva analysis is challenging due to its heterogeneous composition. Recently, there has been an upsurge in saliva exploration using optical techniques. Forms of saliva such as precipitate and supernatant have been monitored, but this sampling method needs to be standardized due to the obvious loss of analytes in processing. In that context, present work details the comparison of four different saliva sampling methodologies, i.e., air-dried, lyophilized, pellet, and supernatant using Raman spectroscopy collected from 10 healthy samples. Composition-driven spectral features of all forms were compared and classified using principal component analysis and linear discriminant analysis. Analysis was carried out on all four groups in the first step. In the second step, groups of pellet and supernatant , and air-dried and lyophilized were analyzed. Findings suggest that pellet and supernatant exhibit discrete spectroscopic features and demonstrate high classification efficiency, which is indicative of their distinctive biochemical composition. On the other hand, air-dried and lyophilized forms showed overlapping spectral features and low classification, suggesting these forms retain majority spectroscopic features of whole saliva and are less prone to sampling losses. Thus, this study indicates air-dried and lyophilized forms may be more appropriate for saliva sampling using Raman spectroscopy providing the comprehensive information required for cancer diagnosis. Furthermore, the method was also tested for the classification of oral cancer and healthy subjects (n=27) which yielded 90% stratification. The findings of the study indicate the utility of minimally invasive salivary Raman-based diagnostics in oral cancers.

PMID

33107759 [<https://www.ncbi.nlm.nih.gov/pubmed/?term=33107759>]

Author NameID

Krishna, C Murali; ORCID: <https://orcid.org/0000-0002-4974-8533>

Institution

(Hole, Tyagi, Krishna) Chilakapati Laboratory, 29435Advanced Centre for Treatment Research and Education in Cancer, Tata Memorial Centre, Navi Mumbai, India (Deshmukh) Centre for Interdisciplinary Research, D.Y. Patil University, Navi Mumbai, India

(Deshpande, Gota) Clinical Pharmacology Laboratory, 29435Advanced Centre for Treatment Research and Education in Cancer, Tata Memorial Centre, Navi Mumbai, India

(Gota, Chaturvedi, Krishna) Homi Bhabha National Institute, Training School Complex, India

(Chaturvedi) Department of Surgical Oncology, Tata Memorial Hospital, Mumbai, India

Publisher

NLM (Medline)

Emtree Heading

discriminant analysis; human; *mouth tumor/di [Diagnosis]; *Raman spectrometry; saliva; standard.

Other Index Terms

discriminant analysis; human; *mouth tumor / *diagnosis; *Raman spectrometry; saliva; standard.

Link to the Ovid Full Text or citation:

[Click here for full text options](https://ovidsp.ovid.com/ovidweb.cgi?T=JS&CSC=Y&NEWS=N&PAGE=fulltext&D=emed22&AN=633278953)

Link to the External Link Resolver:

[SFX](https://sfx-86scu.hosted.exlibrisgroup.com.cn/86scu?sid=OVID:embase&id=pmid:33107759&id=doi:10.1177%2F0003702820973260&issn=19433530&isbn=&volume=75&issue=5&spage=581&pages=581-588&date=2021&title=Applied+spectroscopy&atitle=Salivary+Raman+Spectroscopy%3A+Standardization+of+Sampling+Protocols+and+Stratification+of+Healthy+and+Oral+Cancer+Subjects&aulast=Hole&pid=<author>Hole+A.%3BTyagi+G.%3BDeshmukh+A.%3BDeshpande+R.%3BGota+V.%3BChaturvedi+P.%3BKrishna+C.M.<%2Fauthor><AN>633278953<%2FAN><DT>Article<%2FDT>)

80.

Single cell detection using intracellularly-grown-Au-nanoparticle based surface-enhanced Raman scattering spectroscopy for nasopharyngeal cell line classification.

Chen W., Xu S., Wang X., Wei G., Hong Q., Huang H., Yu Y.

Analytical methods : advancing methods and applications. 13(28) (pp 3147-3153), 2021. Date of Publication: 28 Jul 2021.

AN: 635431046

The aim of this study was to evaluate the feasibility of applying intracellularly-grown-Au-nanoparticle (IGAuNP)-based surface-enhanced Raman scattering (SERS) technology to classify two types of nasopharyngeal cancer (NPC) cell lines (CNE2 and CNE1). The IGAuNP technology provides excellent delivery efficiency of Au NPs to the cytoplasm and nucleus, thus leading to an extraordinary enhancement of the Raman signals of cells. Compared with normal Raman scattering (NRS) spectra of cells, IGAuNP-based SERS spectra not only have a high signal-to-noise ratio, but also can detect more characteristic Raman peaks, which can be used to explore more differences when comparing the biochemical components of different nasopharyngeal carcinoma cell lines. Based on the linear discriminant analysis (LDA) and support vector machine (SVM) analysis of SERS spectral data, an exciting result with a diagnostic sensitivity of 100%, specificity of 100%, and accuracy of 100%, could be achieved to differentiate CNE2 and CNE1 cells, which is better than the result obtained by NRS spectroscopy. This exploratory study indicated that the SERS technology based on IGAuNPs in conjunction with multivariate statistical analysis methods has great potential in the identification of nasopharyngeal carcinoma cell lines.

PMID

34159968 [<https://www.ncbi.nlm.nih.gov/pubmed/?term=34159968>]

Institution

(Chen) College of Integrated Traditional Chinese and Western Medicine, Fujian University of Traditional Chinese Medicine, Fuzhou 350122, China

Publisher

NLM (Medline)

Emtree Heading

cell line; human; nasopharynx carcinoma/di [Diagnosis]; *nasopharynx tumor/di [Diagnosis]; Raman spectrometry; *nanoparticle.

Drug Index Terms

*nanoparticle.

Other Index Terms

cell line; human; nasopharynx carcinoma / diagnosis; *nasopharynx tumor / *diagnosis; Raman spectrometry.

Link to the Ovid Full Text or citation:

[Click here for full text options](https://ovidsp.ovid.com/ovidweb.cgi?T=JS&CSC=Y&NEWS=N&PAGE=fulltext&D=emed22&AN=635431046)

Link to the External Link Resolver:

[SFX](https://sfx-86scu.hosted.exlibrisgroup.com.cn/86scu?sid=OVID:embase&id=pmid:34159968&id=doi:10.1039%2Fd1ay00554e&issn=17599679&isbn=&volume=13&issue=28&spage=3147&pages=3147-3153&date=2021&title=Analytical+methods+%3A+advancing+methods+and+applications&atitle=Single+cell+detection+using+intracellularly-grown-Au-nanoparticle+based+surface-enhanced+Raman+scattering+spectroscopy+for+nasopharyngeal+cell+line+classification&aulast=Chen&pid=<author>Chen+W.%3BXu+S.%3BWang+X.%3BWei+G.%3BHong+Q.%3BHuang+H.%3BYu+Y.<%2Fauthor><AN>635431046<%2FAN><DT>Article<%2FDT>)

81.

Label-free electrochemical immunosensor based on gold nanoparticle/polyethyleneimine/reduced graphene oxide nanocomposites for the ultrasensitive detection of cancer biomarker matrix metalloproteinase-1.

Liu X., Lin L.-Y., Tseng F.-Y., Tan Y.-C., Li J., Feng L., Song L., Lai C.-F., Li X., He J.-H., Sakthivel R., Chung R.-J.

The Analyst. 146(12) (pp 4066-4079), 2021. Date of Publication: 14 Jun 2021.

AN: 635194360

Matrix metalloproteinase-1 (MMP-1) is associated with many types of cancers, including oral, colorectal, and brain cancers. This paper describes the fabrication of an MMP-1 immunosensor based on a gold nanoparticle/polyethyleneimine/reduced graphene oxide (AuNP/PEI/rGO)-modified disposable screen-printed electrode (SPE). A microwave-assisted single-step method was employed for the simultaneous reduction of gold and graphene oxide in a PEI environment to avoid AuNP agglomeration. The crystal structure, chemical composition, optical properties, and interior morphology of the materials were probed by X-ray diffraction, Raman spectroscopy, UV-visible spectrometry, and transmission electron microscopy techniques. To assemble a label-free MMP-1 immunosensor layer-by-layer, 3-mercaptopropionic acid was utilized due to its strong sulfur-gold bonding ability, and its tail end was attached to a carboxyl group, allowing the MMP-1 antibody (anti-MMP-1) to be subsequently cross-linked using the traditional N-(3-dimethylaminopropyl) and N' ethylcarbodiimide hydrochloride method. Differential pulse voltammetry analysis showed a linear relationship with MMP-1 concentration in the range of 1-50 ng ml-1 with an R2 value of ~0.996 (n = 5, RSD < 5%). This immunosensor was successfully applied for MMP-1 detection in urine, saliva, bovine serum, and cell culture media (HSC-3 & C6) of oral and brain cancers showing results comparable to those of the credible ELISA method.

PMID

34048512 [<https://www.ncbi.nlm.nih.gov/pubmed/?term=34048512>]

Author NameID

Chung, Ren-Jei; ORCID: <https://orcid.org/0000-0002-0655-3680>

Institution

(Liu) College of Materials Science and Engineering, Shenzhen University, Shenzhen 518060, China. lxh@szu.edu.cn and Department of Electrical and Computer Engineering, National University of Singapore, Singapore 117583, Singapore (Lin, Tseng, Tan, Sakthivel) Department of Chemical Engineering and Biotechnology, National Taipei University of Technology (Taipei Tech), Taipei 10608, Taiwan (Republic of China)

(Li, Feng, Li) College of Materials Science and Engineering, Shenzhen University, Shenzhen 518060, China

(Song) Research Center of Guangdong Intelligent Charging and System Integration Engineering Technology, Ltd, Shenzhen Winsemi Microelectronics Co., Shenzhen 518000, China

(Lai) DFON Biomedical Technology Inc., Taipei 10608, Taiwan (Republic of China)

(He) Department of Chemical Engineering and Biotechnology, National Taipei University of Technology (Taipei Tech), Taiwan. and Department of Materials Science and Engineering, City University of Hong Kong, Hong Kong, Taipei 10608, Taiwan (Republic of China)

(Chung) Department of Chemical Engineering and Biotechnology, National Taipei University of Technology (Taipei Tech), Taipei 10608, Taiwan (Republic of China)

Publisher

NLM (Medline)

Emtree Heading

animal; bovine; electrochemical analysis; electrode; *genetic procedures; immunoassay; limit of detection; *neoplasm; gold; graphene oxide; *graphite; interstitial collagenase; *metal nanoparticle; *nanocomposite; polyethyleneimine; tumor marker.

Drug Index Terms

gold; graphene oxide; *graphite; interstitial collagenase; *metal nanoparticle; *nanocomposite; polyethyleneimine; tumor marker.

Other Index Terms

animal; bovine; electrochemical analysis; electrode; *genetic procedures; immunoassay; limit of detection; *neoplasm.

Link to the Ovid Full Text or citation:

[Click here for full text options](https://ovidsp.ovid.com/ovidweb.cgi?T=JS&CSC=Y&NEWS=N&PAGE=fulltext&D=emed22&AN=635194360)

Link to the External Link Resolver:

[SFX](https://sfx-86scu.hosted.exlibrisgroup.com.cn/86scu?sid=OVID:embase&id=pmid:34048512&id=doi:10.1039%2Fd1an00537e&issn=13645528&isbn=&volume=146&issue=12&spage=4066&pages=4066-4079&date=2021&title=The+Analyst&atitle=Label-free+electrochemical+immunosensor+based+on+gold+nanoparticle%2Fpolyethyleneimine%2Freduced+graphene+oxide+nanocomposites+for+the+ultrasensitive+detection+of+cancer+biomarker+matrix+metalloproteinase-1&aulast=Liu&pid=<author>Liu+X.%3BLin+L.-Y.%3BTseng+F.-Y.%3BTan+Y.-C.%3BLi+J.%3BFeng+L.%3BSong+L.%3BLai+C.-F.%3BLi+X.%3BHe+J.-H.%3BSakthivel+R.%3BChung+R.-J.<%2Fauthor><AN>635194360<%2FAN><DT>Article<%2FDT>)

82.

Spectroscopic, quantum chemical and molecular docking studies on 1-amino-5-chloroanthraquinone: A targeted drug therapy for thyroid cancer.

Valarmathi T., Premkumar R., Meera M.R., Milton Franklin Benial A.

Spectrochimica acta. Part A, Molecular and biomolecular spectroscopy. 255 (pp 119659), 2021. Date of Publication: 05 Jul 2021.

AN: 634636688

The DFT studies of the 1-Amino-5-chloro-anthraquinone (ACAQ) molecule have been carried out with extensive and accurate investigations of detailed vibrational and spectroscopic investigations and validated by experimentally. The optimized molecular structure and harmonic resonance frequencies were computed based on DFT/B3LYP method with 6-311G++(d,p) basis set using the Gaussian 09 program. The experimental and calculated vibrational wavenumbers were assigned on the basis of PED calculations using VEDA 4.0 program. The 13C NMR isotropic chemical shifts of the molecule were calculated using Gauge-Invariant-Atomic Orbital (GIAO) method in DMSO solution and compared with the experimental data. The absorption spectrum of the molecule was computed in liquid phase (ethanol), which exhibits to * electronic transition and compared with observed UV-Vis spectrum. Frontier molecular orbitals analysis shows the molecular reactivity and kinetic stability of the molecule. The Mulliken atomic charge distribution and molecular electrostatic potential surface analysis of the molecule validate the reactive site of the molecule. The natural bond orbital analysis proves the bioactivity of the molecule. Molecular docking analysis indicate that ACAQ molecule inhibits the action of c-Met Kinase protein, which is associated with the thyroid cancer. Hence, the present study pave the way for the development of novel drugs in the treatment of thyroid cancer.

Copyright © 2021 Elsevier B.V. All rights reserved.

PMID

33751957 [<https://www.ncbi.nlm.nih.gov/pubmed/?term=33751957>]

Institution

(Valarmathi, Premkumar, Milton Franklin Benial) P.G. and Research Department of Physics, N.M.S.S.V.N. College, Madurai, Tamil Nadu 625019, India (Meera) Department of Physics, Sree Ayyappa College for Women, Tamil Nadu, India

Publisher

NLM (Medline)

Emtree Heading

conformation; human; infrared spectroscopy; molecular docking; molecular model; quantum theory; Raman spectrometry; thermodynamics; *thyroid tumor; ultraviolet spectrophotometry; *drug.

Drug Index Terms

*drug.

Other Index Terms

conformation; human; infrared spectroscopy; molecular docking; molecular model; quantum theory; Raman spectrometry; thermodynamics; *thyroid tumor; ultraviolet spectrophotometry.

Link to the Ovid Full Text or citation:

[Click here for full text options](https://ovidsp.ovid.com/ovidweb.cgi?T=JS&CSC=Y&NEWS=N&PAGE=fulltext&D=emed22&AN=634636688)

Link to the External Link Resolver:

[SFX](https://sfx-86scu.hosted.exlibrisgroup.com.cn/86scu?sid=OVID:embase&id=pmid:33751957&id=doi:10.1016%2Fj.saa.2021.119659&issn=18733557&isbn=&volume=255&issue=&spage=119659&pages=119659&date=2021&title=Spectrochimica+acta.+Part+A%2C+Molecular+and+biomolecular+spectroscopy&atitle=Spectroscopic%2C+quantum+chemical+and+molecular+docking+studies+on+1-amino-5-chloroanthraquinone%3A+A+targeted+drug+therapy+for+thyroid+cancer&aulast=Valarmathi&pid=<author>Valarmathi+T.%3BPremkumar+R.%3BMeera+M.R.%3BMilton+Franklin+Benial+A.<%2Fauthor><AN>634636688<%2FAN><DT>Article<%2FDT>)

83.

Biomedical applications of vibrational spectroscopy: Oral cancer diagnostics.

Byrne H.J., Behl I., Calado G., Ibrahim O., Toner M., Galvin S., Healy C.M., Flint S., Lyng F.M.

Spectrochimica acta. Part A, Molecular and biomolecular spectroscopy. 252 (pp 119470), 2021. Date of Publication: 05 May 2021.

AN: 634139843

Vibrational spectroscopy, based on either infrared absorption or Raman scattering, has attracted increasing attention for biomedical applications. Proof of concept explorations for diagnosis of oral potentially malignant disorders and cancer are reviewed, and recent advances critically appraised. Specific examples of applications of Raman microspectroscopy for analysis of histological, cytological and saliva samples are presented for illustrative purposes, and the future prospects, ultimately for routine, chairside in vivo screening are discussed.

Copyright © 2021 Elsevier B.V. All rights reserved.

PMID

33503511 [<https://www.ncbi.nlm.nih.gov/pubmed/?term=33503511>]

Institution

(Byrne) FOCAS Research Institute, Technological University Dublin, City Campus, Dublin 8, Ireland (Behl, Calado, Lyng) School of Physics and Clinical and Optometric Sciences, Technological University Dublin, City Campus, Dublin 8, Ireland; Radiation and Environmental Science Centre, FOCAS Research Institute, Technological University Dublin, City Campus, Dublin 8, Ireland

(Ibrahim) School of Dental Science, Trinity College Dublin, Lincoln Place, Dublin 2, Ireland

(Toner) Central Pathology Laboratory, St. James Hospital, James Street, Dublin 8, Ireland

(Galvin, Healy, Flint) Oral Medicine Unit, Dublin Dental University Hospital, Trinity College Dublin, Lincoln Place, Dublin 2, Ireland

Publisher

NLM (Medline)

Emtree Heading

human; *mouth tumor/di [Diagnosis]; *Raman spectrometry; vibration.

Other Index Terms

human; *mouth tumor / *diagnosis; *Raman spectrometry; vibration.

Link to the Ovid Full Text or citation:

[Click here for full text options](https://ovidsp.ovid.com/ovidweb.cgi?T=JS&CSC=Y&NEWS=N&PAGE=fulltext&D=emed22&AN=634139843)

Link to the External Link Resolver:

[SFX](https://sfx-86scu.hosted.exlibrisgroup.com.cn/86scu?sid=OVID:embase&id=pmid:33503511&id=doi:10.1016%2Fj.saa.2021.119470&issn=18733557&isbn=&volume=252&issue=&spage=119470&pages=119470&date=2021&title=Spectrochimica+acta.+Part+A%2C+Molecular+and+biomolecular+spectroscopy&atitle=Biomedical+applications+of+vibrational+spectroscopy%3A+Oral+cancer+diagnostics&aulast=Byrne&pid=<author>Byrne+H.J.%3BBehl+I.%3BCalado+G.%3BIbrahim+O.%3BToner+M.%3BGalvin+S.%3BHealy+C.M.%3BFlint+S.%3BLyng+F.M.<%2Fauthor><AN>634139843<%2FAN><DT>Review<%2FDT>)

84.

Rapid and noninvasive diagnosis of oral and oropharyngeal cancer based on micro-Raman and FT-IR spectra of saliva.

Falamas A., Faur C.I., Ciupe S., Chirila M., Rotaru H., Hedesiu M., Cinta Pinzaru S.

Spectrochimica acta. Part A, Molecular and biomolecular spectroscopy. 252 (pp 119477), 2021. Date of Publication: 05 May 2021.

AN: 634209717

Fast, sensitive, and noninvasive techniques are needed for better health care management, particularly when traditional biopsies could be replaced with appropriate analyses of body fluids, such as saliva. Here is presented a proof-of-concept study, which aims to test a recently developed saliva samples preparation method, for oral and oropharyngeal cancer diagnosis, using micro-Raman and Fourier transform infrared (FT-IR) spectroscopic techniques. The detected biomarker bands and the cancer classification rates are compared and discussed. Saliva samples were collected from healthy donors and pathologically confirmed oral and oropharyngeal cancer patients. Principal components analysis (PCA) and principal components analysis-linear discriminant analysis (PCA-LDA) chemometric methods were applied to build discrimination models for the test and control groups. Based on the differences between salivary spectra of healthy and cancer patients, several biomarker bands were identified. Noteworthy, a significant vibrational biomarker band at 2064cm-1, assigned to thiocyanate, was observed in both the FT-IR and Raman data-set. Other cancer characteristic Raman bands were 754cm-1 (tryptophan), 530 and 927cm-1 (lysozyme), 1001cm-1 (phenylalanine), while the FT-IR biomarker band was located at 1075cm-1 (phosphodiester bonds stretching in DNA, RNA). The oral and oropharyngeal cancer was classified with an accuracy of 90% based on the micro-Raman data and 82% based on the FT-IR data set, respectively. The study showed that oral and oropharyngeal cancer can be differentiated from control saliva samples based on their respective micro-Raman and FT-IR spectral signatures, due to the biomolecular modifications induced by the disease.

Copyright © 2021 Elsevier B.V. All rights reserved.

PMID

33545509 [<https://www.ncbi.nlm.nih.gov/pubmed/?term=33545509>]

Institution

(Falamas, Ciupe) Molecular and Biomolecular Physics, National Institute for Research and Development of Isotopic and Molecular Technologies, Cluj-Napoca, Romania (Faur, Rotaru, Hedesiu) Department of Maxillofacial Surgery and Radiology, "Iuliu Hatieganu" University of Medicine and Pharmacy, Cluj-Napoca, Romania

(Chirila) Department of ENT, "Iuliu Hatieganu" University of Medicine and Pharmacy, Cluj-Napoca, Romania

(Cinta Pinzaru) Biomolecular Physics Department, Faculty of Physics, Babes-Bolyai University, Cluj-Napoca, Romania

Publisher

NLM (Medline)

Emtree Heading

discriminant analysis; Fourier analysis; human; *infrared spectroscopy; *oropharynx tumor/di [Diagnosis]; Raman spectrometry; *saliva.

Other Index Terms

discriminant analysis; Fourier analysis; human; *infrared spectroscopy; *oropharynx tumor / *diagnosis; Raman spectrometry; *saliva.

Link to the Ovid Full Text or citation:

[Click here for full text options](https://ovidsp.ovid.com/ovidweb.cgi?T=JS&CSC=Y&NEWS=N&PAGE=fulltext&D=emed22&AN=634209717)

Link to the External Link Resolver:

[SFX](https://sfx-86scu.hosted.exlibrisgroup.com.cn/86scu?sid=OVID:embase&id=pmid:33545509&id=doi:10.1016%2Fj.saa.2021.119477&issn=18733557&isbn=&volume=252&issue=&spage=119477&pages=119477&date=2021&title=Spectrochimica+acta.+Part+A%2C+Molecular+and+biomolecular+spectroscopy&atitle=Rapid+and+noninvasive+diagnosis+of+oral+and+oropharyngeal+cancer+based+on+micro-Raman+and+FT-IR+spectra+of+saliva&aulast=Falamas&pid=<author>Falamas+A.%3BFaur+C.I.%3BCiupe+S.%3BChirila+M.%3BRotaru+H.%3BHedesiu+M.%3BCinta+Pinzaru+S.<%2Fauthor><AN>634209717<%2FAN><DT>Article<%2FDT>)

85.

Centimeter-scale gas-sieving nanoporous single-layer graphene membrane.

Lee W.-C., Bondaz L., Huang S., He G., Dakhchoune M., Agrawal K.V.

Journal of Membrane Science. 618 (no pagination), 2021. Article Number: 118745. Date of Publication: 15 January 2021.

AN: 2007973956

High-permeance, molecular-sieving, nanoporous single-layer graphene (NSLG) membranes are highly promising for gas separation. However, the formation of cracks during the transfer of NSLG to a low-cost porous support is difficult to avoid. These cracks are detrimental to gas selectivity, and therefore, make the scale-up of the gas-sieving NSLG membranes challenging. To mitigate the crack formation on low-cost macroporous supports, herein, we demonstrate mechanical reinforcement of the graphene film with a two-layer composite carbon film. The bottom layer of the composite film is a 100-nm-thick block-copolymer film derived nanoporous carbon (NPC) film with a pore size of 20-30 nm. This layer makes an intimate contact with NSLG and prevents generation of crack. However, the NPC film by itself is not robust enough to cover the rough surface of low-cost macroporous supports and tends to generate occasional cracks. This is prevented by spin-coating a 500-nm-thick multi-walled carbon nanotube (MWNT) film, hosting pore size of 200-300 nm, on top of the NPC film. This imparts enough mechanical strength to NSLG/NPC film to be successfully suspended on a low-cost, macroporous, nonwoven metal wire mesh on a centimeter-scale while completely avoiding cracks. As a result, H2/CH4 and H2/CO2 selectivities of 11-23 and 5-8, respectively, higher than the corresponding Knudsen selectivities of 2.8 and 4.7, respectively, are obtained from the centimeter-scale NSLG membranes. The reinforced membranes are mechanically robust and can successfully withstand transmembrane pressure difference of 4 bar. When the MWNT film is directly coated on NSLG without using the intermediate NPC layer, the gas sieving behavior is not observed, likely due to the development of nanoscale cracks. This underlines the crucial role of the hierarchical pore structure in the composite carbon film in realizing the gas-sieving graphene membranes.

Copyright © 2020 The Author(s)

Author NameID

Bondaz, Luc; ORCID: <https://orcid.org/0000-0002-0675-0289>

Institution

(Lee, Bondaz, Huang, He, Dakhchoune, Agrawal) Laboratory of Advanced Separations, Ecole Polytechnique Federale de Lausanne, Sion, Switzerland

Publisher

Elsevier B.V.

Emtree Heading

article; chemical vapor deposition; concentration (parameter); cost; *gas; gas permeability; gas transport; *material state; mechanics; molecular mechanics; *nanofabrication; phase separation; pore size; pressure; priority journal; Raman spectrometry; scale up; scanning electron microscopy; spin coating; strength; surface property; transmission electron microscopy; carbon dioxide; copolymer; *graphene; hydrogen; methane; multi walled nanotube; nanocoating; nanocomposite; nanofilm; stainless steel; unclassified drug; crack; low pressure chemical vapor deposition; *molecular sieving; *nanoporous single layer graphene; poly(styrene b 4 vinyl pyridine).

Candidate Terms

crack [other term] low pressure chemical vapor deposition [other term]

*molecular sieving [other term]

*nanoporous single layer graphene [drug term]

poly(styrene b 4 vinyl pyridine) [drug term]

Drug Index Terms

carbon dioxide; copolymer; *graphene; hydrogen; methane; multi walled nanotube; nanocoating; nanocomposite; nanofilm; stainless steel; unclassified drug.

Other Index Terms

Article; chemical vapor deposition; concentration (parameter); cost; *gas; gas permeability; gas transport; *material state; mechanics; molecular mechanics; *nanofabrication; phase separation; pore size; pressure; priority journal; Raman spectrometry; scale up; scanning electron microscopy; spin coating; strength; surface property; transmission electron microscopy.

Link to the Ovid Full Text or citation:

[Click here for full text options](https://ovidsp.ovid.com/ovidweb.cgi?T=JS&CSC=Y&NEWS=N&PAGE=fulltext&D=emed22&AN=2007973956)

Link to the External Link Resolver:

[SFX](https://sfx-86scu.hosted.exlibrisgroup.com.cn/86scu?sid=OVID:embase&id=pmid:&id=doi:10.1016%2Fj.memsci.2020.118745&issn=03767388&isbn=&volume=618&issue=&spage=118745&pages=&date=2021&title=Journal+of+Membrane+Science&atitle=Centimeter-scale+gas-sieving+nanoporous+single-layer+graphene+membrane&aulast=Lee&pid=<author>Lee+W.-C.%3BBondaz+L.%3BHuang+S.%3BHe+G.%3BDakhchoune+M.%3BAgrawal+K.V.<%2Fauthor><AN>2007973956<%2FAN><DT>Article<%2FDT>)

86.

Erratum: Assessment of radiotherapy effect for nasopharyngeal cancer using plasma surface-enhanced Raman spectroscopy technology (Biomedical Optics Express (2018) 9 (3413) DOI: 10.1364/BOE.9.003413).

Wu Q., Qiu S., Yu Y., Chen W., Lin H., Lin D., Feng S., Chen R.

Biomedical Optics Express. 12(5) (pp 2557-2558), 2021. Date of Publication: May 2021.

AN: 2011856866

The authors regret that in the original manuscript the TEM image of Ag nanoparticles was accidentally misused in Fig. 1 in [1]. The correct Fig. 1 is shown below. The authors apologize for this error and any inconvenience to readers. (Figure Presented).

Copyright © 2021 OSA - The Optical Society. All rights reserved.

Institution

(Wu, Yu, Lin, Lin, Feng, Chen) Fujian Normal University, Key Laboratory of OptoElectronic Science and Technology for Medicine, Ministry of Education, Fujian Provincial Key Laboratory for Photonics Technology, Fuzhou 350007, China (Qiu) Department of Radiation Oncology, Fujian Provincial Cancer Hospital, Fujian Medical University Cancer Hospital, Fujian Provincial Key Laboratory of Translational Cancer Medicine, Fuzhou 350014, China

(Yu, Chen, Lin) College of Integrated Traditional Chinese and Western Medicine, Fujian University of Traditional Chinese Medicine, Fuzhou 350122, China

Publisher

The Optical Society

Emtree Heading

*erratum.

Other Index Terms

*erratum.

Link to the Ovid Full Text or citation:

[Click here for full text options](https://ovidsp.ovid.com/ovidweb.cgi?T=JS&CSC=Y&NEWS=N&PAGE=fulltext&D=emed22&AN=2011856866)

Link to the External Link Resolver:

[SFX](https://sfx-86scu.hosted.exlibrisgroup.com.cn/86scu?sid=OVID:embase&id=pmid:&id=doi:10.1364%2FBOE.426301&issn=21567085&isbn=&volume=12&issue=5&spage=2557&pages=2557-2558&date=2021&title=Biomedical+Optics+Express&atitle=Erratum%3A+Assessment+of+radiotherapy+effect+for+nasopharyngeal+cancer+using+plasma+surface-enhanced+Raman+spectroscopy+technology+(Biomedical+Optics+Express+(2018)+9+(3413)+DOI%3A+10.1364%2FBOE.9.003413)&aulast=Wu&pid=<author>Wu+Q.%3BQiu+S.%3BYu+Y.%3BChen+W.%3BLin+H.%3BLin+D.%3BFeng+S.%3BChen+R.<%2Fauthor><AN>2011856866<%2FAN><DT>Erratum<%2FDT>)

adult; cytology; female; human; male; middle aged; mouth; pathology; pilot study; *Raman spectrometry; receiver operating characteristic.

Link to the Ovid Full Text or citation:

[Click here for full text options](https://ovidsp.ovid.com/ovidweb.cgi?T=JS&CSC=Y&NEWS=N&PAGE=fulltext&D=emed15&AN=606344553)

Link to the External Link Resolver:

[SFX](https://sfx-86scu.hosted.exlibrisgroup.com.cn/86scu?sid=OVID:embase&id=pmid:23821433&id=doi:10.1002%2Fjbio.201300030&issn=18640648&isbn=&volume=7&issue=9&spage=690&pages=690-702&date=2014&title=Journal+of+biophotonics&atitle=In+vivo+Raman+spectroscopy+for+detection+of+oral+neoplasia%3A+a+pilot+clinical+study&aulast=Krishna&pid=<author>Krishna+H.%3BMajumder+S.K.%3BChaturvedi+P.%3BSidramesh+M.%3BGupta+P.K.<%2Fauthor><AN>606344553<%2FAN><DT>Article<%2FDT>)

283.

Raman mapping of oral buccal mucosa: a spectral histopathology approach.

Behl I., Kukreja L., Deshmukh A., Singh S.P., Mamgain H., Hole A.R., Krishna C.M.

Journal of biomedical optics. 19(12) (pp 126005), 2014. Date of Publication: 01 Dec 2014.

AN: 605431243

Oral cancer is one of the most common cancers worldwide. One-fifth of the world's oral cancer subjects are from India and other South Asian countries. The present Raman mapping study was carried out to understand biochemical variations in normal and malignant oral buccal mucosa. Data were acquired using WITec alpha 300R instrument from 10 normal and 10 tumors unstained tissue sections. Raman maps of normal sections could resolve the layers of epithelium, i.e. basal, intermediate, and superficial. Inflammatory, tumor, and stromal regions are distinctly depicted on Raman maps of tumor sections. Mean and difference spectra of basal and inflammatory cells suggest abundance of DNA and carotenoids features. Strong cytochrome bands are observed in intermediate layers of normal and stromal regions of tumor. Epithelium and stromal regions of normal cells are classified by principal component analysis. Classification among cellular components of normal and tumor sections is also observed. Thus, the findings of the study further support the applicability of Raman mapping for providing molecular level insights in normal and malignant conditions.

PMID

25478870 [<https://www.ncbi.nlm.nih.gov/pubmed/?term=25478870>]

Institution

(Behl) Chilakapati Lab, ACTREC, Kharghar, Navi-Mumbai 410210, India (Kukreja) Chilakapati Lab, ACTREC, Kharghar, Navi-Mumbai 410210, India

(Deshmukh) Chilakapati Lab, ACTREC, Kharghar, Navi-Mumbai 410210, India

(Singh) Chilakapati Lab, ACTREC, Kharghar, Navi-Mumbai 410210, India

(Mamgain) WITec GmbH, Lisemeitner-str.6, D-89081 Ulm, Germany

(Hole) Chilakapati Lab, ACTREC, Kharghar, Navi-Mumbai 410210, India

(Krishna) Chilakapati Lab, ACTREC, Kharghar, Navi-Mumbai 410210, India

Emtree Heading

*chemistry; cytochemistry; human; mouth mucosa; mouth tumor; *pathology; principal component analysis; *procedures; Raman spectrometry.

Other Index Terms

*chemistry; cytochemistry; human; mouth mucosa; mouth tumor; *pathology; principal component analysis; *procedures; Raman spectrometry.

Link to the Ovid Full Text or citation:

[Click here for full text options](https://ovidsp.ovid.com/ovidweb.cgi?T=JS&CSC=Y&NEWS=N&PAGE=fulltext&D=emed15&AN=605431243)

Link to the External Link Resolver:

[SFX](https://sfx-86scu.hosted.exlibrisgroup.com.cn/86scu?sid=OVID:embase&id=pmid:25478870&id=doi:10.1117%2F1.JBO.19.12.126005&issn=15602281&isbn=&volume=19&issue=12&spage=126005&pages=126005&date=2014&title=Journal+of+biomedical+optics&atitle=Raman+mapping+of+oral+buccal+mucosa%3A+a+spectral+histopathology+approach&aulast=Behl&pid=<author>Behl+I.%3BKukreja+L.%3BDeshmukh+A.%3BSingh+S.P.%3BMamgain+H.%3BHole+A.R.%3BKrishna+C.M.<%2Fauthor><AN>605431243<%2FAN><DT>Article<%2FDT>)

284.

A new nanotechnology technique for determining drug efficacy using targeted plasmonically enhanced single cell imaging spectroscopy.

Austin L.A., Kang B., El-Sayed M.A.

Journal of the American Chemical Society. 135(12) (pp 4688-4691), 2013. Date of Publication: 27 Mar 2013.

AN: 368642476

Recently, we described a new technique, targeted plasmonically enhanced single cell imaging spectroscopy (T-PESCIS), which exploits the plasmonic properties of gold nanoparticles, e.g. gold nanospheres, to simultaneously obtain enhanced intracellular Raman molecular spectra and enhanced Rayleigh cell scattering images throughout the entire span of a single cell cycle. In the present work, we demonstrate the use of T-PESCIS in evaluating the relative efficacy and dynamics of two popular chemotherapy drugs on human oral squamous carcinoma (HSC-3) cells. T-PESCIS revealed three plasmonically enhanced Raman scattering vibration bands, 500, 1000, and 1585 cm-1, associated with the cellular death dynamics. Detailed analysis indicated that the decrease in the 500 cm-1 band did not correlate well with drug efficacy but could indicate death initiation. The time it takes for the relative intensity of either the 1000 or 1585 cm-1 band ("SERS death" bands) to appear and increase to its maximum value after the injection of a known concentration of the drug can be related to the drug's efficacy. The inverse ratio, termed cell death enhancement factor, of these characteristic death times when using either band, especially the spectrally sharp band at 1000 cm -1, gave the correct drug efficacy ratio as determined by the commonly used XTT cell viability assay method. These results strongly suggest the potential future use of this technique in determining the efficacy, dynamics, and molecular mechanisms of various drugs against different diseases. © 2013 American Chemical Society.

PMID

23469948 [<https://www.ncbi.nlm.nih.gov/pubmed/?term=23469948>]

Institution

(Austin, Kang, El-Sayed) Laser Dynamics Laboratory, School of Chemistry and Biochemistry, Georgia Institute of Technology, Atlanta, GA 30332-0400, United States (Kang) College of Material Science and Technology, Nanjing University of Aeronautics and Astronautics, Nanjing 210016, China

Publisher

American Chemical Society (2540 Olentangy River Road, P.O. Box 3337, Columbus OH 43210-3337, United States)

Emtree Heading

article; cell death; cell viability; concentration (parameters); drug determination; drug efficacy; drug targeting; human; *nanotechnology; Raman spectrometry; *spectroscopy; squamous cell carcinoma; cisplatin; fluorouracil; *targeted plasmonically enhanced single cell imaging spectroscopy.

Candidate Terms

*targeted plasmonically enhanced single cell imaging spectroscopy [other term]

Drug Index Terms

cisplatin; fluorouracil.

Other Index Terms

article; cell death; cell viability; concentration (parameters); drug determination; drug efficacy; drug targeting; human; *nanotechnology; Raman spectrometry; *spectroscopy; squamous cell carcinoma.

Link to the Ovid Full Text or citation:

[Click here for full text options](https://ovidsp.ovid.com/ovidweb.cgi?T=JS&CSC=Y&NEWS=N&PAGE=fulltext&D=emed14&AN=368642476)

Link to the External Link Resolver:

[SFX](https://sfx-86scu.hosted.exlibrisgroup.com.cn/86scu?sid=OVID:embase&id=pmid:23469948&id=doi:10.1021%2Fja4011145&issn=00027863&isbn=&volume=135&issue=12&spage=4688&pages=4688-4691&date=2013&title=Journal+of+the+American+Chemical+Society&atitle=A+new+nanotechnology+technique+for+determining+drug+efficacy+using+targeted+plasmonically+enhanced+single+cell+imaging+spectroscopy&aulast=Austin&pid=<author>Austin+L.A.%3BKang+B.%3BEl-Sayed+M.A.<%2Fauthor><AN>368642476<%2FAN><DT>Article<%2FDT>)

285.

Raman spectroscopy demonstrates Amifostine induced preservation of bone mineralization patterns in the irradiated murine mandible.

Tchanque-Fossuo C.N., Gong B., Poushanchi B., Donneys A., Sarhaddi D., Gallagher K.K., Deshpande S.S., Goldstein S.A., Morris M.D., Buchman S.R.

Bone. 52(2) (pp 712-717), 2013. Date of Publication: February 2013.

AN: 52337028

Purpose: Adjuvant radiotherapy in the management of head and neck cancer remains severely debilitating. Fortunately, newly developed agents aimed at decreasing radiation-induced damage have shown great promise. Amifostine (AMF) is a compound, which confers radio-protection to the exposed normal tissues, such as bone. Our intent is to utilize Raman spectroscopy to demonstrate how AMF preserves the mineral composition of the murine mandible following human equivalent radiation.

Method(s): Sprague Dawley rats were randomized into 3 experimental groups: control (n = 5), XRT (n = 5), and AMF-XRT (n = 5). Both XRT and AMF groups underwent bioequivalent radiation of 70. Gy in 5 fractions to the left hemimandible. AMF-XRT received Amifostine prior to radiation. Fifty-six days post-radiation, the hemimandibles were harvested, and Raman spectra were taken in the region of interest spanning 2. mm behind the last molar. Bone mineral and matrix-specific Raman bands were analyzed using one-way ANOVA, with statistical significance at p. <. 0.05.

Result(s): The full-width at half-maximum of the primary phosphate band (FWHM) and the ratio of carbonate/phosphate intensities demonstrated significant differences between AMF-XRT versus XRT (p. <. 0.01) and XRT versus control (p. <. 0.01). There was no difference between AMF-XRT and control (p. >. 0.05) in both Raman metrics. Computer-aided spectral subtraction further confirmed these results where AMF-XRT was spectrally similar to the control. Interestingly, the collagen cross-link ratio did not differ between XRT and AMF-XRT (p. <. 0.01) but was significantly different from the control (p. <. 0.01).

Conclusion(s): Our novel findings demonstrate that AMF prophylaxis maintains and protects bone mineral quality in the setting of radiation. Raman spectroscopy is an emerging and exceptionally attractive clinical translational technology to investigate and monitor both the destructive effects of radiation and the therapeutic remediation of AMF on the structural, physical and chemical qualities of bone. © 2012 Elsevier Inc.

PMID

22885239 [<https://www.ncbi.nlm.nih.gov/pubmed/?term=22885239>]

Institution

(Tchanque-Fossuo, Poushanchi, Donneys, Sarhaddi, Deshpande, Buchman) University of Michigan Hospital and Health Systems, Pediatric Plastic Surgery Section, 1540 E. Hospital Drive, MI 48109, United States (Gong, Morris) University of Michigan Hospital and Health Systems, Department of Chemistry, 930 N. University, Ann Arbor, MI 48109, United States

(Gallagher) University of Michigan Hospital and Health Systems, Department of Otolaryngology-Head and Neck Surgery, University of Michigan Medical School, 1500 E. Medical Center Drive, Ann Arbor, MI 48109, United States

(Goldstein) University of Michigan, Department of Orthopaedic Surgery, A. Alfred Taubman Biomedical Science Research Building, 109 Zina Pitcher Pl, Ann Arbor, MI, 48109, United States

Publisher

Elsevier Inc. (360 Park Avenue South, New York NY 10010, United States)

Emtree Heading

animal experiment; animal tissue; article; bone mineral; *bone mineralization; bone strength; bone structure; controlled study; diode laser; drug mechanism; intermethod comparison; male; *mandible; microscope; musculoskeletal system parameters; nonhuman; physical chemistry; protein cross linking; radiation dose; radiation protection; radiological parameters; *Raman spectrometry; randomization; rat; statistical significance; *tissue preservation; *amifostine/pd [Pharmacology]; *amifostine/sc [Subcutaneous Drug Administration]; carbonic acid; collagen; phosphate; bone quality.

Candidate Terms

bone quality [other term]

Drug Index Terms

*amifostine / *pharmacology / *subcutaneous drug administration; carbonic acid; collagen; phosphate.

Other Index Terms

animal experiment; animal tissue; article; bone mineral; *bone mineralization; bone strength; bone structure; controlled study; diode laser; drug mechanism; intermethod comparison; male; *mandible; microscope; musculoskeletal system parameters; nonhuman; physical chemistry; protein cross linking; radiation dose; radiation protection; radiological parameters; *Raman spectrometry; randomization; rat; statistical significance; *tissue preservation.

Drug Trade Name

ethyol: Medimmune [United States]

Link to the Ovid Full Text or citation:

[Click here for full text options](https://ovidsp.ovid.com/ovidweb.cgi?T=JS&CSC=Y&NEWS=N&PAGE=fulltext&D=emed14&AN=52337028)

Link to the External Link Resolver:

[SFX](https://sfx-86scu.hosted.exlibrisgroup.com.cn/86scu?sid=OVID:embase&id=pmid:22885239&id=doi:10.1016%2Fj.bone.2012.07.029&issn=87563282&isbn=&volume=52&issue=2&spage=712&pages=712-717&date=2013&title=Bone&atitle=Raman+spectroscopy+demonstrates+Amifostine+induced+preservation+of+bone+mineralization+patterns+in+the+irradiated+murine+mandible&aulast=Tchanque-Fossuo&pid=<author>Tchanque-Fossuo+C.N.%3BGong+B.%3BPoushanchi+B.%3BDonneys+A.%3BSarhaddi+D.%3BGallagher+K.K.%3BDeshpande+S.S.%3BGoldstein+S.A.%3BMorris+M.D.%3BBuchman+S.R.<%2Fauthor><AN>52337028<%2FAN><DT>Article<%2FDT>)

286.

Raman spectral analysis of nasopharyngeal carcinoma cell line CNE2 after microwave radiation.

Ye Y., Chen Y., Su Y., Zou C., Huang Y., Ou L., Chen R.

Biochemistry and Cell Biology. 91(2) (pp 67-71), 2013. Date of Publication: April 2013.

AN: 368653757

This study aimed to study the effects of microwave radiation on the nasopharyngeal carcinoma cell line CNE2 by Raman spectroscopy. The cells were separated into a control group and radiated groups with radiation times of 2, 5, 10, and 25 min, respectively. Both principal components analysis and support vector machine were employed for statistical analysis of Raman spectra. The results show that the relative content of C-H deformation and amide I begin to change when the radiation time is over 10 min, and principal components analysis further confirms there are significant differences after 10 min of radiation. Moreover, support vector machine is simultaneously used to classify radiated samples from control samples. The classification accuracy is low until the radiation time reaches over 10 min. In conclusion, this study reveals the Raman spectral characteristics of CNE2 under different microwave radiation exposure timesand demonstrates Raman spectroscopy can be a potential method to explore cellular characterization after radiation. The final results may help in elucidating the mechanism by which microwave radiation interacts with tumor cells. © 2013 Published by NRC Research Press.

PMID

23527634 [<https://www.ncbi.nlm.nih.gov/pubmed/?term=23527634>]

Institution

(Ye) College of Physics and Information, Fuzhou University, Fuzhou 350002, Fujian, China (Ye, Chen, Ou, Chen) Zhicheng College, Fuzhou University, Fuzhou 350002, Fujian, China

(Chen) Key Laboratory of Optoelectronic Science and Technology for Medicine, Ministry of Education, Fujian Normal University, Fuzhou 350007, Fujian, China

(Su, Zou) Fujian Provincial Tumor Hospital, Fuzhou 350014, Fujian, China

(Huang) Key Laboratory of Instrumentation Science and Dynamic Measurement (North University of China), Ministry of Education, North University of China, Taiyuan 030051, Shanxi, China

(Ye) No. 50, Yangqiao West Road, Gulou Area, Fuzhou, China

Publisher

National Research Council of Canada (Buiding M 55, Ottawa ONT K1A 0R6, Canada)

Emtree Heading

accuracy; article; cancer cell culture; controlled study; human; human cell; *microwave radiation; *nasopharynx carcinoma; principal component analysis; radiation exposure; *Raman spectrometry; statistical analysis; support vector machine; amide; carbon; hydrogen.

Drug Index Terms

amide; carbon; hydrogen.

Other Index Terms

accuracy; article; cancer cell culture; controlled study; human; human cell; *microwave radiation; *nasopharynx carcinoma; principal component analysis; radiation exposure; *Raman spectrometry; statistical analysis; support vector machine.

Link to the Ovid Full Text or citation:

[Click here for full text options](https://ovidsp.ovid.com/ovidweb.cgi?T=JS&CSC=Y&NEWS=N&PAGE=fulltext&D=emed14&AN=368653757)

Link to the External Link Resolver:

[SFX](https://sfx-86scu.hosted.exlibrisgroup.com.cn/86scu?sid=OVID:embase&id=pmid:23527634&id=doi:10.1139%2Fbcb-2012-0040&issn=08298211&isbn=&volume=91&issue=2&spage=67&pages=67-71&date=2013&title=Biochemistry+and+Cell+Biology&atitle=Raman+spectral+analysis+of+nasopharyngeal+carcinoma+cell+line+CNE2+after+microwave+radiation&aulast=Ye&pid=<author>Ye+Y.%3BChen+Y.%3BSu+Y.%3BZou+C.%3BHuang+Y.%3BOu+L.%3BChen+R.<%2Fauthor><AN>368653757<%2FAN><DT>Article<%2FDT>)

287.

Recent advancements in the diagnosis of oral premalignant and malignant lesions: A comprehensive review.

Gupta M., Aggarwal A., Ahuja R.

Clinical Cancer Investigation Journal. 2(3) (pp 181-184), 2013. Date of Publication: July-September 2013.

AN: 370127939

The diagnosis and treatment of lesions are currently based on histopathology, which is the gold standard for the diagnosis of cancer and dysplasia at the molecular level. Recently, there has been an increasing trend of optical spectroscopy methods for the detection of oral premalignant and malignant lesions and the same is highlighted in this paper. It depends on the optical spectrum derived from any tissue that contains information about the histological and biochemical make-up of that tissue. It provides tissue diagnosis in real-time, non-invasively, and in situ.

Institution

(Gupta) Department of Oral Medicine and Radiology, Shree Bankey Bihari Dental College and Research Centre, Ghaziabad, Uttar Pradesh, India (Gupta) Department of Oral Medicine and Radiology, Rishiraj College of Dental Sciences and Research Centre, Bhopal, Madhya Pradesh, India

(Aggarwal) Department of Oral Medicine and Radiology, Rajasthan Dental College, Jaipur, Rajasthan, India

(Ahuja) Department of Oral Medicine and Radiology, Kalka Dental College Hospital and Research Centre, Meerut, Uttar Pradesh, India

Publisher

Medknow Publications and Media Pvt. Ltd (B9, Kanara Business Centre, off Link Road, Ghatkopar (E), Mumbai 400 075, India)

Emtree Heading

antigen binding; autofluorescence; cancer prognosis; chromosome translocation; chronic myeloid leukemia; cytopathology; diagnostic test accuracy study; DNA microarray; DNA sequence; DNA strand; electrochemical impedance spectroscopy; flow cytometry; fluorescence; genetic transcription; histopathology; human; immunocytochemistry; immunohistochemistry; keratinocyte; malignant transformation; *mouth cancer/di [Diagnosis]; mouth squamous cell carcinoma/di [Diagnosis]; non invasive measurement; nuclear magnetic resonance spectroscopy; optical coherence tomography; optical resolution; oxygenation; photodynamic therapy; polymerase chain reaction; *precancer/di [Diagnosis]; priority journal; Raman spectrometry; review; RNA probe; signal transduction; T cell lymphoma; tumor suppressor gene; collagen/ec [Endogenous Compound]; elastin/ec [Endogenous Compound]; photosensitizing agent; protoporphyrin/ec [Endogenous Compound]; reduced nicotinamide adenine dinucleotide/ec [Endogenous Compound].

Drug Index Terms

collagen / endogenous compound; elastin / endogenous compound; photosensitizing agent; protoporphyrin / endogenous compound; reduced nicotinamide adenine dinucleotide / endogenous compound.

Other Index Terms

antigen binding; autofluorescence; cancer prognosis; chromosome translocation; chronic myeloid leukemia; cytopathology; diagnostic test accuracy study; DNA microarray; DNA sequence; DNA strand; electrochemical impedance spectroscopy; flow cytometry; fluorescence; genetic transcription; histopathology; human; immunocytochemistry; immunohistochemistry; keratinocyte; malignant transformation; *mouth cancer / *diagnosis; mouth squamous cell carcinoma / diagnosis; non invasive measurement; nuclear magnetic resonance spectroscopy; optical coherence tomography; optical resolution; oxygenation; photodynamic therapy; polymerase chain reaction; *precancer / *diagnosis; priority journal; Raman spectrometry; review; RNA probe; signal transduction; T cell lymphoma; tumor suppressor gene.

Link to the Ovid Full Text or citation:

[Click here for full text options](https://ovidsp.ovid.com/ovidweb.cgi?T=JS&CSC=Y&NEWS=N&PAGE=fulltext&D=emed14&AN=370127939)

Link to the External Link Resolver:

[SFX](https://sfx-86scu.hosted.exlibrisgroup.com.cn/86scu?sid=OVID:embase&id=pmid:&id=doi:10.4103%2F2278-0513.119249&issn=22780513&isbn=&volume=2&issue=3&spage=181&pages=181-184&date=2013&title=Clinical+Cancer+Investigation+Journal&atitle=Recent+advancements+in+the+diagnosis+of+oral+premalignant+and+malignant+lesions%3A+A+comprehensive+review&aulast=Gupta&pid=<author>Gupta+M.%3BAggarwal+A.%3BAhuja+R.<%2Fauthor><AN>370127939<%2FAN><DT>Review<%2FDT>)

288.

Optical techniques for the intraoperative assessment of nodal status.

Grootendorst D.J., Steenbergen W., Manohar S., Ruers T.J.M.

Future Oncology. 9(11) (pp 1741-1755), 2013. Date of Publication: November 2013.

AN: 370211030

The lymphatic system is an important pathway in the metastatic spread of many malignancies and a key prognostic indicator. Nondestructive assessment of the nodal status during surgery could limit the amount of lymph nodes that need to be resected and allow for immediate regional lymphadenectomy during sentinel lymph node biopsy procedures. This review looks into the possibilities of conventional medical imaging methods that are capable of intraoperative nodal assessment and discusses multiple newly developed optical techniques. The physical background behind these techniques is reviewed and a concise overview of their main advantages and disadvantages is provided. These recent innovations show that while the application of optical modalities for intraoperative nodal staging is not yet applied routinely, there is reason enough to expect their introduction in the near future. © 2013 Future Medicine Ltd.

PMID

24156334 [<https://www.ncbi.nlm.nih.gov/pubmed/?term=24156334>]

Institution

(Grootendorst, Steenbergen, Manohar) Biomedical Photonic Imaging Group, MIRA Institute for Biomedical Technology and Technical Medicine, Science and Technology, University of Twente, PO Box 217, 7500 AE Enschede, Netherlands (Ruers) Netherlands Cancer Institute, Antoni Van Leeuwenhoek Hospital, Netherlands

(Ruers) Nanobiophysics Group, MIRA Institute for Biomedical Technology and Technical Medicine, Science and Technology, University of Twente, PO Box 217, 7500 AE Enschede, Netherlands

Publisher

Future Medicine Ltd. (2nd Albert Place, Finchley Central, London N3 1QB, United Kingdom)

Emtree Heading

adenocarcinoma/di [Diagnosis]; breast cancer/di [Diagnosis]; *cancer staging; computer assisted tomography; diagnostic accuracy; *diagnostic imaging; diagnostic test accuracy study; diffuse reflectance spectroscopy; fluorescence imaging; head and neck squamous cell carcinoma/di [Diagnosis]; histopathology; Hodgkin disease/di [Diagnosis]; human; infrared spectroscopy; intraoperative period; *lymph node; lymph node dissection; lymph node metastasis; lymphatic system; melanoma/di [Diagnosis]; mouth cancer/di [Diagnosis]; nonhodgkin lymphoma/di [Diagnosis]; nonhuman; optical coherence tomography; photoacoustics; positron emission tomography; priority journal; prognosis; Raman spectrometry; review; sensitivity and specificity; sentinel lymph node biopsy; spectroscopy; elastic scattering spectroscopy; near infrared fluorescence.

Candidate Terms

elastic scattering spectroscopy [other term] near infrared fluorescence [other term]

Other Index Terms

adenocarcinoma / diagnosis; breast cancer / diagnosis; *cancer staging; computer assisted tomography; diagnostic accuracy; *diagnostic imaging; diagnostic test accuracy study; diffuse reflectance spectroscopy; fluorescence imaging; head and neck squamous cell carcinoma / diagnosis; histopathology; Hodgkin disease / diagnosis; human; infrared spectroscopy; intraoperative period; *lymph node; lymph node dissection; lymph node metastasis; lymphatic system; melanoma / diagnosis; mouth cancer / diagnosis; nonhodgkin lymphoma / diagnosis; nonhuman; optical coherence tomography; photoacoustics; positron emission tomography; priority journal; prognosis; Raman spectrometry; review; sensitivity and specificity; sentinel lymph node biopsy; spectroscopy.

Link to the Ovid Full Text or citation:

[Click here for full text options](https://ovidsp.ovid.com/ovidweb.cgi?T=JS&CSC=Y&NEWS=N&PAGE=fulltext&D=emed14&AN=370211030)

Link to the External Link Resolver:

[SFX](https://sfx-86scu.hosted.exlibrisgroup.com.cn/86scu?sid=OVID:embase&id=pmid:24156334&id=doi:10.2217%2Ffon.13.125&issn=14796694&isbn=&volume=9&issue=11&spage=1741&pages=1741-1755&date=2013&title=Future+Oncology&atitle=Optical+techniques+for+the+intraoperative+assessment+of+nodal+status&aulast=Grootendorst&pid=<author>Grootendorst+D.J.%3BSteenbergen+W.%3BManohar+S.%3BRuers+T.J.M.<%2Fauthor><AN>370211030<%2FAN><DT>Review<%2FDT>)

289.

Multimodal nonlinear microscopic investigations on head and neck squamous cell carcinoma: Toward intraoperative imaging.

Meyer T., Guntinas-Lichius O., Von Eggeling F., Ernst G., Akimov D., Schmitt M., Dietzek B., Popp J.

Head and Neck. 35(9) (pp E280-E287), 2013. Date of Publication: September 2013.

AN: 369650881

Background Prognosis and appropriate treatment of head and neck squamous cell carcinoma (HNSCC) depend on the tumor type routinely derived by invasive histopathology. A promising noninvasive alternative is nonlinear optical imaging, which is capable of in vivo tissue visualization for tumor typing and grading. Methods and Results Thin tissue sections from 3 patients aged 56 to 60 years presenting advanced carcinoma of the hypopharynx, larynx, and left tonsil were investigated by coherent anti-Stokes Raman scattering (CARS), second-harmonic generation (SHG), and 2 photon excited fluorescence (TPEF) to study the morphochemistry of the tissues. Morphologic alterations of prognostic significance, such as cell density, nuclear to cytoplasm ratio, and keratinization as well as the underlying compositional changes during malignant transformation were determined, such as the distributions of lipids, collagen, and autofluorophors. Conclusions Nonlinear imaging provides a noninvasive optical biopsy of the epithelial layer comparable to staining microscopy. By integration into an operational microscope, routine screening of suspicious lesions and surgical guidance can be realized. © 2012 Wiley Periodicals, Inc.

PMID

22987435 [<https://www.ncbi.nlm.nih.gov/pubmed/?term=22987435>]

Institution

(Meyer, Akimov, Schmitt, Dietzek, Popp) Institute of Photonic Technology, Jena, Germany (Guntinas-Lichius) Department of Otorhinolaryngology, University Hospital, Jena, Germany

(Von Eggeling, Ernst) Institute of Human Genetics, Core Unit Chip Application, University Hospital, Jena, Germany

(Dietzek, Popp) Institute of Physical Chemistry, Abbe Center of Photonics, Friedrich-Schiller-University, Jena, Germany

Publisher

John Wiley and Sons Inc. (P.O.Box 18667, Newark NJ 07191-8667, United States)

Emtree Heading

adult; article; basal cell; basement membrane; biopsy technique; cancer prognosis; case report; cell density; cell nucleus; cell shape; cell size; chemoradiotherapy; comorbidity; connective tissue; cytoplasm; diagnostic imaging; dyskeratosis; fluorescence imaging; *head and neck squamous cell carcinoma/rt [Radiotherapy]; *head and neck squamous cell carcinoma/su [Surgery]; histopathology; human; human tissue; hypopharynx; hypopharynx tumor; *intraoperative period; keratinization; larynx; male; malignant transformation; *microscopy; neck dissection; optical coherence tomography; postoperative care; primary tumor; priority journal; tissue section; tonsil; tracheostomy; collagen; lipid; *multimodal nonlinear microscopy.

Candidate Terms

*multimodal nonlinear microscopy [other term]

Drug Index Terms

collagen; lipid.

Other Index Terms

adult; article; basal cell; basement membrane; biopsy technique; cancer prognosis; case report; cell density; cell nucleus; cell shape; cell size; chemoradiotherapy; comorbidity; connective tissue; cytoplasm; diagnostic imaging; dyskeratosis; fluorescence imaging; *head and neck squamous cell carcinoma / *radiotherapy / *surgery; histopathology; human; human tissue; hypopharynx; hypopharynx tumor; *intraoperative period; keratinization; larynx; male; malignant transformation; *microscopy; neck dissection; optical coherence tomography; postoperative care; primary tumor; priority journal; tissue section; tonsil; tracheostomy.

Link to the Ovid Full Text or citation:

[Click here for full text options](https://ovidsp.ovid.com/ovidweb.cgi?T=JS&CSC=Y&NEWS=N&PAGE=fulltext&D=emed14&AN=369650881)

Link to the External Link Resolver:

[SFX](https://sfx-86scu.hosted.exlibrisgroup.com.cn/86scu?sid=OVID:embase&id=pmid:22987435&id=doi:10.1002%2Fhed.23139&issn=10433074&isbn=&volume=35&issue=9&spage=E280&pages=E280-E287&date=2013&title=Head+and+Neck&atitle=Multimodal+nonlinear+microscopic+investigations+on+head+and+neck+squamous+cell+carcinoma%3A+Toward+intraoperative+imaging&aulast=Meyer&pid=<author>Meyer+T.%3BGuntinas-Lichius+O.%3BVon+Eggeling+F.%3BErnst+G.%3BAkimov+D.%3BSchmitt+M.%3BDietzek+B.%3BPopp+J.<%2Fauthor><AN>369650881<%2FAN><DT>Article<%2FDT>)

290.

Raman microspectroscopy as a diagnostic tool to study single living nasopharyngeal carcinoma cell lines.

Li Z., Chen Y., Li Y., Chen W., Pan J., Su Y., Zou C.

Biochemistry and Cell Biology. 91(3) (pp 182-186), 2013. Date of Publication: June 2013.

AN: 369477782

Raman spectroscopy can provide molecular-level fingerprint information about the biochemical composition and structure of cells and tissues with excellent spatial resolution. In this study, Raman spectroscopy of 3 different nasopharyngeal carcinoma cell lines C666-1, CNE1, and CNE2 and 1 nasopharyngeal normal cell line NP69 acquired on a piece of silica glass slide are presented to investigate the differences among them. The results show the ratio of I 1657/I1449 (= 0.7) could provide good distinction between tumor and normal cell lines very easily, which coincides with existing reports about the study of different cell lines and bronchial tissue. In addition, several statistical analytical methods were used to classify these 4 different cell lines and then achieved an exciting result with great sensitivity and specificity of >90%, respectively. The findings of this work further support former work where cells' Raman spectra were acquired on a different substrate. All of these results indicate Raman spectroscopy has the potential to discriminate between normal and tumor cells and have potential use in early diagnosis of nasopharyngeal carcinoma. © 2013 Published by NRC Research Press.

PMID

23668791 [<https://www.ncbi.nlm.nih.gov/pubmed/?term=23668791>]

Institution

(Li) Fujian Academy of Integrative Medicine, Fujian University of Traditional Chinese Medicine, Fuzhou 350122, Fujian, China (Chen) Zhicheng College, Fuzhou University, Fuzhou 350007, Fujian, China

(Li) Key Laboratory of Optoelectronic Science and Technology for Medicine, Ministry of Education, Fujian Normal University, Fuzhou 350002, Fujian, China

(Chen) College of Integrative Medicine, Fujian University of Traditional Chinese Medicine, Fuzhou 350122, Fujian, China

(Pan, Su, Zou) Fujian Provincial Tumor Hospital, Fuzhou 350014, Fujian, China

Publisher

National Research Council of Canada (Buiding M 55, Ottawa ONT K1A 0R6, Canada)

Emtree Heading

article; cancer cell culture; cancer diagnosis; diagnostic accuracy; early diagnosis; human; human cell; human cell culture; *nasopharynx carcinoma; *Raman spectrometry; sensitivity and specificity.

Other Index Terms

article; cancer cell culture; cancer diagnosis; diagnostic accuracy; early diagnosis; human; human cell; human cell culture; *nasopharynx carcinoma; *Raman spectrometry; sensitivity and specificity.

Link to the Ovid Full Text or citation:

[Click here for full text options](https://ovidsp.ovid.com/ovidweb.cgi?T=JS&CSC=Y&NEWS=N&PAGE=fulltext&D=emed14&AN=369477782)

Link to the External Link Resolver:

[SFX](https://sfx-86scu.hosted.exlibrisgroup.com.cn/86scu?sid=OVID:embase&id=pmid:23668791&id=doi:10.1139%2Fbcb-2012-0024&issn=08298211&isbn=&volume=91&issue=3&spage=182&pages=182-186&date=2013&title=Biochemistry+and+Cell+Biology&atitle=Raman+microspectroscopy+as+a+diagnostic+tool+to+study+single+living+nasopharyngeal+carcinoma+cell+lines&aulast=Li&pid=<author>Li+Z.%3BChen+Y.%3BLi+Y.%3BChen+W.%3BPan+J.%3BSu+Y.%3BZou+C.<%2Fauthor><AN>369477782<%2FAN><DT>Article<%2FDT>)

291.

Quantification of circulating tumor cells using multiplexed Surface-Enhanced Raman Scattering Nanoparticles.

Wang X., Qian X., Beitler J.J., Chen Z., Khuri F.R., Lewis M.M., Shin H.J., Nie S., Shin D.M.

Cancer Research. Conference: 104th Annual Meeting of the American Association for Cancer Research, AACR 2013. Washington, DC United States. Conference Publication: (var.pagings). 73(8 SUPPL. 1) (no pagination), 2013. Date of Publication: 15 Apr 2013.

AN: 71348438

Background: Detection, quantification, and molecular characterization of CTCs may aid physicians in treatment decision making and optimizing. However, the major challenge in the detection and characterization of CTCs is the lack of a highly specific and sensitive assay to isolate and identify the rare population of CTCs in complex blood samples. Moreover, current approaches for CTC detection including CellSearch (FDA approved CTC detection method) which primarily relays on single biomarker are suboptimal because of tumor heterogeneity. Here we develop a multiplexed single-tube assay for directly detection of CTCs in blood sample using two targeted Surface-Enhanced Raman scattering (SERS) nanoparticles.

Method(s): SERS nanoparticles (60 nm) were prepared by spectral encoding with a Raman reporter molecule (QSY or BHQ) and then stabilized and protected by low molecular weight polyethylene glycols (PEGs). A targeting ligand, epidermal growth factor (EGF) peptide or epithelial cell adhesion molecule (EpCAM) antibody, was covalently conjugated to PEG layer of SERS nanoparticle. The functionalized EGF- or EpCAM- SERS nanoparticle was able to identify CTCs in the peripheral blood with high specificity and affinity.

Result(s): We collected and examined fresh blood samples from 20 SCCHN patients with various histologies and successfully detected positive SERS signals that were greater than background in 9 of 12 (75%) of patients. This technique was demonstrated to be capable of identifying rare CTCs by two biomarkers simultaneously, with a range of 5-720 cells per ml of whole blood. Blood samples from 3 normal controls did not show any CTCs. In one patient who had 0 count of CTC detected by EGFR-targeting SERS tag showed 170 CTCs/ml of blood detected by EpCAM-targeting SERS tags.

Conclusion(s): Special design of the two SERS nanoparticles, distinguished with unique spectral signatures and bio-conjugation of EGF or EpCAM antibody as targeting molecule, allowed simultaneous detection of epidermal growth factor receptor (EGFR) or EpCAM on CTCs in one blood sample. Our technique based on two biomarkers rather than those relayed on single biomarker may improve CTC assessment in clinical settings.

Institution

(Wang, Beitler, Chen, Khuri, Lewis, Shin) Emory Univ., Winship Cancer Inst., Atlanta, GA, United States (Qian, Nie) Emory Univ., Atlanta, GA, United States

(Shin) Quest Diagnostics, Atlanta, GA, United States

Publisher

American Association for Cancer Research Inc.

Emtree Heading

*circulating tumor cell; *Raman spectrometry; *cancer research; blood sampling; human; blood; patient; assay; population; physician; conjugation; molecular weight; decision making; tube; neoplasm; histology; *nanoparticle; epithelial cell adhesion molecule; biological marker; antibody; peptide; epidermal growth factor; ligand; macrogol derivative; epidermal growth factor receptor.

Drug Index Terms

*nanoparticle; epithelial cell adhesion molecule; biological marker; antibody; peptide; epidermal growth factor; ligand; macrogol derivative; epidermal growth factor receptor.

Other Index Terms

*circulating tumor cell; *Raman spectrometry; *cancer research; blood sampling; human; blood; patient; assay; population; physician; conjugation; molecular weight; decision making; tube; neoplasm; histology.

Link to the Ovid Full Text or citation:

[Click here for full text options](https://ovidsp.ovid.com/ovidweb.cgi?T=JS&CSC=Y&NEWS=N&PAGE=fulltext&D=emed14&AN=71348438)

Link to the External Link Resolver:

[SFX](https://sfx-86scu.hosted.exlibrisgroup.com.cn/86scu?sid=OVID:embase&id=pmid:&id=doi:10.1158%2F1538-7445.AM2013-1460&issn=00085472&isbn=&volume=73&issue=8+SUPPL.+1&spage=1460&pages=&date=2013&title=Cancer+Research&atitle=Quantification+of+circulating+tumor+cells+using+multiplexed+Surface-Enhanced+Raman+Scattering+Nanoparticles&aulast=Wang&pid=<author>Wang+X.%3BQian+X.%3BBeitler+J.J.%3BChen+Z.%3BKhuri+F.R.%3BLewis+M.M.%3BShin+H.J.%3BNie+S.%3BShin+D.M.<%2Fauthor><AN>71348438<%2FAN><DT>Conference+Abstract<%2FDT>)

292.

Novel nanogold sers based photosensitizers as optical diagnostic probes for oral cancer.

Davies K., Connolly J., Owens P., Dockery P., Olivo M., Keogh I.

Irish Journal of Medical Science. Conference: 54th Annual Meeting of the Irish Otorhinolaryngology/Head and Neck Society. Cong, Co. Mayo Ireland. Conference Publication: (var.pagings). 182(12 SUPPL. 1) (pp S515), 2013. Date of Publication: December 2013.

AN: 71283191

Objectives: Despite significant advances in treatment modalities, 5 year survival in oral squamous cell carcinoma is less than 50 %. Clinical examination, white light endoscopy and histopathological analysis remain the gold standard for diagnostic surveillance of oral cancer with a limited diagnostic accuracy of 55 %. Novel optical based diagnostic methods are promising new technologies for improving both screening and detection of cancer.

Method(s): We report the use of novel nanogold SERS (Surface Enhanced Raman Spectroscopy) based nano(photo) sensitisers as optical diagnostic probes using an in vitro oral cancer cell model. Such multi-modal nanosensitisers were constructed by layering fluorescent photosensitisers (Hypericin, Chlorin e6) and a Raman reporter (DTTCI; 3,3-diethyl-thiatricarbocyanine) onto multi-branched gold nanoparticles. Nanosensitisers were also conjugated to tumour-specific antibodies to confer cancer cell specificity. A number of cell lines were cultured in vitro with various concentrations of nanosensitisers. Cellular uptake and intracellular localization of nanosensitisers was assessed using confocal fluorescence microscopy and transmission electron microscopy. To demonstrate label free detection of nanosensitiser uptake, SERS spectra and large area scans were also examined. Data was collected using a WITEC Alpha 500 system using 785 nm excitation.

Result(s): It was observed that nanosensitisers were taken up by tumour cells and internalized, they were found to localize primarily to lyso-somes. Raman images were collected, enabling label free visualization of nanosensitiser uptake in cells.

Conclusion(s): While preliminary, results from this study suggest bio-compatibility of these novel gold nanosensitisers in vitro and highlight it's potential as multifunctional optical agents for cancer diagnosis.

Publisher

Springer London

Emtree Heading

*optics; *diagnosis; *mouth cancer; *neck; *society; in vitro study; cancer cell; neoplasm; mouth squamous cell carcinoma; screening; technology; diagnostic procedure; diagnostic accuracy; cell line; excitation; Raman spectrometry; gold standard; survival; white light endoscopy; clinical examination; cell specificity; fluorescence microscopy; transmission electron microscopy; tumor cell; cancer diagnosis; *photosensitizing agent; gold; hypericin; gold nanoparticle; antibody.

Drug Index Terms

*photosensitizing agent; gold; hypericin; gold nanoparticle; antibody.

Other Index Terms

*optics; *diagnosis; *mouth cancer; *neck; *society; in vitro study; cancer cell; neoplasm; mouth squamous cell carcinoma; screening; technology; diagnostic procedure; diagnostic accuracy; cell line; excitation; Raman spectrometry; gold standard; survival; white light endoscopy; clinical examination; cell specificity; fluorescence microscopy; transmission electron microscopy; tumor cell; cancer diagnosis.

Link to the Ovid Full Text or citation:

[Click here for full text options](https://ovidsp.ovid.com/ovidweb.cgi?T=JS&CSC=Y&NEWS=N&PAGE=fulltext&D=emed14&AN=71283191)

Link to the External Link Resolver:

[SFX](https://sfx-86scu.hosted.exlibrisgroup.com.cn/86scu?sid=OVID:embase&id=pmid:&id=doi:10.1007%2Fs11845-013-1048-1&issn=00211265&isbn=&volume=182&issue=12+SUPPL.+1&spage=S515&pages=S515&date=2013&title=Irish+Journal+of+Medical+Science&atitle=Novel+nanogold+sers+based+photosensitizers+as+optical+diagnostic+probes+for+oral+cancer&aulast=Davies&pid=<author>Davies+K.%3BConnolly+J.%3BOwens+P.%3BDockery+P.%3BOlivo+M.%3BKeogh+I.<%2Fauthor><AN>71283191<%2FAN><DT>Conference+Abstract<%2FDT>)

293.

A minimally-invasive approach to the challenge of oral neoplasia.

Mittal R., Balu M., Liu G., Chen Z., Tromberg B., Wilder-Smith P., Potma E.

Lasers in Surgery and Medicine. Conference: 33rd Annual Conference of the American Society for Laser Medicine and Surgery, ASLMS 2013. Boston, MA United States. Conference Publication: (var.pagings). 45(SUPPL. 25) (pp 39-40), 2013. Date of Publication: March 2013.

AN: 71034048

Background: Despite significant advancements in the treatment of oral cancer, it still results in 10,000 U.S. deaths annually. Squamous cell carcinomas (SCC) account for 96% of oral cancers and are usually preceded by dysplasia. Current techniques require surgical biopsy of oral lesions, which are often benign and detect malignant changes too late for optimal treatment. A noninvasive capability for early detection of oral dysplasia is required for patient survival. In this study we aim to develop a fiber coupled miniature probe suitable for examination of oral cavity. The probe includes coherent Raman scattering (CRS) along with two-photon excited florescence (TPEF) imaging capabilities. Study: The probe design includes optimized objective lens, efficient light delivery and collection through optical fiber, real time fiber scanning capabilities and home build photodetector. Each of these parts is individually optimized for improved performance. For preliminary experiments we imaged SCC excised tissue.

Result(s): We have designed and constructed a miniature lens assembly with achromatic performance, numerical aperture of 0.5 and lateral resolution of 1 mum. We incorporated a fiber tip scanner, including a piezo-electric actuator and fiber cantilever. The fiber tip spiral scans at the rate of 20 Hz, resulting in faster imaging of field of view of 100 x 100 mum2. All components are enclosed in a compact probe of 10.5mm diameter and length of 38mm, suitable for imaging of oral cavity. A SCC sample imaged with TPEF/CRS clearly separates the nuclei from the cytosolic cell content and shows similarities to hemotoxylin/eosin (H&E) stained images in the epidermal layers.

Conclusion(s): Advances made in this work enables discrimination of the cell cytoplasm and nucleus similar to conventional H&E staining contrast, using CRS imaging. The improved imaging resolution of TPEF/CRS probe makes it possible to visualize cell nuclei and cell boundaries, without the need of histopathology of biopsy, crucial for diagnostic of oral cancer.

Institution

(Mittal, Balu, Liu, Chen, Tromberg, Wilder-Smith, Potma) Beckman Laser Institute and Medical Clinic, University of California, Irvine, CA, United States

Publisher

Wiley-Liss Inc.

Emtree Heading

*society; *laser; *surgery; *neoplasm; fiber; imaging; mouth cancer; mouth cavity; dysplasia; biopsy; lens; squamous cell carcinoma; photon; human; examination; survival; death; diagnosis; patient; mouth lesion; staining; tissues; cytoplasm; cell nucleus; histopathology; United States; glass fiber.

Drug Index Terms

glass fiber.

Other Index Terms

*society; *laser; *surgery; *neoplasm; fiber; imaging; mouth cancer; mouth cavity; dysplasia; biopsy; lens; squamous cell carcinoma; photon; human; examination; survival; death; diagnosis; patient; mouth lesion; staining; tissues; cytoplasm; cell nucleus; histopathology; United States.

Link to the Ovid Full Text or citation:

[Click here for full text options](https://ovidsp.ovid.com/ovidweb.cgi?T=JS&CSC=Y&NEWS=N&PAGE=fulltext&D=emed14&AN=71034048)

Link to the External Link Resolver:

[SFX](https://sfx-86scu.hosted.exlibrisgroup.com.cn/86scu?sid=OVID:embase&id=pmid:&id=doi:10.1002%2Flsm.22127&issn=01968092&isbn=&volume=45&issue=SUPPL.+25&spage=39&pages=39-40&date=2013&title=Lasers+in+Surgery+and+Medicine&atitle=A+minimally-invasive+approach+to+the+challenge+of+oral+neoplasia&aulast=Mittal&pid=<author>Mittal+R.%3BBalu+M.%3BLiu+G.%3BChen+Z.%3BTromberg+B.%3BWilder-Smith+P.%3BPotma+E.<%2Fauthor><AN>71034048<%2FAN><DT>Conference+Abstract<%2FDT>)

294.

Discrimination between benign, primary and secondary malignancies in lymph nodes from the head and neck utilising Raman spectroscopy and multivariate analysis.

Lloyd G.R., Orr L.E., Christie-Brown J., McCarthy K., Rose S., Thomas M., Stone N.

The Analyst. 138(14) (pp 3900-3908), 2013. Date of Publication: 21 Jul 2013.

AN: 602409358

BACKGROUND: The potential use of Raman spectroscopy (RS) for the detection of malignancy within lymph nodes of the head and neck was evaluated. RS measures the presence of biomolecules by the inelastic scattering of light within cells and tissues. This can be performed in vivo in real-time. METHODS: 103 lymph nodes were collected from 23 patients undergoing surgery for suspicious lymph nodes. Five pathologies, defined by consensus histopathology, were collected including reactive nodes (benign), Hodgkin's and non-Hodgkin's lymphomas, metastases from both squamous cell carcinomas and adenocarcinomas. Raman spectra were measured with 830 nm excitation from numerous positions on each biopsy. Spectral diagnostic models were constructed using principal component analysis followed by linear discriminant analysis (PCA-LDA), and by partial least squares discriminant analysis (PLS-DA) for comparison. Two-group models were constructed to distinguish between reactive and malignant nodes, and three-group models to distinguish between the benign, primary and secondary conditions.

RESULTS: Results were validated using a repeated subsampling procedure. Sensitivities and specificities of 90% and 86% were obtained using PCA-LDA, and 89% and 88% using PLS-DA, for the two-group models. Both PCA-LDA and PLS-DA models were also found to be very successful at discriminating between pathologies in the three-group models achieving sensitivities and specificities of over 78% and 89% for PCA-LDA, and over 81% and 89% for PLS-DA for all three pathology groups.

CONCLUSION: Raman spectroscopy and chemometric techniques can be successfully utilised in combination for discriminating between different cancerous conditions of lymph nodes from the head and neck.

PMID

23295372 [<https://www.ncbi.nlm.nih.gov/pubmed/?term=23295372>]

Institution

(Lloyd, Orr, Christie-Brown, McCarthy, Rose, Thomas, Stone) Biophotonics Research Group, Gloucestershire Royal Hospital, Great Western Road, Gloucester, GL1 3NN, UK

Emtree Heading

adenocarcinoma; comparative study; discriminant analysis; head and neck tumor; Hodgkin disease; human; lymph node metastasis; multivariate analysis; nonhodgkin lymphoma; *pathology; principal component analysis; *Raman spectrometry; regression analysis; second cancer; sensitivity and specificity; squamous cell carcinoma; *secondary.

Candidate Terms

*secondary [other term]

Other Index Terms

adenocarcinoma; comparative study; discriminant analysis; head and neck tumor; Hodgkin disease; human; lymph node metastasis; multivariate analysis; nonhodgkin lymphoma; *pathology; principal component analysis; *Raman spectrometry; regression analysis; second cancer; sensitivity and specificity; squamous cell carcinoma.

Link to the Ovid Full Text or citation:

[Click here for full text options](https://ovidsp.ovid.com/ovidweb.cgi?T=JS&CSC=Y&NEWS=N&PAGE=fulltext&D=emed14&AN=602409358)

Link to the External Link Resolver:

[SFX](https://sfx-86scu.hosted.exlibrisgroup.com.cn/86scu?sid=OVID:embase&id=pmid:23295372&id=doi:10.1039%2Fc2an36579k&issn=13645528&isbn=&volume=138&issue=14&spage=3900&pages=3900-3908&date=2013&title=The+Analyst&atitle=Discrimination+between+benign%2C+primary+and+secondary+malignancies+in+lymph+nodes+from+the+head+and+neck+utilising+Raman+spectroscopy+and+multivariate+analysis&aulast=Lloyd&pid=<author>Lloyd+G.R.%3BOrr+L.E.%3BChristie-Brown+J.%3BMcCarthy+K.%3BRose+S.%3BThomas+M.%3BStone+N.<%2Fauthor><AN>602409358<%2FAN><DT>Article<%2FDT>)

295.

Raman spectroscopy of serum: an exploratory study for detection of oral cancers.

Sahu A., Sawant S., Mamgain H., Krishna C.M.

The Analyst. 138(14) (pp 4161-4174), 2013. Date of Publication: 21 Jul 2013.

AN: 602409154

Early diagnosis of oral cancers, one of the major cancers, is of utmost importance as 5-year disease-free survival rates are some of the lowest, despite advances in treatment and surgical modalities. In vivo Raman spectroscopy has shown efficacy in the detection of normal, premalignant and malignant lesions and even of early changes such as cancer-field-effects/malignancy-associated-changes. However, the need for a dedicated instrument and stringent laboratory conditions, at all diagnostic centers, limits wide screening applications of this method. In light of this, it is pertinent to explore ex vivo samples like serum due to its ease of collection, storage, transport and analysis at a centralized facility. Hence, Raman studies were carried out on serum from 14 buccal mucosa and 40 tongue cancers as well as 16 healthy control samples. Spectral features indicate differential contributions of proteins, DNA, and amino acids like Phe, Trp and Tyr and beta-carotene in the analyzed groups. Highly intense Raman bands assigned to beta-carotene could be due to resonance Raman, and were observed in all sera with the highest relative intensity in normal samples. Higher DNA and protein content were observed in the mean cancer spectra. Principal component-linear discriminant analysis (PC-LDA) followed by cross-validation using leave-one-out cross-validation (LOOCV) were employed for data analysis which was carried out both spectra- and patient-wise. Findings indicate the possibility of classifying normal and oral cancer sera in both these approaches; however, the patient-wise approach could be the preferred mode for prospective studies. Besides, a tendency of classification for buccal mucosa and tongue cancers was also observed. Prospective validation of these results on a large sample size may help in the translation of this methodology to clinics.

PMID

23736856 [<https://www.ncbi.nlm.nih.gov/pubmed/?term=23736856>]

Institution

(Sahu, Sawant, Mamgain, Krishna) Chilakapati lab, ACTREC, Tata Memorial Center, Kharghar, Navi Mumbai-410210, India

Emtree Heading

adult; algorithm; blood; case control study; comparative study; controlled study; discriminant analysis; female; human; male; middle aged; mouth mucosa; *pathology; principal component analysis; *procedures; Raman spectrometry; randomized controlled trial; Carcinoma, Squamous Cell/di [Diagnosis]; Mouth Neoplasms/di [Diagnosis]; Precancerous Conditions/di [Diagnosis].

Candidate Terms

Carcinoma, Squamous Cell / diagnosis [other term] Mouth Neoplasms / diagnosis [other term]

Precancerous Conditions / diagnosis [other term]

Other Index Terms

adult; algorithm; blood; case control study; comparative study; controlled study; discriminant analysis; female; human; male; middle aged; mouth mucosa; *pathology; principal component analysis; *procedures; Raman spectrometry; randomized controlled trial.

Link to the Ovid Full Text or citation:

[Click here for full text options](https://ovidsp.ovid.com/ovidweb.cgi?T=JS&CSC=Y&NEWS=N&PAGE=fulltext&D=emed14&AN=602409154)

Link to the External Link Resolver:

[SFX](https://sfx-86scu.hosted.exlibrisgroup.com.cn/86scu?sid=OVID:embase&id=pmid:23736856&id=doi:10.1039%2Fc3an00308f&issn=13645528&isbn=&volume=138&issue=14&spage=4161&pages=4161-4174&date=2013&title=The+Analyst&atitle=Raman+spectroscopy+of+serum%3A+an+exploratory+study+for+detection+of+oral+cancers&aulast=Sahu&pid=<author>Sahu+A.%3BSawant+S.%3BMamgain+H.%3BKrishna+C.M.<%2Fauthor><AN>602409154<%2FAN><DT>Article<%2FDT>)

296.

Discrimination between benign, primary and secondary malignancies in lymph nodes from the head and neck utilising Raman spectroscopy and multivariate analysis.

Lloyd G.R., Orr L.E., Christie-Brown J., McCarthy K., Rose S., Thomas M., Stone N.

The Analyst. 138(14) (pp 3900-3908), 2013. Date of Publication: 21 Jul 2013.

AN: 563014916

The potential use of Raman spectroscopy (RS) for the detection of malignancy within lymph nodes of the head and neck was evaluated. RS measures the presence of biomolecules by the inelastic scattering of light within cells and tissues. This can be performed in vivo in real-time. 103 lymph nodes were collected from 23 patients undergoing surgery for suspicious lymph nodes. Five pathologies, defined by consensus histopathology, were collected including reactive nodes (benign), Hodgkin's and non-Hodgkin's lymphomas, metastases from both squamous cell carcinomas and adenocarcinomas. Raman spectra were measured with 830 nm excitation from numerous positions on each biopsy. Spectral diagnostic models were constructed using principal component analysis followed by linear discriminant analysis (PCA-LDA), and by partial least squares discriminant analysis (PLS-DA) for comparison. Two-group models were constructed to distinguish between reactive and malignant nodes, and three-group models to distinguish between the benign, primary and secondary conditions. Results were validated using a repeated subsampling procedure. Sensitivities and specificities of 90% and 86% were obtained using PCA-LDA, and 89% and 88% using PLS-DA, for the two-group models. Both PCA-LDA and PLS-DA models were also found to be very successful at discriminating between pathologies in the three-group models achieving sensitivities and specificities of over 78% and 89% for PCA-LDA, and over 81% and 89% for PLS-DA for all three pathology groups. Raman spectroscopy and chemometric techniques can be successfully utilised in combination for discriminating between different cancerous conditions of lymph nodes from the head and neck.

PMID

23295372 [<https://www.ncbi.nlm.nih.gov/pubmed/?term=23295372>]

Institution

(Lloyd) Biophotonics Research Group, Gloucestershire Royal Hospital, Great Western Road, Gloucester, GL1 3NN, UK.

Emtree Heading

*adenocarcinoma; article; comparative study; discriminant analysis; *head and neck tumor; *Hodgkin disease; human; lymph node metastasis; metastasis; multivariate analysis; *nonhodgkin lymphoma; pathology; principal component analysis; *Raman spectrometry; regression analysis; *second cancer; sensitivity and specificity; *squamous cell carcinoma.

Other Index Terms

*adenocarcinoma; article; comparative study; discriminant analysis; *head and neck tumor; *Hodgkin disease; human; lymph node metastasis; metastasis; multivariate analysis; *nonhodgkin lymphoma; pathology; principal component analysis; *Raman spectrometry; regression analysis; *second cancer; sensitivity and specificity; *squamous cell carcinoma.

Link to the Ovid Full Text or citation:

[Click here for full text options](https://ovidsp.ovid.com/ovidweb.cgi?T=JS&CSC=Y&NEWS=N&PAGE=fulltext&D=emed14&AN=563014916)

Link to the External Link Resolver:

[SFX](https://sfx-86scu.hosted.exlibrisgroup.com.cn/86scu?sid=OVID:embase&id=pmid:23295372&id=doi:&issn=13645528&isbn=&volume=138&issue=14&spage=3900&pages=3900-3908&date=2013&title=The+Analyst&atitle=Discrimination+between+benign%2C+primary+and+secondary+malignancies+in+lymph+nodes+from+the+head+and+neck+utilising+Raman+spectroscopy+and+multivariate+analysis&aulast=Lloyd&pid=<author>Lloyd+G.R.%3BOrr+L.E.%3BChristie-Brown+J.%3BMcCarthy+K.%3BRose+S.%3BThomas+M.%3BStone+N.<%2Fauthor><AN>563014916<%2FAN><DT>Article<%2FDT>)

297.

Raman spectroscopy of serum: an exploratory study for detection of oral cancers.

Sahu A., Sawant S., Mamgain H., Krishna C.M.

The Analyst. 138(14) (pp 4161-4174), 2013. Date of Publication: 21 Jul 2013.

AN: 563014909

Early diagnosis of oral cancers, one of the major cancers, is of utmost importance as 5-year disease-free survival rates are some of the lowest, despite advances in treatment and surgical modalities. In vivo Raman spectroscopy has shown efficacy in the detection of normal, premalignant and malignant lesions and even of early changes such as cancer-field-effects/malignancy-associated-changes. However, the need for a dedicated instrument and stringent laboratory conditions, at all diagnostic centers, limits wide screening applications of this method. In light of this, it is pertinent to explore ex vivo samples like serum due to its ease of collection, storage, transport and analysis at a centralized facility. Hence, Raman studies were carried out on serum from 14 buccal mucosa and 40 tongue cancers as well as 16 healthy control samples. Spectral features indicate differential contributions of proteins, DNA, and amino acids like Phe, Trp and Tyr and beta-carotene in the analyzed groups. Highly intense Raman bands assigned to beta-carotene could be due to resonance Raman, and were observed in all sera with the highest relative intensity in normal samples. Higher DNA and protein content were observed in the mean cancer spectra. Principal component-linear discriminant analysis (PC-LDA) followed by cross-validation using leave-one-out cross-validation (LOOCV) were employed for data analysis which was carried out both spectra- and patient-wise. Findings indicate the possibility of classifying normal and oral cancer sera in both these approaches; however, the patient-wise approach could be the preferred mode for prospective studies. Besides, a tendency of classification for buccal mucosa and tongue cancers was also observed. Prospective validation of these results on a large sample size may help in the translation of this methodology to clinics.

PMID

23736856 [<https://www.ncbi.nlm.nih.gov/pubmed/?term=23736856>]

Institution

(Sahu) Chilakapati lab, ACTREC, Tata Memorial Center, Kharghar, Navi Mumbai-410210, India.

Emtree Heading

adult; algorithm; article; blood; case control study; comparative study; controlled clinical trial; controlled study; discriminant analysis; female; human; male; methodology; middle aged; *mouth mucosa; *mouth tumor/di [Diagnosis]; pathology; *precancer/di [Diagnosis]; principal component analysis; *Raman spectrometry; randomized controlled trial; *squamous cell carcinoma/di [Diagnosis].

Other Index Terms

adult; algorithm; article; blood; case control study; comparative study; controlled clinical trial; controlled study; discriminant analysis; female; human; male; methodology; middle aged; *mouth mucosa; *mouth tumor / *diagnosis; pathology; *precancer / *diagnosis; principal component analysis; *Raman spectrometry; randomized controlled trial; *squamous cell carcinoma / *diagnosis.

Link to the Ovid Full Text or citation:

[Click here for full text options](https://ovidsp.ovid.com/ovidweb.cgi?T=JS&CSC=Y&NEWS=N&PAGE=fulltext&D=emed14&AN=563014909)

Link to the External Link Resolver:

[SFX](https://sfx-86scu.hosted.exlibrisgroup.com.cn/86scu?sid=OVID:embase&id=pmid:23736856&id=doi:&issn=13645528&isbn=&volume=138&issue=14&spage=4161&pages=4161-4174&date=2013&title=The+Analyst&atitle=Raman+spectroscopy+of+serum%3A+an+exploratory+study+for+detection+of+oral+cancers&aulast=Sahu&pid=<author>Sahu+A.%3BSawant+S.%3BMamgain+H.%3BKrishna+C.M.<%2Fauthor><AN>563014909<%2FAN><DT>Article<%2FDT>)

298.

A compact microscope setup for multimodal nonlinear imaging in clinics and its application to disease diagnostics.

Meyer T., Baumgartl M., Gottschall T., Pascher T., Wuttig A., Matthaus C., Romeike B.F., Brehm B.R., Limpert J., Tunnermann A., Guntinas-Lichius O., Dietzek B., Schmitt M., Popp J.

The Analyst. 138(14) (pp 4048-4057), 2013. Date of Publication: 21 Jul 2013.

AN: 563014900

The past years have seen increasing interest in nonlinear optical microscopic imaging approaches for the investigation of diseases due to the method's unique capabilities of deep tissue penetration, 3D sectioning and molecular contrast. Its application in clinical routine diagnostics, however, is hampered by large and costly equipment requiring trained staff and regular maintenance, hence it has not yet matured to a reliable tool for application in clinics. In this contribution implementing a novel compact fiber laser system into a tailored designed laser scanning microscope results in a small footprint easy to use multimodal imaging platform enabling simultaneously highly efficient generation and acquisition of second harmonic generation (SHG), two-photon excited fluorescence (TPEF) as well as coherent anti-Stokes Raman scattering (CARS) signals with optimized CARS contrast for lipid imaging for label-free investigation of tissue samples. The instrument combining a laser source and a microscope features a unique combination of the highest NIR transmission and a fourfold enlarged field of view suited for investigating large tissue specimens. Despite its small size and turnkey operation rendering daily alignment dispensable the system provides the highest flexibility, an imaging speed of 1 megapixel per second and diffraction limited spatial resolution. This is illustrated by imaging samples of squamous cell carcinoma of the head and neck (HNSCC) and an animal model of atherosclerosis allowing for a complete characterization of the tissue composition and morphology, i.e. the tissue's morphochemistry. Highly valuable information for clinical diagnostics, e.g. monitoring the disease progression at the cellular level with molecular specificity, can be retrieved. Future combination with microscopic probes for in vivo imaging or even implementation in endoscopes will allow for in vivo grading of HNSCC and characterization of plaque deposits towards the detection of high risk plaques.

PMID

23632421 [<https://www.ncbi.nlm.nih.gov/pubmed/?term=23632421>]

Institution

(Meyer) Institute of Photonic Technology Jena IPHT, Albert-Einstein-Strasse 9, 07745 Jena, Germany.

Emtree Heading

animal; article; *atherosclerosis/di [Diagnosis]; *atherosclerosis/et [Etiology]; *confocal microscopy; disease course; disease model; *head and neck tumor/di [Diagnosis]; human; image processing; male; methodology; photon; rabbit; *Raman spectrometry; *squamous cell carcinoma/di [Diagnosis]; lipid/an [Drug Analysis].

Drug Index Terms

lipid / drug analysis.

Other Index Terms

animal; article; *atherosclerosis / *diagnosis / *etiology; *confocal microscopy; disease course; disease model; *head and neck tumor / *diagnosis; human; image processing; male; methodology; photon; rabbit; *Raman spectrometry; *squamous cell carcinoma / *diagnosis.

Link to the Ovid Full Text or citation:

[Click here for full text options](https://ovidsp.ovid.com/ovidweb.cgi?T=JS&CSC=Y&NEWS=N&PAGE=fulltext&D=emed14&AN=563014900)

Link to the External Link Resolver:

[SFX](https://sfx-86scu.hosted.exlibrisgroup.com.cn/86scu?sid=OVID:embase&id=pmid:23632421&id=doi:&issn=13645528&isbn=&volume=138&issue=14&spage=4048&pages=4048-4057&date=2013&title=The+Analyst&atitle=A+compact+microscope+setup+for+multimodal+nonlinear+imaging+in+clinics+and+its+application+to+disease+diagnostics&aulast=Meyer&pid=<author>Meyer+T.%3BBaumgartl+M.%3BGottschall+T.%3BPascher+T.%3BWuttig+A.%3BMatthaus+C.%3BRomeike+B.F.%3BBrehm+B.R.%3BLimpert+J.%3BTunnermann+A.%3BGuntinas-Lichius+O.%3BDietzek+B.%3BSchmitt+M.%3BPopp+J.<%2Fauthor><AN>563014900<%2FAN><DT>Article<%2FDT>)

299.

In vivo Raman spectroscopy of oral buccal mucosa: a study on malignancy associated changes (MAC)/cancer field effects (CFE).

Singh S.P., Sahu A., Deshmukh A., Chaturvedi P., Krishna C.M.

The Analyst. 138(14) (pp 4175-4182), 2013. Date of Publication: 21 Jul 2013.

AN: 563014895

Occurrence of metachronous and synchronous secondary tumors in oral cavities has been associated with poor prognosis and decreased 5-year disease-free survival rates. The origin of secondary tumors in the oral cavity has been primarily attributed to cancer field effects (CFE) or malignancy-associated changes (MAC) in uninvolved areas. Classification of normal, cancerous and pre-cancerous oral lesions by in vivo Raman spectroscopy (RS) has already been demonstrated. In the present study, MAC/CFE in oral buccal mucosa were explored. In vivo Raman spectra from 84 subjects (722 spectra) under five categories - cancer and contralateral normal (opposite side of tumor), healthy controls (no tobacco habit, no cancer), habitues healthy controls (tobacco habit, no cancer) and non-habitues contralateral normal (no tobacco habit with cancer) were acquired. Mean and difference spectra suggest that loss of lipids and additional features representing proteins and DNA are characteristics of all pathological conditions, with respect to healthy controls. Spectral data were analyzed by PC-LDA followed by leave-one-out cross-validation. Results suggest that Raman characteristics of mucosa of healthy controls are exclusive, while those of habitues healthy controls are similar to those of contralateral normal mucosa. It was observed that the cluster of non-habitues contralateral normal mucosa is different from habitues healthy controls, suggesting that malignancy associated changes can be identified and also indicating that transformation of uninvolved oral mucosa due to tobacco habit or malignancy is different. The findings of the study demonstrate the potential of RS in identifying early transformation changes in oral mucosa and the efficacy of this approach in oral cancer applications.

PMID

23392131 [<https://www.ncbi.nlm.nih.gov/pubmed/?term=23392131>]

Institution

(Singh) Chilakapati lab, ACTREC, Tata Memorial Center, Kharghar, Navi Mumbai 410210, India.

Emtree Heading

adult; aged; algorithm; article; blood; case control study; chemically induced disorder; comparative study; discriminant analysis; feasibility study; female; human; male; methodology; middle aged; *mouth mucosa; *mouth tumor/di [Diagnosis]; pathology; principal component analysis; *Raman spectrometry; spectrofluorometry; *squamous cell carcinoma/di [Diagnosis]; *tobacco/ae [Adverse Drug Reaction]; young adult.

Other Index Terms

adult; aged; algorithm; article; blood; case control study; chemically induced disorder; comparative study; discriminant analysis; feasibility study; female; human; male; methodology; middle aged; *mouth mucosa; *mouth tumor / *diagnosis; pathology; principal component analysis; *Raman spectrometry; spectrofluorometry; *squamous cell carcinoma / *diagnosis; *tobacco / *adverse drug reaction; young adult.

Link to the Ovid Full Text or citation:

[Click here for full text options](https://ovidsp.ovid.com/ovidweb.cgi?T=JS&CSC=Y&NEWS=N&PAGE=fulltext&D=emed14&AN=563014895)

Link to the External Link Resolver:

[SFX](https://sfx-86scu.hosted.exlibrisgroup.com.cn/86scu?sid=OVID:embase&id=pmid:23392131&id=doi:&issn=13645528&isbn=&volume=138&issue=14&spage=4175&pages=4175-4182&date=2013&title=The+Analyst&atitle=In+vivo+Raman+spectroscopy+of+oral+buccal+mucosa%3A+a+study+on+malignancy+associated+changes+(MAC)%2Fcancer+field+effects+(CFE)&aulast=Singh&pid=<author>Singh+S.P.%3BSahu+A.%3BDeshmukh+A.%3BChaturvedi+P.%3BKrishna+C.M.<%2Fauthor><AN>563014895<%2FAN><DT>Article<%2FDT>)

300.

A compact microscope setup for multimodal nonlinear imaging in clinics and its application to disease diagnostics.

Meyer T., Baumgartl M., Gottschall T., Pascher T., Wuttig A., Matthaus C., Romeike B.F., Brehm B.R., Limpert J., Tunnermann A., Guntinas-Lichius O., Dietzek B., Schmitt M., Popp J.

The Analyst. 138(14) (pp 4048-4057), 2013. Date of Publication: 21 Jul 2013.

AN: 603380241

The past years have seen increasing interest in nonlinear optical microscopic imaging approaches for the investigation of diseases due to the method's unique capabilities of deep tissue penetration, 3D sectioning and molecular contrast. Its application in clinical routine diagnostics, however, is hampered by large and costly equipment requiring trained staff and regular maintenance, hence it has not yet matured to a reliable tool for application in clinics. In this contribution implementing a novel compact fiber laser system into a tailored designed laser scanning microscope results in a small footprint easy to use multimodal imaging platform enabling simultaneously highly efficient generation and acquisition of second harmonic generation (SHG), two-photon excited fluorescence (TPEF) as well as coherent anti-Stokes Raman scattering (CARS) signals with optimized CARS contrast for lipid imaging for label-free investigation of tissue samples. The instrument combining a laser source and a microscope features a unique combination of the highest NIR transmission and a fourfold enlarged field of view suited for investigating large tissue specimens. Despite its small size and turnkey operation rendering daily alignment dispensable the system provides the highest flexibility, an imaging speed of 1 megapixel per second and diffraction limited spatial resolution. This is illustrated by imaging samples of squamous cell carcinoma of the head and neck (HNSCC) and an animal model of atherosclerosis allowing for a complete characterization of the tissue composition and morphology, i.e. the tissue's morphochemistry. Highly valuable information for clinical diagnostics, e.g. monitoring the disease progression at the cellular level with molecular specificity, can be retrieved. Future combination with microscopic probes for in vivo imaging or even implementation in endoscopes will allow for in vivo grading of HNSCC and characterization of plaque deposits towards the detection of high risk plaques.

PMID

23632421 [<https://www.ncbi.nlm.nih.gov/pubmed/?term=23632421>]

Institution

(Meyer, Baumgartl, Gottschall, Pascher, Wuttig, Matthaus, Romeike, Brehm, Limpert, Tunnermann, Guntinas-Lichius, Dietzek, Schmitt, Popp) Institute of Photonic Technology Jena IPHT, Albert-Einstein-Strasse 9, 07745 Jena, Germany

Emtree Heading

animal; atherosclerosis/di [Diagnosis]; atherosclerosis/et [Etiology]; *confocal microscopy; disease course; disease model; human; image processing; male; photon; *procedures; rabbit; Raman spectrometry; lipid/an [Drug Analysis]; Carcinoma, Squamous Cell/di [Diagnosis]; Head and Neck Neoplasms/di [Diagnosis].

Candidate Terms

Carcinoma, Squamous Cell / diagnosis [other term] Head and Neck Neoplasms / diagnosis [other term]

Drug Index Terms

lipid / drug analysis.

Other Index Terms

animal; atherosclerosis / diagnosis / etiology; *confocal microscopy; disease course; disease model; human; image processing; male; photon; *procedures; rabbit; Raman spectrometry.

Link to the Ovid Full Text or citation:

[Click here for full text options](https://ovidsp.ovid.com/ovidweb.cgi?T=JS&CSC=Y&NEWS=N&PAGE=fulltext&D=emed14&AN=603380241)

Link to the External Link Resolver:

[SFX](https://sfx-86scu.hosted.exlibrisgroup.com.cn/86scu?sid=OVID:embase&id=pmid:23632421&id=doi:10.1039%2Fc3an00354j&issn=13645528&isbn=&volume=138&issue=14&spage=4048&pages=4048-4057&date=2013&title=The+Analyst&atitle=A+compact+microscope+setup+for+multimodal+nonlinear+imaging+in+clinics+and+its+application+to+disease+diagnostics&aulast=Meyer&pid=<author>Meyer+T.%3BBaumgartl+M.%3BGottschall+T.%3BPascher+T.%3BWuttig+A.%3BMatthaus+C.%3BRomeike+B.F.%3BBrehm+B.R.%3BLimpert+J.%3BTunnermann+A.%3BGuntinas-Lichius+O.%3BDietzek+B.%3BSchmitt+M.%3BPopp+J.<%2Fauthor><AN>603380241<%2FAN><DT>Article<%2FDT>)

301.

In vivo Raman spectroscopy of oral buccal mucosa: a study on malignancy associated changes (MAC)/cancer field effects (CFE).

Singh S.P., Sahu A., Deshmukh A., Chaturvedi P., Krishna C.M.

The Analyst. 138(14) (pp 4175-4182), 2013. Date of Publication: 21 Jul 2013.

AN: 603380096

Occurrence of metachronous and synchronous secondary tumors in oral cavities has been associated with poor prognosis and decreased 5-year disease-free survival rates. The origin of secondary tumors in the oral cavity has been primarily attributed to cancer field effects (CFE) or malignancy-associated changes (MAC) in uninvolved areas. Classification of normal, cancerous and pre-cancerous oral lesions by in vivo Raman spectroscopy (RS) has already been demonstrated. In the present study, MAC/CFE in oral buccal mucosa were explored. In vivo Raman spectra from 84 subjects (722 spectra) under five categories - cancer and contralateral normal (opposite side of tumor), healthy controls (no tobacco habit, no cancer), habitues healthy controls (tobacco habit, no cancer) and non-habitues contralateral normal (no tobacco habit with cancer) were acquired. Mean and difference spectra suggest that loss of lipids and additional features representing proteins and DNA are characteristics of all pathological conditions, with respect to healthy controls. Spectral data were analyzed by PC-LDA followed by leave-one-out cross-validation. Results suggest that Raman characteristics of mucosa of healthy controls are exclusive, while those of habitues healthy controls are similar to those of contralateral normal mucosa. It was observed that the cluster of non-habitues contralateral normal mucosa is different from habitues healthy controls, suggesting that malignancy associated changes can be identified and also indicating that transformation of uninvolved oral mucosa due to tobacco habit or malignancy is different. The findings of the study demonstrate the potential of RS in identifying early transformation changes in oral mucosa and the efficacy of this approach in oral cancer applications.

PMID

23392131 [<https://www.ncbi.nlm.nih.gov/pubmed/?term=23392131>]

Institution

(Singh, Sahu, Deshmukh, Chaturvedi, Krishna) Chilakapati lab, ACTREC, Tata Memorial Center, Kharghar, Navi Mumbai 410210, India

Emtree Heading

adult; aged; algorithm; blood; case control study; comparative study; discriminant analysis; feasibility study; female; human; male; middle aged; mouth mucosa; *pathology; principal component analysis; *procedures; Raman spectrometry; spectrofluorometry; tobacco; young adult; Carcinoma, Squamous Cell/di [Diagnosis]; chemically induced; Mouth Neoplasms/di [Diagnosis].

Candidate Terms

Carcinoma, Squamous Cell / diagnosis [other term] chemically induced [other term]

Mouth Neoplasms / diagnosis [other term]

Other Index Terms

adult; aged; algorithm; blood; case control study; comparative study; discriminant analysis; feasibility study; female; human; male; middle aged; mouth mucosa; *pathology; principal component analysis; *procedures; Raman spectrometry; spectrofluorometry; tobacco; young adult.

Link to the Ovid Full Text or citation:

[Click here for full text options](https://ovidsp.ovid.com/ovidweb.cgi?T=JS&CSC=Y&NEWS=N&PAGE=fulltext&D=emed14&AN=603380096)

Link to the External Link Resolver:

[SFX](https://sfx-86scu.hosted.exlibrisgroup.com.cn/86scu?sid=OVID:embase&id=pmid:23392131&id=doi:10.1039%2Fc3an36761d&issn=13645528&isbn=&volume=138&issue=14&spage=4175&pages=4175-4182&date=2013&title=The+Analyst&atitle=In+vivo+Raman+spectroscopy+of+oral+buccal+mucosa%3A+a+study+on+malignancy+associated+changes+(MAC)%2Fcancer+field+effects+(CFE)&aulast=Singh&pid=<author>Singh+S.P.%3BSahu+A.%3BDeshmukh+A.%3BChaturvedi+P.%3BKrishna+C.M.<%2Fauthor><AN>603380096<%2FAN><DT>Article<%2FDT>)

302.

Raman spectroscopic investigation of the chemopreventive response of naringenin and its nanoparticles in DMBA-induced oral carcinogenesis.

Krishnakumar N., Sulfikkarali N.K., Manoharan S., Venkatachalam P.

Spectrochimica Acta - Part A: Molecular and Biomolecular Spectroscopy. 115 (pp 648-653), 2013. Date of Publication: 2013.

AN: 370088526

Raman spectroscopy is a vibrational spectroscopic technique that can be used to optically probe the bio-molecular changes associated with tumor progression. The aim of the present study is to investigate the biomolecular changes in chemopreventive response of prepared naringenin-loaded nanoparticles (NARNPs) relative to efficacy of free naringenin (NAR) during 7,12-dimethyl benz(a)anthracene (DMBA)-induced oral carcinogenesis by Fourier Transform Raman (FT-Raman) spectroscopy. Oral squamous cell carcinoma (OSCC) was developed in the buccal pouch of golden Syrian hamsters by painting with 0.5% DMBA in liquid paraffin three times a week for 14 weeks. Raman spectra differed significantly between the control and tumor tissues, with tumors showing higher percentage signals for nucleic acids, phenylalanine and tryptophan and a lower in the percentage of phospholipids. Moreover, oral administration of free NAR and NARNPs significantly increased phospholipids and decreased the levels of tryptophan, phenylalanine and nucleic acid contents. On a comparative basis, NARNPs was found to have a more potent antitumor effect than free NAR in completely preventing the formation of squamous cell carcinoma and in improving the biochemical status to a normal range in DMBA-induced oral carcinogenesis. The present study further suggest that Raman spectroscopy could be a valuable tool for rapid and sensitive detection of specific biomolecular changes in response to chemopreventive agents. © 2013 Elsevier B.V. All rights reserved.

PMID

23880406 [<https://www.ncbi.nlm.nih.gov/pubmed/?term=23880406>]

Institution

(Krishnakumar, Sulfikkarali) Department of Physics, Annamalai University, Annamalainagar 608 002, Tamil Nadu, India (Manoharan) Department of Biochemistry and Biotechnology, Annamalai University, Annamalainagar 608 002, Tamil Nadu, India

(Venkatachalam) Physics Wing, DDE, Annamalai University, Annamalainagar 608 002, Tamil Nadu, India

Publisher

Elsevier (P.O. Box 211, Amsterdam 1000 AE, Netherlands)

Emtree Heading

animal; article; *carcinogenesis; chemoprophylaxis; drug effect; Fourier analysis; hamster; light; male; Mesocricetus; mouth mucosa; *mouth tumor/dt [Drug Therapy]; *mouth tumor/pc [Prevention]; particle size; pathology; radiation scattering; *Raman spectrometry; squamous cell carcinoma/dt [Drug Therapy]; squamous cell carcinoma/pc [Prevention]; ultrastructure; 7,12 dimethylbenz[a]anthracene; *flavanone derivative/dt [Drug Therapy]; *flavanone derivative/pd [Pharmacology]; *nanoparticle/dt [Drug Therapy]; naringenin; Hamster buccal pouch carcinogenesis.

Candidate Terms

Hamster buccal pouch carcinogenesis [other term]

Drug Index Terms

7,12 dimethylbenz[a]anthracene; *flavanone derivative / *drug therapy / *pharmacology; *nanoparticle / *drug therapy; naringenin.

Other Index Terms

animal; article; *carcinogenesis; chemoprophylaxis; drug effect; Fourier analysis; hamster; light; male; Mesocricetus; mouth mucosa; *mouth tumor / *drug therapy / *prevention; particle size; pathology; radiation scattering; *Raman spectrometry; squamous cell carcinoma / drug therapy / prevention; ultrastructure.

Link to the Ovid Full Text or citation:

[Click here for full text options](https://ovidsp.ovid.com/ovidweb.cgi?T=JS&CSC=Y&NEWS=N&PAGE=fulltext&D=emed14&AN=370088526)

Link to the External Link Resolver:

[SFX](https://sfx-86scu.hosted.exlibrisgroup.com.cn/86scu?sid=OVID:embase&id=pmid:23880406&id=doi:10.1016%2Fj.saa.2013.05.076&issn=13861425&isbn=&volume=115&issue=&spage=648&pages=648-653&date=2013&title=Spectrochimica+Acta+-+Part+A%3A+Molecular+and+Biomolecular+Spectroscopy&atitle=Raman+spectroscopic+investigation+of+the+chemopreventive+response+of+naringenin+and+its+nanoparticles+in+DMBA-induced+oral+carcinogenesis&aulast=Krishnakumar&pid=<author>Krishnakumar+N.%3BSulfikkarali+N.K.%3BManoharan+S.%3BVenkatachalam+P.<%2Fauthor><AN>370088526<%2FAN><DT>Article<%2FDT>)

303.

Raman spectroscopy of oral buccal mucosa: A study on age-related physiological changes and tobacco-related pathological changes.

Sahu A., Deshmukh A., Ghanate A.D., Singh S.P., Chaturvedi P., Murali Krishna C.

Technology in Cancer Research and Treatment. 11(6) (pp 529-541), 2012. Date of Publication: December 2012.

AN: 365971507

Biophysical techniques play an important role in detecting physiological alterations during pathogenesis. Raman spectroscopy has shown immense potential in identifying several diseased conditions, including oral cancers. Classification of normal, inflammatory, premalignant and malignant conditions has been demonstrated using ex vivo Raman spectroscopy. Feasibility of recording in vivo spectra in clinically implementable time has also been shown. Translation of this technology to clinics requires extensive validation of methodologies, building of robust models and testing the same under stringent conditions as well as on diverse populations. In this context, the ability of Raman spectroscopy in identifying subtle changes in oral mucosa with increasing age, and the influence of these aging related changes on classification with tobacco-related pathological changes was evaluated. A total of 451 spectra from 62 subjects were recorded from buccal mucosa of healthy subjects of 4 different age groups (aged 20-60 years). Also, 478 spectra from 85 subjects belonging to 4 different categories, tobacco exposed mucosa, contralateral normal (opposite side of tumor), premalignant patches and tumors on buccal mucosa were recorded using fiber optic probe-coupled commercial Raman spectrometer. Differences in spectra were explored by unsupervised Principal Component Analysis (PCA) and supervised Linear Discriminant Analysis (LDA), followed by Leave one out cross validation. Results indicate feasibility of classifying early and late age groups. Also, clear classification is observed between healthy and pathological groups, thus inherent heterogeneity in healthy groups seems to have no bearing on classification of normal with abnormal conditions. Findings of the study indicate high sensitivity of Raman spectroscopy in detecting subtle mucosal changes, further supporting efficacy of Raman spectroscopic approaches in oral cancer applications. Prospectively, more vigorous validation studies of Raman methodology would enable routine clinical applications. © Adenine Press (2012).

PMID

23101756 [<https://www.ncbi.nlm.nih.gov/pubmed/?term=23101756>]

Institution

(Sahu, Deshmukh, Ghanate, Singh, Murali Krishna) Chilakapati Lab, ACTREC, Kharghar, Navi-Mumbai, 410210, India (Chaturvedi) Department of Surgical Oncology, Tata Memorial Hospital, Mumbai-400012, India

Publisher

Adenine Press

Emtree Heading

aging; *cheek mucosa; classification; discriminant analysis; ex vivo study; fiber optics; groups by age; hospital; human; methodology; model; *mouth cancer; mouth mucosa; mucosa; neoplasm; normal human; pathogenesis; population; principal component analysis; *Raman spectrometry; recording; spectrometer; technology; *tobacco; validation study.

Other Index Terms

aging [m]; *cheek mucosa [m]; classification [m]; discriminant analysis [m]; ex vivo study [m]; fiber optics [m]; groups by age [m]; hospital [m]; human [m]; methodology [m]; model [m]; *mouth cancer [m]; mouth mucosa [m]; mucosa [m]; neoplasm [m]; normal human [m]; pathogenesis [m]; population [m]; principal component analysis [m]; *Raman spectrometry [m]; recording [m]; spectrometer [m]; technology [m]; *tobacco [m]; validation study [m].

Link to the Ovid Full Text or citation:

[Click here for full text options](https://ovidsp.ovid.com/ovidweb.cgi?T=JS&CSC=Y&NEWS=N&PAGE=fulltext&D=emed13&AN=365971507)

Link to the External Link Resolver:

[SFX](https://sfx-86scu.hosted.exlibrisgroup.com.cn/86scu?sid=OVID:embase&id=pmid:23101756&id=doi:10.7785%2Ftcrt.2012.500304&issn=15330346&isbn=&volume=11&issue=6&spage=529&pages=529-541&date=2012&title=Technology+in+Cancer+Research+and+Treatment&atitle=Raman+spectroscopy+of+oral+buccal+mucosa%3A+A+study+on+age-related+physiological+changes+and+tobacco-related+pathological+changes&aulast=Sahu&pid=<author>Sahu+A.%3BDeshmukh+A.%3BGhanate+A.D.%3BSingh+S.P.%3BChaturvedi+P.%3BMurali+Krishna+C.<%2Fauthor><AN>365971507<%2FAN><DT>Article<%2FDT>)

304.

Targeting carbon nanotubes against cancer.

Fabbro C., Ali-Boucetta H., Ros T.D., Kostarelos K., Bianco A., Prato M.

Chemical Communications. 48(33) (pp 3911-3926), 2012. Date of Publication: 25 Apr 2012.

AN: 364534318

The use of carbon nanotubes (CNTs) as polyvalent tools for cancer treatment is progressing at a very fast pace. The most promising approach is the targeted delivery of drugs, designed to selectively direct the therapeutic treatment towards the tumours. CNTs may offer several advantages to overcome one of the main limitations of most existing anticancer therapies, namely the lack of selectivity. Herein, an account of the existing literature on CNT-based nanomedicine for cancer treatment is given. The most significant results obtained so far in the field of drug delivery are presented for many anticancer chemotherapeutics (doxorubicin, methotrexate, taxanes, platinum analogues, camptothecine and gemcitabine), but also for immunotherapeutics and nucleic acids. Moreover, the alternative anticancer therapies based on thermal ablation and radiotherapy are discussed. The attention throughout the review is focused on the different targeting strategies proposed so far, mainly based on antibodies, but also on other specifically recognised molecules or on the application of an external magnetic field. © 2012 The Royal Society of Chemistry.

PMID

22428156 [<https://www.ncbi.nlm.nih.gov/pubmed/?term=22428156>]

Institution

(Fabbro, Ros, Prato) Dipartimento di Scienze Chimiche e Farmaceutiche, Center of Excellence for Nanostructured Materials (CENMAT), Universita di Trieste, Trieste, 34127, Italy (Ali-Boucetta, Kostarelos) Nanomedicine Lab, UCL School of Pharmacy, University College London, WC1N 1AX, United Kingdom

(Bianco) CNRS, Institut de Biologie Moleculaire et Cellulaire, Laboratoire d'Immunologie et Chimie Therapeutiques, Strasbourg, 67000, France

Publisher

Royal Society of Chemistry

Emtree Heading

antigen presenting cell; apoptosis; breast cancer; cancer chemotherapy; cancer immunotherapy; cancer radiotherapy; cell death; cell proliferation; competitive inhibition; concentration response; confocal microscopy; controlled drug release; cycloaddition; drug bioavailability; drug cytotoxicity; *drug delivery system; drug dose comparison; drug efficacy; drug excretion; drug half life; glioblastoma; head and neck carcinoma/dt [Drug Therapy]; HeLa cell; human; humoral immunity; hydrophilicity; IC 50; in vivo study; liposomal delivery; liver cell carcinoma/dt [Drug Therapy]; lung carcinoma; magnetic field; melanoma; nanomedicine; nonhuman; protein targeting; Raman spectrometry; review; stomach carcinoma; structure activity relation; 10 hydroxycamptothecin/pr [Pharmaceutics]; 10 hydroxycamptothecin/pk [Pharmacokinetics]; arginylglycylaspartic acid; camptothecin/an [Drug Analysis]; camptothecin/dt [Drug Therapy]; camptothecin/pr [Pharmaceutics]; camptothecin/pk [Pharmacokinetics]; camptothecin/pd [Pharmacology]; *carbon nanotube; carboplatin/an [Drug Analysis]; carboplatin/pr [Pharmaceutics]; carboplatin/pk [Pharmacokinetics]; cisplatin/an [Drug Analysis]; cisplatin/dt [Drug Therapy]; cisplatin/pr [Pharmaceutics]; cisplatin/pk [Pharmacokinetics]; doxorubicin/an [Drug Analysis]; doxorubicin/do [Drug Dose]; doxorubicin/pr [Pharmaceutics]; doxorubicin/pk [Pharmacokinetics]; doxorubicin/pd [Pharmacology]; epidermal growth factor/ec [Endogenous Compound]; epidermal growth factor receptor/ec [Endogenous Compound]; gemcitabine/an [Drug Analysis]; gemcitabine/pr [Pharmaceutics]; gemcitabine/pk [Pharmacokinetics]; gemcitabine/pd [Pharmacology]; macrogol; methotrexate/an [Drug Analysis]; methotrexate/pr [Pharmaceutics]; methotrexate/pk [Pharmacokinetics]; methotrexate/pd [Pharmacology]; multi walled nanotube; paclitaxel/an [Drug Analysis]; paclitaxel/pr [Pharmaceutics]; paclitaxel/pk [Pharmacokinetics]; quantum dot; single walled nanotube; tumor cell vaccine/dt [Drug Therapy]; tumor cell vaccine/pr [Pharmaceutics]; tumor cell vaccine/pk [Pharmacokinetics]; tumor cell vaccine/sc [Subcutaneous Drug Administration].

Drug Index Terms

10 hydroxycamptothecin / pharmaceutics / pharmacokinetics; arginylglycylaspartic acid; camptothecin / drug analysis / drug therapy / pharmaceutics / pharmacokinetics / pharmacology; *carbon nanotube; carboplatin / drug analysis / pharmaceutics / pharmacokinetics; cisplatin / drug analysis / drug therapy / pharmaceutics / pharmacokinetics; doxorubicin / drug analysis / drug dose / pharmaceutics / pharmacokinetics / pharmacology; epidermal growth factor / endogenous compound; epidermal growth factor receptor / endogenous compound; gemcitabine / drug analysis / pharmaceutics / pharmacokinetics / pharmacology; macrogol; methotrexate / drug analysis / pharmaceutics / pharmacokinetics / pharmacology; multi walled nanotube; paclitaxel / drug analysis / pharmaceutics / pharmacokinetics; quantum dot; single walled nanotube; tumor cell vaccine / drug therapy / pharmaceutics / pharmacokinetics / subcutaneous drug administration.

Other Index Terms

antigen presenting cell; apoptosis; breast cancer; cancer chemotherapy; cancer immunotherapy; cancer radiotherapy; cell death; cell proliferation; competitive inhibition; concentration response; confocal microscopy; controlled drug release; cycloaddition; drug bioavailability; drug cytotoxicity; *drug delivery system; drug dose comparison; drug efficacy; drug excretion; drug half life; glioblastoma; head and neck carcinoma / drug therapy; HeLa cell; human; humoral immunity; hydrophilicity; IC 50; in vivo study; liposomal delivery; liver cell carcinoma / drug therapy; lung carcinoma; magnetic field; melanoma; nanomedicine; nonhuman; protein targeting; Raman spectrometry; review; stomach carcinoma; structure activity relation.

Drug Trade Name

doxil, taxol

Link to the Ovid Full Text or citation:

[Click here for full text options](https://ovidsp.ovid.com/ovidweb.cgi?T=JS&CSC=Y&NEWS=N&PAGE=fulltext&D=emed13&AN=364534318)

Link to the External Link Resolver:

[SFX](https://sfx-86scu.hosted.exlibrisgroup.com.cn/86scu?sid=OVID:embase&id=pmid:22428156&id=doi:10.1039%2Fc2cc17995d&issn=13597345&isbn=&volume=48&issue=33&spage=3911&pages=3911-3926&date=2012&title=Chemical+Communications&atitle=Targeting+carbon+nanotubes+against+cancer&aulast=Fabbro&pid=<author>Fabbro+C.%3BAli-Boucetta+H.%3BRos+T.D.%3BKostarelos+K.%3BBianco+A.%3BPrato+M.<%2Fauthor><AN>364534318<%2FAN><DT>Review<%2FDT>)

305.

Oncology scan - Cancer biology.

Marples B., Madlambayan G.

International Journal of Radiation Oncology Biology Physics. 83(1) (pp 3-5), 2012. Date of Publication: 01 May 2012.

AN: 364636586

Institution

(Marples, Madlambayan) Oakland University, Rochester, MI, United States

Publisher

Elsevier Inc. (360 Park Avenue South, New York NY 10010, United States)

Emtree Heading

amino terminal sequence; breast cancer/rt [Radiotherapy]; cancer radiotherapy; cancer stem cell; cell growth; *circulating tumor cell; Drosophila; epithelial mesenchymal transition; gene mutation; head and neck carcinoma; human; nonhuman; note; pancreas tumor; priority journal; Raman spectrometry; squamous cell carcinoma; CD34 antigen/ec [Endogenous Compound]; epidermal growth factor/ec [Endogenous Compound]; epidermal growth factor receptor/ec [Endogenous Compound]; nanoparticle; Notch receptor/ec [Endogenous Compound].

Drug Index Terms

CD34 antigen / endogenous compound; epidermal growth factor / endogenous compound; epidermal growth factor receptor / endogenous compound; nanoparticle; Notch receptor / endogenous compound.

Other Index Terms

amino terminal sequence; breast cancer / radiotherapy; cancer radiotherapy; cancer stem cell; cell growth; *circulating tumor cell; Drosophila; epithelial mesenchymal transition; gene mutation; head and neck carcinoma; human; nonhuman; note; pancreas tumor; priority journal; Raman spectrometry; squamous cell carcinoma.

Link to the Ovid Full Text or citation:

[Click here for full text options](https://ovidsp.ovid.com/ovidweb.cgi?T=JS&CSC=Y&NEWS=N&PAGE=fulltext&D=emed13&AN=364636586)

Link to the External Link Resolver:

[SFX](https://sfx-86scu.hosted.exlibrisgroup.com.cn/86scu?sid=OVID:embase&id=pmid:&id=doi:10.1016%2FS0360-3016%252812%252900378-1&issn=03603016&isbn=&volume=83&issue=1&spage=3&pages=3-5&date=2012&title=International+Journal+of+Radiation+Oncology+Biology+Physics&atitle=Oncology+scan+-+Cancer+biology&aulast=Marples&pid=<author>Marples+B.%3BMadlambayan+G.<%2Fauthor><AN>364636586<%2FAN><DT>Note<%2FDT>)

306.

Analysis of protein from nasopharyngeal carcinoma cell line CNE2 irradiated by X-ray using Raman spectroscopy.

Ou L., Chen Y., Huang Y., Su Y., Lin D., Zou C., Lei J.

Spectroscopy. 27(1) (pp 1-7), 2012. Date of Publication: 2012.

AN: 364473859

Our work aims to explore Raman spectroscopy (RS) to study the effects of total protein of nasopharyngeal carcinoma (NPC) cell line CNE2 treated by different doses of X-ray irradiation. The total protein is extracted from the cell line after irradiation with no incubation (0 h) and with incubation for 72 h, respectively. Both paired-samples t test and support vector machine (SVM) are employed for statistical analysis of Raman spectrum. The results show that several Raman bands assigned to total protein can be detected; and the X-ray irradiation have various effects on different components in total protein, especially for the dose higher than 6 Gy, and also associated with the incubation time after irradiation. The differences of content between 0 h groups and 72 h groups appear at bands of C-S stretching, C-H stretching, O=C-O - stretching and L-phenylalanine. It indicated no significant diversification on structures of protein, while the content may be varied during irradiation. Our spectroscopic results reveal that RS analysis of total protein of cell line irradiated by X-ray, in conjunction with mathematical statistical model, can be a potential method to explore the Raman characterization target of cell radiosensitivity, and then for making known the mechanism of radiosensitivity of tumor further. © 2012 - IOS Press and the authors. All rights reserved.

Institution

(Ou, Lin, Lei) Key Laboratory of Optoelectronic Science and Technology for Medicine, Ministry of Education, Fujian Normal University, Fuzhou, China (Chen) Zhicheng College, Fuzhou University, Fuzhou, China

(Huang) Key Laboratory of Instrumentation Science and Dynamic Measurement, Ministry of Education, North University of China, Taiyuan, China

(Su, Zou) Fujian Provincial Tumor Hospital, Fuzhou, China

Publisher

Hindawi Publishing Corporation (410 Park Avenue, 15th Floor, 287 pmb, New York NY 10022, United States)

Emtree Heading

article; cancer cell culture; controlled study; human; human cell; mathematical model; *nasopharynx carcinoma; protein analysis; radiation dose; Raman spectrometry; X irradiation; *protein/ec [Endogenous Compound].

Drug Index Terms

*protein / *endogenous compound.

Other Index Terms

article; cancer cell culture; controlled study; human; human cell; mathematical model; *nasopharynx carcinoma; protein analysis; radiation dose; Raman spectrometry; X irradiation.

Link to the Ovid Full Text or citation:

[Click here for full text options](https://ovidsp.ovid.com/ovidweb.cgi?T=JS&CSC=Y&NEWS=N&PAGE=fulltext&D=emed13&AN=364473859)

Link to the External Link Resolver:

[SFX](https://sfx-86scu.hosted.exlibrisgroup.com.cn/86scu?sid=OVID:embase&id=pmid:&id=doi:10.3233%2FSPE-2012-0569&issn=07124813&isbn=&volume=27&issue=1&spage=1&pages=1-7&date=2012&title=Spectroscopy&atitle=Analysis+of+protein+from+nasopharyngeal+carcinoma+cell+line+CNE2+irradiated+by+X-ray+using+Raman+spectroscopy&aulast=Ou&pid=<author>Ou+L.%3BChen+Y.%3BHuang+Y.%3BSu+Y.%3BLin+D.%3BZou+C.%3BLei+J.<%2Fauthor><AN>364473859<%2FAN><DT>Article<%2FDT>)

307.

Immunoassay for LMP1 in nasopharyngeal tissue based on surface-enhanced Raman scattering.

Chen Y., Zheng X., Chen G., He C., Zhu W., Feng S., Xi G., Chen R., Lan F., Zeng H.

International Journal of Nanomedicine. 7 (pp 73-82), 2012. Date of Publication: 2012.

AN: 364206199

Background: Previous studies have shown that Epstein-Barr virus (EBV)-encoded latent membrane protein 1 (LMP1) is closely associated with the occurrence and development of nasopharyngeal carcinoma, and can be used as a tumor marker in screening for the disease. Here we report a new methodology based on highly specific and sensitive surface-enhanced Raman scattering (SERS) technology to detect LMP1 in nasopharyngeal tissue sections directly with no need of tedious procedures as with conventional immunohistochemistry methods.

Method(s): LMP1-functionalized 4-mercaptobenzoic acid (4-MBA)-labeled Au/Ag core-shell bimetallic nanoparticles were prepared frst and then applied for analyzing LMP1 in formalin-fxed paraffn-embedded nasopharyngeal tissue sections obtained from 34 cancer patients and 20 healthy controls. SERS spectra were acquired from a 25 x 25 spot square area on each tissue section and used to generate SERS images.

Result(s): Data from SERS spectra and images show that this new SERS-based immunoassay detected LMP1 in formalin-fixed paraffn-embedded nasopharyngeal tissue sections with high sensitivity and specificity. The results from the new LMP1-SERS probe method are superior to those of conventional immunohistochemistry staining for LMP1, and in excellent agreement with those of in situ hybridization for EBV-encoded small RNA (EBER).

Conclusion(s): This new SERS technique has the potential to be developed into a new clinical tool for detection and differential diagnosis of nasopharyngeal carcinoma as well as for predicting metastasis and immune-targeted treatment of nasopharyngeal carcinoma. © 2012 Chen et al.

PMID

22275824 [<https://www.ncbi.nlm.nih.gov/pubmed/?term=22275824>]

Institution

(Chen, Zheng, Chen, He, Zhu) Pathology Department of Fujian Provincial Tumor Hospital, Teaching Hospital of Fujian Medical University, China (Feng, Xi, Chen) Key Laboratory of OptoElectronic Science and Technology for Medicine, Ministry of Education, Fujian Normal University, China

(Lan) Research Center for Molecular Diagnosis of Genetic Diseases, Fuzhou General Hospital, Clinical College of Fujian Medical University, Fuzhou, Fujian, China

(Zeng) Imaging Unit, Integrative Oncology Department, British Columbia Cancer Agency Research Centre, Vancouver, Canada

Publisher

DOVE Medical Press Ltd. (PO Box 300-008, Albany, Auckland, New Zealand)

Emtree Heading

article; cancer screening; cervical lymph node; clinical article; controlled study; differential diagnosis; Epstein Barr virus; human; human tissue; *immunoassay; immunohistochemistry; in situ hybridization; metastasis; *nasopharynx carcinoma/di [Diagnosis]; prognosis; *Raman spectrometry; sensitivity and specificity; socioeconomics; *latent membrane protein 1/ec [Endogenous Compound]; monoclonal antibody/ec [Endogenous Compound]; nanoparticle; thiosalicylic acid; tumor marker/ec [Endogenous Compound].

Drug Index Terms

*latent membrane protein 1 / *endogenous compound; monoclonal antibody / endogenous compound; nanoparticle; thiosalicylic acid; tumor marker / endogenous compound.

Other Index Terms

article; cancer screening; cervical lymph node; clinical article; controlled study; differential diagnosis; Epstein Barr virus; human; human tissue; *immunoassay; immunohistochemistry; in situ hybridization; metastasis; *nasopharynx carcinoma / *diagnosis; prognosis; *Raman spectrometry; sensitivity and specificity; socioeconomics.

Link to the Ovid Full Text or citation:

[Click here for full text options](https://ovidsp.ovid.com/ovidweb.cgi?T=JS&CSC=Y&NEWS=N&PAGE=fulltext&D=emed13&AN=364206199)

Link to the External Link Resolver:

[SFX](https://sfx-86scu.hosted.exlibrisgroup.com.cn/86scu?sid=OVID:embase&id=pmid:22275824&id=doi:&issn=11769114&isbn=&volume=7&issue=&spage=73&pages=73-82&date=2012&title=International+Journal+of+Nanomedicine&atitle=Immunoassay+for+LMP1+in+nasopharyngeal+tissue+based+on+surface-enhanced+Raman+scattering&aulast=Chen&pid=<author>Chen+Y.%3BZheng+X.%3BChen+G.%3BHe+C.%3BZhu+W.%3BFeng+S.%3BXi+G.%3BChen+R.%3BLan+F.%3BZeng+H.<%2Fauthor><AN>364206199<%2FAN><DT>Article<%2FDT>)

308.

Optical diagnosis of laryngeal cancer using high wavenumber Raman spectroscopy.

Lin K., Cheng D.L.P., Huang Z.

Biosensors and Bioelectronics. 35(1) (pp 213-217), 2012. Date of Publication: 15 May 2012.

AN: 51938194

We report the implementation of the transnasal image-guided high wavenumber (HW) Raman spectroscopy to differentiate tumor from normal laryngeal tissue at endoscopy. A rapid-acquisition Raman spectroscopy system coupled with a miniaturized fiber-optic Raman probe was utilized to realize real-time HW Raman (2800-3020cm-1) measurements in the larynx. A total of 94 HW Raman spectra (22 normal sites, 72 tumor sites) were acquired from 39 patients who underwent laryngoscopic screening. Significant differences in Raman intensities of prominent Raman bands at 2845, 2880 and 2920cm-1 (CH2 stretching of lipids), and 2940cm-1 (CH3 stretching of proteins) were observed between normal and cancer laryngeal tissue. The diagnostic algorithms based on principal components analysis (PCA) and linear discriminant analysis (LDA) together with the leave-one subject-out, cross-validation method on HW Raman spectra yielded a diagnostic sensitivity of 90.3% (65/72) and specificity of 90.9% (20/22) for laryngeal cancer identification. This study demonstrates that HW Raman spectroscopy has the potential for the noninvasive, real-time diagnosis and detection of laryngeal cancer at the molecular level. © 2012 Elsevier B.V.

PMID

22465448 [<https://www.ncbi.nlm.nih.gov/pubmed/?term=22465448>]

Institution

(Lin, Huang) Optical Bioimaging Laboratory, Department of Bioengineering, Faculty of Engineering, National University of Singapore, Singapore 117576, Singapore (Cheng) Department of Otolaryngology, Singapore General Hospital, Singapore 169608, Singapore

Publisher

Elsevier Ltd (Langford Lane, Kidlington, Oxford OX5 1GB, United Kingdom)

Emtree Heading

adult; algorithm; article; *cancer diagnosis; clinical article; controlled study; diagnostic value; differential diagnosis; discriminant analysis; fiber optics; human; human tissue; laryngoscopy; *larynx cancer/di [Diagnosis]; linear system; measurement; molecular diagnosis; non invasive procedure; principal component analysis; *Raman spectrometry; receiver operating characteristic; sensitivity and specificity; validation process; lipid/ec [Endogenous Compound]; protein/ec [Endogenous Compound]; *high wavenumber Raman spectrometry.

Candidate Terms

*high wavenumber Raman spectrometry [other term]

Drug Index Terms

lipid / endogenous compound; protein / endogenous compound.

Other Index Terms

adult; algorithm; article; *cancer diagnosis; clinical article; controlled study; diagnostic value; differential diagnosis; discriminant analysis; fiber optics; human; human tissue; laryngoscopy; *larynx cancer / *diagnosis; linear system; measurement; molecular diagnosis; non invasive procedure; principal component analysis; *Raman spectrometry; receiver operating characteristic; sensitivity and specificity; validation process.

Link to the Ovid Full Text or citation:

[Click here for full text options](https://ovidsp.ovid.com/ovidweb.cgi?T=JS&CSC=Y&NEWS=N&PAGE=fulltext&D=emed13&AN=51938194)

Link to the External Link Resolver:

[SFX](https://sfx-86scu.hosted.exlibrisgroup.com.cn/86scu?sid=OVID:embase&id=pmid:22465448&id=doi:10.1016%2Fj.bios.2012.02.050&issn=09565663&isbn=&volume=35&issue=1&spage=213&pages=213-217&date=2012&title=Biosensors+and+Bioelectronics&atitle=Optical+diagnosis+of+laryngeal+cancer+using+high+wavenumber+Raman+spectroscopy&aulast=Lin&pid=<author>Lin+K.%3BCheng+D.L.P.%3BHuang+Z.<%2Fauthor><AN>51938194<%2FAN><DT>Article<%2FDT>)

309.

Nanogoldtechnology-imaging, sensing and target therapy in head and neck cancer.

Sultana N., Shenoy S.B., Sham M., Keshav S., Kaul R.

Clinical Cancer Investigation Journal. 1(1) (pp 6-12), 2012. Date of Publication: January-March 2012.

AN: 368089361

Innovation in the last decade has endowed nanotechnology with an assortment of tools for delivery, imaging, and sensing in cancer research. Cancer nanotherapeutics are rapidly progressing and are being implemented to solve several limitations of conventional drug delivery systems such as nonspecific biodistribution and targeting, lack of water solubility, poor oral bioavailability, and low therapeutic indices. To improve the biodistribution of cancer drugs, nanoparticles have been designed for optimal size and surface characteristics to increase their circulation time in the bloodstream. Nanoparticles have the ability to accumulate in cells without being recognized by P-glycoprotein, one of themainmediators of multidrug resistance, resulting in the increased intracellular concentration of drugs. Multifunctional and multiplex nanoparticles are now being actively investigated and are on the horizon as the next generation of nanoparticles, facilitating personalized and tailored cancer treatment.

Institution

(Sultana, Shenoy, Keshav, Kaul) Departments of Oral Medicine and Radiology, Vydehi Institute of Dental Sciences, No-37, 1st Cross, Silver Oak Street, JP Nagar, Bangalore-78, India (Sham) Departments of Maxillofacial Surgery, Vydehi Institute of Dental Sciences, Bangalore, India

Publisher

Medknow Publications and Media Pvt. Ltd (B9, Kanara Business Centre, off Link Road, Ghatkopar (E), Mumbai 400 075, India)

Emtree Heading

aphthous stomatitis; bioinformatics; cancer cell; cancer therapy; colorectal cancer/dt [Drug Therapy]; drug delivery system; drug resistance; drug targeting; erythema; *head and neck cancer; head and neck squamous cell carcinoma/dt [Drug Therapy]; in vitro study; in vivo study; magnetic resonance angiography; molecular diagnosis; molecular imaging; *nanomedicine; *nanosensor; nuclear magnetic resonance imaging; optical coherence tomography; oral mucositis/co [Complication]; photoacoustic tomography; priority journal; Raman spectrometry; review; synthesis; tumor microenvironment; tumor vascularization; carbon nanotube; cetuximab/dt [Drug Therapy]; dendrimer; *gold nanoparticle; liposome; *nanocarrier; nanoparticle; nanoshell; unclassified drug; virus nanoparticle.

Candidate Terms

virus nanoparticle [drug term]

Drug Index Terms

carbon nanotube; cetuximab / drug therapy; dendrimer; *gold nanoparticle; liposome; *nanocarrier; nanoparticle; nanoshell; unclassified drug.

Other Index Terms

aphthous stomatitis; bioinformatics; cancer cell; cancer therapy; colorectal cancer / drug therapy; drug delivery system; drug resistance; drug targeting; erythema; *head and neck cancer; head and neck squamous cell carcinoma / drug therapy; in vitro study; in vivo study; magnetic resonance angiography; molecular diagnosis; molecular imaging; *nanomedicine; *nanosensor; nuclear magnetic resonance imaging; optical coherence tomography; oral mucositis / complication; photoacoustic tomography; priority journal; Raman spectrometry; review; synthesis; tumor microenvironment; tumor vascularization.

Drug Trade Name

erbitux: Imclone

Link to the Ovid Full Text or citation:

[Click here for full text options](https://ovidsp.ovid.com/ovidweb.cgi?T=JS&CSC=Y&NEWS=N&PAGE=fulltext&D=emed13&AN=368089361)

Link to the External Link Resolver:

[SFX](https://sfx-86scu.hosted.exlibrisgroup.com.cn/86scu?sid=OVID:embase&id=pmid:&id=doi:10.4103%2F2278-0513.95012&issn=22780513&isbn=&volume=1&issue=1&spage=6&pages=6-12&date=2012&title=Clinical+Cancer+Investigation+Journal&atitle=Nanogoldtechnology-imaging%2C+sensing+and+target+therapy+in+head+and+neck+cancer&aulast=Sultana&pid=<author>Sultana+N.%3BShenoy+S.B.%3BSham+M.%3BKeshav+S.%3BKaul+R.<%2Fauthor><AN>368089361<%2FAN><DT>Review<%2FDT>)

310.

Direct carbonization of Al-based porous coordination polymer for synthesis of nanoporous carbon.

Hu M., Reboul J., Furukawa S., Torad N.L., Ji Q., Srinivasu P., Ariga K., Kitagawa S., Yamauchi Y.

Journal of the American Chemical Society. 134(6) (pp 2864-2867), 2012. Date of Publication: 15 Feb 2012.

AN: 364258192

Nanoporous carbon (NPC) is prepared by direct carbonization of Al-based porous coordination polymers (Al-PCP). By applying the appropriate carbonization temperature, both high surface area and large pore volume are realized for the first time. Our NPC shows much higher porosity than other carbon materials (such as activated carbons and mesoporous carbons). This new type of carbon material exhibits superior sensing capabilities toward toxic aromatic substances. © 2012 American Chemical Society.

Institution

(Hu, Torad, Ji, Srinivasu, Ariga, Yamauchi) World Premier International (WPI) Research Center for Materials Nanoarchitectonics (MANA), National Institute for Materials Science (NIMS), 1-1 Namiki, Tsukuba, Ibaraki 305-0044, Japan (Reboul, Furukawa, Kitagawa) ERATO Kitagawa Integrated Pores Project, Japan Science and Technology Agency (JST), Kyoto Research Park Bldg no 3, Shimogyo, Kyoto 600-8815, Japan

(Reboul, Furukawa, Kitagawa) Institute for Integrated Cell-Material Sciences (ICeMS), Kyoto University, Yoshida, Sakyo, Kyoto 606-8501, Japan

(Torad, Yamauchi) Faculty of Science and Engineering, Waseda University, 3-4-1 Okubo, Shinjuku, Tokyo 169-8555, Japan

(Ariga, Yamauchi) Core Research for Evolutional Science and Technology (CREST), Precursory Research for Embryonic Science and Technology (PRESTO), Japan Science and Technology Agency (JST), 4-1-8, Honcho, Kawaguchi, Saitama 332-0012, Japan

Publisher

American Chemical Society (2540 Olentangy River Road, P.O. Box 3337, Columbus OH 43210-3337, United States)

Emtree Heading

adsorption; article; *materials; molecular weight; Raman spectrometry; scanning electron microscopy; synthesis; temperature; thermostability; transmission electron microscopy; X ray diffraction; *aluminum; aromatic hydrocarbon; *carbon; coordination compound; cyclohexane; hexane; organometallic compound; *polymer; toluene; unclassified drug; zeolite; *nanoporous carbon.

Candidate Terms

*nanoporous carbon [drug term]

Drug Index Terms

*aluminum; aromatic hydrocarbon; *carbon; coordination compound; cyclohexane; hexane; organometallic compound; *polymer; toluene; unclassified drug; zeolite.

Other Index Terms

adsorption; article; *materials; molecular weight; Raman spectrometry; scanning electron microscopy; synthesis; temperature; thermostability; transmission electron microscopy; X ray diffraction.

Link to the Ovid Full Text or citation:

[Click here for full text options](https://ovidsp.ovid.com/ovidweb.cgi?T=JS&CSC=Y&NEWS=N&PAGE=fulltext&D=emed13&AN=364258192)

Link to the External Link Resolver:

[SFX](https://sfx-86scu.hosted.exlibrisgroup.com.cn/86scu?sid=OVID:embase&id=pmid:&id=doi:10.1021%2Fja208940u&issn=00027863&isbn=&volume=134&issue=6&spage=2864&pages=2864-2867&date=2012&title=Journal+of+the+American+Chemical+Society&atitle=Direct+carbonization+of+Al-based+porous+coordination+polymer+for+synthesis+of+nanoporous+carbon&aulast=Hu&pid=<author>Hu+M.%3BReboul+J.%3BFurukawa+S.%3BTorad+N.L.%3BJi+Q.%3BSrinivasu+P.%3BAriga+K.%3BKitagawa+S.%3BYamauchi+Y.<%2Fauthor><AN>364258192<%2FAN><DT>Article<%2FDT>)

311.

Raman spectroscopy in head and neck cancers: Toward oncological applications.

Singh S.P., Deshmukh A., Chaturvedi P., Krishna C.M.

Journal of Cancer Research and Therapeutics. 8(SUPPL. 2) (pp S126-S132), 2012. Date of Publication: January 2012.

AN: 364257146

Aims: To test the spectral reproducibility of earlier findings under ex vivo conditions and to record good quality in vivo spectra in clinically implementable time in finger print region.

Material(s) and Method(s): Spectra from 20 ex vivo tissues (10 normal and 10 tumor) were recorded using fiber optic probe coupled Raman spectrometer. In vivo spectra from 10 healthy volunteers were also recorded. Spectral differences were analyzed by PC-LDA method followed by validation by leave-one-out and test samples.

Result(s): Spectral features of ex vivo normal tissue suggest predominant lipid features while protein is high in tumor conditions. Major spectral features of in vivo healthy volunteers share several commonalities with ex vivo normal tissues except minor differences in amide III region. Classification efficiency of 90, 85 and 96% for ex vivo tumor, normal and in vivo normal standard models, respectively after leave-one-out cross validation, was observed. Test prediction efficiency of standard models of ex vivo normal, ex vivo tumor and in vivo healthy volunteers were 82.5, 92.5 and 100%, respectively.

Conclusion(s): Our findings corroborate with the reported ex vivo and in vivo normal spectral features. Features of in vivo Raman spectra show strong similarities with ex vivo normal spectra minor but significant differences were also observed. Findings of this study indicate that with our instrument in vivo Raman spectra in finger print region can be recorded in short and clinically implementable time.

PMID

22322731 [<https://www.ncbi.nlm.nih.gov/pubmed/?term=22322731>]

Institution

(Singh, Deshmukh, Krishna) Chilakapati Laboratory, Cancer Research Institute, ACTREC TMC, Kharghar, Sector '22', Navi Mumbai - 410210, India (Chaturvedi) Department of Surgical Oncology, Tata Memorial Hospital, Mumbai 400012, India

Publisher

Medknow Publications and Media Pvt. Ltd (B9, Kanara Business Centre, off Link Road, Ghatkopar (E), Mumbai 400 075, India)

Emtree Heading

article; cancer classification; controlled study; ex vivo study; fiber optics; *head and neck cancer/di [Diagnosis]; human; human tissue; in vivo study; lipid blood level; prediction; *Raman spectrometry; spectrometer; tumor/di [Diagnosis]; amide; lipid/ec [Endogenous Compound].

Drug Index Terms

amide; lipid / endogenous compound.

Other Index Terms

article; cancer classification; controlled study; ex vivo study; fiber optics; *head and neck cancer / *diagnosis; human; human tissue; in vivo study; lipid blood level; prediction; *Raman spectrometry; spectrometer; tumor / diagnosis.

Link to the Ovid Full Text or citation:

[Click here for full text options](https://ovidsp.ovid.com/ovidweb.cgi?T=JS&CSC=Y&NEWS=N&PAGE=fulltext&D=emed13&AN=364257146)

Link to the External Link Resolver:

[SFX](https://sfx-86scu.hosted.exlibrisgroup.com.cn/86scu?sid=OVID:embase&id=pmid:22322731&id=doi:10.4103%2F0973-1482.92227&issn=09731482&isbn=&volume=8&issue=SUPPL.+2&spage=S126&pages=S126-S132&date=2012&title=Journal+of+Cancer+Research+and+Therapeutics&atitle=Raman+spectroscopy+in+head+and+neck+cancers%3A+Toward+oncological+applications&aulast=Singh&pid=<author>Singh+S.P.%3BDeshmukh+A.%3BChaturvedi+P.%3BKrishna+C.M.<%2Fauthor><AN>364257146<%2FAN><DT>Article<%2FDT>)

312.

Behavior of circulating tumor cells detected by EGF tagged SERS nanoparticles in head and neck cancer patients.

Beitler J.J., Wang X., Qian X., Wang H., Chen Z.G., Khuri F.R., Nie S., Shin D.M.

Journal of Clinical Oncology. Conference: 2012 Annual Meeting of the American Society of Clinical Oncology, ASCO. Chicago, IL United States. Conference Publication: (var.pagings). 30(15 SUPPL. 1) (no pagination), 2012. Date of Publication: 20 May 2012.

AN: 71007235

Background: Quantification of circulating tumor cells(CTCs) is becoming central to the management of metastatic breast, colorectal and prostate cancers and the potential to improve management of head and neck squamous cell cancer (HNSCC) patients is very high. Our group has published our experience with nanotechnology, highly specific, surface-enhanced Raman spectroscopy(SERS) and Epidermal Growth Factor Receptor(EGFR) ligands to identify 7-720 CTCs/ml in 19 samples from HNSCC patients[1]. Our goal here is to understand the clinical behavior of CTCs in HNSCC for selected patients whom we believe our single channel assay has been accurate.

Method(s): Our methods for detection of CTCs using EGFR ligands have been previously described[1]. After comparing CTCs in 116 patient samples with and without gross disease, we examined 8 patients with decreasing sequential single channel assays of CTCs and no evidence of disease at last follow-up.

Result(s): For 56 samples from patients with no gross disease, the mean CTC was 94/ml. For 61 samples from patients with gross disease the mean CTC was 193/ml (p=0.002). Among our 4 samples where we had initial disease and a non-zero, no evidence of disease, second sample, the absolute and percentage decrease per month in CTC was 121/ml/month(range 43-160) and 17%/month(range 11-25%). The decrease of 121 CTC/ml/month correlated well with 5 out of 6 other data sets.

Conclusion(s): Targeting of EGFR for detection of CTCs in HNSCC patients appears very promising. Among +EGFR HNSCC patients who appear cured, the average decrease in CTC per ml was 121 per month and 17% per month. To further enhance the sensitivity and specificity of detection of CTCs, we are developing 3 SERS nanoparticles (multiplexed SERS) to target specific and distinct targets on the CTC surface while still maintaining a single tube assay system. 1. Wang, X., et al., Detection of circulating tumor cells in human peripheral blood using surface-enhanced Raman scattering nanoparticles.

Institution

(Beitler, Wang, Qian, Wang, Chen, Khuri, Nie, Shin) Winship Cancer Institute, Emory University, Atlanta, GA, United States

Publisher

American Society of Clinical Oncology

Emtree Heading

*head and neck cancer; *cancer patient; *human; *society; *oncology; *circulating tumor cell; patient; assay; Raman spectrometry; prostate cancer; squamous cell carcinoma; nanotechnology; neck; tube; follow up; breast; sensitivity and specificity; blood; *nanoparticle; ligand; epidermal growth factor receptor.

Drug Index Terms

*nanoparticle; ligand; epidermal growth factor receptor.

Other Index Terms

*head and neck cancer; *cancer patient; *human; *society; *oncology; *circulating tumor cell; patient; assay; Raman spectrometry; prostate cancer; squamous cell carcinoma; nanotechnology; neck; tube; follow up; breast; sensitivity and specificity; blood.

Link to the Ovid Full Text or citation:

[Click here for full text options](https://ovidsp.ovid.com/ovidweb.cgi?T=JS&CSC=Y&NEWS=N&PAGE=fulltext&D=emed13&AN=71007235)

Link to the External Link Resolver:

[SFX](https://sfx-86scu.hosted.exlibrisgroup.com.cn/86scu?sid=OVID:embase&id=pmid:&id=doi:&issn=0732183X&isbn=&volume=30&issue=15+SUPPL.+1&spage=&pages=&date=2012&title=Journal+of+Clinical+Oncology&atitle=Behavior+of+circulating+tumor+cells+detected+by+EGF+tagged+SERS+nanoparticles+in+head+and+neck+cancer+patients&aulast=Beitler&pid=<author>Beitler+J.J.%3BWang+X.%3BQian+X.%3BWang+H.%3BChen+Z.G.%3BKhuri+F.R.%3BNie+S.%3BShin+D.M.<%2Fauthor><AN>71007235<%2FAN><DT>Conference+Abstract<%2FDT>)

313.

Identification of radioresistance related proteins in human oral cancer cells by fractionated ionizing radiation.

Yasser M., Pawar S., Govekar R., Zingde S., Teni T.

Journal of Cancer Research and Therapeutics. Conference: International Conference on Radiation Biology, ICRB-2012 on Cosmic Radiation to Cancer Therapeutics and 11th Biennial Meeting of the Indian Society of Radiation Biology. Navi Mumbai India. Conference Publication: (var.pagings). 8(3) (pp 473-474), 2012. Date of Publication: July-September 2012.

AN: 70989515

Tumor cell resistance to ionizing radiation and chemotherapy is a major obstacle in cancer therapy. Identification of proteins that are differentially-expressed between radiosensitive and radioresistant cancer cells is important for the ability to predict clinical effectiveness of radiotherapy. We selected two oral cancer cell lines AW13516 and SCC29B of different sites with differing radiosensitivity. Radioresistant sublines of AW13516 and SCC29B were established by treatment with 2Gy Fractionated Ionizing Radiation using 60Co-gamma Linear Accelerator (Bhabhatron, ACTREC) up to a total dose of 70Gy. Protein lysates of parental and radioresistant AW13516, SCC29B cells were separated using 2-Dimensional gel electrophoresis in the PI range 3-10, coomassie stained and differentially expressed spots were identified. The differential spots were eluted and analyzed by MALDI-TOF. A total of 45 proteins were identified to be differentially expressed between parental and radioresistant sublines. Among them 14 proteins were found to be differentially expressed in AW 13516, 9 proteins in SCC 29B and 22 common proteins in both cell lines. The parental and radioresistant sublines were further characterized by morphological evaluation, cell cycle analysis, Annexin V/PI staining and Raman spectroscopy. Protein analysis of radioresistant sublines may provide new insights into the mechanism underlying clinical radioresistance and help in improving the efficacy of radiotherapy of oral cancers.

Institution

(Yasser, Pawar, Teni) Teni Lab, Navi Mumbai, India (Govekar, Zingde) Zingde Lab Advanced Centre for Treatment Research and Education in Cancer ACTREC, Navi Mumbai, India

Publisher

Medknow Publications and Media Pvt. Ltd

Emtree Heading

*human; *radiobiology; *mouth cancer; *neoplasm; *cancer cell; *ionizing radiation; *cosmic radiation; *radiosensitivity; *therapy; *Indian; *society; radiotherapy; Raman spectrometry; cancer therapy; cell cycle; cell line; chemotherapy; gel electrophoresis; linear accelerator; cancer cell culture; staining; protein analysis; clinical effectiveness; tumor cell; *protein; annexin; cobalt 60.

Drug Index Terms

*protein; annexin; cobalt 60.

Other Index Terms

*human; *radiobiology; *mouth cancer; *neoplasm; *cancer cell; *ionizing radiation; *cosmic radiation; *radiosensitivity; *therapy; *Indian; *society; radiotherapy; Raman spectrometry; cancer therapy; cell cycle; cell line; chemotherapy; gel electrophoresis; linear accelerator; cancer cell culture; staining; protein analysis; clinical effectiveness; tumor cell.

Link to the Ovid Full Text or citation:

[Click here for full text options](https://ovidsp.ovid.com/ovidweb.cgi?T=JS&CSC=Y&NEWS=N&PAGE=fulltext&D=emed13&AN=70989515)

Link to the External Link Resolver:

[SFX](https://sfx-86scu.hosted.exlibrisgroup.com.cn/86scu?sid=OVID:embase&id=pmid:&id=doi:&issn=09731482&isbn=&volume=8&issue=3&spage=473&pages=473-474&date=2012&title=Journal+of+Cancer+Research+and+Therapeutics&atitle=Identification+of+radioresistance+related+proteins+in+human+oral+cancer+cells+by+fractionated+ionizing+radiation&aulast=Yasser&pid=<author>Yasser+M.%3BPawar+S.%3BGovekar+R.%3BZingde+S.%3BTeni+T.<%2Fauthor><AN>70989515<%2FAN><DT>Conference+Abstract<%2FDT>)

314.

AAOMS 94th Annual Meeting Scientific Sessions and Exhibition.

Anonymous

Journal of Oral and Maxillofacial Surgery. Conference: 94th Annual Meeting Scientific Sessions and Exhibition of the American Association of Oral and Maxillofacial Surgeons, AAOMS 2012. San Diego, CA United States. Conference Publication: (var.pagings). 70(9 SUPPL. 2) (no pagination), 2012. Date of Publication: September 2012.

AN: 70875439

The proceedings contain 169 papers. The topics discussed include: upper airway stimulation in patients with obstructive sleep apnea undergoing maxillomandibular advancement; incidence, survival and mortality of oral and oropharyngeal cancer: a surveillance, epidemiology and end results program-based analysis; prostate specific antigen (human kallikrein protein 3) expression in maxillofacial cysts and tumors; postoperative stress-induced gastrointestinal bleeding in healthy adolescent patients following orthognathic surgery: report of two cases and review of literature; soft tissue changes during maxillofacial surgery; is virtual surgical planning in orthognathic surgery error proof?; using Raman spectroscopy to evaluate bone graft and implant osseointegration non-invasively: a novel and groundbreaking technique; and a survey of methods used to secure an airway in patients with facial burn injuries.

Publisher

W.B. Saunders

Emtree Heading

*maxillofacial surgery; human; patient; orthognathic surgery; sleep apnea syndrome; oropharynx cancer; survival; mortality; tumor; epidemiology; cyst; gastrointestinal hemorrhage; adolescent; soft tissue; stimulation; planning; Raman spectrometry; bone graft; implant; bone regeneration; airway; burn; upper respiratory tract; prostate specific antigen; kallikrein; synapsin II.

Drug Index Terms

prostate specific antigen; kallikrein; synapsin II.

Other Index Terms

*maxillofacial surgery; human; patient; orthognathic surgery; sleep apnea syndrome; oropharynx cancer; survival; mortality; tumor; epidemiology; cyst; gastrointestinal hemorrhage; adolescent; soft tissue; stimulation; planning; Raman spectrometry; bone graft; implant; bone regeneration; airway; burn; upper respiratory tract.

Link to the Ovid Full Text or citation:

[Click here for full text options](https://ovidsp.ovid.com/ovidweb.cgi?T=JS&CSC=Y&NEWS=N&PAGE=fulltext&D=emed13&AN=70875439)

Link to the External Link Resolver:

[SFX](https://sfx-86scu.hosted.exlibrisgroup.com.cn/86scu?sid=OVID:embase&id=pmid:&id=doi:&issn=02782391&isbn=&volume=70&issue=9+SUPPL.+2&spage=&pages=&date=2012&title=Journal+of+Oral+and+Maxillofacial+Surgery&atitle=AAOMS+94th+Annual+Meeting+Scientific+Sessions+and+Exhibition&aulast=&pid=<author>anonymous<%2Fauthor><AN>70875439<%2FAN><DT>Conference+Review<%2FDT>)

315.

Investigation of raman spectroscopy and optical coherence tomography to aid in diagnosis of oral cancer.

Hearnden V., Rehman I.U., MacNeil S., Murdoch C., Thornhill M.H., Hunter K., Colley H.E.

European Journal of Cancer. Conference: 22nd Biennial Congress of the European Association for Cancer Research, EACR 2012. Barcelona Spain. Conference Publication: (var.pagings). 48(SUPPL. 5) (pp S287), 2012. Date of Publication: July 2012.

AN: 70820904

Introduction: The diagnosis of oral cancer (OC) currently requires surgical biopsy and histological analysis which is invasive and time consuming. The prognosis is far better if OC is diagnosed early. Therefore, technologies that aid detection, improve diagnostic accuracy and are non-invasive, particularly if the results are available at the point of care and in real-time, will be of great patient benefit.

Aim(s): To investigate two novel methods for the diagnosis of OC. The first of these is Raman spectroscopy, a technique able to determine the specific chemical bonds present in a sample and their relative quantities. The second technique is optical coherence tomography (OCT), a non-invasive imaging technique which is commonly used in ophthalmology. Here we investigate the potential of this technology to visualise the oral epithelium.

Material(s) and Method(s): A DXR Raman microscope was used to obtain spectra from healthy, pre-cancerous and cancerous tissue sections. These sections were from either tissue engineered models of OC and the oral mucosa or from archived patient samples. Raman spectra (RS) were also obtained from different cell lines (cancer and dysplastic), healthy oral cells isolated from excised patient tissue and tissue engineered models of mucosa and OC. Tissue engineered models were cultured as described previously (Colley et al., Br J Cancer 2011). OCT images of tissue engineered models and healthy human volunteers were obtained using an OCT device. Results and Discussion: It is possible to obtain RS from tissue sections, cells and tissue engineered models, and differences were observed between normal and cancerous samples. OCT could non-invasively image over 500 mm into oral tissue in real-time. It was possible to identify the epithelial-connective tissue architecture and superficial connective tissue structures such as blood vessels and minor salivary glands.

Conclusion(s): RS of in vitro samples and ex vivo sections show variations between healthy tissue and cancerous tissues which could have potential as a diagnostic aid for OC. Further studies are required to determine the potential use in patients. OCT images show differences between tissue engineered models of OC and normal mucosa. Real-time images of healthy volunteers were easily obtained and showed the epithelial connective tissue boundary. Studies in patients with potentially malignant lesions will further test the clinical benefits of OCT.

Institution

(Hearnden, Murdoch, Thornhill, Hunter, Colley) University of Sheffield, School of Clinical Dentistry, Sheffield, United Kingdom (Rehman, MacNeil) University of Sheffield, Materials Science and Engineering, Sheffield, United Kingdom

Publisher

Elsevier Ltd

Emtree Heading

*optical coherence tomography; *diagnosis; *mouth cancer; *cancer research; *Raman spectrometry; human; tissues; model; patient; connective tissue; technology; mucosa; normal human; tissue section; mouth mucosa; neoplasm; cell line; diagnostic accuracy; microscope; ex vivo study; in vitro study; prognosis; minor saliva gland; tissue structure; ophthalmology; imaging; architecture; chemical bond; mouth; blood vessel; devices; volunteer; biopsy.

Other Index Terms

*optical coherence tomography; *diagnosis; *mouth cancer; *cancer research; *Raman spectrometry; human; tissues; model; patient; connective tissue; technology; mucosa; normal human; tissue section; mouth mucosa; neoplasm; cell line; diagnostic accuracy; microscope; ex vivo study; in vitro study; prognosis; minor saliva gland; tissue structure; ophthalmology; imaging; architecture; chemical bond; mouth; blood vessel; devices; volunteer; biopsy.

Link to the Ovid Full Text or citation:

[Click here for full text options](https://ovidsp.ovid.com/ovidweb.cgi?T=JS&CSC=Y&NEWS=N&PAGE=fulltext&D=emed13&AN=70820904)

Link to the External Link Resolver:

[SFX](https://sfx-86scu.hosted.exlibrisgroup.com.cn/86scu?sid=OVID:embase&id=pmid:&id=doi:10.1016%2FS0959-8049%252812%252971785-9&issn=09598049&isbn=&volume=48&issue=SUPPL.+5&spage=S287&pages=S287&date=2012&title=European+Journal+of+Cancer&atitle=Investigation+of+raman+spectroscopy+and+optical+coherence+tomography+to+aid+in+diagnosis+of+oral+cancer&aulast=Hearnden&pid=<author>Hearnden+V.%3BRehman+I.U.%3BMacNeil+S.%3BMurdoch+C.%3BThornhill+M.H.%3BHunter+K.%3BColley+H.E.<%2Fauthor><AN>70820904<%2FAN><DT>Conference+Abstract<%2FDT>)

316.

Future role of Raman spectroscopy in head and neck oncologic surgery and follow-up.

Cals F., Schut T.B., Koljenovic S., Hardillo J., Puppels G., De Jong R.B.

European Archives of Oto-Rhino-Laryngology. Conference: 5th European Conference on Head and Neck Oncology. Poznan Poland. Conference Publication: (var.pagings). 269(4) (pp 1324), 2012. Date of Publication: April 2012.

AN: 70812945

Background: The high morbidity and mortality associated with head and neck squamous cell carcinoma (HNSCC) are due mainly to the mutilating character of treatment and the development of recurrences over time. The problems in clinical practice are the lack of reliable techniques to: (1) ensure adequate tumor-free margins during surgery to minimize the risk of recurrences, and (2) identify early recurrences during follow-up. Current techniques are subjective depending on the experience of the surgeon, and are time-consuming. A new technique is, therefore, desired that can provide objective real-time information on tissue pathology. Raman spectroscopy is a non-invasive optical technique which yields information about the molecular composition of tissues. Because the technique needs no tissue preparation, it can be used in vivo as well as ex vivo. Our objective is to identify the molecular composition of normal, irradiated and malignant mucosa of the oral cavity by means of Raman spectroscopy to lay the groundwork for the development of ex vivo and in vivo Raman spectroscopic tools for applications in surgery and follow-up monitoring of HNSCC.

Method(s): Samples of normal and malignant mucosa of the oral cavity were collected. Frozen tissue sections of these samples were scanned with micrometer spatial resolution. After the Raman measurements, the frozen sections were routinely stained with haematoxylin-eosine (HE). Using cluster analysis the Raman measurements were converted in pseudocolor images (so-called Raman maps) in which the contrast is based on differences in local molecular composition, as reflected in the Raman spectra. The structures found in the Raman maps were subsequently correlated with the tissue structures identified in HE-stained sections.

Result(s): By scanning large areas with high resolution steps, an accurate correlation of Raman spectra with histopathological structures was possible, resulting in a library of tissue-specific spectra; including normal squamous cell epithelium, stroma, fat, muscle, seromucinous gland, mucus and squamous cell carcinoma.

Conclusion(s): Our first in vitro results show that Raman spectroscopy can be used to differentiate between several tissue structures in the oral cavity, more specifically between normal and malignant mucosa. This is the first step towards ex vivo as well as in vivo clinical applications of Raman spectroscopy.

Institution

(Cals, Hardillo, De Jong) Department of Otorhinolaryngology and Head and Neck Surgery, Erasmus Medical Center, Rotterdam, Netherlands (Schut, Puppels) Department of Dermatology, Center of Optical Diagnostics and Therapy, Erasmus Medical Center, Rotterdam, Netherlands

(Koljenovic) Department of Pathology, Erasmus Medical Center, Rotterdam, Netherlands

Publisher

Springer Verlag

Emtree Heading

*neck; *surgery; *oncology; *follow up; *Raman spectrometry; mouth cavity; tissues; mucosa; ex vivo study; tissue structure; human; monitoring; head and neck squamous cell carcinoma; optics; pathology; stroma; mortality; squamous cell carcinoma; tissue preparation; surgeon; risk; tissue section; cluster analysis; tumor; frozen section; clinical practice; library; squamous epithelium; muscle; mucus; morbidity; in vitro study; eosin; hematoxylin.

Drug Index Terms

eosin; hematoxylin.

Other Index Terms

*neck; *surgery; *oncology; *follow up; *Raman spectrometry; mouth cavity; tissues; mucosa; ex vivo study; tissue structure; human; monitoring; head and neck squamous cell carcinoma; optics; pathology; stroma; mortality; squamous cell carcinoma; tissue preparation; surgeon; risk; tissue section; cluster analysis; tumor; frozen section; clinical practice; library; squamous epithelium; muscle; mucus; morbidity; in vitro study.

Link to the Ovid Full Text or citation:

[Click here for full text options](https://ovidsp.ovid.com/ovidweb.cgi?T=JS&CSC=Y&NEWS=N&PAGE=fulltext&D=emed13&AN=70812945)

Link to the External Link Resolver:

[SFX](https://sfx-86scu.hosted.exlibrisgroup.com.cn/86scu?sid=OVID:embase&id=pmid:&id=doi:10.1007%2Fs00405-012-1960-4&issn=09374477&isbn=&volume=269&issue=4&spage=1324&pages=1324&date=2012&title=European+Archives+of+Oto-Rhino-Laryngology&atitle=Future+role+of+Raman+spectroscopy+in+head+and+neck+oncologic+surgery+and+follow-up&aulast=Cals&pid=<author>Cals+F.%3BSchut+T.B.%3BKoljenovic+S.%3BHardillo+J.%3BPuppels+G.%3BDe+Jong+R.B.<%2Fauthor><AN>70812945<%2FAN><DT>Conference+Abstract<%2FDT>)

317.

In vivo Raman spectroscopic identification of premalignant lesions in oral buccal mucosa.

Singh S.P., Deshmukh A., Chaturvedi P., Murali Krishna C.

Journal of biomedical optics. 17(10) (pp 105002), 2012. Date of Publication: Oct 2012.

AN: 366400922

Cancers of oral cavities are one of the most common malignancies in India and other south-Asian countries. Tobacco habits are the main etiological factors for oral cancer. Identification of premalignant lesions is required for improving survival rates related to oral cancer. Optical spectroscopy methods are projected as alternative/adjunct for cancer diagnosis. Earlier studies have demonstrated the feasibility of classifying normal, premalignant, and malignant oral ex-vivo tissues. We intend to evaluate potentials of Raman spectroscopy in detecting premalignant conditions. Spectra were recorded from premalignant patches, contralateral normal (opposite to tumor site), and cancerous sites of subjects with oral cancers and also from age-matched healthy subjects with and without tobacco habits. A total of 861 spectra from 104 subjects were recorded using a fiber-optic probe-coupled HE-785 Raman spectrometer. Spectral differences in the 1200- to 1800-cm-1 region were subjected to unsupervised principal component analysis and supervised linear discriminant analysis followed by validation with leave-one-out and an independent test data set. Results suggest that premalignant conditions can be objectively discriminated with both normal and cancerous sites as well as from healthy controls with and without tobacco habits. Findings of the study further support efficacy of Raman spectroscopic approaches in oral-cancer applications.

PMID

23223996 [<https://www.ncbi.nlm.nih.gov/pubmed/?term=23223996>]

Institution

(Singh) Advanced Center for Treatment Research and Education in Cancer, Chilakapati Laboratory, Kharghar, Navi-Mumbai 410210, India.

Emtree Heading

adult; algorithm; article; case control study; chemistry; human; methodology; middle aged; *mouth mucosa; *mouth tumor/di [Diagnosis]; pathology; *precancer/di [Diagnosis]; principal component analysis; *Raman spectrometry; smokeless tobacco; smoking; *squamous cell carcinoma.

Other Index Terms

adult; algorithm; article; case control study; chemistry; human; methodology; middle aged; *mouth mucosa; *mouth tumor / *diagnosis; pathology; *precancer / *diagnosis; principal component analysis; *Raman spectrometry; smokeless tobacco; smoking; *squamous cell carcinoma.

Link to the Ovid Full Text or citation:

[Click here for full text options](https://ovidsp.ovid.com/ovidweb.cgi?T=JS&CSC=Y&NEWS=N&PAGE=fulltext&D=emed13&AN=366400922)

Link to the External Link Resolver:

[SFX](https://sfx-86scu.hosted.exlibrisgroup.com.cn/86scu?sid=OVID:embase&id=pmid:23223996&id=doi:&issn=15602281&isbn=&volume=17&issue=10&spage=105002&pages=105002&date=2012&title=Journal+of+biomedical+optics&atitle=In+vivo+Raman+spectroscopic+identification+of+premalignant+lesions+in+oral+buccal+mucosa&aulast=Singh&pid=<author>Singh+S.P.%3BDeshmukh+A.%3BChaturvedi+P.%3BMurali+Krishna+C.<%2Fauthor><AN>366400922<%2FAN><DT>Article<%2FDT>)

318.

Conformational landscape and hydrogen bonding in (S)-(-)-perillyc acid: Experimental VCD, IR, Raman, and theoretical DFT studies.

Moreno J.R.A., Urena F.P., Gonzalez J.J.L.

Tetrahedron Asymmetry. 23(10) (pp 780-788), 2012. Date of Publication: 31 May 2012.

AN: 52066094

(S)-(-)-Perillyc acid (4-isopropenylcyclohexene-1-carboxylic acid) is an intermediate in the limonene and pinene pathway degradation and its measurement in urine is used to monitor cancer patients receiving oral limonene. For the first time, a theoretical study of the conformational preference in the monomer and H-bonded dimers complemented with a theoretical and experimental analysis of the infrared, raman, and vibrational vircular dichroism spectra of (S)-(-)-perillyc acid in solution and solid phases is presented. With regard to the monomer, theoretical calculations revealed the existence of two conformers depending on the position of the isopropenyl group (axial and equatorial) and 24 rotamers (12 equatorials and 12 axials). The study of the H-bonded dimers revealed great complexity in the conformational landscape with a total of 36 structures studied. Herein, from a reliable assignment of the IR and Raman spectra and with help from the study of the VCD spectrum of the title compound, the most stable rotamers of the H-bonded complexes have been detected experimentally in the liquid and solid phases. Additionally, natural bond orbitals (NBO) analysis indicates an electronic delocalization between the two subunits in the dimer. The IR, Raman, and VCD are helpful and complementary techniques to characterize flexible systems, such as terpenes, which present several conformers and H-bonded aggregates. © 2012 Elsevier Ltd. All rights reserved.

Institution

(Moreno, Urena, Gonzalez) University of Jaen, Department of Physical and Analytical Chemistry, Campus Las Lagunillas, E-23071 Jaen, Spain (Moreno) Grupo de Quimica Fisica Teorica y Experimental FQM173, Facultad de Ciencias Experimentales, Universidad de Jaen, E-23071 Jaen, Spain

Publisher

Elsevier Ltd (Langford Lane, Kidlington, Oxford OX5 1GB, United Kingdom)

Emtree Heading

article; circular dichroism; density functional theory; drug conformation; hydrogen bond; infrared spectroscopy; liquid; priority journal; Raman spectrometry; solid; *carboxylic acid derivative/an [Drug Analysis]; limonene/po [Oral Drug Administration]; pinene; terpene derivative; unclassified drug; *perillyc acid/an [Drug Analysis].

Candidate Terms

*perillyc acid / *drug analysis [drug term]

Drug Index Terms

*carboxylic acid derivative / *drug analysis; limonene / oral drug administration; pinene; terpene derivative; unclassified drug.

Other Index Terms

article; circular dichroism; density functional theory; drug conformation; hydrogen bond; infrared spectroscopy; liquid; priority journal; Raman spectrometry; solid.

Link to the Ovid Full Text or citation:

[Click here for full text options](https://ovidsp.ovid.com/ovidweb.cgi?T=JS&CSC=Y&NEWS=N&PAGE=fulltext&D=emed13&AN=52066094)

Link to the External Link Resolver:

[SFX](https://sfx-86scu.hosted.exlibrisgroup.com.cn/86scu?sid=OVID:embase&id=pmid:&id=doi:10.1016%2Fj.tetasy.2012.05.015&issn=09574166&isbn=&volume=23&issue=10&spage=780&pages=780-788&date=2012&title=Tetrahedron+Asymmetry&atitle=Conformational+landscape+and+hydrogen+bonding+in+(S)-(-)-perillyc+acid%3A+Experimental+VCD%2C+IR%2C+Raman%2C+and+theoretical+DFT+studies&aulast=Moreno&pid=<author>Moreno+J.R.A.%3BUrena+F.P.%3BGonzalez+J.J.L.<%2Fauthor><AN>52066094<%2FAN><DT>Article<%2FDT>)

319.

Immunoassay for LMP1 in nasopharyngeal tissue based on surface-enhanced Raman scattering.

Chen Y., Zheng X., Chen G., He C., Zhu W., Feng S., Xi G., Chen R., Lan F., Zeng H.

International Journal of Nanomedicine. 7 (pp 73-82), 2012. Date of Publication: 2012.

AN: 366162795

Previous studies have shown that Epstein-Barr virus (EBV)-encoded latent membrane protein 1 (LMP1) is closely associated with the occurrence and development of nasopharyngeal carcinoma, and can be used as a tumor marker in screening for the disease. Here we report a new methodology based on highly specific and sensitive surface-enhanced Raman scattering (SERS) technology to detect LMP1 in nasopharyngeal tissue sections directly with no need of tedious procedures as with conventional immunohistochemistry methods.

Method(s): LMP1-functionalized 4-mercaptobenzoic acid (4-MBA)-labeled Au/Ag core-shell bimetallic nanoparticles were prepared first and then applied for analyzing LMP1 in formalinfixed paraffin-embedded nasopharyngeal tissue sections obtained from 34 cancer patients and 20 healthy controls. SERS spectra were acquired from a 25 x 25 spot square area on each tissue section and used to generate SERS images.

Result(s): Data from SERS spectra and images show that this new SERS-based immunoassay detected LMP1 in formalin-fixed paraffin-embedded nasopharyngeal tissue sections with high sensitivity and specificity. The results from the new LMP1-SERS probe method are superior to those of conventional immunohistochemistry staining for LMP1, and in excellent agreement with those of in situ hybridization for EBV-encoded small RNA (EBER).

Conclusion(s): This new SERS technique has the potential to be developed into a new clinical tool for detection and differential diagnosis of nasopharyngeal carcinoma as well as for predicting metastasis and immune-targeted treatment of nasopharyngeal carcinoma. © 2012 Chen et al, publisher and licensee Dove Medical Press Ltd.

Institution

(Chen, Zheng, Chen, He, Zhu) Pathology Department of Fujian, Provincial Tumor Hospital, Teaching Hospital of Fujian Medical University, China (Feng, Xi, Chen) Key Laboratory of OptoElectronic Science and Technology for Medicine, Ministry of Education, Fujian Normal University, China

(Lan) Research Center for Molecular Diagnosis of Genetic Diseases, Fuzhou General Hospital, Clinical College of Fujian Medical University, Fuzhou, Fujian 350025, China

(Zeng) Imaging Unit, Integrative Oncology Department, British Columbia Cancer Agency Research Centre, Vancouver, Canada

Publisher

DOVE Medical Press Ltd. (PO Box 300-008, Albany, Auckland, New Zealand)

Emtree Heading

article; clinical article; controlled study; Epstein Barr virus; human; human tissue; *immunoassay; immunohistochemistry; in situ hybridization; molecular imaging; molecular probe; nasopharynx; nasopharynx carcinoma; protein determination; *Raman spectrometry; sensitivity and specificity; tissue section; formaldehyde; gold nanoparticle; *latent membrane protein 1; paraffin; silver nanoparticle; thiolacid; unclassified drug; virus RNA; mercaptobenzoic acid.

Candidate Terms

mercaptobenzoic acid [drug term]

Drug Index Terms

formaldehyde; gold nanoparticle; *latent membrane protein 1; paraffin; silver nanoparticle; thiolacid; unclassified drug; virus RNA.

Other Index Terms

article; clinical article; controlled study; Epstein Barr virus; human; human tissue; *immunoassay; immunohistochemistry; in situ hybridization; molecular imaging; molecular probe; nasopharynx; nasopharynx carcinoma; protein determination; *Raman spectrometry; sensitivity and specificity; tissue section.

Link to the Ovid Full Text or citation:

[Click here for full text options](https://ovidsp.ovid.com/ovidweb.cgi?T=JS&CSC=Y&NEWS=N&PAGE=fulltext&D=emed13&AN=366162795)

Link to the External Link Resolver:

[SFX](https://sfx-86scu.hosted.exlibrisgroup.com.cn/86scu?sid=OVID:embase&id=pmid:&id=doi:10.2147%2FIJN.S26854&issn=11769114&isbn=&volume=7&issue=&spage=73&pages=73-82&date=2012&title=International+Journal+of+Nanomedicine&atitle=Immunoassay+for+LMP1+in+nasopharyngeal+tissue+based+on+surface-enhanced+Raman+scattering&aulast=Chen&pid=<author>Chen+Y.%3BZheng+X.%3BChen+G.%3BHe+C.%3BZhu+W.%3BFeng+S.%3BXi+G.%3BChen+R.%3BLan+F.%3BZeng+H.<%2Fauthor><AN>366162795<%2FAN><DT>Article<%2FDT>)

320.

Salivary biomarker development using genomic, proteomic and metabolomic approaches.

Bonne N.J., Wong D.T.W.

Genome Medicine. 4(10) (no pagination), 2012. Article Number: 82. Date of Publication: 30 Oct 2012.

AN: 52282498

The use of saliva as a diagnostic sample provides a non-invasive, cost-efficient method of sample collection for disease screening without the need for highly trained professionals. Saliva collection is far more practical and safe compared with invasive methods of sample collection, because of the infection risk from contaminated needles during, for example, blood sampling. Furthermore, the use of saliva could increase the availability of accurate diagnostics for remote and impoverished regions. However, the development of salivary diagnostics has required technical innovation to allow stabilization and detection of analytes in the complex molecular mixture that is saliva. The recent development of cost-effective room temperature analyte stabilization methods, nucleic acid pre-amplification techniques and direct saliva transcriptomic analysis have allowed accurate detection and quantification of transcripts found in saliva. Novel protein stabilization methods have also facilitated improved proteomic analyses. Although candidate biomarkers have been discovered using epigenetic, transcriptomic, proteomic and metabolomic approaches, transcriptomic analyses have so far achieved the most progress in terms of sensitivity and specificity, and progress towards clinical implementation. Here, we review recent developments in salivary diagnostics that have been accomplished using genomic, transcriptomic, proteomic and metabolomic approaches. © 2012 BioMed Central Ltd.

Institution

(Bonne, Wong) School of Dentistry and Dental Research Institute, University of California Los Angeles, 650 Charles Young Drive, CHS 73-032, Los Angeles, CA, United States

Publisher

BioMed Central Ltd. (Floor 6, 236 Gray's Inn Road, London WC1X 8HB, United Kingdom)

Emtree Heading

blood sampling; breast cancer/di [Diagnosis]; cancer diagnosis; capillary electrophoresis; cost effectiveness analysis; diagnostic accuracy; diagnostic imaging; diagnostic value; DNA DNA hybridization; DNA methylation; enzyme linked immunosorbent assay; epigenetics; Epstein Barr virus; exosome; genetic marker; *genomics; genotype; head and neck squamous cell carcinoma/di [Diagnosis]; hepatitis C/di [Diagnosis]; immunoblotting; infection risk; leukoplakia/di [Diagnosis]; liquid chromatography; lung cancer/di [Diagnosis]; metabolome; *metabolomics; molecular stability; mouth squamous cell carcinoma/di [Diagnosis]; nasopharynx carcinoma/di [Diagnosis]; needle; non invasive measurement; nucleic acid amplification; pancreas cancer/di [Diagnosis]; pancreatitis/di [Diagnosis]; periodontal disease/di [Diagnosis]; periodontitis/di [Diagnosis]; physical examination; polymerase chain reaction; Porphyromonas gingivalis; precancer/di [Diagnosis]; priority journal; *proteomics; quadrupole mass spectrometry; quantitative analysis; Raman spectrometry; reverse transcription polymerase chain reaction; reversed phase liquid chromatography; review; room temperature; saliva; *saliva analysis; screening; sensitivity and specificity; surface enhanced laser desorption ionization time of flight mass spectrometry; tandem mass spectrometry; time of flight mass spectrometry; transcriptomics; tumor suppressor gene; two dimensional difference gel electrophoresis; ultra performance liquid chromatography; virus carcinogenesis/di [Diagnosis]; alanine aminotransferase/ec [Endogenous Compound]; alpha 2 macroglobulin/ec [Endogenous Compound]; azurocidin/ec [Endogenous Compound]; B Raf kinase/ec [Endogenous Compound]; *biological marker/ec [Endogenous Compound]; carcinoembryonic antigen/ec [Endogenous Compound]; cyclin I/ec [Endogenous Compound]; DNA/ec [Endogenous Compound]; endothelin receptor/ec [Endogenous Compound]; epidermal growth factor/ec [Endogenous Compound]; epidermal growth factor receptor 2/ec [Endogenous Compound]; fibroblast growth factor receptor 2/ec [Endogenous Compound]; glyceraldehyde 3 phosphate dehydrogenase/ec [Endogenous Compound]; interleukin 17/ec [Endogenous Compound]; interleukin 18/ec [Endogenous Compound]; interleukin 1beta/ec [Endogenous Compound]; interleukin 6/ec [Endogenous Compound]; interleukin 8/ec [Endogenous Compound]; macrophage inflammatory protein 1alpha/ec [Endogenous Compound]; messenger RNA/ec [Endogenous Compound]; microRNA/ec [Endogenous Compound]; neutrophil collagenase/ec [Endogenous Compound]; osteoprotegerin/ec [Endogenous Compound]; protein/ec [Endogenous Compound]; proteome/ec [Endogenous Compound]; serum amyloid A/ec [Endogenous Compound]; transcriptome/ec [Endogenous Compound]; transferrin/ec [Endogenous Compound]; tumor suppressor protein/ec [Endogenous Compound]; vasculotropin/ec [Endogenous Compound]; isobaric tag for relative and absolute quantitation.

Candidate Terms

isobaric tag for relative and absolute quantitation [other term]

Drug Index Terms

alanine aminotransferase / endogenous compound; alpha 2 macroglobulin / endogenous compound; azurocidin / endogenous compound; B Raf kinase / endogenous compound; *biological marker / *endogenous compound; carcinoembryonic antigen / endogenous compound; cyclin I / endogenous compound; DNA / endogenous compound; endothelin receptor / endogenous compound; epidermal growth factor / endogenous compound; epidermal growth factor receptor 2 / endogenous compound; fibroblast growth factor receptor 2 / endogenous compound; glyceraldehyde 3 phosphate dehydrogenase / endogenous compound; interleukin 17 / endogenous compound; interleukin 18 / endogenous compound; interleukin 1beta / endogenous compound; interleukin 6 / endogenous compound; interleukin 8 / endogenous compound; macrophage inflammatory protein 1alpha / endogenous compound; messenger RNA / endogenous compound; microRNA / endogenous compound; neutrophil collagenase / endogenous compound; osteoprotegerin / endogenous compound; protein / endogenous compound; proteome / endogenous compound; serum amyloid A / endogenous compound; transcriptome / endogenous compound; transferrin / endogenous compound; tumor suppressor protein / endogenous compound; vasculotropin / endogenous compound.

Other Index Terms

blood sampling; breast cancer / diagnosis; cancer diagnosis; capillary electrophoresis; cost effectiveness analysis; diagnostic accuracy; diagnostic imaging; diagnostic value; DNA DNA hybridization; DNA methylation; enzyme linked immunosorbent assay; epigenetics; Epstein Barr virus; exosome; genetic marker; *genomics; genotype; head and neck squamous cell carcinoma / diagnosis; hepatitis C / diagnosis; immunoblotting; infection risk; leukoplakia / diagnosis; liquid chromatography; lung cancer / diagnosis; metabolome; *metabolomics; molecular stability; mouth squamous cell carcinoma / diagnosis; nasopharynx carcinoma / diagnosis; needle; non invasive measurement; nucleic acid amplification; pancreas cancer / diagnosis; pancreatitis / diagnosis; periodontal disease / diagnosis; periodontitis / diagnosis; physical examination; polymerase chain reaction; Porphyromonas gingivalis; precancer / diagnosis; priority journal; *proteomics; quadrupole mass spectrometry; quantitative analysis; Raman spectrometry; reverse transcription polymerase chain reaction; reversed phase liquid chromatography; review; room temperature; saliva; *saliva analysis; screening; sensitivity and specificity; surface enhanced laser desorption ionization time of flight mass spectrometry; tandem mass spectrometry; time of flight mass spectrometry; transcriptomics; tumor suppressor gene; two dimensional difference gel electrophoresis; ultra performance liquid chromatography; virus carcinogenesis / diagnosis.

Link to the Ovid Full Text or citation:

[Click here for full text options](https://ovidsp.ovid.com/ovidweb.cgi?T=JS&CSC=Y&NEWS=N&PAGE=fulltext&D=emed13&AN=52282498)

Link to the External Link Resolver:

[SFX](https://sfx-86scu.hosted.exlibrisgroup.com.cn/86scu?sid=OVID:embase&id=pmid:&id=doi:10.1186%2Fgm383&issn=1756994X&isbn=&volume=4&issue=10&spage=82&pages=&date=2012&title=Genome+Medicine&atitle=Salivary+biomarker+development+using+genomic%2C+proteomic+and+metabolomic+approaches&aulast=Bonne&pid=<author>Bonne+N.J.%3BWong+D.T.W.<%2Fauthor><AN>52282498<%2FAN><DT>Review<%2FDT>)

321.

Detection of nasopharyngeal cancer using confocal Raman spectroscopy and genetic algorithm technique.

Li S.X., Chen Q.Y., Zhang Y.J., Liu Z.M., Xiong H.L., Guo Z.Y., Mai H.Q., Liu S.H.

Journal of biomedical optics. 17(12) (pp 125003), 2012. Date of Publication: Dec 2012.

AN: 366409533

Raman spectroscopy (RS) and a genetic algorithm (GA) were applied to distinguish nasopharyngeal cancer (NPC) from normal nasopharyngeal tissue. A total of 225 Raman spectra are acquired from 120 tissue sites of 63 nasopharyngeal patients, 56 Raman spectra from normal tissue and 169 Raman spectra from NPC tissue. The GA integrated with linear discriminant analysis (LDA) is developed to differentiate NPC and normal tissue according to spectral variables in the selected regions of 792-805, 867-880, 996-1009, 1086-1099, 1288-1304, 1663-1670, and 1742-1752 cm-1 related to proteins, nucleic acids and lipids of tissue. The GA-LDA algorithms with the leave-one-out cross-validation method provide a sensitivity of 69.2% and specificity of 100%. The results are better than that of principal component analysis which is applied to the same Raman dataset of nasopharyngeal tissue with a sensitivity of 63.3% and specificity of 94.6%. This demonstrates that Raman spectroscopy associated with GA-LDA diagnostic algorithm has enormous potential to detect and diagnose nasopharyngeal cancer.

PMID

23208211 [<https://www.ncbi.nlm.nih.gov/pubmed/?term=23208211>]

Institution

(Li) South China Normal University, School of Information and Optoelectronic Science and Engineering, Guangzhou 510631, China.

Emtree Heading

adult; *algorithm; article; biological model; *computer assisted diagnosis; *confocal microscopy; female; human; male; methodology; *nasopharynx tumor/di [Diagnosis]; *Raman spectrometry; reproducibility; sensitivity and specificity.

Other Index Terms

adult; *algorithm; article; biological model; *computer assisted diagnosis; *confocal microscopy; female; human; male; methodology; *nasopharynx tumor / *diagnosis; *Raman spectrometry; reproducibility; sensitivity and specificity.

Link to the Ovid Full Text or citation:

[Click here for full text options](https://ovidsp.ovid.com/ovidweb.cgi?T=JS&CSC=Y&NEWS=N&PAGE=fulltext&D=emed13&AN=366409533)

Link to the External Link Resolver:

[SFX](https://sfx-86scu.hosted.exlibrisgroup.com.cn/86scu?sid=OVID:embase&id=pmid:23208211&id=doi:&issn=15602281&isbn=&volume=17&issue=12&spage=125003&pages=125003&date=2012&title=Journal+of+biomedical+optics&atitle=Detection+of+nasopharyngeal+cancer+using+confocal+Raman+spectroscopy+and+genetic+algorithm+technique&aulast=Li&pid=<author>Li+S.X.%3BChen+Q.Y.%3BZhang+Y.J.%3BLiu+Z.M.%3BXiong+H.L.%3BGuo+Z.Y.%3BMai+H.Q.%3BLiu+S.H.<%2Fauthor><AN>366409533<%2FAN><DT>Article<%2FDT>)

322.

The future of medical diagnostics: Review paper.

Jerjes W.K., Upile T., Wong B.J., Betz C.S., Sterenborg H.J., Witjes M.J., Berg K., Van Veen R., Biel M.A., El-Naggar A.K., Mosse C.A., Olivo M., Richards-Kortum R., Robinson D.J., Rosen J., Yodh A.G., Kendall C., Ilgner J.F., Amelink A., Bagnato V., Barr H., Bolotine L., Bigio I., Chen Z., Choo-Smith L.-P., D'Cruz A.K., Gillenwater A., Leunig A., MacRobert A.J., McKenzie G., Sandison A., Soo K.C., Stepp H., Stone N., Svanberg K., Tan I.B., Wilson B.C., Wolfsen H., Hopper C.

Head and Neck Oncology. 3(1) (no pagination), 2011. Article Number: 38. Date of Publication: 2011.

AN: 51596642

While histopathology of excised tissue remains the gold standard for diagnosis, several new, non-invasive diagnostic techniques are being developed. They rely on physical and biochemical changes that precede and mirror malignant change within tissue. The basic principle involves simple optical techniques of tissue interrogation. Their accuracy, expressed as sensitivity and specificity, are reported in a number of studies suggests that they have a potential for cost effective, real-time, in situ diagnosis. We review the Third Scientific Meeting of the Head and Neck Optical Diagnostics Society held in Congress Innsbruck, Innsbruck, Austria on the 11th May 2011. For the first time the HNODS Annual Scientific Meeting was held in association with the International Photodynamic Association (IPA) and the European Platform for Photodynamic Medicine (EPPM). The aim was to enhance the interdisciplinary aspects of optical diagnostics and other photodynamic applications. The meeting included 2 sections: oral communication sessions running in parallel to the IPA programme and poster presentation sessions combined with the IPA and EPPM posters sessions. © 2011 Jerjes et al; licensee BioMed Central Ltd.

PMID

21861912 [<https://www.ncbi.nlm.nih.gov/pubmed/?term=21861912>]

Institution

(Jerjes, Upile, Wong, Betz, Sterenborg, Witjes, Berg, Van Veen, Biel, El-Naggar, Mosse, Olivo, Richards-Kortum, Robinson, Yodh, Kendall, Amelink, Bagnato, Barr, Bolotine, Bigio, Chen, Choo-Smith, D'Cruz, Gillenwater, Leunig, MacRobert, McKenzie, Sandison, Soo, Stepp, Stone, Svanberg, Tan, Wilson, Wolfsen, Hopper) Head and Neck Optical Diagnostics Society Council, International Society of Minimally Invasive Diagnostics, University College London, London, United Kingdom (Jerjes) Department of Trauma and Orthopaedic Surgery, Leeds Teaching Hospitals NHS Trust, Leeds, United Kingdom

(Jerjes) Academic Department of Trauma and Orthopaedic Surgery, School of Medicine, University of Leeds, Leeds, United Kingdom

(Jerjes, Hopper) UCL Department of Surgery, Division of Surgery and Interventional Science, London, United Kingdom

(Upile) Department of Otolaryngology/Head and Neck Surgery, Barnet and Chase Farm Hospitals NHS Trust, London, United Kingdom

(Upile, McKenzie, Hopper) UCLH Head and Neck Centre, University College London Hospitals, London, United Kingdom

(Wong) Beckman Laser Institute and Medical Clinic, University of California Irvine, Irvine, CA, United States

(Betz, Leunig) Department of Otorhinolaryngology, Head and Neck Surgery, Ludwig Maximilian University, Munich, Germany

(Sterenborg, Van Veen, Amelink) Center for Optical Diagnostics and Therapy, Erasmus University Medical Center, Rotterdam, Netherlands

(Witjes) Department of Oral and Maxillofacial Surgery, University Medical Center Groningen, Groningen, Netherlands

(Berg) Dept. of Radiation Biology, Norwegian Radium Hospital, Montebello, Norway

(Biel) Virginia Piper Cancer Institute, Abbott Northwestern Hospital, MN, United States

(El-Naggar) Department of Pathology, University of Texas, M.D. Anderson Cancer Center, Houston, TX, United States

(Mosse, MacRobert) National Medical Laser Centre, University College London, Department of Surgery, London, United Kingdom

(Olivo) School of Physics, National University of Ireland, Galway, Ireland

(Richards-Kortum) Department of Bioengineering, Rice University, Houston, United States

(Robinson) Department of Radiation Oncology, Erasmus University Medical Center, Rotterdam, Netherlands

(Rosen) Department of Surgery, Boston University, School of Medicine, Boston, United States

(Yodh) Physics and Astronomy Department, University of Pennsylvania, Philadelphia, United States

(Kendall, Barr, Stone) Biophotonics Research Group, Gloucestershire Hospitals NHS Foundation Trust, Gloucester, United Kingdom

(Ilgner) Department of Otorhinolaryngology, Plastic Head and Neck Surgery, Aachen University Hospital, RWTH Aachen, Germany

(Bagnato) Centro de Pesquisa em Ptica e Fotonica, University of Sao Paulo, Sao Carlos, SP, Brazil

(Bolotine) Research Centre for Automatic Control (CRAN), Nancy-University, UMR CNRS, France

(Bigio) Department of Biomedical Engineering, Electrical and Computer Engineering, Physics, Boston University, Boston, United States

(Chen) Department of Biomedical Engineering, Beckman Laser Institute, University of California, Irvine, United States

(Choo-Smith) National Research Council Canada-Institute for Biodiagnostics, Winnipeg, MB, Canada

(D'Cruz) Department of Oral and Maxillofacial Surgery, Tata Memorial Hospital, Mumbai, India

(Gillenwater) Department of Head and Neck Surgery, M. D. Anderson Cancer Center, University of Texas, Houston, TX, United States

(McKenzie) Michelson Diagnostics, Orpington, Kent, United Kingdom

(Sandison) Department of Histopathology, Imperial College, Hammersmith Hospitals, London, United Kingdom

(Soo) National Cancer Centre, Singapore, Singapore

(Stepp) LIFE Center, University Clinic Munich, Munich, Germany

(Svanberg) Division of Oncology, Lund University Hospital, Lund, Sweden

(Tan) Department of Head and Neck Oncology and Surgery, Netherlands Cancer Institute, Antoni Van Leeuwenhoek Hospital, Amsterdam, Netherlands

(Wilson) Division of BioPhysics and BioImaging, Ontario Cancer Institute, ON, Canada

(Wilson, Wolfsen) Department of Medical Biophysics, Faculty of Medicine, University of Toronto, Toronto, Canada

(Wolfsen) Division of Gastroenterology and Hepatology, Mayo Clinic, FL, United States

Publisher

OA Publishing London (Second Floor, 10-12 Maclise Road, London, England W140PR, United States)

Emtree Heading

Austria; biochemistry; biopsy technique; confocal microscopy; diagnostic accuracy; diagnostic procedure; endoscopy; fluorescence analysis; gold standard; *head and neck cancer/di [Diagnosis]; histopathology; human; malignant transformation; medical society; near infrared spectroscopy; non invasive measurement; optical coherence tomography; photodynamics; physical examination; priority journal; Raman spectrometry; review; sensitivity and specificity; spectroscopy; confocal reflectance microscopy; deferential path length spectroscopy; elastic scattering spectroscopy; microendoscopy; optical biopsy.

Candidate Terms

confocal reflectance microscopy [other term] deferential path length spectroscopy [other term]

elastic scattering spectroscopy [other term]

microendoscopy [other term]

optical biopsy [other term]

Other Index Terms

Austria; biochemistry; biopsy technique; confocal microscopy; diagnostic accuracy; diagnostic procedure; endoscopy; fluorescence analysis; gold standard; *head and neck cancer / *diagnosis; histopathology; human; malignant transformation; medical society; near infrared spectroscopy; non invasive measurement; optical coherence tomography; photodynamics; physical examination; priority journal; Raman spectrometry; review; sensitivity and specificity; spectroscopy.

Link to the Ovid Full Text or citation:

[Click here for full text options](https://ovidsp.ovid.com/ovidweb.cgi?T=JS&CSC=Y&NEWS=N&PAGE=fulltext&D=emed12&AN=51596642)

Link to the External Link Resolver:

[SFX](https://sfx-86scu.hosted.exlibrisgroup.com.cn/86scu?sid=OVID:embase&id=pmid:21861912&id=doi:10.1186%2F1758-3284-3-38&issn=17583284&isbn=&volume=3&issue=1&spage=38&pages=&date=2011&title=Head+and+Neck+Oncology&atitle=The+future+of+medical+diagnostics%3A+Review+paper&aulast=Jerjes&pid=<author>Jerjes+W.K.%3BUpile+T.%3BWong+B.J.%3BBetz+C.S.%3BSterenborg+H.J.%3BWitjes+M.J.%3BBerg+K.%3BVan+Veen+R.%3BBiel+M.A.%3BEl-Naggar+A.K.%3BMosse+C.A.%3BOlivo+M.%3BRichards-Kortum+R.%3BRobinson+D.J.%3BRosen+J.%3BYodh+A.G.%3BKendall+C.%3BIlgner+J.F.%3BAmelink+A.%3BBagnato+V.%3BBarr+H.%3BBolotine+L.%3BBigio+I.%3BChen+Z.%3BChoo-Smith+L.-P.%3BD'Cruz+A.K.%3BGillenwater+A.%3BLeunig+A.%3BMacRobert+A.J.%3BMcKenzie+G.%3BSandison+A.%3BSoo+K.C.%3BStepp+H.%3BStone+N.%3BSvanberg+K.%3BTan+I.B.%3BWilson+B.C.%3BWolfsen+H.%3BHopper+C.<%2Fauthor><AN>51596642<%2FAN><DT>Review<%2FDT>)

323.

Raman spectroscopy of normal oral buccal mucosa tissues: study on intact and incised biopsies.

Deshmukh A., Singh S.P., Chaturvedi P., Krishna C.M.

Journal of biomedical optics. 16(12) (pp 127004), 2011. Date of Publication: Dec 2011.

AN: 560066938

Oral squamous cell carcinoma is one of among the top 10 malignancies. Optical spectroscopy, including Raman, is being actively pursued as alternative/adjunct for cancer diagnosis. Earlier studies have demonstrated the feasibility of classifying normal, premalignant, and malignant oral ex vivo tissues. Spectral features showed predominance of lipids and proteins in normal and cancer conditions, respectively, which were attributed to membrane lipids and surface proteins. In view of recent developments in deep tissue Raman spectroscopy, we have recorded Raman spectra from superior and inferior surfaces of 10 normal oral tissues on intact, as well as incised, biopsies after separation of epithelium from connective tissue. Spectral variations and similarities among different groups were explored by unsupervised (principal component analysis) and supervised (linear discriminant analysis, factorial discriminant analysis) methodologies. Clusters of spectra from superior and inferior surfaces of intact tissues show a high overlap; whereas spectra from separated epithelium and connective tissue sections yielded clear clusters, though they also overlap on clusters of intact tissues. Spectra of all four groups of normal tissues gave exclusive clusters when tested against malignant spectra. Thus, this study demonstrates that spectra recorded from the superior surface of an intact tissue may have contributions from deeper layers but has no bearing from the classification of a malignant tissues point of view.

PMID

22191934 [<https://www.ncbi.nlm.nih.gov/pubmed/?term=22191934>]

Institution

(Deshmukh) Chilakapati Laboratory, ACTREC, Kharghar, Navi-Mumbai, India.

Emtree Heading

article; biopsy; chemistry; connective tissue; *cytochemistry; cytology; discriminant analysis; epithelium; human; methodology; *mouth mucosa; mouth tumor; pathology; principal component analysis; *Raman spectrometry; signal processing.

Other Index Terms

article; biopsy; chemistry; connective tissue; *cytochemistry; cytology; discriminant analysis; epithelium; human; methodology; *mouth mucosa; mouth tumor; pathology; principal component analysis; *Raman spectrometry; signal processing.

Link to the Ovid Full Text or citation:

[Click here for full text options](https://ovidsp.ovid.com/ovidweb.cgi?T=JS&CSC=Y&NEWS=N&PAGE=fulltext&D=emed12&AN=560066938)

Link to the External Link Resolver:

[SFX](https://sfx-86scu.hosted.exlibrisgroup.com.cn/86scu?sid=OVID:embase&id=pmid:22191934&id=doi:&issn=15602281&isbn=&volume=16&issue=12&spage=127004&pages=127004&date=2011&title=Journal+of+biomedical+optics&atitle=Raman+spectroscopy+of+normal+oral+buccal+mucosa+tissues%3A+study+on+intact+and+incised+biopsies&aulast=Deshmukh&pid=<author>Deshmukh+A.%3BSingh+S.P.%3BChaturvedi+P.%3BKrishna+C.M.<%2Fauthor><AN>560066938<%2FAN><DT>Article<%2FDT>)

324.

PET imaging of integrin alphavbeta3 expression.

Beer A.J., Kessler H., Wester H.-J., Schwaiger M.

Theranostics. 1(1) (pp 48-57), 2011. Date of Publication: 2011.

AN: 364269444

PET imaging of integrin alphavbeta3 expression has been studied intensely by the academia and recently also by the industry. Imaging of integrin alphavbeta3 expression is of great potential value, as the integrin alphavbeta3 is a key player in tumor metastasis and angiogenesis. Therefore PET imaging of this target might be a suitable in-vivo biomarker of angiogenesis and metastatic potential of tumors. In this manuscript, the various strategies for PET imaging of the integrin alphavbeta3 will be summarized, including monomeric and multimeric radiolabelled RGD peptides and nanoparticles. While most experiments have been performed using preclinical tumor models, more and more clinical results on PET imaging of alphavbeta3 expression are available and will be discussed in detail. However, while a multitude of radiotracer strategies have been successfully evaluated for PET imaging of alphavbeta3, the ultimate clinical value of this new imaging biomarker still has to be evaluated in large clinical trials. © Ivyspring International Publisher.

Institution

(Beer, Schwaiger) Department of Nuclear Medicine, Klinikum rechts der Isar, Technische Universitat Munchen, Munich, Germany (Kessler) Institute for Advanced Study und Center of Integrated Protein Science, Technische Universitat Munchen, Department Chemie, Garching, Germany

(Wester) Chair for Pharmaceutical Radiochemistry, Garching, Germany

Publisher

Ivyspring International Publisher (Level 32, 1 Market Street, Sedney, New South Wales 2000, Australia)

Emtree Heading

angiogenesis; antiangiogenic therapy; autoradiography; bioaccumulation; biological monitoring; breast adenocarcinoma; cancer model; cancer radiotherapy; carcinogenesis; cell proliferation; computer assisted tomography; contrast enhancement; correlation coefficient; cyclotron; diagnostic value; dosimetry; drug excretion; drug uptake; fluorescence imaging; glucose metabolism; glycosylation; half life time; head and neck cancer; human; hydrophilicity; image analysis; immunohistochemistry; isotope labeling; Lewis carcinoma/dt [Drug Therapy]; liver metastasis; metastasis; microvasculature; *molecular imaging; nonhuman; nuclear magnetic resonance imaging; peptide synthesis; phenotype; polyacrylamide gel electrophoresis; *positron emission tomography; protein analysis; protein expression; protein protein interaction; protein targeting; quantitative analysis; radiation dose; Raman spectrometry; review; sequence analysis; single photon emission computer tomography; squamous cell carcinoma; structure activity relation; treatment response; tumor differentiation; urinary excretion; arginylglycylaspartic acid/pk [Pharmacokinetics]; biological marker; fibrinogen/ec [Endogenous Compound]; fluciclatide f 18/ct [Clinical Trial]; fluciclatide f 18/pk [Pharmacokinetics]; fluorine 18; fluorodeoxyglucose f 18/pk [Pharmacokinetics]; iodine 123; iodine 125; macrogol; maraciclatide tc 99m/pk [Pharmacokinetics]; monoclonal antibody; monomer; nanoparticle; paclitaxel/dt [Drug Therapy]; single walled nanotube; technetium 99m; tetraxetan; tracer; vitronectin/ec [Endogenous Compound]; *vitronectin receptor/ec [Endogenous Compound].

Drug Index Terms

arginylglycylaspartic acid / pharmacokinetics; biological marker; fibrinogen / endogenous compound; fluciclatide f 18 / clinical trial / pharmacokinetics; fluorine 18; fluorodeoxyglucose f 18 / pharmacokinetics; iodine 123; iodine 125; macrogol; maraciclatide tc 99m / pharmacokinetics; monoclonal antibody; monomer; nanoparticle; paclitaxel / drug therapy; single walled nanotube; technetium 99m; tetraxetan; tracer; vitronectin / endogenous compound; *vitronectin receptor / *endogenous compound.

Other Index Terms

angiogenesis; antiangiogenic therapy; autoradiography; bioaccumulation; biological monitoring; breast adenocarcinoma; cancer model; cancer radiotherapy; carcinogenesis; cell proliferation; computer assisted tomography; contrast enhancement; correlation coefficient; cyclotron; diagnostic value; dosimetry; drug excretion; drug uptake; fluorescence imaging; glucose metabolism; glycosylation; half life time; head and neck cancer; human; hydrophilicity; image analysis; immunohistochemistry; isotope labeling; Lewis carcinoma / drug therapy; liver metastasis; metastasis; microvasculature; *molecular imaging; nonhuman; nuclear magnetic resonance imaging; peptide synthesis; phenotype; polyacrylamide gel electrophoresis; *positron emission tomography; protein analysis; protein expression; protein protein interaction; protein targeting; quantitative analysis; radiation dose; Raman spectrometry; review; sequence analysis; single photon emission computer tomography; squamous cell carcinoma; structure activity relation; treatment response; tumor differentiation; urinary excretion.

Link to the Ovid Full Text or citation:

[Click here for full text options](https://ovidsp.ovid.com/ovidweb.cgi?T=JS&CSC=Y&NEWS=N&PAGE=fulltext&D=emed12&AN=364269444)

Link to the External Link Resolver:

[SFX](https://sfx-86scu.hosted.exlibrisgroup.com.cn/86scu?sid=OVID:embase&id=pmid:&id=doi:10.7150%2Fthno%2Fv01p0048&issn=18387640&isbn=&volume=1&issue=1&spage=48&pages=48-57&date=2011&title=Theranostics&atitle=PET+imaging+of+integrin+alphavbeta3+expression&aulast=Beer&pid=<author>Beer+A.J.%3BKessler+H.%3BWester+H.-J.%3BSchwaiger+M.<%2Fauthor><AN>364269444<%2FAN><DT>Review<%2FDT>)

325.

Potential applications of the chemiluminescent methods in tumoral diseases investigation.

Araujo-Filho J.L.S., Melo-Junior M.R., Carvalho L.B.

International Journal of Pharma and Bio Sciences. 2(2) (pp 392-400), 2011. Date of Publication: 2011.

AN: 364051723

Nowadays, in the clinic routine, protein biomarkers of efficacy, or mode of action, are used to better monitor and predict patient responses to drug treatment and diagnosis. Currently, the chemiluminescence biomarkers are applied for validation methods and screening. Here, was revised the development of the new analytical tool through immunological (antibody) and chemiluminescent methods combination, named chemiluminescence immunoassay (CLIA). In the tumoral process, increased expression of specific antigens is associated in patients with certain tumors. Thus, in our laboratories CLIA has been tested to study different tumoral lesions from glandular tissues, like prostate and thyroid. In conclusion, there is a great opportunity for introducing the adaptive chemiluminescence devices, since clinical diagnostics represents a huge, well-established and important analytical field.

Institution

(Araujo-Filho, Melo-Junior, Carvalho) Laboratory of Immunopathology Keizo Asami, Federal University of Pernambuco, Recife, Brazil (Carvalho) Department of Biochemistry, Federal University of Pernambuco, Recife, Brazil

Publisher

International Journal of Pharma and Bio Sciences (103, Bhagwan Towers, 7th Line, Muthyal Reddy Nagar, Amaravathi Road, Guntur 522002, India)

Emtree Heading

antigen expression; carcinomatosis; *chemoluminescence; disease marker; early diagnosis; enzyme linked immunosorbent assay; human; immunoassay; immunohistochemistry; in situ hybridization; intermethod comparison; limit of detection; liver cell carcinoma/di [Diagnosis]; *neoplasm; nonhuman; process development; prostate tumor; protein blood level; Raman spectrometry; review; sensitivity and specificity; thyroid tumor; uterine cervix cancer/di [Diagnosis]; uterine cervix carcinoma in situ/di [Diagnosis]; acridine derivative; alkaline phosphatase; alpha fetoprotein/ec [Endogenous Compound]; carcinoembryonic antigen/ec [Endogenous Compound]; fluorescein isothiocyanate; horseradish peroxidase; luminol; prostate specific antigen/ec [Endogenous Compound]; tumor antigen/ec [Endogenous Compound]; chemiluminescence enzyme immunoassay; chemiluminescence immunoassay.

Candidate Terms

chemiluminescence enzyme immunoassay [other term] chemiluminescence immunoassay [other term]

Drug Index Terms

acridine derivative; alkaline phosphatase; alpha fetoprotein / endogenous compound; carcinoembryonic antigen / endogenous compound; fluorescein isothiocyanate; horseradish peroxidase; luminol; prostate specific antigen / endogenous compound; tumor antigen / endogenous compound.

Other Index Terms

antigen expression; carcinomatosis; *chemoluminescence; disease marker; early diagnosis; enzyme linked immunosorbent assay; human; immunoassay; immunohistochemistry; in situ hybridization; intermethod comparison; limit of detection; liver cell carcinoma / diagnosis; *neoplasm; nonhuman; process development; prostate tumor; protein blood level; Raman spectrometry; review; sensitivity and specificity; thyroid tumor; uterine cervix cancer / diagnosis; uterine cervix carcinoma in situ / diagnosis.

Link to the Ovid Full Text or citation:

[Click here for full text options](https://ovidsp.ovid.com/ovidweb.cgi?T=JS&CSC=Y&NEWS=N&PAGE=fulltext&D=emed12&AN=364051723)

Link to the External Link Resolver:

[SFX](https://sfx-86scu.hosted.exlibrisgroup.com.cn/86scu?sid=OVID:embase&id=pmid:&id=doi:&issn=09756299&isbn=&volume=2&issue=2&spage=392&pages=392-400&date=2011&title=International+Journal+of+Pharma+and+Bio+Sciences&atitle=Potential+applications+of+the+chemiluminescent+methods+in+tumoral+diseases+investigation&aulast=Araujo-Filho&pid=<author>Araujo-Filho+J.L.S.%3BMelo-Junior+M.R.%3BCarvalho+L.B.<%2Fauthor><AN>364051723<%2FAN><DT>Review<%2FDT>)

326.

SERS spectra of a single nasopharyngeal carcinoma cell based on intracellularly grown and passive uptake Au nanoparticles.

Huang H., Chen W., Pan J., Chen Q., Feng S., Yu Y., Chen Y., Su Y., Chen R.

Spectroscopy. 26(3) (pp 187-194), 2011. Date of Publication: 2011.

AN: 364424552

The intracellularly-grown-Au-nanoparticles (IGAuNs) technique was employed to analyze the surface-enhanced Raman scattering (SERS) spectra of nasopharyngeal carcinoma cells (CNE-1 cell line). There are only six obvious Raman bands (718, 1001, 1123, 1336, 1446, 1660 cm-1) in the normal Raman spectrum of living CNE-1 cells. However, over twenty SERS Raman bands have been detected in the SERS spectra of IGAuNs-induced cells, among which five bands are of the DNA backbone (673, 1097, 1306, 1336 and 1585 cm-1). There are four vibrations of the DNA backbone (1026, 1097, 1336 and 1585 cm -1) in the SERS spectra of living CNE-1 cells induced by the passive uptake gold nanoparticles (PUAuNS), but one more DNA backbone and many nucleus Raman peaks appeared in the IGAuNs-induced SERS spectra. Many Raman peaks in the PUAuNs-induced SERS spectra are stronger than those in the IGAuNs-induced ones. This study has shown that the PUAuNs technique can achieve stronger Raman signals, and that the IGAuNs technique can enable the gold element to access to the nucleus more easily, which could help to obtain more surface-enhanced Raman signals of the intracellular biochemical molecules. Thus, the two techniques can work together to attain the Raman spectral information of the cytoplasm and the nucleus in a better way, which might provide a sensitive method for broad biomedical applications such as intracellular SERS analysis of living cells. © 2011-IOS Press and the authors. All rights reserved.

Institution

(Huang, Chen, Yu) College of Integrated Traditional Chinese and Western Medicine, Fujian University of Traditional Chinese Medicine, Fuzhou, China (Chen, Feng, Yu, Chen) Key Laboratory of Optoelectronic Science and Technology for Medicine, Ministry of Education, Fujian Normal University, Fuzhou 350007, China

(Pan, Chen, Su) Fujian Provincial Cancer Hospital, Fuzhou, China

(Chen) Fujian Provincial Tumor Hospital, Fuzhou, China

Publisher

Hindawi Publishing Corporation (410 Park Avenue, 15th Floor, 287 pmb, New York NY 10022, United States)

Emtree Heading

article; *carcinoma cell; cell nucleus; controlled study; human; human cell; microscopy; molecular dynamics; nasopharynx carcinoma; *passive transport; *Raman spectrometry; DNA/ec [Endogenous Compound]; *gold nanoparticle.

Drug Index Terms

DNA / endogenous compound; *gold nanoparticle.

Other Index Terms

article; *carcinoma cell; cell nucleus; controlled study; human; human cell; microscopy; molecular dynamics; nasopharynx carcinoma; *passive transport; *Raman spectrometry.

Link to the Ovid Full Text or citation:

[Click here for full text options](https://ovidsp.ovid.com/ovidweb.cgi?T=JS&CSC=Y&NEWS=N&PAGE=fulltext&D=emed12&AN=364424552)

Link to the External Link Resolver:

[SFX](https://sfx-86scu.hosted.exlibrisgroup.com.cn/86scu?sid=OVID:embase&id=pmid:&id=doi:10.3233%2FSPE-2011-0540&issn=07124813&isbn=&volume=26&issue=3&spage=187&pages=187-194&date=2011&title=Spectroscopy&atitle=SERS+spectra+of+a+single+nasopharyngeal+carcinoma+cell+based+on+intracellularly+grown+and+passive+uptake+Au+nanoparticles&aulast=Huang&pid=<author>Huang+H.%3BChen+W.%3BPan+J.%3BChen+Q.%3BFeng+S.%3BYu+Y.%3BChen+Y.%3BSu+Y.%3BChen+R.<%2Fauthor><AN>364424552<%2FAN><DT>Article<%2FDT>)

327.

Raman spectral statistical classification of nasopharyngeal carcinoma and nasopharyngeal normal cell lines based on support vector classification.

Chen Y., Sun L., Huang Y., Ou L., Su Y.

Spectroscopy. 26(4-5) (pp 231-236), 2011. Date of Publication: 2011.

AN: 364424545

Raman spectroscopy (RS) has been used in the discrimination of normal and tumor cells for years. It is very important to validate an existing classification model using different algorithms. In this work, two algorithms of support vector classification (SVC) are utilized to validate our previous work about a LDA classification model of nasopharyngeal carcinoma (NPC) cell lines C666-1, CNE2 and nasopharyngeal normal cell line NP69. All of these two SVC algorithms use the same data set as the previous LDA model and, achieve great sensitivity and specificity. The final results show that our previous LDA classification model could be supported by different SVC algorithms and this demonstrates our classification model is reliable and may be helpful to the realization of RS to be one of diagnostic techniques of NPC. © 2011 - IOS Press and the authors. All rights reserved.

Institution

(Chen, Sun) Zhicheng College, Fuzhou University, Fuzhou, China (Huang) Key Laboratory of Instrumentation Science, Dynamic Measurement Ministry of Education, North University of China, Taiyuan, China

(Ou) College of Physics and OptoElectronics Technology, Fujian Normal University, Building 7, Shangsan Road, Cangshan District, Fuzhou, China

(Su) Fujian Provincial Tumor Hospital, Fuzhou, China

Publisher

Hindawi Publishing Corporation (410 Park Avenue, 15th Floor, 287 pmb, New York NY 10022, United States)

Emtree Heading

algorithm; article; cancer cell culture; classification; controlled study; diagnostic procedure; discriminant analysis; human; human tissue; *nasopharynx carcinoma; *Raman spectrometry; sensitivity and specificity; *support vector machine.

Other Index Terms

algorithm; article; cancer cell culture; classification; controlled study; diagnostic procedure; discriminant analysis; human; human tissue; *nasopharynx carcinoma; *Raman spectrometry; sensitivity and specificity; *support vector machine.

Link to the Ovid Full Text or citation:

[Click here for full text options](https://ovidsp.ovid.com/ovidweb.cgi?T=JS&CSC=Y&NEWS=N&PAGE=fulltext&D=emed12&AN=364424545)

Link to the External Link Resolver:

[SFX](https://sfx-86scu.hosted.exlibrisgroup.com.cn/86scu?sid=OVID:embase&id=pmid:&id=doi:10.3233%2FSPE-2011-0548&issn=07124813&isbn=&volume=26&issue=4-5&spage=231&pages=231-236&date=2011&title=Spectroscopy&atitle=Raman+spectral+statistical+classification+of+nasopharyngeal+carcinoma+and+nasopharyngeal+normal+cell+lines+based+on+support+vector+classification&aulast=Chen&pid=<author>Chen+Y.%3BSun+L.%3BHuang+Y.%3BOu+L.%3BSu+Y.<%2Fauthor><AN>364424545<%2FAN><DT>Article<%2FDT>)

328.

Raman spectroscopy demonstrates amifostine-induced protection of bone quality in the irradiated murine mandible.

Tchanque-Fossuo C., Gong B., Poushanchi B., Gallagher K.K., Sarhaddi D., Donneys A., Deshpande S.S., Morris M., Goldstein S., Buchman S.R.

Journal of Bone and Mineral Research. Conference: 2011 Annual Meeting of the American Society for Bone and Mineral Research, ASBMR 2011. San Diego, CA United States. Conference Publication: (var.pagings). 26(SUPPL. 1) (no pagination), 2011. Date of Publication: 2011.

AN: 71519770

Purpose: Adjuvant radiotherapy (XRT) in the management of head and neck cancer remains severely debilitating. Fortunately, newly developed agents aimed at decreasing XRT-induced damage have shown great promise. Amifostine (AMF) is a drug which confers radio-protection to the exposed normal tissues, such as bone. Our intent is to utilize Raman spectroscopy (RS) to demonstrate how AMF preserves the mineral composition of the murine mandible following human equivalent XRT.

Method(s): 6 Sprague Dawley rats were randomly assigned into 2 groups: XRT (n=3), AMF (n=3). Both groups underwent bioequivalent XRT of 70Gy in 5 fractions to the left hemimandible. AMF was administered 45min prior to XRT. 56 days post-XRT, the hemimandibles were harvested, and Raman spectra with 4 cm-1 spectral resolution taken in the region of interest spanning 5.1 mm posterior to the last molar. A contralateral hemimandible from XRT served as Control. Bone mineral and matrix-specific Raman bands were observed and analyzed using One-way Anova, with statistical significance at p<0.05.

Result(s): The XRT group exhibited greater signs of radiation-induced stress, including alopecia, mucositis and weight loss compared to AMF. Gross examination demonstrated a reduction in angular cortical density. Mineral crystallinity, determined by the width of the primary phosphate band, which is fitted to obtain the full-width half-maximal-FWHM was calculated. FWHM demonstrated significant difference between AMF and XRT (p=0.005) and XRT and Control (p=0.021). There was no difference between AMF and Control (p=0.901;Fig1). There was an important shift in the peak position of the major phosphate band to higher wavenumber. These results are consistent with XRT-induced changes in mineral composition and crystallinity. Additional computer-aided spectral subtraction further confirmed these results (Fig2). AMF was spectrally similar to the Control in the same region thus demonstrating AMF protective effect from XRT-induced mineral damage.

Conclusion(s): Our novel findings demonstrate that AMF prophylaxis maintains and protects bone quality in the setting of XRT. RS is an emerging and exceptionally attractive clinical translational technology to investigate and monitor both the destructive effects of XRT and the therapeutic remediation of AMF on the structural, physical and chemical quality of bone. With noninvasive fiberoptic probe this technology holds the potential for diagnostic use in humans.

Institution

(Tchanque-Fossuo) Plastic Surgery Research Fellow, United States (Gong, Poushanchi, Gallagher, Sarhaddi, Donneys, Deshpande, Morris, Buchman) University of Michigan, United States

(Goldstein) University of Michigan, Orthopaedic Research Lab, United States

Publisher

Wiley-Blackwell

Emtree Heading

*bone; *protection; *mandible; *society; *Raman spectrometry; human; technology; radiation protection; weight reduction; alopecia; head and neck cancer; radiation; statistical significance; bone mineral; fiber optics; mucosa inflammation; Sprague Dawley rat; examination; density; prophylaxis; computer; tissues; adjuvant therapy; diagnosis; *amifostine; *mineral; phosphate.

Drug Index Terms

*amifostine; *mineral; phosphate.

Other Index Terms

*bone; *protection; *mandible; *society; *Raman spectrometry; human; technology; radiation protection; weight reduction; alopecia; head and neck cancer; radiation; statistical significance; bone mineral; fiber optics; mucosa inflammation; Sprague Dawley rat; examination; density; prophylaxis; computer; tissues; adjuvant therapy; diagnosis.

Link to the Ovid Full Text or citation:

[Click here for full text options](https://ovidsp.ovid.com/ovidweb.cgi?T=JS&CSC=Y&NEWS=N&PAGE=fulltext&D=emed12&AN=71519770)

Link to the External Link Resolver:

[SFX](https://sfx-86scu.hosted.exlibrisgroup.com.cn/86scu?sid=OVID:embase&id=pmid:&id=doi:&issn=08840431&isbn=&volume=26&issue=SUPPL.+1&spage=&pages=&date=2011&title=Journal+of+Bone+and+Mineral+Research&atitle=Raman+spectroscopy+demonstrates+amifostine-induced+protection+of+bone+quality+in+the+irradiated+murine+mandible&aulast=Tchanque-Fossuo&pid=<author>Tchanque-Fossuo+C.%3BGong+B.%3BPoushanchi+B.%3BGallagher+K.K.%3BSarhaddi+D.%3BDonneys+A.%3BDeshpande+S.S.%3BMorris+M.%3BGoldstein+S.%3BBuchman+S.R.<%2Fauthor><AN>71519770<%2FAN><DT>Conference+Abstract<%2FDT>)

329.

BMLA Woburn 2011 - BMLA Annual Conference, Scientific Meeting and AGM.

Anonymous

Lasers in Medical Science. Conference: BMLA Annual Conference, Scientific Meeting and AGM, BMLA Woburn 2011. Woburn United Kingdom. Conference Publication: (var.pagings). 26(6) (no pagination), 2011. Date of Publication: November 2011.

AN: 70622506

The proceedings contain 38 papers. The topics discussed include: fluorescence-guided tumor resection: development of quantitative measurements, sub-surface imaging and extensions of indications; in vitro fluorescent behavior and location of porphyrins in cell lines; detecting breast cancer by probing micro-calcifications with deep-Raman; scattering attenuation microscopy (SAM) of oral epithelial dysplasia; singlet oxygen luminescence dosimetry and multi-modal intravital imaging of response to photodynamic therapy in preclinical in vivo tumor models; PDT for vascular anomalies: prospective evaluation of outcome; photodynamic therapy for advanced and/or recurrent tongue base carcinoma; PDT for uncommon broncho-pulmonary neoplastic lesions; from idea to bench to bedside and industry: journeys in translating optical technologies to clinical medicine and the life sciences; and the use of the long-pulsed Nd:YAG laser for venous malformations in the pediatric population.

Publisher

Springer London

Emtree Heading

imaging; photodynamic therapy; cell line; breast cancer; attenuation; calcification; dysplasia; microscopy; dosimetry; luminescence; tumor model; congenital blood vessel malformation; tongue carcinoma; industry; optics; technology; clinical medicine; biomedicine; neodymium laser; congenital malformation; population; cancer surgery; in vitro study; fluorescence; singlet oxygen; porphyrin.

Drug Index Terms

singlet oxygen; porphyrin.

Other Index Terms

imaging; photodynamic therapy; cell line; breast cancer; attenuation; calcification; dysplasia; microscopy; dosimetry; luminescence; tumor model; congenital blood vessel malformation; tongue carcinoma; industry; optics; technology; clinical medicine; biomedicine; neodymium laser; congenital malformation; population; cancer surgery; in vitro study; fluorescence.

Link to the Ovid Full Text or citation:

[Click here for full text options](https://ovidsp.ovid.com/ovidweb.cgi?T=JS&CSC=Y&NEWS=N&PAGE=fulltext&D=emed12&AN=70622506)

Link to the External Link Resolver:

[SFX](https://sfx-86scu.hosted.exlibrisgroup.com.cn/86scu?sid=OVID:embase&id=pmid:&id=doi:&issn=02688921&isbn=&volume=26&issue=6&spage=&pages=&date=2011&title=Lasers+in+Medical+Science&atitle=BMLA+Woburn+2011+-+BMLA+Annual+Conference%2C+Scientific+Meeting+and+AGM&aulast=&pid=<author>anonymous<%2Fauthor><AN>70622506<%2FAN><DT>Conference+Review<%2FDT>)

330.

Early detection of premalignant lesions and oral cancer.

Steele T.O., Meyers A.

Otolaryngologic Clinics of North America. 44(1) (pp 221-229), 2011. Date of Publication: February 2011.

AN: 360000187

Cancers of the oral cavity account for approximately 3% of malignancies diagnosed annually in the United States. As with other upper aerodigestive tract cancers, 5-year survival rates for oral cavity cancers decrease with delayed diagnosis. Cancers of the oral cavity are thought to progress from premalignant/precancerous lesions, beginning as hyperplastic tissue and developing into invasive squamous cell carcinoma. Despite the general accessibility of the oral cavity during physical examination, many malignancies are not diagnosed until late stages of disease. To prevent malignant transformation of these oral precursor lesions, multiple screening and detection techniques have been developed to address this problem. © 2011 Elsevier Inc.

PMID

21093631 [<https://www.ncbi.nlm.nih.gov/pubmed/?term=21093631>]

Institution

(Steele) Department of Otolaryngology, Head and Neck Surgery, University of California Davis, 2521 Stockton Boulevard, Suite 7200, Sacramento, CA 95817, United States (Meyers) Department of Otolaryngology, Dentistry and Engineering, University of Colorado Denver, 12631 East 17th Avenue, B205 Aurora, CO 80045, United States

Publisher

W.B. Saunders (Independence Square West, Philadelphia PA 19106-3399, United States)

Emtree Heading

*cancer screening; cytology; discoid lupus erythematosus/di [Diagnosis]; dyskeratosis/di [Diagnosis]; early diagnosis; epidermolysis bullosa/di [Diagnosis]; erythroplasia/di [Diagnosis]; fluorescence imaging; fluorescence spectroscopy; human; leukoplakia/di [Diagnosis]; lichen planus/di [Diagnosis]; *mouth cancer/di [Diagnosis]; mouth disease/di [Diagnosis]; mouth examination; narrow band imaging; optical coherence tomography; *precancer/di [Diagnosis]; Raman spectrometry; reflectometry; review; staining; tolonium chloride; supravital staining.

Candidate Terms

supravital staining [other term]

Drug Index Terms

tolonium chloride.

Other Index Terms

*cancer screening; cytology; discoid lupus erythematosus / diagnosis; dyskeratosis / diagnosis; early diagnosis; epidermolysis bullosa / diagnosis; erythroplasia / diagnosis; fluorescence imaging; fluorescence spectroscopy; human; leukoplakia / diagnosis; lichen planus / diagnosis; *mouth cancer / *diagnosis; mouth disease / diagnosis; mouth examination; narrow band imaging; optical coherence tomography; *precancer / *diagnosis; Raman spectrometry; reflectometry; review; staining.

Link to the Ovid Full Text or citation:

[Click here for full text options](https://ovidsp.ovid.com/ovidweb.cgi?T=JS&CSC=Y&NEWS=N&PAGE=fulltext&D=emed12&AN=360000187)

Link to the External Link Resolver:

[SFX](https://sfx-86scu.hosted.exlibrisgroup.com.cn/86scu?sid=OVID:embase&id=pmid:21093631&id=doi:10.1016%2Fj.otc.2010.10.002&issn=00306665&isbn=&volume=44&issue=1&spage=221&pages=221-229&date=2011&title=Otolaryngologic+Clinics+of+North+America&atitle=Early+detection+of+premalignant+lesions+and+oral+cancer&aulast=Steele&pid=<author>Steele+T.O.%3BMeyers+A.<%2Fauthor><AN>360000187<%2FAN><DT>Review<%2FDT>)

331.

Inorganic nanoparticles for cancer imaging and therapy.

Huang H.-C., Barua S., Sharma G., Dey S.K., Rege K.

Journal of Controlled Release. 155(3) (pp 344-357), 2011. Date of Publication: 07 Nov 2011.

AN: 51556033

Inorganic nanoparticles have received increased attention in the recent past as potential diagnostic and therapeutic systems in the field of oncology. Inorganic nanoparticles have demonstrated successes in imaging and treatment of tumors both ex vivo and in vivo, with some promise towards clinical trials. This review primarily discusses progress in applications of inorganic nanoparticles for cancer imaging and treatment, with an emphasis on in vivo studies. Advances in the use of semiconductor fluorescent quantum dots, carbon nanotubes, gold nanoparticles (spheres, shells, rods, cages), iron oxide magnetic nanoparticles and ceramic nanoparticles in tumor targeting, imaging, photothermal therapy and drug delivery applications are discussed. Limitations and toxicity issues associated with inorganic nanoparticles in living organisms are also discussed. © 2011 Elsevier B.V. All rights reserved.

PMID

21723891 [<https://www.ncbi.nlm.nih.gov/pubmed/?term=21723891>]

Institution

(Huang, Barua, Rege) Chemical Engineering, Arizona State University, Tempe, AZ 85287, United States (Dey) Materials Science and Engineering, Arizona State University, Tempe, AZ 85287, United States

(Rege) Center for the Convergence of Physical Science and Cancer Biology, Arizona State University, Tempe, AZ 85287, United States

(Sharma) Sanford-Burnham Medical Research Institute, San Diego, CA 92037, United States

Publisher

Elsevier (P.O. Box 211, Amsterdam 1000 AE, Netherlands)

Emtree Heading

ablation therapy; animal venereal tumor/dt [Drug Therapy]; breast cancer; breast carcinoma; breast tumor/dt [Drug Therapy]; cancer diagnosis; clinical trial (topic); colon carcinoma/dt [Drug Therapy]; colorectal cancer; computer assisted tomography; glioma; head and neck cancer; human; kidney tumor/dt [Drug Therapy]; Lewis carcinoma/dt [Drug Therapy]; low drug dose; lung carcinoma/di [Diagnosis]; lymph node metastasis/di [Diagnosis]; lymphoma/dt [Drug Therapy]; mouth carcinoma/dt [Drug Therapy]; multimodality cancer therapy; nonhuman; nuclear magnetic resonance imaging; optical coherence tomography; pancreas cancer; pancreas carcinoma/dt [Drug Therapy]; phase 1 clinical trial (topic); phase 2 clinical trial (topic); priority journal; prostate cancer/dt [Drug Therapy]; protein expression; Raman spectrometry; rectum carcinoma; review; single drug dose; squamous cell carcinoma/dt [Drug Therapy]; X ray; cadmium; carbon nanotube/cb [Drug Combination]; carbon nanotube/dt [Drug Therapy]; carbon nanotube/ip [Intraperitoneal Drug Administration]; carbon nanotube/tu [Intratumoral Drug Administration]; carbon nanotube/pk [Pharmacokinetics]; carbon nanotube/sc [Subcutaneous Drug Administration]; cisplatin/cb [Drug Combination]; cisplatin/dt [Drug Therapy]; cisplatin/pk [Pharmacokinetics]; doxorubicin/cb [Drug Combination]; doxorubicin/dt [Drug Therapy]; epidermal growth factor receptor/ec [Endogenous Compound]; folate receptor/ec [Endogenous Compound]; gamma urogastrone/ec [Endogenous Compound]; gold nanoparticle/ad [Drug Administration]; gold nanoparticle/cb [Drug Combination]; gold nanoparticle/dt [Drug Therapy]; gold nanoparticle/ip [Intraperitoneal Drug Administration]; gold nanoparticle/tu [Intratumoral Drug Administration]; gold nanoparticle/iv [Intravenous Drug Administration]; gold nanoparticle/pk [Pharmacokinetics]; macrogol/cb [Drug Combination]; macrogol/dt [Drug Therapy]; manganese; methotrexate/dt [Drug Therapy]; methotrexate/pr [Pharmaceutics]; nanocage; nanocarrier; nanodiamond; *nanoparticle; nanorod; nanoshell; nanosphere; paclitaxel/cb [Drug Combination]; paclitaxel/dt [Drug Therapy]; podophyllotoxin/pk [Pharmacokinetics]; poly(n isopropylacrylamide)/dt [Drug Therapy]; poly(n isopropylacrylamide)/iv [Intravenous Drug Administration]; polyethyleneimine; quantum dot/dt [Drug Therapy]; quantum dot/iv [Intravenous Drug Administration]; recombinant tumor necrosis factor alpha/cb [Drug Combination]; rituximab/dt [Drug Therapy]; silicon/tu [Intratumoral Drug Administration]; silicon/pk [Pharmacokinetics]; superparamagnetic iron oxide nanoparticle/iv [Intravenous Drug Administration]; vitronectin receptor/ec [Endogenous Compound].

Drug Index Terms

cadmium; carbon nanotube / drug combination / drug therapy / intraperitoneal drug administration / intratumoral drug administration / pharmacokinetics / subcutaneous drug administration; cisplatin / drug combination / drug therapy / pharmacokinetics; doxorubicin / drug combination / drug therapy; epidermal growth factor receptor / endogenous compound; folate receptor / endogenous compound; gamma urogastrone / endogenous compound; gold nanoparticle / drug administration / drug combination / drug therapy / intraperitoneal drug administration / intratumoral drug administration / intravenous drug administration / pharmacokinetics; macrogol / drug combination / drug therapy; manganese; methotrexate / drug therapy / pharmaceutics; nanocage; nanocarrier; nanodiamond; *nanoparticle; nanorod; nanoshell; nanosphere; paclitaxel / drug combination / drug therapy; podophyllotoxin / pharmacokinetics; poly(n isopropylacrylamide) / drug therapy / intravenous drug administration; polyethyleneimine; quantum dot / drug therapy / intravenous drug administration; recombinant tumor necrosis factor alpha / drug combination; rituximab / drug therapy; silicon / intratumoral drug administration / pharmacokinetics; superparamagnetic iron oxide nanoparticle / intravenous drug administration; vitronectin receptor / endogenous compound.

Other Index Terms

ablation therapy; animal venereal tumor / drug therapy; breast cancer; breast carcinoma; breast tumor / drug therapy; cancer diagnosis; clinical trial (topic); colon carcinoma / drug therapy; colorectal cancer; computer assisted tomography; glioma; head and neck cancer; human; kidney tumor / drug therapy; Lewis carcinoma / drug therapy; low drug dose; lung carcinoma / diagnosis; lymph node metastasis / diagnosis; lymphoma / drug therapy; mouth carcinoma / drug therapy; multimodality cancer therapy; nonhuman; nuclear magnetic resonance imaging; optical coherence tomography; pancreas cancer; pancreas carcinoma / drug therapy; phase 1 clinical trial (topic); phase 2 clinical trial (topic); priority journal; prostate cancer / drug therapy; protein expression; Raman spectrometry; rectum carcinoma; review; single drug dose; squamous cell carcinoma / drug therapy; X ray.

Drug Trade Name

taxol

Link to the Ovid Full Text or citation:

[Click here for full text options](https://ovidsp.ovid.com/ovidweb.cgi?T=JS&CSC=Y&NEWS=N&PAGE=fulltext&D=emed12&AN=51556033)

Link to the External Link Resolver:

[SFX](https://sfx-86scu.hosted.exlibrisgroup.com.cn/86scu?sid=OVID:embase&id=pmid:21723891&id=doi:10.1016%2Fj.jconrel.2011.06.004&issn=01683659&isbn=&volume=155&issue=3&spage=344&pages=344-357&date=2011&title=Journal+of+Controlled+Release&atitle=Inorganic+nanoparticles+for+cancer+imaging+and+therapy&aulast=Huang&pid=<author>Huang+H.-C.%3BBarua+S.%3BSharma+G.%3BDey+S.K.%3BRege+K.<%2Fauthor><AN>51556033<%2FAN><DT>Review<%2FDT>)

332.

Gastric cancer detection based on blood plasma surface-enhanced Raman spectroscopy excited by polarized laser light.

Feng S., Chen R., Lin J., Pan J., Wu Y., Li Y., Chen J., Zeng H.

Biosensors and Bioelectronics. 26(7) (pp 3167-3174), 2011. Date of Publication: 15 Mar 2011.

AN: 51223657

We have recently applied surface-enhanced Raman spectroscopy (SERS) for blood plasma analysis for non-invasive nasopharyngeal cancer detection and obtained good preliminary results. The aim of this study was to develop a more robust SERS spectroscopy based blood plasma analysis method for non-invasive gastric cancer detection. The effect of different laser polarizations (non-polarized, linear-polarized, right-handed circularly polarized, and left-handed circularly polarized) on blood plasma SERS spectroscopy was explored for the first time. Silver nanoparticles as the SERS-substrate were directly mixed with blood plasma to enhance the Raman scattering of various biomolecular constituents. High quality SERS spectra were obtained using a fiber optic probe and a dispersive type near infrared Raman system. Blood plasma samples from gastric cancer patients (n= 32) and healthy subjects (n= 33) were analyzed. The diagnostic performance for differentiating gastric cancer plasma from normal plasma was evaluated. Principal component analysis combined with linear discriminant analysis (LDA) of the obtained spectral data was used to develop diagnostic algorithms. Classification results obtained from cross-validation of the LDA model based on the four spectral data sets of different laser polarizations demonstrated different diagnostic sensitivities and specificities: 71.9% and 72.7% for non-polarized laser excitation, 75% and 87.9% for linear-polarized laser excitation, 81.3% and 78.8% for right-handed circularly polarized laser excitation, 100% and 97% for left-handed circularly polarized laser excitation. The results from this exploratory study demonstrated that plasma SERS spectroscopy with left-handed circularly polarized laser excitation has great promise of becoming a clinically useful diagnostic tool for non-invasive gastric cancer detection. © 2010 Elsevier B.V.

PMID

21227679 [<https://www.ncbi.nlm.nih.gov/pubmed/?term=21227679>]

Institution

(Feng, Chen, Lin, Li, Chen) Key Laboratory of OptoElectronic Science and Technology for Medicine, Ministry of Education, Fujian Normal University, Fuzhou 350007, China (Pan) Fujian Provincial Tumor Hospital, Fuzhou 350001, China

(Wu) Fujian Provincial Hospital, Fuzhou 350001, China

(Zeng) Cancer Imaging Department, British Columbia Cancer Agency Research Centre, 675 West 10th Avenue, Vancouver, BC V5Z 1L3, Canada

Publisher

Elsevier Ltd (Langford Lane, Kidlington, Oxford OX5 1GB, United Kingdom)

Emtree Heading

adult; algorithm; article; *blood analysis; *cancer diagnosis; cancer screening; chirality; clinical article; colloid; controlled study; diagnostic test accuracy study; diagnostic value; discriminant analysis; human; *laser; non invasive measurement; polarization; principal component analysis; *Raman spectrometry; sensitivity and specificity; *stomach cancer/di [Diagnosis]; silver nanoparticle.

Drug Index Terms

silver nanoparticle.

Other Index Terms

adult; algorithm; article; *blood analysis; *cancer diagnosis; cancer screening; chirality; clinical article; colloid; controlled study; diagnostic test accuracy study; diagnostic value; discriminant analysis; human; *laser; non invasive measurement; polarization; principal component analysis; *Raman spectrometry; sensitivity and specificity; *stomach cancer / *diagnosis.

Link to the Ovid Full Text or citation:

[Click here for full text options](https://ovidsp.ovid.com/ovidweb.cgi?T=JS&CSC=Y&NEWS=N&PAGE=fulltext&D=emed12&AN=51223657)

Link to the External Link Resolver:

[SFX](https://sfx-86scu.hosted.exlibrisgroup.com.cn/86scu?sid=OVID:embase&id=pmid:21227679&id=doi:10.1016%2Fj.bios.2010.12.020&issn=09565663&isbn=&volume=26&issue=7&spage=3167&pages=3167-3174&date=2011&title=Biosensors+and+Bioelectronics&atitle=Gastric+cancer+detection+based+on+blood+plasma+surface-enhanced+Raman+spectroscopy+excited+by+polarized+laser+light&aulast=Feng&pid=<author>Feng+S.%3BChen+R.%3BLin+J.%3BPan+J.%3BWu+Y.%3BLi+Y.%3BChen+J.%3BZeng+H.<%2Fauthor><AN>51223657<%2FAN><DT>Article<%2FDT>)

333.

Characterization and discrimination of nasopharyngeal carcinoma and nasopharyngeal normal cell lines using confocal Raman microspectroscopy.

Ye Y., Li Y., Su Y., Zou C., Chen Y., Ou L., Chen R., Zeng H.

Spectroscopy. 25(5) (pp 217-224), 2011. Date of Publication: 2011.

AN: 362213587

Raman microspectroscopy can provide molecular-level information about the biochemical composition and structure of cells and tissues with excellent spatial resolution. In this study, Raman spectroscopy of individual cells from nasopharyngeal carcinoma cell lines C666-1, CNE2 and nasopharyngeal normal cell line NP69 are investigated for their differences. The spectral intensity ratio at 1449 and 1657 cm-1 with a decision line of I1449/I 1657=1.10 can very easily separate the tumor and normal cell lines into two groups. Principal component analysis (PCA) and linear discriminant analysis (LDA) are also used to classify different cell lines and achieved a specificity and sensitivity of 100 and 90%, respectively. The results support the potential utility of Raman spectroscopy for nasopharyngeal diagnosis. © 2011 - IOS Press and the authors. All rights reserved.

Institution

(Ye, Chen, Li, Ou, Chen) Key Laboratory of Optoelectronic Science and Technology for Medicine, Ministry of Education, Fujian Normal University, Fuzhou, Fujian 350007, China (Ye, Chen) College of Physics and Information, Fuzhou University, Fuzhou, Fujian, China

(Su, Zou, Chen) Fujian Provincial Tumor Hospital, Fuzhou, Fujian, China

(Zeng) Cancer Imaging Department, British Columbia Cancer Research Centre, Vancouver, BC, Canada

Publisher

Hindawi Publishing Corporation (410 Park Avenue, 15th Floor, 287 pmb, New York NY 10022, United States)

Emtree Heading

article; biochemical composition; cancer cell culture; *carcinoma cell; cell structure; confocal microscopy; human; human cell; *nasopharynx carcinoma; *Raman spectrometry; sensitivity and specificity; tissue characterization; tumor cell line; *cell strain C666 1; *cell strain CNE2; *cell strain NP69; *confocal Raman microspectroscopy.

Candidate Terms

*cell strain C666 1 [other term]	*cell strain CNE2 [other term]

*cell strain NP69 [other term]

*confocal Raman microspectroscopy [other term]

Other Index Terms

article; biochemical composition; cancer cell culture; *carcinoma cell; cell structure; confocal microscopy; human; human cell; *nasopharynx carcinoma; *Raman spectrometry; sensitivity and specificity; tissue characterization; tumor cell line.

Link to the Ovid Full Text or citation:

[Click here for full text options](https://ovidsp.ovid.com/ovidweb.cgi?T=JS&CSC=Y&NEWS=N&PAGE=fulltext&D=emed12&AN=362213587)

Link to the External Link Resolver:

[SFX](https://sfx-86scu.hosted.exlibrisgroup.com.cn/86scu?sid=OVID:embase&id=pmid:&id=doi:10.3233%2FSPE-2011-0511&issn=07124813&isbn=&volume=25&issue=5&spage=217&pages=217-224&date=2011&title=Spectroscopy&atitle=Characterization+and+discrimination+of+nasopharyngeal+carcinoma+and+nasopharyngeal+normal+cell+lines+using+confocal+Raman+microspectroscopy&aulast=Ye&pid=<author>Ye+Y.%3BLi+Y.%3BSu+Y.%3BZou+C.%3BChen+Y.%3BOu+L.%3BChen+R.%3BZeng+H.<%2Fauthor><AN>362213587<%2FAN><DT>Article<%2FDT>)

334.

Screening for oral cancers-Which method is most effective?.

Kao S.-Y., Lo W.-L.

Journal of the Chinese Medical Association. 74(12) (pp 529-530), 2011. Date of Publication: December 2011.

AN: 51742267

PMID

22196466 [<https://www.ncbi.nlm.nih.gov/pubmed/?term=22196466>]

Institution

(Kao) Department of Stomatology, Taipei Veterans General Hospital, Taipei, Taiwan (Republic of China) (Kao) National Yang-Ming University School of Dentistry, Taipei, Taiwan (Republic of China)

(Lo) Section of Oral and Maxillofacial Surgery, Department of Stomatology, Taipei Veterans General Hospital, Taipei, Taiwan (Republic of China)

(Lo) Department of Dentistry and Institute of Oral Biology, National Yang-Ming University School of Dentistry, Taipei, Taiwan (Republic of China)

Publisher

Elsevier Ltd (Langford Lane, Kidlington, Oxford OX5 1GB, United Kingdom)

Emtree Heading

age; alcohol consumption; autofluorescence imaging; cancer risk; *cancer screening; cancer survival; clinical effectiveness; cytometry; diagnostic accuracy; editorial; fluorescence spectroscopy; health care cost; histology; human; immunocytochemistry; medical practice; *mouth cancer/di [Diagnosis]; *mouth cancer/dm [Disease Management]; mouth examination; mouth mucosa; optical instrumentation; oral biopsy; precancer; quality of life; Raman spectrometry; sensitivity and specificity; smoking; spectroscopy; squamous cell carcinoma; survival rate; Taiwan; tolonium chloride.

Drug Index Terms

tolonium chloride.

Other Index Terms

age; alcohol consumption; autofluorescence imaging; cancer risk; *cancer screening; cancer survival; clinical effectiveness; cytometry; diagnostic accuracy; editorial; fluorescence spectroscopy; health care cost; histology; human; immunocytochemistry; medical practice; *mouth cancer / *diagnosis / *disease management; mouth examination; mouth mucosa; optical instrumentation; oral biopsy; precancer; quality of life; Raman spectrometry; sensitivity and specificity; smoking; spectroscopy; squamous cell carcinoma; survival rate; Taiwan.

Link to the Ovid Full Text or citation:

[Click here for full text options](https://ovidsp.ovid.com/ovidweb.cgi?T=JS&CSC=Y&NEWS=N&PAGE=fulltext&D=emed12&AN=51742267)

Link to the External Link Resolver:

[SFX](https://sfx-86scu.hosted.exlibrisgroup.com.cn/86scu?sid=OVID:embase&id=pmid:22196466&id=doi:10.1016%2Fj.jcma.2011.10.003&issn=17264901&isbn=&volume=74&issue=12&spage=529&pages=529-530&date=2011&title=Journal+of+the+Chinese+Medical+Association&atitle=Screening+for+oral+cancers-Which+method+is+most+effective%3F&aulast=Kao&pid=<author>Kao+S.-Y.%3BLo+W.-L.<%2Fauthor><AN>51742267<%2FAN><DT>Editorial<%2FDT>)

335.

Discrimination of parotid neoplasms from the normal parotid gland by use of Raman spectroscopy and support vector machine.

Yan B., Li Y., Yang G., Wen Z.-N., Li M.-L., Li L.-J.

Oral Oncology. 47(5) (pp 430-435), 2011. Date of Publication: May 2011.

AN: 51332185

Preoperative diagnosis of neoplasms in the parotid gland is essential for successful surgical treatment. The purpose of this study is to apply Raman spectroscopy in order to distinguish the spectral differences between pleomorphic adenoma and Warthin tumor from that of normal parotid gland tissues. Furthermore we establish the diagnostic model of the Raman spectra of neoplasms in parotid gland by employing support vector machine (SVM) with Gaussian radial basis function. Firstly, Raman spectra from different histopathological tissues were obtained by near-infrared Raman microscope, SVM was then employed to analyze the different spectra and establish a discriminating model. As a result, the differences of peaks in the region 800-1800 cm-1 demonstrated the biochemical molecular alterations between different histopathological tissues. Compared with normal parotid gland tissues, the content of proteins, lipids and DNA increased in pleomorphic adenoma. The content of DNA increased but proteins and lipids decreased in Warthin tumor. SVM displayed a powerful role in the classification of three different groups. The sensitivities and specificities of discrimination between different groups reached above 95% and 99%, respectively. Raman spectroscopy combined SVM algorithm could have great potential for providing a noninvasive, effective and accurate diagnostic technology for neoplasm diagnosis in the parotid gland. © 2011 Elsevier Ltd. All rights reserved.

PMID

21439894 [<https://www.ncbi.nlm.nih.gov/pubmed/?term=21439894>]

Institution

(Yan, Li, Li) State Key Laboratory of Oral Diseases, Sichuan University, Chengdu 610041, China (Yang, Wen, Li) College of Chemistry, Sichuan University, Chengdu 610041, China

(Li) Department of Head and Neck Oncology, West China Hospital of Stomatology, Sichuan University, Chengdu 610041, China

Publisher

Elsevier Ltd (Langford Lane, Kidlington, Oxford OX5 1GB, United Kingdom)

Emtree Heading

adolescent; adult; aged; article; clinical article; female; histopathology; human; human tissue; male; normal distribution; *pleomorphic adenoma/di [Diagnosis]; priority journal; *Raman spectrometry; sensitivity and specificity; support vector machine; *Warthin tumor/di [Diagnosis]; DNA/ec [Endogenous Compound]; lipid/ec [Endogenous Compound]; protein/ec [Endogenous Compound].

Drug Index Terms

DNA / endogenous compound; lipid / endogenous compound; protein / endogenous compound.

Other Index Terms

adolescent; adult; aged; article; clinical article; female; histopathology; human; human tissue; male; normal distribution; *pleomorphic adenoma / *diagnosis; priority journal; *Raman spectrometry; sensitivity and specificity; support vector machine; *Warthin tumor / *diagnosis.

Link to the Ovid Full Text or citation:

[Click here for full text options](https://ovidsp.ovid.com/ovidweb.cgi?T=JS&CSC=Y&NEWS=N&PAGE=fulltext&D=emed12&AN=51332185)

Link to the External Link Resolver:

[SFX](https://sfx-86scu.hosted.exlibrisgroup.com.cn/86scu?sid=OVID:embase&id=pmid:21439894&id=doi:10.1016%2Fj.oraloncology.2011.02.021&issn=13688375&isbn=&volume=47&issue=5&spage=430&pages=430-435&date=2011&title=Oral+Oncology&atitle=Discrimination+of+parotid+neoplasms+from+the+normal+parotid+gland+by+use+of+Raman+spectroscopy+and+support+vector+machine&aulast=Yan&pid=<author>Yan+B.%3BLi+Y.%3BYang+G.%3BWen+Z.-N.%3BLi+M.-L.%3BLi+L.-J.<%2Fauthor><AN>51332185<%2FAN><DT>Article<%2FDT>)

336.

Detection of circulating tumor cells in human peripheral blood using surface-enhanced raman scattering nanoparticles.

Wang X., Qian X., Beitler J.J., Chen Z.G., Khuri F.R., Lewis M.M., Shin H.J.C., Nie S., Shin D.M.

Cancer Research. 71(5) (pp 1526-1532), 2011. Date of Publication: 01 Mar 2011.

AN: 361363235

The detection and characterization of circulating tumor cells (CTC) holds great promise for personalizing medicine and optimizing systemic therapy. However, low specificity, low sensitivity, and the time consuming nature of current approaches have impeded clinical adoption. Here we report a new method using surface-enhanced Raman spectroscopy (SERS) to directly measure targeted CTCs in the presence of white blood cells. SERS nanoparticles with epidermal growth factor peptide as a targeting ligand have successfully identified CTCs in the peripheral blood of 19 patients with squamous cell carcinoma of the head and neck (SCCHN), with a range of 1 to 720 CTCs per milliliter of whole blood. Our technique may provide an important new clinical tool for management of patients with SCCHN and other cancers. ©2011 AACR.

PMID

21212408 [<https://www.ncbi.nlm.nih.gov/pubmed/?term=21212408>]

Institution

(Wang, Beitler, Chen, Khuri, Shin) Department of Hematology and Medical Oncology, Emory University School of Medicine, Atlanta, GA, United States (Qian, Nie) Department of Biomedical Engineering and Chemistry, Emory University School of Medicine, Atlanta, GA, United States

(Beitler) Department of Radiation Oncology and Otolaryngology, Emory University School of Medicine, Atlanta, GA, United States

(Lewis) Department of Pathology, Emory University School of Medicine, Atlanta, GA, United States

(Shin) Quest Diagnostics, Atlanta, GA, United States

(Shin) Emory University School of Medicine, Winship Cancer Institute, 1365 Clifton Road, C-3094, Atlanta, GA 30322, United States

Publisher

American Association for Cancer Research Inc. (615 Chestnut Street, 17th Floor, Philadelphia PA 19106-3483, United States)

Emtree Heading

article; clinical article; controlled study; head and neck carcinoma; human; leukocyte; mouse; nonhuman; patient care; priority journal; *Raman spectrometry; sensitivity and specificity; squamous cell carcinoma; target cell; *tumor cell; *tumor diagnosis; epidermal growth factor/ec [Endogenous Compound]; *nanoparticle; *circulating tumor cell.

Candidate Terms

*circulating tumor cell [other term]

Drug Index Terms

epidermal growth factor / endogenous compound; *nanoparticle.

Other Index Terms

article; clinical article; controlled study; head and neck carcinoma; human; leukocyte; mouse; nonhuman; patient care; priority journal; *Raman spectrometry; sensitivity and specificity; squamous cell carcinoma; target cell; *tumor cell; *tumor diagnosis.

Link to the Ovid Full Text or citation:

[Click here for full text options](https://ovidsp.ovid.com/ovidweb.cgi?T=JS&CSC=Y&NEWS=N&PAGE=fulltext&D=emed12&AN=361363235)

Link to the External Link Resolver:

[SFX](https://sfx-86scu.hosted.exlibrisgroup.com.cn/86scu?sid=OVID:embase&id=pmid:21212408&id=doi:10.1158%2F0008-5472.CAN-10-3069&issn=00085472&isbn=&volume=71&issue=5&spage=1526&pages=1526-1532&date=2011&title=Cancer+Research&atitle=Detection+of+circulating+tumor+cells+in+human+peripheral+blood+using+surface-enhanced+raman+scattering+nanoparticles&aulast=Wang&pid=<author>Wang+X.%3BQian+X.%3BBeitler+J.J.%3BChen+Z.G.%3BKhuri+F.R.%3BLewis+M.M.%3BShin+H.J.C.%3BNie+S.%3BShin+D.M.<%2Fauthor><AN>361363235<%2FAN><DT>Article<%2FDT>)

337.

Advances in bio-optical imaging for the diagnosis of early oral cancer.

Olivo M., Bhuvaneswari R., Keogh I.

Pharmaceutics. 3(3) (pp 354-378), 2011. Date of Publication: September 2011.

AN: 362600188

Oral cancer is among the most common malignancies worldwide, therefore early detection and treatment is imperative. The 5-year survival rate has remained at a dismal 50% for the past several decades. The main reason for the poor survival rate is the fact that most of the oral cancers, despite the general accessibility of the oral cavity, are not diagnosed until the advanced stage. Early detection of the oral tumors and its precursor lesions may be the most effective means to improve clinical outcome and cure most patients. One of the emerging technologies is the use of non-invasive in vivo tissue imaging to capture the molecular changes at high-resolution to improve the detection capability of early stage disease. This review will discuss the use of optical probes and highlight the role of optical imaging such as autofluorescence, fluorescence diagnosis (FD), laser confocal endomicroscopy (LCE), surface enhanced Raman spectroscopy (SERS), optical coherence tomography (OCT) and confocal reflectance microscopy (CRM) in early oral cancer detection. FD is a promising method to differentiate cancerous lesions from benign, thus helping in the determination of adequate resolution of surgical resection margin. LCE offers in vivo cellular imaging of tissue structures from surface to subsurface layers and has demonstrated the potential to be used as a minimally invasive optical biopsy technique for early diagnosis of oral cancer lesions. SERS was able to differentiate between normal and oral cancer patients based on the spectra acquired from saliva of patients. OCT has been used to visualize the detailed histological features of the oral lesions with an imaging depth down to 2-3 mm. CRM is an optical tool to noninvasively image tissue with near histological resolution. These comprehensive diagnostic modalities can also be used to define surgical margin and to provide a direct assessment of the therapeutic effectiveness. © 2011 by the authors; licensee MDPI, Basel, Switzerland.

Institution

(Olivo) School of Physics, National University of Ireland Galway, Ireland (Olivo) Department of Pharmacy, National University of Singapore, No. 18 Science Drive 4, Block S4, 117543, Singapore

(Bhuvaneswari) National Cancer Centre Singapore, 11 Hospital Drive, 169610, Singapore

(Keogh) Consultant Otolaryngologist, Head and Neck Surgeon, Academic Department of Otorhinolaryngology, National University of Ireland Galway and Galway University Hospitals, Ireland

Publisher

MDPI AG (Kandererstrasse 25, Basel CH-4057, Switzerland)

Emtree Heading

antineoplastic activity; *autofluorescence imaging; camera; cancer diagnosis; cancer morphology; colon carcinoma/dt [Drug Therapy]; *confocal laser microscopy; *confocal microscopy; diagnostic accuracy; diagnostic imaging; differential diagnosis; drug accumulation; drug targeting; *early cancer/di [Diagnosis]; early diagnosis; *fluorescence imaging; Fourier transform mass spectrometry; human; in vivo study; lung non small cell cancer/dt [Drug Therapy]; minimally invasive procedure; molecular imaging; molecular probe; *mouth cancer/di [Diagnosis]; mouth mucosa; non invasive measurement; nonhuman; *optical coherence tomography; optical depth; optical instrumentation; precancer/di [Diagnosis]; *Raman spectrometry; review; surgical anatomy; treatment planning; aminolevulinic acid/cm [Drug Comparison]; aminolevulinic acid/pk [Pharmacokinetics]; bevacizumab/dt [Drug Therapy]; bevacizumab/pd [Pharmacology]; gold nanoparticle; hypericin/cm [Drug Comparison]; hypericin/pk [Pharmacokinetics]; nanoshell; protoporphyrin/ec [Endogenous Compound]; technetium 99m; vasculotropin/ec [Endogenous Compound]; *confocal reflectance microscopy; *fluorescence diagnosis.

Candidate Terms

*confocal reflectance microscopy [other term] *fluorescence diagnosis [other term]

Drug Index Terms

aminolevulinic acid / drug comparison / pharmacokinetics; bevacizumab / drug therapy / pharmacology; gold nanoparticle; hypericin / drug comparison / pharmacokinetics; nanoshell; protoporphyrin / endogenous compound; technetium 99m; vasculotropin / endogenous compound.

Other Index Terms

antineoplastic activity; *autofluorescence imaging; camera; cancer diagnosis; cancer morphology; colon carcinoma / drug therapy; *confocal laser microscopy; *confocal microscopy; diagnostic accuracy; diagnostic imaging; differential diagnosis; drug accumulation; drug targeting; *early cancer / *diagnosis; early diagnosis; *fluorescence imaging; Fourier transform mass spectrometry; human; in vivo study; lung non small cell cancer / drug therapy; minimally invasive procedure; molecular imaging; molecular probe; *mouth cancer / *diagnosis; mouth mucosa; non invasive measurement; nonhuman; *optical coherence tomography; optical depth; optical instrumentation; precancer / diagnosis; *Raman spectrometry; review; surgical anatomy; treatment planning.

Link to the Ovid Full Text or citation:

[Click here for full text options](https://ovidsp.ovid.com/ovidweb.cgi?T=JS&CSC=Y&NEWS=N&PAGE=fulltext&D=emed12&AN=362600188)

Link to the External Link Resolver:

[SFX](https://sfx-86scu.hosted.exlibrisgroup.com.cn/86scu?sid=OVID:embase&id=pmid:&id=doi:10.3390%2Fpharmaceutics3030354&issn=19994923&isbn=&volume=3&issue=3&spage=354&pages=354-378&date=2011&title=Pharmaceutics&atitle=Advances+in+bio-optical+imaging+for+the+diagnosis+of+early+oral+cancer&aulast=Olivo&pid=<author>Olivo+M.%3BBhuvaneswari+R.%3BKeogh+I.<%2Fauthor><AN>362600188<%2FAN><DT>Review<%2FDT>)

338.

Optimizing electroporation assisted silver nanoparticle delivery into living C666 cells for surface-enhanced Raman spectroscopy.

Yu Y., Lin J., Wu Y., Feng S., Li Y., Huang Z., Chen R., Zeng H.

Spectroscopy. 25(1) (pp 13-21), 2011. Date of Publication: 2011.

AN: 361167421

Electroporation assisted metallic nanoparticle delivery has been shown by our previous work to significantly reduce the time of sample preparation for surface-enhanced Raman spectroscopy (SERS) measurements of biological cells. In this paper, we report our experimental work to optimize the electroporation parameters, including adjustment of the pulse pattern, operation temperature, and electroporation buffer, for fastest delivery of silver nanoparticles into living C666 cells (a human nasopharyngeal carcinoma cell line). The delivery efficiency was evaluated by the integrated intensity of whole cell SERS spectrum. Our work concluded that the silver nanoparticle delivery rate is best under the electroporation condition of using 4 consecutive 350 V (875 V/cm) rectangular electric pulses of 1, 10, 10 and 1 ms durations, respectively. Low temperature (0-4degreeC) is necessary for keeping cell viability during the electroporation process and it also improves the delivery efficiency of silver nanoparticles. The serum in the buffer has no obvious effect on the delivery efficiency. © 2011 - IOS Press and the authors. All rights reserved.

Institution

(Yu, Lin, Feng, Li, Huang, Chen) Key Laboratory of OptoElectronic Science and Technology for Medicine, Ministry of Education, Fujian Normal University, Fuzhou, Fujian 350007, China (Wu) Fujian Provincial Hospital, Fuzhou, Fujian, China

(Zeng) Cancer Imaging Department, British Columbia Cancer Agency Research Centre, Vancouver, BC, Canada

Publisher

Hindawi Publishing Corporation (410 Park Avenue, 15th Floor, 287 pmb, New York NY 10022, United States)

Emtree Heading

article; cell viability; controlled study; electron microscopy; electroporation; human; human cell; low temperature; pulse rate; *Raman spectrometry; temperature measurement; whole cell; silver nanoparticle.

Drug Index Terms

silver nanoparticle.

Other Index Terms

article; cell viability; controlled study; electron microscopy; electroporation; human; human cell; low temperature; pulse rate; *Raman spectrometry; temperature measurement; whole cell.

Link to the Ovid Full Text or citation:

[Click here for full text options](https://ovidsp.ovid.com/ovidweb.cgi?T=JS&CSC=Y&NEWS=N&PAGE=fulltext&D=emed12&AN=361167421)

Link to the External Link Resolver:

[SFX](https://sfx-86scu.hosted.exlibrisgroup.com.cn/86scu?sid=OVID:embase&id=pmid:&id=doi:10.3233%2FSPE-2010-0493&issn=07124813&isbn=&volume=25&issue=1&spage=13&pages=13-21&date=2011&title=Spectroscopy&atitle=Optimizing+electroporation+assisted+silver+nanoparticle+delivery+into+living+C666+cells+for+surface-enhanced+Raman+spectroscopy&aulast=Yu&pid=<author>Yu+Y.%3BLin+J.%3BWu+Y.%3BFeng+S.%3BLi+Y.%3BHuang+Z.%3BChen+R.%3BZeng+H.<%2Fauthor><AN>361167421<%2FAN><DT>Article<%2FDT>)

339.

Raman spectroscopy in head and neck cancer.

Harris A.T., Rennie A., Waqar-Uddin H., Wheatley S.R., Ghosh S.K., Martin-Hirsch D.P., Fisher S.E., High A.S., Kirkham J., Upile T.

Head and Neck Oncology. 2(1) (no pagination), 2010. Article Number: 26. Date of Publication: 2010.

AN: 51101211

In recent years there has been much interest in the use of optical diagnostics in cancer detection. Early diagnosis of cancer affords early intervention and greatest chance of cure. Raman spectroscopy is based on the interaction of photons with the target material producing a highly detailed biochemical 'fingerprint' of the sample. It can be appreciated that such a sensitive biochemical detection system could confer diagnostic benefit in a clinical setting. Raman has been used successfully in key health areas such as cardiovascular diseases, and dental care but there is a paucity of literature on Raman spectroscopy in Head and Neck cancer. Following the introduction of health care targets for cancer, and with an ever-aging population the need for rapid cancer detection has never been greater. Raman spectroscopy could confer great patient benefit with early, rapid and accurate diagnosis. This technique is almost labour free without the need for sample preparation. It could reduce the need for whole pathological specimen examination, in theatre it could help to determine margin status, and finally peripheral blood diagnosis may be an achievable target. © 2010 Harris et al; licensee BioMed Central Ltd.

PMID

20923567 [<https://www.ncbi.nlm.nih.gov/pubmed/?term=20923567>]

Institution

(Harris, Rennie, Waqar-Uddin, Wheatley, Ghosh, Martin-Hirsch) Department of Ear, Nose and Throat/Head and Neck Surgery, Calderdale and Huddersfield NHS Trust, Huddersfield, United Kingdom (Fisher) Section of Experimental Therapeutics, Leeds Institute of Molecular Medicine, University of Leeds, Leeds, United Kingdom

(Fisher) School of Health Studies, University of Bradford, Bradford, United Kingdom

(High) Department of Pathology Leeds Dental Institute, University of Leeds, Leeds, United Kingdom

(Kirkham) Department of Oral Biology, Leeds Dental Institute, University of Leeds, Leeds, United Kingdom

(Upile) Department of Otolaryngology with Head and Neck Surgery, Chase Farm Hospital, Enfield, United Kingdom

(Upile) Head and Neck Unit, UCLH, Euston Road, London, United Kingdom

Publisher

OA Publishing London (Second Floor, 10-12 Maclise Road, London, England W140PR, United States)

Emtree Heading

cancer classification; cancer diagnosis; cancer survival; carcinoma cell; diagnostic accuracy; diagnostic imaging; differential diagnosis; early diagnosis; early intervention; genetic algorithm; genotype phenotype correlation; gold standard; *head and neck cancer/di [Diagnosis]; head and neck carcinoma/di [Diagnosis]; histopathology; human; imaging system; intermethod comparison; priority journal; *Raman spectrometry; review; sensitivity and specificity; thyroid cell; tumor biopsy.

Other Index Terms

cancer classification; cancer diagnosis; cancer survival; carcinoma cell; diagnostic accuracy; diagnostic imaging; differential diagnosis; early diagnosis; early intervention; genetic algorithm; genotype phenotype correlation; gold standard; *head and neck cancer / *diagnosis; head and neck carcinoma / diagnosis; histopathology; human; imaging system; intermethod comparison; priority journal; *Raman spectrometry; review; sensitivity and specificity; thyroid cell; tumor biopsy.

Link to the Ovid Full Text or citation:

[Click here for full text options](https://ovidsp.ovid.com/ovidweb.cgi?T=JS&CSC=Y&NEWS=N&PAGE=fulltext&D=emed11&AN=51101211)

Link to the External Link Resolver:

[SFX](https://sfx-86scu.hosted.exlibrisgroup.com.cn/86scu?sid=OVID:embase&id=pmid:20923567&id=doi:10.1186%2F1758-3284-2-26&issn=17583284&isbn=&volume=2&issue=1&spage=26&pages=&date=2010&title=Head+and+Neck+Oncology&atitle=Raman+spectroscopy+in+head+and+neck+cancer&aulast=Harris&pid=<author>Harris+A.T.%3BRennie+A.%3BWaqar-Uddin+H.%3BWheatley+S.R.%3BGhosh+S.K.%3BMartin-Hirsch+D.P.%3BFisher+S.E.%3BHigh+A.S.%3BKirkham+J.%3BUpile+T.<%2Fauthor><AN>51101211<%2FAN><DT>Review<%2FDT>)

340.

Nanotechnology in head and neck cancer: The race is on.

El-Sayed I.H.

Current Oncology Reports. 12(2) (pp 121-128), 2010. Date of Publication: 2010.

AN: 50820132

Rapid advances in the ability to produce nanoparticles of uniform size, shape, and composition have started a revolution in the sciences. Nano-sized structures herald innovative technology with a wide range of potential therapeutic and diagnostic applications. More than 1000 nanostructures have been reported, many with potential medical applications, such as metallic-, dielectric-, magnetic-, liposomal-, and carbon-based structures. Of these, noble metallic nanoparticles are generating significant interest because of their multifunctional capacity for novel methods of laboratory-based diagnostics, in vivo clinical diagnostic imaging, and therapeutic treatments. This review focuses on recent advances in the applications of nanotechnology in head and neck cancer, with special emphasis on the particularly promising plasmonic gold nanotechnology. © 2010 The Author(s).

PMID

20425597 [<https://www.ncbi.nlm.nih.gov/pubmed/?term=20425597>]

Institution

(El-Sayed) Department of Otolaryngology-Head and Neck Surgery, Otolaryngology Minimally Invasive Skull Base Program, University of California, San Francisco, 2380 Sutter Street, San Francisco, CA 94115, United States

Publisher

Current Medicine Group LLC (400 Market St, Ste 700 Philadelphia PA 19106, United States)

Emtree Heading

cancer chemotherapy; cancer diagnosis; cancer radiotherapy; cancer survival; clinical effectiveness; colon carcinoma/dt [Drug Therapy]; diagnostic imaging; diagnostic value; drug delivery system; drug targeting; fluorescence imaging; *head and neck cancer/di [Diagnosis]; *head and neck cancer/th [Therapy]; human; ionizing radiation; molecular dynamics; mouth cancer/th [Therapy]; *nanomedicine; nonhuman; phototherapy; quality of life; Raman spectrometry; review; squamous cell carcinoma/th [Therapy]; surface plasmon resonance; total quality management; gold nanoparticle/pr [Pharmaceutics]; nanorod; tumor necrosis factor/dt [Drug Therapy]; tumor necrosis factor/pr [Pharmaceutics]; plasmonic photothermal therapy.

Candidate Terms

plasmonic photothermal therapy [other term]

Drug Index Terms

gold nanoparticle / pharmaceutics; nanorod; tumor necrosis factor / drug therapy / pharmaceutics.

Other Index Terms

cancer chemotherapy; cancer diagnosis; cancer radiotherapy; cancer survival; clinical effectiveness; colon carcinoma / drug therapy; diagnostic imaging; diagnostic value; drug delivery system; drug targeting; fluorescence imaging; *head and neck cancer / *diagnosis / *therapy; human; ionizing radiation; molecular dynamics; mouth cancer / therapy; *nanomedicine; nonhuman; phototherapy; quality of life; Raman spectrometry; review; squamous cell carcinoma / therapy; surface plasmon resonance; total quality management.

Link to the Ovid Full Text or citation:

[Click here for full text options](https://ovidsp.ovid.com/ovidweb.cgi?T=JS&CSC=Y&NEWS=N&PAGE=fulltext&D=emed11&AN=50820132)

Link to the External Link Resolver:

[SFX](https://sfx-86scu.hosted.exlibrisgroup.com.cn/86scu?sid=OVID:embase&id=pmid:20425597&id=doi:10.1007%2Fs11912-010-0087-2&issn=15233790&isbn=&volume=12&issue=2&spage=121&pages=121-128&date=2010&title=Current+Oncology+Reports&atitle=Nanotechnology+in+head+and+neck+cancer%3A+The+race+is+on&aulast=El-Sayed&pid=<author>El-Sayed+I.H.<%2Fauthor><AN>50820132<%2FAN><DT>Review<%2FDT>)

341.

Nasopharyngeal cancer detection based on blood plasma surface-enhanced Raman spectroscopy and multivariate analysis.

Feng S., Chen R., Lin J., Pan J., Chen G., Li Y., Cheng M., Huang Z., Chen J., Zeng Haishan H.

Biosensors and Bioelectronics. 25(11) (pp 2414-2419), 2010. Date of Publication: July 2010.

AN: 358984532

A surface-enhanced Raman spectroscopy (SERS) method was developed for blood plasma biochemical analysis for the first time with the aim to develop a simple blood test for non-invasive nasopharyngeal cancer detection. Silver nanoparticles (Ag NP) as the SERS-active nanostructures were directly mixed with blood plasma to enhance the Raman scattering signals of various biomolecular constituents such as proteins, lipids, and nucleic acids. High quality SERS spectrum from blood plasma-Ag NP mixture can be obtained within 10. s using a Renishaw micro-Raman system. SERS measurements were performed on two groups of blood plasma samples: one group from patients (n= 43) with pathologically confirmed nasopharyngeal carcinomas (WHO type I, II, and III) and the other group from healthy volunteers (control subjects, n= 33). Tentative assignments of the Raman bands in the measured SERS spectra suggest interesting cancer specific biomolecular differences, including an increase in the relative amounts of nucleic acid, collagen, phospholipids and phenylalanine and a decrease in the percentage of amino acids and saccharide contents in the blood plasma of nasopharyngeal cancer patients as compared to that of healthy subjects. Principal component analysis (PCA) of the measured SERS spectra separated the spectral features of the two groups into two distinct clusters with little overlaps. Linear discriminate analysis (LDA) based on the PCA generated features differentiated the nasopharyngeal cancer SERS spectra from normal SERS spectra with high sensitivity (90.7%) and specificity (100%). The results from this exploratory study demonstrated great potentials for developing SERS blood plasma analysis into a novel clinical tool for non-invasive detection of nasopharyngeal cancers. © 2010 Elsevier B.V.

PMID

20427174 [<https://www.ncbi.nlm.nih.gov/pubmed/?term=20427174>]

Institution

(Feng, Chen, Lin, Chen, Li, Huang, Chen) Key Laboratory of OptoElectronic Science and Technology for Medicine, Ministry of Education, Fujian Normal University, Fuzhou 350007, China (Pan) Fujian Provincial Tumor Hospital, Fuzhou 350001, China

(Feng) College of Chemistry and Material Science, Experimental Center, Fujian Normal University, Fuzhou 350007, China

(Cheng) College of Minjiang, Fuzhou 350001, China

(Zeng Haishan) Cancer Imaging Department, British Columbia Cancer Research Centre, 675 West 10th Avenue, Vancouver, BC V5Z 1L3, Canada

Publisher

Elsevier Ltd

Emtree Heading

adult; article; biochemistry; *blood sampling; *cancer diagnosis; colloid; controlled study; human; major clinical study; *multivariate analysis; *nasopharynx cancer/di [Diagnosis]; nasopharynx carcinoma/di [Diagnosis]; principal component analysis; *Raman spectrometry; sensitivity and specificity; squamous cell carcinoma; amino acid; carbohydrate; collagen; nucleic acid; phenylalanine; phospholipid; silver nanoparticle.

Drug Index Terms

amino acid; carbohydrate; collagen; nucleic acid; phenylalanine; phospholipid; silver nanoparticle.

Other Index Terms

adult; article; biochemistry; *blood sampling; *cancer diagnosis; colloid; controlled study; human; major clinical study; *multivariate analysis; *nasopharynx cancer / *diagnosis; nasopharynx carcinoma / diagnosis; principal component analysis; *Raman spectrometry; sensitivity and specificity; squamous cell carcinoma.

Link to the Ovid Full Text or citation:

[Click here for full text options](https://ovidsp.ovid.com/ovidweb.cgi?T=JS&CSC=Y&NEWS=N&PAGE=fulltext&D=emed11&AN=358984532)

Link to the External Link Resolver:

[SFX](https://sfx-86scu.hosted.exlibrisgroup.com.cn/86scu?sid=OVID:embase&id=pmid:20427174&id=doi:10.1016%2Fj.bios.2010.03.033&issn=09565663&isbn=&volume=25&issue=11&spage=2414&pages=2414-2419&date=2010&title=Biosensors+and+Bioelectronics&atitle=Nasopharyngeal+cancer+detection+based+on+blood+plasma+surface-enhanced+Raman+spectroscopy+and+multivariate+analysis&aulast=Feng&pid=<author>Feng+S.%3BChen+R.%3BLin+J.%3BPan+J.%3BChen+G.%3BLi+Y.%3BCheng+M.%3BHuang+Z.%3BChen+J.%3BZeng+Haishan+H.<%2Fauthor><AN>358984532<%2FAN><DT>Article<%2FDT>)

342.

Quantification of circulating tumor cells in peripheral blood using EGFR-targeting gold nanoparticles.

Wang X., Qian X., Beitler J.J., Chen Z.G., Nie S., Shin D.M.

Cancer Research. Conference: 101st Annual Meeting of the American Association for Cancer Research, AACR 2010. Washington, DC United States. Conference Publication: (var.pagings). 70(8 SUPPL. 1) (no pagination), 2010. Date of Publication: 15 Apr 2010.

AN: 71451293

Background: The prognostic significance of circulating tumor cells (CTCs) in patients with squamous cell carcinoma of the head and neck (HNSCC) is of great clinical interest. HNSCCs are overwhelmingly EGFR positive.

Objective(s): Our goal was to pilot nanotechnology to identify CTCs in human xenograft mouse model as a prelude to clinical use.

Method(s): Surface-enhanced Raman Scattering (SERS)-gold nanocrystals (60 nm) were prepared by spectral encoding with a Raman reporter molecule and then stabilized and protected by low molecular weight polyethylene glycols (PEGs). Heterofunctional PEG was covalently conjugated to epidermal growth factor (EGF) peptide, a ligand that binds to EGFR with high specificity and affinity. To identify CTC in normal mouse blood, varying quantities of Tu212 cells were added into mice blood samples, then our nanoparticle was added to the sample and the presence of Tu212 cells were detected in vitro using laser excitation of the Raman reporters. Different types of cancer cells with variable EGFR expression were also examined in blood to confirm the specificity of the EGF-targeted SERS nanoparticles. Normal mouse blood served as the experimental control to account for non-specific binding.

Result(s): The cellular uptake of the EGFR-targeting nanoparticle in high EGFR expression Tu212 cells was about 10-fold higher than that in low EGFR expression H460 cells in vitro, suggesting a specific EGFR targeting of the gold nanoparticle. Tu212 cells could be selectively labeled and quantified (5.6 x 10^4^ nanoparticles/cell) at a concentration of approximately 10 cells per ml of normal mouse blood. Comparing the labeling of Tu212 cells by our nanoparticles with H460 cells in the mouse blood samples showed that the signal intensity in Tu212 cells was about 9-fold higher than that in H460 cells ((3750 +/- 230 vs. 350 +/- 120, respectively, p <0.05). These results indicated high sensitivity and specificity of using SERS-gold nanoparticles for detection of CTCs.

Conclusion(s): We have demonstrated the ability to use targeted surface-enhanced Raman gold nanoparticles to quantify CTCs with EGFR expression.

Institution

(Wang, Beitler, Chen, Shin) Emory Univ., Winship Cancer Inst., Atlanta, GA, United States (Qian, Nie) Emory Univ., Department of Biomedical Engineering, Atlanta, GA, United States

Publisher

American Association for Cancer Research Inc.

Emtree Heading

*blood; *cancer research; *circulating tumor cell; mouse; human; in vitro study; blood sampling; xenograft; nanotechnology; neck; molecular weight; squamous cell carcinoma; excitation; Raman spectrometry; laser; mouse model; cancer cell; sensitivity and specificity; patient; *gold nanoparticle; nanoparticle; peptide; epidermal growth factor; macrogol derivative; gold; ligand.

Drug Index Terms

*gold nanoparticle; nanoparticle; peptide; epidermal growth factor; macrogol derivative; gold; ligand.

Other Index Terms

*blood; *cancer research; *circulating tumor cell; mouse; human; in vitro study; blood sampling; xenograft; nanotechnology; neck; molecular weight; squamous cell carcinoma; excitation; Raman spectrometry; laser; mouse model; cancer cell; sensitivity and specificity; patient.

Link to the Ovid Full Text or citation:

[Click here for full text options](https://ovidsp.ovid.com/ovidweb.cgi?T=JS&CSC=Y&NEWS=N&PAGE=fulltext&D=emed11&AN=71451293)

Link to the External Link Resolver:

[SFX](https://sfx-86scu.hosted.exlibrisgroup.com.cn/86scu?sid=OVID:embase&id=pmid:&id=doi:10.1158%2F1538-7445.AM10-1155&issn=00085472&isbn=&volume=70&issue=8+SUPPL.+1&spage=1155&pages=&date=2010&title=Cancer+Research&atitle=Quantification+of+circulating+tumor+cells+in+peripheral+blood+using+EGFR-targeting+gold+nanoparticles&aulast=Wang&pid=<author>Wang+X.%3BQian+X.%3BBeitler+J.J.%3BChen+Z.G.%3BNie+S.%3BShin+D.M.<%2Fauthor><AN>71451293<%2FAN><DT>Conference+Abstract<%2FDT>)

343.

Near infrared Raman spectral character and diagnostic value of squamous cell carcinoma of oral mucosa.

Li Y., Wen Z.-N., Li L.-J., Li M.-L., Zhang Z., Gao N.

Hua xi kou qiang yi xue za zhi = Huaxi kouqiang yixue zazhi = West China journal of stomatology. 28(1) (pp 61-64), 2010. Date of Publication: 01 Feb 2010.

AN: 613660404

RESULTS: By analyzing the subtracted mean spectra, the increasing peak intensity in wavenumber range of 500-2 200 cm(-1) hinted us of the high contents of DNA, protein and lipid in OSCC, which elucidate the high proliferative activity. The increasing peak intensity in the wavenumber range of 500-2 200 cm(-1) hinted us of the high contents of DNA, protein and lipid in OSCC, which elucidate the high proliferative activity, but the difference between OLK and OSCC was not as much as that between normal and OSCC. The Gaussian radial basis function support vector machine showed powerful ability in grouping and modeling of normal and OSCC, and the specificity, sensitivity and accuracy were 100%, 97.44% and 98.81% correspondingly. The algorithm showed good ability in grouping and modeling of OLK and OSCC, the specificity, sensitivity and accuracy were 95.00%, 86.36% and 96.30%. CONCLUSION: Combined with support vector machines, near infrared Raman spectroscopy could detect the biochemical variations in oral normal, OLK and OSCC, and establish diagnostic model accurately.

METHODS: Near infrared Raman spectra of ten normal mucosa, twenty OSCC and thirty oral leukoplakia (OLK) cases were collected in the research. Based on the previous researches, the information of the subtracted spectra of compared group was gained by the characteristic band in them. A Gaussian radial basis function support vector machine was used to classify spectra and establish the diagnostic models. The efficacy and validity of the algorithm were evaluated.

OBJECTIVE: To evaluate the value of the near infrared Raman spectroscope in diagnosing oral squamous cell carcinoma (OSCC).

PMID

20337078 [<https://www.ncbi.nlm.nih.gov/pubmed/?term=20337078>]

Institution

(Li, Wen, Li, Li, Zhang, Gao) State Key Laboratory of Oral Diseases, Sichuan University, Chengdu 610041, China

Emtree Heading

human; *leukoplakia; *mouth mucosa; Raman spectrometry; sensitivity and specificity; squamous cell carcinoma.

Other Index Terms

human; *leukoplakia; *mouth mucosa; Raman spectrometry; sensitivity and specificity; squamous cell carcinoma.

Link to the Ovid Full Text or citation:

[Click here for full text options](https://ovidsp.ovid.com/ovidweb.cgi?T=JS&CSC=Y&NEWS=N&PAGE=fulltext&D=emed11&AN=613660404)

Link to the External Link Resolver:

[SFX](https://sfx-86scu.hosted.exlibrisgroup.com.cn/86scu?sid=OVID:embase&id=pmid:20337078&id=doi:&issn=10001182&isbn=&volume=28&issue=1&spage=61&pages=61-64&date=2010&title=Hua+xi+kou+qiang+yi+xue+za+zhi+%3D+Huaxi+kouqiang+yixue+zazhi+%3D+West+China+journal+of+stomatology&atitle=Near+infrared+Raman+spectral+character+and+diagnostic+value+of+squamous+cell+carcinoma+of+oral+mucosa&aulast=Li&pid=<author>Li+Y.%3BWen+Z.-N.%3BLi+L.-J.%3BLi+M.-L.%3BZhang+Z.%3BGao+N.<%2Fauthor><AN>613660404<%2FAN><DT>Article<%2FDT>)

344.

Who should have Mohs micrographic surgery?.

Perkins W.

Current Opinion in Otolaryngology and Head and Neck Surgery. 18(4) (pp 283-289), 2010. Date of Publication: August 2010.

AN: 50983923

Purpose of review: To review the indications for Mohs micrographic surgery in skin cancer particularly with relationship to tumours of the head and neck and any recent developments which may influence those indications in the near future. Recent findings: There is increasing evidence to support the use of Mohs micrographic surgery in the treatment of recurrent and primary basal cell carcinoma and in squamous cell carcinoma, particularly when there is evidence of perineural invasion. Mohs micrographic surgery is particularly effective in the treatment of dermatofibosarcoma protuberans, especially in the high-risk head and neck area in which wide local excision may not be possible. Developments in freshly excised tissue imaging by confocal fluorescence microscopy and/or Raman spectroscopy may further refine the technique of Mohs micrographic surgery. Developments in immunohistochemistry with rapid staining of frozen sections may make Mohs micrographic surgery for lentigo maligna and melanoma in situ more feasible in the future, which would be an advantage in the head and neck.

Summary: The evidence base for the use of Mohs micrographic surgery for a wide range of cutaneous tumours is growing and the technique continues to develop. Close collaboration between Mohs micrographic surgeons, specialist surgical oncologists and reconstructive surgeons will provide the highest quality care for our patients with some of the most challenging cutaneous tumours. © 2010 Wolters Kluwer Health

Lippincott Williams and Wilkins.

PMID

20613530 [<https://www.ncbi.nlm.nih.gov/pubmed/?term=20613530>]

Institution

(Perkins) Nottingham University Hospitals NHS Trust, Queens Medical Centre, Nottingham NG7 2UH, United Kingdom

Publisher

Lippincott Williams and Wilkins (530 Walnut Street,P O Box 327, Philadelphia PA 19106-3621, United States)

Emtree Heading

adenoid cystic carcinoma/su [Surgery]; adenosquamous carcinoma/su [Surgery]; basal cell carcinoma/su [Surgery]; cancer invasion; cancer patient; dermatofibrosarcoma protuberans/su [Surgery]; fibroxanthoma/su [Surgery]; fluorescence microscopy; frozen section; head and neck tumor/su [Surgery]; health care quality; human; immunohistochemistry; malignant lentigo/su [Surgery]; melanoma/su [Surgery]; myoepithelioma/su [Surgery]; priority journal; Raman spectrometry; recurrent cancer/su [Surgery]; review; skin cancer/su [Surgery]; *skin surgery; squamous cell carcinoma/su [Surgery]; sweat gland carcinoma/su [Surgery]; treatment indication; trichoepithelioma/su [Surgery]; *Mohs micrographic surgery.

Candidate Terms

*Mohs micrographic surgery [other term]

Other Index Terms

adenoid cystic carcinoma / surgery; adenosquamous carcinoma / surgery; basal cell carcinoma / surgery; cancer invasion; cancer patient; dermatofibrosarcoma protuberans / surgery; fibroxanthoma / surgery; fluorescence microscopy; frozen section; head and neck tumor / surgery; health care quality; human; immunohistochemistry; malignant lentigo / surgery; melanoma / surgery; myoepithelioma / surgery; priority journal; Raman spectrometry; recurrent cancer / surgery; review; skin cancer / surgery; *skin surgery; squamous cell carcinoma / surgery; sweat gland carcinoma / surgery; treatment indication; trichoepithelioma / surgery.

Link to the Ovid Full Text or citation:

[Click here for full text options](https://ovidsp.ovid.com/ovidweb.cgi?T=JS&CSC=Y&NEWS=N&PAGE=fulltext&D=emed11&AN=50983923)

Link to the External Link Resolver:

[SFX](https://sfx-86scu.hosted.exlibrisgroup.com.cn/86scu?sid=OVID:embase&id=pmid:20613530&id=doi:10.1097%2FMOO.0b013e32833b6f19&issn=10689508&isbn=&volume=18&issue=4&spage=283&pages=283-289&date=2010&title=Current+Opinion+in+Otolaryngology+and+Head+and+Neck+Surgery&atitle=Who+should+have+Mohs+micrographic+surgery%3F&aulast=Perkins&pid=<author>Perkins+W.<%2Fauthor><AN>50983923<%2FAN><DT>Review<%2FDT>)

345.

Optical and molecular techniques to identify tumor margins within the larynx.

Hughes O.R., Stone N., Kraft M., Arens C., Birchall M.A.

Head and Neck. 32(11) (pp 1544-1553), 2010. Date of Publication: November 2010.

AN: 360042471

Failure to remove tumor cells from the larynx significantly increases the risk of local recurrence following surgical excision. Healthy tissue must be preserved to optimize long-term vocal and swallowing function. It is essential to accurately distinguish between healthy mucosa, dysplasia, and invasive carcinoma. Optical and molecular examining technologies have been developed to improve tumor margin identification in vivo. We aimed to review the efficacy of these technologies. Published articles were identified using MEDLINE, EMBASE, and Cochrane central register of controlled trials (CENTRAL). Randomized clinical trials are required to establish the benefit to patients and cost to the health service of using 5-aminolevulinic acid (ALA)-induced fluorescent imaging, contact endoscopy, and optical coherence tomography (OCT). Furthermore, primary research is required to validate other techniques, such as confocal endomicroscopy and Raman spectroscopy, and to develop their clinical applications in the larynx. © 2010 Wiley Periodicals, Inc.

PMID

20091681 [<https://www.ncbi.nlm.nih.gov/pubmed/?term=20091681>]

Institution

(Hughes, Birchall) Department of Otorhinolaryngology-Head and Neck Surgery, Royal National Throat, Nose, and Ear Hospital, London, United Kingdom (Stone) Biophotonics Research Unit, Gloucestershire Royal Hospital, Gloucester, United Kingdom

(Kraft) Department of Otorhinolaryngology-Head and Neck Surgery, Kantonsspital Aarau, Aarau, Switzerland

(Arens) Department of Otorhinolaryngology, Head and Neck Surgery, University Hospital Magdeburg, Magdeburg, Germany

Publisher

John Wiley and Sons Inc. (P.O.Box 18667, Newark NJ 07191-8667, United States)

Emtree Heading

autofluorescence imaging; cancer diagnosis; cancer radiotherapy; clinical practice; clinical research; clinical trial; confocal microscopy; dysplasia/di [Diagnosis]; endoscopy; fluorescence imaging; fluorescence spectroscopy; health care cost; health services research; histopathology; human; illumination; laryngeal biopsy; larynx; larynx cancer/di [Diagnosis]; larynx cancer/rt [Radiotherapy]; larynx cancer/su [Surgery]; *larynx carcinoma/di [Diagnosis]; *larynx carcinoma/rt [Radiotherapy]; *larynx carcinoma/su [Surgery]; larynx disorder/di [Diagnosis]; *larynx tumor/di [Diagnosis]; *larynx tumor/rt [Radiotherapy]; *larynx tumor/su [Surgery]; *molecular imaging; *optical coherence tomography; priority journal; Raman spectrometry; review; sensitivity and specificity; aminolevulinic acid; fluorescent dye; methylene blue; protoporphyrin; contact endoscopy; laryngeal dysplasia/di [Diagnosis].

Candidate Terms

contact endoscopy [other term]	laryngeal dysplasia / diagnosis [other term]

Drug Index Terms

aminolevulinic acid; fluorescent dye; methylene blue; protoporphyrin.

Other Index Terms

autofluorescence imaging; cancer diagnosis; cancer radiotherapy; clinical practice; clinical research; clinical trial; confocal microscopy; dysplasia / diagnosis; endoscopy; fluorescence imaging; fluorescence spectroscopy; health care cost; health services research; histopathology; human; illumination; laryngeal biopsy; larynx; larynx cancer / diagnosis / radiotherapy / surgery; *larynx carcinoma / *diagnosis / *radiotherapy / *surgery; larynx disorder / diagnosis; *larynx tumor / *diagnosis / *radiotherapy / *surgery; *molecular imaging; *optical coherence tomography; priority journal; Raman spectrometry; review; sensitivity and specificity.

Link to the Ovid Full Text or citation:

[Click here for full text options](https://ovidsp.ovid.com/ovidweb.cgi?T=JS&CSC=Y&NEWS=N&PAGE=fulltext&D=emed11&AN=360042471)

Link to the External Link Resolver:

[SFX](https://sfx-86scu.hosted.exlibrisgroup.com.cn/86scu?sid=OVID:embase&id=pmid:20091681&id=doi:10.1002%2Fhed.21321&issn=10433074&isbn=&volume=32&issue=11&spage=1544&pages=1544-1553&date=2010&title=Head+and+Neck&atitle=Optical+and+molecular+techniques+to+identify+tumor+margins+within+the+larynx&aulast=Hughes&pid=<author>Hughes+O.R.%3BStone+N.%3BKraft+M.%3BArens+C.%3BBirchall+M.A.<%2Fauthor><AN>360042471<%2FAN><DT>Review<%2FDT>)

346.

Surfaced-enhanced Raman spectroscopic study on single living human nasopharyngeal carcinoma cells incubated with colloidal gold.

Huang H., Pan J., Chen W., Chen Q., Feng S., Su Y., Xu X., Chen R.

Guang pu xue yu guang pu fen xi = Guang pu. 30(11) (pp 2981-2984), 2010. Date of Publication: 01 Nov 2010.

AN: 605154533

The surface-enhanced Raman scattering (SERS) spectroscopy and normal Raman spectroscopy of single living human nasopharyngeal carcinoma cells(CNE-1) were tested and analyzed by gold nanoparticles incubation into cells. Six obvious Raman bands (718, 1001, 1123, 1336, 1446 and 1660 cm(-1)) were observed in the normal Raman spectroscopy of living CNE-1 cells. The characteristic Raman bands in the SERS spectra of living cells were tentatively assigned. Colloidal gold particles that were introduced inside cells result in strongly enhanced Raman signals of the native chemical constituents of the cells, and over twenty SERS Raman bands were observed in the SERS spectroscopy of living CNE-1 cells. The Raman lines of 1026, 1097, 1336 and 1585 cm(-1) were assigned to vibrations of the DNA backbone, which confirms that some gold nanoparticles were able to enter the nucleus. The results showed that, based on colloidal gold, the SERS spectroscopy might provide a sensitive and structurally selective detecting method for native chemicals inside a cell, such as DNA and phenylalanine.

PMID

21284167 [<https://www.ncbi.nlm.nih.gov/pubmed/?term=21284167>]

Institution

(Huang, Pan, Chen, Chen, Feng, Su, Xu, Chen) Department of Integrative Medicine, Fujian University of Traditional Chinese Medicine, Fuzhou 350108, China

Emtree Heading

cell nucleus; human; *nasopharynx tumor; *Raman spectrometry; tumor cell line; *colloidal gold; DNA; *metal nanoparticle.

Drug Index Terms

*colloidal gold; DNA; *metal nanoparticle.

Other Index Terms

cell nucleus; human; *nasopharynx tumor; *Raman spectrometry; tumor cell line.

Link to the Ovid Full Text or citation:

[Click here for full text options](https://ovidsp.ovid.com/ovidweb.cgi?T=JS&CSC=Y&NEWS=N&PAGE=fulltext&D=emed11&AN=605154533)

Link to the External Link Resolver:

[SFX](https://sfx-86scu.hosted.exlibrisgroup.com.cn/86scu?sid=OVID:embase&id=pmid:21284167&id=doi:&issn=10000593&isbn=&volume=30&issue=11&spage=2981&pages=2981-2984&date=2010&title=Guang+pu+xue+yu+guang+pu+fen+xi+%3D+Guang+pu&atitle=Surfaced-enhanced+Raman+spectroscopic+study+on+single+living+human+nasopharyngeal+carcinoma+cells+incubated+with+colloidal+gold&aulast=Huang&pid=<author>Huang+H.%3BPan+J.%3BChen+W.%3BChen+Q.%3BFeng+S.%3BSu+Y.%3BXu+X.%3BChen+R.<%2Fauthor><AN>605154533<%2FAN><DT>Article<%2FDT>)

347.

Distribution and clearance of PEG-single-walled carbon nanotube cancer drug delivery vehicles in mice.

Bhirde A.A., Patel S., Sousa A.A., Patel V., Molinolo A.A., Ji Y., Leapman R.D., Gutkind J.S., Rusling J.F.

Nanomedicine. 5(10) (pp 1535-1546), 2010. Date of Publication: December 2010.

AN: 360219333

Aims: To study the distribution and clearance of polyethylene glycol (PEG)-ylated single-walled carbon nanotube (SWCNTs) as drug delivery vehicles for the anticancer drug cisplatin in mice.

Material(s) and Method(s): PEG layers were attached to SWCNTs and dispersed in aqueous media and characterized using dynamic light scattering, scanning transmission electron microscopy and Raman spectroscopy. Cytotoxicity was assessed in vitro using Annexin-V assay, and the distribution and clearance pathways in mice were studied by histological staining and Raman spectroscopy. Efficacy of PEG-SWCNT-cisplatin for tumor growth inhibition was studied in mice. Results & discussion: PEG-SWCNTs were efficiently dispersed in aqueous media compared with controls, and did not induce apoptosis in vitro. Hematoxylin and eosin staining, and Raman bands for SWCNTs in tissues from several vital organs from mice injected intravenously with nanotube bioconjugates revealed that control SWCNTs were lodged in lung tissue as large aggregates compared with the PEG-SWCNTs, which showed little or no accumulation. Characteristic SWCNT Raman bands in feces revealed the presence of bilary or renal excretion routes. Attachment of cisplatin on bioconjugates was visualized with Z-contrast scanning transmission electron microscopy. PEG-SWCNT-cisplatin with the attached targeting ligand EGF successfully inhibited growth of head and neck tumor xenografts in mice.

Conclusion(s): PEG-SWCNTs, as opposed to control SWCNTs, form more highly dispersed delivery vehicles that, when loaded with both cisplatin and EGF, inhibit growth of squamous cell tumors. © 2010 Future Medicine Ltd.

PMID

21143032 [<https://www.ncbi.nlm.nih.gov/pubmed/?term=21143032>]

Institution

(Bhirde, Rusling) Department of Chemistry, University of Connecticut, Storrs, CT, United States (Patel, Patel, Molinolo, Gutkind) Oral and Pharyngeal Cancer Branch, National Institute of Dental and Craniofacial Research, National Institutes of Health (NIH), Bethesda, MD 20892, United States

(Sousa, Leapman) Laboratory of Cellular Imaging and Macromolecular Biophysics, National Institute of Biomedical Imaging and Bioengineering, NIH, Bethesda, MD 20892, United States

(Ji) Clinical and Experimental Orthopedics, National Institute of Arthritis, and Musculoskeletal and Skin Diseases, NIH, Bethesda, MD, United States

(Rusling) Department of Cell Biology, University of Connecticut Health Center, Farmington, CT, United States

(Rusling) Institute of Materials Science, University of Connecticut, Storrs, CT, United States

Publisher

Future Medicine Ltd. (2nd Albert Place, Finchley Central, London N3 1QB, United Kingdom)

Emtree Heading

animal experiment; animal model; animal tissue; apoptosis; aqueous solution; article; bile flow; cancer inhibition; controlled study; cytotoxicity test; dispersion; drug clearance; drug delivery system; drug distribution; female; head and neck tumor; light scattering; lung parenchyma; mouse; nonhuman; priority journal; Raman spectrometry; scanning transmission electron microscopy; staining; tumor xenograft; urinary excretion; cisplatin/pr [Pharmaceutics]; eosin; hematoxylin; lipocortin 5; *macrogol; *single walled nanotube.

Drug Index Terms

cisplatin / pharmaceutics; eosin; hematoxylin; lipocortin 5; *macrogol; *single walled nanotube.

Other Index Terms

animal experiment; animal model; animal tissue; apoptosis; aqueous solution; article; bile flow; cancer inhibition; controlled study; cytotoxicity test; dispersion; drug clearance; drug delivery system; drug distribution; female; head and neck tumor; light scattering; lung parenchyma; mouse; nonhuman; priority journal; Raman spectrometry; scanning transmission electron microscopy; staining; tumor xenograft; urinary excretion.

Link to the Ovid Full Text or citation:

[Click here for full text options](https://ovidsp.ovid.com/ovidweb.cgi?T=JS&CSC=Y&NEWS=N&PAGE=fulltext&D=emed11&AN=360219333)

Link to the External Link Resolver:

[SFX](https://sfx-86scu.hosted.exlibrisgroup.com.cn/86scu?sid=OVID:embase&id=pmid:21143032&id=doi:10.2217%2Fnnm.10.90&issn=17435889&isbn=&volume=5&issue=10&spage=1535&pages=1535-1546&date=2010&title=Nanomedicine&atitle=Distribution+and+clearance+of+PEG-single-walled+carbon+nanotube+cancer+drug+delivery+vehicles+in+mice&aulast=Bhirde&pid=<author>Bhirde+A.A.%3BPatel+S.%3BSousa+A.A.%3BPatel+V.%3BMolinolo+A.A.%3BJi+Y.%3BLeapman+R.D.%3BGutkind+J.S.%3BRusling+J.F.<%2Fauthor><AN>360219333<%2FAN><DT>Article<%2FDT>)

348.

Thyroid tissue analysis through Raman spectroscopy.

Teixeira C.S.B., Bitar R.A., Martinho H.S., Santos A.B.O., Kulcsar M.A.V., Friguglietti C.U.M., Da Costa R.B., Arisawa E.A.L., Martin A.A.

Analyst. 134(11) (pp 2361-2370), 2009. Date of Publication: 2009.

AN: 355832454

The diagnosis of thyroid pathologies is usually made by cytologic analysis of the fine needle aspiration (FNA) material. However, this procedure has a low sensitivity at times, presenting a variation of 2-37%. The application of optical spectroscopy in the characterization of alterations could result in the development of a minimally invasive and non-destructive method for the diagnosis of thyroid diseases. Thus, the objective of this work was to study the biochemical alterations of tissues and hormones (T3 and T4) of the thyroid gland by means of molecular vibrations probed by FT-Raman spectroscopy. Through the discriminative linear analysis of the Raman spectra of the tissue, it was possible to establish (in percentages) the correct classification index among the groups: goitre adjacent tissue, goitre nodular region, follicular adenoma, follicular carcinoma and papillary carcinoma. As a result of the comparison between the groups goitre adjacent tissue versus goitre nodular region, an index of 58.3% of correct classification was obtained; this percentage was considered low, and it was not possible to distinguish the Raman spectra of these groups. Between goitre (nodular region and adjacent tissue) versus papillary carcinoma, the index of correct classification was 64.9%, which was considered good. A relevant result was obtained in the analysis of the benign tissues (goitre and follicular adenoma) versus malignant tissues (papillary and follicular carcinomas), for which the index was 72.5% and considered good. It was also possible, by means of visual observation, to find similar vibrational modes in the hormones and pathologic tissues. In conclusion, some biochemical alterations, represented by the FT-Raman spectra, were identified that could possibly be used to classify histologic groups of the thyroid. However, more studies are necessary due to the difficulty in setting a standard for pathologic groups. © The Royal Society of Chemistry 2009.

PMID

19838427 [<https://www.ncbi.nlm.nih.gov/pubmed/?term=19838427>]

Institution

(Teixeira, Santos, Kulcsar, Friguglietti, Da Costa, Arisawa, Martin) Universidade Do Vale Do Paraiba, Instituto de Pesquisa e Desenvolvimento, Laboratorio de Espectroscopia Vibracional Biomedica, Av.Shishima Hifumi 2911, Urbanova CEP 12244-000, Sao Jose dos Campos Sao Paulo, Brazil (Bitar, Martinho) Centro de Ciencias Naturais e Humanas, Universidade Federal Do ABC, 09090-400 Santo Andre Sao Paulo, Brazil

Publisher

Royal Society of Chemistry

Emtree Heading

article; discriminant analysis; Fourier transform mass spectrometry; goiter/di [Diagnosis]; *histopathology; hormone determination; human; human tissue; *Raman spectrometry; thyroid adenoma/di [Diagnosis]; thyroid follicular carcinoma/di [Diagnosis]; *thyroid gland; thyroid papillary carcinoma/di [Diagnosis]; tissue distribution; vibration; liothyronine/ec [Endogenous Compound]; thyroxine/ec [Endogenous Compound].

Drug Index Terms

liothyronine / endogenous compound; thyroxine / endogenous compound.

Other Index Terms

article; discriminant analysis; Fourier transform mass spectrometry; goiter / diagnosis; *histopathology; hormone determination; human; human tissue; *Raman spectrometry; thyroid adenoma / diagnosis; thyroid follicular carcinoma / diagnosis; *thyroid gland; thyroid papillary carcinoma / diagnosis; tissue distribution; vibration.

Link to the Ovid Full Text or citation:

[Click here for full text options](https://ovidsp.ovid.com/ovidweb.cgi?T=JS&CSC=Y&NEWS=N&PAGE=fulltext&D=emed11&AN=355832454)

Link to the External Link Resolver:

[SFX](https://sfx-86scu.hosted.exlibrisgroup.com.cn/86scu?sid=OVID:embase&id=pmid:19838427&id=doi:10.1039%2Fb822578h&issn=00032654&isbn=&volume=134&issue=11&spage=2361&pages=2361-2370&date=2009&title=Analyst&atitle=Thyroid+tissue+analysis+through+Raman+spectroscopy&aulast=Teixeira&pid=<author>Teixeira+C.S.B.%3BBitar+R.A.%3BMartinho+H.S.%3BSantos+A.B.O.%3BKulcsar+M.A.V.%3BFriguglietti+C.U.M.%3BDa+Costa+R.B.%3BArisawa+E.A.L.%3BMartin+A.A.<%2Fauthor><AN>355832454<%2FAN><DT>Article<%2FDT>)

349.

Editorial - A light diagnosis.

Graham D., Deckert V.

Analyst. 134(6) (pp 1027-1028), 2009. Date of Publication: 2009.

AN: 354733598

PMID

19475127 [<https://www.ncbi.nlm.nih.gov/pubmed/?term=19475127>]

Institution

(Graham) University of Strathclyde, Glasgow, United Kingdom (Deckert) Friedrich-Schiller University, Jena, Germany

Publisher

Royal Society of Chemistry

Emtree Heading

atherosclerosis; bacterial spore; Barrett esophagus; breast cancer/di [Diagnosis]; breast metastasis/di [Diagnosis]; cancer cell; cancer diagnosis; chemometric analysis; confocal microscopy; editorial; esophageal adenocarcinoma; human; infrared spectroscopy; interstitial cystitis; larynx carcinoma/di [Diagnosis]; malaria; matrix assisted laser desorption ionization time of flight mass spectrometry; micromorphology; microscopy; near infrared spectroscopy; nonhuman; prostate cancer; radiation scattering; Raman spectrometry; sensitivity and specificity; skin carcinoma; *spectroscopy.

Other Index Terms

atherosclerosis; bacterial spore; Barrett esophagus; breast cancer / diagnosis; breast metastasis / diagnosis; cancer cell; cancer diagnosis; chemometric analysis; confocal microscopy; editorial; esophageal adenocarcinoma; human; infrared spectroscopy; interstitial cystitis; larynx carcinoma / diagnosis; malaria; matrix assisted laser desorption ionization time of flight mass spectrometry; micromorphology; microscopy; near infrared spectroscopy; nonhuman; prostate cancer; radiation scattering; Raman spectrometry; sensitivity and specificity; skin carcinoma; *spectroscopy.

Link to the Ovid Full Text or citation:

[Click here for full text options](https://ovidsp.ovid.com/ovidweb.cgi?T=JS&CSC=Y&NEWS=N&PAGE=fulltext&D=emed11&AN=354733598)

Link to the External Link Resolver:

[SFX](https://sfx-86scu.hosted.exlibrisgroup.com.cn/86scu?sid=OVID:embase&id=pmid:19475127&id=doi:10.1039%2Fb907715b&issn=00032654&isbn=&volume=134&issue=6&spage=1027&pages=1027-1028&date=2009&title=Analyst&atitle=Editorial+-+A+light+diagnosis&aulast=Graham&pid=<author>Graham+D.%3BDeckert+V.<%2Fauthor><AN>354733598<%2FAN><DT>Editorial<%2FDT>)

350.

Vibrational spectroscopy: A clinical tool for cancer diagnostics.

Kendall C., Isabelle M., Bazant-Hegemark F., Hutchings J., Orr L., Babrah J., Baker R., Stone N.

Analyst. 134(6) (pp 1029-1045), 2009. Date of Publication: 2009.

AN: 354733589

Vibrational spectroscopy techniques have demonstrated potential to provide non-destructive, rapid, clinically relevant diagnostic information. Early detection is the most important factor in the prevention of cancer. Raman and infrared spectroscopy enable the biochemical signatures from biological tissues to be extracted and analysed. In conjunction with advanced chemometrics such measurements can contribute to the diagnostic assessment of biological material. This paper also illustrates the complementary advantage of using Raman and FTIR spectroscopy technologies together. Clinical requirements are increasingly met by technological developments which show promise to become a clinical reality. This review summarises recent advances in vibrational spectroscopy and their impact on the diagnosis of cancer. © 2009 Royal Society of Chemistry.

PMID

19475128 [<https://www.ncbi.nlm.nih.gov/pubmed/?term=19475128>]

Institution

(Kendall, Isabelle, Bazant-Hegemark, Hutchings, Orr, Babrah, Baker, Stone) Biophotonics Research Unit, Leadon House, Gloucestershire Hospitals NHS Foundation Trust, Gloucester GL1 3NN, United Kingdom

Publisher

Royal Society of Chemistry

Emtree Heading

absorption; accuracy; brain cancer/di [Diagnosis]; breast cancer/di [Diagnosis]; cancer classification; *cancer diagnosis; digestive system cancer/di [Diagnosis]; head and neck cancer/di [Diagnosis]; histopathology; human; in vitro study; in vivo study; *infrared spectroscopy; laser; leukemia/di [Diagnosis]; lung cancer/di [Diagnosis]; lymph node metastasis/di [Diagnosis]; medical instrumentation; nonhuman; *Raman spectrometry; review; sensitivity and specificity; skin cancer/di [Diagnosis]; synchrotron; thickness; tumor differentiation; urinary tract cancer/di [Diagnosis]; uterine cervix cancer/di [Diagnosis]; vibration; paraffin.

Drug Index Terms

paraffin.

Other Index Terms

absorption; accuracy; brain cancer / diagnosis; breast cancer / diagnosis; cancer classification; *cancer diagnosis; digestive system cancer / diagnosis; head and neck cancer / diagnosis; histopathology; human; in vitro study; in vivo study; *infrared spectroscopy; laser; leukemia / diagnosis; lung cancer / diagnosis; lymph node metastasis / diagnosis; medical instrumentation; nonhuman; *Raman spectrometry; review; sensitivity and specificity; skin cancer / diagnosis; synchrotron; thickness; tumor differentiation; urinary tract cancer / diagnosis; uterine cervix cancer / diagnosis; vibration.

Link to the Ovid Full Text or citation:

[Click here for full text options](https://ovidsp.ovid.com/ovidweb.cgi?T=JS&CSC=Y&NEWS=N&PAGE=fulltext&D=emed11&AN=354733589)

Link to the External Link Resolver:

[SFX](https://sfx-86scu.hosted.exlibrisgroup.com.cn/86scu?sid=OVID:embase&id=pmid:19475128&id=doi:10.1039%2Fb822130h&issn=00032654&isbn=&volume=134&issue=6&spage=1029&pages=1029-1045&date=2009&title=Analyst&atitle=Vibrational+spectroscopy%3A+A+clinical+tool+for+cancer+diagnostics&aulast=Kendall&pid=<author>Kendall+C.%3BIsabelle+M.%3BBazant-Hegemark+F.%3BHutchings+J.%3BOrr+L.%3BBabrah+J.%3BBaker+R.%3BStone+N.<%2Fauthor><AN>354733589<%2FAN><DT>Review<%2FDT>)

351.

Spectroscopic diagnosis of laryngeal carcinoma using near-infrared Raman spectroscopy and random recursive partitioning ensemble techniques.

Teh S.K., Zheng W., Lau D.P., Huang Z.

Analyst. 134(6) (pp 1232-1239), 2009. Date of Publication: 2009.

AN: 354733574

In this work, we evaluated the diagnostic ability of near-infrared (NIR) Raman spectroscopy associated with the ensemble recursive partitioning algorithm based on random forests for identifying cancer from normal tissue in the larynx. A rapid-acquisition NIR Raman system was utilized for tissue Raman measurements at 785 nm excitation, and 50 human laryngeal tissue specimens (20 normal; 30 malignant tumors) were used for NIR Raman studies. The random forests method was introduced to develop effective diagnostic algorithms for classification of Raman spectra of different laryngeal tissues. High-quality Raman spectra in the range of 800-1800 cm-1 can be acquired from laryngeal tissue within 5 seconds. Raman spectra differed significantly between normal and malignant laryngeal tissues. Classification results obtained from the random forests algorithm on tissue Raman spectra yielded a diagnostic sensitivity of 88.0% and specificity of 91.4% for laryngeal malignancy identification. The random forests technique also provided variables importance that facilitates correlation of significant Raman spectral features with cancer transformation. This study shows that NIR Raman spectroscopy in conjunction with random forests algorithm has a great potential for the rapid diagnosis and detection of malignant tumors in the larynx. © 2009 Royal Society of Chemistry.

PMID

19475153 [<https://www.ncbi.nlm.nih.gov/pubmed/?term=19475153>]

Institution

(Teh, Zheng, Huang) Department of Bioengineering, Faculty of Engineering, National University of Singapore, 9 Engineering Drive 1, Singapore 117576, Singapore (Lau) Department of Otolaryngology, Singapore General Hospital, Singapore 169608, Singapore

Publisher

Royal Society of Chemistry

Emtree Heading

algorithm; analytical equipment; article; cancer diagnosis; cancer tissue; clinical article; clinical evaluation; clinical trial; human; human tissue; *larynx carcinoma/di [Diagnosis]; malignant neoplastic disease/di [Diagnosis]; *near infrared spectroscopy; outcome assessment; quality control; *Raman spectrometry; random forest; sensitivity and specificity.

Other Index Terms

algorithm; analytical equipment; article; cancer diagnosis; cancer tissue; clinical article; clinical evaluation; clinical trial; human; human tissue; *larynx carcinoma / *diagnosis; malignant neoplastic disease / diagnosis; *near infrared spectroscopy; outcome assessment; quality control; *Raman spectrometry; random forest; sensitivity and specificity.

Link to the Ovid Full Text or citation:

[Click here for full text options](https://ovidsp.ovid.com/ovidweb.cgi?T=JS&CSC=Y&NEWS=N&PAGE=fulltext&D=emed11&AN=354733574)

Link to the External Link Resolver:

[SFX](https://sfx-86scu.hosted.exlibrisgroup.com.cn/86scu?sid=OVID:embase&id=pmid:19475153&id=doi:10.1039%2Fb811008e&issn=00032654&isbn=&volume=134&issue=6&spage=1232&pages=1232-1239&date=2009&title=Analyst&atitle=Spectroscopic+diagnosis+of+laryngeal+carcinoma+using+near-infrared+Raman+spectroscopy+and+random+recursive+partitioning+ensemble+techniques&aulast=Teh&pid=<author>Teh+S.K.%3BZheng+W.%3BLau+D.P.%3BHuang+Z.<%2Fauthor><AN>354733574<%2FAN><DT>Article<%2FDT>)

352.

Surfing the wave.

Heber J.

Nature. 461(7265) (pp 720-722), 2009. Date of Publication: 08 Oct 2009.

AN: 355417373

PMID

19812649 [<https://www.ncbi.nlm.nih.gov/pubmed/?term=19812649>]

Publisher

Nature Publishing Group (Houndmills, Basingstoke, Hampshire RG21 6XS, United Kingdom)

Emtree Heading

cancer therapy; electrical equipment; electron; head and neck cancer; human; nanosensor; *nanotechnology; nonhuman; oscillation; priority journal; Raman spectrometry; short survey; solar energy; surface plasmon resonance; telecommunication; nanoparticle; *plasmonics.

Candidate Terms

*plasmonics [other term]

Drug Index Terms

nanoparticle.

Other Index Terms

cancer therapy; electrical equipment; electron; head and neck cancer; human; nanosensor; *nanotechnology; nonhuman; oscillation; priority journal; Raman spectrometry; short survey; solar energy; surface plasmon resonance; telecommunication.

Link to the Ovid Full Text or citation:

[Click here for full text options](https://ovidsp.ovid.com/ovidweb.cgi?T=JS&CSC=Y&NEWS=N&PAGE=fulltext&D=emed11&AN=355417373)

Link to the External Link Resolver:

[SFX](https://sfx-86scu.hosted.exlibrisgroup.com.cn/86scu?sid=OVID:embase&id=pmid:19812649&id=doi:10.1038%2F461720a&issn=00280836&isbn=&volume=461&issue=7265&spage=720&pages=720-722&date=2009&title=Nature&atitle=Surfing+the+wave&aulast=Heber&pid=<author>Heber+J.<%2Fauthor><AN>355417373<%2FAN><DT>Short+Survey<%2FDT>)

353.

Gold nanoparticle based surface-enhanced Raman scattering Spectroscopy of cancerous and normal nasopharyngeal tissues under near-infrared laser excitation.

Feng S., Lin J., Cheng M., Li Y.-Z., Chen G., Huang Z., Yu Y., Chen R., Zeng H.

Applied Spectroscopy. 63(10) (pp 1089-1094), 2009. Date of Publication: 2009.

AN: 355684887

The capabilities of using gold nanoparticle based near-infrared surfaceenhanced Raman scattering (SERS) to obtain biochemical information with high spatial resolution from human nasopharyngeal tissue were presented in this paper. The gold nanoparticles used have a mean diameter of 43 nm with a standard deviation of 6 nm. The SERS bands of nasopharyngeal tissue were assigned to known molecular vibrations of nucleic acids, amino acids, proteins, and metabolites. We also observed the blinking phenomenon at the tissue level when measuring the nasopharyngeal tissue SERS spectra, most frequently in signal intensity but also occasionally in peak positions. This phenomenon is excitation light intensity dependent. This work demonstrated great potential for using SERS imaging for distinguishing cancerous and normal nasopharyngeal tissues on frozen sections without using any dye labeling or other chemical species as functionalized binding sites. © 2009 Society for Applied Spectroscopy.

PMID

19843357 [<https://www.ncbi.nlm.nih.gov/pubmed/?term=19843357>]

Institution

(Feng, Lin, Li, Chen, Huang, Yu, Chen) Key Laboratory of OptoElectronic Science and Technology for Medicine, Ministry of Education, Fujian Normal University, Fuzhou 350007, China (Feng) College of Chemistry and Material Science, Experimental Center, Fujian Normal University, Fuzhou 350007, China

(Cheng) College of Minjiang, Fuzhou 350001, China

(Zeng) Cancer Imaging Department, British Columbia Cancer Research Centre, Vancouver, BC, Canada

Publisher

Society for Applied Spectroscopy (201B Broadway Street, Frederick MD 21702-6501, United States)

Emtree Heading

article; chemistry; human; laser; methodology; *nasopharynx; *nasopharynx tumor; near infrared spectroscopy; particle size; *Raman spectrometry; surface property; transmission electron microscopy; ultrastructure; adenine; amino acid; *gold; *metal nanoparticle; thymine.

Drug Index Terms

adenine; amino acid; *gold; *metal nanoparticle; thymine.

Other Index Terms

article; chemistry; human; laser; methodology; *nasopharynx; *nasopharynx tumor; near infrared spectroscopy; particle size; *Raman spectrometry; surface property; transmission electron microscopy; ultrastructure.

Link to the Ovid Full Text or citation:

[Click here for full text options](https://ovidsp.ovid.com/ovidweb.cgi?T=JS&CSC=Y&NEWS=N&PAGE=fulltext&D=emed11&AN=355684887)

Link to the External Link Resolver:

[SFX](https://sfx-86scu.hosted.exlibrisgroup.com.cn/86scu?sid=OVID:embase&id=pmid:19843357&id=doi:10.1366%2F000370209789553291&issn=00037028&isbn=&volume=63&issue=10&spage=1089&pages=1089-1094&date=2009&title=Applied+Spectroscopy&atitle=Gold+nanoparticle+based+surface-enhanced+Raman+scattering+Spectroscopy+of+cancerous+and+normal+nasopharyngeal+tissues+under+near-infrared+laser+excitation&aulast=Feng&pid=<author>Feng+S.%3BLin+J.%3BCheng+M.%3BLi+Y.-Z.%3BChen+G.%3BHuang+Z.%3BYu+Y.%3BChen+R.%3BZeng+H.<%2Fauthor><AN>355684887<%2FAN><DT>Article<%2FDT>)

354.

Measurement of nasopharyngeal carcinoma tissue ex vivo by Raman spectroscopy.

Huang W., Pan J.J., Chen R., Li Y.Z., Feng S.Y., Xie S.S., Zeng H.S.

Guang pu xue yu guang pu fen xi = Guang pu. 29(5) (pp 1304-1307), 2009. Date of Publication: May 2009.

AN: 355895964

Raman spectroscopy has shown its potential and advantages in detecting molecular changes associated with tissue pathology, which makes it possible to diagnose with optical methods non-invasively and real-time. A compact and rapid near-infrared (NIR)Raman system was developed using 785 nm diode laser, volume phase technology (VPT)holographic grating system and NIR intensified charge-coupled device (CCD)with a specially designed Raman fibre probe which can effectively reduce the interference of fluorescence and Rayleigh scattering, maximize the ability of Raman collection as well as correct the image aberration of a planar grating diffraction. Adopting this method, signal-to-noise ratio has been greatly improved and human tissue signals can be acquired in a short time. Raman signals from fat and musculature of fresh pork were measured and referenced for further optimization, then Raman spectra of nasopharyngeal carcinoma in vitro and the effect of storage time on them were measured in 1-5 s and discussed. The sensitivities and performance of the system will be further enhanced and more Raman data will be acquired and compared between normal and cancerous nasopharyngeal tissue, expecting to discover the statistical characteristics, which will benefit the diagnosis and treatment of early nasopharyngeal carcinoma or other tumors.

PMID

19650477 [<https://www.ncbi.nlm.nih.gov/pubmed/?term=19650477>]

Institution

(Huang, Pan, Chen, Li, Feng, Xie, Zeng) Key Laboratory of Optoelectronic Science and Technology for Medicine (Fujian Normal University), Ministry of Education, Fuzhou 350007, China.

Emtree Heading

adult; aged; article; human; middle aged; *nasopharynx tumor; pathology; *Raman spectrometry; time.

Other Index Terms

adult; aged; article; human; middle aged; *nasopharynx tumor; pathology; *Raman spectrometry; time.

Link to the Ovid Full Text or citation:

[Click here for full text options](https://ovidsp.ovid.com/ovidweb.cgi?T=JS&CSC=Y&NEWS=N&PAGE=fulltext&D=emed11&AN=355895964)

Link to the External Link Resolver:

[SFX](https://sfx-86scu.hosted.exlibrisgroup.com.cn/86scu?sid=OVID:embase&id=pmid:19650477&id=doi:&issn=10000593&isbn=&volume=29&issue=5&spage=1304&pages=1304-1307&date=2009&title=Guang+pu+xue+yu+guang+pu+fen+xi+%3D+Guang+pu&atitle=Measurement+of+nasopharyngeal+carcinoma+tissue+ex+vivo+by+Raman+spectroscopy&aulast=Huang&pid=<author>Huang+W.%3BPan+J.J.%3BChen+R.%3BLi+Y.Z.%3BFeng+S.Y.%3BXie+S.S.%3BZeng+H.S.<%2Fauthor><AN>355895964<%2FAN><DT>Article<%2FDT>)

355.

Raman spectroscopy and advanced mathematical modelling in the discrimination of human thyroid cell lines.

Harris A.T., Garg M., Yang X.B., Fisher S.E., Kirkham J., Smith D.A., Martin-Hirsch D.P., High A.S.

Head & neck oncology. 1 (pp 38), 2009. Date of Publication: 2009.

AN: 355900757

Raman spectroscopy could offer non-invasive, rapid and an objective nature to cancer diagnostics. However, much work in this field has focused on resolving differences between cancerous and non-cancerous tissues, and lacks the reproducibility and interpretation to be put into clinical practice. Much work is needed on basic cellular differences between malignancy and normal. This would allow the establishment of a clinically relevant cellular based model to translate to tissue classification. Raman spectroscopy provides a very detailed biochemical analysis of the target material and to 'unlock' this potential requires sophisticated mathematical modelling such as neural networks as an adjunct to data interpretation. Commercially obtained cancerous and non-cancerous cells, cultured in the laboratory were used in Raman spectral measurements. Data trends were visualised through PCA and then subjected to neural network analysis based on self-organising maps; consisting of m maps, where m is the number of classes to be recognised. Each map approximates the statistical distribution of a given class. The neural network analysis provided a 95% accuracy for identification of the cancerous cell line and 92% accuracy for normal cell line. In this preliminary study we have demonstrated th ability to distinguish between "normal" and cancerous commercial cell lines. This encourages future work to establish the reasons underpinning these spectral differences and to move forward to more complex systems involving tissues. We have also shown that the use of sophisticated mathematical modelling allows a high degree of discrimination of 'raw' spectral data.

PMID

19863815 [<https://www.ncbi.nlm.nih.gov/pubmed/?term=19863815>]

Institution

(Harris) Department of Oral Biology, Level 6 Worsley Building, University of Leeds, Clarendon Way, Leeds, LS2 9LU, UK.

Emtree Heading

article; *artificial neural network; cell line; cytology; human; methodology; pathology; principal component analysis; *Raman spectrometry; theoretical model; *thyroid gland; *thyroid tumor.

Other Index Terms

article; *artificial neural network; cell line; cytology; human; methodology; pathology; principal component analysis; *Raman spectrometry; theoretical model; *thyroid gland; *thyroid tumor.

Link to the Ovid Full Text or citation:

[Click here for full text options](https://ovidsp.ovid.com/ovidweb.cgi?T=JS&CSC=Y&NEWS=N&PAGE=fulltext&D=emed11&AN=355900757)

Link to the External Link Resolver:

[SFX](https://sfx-86scu.hosted.exlibrisgroup.com.cn/86scu?sid=OVID:embase&id=pmid:19863815&id=doi:10.1186%2F1758-3284-1-38&issn=17583284&isbn=&volume=1&issue=&spage=38&pages=38&date=2009&title=Head+%26+neck+oncology&atitle=Raman+spectroscopy+and+advanced+mathematical+modelling+in+the+discrimination+of+human+thyroid+cell+lines&aulast=Harris&pid=<author>Harris+A.T.%3BGarg+M.%3BYang+X.B.%3BFisher+S.E.%3BKirkham+J.%3BSmith+D.A.%3BMartin-Hirsch+D.P.%3BHigh+A.S.<%2Fauthor><AN>355900757<%2FAN><DT>Article<%2FDT>)

356.

Potential for Raman spectroscopy to provide cancer screening using a peripheral blood sample.

Harris A.T., Lungari A., Needham C.J., Smith S.L., Lones M.A., Fisher S.E., Yang X.B., Cooper N., Kirkham J., Smith D.A., Martin-Hirsch D.P., High A.S.

Head & neck oncology. 1 (pp 34), 2009. Date of Publication: 2009.

AN: 355900754

Cancer poses a massive health burden with incidence rates expected to double globally over the next decade. In the United Kingdom screening programmes exists for cervical, breast, and colorectal cancer. The ability to screen individuals for solid malignant tumours using only a peripheral blood sample would revolutionise cancer services and permit early diagnosis and intervention. Raman spectroscopy interrogates native biochemistry through the interaction of light with matter, producing a high definition biochemical 'fingerprint' of the target material. This paper explores the possibility of using Raman spectroscopy to discriminate between cancer and non-cancer patients through a peripheral blood sample. Forty blood samples were obtained from patients with Head and Neck cancer and patients with respiratory illnesses to act as a positive control. Raman spectroscopy was carried out on all samples with the resulting spectra being used to build a classifier in order to distinguish between the cancer and respiratory patients' spectra; firstly using principal component analysis (PCA)/linear discriminant analysis (LDA), and secondly with a genetic evolutionary algorithm. The PCA/LDA classifier gave a 65% sensitivity and specificity for discrimination between the cancer and respiratory groups. A sensitivity score of 75% with a specificity of 75% was achieved with a 'trained' evolutionary algorithm. In conclusion this preliminary study has demonstrated the feasibility of using Raman spectroscopy in cancer screening and diagnostics of solid tumours through a peripheral blood sample. Further work needs to be carried out for this technique to be implemented in the clinical setting.

PMID

19761601 [<https://www.ncbi.nlm.nih.gov/pubmed/?term=19761601>]

Institution

(Harris) Oral Biology, Leeds Dental Institute, University of Leeds, Leeds, UK.

Emtree Heading

article; blood; *early diagnosis; female; human; male; methodology; middle aged; *neoplasm/di [Diagnosis]; principal component analysis; *Raman spectrometry.

Other Index Terms

article; blood; *early diagnosis; female; human; male; methodology; middle aged; *neoplasm / *diagnosis; principal component analysis; *Raman spectrometry.

Link to the Ovid Full Text or citation:

[Click here for full text options](https://ovidsp.ovid.com/ovidweb.cgi?T=JS&CSC=Y&NEWS=N&PAGE=fulltext&D=emed11&AN=355900754)

Link to the External Link Resolver:

[SFX](https://sfx-86scu.hosted.exlibrisgroup.com.cn/86scu?sid=OVID:embase&id=pmid:19761601&id=doi:10.1186%2F1758-3284-1-34&issn=17583284&isbn=&volume=1&issue=&spage=34&pages=34&date=2009&title=Head+%26+neck+oncology&atitle=Potential+for+Raman+spectroscopy+to+provide+cancer+screening+using+a+peripheral+blood+sample&aulast=Harris&pid=<author>Harris+A.T.%3BLungari+A.%3BNeedham+C.J.%3BSmith+S.L.%3BLones+M.A.%3BFisher+S.E.%3BYang+X.B.%3BCooper+N.%3BKirkham+J.%3BSmith+D.A.%3BMartin-Hirsch+D.P.%3BHigh+A.S.<%2Fauthor><AN>355900754<%2FAN><DT>Article<%2FDT>)

357.

Head & neck optical diagnostics: vision of the future of surgery.

Upile T., Jerjes W., Sterenborg H.J., El-Naggar A.K., Sandison A., Witjes M.J., Biel M.A., Bigio I., Wong B.J., Gillenwater A., MacRobert A.J., Robinson D.J., Betz C.S., Stepp H., Bolotine L., McKenzie G., Mosse C.A., Barr H., Chen Z., Berg K., D'Cruz A.K., Stone N., Kendall C., Fisher S., Leunig A., Olivo M., Richards-Kortum R., Soo K.C., Bagnato V., Choo-Smith L.P., Svanberg K., Tan I.B., Wilson B.C., Wolfsen H., Yodh A.G., Hopper C.

Head & neck oncology. 1 (pp 25), 2009. Date of Publication: 2009.

AN: 355900747

Review paper and Proceedings of the Inaugural Meeting of the Head and Neck Optical Diagnostics Society (HNODS) on March 14th 2009 at University College London. The aim of our research must be to provide breakthrough translational research which can be applied clinically in the immediate rather than the near future. We are fortunate that this is indeed a possibility and may fundamentally change current clinical and surgical practice to improve our patients' lives.

PMID

19594907 [<https://www.ncbi.nlm.nih.gov/pubmed/?term=19594907>]

Institution

(Upile) The Head and Neck Optical Diagnostics Society Council, Head & Neck Centre, University College Hospital, 250 Euston Road, London, NW1 2PG, UK.

Emtree Heading

article; confocal microscopy; fluorescence; *head and neck tumor/di [Diagnosis]; *head and neck tumor/su [Surgery]; human; infrared spectrophotometry; optical coherence tomography; pathology; Raman spectrometry; spectroscopy.

Other Index Terms

article; confocal microscopy; fluorescence; *head and neck tumor / *diagnosis / *surgery; human; infrared spectrophotometry; optical coherence tomography; pathology; Raman spectrometry; spectroscopy.

Link to the Ovid Full Text or citation:

[Click here for full text options](https://ovidsp.ovid.com/ovidweb.cgi?T=JS&CSC=Y&NEWS=N&PAGE=fulltext&D=emed11&AN=355900747)

Link to the External Link Resolver:

[SFX](https://sfx-86scu.hosted.exlibrisgroup.com.cn/86scu?sid=OVID:embase&id=pmid:19594907&id=doi:10.1186%2F1758-3284-1-25&issn=17583284&isbn=&volume=1&issue=&spage=25&pages=25&date=2009&title=Head+%26+neck+oncology&atitle=Head+%26+neck+optical+diagnostics%3A+vision+of+the+future+of+surgery&aulast=Upile&pid=<author>Upile+T.%3BJerjes+W.%3BSterenborg+H.J.%3BEl-Naggar+A.K.%3BSandison+A.%3BWitjes+M.J.%3BBiel+M.A.%3BBigio+I.%3BWong+B.J.%3BGillenwater+A.%3BMacRobert+A.J.%3BRobinson+D.J.%3BBetz+C.S.%3BStepp+H.%3BBolotine+L.%3BMcKenzie+G.%3BMosse+C.A.%3BBarr+H.%3BChen+Z.%3BBerg+K.%3BD'Cruz+A.K.%3BStone+N.%3BKendall+C.%3BFisher+S.%3BLeunig+A.%3BOlivo+M.%3BRichards-Kortum+R.%3BSoo+K.C.%3BBagnato+V.%3BChoo-Smith+L.P.%3BSvanberg+K.%3BTan+I.B.%3BWilson+B.C.%3BWolfsen+H.%3BYodh+A.G.%3BHopper+C.<%2Fauthor><AN>355900747<%2FAN><DT>Article<%2FDT>)

358.

The role of molecular strategies in the evaluation of surgical margins in oropharyngeal squamous cell carcinoma.

Kanatas A.N., Harris A.T., Messenger M., Houghton D., Rogers S.N.

Oncology Reviews. 3(3) (pp 161-167), 2009. Date of Publication: September 2009.

AN: 50625689

The recurrence of a tumour at the resection margins in head and neck squamous cell carcinoma (HNSCC) has profound implications on the morbidity and mortality of the patient. At present HNSCC does not undergo any form of molecular analysis to aid treatment strategy and prognosticate for those individuals at higher risk of recurrence. This article aims to review current research into molecular strategies for tumour evaluation, highlighting conflicting evidence and possible novel concepts for further exploration. © 2009 Springer-Verlag.

Institution

(Kanatas) Oral and Maxillofacial Department, Leeds Dental Institute, Worsley Building, Clarendon Way, Leeds LS2 9LU, United Kingdom (Harris) Oral Biology, University of Leeds, Level 6 Worsley Building, Clarendon Way, Leeds LS2 9LU, United Kingdom

(Messenger) Department of Oral Biology, Tissue Engineering Research, Leeds Dental Institute, Leeds LS2 9LU, United Kingdom

(Houghton) Oral and Facial Specialties Department, Pinderfields General Hospital, Aberford Road, Wakefield WF1 4DG, United Kingdom

(Rogers) Faculty of Health, Edge Hill University, University Hospital Aintree, Liverpool L9 7LN, United Kingdom

Publisher

Springer Milan (Via Podgora 4, Milan I-20122, Italy)

Emtree Heading

cancer surgery; DNA methylation; epigenetics; flow cytometry; gene expression; gene locus; gene mutation; gene silencing; genome analysis; human; immunohistochemistry; infrared spectroscopy; microsatellite marker; *oropharynx carcinoma/su [Surgery]; priority journal; promoter region; Raman spectrometry; review; *squamous cell carcinoma/su [Surgery]; stem cell; tumor suppressor gene; Wart virus; cyclin dependent kinase inhibitor 2A; Ki 67 antigen/ec [Endogenous Compound]; methylated DNA protein cysteine methyltransferase; protein p53; Ras association domain family protein 1A; *tumor marker/ec [Endogenous Compound].

Drug Index Terms

cyclin dependent kinase inhibitor 2A; Ki 67 antigen / endogenous compound; methylated DNA protein cysteine methyltransferase; protein p53; Ras association domain family protein 1A; *tumor marker / *endogenous compound.

Other Index Terms

cancer surgery; DNA methylation; epigenetics; flow cytometry; gene expression; gene locus; gene mutation; gene silencing; genome analysis; human; immunohistochemistry; infrared spectroscopy; microsatellite marker; *oropharynx carcinoma / *surgery; priority journal; promoter region; Raman spectrometry; review; *squamous cell carcinoma / *surgery; stem cell; tumor suppressor gene; Wart virus.

Link to the Ovid Full Text or citation:

[Click here for full text options](https://ovidsp.ovid.com/ovidweb.cgi?T=JS&CSC=Y&NEWS=N&PAGE=fulltext&D=emed11&AN=50625689)

Link to the External Link Resolver:

[SFX](https://sfx-86scu.hosted.exlibrisgroup.com.cn/86scu?sid=OVID:embase&id=pmid:&id=doi:10.1007%2Fs12156-009-0020-9&issn=19705557&isbn=&volume=3&issue=3&spage=161&pages=161-167&date=2009&title=Oncology+Reviews&atitle=The+role+of+molecular+strategies+in+the+evaluation+of+surgical+margins+in+oropharyngeal+squamous+cell+carcinoma&aulast=Kanatas&pid=<author>Kanatas+A.N.%3BHarris+A.T.%3BMessenger+M.%3BHoughton+D.%3BRogers+S.N.<%2Fauthor><AN>50625689<%2FAN><DT>Review<%2FDT>)

359.

Peptide-conjugated gold nanorods for nuclear targeting.

Oyelere A.K., Chen P.C., Huang X., El-Sayed I.H., El-Sayed M.A.

Bioconjugate Chemistry. 18(5) (pp 1490-1497), 2007. Date of Publication: September/October 2007.

AN: 47464056

Resonant electron oscillations on the surface of noble metal nanoparticles (Au, Ag, Cu) create the surface plasmon resonance (SPR) that greatly enhances the absorption and Rayleigh (Mie) scattering of light by these particles. By adjusting the size and shape of the particles from spheres to rods, the SPR absorption and scattering can be tuned from the visible to the near-infrared region (NIR) where biologic tissues are relatively transparent. Further, gold nanorods greatly enhance surface Raman scattering of adsorbed molecules. These unique properties make gold nanorods especially attractive as optical sensors for biological and medical applications. In the present work, gold nanorods are covalently conjugated with a nuclear localization signal peptide through a thioalkyl-triazole linker and incubated with an immortalized benign epithelial cell line and an oral cancer cell line. Dark field light SPR scattering images demonstrate that nanorods are located in both the cytoplasm and nucleus of both cell lines. Single cell micro-Raman spectra reveal enhanced Raman bands of the peptide as well as molecules in the cytoplasm and the nucleus. Further, the Raman spectra reveal a difference between benign and cancer cell lines. This work represents an important step toward both imaging and Raman-based intracellular biosensing with covalently linked ligand-nanorod probes. © 2007 American Chemical Society.

PMID

17630680 [<https://www.ncbi.nlm.nih.gov/pubmed/?term=17630680>]

Institution

(Oyelere, Chen) Parker H. Petit Institute for Bioengineering and Biosciences, School of Chemistry and Biochemistry, Georgia Institute of Technology, Atlanta, GA 30332, United States (Huang, El-Sayed) Laser Dynamics Laboratory, School of Chemistry and Biochemistry, Georgia Institute of Technology, 901 Atlantic Drive, Atlanta, GA 30332, United States

(El-Sayed) Otolaryngology-Head and Neck Surgery, Comprehensive Cancer Center, University of California, San Francisco, 400 Parnassus Avenue, San Francisco, CA 94143-0342, United States

Publisher

American Chemical Society

Emtree Heading

article; biosensor; cancer cell; cell differentiation; cell immortalization; cell nucleus; *conjugation; covalent bond; cytoplasm; epithelium cell; human; human cell; incubation time; interferometer; intracellular transport; ligand binding; light scattering; *metal binding; molecular model; molecular sensor; mouth cancer/et [Etiology]; near infrared spectroscopy; *nuclear localization signal; oscillation; *protein binding; Raman spectrometry; signal detection; Simian virus 40; surface plasmon resonance; surface property; copper; *gold; nanoparticle; *nanorod; *signal peptide; silver; triazole; dark field light scattering; micro Raman spectroscopy.

Candidate Terms

dark field light scattering [other term]	micro Raman spectroscopy [other term]

Drug Index Terms

copper; *gold; nanoparticle; *nanorod; *signal peptide; silver; triazole.

Other Index Terms

article; biosensor; cancer cell; cell differentiation; cell immortalization; cell nucleus; *conjugation; covalent bond; cytoplasm; epithelium cell; human; human cell; incubation time; interferometer; intracellular transport; ligand binding; light scattering; *metal binding; molecular model; molecular sensor; mouth cancer / etiology; near infrared spectroscopy; *nuclear localization signal; oscillation; *protein binding; Raman spectrometry; signal detection; Simian virus 40; surface plasmon resonance; surface property.

Link to the Ovid Full Text or citation:

[Click here for full text options](https://ovidsp.ovid.com/ovidweb.cgi?T=JS&CSC=Y&NEWS=N&PAGE=fulltext&D=emed10&AN=47464056)

Link to the External Link Resolver:

[SFX](https://sfx-86scu.hosted.exlibrisgroup.com.cn/86scu?sid=OVID:embase&id=pmid:17630680&id=doi:10.1021%2Fbc070132i&issn=10431802&isbn=&volume=18&issue=5&spage=1490&pages=1490-1497&date=2007&title=Bioconjugate+Chemistry&atitle=Peptide-conjugated+gold+nanorods+for+nuclear+targeting&aulast=Oyelere&pid=<author>Oyelere+A.K.%3BChen+P.C.%3BHuang+X.%3BEl-Sayed+I.H.%3BEl-Sayed+M.A.<%2Fauthor><AN>47464056<%2FAN><DT>Article<%2FDT>)

360.

Cancer cells assemble and align gold nanorods conjugated to antibodies to produce highly enhanced, sharp, and polarized surface Raman spectra: a potential cancer diagnostic marker.

Huang X., El-Sayed I.H., Qian W., El-Sayed M.A.

Nano letters. 7(6) (pp 1591-1597), 2007. Date of Publication: Jun 2007.

AN: 47286050

Human oral cancer cells are found to assemble and align gold nanorods conjugated to anti-epidermal growth factor receptor (anti-EGFR) antibodies. Immnoconjugated gold nanorods and nanospheres were shown previously to exhibit strong Rayleigh (Mie) scattering useful for imaging. In the present letter, molecules near the nanorods on the cancer cells are found to give a Raman spectrum that is greatly enhanced (due to the high surface plasmon field of the nanorod assembly in which their extended surface plasmon fields overlap), sharp (due to a homogeneous environment), and polarized (due to anisotropic alignments). These observed properties can be used as diagnostic signatures for cancer cells.

PMID

17474783 [<https://www.ncbi.nlm.nih.gov/pubmed/?term=17474783>]

Institution

(Huang, El-Sayed, Qian, El-Sayed) Laser Dynamics Laboratory, School of Chemistry and Biochemistry, Georgia Institute of Technology, Atlanta, Georgia 30332, USA.

Emtree Heading

article; chemistry; evaluation; *genetic procedures; human; immunoassay; immunology; methodology; nanotechnology; pathology; *Raman spectrometry; *refractometry; *squamous cell carcinoma; tumor cell line; ultrastructure; contrast medium; diagnostic agent; *epidermal growth factor receptor; *gold; *nanotube; unclassified drug; HER1 protein, human.

Candidate Terms

HER1 protein, human [drug term]

Drug Index Terms

contrast medium; diagnostic agent; *epidermal growth factor receptor; *gold; *nanotube; unclassified drug.

Other Index Terms

article; chemistry; evaluation; *genetic procedures; human; immunoassay; immunology; methodology; nanotechnology; pathology; *Raman spectrometry; *refractometry; *squamous cell carcinoma; tumor cell line; ultrastructure.

Link to the Ovid Full Text or citation:

[Click here for full text options](https://ovidsp.ovid.com/ovidweb.cgi?T=JS&CSC=Y&NEWS=N&PAGE=fulltext&D=emed10&AN=47286050)

Link to the External Link Resolver:

[SFX](https://sfx-86scu.hosted.exlibrisgroup.com.cn/86scu?sid=OVID:embase&id=pmid:17474783&id=doi:10.1021%2Fnl070472c&issn=15306984&isbn=&volume=7&issue=6&spage=1591&pages=1591-1597&date=2007&title=Nano+letters&atitle=Cancer+cells+assemble+and+align+gold+nanorods+conjugated+to+antibodies+to+produce+highly+enhanced%2C+sharp%2C+and+polarized+surface+Raman+spectra%3A+a+potential+cancer+diagnostic+marker&aulast=Huang&pid=<author>Huang+X.%3BEl-Sayed+I.H.%3BQian+W.%3BEl-Sayed+M.A.<%2Fauthor><AN>47286050<%2FAN><DT>Article<%2FDT>)

361.

Study of Raman spectra of single carcinoma of nasopharynx cell.

Yao H.L., Zhu M., Wang G.W., Peng L.X., He B.J., He M., Li Y.Q.

Guang pu xue yu guang pu fen xi = Guang pu. 27(9) (pp 1761-1764), 2007. Date of Publication: Sep 2007.

AN: 350359435

The Raman spectra from carcinoma of nasopharynx cell lines (CNE2) and normal airway epithelial cell lines (HBE) were investigated using a laser tweezers Raman spectroscopy (LTRS). The Raman scattering measurements were obtained from three different places in every single cell. Visual inspection of the spectra shows that the differences observed in spectra of the cancer cells and normal cells are obvious. The peak ratio I1 304/I1 336 is 1.05 for the normal cell and 1.22 for the cancer cell. Using a combination of principal component analysis (PCA) and discriminant function analysis (DFA), the authors are able to predict cancer cells, and normal cells and the DFA is better for single Raman spectrum. The sampling locations did not seriously affect the result of PCA and DFA. PCA and DFA also show that the uniformity of normal cells is better than that of cancer cells. The results indicate that the Raman spectra may offer the experimental basis for colorectal cancer diagnosis.

PMID

18051524 [<https://www.ncbi.nlm.nih.gov/pubmed/?term=18051524>]

Institution

(Yao, Zhu, Wang, Peng, He, He, Li) Laboratory for Biophysics, Guangxi Academy of Sciences, Nanning 530003, China.

Emtree Heading

article; *carcinoma/di [Diagnosis]; *cell; chemistry; cytology; *diagnostic procedure; evaluation; human; methodology; *nasopharynx tumor/di [Diagnosis]; *Raman spectrometry; tumor cell line.

Other Index Terms

article; *carcinoma / *diagnosis; *cell; chemistry; cytology; *diagnostic procedure; evaluation; human; methodology; *nasopharynx tumor / *diagnosis; *Raman spectrometry; tumor cell line.

Link to the Ovid Full Text or citation:

[Click here for full text options](https://ovidsp.ovid.com/ovidweb.cgi?T=JS&CSC=Y&NEWS=N&PAGE=fulltext&D=emed10&AN=350359435)

Link to the External Link Resolver:

[SFX](https://sfx-86scu.hosted.exlibrisgroup.com.cn/86scu?sid=OVID:embase&id=pmid:18051524&id=doi:&issn=10000593&isbn=&volume=27&issue=9&spage=1761&pages=1761-1764&date=2007&title=Guang+pu+xue+yu+guang+pu+fen+xi+%3D+Guang+pu&atitle=Study+of+Raman+spectra+of+single+carcinoma+of+nasopharynx+cell&aulast=Yao&pid=<author>Yao+H.L.%3BZhu+M.%3BWang+G.W.%3BPeng+L.X.%3BHe+B.J.%3BHe+M.%3BLi+Y.Q.<%2Fauthor><AN>350359435<%2FAN><DT>Article<%2FDT>)

362.

Diagnosis of nonmelanoma skin cancer/keratinocyte carcinoma: A review of diagnostic accuracy of nonmelanoma skin cancer diagnostic tests and technologies.

Mogensen M., Jemec G.B.E.

Dermatologic Surgery. 33(10) (pp 1158-1174), 2007. Date of Publication: October 2007.

AN: 47508663

BACKGROUND: Nonmelanoma skin cancer (NMSC) is the most prevalent cancer in the light-skinned population. Noninvasive treatment is increasingly used for NMSC patients with superficial lesions, making the development of noninvasive diagnostic technologies highly relevant.

OBJECTIVE(S): The scope of this review is to present data on the current state-of-the-art diagnostic methods for keratinocyte carcinoma: basal cell carcinoma, squamous cell carcinoma, and actinic keratosis. METHODS AND MATERIALS: MEDLINE, BIOSIS, and EMBASE searches on NMSC and physical and clinical examination, biopsy, molecular marker, ultrasonography, Doppler, optical coherence tomography, dermoscopy, spectroscopy, fluorescence imaging, confocal microscopy, positron emission tomography, computed tomography, magnetic resonance imaging, terahertz imaging, electrical impedance and sensitivity, specificity, and diagnostic accuracy.

RESULT(S): State-of-the-art diagnostic research has been limited in this field, but encouraging results from the reviewed diagnostic trials have suggested a high diagnostic accuracy for many of the technologies. Most of the studies, however, were pilot or small studies and the results would need to be validated in larger trials.

CONCLUSION(S): Some of these new imaging technologies have the capability of providing new, three-dimensional in vivo, in situ understanding of NMSC development over time. Some of the new technologies described here have the potential to make it from the bench to the clinic. © 2007 by the American Society for Dermatologic Surgery, Inc.

PMID

17903149 [<https://www.ncbi.nlm.nih.gov/pubmed/?term=17903149>]

Institution

(Mogensen, Jemec) Department of Dermatology, University of Copenhagen, Roskilde Hospital, Roskilde, Denmark (Mogensen) Department of Dermatology, University of Copenhagen, Roskilde Hospital, Kogevej 7-13, DK-4000 Roskilde, Denmark

Publisher

Blackwell Publishing Ltd (9600 Garsington Road, Oxford OX4 2XG, United Kingdom)

Emtree Heading

actinic keratosis/di [Diagnosis]; article; *basal cell carcinoma/di [Diagnosis]; Bowen disease/di [Diagnosis]; *cancer diagnosis; cancer survival; clinical examination; clinical trial; computer assisted tomography; confocal microscopy; diagnostic accuracy; diagnostic imaging; *diagnostic test; diagnostic value; diffuse reflectance spectroscopy; Doppler echography; echography; Embase; epiluminescence microscopy; eyelid tumor/di [Diagnosis]; fluorescence analysis; human; impedance; infrared spectroscopy; intermethod comparison; keratinocyte; medical literature; medical research; Medline; melanoma; near infrared spectroscopy; non invasive procedure; nose cancer/di [Diagnosis]; nuclear magnetic resonance imaging; optical coherence tomography; physical examination; positron emission tomography; priority journal; radiodiagnosis; Raman spectrometry; sensitivity and specificity; skin biopsy; *skin cancer/di [Diagnosis]; *skin carcinoma/di [Diagnosis]; spectroscopy; *squamous cell carcinoma/di [Diagnosis]; survival time; validity; fluorodeoxyglucose f 18; molecular marker/ec [Endogenous Compound]; terahertz pulsed imaging.

Candidate Terms

terahertz pulsed imaging [other term]

Drug Index Terms

fluorodeoxyglucose f 18; molecular marker / endogenous compound.

Other Index Terms

actinic keratosis / diagnosis; article; *basal cell carcinoma / *diagnosis; Bowen disease / diagnosis; *cancer diagnosis; cancer survival; clinical examination; clinical trial; computer assisted tomography; confocal microscopy; diagnostic accuracy; diagnostic imaging; *diagnostic test; diagnostic value; diffuse reflectance spectroscopy; Doppler echography; echography; EMBASE; epiluminescence microscopy; eyelid tumor / diagnosis; fluorescence analysis; human; impedance; infrared spectroscopy; intermethod comparison; keratinocyte; medical literature; medical research; MEDLINE; melanoma; near infrared spectroscopy; non invasive procedure; nose cancer / diagnosis; nuclear magnetic resonance imaging; optical coherence tomography; physical examination; positron emission tomography; priority journal; radiodiagnosis; Raman spectrometry; sensitivity and specificity; skin biopsy; *skin cancer / *diagnosis; *skin carcinoma / *diagnosis; spectroscopy; *squamous cell carcinoma / *diagnosis; survival time; validity.

Link to the Ovid Full Text or citation:

[Click here for full text options](https://ovidsp.ovid.com/ovidweb.cgi?T=JS&CSC=Y&NEWS=N&PAGE=fulltext&D=emed10&AN=47508663)

Link to the External Link Resolver:

[SFX](https://sfx-86scu.hosted.exlibrisgroup.com.cn/86scu?sid=OVID:embase&id=pmid:17903149&id=doi:10.1111%2Fj.1524-4725.2007.33251.x&issn=10760512&isbn=&volume=33&issue=10&spage=1158&pages=1158-1174&date=2007&title=Dermatologic+Surgery&atitle=Diagnosis+of+nonmelanoma+skin+cancer%2Fkeratinocyte+carcinoma%3A+A+review+of+diagnostic+accuracy+of+nonmelanoma+skin+cancer+diagnostic+tests+and+technologies&aulast=Mogensen&pid=<author>Mogensen+M.%3BJemec+G.B.E.<%2Fauthor><AN>47508663<%2FAN><DT>Article<%2FDT>)

363.

Metabolic fingerprinting as a diagnostic tool.

Ellis D.I., Dunn W.B., Griffin J.L., Allwood J.W., Goodacre R.

Pharmacogenomics. 8(9) (pp 1243-1266), 2007. Date of Publication: September 2007.

AN: 47578232

Within the framework of systems biology, functional analyses at all 'omic levels have seen an intense level of activity during the first decade of the twenty-first century. These include genomics, transcriptomics, proteomics, metabolomics and lipidomics. It could be said that metabolomics offers some unique advantages over the other 'omics disciplines and one of the core approaches of metabolomics for disease diagnostics is metabolic fingerprinting. This review provides an overview of the main metabolic fingerprinting approaches used for disease diagnostics and includes: infrared and Raman spectroscopy, Nuclear magnetic resonance (NMR) spectroscopy, followed by an introduction to a wide range of novel mass spectrometry-based methods, which are currently under intense investigation and developmental activity in laboratories worldwide. It is hoped that this review will act as a springboard for researchers and clinicians across a wide range of disciplines in this exciting era of multidisciplinary and novel approaches to disease diagnostics. © 2007 Future Medicine Ltd.

PMID

17924839 [<https://www.ncbi.nlm.nih.gov/pubmed/?term=17924839>]

Institution

(Ellis, Dunn, Allwood, Goodacre) University of Manchester, School of Chemistry, Manchester Interdisciplinary Biocentre, 131 Princess Street, Manchester M1 7ND, United Kingdom (Dunn) University of Manchester, Manchester Centre for Integrative Systems Biology, Manchester Interdisciplinary Biocentre, 131 Princess Street, Manchester M1 7ND, United Kingdom

(Griffin) University of Cambridge, Department of Biochemistry, Cambridge CB2 1QW, United Kingdom

Publisher

Future Medicine Ltd. (2nd Albert Place, Finchley Central, London N3 1QB, United Kingdom)

Emtree Heading

arthritis/di [Diagnosis]; Barrett esophagus/di [Diagnosis]; bladder cancer/di [Diagnosis]; brain cancer/di [Diagnosis]; breast cancer/di [Diagnosis]; childhood leukemia/di [Diagnosis]; childhood leukemia/dt [Drug Therapy]; chronic lymphatic leukemia/di [Diagnosis]; colorectal carcinoma/di [Diagnosis]; diabetes mellitus/di [Diagnosis]; diagnostic procedure; *DNA fingerprinting; genomics; human; inborn error of metabolism/di [Diagnosis]; infrared spectroscopy; intermethod comparison; laboratory test; larynx cancer/di [Diagnosis]; malignant neoplastic disease/di [Diagnosis]; mass spectrometry; matrix assisted laser desorption ionization time of flight mass spectrometry; *metabolomics; molecular biology; nonhuman; nuclear magnetic resonance spectroscopy; ovary cancer/di [Diagnosis]; precancer/di [Diagnosis]; prion disease/di [Diagnosis]; prostate cancer/di [Diagnosis]; prostate hypertrophy/di [Diagnosis]; proteomics; Raman spectrometry; review; stomach cancer/di [Diagnosis]; thyroid tumor/di [Diagnosis]; transcriptomics; uterine cervix cancer/di [Diagnosis]; etoposide/dt [Drug Therapy].

Drug Index Terms

etoposide / drug therapy.

Other Index Terms

arthritis / diagnosis; Barrett esophagus / diagnosis; bladder cancer / diagnosis; brain cancer / diagnosis; breast cancer / diagnosis; childhood leukemia / diagnosis / drug therapy; chronic lymphatic leukemia / diagnosis; colorectal carcinoma / diagnosis; diabetes mellitus / diagnosis; diagnostic procedure; *DNA fingerprinting; genomics; human; inborn error of metabolism / diagnosis; infrared spectroscopy; intermethod comparison; laboratory test; larynx cancer / diagnosis; malignant neoplastic disease / diagnosis; mass spectrometry; matrix assisted laser desorption ionization time of flight mass spectrometry; *metabolomics; molecular biology; nonhuman; nuclear magnetic resonance spectroscopy; ovary cancer / diagnosis; precancer / diagnosis; prion disease / diagnosis; prostate cancer / diagnosis; prostate hypertrophy / diagnosis; proteomics; Raman spectrometry; review; stomach cancer / diagnosis; thyroid tumor / diagnosis; transcriptomics; uterine cervix cancer / diagnosis.

Link to the Ovid Full Text or citation:

[Click here for full text options](https://ovidsp.ovid.com/ovidweb.cgi?T=JS&CSC=Y&NEWS=N&PAGE=fulltext&D=emed10&AN=47578232)

Link to the External Link Resolver:

[SFX](https://sfx-86scu.hosted.exlibrisgroup.com.cn/86scu?sid=OVID:embase&id=pmid:17924839&id=doi:10.2217%2F14622416.8.9.1243&issn=14622416&isbn=&volume=8&issue=9&spage=1243&pages=1243-1266&date=2007&title=Pharmacogenomics&atitle=Metabolic+fingerprinting+as+a+diagnostic+tool&aulast=Ellis&pid=<author>Ellis+D.I.%3BDunn+W.B.%3BGriffin+J.L.%3BAllwood+J.W.%3BGoodacre+R.<%2Fauthor><AN>47578232<%2FAN><DT>Review<%2FDT>)

364.

Early diagnosis of oral cancer based on the surface plasmon resonance of gold nanoparticles.

Kah J.C.Y., Kho K.W., Lee C.G.L., Sheppard C.J.R., Shen Z.X., Soo K.C., Olivo M.C.

International Journal of Nanomedicine. 2(4) (pp 785-798), 2007. Date of Publication: 2007.

AN: 351068320

The high mortality rate in cancer such as oral squamous cell carcinoma is commonly attributed to the difficulties in detecting the disease at an early treatable stage. In this study, we exploited the ability of gold nanoparticles to undergo coupled surface plasmon resonance and set up strong electric fields when closely-spaced to improve the molecular contrast signal in reflectance-based imaging and also to enhance the Raman signal of bioanalytes in cancer. Colloidal gold nanoparticles were synthesized and conjugated to anti-epidermal growth factor receptor (EGFR) for imaging. A self-assembled surface enhanced Raman scattering (SERS)-active gold nanoparticle monolayer film was also developed as a biosensing surface using a simple drop-dry approach. We have shown that gold nanoparticles could elicit an optical contrast to discriminate between cancerous and normal cells and their conjugation with antibodies allowed them to map the expression of relevant biomarkers for molecular imaging under confocal reflectance microscopy. We have also shown that the SERS spectra of saliva from the closely-packed gold nanoparticles films was differentiable between those acquired from normal individuals and oral cancer patients, thus showing promise of a simple SERS-based saliva assay for early diagnosis of oral cancer. © 2007 Dove Medical Press Limited. All rights reserved.

PMID

18203445 [<https://www.ncbi.nlm.nih.gov/pubmed/?term=18203445>]

Institution

(Kah, Sheppard) Division of Bioengineering, National University of Singapore, Singapore, Singapore (Kho, Lee, Soo, Olivo) Division of Medical Sciences, National Cancer Centre Singapore, 11 Hospital Drive, Singapore 169610, Singapore

(Shen) School of Physical and Mathematical Sciences, Nanyang Technological University, Singapore, Singapore

Publisher

DOVE Medical Press Ltd. (PO Box 300-008, Albany, Auckland, New Zealand)

Emtree Heading

antigen binding; article; biosensor; cancer cell; cancer mortality; cancer patient; cancer staging; clinical article; colloid; confocal microscopy; contrast enhancement; controlled study; diagnostic value; early diagnosis; electric field; human; human cell; *mouth cancer/di [Diagnosis]; protein expression; Raman spectrometry; saliva analysis; signal processing; squamous cell carcinoma/di [Diagnosis]; *surface plasmon resonance; *epidermal growth factor receptor antibody; *gold; *nanoparticle.

Drug Index Terms

*epidermal growth factor receptor antibody; *gold; *nanoparticle.

Other Index Terms

antigen binding; article; biosensor; cancer cell; cancer mortality; cancer patient; cancer staging; clinical article; colloid; confocal microscopy; contrast enhancement; controlled study; diagnostic value; early diagnosis; electric field; human; human cell; *mouth cancer / *diagnosis; protein expression; Raman spectrometry; saliva analysis; signal processing; squamous cell carcinoma / diagnosis; *surface plasmon resonance.

Link to the Ovid Full Text or citation:

[Click here for full text options](https://ovidsp.ovid.com/ovidweb.cgi?T=JS&CSC=Y&NEWS=N&PAGE=fulltext&D=emed10&AN=351068320)

Link to the External Link Resolver:

[SFX](https://sfx-86scu.hosted.exlibrisgroup.com.cn/86scu?sid=OVID:embase&id=pmid:18203445&id=doi:&issn=11769114&isbn=&volume=2&issue=4&spage=785&pages=785-798&date=2007&title=International+Journal+of+Nanomedicine&atitle=Early+diagnosis+of+oral+cancer+based+on+the+surface+plasmon+resonance+of+gold+nanoparticles&aulast=Kah&pid=<author>Kah+J.C.Y.%3BKho+K.W.%3BLee+C.G.L.%3BSheppard+C.J.R.%3BShen+Z.X.%3BSoo+K.C.%3BOlivo+M.C.<%2Fauthor><AN>351068320<%2FAN><DT>Article<%2FDT>)

365.

Catalytic polymerization and facile grafting of poly(furfuryl alcohol) to single-wall carbon nanotube: Preparation of nanocomposite carbon.

Yi B., Rajagopalan R., Foley H.C., Kim U.J., Liu X., Eklund P.C.

Journal of the American Chemical Society. 128(34) (pp 11307-11313), 2006. Date of Publication: 30 Aug 2006.

AN: 44300141

A nanocomposite carbon was prepared by grafting a carbonizable polymer, poly(furfuryl alcohol) (PFA), to a single-wall carbon nanotube (SWNT). The SWNT was first functionalized with arylsulfonic acid groups on the sidewall via a method using a diazonium reagent. Both Raman and FTIR spectroscopies were used to identify the functional groups on the nanotube surface. HRTEM imaging shows that the SWNT bundles are exfoliated after functionalization. Once this state of the SWNTs was accomplished, the PFA-functionalized SWNT (PFA-SWNT) was prepared by in situ polymerization of furfuryl alcohol (FA). The sulfonic acid groups on the surface of the SWNT acted as a catalyst for FA polymerization, and the resulting PFA then grafted to the SWNTs. The surfaces of the SWNTs converted from hydrophilic to hydrophobic when they were wrapped with PFA. The formation of the polymer and the attraction between it and the sulfonic acid groups were confirmed by IR spectra. A nanocomposite carbon was generated by heating the PFA-SWNT in argon at 600 degreeC, a process during which the PFA was transformed to nanoporous carbon (NPC) and the sulfonic acid groups were cleaved from the SWNT. Based upon the Raman spectra and HRTEM images of the composite, it is concluded that SWNTs survive this process and a continuous phase is formed between the NPC and the SWNT. © 2006 American Chemical Society.

Institution

(Foley) Department of Chemistry, Pennsylvania State University, University Park, PA 16802, United States (Yi, Foley) Department of Chemical Engineering, Pennsylvania State University, University Park, PA 16802, United States

(Rajagopalan, Foley, Eklund) Materials Research Institute, Pennsylvania State University, University Park, PA 16802, United States

(Kim, Liu, Eklund) Department of Physics, Pennsylvania State University, University Park, PA 16802, United States

(Kim) Nano Fabrication Technology Center, Samsung Advanced Institute of Technology, Kyeongki-Do 449-712, South Korea

Publisher

American Chemical Society

Emtree Heading

article; *catalysis; infrared spectroscopy; *polymerization; Raman spectrometry; arylsulfonic acid derivative; *carbon; *carbon nanotube; diazonium compound; functional group; *furfuryl alcohol; *nanotube; sulfonic acid derivative.

Drug Index Terms

arylsulfonic acid derivative; *carbon; *carbon nanotube; diazonium compound; functional group; *furfuryl alcohol; *nanotube; sulfonic acid derivative.

Other Index Terms

article; *catalysis; infrared spectroscopy; *polymerization; Raman spectrometry.

Link to the Ovid Full Text or citation:

[Click here for full text options](https://ovidsp.ovid.com/ovidweb.cgi?T=JS&CSC=Y&NEWS=N&PAGE=fulltext&D=emed9&AN=44300141)

Link to the External Link Resolver:

[SFX](https://sfx-86scu.hosted.exlibrisgroup.com.cn/86scu?sid=OVID:embase&id=pmid:&id=doi:10.1021%2Fja063518x&issn=00027863&isbn=&volume=128&issue=34&spage=11307&pages=11307-11313&date=2006&title=Journal+of+the+American+Chemical+Society&atitle=Catalytic+polymerization+and+facile+grafting+of+poly(furfuryl+alcohol)+to+single-wall+carbon+nanotube%3A+Preparation+of+nanocomposite+carbon&aulast=Yi&pid=<author>Yi+B.%3BRajagopalan+R.%3BFoley+H.C.%3BKim+U.J.%3BLiu+X.%3BEklund+P.C.<%2Fauthor><AN>44300141<%2FAN><DT>Article<%2FDT>)

366.

FT-Raman spectroscopic investigation on hypothyroid cancer.

Li R., Zhou G.M., Peng H.J., Deng Y.L.

Guang pu xue yu guang pu fen xi = Guang pu. 26(10) (pp 1868-1870), 2006. Date of Publication: Oct 2006.

AN: 350359350

The normal and malignant tissues from hypothyroid were investigated by FT-Raman spectroscopy. The results show that two characteristic Raman spectral peaks of normal tissues appeared at 503 and 758 cm(-1) (corresponding to theextension vibration of C--I band and the vibration of benzene rings respectively) in the range of 400-3500 cm(-1). The two peaks disappeared at malignant tissues. The intensity of the characteristic vibration peaks of normal tissues appearing at 3 062 and 1 003 cm(-1) corresponding to tyrosine weakened in malignant tissues. Thus, FT-Raman spectroscopy is a potential method in the diagnosis of hypothyroid cancer.

PMID

17205740 [<https://www.ncbi.nlm.nih.gov/pubmed/?term=17205740>]

Institution

(Li, Zhou, Peng, Deng) Department of Chemistry, Guiyang Medical College, Guiyang 550004, China.

Emtree Heading

article; human; methodology; *Raman spectrometry; *thyroid tumor/di [Diagnosis].

Other Index Terms

article; human; methodology; *Raman spectrometry; *thyroid tumor / *diagnosis.

Link to the Ovid Full Text or citation:

[Click here for full text options](https://ovidsp.ovid.com/ovidweb.cgi?T=JS&CSC=Y&NEWS=N&PAGE=fulltext&D=emed9&AN=350359350)

Link to the External Link Resolver:

[SFX](https://sfx-86scu.hosted.exlibrisgroup.com.cn/86scu?sid=OVID:embase&id=pmid:17205740&id=doi:&issn=10000593&isbn=&volume=26&issue=10&spage=1868&pages=1868-1870&date=2006&title=Guang+pu+xue+yu+guang+pu+fen+xi+%3D+Guang+pu&atitle=FT-Raman+spectroscopic+investigation+on+hypothyroid+cancer&aulast=Li&pid=<author>Li+R.%3BZhou+G.M.%3BPeng+H.J.%3BDeng+Y.L.<%2Fauthor><AN>350359350<%2FAN><DT>Article<%2FDT>)

367.

Spectroscopy and fluorescence in esophageal diseases.

DaCosta R.S., Wilson B.C., Marcon N.E.

Best Practice and Research: Clinical Gastroenterology. 20(1) (pp 41-57), 2006. Date of Publication: February 2006.

AN: 43210626

Malignant tumors of the esophagus continue to be a major health issue associated with high mortality primarily because most present with symptoms of dysphagia or anaemia. The disease at that stage is advanced and not likely curable. The big issue for squamous dysplasia and that associated with BE is that only a small proportion are discovered in surveillance programs when they are asymptomatic, either because the patient lives in a high-incidence geographical area, has a family history, previously diagnosed head and neck cancer or chronic reflux, as in Barrett's. Current endoscopic methods are hampered by the endoscopist's inability to recognize subtle topographic clues of dysplasia, sampling errors related to biopsy protocols, and confounding inflammation-induced artifacts both for the endoscopist and pathologist. What is desperately needed would be a biomarker (e.g. serological, fecal, urinary) that selects patients for endoscopy. However, such a test is not yet on the horizon. This article examines the current status in practice and research of novel optically based 'bioendoscopic' devices (i.e. fluorescence spectroscopy and imaging, confocal fluorescence microendoscopy (CFM), light scattering spectroscopy (LSS), Raman spectroscopy (RS), and immunophotodiagnostic endoscopy) which may enhance the diagnosis of dysplasia in all patients undergoing conventional white light endoscopy. Perhaps these new technologies will lead to more cost-effective diagnosis, mapping (e.g. surface), and staging (e.g. depth) of dysplasia, thereby allowing timely cure by endoscopic means (e.g. EMR and/or PDT), biological interventions (e.g. Cox-2 inhibitors) rather than esophajectomy. © 2005 Elsevier Ltd. All rights reserved.

PMID

16473800 [<https://www.ncbi.nlm.nih.gov/pubmed/?term=16473800>]

Institution

(DaCosta, Wilson) Department of Medical Biophysics, University of Toronto, Ontario Cancer Institute, Toronto, Ont., Canada (Marcon) St. Micheal's Hospital, Center for the Therapeutic Endoscopy and Endoscopic Oncology, 30 Bond Street, Toronto, Ont. M5B 1W8, Canada

Publisher

Bailliere Tindall Ltd (32 Jamestown Road, London NW1 7BY, United Kingdom)

Emtree Heading

artifact; Barrett esophagus/di [Diagnosis]; cancer staging; clinical effectiveness; clinical practice; clinical trial; computer program; contrast enhancement; cost effectiveness analysis; diagnostic accuracy; diagnostic value; disease severity; early diagnosis; endoscopic mucosal resection; *esophagoscopy; esophagus biopsy; *esophagus cancer/di [Diagnosis]; *esophagus cancer/dm [Disease Management]; *esophagus cancer/su [Surgery]; *esophagus cancer/th [Therapy]; ex vivo study; *fluorescence spectroscopy; health care cost; human; image quality; in vivo study; medical research; non invasive measurement; nonhuman; optical coherence tomography; photodynamic therapy; prediction; Raman spectrometry; review; sensitivity and specificity; statistical significance; biological marker; fluorescein/iv [Intravenous Drug Administration]; indocyanine green.

Drug Index Terms

biological marker; fluorescein / intravenous drug administration; indocyanine green.

Other Index Terms

artifact; Barrett esophagus / diagnosis; cancer staging; clinical effectiveness; clinical practice; clinical trial; computer program; contrast enhancement; cost effectiveness analysis; diagnostic accuracy; diagnostic value; disease severity; early diagnosis; endoscopic mucosal resection; *esophagoscopy; esophagus biopsy; *esophagus cancer / *diagnosis / *disease management / *surgery / *therapy; ex vivo study; *fluorescence spectroscopy; health care cost; human; image quality; in vivo study; medical research; non invasive measurement; nonhuman; optical coherence tomography; photodynamic therapy; prediction; Raman spectrometry; review; sensitivity and specificity; statistical significance.

Link to the Ovid Full Text or citation:

[Click here for full text options](https://ovidsp.ovid.com/ovidweb.cgi?T=JS&CSC=Y&NEWS=N&PAGE=fulltext&D=emed9&AN=43210626)

Link to the External Link Resolver:

[SFX](https://sfx-86scu.hosted.exlibrisgroup.com.cn/86scu?sid=OVID:embase&id=pmid:16473800&id=doi:10.1016%2Fj.bpg.2005.08.003&issn=15216918&isbn=&volume=20&issue=1&spage=41&pages=41-57&date=2006&title=Best+Practice+and+Research%3A+Clinical+Gastroenterology&atitle=Spectroscopy+and+fluorescence+in+esophageal+diseases&aulast=DaCosta&pid=<author>DaCosta+R.S.%3BWilson+B.C.%3BMarcon+N.E.<%2Fauthor><AN>43210626<%2FAN><DT>Review<%2FDT>)

368.

Near-infrared Raman spectroscopy for oral carcinoma diagnosis.

Oliveira A.P., Bitar R.A., Silveira Jr. L., Zangaro R.A., Martin A.A.

Photomedicine and Laser Surgery. 24(3) (pp 348-353), 2006. Date of Publication: June 2006.

AN: 44115217

Objective: Fourier-transform (FT)-Raman spectroscopy has been used to explore the changes in the vibrational bands of normal, dysplastic (DE) and squamous cell carcinoma (SCC) tissues. Background Data: Raman spectroscopy has been applied as a diagnostic tool for the detection of cancers, due to its sensitivity to the changes in molecular composition and conformation that occurs in malignant tissues. The detection of weak Raman signals from biotissues becomes easier by FT-Raman due to fluorescence suppression.

Method(s): A carcinogen (7,12-dimethybenz[a]anthracene [DMBA]) was applied daily in the oral pouch of 21 hamster to induce oral carcinoma. After 14 weeks, the fragments of squamous cell carcinomas and oral normal tissue were collected and analyzed by FT-Raman spectroscopy, using a 1064-nm Nd:YAG laser line as an excitation source. A total of 123 spectra were obtained and divided in normal and malignant tissue groups, and analyzed statistically through principal components analysis (PCA) and classified using Mahalanobis distance.

Result(s): Major differences between normal and malignant spectra seem to arise from the composition, conformational, and structural changes of proteins, and possible increase of its content in malignant epithelia. An algorithm based on PCA was able to separate the samples into two groups-normal and carcinoma. For the algorithm training group, 91% sensitivity and 69% specificity were observed, while the prospective group had 100% sensitivity and 55% specificity.

Conclusion(s): The algorithm based on PCA has the potential for classifying Raman spectra and can be useful for detection of dysplastic and malign oral lesion. © Mary Ann Liebert, Inc.

PMID

16875443 [<https://www.ncbi.nlm.nih.gov/pubmed/?term=16875443>]

Institution

(Oliveira, Silveira Jr., Zangaro) Grupo de Optica Biomedica, Instituto de Pesquisa e Desenvolvimento (IP and D), Universidade do Vale do Paraiba (UNIVAP), Sao Jose dos Campos, SP, Brazil (Bitar, Martin) Laboratorio de Espectroscopia Vibracional Biomedica, Instituto de Pesquisa e Desenvolvimento (IP and D), Universidade do Vale do Paraiba (UNIVAP), Sao Jose dos Campos, SP, Brazil

(Martin) Universidade do Vale do Paraiba (UNIVAP), Instituto de Pesquisa e Desenvolvimento (IP and D), Av. Shishima Hifumi, 2911, 12244-000 Sao Jose dos Campos, SP, Brazil

Publisher

Mary Ann Liebert Inc. (140 Huguenot Street, New Rochelle NY 10801-5215, United States)

Emtree Heading

algorithm; animal cell; animal experiment; animal model; animal tissue; article; *cancer diagnosis; conformation; controlled study; diagnostic value; hamster; infrared spectroscopy; male; *mouth carcinoma/di [Diagnosis]; neodymium laser; nonhuman; principal component analysis; protein structure; *Raman spectrometry; sensitivity and specificity; signal transduction; squamous cell carcinoma/di [Diagnosis]; vibration; *carcinogen; *dimethylbenz[a]anthracene.

Drug Index Terms

*carcinogen; *dimethylbenz[a]anthracene.

Other Index Terms

algorithm; animal cell; animal experiment; animal model; animal tissue; article; *cancer diagnosis; conformation; controlled study; diagnostic value; hamster; infrared spectroscopy; male; *mouth carcinoma / *diagnosis; neodymium laser; nonhuman; principal component analysis; protein structure; *Raman spectrometry; sensitivity and specificity; signal transduction; squamous cell carcinoma / diagnosis; vibration.

Link to the Ovid Full Text or citation:

[Click here for full text options](https://ovidsp.ovid.com/ovidweb.cgi?T=JS&CSC=Y&NEWS=N&PAGE=fulltext&D=emed9&AN=44115217)

Link to the External Link Resolver:

[SFX](https://sfx-86scu.hosted.exlibrisgroup.com.cn/86scu?sid=OVID:embase&id=pmid:16875443&id=doi:10.1089%2Fpho.2006.24.348&issn=15495418&isbn=&volume=24&issue=3&spage=348&pages=348-353&date=2006&title=Photomedicine+and+Laser+Surgery&atitle=Near-infrared+Raman+spectroscopy+for+oral+carcinoma+diagnosis&aulast=Oliveira&pid=<author>Oliveira+A.P.%3BBitar+R.A.%3BSilveira+Jr.+L.%3BZangaro+R.A.%3BMartin+A.A.<%2Fauthor><AN>44115217<%2FAN><DT>Article<%2FDT>)

369.

Discrimination of normal, inflammatory, premalignant, and malignant oral tissue: A Raman spectroscopy study.

Malini R., Venkatakrishna K., Kurien J., Pai K.M., Rao L., Kartha V.B., Krishna C.M.

Biopolymers. 81(3) (pp 179-193), 2006. Date of Publication: 15 Feb 2006.

AN: 43294778

Optical spectroscopy methods are fast emerging as potential alternatives for early diagnosis of cancer. A Raman spectroscopy method for discrimination of normal and malignant oral tissues has been developed by us earlier. It is necessary to evaluate and establish the validity of the approach before it can be routinely used. In the present study, our Raman spectroscopy investigations are extended further to evaluate the efficacy of the technique to discriminate between normal, inflammatory, premalignant, and malignant conditions in oral tissue. Spectral profiles of normal, malignant, premalignant, and inflammatory conditions show pronounced differences between one another. Spectra of normal tissues can be attributed mainly to lipids whereas pathological tissue spectra are dominated by proteins. Principal components analysis (PCA) of the spectral data sets belonging to the four different categories showed that scores of factors differentiated between normal and all pathological conditions but gave only poor discrimination among the three pathological states. PCA combined with multiparameter limit tests allow match/mismatch criteria to be applied to test samples when pathologically certified calibration sets are available in each class. It is shown that by this method all the four tissue types could be discriminated and diagnosed correctly. The biochemical differences between normal and pathological conditions of oral tissue are also discussed from spectral differences of the different classes of spectra. © 2005 Wiley Periodicals, Inc.

PMID

16231284 [<https://www.ncbi.nlm.nih.gov/pubmed/?term=16231284>]

Institution

(Malini, Venkatakrishna, Kartha, Krishna) Center for Laser Spectroscopy, Manipal Academy of Higher Education, Manipal, India (Kurien) Department of Surgical Oncology, Sai Baba Cancer Hospital and Research Center, Manipal Academy of Higher Education, Manipal, India

(Pai) Department of Oral Medicine and Radiology, College of Dental Sciences, Manipal Academy of Higher Education, Manipal, India

(Rao) Department of Pathology, Kasturba Medical College, Manipal Academy of Higher Education, Manipal, India

Publisher

John Wiley and Sons Inc. (P.O.Box 18667, Newark NJ 07191-8667, United States)

Emtree Heading

article; calibration; controlled study; diagnostic procedure; diagnostic value; early diagnosis; human; human tissue; *mouth cancer/di [Diagnosis]; *mouth inflammation/di [Diagnosis]; *precancer/di [Diagnosis]; principal component analysis; *Raman spectrometry; lipid/ec [Endogenous Compound].

Drug Index Terms

lipid / endogenous compound.

Other Index Terms

article; calibration; controlled study; diagnostic procedure; diagnostic value; early diagnosis; human; human tissue; *mouth cancer / *diagnosis; *mouth inflammation / *diagnosis; *precancer / *diagnosis; principal component analysis; *Raman spectrometry.

Link to the Ovid Full Text or citation:

[Click here for full text options](https://ovidsp.ovid.com/ovidweb.cgi?T=JS&CSC=Y&NEWS=N&PAGE=fulltext&D=emed9&AN=43294778)

Link to the External Link Resolver:

[SFX](https://sfx-86scu.hosted.exlibrisgroup.com.cn/86scu?sid=OVID:embase&id=pmid:16231284&id=doi:10.1002%2Fbip.20398&issn=00063525&isbn=&volume=81&issue=3&spage=179&pages=179-193&date=2006&title=Biopolymers&atitle=Discrimination+of+normal%2C+inflammatory%2C+premalignant%2C+and+malignant+oral+tissue%3A+A+Raman+spectroscopy+study&aulast=Malini&pid=<author>Malini+R.%3BVenkatakrishna+K.%3BKurien+J.%3BPai+K.M.%3BRao+L.%3BKartha+V.B.%3BKrishna+C.M.<%2Fauthor><AN>43294778<%2FAN><DT>Article<%2FDT>)

370.

Optical techniques in diagnosis of head and neck malignancy.

Swinson B., Jerjes W., El-Maaytah M., Norris P., Hopper C.

Oral Oncology. 42(3) (pp 221-228), 2006. Date of Publication: March 2006.

AN: 43246754

The "gold standard" of assessing pathological changes in tissue is currently histopathology. However, the processing of biopsy material and the interpretation of the results inevitably leads to diagnostic delay and the added possibility of taking an unrepresentative sample. Recently, there has been increasing interest in the use of optical spectroscopy systems to be able to provide tissue diagnosis in real-time, non-invasively and in situ. These systems rely on the fact that the optical spectrum derived from any tissue will contain information about the histological and biochemical make up of that tissue. The technique has not only been shown to have a role in the detection of dysplasia and malignancy but also in performing guided biopsies, monitoring of haemoglobin tissue perfusion in free-flaps and therapeutic drug levels during chemo- and photodynamic therapy. The assessment of surgical margins and a role in sentinel node biopsy are also interesting developments. The obvious advantages of being able to accurately diagnose pathology without the need to remove a tissue sample diminish patient trauma as well as having financial implications.

PMID

16140566 [<https://www.ncbi.nlm.nih.gov/pubmed/?term=16140566>]

Institution

(Swinson, Jerjes, El-Maaytah, Norris, Hopper) Oral and Maxillofacial Surgery Unit, Eastman Dental Institute for Oral Healthcare Sciences, 256 Gray's Inn Road, London WC1X 8LD, United Kingdom (Swinson) Department of Maxillofacial Surgery, St. Georges Hospital, Blackshaw Rd, Tooting, London SW17 0QT, United Kingdom

Publisher

Elsevier Ltd (Langford Lane, Kidlington, Oxford OX5 1GB, United Kingdom)

Emtree Heading

autofluorescence; Barrett esophagus; bone necrosis/co [Complication]; cancer chemotherapy; *cancer diagnosis; cell membrane; cervical lymph node; diagnostic accuracy; dysplasia; fluorescence spectroscopy; *head and neck cancer/di [Diagnosis]; histopathology; human; hypoxia; intermethod comparison; larynx carcinoma/di [Diagnosis]; microcirculation; mouth cancer; mouth cavity; mouth lesion; mouth mucosa; non invasive measurement; oxygen saturation; pharynx carcinoma/di [Diagnosis]; photodynamic therapy; priority journal; Raman spectrometry; reliability; review; sensitivity and specificity; sentinel lymph node biopsy; spectroscopy; squamous cell carcinoma; thrombosis; trabecular bone; aminolevulinic acid; glass fiber; hemoglobin; protoporphyrin; elastic scattering spectroscopy; trimodal spectroscopy.

Candidate Terms

elastic scattering spectroscopy [other term]	trimodal spectroscopy [other term]

Drug Index Terms

aminolevulinic acid; glass fiber; hemoglobin; protoporphyrin.

Other Index Terms

autofluorescence; Barrett esophagus; bone necrosis / complication; cancer chemotherapy; *cancer diagnosis; cell membrane; cervical lymph node; diagnostic accuracy; dysplasia; fluorescence spectroscopy; *head and neck cancer / *diagnosis; histopathology; human; hypoxia; intermethod comparison; larynx carcinoma / diagnosis; microcirculation; mouth cancer; mouth cavity; mouth lesion; mouth mucosa; non invasive measurement; oxygen saturation; pharynx carcinoma / diagnosis; photodynamic therapy; priority journal; Raman spectrometry; reliability; review; sensitivity and specificity; sentinel lymph node biopsy; spectroscopy; squamous cell carcinoma; thrombosis; trabecular bone.

Link to the Ovid Full Text or citation:

[Click here for full text options](https://ovidsp.ovid.com/ovidweb.cgi?T=JS&CSC=Y&NEWS=N&PAGE=fulltext&D=emed9&AN=43246754)

Link to the External Link Resolver:

[SFX](https://sfx-86scu.hosted.exlibrisgroup.com.cn/86scu?sid=OVID:embase&id=pmid:16140566&id=doi:10.1016%2Fj.oraloncology.2005.05.001&issn=13688375&isbn=&volume=42&issue=3&spage=221&pages=221-228&date=2006&title=Oral+Oncology&atitle=Optical+techniques+in+diagnosis+of+head+and+neck+malignancy&aulast=Swinson&pid=<author>Swinson+B.%3BJerjes+W.%3BEl-Maaytah+M.%3BNorris+P.%3BHopper+C.<%2Fauthor><AN>43246754<%2FAN><DT>Review<%2FDT>)

371.

Raman spectroscopy for optical diagnosis in the larynx: Preliminary findings.

Lau D.P., Huang Z., Lui H., Anderson D.W., Berean K., Morrison M.D., Shen L., Zeng H.

Lasers in Surgery and Medicine. 37(3) (pp 192-200), 2005. Date of Publication: September 2005.

AN: 41464820

Background and Objectives: Raman spectroscopy (RS) provides information about molecular structure and is a potential tool for non-invasive tissue diagnosis. To determine if Raman spectra could be obtained rapidly from laryngeal tissue in vitro, and compare Raman spectra from normal, benign, and cancerous laryngeal tissue. Study Design/Materials and Methods: Forty-seven laryngeal specimens were studied using RS with signal acquisition times (SAT) between 1 and 30 second(s). Multivariate analysis was used to determine the diagnostic ability of RS compared to standard histology (n = 18, 13, and 16 respectively for normal tissue, carcinoma, and squamous papilloma).

Result(s): Good quality spectra were obtained with 5-second SAT. Spectral peak analysis showed prediction sensitivities of 89%, 69%, and 88%, and specificities of 86%, 94%, and 94% for normal tissue, carcinoma, and papilloma.

Conclusion(s): In the larynx, spectral differences appear to exist between normal tissue, carcinoma, and papilloma. The ability to obtain spectra rapidly supports potential for future in vivo studies. © 2005 Wiley-Liss, Inc.

PMID

16127671 [<https://www.ncbi.nlm.nih.gov/pubmed/?term=16127671>]

Institution

(Lau) Department of Otolaryngology, Singapore General Hospital, Singapore 169608, Singapore (Lau, Anderson, Morrison) Division of Otolaryngology, University of British Columbia, Vancouver General Hospital, Vancouver, BC V5Z 1M9, Canada

(Huang, Zeng) Department of Cancer Imaging, British Columbia Cancer Research Center, Vancouver, BC V5Z 1L3, Canada

(Lui) Division of Dermatology, University of British Columbia, Vancouver General Hospital, Vancouver, BC V5Z 4E8, Canada

(Berean) Department of Pathology, University of British Columbia, Vancouver General Hospital, Vancouver, BC V6T 2B5, Canada

(Shen) Clinical Trials and Epidemiology Research Unit, 226 Outram Road, Singapore 169039, Singapore

(Lau) Department of Otolaryngology, Singapore General Hospital, Outram Road, Singapore 169609, Singapore

Publisher

Wiley-Liss Inc. (111 River Street, Hoboken NJ 07030-5774, United States)

Emtree Heading

adult; aged; analysis of variance; article; clinical article; controlled study; diagnostic accuracy; diagnostic imaging; differential diagnosis; diode laser; discriminant analysis; histology; human; human tissue; image quality; intermethod comparison; *larynx; *larynx carcinoma/di [Diagnosis]; larynx papillomatosis/di [Diagnosis]; optics; priority journal; *Raman spectrometry.

Other Index Terms

adult; aged; analysis of variance; article; clinical article; controlled study; diagnostic accuracy; diagnostic imaging; differential diagnosis; diode laser; discriminant analysis; histology; human; human tissue; image quality; intermethod comparison; *larynx; *larynx carcinoma / *diagnosis; larynx papillomatosis / diagnosis; optics; priority journal; *Raman spectrometry.

Link to the Ovid Full Text or citation:

[Click here for full text options](https://ovidsp.ovid.com/ovidweb.cgi?T=JS&CSC=Y&NEWS=N&PAGE=fulltext&D=emed9&AN=41464820)

Link to the External Link Resolver:

[SFX](https://sfx-86scu.hosted.exlibrisgroup.com.cn/86scu?sid=OVID:embase&id=pmid:16127671&id=doi:10.1002%2Flsm.20226&issn=01968092&isbn=&volume=37&issue=3&spage=192&pages=192-200&date=2005&title=Lasers+in+Surgery+and+Medicine&atitle=Raman+spectroscopy+for+optical+diagnosis+in+the+larynx%3A+Preliminary+findings&aulast=Lau&pid=<author>Lau+D.P.%3BHuang+Z.%3BLui+H.%3BAnderson+D.W.%3BBerean+K.%3BMorrison+M.D.%3BShen+L.%3BZeng+H.<%2Fauthor><AN>41464820<%2FAN><DT>Article<%2FDT>)

372.

Autofluorescence and Raman microspectroscopy of tissue sections of oral lesions.

De Veld D.C.G., Bakker Schut T.C., Skurichina M., Witjes M.J.H., Van Der Wal J.E., Roodenburg J.L.N., Sterenborg H.J.C.M.

Lasers in Medical Science. 19(4) (pp 203-209), 2005. Date of Publication: April 2005.

AN: 40603174

Autofluorescence spectroscopy and Raman spectroscopy have been suggested for lesion diagnostics. We investigate the information contained in autofluorescence and Raman spectra recorded from oral tissue slices of various lesion types. Thirty-seven human oral mucosa lesions were biopsied and freeze-dried. Complete autofluorescence images and spectra were recorded from 20 mum sections. Raman spectra were acquired from the same positions for 12 of the sections. Cluster analysis was applied to find any relationship between spectral shape and lesion type or cell layer. Autofluorescence images showed high intensities for keratin layers and connective tissue, but hardly any for the epithelium. Autofluorescence spectra were centered around 520 nm and did not show specific spectral features. No clustering with regard to lesion type or cell layer was observed. Raman spectra allowed for reliable classification into cell layers, but differences between lesion types were not significant in this study. Autofluorescence spectra of freeze-dried oral mucosa sections did not contain useful information. A more comprehensive study is required for Raman spectra. © Springer-Verlag London Limited 2005.

PMID

15772873 [<https://www.ncbi.nlm.nih.gov/pubmed/?term=15772873>]

Institution

(De Veld, Witjes, Roodenburg) Dept. Oral and Maxillofacial Surg., Division of Oncology, University Hospital Groningen, Groningen, Netherlands (De Veld, Sterenborg) Photodynamic Ther. Optical S., Department of Radiation Oncology, Erasmus Medical Center, P.O. Box 2040, 3000 CA Rotterdam, Netherlands

(Bakker Schut) Ctr. Optical Diagnostics and Ther., Department of General Surgery, Erasmus Medical Center, Rotterdam, Netherlands

(Skurichina) Info. and Communication Theory Group, Fac. Elec. Eng., Math. Comp. Sci., Delft University of Technology, Delft, Netherlands

(Van Der Wal) Department of Pathology, University Hospital Groningen, Groningen, Netherlands

Publisher

Springer London (The Guildway, Old Portsmouth Road, Artington, Guildford GU3 1LP, United Kingdom)

Emtree Heading

article; *autofluorescence; cluster analysis; connective tissue; controlled study; epithelium; freeze drying; human; human tissue; image display; medical information; *mouth lesion; mouth mucosa; observation; oral biopsy; priority journal; *Raman spectrometry; recording; reliability; tissue slice; keratin/ec [Endogenous Compound].

Drug Index Terms

keratin / endogenous compound.

Other Index Terms

article; *autofluorescence; cluster analysis; connective tissue; controlled study; epithelium; freeze drying; human; human tissue; image display; medical information; *mouth lesion; mouth mucosa; observation; oral biopsy; priority journal; *Raman spectrometry; recording; reliability; tissue slice.

Link to the Ovid Full Text or citation:

[Click here for full text options](https://ovidsp.ovid.com/ovidweb.cgi?T=JS&CSC=Y&NEWS=N&PAGE=fulltext&D=emed9&AN=40603174)

Link to the External Link Resolver:

[SFX](https://sfx-86scu.hosted.exlibrisgroup.com.cn/86scu?sid=OVID:embase&id=pmid:15772873&id=doi:10.1007%2Fs10103-004-0325-7&issn=02688921&isbn=&volume=19&issue=4&spage=203&pages=203-209&date=2005&title=Lasers+in+Medical+Science&atitle=Autofluorescence+and+Raman+microspectroscopy+of+tissue+sections+of+oral+lesions&aulast=De+Veld&pid=<author>De+Veld+D.C.G.%3BBakker+Schut+T.C.%3BSkurichina+M.%3BWitjes+M.J.H.%3BVan+Der+Wal+J.E.%3BRoodenburg+J.L.N.%3BSterenborg+H.J.C.M.<%2Fauthor><AN>40603174<%2FAN><DT>Article<%2FDT>)

373.

Micro-Raman spectroscopy for optical pathology of oral squamous cell carcinoma.

Krishna C.M., Sockalingum G.D., Kurien J., Rao L., Venteo L., Pluot M., Manfait M., Kartha V.B.

Applied Spectroscopy. 58(9) (pp 1128-1135), 2004. Date of Publication: September 2004.

AN: 39309014

Micro-Raman spectra of formalin-fixed oral squamous normal and carcinoma tissues, stored at room temperature for 2 months, have been recorded. Spectra were recorded both in the epithelial and subepithelial regions of the tissues. No noticeable spectral contamination due to formalin was observed. Very significant differences between spectra of normal epithelial and malignant epithelial samples were found. No such differences in spectra of subepithelial malignant and subepithelial normal samples could be observed. This study shows that spectra from the epithelial region changes drastically because of malignancy-induced biochemical changes in this region. Major differences between normal and malignant spectra seem to arise from the protein composition, conformational/structural changes, and possible increase in protein content in malignant epithelia. The differences between normal epithelial and subepithelial spectra, as expected, arise mainly from the collagen in subepithelial tissue. Principal component analysis of the combined sets of spectra-epithelial and subepithelial, normal and malignant-showed that very good discrimination can be achieved by Raman microspectroscopy. This study thus validates the suitability of formalin-fixed tissues for optical pathology in oral malignancy. © 2004 Society for Applied Spectroscopy.

PMID

15479531 [<https://www.ncbi.nlm.nih.gov/pubmed/?term=15479531>]

Institution

(Krishna, Kartha) Center for Laser Spectroscopy, Manipal Academy of Higher Education, Manipal-576104, India (Kurien) Department of Surgical Oncology, Manipal Academy of Higher Education, Manipal-576104, India

(Rao) Department of Pathology, Manipal Academy of Higher Education, Manipal-576104, India

(Krishna, Sockalingum, Manfait) Unite MeDIAN, CNRS UMR 6142, UFR Pharmacie, Reims, France

(Venteo, Pluot) Lab. Ctrl. d'Anat./Cytol. Pathol., CHU de Reims, Hopital Robert Debre, Reims, France

Publisher

Society for Applied Spectroscopy (201B Broadway Street, Frederick MD 21702-6501, United States)

Emtree Heading

article; comparative study; evaluation; human; metabolism; methodology; *mouth tumor/di [Diagnosis]; pathology; *Raman spectrometry; reproducibility; sensitivity and specificity; *squamous cell carcinoma/di [Diagnosis]; *tissue fixation; validation study; *formaldehyde; *tumor marker/an [Drug Analysis]; tumor protein/an [Drug Analysis].

Drug Index Terms

*formaldehyde; *tumor marker / *drug analysis; tumor protein / drug analysis.

Other Index Terms

article; comparative study; evaluation; human; metabolism; methodology; *mouth tumor / *diagnosis; pathology; *Raman spectrometry; reproducibility; sensitivity and specificity; *squamous cell carcinoma / *diagnosis; *tissue fixation; validation study.

Link to the Ovid Full Text or citation:

[Click here for full text options](https://ovidsp.ovid.com/ovidweb.cgi?T=JS&CSC=Y&NEWS=N&PAGE=fulltext&D=emed8&AN=39309014)

Link to the External Link Resolver:

[SFX](https://sfx-86scu.hosted.exlibrisgroup.com.cn/86scu?sid=OVID:embase&id=pmid:15479531&id=doi:10.1366%2F0003702041959460&issn=00037028&isbn=&volume=58&issue=9&spage=1128&pages=1128-1135&date=2004&title=Applied+Spectroscopy&atitle=Micro-Raman+spectroscopy+for+optical+pathology+of+oral+squamous+cell+carcinoma&aulast=Krishna&pid=<author>Krishna+C.M.%3BSockalingum+G.D.%3BKurien+J.%3BRao+L.%3BVenteo+L.%3BPluot+M.%3BManfait+M.%3BKartha+V.B.<%2Fauthor><AN>39309014<%2FAN><DT>Article<%2FDT>)

374.

FTIR spectroscopic study on gastric tissue samples from gastroscopy.

Sun X.J., Li Q.B., Shi J.S., Ren Y., Zhang Y.F., Xu D.F., Wu J.G.

Guang pu xue yu guang pu fen xi = Guang pu. 24(8) (pp 933-935), 2004. Date of Publication: Aug 2004.

AN: 41897865

A total of 184 specimens from gastroscopy were studied using Fourier transform infrared (FTIR) spectroscopy. The spectra of small-sized gastric endoscope samples with high quality were measured using the modified FTIR spectrometer with a MCT detector. The results show that the spectra of chronic superficial gastritis are similar to those of the normal block tissue in our previous study. But there are significant differences in band location and relative intensity between the spectra of chronic superficial gastritis and those of normal gastric tissues. Similarly, the differences exist in the spectra of chronic atrophic gastritis and those of malignant gastric tissues. In addition, the subtraction technique can provide more information to identify well the gastric samples of chronic atrophic gastritis, superficial gastritis and gastric cancer from endoscope the detection. These results indicate that FTIR spectroscopy exhibits potential to diagnose the gastric samples from gastroscopy.

PMID

15766111 [<https://www.ncbi.nlm.nih.gov/pubmed/?term=15766111>]

Institution

(Sun) First Hospital of Xi'an Jiaotong University, Xi'an 710061, China.

Emtree Heading

article; atrophic gastritis/di [Diagnosis]; blood; breast tumor/di [Diagnosis]; cancer staging; chemistry; differential diagnosis; fiber optics; *gastritis/di [Diagnosis]; *gastroscope; gastroscopy; Helicobacter infection; human; *infrared spectroscopy; methodology; mouth tumor; pathology; Raman spectrometry; reference value; standard; stomach; stomach mucosa; *stomach tumor/di [Diagnosis]; utilization review; endothelium derived relaxing factor; nitric oxide; tumor marker/an [Drug Analysis]; tumor protein.

Drug Index Terms

endothelium derived relaxing factor; nitric oxide; tumor marker / drug analysis; tumor protein.

Other Index Terms

article; atrophic gastritis / diagnosis; blood; breast tumor / diagnosis; cancer staging; chemistry; differential diagnosis; fiber optics; *gastritis / *diagnosis; *gastroscope; gastroscopy; Helicobacter infection; human; *infrared spectroscopy; methodology; mouth tumor; pathology; Raman spectrometry; reference value; standard; stomach; stomach mucosa; *stomach tumor / *diagnosis; utilization review.

Link to the Ovid Full Text or citation:

[Click here for full text options](https://ovidsp.ovid.com/ovidweb.cgi?T=JS&CSC=Y&NEWS=N&PAGE=fulltext&D=emed8&AN=41897865)

Link to the External Link Resolver:

[SFX](https://sfx-86scu.hosted.exlibrisgroup.com.cn/86scu?sid=OVID:embase&id=pmid:15766111&id=doi:&issn=10000593&isbn=&volume=24&issue=8&spage=933&pages=933-935&date=2004&title=Guang+pu+xue+yu+guang+pu+fen+xi+%3D+Guang+pu&atitle=FTIR+spectroscopic+study+on+gastric+tissue+samples+from+gastroscopy&aulast=Sun&pid=<author>Sun+X.J.%3BLi+Q.B.%3BShi+J.S.%3BRen+Y.%3BZhang+Y.F.%3BXu+D.F.%3BWu+J.G.<%2Fauthor><AN>41897865<%2FAN><DT>Article<%2FDT>)

375.

Raman spectroscopy in chemical bioanalysis.

Baena J.R., Lendl B.

Current Opinion in Chemical Biology. 8(5) (pp 534-539), 2004. Date of Publication: October 2004.

AN: 39278351

Advances in instrumentation are making Raman spectroscopy the tool of choice for an increasing number of (bio)chemical applications. Raman is an interesting option for several reasons, including the sensitivity to small structural changes, non-invasive sampling capability, minimal sample preparation, and high spatial resolution in the case of Raman micro-spectroscopy. Herein we discuss the most recent technical approaches employed, from the well-known surface enhanced resonance Raman spectroscopy to non-linear Raman techniques such as coherent anti-stokes Raman spectroscopy (CARS) and related techniques. Relevant applications of Raman spectroscopy in the fields of clinical pathology, in vivo and ex vivo imaging, classification and detection of microorganisms and chemical analysis in the past three years are also included.

PMID

15450497 [<https://www.ncbi.nlm.nih.gov/pubmed/?term=15450497>]

Institution

(Baena) Analytical Chemistry Department, Annex C-3 Building, Camp. Rabanales, Univ. C. (Lendl) Inst. Chem. Technol. and Analytics, Vienna University of Technology, Getreidemarkt 164-AC, 1060 V., Austria

Publisher

Elsevier Ltd (Langford Lane, Kidlington, Oxford OX5 1GB, United Kingdom)

Emtree Heading

arteriosclerosis/di [Diagnosis]; bacterium identification; Barrett esophagus/di [Diagnosis]; brain cancer/di [Diagnosis]; breast cancer/di [Diagnosis]; cancer diagnosis; cancer research; chemical analysis; confocal laser microscopy; diagnostic procedure; food quality; high performance liquid chromatography; human; imaging; intestine cancer/di [Diagnosis]; larynx cancer/di [Diagnosis]; malaria; microorganism detection; nonhuman; pathology; *Raman spectrometry; review; skin cancer/di [Diagnosis]; uterus cancer/di [Diagnosis]; antimalarial agent/dv [Drug Development]; hematin; silver.

Drug Index Terms

antimalarial agent / drug development; hematin; silver.

Other Index Terms

arteriosclerosis / diagnosis; bacterium identification; Barrett esophagus / diagnosis; brain cancer / diagnosis; breast cancer / diagnosis; cancer diagnosis; cancer research; chemical analysis; confocal laser microscopy; diagnostic procedure; food quality; high performance liquid chromatography; human; imaging; intestine cancer / diagnosis; larynx cancer / diagnosis; malaria; microorganism detection; nonhuman; pathology; *Raman spectrometry; review; skin cancer / diagnosis; uterus cancer / diagnosis.

Link to the Ovid Full Text or citation:

[Click here for full text options](https://ovidsp.ovid.com/ovidweb.cgi?T=JS&CSC=Y&NEWS=N&PAGE=fulltext&D=emed8&AN=39278351)

Link to the External Link Resolver:

[SFX](https://sfx-86scu.hosted.exlibrisgroup.com.cn/86scu?sid=OVID:embase&id=pmid:15450497&id=doi:10.1016%2Fj.cbpa.2004.08.014&issn=13675931&isbn=&volume=8&issue=5&spage=534&pages=534-539&date=2004&title=Current+Opinion+in+Chemical+Biology&atitle=Raman+spectroscopy+in+chemical+bioanalysis&aulast=Baena&pid=<author>Baena+J.R.%3BLendl+B.<%2Fauthor><AN>39278351<%2FAN><DT>Review<%2FDT>)

376.

Normal coordinate analyses of 3,5-dichlorophenylcyanamide.

Badawi H.M., Forner W.

Journal of Molecular Modeling. 10(3) (pp 178-184), 2004. Date of Publication: June 2004.

AN: 38821253

The structure of 3,5-dichlorophenylcyanamide c-C6H3Cl2-NHCN was investigated by DFT-B3LYP and ab initio MP2 calculations with the 6-311+G** basis set. The planar to perpendicular rotational barrier was calculated to be of about 5 kcal mol-1 at both levels of calculation. The stability of the planar structure of the molecule was explained on the basis of conjugation effects between the cyanamide-NHCN moiety and the phenyl c-C6H5 ring in agreement with earlier NMR results. The CNC and the HNC bond angles were calculated to be about 120degree especially by MP2 calculation, which is consistent with sp2 (planar -NH-CN group) and not sp3 (pyramidal -NH-CN group) structure. The vibrational frequencies of the d0, d1 and d3 species of 3,5-dichlorophenylcyanamide and the potential energy distributions among symmetry coordinates of the normal modes of the parent molecule were computed at the DFT-B3LYP level. The calculated infrared and Raman spectra of the molecule were plotted. Complete vibrational assignments were made on the basis of isotopic substitution and normal coordinate calculations. © Springer-Verlag 2004.

PMID

15024632 [<https://www.ncbi.nlm.nih.gov/pubmed/?term=15024632>]

Institution

(Badawi, Forner) Department of Chemistry, King Fahd Univ. of Petroleum/Mineral, 31261 Dhahran, Saudi Arabia

Publisher

Springer Verlag (Tiergartenstrasse 17, Heidelberg D-69121, Germany)

Emtree Heading

ab initio calculation; analytic method; article; chemical bond; chemical structure; computer program; conjugation; controlled study; energy; infrared spectroscopy; intermethod comparison; mathematical computing; molecular stability; nuclear magnetic resonance; plots and curves; priority journal; Raman spectrometry; rotation; structure analysis; substitution reaction; vibration; benzene derivative; *coordination compound; *cyanamide; unclassified drug; vinyl derivative; *3,5 dichlorophenylcyanamide.

Candidate Terms

*3,5 dichlorophenylcyanamide [drug term]

Drug Index Terms

benzene derivative; *coordination compound; *cyanamide; unclassified drug; vinyl derivative.

Other Index Terms

ab initio calculation; analytic method; article; chemical bond; chemical structure; computer program; conjugation; controlled study; energy; infrared spectroscopy; intermethod comparison; mathematical computing; molecular stability; nuclear magnetic resonance; plots and curves; priority journal; Raman spectrometry; rotation; structure analysis; substitution reaction; vibration.

Link to the Ovid Full Text or citation:

[Click here for full text options](https://ovidsp.ovid.com/ovidweb.cgi?T=JS&CSC=Y&NEWS=N&PAGE=fulltext&D=emed8&AN=38821253)

Link to the External Link Resolver:

[SFX](https://sfx-86scu.hosted.exlibrisgroup.com.cn/86scu?sid=OVID:embase&id=pmid:15024632&id=doi:10.1007%2Fs00894-004-0180-2&issn=16102940&isbn=&volume=10&issue=3&spage=178&pages=178-184&date=2004&title=Journal+of+Molecular+Modeling&atitle=Normal+coordinate+analyses+of+3%2C5-dichlorophenylcyanamide&aulast=Badawi&pid=<author>Badawi+H.M.%3BForner+W.<%2Fauthor><AN>38821253<%2FAN><DT>Article<%2FDT>)

377.

Osteoradionecrosis (ORN) of the mandible: a laser Raman spectroscopic study.

Lakshmi R.J., Alexander M., Kurien J., Mahato K.K., Kartha V.B.

Applied spectroscopy. 57(9) (pp 1100-1116), 2003. Date of Publication: Sep 2003.

AN: 137584639

Laser Raman spectroscopy has been used in this study to characterize mandibular bone samples from patients who had undergone radiation therapy for oral cancer. The paper discusses spectral changes resulting in osteoradionecrosis (ORN) of the mandibular bone, a serious complication that may occur after radiation therapy. Histopathological studies normally reveal the radiation damage on vascular canals and loss in bone cells, but will not reveal any structural or biochemical changes. All radiation-induced side effects are attributed to this hypovascularity and hypocellularity caused by early- and/or late-delayed effects. Our Raman studies on normal and ORN bone and on bone exposed to radiation, but not in the ORN state, show that irradiation produces immediate structural changes in the inorganic bone matrix with a slight loss in cells. ORN bone, in addition to the structural changes that had already occurred on radiation exposure, shows almost complete loss of cellular components. Since bone tissue is continuously being remodeled (dissolved and rebuilt) under normal conditions, our results suggest that the immediate structural changes in the calcium hydroxy apatite mineral part is not repaired in ORN, due to loss of the highly transient osteoblasts and osteoclasts resulting from destruction of stem cells. The spectral studies also show changes in the organic matrix, which is mostly type I collagen.

PMID

14611040 [<https://www.ncbi.nlm.nih.gov/pubmed/?term=14611040>]

Institution

(Lakshmi, Alexander, Kurien, Mahato, Kartha) Department of Physics, Manipal Institute of Technology, Manipal Academy of Higher Education, Manipal, Karnataka, India, 576 119.

Emtree Heading

adult; aged; article; *bone necrosis/et [Etiology]; chemistry; comparative study; extracellular matrix; female; human; *jaw disease/et [Etiology]; laser; male; *mandible; metabolism; methodology; middle aged; *mouth tumor/rt [Radiotherapy]; radiation exposure; *Raman spectrometry; reference value; *skull irradiation/ae [Adverse Drug Reaction]; hydroxyapatite.

Drug Index Terms

hydroxyapatite.

Other Index Terms

adult; aged; article; *bone necrosis / *etiology; chemistry; comparative study; extracellular matrix; female; human; *jaw disease / *etiology; laser; male; *mandible; metabolism; methodology; middle aged; *mouth tumor / *radiotherapy; radiation exposure; *Raman spectrometry; reference value; *skull irradiation / *adverse drug reaction.

Link to the Ovid Full Text or citation:

[Click here for full text options](https://ovidsp.ovid.com/ovidweb.cgi?T=JS&CSC=Y&NEWS=N&PAGE=fulltext&D=emed8&AN=137584639)

Link to the External Link Resolver:

[SFX](https://sfx-86scu.hosted.exlibrisgroup.com.cn/86scu?sid=OVID:embase&id=pmid:14611040&id=doi:&issn=00037028&isbn=&volume=57&issue=9&spage=1100&pages=1100-1116&date=2003&title=Applied+spectroscopy&atitle=Osteoradionecrosis+(ORN)+of+the+mandible%3A+a+laser+Raman+spectroscopic+study&aulast=Lakshmi&pid=<author>Lakshmi+R.J.%3BAlexander+M.%3BKurien+J.%3BMahato+K.K.%3BKartha+V.B.<%2Fauthor><AN>137584639<%2FAN><DT>Article<%2FDT>)

378.

Raman spectroscopy for optical diagnosis in normal and cancerous tissue of the nasopharynx - Preliminary findings.

Lau D.P., Huang Z., Lui H., Man C.S., Berean K., Morrison M.D., Zeng H.

Lasers in Surgery and Medicine. 32(3) (pp 210-214), 2003. Date of Publication: 2003.

AN: 36389963

Background and Objectives: Raman spectroscopy (RS), which can detect molecular changes associated with cancer, was explored as a means of distinguishing normal and cancerous nasopharyngeal tissue. Study Design/Patients and Methods: Tissue from six patients with normal and cancerous biopsies was studied using a rapid acquisition Raman spectrometer.

Result(s): Spectra were obtainable within 5 seconds. Consistent differences were noted between normal and cancer tissue in three bands 1,290-1,320 cm-1 (P = 0.005), 1,4201,470 cm-1 (P = 0.006), and 1,530-1,580 cm-1 (P = 0.002).

Conclusion(s): Spectral differences appear to exist between normal and cancerous nasopharyngeal tissue. The ability to obtain spectra rapidly supports the potential for future in vivo application. © 2003 Wiley-Liss, Inc.

PMID

12605428 [<https://www.ncbi.nlm.nih.gov/pubmed/?term=12605428>]

Institution

(Lau, Man, Morrison) Division of Otolaryngology, University of British Columbia, Vancouver General Hospital, Vancouver, BC V5Z 1M9, Canada (Huang, Zeng) Department of Cancer Imaging, British Columbia Cancer Agency, Vancouver General Hospital, Vancouver, BC V5Z 4E6, Canada

(Lui) Division of Dermatology, University of British Columbia, Vancouver General Hospital, Vancouver, BC V5Z 4E8, Canada

(Berean) Department of Pathology, University of British Columbia, Vancouver General Hospital, Vancouver, BC V6T 2B5, Canada

(Zeng) Cancer Imaging Department, BC Cancer Agency, Vancouver Cancer Centre, 600 W 10th Avenue, Vancouver, BC V52 4E6, Canada

Publisher

Wiley-Liss Inc. (111 River Street, Hoboken NJ 07030-5774, United States)

Emtree Heading

adult; article; clinical article; controlled study; diagnostic procedure; disease association; futurology; human; human cell; human tissue; male; nasal biopsy; *nasopharynx cancer/di [Diagnosis]; priority journal; *Raman spectrometry; statistical significance.

Other Index Terms

adult; article; clinical article; controlled study; diagnostic procedure; disease association; futurology; human; human cell; human tissue; male; nasal biopsy; *nasopharynx cancer / *diagnosis; priority journal; *Raman spectrometry; statistical significance.

Link to the Ovid Full Text or citation:

[Click here for full text options](https://ovidsp.ovid.com/ovidweb.cgi?T=JS&CSC=Y&NEWS=N&PAGE=fulltext&D=emed8&AN=36389963)

Link to the External Link Resolver:

[SFX](https://sfx-86scu.hosted.exlibrisgroup.com.cn/86scu?sid=OVID:embase&id=pmid:12605428&id=doi:10.1002%2Flsm.10084&issn=01968092&isbn=&volume=32&issue=3&spage=210&pages=210-214&date=2003&title=Lasers+in+Surgery+and+Medicine&atitle=Raman+spectroscopy+for+optical+diagnosis+in+normal+and+cancerous+tissue+of+the+nasopharynx+-+Preliminary+findings&aulast=Lau&pid=<author>Lau+D.P.%3BHuang+Z.%3BLui+H.%3BMan+C.S.%3BBerean+K.%3BMorrison+M.D.%3BZeng+H.<%2Fauthor><AN>36389963<%2FAN><DT>Article<%2FDT>)

379.

Fourier-transform infrared and Raman spectra, and ab initio calculations for cadmium-n-di-iso-propylphosphorylguanidine-di-chloride (CdDPGCl2) complex.

Tellez C.A., Hollauer E., Felcman J., Lopes D.C., Cattapan R.A.

Spectrochimica acta. Part A, Molecular and biomolecular spectroscopy. 58(9) (pp 1853-1866), 2002. Date of Publication: Jul 2002.

AN: 35542203

Cadmium-n-di-isopropylphosphorylguanidine-di-chloride (CdDPGCl2) was synthesized in the solid phase and characterized previously. The Fourier transform infrared and Raman spectra of (CdDPGCl2) in the solid state were recorded and analyzed. Emphasis was placed on the vibrational assignment of the [(O2P=O-[CdCl2]-HN=C) fragment of the complete molecular structure. With the aim of assisting the vibrational assignment of the experimental spectra, a comparison with the spectra of N-di-isopropylphosphorylguanidine ligand was carried out and ab initio calculations have been performed with several effective core potentials and valence basis sets (Hay-Wadt (HW) and Stevens-Basch-Krauss (SBK)). Due to our limited computational resources, hydrogen atoms replaced the isopropyl groups. The calculated geometrical parameters showed excellent agreement with the experimental, as well as the RHF/MP2 calculated infrared wave numbers, when compared to the IR/Raman experimental wave numbers.

PMID

12164485 [<https://www.ncbi.nlm.nih.gov/pubmed/?term=12164485>]

Institution

(Tellez, Hollauer, Felcman, Lopes, Cattapan) Departamento de Fisico-Quimica, Instituto de Quimica, Universidade Federal Fluminense, Rio de Janeiro, Brazil.

Emtree Heading

article; chemistry; infrared spectroscopy; Raman spectrometry; *cadmium derivative; guanidine derivative; N,N diisopropylphosphorylguanidine; N,N-diisopropylphosphorylguanidine.

Candidate Terms

N,N diisopropylphosphorylguanidine [drug term] N,N-diisopropylphosphorylguanidine [drug term]

Drug Index Terms

*cadmium derivative; guanidine derivative.

Other Index Terms

article; chemistry; infrared spectroscopy; Raman spectrometry.

Link to the Ovid Full Text or citation:

[Click here for full text options](https://ovidsp.ovid.com/ovidweb.cgi?T=JS&CSC=Y&NEWS=N&PAGE=fulltext&D=emed7&AN=35542203)

Link to the External Link Resolver:

[SFX](https://sfx-86scu.hosted.exlibrisgroup.com.cn/86scu?sid=OVID:embase&id=pmid:12164485&id=doi:&issn=13861425&isbn=&volume=58&issue=9&spage=1853&pages=1853-1866&date=2002&title=Spectrochimica+acta.+Part+A%2C+Molecular+and+biomolecular+spectroscopy&atitle=Fourier-transform+infrared+and+Raman+spectra%2C+and+ab+initio+calculations+for+cadmium-n-di-iso-propylphosphorylguanidine-di-chloride+(CdDPGCl2)+complex&aulast=Tellez&pid=<author>Tellez+C.A.%3BHollauer+E.%3BFelcman+J.%3BLopes+D.C.%3BCattapan+R.A.<%2Fauthor><AN>35542203<%2FAN><DT>Article<%2FDT>)

380.

Tissue Raman spectroscopy for the study of radiation damage: Brain irradiation of mice.

Lakshmi R.J., Kartha V.B., Krishna C.M., Solomon J.G.R., Ullas G., Devi P.U.

Radiation Research. 157(2) (pp 175-182), 2002. Date of Publication: 2002.

AN: 34101369

Radiotherapy is routinely employed in the treatment of head and neck cancers. Acute cell death, radiation-activated chemical cascades, and the induction of genes coding for protective factors like cytokines are considered to be the major processes involved in radiation damage and repair. It should be possible to follow these processes by monitoring the biochemical interactions initiated by radiation. We have carried out Raman spectroscopy studies on tissue from mice subjected to brain irradiation to identify the biochemical changes occurring in tissue and brain as a result of radiation injury. These studies show that brain irradiation produces drastic spectral changes even in tissue far removed from the irradiation site. The changes are very similar to those produced by the stress of inoculation and restraint and the administration of an anesthetic drug. While the changes produced by stress or anesthetics last for only a short time (a few hours to 1 or 2 days), radiation-induced changes persist even after 1 week. The spectral changes can be interpreted in terms of the observation of new spectra that are dominated by bands due to proteins. The results thus support the hypothesis that various protective factors are released throughout the body when the central nervous system (CNS) is exposed to radiation. © 2002 by Radiation Research Society.

PMID

11835681 [<https://www.ncbi.nlm.nih.gov/pubmed/?term=11835681>]

Institution

(Lakshmi, Kartha, Krishna, Solomon, Ullas, Devi) Center for Laser Spectroscopy, Manipal Academy of Higher Education, Manipal-576 119, India

Publisher

Radiation Research Society (10105 Cottesmore Court, Great Falls VA 22066-3540, United States)

Emtree Heading

animal experiment; animal tissue; article; *brain injury/di [Diagnosis]; cancer radiotherapy; controlled study; female; head and neck cancer/rt [Radiotherapy]; irradiation; male; mouse; nonhuman; priority journal; radiation hazard; *radiation injury/di [Diagnosis]; Raman spectrometry.

Other Index Terms

animal experiment; animal tissue; article; *brain injury / *diagnosis; cancer radiotherapy; controlled study; female; head and neck cancer / radiotherapy; irradiation; male; mouse; nonhuman; priority journal; radiation hazard; *radiation injury / *diagnosis; Raman spectrometry.

Link to the Ovid Full Text or citation:

[Click here for full text options](https://ovidsp.ovid.com/ovidweb.cgi?T=JS&CSC=Y&NEWS=N&PAGE=fulltext&D=emed7&AN=34101369)

Link to the External Link Resolver:

[SFX](https://sfx-86scu.hosted.exlibrisgroup.com.cn/86scu?sid=OVID:embase&id=pmid:11835681&id=doi:10.1667%2F0033-7587(2002)157%5B0175%3ATRSFTS%5D2.0.CO%3B2&issn=00337587&isbn=&volume=157&issue=2&spage=175&pages=175-182&date=2002&title=Radiation+Research&atitle=Tissue+Raman+spectroscopy+for+the+study+of+radiation+damage%3A+Brain+irradiation+of+mice&aulast=Lakshmi&pid=<author>Lakshmi+R.J.%3BKartha+V.B.%3BKrishna+C.M.%3BSolomon+J.G.R.%3BUllas+G.%3BDevi+P.U.<%2Fauthor><AN>34101369<%2FAN><DT>Article<%2FDT>)

381.

Raman spectra of the serums from cancerous persons.

Guo P., Yi G., Xiong P., Yuan Y., Xie Q., Chen C.

Guang pu xue yu guang pu fen xi = Guang pu. 20(6) (pp 844-846), 2000. Date of Publication: Dec 2000.

AN: 137582883

The serums of different categories, those who are normal people and those who are patient suffering from nasopharyngeal cancer, liver cancer and chronic leukaemia, were determined by laser Raman spectra at the range of 0-2,000 cm-1. The results demonstrated that the serums of those who are suffering from the same disease show different characteristic peak height, but their Raman spectrogram characteristics are almost the same. There exists great difference in the laser Raman spectrogram for the serums of the different kinds of cancerous patients. Near the characteristic peaks of laser Raman spectrograms of serums from the cancerous patients, the normal person's serum has no Raman spectrogram characteristic peak.

PMID

12938490 [<https://www.ncbi.nlm.nih.gov/pubmed/?term=12938490>]

Institution

(Guo, Yi, Xiong, Yuan, Xie, Chen) Department of Physics, Hengyang Medical College, 421001 Hengyang.

Emtree Heading

article; blood; human; laser; *leukemia; *liver tumor; methodology; *nasopharynx tumor; Raman spectrometry.

Other Index Terms

article; blood; human; laser; *leukemia; *liver tumor; methodology; *nasopharynx tumor; Raman spectrometry.

Link to the Ovid Full Text or citation:

[Click here for full text options](https://ovidsp.ovid.com/ovidweb.cgi?T=JS&CSC=Y&NEWS=N&PAGE=fulltext&D=emed7&AN=137582883)

Link to the External Link Resolver:

[SFX](https://sfx-86scu.hosted.exlibrisgroup.com.cn/86scu?sid=OVID:embase&id=pmid:12938490&id=doi:&issn=10000593&isbn=&volume=20&issue=6&spage=844&pages=844-846&date=2000&title=Guang+pu+xue+yu+guang+pu+fen+xi+%3D+Guang+pu&atitle=Raman+spectra+of+the+serums+from+cancerous+persons&aulast=Guo&pid=<author>Guo+P.%3BYi+G.%3BXiong+P.%3BYuan+Y.%3BXie+Q.%3BChen+C.<%2Fauthor><AN>137582883<%2FAN><DT>Article<%2FDT>)

382.

Raman spectroscopy for early detection of laryngeal malignancy: Preliminary results.

Stone N., Stavroulaki P., Kendall C., Birchall M., Barr H.

Laryngoscope. 110(10 I) (pp 1756-1763), 2000. Date of Publication: 2000.

AN: 30764260

Objective: Raman spectroscopy, the analysis of scattered photons after monochromatic laser excitation, is well established in nonbiological sciences. Recently this method has been used to differentiate premalignant and malignant lesions from normal tissue. Its application for early diagnosis has been explored in a variety of sites (e.g., esophagus, cervix), but not, to date, in laryngeal cancer. The objective of this study was to perform a feasibility study of the use of Raman spectroscopy for early diagnosis of laryngeal malignancy.

Method(s): Biopsy specimens were snap-frozen, and top sections were sent for histopathological analysis. Only homogenous samples with clearly defined pathological findings were used in this study: seven histologically normal samples, four exhibiting dysplasia, and four with carcinoma. Samples were defrosted and placed under a Renishaw (Wotton-Under-Edge, UK) System 1000 Raman microspectrometer for analysis. Between 5 and 12 spectra were acquired from each sample, with an excitation wavelength of 830 nm. Average characteristic spectra for each disease or condition were compared. Further multivariate statistical analysis of the data was carried out to evaluate and maximize the differences in the spectra for each disease or condition.

Result(s): The most visible differences in the spectra occur between 850 and 950 cm-1 and 1200 and 1350 cm-1. The later peaks are directly related to protein conformation and C-H bond stretch in nucleic acid bases. The relative intensity of the nucleic acid peak increases with progression to malignancy. Use of linear discriminant analysis made it possible to separate the spectra with disease to a greater degree of accuracy than using empirical peak ratio methods alone. Classification results obtained from cross-validation of the discriminant model showed prediction sensitivities of 83%, 76%, and 92% and specificities of 94%, 91%, and 90% for normal, dysplastic, and squamous cell carcinoma of the larynx, respectively.

Conclusion(s): There was strong evidence to support spectral identification of malignancy and earlier abnormal changes. More substantive studies of the spectral differences between malignant and non-neoplastic tissue are warranted. Raman spectroscopy may become a useful adjunct to pathological diagnosis allowing directed or guided biopsies and assessment of adequacy of resection margins.

PMID

11037840 [<https://www.ncbi.nlm.nih.gov/pubmed/?term=11037840>]

Institution

(Stone, Stavroulaki, Kendall, Birchall, Barr) Senior Clinical Scientist, Cranfield Post Graduate Med. School, Gloucestershire Royal Hospital, Great Western Road, Gloucester GL1 3NN, United Kingdom

Publisher

John Wiley and Sons Inc. (P.O.Box 18667, Newark NJ 07191-8667, United States)

Emtree Heading

adolescent; adult; aged; article; clinical article; controlled study; diagnostic accuracy; diagnostic imaging; early diagnosis; female; human; *larynx carcinoma/di [Diagnosis]; male; priority journal; *Raman spectrometry; spectrometer; squamous epithelium.

Other Index Terms

adolescent; adult; aged; article; clinical article; controlled study; diagnostic accuracy; diagnostic imaging; early diagnosis; female; human; *larynx carcinoma / *diagnosis; male; priority journal; *Raman spectrometry; spectrometer; squamous epithelium.

Link to the Ovid Full Text or citation:

[Click here for full text options](https://ovidsp.ovid.com/ovidweb.cgi?T=JS&CSC=Y&NEWS=N&PAGE=fulltext&D=emed7&AN=30764260)

Link to the External Link Resolver:

[SFX](https://sfx-86scu.hosted.exlibrisgroup.com.cn/86scu?sid=OVID:embase&id=pmid:11037840&id=doi:&issn=0023852X&isbn=&volume=110&issue=10+I&spage=1756&pages=1756-1763&date=2000&title=Laryngoscope&atitle=Raman+spectroscopy+for+early+detection+of+laryngeal+malignancy%3A+Preliminary+results&aulast=Stone&pid=<author>Stone+N.%3BStavroulaki+P.%3BKendall+C.%3BBirchall+M.%3BBarr+H.<%2Fauthor><AN>30764260<%2FAN><DT>Article<%2FDT>)

383.

Nasopharyngeal cancer detection based on blood plasma surface-enhanced Raman spectroscopy and multivariate analysis.

Feng S., Chen R., Lin J., Pan J., Chen G., Li Y., Cheng M., Huang Z., Chen J., Zeng H.

Biosensors and Bioelectronics. (no pagination)

AN: 50887317

A surface-enhanced Raman spectroscopy (SERS) method was developed for blood plasma biochemical analysis for the first time with the aim to develop a simple blood test for non-invasive nasopharyngeal cancer detection. Silver nanoparticles (Ag NP) as the SERS-active nanostructures were directly mixed with blood plasma to enhance the Raman scattering signals of various biomolecular constituents such as proteins, lipids, and nucleic acids. High quality SERS spectrum from blood plasma-Ag NP mixture can be obtained within 10 s using a Renishaw micro-Raman system. SERS measurements were performed on two groups of blood plasma samples: one group from patients (n = 43) with pathologically confirmed nasopharyngeal carcinomas (WHO type I, II, and III) and the other group from healthy volunteers (control subjects, n = 33). Tentative assignments of the Raman bands in the measured SERS spectra suggest interesting cancer specific biomolecular differences, including an increase in the relative amounts of nucleic acid, collagen, phospholipids and phenylalanine and a decrease in the percentage of amino acids and saccharide contents in the blood plasma of nasopharyngeal cancer patients as compared to that of healthy subjects. Principal component analysis (PCA) of the measured SERS spectra separated the spectral features of the two groups into two distinct clusters with little overlaps. Linear discriminate analysis (LDA) based on the PCA generated features differentiated the nasopharyngeal cancer SERS spectra from normal SERS spectra with high sensitivity (90.7%) and specificity (100%). The results from this exploratory study demonstrated great potentials for developing SERS blood plasma analysis into a novel clinical tool for non-invasive detection of nasopharyngeal cancers. © 2010 Elsevier B.V. All rights reserved.

Institution

(Feng, Chen, Lin, Chen, Li, Huang, Chen) Key Laboratory of OptoElectronic Science and Technology for Medicine, Ministry of Education, Fujian Normal University, Fuzhou 350007, China (Pan) Fujian Provincial Tumor Hospital, Fuzhou 350001, China

(Feng) College of Chemistry and Material Science, Experimental Center, Fujian Normal University, Fuzhou 350007 China

(Cheng) College of Minjiang, Fuzhou 350001 China

(Zeng) Cancer Imaging Department, British Columbia Cancer Research Centre, 675 West 10th Avenue, Vancouver, BC V5Z 1L3, Canada

Emtree Heading

*plasma; *Raman spectrometry; *nasopharynx cancer; *cancer diagnosis; *multivariate analysis; normal human; chemical analysis; blood; patient; nasopharynx carcinoma; neoplasm; cancer patient; principal component analysis; sensitivity and specificity; *silver nanoparticle; nucleic acid; silver; nanomaterial; protein; lipid; collagen; phospholipid; phenylalanine; amino acid; carbohydrate.

Drug Index Terms

*silver nanoparticle; nucleic acid; silver; nanomaterial; protein; lipid; collagen; phospholipid; phenylalanine; amino acid; carbohydrate.

Other Index Terms

*plasma; *Raman spectrometry; *nasopharynx cancer; *cancer diagnosis; *multivariate analysis; normal human; chemical analysis; blood; patient; nasopharynx carcinoma; neoplasm; cancer patient; principal component analysis; sensitivity and specificity.

Link to the Ovid Full Text or citation:

[Click here for full text options](https://ovidsp.ovid.com/ovidweb.cgi?T=JS&CSC=Y&NEWS=N&PAGE=fulltext&D=emexb&AN=50887317)

Link to the External Link Resolver:

[SFX](https://sfx-86scu.hosted.exlibrisgroup.com.cn/86scu?sid=OVID:embase&id=pmid:&id=doi:10.1016%2Fj.bios.2010.03.033&issn=09565663&isbn=&volume=25&issue=11&spage=2414&pages=&date=2010&title=Biosensors+and+Bioelectronics&atitle=Nasopharyngeal+cancer+detection+based+on+blood+plasma+surface-enhanced+Raman+spectroscopy+and+multivariate+analysis&aulast=Feng&pid=<author>Feng+S.%3BChen+R.%3BLin+J.%3BPan+J.%3BChen+G.%3BLi+Y.%3BCheng+M.%3BHuang+Z.%3BChen+J.%3BZeng+H.<%2Fauthor><AN>50887317<%2FAN><DT>Article+In+Press<%2FDT>)
