# Supplementary material for: The accuracy of Fiber-Optic Raman Spectroscopy in the detection and diagnosis of head and neck neoplasm in vivo: a systematic review and meta-analysis
Source: PeerJ. 2023 Dec 11;11:e16536. doi: 10.7717/peerj.16536 (PMC10720414; doi:10.7717/peerj.16536)
Supplement: Supplemental Information 3 [file peerj-11-16536-s003.doc]

Database: Ovid MEDLINE(R) ALL <1946 to March 17, 2023>

Search Strategy:

--------------------------------------------------------------------------------

1 (head and neck neoplasms).mp. [mp=title, book title, abstract, original title, name of substance word, subject heading word, floating sub-heading word, keyword heading word, organism supplementary concept word, protocol supplementary concept word, rare disease supplementary concept word, unique identifier, synonyms, population supplementary concept word, anatomy supplementary concept word] (64565)

2 Facial Neoplasms.mp. or Facial Neoplasms/ (8498)

3 Eyelid Neoplasms.mp. or Eyelid Neoplasms/ (4860)

4 Mouth Neoplasms.mp. or Mouth Neoplasms/ (39809)

5 Gingival Neoplasms.mp. or Gingival Neoplasms/ (2457)

6 Leukoplakia, Oral.mp. or Leukoplakia, Oral/ (3690)

7 Leukoplakia, Hairy.mp. or Leukoplakia, Hairy/ (349)

8 Lip Neoplasms.mp. or Lip Neoplasms/ (4351)

9 Palatal Neoplasms.mp. or Palatal Neoplasms/ (3040)

10 Salivary Gland Neoplasms.mp. or Salivary Gland Neoplasms/ (9973)

11 Parotid Neoplasms.mp. or Parotid Neoplasms/ (9490)

12 Sublingual Gland Neoplasms.mp. or Sublingual Gland Neoplasms/ (151)

13 Submandibular Gland Neoplasms.mp. or Submandibular Gland Neoplasms/ (1197)

14 Tongue Neoplasms.mp. or Tongue Neoplasms/ (10875)

15 Otorhinolaryngologic Neoplasms.mp. or Otorhinolaryngologic Neoplasms/ (1709)

16 Laryngeal Neoplasms.mp. or Laryngeal Neoplasms/ (29059)

17 Nose Neoplasms.mp. or Nose Neoplasms/ (11038)

18 Paranasal Sinus Neoplasms.mp. or Paranasal Sinus Neoplasms/ (8196)

19 Maxillary Sinus Neoplasms.mp. or Maxillary Sinus Neoplasms/ (2061)

20 Pharyngeal Neoplasms.mp. or Pharyngeal Neoplasms/ (7929)

21 Hypopharyngeal Neoplasms.mp. or Hypopharyngeal Neoplasms/ (3519)

22 Nasopharyngeal Neoplasms.mp. or Nasopharyngeal Neoplasms/ (18492)

23 Nasopharyngeal Carcinoma.mp. or Nasopharyngeal Carcinoma/ (15943)

24 Oropharyngeal Neoplasms.mp. or Oropharyngeal Neoplasms/ (7233)

25 Tonsillar Neoplasms.mp. or Tonsillar Neoplasms/ (2886)

26 Parathyroid Neoplasms.mp. or Parathyroid Neoplasms/ (8468)

27 (Squamous Cell Carcinoma of Head and Neck).mp. [mp=title, book title, abstract, original title, name of substance word, subject heading word, floating sub-heading word, keyword heading word, organism supplementary concept word, protocol supplementary concept word, rare disease supplementary concept word, unique identifier, synonyms, population supplementary concept word, anatomy supplementary concept word] (11119)

28 Thyroid Neoplasms.mp. or Thyroid Neoplasms/ (57032)

29 Thyroid Cancer, Papillary.mp. or Thyroid Cancer, Papillary/ (6543)

30 Thyroid Nodule/ or Thyroid Nodule.mp. (10074)

31 Tracheal Neoplasms.mp. or Tracheal Neoplasms/ (3909)

32 (Neoplasms, Head and Neck).mp. [mp=title, book title, abstract, original title, name of substance word, subject heading word, floating sub-heading word, keyword heading word, organism supplementary concept word, protocol supplementary concept word, rare disease supplementary concept word, unique identifier, synonyms, population supplementary concept word, anatomy supplementary concept word] (6)

33 Head, Neck Neoplasms.mp. or "Head and Neck Neoplasms"/ (63783)

34 (Cancer of Head and Neck).mp. [mp=title, book title, abstract, original title, name of substance word, subject heading word, floating sub-heading word, keyword heading word, organism supplementary concept word, protocol supplementary concept word, rare disease supplementary concept word, unique identifier, synonyms, population supplementary concept word, anatomy supplementary concept word] (1191)

35 (Head and Neck Cancer).mp. [mp=title, book title, abstract, original title, name of substance word, subject heading word, floating sub-heading word, keyword heading word, organism supplementary concept word, protocol supplementary concept word, rare disease supplementary concept word, unique identifier, synonyms, population supplementary concept word, anatomy supplementary concept word] (31188)

36 (Cancer of the Head and Neck).mp. [mp=title, book title, abstract, original title, name of substance word, subject heading word, floating sub-heading word, keyword heading word, organism supplementary concept word, protocol supplementary concept word, rare disease supplementary concept word, unique identifier, synonyms, population supplementary concept word, anatomy supplementary concept word] (3150)

37 Head Neoplasms.mp. or "Head and Neck Neoplasms"/ (63796)

38 Neoplasms, Head.mp. or "Head and Neck Neoplasms"/ (63781)

39 Neck Neoplasms.mp. or "Head and Neck Neoplasms"/ (64581)

40 Neoplasms, Neck.mp. or "Head and Neck Neoplasms"/ (63779)

41 Cancer of Head.mp. or "Head and Neck Neoplasms"/ (64578)

42 Head Cancer.mp. or "Head and Neck Neoplasms"/ (64533)

43 Cancer of the Head.mp. or "Head and Neck Neoplasms"/ (65507)

44 Cancer of Neck.mp. or "Head and Neck Neoplasms"/ (63867)

45 Neck Cancer.mp. or "Head and Neck Neoplasms"/ (74613)

46 Cancer of the Neck.mp. or "Head and Neck Neoplasms"/ (64359)

47 ((nasopharyn$ or oropharyn$ or laryn$ or glotti$ or tonsil$ or epiglotti$ or oral cavity or oral or tongue or gingiva$ or bucca$ or lip or palat$ or gum or mouth floor or floor of mouth or lingual or (head and neck) or HN) adj4 (cancer$ or tumor$ or tumor$ or neoplasm$ or carcinoma$ or squamous cell carcinoma or SCC)).mp. [mp=title, book title, abstract, original title, name of substance word, subject heading word, floating sub-heading word, keyword heading word, organism supplementary concept word, protocol supplementary concept word, rare disease supplementary concept word, unique identifier, synonyms, population supplementary concept word, anatomy supplementary concept word] (200983)

48 ((cancer$ or tumor$ or tumor$ or neoplasm$ or carcinoma$ or squamous cell carcinoma or SCC) adj4 (nasopharyn$ or oropharyn$ or laryn$ or glotti$ or tonsil$ or epiglotti$ or oral cavity or oral or tongue or gingiva$ or bucca$ or lip or palat$ or gum or mouth floor or floor of mouth or lingual or (head and neck) or HN)).mp. [mp=title, book title, abstract, original title, name of substance word, subject heading word, floating sub-heading word, keyword heading word, organism supplementary concept word, protocol supplementary concept word, rare disease supplementary concept word, unique identifier, synonyms, population supplementary concept word, anatomy supplementary concept word] (200983)

49 (HNSCC or SCCHN or HNC or OSCC or OCSCC or OPSCC or LSCC or NPC).mp. [mp=title, book title, abstract, original title, name of substance word, subject heading word, floating sub-heading word, keyword heading word, organism supplementary concept word, protocol supplementary concept word, rare disease supplementary concept word, unique identifier, synonyms, population supplementary concept word, anatomy supplementary concept word] (46390)

50 spectrum analysis, raman.mp. or Spectrum Analysis, Raman/ (26423)

51 Raman Spectrum Analysis.mp. or Spectrum Analysis, Raman/ (26456)

52 Raman Spectroscopy.mp. or Spectrum Analysis, Raman/ (46743)

53 Spectroscopy, Raman.mp. or Spectrum Analysis, Raman/ (27276)

54 Analysis, Raman Spectrum.mp. or Spectrum Analysis, Raman/ (26420)

55 Raman Optical Activity Spectroscopy.mp. or Spectrum Analysis, Raman/ (26425)

56 Raman Scattering.mp. or Spectrum Analysis, Raman/ (35688)

57 Scattering, Raman.mp. or Spectrum Analysis, Raman/ (26480)

58 1 or 2 or 3 or 4 or 5 or 6 or 7 or 8 or 9 or 10 or 11 or 12 or 13 or 14 or 15 or 16 or 17 or 18 or 19 or 20 or 21 or 22 or 23 or 24 or 25 or 26 or 27 or 28 or 29 or 30 or 31 or 32 or 33 or 34 or 35 or 36 or 37 or 38 or 39 or 40 or 41 or 42 or 43 or 44 or 45 or 46 or 47 or 48 or 49 (340903)

59 50 or 51 or 52 or 53 or 54 or 55 or 56 or 57 (54964)

60 58 and 59 (257)

***************************

1.

Serum Raman spectroscopy: Prognostic applications in oral cancers.

Saha P, Sawant S, Deshmukh A, Hole A, Murali Krishna C

Head & Neck. 2023 Mar 15.

[Journal Article]

UI: 36919570

BACKGROUND: Loco-regional recurrences attributable to field cancerization and minimal residual cancer, remain prime causes of mortality in oral cancer (OC) subjects. The current study evaluates potential of serum Raman spectroscopy (SRS) to identify recurrence-prone OC subjects.

METHODS: Raman spectra of serum from eight healthy subjects (H) and 57 OC subjects (with-recurrence [R], without-recurrence [NR], and with suspicious-lesions [S]), before (BS) and after (AS) surgical excision of tumor were recorded. OC subjects were followed-up for 7-years.

RESULTS: DNA and protein alterations were observed in AS sera of all groups. 4-, 3-, and 2-model multivariate analyses were used to stratify BS and AS groups. H spectra were 100% distinguishable from all other groups. AS, R and NR were distinguished with high accuracy (84%) in all models. No stratification (~50%) was observed BS.

CONCLUSION: SRS shows potential to identify recurrence prone subjects, post-surgery, using serum collected as early as 1 week after surgery.

Copyright © 2023 Wiley Periodicals LLC.

Version ID

1

Record Owner

From MEDLINE, a database of the U.S. National Library of Medicine.

Status

Publisher

Author NameID

Murali Krishna, C; ORCID: <https://orcid.org/0000-0002-4974-8533>

Authors Full Name

Saha, Panchali, Sawant, Sharada, Deshmukh, Atul, Hole, Arti, Murali Krishna, C

Institution

Saha, Panchali. Tata Memorial Centre, Advanced Centre for Treatment, Education and Research in Cancer, Navi Mumbai, Maharashtra, India. Saha, Panchali. Training School Complex, Homi Bhabha National Institute, Anushakti Nagar, Maharashtra, India.

Sawant, Sharada. Tata Memorial Centre, Advanced Centre for Treatment, Education and Research in Cancer, Navi Mumbai, Maharashtra, India.

Deshmukh, Atul. Centre for Interdisciplinary Research, D.Y. Patil University, Navi Mumbai, Maharashtra, India.

Hole, Arti. Tata Memorial Centre, Advanced Centre for Treatment, Education and Research in Cancer, Navi Mumbai, Maharashtra, India.

Murali Krishna, C. Tata Memorial Centre, Advanced Centre for Treatment, Education and Research in Cancer, Navi Mumbai, Maharashtra, India.

Murali Krishna, C. Training School Complex, Homi Bhabha National Institute, Anushakti Nagar, Maharashtra, India.

Keyword Heading

Raman spectroscopy loco-regional recurrence

oral squamous cell carcinoma

serum.

Keyword Heading Owner

NOTNLM

Year of Publication

2023

Link to the Ovid Full Text or citation:

[Click here for full text options](https://ovidsp.ovid.com/ovidweb.cgi?T=JS&CSC=Y&NEWS=N&PAGE=fulltext&D=medp&AN=36919570)

Link to the External Link Resolver:

[SFX](https://sfx-86scu.hosted.exlibrisgroup.com.cn/86scu?sid=OVID:medline&id=pmid:36919570&id=doi:10.1002%2Fhed.27338&issn=10433074&isbn=&volume=&issue=&spage=&pages=&date=2023&title=Head+%26+Neck&atitle=Serum+Raman+spectroscopy%3A+Prognostic+applications+in+oral+cancers.&aulast=Saha&pid=<author>Saha+P%3BSawant+S%3BDeshmukh+A%3BHole+A%3BMurali+Krishna+C<%2Fauthor><AN>36919570<%2FAN><DT>Journal+Article<%2FDT>)

2.

Mobile multi-configuration clinical translational Raman system for oral cancer application.

Maryam S, Konugolu Venkata Sekar S, Ghauri MD, Fahy E, Nogueira MS, Lu H, Beffara F, Humbert G, Ni Riordain R, Sheahan P, Burke R, Wei Kho K, Gautam R, Andersson-Engels S

Analyst. 2023 Mar 10.

[Journal Article]

UI: 36896767

Early diagnosis of oral cancer is critical to improve the survival rate of patients. Raman spectroscopy, a non-invasive spectroscopic technique, has shown potential in identifying early-stage oral cancer biomarkers in the oral cavity environment. However, inherently weak signals necessitate highly sensitive detectors, which restricts widespread usage due to high setup costs. In this research, the fabrication and assembly of a customised Raman system that can adapt three different configurations for the in vivo and ex vivo analysis is reported. This novel design will help in reducing the cost required to have multiple Raman instruments specific for a given application. First, we demonstrated the capability of a customized microscope for acquiring Raman signals from a single cell with high signal-to-noise ratio. Generally, when working with liquid samples with low concentration of analytes (such as saliva) under a microscope, excitation light interacts with a small sample volume, which may not be representative of whole sample. To address this issue, we have designed a novel long-path transmission set-up, which was found to be sensitive towards low concentration of analytes in aqueous solution. We further demonstrated that the same Raman system can be incorporated with the multimodal fibre optical probe to collect in vivo data from oral tissues. In summary, this flexible, portable, multi-configuration Raman system has the potential to provide a cost-effective solution for complete screening of precancer oral lesions.

Version ID

1

Record Owner

From MEDLINE, a database of the U.S. National Library of Medicine.

Status

Publisher

Author NameID

Maryam, Siddra; ORCID: <http://orcid.org/0000-0003-2644-2088> Gautam, Rekha; ORCID: <http://orcid.org/0000-0002-1176-8491>

Andersson-Engels, Stefan; ORCID: <http://orcid.org/0000-0001-5640-3122>

Authors Full Name

Maryam, Siddra, Konugolu Venkata Sekar, Sanathana, Ghauri, M Daniyal, Fahy, Edward, Nogueira, Marcelo Saito, Lu, Huihui, Beffara, Flavien, Humbert, Georges, Ni Riordain, Richeal, Sheahan, Patrick, Burke, Ray, Wei Kho, Kiang, Gautam, Rekha, Andersson-Engels, Stefan

Institution

Maryam, Siddra. Tyndall National Institute, University College Cork, Cork, Ireland. siddra.maryam@tyndall.ie. Konugolu Venkata Sekar, Sanathana. Tyndall National Institute, University College Cork, Cork, Ireland. siddra.maryam@tyndall.ie.

Ghauri, M Daniyal. Tyndall National Institute, University College Cork, Cork, Ireland. siddra.maryam@tyndall.ie.

Fahy, Edward. Cork University Dental School and Hospital, Wilton, Cork, Ireland.

Nogueira, Marcelo Saito. Tyndall National Institute, University College Cork, Cork, Ireland. siddra.maryam@tyndall.ie.

Lu, Huihui. Tyndall National Institute, University College Cork, Cork, Ireland. siddra.maryam@tyndall.ie.

Beffara, Flavien. XLIM Research Institute, UMR 7252 CNRS/Limoges University, Limoges, France.

Humbert, Georges. XLIM Research Institute, UMR 7252 CNRS/Limoges University, Limoges, France.

Ni Riordain, Richeal. Cork University Dental School and Hospital, Wilton, Cork, Ireland.

Ni Riordain, Richeal. ENTO Research Institute, University College Cork, Cork, Ireland.

Sheahan, Patrick. South Infirmary Victoria University Hospital, Cork, Ireland.

Burke, Ray. Tyndall National Institute, University College Cork, Cork, Ireland. siddra.maryam@tyndall.ie.

Wei Kho, Kiang. Tyndall National Institute, University College Cork, Cork, Ireland. siddra.maryam@tyndall.ie.

Gautam, Rekha. Tyndall National Institute, University College Cork, Cork, Ireland. siddra.maryam@tyndall.ie.

Andersson-Engels, Stefan. Tyndall National Institute, University College Cork, Cork, Ireland. siddra.maryam@tyndall.ie.

Year of Publication

2023

Link to the Ovid Full Text or citation:

[Click here for full text options](https://ovidsp.ovid.com/ovidweb.cgi?T=JS&CSC=Y&NEWS=N&PAGE=fulltext&D=medp&AN=36896767)

Link to the External Link Resolver:

[SFX](https://sfx-86scu.hosted.exlibrisgroup.com.cn/86scu?sid=OVID:medline&id=pmid:36896767&id=doi:10.1039%2Fd2an01921c&issn=00032654&isbn=&volume=&issue=&spage=&pages=&date=2023&title=Analyst&atitle=Mobile+multi-configuration+clinical+translational+Raman+system+for+oral+cancer+application.&aulast=Maryam&pid=<author>Maryam+S%3BKonugolu+Venkata+Sekar+S%3BGhauri+MD%3BFahy+E%3BNogueira+MS%3BLu+H%3BBeffara+F%3BHumbert+G%3BNi+Riordain+R%3BSheahan+P%3BBurke+R%3BWei+Kho+K%3BGautam+R%3BAndersson-Engels+S<%2Fauthor><AN>36896767<%2FAN><DT>Journal+Article<%2FDT>)

3.

Shifted-excitation Raman difference spectroscopy for improving in vivo detection of nasopharyngeal carcinoma.

Lin J, Lin D, Qiu S, Huang Z, Liu F, Huang W, Xu Y, Zhang X, Feng S

Talanta. 257:124330, 2023 May 15.

[Journal Article]

UI: 36773510

A strong fluorescence background is one of the common interference factors of Raman spectroscopic analysis in biological tissue. This study developed an endoscopic shifted-excitation Raman difference spectroscopy (SERDS) system for real-time in vivo detection of nasopharyngeal carcinoma (NPC) for the first time. Owing to the use of the SERDS method, the high-quality Raman signals of nasopharyngeal tissue could be well extracted and characterized from the complex raw spectra by removing the fluorescence interference signals. Significant spectral differences relating to proteins, phospholipids, glucose, and DNA were found between 42 NPC and 42 normal tissue sites. Using linear discriminant analysis, the diagnostic accuracy of SERDS for NPC detection was 100%, which was much higher than that of raw Raman spectroscopy (75.0%), showing the great potential of SERDS for improving the accurate in vivo detection of NPC.

Copyright © 2023. Published by Elsevier B.V.

Version ID

1

Record Owner

From MEDLINE, a database of the U.S. National Library of Medicine.

Status

MEDLINE

Authors Full Name

Lin, Jinyong, Lin, Duo, Qiu, Sufang, Huang, Zufang, Liu, Feng, Huang, Wei, Xu, Yuanji, Zhang, Xianzeng, Feng, Shangyuan

Institution

Lin, Jinyong. Clinical Oncology School of Fujian Medical University, Fujian Cancer Hospital, Fuzhou, 350014, China; Key Laboratory of OptoElectronic Science and Technology for Medicine, Ministry of Education, Fujian Provincial Key Laboratory for Photonics Technology, Fujian Normal University, Fuzhou, 350007, China. Lin, Duo. Key Laboratory of OptoElectronic Science and Technology for Medicine, Ministry of Education, Fujian Provincial Key Laboratory for Photonics Technology, Fujian Normal University, Fuzhou, 350007, China.

Qiu, Sufang. Clinical Oncology School of Fujian Medical University, Fujian Cancer Hospital, Fuzhou, 350014, China.

Huang, Zufang. Key Laboratory of OptoElectronic Science and Technology for Medicine, Ministry of Education, Fujian Provincial Key Laboratory for Photonics Technology, Fujian Normal University, Fuzhou, 350007, China. Electronic address: zfhuang@fjnu.edu.cn.

Liu, Feng. Simple & Smart Instrument (Beijing) Co.,Ltd, China.

Huang, Wei. Department of Forensic Science, Fujian Police College, Fuzhou, 350007, PR China.

Xu, Yuanji. Clinical Oncology School of Fujian Medical University, Fujian Cancer Hospital, Fuzhou, 350014, China.

Zhang, Xianzeng. Key Laboratory of OptoElectronic Science and Technology for Medicine, Ministry of Education, Fujian Provincial Key Laboratory for Photonics Technology, Fujian Normal University, Fuzhou, 350007, China. Electronic address: xzzhang@fjnu.edu.cn.

Feng, Shangyuan. Key Laboratory of OptoElectronic Science and Technology for Medicine, Ministry of Education, Fujian Provincial Key Laboratory for Photonics Technology, Fujian Normal University, Fuzhou, 350007, China. Electronic address: syfeng@fjnu.edu.cn.

MeSH Heading

Humans. Nasopharyngeal Carcinoma. Spectrum Analysis, Raman/mt [Methods]. *Spectrum Analysis, Raman. Discriminant Analysis. DNA. Nasopharyngeal Neoplasms/ch [Chemistry]. Nasopharyngeal Neoplasms/di [Diagnosis]. *Nasopharyngeal Neoplasms.

Keyword Heading

Endoscopy system In vivo detection

Nasopharyngeal carcinoma

Shifted-excitation Raman difference spectroscopy (SERDS).

Keyword Heading Owner

NOTNLM

Registry Number/Name of Substance

9007-49-2 (DNA).

Year of Publication

2023

Link to the Ovid Full Text or citation:

[Click here for full text options](https://ovidsp.ovid.com/ovidweb.cgi?T=JS&CSC=Y&NEWS=N&PAGE=fulltext&D=mesx&AN=36773510)

Link to the External Link Resolver:

[SFX](https://sfx-86scu.hosted.exlibrisgroup.com.cn/86scu?sid=OVID:medline&id=pmid:36773510&id=doi:10.1016%2Fj.talanta.2023.124330&issn=00399140&isbn=&volume=257&issue=&spage=124330&pages=124330&date=2023&title=Talanta&atitle=Shifted-excitation+Raman+difference+spectroscopy+for+improving+in+vivo+detection+of+nasopharyngeal+carcinoma.&aulast=Lin&pid=<author>Lin+J%3BLin+D%3BQiu+S%3BHuang+Z%3BLiu+F%3BHuang+W%3BXu+Y%3BZhang+X%3BFeng+S<%2Fauthor><AN>36773510<%2FAN><DT>Journal+Article<%2FDT>)

4.

CuO decorated vacancy-rich CeO2 nanopencils for highly efficient catalytic NO reduction by CO at low temperature.

Wang F, Yu Z, Zhai S, Li Y, Xu Y, Ye Y, Wei X, Xu J, Xue B

Environmental Science & Pollution Research. 30(11):31895-31904, 2023 Mar.

[Journal Article]

UI: 36459322

With the rapid development of transportation and vehicles, the elimination of NOx and CO has highly attracted public attention. In this work, vacancy-rich CeO2 nanopencil supported CuO catalysts (CuO/CeO2-NPC) were successfully prepared for NO reduction by CO. Importantly, CeO2 with nanopencil-like shape (CeO2-NPC) have been synthesis by solvothermal method for the first time. The physicochemical properties of all samples were studied in detail by combining the means of X-ray diffraction (XRD), Raman spectroscopy, electron paramagnetic resonance (EPR), X-ray photoelectron spectroscopy (XPS), H2-temperature-programmed reduction (H2-TPR), transmission electron microscopy (TEM), scanning electron microscopy (SEM), N2 physisorption (Brunauer-Emmett-Teller), and NO and CO temperature-programmed desorption (NO-TPD and CO-TPD) techniques. Compared with CeO2 nanorods and nanoparticles supported CuO catalysts (CuO/CeO2-NR and CuO/CeO2-NP), the CuO/CeO2-NPC catalysts showed the highest catalytic activity, affording more than 90% NO conversion at 69 degreeC as well as excellent H2O tolerance at 150 degreeC, which is superior to catalysts previously reported. Characterization results indicated that the synergistic effect between the well-dispersed CuO and the CeO2 nanopencil support enables a favorable electron transfer between these components and enhances the density of surface oxygen vacancies and Cu+ species, which consequently accelerating the redox cycle. The results indicated that the morphology control of CeO2 support could be an efficient way to evidently enhance the catalytic performance for NO + CO reaction.

Copyright © 2022. The Author(s), under exclusive licence to Springer-Verlag GmbH Germany, part of Springer Nature.

Version ID

1

Record Owner

From MEDLINE, a database of the U.S. National Library of Medicine.

Status

MEDLINE

Author NameID

Wang, Fei; ORCID: <http://orcid.org/0000-0003-3475-1163>

Authors Full Name

Wang, Fei, Yu, Zairan, Zhai, Shuai, Li, Yuanyuan, Xu, Yang, Ye, Yuyang, Wei, Xuejiao, Xu, Jie, Xue, Bing

Institution

Wang, Fei. Advanced Catalysis and Green Manufacturing Collaborative Innovation Center, School of Petrochemical and Engineering, Changzhou University, Changzhou, 213164, People's Republic of China. wangfei@cczu.edu.cn. Yu, Zairan. Advanced Catalysis and Green Manufacturing Collaborative Innovation Center, School of Petrochemical and Engineering, Changzhou University, Changzhou, 213164, People's Republic of China.

Zhai, Shuai. Advanced Catalysis and Green Manufacturing Collaborative Innovation Center, School of Petrochemical and Engineering, Changzhou University, Changzhou, 213164, People's Republic of China.

Li, Yuanyuan. Advanced Catalysis and Green Manufacturing Collaborative Innovation Center, School of Petrochemical and Engineering, Changzhou University, Changzhou, 213164, People's Republic of China.

Xu, Yang. Advanced Catalysis and Green Manufacturing Collaborative Innovation Center, School of Petrochemical and Engineering, Changzhou University, Changzhou, 213164, People's Republic of China.

Ye, Yuyang. Advanced Catalysis and Green Manufacturing Collaborative Innovation Center, School of Petrochemical and Engineering, Changzhou University, Changzhou, 213164, People's Republic of China.

Wei, Xuejiao. School of Chemical Engineering and Materials, Changzhou Institute of Technology, Changzhou, 213032, People's Republic of China.

Xu, Jie. Advanced Catalysis and Green Manufacturing Collaborative Innovation Center, School of Petrochemical and Engineering, Changzhou University, Changzhou, 213164, People's Republic of China.

Xue, Bing. Advanced Catalysis and Green Manufacturing Collaborative Innovation Center, School of Petrochemical and Engineering, Changzhou University, Changzhou, 213164, People's Republic of China.

MeSH Heading

Temperature. Cerium/ch [Chemistry]. *Cerium. Cold Temperature. Copper/ch [Chemistry].

Keyword Heading

CuO/CeO2 NO reduction by CO

Nanopencils

Oxygen vacancies.

Keyword Heading Owner

NOTNLM

Registry Number/Name of Substance

V1XJQ704R4 (cupric oxide). 30K4522N6T (Cerium). 789U1901C5 (Copper).

Year of Publication

2023

Link to the Ovid Full Text or citation:

[Click here for full text options](https://ovidsp.ovid.com/ovidweb.cgi?T=JS&CSC=Y&NEWS=N&PAGE=fulltext&D=medl&AN=36459322)

Link to the External Link Resolver:

[SFX](https://sfx-86scu.hosted.exlibrisgroup.com.cn/86scu?sid=OVID:medline&id=pmid:36459322&id=doi:10.1007%2Fs11356-022-24508-1&issn=09441344&isbn=&volume=30&issue=11&spage=31895&pages=31895-31904&date=2023&title=Environmental+Science+%26+Pollution+Research&atitle=CuO+decorated+vacancy-rich+CeO2+nanopencils+for+highly+efficient+catalytic+NO+reduction+by+CO+at+low+temperature.&aulast=Wang&pid=<author>Wang+F%3BYu+Z%3BZhai+S%3BLi+Y%3BXu+Y%3BYe+Y%3BWei+X%3BXu+J%3BXue+B<%2Fauthor><AN>36459322<%2FAN><DT>Journal+Article<%2FDT>)

5.

High-Precision Detection of Cellular Drug Response Based on SERS Spectrum and Multivariate Statistical Analysis.

Wu F, Wu Z, Wang X, Liu Y, Ye Q

Biosensors. 13(2), 2023 Feb 08.

[Journal Article]

UI: 36832007

The rapid development of personalized medicine places high demands on the control of drug dose and cellular drug response to provide patients with better curative effects and low side effects. To solve the problem of low detection accuracies of the cell-counting kit-8 (CCK8) method, a detection method based on surface-enhanced Raman spectroscopy (SERS) of cell-secreted proteins was adopted to evaluate the concentration of the anticancer drug cisplatin and the cellular drug response of nasopharyngeal carcinoma. CNE1 and NP69 cell lines were used to evaluate cisplatin response. The results showed that the combination of the SERS spectrum with principal component analysis-linear discriminant analysis could detect the difference in the response of cisplatin with a concentration difference of 1 mug/mL, which considerably exceeded that of CCK8. In addition, the SERS spectral peak intensity of the cell-secreted proteins strongly correlated with the cisplatin concentration. Furthermore, the mass spectrum of the secreted proteins of the nasopharyngeal carcinoma cells was analyzed to verify the results obtained using the SERS spectrum. The results demonstrated that SERS of secreted proteins has great potential for high-precision detection of chemotherapeutic drug response.

Version ID

1

Record Owner

From MEDLINE, a database of the U.S. National Library of Medicine.

Status

MEDLINE

Author NameID

Wu, Fengfang; ORCID: <https://orcid.org/0000-0002-4415-8171>

Authors Full Name

Wu, Fengfang, Wu, Zhiwei, Wang, Xiaoyan, Liu, Yunliang, Ye, Qing

Institution

Wu, Fengfang. Shengli Clinical Medical College of Fujian Medical University, Fuzhou 350001, China. Wu, Fengfang. Department of Otolaryngology, Head and Neck Surgery, Quanzhou First Hospital Affiliated to Fujian Medical University, Quanzhou 362000, China.

Wu, Zhiwei. Department of Otolaryngology, Head and Neck Surgery, Fujian Provincial Hospital, Fuzhou 350001, China.

Wang, Xiaoyan. Shengli Clinical Medical College of Fujian Medical University, Fuzhou 350001, China.

Liu, Yunliang. Shengli Clinical Medical College of Fujian Medical University, Fuzhou 350001, China.

Ye, Qing. Shengli Clinical Medical College of Fujian Medical University, Fuzhou 350001, China.

Ye, Qing. Department of Otolaryngology, Head and Neck Surgery, Fujian Provincial Hospital, Fuzhou 350001, China.

MeSH Heading

Humans. Nasopharyngeal Carcinoma. Cisplatin. Multivariate Analysis. Discriminant Analysis. Spectrum Analysis, Raman/mt [Methods]. Nasopharyngeal Neoplasms/di [Diagnosis]. *Nasopharyngeal Neoplasms. Metal Nanoparticles/ch [Chemistry]. *Metal Nanoparticles. Principal Component Analysis.

Keyword Heading

cisplatin nasopharyngeal carcinoma

secreted protein

surface-enhanced Raman scattering (SERS).

Keyword Heading Owner

NOTNLM

Registry Number/Name of Substance

Q20Q21Q62J (Cisplatin).

Year of Publication

2023

Link to the Ovid Full Text or citation:

[Click here for full text options](https://ovidsp.ovid.com/ovidweb.cgi?T=JS&CSC=Y&NEWS=N&PAGE=fulltext&D=medl&AN=36832007)

Link to the External Link Resolver:

[SFX](https://sfx-86scu.hosted.exlibrisgroup.com.cn/86scu?sid=OVID:medline&id=pmid:36832007&id=doi:10.3390%2Fbios13020241&issn=20796374&isbn=&volume=13&issue=2&spage=&pages=&date=2023&title=Biosensors&atitle=High-Precision+Detection+of+Cellular+Drug+Response+Based+on+SERS+Spectrum+and+Multivariate+Statistical+Analysis.&aulast=Wu&pid=<author>Wu+F%3BWu+Z%3BWang+X%3BLiu+Y%3BYe+Q<%2Fauthor><AN>36832007<%2FAN><DT>Journal+Article<%2FDT>)

6.

Rapid adsorption of triclosan and p-chloro-m-xylenol by nitrogen-doped magnetic porous carbon.

Li Q, Huang L, Zhu P, Zhong M, Xu S

Environmental Science & Pollution Research. 30(1):1640-1655, 2023 Jan.

[Journal Article]

UI: 35921007

Contamination of water resources with organic substances like phenolic fungicides is undesirable due to the improvement of living standards, the huge production of chemicals, the heavy consumption of daily chemical products, and the growth of the population. In this study, Co-based zeolitic imidazole framework-67 (ZIF-67(Co)) was synthesized using the "one-pot method," and the best Co-based N-doped magnetic porous carbon (Co-NPC) was prepared by ZIF-67(Co) carbonization in an atmosphere of N2. The materials were tested using an X-ray diffractometer (XRD), scanning electron microscope (SEM), infrared spectroscopy (IR), Raman spectroscopy, X-ray photoelectron spectroscopy (XPS), N2 adsorption-desorption, and magnetization analysis. These characterizations indicated that the Co-NPC was successfully prepared. With the original morphology of ZIF-67(Co) crystals, the Co-NPC also has good porosity, magnetic properties, and a large specific surface area. In water, Co-NPC-800 has a good adsorption capacity for triclosan (TCS) and p-chloro-m-xylenol (PCMX), which are kinds of aromatic fungicides. The adsorption of Co-NPC-800 on both reached equilibrium within 3 min, which is in accordance with the quasi-second-order kinetic model. At 298 K, the maximum adsorption capacity of Co-NPC-800 for TCS and PCMX was 163 and 39 mg.g-1, respectively. The adsorption of TCS and PCMX by Co-NPC-800 is a spontaneous endothermic process with reduced entropy. The combination of Co-NPC-800 and phenols come from multiple actions of electrostatic, pi-pi, and hydrogen bond effects. Moreover, Co-NPC-800 can be regenerated through simple washing and can be reused at least three times by a magnet. The Co-NPC-800 has good porosity, large specific surface area, comparable adsorption capacity, rapid adsorption time, so it could be broadly used in sewage treatments and other environmental fields.

Copyright © 2022. The Author(s), under exclusive licence to Springer-Verlag GmbH Germany, part of Springer Nature.

Version ID

1

Record Owner

From MEDLINE, a database of the U.S. National Library of Medicine.

Status

MEDLINE

Author NameID

Xu, Shuxia; ORCID: <http://orcid.org/0000-0002-4235-7847>

Authors Full Name

Li, Qiuxing, Huang, Li, Zhu, Paijin, Zhong, Min, Xu, Shuxia

Institution

Li, Qiuxing. College of Earth Science, Chengdu University of Technology, Chengdu, 610059, Sichuan, China. Huang, Li. College of Ecology and Environment, Chengdu University of Technology, Chengdu, 610059, Sichuan, China.

Zhu, Paijin. College of Ecology and Environment, Chengdu University of Technology, Chengdu, 610059, Sichuan, China.

Zhong, Min. College of Ecology and Environment, Chengdu University of Technology, Chengdu, 610059, Sichuan, China.

Zhong, Min. State Environmental Protection Key Laboratory of Synergetic Control and Joint Remediation for Soil & Water Pollution, Chengdu University of Technology, Chengdu, 610059, China.

Xu, Shuxia. College of Ecology and Environment, Chengdu University of Technology, Chengdu, 610059, Sichuan, China. xushux@cdut.edu.cn.

Xu, Shuxia. State Environmental Protection Key Laboratory of Synergetic Control and Joint Remediation for Soil & Water Pollution, Chengdu University of Technology, Chengdu, 610059, China. xushux@cdut.edu.cn.

MeSH Heading

Carbon. Adsorption. *Triclosan. Porosity. Nitrogen/ch [Chemistry]. *Fungicides, Industrial. Magnetic Phenomena. Water Pollutants, Chemical/ch [Chemistry]. *Water Pollutants, Chemical. Kinetics.

Keyword Heading

Adsorption Kinetics

Magnetic porous carbon

Nitrogen doping

P-chloro-m-xylenol

Thermodynamics

Triclosan.

Keyword Heading Owner

NOTNLM

Registry Number/Name of Substance

7440-44-0 (Carbon). 0F32U78V2Q (chloroxylenol). 4NM5039Y5X (Triclosan). N762921K75 (Nitrogen). 0 (Fungicides, Industrial). 0 (Water Pollutants, Chemical).

Year of Publication

2023

Link to the Ovid Full Text or citation:

[Click here for full text options](https://ovidsp.ovid.com/ovidweb.cgi?T=JS&CSC=Y&NEWS=N&PAGE=fulltext&D=medl&AN=35921007)

Link to the External Link Resolver:

[SFX](https://sfx-86scu.hosted.exlibrisgroup.com.cn/86scu?sid=OVID:medline&id=pmid:35921007&id=doi:10.1007%2Fs11356-022-22084-y&issn=09441344&isbn=&volume=30&issue=1&spage=1640&pages=1640-1655&date=2023&title=Environmental+Science+%26+Pollution+Research&atitle=Rapid+adsorption+of+triclosan+and+p-chloro-m-xylenol+by+nitrogen-doped+magnetic+porous+carbon.&aulast=Li&pid=<author>Li+Q%3BHuang+L%3BZhu+P%3BZhong+M%3BXu+S<%2Fauthor><AN>35921007<%2FAN><DT>Journal+Article<%2FDT>)

7.

Profiling of Tumor Cell-Delivered Exosome by Surface Enhanced Raman Spectroscopy-Based Biosensor for Evaluation of Nasopharyngeal Cancer Radioresistance.

Wu Q, Ding Q, Lin W, Weng Y, Feng S, Chen R, Chen C, Qiu S, Lin D

Advanced Healthcare Materials. e2202482, 2022 Dec 17.

[Journal Article]

UI: 36528342

Although the advancement of radiotherapy significantly improves the survival of nasopharyngeal cancer (NPC), radioresistance associated with recurrence and poor outcomes still remains a daunting challenge in the clinical scenario. Currently, effective biomarkers and convenient detection methods for predicting radioresistance have not been well established. Here, the surface-enhanced Raman spectroscopy combined with proteomics is used to firstly profile the characteristic spectral patterns of exosomes secreted from self-established NPC radioresistance cells, and reveals specific variations of proteins expression during radioresistance formation, including collagen alpha-2 (I) chain (COL1A2) that is associated with a favorable prognosis in NPC and is negatively associated with DNA repair scores and DNA repair-related genes via bioinformatic analysis. Furthermore, deep learning model-based diagnostic model is generated to accurately identify the exosomes from radioresistance group. This work demonstrates the promising potential of exosomes as a novel biomarker for predicting the radioresistance and develops a rapid and sensitive liquid biopsy method that will provide a personalized and precise strategy for clinical NPC treatment.

Copyright © 2022 Wiley-VCH GmbH.

Version ID

1

Record Owner

From MEDLINE, a database of the U.S. National Library of Medicine.

Status

Publisher

Author NameID

Lin, Duo; ORCID: <https://orcid.org/0000-0001-6959-5995>

Authors Full Name

Wu, Qiong, Ding, Qin, Lin, Wanzun, Weng, Youliang, Feng, Shangyuan, Chen, Rong, Chen, Chuanben, Qiu, Sufang, Lin, Duo

Institution

Wu, Qiong. Key Laboratory of OptoElectronic Science and Technology for Medicine, Ministry of Education, Fujian Provincial Key Laboratory for Photonics Technology, Fujian Normal University, Fuzhou, Fujian, 350001, China. Wu, Qiong. College of Physics and Electronic Information Engineering, Minjiang University, Fuzhou, Fujian, 350001, China.

Ding, Qin. Department of Radiation Oncology, Clinical Oncology School of Fujian Medical University, Fujian Cancer Hospital, Fuzhou, Fujian, 350001, China.

Lin, Wanzun. Department of Radiation Oncology, Shanghai Proton and Heavy Ion Center, Fudan University Cancer Hospital, Shanghai, 201321, China.

Weng, Youliang. Department of Radiation Oncology, Clinical Oncology School of Fujian Medical University, Fujian Cancer Hospital, Fuzhou, Fujian, 350001, China.

Feng, Shangyuan. Key Laboratory of OptoElectronic Science and Technology for Medicine, Ministry of Education, Fujian Provincial Key Laboratory for Photonics Technology, Fujian Normal University, Fuzhou, Fujian, 350001, China.

Chen, Rong. Key Laboratory of OptoElectronic Science and Technology for Medicine, Ministry of Education, Fujian Provincial Key Laboratory for Photonics Technology, Fujian Normal University, Fuzhou, Fujian, 350001, China.

Chen, Chuanben. Department of Radiation Oncology, Clinical Oncology School of Fujian Medical University, Fujian Cancer Hospital, Fuzhou, Fujian, 350001, China.

Qiu, Sufang. Department of Radiation Oncology, Clinical Oncology School of Fujian Medical University, Fujian Cancer Hospital, Fuzhou, Fujian, 350001, China.

Lin, Duo. Key Laboratory of OptoElectronic Science and Technology for Medicine, Ministry of Education, Fujian Provincial Key Laboratory for Photonics Technology, Fujian Normal University, Fuzhou, Fujian, 350001, China.

Keyword Heading

biomarkers exosomes

nasopharyngeal cancer

radiotherapy

surface-enhanced Raman spectroscopy.

Keyword Heading Owner

NOTNLM

Year of Publication

2022

Link to the Ovid Full Text or citation:

[Click here for full text options](https://ovidsp.ovid.com/ovidweb.cgi?T=JS&CSC=Y&NEWS=N&PAGE=fulltext&D=medp&AN=36528342)

Link to the External Link Resolver:

[SFX](https://sfx-86scu.hosted.exlibrisgroup.com.cn/86scu?sid=OVID:medline&id=pmid:36528342&id=doi:10.1002%2Fadhm.202202482&issn=21922640&isbn=&volume=&issue=&spage=e2202482&pages=e2202482&date=2022&title=Advanced+Healthcare+Materials&atitle=Profiling+of+Tumor+Cell-Delivered+Exosome+by+Surface+Enhanced+Raman+Spectroscopy-Based+Biosensor+for+Evaluation+of+Nasopharyngeal+Cancer+Radioresistance.&aulast=Wu&pid=<author>Wu+Q%3BDing+Q%3BLin+W%3BWeng+Y%3BFeng+S%3BChen+R%3BChen+C%3BQiu+S%3BLin+D<%2Fauthor><AN>36528342<%2FAN><DT>Journal+Article<%2FDT>)

8.

Analysis of urine using electronic tongue towards non-invasive cancer diagnosis. [Review]

Zniber M, Vahdatiyekta P, Huynh TP

Biosensors & Bioelectronics. 219:114810, 2022 Oct 15.

[Journal Article. Review]

UI: 36272349

Electronic tongues (e-tongues) have been broadly employed in monitoring the quality of food, beverage, cosmetics, and pharmaceutical products, and in diagnosis of diseases, as the e-tongues can discriminate samples of high complexity, reduce interference of the matrix, offer rapid response. Compared to other analytical approaches using expensive and complex instrumentation as well as required sample preparation, the e-tongue is non-destructive, miniaturizable and on-site method with little or no preparation of samples. Even though e-tongues are successfully commercialized, their application in cancer diagnosis from urine samples is underestimated. In this review, we would like to highlight the various analytical techniques such as Raman spectroscopy, infrared spectroscopy, fluorescence spectroscopy, and electrochemical methods (potentiometry and voltammetry) used as e-tongues for urine analysis towards non-invasive cancer diagnosis. Besides, different machine learning approaches, for instance, supervised and unsupervised learning algorithms are introduced to analyze extracted chemical data. Finally, capabilities of e-tongues in distinguishing between patients diagnosed with cancer and healthy controls are highlighted.

Copyright © 2022 The Authors. Published by Elsevier B.V. All rights reserved.

Version ID

1

Record Owner

From MEDLINE, a database of the U.S. National Library of Medicine.

Status

Publisher

Authors Full Name

Zniber, Mohammed, Vahdatiyekta, Parastoo, Huynh, Tan-Phat

Institution

Zniber, Mohammed. Laboratory of Molecular Science and Engineering, Abo Akademi University, 20500, Turku, Finland. Vahdatiyekta, Parastoo. Laboratory of Molecular Science and Engineering, Abo Akademi University, 20500, Turku, Finland.

Huynh, Tan-Phat. Laboratory of Molecular Science and Engineering, Abo Akademi University, 20500, Turku, Finland. Electronic address: tan.huynh@abo.fi.

Keyword Heading

Cancer diagnosis Electronic tongue

Machine learning

Urine.

Keyword Heading Owner

NOTNLM

Year of Publication

2022

Link to the Ovid Full Text or citation:

[Click here for full text options](https://ovidsp.ovid.com/ovidweb.cgi?T=JS&CSC=Y&NEWS=N&PAGE=fulltext&D=medp&AN=36272349)

Link to the External Link Resolver:

[SFX](https://sfx-86scu.hosted.exlibrisgroup.com.cn/86scu?sid=OVID:medline&id=pmid:36272349&id=doi:10.1016%2Fj.bios.2022.114810&issn=09565663&isbn=&volume=219&issue=&spage=114810&pages=114810&date=2022&title=Biosensors+%26+Bioelectronics&atitle=Analysis+of+urine+using+electronic+tongue+towards+non-invasive+cancer+diagnosis.&aulast=Zniber&pid=<author>Zniber+M%3BVahdatiyekta+P%3BHuynh+TP<%2Fauthor><AN>36272349<%2FAN><DT>Journal+Article<%2FDT>)

9.

Label-Free Optical Spectroscopy for Early Detection of Oral Cancer. [Review]

Maryam S, Nogueira MS, Gautam R, Krishnamoorthy S, Venkata Sekar SK, Kho KW, Lu H, Ni Riordain R, Feeley L, Sheahan P, Burke R, Andersson-Engels S

Diagnostics. 12(12), 2022 Nov 22.

[Journal Article. Review]

UI: 36552903

Oral cancer is the 16th most common cancer worldwide. It commonly arises from painless white or red plaques within the oral cavity. Clinical outcome is highly related to the stage when diagnosed. However, early diagnosis is complex owing to the impracticality of biopsying every potentially premalignant intraoral lesion. Therefore, there is a need to develop a non-invasive cost-effective diagnostic technique to differentiate non-malignant and early-stage malignant lesions. Optical spectroscopy may provide an appropriate solution to facilitate early detection of these lesions. It has many advantages over traditional approaches including cost, speed, objectivity, sensitivity, painlessness, and ease-of use in clinical setting for real-time diagnosis. This review consists of a comprehensive overview of optical spectroscopy for oral cancer diagnosis, epidemiology, and recent improvements in this field for diagnostic purposes. It summarizes major developments in label-free optical spectroscopy, including Raman, fluorescence, and diffuse reflectance spectroscopy during recent years. Among the wide range of optical techniques available, we chose these three for this review because they have the ability to provide biochemical information and show great potential for real-time deep-tissue point-based in vivo analysis. This review also highlights the importance of saliva-based potential biomarkers for non-invasive early-stage diagnosis. It concludes with the discussion on the scope of development and future demands from a clinical point of view.

Version ID

1

Record Owner

From MEDLINE, a database of the U.S. National Library of Medicine.

Status

PubMed-not-MEDLINE

Author NameID

Nogueira, Marcelo Saito; ORCID: <https://orcid.org/0000-0002-5611-9620> Gautam, Rekha; ORCID: <https://orcid.org/0000-0002-1176-8491>

Venkata Sekar, Sanathana Konugolu; ORCID: <https://orcid.org/0000-0003-0912-1282>

Lu, Huihui; ORCID: <https://orcid.org/0000-0002-7038-202X>

Ni Riordain, Richeal; ORCID: <https://orcid.org/0000-0002-1937-8105>

Andersson-Engels, Stefan; ORCID: <https://orcid.org/0000-0001-5640-3122>

Authors Full Name

Maryam, Siddra, Nogueira, Marcelo Saito, Gautam, Rekha, Krishnamoorthy, Shree, Venkata Sekar, Sanathana Konugolu, Kho, Kiang Wei, Lu, Huihui, Ni Riordain, Richeal, Feeley, Linda, Sheahan, Patrick, Burke, Ray, Andersson-Engels, Stefan

Institution

Maryam, Siddra. Tyndall National Institute, University College Cork, T12 R229 Cork, Ireland. Nogueira, Marcelo Saito. Tyndall National Institute, University College Cork, T12 R229 Cork, Ireland.

Gautam, Rekha. Tyndall National Institute, University College Cork, T12 R229 Cork, Ireland.

Krishnamoorthy, Shree. Tyndall National Institute, University College Cork, T12 R229 Cork, Ireland.

Venkata Sekar, Sanathana Konugolu. Tyndall National Institute, University College Cork, T12 R229 Cork, Ireland.

Kho, Kiang Wei. Tyndall National Institute, University College Cork, T12 R229 Cork, Ireland.

Lu, Huihui. Tyndall National Institute, University College Cork, T12 R229 Cork, Ireland.

Ni Riordain, Richeal. ENTO Research Institute, University College Cork, T12 R229 Cork, Ireland.

Ni Riordain, Richeal. Cork University Dental School and Hospital, Wilton, T12 E8YV Cork, Ireland.

Feeley, Linda. ENTO Research Institute, University College Cork, T12 R229 Cork, Ireland.

Feeley, Linda. Cork University Hospital, T12 DC4A Cork, Ireland.

Sheahan, Patrick. ENTO Research Institute, University College Cork, T12 R229 Cork, Ireland.

Sheahan, Patrick. South Infirmary Victoria University Hospital, T12 X23H Cork, Ireland.

Burke, Ray. Tyndall National Institute, University College Cork, T12 R229 Cork, Ireland.

Andersson-Engels, Stefan. Tyndall National Institute, University College Cork, T12 R229 Cork, Ireland.

Keyword Heading

Raman spectroscopy biomarkers

diffuse reflectance spectroscopy

fluorescence spectroscopy

oral cancer

saliva analysis.

Keyword Heading Owner

NOTNLM

Year of Publication

2022

Link to the Ovid Full Text or citation:

[Click here for full text options](https://ovidsp.ovid.com/ovidweb.cgi?T=JS&CSC=Y&NEWS=N&PAGE=fulltext&D=pmnm&AN=36552903)

Link to the External Link Resolver:

[SFX](https://sfx-86scu.hosted.exlibrisgroup.com.cn/86scu?sid=OVID:medline&id=pmid:36552903&id=doi:10.3390%2Fdiagnostics12122896&issn=20754418&isbn=&volume=12&issue=12&spage=&pages=&date=2022&title=Diagnostics&atitle=Label-Free+Optical+Spectroscopy+for+Early+Detection+of+Oral+Cancer.&aulast=Maryam&pid=<author>Maryam+S%3BNogueira+MS%3BGautam+R%3BKrishnamoorthy+S%3BVenkata+Sekar+SK%3BKho+KW%3BLu+H%3BNi+Riordain+R%3BFeeley+L%3BSheahan+P%3BBurke+R%3BAndersson-Engels+S<%2Fauthor><AN>36552903<%2FAN><DT>Journal+Article<%2FDT>)

10.

Plasmonic Nanostructures-Decorated ZIF-8-Derived Nanoporous Carbon for Surface-Enhanced Raman Scattering.

Liao GY, Lien MC, Tadepalli S, Liu KK

ACS Omega. 7(41):36427-36433, 2022 Oct 18.

[Journal Article]

UI: 36278097

Surface-enhanced Raman scattering (SERS) is considered to be a highly sensitive platform for chemical and biological sensing. Recently, owing to their high porosity and large surface area, metal-organic frameworks (MOFs) have attracted considerable attention in sensing applications. Porous carbon nanostructures are promising SERS substrates due to their strong broadband charge-transfer resonance and reproducible fabrication. Furthermore, an extraordinarily large enhancement of the electromagnetic field enables plasmonic nanomaterials to be ideal SERS substrates. Here, we demonstrate the porous Au@Ag nanostructure-decorated MOF-derived nanoporous carbon (NPC) for highly efficient SERS sensing. Specifically, this plasmonic nanomaterial-NPC composite offers high Raman signal enhancement with the ability to detect the model Raman reporter 2-naphthalenethiol (2-NT) at picomolar concentration levels.

Copyright © 2022 The Authors. Published by American Chemical Society.

Version ID

1

Record Owner

From MEDLINE, a database of the U.S. National Library of Medicine.

Status

PubMed-not-MEDLINE

Author NameID

Tadepalli, Sirimuvva; ORCID: <https://orcid.org/0000-0001-9658-9988> Liu, Keng-Ku; ORCID: <https://orcid.org/0000-0002-1427-3494>

Authors Full Name

Liao, Guan-Ye, Lien, Mei-Chin, Tadepalli, Sirimuvva, Liu, Keng-Ku

Institution

Liao, Guan-Ye. Department of Biomedical Engineering and Environmental Sciences, National Tsing Hua University, Hsinchu 300044, Taiwan. Lien, Mei-Chin. Department of Biomedical Engineering and Environmental Sciences, National Tsing Hua University, Hsinchu 300044, Taiwan.

Tadepalli, Sirimuvva. Microbiology & Immunology Department and Immunology Program, Stanford University School of Medicine, Stanford, California 94305, United States.

Liu, Keng-Ku. Department of Biomedical Engineering and Environmental Sciences, National Tsing Hua University, Hsinchu 300044, Taiwan.

Year of Publication

2022

Link to the Ovid Full Text or citation:

[Click here for full text options](https://ovidsp.ovid.com/ovidweb.cgi?T=JS&CSC=Y&NEWS=N&PAGE=fulltext&D=pmnm7&AN=36278097)

Link to the External Link Resolver:

[SFX](https://sfx-86scu.hosted.exlibrisgroup.com.cn/86scu?sid=OVID:medline&id=pmid:36278097&id=doi:10.1021%2Facsomega.2c04183&issn=24701343&isbn=&volume=7&issue=41&spage=36427&pages=36427-36433&date=2022&title=ACS+Omega&atitle=Plasmonic+Nanostructures-Decorated+ZIF-8-Derived+Nanoporous+Carbon+for+Surface-Enhanced+Raman+Scattering.&aulast=Liao&pid=<author>Liao+GY%3BLien+MC%3BTadepalli+S%3BLiu+KK<%2Fauthor><AN>36278097<%2FAN><DT>Journal+Article<%2FDT>)

11.

Corrigendum: Diagnostic accuracy of Raman spectroscopy in oral squamous cell carcinoma.

Han R, Lin N, Huang J, Ma X

Frontiers in Oncology. 12:1030058, 2022.

[Published Erratum]

UI: 36212486

[This corrects the article DOI: 10.3389/fonc.2022.925032.].

Copyright © 2022 Han, Lin, Huang and Ma.

Version ID

1

Record Owner

From MEDLINE, a database of the U.S. National Library of Medicine.

Status

PubMed-not-MEDLINE

Authors Full Name

Han, Ruiying, Lin, Nan, Huang, Juan, Ma, Xuelei

Institution

Han, Ruiying. Department of Biotherapy, West China Hospital and State Key Laboratory of Biotherapy, Sichuan University, Chengdu, China. Han, Ruiying. State Key Laboratory of Oral Diseases, National Clinical Research Center for Oral Diseases, Sichuan University, Chengdu, China.

Lin, Nan. Department of Biotherapy, West China Hospital and State Key Laboratory of Biotherapy, Sichuan University, Chengdu, China.

Huang, Juan. Department of Hematology, Sichuan Academy of Medical Sciences and Sichuan Provincial People's Hospital, University of Electronic Science and Technology of China, Chengdu, China.

Ma, Xuelei. Department of Biotherapy, West China Hospital and State Key Laboratory of Biotherapy, Sichuan University, Chengdu, China.

Comments

Erratum for (EFR)

Keyword Heading

OSCC artificial intelligence

diagnosis

raman spectroscopy

systematic review.

Keyword Heading Owner

NOTNLM

Year of Publication

2022

Link to the Ovid Full Text or citation:

[Click here for full text options](https://ovidsp.ovid.com/ovidweb.cgi?T=JS&CSC=Y&NEWS=N&PAGE=fulltext&D=pmnm7&AN=36212486)

Link to the External Link Resolver:

[SFX](https://sfx-86scu.hosted.exlibrisgroup.com.cn/86scu?sid=OVID:medline&id=pmid:36212486&id=doi:10.3389%2Ffonc.2022.1030058&issn=2234943X&isbn=&volume=12&issue=&spage=1030058&pages=1030058&date=2022&title=Frontiers+in+Oncology&atitle=Corrigendum%3A+Diagnostic+accuracy+of+Raman+spectroscopy+in+oral+squamous+cell+carcinoma.&aulast=Han&pid=<author>Han+R%3BLin+N%3BHuang+J%3BMa+X<%2Fauthor><AN>36212486<%2FAN><DT>Published+Erratum<%2FDT>)

12.

Corrigendum to <Diverse spectral band-based deep residual network for tongue squamous cell carcinoma classification using fiber optic Raman spectroscopy> <[Photodiagnosis and Photodynamic Therapy, Volume 32, December 2020, 102048]>.

Ding J, Yu M, Zhu L, Zhang T, Xia J, Sun G

Photodiagnosis & Photodynamic Therapy. 39:102981, 2022 Sep.

[Published Erratum]

UI: 36068732

Version ID

1

Record Owner

From MEDLINE, a database of the U.S. National Library of Medicine.

Status

PubMed-not-MEDLINE

Authors Full Name

Ding, Jingya, Yu, Mingxin, Zhu, Lianqing, Zhang, Tao, Xia, Jiabin, Sun, Guangkai

Institution

Ding, Jingya. Key Laboratory of the Ministry of Education for Optoelectronic Measurement Technology and Instrument, Beijing Information Science and Technology University, Beijing, 100192, China. Yu, Mingxin. Key Laboratory of the Ministry of Education for Optoelectronic Measurement Technology and Instrument, Beijing Information Science and Technology University, Beijing, 100192, China. Electronic address: yumingxin@bistu.edu.cn.

Zhu, Lianqing. Key Laboratory of the Ministry of Education for Optoelectronic Measurement Technology and Instrument, Beijing Information Science and Technology University, Beijing, 100192, China; School of Instrument Science and Opto-electronics Engineering, Hefei University of Technology, Hefei, 230009, China. Electronic address: lianqingbistu@sina.com.

Zhang, Tao. Department of stomatology, Peking Union Medical College Hospital, No. 1 Shuaifuyuan Wangfujing, Dongcheng District, Beijing 100730, China.

Xia, Jiabin. Key Laboratory of the Ministry of Education for Optoelectronic Measurement Technology and Instrument, Beijing Information Science and Technology University, Beijing, 100192, China; School of Instrument Science and Opto-electronics Engineering, Hefei University of Technology, Hefei, 230009, China.

Sun, Guangkai. Key Laboratory of the Ministry of Education for Optoelectronic Measurement Technology and Instrument, Beijing Information Science and Technology University, Beijing, 100192, China.

Comments

Erratum for (EFR)

Year of Publication

2022

Link to the Ovid Full Text or citation:

[Click here for full text options](https://ovidsp.ovid.com/ovidweb.cgi?T=JS&CSC=Y&NEWS=N&PAGE=fulltext&D=pmnm7&AN=36068732)

Link to the External Link Resolver:

[SFX](https://sfx-86scu.hosted.exlibrisgroup.com.cn/86scu?sid=OVID:medline&id=pmid:36068732&id=doi:10.1016%2Fj.pdpdt.2022.102981&issn=15721000&isbn=&volume=39&issue=&spage=102981&pages=102981&date=2022&title=Photodiagnosis+%26+Photodynamic+Therapy&atitle=Corrigendum+to+<Diverse+spectral+band-based+deep+residual+network+for+tongue+squamous+cell+carcinoma+classification+using+fiber+optic+Raman+spectroscopy>+<%5BPhotodiagnosis+and+Photodynamic+Therapy%2C+Volume+32%2C+December+2020%2C+102048%5D>.&aulast=Ding&pid=<author>Ding+J%3BYu+M%3BZhu+L%3BZhang+T%3BXia+J%3BSun+G<%2Fauthor><AN>36068732<%2FAN><DT>Published+Erratum<%2FDT>)

13.

Simultaneous detection of circulating tumor DNAs using a SERS-based lateral flow assay biosensor for point-of-care diagnostics of head and neck cancer.

Li G, Ge S, Niu P, Zhang J, Mao Y, Wang Y, Sun A

Biomedical Optics Express. 13(8):4102-4117, 2022 Aug 01.

[Journal Article]

UI: 36032568

Circulating tumor DNA (ctDNA) has recently emerged as an ideal target for biomarker analytes. Thus, the development of rapid and ultrasensitive ctDNA detection methods is essential. In this study, a high-throughput surface-enhanced Raman scattering (SERS)-based lateral flow assay (LFA) strip is proposed. The aim of this method is to achieve accurate quantification of TP53 and PIK3CA E545K, two types of ctDNAs associated with head and neck squamous cell carcinoma (HNSCC), particularly for point-of-care testing (POCT). Raman reporters and hairpin DNAs are used to functionalize the Pd-Au core-shell nanorods (Pd-AuNRs), which serve as the SERS probes. During the detection process, the existence of targets could open the hairpins on the surface of Pd-AuNRs and trigger the first step of catalytic hairpin assembly (CHA) amplification. The next stage of CHA amplification is initiated by the hairpins prefixed on the test lines, generating numerous "hot spots" to enhance the SERS signal significantly. By the combination of high-performing SERS probes and a target-specific signal amplification strategy, TP53 and PIK3CA E545K are directly quantified in the range of 100 aM-1 nM, with the respective limits of detection (LOD) calculated as 33.1 aM and 20.0 aM in the PBS buffer and 37.8 aM and 23.1 aM in human serum, which are significantly lower than for traditional colorimetric LFA methods. The entire detection process is completed within 45 min, and the multichannel design realizes the parallel detection of multiple groups of samples. Moreover, the analytical performance is validated, including reproducibility, uniformity, and specificity. Finally, the SERS-LFA biosensor is employed to analyze the expression levels of TP53 and PIK3CA E545K in the serum of patients with HNSCC. The results are verified as consistent with those of qRT-PCR. Thus, the SERS-LFA biosensor can be considered as a noninvasive liquid biopsy assay for clinical cancer diagnosis.

Copyright © 2022 Optica Publishing Group under the terms of the Optica Open Access Publishing Agreement.

Version ID

1

Record Owner

From MEDLINE, a database of the U.S. National Library of Medicine.

Status

PubMed-not-MEDLINE

Authors Full Name

Li, Guang, Ge, Shengjie, Niu, Ping, Zhang, Jianyou, Mao, Yu, Wang, Youwei, Sun, Aidong

Institution

Li, Guang. Department of Otorhinolaryngology-Head and Neck Surgery, The Affiliated Hospital of Yangzhou University, Yangzhou University, Yangzhou, 225001, P. R., China. Ge, Shengjie. Department of Otorhinolaryngology-Head and Neck Surgery, The Affiliated Hospital of Yangzhou University, Yangzhou University, Yangzhou, 225001, P. R., China.

Ge, Shengjie. Institute of Translational Medicine, Medical College, Yangzhou University, Yangzhou, 225001, P. R., China.

Niu, Ping. Departments of Otolaryngology, Qingzhou People's Hospital, Qingzhou, 262500, P. R., China.

Zhang, Jianyou. Department of Anesthesiology, The Affiliated Hospital of Yangzhou University, Yangzhou University, Yangzhou, 225001, P. R., China.

Mao, Yu. Institute of Translational Medicine, Medical College, Yangzhou University, Yangzhou, 225001, P. R., China.

Wang, Youwei. Department of Neurosurgery, The Affiliated Hospital of Yangzhou University, Yangzhou University, Yangzhou, 225001, P. R., China.

Wang, Youwei. wangyouwei19@126.com.

Sun, Aidong. Department of Otorhinolaryngology-Head and Neck Surgery, The Affiliated Hospital of Yangzhou University, Yangzhou University, Yangzhou, 225001, P. R., China.

Sun, Aidong. entsunaidong@163.com.

Year of Publication

2022

Link to the Ovid Full Text or citation:

[Click here for full text options](https://ovidsp.ovid.com/ovidweb.cgi?T=JS&CSC=Y&NEWS=N&PAGE=fulltext&D=pmnm7&AN=36032568)

Link to the External Link Resolver:

[SFX](https://sfx-86scu.hosted.exlibrisgroup.com.cn/86scu?sid=OVID:medline&id=pmid:36032568&id=doi:10.1364%2FBOE.463612&issn=21567085&isbn=&volume=13&issue=8&spage=4102&pages=4102-4117&date=2022&title=Biomedical+Optics+Express&atitle=Simultaneous+detection+of+circulating+tumor+DNAs+using+a+SERS-based+lateral+flow+assay+biosensor+for+point-of-care+diagnostics+of+head+and+neck+cancer.&aulast=Li&pid=<author>Li+G%3BGe+S%3BNiu+P%3BZhang+J%3BMao+Y%3BWang+Y%3BSun+A<%2Fauthor><AN>36032568<%2FAN><DT>Journal+Article<%2FDT>)

14.

Diagnostic accuracy of Raman spectroscopy in oral squamous cell carcinoma.

Han R, Lin N, Huang J, Ma X

Frontiers in Oncology. 12:925032, 2022.

[Systematic Review]

UI: 35992884

Background: Raman spectroscopy (RS) has shown great potential in the diagnosis of oral squamous cell carcinoma (OSCC). Although many single-central original studies have been carried out, it is difficult to use RS in real clinical settings based on the current limited evidence. Herein, we conducted this meta-analysis of diagnostic studies to evaluate the overall performance of RS in OSCC diagnosis.

Methods: We systematically searched databases including Medline, Embase, and Web of Science for studies up to March 2022 with no start date limited. Data of true positives, true negatives, false positives, and false negatives were extracted from the included studies to calculate the pooled sensitivity, specificity, accuracy, positive and negative likelihood ratios (LRs), and diagnostic odds ratio (DOR) with 95% confidence intervals, then we plotted the summary receiver operating characteristic (SROC) curve and the area under the curve (AUC) to evaluate the overall performance of RS. Quality assessments and publication bias were evaluated by Quality Assessment of Diagnostic Accuracy Studies 2 (QUADAS-2) checklist in Review Manager 5.3. The statistical parameters were calculated with StataSE version 12 and MetaDiSc 1.4.

Results: In total, 13 studies were included in our meta-analysis. The pooled diagnostic sensitivity and specificity of RS in OSCC were 0.89 (95% CI, 0.85-0.92) and 0.84 (95% CI, 0.78-0.89). The AUC of SROC curve was 0.93 (95% CI, 0.91-0.95).

Conclusions: RS is a non-invasive diagnostic technology with high specificity and sensitivity for detecting OSCC and has the potential to be applied clinically.

Copyright © 2022 Han, Lin, Huang and Ma.

Version ID

1

Record Owner

From MEDLINE, a database of the U.S. National Library of Medicine.

Status

PubMed-not-MEDLINE

Authors Full Name

Han, Ruiying, Lin, Nan, Huang, Juan, Ma, Xuelei

Institution

Han, Ruiying. State Key Laboratory of Oral Diseases, National Clinical Research Center for Oral Diseases, Sichuan University, Chengdu, China. Lin, Nan. Department of Biotherapy, West China Hospital and State Key Laboratory of Biotherapy, Sichuan University, Chengdu, China.

Huang, Juan. Department of Hematology, Sichuan Academy of Medical Sciences and Sichuan Provincial People's Hospital, University of Electronic Science and Technology of China, Chengdu, China.

Ma, Xuelei. Department of Biotherapy, West China Hospital and State Key Laboratory of Biotherapy, Sichuan University, Chengdu, China.

Comments

Erratum in (EIN)

Keyword Heading

OSCC artificial intelligence

diagnosis

raman spectroscopy

systematic review.

Keyword Heading Owner

NOTNLM

Year of Publication

2022

Link to the Ovid Full Text or citation:

[Click here for full text options](https://ovidsp.ovid.com/ovidweb.cgi?T=JS&CSC=Y&NEWS=N&PAGE=fulltext&D=pmnm7&AN=35992884)

Link to the External Link Resolver:

[SFX](https://sfx-86scu.hosted.exlibrisgroup.com.cn/86scu?sid=OVID:medline&id=pmid:35992884&id=doi:10.3389%2Ffonc.2022.925032&issn=2234943X&isbn=&volume=12&issue=&spage=925032&pages=925032&date=2022&title=Frontiers+in+Oncology&atitle=Diagnostic+accuracy+of+Raman+spectroscopy+in+oral+squamous+cell+carcinoma.&aulast=Han&pid=<author>Han+R%3BLin+N%3BHuang+J%3BMa+X<%2Fauthor><AN>35992884<%2FAN><DT>Systematic+Review<%2FDT>)

15.

Highly Efficient Blood Protein Analysis Using Membrane Purification Technique and Super-Hydrophobic SERS Platform for Precise Screening and Staging of Nasopharyngeal Carcinoma.

Lin J, Weng Y, Lin X, Qiu S, Huang Z, Pan C, Li Y, Kong KV, Zhang X, Feng S

Nanomaterials. 12(15), 2022 Aug 08.

[Journal Article]

UI: 35957154

Early screening and precise staging are crucial for reducing mortality in patients with nasopharyngeal carcinoma (NPC). This study aimed to assess the performance of blood protein surface-enhanced Raman scattering (SERS) spectroscopy, combined with deep learning, for the precise detection of NPC. A highly efficient protein SERS analysis, based on a membrane purification technique and super-hydrophobic platform, was developed and applied to blood samples from 1164 subjects, including 225 healthy volunteers, 120 stage I, 249 stage II, 291 stage III, and 279 stage IV NPC patients. The proteins were rapidly purified from only 10 microL of blood plasma using the membrane purification technique. Then, the super-hydrophobic platform was prepared to pre-concentrate tiny amounts of proteins by forming a uniform deposition to provide repeatable SERS spectra. A total of 1164 high-quality protein SERS spectra were rapidly collected using a self-developed macro-Raman system. A convolutional neural network-based deep-learning algorithm was used to classify the spectra. An accuracy of 100% was achieved for distinguishing between the healthy and NPC groups, and accuracies of 96%, 96%, 100%, and 100% were found for the differential classification among the four NPC stages. This study demonstrated the great promise of SERS- and deep-learning-based blood protein testing for rapid, non-invasive, and precise screening and staging of NPC.

Version ID

1

Record Owner

From MEDLINE, a database of the U.S. National Library of Medicine.

Status

PubMed-not-MEDLINE

Author NameID

Qiu, Sufang; ORCID: <https://orcid.org/0000-0001-7660-6955> Huang, Zufang; ORCID: <https://orcid.org/0000-0001-8290-5281>

Authors Full Name

Lin, Jinyong, Weng, Youliang, Lin, Xueliang, Qiu, Sufang, Huang, Zufang, Pan, Changbin, Li, Ying, Kong, Kien Voon, Zhang, Xianzeng, Feng, Shangyuan

Institution

Lin, Jinyong. Key Laboratory of OptoElectronic Science and Technology for Medicine, Ministry of Education, Fujian Provincial Key Laboratory for Photonics Technology, Fujian Normal University, Fuzhou 350007, China. Lin, Jinyong. Clinical Oncology School of Fujian Medical University, Fujian Cancer Hospital, Fuzhou 350014, China.

Weng, Youliang. Clinical Oncology School of Fujian Medical University, Fujian Cancer Hospital, Fuzhou 350014, China.

Lin, Xueliang. Fujian Provincial Key Laboratory for Advanced Micro-Nano Photonics Technology and Devices, Research Center for Photonics Technology, Quanzhou Normal University, Quanzhou 362046, China.

Qiu, Sufang. Clinical Oncology School of Fujian Medical University, Fujian Cancer Hospital, Fuzhou 350014, China.

Huang, Zufang. Key Laboratory of OptoElectronic Science and Technology for Medicine, Ministry of Education, Fujian Provincial Key Laboratory for Photonics Technology, Fujian Normal University, Fuzhou 350007, China.

Pan, Changbin. Key Laboratory of OptoElectronic Science and Technology for Medicine, Ministry of Education, Fujian Provincial Key Laboratory for Photonics Technology, Fujian Normal University, Fuzhou 350007, China.

Li, Ying. Clinical Oncology School of Fujian Medical University, Fujian Cancer Hospital, Fuzhou 350014, China.

Kong, Kien Voon. Department of Chemistry, National Taiwan University, Taipei 10617, Taiwan.

Zhang, Xianzeng. Key Laboratory of OptoElectronic Science and Technology for Medicine, Ministry of Education, Fujian Provincial Key Laboratory for Photonics Technology, Fujian Normal University, Fuzhou 350007, China.

Feng, Shangyuan. Key Laboratory of OptoElectronic Science and Technology for Medicine, Ministry of Education, Fujian Provincial Key Laboratory for Photonics Technology, Fujian Normal University, Fuzhou 350007, China.

Keyword Heading

deep learning nasopharyngeal carcinoma

protein SERS

super-hydrophobic platform.

Keyword Heading Owner

NOTNLM

Year of Publication

2022

Link to the Ovid Full Text or citation:

[Click here for full text options](https://ovidsp.ovid.com/ovidweb.cgi?T=JS&CSC=Y&NEWS=N&PAGE=fulltext&D=pmnm7&AN=35957154)

Link to the External Link Resolver:

[SFX](https://sfx-86scu.hosted.exlibrisgroup.com.cn/86scu?sid=OVID:medline&id=pmid:35957154&id=doi:10.3390%2Fnano12152724&issn=20794991&isbn=&volume=12&issue=15&spage=&pages=&date=2022&title=Nanomaterials&atitle=Highly+Efficient+Blood+Protein+Analysis+Using+Membrane+Purification+Technique+and+Super-Hydrophobic+SERS+Platform+for+Precise+Screening+and+Staging+of+Nasopharyngeal+Carcinoma.&aulast=Lin&pid=<author>Lin+J%3BWeng+Y%3BLin+X%3BQiu+S%3BHuang+Z%3BPan+C%3BLi+Y%3BKong+KV%3BZhang+X%3BFeng+S<%2Fauthor><AN>35957154<%2FAN><DT>Journal+Article<%2FDT>)

16.

Gold nanomaterials for oral cancer diagnosis and therapy: Advances, challenges, and prospects.

Zhang Q, Hou D, Wen X, Xin M, Li Z, Wu L, Pathak JL

Materials Today. Bio.. 15:100333, 2022 Jun.

[Journal Article]

UI: 35774196

Early diagnosis and treatment of oral cancer are vital for patient survival. Since the oral cavity accommodates the second largest and most diverse microbiome community after the gut, the diagnostic and therapeutic approaches with low invasiveness and minimal damage to surrounding tissues are keys to preventing clinical intervention-related infections. Gold nanoparticles (AuNPs) are widely used in the research of cancer diagnosis and therapy due to their excellent properties such as surface-enhanced Raman spectroscopy, surface plasma resonance, controlled synthesis, the plasticity of surface morphology, biological safety, and stability. AuNPs had been used in oral cancer detection reagents, tumor-targeted therapy, photothermal therapy, photodynamic therapy, and other combination therapies for oral cancer. AuNPs-based noninvasive diagnosis and precise treatments further reduce the clinical intervention-related infections. This review is focused on the recent advances in research and application of AuNPs for early screening, diagnostic typing, drug delivery, photothermal therapy, radiotherapy sensitivity treatment, and combination therapy of oral cancer. Distinctive reports from the literature are summarized to highlight the latest advances in the development and application of AuNPs in oral cancer diagnosis and therapy. Finally, this review points out the challenges and prospects of possible applications of AuNPs in oral cancer diagnosis and therapy.

Copyright © 2022 The Authors.

Version ID

1

Record Owner

From MEDLINE, a database of the U.S. National Library of Medicine.

Status

PubMed-not-MEDLINE

Authors Full Name

Zhang, Qing, Hou, Dan, Wen, Xueying, Xin, Mengyu, Li, Ziling, Wu, Lihong, Pathak, Janak L

Institution

Zhang, Qing. Affiliated Stomatology Hospital of Guangzhou Medical University, Guangdong Engineering Research Center of Oral Restoration and Reconstruction, Guangzhou Key Laboratory of Basic and Applied Research of Oral Regenerative Medicine, Guangzhou, 510182, China. Zhang, Qing. Laboratory for Myology, Department of Human Movement Sciences, Faculty of Behavioural and Movement Sciences, Vrije Universiteit Amsterdam, Amsterdam Movement Sciences, 1081 BT Amsterdam, the Netherlands.

Hou, Dan. Affiliated Stomatology Hospital of Guangzhou Medical University, Guangdong Engineering Research Center of Oral Restoration and Reconstruction, Guangzhou Key Laboratory of Basic and Applied Research of Oral Regenerative Medicine, Guangzhou, 510182, China.

Wen, Xueying. Affiliated Stomatology Hospital of Guangzhou Medical University, Guangdong Engineering Research Center of Oral Restoration and Reconstruction, Guangzhou Key Laboratory of Basic and Applied Research of Oral Regenerative Medicine, Guangzhou, 510182, China.

Xin, Mengyu. Affiliated Stomatology Hospital of Guangzhou Medical University, Guangdong Engineering Research Center of Oral Restoration and Reconstruction, Guangzhou Key Laboratory of Basic and Applied Research of Oral Regenerative Medicine, Guangzhou, 510182, China.

Li, Ziling. Affiliated Stomatology Hospital of Guangzhou Medical University, Guangdong Engineering Research Center of Oral Restoration and Reconstruction, Guangzhou Key Laboratory of Basic and Applied Research of Oral Regenerative Medicine, Guangzhou, 510182, China.

Wu, Lihong. Affiliated Stomatology Hospital of Guangzhou Medical University, Guangdong Engineering Research Center of Oral Restoration and Reconstruction, Guangzhou Key Laboratory of Basic and Applied Research of Oral Regenerative Medicine, Guangzhou, 510182, China.

Pathak, Janak L. Affiliated Stomatology Hospital of Guangzhou Medical University, Guangdong Engineering Research Center of Oral Restoration and Reconstruction, Guangzhou Key Laboratory of Basic and Applied Research of Oral Regenerative Medicine, Guangzhou, 510182, China.

Keyword Heading

Cancer therapy Diagnosis

Gold nanomaterials

Oral cancer

Theranostics.

Keyword Heading Owner

NOTNLM

Year of Publication

2022

Link to the Ovid Full Text or citation:

[Click here for full text options](https://ovidsp.ovid.com/ovidweb.cgi?T=JS&CSC=Y&NEWS=N&PAGE=fulltext&D=pmnm7&AN=35774196)

Link to the External Link Resolver:

[SFX](https://sfx-86scu.hosted.exlibrisgroup.com.cn/86scu?sid=OVID:medline&id=pmid:35774196&id=doi:10.1016%2Fj.mtbio.2022.100333&issn=25900064&isbn=&volume=15&issue=&spage=100333&pages=100333&date=2022&title=Materials+Today.+Bio.&atitle=Gold+nanomaterials+for+oral+cancer+diagnosis+and+therapy%3A+Advances%2C+challenges%2C+and+prospects.&aulast=Zhang&pid=<author>Zhang+Q%3BHou+D%3BWen+X%3BXin+M%3BLi+Z%3BWu+L%3BPathak+JL<%2Fauthor><AN>35774196<%2FAN><DT>Journal+Article<%2FDT>)

17.

Fused Raman spectroscopic analysis of blood and saliva delivers high accuracy for head and neck cancer diagnostics.

Koster HJ, Guillen-Perez A, Gomez-Diaz JS, Navas-Moreno M, Birkeland AC, Carney RP

Scientific Reports. 12(1):18464, 2022 11 02.

[Journal Article. Research Support, Non-U.S. Gov't. Research Support, N.I.H., Extramural]

UI: 36323705

As a rapid, label-free, non-destructive analytical measurement requiring little to no sample preparation, Raman spectroscopy shows great promise for liquid biopsy cancer detection and diagnosis. We carried out Raman analysis and mass spectrometry of plasma and saliva from more than 50 subjects in a cohort of head and neck cancer patients and benign controls (e.g., patients with benign oral masses). Unsupervised data models were built to assess diagnostic performance. Raman spectra collected from either biofluid provided moderate performance to discriminate cancer samples. However, by fusing together the Raman spectra of plasma and saliva for each patient, subsequent analytical models delivered an impressive sensitivity, specificity, and accuracy of 96.3%, 85.7%, and 91.7%, respectively. We further confirmed that the metabolites driving the differences in Raman spectra for our models are among the same ones that drive mass spectrometry models, unifying the two techniques and validating the underlying ability of Raman to assess metabolite composition. This study bolsters the relevance of Raman to provide additive value by probing the unique chemical compositions across biofluid sources. Ultimately, we show that a simple data augmentation routine of fusing plasma and saliva spectra provided significantly higher clinical value than either biofluid alone, pushing forward the potential of clinical translation of Raman spectroscopy for liquid biopsy cancer diagnostics.

Copyright © 2022. The Author(s).

Version ID

1

Record Owner

From MEDLINE, a database of the U.S. National Library of Medicine.

Status

MEDLINE

Authors Full Name

Koster, Hanna J, Guillen-Perez, Antonio, Gomez-Diaz, Juan Sebastian, Navas-Moreno, Maria, Birkeland, Andrew C, Carney, Randy P

Institution

Koster, Hanna J. Biomedical Engineering, University of California, Davis, CA, USA. Guillen-Perez, Antonio. Electrical and Computer Engineering, University of California, Davis, CA, USA.

Gomez-Diaz, Juan Sebastian. Electrical and Computer Engineering, University of California, Davis, CA, USA.

Navas-Moreno, Maria. illumifyDx, Inc., Broomfield, CO, USA.

Birkeland, Andrew C. Department of Otolaryngology, University of California, CA, Davis, USA.

Carney, Randy P. Biomedical Engineering, University of California, Davis, CA, USA. rcarney@ucdavis.com.

MeSH Heading

Humans. Spectrum Analysis, Raman/mt [Methods]. *Spectrum Analysis, Raman. Saliva. Head and Neck Neoplasms/di [Diagnosis]. *Head and Neck Neoplasms. Specimen Handling.

Year of Publication

2022

Link to the Ovid Full Text or citation:

[Click here for full text options](https://ovidsp.ovid.com/ovidweb.cgi?T=JS&CSC=Y&NEWS=N&PAGE=fulltext&D=med22&AN=36323705)

Link to the External Link Resolver:

[SFX](https://sfx-86scu.hosted.exlibrisgroup.com.cn/86scu?sid=OVID:medline&id=pmid:36323705&id=doi:10.1038%2Fs41598-022-22197-x&issn=20452322&isbn=&volume=12&issue=1&spage=18464&pages=18464&date=2022&title=Scientific+Reports&atitle=Fused+Raman+spectroscopic+analysis+of+blood+and+saliva+delivers+high+accuracy+for+head+and+neck+cancer+diagnostics.&aulast=Koster&pid=<author>Koster+HJ%3BGuillen-Perez+A%3BGomez-Diaz+JS%3BNavas-Moreno+M%3BBirkeland+AC%3BCarney+RP<%2Fauthor><AN>36323705<%2FAN><DT>Journal+Article<%2FDT>)

18.

Gefitinib-resveratrol Cocrystal with Optimized Performance in Dissolution and Stability.

Zhai L, Zhang Z, Guo L, Dong H, Yu J, Zhang G

Journal of Pharmaceutical Sciences. 111(12):3224-3231, 2022 12.

[Journal Article. Research Support, Non-U.S. Gov't]

UI: 36202251

Gefitinib (GEF) is an anti-tumor oral solid formulation with a superior advantage for lung tumors. However, it has poor aqueous solubility which limits its utility in vivo. Herein, a novel cocrystal (GEF-RES) assembled by GEF and RES (Resveratrol) has been successfully prepared and comprehensively characterized by differential scanning calorimetry, thermogravimetric analysis, Raman spectroscopy and powder X-ray diffraction. A single-crystal structure of the GEF-RES cocrystal was solved and illustrated in detail. In aqueous hydrochloric acid, the GEF-RES cocrystal showed that the maximum concentration of GEF was slightly higher than that of raw GEF. Furthermore, the thermal and physical stability of the GEF-RES cocrystal were also evaluated in this paper. The enhanced solubility and excellent solid-state stability results may provide new potential to the application of key GEF in clinical.

Copyright © 2022. Published by Elsevier Inc.

Version ID

1

Record Owner

From MEDLINE, a database of the U.S. National Library of Medicine.

Status

MEDLINE

Authors Full Name

Zhai, Lihai, Zhang, Zhaohua, Guo, Lihong, Dong, Huaimin, Yu, Junhou, Zhang, Guimin

Institution

Zhai, Lihai. Lunan Pharmaceutical Group Co., Ltd, Linyi 273400, PR China; National Engineering and Technology Research Centre of Chiral Pharmaceutical, Linyi, 273400, PR China. Zhang, Zhaohua. Lunan Pharmaceutical Group Co., Ltd, Linyi 273400, PR China; National Engineering and Technology Research Centre of Chiral Pharmaceutical, Linyi, 273400, PR China.

Guo, Lihong. Lunan Pharmaceutical Group Co., Ltd, Linyi 273400, PR China; National Engineering and Technology Research Centre of Chiral Pharmaceutical, Linyi, 273400, PR China.

Dong, Huaimin. Lunan Pharmaceutical Group Co., Ltd, Linyi 273400, PR China; National Engineering and Technology Research Centre of Chiral Pharmaceutical, Linyi, 273400, PR China.

Yu, Junhou. Lunan Pharmaceutical Group Co., Ltd, Linyi 273400, PR China.

Zhang, Guimin. Lunan Pharmaceutical Group Co., Ltd, Linyi 273400, PR China; National Engineering and Technology Research Centre of Chiral Pharmaceutical, Linyi, 273400, PR China. Electronic address: ln_zhangguimin@163.com.

MeSH Heading

*Solubility. Resveratrol. Gefitinib. Crystallization/mt [Methods]. Calorimetry, Differential Scanning. X-Ray Diffraction. Powder Diffraction.

Keyword Heading

Anti-tumor Cocrystal

Gefitinib

Resveratrol

Solid-state stability

Solubility.

Keyword Heading Owner

NOTNLM

Registry Number/Name of Substance

Q369O8926L (Resveratrol). S65743JHBS (Gefitinib).

Year of Publication

2022

Link to the Ovid Full Text or citation:

[Click here for full text options](https://ovidsp.ovid.com/ovidweb.cgi?T=JS&CSC=Y&NEWS=N&PAGE=fulltext&D=med22&AN=36202251)

Link to the External Link Resolver:

[SFX](https://sfx-86scu.hosted.exlibrisgroup.com.cn/86scu?sid=OVID:medline&id=pmid:36202251&id=doi:10.1016%2Fj.xphs.2022.09.031&issn=00223549&isbn=&volume=111&issue=12&spage=3224&pages=3224-3231&date=2022&title=Journal+of+Pharmaceutical+Sciences&atitle=Gefitinib-resveratrol+Cocrystal+with+Optimized+Performance+in+Dissolution+and+Stability.&aulast=Zhai&pid=<author>Zhai+L%3BZhang+Z%3BGuo+L%3BDong+H%3BYu+J%3BZhang+G<%2Fauthor><AN>36202251<%2FAN><DT>Journal+Article<%2FDT>)

19.

Sensitive SERS detection of oral squamous cell carcinoma-related miRNAs in saliva via a gold nanohexagon array coupled with hybridization chain reaction amplification.

Wang Y, Zhang Y, Du Q, Cao D, Lu X, Meng Z

Analytical Methods. 14(44):4563-4575, 2022 11 18.

[Journal Article. Research Support, Non-U.S. Gov't]

UI: 36317581

In this work, a highly specific and sensitive method for the detection of dual miRNAs was successfully developed by a hybridization chain reaction (HCR) amplification coupled with surface-enhanced Raman scattering (SERS) on Au-Ag hollow nanoparticles (Au-Ag HNPs) and a gold nanohexagon (AuNH) array. Two Raman reporter-labelled and hairpin DNA-modified Au-Ag HNPs acted as SERS probes (Au-Ag HNPs@4-MBA@HP1-1, Au-Ag HNPs@4-MBA@HP2-1, Au-Ag HNPs@DTNB@HP1-2, and Au-Ag HNPs@DTNB@HP2-2), and the hairpin DNA-modified AuNH array acted as the capture substrate. The HCR process could be triggered by the presence of target miRNAs, and long DNA hybridization chains on the substrate were formed by self-assembly rapidly, causing significant signal enhancement. Using the mentioned strategy, a low detection limit (LOD) of 6.51 aM for miR-31 and 6.52 aM for miR-21 in human saliva were obtained, showing the biosensor's remarkable sensitivity. The proposed biosensor also displays a significant specificity in detecting target miRNAs by introducing different interfering factors. This method has been successfully applied to detect and identify miR-21 and miR-31 in saliva from oral squamous cell carcinoma (OSCC) patients and healthy subjects. The results were consistent with those of the traditional test method in detecting target miRNAs, which confirmed the good accuracy of our method. Hence, the new assay method has great potential to be a valuable platform for detecting miRNAs in the early diagnosis of OSCC.

Version ID

1

Record Owner

From MEDLINE, a database of the U.S. National Library of Medicine.

Status

MEDLINE

Author NameID

Meng, Zhibing; ORCID: <https://orcid.org/0000-0003-2402-6056>

Authors Full Name

Wang, Youwei, Zhang, Yatong, Du, Qiu, Cao, Demao, Lu, Xiaoxia, Meng, Zhibing

Institution

Wang, Youwei. Department of Neurosurgery, Affiliated Hospital of Yangzhou University, Yangzhou, Jiangsu, 225000, China. Zhang, Yatong. Graduate School of Dalian Medical University, Dalian, Liaoning, 116011, China.

Du, Qiu. Department of Neurosurgery, Affiliated Hospital of Yangzhou University, Yangzhou, Jiangsu, 225000, China.

Cao, Demao. Department of Neurosurgery, Affiliated Hospital of Yangzhou University, Yangzhou, Jiangsu, 225000, China.

Lu, Xiaoxia. Department of Oncology, Affiliated Hospital of Yangzhou University, Yangzhou, Jiangsu, 225000, China. luxiaoxia19870318@126.com.

Meng, Zhibing. Department of Oral and Maxillofacial Surgery, Affiliated Hospital of Yangzhou University, Yangzhou, Jiangsu, 225000, China. mengzhibing2007@163.com.

MeSH Heading

Humans. Gold. Silver. MicroRNAs/ge [Genetics]. *MicroRNAs. Saliva. Squamous Cell Carcinoma of Head and Neck. Carcinoma, Squamous Cell/di [Diagnosis]. Carcinoma, Squamous Cell/ge [Genetics]. *Carcinoma, Squamous Cell. Dithionitrobenzoic Acid. Mouth Neoplasms/di [Diagnosis]. Mouth Neoplasms/ge [Genetics]. *Mouth Neoplasms. DNA. *Head and Neck Neoplasms.

Registry Number/Name of Substance

7440-57-5 (Gold). 124985-62-2 (4-mercaptobutyramidine). 3M4G523W1G (Silver). 0 (MicroRNAs). 9BZQ3U62JX (Dithionitrobenzoic Acid). 9007-49-2 (DNA).

Year of Publication

2022

Link to the Ovid Full Text or citation:

[Click here for full text options](https://ovidsp.ovid.com/ovidweb.cgi?T=JS&CSC=Y&NEWS=N&PAGE=fulltext&D=med22&AN=36317581)

Link to the External Link Resolver:

[SFX](https://sfx-86scu.hosted.exlibrisgroup.com.cn/86scu?sid=OVID:medline&id=pmid:36317581&id=doi:10.1039%2Fd2ay01180h&issn=17599660&isbn=&volume=14&issue=44&spage=4563&pages=4563-4575&date=2022&title=Analytical+Methods&atitle=Sensitive+SERS+detection+of+oral+squamous+cell+carcinoma-related+miRNAs+in+saliva+via+a+gold+nanohexagon+array+coupled+with+hybridization+chain+reaction+amplification.&aulast=Wang&pid=<author>Wang+Y%3BZhang+Y%3BDu+Q%3BCao+D%3BLu+X%3BMeng+Z<%2Fauthor><AN>36317581<%2FAN><DT>Journal+Article<%2FDT>)

20.

Utilization of Raman spectroscopy in biochemical fingerprint analysis for oral cancer screening and diagnosis.

Sundramoorthy AK, Atchudan R, Arya S

Oral Oncology. 135:106192, 2022 12.

[Letter. Research Support, Non-U.S. Gov't]

UI: 36270203

Version ID

1

Record Owner

From MEDLINE, a database of the U.S. National Library of Medicine.

Status

MEDLINE

Authors Full Name

Sundramoorthy, Ashok K, Atchudan, Raji, Arya, Sandeep

Institution

Sundramoorthy, Ashok K. Centre for Nano-Biosensors, Department of Prosthodontics, Saveetha Dental College and Hospitals, Saveetha Institute of Medical and Technical Sciences, Chennai 600077, Tamil Nadu, India. Electronic address: ashok.sundramoorthy@gmail.com. Atchudan, Raji. School of Chemical Engineering, Yeungnam University, Gyeongsan 38541, Republic of Korea.

Arya, Sandeep. Department of Physics, University of Jammu, Jammu, Jammu and Kashmir 180006, India.

MeSH Heading

Humans. Spectrum Analysis, Raman/mt [Methods]. *Spectrum Analysis, Raman. Early Detection of Cancer/mt [Methods]. Mouth Neoplasms/di [Diagnosis]. *Mouth Neoplasms.

Year of Publication

2022

Link to the Ovid Full Text or citation:

[Click here for full text options](https://ovidsp.ovid.com/ovidweb.cgi?T=JS&CSC=Y&NEWS=N&PAGE=fulltext&D=med22&AN=36270203)

Link to the External Link Resolver:

[SFX](https://sfx-86scu.hosted.exlibrisgroup.com.cn/86scu?sid=OVID:medline&id=pmid:36270203&id=doi:10.1016%2Fj.oraloncology.2022.106192&issn=13688375&isbn=&volume=135&issue=&spage=106192&pages=106192&date=2022&title=Oral+Oncology&atitle=Utilization+of+Raman+spectroscopy+in+biochemical+fingerprint+analysis+for+oral+cancer+screening+and+diagnosis.&aulast=Sundramoorthy&pid=<author>Sundramoorthy+AK%3BAtchudan+R%3BArya+S<%2Fauthor><AN>36270203<%2FAN><DT>Letter<%2FDT>)

21.

Clinical Use of Raman Spectroscopy Improves Diagnostic Accuracy for Indeterminate Thyroid Nodules.

Palermo A, Sodo A, Naciu AM, Di Gioacchino M, Paolucci A, di Masi A, Maggi D, Crucitti P, Longo F, Perrella E, Taffon C, Verri M, Ricci MA, Crescenzi A

Journal of Clinical Endocrinology & Metabolism. 107(12):3309-3319, 2022 11 25.

[Journal Article. Research Support, Non-U.S. Gov't]

UI: 36103268

BACKGROUND AND OBJECTIVE: Molecular analysis of thyroid fine-needle aspiration (FNA) specimens is believed to improve the management of indeterminate nodules. Raman spectroscopy (RS) can differentiate benign and malignant thyroid lesions in surgically removed tissues, generating distinctive structural profiles. Herein, the diagnostic performance of RS was tested on FNA biopsies of thyroid gland.

DESIGN: Prospective, blinded, and single-center study.

METHODS: We enrolled 123 patients with indeterminate or more ominous cytologic diagnoses (TIR3A-low-risk indeterminate lesion, TIR3B-high-risk indeterminate lesion, TIR4-suspicious of malignancy, TIR5-malignant). All subjects were surgical candidates (defined by international guidelines) and submitted to FNA procedures for RS analysis. We compared RS data, cytologic findings, and final histologic assessments (as reference standard) using various statistical techniques.

RESULTS: The distribution of our study population was as follows: TIR3A:37, TIR3B:32, TIR4:16, and TIR5:38. In 30.9% of patients, histologic diagnoses were benign. For predicting thyroid malignancy in FNA samples, the overall specificity of RS was 86.8%, with 86.5% specificity in indeterminate cytologic categories. In patients with high-risk ultrasound categories, the specificity of RS increased to 87.5% for TIR3A, reaching 100% for TIR3B. Benign histologic diagnoses accounted for 72.9% of patients classified as TIR3A and 31.3% of those classified as TIR3B. Based on positive RS testing, unnecessary surgery was reduced to 7.4% overall (TIR3A-33.3%, TIR3B-6.7%).

CONCLUSIONS: This premier use of RS for thyroid cytology confirms its role as a valuable diagnostic tool and a valid alternative to molecular studies, capable of improving the management of indeterminate nodules and reducing unnecessary surgery.

Copyright © The Author(s) 2022. Published by Oxford University Press on behalf of the Endocrine Society. All rights reserved. For permissions, please e-mail: journals.permissions@oup.com.

Version ID

1

Record Owner

From MEDLINE, a database of the U.S. National Library of Medicine.

Status

MEDLINE

Author NameID

Palermo, Andrea; ORCID: <https://orcid.org/0000-0002-1143-4926> Naciu, Anda Mihaela; ORCID: <https://orcid.org/0000-0002-4576-3381>

Crescenzi, Anna; ORCID: <https://orcid.org/0000-0002-8156-5753>

Authors Full Name

Palermo, Andrea, Sodo, Armida, Naciu, Anda Mihaela, Di Gioacchino, Michael, Paolucci, Alessio, di Masi, Alessandra, Maggi, Daria, Crucitti, Pierfilippo, Longo, Filippo, Perrella, Eleonora, Taffon, Chiara, Verri, Martina, Ricci, Maria Antonietta, Crescenzi, Anna

Institution

Palermo, Andrea. Unit of Metabolic Bone and Thyroid Disorders, Fondazione Policlinico Universitario Campus Bio-Medico, Via Alvaro del Portillo, 200 - 00128 Roma, Italy. Palermo, Andrea. Unit of Endocrinology and Diabetes, Department of Medicine and Surgery, Universita Campus Bio-Medico di Roma, Via Alvaro del Portillo, 21 - 00128 Roma, Italy.

Sodo, Armida. Dipartimento di Scienze, Universita Roma Tre, Rome, Italy.

Naciu, Anda Mihaela. Unit of Metabolic Bone and Thyroid Disorders, Fondazione Policlinico Universitario Campus Bio-Medico, Via Alvaro del Portillo, 200 - 00128 Roma, Italy.

Di Gioacchino, Michael. Dipartimento di Scienze, Universita Roma Tre, Rome, Italy.

Paolucci, Alessio. Dipartimento di Scienze, Universita Roma Tre, Rome, Italy.

di Masi, Alessandra. Dipartimento di Scienze, Universita Roma Tre, Rome, Italy.

Maggi, Daria. Unit of Endocrinology and Diabetes, Fondazione Policlinico Universitario Campus Bio-Medico, Rome, Italy.

Crucitti, Pierfilippo. Unit of Thoracic Surgery, Fondazione Policlinico Universitario Campus Bio-Medico, Rome, Italy.

Longo, Filippo. Unit of Thoracic Surgery, Fondazione Policlinico Universitario Campus Bio-Medico, Rome, Italy.

Perrella, Eleonora. Unit of Pathology, Fondazione Policlinico Universitario Campus Bio-Medico, Rome, Italy.

Taffon, Chiara. Unit of Pathology, Fondazione Policlinico Universitario Campus Bio-Medico, Rome, Italy.

Verri, Martina. Unit of Pathology, Fondazione Policlinico Universitario Campus Bio-Medico, Rome, Italy.

Ricci, Maria Antonietta. Dipartimento di Scienze, Universita Roma Tre, Rome, Italy.

Crescenzi, Anna. Unit of Pathology, Fondazione Policlinico Universitario Campus Bio-Medico, Rome, Italy.

MeSH Heading

Humans. Thyroid Nodule/di [Diagnosis]. Thyroid Nodule/su [Surgery]. Thyroid Nodule/pa [Pathology]. *Thyroid Nodule. Prospective Studies. Spectrum Analysis, Raman. Biopsy, Fine-Needle. Thyroid Neoplasms/di [Diagnosis]. Thyroid Neoplasms/su [Surgery]. Thyroid Neoplasms/pa [Pathology]. *Thyroid Neoplasms. Retrospective Studies.

Keyword Heading

Raman spectroscopy indeterminate cytology

thyroid

thyroid nodule.

Keyword Heading Owner

NOTNLM

Year of Publication

2022

Link to the Ovid Full Text or citation:

[Click here for full text options](https://ovidsp.ovid.com/ovidweb.cgi?T=JS&CSC=Y&NEWS=N&PAGE=fulltext&D=med22&AN=36103268)

Link to the External Link Resolver:

[SFX](https://sfx-86scu.hosted.exlibrisgroup.com.cn/86scu?sid=OVID:medline&id=pmid:36103268&id=doi:10.1210%2Fclinem%2Fdgac537&issn=0021972X&isbn=&volume=107&issue=12&spage=3309&pages=3309-3319&date=2022&title=Journal+of+Clinical+Endocrinology+%26+Metabolism&atitle=Clinical+Use+of+Raman+Spectroscopy+Improves+Diagnostic+Accuracy+for+Indeterminate+Thyroid+Nodules.&aulast=Palermo&pid=<author>Palermo+A%3BSodo+A%3BNaciu+AM%3BDi+Gioacchino+M%3BPaolucci+A%3Bdi+Masi+A%3BMaggi+D%3BCrucitti+P%3BLongo+F%3BPerrella+E%3BTaffon+C%3BVerri+M%3BRicci+MA%3BCrescenzi+A<%2Fauthor><AN>36103268<%2FAN><DT>Journal+Article<%2FDT>)

22.

Simulated fine-needle aspiration diagnosis of follicular thyroid nodules by hyperspectral Raman microscopy and chemometric analysis.

Soares de Oliveira MA, Campbell M, Afify AM, Huang EC, Chan JW

Journal of Biomedical Optics. 27(9), 2022 09.

[Journal Article. Research Support, N.I.H., Extramural. Research Support, Non-U.S. Gov't]

UI: 36071559

SIGNIFICANCE: Follicular thyroid carcinoma carries a substantially poor prognosis due to its unique biological behavior and less favorable outcomes. In particular, fine-needle aspiration (FNA) biopsies, which play a key role in screening thyroid nodules, cannot differentiate benign from malignant follicular neoplasm.

AIM: We report on the use of hyperspectral Raman microscopy in combination with chemometric analysis for identifying and classifying single cells obtained from clinical samples of human follicular thyroid neoplasms.

APPROACH: We used a method intended to simulate the FNA procedure to obtain single cells from thyroid nodules. A total of 392 hyperspectral Raman images of single cells from follicular thyroid neoplasms were collected.

RESULTS: Malignant cells were identified based on their intrinsic Raman spectral signatures with an overall diagnostic accuracy of up to 83.7%.

CONCLUSIONS: Our findings indicate that hyperspectral Raman microscopy can potentially be developed into an ancillary test for analyzing single cells from thyroid FNA biopsies to better stratify "indeterminate" nodules and other cytologically challenging cases.

Version ID

1

Record Owner

From MEDLINE, a database of the U.S. National Library of Medicine.

Status

MEDLINE

Authors Full Name

Soares de Oliveira, Marcos A, Campbell, Michael, Afify, Alaa M, Huang, Eric C, Chan, James W

Institution

Soares de Oliveira, Marcos A. University of California Davis, Department of Pathology and Laboratory Medicine, Sacramento, California, United States, United States. Campbell, Michael. University of California Davis, Department of Surgery, Sacramento, California, United States, United States.

Afify, Alaa M. University of California Davis, Department of Pathology and Laboratory Medicine, Sacramento, California, United States, United States.

Huang, Eric C. University of Washington, Department of Laboratory Medicine and Pathology, Seattle, Washington, United States, United States.

Chan, James W. University of California Davis, Department of Pathology and Laboratory Medicine, Sacramento, California, United States, United States.

MeSH Heading

Biopsy, Fine-Needle. Chemometrics. Humans. Microscopy. Thyroid Neoplasms/dg [Diagnostic Imaging]. *Thyroid Neoplasms. Thyroid Nodule/dg [Diagnostic Imaging]. Thyroid Nodule/pa [Pathology]. *Thyroid Nodule.

Keyword Heading

Raman spectroscopy chemometric analysis

follicular thyroid neoplasm

hyperspectral Raman imaging

thyroid cancer.

Keyword Heading Owner

NOTNLM

Year of Publication

2022

Link to the Ovid Full Text or citation:

[Click here for full text options](https://ovidsp.ovid.com/ovidweb.cgi?T=JS&CSC=Y&NEWS=N&PAGE=fulltext&D=med22&AN=36071559)

Link to the External Link Resolver:

[SFX](https://sfx-86scu.hosted.exlibrisgroup.com.cn/86scu?sid=OVID:medline&id=pmid:36071559&id=doi:10.1117%2F1.JBO.27.9.095001&issn=10833668&isbn=&volume=27&issue=9&spage=&pages=&date=2022&title=Journal+of+Biomedical+Optics&atitle=Simulated+fine-needle+aspiration+diagnosis+of+follicular+thyroid+nodules+by+hyperspectral+Raman+microscopy+and+chemometric+analysis.&aulast=Soares+de+Oliveira&pid=<author>Soares+de+Oliveira+MA%3BCampbell+M%3BAfify+AM%3BHuang+EC%3BChan+JW<%2Fauthor><AN>36071559<%2FAN><DT>Journal+Article<%2FDT>)

23.

Synthesis of Electron-Rich Porous Organic Polymers via Schiff-Base Chemistry for Efficient Iodine Capture.

Tian P, Ai Z, Hu H, Wang M, Li Y, Gao X, Qian J, Su X, Xiao S, Xu H, Lu F, Gao Y

Molecules. 27(16), 2022 Aug 12.

[Journal Article]

UI: 36014397

As one of the main nuclear wastes generated in the process of nuclear fission, radioactive iodine has attracted worldwide attention due to its harm to public safety and environmental pollution. Therefore, it is of crucial importance to develop materials that can rapidly and efficiently capture radioactive iodine. Herein, we report the construction of three electron-rich porous organic polymers (POPs), denoted as POP-E, POP-T and POP-P via Schiff base polycondensations reactions between Td-symmetric adamantane knot and four-branched "linkage" molecules. We demonstrated that all the three POPs showed high iodine adsorption capability, among which the adsorption capacity of POP-T for iodine vapor reached up to 3.94 g.g-1 and the removal rate of iodine in n-hexane solution was up to 99%. The efficient iodine capture mechanism of the POP-T was investigated through systematic comparison of Fourier transform infrared spectroscopy (FT-IR), Raman spectroscopy and X-ray photoelectron spectroscopy (XPS) before and after iodine adsorption. The unique pi-pi conjugated system between imine bonds linked aromatic rings with iodine result in charge-transfer complexes, which explains the exceptional iodine capture capacity. Additionally, the introduction of heteroatoms into the framework would also enhance the iodine adsorption capability of POPs. Good retention behavior and recycling capacity were also observed for the POPs.

Version ID

1

Record Owner

From MEDLINE, a database of the U.S. National Library of Medicine.

Status

MEDLINE

Author NameID

Gao, Yanan; ORCID: <https://orcid.org/0000-0002-5543-6296>

Authors Full Name

Tian, Peng, Ai, Zhiting, Hu, Hui, Wang, Ming, Li, Yaling, Gao, Xinpei, Qian, Jiaying, Su, Xiaofang, Xiao, Songtao, Xu, Huanjun, Lu, Fei, Gao, Yanan

Institution

Tian, Peng. Key Laboratory of Ministry of Education for Advanced Materials in Tropical Island Resources, Department of Chemistry and Chemical Engineering, Hainan University, No 58, Renmin Avenue, Haikou 570228, China. Ai, Zhiting. Key Laboratory of Ministry of Education for Advanced Materials in Tropical Island Resources, Department of Chemistry and Chemical Engineering, Hainan University, No 58, Renmin Avenue, Haikou 570228, China.

Hu, Hui. Key Laboratory of Ministry of Education for Advanced Materials in Tropical Island Resources, Department of Chemistry and Chemical Engineering, Hainan University, No 58, Renmin Avenue, Haikou 570228, China.

Wang, Ming. Key Laboratory of Ministry of Education for Advanced Materials in Tropical Island Resources, Department of Chemistry and Chemical Engineering, Hainan University, No 58, Renmin Avenue, Haikou 570228, China.

Li, Yaling. Key Laboratory of Ministry of Education for Advanced Materials in Tropical Island Resources, Department of Chemistry and Chemical Engineering, Hainan University, No 58, Renmin Avenue, Haikou 570228, China.

Gao, Xinpei. Key Laboratory of Ministry of Education for Advanced Materials in Tropical Island Resources, Department of Chemistry and Chemical Engineering, Hainan University, No 58, Renmin Avenue, Haikou 570228, China.

Qian, Jiaying. Key Laboratory of Ministry of Education for Advanced Materials in Tropical Island Resources, Department of Chemistry and Chemical Engineering, Hainan University, No 58, Renmin Avenue, Haikou 570228, China.

Su, Xiaofang. Key Laboratory of Ministry of Education for Advanced Materials in Tropical Island Resources, Department of Chemistry and Chemical Engineering, Hainan University, No 58, Renmin Avenue, Haikou 570228, China.

Xiao, Songtao. China Institute of Atomic Energy, Beijing 102413, China.

Xu, Huanjun. School of Science, Qiongtai Normal University, Haikou 571127, China.

Lu, Fei. Key Laboratory of Ministry of Education for Advanced Materials in Tropical Island Resources, Department of Chemistry and Chemical Engineering, Hainan University, No 58, Renmin Avenue, Haikou 570228, China.

Gao, Yanan. Key Laboratory of Ministry of Education for Advanced Materials in Tropical Island Resources, Department of Chemistry and Chemical Engineering, Hainan University, No 58, Renmin Avenue, Haikou 570228, China.

MeSH Heading

Electrons. Humans. Iodides. Iodine/ch [Chemistry]. *Iodine. Iodine Radioisotopes. Polymers/ch [Chemistry]. Porosity. Schiff Bases. Spectroscopy, Fourier Transform Infrared. *Thyroid Neoplasms.

Keyword Heading

charge-transfer complexes electron-rich framework

iodine capture

porous organic polymers

radioiodine.

Keyword Heading Owner

NOTNLM

Registry Number/Name of Substance

0 (Iodides). 0 (Iodine Radioisotopes). 0 (Polymers). 0 (Schiff Bases). 9679TC07X4 (Iodine).

Year of Publication

2022

Link to the Ovid Full Text or citation:

[Click here for full text options](https://ovidsp.ovid.com/ovidweb.cgi?T=JS&CSC=Y&NEWS=N&PAGE=fulltext&D=med22&AN=36014397)

Link to the External Link Resolver:

[SFX](https://sfx-86scu.hosted.exlibrisgroup.com.cn/86scu?sid=OVID:medline&id=pmid:36014397&id=doi:10.3390%2Fmolecules27165161&issn=14203049&isbn=&volume=27&issue=16&spage=5161&pages=&date=2022&title=Molecules&atitle=Synthesis+of+Electron-Rich+Porous+Organic+Polymers+via+Schiff-Base+Chemistry+for+Efficient+Iodine+Capture.&aulast=Tian&pid=<author>Tian+P%3BAi+Z%3BHu+H%3BWang+M%3BLi+Y%3BGao+X%3BQian+J%3BSu+X%3BXiao+S%3BXu+H%3BLu+F%3BGao+Y<%2Fauthor><AN>36014397<%2FAN><DT>Journal+Article<%2FDT>)

24.

Histologically resolved multiomics enables precise molecular profiling of human intratumor heterogeneity.

Chen T, Cao C, Zhang J, Streets A, Li T, Huang Y

Plos Biology. 20(7):e3001699, 2022 07.

[Journal Article. Research Support, Non-U.S. Gov't. Research Support, U.S. Gov't, Non-P.H.S.]

UI: 35776767

Both the composition of cell types and their spatial distribution in a tissue play a critical role in cellular function, organ development, and disease progression. For example, intratumor heterogeneity and the distribution of transcriptional and genetic events in single cells drive the genesis and development of cancer. However, it can be challenging to fully characterize the molecular profile of cells in a tissue with high spatial resolution because microscopy has limited ability to extract comprehensive genomic information, and the spatial resolution of genomic techniques tends to be limited by dissection. There is a growing need for tools that can be used to explore the relationship between histological features, gene expression patterns, and spatially correlated genomic alterations in healthy and diseased tissue samples. Here, we present a technique that combines label-free histology with spatially resolved multiomics in unfixed and unstained tissue sections. This approach leverages stimulated Raman scattering microscopy to provide chemical contrast that reveals histological tissue architecture, allowing for high-resolution in situ laser microdissection of regions of interests. These microtissue samples are then processed for DNA and RNA sequencing to identify unique genetic profiles that correspond to distinct anatomical regions. We demonstrate the capabilities of this technique by mapping gene expression and copy number alterations to histologically defined regions in human oral squamous cell carcinoma (OSCC). Our approach provides complementary insights in tumorigenesis and offers an integrative tool for macroscale cancer tissues with spatial multiomics assessments.

Version ID

1

Record Owner

From MEDLINE, a database of the U.S. National Library of Medicine.

Status

MEDLINE

Author NameID

Streets, Aaron; ORCID: <https://orcid.org/0000-0002-3909-8389> Huang, Yanyi; ORCID: <https://orcid.org/0000-0002-7297-1266>

Authors Full Name

Chen, Tao, Cao, Chen, Zhang, Jianyun, Streets, Aaron, Li, Tiejun, Huang, Yanyi

Institution

Chen, Tao. Biomedical Pioneering Innovation Center (BIOPIC), School of Life Sciences, Peking University, Beijing, China. Chen, Tao. College of Engineering, Peking University, Beijing, China.

Cao, Chen. Biomedical Pioneering Innovation Center (BIOPIC), School of Life Sciences, Peking University, Beijing, China.

Zhang, Jianyun. Department of Oral Pathology, Peking University School and Hospital of Stomatology & National Center of Stomatology & National Clinical Research Center for Oral Diseases & National Engineering Research Center of Oral Biomaterials and Digital Medical Devices, Beijing, China.

Zhang, Jianyun. Beijing Key Laboratory of Digital Stomatology.

Streets, Aaron. Biomedical Pioneering Innovation Center (BIOPIC), School of Life Sciences, Peking University, Beijing, China.

Li, Tiejun. Department of Oral Pathology, Peking University School and Hospital of Stomatology & National Center of Stomatology & National Clinical Research Center for Oral Diseases & National Engineering Research Center of Oral Biomaterials and Digital Medical Devices, Beijing, China.

Li, Tiejun. Beijing Key Laboratory of Digital Stomatology.

Li, Tiejun. Research Unit of Precision Pathologic Diagnosis in Tumors of the Oral and Maxillofacial Regions, Chinese Academy of Medical Sciences (2019RU034), Beijing, China.

Huang, Yanyi. Biomedical Pioneering Innovation Center (BIOPIC), School of Life Sciences, Peking University, Beijing, China.

Huang, Yanyi. College of Engineering, Peking University, Beijing, China.

Huang, Yanyi. Peking-Tsinghua Center for Life Sciences, Peking University, Beijing, China.

Huang, Yanyi. Beijing Advanced Innovation Center for Genomics (ICG), Peking University, Beijing, China.

Huang, Yanyi. College of Chemistry and Molecular Engineering, Peking University, Beijing, China.

Huang, Yanyi. Institute for Cell Analysis, Shenzhen Bay Laboratory, Guangdong, China.

MeSH Heading

Carcinoma, Squamous Cell/ge [Genetics]. *Carcinoma, Squamous Cell. DNA Copy Number Variations/ge [Genetics]. Gene Expression Profiling/mt [Methods]. Genomics. Humans. *Mouth Neoplasms. Sequence Analysis, RNA.

Year of Publication

2022

Link to the Ovid Full Text or citation:

[Click here for full text options](https://ovidsp.ovid.com/ovidweb.cgi?T=JS&CSC=Y&NEWS=N&PAGE=fulltext&D=med22&AN=35776767)

Link to the External Link Resolver:

[SFX](https://sfx-86scu.hosted.exlibrisgroup.com.cn/86scu?sid=OVID:medline&id=pmid:35776767&id=doi:10.1371%2Fjournal.pbio.3001699&issn=15449173&isbn=&volume=20&issue=7&spage=e3001699&pages=e3001699&date=2022&title=Plos+Biology&atitle=Histologically+resolved+multiomics+enables+precise+molecular+profiling+of+human+intratumor+heterogeneity.&aulast=Chen&pid=<author>Chen+T%3BCao+C%3BZhang+J%3BStreets+A%3BLi+T%3BHuang+Y<%2Fauthor><AN>35776767<%2FAN><DT>Journal+Article<%2FDT>)

25.

Multi-Modal Optical Imaging and Combined Phototherapy of Nasopharyngeal Carcinoma Based on a Nanoplatform. [Review]

Lin Y, Qiu T, Lan Y, Li Z, Wang X, Zhou M, Li Q, Li Y, Liang J, Zhang J

International Journal of Nanomedicine. 17:2435-2446, 2022.

[Journal Article. Review]

UI: 35656166

Nasopharyngeal carcinoma (NPC) is a common malignant tumor of the head and neck with a high incidence rate worldwide, especially in southern China. Phototheranostics in combination with nanoparticles is an integrated strategy for enabling simultaneous diagnosis, real-time monitoring, and administration of precision therapy for nasopharyngeal carcinoma (NPC). It has shown great potential in the field of cancer diagnosis and treatment owing to its unique noninvasive advantages. Many Chinese and international research teams have applied nano-targeted drugs to optical diagnosis and treatment technology to conduct multimodal imaging and collaborative treatment of NPC, which has become a hot research topic. In this review, we aimed to introduce the recent developments in phototheranostics of NPC based on a nanoplatform. This study aimed to elaborate on the applications of nanoplatform-based optical imaging strategies and treatment modalities, including fluorescence imaging, photoacoustic imaging, Raman spectroscopy imaging, photodynamic therapy, and photothermal therapy. This study is expected to provide a scientific basis for further research and development of NPC diagnosis and treatment.

Copyright © 2022 Lin et al.

Version ID

1

Record Owner

From MEDLINE, a database of the U.S. National Library of Medicine.

Status

MEDLINE

Author NameID

Zhang, Jian; ORCID: <https://orcid.org/0000-0002-9665-9322>

Authors Full Name

Lin, Yanping, Qiu, Ting, Lan, Yintao, Li, Zhaoyong, Wang, Xin, Zhou, Mengyu, Li, Qiuyu, Li, Yao, Liang, Junsheng, Zhang, Jian

Institution

Lin, Yanping. Department of Radiology, DongGuan Tungwah Hospital, DongGuan, Guangdong, 523000, People's Republic of China. Qiu, Ting. Department of Radiology, Zhuhai People's Hospital (Zhuhai Hospital Affiliated with Jinan University), Zhuhai, Guangdong, 519000, People's Republic of China.

Lan, Yintao. Department of Biomedical Engineering, School of Basic Medical Sciences, Guangzhou Medical University, Guangzhou, Guangdong, 511436, People's Republic of China.

Li, Zhaoyong. Department of Radiology, DongGuan Tungwah Hospital, DongGuan, Guangdong, 523000, People's Republic of China.

Wang, Xin. Department of Oncology, The Sixth Affiliated Hospital of Guangzhou Medical University, Qingyuan People's Hospital, Qingyuan, Guangdong, 511500, People's Republic of China.

Zhou, Mengyu. Department of Biomedical Engineering, School of Basic Medical Sciences, Guangzhou Medical University, Guangzhou, Guangdong, 511436, People's Republic of China.

Li, Qiuyu. Department of Radiology, DongGuan Tungwah Hospital, DongGuan, Guangdong, 523000, People's Republic of China.

Li, Yao. Department of Radiology, DongGuan Tungwah Hospital, DongGuan, Guangdong, 523000, People's Republic of China.

Liang, Junsheng. Department of Radiology, DongGuan Tungwah Hospital, DongGuan, Guangdong, 523000, People's Republic of China.

Zhang, Jian. Department of Biomedical Engineering, School of Basic Medical Sciences, Guangzhou Medical University, Guangzhou, Guangdong, 511436, People's Republic of China.

Zhang, Jian. Department of Oncology, The Sixth Affiliated Hospital of Guangzhou Medical University, Qingyuan People's Hospital, Qingyuan, Guangdong, 511500, People's Republic of China.

MeSH Heading

Humans. Nasopharyngeal Carcinoma/dg [Diagnostic Imaging]. Nasopharyngeal Carcinoma/th [Therapy]. Nasopharyngeal Neoplasms/dg [Diagnostic Imaging]. Nasopharyngeal Neoplasms/th [Therapy]. *Nasopharyngeal Neoplasms. Optical Imaging. *Phototherapy. Photothermal Therapy.

Keyword Heading

nanoparticle nasopharyngeal carcinoma

optical imaging

phototherapy.

Keyword Heading Owner

NOTNLM

Year of Publication

2022

Link to the Ovid Full Text or citation:

[Click here for full text options](https://ovidsp.ovid.com/ovidweb.cgi?T=JS&CSC=Y&NEWS=N&PAGE=fulltext&D=med22&AN=35656166)

Link to the External Link Resolver:

[SFX](https://sfx-86scu.hosted.exlibrisgroup.com.cn/86scu?sid=OVID:medline&id=pmid:35656166&id=doi:10.2147%2FIJN.S357493&issn=11769114&isbn=&volume=17&issue=&spage=2435&pages=2435-2446&date=2022&title=International+Journal+of+Nanomedicine&atitle=Multi-Modal+Optical+Imaging+and+Combined+Phototherapy+of+Nasopharyngeal+Carcinoma+Based+on+a+Nanoplatform.&aulast=Lin&pid=<author>Lin+Y%3BQiu+T%3BLan+Y%3BLi+Z%3BWang+X%3BZhou+M%3BLi+Q%3BLi+Y%3BLiang+J%3BZhang+J<%2Fauthor><AN>35656166<%2FAN><DT>Journal+Article<%2FDT>)

26.

Machine-learning-assisted spontaneous Raman spectroscopy classification and feature extraction for the diagnosis of human laryngeal cancer.

Li Z, Li Z, Chen Q, Zhang J, Dunham ME, McWhorter AJ, Feng JM, Li Y, Yao S, Xu J

Computers in Biology & Medicine. 146:105617, 2022 07.

[Journal Article. Research Support, Non-U.S. Gov't. Research Support, U.S. Gov't, Non-P.H.S.]

UI: 35605486

The early detection of laryngeal cancer significantly increases the survival rates, permits more conservative larynx sparing treatments, and reduces healthcare costs. A non-invasive optical form of biopsy for laryngeal carcinoma can increase the early detection rate, allow for more accurate monitoring of its recurrence, and improve intraoperative margin control. In this study, we evaluated a Raman spectroscopy system for the rapid intraoperative detection of human laryngeal carcinoma. The spectral analysis methods included principal component analysis (PCA), random forest (RF), and one-dimensional (1D) convolutional neural network (CNN) methods. We measured the Raman spectra from 207 normal and 500 tumor sites collected from 10 human laryngeal cancer surgical specimens. Random Forest analysis yielded an overall accuracy of 90.5%, sensitivity of 88.2%, and specificity of 92.8% on average over 10 trials. The 1D CNN demonstrated the highest performance with an accuracy of 96.1%, sensitivity of 95.2%, and specificity of 96.9% on average over 50 trials. In predicting the first three principal components (PCs) of normal and tumor data, both RF and CNN demonstrated high performances, except for the tumor PC2. This is the first study in which CNN-assisted Raman spectroscopy was used to identify human laryngeal cancer tissue with extracted feature weights. The proposed Raman spectroscopy feature extraction approach has not been previously applied to human cancer diagnosis. Raman spectroscopy, as assisted by machine learning (ML) methods, has the potential to serve as an intraoperative, non-invasive tool for the rapid diagnosis of laryngeal cancer and margin detection.

Copyright © 2022 Elsevier Ltd. All rights reserved.

Version ID

1

Record Owner

From MEDLINE, a database of the U.S. National Library of Medicine.

Status

MEDLINE

Authors Full Name

Li, Zheng, Li, Zhongqiang, Chen, Qing, Zhang, Jian, Dunham, Michael E, McWhorter, Andrew J, Feng, Ji-Ming, Li, Yanping, Yao, Shaomian, Xu, Jian

Institution

Li, Zheng. Division of Electrical and Computer Engineering, College of Engineering, Louisiana State University, Baton Rouge, LA, 70803, USA. Li, Zhongqiang. Division of Electrical and Computer Engineering, College of Engineering, Louisiana State University, Baton Rouge, LA, 70803, USA.

Chen, Qing. Division of Computer Science & Engineering, College of Engineering, Louisiana State University, Baton Rouge, LA, 70803, USA.

Zhang, Jian. Division of Computer Science & Engineering, College of Engineering, Louisiana State University, Baton Rouge, LA, 70803, USA.

Dunham, Michael E. Department of Otolaryngology, School of Medicine, Louisiana State University Health Science Center, New Orleans, LA, 70112, USA.

McWhorter, Andrew J. Department of Otolaryngology, School of Medicine, Louisiana State University Health Science Center, New Orleans, LA, 70112, USA.

Feng, Ji-Ming. Department of Comparative Biomedical Science, School of Veterinary Medicine, Louisiana State University, Baton Rouge, LA, 70803, USA.

Li, Yanping. School of Environment and Sustainability, University of Saskatchewan, Saskatoon, SK S7N 5C9, Canada.

Yao, Shaomian. Department of Comparative Biomedical Science, School of Veterinary Medicine, Louisiana State University, Baton Rouge, LA, 70803, USA.

Xu, Jian. Division of Electrical and Computer Engineering, College of Engineering, Louisiana State University, Baton Rouge, LA, 70803, USA. Electronic address: jianxu1@lsu.edu.

MeSH Heading

*Carcinoma. Humans. Laryngeal Neoplasms/dg [Diagnostic Imaging]. *Laryngeal Neoplasms. Machine Learning. Neural Networks, Computer. Spectrum Analysis, Raman/mt [Methods].

Keyword Heading

Convolutional neural network Laryngeal cancer

Principal component analysis

Raman spectroscopy

Random forest.

Keyword Heading Owner

NOTNLM

Year of Publication

2022

Link to the Ovid Full Text or citation:

[Click here for full text options](https://ovidsp.ovid.com/ovidweb.cgi?T=JS&CSC=Y&NEWS=N&PAGE=fulltext&D=med22&AN=35605486)

Link to the External Link Resolver:

[SFX](https://sfx-86scu.hosted.exlibrisgroup.com.cn/86scu?sid=OVID:medline&id=pmid:35605486&id=doi:10.1016%2Fj.compbiomed.2022.105617&issn=00104825&isbn=&volume=146&issue=&spage=105617&pages=105617&date=2022&title=Computers+in+Biology+%26+Medicine&atitle=Machine-learning-assisted+spontaneous+Raman+spectroscopy+classification+and+feature+extraction+for+the+diagnosis+of+human+laryngeal+cancer.&aulast=Li&pid=<author>Li+Z%3BLi+Z%3BChen+Q%3BZhang+J%3BDunham+ME%3BMcWhorter+AJ%3BFeng+JM%3BLi+Y%3BYao+S%3BXu+J<%2Fauthor><AN>35605486<%2FAN><DT>Journal+Article<%2FDT>)

27.

The effect of therapeutic radiation on dental enamel and dentin: A systematic review. [Review]

Douchy L, Gauthier R, Abouelleil-Sayed H, Colon P, Grosgogeat B, Bosco J

Dental Materials. 38(7):e181-e201, 2022 07.

[Journal Article. Review. Systematic Review]

UI: 35570008

OBJECTIVES: The conventional radiotherapy protocol to treat head-and-neck cancer is usually followed by tooth-decay onset. Radiation impact on mineralized tooth structures is not well-understood. This systematic review aimed to collect the recorded effects of therapeutic radiation on tooth chemical, structural and mechanical properties, in relation with their means of investigation.

DATA: Systematic search (January 01 2012 - September 30 2021) terms were "Radiotherapy", "Radiation effects", "Dental enamel", "Dentin", "Human" and "Radiotherapy" NOT "Laser".

SOURCES: PubMed, DOSS and Embase databases were searched.

STUDY SELECTION: Selected studies compared dental enamel, coronal and root dentin properties before and after in vitro or in vivo irradiation up to 80 Gy.

RESULTS: The systematic search identified 353 different articles, with 28 satisfying inclusion criteria. Their reference lists provided two more. Twenty-two studies evaluated dental enamel evolution, nine assessed coronal dentin and eight concerned root dentin. Coronal and root dentin results indicate a major impact of the radiation on their organic matrix. Dental enamel's chemical properties are less modified. Enamel and root dentin's hardness are decreased by therapeutic radiation, but no consensus arises for coronal dentin.

CONCLUSIONS: Our findings revealed some interesting information about enzymatic degradation mechanisms of dentin organic matrix and highlighted that dental hard-tissue characterization requires highly specific expertise in materials science. That scientific knowledge is necessary to design suitable protocols, adequately analyze the obtained data, and, thus, provide relevant conclusions.

CLINICAL SIGNIFICANCE STATEMENT: Better knowledge and understanding of the mechanisms involved in the degradation of enamel and dentin would enable development of new preventive and therapeutic methods for improved medical care of patients undergoing radiotherapy.

Copyright © 2022 Elsevier Inc. All rights reserved.

Version ID

1

Record Owner

From MEDLINE, a database of the U.S. National Library of Medicine.

Status

MEDLINE

Authors Full Name

Douchy, Laura, Gauthier, Remy, Abouelleil-Sayed, Hazem, Colon, Pierre, Grosgogeat, Brigitte, Bosco, Julia

Institution

Douchy, Laura. Universite de Lyon, Universite Lyon 1, Laboratoire des Multimateriaux et des Interfaces, UMR CNRS, 5615, Lyon, France; Universite de Paris, Faculte d'Odontologie, Paris, France; Assistance Publique-Hopitaux de Paris, Hopital Rothschild, Service Odontologie, Paris, France. Electronic address: laura.douchy@etu.u-paris.fr. Gauthier, Remy. Universite de Lyon, Universite Lyon 1, Laboratoire des Multimateriaux et des Interfaces, UMR CNRS, 5615, Lyon, France.

Abouelleil-Sayed, Hazem. Universite de Lyon, Universite Lyon 1, Laboratoire des Multimateriaux et des Interfaces, UMR CNRS, 5615, Lyon, France; Universite de Lyon, Universite Lyon 1, Faculte d'Odontologie, Lyon, France.

Colon, Pierre. Universite de Lyon, Universite Lyon 1, Laboratoire des Multimateriaux et des Interfaces, UMR CNRS, 5615, Lyon, France; Universite de Paris, Faculte d'Odontologie, Paris, France; Assistance Publique-Hopitaux de Paris, Hopital Rothschild, Service Odontologie, Paris, France.

Grosgogeat, Brigitte. Universite de Lyon, Universite Lyon 1, Laboratoire des Multimateriaux et des Interfaces, UMR CNRS, 5615, Lyon, France; Hospices Civils de Lyon, Service d'Odontologie, Lyon, France; Universite de Lyon, Universite Lyon 1, Faculte d'Odontologie, Lyon, France.

Bosco, Julia. Universite de Lyon, Universite Lyon 1, Laboratoire des Multimateriaux et des Interfaces, UMR CNRS, 5615, Lyon, France; Universite de Paris, Faculte d'Odontologie, Paris, France; Assistance Publique-Hopitaux de Paris, Hopital Pitie-Salpetriere, Service Odontologie, Paris, France.

MeSH Heading

Dental Caries/et [Etiology]. *Dental Caries. Dental Enamel/ch [Chemistry]. Dental Enamel/re [Radiation Effects]. Dentin/ch [Chemistry]. Dentin/re [Radiation Effects]. Head and Neck Neoplasms/rt [Radiotherapy]. *Head and Neck Neoplasms. Humans. *Radiation Injuries. Tooth/re [Radiation Effects]. *Tooth.

Keyword Heading

Dental enamel Dentin

Fourier-transform infrared spectroscopy

Hardness test

Head and neck neoplasms

Radiotherapy

Raman Spectroscopy

Systematic review

X-ray diffraction.

Keyword Heading Owner

NOTNLM

Year of Publication

2022

Link to the Ovid Full Text or citation:

[Click here for full text options](https://ovidsp.ovid.com/ovidweb.cgi?T=JS&CSC=Y&NEWS=N&PAGE=fulltext&D=med22&AN=35570008)

Link to the External Link Resolver:

[SFX](https://sfx-86scu.hosted.exlibrisgroup.com.cn/86scu?sid=OVID:medline&id=pmid:35570008&id=doi:10.1016%2Fj.dental.2022.04.014&issn=01095641&isbn=&volume=38&issue=7&spage=e181&pages=e181-e201&date=2022&title=Dental+Materials&atitle=The+effect+of+therapeutic+radiation+on+dental+enamel+and+dentin%3A+A+systematic+review.&aulast=Douchy&pid=<author>Douchy+L%3BGauthier+R%3BAbouelleil-Sayed+H%3BColon+P%3BGrosgogeat+B%3BBosco+J<%2Fauthor><AN>35570008<%2FAN><DT>Journal+Article<%2FDT>)

28.

Pump-free microfluidic chip based laryngeal squamous cell carcinoma-related microRNAs detection through the combination of surface-enhanced Raman scattering techniques and catalytic hairpin assembly amplification.

Ge S, Li G, Zhou X, Mao Y, Gu Y, Li Z, Gu Y, Cao X

Talanta. 245:123478, 2022 Aug 01.

[Journal Article]

UI: 35436733

MicroRNA (miRNA), as one of the ideal target biomarker analytes, plays an essential role in biological processes; thus, the development of rapidly sensitive detection methods is imperative. Herein, we proposed a pump-free surface-enhanced Raman scatting (SERS) microfluidic chip for the rapid and ultrasensitive detection of miR-106b and miR-196b, laryngeal squamous cell carcinoma (LSCC)-related miRNAs. Ag-Au core-shell nanorods (Ag-AuNRs) were applied to prepare SERS tags by modifying Raman reporters and hairpin DNAs. The capture probes were synthesized by labeling hairpin DNAs onto the magnetic beads (MBs) surface. In the presence of targets, the catalytic hairpin assembly (CHA) reactions between SERS tags and capture probes could be triggered, causing the aggregation of Ag-AuNRs. The tiny magnets installed under the rectangular chamber could magnetically gather the CHA products, leading to the further aggregation of Ag-AuNRs. Thus, this strategy could achieve the double aggregation of Ag-AuNRs, resulting in the significant amplification of the SERS signal. The proposed strategy achieved simultaneous and sensitive detection of miR-106b and miR-196b, with limits of detection low to aM level. The whole detection process could be completed within 5 min. Moreover, this microfluidic chip exhibited excellent reproducibility, stability, and specificity. The high accuracy of this SERS microfluidic chip was proved by practical analysis in LSCC patients' serum. The results demonstrated that SERS could be a promising alternative clinical diagnosis tool and exhibited potential application for the dynamic monitoring of cancer staging.

Copyright © 2022 Elsevier B.V. All rights reserved.

Version ID

1

Record Owner

From MEDLINE, a database of the U.S. National Library of Medicine.

Status

MEDLINE

Authors Full Name

Ge, Shengjie, Li, Guang, Zhou, Xinyu, Mao, Yu, Gu, Yingyan, Li, Zhiyue, Gu, Yuexing, Cao, Xiaowei

Institution

Ge, Shengjie. Institute of Translational Medicine, Medical College, Yangzhou University, Yangzhou, 225001, PR China; Jiangsu Key Laboratory of Integrated Traditional Chinese and Western Medicine for Prevention and Treatment of Senile Diseases, Yangzhou University, Yangzhou, PR China; Jiangsu Key Laboratory of Experimental & Translational Noncoding RNA Research, Medical College, Yangzhou University, Yangzhou, China. Li, Guang. Department of Otorhinolaryngology-Head and Neck Surgery, The Affiliated Hospital of Yangzhou University, Yangzhou University, Yangzhou, 225001, PR China.

Zhou, Xinyu. Institute of Translational Medicine, Medical College, Yangzhou University, Yangzhou, 225001, PR China; Jiangsu Key Laboratory of Integrated Traditional Chinese and Western Medicine for Prevention and Treatment of Senile Diseases, Yangzhou University, Yangzhou, PR China; Jiangsu Key Laboratory of Experimental & Translational Noncoding RNA Research, Medical College, Yangzhou University, Yangzhou, China.

Mao, Yu. Institute of Translational Medicine, Medical College, Yangzhou University, Yangzhou, 225001, PR China; Jiangsu Key Laboratory of Integrated Traditional Chinese and Western Medicine for Prevention and Treatment of Senile Diseases, Yangzhou University, Yangzhou, PR China; Jiangsu Key Laboratory of Experimental & Translational Noncoding RNA Research, Medical College, Yangzhou University, Yangzhou, China.

Gu, Yingyan. Institute of Translational Medicine, Medical College, Yangzhou University, Yangzhou, 225001, PR China; Jiangsu Key Laboratory of Integrated Traditional Chinese and Western Medicine for Prevention and Treatment of Senile Diseases, Yangzhou University, Yangzhou, PR China; Jiangsu Key Laboratory of Experimental & Translational Noncoding RNA Research, Medical College, Yangzhou University, Yangzhou, China.

Li, Zhiyue. The First Clinical College, Dalian Medical University, Dalian, PR China.

Gu, Yuexing. Institute of Translational Medicine, Medical College, Yangzhou University, Yangzhou, 225001, PR China; Jiangsu Key Laboratory of Integrated Traditional Chinese and Western Medicine for Prevention and Treatment of Senile Diseases, Yangzhou University, Yangzhou, PR China; Jiangsu Key Laboratory of Experimental & Translational Noncoding RNA Research, Medical College, Yangzhou University, Yangzhou, China.

Cao, Xiaowei. Institute of Translational Medicine, Medical College, Yangzhou University, Yangzhou, 225001, PR China; Jiangsu Key Laboratory of Integrated Traditional Chinese and Western Medicine for Prevention and Treatment of Senile Diseases, Yangzhou University, Yangzhou, PR China; Jiangsu Key Laboratory of Experimental & Translational Noncoding RNA Research, Medical College, Yangzhou University, Yangzhou, China. Electronic address: cxw19861121@163.com.

MeSH Heading

Biosensing Techniques/mt [Methods]. *Biosensing Techniques. Gold. *Head and Neck Neoplasms. Humans. Limit of Detection. *Metal Nanoparticles. MicroRNAs/an [Analysis]. *MicroRNAs. Microfluidics. Reproducibility of Results. Spectrum Analysis, Raman/mt [Methods]. Squamous Cell Carcinoma of Head and Neck.

Keyword Heading

Catalytic hairpin assembly Laryngeal squamous cell carcinoma

Microfluidic chip

Surface-enhanced Raman scattering

miRNA.

Keyword Heading Owner

NOTNLM

Registry Number/Name of Substance

0 (MicroRNAs). 7440-57-5 (Gold).

Year of Publication

2022

Link to the Ovid Full Text or citation:

[Click here for full text options](https://ovidsp.ovid.com/ovidweb.cgi?T=JS&CSC=Y&NEWS=N&PAGE=fulltext&D=med22&AN=35436733)

Link to the External Link Resolver:

[SFX](https://sfx-86scu.hosted.exlibrisgroup.com.cn/86scu?sid=OVID:medline&id=pmid:35436733&id=doi:10.1016%2Fj.talanta.2022.123478&issn=00399140&isbn=&volume=245&issue=&spage=123478&pages=123478&date=2022&title=Talanta&atitle=Pump-free+microfluidic+chip+based+laryngeal+squamous+cell+carcinoma-related+microRNAs+detection+through+the+combination+of+surface-enhanced+Raman+scattering+techniques+and+catalytic+hairpin+assembly+amplification.&aulast=Ge&pid=<author>Ge+S%3BLi+G%3BZhou+X%3BMao+Y%3BGu+Y%3BLi+Z%3BGu+Y%3BCao+X<%2Fauthor><AN>35436733<%2FAN><DT>Journal+Article<%2FDT>)

29.

Raman spectroscopy in oral cavity and oropharyngeal cancer: a systematic review. [Review]

Faur CI, Falamas A, Chirila M, Roman RC, Rotaru H, Moldovan MA, Albu S, Baciut M, Robu I, Hedesiu M

International Journal of Oral & Maxillofacial Surgery. 51(11):1373-1381, 2022 Nov.

[Systematic Review. Journal Article. Review]

UI: 35282942

Due to the high incidence of head and neck cancer and under-diagnosis in the early stages, non-invasive and highly accurate diagnostic tests are required for cancer detection. Recent advances in Raman spectroscopy techniques have yielded promising sensitivity and specificity results in the evaluation of cancer. The aim of this study was to investigate the potential value of Raman spectroscopy in oral cavity and oropharyngeal cancer diagnosis based on currently available scientific papers. A search of the PubMed database was performed using a specific strategy and according to the PRISMA guidelines. Raman spectroscopy achieved a maximum accuracy of 98% in cancer detection, while accuracy was 97.24% for tumour grading evaluation, 95% for cancer treatment assessment, and 77% for the detection of cancer recurrence. Moreover, early-stage cancer can be identified by Raman spectroscopy investigation of liquid biopsy samples. An in vivo technique with direct mucosa examination by fibre-optic Raman spectroscopy obtained a maximum accuracy of 94% in cancer diagnosis. The most prominent markers of the presence of malignancy were an increase in Raman signal intensity for proteins, nucleic acids, and water and a decrease for lipids. These cancer discriminants were detected in both fingerprint and high wavenumber regions. In conclusion, Raman spectroscopy is a promising tool for oral cavity and oropharyngeal cancer screening.

Copyright © 2022 International Association of Oral and Maxillofacial Surgeons. Published by Elsevier Inc. All rights reserved.

Version ID

1

Record Owner

From MEDLINE, a database of the U.S. National Library of Medicine.

Status

MEDLINE

Authors Full Name

Faur, C I, Falamas, A, Chirila, M, Roman, R C, Rotaru, H, Moldovan, M A, Albu, S, Baciut, M, Robu, I, Hedesiu, M

Institution

Faur, C I. Department of Oral Radiology, Iuliu Hatieganu University of Medicine and Pharmacy, Cluj-Napoca, Romania. Electronic address: cosmin.faur@yahoo.com. Falamas, A. Department of Molecular and Biomolecular Physics, National Institute for Research and Development of Isotopic and Molecular Technologies, Cluj-Napoca, Romania.

Chirila, M. Department of Otorhinolaryngology, Iuliu Hatieganu University of Medicine and Pharmacy, Cluj-Napoca, Romania.

Roman, R C. Department of Oral and Craniomaxillofacial Surgery, Iuliu Hatieganu University of Medicine and Pharmacy, Cluj-Napoca, Romania.

Rotaru, H. Department of Oral and Craniomaxillofacial Surgery, Iuliu Hatieganu University of Medicine and Pharmacy, Cluj-Napoca, Romania.

Moldovan, M A. Department of Oral and Craniomaxillofacial Surgery, Iuliu Hatieganu University of Medicine and Pharmacy, Cluj-Napoca, Romania.

Albu, S. Department of Oral and Craniomaxillofacial Surgery, Iuliu Hatieganu University of Medicine and Pharmacy, Cluj-Napoca, Romania.

Baciut, M. Department of Maxillofacial Surgery and Implantology, Iuliu Hatieganu University of Medicine and Pharmacy Cluj-Napoca, Romania.

Robu, I. Doctoral School, Iuliu Hatieganu University of Medicine and Pharmacy, Cluj-Napoca, Romania.

Hedesiu, M. Department of Oral Radiology, Iuliu Hatieganu University of Medicine and Pharmacy, Cluj-Napoca, Romania.

MeSH Heading

Humans. Spectrum Analysis, Raman/mt [Methods]. *Spectrum Analysis, Raman. Neoplasm Recurrence, Local. Oropharyngeal Neoplasms/di [Diagnosis]. *Oropharyngeal Neoplasms. Early Detection of Cancer. Mouth.

Keyword Heading

Raman spectroscopy early detection of cancer

head and neck cancer

liquid biopsy

oral cancer.

Keyword Heading Owner

NOTNLM

Year of Publication

2022

Link to the Ovid Full Text or citation:

[Click here for full text options](https://ovidsp.ovid.com/ovidweb.cgi?T=JS&CSC=Y&NEWS=N&PAGE=fulltext&D=med21&AN=35282942)

Link to the External Link Resolver:

[SFX](https://sfx-86scu.hosted.exlibrisgroup.com.cn/86scu?sid=OVID:medline&id=pmid:35282942&id=doi:10.1016%2Fj.ijom.2022.02.015&issn=09015027&isbn=&volume=51&issue=11&spage=1373&pages=1373-1381&date=2022&title=International+Journal+of+Oral+%26+Maxillofacial+Surgery&atitle=Raman+spectroscopy+in+oral+cavity+and+oropharyngeal+cancer%3A+a+systematic+review.&aulast=Faur&pid=<author>Faur+CI%3BFalamas+A%3BChirila+M%3BRoman+RC%3BRotaru+H%3BMoldovan+MA%3BAlbu+S%3BBaciut+M%3BRobu+I%3BHedesiu+M<%2Fauthor><AN>35282942<%2FAN><DT>Systematic+Review<%2FDT>)

30.

Raman Spectroscopy: A Potential Diagnostic Tool for Oral Diseases. [Review]

Zhang Y, Ren L, Wang Q, Wen Z, Liu C, Ding Y

Frontiers in Cellular & Infection Microbiology. 12:775236, 2022.

[Journal Article. Research Support, Non-U.S. Gov't. Review]

UI: 35186787

Oral diseases impose a major health burden worldwide and have a profound effect on general health. Dental caries, periodontal diseases, and oral cancers are the most common oral health conditions. Their occurrence and development are related to oral microbes, and effective measures for their prevention and the promotion of oral health are urgently needed. Raman spectroscopy detects molecular vibration information by collecting inelastic scattering light, allowing a "fingerprint" of a sample to be acquired. It provides the advantages of rapid, sensitive, accurate, and minimally invasive detection as well as minimal interference from water in the "fingerprint region." Owing to these characteristics, Raman spectroscopy has been used in medical detection in various fields to assist diagnosis and evaluate prognosis, such as detecting and differentiating between bacteria or between neoplastic and normal brain tissues. Many oral diseases are related to oral microbial dysbiosis, and their lesions differ from normal tissues in essential components. The colonization of keystone pathogens, such as Porphyromonas gingivalis, resulting in microbial dysbiosis in subgingival plaque, is the main cause of periodontitis. Moreover, the components in gingival crevicular fluid, such as infiltrating inflammatory cells and tissue degradation products, are markedly different between individuals with and without periodontitis. Regarding dental caries, the compositions of decayed teeth are transformed, accompanied by an increase in acid-producing bacteria. In oral cancers, the compositions and structures of lesions and normal tissues are different. Thus, the changes in bacteria and the components of saliva and tissue can be used in examinations as special markers for these oral diseases, and Raman spectroscopy has been acknowledged as a promising measure for detecting these markers. This review summarizes and discusses key research and remaining problems in this area. Based on this, suggestions for further study are proposed.

Copyright © 2022 Zhang, Ren, Wang, Wen, Liu and Ding.

Version ID

1

Record Owner

From MEDLINE, a database of the U.S. National Library of Medicine.

Status

MEDLINE

Authors Full Name

Zhang, Yuwei, Ren, Liang, Wang, Qi, Wen, Zhining, Liu, Chengcheng, Ding, Yi

Institution

Zhang, Yuwei. State Key Laboratory of Oral Diseases, National Clinical Research Center for Oral Diseases, Department of Periodontics, West China Hospital of Stomatology, Sichuan University, Chengdu, China. Ren, Liang. State Key Laboratory of Oral Diseases, National Clinical Research Center for Oral Diseases, Department of Periodontics, West China Hospital of Stomatology, Sichuan University, Chengdu, China.

Wang, Qi. State Key Laboratory of Oral Diseases, National Clinical Research Center for Oral Diseases, Department of Prosthodontics, West China Hospital of Stomatology, Sichuan University, Chengdu, China.

Wen, Zhining. College of Chemistry, Sichuan University, Chengdu, China.

Liu, Chengcheng. State Key Laboratory of Oral Diseases, National Clinical Research Center for Oral Diseases, Department of Periodontics, West China Hospital of Stomatology, Sichuan University, Chengdu, China.

Ding, Yi. State Key Laboratory of Oral Diseases, National Clinical Research Center for Oral Diseases, Department of Periodontics, West China Hospital of Stomatology, Sichuan University, Chengdu, China.

MeSH Heading

Dental Caries/di [Diagnosis]. *Dental Caries. Dysbiosis/mi [Microbiology]. Humans. Periodontitis/mi [Microbiology]. *Periodontitis. Porphyromonas gingivalis. Spectrum Analysis, Raman.

Keyword Heading

Raman spectroscopy dental caries

oral cancer

oral microbiota

periodontitis.

Keyword Heading Owner

NOTNLM

Year of Publication

2022

Link to the Ovid Full Text or citation:

[Click here for full text options](https://ovidsp.ovid.com/ovidweb.cgi?T=JS&CSC=Y&NEWS=N&PAGE=fulltext&D=med21&AN=35186787)

Link to the External Link Resolver:

[SFX](https://sfx-86scu.hosted.exlibrisgroup.com.cn/86scu?sid=OVID:medline&id=pmid:35186787&id=doi:10.3389%2Ffcimb.2022.775236&issn=22352988&isbn=&volume=12&issue=&spage=775236&pages=775236&date=2022&title=Frontiers+in+Cellular+%26+Infection+Microbiology&atitle=Raman+Spectroscopy%3A+A+Potential+Diagnostic+Tool+for+Oral+Diseases.&aulast=Zhang&pid=<author>Zhang+Y%3BRen+L%3BWang+Q%3BWen+Z%3BLiu+C%3BDing+Y<%2Fauthor><AN>35186787<%2FAN><DT>Journal+Article<%2FDT>)

31.

Rapid identification of papillary thyroid carcinoma and papillary microcarcinoma based on serum Raman spectroscopy combined with machine learning models.

Song H, Dong C, Zhang X, Wu W, Chen C, Ma B, Chen F, Chen C, Lv X

Photodiagnosis & Photodynamic Therapy. 37:102647, 2022 Mar.

[Journal Article]

UI: 34818598

Thyroid carcinoma is with the highest diagnosis rate in the endocrine system, and its main histological subtype is papillary thyroid carcinoma (PTC) accounting for 80% of thyroid malignancies. In recent years, the incidence of thyroid cancer has increased exponentially, and its substantial increase was closely related to the overdiagnosis of papillary microcarcinoma (PMC). Therefore, early and accurate identification of PTC and PMC can prevent patients from over treatment. This study aimed to identify PTC and PMC using Raman spectroscopy. We collected serum Raman spectra from 16 patients with PTC and 31 patients with PMC. Firstly, the collected imbalance data were preprocessed using the synthetic minority over-sampling technique (SMOTE). Then, the equalized data were dimensionality reduced by principal component analysis (PCA). Finally, the processed data were fed into the single decision tree (DT) classifier, as well as the random forest (RF) built on the idea of Boosting ensemble and the Adaptive Boosting (Adaboost) model built on the idea of Bagging ensemble for classification. The classification accuracy of the three models in the testing set were 75.38%, 81.54%, and 84.61%, respectively. Compared with the DT classifier, the accuracy of the models introducing the idea of ensemble learning was enhanced by 6.16% and 9.23%, respectively. The best model was the Adaboost. This result demonstrates that serum Raman spectroscopy combined with an ensemble learning algorithm was feasible in rapidly identifying PTC and PMC. At the same time, the method has great potential for application in the field of clinical diagnosis.

Copyright © 2021 Elsevier B.V. All rights reserved.

Version ID

1

Record Owner

From MEDLINE, a database of the U.S. National Library of Medicine.

Status

MEDLINE

Authors Full Name

Song, Haitao, Dong, Chao, Zhang, Xudan, Wu, Wei, Chen, Cheng, Ma, Binlin, Chen, Fangfang, Chen, Chen, Lv, Xiaoyi

Institution

Song, Haitao. Department of Breast, Head and Neck Surgery, Xinjiang Medical University Affiliated Tumor Hospital, Urumqi, China. Dong, Chao. Department of Breast, Head and Neck Surgery, Xinjiang Medical University Affiliated Tumor Hospital, Urumqi, China.

Zhang, Xudan. College of Information Science and Engineering, Xinjiang University, Urumqi 830046, China.

Wu, Wei. College of Software, Xinjiang University, Urumqi 830046, China.

Chen, Cheng. College of Software, Xinjiang University, Urumqi 830046, China; Key Laboratory of Signal Detection and Processing, Xinjiang University, Urumqi 830046, China.

Ma, Binlin. Department of Breast, Head and Neck Surgery, Xinjiang Medical University Affiliated Tumor Hospital, Urumqi, China. Electronic address: mbldoctor@126.com.

Chen, Fangfang. College of Information Science and Engineering, Xinjiang University, Urumqi 830046, China.

Chen, Chen. College of Information Science and Engineering, Xinjiang University, Urumqi 830046, China.

Lv, Xiaoyi. College of Software, Xinjiang University, Urumqi 830046, China; Key Laboratory of Signal Detection and Processing, Xinjiang University, Urumqi 830046, China; College of Information Science and Engineering, Xinjiang University, Urumqi 830046, China.

MeSH Heading

Humans. Machine Learning. Photochemotherapy/mt [Methods]. *Photochemotherapy. Spectrum Analysis, Raman. Thyroid Cancer, Papillary/di [Diagnosis]. Thyroid Neoplasms/di [Diagnosis]. Thyroid Neoplasms/pa [Pathology]. *Thyroid Neoplasms.

Keyword Heading

Ensemble learning Papillary microcarcinoma (PMC)

Papillary thyroid carcinoma (PTC)

Raman spectroscopy

Serum.

Keyword Heading Owner

NOTNLM

Year of Publication

2022

Link to the Ovid Full Text or citation:

[Click here for full text options](https://ovidsp.ovid.com/ovidweb.cgi?T=JS&CSC=Y&NEWS=N&PAGE=fulltext&D=med21&AN=34818598)

Link to the External Link Resolver:

[SFX](https://sfx-86scu.hosted.exlibrisgroup.com.cn/86scu?sid=OVID:medline&id=pmid:34818598&id=doi:10.1016%2Fj.pdpdt.2021.102647&issn=15721000&isbn=&volume=37&issue=&spage=102647&pages=102647&date=2022&title=Photodiagnosis+%26+Photodynamic+Therapy&atitle=Rapid+identification+of+papillary+thyroid+carcinoma+and+papillary+microcarcinoma+based+on+serum+Raman+spectroscopy+combined+with+machine+learning+models.&aulast=Song&pid=<author>Song+H%3BDong+C%3BZhang+X%3BWu+W%3BChen+C%3BMa+B%3BChen+F%3BChen+C%3BLv+X<%2Fauthor><AN>34818598<%2FAN><DT>Journal+Article<%2FDT>)

32.

Classification of cytological samples from oral potentially malignant lesions through Raman spectroscopy: A pilot study.

Behl I, Calado G, Vishwakarma A, Traynor D, Flint S, Galvin S, Healy CM, Pimentel ML, Malkin A, Byrne HJ, Lyng FM

Spectrochimica Acta. Part A, Molecular & Biomolecular Spectroscopy. 266:120437, 2022 Feb 05.

[Journal Article]

UI: 34627019

The potential of Raman microspectroscopy of exfoliated cells has been demonstrated for oral cancer diagnosis. In this study, brush biopsies were collected from the buccal mucosa/tongue of healthy donors (n=31) and from oral mucosal dysplastic lesions (n=31 patients). Raman spectra were acquired and subjected to partial least squares-discriminant analysis (PLS-DA). The patient samples could be differentiated from healthy donor samples with 96% sensitivity and 95% specificity. Furthermore, PLS-DA models were developed based on cytopathological and histopathological assessment. Low and high grade dysplasia could be discriminated with 64% sensitivity and 65% specificity based on cytopathological assessment, while 81% sensitivity and 86% specificity could be achieved when histopathological assessment was within six months of the brush biopsy sampling. Therefore, this explorative study has successfully demonstrated that Raman spectroscopy may have a role in monitoring patients with dysplasia and may reduce the need for multiple biopsies.

Copyright © 2021 Elsevier B.V. All rights reserved.

Version ID

1

Record Owner

From MEDLINE, a database of the U.S. National Library of Medicine.

Status

MEDLINE

Authors Full Name

Behl, Isha, Calado, Genecy, Vishwakarma, Anika, Traynor, Damien, Flint, Stephen, Galvin, Sheila, Healy, Claire M, Pimentel, Marina Leite, Malkin, Alison, Byrne, Hugh J, Lyng, Fiona M

Institution

Behl, Isha. Centre for Radiation and Environmental Science, FOCAS Research Institute, Technological University Dublin, City Campus, Dublin, Ireland; School of Physics & Clinical & Optometric Sciences, Technological University Dublin, City Campus, Dublin, Ireland. Electronic address: behlisha86@gmail.com. Calado, Genecy. Centre for Radiation and Environmental Science, FOCAS Research Institute, Technological University Dublin, City Campus, Dublin, Ireland; School of Physics & Clinical & Optometric Sciences, Technological University Dublin, City Campus, Dublin, Ireland.

Vishwakarma, Anika. Centre for Radiation and Environmental Science, FOCAS Research Institute, Technological University Dublin, City Campus, Dublin, Ireland; School of Physics & Clinical & Optometric Sciences, Technological University Dublin, City Campus, Dublin, Ireland.

Traynor, Damien. Centre for Radiation and Environmental Science, FOCAS Research Institute, Technological University Dublin, City Campus, Dublin, Ireland; School of Physics & Clinical & Optometric Sciences, Technological University Dublin, City Campus, Dublin, Ireland.

Flint, Stephen. Oral Medicine Unit, Dublin Dental University Hospital, Trinity College Dublin, Ireland.

Galvin, Sheila. Oral Medicine Unit, Dublin Dental University Hospital, Trinity College Dublin, Ireland.

Healy, Claire M. Oral Medicine Unit, Dublin Dental University Hospital, Trinity College Dublin, Ireland.

Pimentel, Marina Leite. Division of Restorative Dentistry and Periodontology, Dublin Dental University Hospital, Trinity College Dublin, Dublin, Ireland.

Malkin, Alison. School of Biological and Health Sciences, Technological University Dublin, City Campus, Dublin, Ireland.

Byrne, Hugh J. FOCAS Research Institute, Technological University Dublin, City Campus, Dublin, Ireland.

Lyng, Fiona M. Centre for Radiation and Environmental Science, FOCAS Research Institute, Technological University Dublin, City Campus, Dublin, Ireland; School of Physics & Clinical & Optometric Sciences, Technological University Dublin, City Campus, Dublin, Ireland.

MeSH Heading

Discriminant Analysis. Humans. Least-Squares Analysis. Mouth Neoplasms/di [Diagnosis]. *Mouth Neoplasms. Pilot Projects. Sensitivity and Specificity. *Spectrum Analysis, Raman.

Keyword Heading

Binary grading system Brush biopsy

Cytological samples

High grade dysplasia

Low grade dysplasia

Oral potentially malignant lesions

Raman microspectroscopy

Sensitivity

Specificity.

Keyword Heading Owner

NOTNLM

Year of Publication

2022

Link to the Ovid Full Text or citation:

[Click here for full text options](https://ovidsp.ovid.com/ovidweb.cgi?T=JS&CSC=Y&NEWS=N&PAGE=fulltext&D=med21&AN=34627019)

Link to the External Link Resolver:

[SFX](https://sfx-86scu.hosted.exlibrisgroup.com.cn/86scu?sid=OVID:medline&id=pmid:34627019&id=doi:10.1016%2Fj.saa.2021.120437&issn=13861425&isbn=&volume=266&issue=&spage=120437&pages=120437&date=2022&title=Spectrochimica+Acta.+Part+A%2C+Molecular+%26+Biomolecular+Spectroscopy&atitle=Classification+of+cytological+samples+from+oral+potentially+malignant+lesions+through+Raman+spectroscopy%3A+A+pilot+study.&aulast=Behl&pid=<author>Behl+I%3BCalado+G%3BVishwakarma+A%3BTraynor+D%3BFlint+S%3BGalvin+S%3BHealy+CM%3BPimentel+ML%3BMalkin+A%3BByrne+HJ%3BLyng+FM<%2Fauthor><AN>34627019<%2FAN><DT>Journal+Article<%2FDT>)

33.

SERS Based Lateral Flow Assay for Rapid and Ultrasensitive Quantification of Dual Laryngeal Squamous Cell Carcinoma-Related miRNA Biomarkers in Human Serum Using Pd-Au Core-Shell Nanorods and Catalytic Hairpin Assembly.

Li G, Niu P, Ge S, Cao D, Sun A

Frontiers in Molecular Biosciences. 8:813007, 2021.

[Journal Article]

UI: 35223986

Non-invasive early diagnosis is of great significant in disease pathologic development and subsequent medical treatments, and microRNA (miRNA) detection has attracted critical attention in early cancer screening and diagnosis. However, it was still a challenge to report an accurate and sensitive method for the detection of miRNA during cancer development, especially in the presence of its analogs that produce intense background noise. Herein, we developed a surface-enhanced Raman scattering (SERS)-based lateral flow assay (LFA) biosensor, assisted with catalytic hairpin assembly (CHA) amplification strategy, for the dynamic monitoring of miR-106b and miR-196b, associated with laryngeal squamous cell carcinoma (LSCC). In the presence of target miRNAs, two hairpin DNAs could self-assemble into double-stranded DNA, exposing the biotin molecules modified on the surface of palladium (Pd)-gold (Au) core-shell nanorods (Pd-AuNRs). Then, the biotin molecules could be captured by the streptavidin (SA), which was fixed on the test lines (T1 line and T2 line) beforehand. The core-shell spatial structures and aggregation Pd-AuNRs generated abundant active "hot spots" on the T line, significantly amplifying the SERS signals. Using this strategy, the limits of detections were low to aM level, and the selectivity, reproducibility, and uniformity of the proposed SERS-LFA biosensor were satisfactory. Finally, this rapid analysis strategy was successfully applied to quantitatively detect the target miRNAs in clinical serum obtained from healthy subjects and patients with LSCC at different stages. The results were consistent with the quantitative real-time PCR (qRT-PCR). Thus, the CHA-assisted SERS-LFA biosensor would become a promising alternative tool for miRNAs detection, which showed a tremendous clinical application prospect in diagnosing LSCC.

Copyright © 2022 Li, Niu, Ge, Cao and Sun.

Version ID

1

Record Owner

From MEDLINE, a database of the U.S. National Library of Medicine.

Status

PubMed-not-MEDLINE

Authors Full Name

Li, Guang, Niu, Ping, Ge, Shengjie, Cao, Dawei, Sun, Aidong

Institution

Li, Guang. Department of Otorhinolaryngology-Head and Neck Surgery, The Affiliated Hospital of Yangzhou University, Yangzhou University, Yangzhou, China. Niu, Ping. Departments of Otolaryngology, The Affiliated Hospital of Shandong First Medical University, Qingzhou People's Hospital, Qingzhou, China.

Ge, Shengjie. Institute of Translational Medicine, Medical College, Yangzhou University, Yangzhou, China.

Cao, Dawei. College of Mathematics and Computer Science, Zhejiang Normal University, Jinhua, China.

Sun, Aidong. Department of Otorhinolaryngology-Head and Neck Surgery, The Affiliated Hospital of Yangzhou University, Yangzhou University, Yangzhou, China.

Keyword Heading

Pd-Au core-shell nanorods catalytic hairpin assembly

laryngeal squamous cell carcinoma

lateral flow assay

miRNA

surface-enhanced Raman scattering.

Keyword Heading Owner

NOTNLM

Year of Publication

2021

Link to the Ovid Full Text or citation:

[Click here for full text options](https://ovidsp.ovid.com/ovidweb.cgi?T=JS&CSC=Y&NEWS=N&PAGE=fulltext&D=pmnm6&AN=35223986)

Link to the External Link Resolver:

[SFX](https://sfx-86scu.hosted.exlibrisgroup.com.cn/86scu?sid=OVID:medline&id=pmid:35223986&id=doi:10.3389%2Ffmolb.2021.813007&issn=2296889X&isbn=&volume=8&issue=&spage=813007&pages=813007&date=2021&title=Frontiers+in+Molecular+Biosciences&atitle=SERS+Based+Lateral+Flow+Assay+for+Rapid+and+Ultrasensitive+Quantification+of+Dual+Laryngeal+Squamous+Cell+Carcinoma-Related+miRNA+Biomarkers+in+Human+Serum+Using+Pd-Au+Core-Shell+Nanorods+and+Catalytic+Hairpin+Assembly.&aulast=Li&pid=<author>Li+G%3BNiu+P%3BGe+S%3BCao+D%3BSun+A<%2Fauthor><AN>35223986<%2FAN><DT>Journal+Article<%2FDT>)

34.

Developing an Algorithm for Discriminating Oral Cancerous and Normal Tissues Using Raman Spectroscopy.

Sharma M, Jeng MJ, Young CK, Huang SF, Chang LB

Journal of Personalized Medicine. 11(11), 2021 Nov 09.

[Journal Article]

UI: 34834517

The aim of this study was to investigate the clinical potential of Raman spectroscopy (RS) in detecting oral squamous cell carcinoma (OSCC) in tumor and healthy tissues in surgical resection specimens during surgery. Raman experiments were performed on cryopreserved specimens from patients with OSCC. Univariate and multivariate analysis was performed based on the fingerprint region (700-1800 cm-1) of the Raman spectra. One hundred thirty-one ex-vivo Raman experiments were performed on 131 surgical resection specimens obtained from 67 patients. The principal component analysis (PCA) and partial least square (PLS) methods with linear discriminant analysis (LDA) were applied on an independent validation dataset. Both models were able to differentiate between the tissue types, but PLS-LDA showed 100% accuracy, sensitivity, and specificity. In this study, Raman measurements of fresh resection tissue specimens demonstrated that OSCC had significantly higher nucleic acid, protein, and several amino acid contents than adjacent healthy tissues. The specific spectral information obtained in this study can be used to develop an in vivo Raman spectroscopic method for the tumor-free resection boundary during surgery.

Version ID

1

Record Owner

From MEDLINE, a database of the U.S. National Library of Medicine.

Status

PubMed-not-MEDLINE

Author NameID

Jeng, Ming-Jer; ORCID: <https://orcid.org/0000-0001-6130-1980> Huang, Shiang-Fu; ORCID: <https://orcid.org/0000-0003-3582-9938>

Chang, Liann-Be; ORCID: <https://orcid.org/0000-0003-2174-2022>

Authors Full Name

Sharma, Mukta, Jeng, Ming-Jer, Young, Chi-Kuang, Huang, Shiang-Fu, Chang, Liann-Be

Institution

Sharma, Mukta. Department of Electronic Engineering, Chang Gung University, Taoyuan 333, Taiwan. Jeng, Ming-Jer. Department of Electronic Engineering, Chang Gung University, Taoyuan 333, Taiwan.

Jeng, Ming-Jer. Department of Otolaryngology-Head and Neck Surgery, Chang Gung Memorial Hospital, Linkou 244, Taiwan.

Young, Chi-Kuang. Department of Otolaryngology, Head and Neck Surgery, Chang Gung Memorial Hospital, Keelung Branch, Keelung 204, Taiwan.

Huang, Shiang-Fu. Department of Otolaryngology-Head and Neck Surgery, Chang Gung Memorial Hospital, Linkou 244, Taiwan.

Huang, Shiang-Fu. Department of Public Health, Chang Gung University, Taoyuan 333, Taiwan.

Chang, Liann-Be. Department of Electronic Engineering, Chang Gung University, Taoyuan 333, Taiwan.

Chang, Liann-Be. Department of Otolaryngology-Head and Neck Surgery, Chang Gung Memorial Hospital, Linkou 244, Taiwan.

Chang, Liann-Be. Green Technology Research Center, Chang Gung University, Taoyuan 333, Taiwan.

Keyword Heading

PCA-LDA PLS-LDA

Raman spectroscopy

cryopreserved tissue

oral cancer.

Keyword Heading Owner

NOTNLM

Year of Publication

2021

Link to the Ovid Full Text or citation:

[Click here for full text options](https://ovidsp.ovid.com/ovidweb.cgi?T=JS&CSC=Y&NEWS=N&PAGE=fulltext&D=pmnm6&AN=34834517)

Link to the External Link Resolver:

[SFX](https://sfx-86scu.hosted.exlibrisgroup.com.cn/86scu?sid=OVID:medline&id=pmid:34834517&id=doi:10.3390%2Fjpm11111165&issn=20754426&isbn=&volume=11&issue=11&spage=&pages=&date=2021&title=Journal+of+Personalized+Medicine&atitle=Developing+an+Algorithm+for+Discriminating+Oral+Cancerous+and+Normal+Tissues+Using+Raman+Spectroscopy.&aulast=Sharma&pid=<author>Sharma+M%3BJeng+MJ%3BYoung+CK%3BHuang+SF%3BChang+LB<%2Fauthor><AN>34834517<%2FAN><DT>Journal+Article<%2FDT>)

35.

Brachytherapy Approach Using 177Lu Conjugated Gold Nanostars and Evaluation of Biodistribution, Tumor Retention, Dosimetry and Therapeutic Efficacy in Head and Neck Tumor Model.

Lin MY, Hsieh HH, Chen JC, Chen CL, Sheu NC, Huang WS, Ho SY, Chen TW, Lee YJ, Wu CY

Pharmaceutics. 13(11), 2021 Nov 09.

[Journal Article]

UI: 34834318

Brachytherapy can provide sufficient doses to head and neck squamous cell carcinoma (HNSCC) with minimal damage to nearby normal tissues. In this study, the beta--emitter 177Lu was conjugated to DTPA-polyethylene glycol (PEG) decorated gold nanostars (177Lu-DTPA-pAuNS) used in surface-enhanced Raman scattering and photothermal therapy (PTT). The accumulation and therapeutic efficacy of 177Lu-DTPA-pAuNS were compared with those of 177Lu-DTPA on an orthotopic HNSCC tumor model. The SPECT/CT imaging and biodistribution studies showed that 177Lu-DTPA-pAuNS can be accumulated in the tumor up to 15 days, but 177Lu-DTPA could not be detected at 24 h after injection. The tumor viability and growth were suppressed by injected 177Lu-DTPA-pAuNS but not nonconjugated 177Lu-DTPA, as evaluated by bioluminescent imaging. The radiation-absorbed dose of the normal organ was the highest in the liver (0.33 mSv/MBq) estimated in a 73 kg adult, but that of tumorsphere (0.5 g) was 3.55 mGy/MBq, while intravenous injection of 177Lu-DTPA-pAuNS resulted in 1.97 mSv/MBq and 0.13 mGy/MBq for liver and tumorsphere, respectively. We also observed further enhancement of tumor-suppressive effects by a combination of 177Lu-DTPA-pAuNS and PTT compared to 177Lu-DTPA-pAuNS alone. In conclusion, 177Lu-DTPA-pAuNS may be considered as a potential radiopharmaceutical agent for HNSCC brachytherapy.

Version ID

1

Record Owner

From MEDLINE, a database of the U.S. National Library of Medicine.

Status

PubMed-not-MEDLINE

Author NameID

Lin, Min-Ying; ORCID: <https://orcid.org/0000-0002-2363-7085> Chen, Jyh-Cheng; ORCID: <https://orcid.org/0000-0003-3619-1358>

Chen, Chuan-Lin; ORCID: <https://orcid.org/0000-0002-6295-1034>

Chen, Ting-Wen; ORCID: <https://orcid.org/0000-0003-4028-2375>

Lee, Yi-Jang; ORCID: <https://orcid.org/0000-0002-0340-7557>

Wu, Chun-Yi; ORCID: <https://orcid.org/0000-0002-8217-1692>

Authors Full Name

Lin, Min-Ying, Hsieh, Hsin-Hua, Chen, Jyh-Cheng, Chen, Chuan-Lin, Sheu, Nin-Chu, Huang, Wen-Sheng, Ho, Shinn-Ying, Chen, Ting-Wen, Lee, Yi-Jang, Wu, Chun-Yi

Institution

Lin, Min-Ying. Department of Biomedical Imaging and Radiological Sciences, National Yang Ming Chiao Tung University, Taipei Branch, Taipei 112, Taiwan. Hsieh, Hsin-Hua. Department of Biomedical Imaging and Radiological Sciences, National Yang Ming Chiao Tung University, Taipei Branch, Taipei 112, Taiwan.

Chen, Jyh-Cheng. Department of Biomedical Imaging and Radiological Sciences, National Yang Ming Chiao Tung University, Taipei Branch, Taipei 112, Taiwan.

Chen, Chuan-Lin. Department of Biomedical Imaging and Radiological Sciences, National Yang Ming Chiao Tung University, Taipei Branch, Taipei 112, Taiwan.

Sheu, Nin-Chu. Department of Biomedical Imaging and Radiological Sciences, National Yang Ming Chiao Tung University, Taipei Branch, Taipei 112, Taiwan.

Huang, Wen-Sheng. Department of Nuclear Medicine, Taipei Medical University Hospital, Taipei 11031, Taiwan.

Ho, Shinn-Ying. Institute of Bioinformatics and Systems Biology, National Yang Ming Chiao Tung University, Hsinchu Branch, Hsinchu 30068, Taiwan.

Chen, Ting-Wen. Institute of Bioinformatics and Systems Biology, National Yang Ming Chiao Tung University, Hsinchu Branch, Hsinchu 30068, Taiwan.

Chen, Ting-Wen. Department of Biological Science and Technology, National Yang Ming Chiao Tung University, Hsinchu Branch, Hsinchu 30068, Taiwan.

Lee, Yi-Jang. Department of Biomedical Imaging and Radiological Sciences, National Yang Ming Chiao Tung University, Taipei Branch, Taipei 112, Taiwan.

Lee, Yi-Jang. Cancer Progression Research Center, National Yang Ming Chiao Tung University, Taipei Branch, Taipei 112, Taiwan.

Wu, Chun-Yi. Department of Biomedical Imaging and Radiological Sciences, National Yang Ming Chiao Tung University, Taipei Branch, Taipei 112, Taiwan.

Keyword Heading

177Lu-DTPA-pAuNS brachytherapy

head and neck cancer

photothermal therapy.

Keyword Heading Owner

NOTNLM

Year of Publication

2021

Link to the Ovid Full Text or citation:

[Click here for full text options](https://ovidsp.ovid.com/ovidweb.cgi?T=JS&CSC=Y&NEWS=N&PAGE=fulltext&D=pmnm6&AN=34834318)

Link to the External Link Resolver:

[SFX](https://sfx-86scu.hosted.exlibrisgroup.com.cn/86scu?sid=OVID:medline&id=pmid:34834318&id=doi:10.3390%2Fpharmaceutics13111903&issn=19994923&isbn=&volume=13&issue=11&spage=&pages=&date=2021&title=Pharmaceutics&atitle=Brachytherapy+Approach+Using+177Lu+Conjugated+Gold+Nanostars+and+Evaluation+of+Biodistribution%2C+Tumor+Retention%2C+Dosimetry+and+Therapeutic+Efficacy+in+Head+and+Neck+Tumor+Model.&aulast=Lin&pid=<author>Lin+MY%3BHsieh+HH%3BChen+JC%3BChen+CL%3BSheu+NC%3BHuang+WS%3BHo+SY%3BChen+TW%3BLee+YJ%3BWu+CY<%2Fauthor><AN>34834318<%2FAN><DT>Journal+Article<%2FDT>)

36.

A New Look into Cancer-A Review on the Contribution of Vibrational Spectroscopy on Early Diagnosis and Surgery Guidance. [Review]

Mamede AP, Santos IP, Batista de Carvalho ALM, Figueiredo P, Silva MC, Tavares MV, Marques MPM, Batista de Carvalho LAE

Cancers. 13(21), 2021 Oct 24.

[Journal Article. Review]

UI: 34771500

In 2020, approximately 10 million people died of cancer, rendering this disease the second leading cause of death worldwide. Detecting cancer in its early stages is paramount for patients' prognosis and survival. Hence, the scientific and medical communities are engaged in improving both therapeutic strategies and diagnostic methodologies, beyond prevention. Optical vibrational spectroscopy has been shown to be an ideal diagnostic method for early cancer diagnosis and surgical margins assessment, as a complement to histopathological analysis. Being highly sensitive, non-invasive and capable of real-time molecular imaging, Raman and Fourier transform infrared (FTIR) spectroscopies give information on the biochemical profile of the tissue under analysis, detecting the metabolic differences between healthy and cancerous portions of the same sample. This constitutes tremendous progress in the field, since the cancer-prompted morphological alterations often occur after the biochemical imbalances in the oncogenic process. Therefore, the early cancer-associated metabolic changes are unnoticed by the histopathologist. Additionally, Raman and FTIR spectroscopies significantly reduce the subjectivity linked to cancer diagnosis. This review focuses on breast and head and neck cancers, their clinical needs and the progress made to date using vibrational spectroscopy as a diagnostic technique prior to surgical intervention and intraoperative margin assessment.

Version ID

1

Record Owner

From MEDLINE, a database of the U.S. National Library of Medicine.

Status

PubMed-not-MEDLINE

Author NameID

Mamede, Adriana P; ORCID: <https://orcid.org/0000-0002-0647-4771> Santos, Ines P; ORCID: <https://orcid.org/0000-0003-2463-246X>

Batista de Carvalho, Ana L M; ORCID: <https://orcid.org/0000-0003-1280-3321>

Marques, Maria P M; ORCID: <https://orcid.org/0000-0002-8391-0055>

Batista de Carvalho, Luis A E; ORCID: <https://orcid.org/0000-0002-8059-8537>

Authors Full Name

Mamede, Adriana P, Santos, Ines P, Batista de Carvalho, Ana L M, Figueiredo, Paulo, Silva, Maria C, Tavares, Mariana V, Marques, Maria P M, Batista de Carvalho, Luis A E

Institution

Mamede, Adriana P. "Unidade de I&D Quimica-Fisica Molecular" (QFM-UC), Departament of Chemistry, University of Coimbra, 3004-504 Coimbra, Portugal. Santos, Ines P. "Unidade de I&D Quimica-Fisica Molecular" (QFM-UC), Departament of Chemistry, University of Coimbra, 3004-504 Coimbra, Portugal.

Batista de Carvalho, Ana L M. "Unidade de I&D Quimica-Fisica Molecular" (QFM-UC), Departament of Chemistry, University of Coimbra, 3004-504 Coimbra, Portugal.

Figueiredo, Paulo. Pathology Department, Portuguese Institute of Oncology Francisco Gentil (IPOFG), 3000-075 Coimbra, Portugal.

Silva, Maria C. Surgery Department, Portuguese Institute of Oncology Francisco Gentil (IPOFG), 3000-075 Coimbra, Portugal.

Tavares, Mariana V. "Unidade de I&D Quimica-Fisica Molecular" (QFM-UC), Departament of Chemistry, University of Coimbra, 3004-504 Coimbra, Portugal.

Tavares, Mariana V. Gynaecology Department, Portuguese Institute of Oncology Francisco Gentil (IPOFG), 4200-072 Porto, Portugal.

Marques, Maria P M. "Unidade de I&D Quimica-Fisica Molecular" (QFM-UC), Departament of Chemistry, University of Coimbra, 3004-504 Coimbra, Portugal.

Marques, Maria P M. Department of Life Sciences, University of Coimbra, 3000-456 Coimbra, Portugal.

Batista de Carvalho, Luis A E. "Unidade de I&D Quimica-Fisica Molecular" (QFM-UC), Departament of Chemistry, University of Coimbra, 3004-504 Coimbra, Portugal.

Keyword Heading

FTIR spectroscopy Raman spectroscopy

alternative and complementary methodologies

breast cancer

diagnosis

head and neck cancer

surgical margins assessment.

Keyword Heading Owner

NOTNLM

Year of Publication

2021

Link to the Ovid Full Text or citation:

[Click here for full text options](https://ovidsp.ovid.com/ovidweb.cgi?T=JS&CSC=Y&NEWS=N&PAGE=fulltext&D=pmnm6&AN=34771500)

Link to the External Link Resolver:

[SFX](https://sfx-86scu.hosted.exlibrisgroup.com.cn/86scu?sid=OVID:medline&id=pmid:34771500&id=doi:10.3390%2Fcancers13215336&issn=20726694&isbn=&volume=13&issue=21&spage=&pages=&date=2021&title=Cancers&atitle=A+New+Look+into+Cancer-A+Review+on+the+Contribution+of+Vibrational+Spectroscopy+on+Early+Diagnosis+and+Surgery+Guidance.&aulast=Mamede&pid=<author>Mamede+AP%3BSantos+IP%3BBatista+de+Carvalho+ALM%3BFigueiredo+P%3BSilva+MC%3BTavares+MV%3BMarques+MPM%3BBatista+de+Carvalho+LAE<%2Fauthor><AN>34771500<%2FAN><DT>Journal+Article<%2FDT>)

37.

Erratum: Assessment of radiotherapy effect for nasopharyngeal cancer using plasma surface-enhanced Raman spectroscopy technology: errata.

Wu Q, Qiu S, Yu Y, Chen W, Lin H, Lin D, Feng S, Chen R

Biomedical Optics Express. 12(5):2557-2558, 2021 May 01.

[Published Erratum]

UI: 34123487

[This corrects the article on p. 3413 in vol. 9, PMID: 29984106.].

Copyright © 2021 Optical Society of America under the terms of the OSA Open Access Publishing Agreement.

Version ID

1

Record Owner

From MEDLINE, a database of the U.S. National Library of Medicine.

Status

PubMed-not-MEDLINE

Author NameID

Yu, Yun; ORCID: <https://orcid.org/0000-0002-2875-1928> Lin, Huijing; ORCID: <https://orcid.org/0000-0001-6959-5995>

Authors Full Name

Wu, Qiong, Qiu, Sufang, Yu, Yun, Chen, Weiwei, Lin, Huijing, Lin, Duo, Feng, Shangyuan, Chen, Rong

Institution

Wu, Qiong. Fujian Normal University, Key Laboratory of OptoElectronic Science and Technology for Medicine, Ministry of Education, Fujian Provincial Key Laboratory for Photonics Technology, Fuzhou, 350007, China. Wu, Qiong. These authors contributed equally to this work.

Qiu, Sufang. Department of Radiation Oncology, Fujian Provincial Cancer Hospital; Fujian Medical University Cancer Hospital; Fujian Provincial Key Laboratory of Translational Cancer Medicine, Fuzhou, 350014, China.

Qiu, Sufang. These authors contributed equally to this work.

Yu, Yun. Fujian Normal University, Key Laboratory of OptoElectronic Science and Technology for Medicine, Ministry of Education, Fujian Provincial Key Laboratory for Photonics Technology, Fuzhou, 350007, China.

Yu, Yun. College of Integrated Traditional Chinese and Western Medicine, Fujian University of Traditional Chinese Medicine, Fuzhou, 350122, China.

Chen, Weiwei. College of Integrated Traditional Chinese and Western Medicine, Fujian University of Traditional Chinese Medicine, Fuzhou, 350122, China.

Lin, Huijing. Fujian Normal University, Key Laboratory of OptoElectronic Science and Technology for Medicine, Ministry of Education, Fujian Provincial Key Laboratory for Photonics Technology, Fuzhou, 350007, China.

Lin, Duo. Fujian Normal University, Key Laboratory of OptoElectronic Science and Technology for Medicine, Ministry of Education, Fujian Provincial Key Laboratory for Photonics Technology, Fuzhou, 350007, China.

Lin, Duo. College of Integrated Traditional Chinese and Western Medicine, Fujian University of Traditional Chinese Medicine, Fuzhou, 350122, China.

Lin, Duo. linduo1986@163.com.

Feng, Shangyuan. Fujian Normal University, Key Laboratory of OptoElectronic Science and Technology for Medicine, Ministry of Education, Fujian Provincial Key Laboratory for Photonics Technology, Fuzhou, 350007, China.

Feng, Shangyuan. syfeng@fjnu.edu.cn.

Chen, Rong. Fujian Normal University, Key Laboratory of OptoElectronic Science and Technology for Medicine, Ministry of Education, Fujian Provincial Key Laboratory for Photonics Technology, Fuzhou, 350007, China.

Comments

Erratum for (EFR)

Year of Publication

2021

Link to the Ovid Full Text or citation:

[Click here for full text options](https://ovidsp.ovid.com/ovidweb.cgi?T=JS&CSC=Y&NEWS=N&PAGE=fulltext&D=pmnm6&AN=34123487)

Link to the External Link Resolver:

[SFX](https://sfx-86scu.hosted.exlibrisgroup.com.cn/86scu?sid=OVID:medline&id=pmid:34123487&id=doi:10.1364%2FBOE.426301&issn=21567085&isbn=&volume=12&issue=5&spage=2557&pages=2557-2558&date=2021&title=Biomedical+Optics+Express&atitle=Erratum%3A+Assessment+of+radiotherapy+effect+for+nasopharyngeal+cancer+using+plasma+surface-enhanced+Raman+spectroscopy+technology%3A+errata.&aulast=Wu&pid=<author>Wu+Q%3BQiu+S%3BYu+Y%3BChen+W%3BLin+H%3BLin+D%3BFeng+S%3BChen+R<%2Fauthor><AN>34123487<%2FAN><DT>Published+Erratum<%2FDT>)

38.

Biomass Nanoporous Carbon-Supported Pd Catalysts for Partial Hydrogenation of Biodiesel: Effects of Surface Chemistry on Pd Particle Size and Catalytic Performance.

Udomsap P, Meesiri S, Chollacoop N, Eiad-Ua A

Nanomaterials. 11(6), 2021 May 28.

[Journal Article]

UI: 34071581

Two types of cattail flower-derived nanoporous carbon (NPC), i.e., NPC activated with KOH and H3PO4, were produced and characterized using several techniques (e.g., Raman spectroscopy, nitrogen adsorption, and X-ray photoelectron spectroscopy). The influence of the carbon support characteristics on the particle sizes and chemical states of Pd in the synthesized Pd/NPC catalysts, which affect the catalytic activity and product selectivity, was analyzed. The surface chemistry properties of NPC were the main factors influencing the Pd particle size; by contrast, the textural properties did not significantly affect the size of the Pd particles on NPC supports. The use of Pd nanoparticles supported on the rich-functionalized surface carbons obtained by H3PO4 activation led to superior catalytic activity for the polyunsaturated fatty acid methyl ester (poly-FAME) hydrogenation, which could achieve 90% poly-FAME conversion and 84% selectivity towards monounsaturated FAME after a 45-min reaction time. This is due to the small Pd nanoparticle size and the high acidity of the catalysts, which are beneficial for the partial hydrogenation of poly-FAME in biodiesel. Conversely, the Pd nanoparticles supported on the high-surface-area carbon by KOH activation, with large Pd particle size and low acidity, required a longer reaction time to reach similar conversion and product selectivity levels.

Version ID

1

Record Owner

From MEDLINE, a database of the U.S. National Library of Medicine.

Status

PubMed-not-MEDLINE

Author NameID

Eiad-Ua, Apiluck; ORCID: <https://orcid.org/0000-0002-2090-7334>

Authors Full Name

Udomsap, Parncheewa, Meesiri, Sirasit, Chollacoop, Nuwong, Eiad-Ua, Apiluck

Institution

Udomsap, Parncheewa. College of Materials Innovation and Technology, King Mongkut's Institute of Technology Ladkrabang, Chalongkrung Rd., Ladkrabang, Bangkok 10520, Thailand. Meesiri, Sirasit. School of Energy and Environment, University of Phayao, Phaholyothin Rd., Mae Ka, Mueang Phayao, Phayao 56000, Thailand.

Chollacoop, Nuwong. Energy Innovation Research Group, National Energy Technology Center, 114 Thailand Science Park, Phaholyothin Rd., Klong 1, Klong Luang, Pathumthani 12120, Thailand.

Eiad-Ua, Apiluck. College of Materials Innovation and Technology, King Mongkut's Institute of Technology Ladkrabang, Chalongkrung Rd., Ladkrabang, Bangkok 10520, Thailand.

Keyword Heading

Pd catalyst chemical activation

nanoporous carbon

partial hydrogenation

particle size effect

surface chemistry.

Keyword Heading Owner

NOTNLM

Year of Publication

2021

Link to the Ovid Full Text or citation:

[Click here for full text options](https://ovidsp.ovid.com/ovidweb.cgi?T=JS&CSC=Y&NEWS=N&PAGE=fulltext&D=pmnm6&AN=34071581)

Link to the External Link Resolver:

[SFX](https://sfx-86scu.hosted.exlibrisgroup.com.cn/86scu?sid=OVID:medline&id=pmid:34071581&id=doi:10.3390%2Fnano11061431&issn=20794991&isbn=&volume=11&issue=6&spage=&pages=&date=2021&title=Nanomaterials&atitle=Biomass+Nanoporous+Carbon-Supported+Pd+Catalysts+for+Partial+Hydrogenation+of+Biodiesel%3A+Effects+of+Surface+Chemistry+on+Pd+Particle+Size+and+Catalytic+Performance.&aulast=Udomsap&pid=<author>Udomsap+P%3BMeesiri+S%3BChollacoop+N%3BEiad-Ua+A<%2Fauthor><AN>34071581<%2FAN><DT>Journal+Article<%2FDT>)

39.

Current Intraoperative Imaging Techniques to Improve Surgical Resection of Laryngeal Cancer: A Systematic Review. [Review]

Lauwerends LJ, Galema HA, Hardillo JAU, Sewnaik A, Monserez D, van Driel PBAA, Verhoef C, Baatenburg de Jong RJ, Hilling DE, Keereweer S

Cancers. 13(8), 2021 Apr 15.

[Journal Article. Review]

UI: 33920824

Laryngeal cancer is a prevalent head and neck malignancy, with poor prognosis and low survival rates for patients with advanced disease. Treatment consists of unimodal therapy through surgery or radiotherapy in early staged tumors, while advanced stage tumors are generally treated with multimodal chemoradiotherapy or (total) laryngectomy followed by radiotherapy. Still, the recurrence rate for advanced laryngeal cancer is between 25 and 50%. In order to improve surgical resection of laryngeal cancer and reduce local recurrence rates, various intraoperative optical imaging techniques have been investigated. In this systematic review, we identify these technologies, evaluating the current state and future directions of optical imaging for this indication. Narrow-band imaging (NBI) and autofluorescence (AF) are established tools for early detection of laryngeal cancer. Nonetheless, their intraoperative utility is limited by an intrinsic inability to image beyond the (sub-)mucosa. Likewise, contact endoscopy (CE) and optical coherence tomography (OCT) are technically cumbersome and only useful for mucosal margin assessment. Research on fluorescence imaging (FLI) for this application is sparse, dealing solely with nonspecific fluorescent agents. Evidently, the imaging modalities that have been investigated thus far are generally unsuitable for deep margin assessment. We discuss two optical imaging techniques that can overcome these limitations and suggest how they can be used to achieve adequate margins in laryngeal cancer at all stages.

Version ID

1

Record Owner

From MEDLINE, a database of the U.S. National Library of Medicine.

Status

PubMed-not-MEDLINE

Author NameID

Lauwerends, Lorraine J; ORCID: <https://orcid.org/0000-0001-9704-7806> Galema, Hidde A; ORCID: <https://orcid.org/0000-0002-7777-0754>

Hardillo, Jose A U; ORCID: <https://orcid.org/0000-0001-5580-5613>

Monserez, Dominiek; ORCID: <https://orcid.org/0000-0002-7117-7300>

Verhoef, Cornelis; ORCID: <https://orcid.org/0000-0001-9980-8613>

Baatenburg de Jong, Robert J; ORCID: <https://orcid.org/0000-0001-7236-264X>

Hilling, Denise E; ORCID: <https://orcid.org/0000-0002-5875-6164>

Keereweer, Stijn; ORCID: <https://orcid.org/0000-0003-4665-6868>

Authors Full Name

Lauwerends, Lorraine J, Galema, Hidde A, Hardillo, Jose A U, Sewnaik, Aniel, Monserez, Dominiek, van Driel, Pieter B A A, Verhoef, Cornelis, Baatenburg de Jong, Robert J, Hilling, Denise E, Keereweer, Stijn

Institution

Lauwerends, Lorraine J. Department of Otorhinolaryngology, Head and Neck Surgery, Erasmus MC Cancer Institute, 3015 GD Rotterdam, The Netherlands. Galema, Hidde A. Department of Otorhinolaryngology, Head and Neck Surgery, Erasmus MC Cancer Institute, 3015 GD Rotterdam, The Netherlands.

Galema, Hidde A. Department of Surgical Oncology and Gastrointestinal Surgery, Erasmus MC Cancer Institute, 3015 GD Rotterdam, The Netherlands.

Hardillo, Jose A U. Department of Otorhinolaryngology, Head and Neck Surgery, Erasmus MC Cancer Institute, 3015 GD Rotterdam, The Netherlands.

Sewnaik, Aniel. Department of Otorhinolaryngology, Head and Neck Surgery, Erasmus MC Cancer Institute, 3015 GD Rotterdam, The Netherlands.

Monserez, Dominiek. Department of Otorhinolaryngology, Head and Neck Surgery, Erasmus MC Cancer Institute, 3015 GD Rotterdam, The Netherlands.

van Driel, Pieter B A A. Department of Orthopedic Surgery, Isala Hospital, 8025 AB Zwolle, The Netherlands.

Verhoef, Cornelis. Department of Surgical Oncology and Gastrointestinal Surgery, Erasmus MC Cancer Institute, 3015 GD Rotterdam, The Netherlands.

Baatenburg de Jong, Robert J. Department of Otorhinolaryngology, Head and Neck Surgery, Erasmus MC Cancer Institute, 3015 GD Rotterdam, The Netherlands.

Hilling, Denise E. Department of Surgical Oncology and Gastrointestinal Surgery, Erasmus MC Cancer Institute, 3015 GD Rotterdam, The Netherlands.

Keereweer, Stijn. Department of Otorhinolaryngology, Head and Neck Surgery, Erasmus MC Cancer Institute, 3015 GD Rotterdam, The Netherlands.

Keyword Heading

Raman spectroscopy fluorescence imaging

intraoperative imaging

laryngeal cancer

narrow-band imaging

surgical margins.

Keyword Heading Owner

NOTNLM

Year of Publication

2021

Link to the Ovid Full Text or citation:

[Click here for full text options](https://ovidsp.ovid.com/ovidweb.cgi?T=JS&CSC=Y&NEWS=N&PAGE=fulltext&D=pmnm6&AN=33920824)

Link to the External Link Resolver:

[SFX](https://sfx-86scu.hosted.exlibrisgroup.com.cn/86scu?sid=OVID:medline&id=pmid:33920824&id=doi:10.3390%2Fcancers13081895&issn=20726694&isbn=&volume=13&issue=8&spage=&pages=&date=2021&title=Cancers&atitle=Current+Intraoperative+Imaging+Techniques+to+Improve+Surgical+Resection+of+Laryngeal+Cancer%3A+A+Systematic+Review.&aulast=Lauwerends&pid=<author>Lauwerends+LJ%3BGalema+HA%3BHardillo+JAU%3BSewnaik+A%3BMonserez+D%3Bvan+Driel+PBAA%3BVerhoef+C%3BBaatenburg+de+Jong+RJ%3BHilling+DE%3BKeereweer+S<%2Fauthor><AN>33920824<%2FAN><DT>Journal+Article<%2FDT>)

40.

Optical diagnosis of oral cavity lesions by label-free Raman spectroscopy.

Matthies L, Gebrekidan MT, Tegtmeyer JF, Oetter N, Rohde M, Vollkommer T, Smeets R, Wilczak W, Stelzle F, Gosau M, Braeuer AS, Knipfer C

Biomedical Optics Express. 12(2):836-851, 2021 Feb 01.

[Journal Article]

UI: 33680545

Oral squamous cell carcinoma (OSCC) is one of the most prevalent cancers and frequently preceded by non-malignant lesions. Using Shifted-Excitation Raman Difference Spectroscopy (SERDS), principal component and linear discriminant analysis in native tissue specimens, 9500 raw Raman spectra of OSCC, 4300 of non-malignant lesions and 4200 of physiological mucosa were evaluated. Non-malignant lesions were distinguished from physiological mucosa with a classification accuracy of 95.3% (95.4% sensitivity, 95.2% specificity, area under the curve (AUC) 0.99). Discriminating OSCC from non-malignant lesions showed an accuracy of 88.4% (93.7% sensitivity, 76.7% specificity, AUC 0.93). OSCC was identified against physiological mucosa with an accuracy of 89.8% (93.7% sensitivity, 81.0% specificity, AUC 0.90). These findings underline the potential of SERDS for the diagnosis of oral cavity lesions.

Copyright © 2021 Optical Society of America under the terms of the OSA Open Access Publishing Agreement.

Version ID

1

Record Owner

From MEDLINE, a database of the U.S. National Library of Medicine.

Status

PubMed-not-MEDLINE

Author NameID

Matthies, Levi; ORCID: <https://orcid.org/0000-0001-7168-4428>

Authors Full Name

Matthies, Levi, Gebrekidan, Medhanie T, Tegtmeyer, Jasper F, Oetter, Nicolai, Rohde, Maximilian, Vollkommer, Tobias, Smeets, Ralf, Wilczak, Waldemar, Stelzle, Florian, Gosau, Martin, Braeuer, Andreas S, Knipfer, Christian

Institution

Matthies, Levi. University Medical Center Hamburg-Eppendorf (UKE), Department of Oral and Maxillofacial Surgery, Martinistrase 52, D-20246 Hamburg, Germany. Matthies, Levi. These authors contributed equally.

Gebrekidan, Medhanie T. Friedrich-Alexander-Universitat Erlangen-Nurnberg (FAU), Erlangen Graduate School in Advanced Optical Technologies (SAOT), Paul-Gordan-Strase 6, D-91054 Erlangen, Germany.

Gebrekidan, Medhanie T. Technische Universitat Bergakademie Freiberg (TUBAF), Institute of Thermal-, Environmental- and Resources' Process Engineering (ITUN), Leipziger Strase 28, D-09599 Freiberg, Germany.

Gebrekidan, Medhanie T. These authors contributed equally.

Tegtmeyer, Jasper F. University Medical Center Hamburg-Eppendorf (UKE), Department of Oral and Maxillofacial Surgery, Martinistrase 52, D-20246 Hamburg, Germany.

Oetter, Nicolai. Friedrich-Alexander-Universitat Erlangen-Nurnberg (FAU), Erlangen Graduate School in Advanced Optical Technologies (SAOT), Paul-Gordan-Strase 6, D-91054 Erlangen, Germany.

Oetter, Nicolai. Friedrich-Alexander-Universitat Erlangen-Nurnberg (FAU), Department of Oral and Maxillofacial Surgery, Gluckstrase 11, D-91054 Erlangen, Germany.

Rohde, Maximilian. Friedrich-Alexander-Universitat Erlangen-Nurnberg (FAU), Department of Oral and Maxillofacial Surgery, Gluckstrase 11, D-91054 Erlangen, Germany.

Vollkommer, Tobias. University Medical Center Hamburg-Eppendorf (UKE), Department of Oral and Maxillofacial Surgery, Martinistrase 52, D-20246 Hamburg, Germany.

Smeets, Ralf. University Medical Center Hamburg-Eppendorf (UKE), Department of Oral and Maxillofacial Surgery, Martinistrase 52, D-20246 Hamburg, Germany.

Wilczak, Waldemar. University Medical Center Hamburg-Eppendorf (UKE), Institute of Pathology, Martinistrase 52, D-20246 Hamburg, Germany.

Stelzle, Florian. Friedrich-Alexander-Universitat Erlangen-Nurnberg (FAU), Erlangen Graduate School in Advanced Optical Technologies (SAOT), Paul-Gordan-Strase 6, D-91054 Erlangen, Germany.

Stelzle, Florian. Friedrich-Alexander-Universitat Erlangen-Nurnberg (FAU), Department of Oral and Maxillofacial Surgery, Gluckstrase 11, D-91054 Erlangen, Germany.

Gosau, Martin. University Medical Center Hamburg-Eppendorf (UKE), Department of Oral and Maxillofacial Surgery, Martinistrase 52, D-20246 Hamburg, Germany.

Braeuer, Andreas S. Technische Universitat Bergakademie Freiberg (TUBAF), Institute of Thermal-, Environmental- and Resources' Process Engineering (ITUN), Leipziger Strase 28, D-09599 Freiberg, Germany.

Knipfer, Christian. University Medical Center Hamburg-Eppendorf (UKE), Department of Oral and Maxillofacial Surgery, Martinistrase 52, D-20246 Hamburg, Germany.

Year of Publication

2021

Link to the Ovid Full Text or citation:

[Click here for full text options](https://ovidsp.ovid.com/ovidweb.cgi?T=JS&CSC=Y&NEWS=N&PAGE=fulltext&D=pmnm6&AN=33680545)

Link to the External Link Resolver:

[SFX](https://sfx-86scu.hosted.exlibrisgroup.com.cn/86scu?sid=OVID:medline&id=pmid:33680545&id=doi:10.1364%2FBOE.409456&issn=21567085&isbn=&volume=12&issue=2&spage=836&pages=836-851&date=2021&title=Biomedical+Optics+Express&atitle=Optical+diagnosis+of+oral+cavity+lesions+by+label-free+Raman+spectroscopy.&aulast=Matthies&pid=<author>Matthies+L%3BGebrekidan+MT%3BTegtmeyer+JF%3BOetter+N%3BRohde+M%3BVollkommer+T%3BSmeets+R%3BWilczak+W%3BStelzle+F%3BGosau+M%3BBraeuer+AS%3BKnipfer+C<%2Fauthor><AN>33680545<%2FAN><DT>Journal+Article<%2FDT>)

41.

The Potential of Raman Spectroscopy in the Diagnosis of Dysplastic and Malignant Oral Lesions.

Ibrahim O, Toner M, Flint S, Byrne HJ, Lyng FM

Cancers. 13(4), 2021 Feb 04.

[Journal Article]

UI: 33557195

Early diagnosis, treatment and/or surveillance of oral premalignant lesions are important in preventing progression to oral squamous cell carcinoma (OSCC). The current gold standard is through histopathological diagnosis, which is limited by inter- and intra-observer errors and sampling errors. The objective of this work was to use Raman spectroscopy to discriminate between benign, mild, moderate and severe dysplasia and OSCC in formalin fixed paraffin preserved (FFPP) tissues. The study included 72 different pathologies from which 17 were benign lesions, 20 mildly dysplastic, 20 moderately dysplastic, 10 severely dysplastic and 5 invasive OSCC. The glass substrate and paraffin wax background were digitally removed and PLSDA with LOPO cross-validation was used to differentiate the pathologies. OSCC could be differentiated from the other pathologies with an accuracy of 70%, while the accuracy of the classifier for benign, moderate and severe dysplasia was ~60%. The accuracy of the classifier was lowest for mild dysplasia (~46%). The main discriminating features were increased nucleic acid contributions and decreased protein and lipid contributions in the epithelium and decreased collagen contributions in the connective tissue. Smoking and the presence of inflammation were found to significantly influence the Raman classification with respective accuracies of 76% and 94%.

Version ID

1

Record Owner

From MEDLINE, a database of the U.S. National Library of Medicine.

Status

PubMed-not-MEDLINE

Author NameID

Ibrahim, Ola; ORCID: <https://orcid.org/0000-0002-8196-0307> Byrne, Hugh J; ORCID: <https://orcid.org/0000-0002-1735-8610>

Lyng, Fiona M; ORCID: <https://orcid.org/0000-0002-9876-963X>

Authors Full Name

Ibrahim, Ola, Toner, Mary, Flint, Stephen, Byrne, Hugh J, Lyng, Fiona M

Institution

Ibrahim, Ola. School of Dental Science, Trinity College Dublin, Lincoln Place, D02 Dublin 2, Ireland. Toner, Mary. Central Pathology Laboratory, St. James Hospital, James Street, D08 Dublin 8, Ireland.

Flint, Stephen. Oral Medicine Unit, Dublin Dental University Hospital, Trinity College Dublin, Lincoln Place, D02 Dublin 2, Ireland.

Byrne, Hugh J. FOCAS Research Institute, City Campus, Technological University Dublin, Kevin Street, D08 Dublin 8, Ireland.

Lyng, Fiona M. Radiation and Environmental Science Centre FOCAS Research Institute, City Campus, Technological University Dublin, Kevin Street, D08 Dublin 8, Ireland.

Lyng, Fiona M. School of Physics & Clinical & Optometric Sciences, City Campus, Technological University Dublin, Kevin Street, D08 Dublin 8, Ireland.

Keyword Heading

Raman spectroscopy oral cancer

oral dysplasia

oral pre-cancer

potentially malignant lesions

premalignant lesions.

Keyword Heading Owner

NOTNLM

Year of Publication

2021

Link to the Ovid Full Text or citation:

[Click here for full text options](https://ovidsp.ovid.com/ovidweb.cgi?T=JS&CSC=Y&NEWS=N&PAGE=fulltext&D=pmnm6&AN=33557195)

Link to the External Link Resolver:

[SFX](https://sfx-86scu.hosted.exlibrisgroup.com.cn/86scu?sid=OVID:medline&id=pmid:33557195&id=doi:10.3390%2Fcancers13040619&issn=20726694&isbn=&volume=13&issue=4&spage=&pages=&date=2021&title=Cancers&atitle=The+Potential+of+Raman+Spectroscopy+in+the+Diagnosis+of+Dysplastic+and+Malignant+Oral+Lesions.&aulast=Ibrahim&pid=<author>Ibrahim+O%3BToner+M%3BFlint+S%3BByrne+HJ%3BLyng+FM<%2Fauthor><AN>33557195<%2FAN><DT>Journal+Article<%2FDT>)

42.

Tracking of the biochemical changes upon pleomorphic adenoma progression using vibrational microspectroscopy.

Paluszkiewicz C, Roman M, Piergies N, Pieta E, Wozniak M, Guidi MC, Miskiewicz-Orczyk K, Markow M, Scierski W, Misiolek M, Drozdzowska B, Kwiatek WM

Scientific Reports. 11(1):18010, 2021 09 09.

[Journal Article. Research Support, Non-U.S. Gov't]

UI: 34504182

Head and neck tumors can be very challenging to treat because of the risk of problems or complications after surgery. Therefore, prompt and accurate diagnosis is extremely important to drive appropriate treatment decisions, which may reduce the chance of recurrence. This paper presents the original research exploring the feasibility of Fourier transform infrared (FT-IR) and Raman spectroscopy (RS) methods to investigate biochemical alterations upon the development of the pleomorphic adenoma. Principal component analysis (PCA) was used for a detailed assessment of the observed changes and to determine the spectroscopic basis for salivary gland neoplastic pathogenesis. It is implied that within the healthy margin, as opposed to the tumoral tissue, there are parts that differ significantly in lipid content. This observation shed new light on the crucial role of lipids in tissue physiology and tumorigenesis. Thus, a novel approach that eliminates the influence of lipids on the elucidation of biochemical changes is proposed. The performed analysis suggests that the highly heterogeneous healthy margin contains more unsaturated triacylglycerols, while the tumoral section is rich in proteins. The difference in protein content was also observed for these two tissue types, i.e. the healthy tissue possesses more proteins in the anti-parallel beta-sheet conformation, whereas the tumoral tissue is dominated by proteins rich in unordered random coils. Furthermore, the pathogenic tissue shows a higher content of carbohydrates and reveals noticeable differences in nucleic acid content. Finally, FT-IR and Raman spectroscopy methods were proposed as very promising methods in the discrimination of tumoral and healthy tissues of the salivary gland.

Copyright © 2021. The Author(s).

Version ID

1

Record Owner

From MEDLINE, a database of the U.S. National Library of Medicine.

Status

MEDLINE

Author NameID

Paluszkiewicz, Czeslawa; ORCID: <http://orcid.org/0000-0003-0186-8090> Roman, Maciej; ORCID: <http://orcid.org/0000-0003-0921-426X>

Piergies, Natalia; ORCID: <http://orcid.org/0000-0003-4899-3534>

Pieta, Ewa; ORCID: <http://orcid.org/0000-0001-7071-0284>

Wozniak, Monika; ORCID: <http://orcid.org/0000-0002-3709-8318>

Guidi, Mariangela Cestelli; ORCID: <http://orcid.org/0000-0002-6884-3915>

Miskiewicz-Orczyk, Katarzyna; ORCID: <http://orcid.org/0000-0001-8088-3437>

Markow, Magdalena; ORCID: <http://orcid.org/0000-0002-1072-2125>

Scierski, Wojciech; ORCID: <http://orcid.org/0000-0003-3242-8047>

Misiolek, Maciej; ORCID: <http://orcid.org/0000-0002-8476-9153>

Drozdzowska, Bogna; ORCID: <http://orcid.org/0000-0002-2287-6842>

Kwiatek, Wojciech M; ORCID: <http://orcid.org/0000-0002-2197-8572>

Authors Full Name

Paluszkiewicz, Czeslawa, Roman, Maciej, Piergies, Natalia, Pieta, Ewa, Wozniak, Monika, Guidi, Mariangela Cestelli, Miskiewicz-Orczyk, Katarzyna, Markow, Magdalena, Scierski, Wojciech, Misiolek, Maciej, Drozdzowska, Bogna, Kwiatek, Wojciech M

Institution

Paluszkiewicz, Czeslawa. Institute of Nuclear Physics, Polish Academy of Sciences, Radzikowskiego 152, 31-342, Krakow, Poland. Czeslawa.Paluszkiewicz@ifj.edu.pl. Roman, Maciej. Institute of Nuclear Physics, Polish Academy of Sciences, Radzikowskiego 152, 31-342, Krakow, Poland. Maciej.Roman@ifj.edu.pl.

Piergies, Natalia. Institute of Nuclear Physics, Polish Academy of Sciences, Radzikowskiego 152, 31-342, Krakow, Poland.

Pieta, Ewa. Institute of Nuclear Physics, Polish Academy of Sciences, Radzikowskiego 152, 31-342, Krakow, Poland.

Wozniak, Monika. Institute of Nuclear Physics, Polish Academy of Sciences, Radzikowskiego 152, 31-342, Krakow, Poland.

Guidi, Mariangela Cestelli. INFN-Laboratori Nazionali di Frascati, Via E. Fermi 40, 00044, Frascati, Italy.

Miskiewicz-Orczyk, Katarzyna. Department of Otorhinolaryngology and Laryngological Oncology in Zabrze, Medical University of Silesia Katowice, 41800, Zabrze, Poland.

Markow, Magdalena. Department of Otorhinolaryngology and Laryngological Oncology in Zabrze, Medical University of Silesia Katowice, 41800, Zabrze, Poland.

Scierski, Wojciech. Department of Otorhinolaryngology and Laryngological Oncology in Zabrze, Medical University of Silesia Katowice, 41800, Zabrze, Poland.

Misiolek, Maciej. Department of Otorhinolaryngology and Laryngological Oncology in Zabrze, Medical University of Silesia Katowice, 41800, Zabrze, Poland.

Drozdzowska, Bogna. Department of Pathomorphology Zabrze, Medical University of Silesia, Katowice, Poland.

Kwiatek, Wojciech M. Institute of Nuclear Physics, Polish Academy of Sciences, Radzikowskiego 152, 31-342, Krakow, Poland.

MeSH Heading

*Adenoma, Pleomorphic/di [Diagnosis]. Adenoma, Pleomorphic/me [Metabolism]. Adenoma, Pleomorphic/pa [Pathology]. Adenoma, Pleomorphic/su [Surgery]. Carbohydrates/ch [Chemistry]. Carcinogenesis/me [Metabolism]. Carcinogenesis/pa [Pathology]. Datasets as Topic. Eosine Yellowish-(YS). Female. Hematoxylin. *Histocytochemistry/mt [Methods]. Humans. Male. Middle Aged. Neoplasm Proteins/me [Metabolism]. Nucleic Acids/me [Metabolism]. Organ Specificity. Principal Component Analysis. Protein Conformation, alpha-Helical. Protein Conformation, beta-Strand. *Salivary Gland Neoplasms/di [Diagnosis]. Salivary Gland Neoplasms/me [Metabolism]. Salivary Gland Neoplasms/pa [Pathology]. Salivary Gland Neoplasms/su [Surgery]. *Spectroscopy, Fourier Transform Infrared/mt [Methods]. *Spectrum Analysis, Raman/mt [Methods]. Triglycerides/me [Metabolism].

Registry Number/Name of Substance

0 (Carbohydrates). 0 (Neoplasm Proteins). 0 (Nucleic Acids). 0 (Triglycerides). TDQ283MPCW (Eosine Yellowish-(YS)). YKM8PY2Z55 (Hematoxylin).

Year of Publication

2021

Link to the Ovid Full Text or citation:

[Click here for full text options](https://ovidsp.ovid.com/ovidweb.cgi?T=JS&CSC=Y&NEWS=N&PAGE=fulltext&D=med20&AN=34504182)

Link to the External Link Resolver:

[SFX](https://sfx-86scu.hosted.exlibrisgroup.com.cn/86scu?sid=OVID:medline&id=pmid:34504182&id=doi:10.1038%2Fs41598-021-97377-2&issn=20452322&isbn=&volume=11&issue=1&spage=18010&pages=18010&date=2021&title=Scientific+Reports&atitle=Tracking+of+the+biochemical+changes+upon+pleomorphic+adenoma+progression+using+vibrational+microspectroscopy.&aulast=Paluszkiewicz&pid=<author>Paluszkiewicz+C%3BRoman+M%3BPiergies+N%3BPieta+E%3BWozniak+M%3BGuidi+MC%3BMiskiewicz-Orczyk+K%3BMarkow+M%3BScierski+W%3BMisiolek+M%3BDrozdzowska+B%3BKwiatek+WM<%2Fauthor><AN>34504182<%2FAN><DT>Journal+Article<%2FDT>)

43.

Raman spectral cytopathology for cancer diagnostic applications.

Traynor D, Behl I, O'Dea D, Bonnier F, Nicholson S, O'Connell F, Maguire A, Flint S, Galvin S, Healy CM, Martin CM, O'Leary JJ, Malkin A, Byrne HJ, Lyng FM

Nature Protocols. 16(7):3716-3735, 2021 07.

[Journal Article. Research Support, Non-U.S. Gov't]

UI: 34117476

Raman spectroscopy can provide a rapid, label-free, nondestructive measurement of the chemical fingerprint of a sample and has shown potential for cancer screening and diagnosis. Here we report a protocol for Raman microspectroscopic analysis of different exfoliative cytology samples (cervical, oral and lung), covering sample preparation, spectral acquisition, preprocessing and data analysis. The protocol takes 2 h 20 min for sample preparation, measurement and data preprocessing and up to 8 h for a complete analysis. A key feature of the protocol is that it uses the same sample preparation procedure as commonly used in diagnostic cytology laboratories (i.e., liquid-based cytology on glass slides), ensuring compatibility with clinical workflows. Our protocol also covers methods to correct for the spectral contribution of glass and sample pretreatment methods to remove contaminants (such as blood and mucus) that can obscure spectral features in the exfoliated cells and lead to variability. The protocol establishes a standardized clinical routine allowing the collection of highly reproducible data for Raman spectral cytopathology for cancer diagnostic applications for cervical and lung cancer and for monitoring suspicious lesions for oral cancer.

Version ID

1

Record Owner

From MEDLINE, a database of the U.S. National Library of Medicine.

Status

MEDLINE

Author NameID

Lyng, Fiona M; ORCID: <http://orcid.org/0000-0002-9876-963X>

Authors Full Name

Traynor, Damien, Behl, Isha, O'Dea, Declan, Bonnier, Franck, Nicholson, Siobhan, O'Connell, Finbar, Maguire, Aoife, Flint, Stephen, Galvin, Sheila, Healy, Claire M, Martin, Cara M, O'Leary, John J, Malkin, Alison, Byrne, Hugh J, Lyng, Fiona M

Institution

Traynor, Damien. Centre for Radiation and Environmental Science, FOCAS Research Institute, Technological University Dublin, Dublin, Ireland. Traynor, Damien. School of Physics & Clinical & Optometric Sciences, Technological University Dublin, Dublin, Ireland.

Behl, Isha. Centre for Radiation and Environmental Science, FOCAS Research Institute, Technological University Dublin, Dublin, Ireland.

Behl, Isha. School of Physics & Clinical & Optometric Sciences, Technological University Dublin, Dublin, Ireland.

O'Dea, Declan. Centre for Radiation and Environmental Science, FOCAS Research Institute, Technological University Dublin, Dublin, Ireland.

O'Dea, Declan. School of Biological and Health Sciences, Technological University Dublin, Dublin, Ireland.

Bonnier, Franck. EA 6295 Nanomedicaments et Nanosondes, Universite de Tours, Tours, France.

Nicholson, Siobhan. St James's Hospital, Dublin, Ireland.

O'Connell, Finbar. St James's Hospital, Dublin, Ireland.

Maguire, Aoife. St James's Hospital, Dublin, Ireland.

Flint, Stephen. Oral Medicine Unit, Dublin Dental University Hospital, Trinity College, Dublin, Ireland.

Galvin, Sheila. Oral Medicine Unit, Dublin Dental University Hospital, Trinity College, Dublin, Ireland.

Healy, Claire M. Oral Medicine Unit, Dublin Dental University Hospital, Trinity College, Dublin, Ireland.

Martin, Cara M. Discipline of Histopathology, University of Dublin Trinity College, Dublin, Ireland.

Martin, Cara M. Emer Casey Molecular Pathology Research Laboratory, The Coombe Women and Infants University Hospital, Dublin, Ireland.

Martin, Cara M. CERVIVA Research Consortium, Dublin, Ireland.

O'Leary, John J. Discipline of Histopathology, University of Dublin Trinity College, Dublin, Ireland.

O'Leary, John J. Emer Casey Molecular Pathology Research Laboratory, The Coombe Women and Infants University Hospital, Dublin, Ireland.

O'Leary, John J. CERVIVA Research Consortium, Dublin, Ireland.

Malkin, Alison. School of Biological and Health Sciences, Technological University Dublin, Dublin, Ireland.

Byrne, Hugh J. FOCAS Research Institute, Technological University Dublin, Dublin, Ireland.

Lyng, Fiona M. Centre for Radiation and Environmental Science, FOCAS Research Institute, Technological University Dublin, Dublin, Ireland. fiona.lyng@tudublin.ie.

Lyng, Fiona M. School of Physics & Clinical & Optometric Sciences, Technological University Dublin, Dublin, Ireland. fiona.lyng@tudublin.ie.

Lyng, Fiona M. CERVIVA Research Consortium, Dublin, Ireland. fiona.lyng@tudublin.ie.

MeSH Heading

Algorithms. Cervix Uteri/pa [Pathology]. *Early Detection of Cancer. Female. Humans. Image Processing, Computer-Assisted. Lung/pa [Pathology]. *Neoplasms/di [Diagnosis]. *Neoplasms/pa [Pathology]. *Spectrum Analysis, Raman/mt [Methods].

Year of Publication

2021

Link to the Ovid Full Text or citation:

[Click here for full text options](https://ovidsp.ovid.com/ovidweb.cgi?T=JS&CSC=Y&NEWS=N&PAGE=fulltext&D=med20&AN=34117476)

Link to the External Link Resolver:

[SFX](https://sfx-86scu.hosted.exlibrisgroup.com.cn/86scu?sid=OVID:medline&id=pmid:34117476&id=doi:10.1038%2Fs41596-021-00559-5&issn=17502799&isbn=&volume=16&issue=7&spage=3716&pages=3716-3735&date=2021&title=Nature+Protocols&atitle=Raman+spectral+cytopathology+for+cancer+diagnostic+applications.&aulast=Traynor&pid=<author>Traynor+D%3BBehl+I%3BO'Dea+D%3BBonnier+F%3BNicholson+S%3BO'Connell+F%3BMaguire+A%3BFlint+S%3BGalvin+S%3BHealy+CM%3BMartin+CM%3BO'Leary+JJ%3BMalkin+A%3BByrne+HJ%3BLyng+FM<%2Fauthor><AN>34117476<%2FAN><DT>Journal+Article<%2FDT>)

44.

Experimental study on needle insertion force to minimize tissue deformation in tongue tissue.

Aaboubout Y, Nunes Soares MR, Barroso EM, van der Sar LC, Bocharnikov A, Usenov I, Artyushenko V, Caspers PJ, Koljenovic S, Bakker Schut TC, van den Dobbelsteen JJ, Puppels GJ

Medical Engineering & Physics. 97:40-46, 2021 11.

[Journal Article. Research Support, Non-U.S. Gov't]

UI: 34756337

This study reports on the effects of insertion velocity, needle tip geometry and needle diameter on tissue deformation and maximum insertion force. Moreover, the effect of multiple insertions with the same needle on the maximum insertion force is reported. The tissue deformation and maximum insertion force strongly depend on the insertion velocity and the tip geometry. No correlation was found between the outer diameter and the maximum insertion force for small needles (30G - 32G). The endurance experiments showed no remarkable difference in the maximum insertion force during 100 insertions.

Copyright © 2021 The Authors. Published by Elsevier Ltd.. All rights reserved.

Version ID

1

Record Owner

From MEDLINE, a database of the U.S. National Library of Medicine.

Status

MEDLINE

Authors Full Name

Aaboubout, Y, Nunes Soares, M R, Barroso, E M, van der Sar, L C, Bocharnikov, A, Usenov, I, Artyushenko, V, Caspers, P J, Koljenovic, S, Bakker Schut, T C, van den Dobbelsteen, J J, Puppels, G J

Institution

Aaboubout, Y. Department of Pathology, Erasmus MC, University Medical Center Rotterdam, Wytemaweg 80 CN, Rotterdam 3015, the Netherlands; Department of Otorhinolaryngology and Head and Neck Surgery, Erasmus MC, University Medical Center Rotterdam, the Netherlands. Electronic address: y.aaboubout@erasmusmc.nl. Nunes Soares, M R. Department of Pathology, Erasmus MC, University Medical Center Rotterdam, Wytemaweg 80 CN, Rotterdam 3015, the Netherlands.

Barroso, E M. Department of Pathology, Erasmus MC, University Medical Center Rotterdam, Wytemaweg 80 CN, Rotterdam 3015, the Netherlands; Department of Oral and Maxillofacial Surgery, Erasmus MC, University Medical Center Rotterdam, the Netherlands; Department of Dermatology, Erasmus MC, University Medical Center Rotterdam, the Netherlands.

van der Sar, L C. Department of Pathology, Erasmus MC, University Medical Center Rotterdam, Wytemaweg 80 CN, Rotterdam 3015, the Netherlands.

Bocharnikov, A. Art Photonics GmbH, Berlin, Germany.

Usenov, I. Art Photonics GmbH, Berlin, Germany.

Artyushenko, V. Art Photonics GmbH, Berlin, Germany.

Caspers, P J. Department of Dermatology, Erasmus MC, University Medical Center Rotterdam, the Netherlands.

Koljenovic, S. Department of Pathology, Erasmus MC, University Medical Center Rotterdam, Wytemaweg 80 CN, Rotterdam 3015, the Netherlands.

Bakker Schut, T C. Department of Dermatology, Erasmus MC, University Medical Center Rotterdam, the Netherlands.

van den Dobbelsteen, J J. Department of Biomechanical Engineering, Delft University of Technology, Delft, the Netherlands.

Puppels, G J. Department of Dermatology, Erasmus MC, University Medical Center Rotterdam, the Netherlands.

MeSH Heading

Gravitation. *Mechanical Phenomena. *Needles. Tongue.

Keyword Heading

Head and neck cancer Intraoperative assessment

Needle-tissue interaction

Oral cavity

Raman spectroscopy

Tissue deformation.

Keyword Heading Owner

NOTNLM

Year of Publication

2021

Link to the Ovid Full Text or citation:

[Click here for full text options](https://ovidsp.ovid.com/ovidweb.cgi?T=JS&CSC=Y&NEWS=N&PAGE=fulltext&D=med20&AN=34756337)

Link to the External Link Resolver:

[SFX](https://sfx-86scu.hosted.exlibrisgroup.com.cn/86scu?sid=OVID:medline&id=pmid:34756337&id=doi:10.1016%2Fj.medengphy.2021.10.003&issn=13504533&isbn=&volume=97&issue=&spage=40&pages=40-46&date=2021&title=Medical+Engineering+%26+Physics&atitle=Experimental+study+on+needle+insertion+force+to+minimize+tissue+deformation+in+tongue+tissue.&aulast=Aaboubout&pid=<author>Aaboubout+Y%3BNunes+Soares+MR%3BBarroso+EM%3Bvan+der+Sar+LC%3BBocharnikov+A%3BUsenov+I%3BArtyushenko+V%3BCaspers+PJ%3BKoljenovic+S%3BBakker+Schut+TC%3Bvan+den+Dobbelsteen+JJ%3BPuppels+GJ<%2Fauthor><AN>34756337<%2FAN><DT>Journal+Article<%2FDT>)

45.

Pilot study on the value of Raman spectroscopy in the entity assignment of salivary gland tumors.

Meyer TJ, Gerhard-Hartmann E, Lodes N, Scherzad A, Hagen R, Steinke M, Hackenberg S

PLoS ONE [Electronic Resource]. 16(9):e0257470, 2021.

[Journal Article. Research Support, Non-U.S. Gov't]

UI: 34529739

BACKGROUND: The entity assignment of salivary gland tumors (SGT) based on histomorphology can be challenging. Raman spectroscopy has been applied to analyze differences in the molecular composition of tissues. The aim of this study was to evaluate the suitability of RS for entity assignment in SGT.

METHODS: Raman data were collected in deparaffinized sections of pleomorphic adenomas (PA) and adenoid cystic carcinomas (ACC). Multivariate data and chemometric analysis were completed using the Unscrambler software.

RESULTS: The Raman spectra detected in ACC samples were mostly assigned to nucleic acids, lipids, and amides. In a principal component-based linear discriminant analysis (LDA) 18 of 20 tumor samples were classified correctly.

CONCLUSION: In this proof of concept study, we show that a reliable SGT diagnosis based on LDA algorithm appears possible, despite variations in the entity-specific mean spectra. However, a standardized workflow for tissue sample preparation, measurement setup, and chemometric algorithms is essential to get reliable results.

Version ID

1

Record Owner

From MEDLINE, a database of the U.S. National Library of Medicine.

Status

MEDLINE

Author NameID

Meyer, Till Jasper; ORCID: <https://orcid.org/0000-0003-2377-3519>

Authors Full Name

Meyer, Till Jasper, Gerhard-Hartmann, Elena, Lodes, Nina, Scherzad, Agmal, Hagen, Rudolf, Steinke, Maria, Hackenberg, Stephan

Institution

Meyer, Till Jasper. Department of Oto-Rhino-Laryngology, Plastic, Aesthetic & Reconstructive Head and Neck Surgery, University Hospital Wurzburg, Wurzburg, Germany. Gerhard-Hartmann, Elena. Institute of Pathology, University of Wurzburg, Wurzburg, Germany.

Lodes, Nina. Chair of Tissue Engineering and Regenerative Medicine, University Hospital Wurzburg, Wurzburg, Germany.

Scherzad, Agmal. Department of Oto-Rhino-Laryngology, Plastic, Aesthetic & Reconstructive Head and Neck Surgery, University Hospital Wurzburg, Wurzburg, Germany.

Hagen, Rudolf. Department of Oto-Rhino-Laryngology, Plastic, Aesthetic & Reconstructive Head and Neck Surgery, University Hospital Wurzburg, Wurzburg, Germany.

Steinke, Maria. Chair of Tissue Engineering and Regenerative Medicine, University Hospital Wurzburg, Wurzburg, Germany.

Steinke, Maria. Fraunhofer Institute for Silicate Research ISC, Wurzburg, Germany.

Hackenberg, Stephan. Department of Oto-Rhino-Laryngology, Plastic, Aesthetic & Reconstructive Head and Neck Surgery, University Hospital Wurzburg, Wurzburg, Germany.

Hackenberg, Stephan. Department of Otorhinolaryngology - Head and Neck Surgery, RWTH Aachen University Hospital, Aachen, Germany.

MeSH Heading

Adult. Aged. Aged, 80 and over. Discriminant Analysis. Female. Humans. Male. Middle Aged. Paraffin Embedding. Pilot Projects. Principal Component Analysis. *Salivary Gland Neoplasms/ch [Chemistry]. Salivary Gland Neoplasms/pa [Pathology]. *Spectrum Analysis, Raman.

Year of Publication

2021

Link to the Ovid Full Text or citation:

[Click here for full text options](https://ovidsp.ovid.com/ovidweb.cgi?T=JS&CSC=Y&NEWS=N&PAGE=fulltext&D=med20&AN=34529739)

Link to the External Link Resolver:

[SFX](https://sfx-86scu.hosted.exlibrisgroup.com.cn/86scu?sid=OVID:medline&id=pmid:34529739&id=doi:10.1371%2Fjournal.pone.0257470&issn=19326203&isbn=&volume=16&issue=9&spage=e0257470&pages=e0257470&date=2021&title=PLoS+ONE+%5BElectronic+Resource%5D&atitle=Pilot+study+on+the+value+of+Raman+spectroscopy+in+the+entity+assignment+of+salivary+gland+tumors.&aulast=Meyer&pid=<author>Meyer+TJ%3BGerhard-Hartmann+E%3BLodes+N%3BScherzad+A%3BHagen+R%3BSteinke+M%3BHackenberg+S<%2Fauthor><AN>34529739<%2FAN><DT>Journal+Article<%2FDT>)

46.

Deep Learning-Guided Fiberoptic Raman Spectroscopy Enables Real-Time In Vivo Diagnosis and Assessment of Nasopharyngeal Carcinoma and Post-treatment Efficacy during Endoscopy.

Shu C, Yan H, Zheng W, Lin K, James A, Selvarajan S, Lim CM, Huang Z

Analytical Chemistry. 93(31):10898-10906, 2021 08 10.

[Journal Article. Research Support, Non-U.S. Gov't]

UI: 34319713

In this work, we develop a deep learning-guided fiberoptic Raman diagnostic platform to assess its ability of real-time in vivo nasopharyngeal carcinoma (NPC) diagnosis and post-treatment follow-up of NPC patients. The robust Raman diagnostic platform is established using innovative multi-layer Raman-specified convolutional neural networks (RS-CNN) together with simultaneous fingerprint and high-wavenumber spectra acquired within sub-seconds using a fiberoptic Raman endoscopy system. We have acquired a total of 15,354 FP/HW in vivo Raman spectra (control: 1761; NPC: 4147; and post-treatment (PT): 9446) from 888 tissue sites of 418 subjects (healthy control: 85; NPC: 82; and PT: 251) during endoscopic examination. The optimized RS-CNN model provides an overall diagnostic accuracy of 82.09% (sensitivity of 92.18% and specificity of 73.99%) for identifying NPC from control and post-treatment patients, which is superior to the best diagnosis performance (accuracy of 73.57%; sensitivity of 89.74%; and specificity of 58.10%) using partial-least-squares linear-discriminate-analysis, proving the robustness and high spectral information sensitiveness of the RS-CNN model developed. We further investigate the saliency map of the best RS-CNN models using the correctly predicted Raman spectra. The specific Raman signatures that are related to the cancer-associated biomolecular variations (e.g., collagens, lipids, and nucleic acids) are uncovered in the map, validating the diagnostic capability of RS-CNN models to correlate with biomolecular signatures. Deep learning-based Raman spectroscopy is a powerful diagnostic tool for rapid screening and surveillance of NPC patients and can also be deployed for longitudinal follow-up monitoring of post-treatment NPC patients to detect early cancer recurrences in the head and neck.

Version ID

1

Record Owner

From MEDLINE, a database of the U.S. National Library of Medicine.

Status

MEDLINE

Author NameID

Huang, Zhiwei; ORCID: <https://orcid.org/0000-0002-0104-9135>

Authors Full Name

Shu, Chi, Yan, Hanshu, Zheng, Wei, Lin, Kan, James, Anne, Selvarajan, Sathiyamoorthy, Lim, Chwee Ming, Huang, Zhiwei

Institution

Shu, Chi. Optical Bioimaging Laboratory, Department of Biomedical Engineering, Faculty of Engineering, National University of Singapore, Singapore 117576, Singapore. Yan, Hanshu. Department of Electrical and Computer Engineering, Faculty of Engineering, National University of Singapore, Singapore 117583, Singapore.

Zheng, Wei. Optical Bioimaging Laboratory, Department of Biomedical Engineering, Faculty of Engineering, National University of Singapore, Singapore 117576, Singapore.

Lin, Kan. Optical Bioimaging Laboratory, Department of Biomedical Engineering, Faculty of Engineering, National University of Singapore, Singapore 117576, Singapore.

James, Anne. Department of Anatomical Pathology, Singapore General Hospital, Singapore 169856, Singapore.

Selvarajan, Sathiyamoorthy. Department of Anatomical Pathology, Singapore General Hospital, Singapore 169856, Singapore.

Lim, Chwee Ming. Department of Otolaryngology, Duke-NUS Graduate Medical School, Singapore General Hospital, Singapore 169608, Singapore.

Huang, Zhiwei. Optical Bioimaging Laboratory, Department of Biomedical Engineering, Faculty of Engineering, National University of Singapore, Singapore 117576, Singapore.

MeSH Heading

*Deep Learning. Endoscopy. Humans. Nasopharyngeal Carcinoma/dg [Diagnostic Imaging]. Nasopharyngeal Neoplasms/dg [Diagnostic Imaging]. *Nasopharyngeal Neoplasms. Spectrum Analysis, Raman. Treatment Outcome.

Year of Publication

2021

Link to the Ovid Full Text or citation:

[Click here for full text options](https://ovidsp.ovid.com/ovidweb.cgi?T=JS&CSC=Y&NEWS=N&PAGE=fulltext&D=med20&AN=34319713)

Link to the External Link Resolver:

[SFX](https://sfx-86scu.hosted.exlibrisgroup.com.cn/86scu?sid=OVID:medline&id=pmid:34319713&id=doi:10.1021%2Facs.analchem.1c01559&issn=00032700&isbn=&volume=93&issue=31&spage=10898&pages=10898-10906&date=2021&title=Analytical+Chemistry&atitle=Deep+Learning-Guided+Fiberoptic+Raman+Spectroscopy+Enables+Real-Time+In+Vivo+Diagnosis+and+Assessment+of+Nasopharyngeal+Carcinoma+and+Post-treatment+Efficacy+during+Endoscopy.&aulast=Shu&pid=<author>Shu+C%3BYan+H%3BZheng+W%3BLin+K%3BJames+A%3BSelvarajan+S%3BLim+CM%3BHuang+Z<%2Fauthor><AN>34319713<%2FAN><DT>Journal+Article<%2FDT>)

47.

Imaging of Oral SCC Cells by Raman Micro-Spectroscopy Technique.

Kinoshita H, Miyoshi N, Ogasawara T

Molecules. 26(12), 2021 Jun 15.

[Journal Article]

UI: 34203597

We used Raman micro-spectroscopy technique to analyze the molecular changes associated with oral squamous cell carcinoma (SCC) cells in the form of frozen tissue. Previously, Raman micro-spectroscopy technique on human tissue was mainly based on spectral analysis, but we worked on imaging of molecular structure. In this study, we evaluated the distribution of four components at the cell level (about 10 mum) to describe the changes in protein and molecular structures of protein belonging to malignant tissue. We analyzed ten oral SCC samples of five patients without special pretreatments of the use of formaldehyde. We obtained cell level images of the oral SCC cells at various components (peak at 935 cm-1: proline and valine, 1004 cm-1: phenylalanine, 1223 cm-1: nucleic acids, and 1650 cm-1: amide I). These mapping images of SCC cells showed the distribution of nucleic acids in the nuclear areas; meanwhile, proline and valine, phenylalanine, and amide I were detected in the cytoplasm areas of the SCC cells. Furthermore, the peak of amide I in the cancer area shifts to the higher wavenumber side, which indicates the alpha-helix component may decrease in its relative amounts of protein in the beta-sheet or random coil conformation. Imaging of SCC cells with Raman micro-spectroscopy technique indicated that such a new observation of cancer cells is useful for analyzing the detailed distribution of various molecular conformation within SCC cells.

Version ID

1

Record Owner

From MEDLINE, a database of the U.S. National Library of Medicine.

Status

MEDLINE

Authors Full Name

Kinoshita, Hidetaka, Miyoshi, Norio, Ogasawara, Toshiyuki

Institution

Kinoshita, Hidetaka. Division of Dentistry and Oral Surgery, Fukui General Hospital, Egami, Fukui 910-8561, Japan. Kinoshita, Hidetaka. Department of Rehabilitation Medicine, Fukui College of Health Sciences, Egami, Fukui 910-3190, Japan.

Miyoshi, Norio. Department of Gastroenterology, Faculty of Medicine, Tsukuba University, Tennoudai, Tsukuba 305-8575, Japan.

Ogasawara, Toshiyuki. Division of Dentistry and Oral Surgery, Fukui General Hospital, Egami, Fukui 910-8561, Japan.

MeSH Heading

Carcinoma, Squamous Cell/dg [Diagnostic Imaging]. Carcinoma, Squamous Cell/pa [Pathology]. Cell Nucleus/me [Metabolism]. Cytoplasm/me [Metabolism]. Diagnostic Imaging/mt [Methods]. Humans. Image Processing, Computer-Assisted/mt [Methods]. Japan. Molecular Conformation. Mouth Neoplasms/dg [Diagnostic Imaging]. Mouth Neoplasms/me [Metabolism]. *Spectrum Analysis, Raman/mt [Methods]. *Squamous Cell Carcinoma of Head and Neck/dg [Diagnostic Imaging]. Squamous Cell Carcinoma of Head and Neck/me [Metabolism]. Squamous Cell Carcinoma of Head and Neck/pa [Pathology].

Keyword Heading

Raman micro-spectroscopy technique mapping images

squamous cell carcinoma (SCC).

Keyword Heading Owner

NOTNLM

Year of Publication

2021

Link to the Ovid Full Text or citation:

[Click here for full text options](https://ovidsp.ovid.com/ovidweb.cgi?T=JS&CSC=Y&NEWS=N&PAGE=fulltext&D=med20&AN=34203597)

Link to the External Link Resolver:

[SFX](https://sfx-86scu.hosted.exlibrisgroup.com.cn/86scu?sid=OVID:medline&id=pmid:34203597&id=doi:10.3390%2Fmolecules26123640&issn=14203049&isbn=&volume=26&issue=12&spage=3640&pages=&date=2021&title=Molecules&atitle=Imaging+of+Oral+SCC+Cells+by+Raman+Micro-Spectroscopy+Technique.&aulast=Kinoshita&pid=<author>Kinoshita+H%3BMiyoshi+N%3BOgasawara+T<%2Fauthor><AN>34203597<%2FAN><DT>Journal+Article<%2FDT>)

48.

Single cell detection using intracellularly-grown-Au-nanoparticle based surface-enhanced Raman scattering spectroscopy for nasopharyngeal cell line classification.

Chen W, Xu S, Wang X, Wei G, Hong Q, Huang H, Yu Y

Analytical Methods. 13(28):3147-3153, 2021 07 28.

[Journal Article. Research Support, Non-U.S. Gov't]

UI: 34159968

The aim of this study was to evaluate the feasibility of applying intracellularly-grown-Au-nanoparticle (IGAuNP)-based surface-enhanced Raman scattering (SERS) technology to classify two types of nasopharyngeal cancer (NPC) cell lines (CNE2 and CNE1). The IGAuNP technology provides excellent delivery efficiency of Au NPs to the cytoplasm and nucleus, thus leading to an extraordinary enhancement of the Raman signals of cells. Compared with normal Raman scattering (NRS) spectra of cells, IGAuNP-based SERS spectra not only have a high signal-to-noise ratio, but also can detect more characteristic Raman peaks, which can be used to explore more differences when comparing the biochemical components of different nasopharyngeal carcinoma cell lines. Based on the linear discriminant analysis (LDA) and support vector machine (SVM) analysis of SERS spectral data, an exciting result with a diagnostic sensitivity of 100%, specificity of 100%, and accuracy of 100%, could be achieved to differentiate CNE2 and CNE1 cells, which is better than the result obtained by NRS spectroscopy. This exploratory study indicated that the SERS technology based on IGAuNPs in conjunction with multivariate statistical analysis methods has great potential in the identification of nasopharyngeal carcinoma cell lines.

Version ID

1

Record Owner

From MEDLINE, a database of the U.S. National Library of Medicine.

Status

MEDLINE

Authors Full Name

Chen, Weiwei, Xu, Shangwen, Wang, Xiaoyang, Wei, Guoqiang, Hong, Quanxing, Huang, Hao, Yu, Yun

Institution

Chen, Weiwei. College of Integrated Traditional Chinese and Western Medicine, Fujian University of Traditional Chinese Medicine, Fuzhou, 350122, China. cfjtcm@126.com yuyunsatan@163.com.

MeSH Heading

Cell Line. Humans. *Nanoparticles. Nasopharyngeal Carcinoma/di [Diagnosis]. Nasopharyngeal Neoplasms/di [Diagnosis]. *Nasopharyngeal Neoplasms. Spectrum Analysis, Raman.

Year of Publication

2021

Link to the Ovid Full Text or citation:

[Click here for full text options](https://ovidsp.ovid.com/ovidweb.cgi?T=JS&CSC=Y&NEWS=N&PAGE=fulltext&D=med20&AN=34159968)

Link to the External Link Resolver:

[SFX](https://sfx-86scu.hosted.exlibrisgroup.com.cn/86scu?sid=OVID:medline&id=pmid:34159968&id=doi:10.1039%2Fd1ay00554e&issn=17599660&isbn=&volume=13&issue=28&spage=3147&pages=3147-3153&date=2021&title=Analytical+Methods&atitle=Single+cell+detection+using+intracellularly-grown-Au-nanoparticle+based+surface-enhanced+Raman+scattering+spectroscopy+for+nasopharyngeal+cell+line+classification.&aulast=Chen&pid=<author>Chen+W%3BXu+S%3BWang+X%3BWei+G%3BHong+Q%3BHuang+H%3BYu+Y<%2Fauthor><AN>34159968<%2FAN><DT>Journal+Article<%2FDT>)

49.

Geometrically encoded SERS nanobarcodes for the logical detection of nasopharyngeal carcinoma-related progression biomarkers.

Lin D, Hsieh CL, Hsu KC, Liao PH, Qiu S, Gong T, Yong KT, Feng S, Kong KV

Nature communications . 12(1):3430, 2021 06 08.

[Journal Article. Research Support, Non-U.S. Gov't]

UI: 34078895

The limited availability of nasopharyngeal carcinoma-related progression biomarker array kits that offer physicians comprehensive information is disadvantageous for monitoring cancer progression. To develop a biomarker array kit, systematic identification and differentiation of a large number of distinct molecular surface-enhanced Raman scattering (SERS) reporters with high spectral temporal resolution is a major challenge. To address this unmet need, we use the chemistry of metal carbonyls to construct a series of unique SERS reporters with the potential to provide logical and highly multiplex information during testing. In this study, we report that geometric control over metal carbonyls on nanotags can produce 14 distinct barcodes that can be decoded unambiguously using commercial Raman spectroscopy. These metal carbonyl nanobarcodes are tested on human blood samples and show strong sensitivity (0.07 ng/mL limit of detection, average CV of 6.1% and >92% degree of recovery) and multiplexing capabilities for MMPs.

Version ID

1

Record Owner

From MEDLINE, a database of the U.S. National Library of Medicine.

Status

MEDLINE

Author NameID

Lin, Duo; ORCID: <https://orcid.org/0000-0001-6959-5995> Gong, Tianxun; ORCID: <https://orcid.org/0000-0001-9383-3666>

Authors Full Name

Lin, Duo, Hsieh, Chang-Lin, Hsu, Keng-Chia, Liao, Pei-Hsuan, Qiu, Sufang, Gong, Tianxun, Yong, Ken-Tye, Feng, Shangyuan, Kong, Kien Voon

Institution

Lin, Duo. Key Laboratory of OptoElectronic Science and Technology for Medicine, Ministry of Education, Fujian Provincial Key Laboratory for Photonics Technology, Fujian Normal University, Fuzhou, Fujian, China. Hsieh, Chang-Lin. Department of Chemistry, National Taiwan University, Taipei, Taiwan.

Hsu, Keng-Chia. Department of Chemistry, National Taiwan University, Taipei, Taiwan.

Liao, Pei-Hsuan. Department of Chemistry, National Taiwan University, Taipei, Taiwan.

Qiu, Sufang. Fujian Medical University Cancer Hospital, Fujian Cancer Hospital, Fuzhou, Fujian, China.

Gong, Tianxun. State Key Laboratory of Electronic Thin Films and Integrated Devices, School of Electronic Science and Engineering (National Exemplary School of Microelectronics), University of Electronic Science and Technology of China, Chengdu, China.

Yong, Ken-Tye. School of Biomedical Engineering, The University of Sydney, Sydney, NSW, Australia.

Yong, Ken-Tye. The University of Sydney Nano Institute, The University of Sydney, Sydney, NSW, Australia.

Feng, Shangyuan. Key Laboratory of OptoElectronic Science and Technology for Medicine, Ministry of Education, Fujian Provincial Key Laboratory for Photonics Technology, Fujian Normal University, Fuzhou, Fujian, China.

Kong, Kien Voon. Department of Chemistry, National Taiwan University, Taipei, Taiwan. kvkong@ntu.edu.tw.

MeSH Heading

Biomarkers, Tumor/bl [Blood]. Biomarkers, Tumor/ch [Chemistry]. *Biosensing Techniques/mt [Methods]. Disease Progression. Matrix Metalloproteinases/bl [Blood]. Matrix Metalloproteinases/ch [Chemistry]. Metal Nanoparticles/ch [Chemistry]. Nanogels/ch [Chemistry]. Nasopharyngeal Carcinoma/bl [Blood]. *Nasopharyngeal Carcinoma/di [Diagnosis]. Nasopharyngeal Carcinoma/pa [Pathology]. Nasopharyngeal Neoplasms/bl [Blood]. *Nasopharyngeal Neoplasms/di [Diagnosis]. Nasopharyngeal Neoplasms/pa [Pathology]. Organometallic Compounds/ch [Chemistry]. Sensitivity and Specificity. *Spectrum Analysis, Raman. Surface Properties.

Registry Number/Name of Substance

0 (Biomarkers, Tumor). 0 (Nanogels). 0 (Organometallic Compounds). EC 3-4-24 (Matrix Metalloproteinases).

Year of Publication

2021

Link to the Ovid Full Text or citation:

[Click here for full text options](https://ovidsp.ovid.com/ovidweb.cgi?T=JS&CSC=Y&NEWS=N&PAGE=fulltext&D=med20&AN=34078895)

Link to the External Link Resolver:

[SFX](https://sfx-86scu.hosted.exlibrisgroup.com.cn/86scu?sid=OVID:medline&id=pmid:34078895&id=doi:10.1038%2Fs41467-021-23789-3&issn=20411723&isbn=&volume=12&issue=1&spage=3430&pages=3430&date=2021&title=Nature+communications+&atitle=Geometrically+encoded+SERS+nanobarcodes+for+the+logical+detection+of+nasopharyngeal+carcinoma-related+progression+biomarkers.&aulast=Lin&pid=<author>Lin+D%3BHsieh+CL%3BHsu+KC%3BLiao+PH%3BQiu+S%3BGong+T%3BYong+KT%3BFeng+S%3BKong+KV<%2Fauthor><AN>34078895<%2FAN><DT>Journal+Article<%2FDT>)

50.

Label-free electrochemical immunosensor based on gold nanoparticle/polyethyleneimine/reduced graphene oxide nanocomposites for the ultrasensitive detection of cancer biomarker matrix metalloproteinase-1.

Liu X, Lin LY, Tseng FY, Tan YC, Li J, Feng L, Song L, Lai CF, Li X, He JH, Sakthivel R, Chung RJ

Analyst. 146(12):4066-4079, 2021 Jun 14.

[Journal Article]

UI: 34048512

Matrix metalloproteinase-1 (MMP-1) is associated with many types of cancers, including oral, colorectal, and brain cancers. This paper describes the fabrication of an MMP-1 immunosensor based on a gold nanoparticle/polyethyleneimine/reduced graphene oxide (AuNP/PEI/rGO)-modified disposable screen-printed electrode (SPE). A microwave-assisted single-step method was employed for the simultaneous reduction of gold and graphene oxide in a PEI environment to avoid AuNP agglomeration. The crystal structure, chemical composition, optical properties, and interior morphology of the materials were probed by X-ray diffraction, Raman spectroscopy, UV-visible spectrometry, and transmission electron microscopy techniques. To assemble a label-free MMP-1 immunosensor layer-by-layer, 3-mercaptopropionic acid was utilized due to its strong sulfur-gold bonding ability, and its tail end was attached to a carboxyl group, allowing the MMP-1 antibody (anti-MMP-1) to be subsequently cross-linked using the traditional N-(3-dimethylaminopropyl) and N' ethylcarbodiimide hydrochloride method. Differential pulse voltammetry analysis showed a linear relationship with MMP-1 concentration in the range of 1-50 ng ml-1 with an R2 value of ~0.996 (n = 5, RSD < 5%). This immunosensor was successfully applied for MMP-1 detection in urine, saliva, bovine serum, and cell culture media (HSC-3 & C6) of oral and brain cancers showing results comparable to those of the credible ELISA method.

Version ID

1

Record Owner

From MEDLINE, a database of the U.S. National Library of Medicine.

Status

MEDLINE

Author NameID

Chung, Ren-Jei; ORCID: <http://orcid.org/0000-0002-0655-3680>

Authors Full Name

Liu, Xinke, Lin, Lu-Yin, Tseng, Fu-Yen, Tan, Yu-Cheng, Li, Jian, Feng, Li, Song, Lijun, Lai, Chih-Fang, Li, Xiaohua, He, Jr-Hau, Sakthivel, Rajalakshmi, Chung, Ren-Jei

Institution

Liu, Xinke. College of Materials Science and Engineering, Shenzhen University, Shenzhen 518060, China. lxh@szu.edu.cn and Department of Electrical and Computer Engineering, National University of Singapore, Singapore 117583, Singapore. Lin, Lu-Yin. Department of Chemical Engineering and Biotechnology, National Taipei University of Technology (Taipei Tech), Taipei 10608, Taiwan. rjchung@ntut.edu.tw rajalakshmicnr@gmail.com.

Tseng, Fu-Yen. Department of Chemical Engineering and Biotechnology, National Taipei University of Technology (Taipei Tech), Taipei 10608, Taiwan. rjchung@ntut.edu.tw rajalakshmicnr@gmail.com.

Tan, Yu-Cheng. Department of Chemical Engineering and Biotechnology, National Taipei University of Technology (Taipei Tech), Taipei 10608, Taiwan. rjchung@ntut.edu.tw rajalakshmicnr@gmail.com.

Li, Jian. College of Materials Science and Engineering, Shenzhen University, Shenzhen 518060, China. lxh@szu.edu.cn.

Feng, Li. College of Materials Science and Engineering, Shenzhen University, Shenzhen 518060, China. lxh@szu.edu.cn.

Song, Lijun. Research Center of Guangdong Intelligent Charging and System Integration Engineering Technology, Shenzhen Winsemi Microelectronics Co., Ltd, Shenzhen, 518000, China.

Lai, Chih-Fang. DFON Biomedical Technology Inc., Taipei 10608, Taiwan.

Li, Xiaohua. College of Materials Science and Engineering, Shenzhen University, Shenzhen 518060, China. lxh@szu.edu.cn.

He, Jr-Hau. Department of Chemical Engineering and Biotechnology, National Taipei University of Technology (Taipei Tech), Taipei 10608, Taiwan. rjchung@ntut.edu.tw rajalakshmicnr@gmail.com and Department of Materials Science and Engineering, City University of Hong Kong, Hong Kong. jrhauhe@cityu.edu.hk.

Sakthivel, Rajalakshmi. Department of Chemical Engineering and Biotechnology, National Taipei University of Technology (Taipei Tech), Taipei 10608, Taiwan. rjchung@ntut.edu.tw rajalakshmicnr@gmail.com.

Chung, Ren-Jei. Department of Chemical Engineering and Biotechnology, National Taipei University of Technology (Taipei Tech), Taipei 10608, Taiwan. rjchung@ntut.edu.tw rajalakshmicnr@gmail.com.

MeSH Heading

Animals. Biomarkers, Tumor. *Biosensing Techniques. Cattle. Electrochemical Techniques. Electrodes. Gold. *Graphite. Immunoassay. Limit of Detection. Matrix Metalloproteinase 1. *Metal Nanoparticles. *Nanocomposites. *Neoplasms. Polyethyleneimine.

Registry Number/Name of Substance

0 (Biomarkers, Tumor). 0 (graphene oxide). 7440-57-5 (Gold). 7782-42-5 (Graphite). 9002-98-6 (Polyethyleneimine). EC 3-4-24-7 (Matrix Metalloproteinase 1).

Year of Publication

2021

Link to the Ovid Full Text or citation:

[Click here for full text options](https://ovidsp.ovid.com/ovidweb.cgi?T=JS&CSC=Y&NEWS=N&PAGE=fulltext&D=med20&AN=34048512)

Link to the External Link Resolver:

[SFX](https://sfx-86scu.hosted.exlibrisgroup.com.cn/86scu?sid=OVID:medline&id=pmid:34048512&id=doi:10.1039%2Fd1an00537e&issn=00032654&isbn=&volume=146&issue=12&spage=4066&pages=4066-4079&date=2021&title=Analyst&atitle=Label-free+electrochemical+immunosensor+based+on+gold+nanoparticle%2Fpolyethyleneimine%2Freduced+graphene+oxide+nanocomposites+for+the+ultrasensitive+detection+of+cancer+biomarker+matrix+metalloproteinase-1.&aulast=Liu&pid=<author>Liu+X%3BLin+LY%3BTseng+FY%3BTan+YC%3BLi+J%3BFeng+L%3BSong+L%3BLai+CF%3BLi+X%3BHe+JH%3BSakthivel+R%3BChung+RJ<%2Fauthor><AN>34048512<%2FAN><DT>Journal+Article<%2FDT>)

51.

Spectroscopic, quantum chemical and molecular docking studies on 1-amino-5-chloroanthraquinone: A targeted drug therapy for thyroid cancer.

Valarmathi T, Premkumar R, Meera MR, Milton Franklin Benial A

Spectrochimica Acta. Part A, Molecular & Biomolecular Spectroscopy. 255:119659, 2021 Jul 05.

[Journal Article]

UI: 33751957

The DFT studies of the 1-Amino-5-chloro-anthraquinone (ACAQ) molecule have been carried out with extensive and accurate investigations of detailed vibrational and spectroscopic investigations and validated by experimentally. The optimized molecular structure and harmonic resonance frequencies were computed based on DFT/B3LYP method with 6-311G++(d,p) basis set using the Gaussian 09 program. The experimental and calculated vibrational wavenumbers were assigned on the basis of PED calculations using VEDA 4.0 program. The 13C NMR isotropic chemical shifts of the molecule were calculated using Gauge-Invariant-Atomic Orbital (GIAO) method in DMSO solution and compared with the experimental data. The absorption spectrum of the molecule was computed in liquid phase (ethanol), which exhibits to * electronic transition and compared with observed UV-Vis spectrum. Frontier molecular orbitals analysis shows the molecular reactivity and kinetic stability of the molecule. The Mulliken atomic charge distribution and molecular electrostatic potential surface analysis of the molecule validate the reactive site of the molecule. The natural bond orbital analysis proves the bioactivity of the molecule. Molecular docking analysis indicate that ACAQ molecule inhibits the action of c-Met Kinase protein, which is associated with the thyroid cancer. Hence, the present study pave the way for the development of novel drugs in the treatment of thyroid cancer.

Copyright © 2021 Elsevier B.V. All rights reserved.

Version ID

1

Record Owner

From MEDLINE, a database of the U.S. National Library of Medicine.

Status

MEDLINE

Authors Full Name

Valarmathi, T, Premkumar, R, Meera, M R, Milton Franklin Benial, A

Institution

Valarmathi, T. P.G. and Research Department of Physics, N.M.S.S.V.N. College, Madurai 625019, Tamil Nadu, India. Premkumar, R. P.G. and Research Department of Physics, N.M.S.S.V.N. College, Madurai 625019, Tamil Nadu, India.

Meera, M R. Department of Physics, Sree Ayyappa College for Women, Chunkankadai, Kanyakumari 629003, Tamil Nadu, India.

Milton Franklin Benial, A. P.G. and Research Department of Physics, N.M.S.S.V.N. College, Madurai 625019, Tamil Nadu, India. Electronic address: miltonfranklin@yahoo.com.

MeSH Heading

Humans. Models, Molecular. Molecular Conformation. Molecular Docking Simulation. *Pharmaceutical Preparations. Quantum Theory. Spectrophotometry, Ultraviolet. Spectroscopy, Fourier Transform Infrared. Spectrum Analysis, Raman. Thermodynamics. *Thyroid Neoplasms.

Keyword Heading

1-Amino-5-chloro-anthraquinone DFT

FT-IR

FT-NMR

FT-Raman

Molecular docking

Thyroid cancer.

Keyword Heading Owner

NOTNLM

Registry Number/Name of Substance

0 (Pharmaceutical Preparations).

Year of Publication

2021

Link to the Ovid Full Text or citation:

[Click here for full text options](https://ovidsp.ovid.com/ovidweb.cgi?T=JS&CSC=Y&NEWS=N&PAGE=fulltext&D=med19&AN=33751957)

Link to the External Link Resolver:

[SFX](https://sfx-86scu.hosted.exlibrisgroup.com.cn/86scu?sid=OVID:medline&id=pmid:33751957&id=doi:10.1016%2Fj.saa.2021.119659&issn=13861425&isbn=&volume=255&issue=&spage=119659&pages=119659&date=2021&title=Spectrochimica+Acta.+Part+A%2C+Molecular+%26+Biomolecular+Spectroscopy&atitle=Spectroscopic%2C+quantum+chemical+and+molecular+docking+studies+on+1-amino-5-chloroanthraquinone%3A+A+targeted+drug+therapy+for+thyroid+cancer.&aulast=Valarmathi&pid=<author>Valarmathi+T%3BPremkumar+R%3BMeera+MR%3BMilton+Franklin+Benial+A<%2Fauthor><AN>33751957<%2FAN><DT>Journal+Article<%2FDT>)

52.

Risk prediction by Raman spectroscopy for disease-free survival in oral cancers.

Bhattacharjee A, Hole A, Malik A, Sahu A, Singh SP, Deshmukh A, Nair S, Chaturvedi P, Murali Krishna C

Lasers in Medical Science. 36(8):1691-1700, 2021 Oct.

[Journal Article]

UI: 33661401

In the present study, the potential of Raman spectroscopy (RS) in predicting disease-free survival (DFS) in oral cancer patients has been explored. Raman spectra were obtained from the tumor and contralateral regions of 94 oral squamous cell carcinoma patients. These patients were managed surgically and recommended for adjuvant therapy. The Cox proportional survival analysis was carried out to identify the spectral regions that can be correlated to DFS. The survival analysis was performed with 95% confidence intervals, hazard ratio, and p-values in the 1200-1800 cm-1 spectral region. Out of a total of 182 spectral points, 76 were found to be correlating with DFS, suggesting their utility to predict the patient outcome. The cut-off points of each correlating RS-point values were defined and tested towards predicting the DFS. The performance of predicting the power of spectral points was validated through Brier value, and it was found to be closer to the actual progression. The 76 spectral points identified from the tumors have the potential to accurately predict DFS in oral squamous cell carcinoma through a relatively simplistic prediction model in the absence of confounding factors.

Copyright © 2021. The Author(s), under exclusive licence to Springer-Verlag London Ltd. part of Springer Nature.

Version ID

1

Record Owner

From MEDLINE, a database of the U.S. National Library of Medicine.

Status

MEDLINE

Author NameID

Murali Krishna, C; ORCID: <http://orcid.org/0000-0002-4974-8533>

Authors Full Name

Bhattacharjee, Atanu, Hole, Arti, Malik, Akshat, Sahu, Aditi, Singh, S P, Deshmukh, Atul, Nair, Sudhir, Chaturvedi, Pankaj, Murali Krishna, C

Institution

Bhattacharjee, Atanu. Section of Biostatistics, Centre for Cancer Epidemiology, Tata Memorial Centre, Mumbai, India. Bhattacharjee, Atanu. Homi Bhabha National Institute, Mumbai, India.

Hole, Arti. Chilakapati Laboratory, Advanced Center for Training, Research, and Education in Cancer (ACTREC), Tata Memorial Center, Kharghar, Sector '22', Navi Mumbai, 410210, India.

Malik, Akshat. Head and Neck Surgical Oncology, Tata Memorial Center, Mumbai, India.

Malik, Akshat. Department of Head Neck Surgery, Tata Memorial Centre, Mumbai, India.

Sahu, Aditi. Chilakapati Laboratory, Advanced Center for Training, Research, and Education in Cancer (ACTREC), Tata Memorial Center, Kharghar, Sector '22', Navi Mumbai, 410210, India.

Singh, S P. Chilakapati Laboratory, Advanced Center for Training, Research, and Education in Cancer (ACTREC), Tata Memorial Center, Kharghar, Sector '22', Navi Mumbai, 410210, India.

Deshmukh, Atul. Chilakapati Laboratory, Advanced Center for Training, Research, and Education in Cancer (ACTREC), Tata Memorial Center, Kharghar, Sector '22', Navi Mumbai, 410210, India.

Nair, Sudhir. Head and Neck Surgical Oncology, Tata Memorial Center, Mumbai, India.

Nair, Sudhir. Department of Head Neck Surgery, Tata Memorial Centre, Mumbai, India.

Chaturvedi, Pankaj. Section of Biostatistics, Centre for Cancer Epidemiology, Tata Memorial Centre, Mumbai, India.

Chaturvedi, Pankaj. Head and Neck Surgical Oncology, Tata Memorial Center, Mumbai, India.

Chaturvedi, Pankaj. Department of Head Neck Surgery, Tata Memorial Centre, Mumbai, India.

Murali Krishna, C. Chilakapati Laboratory, Advanced Center for Training, Research, and Education in Cancer (ACTREC), Tata Memorial Center, Kharghar, Sector '22', Navi Mumbai, 410210, India. mchilakapati@actrec.gov.in.

Murali Krishna, C. Head and Neck Surgical Oncology, Tata Memorial Center, Mumbai, India. mchilakapati@actrec.gov.in.

MeSH Heading

*Carcinoma, Squamous Cell. Disease-Free Survival. Humans. *Mouth Neoplasms. Prognosis. Proportional Hazards Models. Retrospective Studies. Spectrum Analysis, Raman.

Keyword Heading

Cox Disease-free survival

Oral cancer

PH

Raman spectroscopy.

Keyword Heading Owner

NOTNLM

Year of Publication

2021

Link to the Ovid Full Text or citation:

[Click here for full text options](https://ovidsp.ovid.com/ovidweb.cgi?T=JS&CSC=Y&NEWS=N&PAGE=fulltext&D=med19&AN=33661401)

Link to the External Link Resolver:

[SFX](https://sfx-86scu.hosted.exlibrisgroup.com.cn/86scu?sid=OVID:medline&id=pmid:33661401&id=doi:10.1007%2Fs10103-021-03276-3&issn=02688921&isbn=&volume=36&issue=8&spage=1691&pages=1691-1700&date=2021&title=Lasers+in+Medical+Science&atitle=Risk+prediction+by+Raman+spectroscopy+for+disease-free+survival+in+oral+cancers.&aulast=Bhattacharjee&pid=<author>Bhattacharjee+A%3BHole+A%3BMalik+A%3BSahu+A%3BSingh+SP%3BDeshmukh+A%3BNair+S%3BChaturvedi+P%3BMurali+Krishna+C<%2Fauthor><AN>33661401<%2FAN><DT>Journal+Article<%2FDT>)

53.

Rapid and noninvasive diagnosis of oral and oropharyngeal cancer based on micro-Raman and FT-IR spectra of saliva.

Falamas A, Faur CI, Ciupe S, Chirila M, Rotaru H, Hedesiu M, Cinta Pinzaru S

Spectrochimica Acta. Part A, Molecular & Biomolecular Spectroscopy. 252:119477, 2021 May 05.

[Journal Article]

UI: 33545509

Fast, sensitive, and noninvasive techniques are needed for better health care management, particularly when traditional biopsies could be replaced with appropriate analyses of body fluids, such as saliva. Here is presented a proof-of-concept study, which aims to test a recently developed saliva samples preparation method, for oral and oropharyngeal cancer diagnosis, using micro-Raman and Fourier transform infrared (FT-IR) spectroscopic techniques. The detected biomarker bands and the cancer classification rates are compared and discussed. Saliva samples were collected from healthy donors and pathologically confirmed oral and oropharyngeal cancer patients. Principal components analysis (PCA) and principal components analysis-linear discriminant analysis (PCA-LDA) chemometric methods were applied to build discrimination models for the test and control groups. Based on the differences between salivary spectra of healthy and cancer patients, several biomarker bands were identified. Noteworthy, a significant vibrational biomarker band at 2064cm-1, assigned to thiocyanate, was observed in both the FT-IR and Raman data-set. Other cancer characteristic Raman bands were 754cm-1 (tryptophan), 530 and 927cm-1 (lysozyme), 1001cm-1 (phenylalanine), while the FT-IR biomarker band was located at 1075cm-1 (phosphodiester bonds stretching in DNA, RNA). The oral and oropharyngeal cancer was classified with an accuracy of 90% based on the micro-Raman data and 82% based on the FT-IR data set, respectively. The study showed that oral and oropharyngeal cancer can be differentiated from control saliva samples based on their respective micro-Raman and FT-IR spectral signatures, due to the biomolecular modifications induced by the disease.

Copyright © 2021 Elsevier B.V. All rights reserved.

Version ID

1

Record Owner

From MEDLINE, a database of the U.S. National Library of Medicine.

Status

MEDLINE

Authors Full Name

Falamas, A, Faur, C I, Ciupe, S, Chirila, M, Rotaru, H, Hedesiu, M, Cinta Pinzaru, S

Institution

Falamas, A. Molecular and Biomolecular Physics, National Institute for Research and Development of Isotopic and Molecular Technologies, Cluj-Napoca, Romania. Electronic address: alexandra.falamas@itim-cj.ro. Faur, C I. Department of Maxillofacial Surgery and Radiology, "Iuliu Hatieganu" University of Medicine and Pharmacy, Cluj-Napoca, Romania.

Ciupe, S. Molecular and Biomolecular Physics, National Institute for Research and Development of Isotopic and Molecular Technologies, Cluj-Napoca, Romania.

Chirila, M. Department of ENT, "Iuliu Hatieganu" University of Medicine and Pharmacy, Cluj-Napoca, Romania.

Rotaru, H. Department of Maxillofacial Surgery and Radiology, "Iuliu Hatieganu" University of Medicine and Pharmacy, Cluj-Napoca, Romania.

Hedesiu, M. Department of Maxillofacial Surgery and Radiology, "Iuliu Hatieganu" University of Medicine and Pharmacy, Cluj-Napoca, Romania.

Cinta Pinzaru, S. Biomolecular Physics Department, Faculty of Physics, Babes-Bolyai University, Cluj-Napoca, Romania. Electronic address: simona.cinta@phys.ubbcluj.ro.

MeSH Heading

Discriminant Analysis. Fourier Analysis. Humans. Oropharyngeal Neoplasms/di [Diagnosis]. *Oropharyngeal Neoplasms. *Saliva. *Spectroscopy, Fourier Transform Infrared. Spectrum Analysis, Raman.

Keyword Heading

Fourier transform infrared (FT-IR) Micro-Raman spectroscopy

Oral cancer

Oropharyngeal cancer

Saliva diagnosis.

Keyword Heading Owner

NOTNLM

Year of Publication

2021

Link to the Ovid Full Text or citation:

[Click here for full text options](https://ovidsp.ovid.com/ovidweb.cgi?T=JS&CSC=Y&NEWS=N&PAGE=fulltext&D=med19&AN=33545509)

Link to the External Link Resolver:

[SFX](https://sfx-86scu.hosted.exlibrisgroup.com.cn/86scu?sid=OVID:medline&id=pmid:33545509&id=doi:10.1016%2Fj.saa.2021.119477&issn=13861425&isbn=&volume=252&issue=&spage=119477&pages=119477&date=2021&title=Spectrochimica+Acta.+Part+A%2C+Molecular+%26+Biomolecular+Spectroscopy&atitle=Rapid+and+noninvasive+diagnosis+of+oral+and+oropharyngeal+cancer+based+on+micro-Raman+and+FT-IR+spectra+of+saliva.&aulast=Falamas&pid=<author>Falamas+A%3BFaur+CI%3BCiupe+S%3BChirila+M%3BRotaru+H%3BHedesiu+M%3BCinta+Pinzaru+S<%2Fauthor><AN>33545509<%2FAN><DT>Journal+Article<%2FDT>)

54.

Biomedical applications of vibrational spectroscopy: Oral cancer diagnostics. [Review]

Byrne HJ, Behl I, Calado G, Ibrahim O, Toner M, Galvin S, Healy CM, Flint S, Lyng FM

Spectrochimica Acta. Part A, Molecular & Biomolecular Spectroscopy. 252:119470, 2021 May 05.

[Journal Article. Review]

UI: 33503511

Vibrational spectroscopy, based on either infrared absorption or Raman scattering, has attracted increasing attention for biomedical applications. Proof of concept explorations for diagnosis of oral potentially malignant disorders and cancer are reviewed, and recent advances critically appraised. Specific examples of applications of Raman microspectroscopy for analysis of histological, cytological and saliva samples are presented for illustrative purposes, and the future prospects, ultimately for routine, chairside in vivo screening are discussed.

Copyright © 2021 Elsevier B.V. All rights reserved.

Version ID

1

Record Owner

From MEDLINE, a database of the U.S. National Library of Medicine.

Status

MEDLINE

Authors Full Name

Byrne, Hugh J, Behl, Isha, Calado, Genecy, Ibrahim, Ola, Toner, Mary, Galvin, Sheila, Healy, Claire M, Flint, Stephen, Lyng, Fiona M

Institution

Byrne, Hugh J. FOCAS Research Institute, Technological University Dublin, City Campus, Dublin 8, Ireland. Electronic address: Hugh.Byrne@TUDublin.ie. Behl, Isha. School of Physics and Clinical and Optometric Sciences, Technological University Dublin, City Campus, Dublin 8, Ireland; Radiation and Environmental Science Centre, FOCAS Research Institute, Technological University Dublin, City Campus, Dublin 8, Ireland.

Calado, Genecy. School of Physics and Clinical and Optometric Sciences, Technological University Dublin, City Campus, Dublin 8, Ireland; Radiation and Environmental Science Centre, FOCAS Research Institute, Technological University Dublin, City Campus, Dublin 8, Ireland.

Ibrahim, Ola. School of Dental Science, Trinity College Dublin, Lincoln Place, Dublin 2, Ireland.

Toner, Mary. Central Pathology Laboratory, St. James Hospital, James Street, Dublin 8, Ireland.

Galvin, Sheila. Oral Medicine Unit, Dublin Dental University Hospital, Trinity College Dublin, Lincoln Place, Dublin 2, Ireland.

Healy, Claire M. Oral Medicine Unit, Dublin Dental University Hospital, Trinity College Dublin, Lincoln Place, Dublin 2, Ireland.

Flint, Stephen. Oral Medicine Unit, Dublin Dental University Hospital, Trinity College Dublin, Lincoln Place, Dublin 2, Ireland.

Lyng, Fiona M. School of Physics and Clinical and Optometric Sciences, Technological University Dublin, City Campus, Dublin 8, Ireland; Radiation and Environmental Science Centre, FOCAS Research Institute, Technological University Dublin, City Campus, Dublin 8, Ireland.

MeSH Heading

Humans. Mouth Neoplasms/di [Diagnosis]. *Mouth Neoplasms. *Spectrum Analysis, Raman. Vibration.

Keyword Heading

Infrared spectroscopy Oral cancer

Oral pre-cancer

Potentially malignant lesions disease diagnostics

Premalignant lesions

Raman spectroscopy

Vibrational spectroscopy.

Keyword Heading Owner

NOTNLM

Year of Publication

2021

Link to the Ovid Full Text or citation:

[Click here for full text options](https://ovidsp.ovid.com/ovidweb.cgi?T=JS&CSC=Y&NEWS=N&PAGE=fulltext&D=med19&AN=33503511)

Link to the External Link Resolver:

[SFX](https://sfx-86scu.hosted.exlibrisgroup.com.cn/86scu?sid=OVID:medline&id=pmid:33503511&id=doi:10.1016%2Fj.saa.2021.119470&issn=13861425&isbn=&volume=252&issue=&spage=119470&pages=119470&date=2021&title=Spectrochimica+Acta.+Part+A%2C+Molecular+%26+Biomolecular+Spectroscopy&atitle=Biomedical+applications+of+vibrational+spectroscopy%3A+Oral+cancer+diagnostics.&aulast=Byrne&pid=<author>Byrne+HJ%3BBehl+I%3BCalado+G%3BIbrahim+O%3BToner+M%3BGalvin+S%3BHealy+CM%3BFlint+S%3BLyng+FM<%2Fauthor><AN>33503511<%2FAN><DT>Journal+Article<%2FDT>)

55.

Label-Free Follow-Up Surveying of Post-Treatment Efficacy and Recurrence in Nasopharyngeal Carcinoma Patients with Fiberoptic Raman Endoscopy.

Shu C, Zheng W, Lin K, Lim C, Huang Z

Analytical Chemistry. 93(4):2053-2061, 2021 02 02.

[Journal Article. Research Support, Non-U.S. Gov't]

UI: 33406834

Recurrent nasopharyngeal carcinoma (NPC) is the main cause of poor prognosis for NPC patients after chemo- and radiotherapies. Subsequent long-term follow-ups of post-treatment patients are crucial for the early discovery of tumor recurrence with timely intervention. Current clinical imaging methods based on tissue morphology encounter difficulties in differentiating recurrent tumors from post-treatment inflammation and fibrosis. In this work, we apply a unique fiberoptic Raman endoscopy technique to address the challenges for label-free follow-up surveying of post-treatment NPC patients and accurate detection of tumor recurrence. Significant Raman spectral differences can be observed among normal, NPC, and nonrecurring post-treatment patients. Raman endoscopy provides diagnostic accuracy of 100% for detecting recurrent NPC from early post-treatment inflammation and diagnostic accuracy of 98.21% for separating recurrent NPC from long-term post-treatment fibrosis. Further quantitative Raman modeling on in vivo nasopharyngeal tissue Raman data acquired unveils the changes of major tissue biochemicals (e.g., triolein, elastin, keratin, fibrillar collagen, and type IV collagen) associated with primary NPC and post-treatment recurrent NPC tissue compared to normal nasopharyngeal tissue. This work demonstrates that fiberoptic Raman endoscopy can be a clinically powerful diagnostic tool for rapid, label-free post-treatment surveying and recurrent tumor detection in NPC patients at the molecular level.

Version ID

1

Record Owner

From MEDLINE, a database of the U.S. National Library of Medicine.

Status

MEDLINE

Author NameID

Huang, Zhiwei; ORCID: <https://orcid.org/0000-0002-0104-9135>

Authors Full Name

Shu, Chi, Zheng, Wei, Lin, Kan, Lim, Chweeming, Huang, Zhiwei

Institution

Shu, Chi. Optical Bioimaging Laboratory, Department of Biomedical Engineering, Faculty of Engineering, National University of Singapore, 9 Engineering Drive 1, Singapore 117576. Zheng, Wei. Optical Bioimaging Laboratory, Department of Biomedical Engineering, Faculty of Engineering, National University of Singapore, 9 Engineering Drive 1, Singapore 117576.

Lin, Kan. Optical Bioimaging Laboratory, Department of Biomedical Engineering, Faculty of Engineering, National University of Singapore, 9 Engineering Drive 1, Singapore 117576.

Lim, Chweeming. Department of Otolaryngology, Singapore General Hospital, Duke-NUS Graduate Medical School, Singapore 169608.

Huang, Zhiwei. Optical Bioimaging Laboratory, Department of Biomedical Engineering, Faculty of Engineering, National University of Singapore, 9 Engineering Drive 1, Singapore 117576.

MeSH Heading

Case-Control Studies. *Endoscopy/is [Instrumentation]. *Endoscopy/mt [Methods]. *Fiber Optic Technology. Follow-Up Studies. Humans. Nasopharyngeal Carcinoma/pa [Pathology]. *Nasopharyngeal Carcinoma/th [Therapy]. Nasopharyngeal Neoplasms/pa [Pathology]. *Nasopharyngeal Neoplasms/th [Therapy]. *Spectrum Analysis, Raman.

Year of Publication

2021

Link to the Ovid Full Text or citation:

[Click here for full text options](https://ovidsp.ovid.com/ovidweb.cgi?T=JS&CSC=Y&NEWS=N&PAGE=fulltext&D=med19&AN=33406834)

Link to the External Link Resolver:

[SFX](https://sfx-86scu.hosted.exlibrisgroup.com.cn/86scu?sid=OVID:medline&id=pmid:33406834&id=doi:10.1021%2Facs.analchem.0c03778&issn=00032700&isbn=&volume=93&issue=4&spage=2053&pages=2053-2061&date=2021&title=Analytical+Chemistry&atitle=Label-Free+Follow-Up+Surveying+of+Post-Treatment+Efficacy+and+Recurrence+in+Nasopharyngeal+Carcinoma+Patients+with+Fiberoptic+Raman+Endoscopy.&aulast=Shu&pid=<author>Shu+C%3BZheng+W%3BLin+K%3BLim+C%3BHuang+Z<%2Fauthor><AN>33406834<%2FAN><DT>Journal+Article<%2FDT>)

56.

Identifying benign and malignant thyroid nodules based on blood serum surface-enhanced Raman spectroscopy.

Xia L, Lu J, Chen Z, Cui X, Chen S, Pei D

Nanomedicine. 32:102328, 2021 02.

[Journal Article. Research Support, Non-U.S. Gov't]

UI: 33181274

The aim of this study is to evaluate the feasibility of using blood serum surface-enhanced Raman spectroscopy (SERS) to identify benign and malignant thyroid nodules. Blood serum samples collected from three different groups including healthy volunteers (n=22), patients with benign nodules (n=19) and malignant nodules (n=22) were measured by SERS. The spectral analysis results demonstrate that biomolecules in serum, such as amino acids, adenine and nucleic acid bases, change differently due to the different progression of nodules. By further combining with partial least square analysis and linear discriminant analysis (PLS-LDA) method, diagnostic accuracies of 93.65% and 82.93%, sensitivities of 92.68% and 81.82% and specificities of 95.45% and 84.21% can be achieved for differentiating healthy versus thyroid nodular groups and benign versus malignant groups, respectively. The above results have suggested that the blood serum SERS technique is helpful for precise diagnosis and timely treatment for patients with thyroid nodules.

Copyright © 2020 Elsevier Inc. All rights reserved.

Version ID

1

Record Owner

From MEDLINE, a database of the U.S. National Library of Medicine.

Status

MEDLINE

Authors Full Name

Xia, Lu, Lu, Jinjing, Chen, Zhilin, Cui, Xiaoyu, Chen, Shuo, Pei, Dongmei

Institution

Xia, Lu. College of Medicine and Biological Information Engineering, Northeastern University, Shenyang, China. Lu, Jinjing. Department of Health Management & Department of Family Medicine, Shengjing Hospital of China Medical University, Shenyang, China.

Chen, Zhilin. College of Medicine and Biological Information Engineering, Northeastern University, Shenyang, China.

Cui, Xiaoyu. College of Medicine and Biological Information Engineering, Northeastern University, Shenyang, China; Key Laboratory of Intelligent Computing in Medical Image, Ministry of Education, China.

Chen, Shuo. College of Medicine and Biological Information Engineering, Northeastern University, Shenyang, China; Key Laboratory of Intelligent Computing in Medical Image, Ministry of Education, China. Electronic address: chenshuo@bmie.neu.edu.cn.

Pei, Dongmei. Department of Health Management & Department of Family Medicine, Shengjing Hospital of China Medical University, Shenyang, China. Electronic address: peidm1111@hotmail.com.

MeSH Heading

Adult. Colloids/ch [Chemistry]. Discriminant Analysis. Female. Humans. Least-Squares Analysis. Principal Component Analysis. ROC Curve. Silver/ch [Chemistry]. *Spectrum Analysis, Raman. *Thyroid Nodule/bl [Blood]. *Thyroid Nodule/dg [Diagnostic Imaging].

Keyword Heading

Blood serum Surface-enhanced Raman spectroscopy

Thyroid nodule.

Keyword Heading Owner

NOTNLM

Registry Number/Name of Substance

0 (Colloids). 3M4G523W1G (Silver).

Year of Publication

2021

Link to the Ovid Full Text or citation:

[Click here for full text options](https://ovidsp.ovid.com/ovidweb.cgi?T=JS&CSC=Y&NEWS=N&PAGE=fulltext&D=med19&AN=33181274)

Link to the External Link Resolver:

[SFX](https://sfx-86scu.hosted.exlibrisgroup.com.cn/86scu?sid=OVID:medline&id=pmid:33181274&id=doi:10.1016%2Fj.nano.2020.102328&issn=15499634&isbn=&volume=32&issue=&spage=102328&pages=102328&date=2021&title=Nanomedicine&atitle=Identifying+benign+and+malignant+thyroid+nodules+based+on+blood+serum+surface-enhanced+Raman+spectroscopy.&aulast=Xia&pid=<author>Xia+L%3BLu+J%3BChen+Z%3BCui+X%3BChen+S%3BPei+D<%2Fauthor><AN>33181274<%2FAN><DT>Journal+Article<%2FDT>)

57.

Salivary Raman Spectroscopy: Standardization of Sampling Protocols and Stratification of Healthy and Oral Cancer Subjects.

Hole A, Tyagi G, Deshmukh A, Deshpande R, Gota V, Chaturvedi P, Krishna CM

Applied Spectroscopy. 75(5):581-588, 2021 May.

[Journal Article]

UI: 33107759

Minimally invasive cancer detection using bio-fluids has been actively pursued due to practical limitations, though there are better suited noninvasive and online in vivo methods. Saliva is one such clinically informative bio-fluid that offers the advantages of easy and multiple sample collection. Despite its potential in cancer diagnostics, saliva analysis is challenging due to its heterogeneous composition. Recently, there has been an upsurge in saliva exploration using optical techniques. Forms of saliva such as precipitate and supernatant have been monitored, but this sampling method needs to be standardized due to the obvious loss of analytes in processing. In that context, present work details the comparison of four different saliva sampling methodologies, i.e., air-dried, lyophilized, pellet, and supernatant using Raman spectroscopy collected from 10 healthy samples. Composition-driven spectral features of all forms were compared and classified using principal component analysis and linear discriminant analysis. Analysis was carried out on all four groups in the first step. In the second step, groups of pellet and supernatant , and air-dried and lyophilized were analyzed. Findings suggest that pellet and supernatant exhibit discrete spectroscopic features and demonstrate high classification efficiency, which is indicative of their distinctive biochemical composition. On the other hand, air-dried and lyophilized forms showed overlapping spectral features and low classification, suggesting these forms retain majority spectroscopic features of whole saliva and are less prone to sampling losses. Thus, this study indicates air-dried and lyophilized forms may be more appropriate for saliva sampling using Raman spectroscopy providing the comprehensive information required for cancer diagnosis. Furthermore, the method was also tested for the classification of oral cancer and healthy subjects (n = 27) which yielded 90% stratification. The findings of the study indicate the utility of minimally invasive salivary Raman-based diagnostics in oral cancers.

Version ID

1

Record Owner

From MEDLINE, a database of the U.S. National Library of Medicine.

Status

MEDLINE

Author NameID

Krishna, C Murali; ORCID: <https://orcid.org/0000-0002-4974-8533>

Authors Full Name

Hole, Arti, Tyagi, Gunjan, Deshmukh, Atul, Deshpande, Raviraj, Gota, Vikram, Chaturvedi, Pankaj, Krishna, C Murali

Institution

Hole, Arti. Chilakapati Laboratory, 29435Advanced Centre for Treatment Research and Education in Cancer, Tata Memorial Centre, Navi Mumbai, India. Tyagi, Gunjan. Chilakapati Laboratory, 29435Advanced Centre for Treatment Research and Education in Cancer, Tata Memorial Centre, Navi Mumbai, India.

Deshmukh, Atul. Centre for Interdisciplinary Research, D.Y. Patil University, Navi Mumbai, India.

Deshpande, Raviraj. Clinical Pharmacology Laboratory, 29435Advanced Centre for Treatment Research and Education in Cancer, Tata Memorial Centre, Navi Mumbai, India.

Gota, Vikram. Clinical Pharmacology Laboratory, 29435Advanced Centre for Treatment Research and Education in Cancer, Tata Memorial Centre, Navi Mumbai, India.

Gota, Vikram. Homi Bhabha National Institute, Training School Complex, Nagar Mumbai, India.

Chaturvedi, Pankaj. Homi Bhabha National Institute, Training School Complex, Nagar Mumbai, India.

Chaturvedi, Pankaj. Department of Surgical Oncology, Tata Memorial Hospital, Mumbai, India.

Krishna, C Murali. Chilakapati Laboratory, 29435Advanced Centre for Treatment Research and Education in Cancer, Tata Memorial Centre, Navi Mumbai, India.

Krishna, C Murali. Homi Bhabha National Institute, Training School Complex, Nagar Mumbai, India.

MeSH Heading

Discriminant Analysis. Humans. Mouth Neoplasms/di [Diagnosis]. *Mouth Neoplasms. Reference Standards. Saliva. *Spectrum Analysis, Raman.

Keyword Heading

Raman spectroscopy Saliva

noninvasive diagnosis

oral cancer

sampling method.

Keyword Heading Owner

NOTNLM

Year of Publication

2021

Link to the Ovid Full Text or citation:

[Click here for full text options](https://ovidsp.ovid.com/ovidweb.cgi?T=JS&CSC=Y&NEWS=N&PAGE=fulltext&D=med19&AN=33107759)

Link to the External Link Resolver:

[SFX](https://sfx-86scu.hosted.exlibrisgroup.com.cn/86scu?sid=OVID:medline&id=pmid:33107759&id=doi:10.1177%2F0003702820973260&issn=00037028&isbn=&volume=75&issue=5&spage=581&pages=581-588&date=2021&title=Applied+Spectroscopy&atitle=Salivary+Raman+Spectroscopy%3A+Standardization+of+Sampling+Protocols+and+Stratification+of+Healthy+and+Oral+Cancer+Subjects.&aulast=Hole&pid=<author>Hole+A%3BTyagi+G%3BDeshmukh+A%3BDeshpande+R%3BGota+V%3BChaturvedi+P%3BKrishna+CM<%2Fauthor><AN>33107759<%2FAN><DT>Journal+Article<%2FDT>)

58.

Raman profile alterations of irradiated human nasopharyngeal cancer cells detected with laser tweezer Raman spectroscopy.

Qiu S, Weng Y, Li Y, Chen Y, Pan Y, Liu J, Lin W, Chen X, Li M, Lin T, Liu W, Zhang L, Lin D

RSC advances. 10(24):14368-14373, 2020 Apr 06.

[Journal Article]

UI: 35498464

Radiotherapy has been widely used for nasopharyngeal carcinoma (NPC) treatment, which causes DNA damage and alterations of macromolecules of cancer cells. However, the Raman profile alterations of irradiated NPC cells remain unclear. In the present study, we used laser tweezers Raman spectroscopy (LTRS) to monitor internal structural changes and chemical modifications in NPC cells after exposure at a clinical dose (2.3 Gy) to X-ray irradiation (IR) at a single-cell level. Two types of NPC cell lines, CNE2 (EBV-negative cell line) and C666-1 (EBV-positive cell line), were used. The Raman spectra of cells before and after radiation treatment were recorded by LTRS. The analysis of spectral differences indicated that the IR caused Raman profile alterations of intracellular proteins, DNA base and lipids. Moreover, by using the multivariate statistical analysis including principal component analysis (PCA) and linear discriminant analysis (LDA) algorithm, an accuracy of 90.0% for classification between CNE2 cells before and after IR could be achieved, which was 10% better than that of C666-1 cells. The results demonstrated that CNE2 cells were more sensitive to IR in comparison to C666-1 cells, providing useful information for creating a treatment strategy in clinical practice. This exploratory study suggested that LTRS combined with multivariate statistical analysis would be a novel and effective tool for evaluating the radiotherapeutic effect on tumor cells, and for detection of the corresponding alterations at the molecular level.

Copyright This journal is © The Royal Society of Chemistry.

Version ID

1

Record Owner

From MEDLINE, a database of the U.S. National Library of Medicine.

Status

PubMed-not-MEDLINE

Author NameID

Chen, Yang; ORCID: <https://orcid.org/0000-0002-9255-6502> Lin, Duo; ORCID: <https://orcid.org/0000-0001-6959-5995>

Authors Full Name

Qiu, Sufang, Weng, Youliang, Li, Ying, Chen, Yang, Pan, Yuhui, Liu, Jun, Lin, Wanzun, Chen, Xiaochuan, Li, Miaomiao, Lin, Ting, Liu, Wei, Zhang, Lurong, Lin, Duo

Institution

Qiu, Sufang. Fujian Medical University Cancer Hospital, Fujian Cancer Hospital Fuzhou 350014 China sfqiu@126.com. Qiu, Sufang. Fujian Provincial Key Laboratory of Translational Cancer Medicine Fuzhou 350014 China.

Weng, Youliang. Department of Radiation Oncology, Fujian Cancer Hospital, Fujian Medical University Cancer Hospital Fuzhou 350014 China.

Li, Ying. Fujian Medical University Cancer Hospital, Fujian Cancer Hospital Fuzhou 350014 China sfqiu@126.com.

Chen, Yang. Department of Laboratory Medicine, Fujian Medical University Fuzhou 350004 China.

Pan, Yuhui. Department of Radiation Oncology, Fujian Cancer Hospital, Fujian Medical University Cancer Hospital Fuzhou 350014 China.

Liu, Jun. Cancer Bio-immunotherapy Center, Fujian Medical University Cancer Hospital, Fujian Cancer Hospital Fuzhou 350014 China.

Liu, Jun. Department of Medical Oncology, Fujian Medical University Cancer Hospital, Fujian Cancer Hospital Fuzhou 350014 China.

Lin, Wanzun. Fujian Medical University Cancer Hospital, Fujian Cancer Hospital Fuzhou 350014 China sfqiu@126.com.

Chen, Xiaochuan. Fujian Medical University Cancer Hospital, Fujian Cancer Hospital Fuzhou 350014 China sfqiu@126.com.

Li, Miaomiao. Fujian Medical University Cancer Hospital, Fujian Cancer Hospital Fuzhou 350014 China sfqiu@126.com.

Lin, Ting. Fujian Medical University Cancer Hospital, Fujian Cancer Hospital Fuzhou 350014 China sfqiu@126.com.

Liu, Wei. Fujian Medical University Cancer Hospital, Fujian Cancer Hospital Fuzhou 350014 China sfqiu@126.com.

Zhang, Lurong. Laboratory of Radiation Oncology and Radiobiology, Fujian Cancer Hospital, Fujian Medical University Cancer Hospital Fuzhou 350014 China.

Lin, Duo. Key Laboratory of Optoelectronic Science and Technology for Medicine, Ministry of Education, Fujian Provincial Key Laboratory for Photonics Technology, Fujian Normal University Fuzhou 350007 China.

Year of Publication

2020

Link to the Ovid Full Text or citation:

[Click here for full text options](https://ovidsp.ovid.com/ovidweb.cgi?T=JS&CSC=Y&NEWS=N&PAGE=fulltext&D=pmnm5&AN=35498464)

Link to the External Link Resolver:

[SFX](https://sfx-86scu.hosted.exlibrisgroup.com.cn/86scu?sid=OVID:medline&id=pmid:35498464&id=doi:10.1039%2Fd0ra01173h&issn=20462069&isbn=&volume=10&issue=24&spage=14368&pages=14368-14373&date=2020&title=RSC+advances&atitle=Raman+profile+alterations+of+irradiated+human+nasopharyngeal+cancer+cells+detected+with+laser+tweezer+Raman+spectroscopy.&aulast=Qiu&pid=<author>Qiu+S%3BWeng+Y%3BLi+Y%3BChen+Y%3BPan+Y%3BLiu+J%3BLin+W%3BChen+X%3BLi+M%3BLin+T%3BLiu+W%3BZhang+L%3BLin+D<%2Fauthor><AN>35498464<%2FAN><DT>Journal+Article<%2FDT>)

59.

Raman Spectroscopy Discloses Altered Molecular Profile in Thyroid Adenomas.

Sodo A, Verri M, Palermo A, Naciu AM, Sponziello M, Durante C, Di Gioacchino M, Paolucci A, di Masi A, Longo F, Crucitti P, Taffon C, Ricci MA, Crescenzi A

Diagnostics. 11(1), 2020 Dec 29.

[Journal Article]

UI: 33383892

Follicular patterned nodules are sometimes complex to be classified due to ambiguous nuclear features and/or questionable capsular or vascular invasion. In this setting, there is a poor inter-observer concordance even among expert pathologists. Raman spectroscopy was recently used to separate benign and malignant thyroid nodules based on their molecular fingerprint; anyway, some histologically proved follicular adenomas were clustered as having a characteristic profile of malignant lesions. In this study, we analyzed five follicular thyroid adenomas with a malignant spectroscopic profile compared to five follicular adenomas with a benign Raman spectrum in order to assess possible molecular differences between the two groups. Morphological, immunohistochemical, and molecular analyses evidenced expression of malignancy-associated proteins in four out of five malignant clustered adenomas. The remaining malignant clustered adenoma showed a TSHR mutation previously associated with autonomously functioning follicular carcinomas. In conclusion, thyroid follicular adenomas are a group of morphologically benign neoplasms that may have altered the mutational or expression profile; cases of adenomas with altered immunophenotype are recognized as showing a profile associated with malignancy by Raman spectroscopy. This correlation warrants a more extensive evaluation and suggests a potential predictive value of spectroscopic assessment in recognizing characteristics associated with tumor progression in follicular thyroid neoplasms.

Version ID

1

Record Owner

From MEDLINE, a database of the U.S. National Library of Medicine.

Status

PubMed-not-MEDLINE

Author NameID

Palermo, Andrea; ORCID: <https://orcid.org/0000-0002-1143-4926> Sponziello, Marialuisa; ORCID: <https://orcid.org/0000-0003-0736-1047>

Durante, Cosimo; ORCID: <https://orcid.org/0000-0002-1791-5915>

Di Gioacchino, Michael; ORCID: <https://orcid.org/0000-0001-7465-2456>

di Masi, Alessandra; ORCID: <https://orcid.org/0000-0002-1122-8663>

Ricci, Maria Antonietta; ORCID: <https://orcid.org/0000-0002-6904-6686>

Authors Full Name

Sodo, Armida, Verri, Martina, Palermo, Andrea, Naciu, Anda Mihaela, Sponziello, Marialuisa, Durante, Cosimo, Di Gioacchino, Michael, Paolucci, Alessio, di Masi, Alessandra, Longo, Filippo, Crucitti, Pierfilippo, Taffon, Chiara, Ricci, Maria Antonietta, Crescenzi, Anna

Institution

Sodo, Armida. Department of Sciences, University Roma Tre, 00146 Rome, Italy. Verri, Martina. Pathology Unit, Campus Bio-Medico University Hospital, 00128 Rome, Italy.

Palermo, Andrea. Unit of Endocrinology and Diabetes, Campus Bio-Medico University, 00128 Rome, Italy.

Naciu, Anda Mihaela. Unit of Endocrinology and Diabetes, Campus Bio-Medico University, 00128 Rome, Italy.

Sponziello, Marialuisa. Department of Translational and Precision Medicine, Sapienza University of Rome, 00185 Rome, Italy.

Durante, Cosimo. Department of Translational and Precision Medicine, Sapienza University of Rome, 00185 Rome, Italy.

Di Gioacchino, Michael. Department of Sciences, University Roma Tre, 00146 Rome, Italy.

Paolucci, Alessio. Department of Sciences, University Roma Tre, 00146 Rome, Italy.

di Masi, Alessandra. Department of Sciences, University Roma Tre, 00146 Rome, Italy.

Longo, Filippo. Unit of Thoracic Surgery, Campus Bio-Medico University Hospital, 00128 Rome, Italy.

Crucitti, Pierfilippo. Unit of Thoracic Surgery, Campus Bio-Medico University Hospital, 00128 Rome, Italy.

Taffon, Chiara. Pathology Unit, Campus Bio-Medico University Hospital, 00128 Rome, Italy.

Ricci, Maria Antonietta. Department of Sciences, University Roma Tre, 00146 Rome, Italy.

Crescenzi, Anna. Pathology Unit, Campus Bio-Medico University Hospital, 00128 Rome, Italy.

Keyword Heading

Raman spectroscopy follicular adenoma

follicular carcinoma

immunohistochemistry

molecular markers

mutational analysis

thyroid.

Keyword Heading Owner

NOTNLM

Year of Publication

2020

Link to the Ovid Full Text or citation:

[Click here for full text options](https://ovidsp.ovid.com/ovidweb.cgi?T=JS&CSC=Y&NEWS=N&PAGE=fulltext&D=pmnm5&AN=33383892)

Link to the External Link Resolver:

[SFX](https://sfx-86scu.hosted.exlibrisgroup.com.cn/86scu?sid=OVID:medline&id=pmid:33383892&id=doi:10.3390%2Fdiagnostics11010043&issn=20754418&isbn=&volume=11&issue=1&spage=&pages=&date=2020&title=Diagnostics&atitle=Raman+Spectroscopy+Discloses+Altered+Molecular+Profile+in+Thyroid+Adenomas.&aulast=Sodo&pid=<author>Sodo+A%3BVerri+M%3BPalermo+A%3BNaciu+AM%3BSponziello+M%3BDurante+C%3BDi+Gioacchino+M%3BPaolucci+A%3Bdi+Masi+A%3BLongo+F%3BCrucitti+P%3BTaffon+C%3BRicci+MA%3BCrescenzi+A<%2Fauthor><AN>33383892<%2FAN><DT>Journal+Article<%2FDT>)

60.

Novel Quantitative Analysis Using Optical Imaging (VELscope) and Spectroscopy (Raman) Techniques for Oral Cancer Detection.

Jeng MJ, Sharma M, Sharma L, Huang SF, Chang LB, Wu SL, Chow L

Cancers. 12(11), 2020 Nov 13.

[Journal Article]

UI: 33202869

In this study, we developed a novel quantitative analysis method to enhance the detection capability for oral cancer screening. We combined two different optical techniques, a light-based detection technique (visually enhanced lesion scope) and a vibrational spectroscopic technique (Raman spectroscopy). Materials and methods: Thirty-five oral cancer patients who went through surgery were enrolled. Thirty-five cancer lesions and thirty-five control samples with normal oral mucosa (adjacent to the cancer lesion) were analyzed. Thirty-five autofluorescence images and 70 Raman spectra were taken from 35 cancer and 35 control group cryopreserved samples. The normalized intensity and heterogeneity of the 70 regions of interest (ROIs) were calculated along with 70 averaged Raman spectra. Linear discriminant analysis (LDA) and quadratic discriminant analysis (QDA) were used with principal component analysis (PCA) to differentiate the cancer and control groups (normal). The classifications rates were validated using two different validation methods, leave-one-out cross-validation (LOOCV) and k-fold cross-validation.

Results: The cryopreserved normal and tumor tissues were differentiated using the PCA-LDA and PCA-QDA models. The PCA-LDA of Raman spectroscopy (RS) had 82.9% accuracy, 80% sensitivity, and 85.7% specificity, while ROIs on the autofluorescence images were differentiated with 90% accuracy, 100% sensitivity, and 80% specificity. The combination of two optical techniques differentiated cancer and normal group with 97.14% accuracy, 100% sensitivity, and 94.3% specificity. Conclusion: In this study, we combined the data of two different optical techniques. Furthermore, PCA-LDA and PCA-QDA quantitative analysis models were used to differentiate tumor and normal groups, creating a complementary pathway for efficient tumor diagnosis. The error rates of RS and VELcope analysis were 17.10% and 10%, respectively, which was reduced to 3% when the two optical techniques were combined.

Version ID

1

Record Owner

From MEDLINE, a database of the U.S. National Library of Medicine.

Status

PubMed-not-MEDLINE

Author NameID

Sharma, Lokesh; ORCID: <https://orcid.org/0000-0001-8362-510X> Huang, Shiang-Fu; ORCID: <https://orcid.org/0000-0003-3582-9938>

Chang, Liann-Be; ORCID: <https://orcid.org/0000-0003-2174-2022>

Chow, Lee; ORCID: <https://orcid.org/0000-0001-7729-6848>

Authors Full Name

Jeng, Ming-Jer, Sharma, Mukta, Sharma, Lokesh, Huang, Shiang-Fu, Chang, Liann-Be, Wu, Shih-Lin, Chow, Lee

Institution

Jeng, Ming-Jer. Department of Electronic Engineering, Chang Gung University, Taoyuan 333, Taiwan. Jeng, Ming-Jer. Department of Otolaryngology-Head and Neck Surgery, Chang Gung Memorial Hospital, Linkou 244, Taiwan.

Sharma, Mukta. Department of Electronic Engineering, Chang Gung University, Taoyuan 333, Taiwan.

Sharma, Lokesh. Department of Computer Science and Information Engineering, Chang Gung University, Taoyuan 333, Taiwan.

Huang, Shiang-Fu. Department of Otolaryngology-Head and Neck Surgery, Chang Gung Memorial Hospital, Linkou 244, Taiwan.

Huang, Shiang-Fu. Department of Public Health, Chang Gung University, Taoyuan 333, Taiwan.

Huang, Shiang-Fu. Graduate Institute of Clinical Medical Sciences, Chang Gung University, Taoyuan 333, Taiwan.

Chang, Liann-Be. Department of Otolaryngology-Head and Neck Surgery, Chang Gung Memorial Hospital, Linkou 244, Taiwan.

Chang, Liann-Be. Green Technology Research Center, Chang Gung University, Guishan, Taoyuan 333, Taiwan.

Wu, Shih-Lin. Department of Computer Science and Information Engineering, Chang Gung University, Taoyuan 333, Taiwan.

Wu, Shih-Lin. Department of Cardiology, Chang Gung Memorial Hospital, Taoyuan 333, Taiwan.

Chow, Lee. Department of Physics, University of Central Florida, Orlando, FL 32816, USA.

Keyword Heading

PCA-LDA and PCA-QDA Raman spectroscopy

autofluorescence

cryopreserved tissue

oral cancer.

Keyword Heading Owner

NOTNLM

Year of Publication

2020

Link to the Ovid Full Text or citation:

[Click here for full text options](https://ovidsp.ovid.com/ovidweb.cgi?T=JS&CSC=Y&NEWS=N&PAGE=fulltext&D=pmnm5&AN=33202869)

Link to the External Link Resolver:

[SFX](https://sfx-86scu.hosted.exlibrisgroup.com.cn/86scu?sid=OVID:medline&id=pmid:33202869&id=doi:10.3390%2Fcancers12113364&issn=20726694&isbn=&volume=12&issue=11&spage=&pages=&date=2020&title=Cancers&atitle=Novel+Quantitative+Analysis+Using+Optical+Imaging+(VELscope)+and+Spectroscopy+(Raman)+Techniques+for+Oral+Cancer+Detection.&aulast=Jeng&pid=<author>Jeng+MJ%3BSharma+M%3BSharma+L%3BHuang+SF%3BChang+LB%3BWu+SL%3BChow+L<%2Fauthor><AN>33202869<%2FAN><DT>Journal+Article<%2FDT>)

61.

Study on the chemodrug-induced effect in nasopharyngeal carcinoma cells using laser tweezer Raman spectroscopy.

Qiu S, Li M, Liu J, Chen X, Lin T, Xu Y, Chen Y, Weng Y, Pan Y, Feng S, Lin X, Zhang L, Lin D

Biomedical Optics Express. 11(4):1819-1833, 2020 Apr 01.

[Journal Article]

UI: 32341850

To explore the effect in nasopharyngeal carcinoma (NPC) cells after treatment with chemodrugs, Raman profiles were characterized by laser tweezer Raman spectroscopy. Two NPC cell lines (CNE2 and C666-1) were treated with gemcitabine, cisplatin, and paclitaxel, respectively. The high-quality Raman spectra of cells without or with treatments were recorded at the single-cell level with label-free laser tweezers Raman spectroscopy (LTRS) and analyzed for the differences of alterations of Raman profiles. Tentative assignments of Raman peaks indicated that the cellular specific biomolecular changes associated with drug treatment include changes in protein structure (e.g. 1655 cm-1), changes in DNA/RNA content and structure (e.g. 830 cm-1), destruction of DNA/RNA base pairs (e.g. 785 cm-1), and reduction in lipids (e.g. 970 cm-1). Besides, both principal components analysis (PCA) combined with linear discriminant analysis (LDA) and the classification and regression trees (CRT) algorithms were employed to further analyze and classify the spectral data between control group and treated group, with the best discriminant accuracy of 96.7% and 90.0% for CNE2 and C666-1 group treated with paclitaxel, respectively. This exploratory work demonstrated that LTRS technology combined with multivariate statistical analysis has promising potential to be a novel analytical strategy at the single-cell level for the evaluation of NPC-related chemotherapeutic drugs.

Copyright © 2020 Optical Society of America under the terms of the OSA Open Access Publishing Agreement.

Version ID

1

Record Owner

From MEDLINE, a database of the U.S. National Library of Medicine.

Status

PubMed-not-MEDLINE

Author NameID

Chen, Yang; ORCID: <https://orcid.org/0000-0002-9255-6502> Lin, Duo; ORCID: <https://orcid.org/0000-0001-6959-5995>

Authors Full Name

Qiu, Sufang, Li, Miaomiao, Liu, Jun, Chen, Xiaochuan, Lin, Ting, Xu, Yunchao, Chen, Yang, Weng, Youliang, Pan, Yuhui, Feng, Shangyuan, Lin, Xiandong, Zhang, Lurong, Lin, Duo

Institution

Qiu, Sufang. Department of Radiation Oncology, Fujian Medical University Cancer Hospital and Fujian Cancer Hospital, Fuzhou 350014, China. Qiu, Sufang. Fujian Provincial Key Laboratory of Translational Cancer Medicine, Fuzhou 350014, China.

Qiu, Sufang. These authors contributed equally to this work.

Qiu, Sufang. sfqiu@126.com.

Li, Miaomiao. Fujian Medical University Cancer Hospital and Fujian Cancer Hospital, Fuzhou 350014, China.

Li, Miaomiao. These authors contributed equally to this work.

Liu, Jun. Cancer Bio-immunotherapy Center, Fujian Medical University Cancer Hospital and Fujian Cancer Hospital, Fuzhou 350014, China.

Liu, Jun. Department of Medical Oncology, Fujian Medical University Cancer Hospital and Fujian Cancer Hospital, Fuzhou 350014, China.

Chen, Xiaochuan. Fujian Medical University Cancer Hospital and Fujian Cancer Hospital, Fuzhou 350014, China.

Lin, Ting. Fujian Medical University Cancer Hospital and Fujian Cancer Hospital, Fuzhou 350014, China.

Xu, Yunchao. Key Laboratory of Optoelectronic Science and Technology for Medicine, Ministry of Education and Fujian Provincial Key Laboratory for Photonics Technology, Fujian Normal University, Fuzhou 350007, China.

Chen, Yang. Department of Laboratory Medicine, Fujian Medical University, Fuzhou 350004, China.

Weng, Youliang. Department of Radiation Oncology, Fujian Medical University Cancer Hospital and Fujian Cancer Hospital, Fuzhou 350014, China.

Pan, Yuhui. Department of Radiation Oncology, Fujian Medical University Cancer Hospital and Fujian Cancer Hospital, Fuzhou 350014, China.

Feng, Shangyuan. Key Laboratory of Optoelectronic Science and Technology for Medicine, Ministry of Education and Fujian Provincial Key Laboratory for Photonics Technology, Fujian Normal University, Fuzhou 350007, China.

Lin, Xiandong. Laboratory of Radiation Oncology and Radiobiology, Fujian Cancer Hospital and Fujian Medical University Cancer Hospital, Fuzhou 350014, China.

Zhang, Lurong. Laboratory of Radiation Oncology and Radiobiology, Fujian Cancer Hospital and Fujian Medical University Cancer Hospital, Fuzhou 350014, China.

Zhang, Lurong. lz8506@163.com.

Lin, Duo. Key Laboratory of Optoelectronic Science and Technology for Medicine, Ministry of Education and Fujian Provincial Key Laboratory for Photonics Technology, Fujian Normal University, Fuzhou 350007, China.

Lin, Duo. linduo1986@163.com.

Year of Publication

2020

Link to the Ovid Full Text or citation:

[Click here for full text options](https://ovidsp.ovid.com/ovidweb.cgi?T=JS&CSC=Y&NEWS=N&PAGE=fulltext&D=pmnm5&AN=32341850)

Link to the External Link Resolver:

[SFX](https://sfx-86scu.hosted.exlibrisgroup.com.cn/86scu?sid=OVID:medline&id=pmid:32341850&id=doi:10.1364%2FBOE.388785&issn=21567085&isbn=&volume=11&issue=4&spage=1819&pages=1819-1833&date=2020&title=Biomedical+Optics+Express&atitle=Study+on+the+chemodrug-induced+effect+in+nasopharyngeal+carcinoma+cells+using+laser+tweezer+Raman+spectroscopy.&aulast=Qiu&pid=<author>Qiu+S%3BLi+M%3BLiu+J%3BChen+X%3BLin+T%3BXu+Y%3BChen+Y%3BWeng+Y%3BPan+Y%3BFeng+S%3BLin+X%3BZhang+L%3BLin+D<%2Fauthor><AN>32341850<%2FAN><DT>Journal+Article<%2FDT>)

62.

Raman spectroscopy combined with multivariate analysis to study the biochemical mechanism of lung cancer microwave ablation.

Song D, Yu F, Chen S, Chen Y, He Q, Zhang Z, Zhang J, Wang S

Biomedical Optics Express. 11(2):1061-1072, 2020 Feb 01.

[Journal Article]

UI: 32133237

Lung cancer is the leading cause of death in cancer patients, and microwave ablation (MWA) has been extensively used in clinical treatment. In this study, we characterized the spectra of MWA-treated and untreated lung squamous cell carcinoma (LSCC) tissues, as well as healthy lung tissue, and conducted a preliminary analysis of spectral variations associated with MWA treatment. The results of characteristic spectral analysis of different types of tissues indicated that MWA treatment induces an increase in the content of nucleic acids, proteins, and lipid components in lung cancer tissues. The discriminant model based on the principal component analysis - linear discriminant analysis (PCA-LDA) algorithm together with leave-one-out cross validation (LOOCV) method yield the sensitivities of 90%, 80%, and 96%, and specificities of 86.2%, 93.8%, and 100% among untreated and MWA-treated cancerous tissue, and healthy lung tissue, respectively. These results indicate that Raman spectroscopy combined with multivariate analysis techniques can be used to explore the biochemical response mechanism of cancerous tissue to MWA therapy.

Copyright © 2020 Optical Society of America under the terms of the OSA Open Access Publishing Agreement.

Version ID

1

Record Owner

From MEDLINE, a database of the U.S. National Library of Medicine.

Status

PubMed-not-MEDLINE

Author NameID

He, Qingli; ORCID: <https://orcid.org/0000-0002-7413-8411> Wang, Shuang; ORCID: <https://orcid.org/0000-0002-5558-4029>

Authors Full Name

Song, Dongliang, Yu, Fan, Chen, Shilin, Chen, Yishen, He, Qingli, Zhang, Zhe, Zhang, Jingyuan, Wang, Shuang

Institution

Song, Dongliang. Institute of Photonics and Photon-Technology, Northwest University, Xi'an, Shaanxi, 710069, China. Song, Dongliang. Department of physics, Northwest University, Xi'an, Shaanxi, 710069, China.

Yu, Fan. Institute of Photonics and Photon-Technology, Northwest University, Xi'an, Shaanxi, 710069, China.

Chen, Shilin. Thoracic Surgery, Nanjing Medical University Affiliated Cancer Hospital, Jiangsu Cancer Hospital, Nanjing, Jiangsu, 210009, China.

Chen, Shilin. chenshilin@163.com.

Chen, Yishen. Institute of Photonics and Photon-Technology, Northwest University, Xi'an, Shaanxi, 710069, China.

He, Qingli. Department of physics, Northwest University, Xi'an, Shaanxi, 710069, China.

Zhang, Zhe. Department of Pathology, Nanjing Medical University Affiliated Cancer Hospital, Jiangsu Cancer Hospital, Nanjing, Jiangsu, 210009, China.

Zhang, Jingyuan. Department of Pathology, Nanjing Medical University Affiliated Cancer Hospital, Jiangsu Cancer Hospital, Nanjing, Jiangsu, 210009, China.

Wang, Shuang. Institute of Photonics and Photon-Technology, Northwest University, Xi'an, Shaanxi, 710069, China.

Wang, Shuang. swang@nwu.edu.cn.

Year of Publication

2020

Link to the Ovid Full Text or citation:

[Click here for full text options](https://ovidsp.ovid.com/ovidweb.cgi?T=JS&CSC=Y&NEWS=N&PAGE=fulltext&D=pmnm5&AN=32133237)

Link to the External Link Resolver:

[SFX](https://sfx-86scu.hosted.exlibrisgroup.com.cn/86scu?sid=OVID:medline&id=pmid:32133237&id=doi:10.1364%2FBOE.383869&issn=21567085&isbn=&volume=11&issue=2&spage=1061&pages=1061-1072&date=2020&title=Biomedical+Optics+Express&atitle=Raman+spectroscopy+combined+with+multivariate+analysis+to+study+the+biochemical+mechanism+of+lung+cancer+microwave+ablation.&aulast=Song&pid=<author>Song+D%3BYu+F%3BChen+S%3BChen+Y%3BHe+Q%3BZhang+Z%3BZhang+J%3BWang+S<%2Fauthor><AN>32133237<%2FAN><DT>Journal+Article<%2FDT>)

63.

The Expressions of Keratins and P63 in Primary Squamous Cell Carcinoma of the Thyroid Gland: An Application of Raman Spectroscopy.

Wang SS, Ye DX, Wang B, Xie C

OncoTargets and therapy. 13:585-591, 2020.

[Case Reports]

UI: 32021300

PURPOSE: Primary squamous cell carcinoma is a rare malignancy in the thyroid gland (SCCTh). The overall prognosis of this carcinoma is poor. This study aimed to explore the application of Raman spectroscopy in investigating the expression of CK5/6 and P63 in SCCTh.

PATIENTS AND METHODS: Tissues of the SCCTh and adjacent normal thyroid, as well as blood serum, were collected from a patient with pathology-confirmed SCCTh. Whole genome sequencing analysis was performed with the tissue of the SCCTh. The expressions of keratins and TP53 family gene were investigated by the Raman spectroscopy in tissues of the SCCTh and adjacent normal thyroid. The serum was also investigated by the Raman spectroscopy for the expression of keratins and TP53 family gene.

RESULTS: The whole genome sequencing analysis identified the mutation of the TP53 gene (42%) in the tissues of SCCTh. Accordingly, the Raman spectra analyses showed higher expression of keratins and TP53 family gene in the tissues of SCCTh compared with that in the adjacent normal thyroid. Raman spectra analyses of the serum of the patient also showed the expressions of the keratins and TP53 family gene.

CONCLUSION: The expressions of the keratins and TP53 are different in the tissues of SCCTh and adjacent normal thyroid, and the difference could be identified with high sensitivity by the Raman spectra analyses.

Copyright © 2020 Wang et al.

Version ID

1

Record Owner

From MEDLINE, a database of the U.S. National Library of Medicine.

Status

PubMed-not-MEDLINE

Authors Full Name

Wang, Si-Si, Ye, Dao-Xiong, Wang, Bo, Xie, Chao

Institution

Wang, Si-Si. Department of General Surgery, Fujian Medical University Union Hospital, Fuzhou, Fujian Province, The People's Republic of China. Ye, Dao-Xiong. Department of General Surgery, Fujian Medical University Union Hospital, Fuzhou, Fujian Province, The People's Republic of China.

Wang, Bo. Department of General Surgery, Fujian Medical University Union Hospital, Fuzhou, Fujian Province, The People's Republic of China.

Xie, Chao. Department of General Surgery, Fujian Medical University Union Hospital, Fuzhou, Fujian Province, The People's Republic of China.

Keyword Heading

CK5/6 P53

Raman spectrum analysis

SCCTh

thyroid neoplasms.

Keyword Heading Owner

NOTNLM

Year of Publication

2020

Link to the Ovid Full Text or citation:

[Click here for full text options](https://ovidsp.ovid.com/ovidweb.cgi?T=JS&CSC=Y&NEWS=N&PAGE=fulltext&D=pmnm5&AN=32021300)

Link to the External Link Resolver:

[SFX](https://sfx-86scu.hosted.exlibrisgroup.com.cn/86scu?sid=OVID:medline&id=pmid:32021300&id=doi:10.2147%2FOTT.S229436&issn=11786930&isbn=&volume=13&issue=&spage=585&pages=585-591&date=2020&title=OncoTargets+and+therapy&atitle=The+Expressions+of+Keratins+and+P63+in+Primary+Squamous+Cell+Carcinoma+of+the+Thyroid+Gland%3A+An+Application+of+Raman+Spectroscopy.&aulast=Wang&pid=<author>Wang+SS%3BYe+DX%3BWang+B%3BXie+C<%2Fauthor><AN>32021300<%2FAN><DT>Case+Reports<%2FDT>)

64.

Gold Nanoparticles Enhance EGFR Inhibition and Irradiation Effects in Head and Neck Squamous Carcinoma Cells.

Kashin M, Kakei Y, Teraoka S, Hasegawa T, Yamaguchi A, Fukuoka T, Sasaki R, Akashi M

BioMed Research International. 2020:1281645, 2020.

[Journal Article]

UI: 33204681

Cetuximab, an epidermal growth factor receptor inhibitor (EI), is currently the only targeted molecular therapy used in combination with radiotherapy for head and neck squamous cell carcinoma (HNSCC). Gold nanoparticles (AuNPs) are expected to enhance radiotherapy effects in cancers. To investigate whether AuNPs combined with AG1478, an EI, enhanced irradiation effects on HNSCC cells, we first examined AG1478 adsorption on AuNP surfaces, using surface-enhanced Raman scattering, which indicated an adsorption equilibrium of AG1478 to AuNPs. We then used transmission electron microscopy to find internalization rates of AuNP alone and AuNP+AG1478; we found that intracellular uptake of AuNP alone and AuNP+AG1478 did not significantly differ. We compared cell numbers, proliferation, apoptosis, and migration between control cells and those treated with or without 60 nm AuNP (1.0 nM), AG1478 (0.5 muM), and irradiation (4 Gy). We found that AuNP+AG1478 inhibited proliferation more than AG1478 alone; the combination of irradiation+AuNP+AG1478 significantly reduced total cell numbers compared with the combination of irradiation+AuNP; AuNP+AG1478 increased apoptotic reaction to irradiation; the combinations of AuNP+AG1478 and irradiation+AuNP induced more apoptosis than AG1478+irradiation. Whereas AuNP+AG1478 enhanced cytotoxicity in human HNSCC cells by inhibiting proliferation, irradiation+AuNP enhanced cytotoxicity by inducing apoptosis.

Copyright © 2020 Masahiko Kashin et al.

Version ID

1

Record Owner

From MEDLINE, a database of the U.S. National Library of Medicine.

Status

MEDLINE

Author NameID

Kakei, Yasumasa; ORCID: <https://orcid.org/0000-0002-5997-514X>

Authors Full Name

Kashin, Masahiko, Kakei, Yasumasa, Teraoka, Shun, Hasegawa, Takumi, Yamaguchi, Akinobu, Fukuoka, Takao, Sasaki, Ryohei, Akashi, Masaya

Institution

Kashin, Masahiko. Department of Oral and Maxillofacial Surgery, Kobe University Graduate School of Medicine, Kobe 650-0017, Japan. Kakei, Yasumasa. Department of Oral and Maxillofacial Surgery, Kobe University Graduate School of Medicine, Kobe 650-0017, Japan.

Teraoka, Shun. Department of Oral and Maxillofacial Surgery, Kobe University Graduate School of Medicine, Kobe 650-0017, Japan.

Hasegawa, Takumi. Department of Oral and Maxillofacial Surgery, Kobe University Graduate School of Medicine, Kobe 650-0017, Japan.

Yamaguchi, Akinobu. Laboratiory of Advanced Science and Technology for Industry, University of Hyogo, Kamigori 678-1205, Japan.

Fukuoka, Takao. Graduate School of Engineering, Kyoto University, Kyoto 615-8540, Japan.

Sasaki, Ryohei. Division of Radiation Oncology, Kobe University Graduate School of Medicine, Kobe 650-0017, Japan.

Akashi, Masaya. Department of Oral and Maxillofacial Surgery, Kobe University Graduate School of Medicine, Kobe 650-0017, Japan.

MeSH Heading

Antineoplastic Agents, Immunological/ad [Administration & Dosage]. *Antineoplastic Combined Chemotherapy Protocols/pd [Pharmacology]. Apoptosis/de [Drug Effects]. Cell Line, Tumor. Cell Proliferation/de [Drug Effects]. Cell Proliferation/re [Radiation Effects]. ErbB Receptors/ai [Antagonists & Inhibitors]. ErbB Receptors/im [Immunology]. Gold/ch [Chemistry]. *Head and Neck Neoplasms/dt [Drug Therapy]. Head and Neck Neoplasms/pa [Pathology]. *Head and Neck Neoplasms/rt [Radiotherapy]. Humans. Metal Nanoparticles/ad [Administration & Dosage]. Metal Nanoparticles/ch [Chemistry]. Metal Nanoparticles/tu [Therapeutic Use]. Microscopy, Electron, Transmission. Quinazolines/pd [Pharmacology]. Spectrum Analysis, Raman. *Squamous Cell Carcinoma of Head and Neck/dt [Drug Therapy]. Squamous Cell Carcinoma of Head and Neck/pa [Pathology]. *Squamous Cell Carcinoma of Head and Neck/rt [Radiotherapy]. Tyrphostins/pd [Pharmacology].

Registry Number/Name of Substance

0 (Antineoplastic Agents, Immunological). 0 (Quinazolines). 0 (Tyrphostins). 170449-18-0 (RTKI cpd). 7440-57-5 (Gold). EC 2-7-10-1 (EGFR protein, human). EC 2-7-10-1 (ErbB Receptors).

Year of Publication

2020

Link to the Ovid Full Text or citation:

[Click here for full text options](https://ovidsp.ovid.com/ovidweb.cgi?T=JS&CSC=Y&NEWS=N&PAGE=fulltext&D=med18&AN=33204681)

Link to the External Link Resolver:

[SFX](https://sfx-86scu.hosted.exlibrisgroup.com.cn/86scu?sid=OVID:medline&id=pmid:33204681&id=doi:10.1155%2F2020%2F1281645&issn=23146141&isbn=&volume=2020&issue=&spage=1281645&pages=1281645&date=2020&title=BioMed+Research+International&atitle=Gold+Nanoparticles+Enhance+EGFR+Inhibition+and+Irradiation+Effects+in+Head+and+Neck+Squamous+Carcinoma+Cells.&aulast=Kashin&pid=<author>Kashin+M%3BKakei+Y%3BTeraoka+S%3BHasegawa+T%3BYamaguchi+A%3BFukuoka+T%3BSasaki+R%3BAkashi+M<%2Fauthor><AN>33204681<%2FAN><DT>Journal+Article<%2FDT>)

65.

Plasmonic assay for amplification-free cancer biomarkers detection in clinical tissue samples.

Dukes PV, Strobbia P, Ngo HT, Odion RA, Rocke D, Lee WT, Vo-Dinh T

Analytica Chimica Acta. 1139:111-118, 2020 Dec 01.

[Journal Article]

UI: 33190693

Developing countries have seen a rise in cancer incidence and are projected to harbor three-quarters of all cancer-related mortality by 2030. While disproportionally affected by the burden of cancer, these regions are ill-equipped to handle the diagnostic caseload. The low number of trained pathologists per capita results in delayed diagnosis and treatment, ultimately contributing to increased mortality rates. To address this issue, we developed a point-of-care (POC) plasmonic assay for direct detection of cancer as an alternative to pathological review. Whereas our assay has general applicability in many cancer diagnoses that involve tissue biopsies, we use head and neck cancer (HNC) as a model system because these tumors are increasingly prevalent in lower-income and underserved regions, due to risk factors such as smoking, drinking, and viral infection. Our method uses surface-enhanced Raman scattering (SERS) to detect unique RNA biomarkers from human biopsy samples without the need for complex target amplification machinery (e.g., PCR), making it time and resource-efficient. Unlike previous studies that required target amplification, this work represents a significant advance for HNC diagnosis directly in clinical samples, using only our SERS-based assay for RNA biomarkers. In this study, we tested our assay on 20 clinical samples, demonstrating the accuracy of the method in the diagnosis of head and neck squamous cell carcinoma. We reported sensitivity of 100% and specificity of 97%. Furthermore, we used a handheld Raman device to read the results in order to illustrate the applicability of our method for POC diagnosis of cancer in low-resource settings.

Copyright © 2020. Published by Elsevier B.V.

Version ID

1

Record Owner

From MEDLINE, a database of the U.S. National Library of Medicine.

Status

MEDLINE

Authors Full Name

Dukes, Priya V, Strobbia, Pietro, Ngo, Hoan T, Odion, Ren A, Rocke, Daniel, Lee, Walter T, Vo-Dinh, Tuan

Institution

Dukes, Priya V. Fitzpatrick Institute for Photonics, Duke University, Durham, NC, USA; Department of Head and Neck Surgery and Communication Sciences, Duke School of Medicine, Durham, NC, USA. Strobbia, Pietro. Fitzpatrick Institute for Photonics, Duke University, Durham, NC, USA; Biomedical Engineering Department, Duke University, Durham, NC, USA.

Ngo, Hoan T. Fitzpatrick Institute for Photonics, Duke University, Durham, NC, USA; Biomedical Engineering Department, Duke University, Durham, NC, USA; Biomedical Engineering Department, International University, Vietnam National University - Ho Chi Minh City, Ho Chi Minh City, Viet Nam.

Odion, Ren A. Fitzpatrick Institute for Photonics, Duke University, Durham, NC, USA; Biomedical Engineering Department, Duke University, Durham, NC, USA.

Rocke, Daniel. Department of Head and Neck Surgery and Communication Sciences, Duke School of Medicine, Durham, NC, USA.

Lee, Walter T. Fitzpatrick Institute for Photonics, Duke University, Durham, NC, USA; Department of Head and Neck Surgery and Communication Sciences, Duke School of Medicine, Durham, NC, USA; Global Health Institute, Duke University, Durham, NC, USA.

Vo-Dinh, Tuan. Fitzpatrick Institute for Photonics, Duke University, Durham, NC, USA; Biomedical Engineering Department, Duke University, Durham, NC, USA; Chemistry Department, Duke University, Durham, NC, USA. Electronic address: tuan.vodinh@duke.edu.

MeSH Heading

Biological Assay. *Biomarkers, Tumor. Humans. Neoplasms/di [Diagnosis]. *Neoplasms. Point-of-Care Systems. Spectrum Analysis, Raman.

Keyword Heading

Cancer Plasmonics

RNA

Surface-enhanced Raman scattering.

Keyword Heading Owner

NOTNLM

Registry Number/Name of Substance

0 (Biomarkers, Tumor).

Year of Publication

2020

Link to the Ovid Full Text or citation:

[Click here for full text options](https://ovidsp.ovid.com/ovidweb.cgi?T=JS&CSC=Y&NEWS=N&PAGE=fulltext&D=med18&AN=33190693)

Link to the External Link Resolver:

[SFX](https://sfx-86scu.hosted.exlibrisgroup.com.cn/86scu?sid=OVID:medline&id=pmid:33190693&id=doi:10.1016%2Fj.aca.2020.09.003&issn=00032670&isbn=&volume=1139&issue=&spage=111&pages=111-118&date=2020&title=Analytica+Chimica+Acta&atitle=Plasmonic+assay+for+amplification-free+cancer+biomarkers+detection+in+clinical+tissue+samples.&aulast=Dukes&pid=<author>Dukes+PV%3BStrobbia+P%3BNgo+HT%3BOdion+RA%3BRocke+D%3BLee+WT%3BVo-Dinh+T<%2Fauthor><AN>33190693<%2FAN><DT>Journal+Article<%2FDT>)

66.

Diverse spectral band-based deep residual network for tongue squamous cell carcinoma classification using fiber optic Raman spectroscopy.

Ding J, Yu M, Zhu L, Zhang T, Xia J, Sun G

Photodiagnosis & Photodynamic Therapy. 32:102048, 2020 Dec.

[Journal Article]

UI: 33017657

The research is to propose a new classification framework, called diverse spectral band-based deep residual network (DSB-ResNet), which can distinguish tongue squamous cell carcinoma (TSCC) from non-cancerous tissue. A fiber optic Raman spectroscopy system is used to collect Raman spectral data of TSCC and normal tissues. DSB-ResNet takes advantage of diverse spectral band-based spectra without processing to derive spectral representations from different spectral bands of Raman spectra, which improves the ability to identify TSCC. To show the superiority of the proposed method, the existing methods are used as the competitive methods to compare with the DSB-RestNet, the results demonstrate our method has the highest performance with 97.38 %, 98.75 %, and 98.25 % for sensitivity, specificity, and accuracy, respectively. The experimental results show that the DSB-ResNet is able to distinguish TSCC from non-cancerous tissue successfully. The proposed method is expected to provide a theoretical and methodological base for accurate detection of TSCC.

Copyright © 2020. Published by Elsevier B.V.

Version ID

1

Record Owner

From MEDLINE, a database of the U.S. National Library of Medicine.

Status

MEDLINE

Authors Full Name

Ding, Jingya, Yu, Mingxin, Zhu, Lianqing, Zhang, Tao, Xia, Jiabin, Sun, Guangkai

Institution

Ding, Jingya. Key Laboratory of the Ministry of Education for Optoelectronic Measurement Technology and Instrument, Beijing Information Science and Technology University, Beijing, 100192, China. Electronic address: papersDing@163.com. Yu, Mingxin. Key Laboratory of the Ministry of Education for Optoelectronic Measurement Technology and Instrument, Beijing Information Science and Technology University, Beijing, 100192, China. Electronic address: yumingxin@bistu.edu.cn.

Zhu, Lianqing. Key Laboratory of the Ministry of Education for Optoelectronic Measurement Technology and Instrument, Beijing Information Science and Technology University, Beijing, 100192, China; School of Instrument Science and Opto-electronics Engineering, Hefei University of Technology, Hefei, 230009, China. Electronic address: lianqingbistu@sina.com.

Zhang, Tao. Department of stomatology, Peking Union Medical College Hospital, No. 1 Shuaifuyuan Wangfujing, Dongcheng District, Beijing 100730, China. Electronic address: drtzhang@126.com.

Xia, Jiabin. Key Laboratory of the Ministry of Education for Optoelectronic Measurement Technology and Instrument, Beijing Information Science and Technology University, Beijing, 100192, China; School of Instrument Science and Opto-electronics Engineering, Hefei University of Technology, Hefei, 230009, China. Electronic address: xiajiabinxjb@126.com.

Sun, Guangkai. Key Laboratory of the Ministry of Education for Optoelectronic Measurement Technology and Instrument, Beijing Information Science and Technology University, Beijing, 100192, China. Electronic address: tangxiaoyingbitlife@gmail.com.

Comments

Erratum in (EIN)

MeSH Heading

Carcinoma, Squamous Cell/di [Diagnosis]. *Carcinoma, Squamous Cell. Humans. Photochemotherapy/mt [Methods]. *Photochemotherapy. Photosensitizing Agents. Spectrum Analysis, Raman. Tongue. Tongue Neoplasms/di [Diagnosis]. *Tongue Neoplasms.

Keyword Heading

Deep residual network Raman spectroscopy

Tongue squamous cell carcinoma.

Keyword Heading Owner

NOTNLM

Registry Number/Name of Substance

0 (Photosensitizing Agents).

Year of Publication

2020

Link to the Ovid Full Text or citation:

[Click here for full text options](https://ovidsp.ovid.com/ovidweb.cgi?T=JS&CSC=Y&NEWS=N&PAGE=fulltext&D=med18&AN=33017657)

Link to the External Link Resolver:

[SFX](https://sfx-86scu.hosted.exlibrisgroup.com.cn/86scu?sid=OVID:medline&id=pmid:33017657&id=doi:10.1016%2Fj.pdpdt.2020.102048&issn=15721000&isbn=&volume=32&issue=&spage=102048&pages=102048&date=2020&title=Photodiagnosis+%26+Photodynamic+Therapy&atitle=Diverse+spectral+band-based+deep+residual+network+for+tongue+squamous+cell+carcinoma+classification+using+fiber+optic+Raman+spectroscopy.&aulast=Ding&pid=<author>Ding+J%3BYu+M%3BZhu+L%3BZhang+T%3BXia+J%3BSun+G<%2Fauthor><AN>33017657<%2FAN><DT>Journal+Article<%2FDT>)

67.

Thyroid cancer diagnosis by Raman spectroscopy.

Sbroscia M, Di Gioacchino M, Ascenzi P, Crucitti P, di Masi A, Giovannoni I, Longo F, Mariotti D, Naciu AM, Palermo A, Taffon C, Verri M, Sodo A, Crescenzi A, Ricci MA

Scientific Reports. 10(1):13342, 2020 08 07.

[Journal Article. Research Support, Non-U.S. Gov't]

UI: 32770073

Over the last 50 years, the incidence of human thyroid cancer disease has seen a significative increment. This comes along with an even higher increment of surgery, since, according to the international guidelines, patients are sometimes addressed to surgery also when the fine needle aspiration gives undetermined cytological diagnosis. As a matter of fact, only 30% of the thyroid glands removed for diagnostic purpose have a post surgical histological report of malignancy: this implies that about 70% of the patients have suffered an unnecessary thyroid removal. Here we show that Raman spectroscopy investigation of thyroid tissues provides reliable cancer diagnosis. Healthy tissues are consistently distinguished from cancerous ones with an accuracy of [Formula: see text] 90%, and the three cancer typology with highest incidence are clearly identified. More importantly, Raman investigation has evidenced alterations suggesting an early stage of transition of adenoma tissues into cancerous ones. These results suggest that Raman spectroscopy may overcome the limits of current diagnostic tools.

Version ID

1

Record Owner

From MEDLINE, a database of the U.S. National Library of Medicine.

Status

MEDLINE

Authors Full Name

Sbroscia, Marco, Di Gioacchino, Michael, Ascenzi, Paolo, Crucitti, Pierfilippo, di Masi, Alessandra, Giovannoni, Isabella, Longo, Filippo, Mariotti, Davide, Naciu, Anda Mihaela, Palermo, Andrea, Taffon, Chiara, Verri, Martina, Sodo, Armida, Crescenzi, Anna, Ricci, Maria Antonietta

Institution

Sbroscia, Marco. Dipartimento di Scienze, Universita degli Studi Roma Tre, Rome, Italy. Sbroscia, Marco. Dipartimento di Fisica, Sapienza Universita di Roma, Rome, Italy.

Di Gioacchino, Michael. Dipartimento di Scienze, Universita degli Studi Roma Tre, Rome, Italy.

Ascenzi, Paolo. Dipartimento di Scienze, Universita degli Studi Roma Tre, Rome, Italy.

Crucitti, Pierfilippo. Unit of Thoracic Surgery, Campus Bio-Medico University, Rome, Italy.

di Masi, Alessandra. Dipartimento di Scienze, Universita degli Studi Roma Tre, Rome, Italy.

Giovannoni, Isabella. Pathology Unit, Campus Biomedico University Hospital, Rome, Italy.

Longo, Filippo. Unit of Thoracic Surgery, Campus Bio-Medico University, Rome, Italy.

Mariotti, Davide. Dipartimento di Scienze, Universita degli Studi Roma Tre, Rome, Italy.

Naciu, Anda Mihaela. Unit of Endocrinology and Diabetes, Campus Bio-Medico University, Rome, Italy.

Palermo, Andrea. Unit of Endocrinology and Diabetes, Campus Bio-Medico University, Rome, Italy.

Taffon, Chiara. Pathology Unit, Campus Biomedico University Hospital, Rome, Italy.

Verri, Martina. Pathology Unit, Campus Biomedico University Hospital, Rome, Italy.

Sodo, Armida. Dipartimento di Scienze, Universita degli Studi Roma Tre, Rome, Italy. armida.sodo@uniroma3.it.

Crescenzi, Anna. Pathology Unit, Campus Biomedico University Hospital, Rome, Italy.

Ricci, Maria Antonietta. Dipartimento di Scienze, Universita degli Studi Roma Tre, Rome, Italy.

MeSH Heading

Adenoma/di [Diagnosis]. Adenoma/pa [Pathology]. Biopsy, Fine-Needle/mt [Methods]. Diagnosis, Differential. Female. Humans. Male. Middle Aged. Spectrum Analysis, Raman/mt [Methods]. Thyroid Gland/pa [Pathology]. *Thyroid Neoplasms/di [Diagnosis]. Thyroid Neoplasms/pa [Pathology]. Thyroid Nodule/di [Diagnosis]. Thyroid Nodule/pa [Pathology].

Year of Publication

2020

Link to the Ovid Full Text or citation:

[Click here for full text options](https://ovidsp.ovid.com/ovidweb.cgi?T=JS&CSC=Y&NEWS=N&PAGE=fulltext&D=med18&AN=32770073)

Link to the External Link Resolver:

[SFX](https://sfx-86scu.hosted.exlibrisgroup.com.cn/86scu?sid=OVID:medline&id=pmid:32770073&id=doi:10.1038%2Fs41598-020-70165-0&issn=20452322&isbn=&volume=10&issue=1&spage=13342&pages=13342&date=2020&title=Scientific+Reports&atitle=Thyroid+cancer+diagnosis+by+Raman+spectroscopy.&aulast=Sbroscia&pid=<author>Sbroscia+M%3BDi+Gioacchino+M%3BAscenzi+P%3BCrucitti+P%3Bdi+Masi+A%3BGiovannoni+I%3BLongo+F%3BMariotti+D%3BNaciu+AM%3BPalermo+A%3BTaffon+C%3BVerri+M%3BSodo+A%3BCrescenzi+A%3BRicci+MA<%2Fauthor><AN>32770073<%2FAN><DT>Journal+Article<%2FDT>)

68.

A pilot study for early detection of oral premalignant diseases using oral cytology and Raman micro-spectroscopy: Assessment of confounding factors.

Behl I, Calado G, Malkin A, Flint S, Galvin S, Healy CM, Pimentel ML, Byrne HJ, Lyng FM

Journal of Biophotonics. 13(11):e202000079, 2020 11.

[Journal Article. Research Support, Non-U.S. Gov't]

UI: 32686263

This study demonstrates the efficacy of Raman micro-spectroscopy of oral cytological samples for differentiating dysplastic, potentially malignant lesions from those of normal, healthy donors. Cells were collected using brush biopsy from healthy donors (n = 20) and patients attending a Dysplasia Clinic (n = 20). Donors were sampled at four different sites (buccal mucosa, tongue, alveolus, gingiva), to ensure matched normal sites for all lesions, while patient samples were taken from clinically evident, histologically verified dysplastic lesions. Spectra were acquired from the nucleus and cytoplasm of individual cells of all samples and subjected to partial least squares-discriminant analysis. Discriminative sensitivities of 94% and 86% and specificity of 85% were achieved for the cytoplasm and nucleus, respectively, largely based on lipidic contributions of dysplastic cells. Alveolar/gingival samples were differentiated from tongue/buccal samples, indicating that anatomical site is potentially a confounding factor, while age, gender, smoking and alcohol consumption were confirmed not to be.

Copyright © 2020 WILEY-VCH GmbH.

Version ID

1

Record Owner

From MEDLINE, a database of the U.S. National Library of Medicine.

Status

MEDLINE

Author NameID

Behl, Isha; ORCID: <https://orcid.org/0000-0003-0676-8988> Byrne, Hugh J; ORCID: <https://orcid.org/0000-0002-1735-8610>

Lyng, Fiona M; ORCID: <https://orcid.org/0000-0002-9876-963X>

Authors Full Name

Behl, Isha, Calado, Genecy, Malkin, Alison, Flint, Stephen, Galvin, Sheila, Healy, Claire M, Pimentel, Marina Leite, Byrne, Hugh J, Lyng, Fiona M

Institution

Behl, Isha. Centre for Radiation and Environmental Science, FOCAS Research Institute, Technological University Dublin. City Campus, Dublin, Ireland. Behl, Isha. School of Physics & Clinical & Optometric Sciences, Technological University Dublin, City Campus, Dublin, Ireland.

Calado, Genecy. Centre for Radiation and Environmental Science, FOCAS Research Institute, Technological University Dublin. City Campus, Dublin, Ireland.

Calado, Genecy. School of Physics & Clinical & Optometric Sciences, Technological University Dublin, City Campus, Dublin, Ireland.

Malkin, Alison. School of Biological and Health Sciences, Technological University Dublin, City Campus, Dublin, Ireland.

Flint, Stephen. Oral Medicine Unit, Dublin Dental University Hospital, Trinity College Dublin, Dublin, Ireland.

Galvin, Sheila. Oral Medicine Unit, Dublin Dental University Hospital, Trinity College Dublin, Dublin, Ireland.

Healy, Claire M. Oral Medicine Unit, Dublin Dental University Hospital, Trinity College Dublin, Dublin, Ireland.

Pimentel, Marina Leite. Division of Restorative Dentistry and Periodontology, Dublin Dental University Hospital, Trinity College Dublin, Dublin, Ireland.

Byrne, Hugh J. FOCAS Research Institute, Technological University Dublin, City Campus, Dublin, Ireland.

Lyng, Fiona M. Centre for Radiation and Environmental Science, FOCAS Research Institute, Technological University Dublin. City Campus, Dublin, Ireland.

Lyng, Fiona M. School of Physics & Clinical & Optometric Sciences, Technological University Dublin, City Campus, Dublin, Ireland.

MeSH Heading

Humans. Mouth Mucosa. Mouth Neoplasms/di [Diagnosis]. *Mouth Neoplasms. Pilot Projects. *Precancerous Conditions. Spectrum Analysis, Raman.

Keyword Heading

Raman micro-spectroscopy oral brush biopsy cytological samples

oral potentially malignant lesions

partial least squares-discriminant analysis

sensitivity and specificity.

Keyword Heading Owner

NOTNLM

Year of Publication

2020

Link to the Ovid Full Text or citation:

[Click here for full text options](https://ovidsp.ovid.com/ovidweb.cgi?T=JS&CSC=Y&NEWS=N&PAGE=fulltext&D=med18&AN=32686263)

Link to the External Link Resolver:

[SFX](https://sfx-86scu.hosted.exlibrisgroup.com.cn/86scu?sid=OVID:medline&id=pmid:32686263&id=doi:10.1002%2Fjbio.202000079&issn=1864063X&isbn=&volume=13&issue=11&spage=e202000079&pages=e202000079&date=2020&title=Journal+of+Biophotonics&atitle=A+pilot+study+for+early+detection+of+oral+premalignant+diseases+using+oral+cytology+and+Raman+micro-spectroscopy%3A+Assessment+of+confounding+factors.&aulast=Behl&pid=<author>Behl+I%3BCalado+G%3BMalkin+A%3BFlint+S%3BGalvin+S%3BHealy+CM%3BPimentel+ML%3BByrne+HJ%3BLyng+FM<%2Fauthor><AN>32686263<%2FAN><DT>Journal+Article<%2FDT>)

69.

[Application of Raman Spectroscopy in the Diagnosis of Oral Cancer]. [Review] [Chinese]

Zhu Z, Meng F, Xia J, Xu X, Hu Y, Zhang A, Zhang T

Chung-Kuo i Hsueh Ko Hsueh Yuan Hsueh Pao Acta Academiae Medicinae Sinicae. 42(3):399-404, 2020 Jun 30.

[Journal Article. Review]

UI: 32616139

Oral cancer is a common and deadly malignancy.While multidisciplinary treatment(mainly surgery)has been applied in the treatment of cancer treatment,early diagnosis and complete removal of the primary lesion are essential for a better prognosis.Raman spectroscopy is an optical technique that detects inelastic scattered light generated by the interaction of light and matter.It can detect the vibrational spectra of biochemical and biomolecular structures and tissue conformations,and can provide the "molecular fingerprint" for cells,tissues,and biological fluids.With the development of related technologies and optical instruments,Raman spectroscopy has been widely applied in medical fields. This article reviews the research advances and application of Raman spectroscopy in the diagnosis of oral cancer.

Version ID

1

Record Owner

From MEDLINE, a database of the U.S. National Library of Medicine.

Status

MEDLINE

Authors Full Name

Zhu, Zhihui, Meng, Fanhao, Xia, Jiabin, Xu, Xiaofeng, Hu, Yang, Zhang, Aijin, Zhang, Tao

Institution

Zhu, Zhihui. Department of Stomatology,PUMC Hospital,CAMS and PUMC,Beijing 100730,China. Meng, Fanhao. Department of Stomatology,PUMC Hospital,CAMS and PUMC,Beijing 100730,China.

Xia, Jiabin. Key Laboratory of the Ministry of Education for Optoelectronic Measurement Technology and Instrument, Beijing Information Science & Technology University,Beijing 100192,China.

Xu, Xiaofeng. Key Laboratory of the Ministry of Education for Optoelectronic Measurement Technology and Instrument, Beijing Information Science & Technology University,Beijing 100192,China.

Hu, Yang. Key Laboratory of the Ministry of Education for Optoelectronic Measurement Technology and Instrument, Beijing Information Science & Technology University,Beijing 100192,China.

Zhang, Aijin. Department of Stomatology,PUMC Hospital,CAMS and PUMC,Beijing 100730,China.

Zhang, Tao. Department of Stomatology,PUMC Hospital,CAMS and PUMC,Beijing 100730,China.

MeSH Heading

Humans. *Mouth Neoplasms. *Spectrum Analysis, Raman.

Keyword Heading

Raman spectrum diagnosis

molecular fingerprint

oral cancer.

Keyword Heading Owner

NOTNLM

Year of Publication

2020

Link to the Ovid Full Text or citation:

[Click here for full text options](https://ovidsp.ovid.com/ovidweb.cgi?T=JS&CSC=Y&NEWS=N&PAGE=fulltext&D=med18&AN=32616139)

Link to the External Link Resolver:

[SFX](https://sfx-86scu.hosted.exlibrisgroup.com.cn/86scu?sid=OVID:medline&id=pmid:32616139&id=doi:10.3881%2Fj.issn.1000-503X.11270&issn=1000503X&isbn=&volume=42&issue=3&spage=399&pages=399-404&date=2020&title=Chung-Kuo+i+Hsueh+Ko+Hsueh+Yuan+Hsueh+Pao+Acta+Academiae+Medicinae+Sinicae&atitle=%5BApplication+of+Raman+Spectroscopy+in+the+Diagnosis+of+Oral+Cancer%5D.&aulast=Zhu&pid=<author>Zhu+Z%3BMeng+F%3BXia+J%3BXu+X%3BHu+Y%3BZhang+A%3BZhang+T<%2Fauthor><AN>32616139<%2FAN><DT>Journal+Article<%2FDT>)

70.

Raman microspectroscopic study for the detection of oral field cancerisation using brush biopsy samples.

Behl I, Calado G, Vishwakarma A, Flint S, Galvin S, Healy CM, Leite Pimentel M, Malkin A, Byrne HJ, Lyng FM

Journal of Biophotonics. 13(10):e202000131, 2020 10.

[Journal Article. Research Support, Non-U.S. Gov't]

UI: 32602241

Field cancerisation (FC) is potentially an underlying cause of poor treatment outcomes of oral squamous cell carcinoma (OSCC). To explore the phenomenon using Raman microspectroscopy, brush biopsies from the buccal mucosa, tongue, gingiva and alveolus of healthy donors (n = 40) and from potentially malignant lesions (PML) of Dysplasia Clinic patients (n = 40) were examined. Contralateral normal samples (n = 38) were also collected from the patients. Raman spectra were acquired from the nucleus and cytoplasm of each cell, and subjected to partial least squares-discriminant analysis (PLS-DA). High discriminatory accuracy for donor and PML samples was achieved for both cytopalmic and nuclear data sets. Notably, contralateral normal (patient) samples were also accurately discriminated from donor samples and contralateral normal samples from patients with multiple lesions showed a similar spectral profile to PML samples, strongly indicating a FC effect. These findings support the potential of Raman microspectroscopy as a screening tool for PML using oral exfoliated cells.

Copyright © 2020 WILEY-VCH Verlag GmbH & Co. KGaA, Weinheim.

Version ID

1

Record Owner

From MEDLINE, a database of the U.S. National Library of Medicine.

Status

MEDLINE

Author NameID

Behl, Isha; ORCID: <https://orcid.org/0000-0003-0676-8988> Byrne, Hugh J; ORCID: <https://orcid.org/0000-0002-1735-8610>

Lyng, Fiona M; ORCID: <https://orcid.org/0000-0002-9876-963X>

Authors Full Name

Behl, Isha, Calado, Genecy, Vishwakarma, Anika, Flint, Stephen, Galvin, Sheila, Healy, Claire M, Leite Pimentel, Marina, Malkin, Alison, Byrne, Hugh J, Lyng, Fiona M

Institution

Behl, Isha. Centre for Radiation and Environmental Science, FOCAS Research Institute, Technological University Dublin, Dublin, Ireland. Behl, Isha. School of Physics, Technological University Dublin, Dublin, Ireland.

Calado, Genecy. Centre for Radiation and Environmental Science, FOCAS Research Institute, Technological University Dublin, Dublin, Ireland.

Calado, Genecy. School of Physics, Technological University Dublin, Dublin, Ireland.

Vishwakarma, Anika. Centre for Radiation and Environmental Science, FOCAS Research Institute, Technological University Dublin, Dublin, Ireland.

Vishwakarma, Anika. School of Physics, Technological University Dublin, Dublin, Ireland.

Flint, Stephen. Oral Medicine Unit, Dublin Dental University Hospital, Trinity College Dublin, Dublin, Ireland.

Galvin, Sheila. Oral Medicine Unit, Dublin Dental University Hospital, Trinity College Dublin, Dublin, Ireland.

Healy, Claire M. Oral Medicine Unit, Dublin Dental University Hospital, Trinity College Dublin, Dublin, Ireland.

Leite Pimentel, Marina. Division of Restorative Dentistry and Periodontology, Dublin Dental University Hospital, Trinity College Dublin, Dublin, Ireland.

Malkin, Alison. School of Biological Sciences, Technological University Dublin, Dublin, Ireland.

Byrne, Hugh J. FOCAS Research Institute, Technological University Dublin, Dublin, Ireland.

Lyng, Fiona M. Centre for Radiation and Environmental Science, FOCAS Research Institute, Technological University Dublin, Dublin, Ireland.

Lyng, Fiona M. School of Physics, Technological University Dublin, Dublin, Ireland.

MeSH Heading

Biopsy. *Carcinoma, Squamous Cell. *Head and Neck Neoplasms. Humans. Mouth Neoplasms/di [Diagnosis]. *Mouth Neoplasms. Spectrum Analysis, Raman.

Keyword Heading

Raman microspectroscopy contralateral normal

field cancerisation

oral brush biopsy cytological samples

oral potentially malignant lesions

partial least squares-discriminant analysis

sensitivity and specificity.

Keyword Heading Owner

NOTNLM

Year of Publication

2020

Link to the Ovid Full Text or citation:

[Click here for full text options](https://ovidsp.ovid.com/ovidweb.cgi?T=JS&CSC=Y&NEWS=N&PAGE=fulltext&D=med18&AN=32602241)

Link to the External Link Resolver:

[SFX](https://sfx-86scu.hosted.exlibrisgroup.com.cn/86scu?sid=OVID:medline&id=pmid:32602241&id=doi:10.1002%2Fjbio.202000131&issn=1864063X&isbn=&volume=13&issue=10&spage=e202000131&pages=e202000131&date=2020&title=Journal+of+Biophotonics&atitle=Raman+microspectroscopic+study+for+the+detection+of+oral+field+cancerisation+using+brush+biopsy+samples.&aulast=Behl&pid=<author>Behl+I%3BCalado+G%3BVishwakarma+A%3BFlint+S%3BGalvin+S%3BHealy+CM%3BLeite+Pimentel+M%3BMalkin+A%3BByrne+HJ%3BLyng+FM<%2Fauthor><AN>32602241<%2FAN><DT>Journal+Article<%2FDT>)

71.

Silver molybdate nanoparticles based immunosensor for the non-invasive detection of Interleukin-8 biomarker.

Pachauri N, Lakshmi GBVS, Sri S, Gupta PK, Solanki PR

Materials Science & Engineering. C, Materials for Biological Applications. 113:110911, 2020 Aug.

[Journal Article]

UI: 32487373

In this study, we report the silver molybdate nanoparticles (beta-Ag2MoO4 NPs) based non-invasive and sensitive electrochemical immunosensor for label-free detection of Interleukin-8 (IL-8) biomarker. The X-ray diffraction and Raman spectroscopy studies confirm the cubic spinel structures of beta-Ag2MoO4 NPs. High-resolution transmission electron microscopy study depicted average size of beta-Ag2MoO4 NPs as 27.15 nm. The cleaned indium tin oxide coated glass substrates were coated with spin-coated thin films of Ag2MoO4 NPs. These electrodes used for covalently immobilization of antibodies specific to IL-8 (Anti-IL-8) using EDC-NHS chemistry and unbound activated sites blocked by bovine serum albumin. Electrochemical response was obtained in the range of 1 fg mL-1 to 40 ng mL-1 and the sensitivity was found to be 7.03 muA ng-1mL cm-2 with LOD of 90 pg mL-1. Spiked samples prepared by human saliva were tested and found efficient detection with this immunoelectrode.

Copyright © 2020 Elsevier B.V. All rights reserved.

Version ID

1

Record Owner

From MEDLINE, a database of the U.S. National Library of Medicine.

Status

MEDLINE

Authors Full Name

Pachauri, Namrata, Lakshmi, G B V S, Sri, Smriti, Gupta, Pramod K, Solanki, Pratima R

Institution

Pachauri, Namrata. Special Centre for Nanoscience, Jawaharlal Nehru University, New Delhi 110067, India. Lakshmi, G B V S. Special Centre for Nanoscience, Jawaharlal Nehru University, New Delhi 110067, India.

Sri, Smriti. Special Centre for Nanoscience, Jawaharlal Nehru University, New Delhi 110067, India.

Gupta, Pramod K. Special Centre for Nanoscience, Jawaharlal Nehru University, New Delhi 110067, India.

Solanki, Pratima R. Special Centre for Nanoscience, Jawaharlal Nehru University, New Delhi 110067, India. Electronic address: partima.jnu@nic.in.

MeSH Heading

Antibodies, Immobilized/ch [Chemistry]. Antibodies, Immobilized/im [Immunology]. *Biomarkers/an [Analysis]. Electrochemical Techniques. Electrodes. Humans. *Immunoassay/mt [Methods]. *Interleukin-8/an [Analysis]. Interleukin-8/im [Immunology]. Limit of Detection. Molybdenum/ch [Chemistry]. *Nanoparticles/ch [Chemistry]. Particle Size. Reproducibility of Results. Saliva/me [Metabolism]. Silver/ch [Chemistry]. Tin Compounds/ch [Chemistry].

Keyword Heading

Electrochemical Interleukin-8, Immunosensor

Oral cancer

Silver molybdate.

Keyword Heading Owner

NOTNLM

Registry Number/Name of Substance

0 (Antibodies, Immobilized). 0 (Biomarkers). 0 (Interleukin-8). 0 (Tin Compounds). 14259-85-9 (molybdate). 3M4G523W1G (Silver). 71243-84-0 (indium tin oxide). 81AH48963U (Molybdenum).

Year of Publication

2020

Link to the Ovid Full Text or citation:

[Click here for full text options](https://ovidsp.ovid.com/ovidweb.cgi?T=JS&CSC=Y&NEWS=N&PAGE=fulltext&D=med18&AN=32487373)

Link to the External Link Resolver:

[SFX](https://sfx-86scu.hosted.exlibrisgroup.com.cn/86scu?sid=OVID:medline&id=pmid:32487373&id=doi:10.1016%2Fj.msec.2020.110911&issn=09284931&isbn=&volume=113&issue=&spage=110911&pages=110911&date=2020&title=Materials+Science+%26+Engineering.+C%2C+Materials+for+Biological+Applications&atitle=Silver+molybdate+nanoparticles+based+immunosensor+for+the+non-invasive+detection+of+Interleukin-8+biomarker.&aulast=Pachauri&pid=<author>Pachauri+N%3BLakshmi+GBVS%3BSri+S%3BGupta+PK%3BSolanki+PR<%2Fauthor><AN>32487373<%2FAN><DT>Journal+Article<%2FDT>)

72.

Preoperative and Intraoperative Methods of Parathyroid Gland Localization and the Diagnosis of Parathyroid Adenomas. [Review]

Baj J, Sitarz R, Lokaj M, Forma A, Czeczelewski M, Maani A, Garruti G

Molecules. 25(7), 2020 Apr 09.

[Journal Article. Meta-Analysis. Review]

UI: 32283730

Accurate pre-operative determination of parathyroid glands localization is critical in the selection of minimally invasive parathyroidectomy as a surgical treatment approach in patients with primary hyperparathyroidism (PHPT). Its importance cannot be overemphasized as it helps to minimize the harmful side effects associated with damage to the parathyroid glands such as in hypocalcemia, severe hemorrhage or recurrent laryngeal nerve dysfunction. Preoperative and intraoperative methods decrease the incidence of mistakenly injuring the parathyroid glands and allow for the timely diagnosis of various abnormalities, including parathyroid adenomas. This article reviews 139 studies conducted between 1970 and 2020 (49 years). Studies that were reviewed focused on several techniques including application of carbon nanoparticles, carbon nanoparticles with technetium sestamibi (99m Tc-MIBI), Raman spectroscopy, near-infrared autofluorescence, dynamic optical contrast imaging, laser speckle contrast imaging, shear wave elastography, and indocyanine green to test their potential in providing proper parathyroid glands' localization. Apart from reviewing the aforementioned techniques, this study focused on the applications that helped in the detection of parathyroid adenomas. Results suggest that applying all the reviewed techniques significantly improves the possibility of providing proper localization of parathyroid glands, and the application of indocyanine green has proven to be the 'ideal' approach for the diagnosis of parathyroid adenomas.

Version ID

1

Record Owner

From MEDLINE, a database of the U.S. National Library of Medicine.

Status

MEDLINE

Author NameID

Sitarz, Robert; ORCID: <https://orcid.org/0000-0001-7267-9516> Forma, Alicja; ORCID: <https://orcid.org/0000-0001-8714-7627>

Czeczelewski, Marcin; ORCID: <https://orcid.org/0000-0002-5576-4169>

Authors Full Name

Baj, Jacek, Sitarz, Robert, Lokaj, Marek, Forma, Alicja, Czeczelewski, Marcin, Maani, Amr, Garruti, Gabriella

Institution

Baj, Jacek. Chair and Department of Anatomy, Medical University of Lublin, 20-950 Lublin, Poland. Sitarz, Robert. Chair and Department of Anatomy, Medical University of Lublin, 20-950 Lublin, Poland.

Sitarz, Robert. Department of Surgery, Center of Oncology of the Lublin Region St. Jana z Dukli, 20-090 Lublin, Poland.

Lokaj, Marek. Department of Surgery, Center of Oncology of the Lublin Region St. Jana z Dukli, 20-090 Lublin, Poland.

Forma, Alicja. Chair and Department of Anatomy, Medical University of Lublin, 20-950 Lublin, Poland.

Czeczelewski, Marcin. Chair and Department of Forensic Medicine, Medical University of Lublin, 20-950 Lublin, Poland.

Maani, Amr. Chair and Department of Anatomy, Medical University of Lublin, 20-950 Lublin, Poland.

Garruti, Gabriella. Section of Endocrinology, Andrology and Metabolic Diseases, Department of Emergency and Organ Transplantations, University of Bari "Aldo Moro" Medical School, 70124 Bari, Italy.

MeSH Heading

Clinical Decision-Making. Disease Management. Humans. Intraoperative Care/mt [Methods]. *Intraoperative Care. Multimodal Imaging/mt [Methods]. Parathyroid Glands/dg [Diagnostic Imaging]. *Parathyroid Glands/pa [Pathology]. *Parathyroid Glands/su [Surgery]. *Parathyroid Neoplasms/di [Diagnosis]. *Parathyroid Neoplasms/su [Surgery]. Preoperative Care/mt [Methods]. *Preoperative Care. Radiopharmaceuticals. Spectrum Analysis, Raman. Technetium Tc 99m Sestamibi. Theranostic Nanomedicine.

Keyword Heading

Raman spectroscopy autofluorescence

carbon nanoparticles

dynamic optical contrast imaging

imaging techniques

indocyanine green

laser speckle contrast imaging

parathyroid adenoma

parathyroid gland

shear wave elastography.

Keyword Heading Owner

NOTNLM

Registry Number/Name of Substance

0 (Radiopharmaceuticals). 971Z4W1S09 (Technetium Tc 99m Sestamibi).

Year of Publication

2020

Link to the Ovid Full Text or citation:

[Click here for full text options](https://ovidsp.ovid.com/ovidweb.cgi?T=JS&CSC=Y&NEWS=N&PAGE=fulltext&D=med18&AN=32283730)

Link to the External Link Resolver:

[SFX](https://sfx-86scu.hosted.exlibrisgroup.com.cn/86scu?sid=OVID:medline&id=pmid:32283730&id=doi:10.3390%2Fmolecules25071724&issn=14203049&isbn=&volume=25&issue=7&spage=1724&pages=&date=2020&title=Molecules&atitle=Preoperative+and+Intraoperative+Methods+of+Parathyroid+Gland+Localization+and+the+Diagnosis+of+Parathyroid+Adenomas.&aulast=Baj&pid=<author>Baj+J%3BSitarz+R%3BLokaj+M%3BForma+A%3BCzeczelewski+M%3BMaani+A%3BGarruti+G<%2Fauthor><AN>32283730<%2FAN><DT>Journal+Article<%2FDT>)

73.

Filter-Membrane-Based Ultrafiltration Coupled with Surface-Enhanced Raman Spectroscopy for Potential Differentiation of Benign and Malignant Thyroid Tumors from Blood Plasma.

Liang X, Miao X, Xiao W, Ye Q, Wang S, Lin J, Li C, Huang Z

International Journal of Nanomedicine. 15:2303-2314, 2020.

[Journal Article]

UI: 32280222

OBJECTIVE: The objective of this study is to evaluate the performance and feasibility of surface-enhanced Raman spectroscopy coupled with a filter membrane and advanced multivariate data analysis on identifying and differentiating benign and malignant thyroid tumors from blood plasma.

PATIENTS AND METHODS: We proposed a membrane filter SERS technology for the differentiation between benign thyroid tumor and thyroid cancer. That is to say, by using filter membranes with optimal pore size, the blood plasma samples from thyroid tumor patients were pretreated with the macromolecular proteins being filtered out prior to SERS measurement. The SERS spectra of blood plasma ultrafiltrate obtained using filter membranes from 102 patients with thyroid tumors (70 thyroid cancers and 32 benign thyroid tumors) were then analyzed and compared. Two multivariate statistical analyses, principal component analysis-linear discriminate analysis (PCA-LDA) and Lasso-partial least squares-discriminant analysis (Lasso-PLS-DA), were performed on the SERS spectral data after background subtraction and normalization, as well as the first derivative processing, to analyze and compare the differential diagnosis of benign thyroid tumors and thyroid cancer.

RESULTS: SERS measurements were performed in blood plasma acquired from a total of 102 thyroid tumor patients (benign thyroid tumor N=32; thyroid cancer N=70). By using filter membranes, the macromolecular proteins in blood plasma were effectively filtered out to yield high-quality SERS spectra. 84.3% discrimination accuracy between benign and malignant thyroid tumor was achieved using PCA-LDA method, while Lasso-PLS-DA yields a discrimination accuracy of 90.2%.

CONCLUSION: Our results demonstrate that SERS spectroscopy, coupled with ultrafiltration and multivariate analysis has the potential of providing a non-invasive, rapid, and objective detection and differentiation of benign and malignant thyroid tumors.

Copyright © 2020 Liang et al.

Version ID

1

Record Owner

From MEDLINE, a database of the U.S. National Library of Medicine.

Status

MEDLINE

Author NameID

Liang, Xiaozhou; ORCID: <https://orcid.org/0000-0003-4594-0226> Ye, Qin; ORCID: <https://orcid.org/0000-0001-5545-3777>

Authors Full Name

Liang, Xiaozhou, Miao, Xuchao, Xiao, Weijin, Ye, Qin, Wang, Sisi, Lin, Juqiang, Li, Chao, Huang, Zufang

Institution

Liang, Xiaozhou. Fujian Normal University, Ministry of Education, Key Laboratory of Optoelectronic Science and Technology for Medicine, Fujian Provincial Key Laboratory for Photonics Technology, Fuzhou, People's Republic of China. Miao, Xuchao. Fujian Normal University, Ministry of Education, Key Laboratory of Optoelectronic Science and Technology for Medicine, Fujian Provincial Key Laboratory for Photonics Technology, Fuzhou, People's Republic of China.

Xiao, Weijin. Department of Pathology, School of Basic Medical Sciences, Fujian Medical University, Fuzhou, People's Republic of China.

Ye, Qin. Department of Head and Neck Surgery, Fujian Cancer Hospital, Fujian Medical University Cancer Hospital, Fuzhou, People's Republic of China.

Wang, Sisi. Department of General Surgery, Fujian Medical University Union Hospital, Fuzhou, People's Republic of China.

Lin, Juqiang. Fujian Normal University, Ministry of Education, Key Laboratory of Optoelectronic Science and Technology for Medicine, Fujian Provincial Key Laboratory for Photonics Technology, Fuzhou, People's Republic of China.

Li, Chao. Department of Pathology, School of Basic Medical Sciences, Fujian Medical University, Fuzhou, People's Republic of China.

Li, Chao. Department of Pathology, Fujian Cancer Hospital, Fujian Medical University Cancer Hospital, Fuzhou, People's Republic of China.

Li, Chao. Fujian Provincial Key Laboratory of Translational Cancer Medicine, Fuzhou, People's Republic of China.

Huang, Zufang. Fujian Normal University, Ministry of Education, Key Laboratory of Optoelectronic Science and Technology for Medicine, Fujian Provincial Key Laboratory for Photonics Technology, Fuzhou, People's Republic of China.

MeSH Heading

Adult. Diagnosis, Differential. Discriminant Analysis. Humans. Membranes, Artificial. Metal Nanoparticles/ch [Chemistry]. Middle Aged. Multivariate Analysis. *Plasma/ch [Chemistry]. Principal Component Analysis. Proof of Concept Study. Silver/ch [Chemistry]. *Spectrum Analysis, Raman/mt [Methods]. *Thyroid Neoplasms/bl [Blood]. Thyroid Neoplasms/di [Diagnosis]. Thyroid Neoplasms/pa [Pathology]. Ultrafiltration/is [Instrumentation]. *Ultrafiltration/mt [Methods].

Keyword Heading

blood plasma filter membrane

silver nanoparticles

surface-enhanced Raman spectroscopy

thyroid tumor.

Keyword Heading Owner

NOTNLM

Registry Number/Name of Substance

0 (Membranes, Artificial). 3M4G523W1G (Silver).

Year of Publication

2020

Link to the Ovid Full Text or citation:

[Click here for full text options](https://ovidsp.ovid.com/ovidweb.cgi?T=JS&CSC=Y&NEWS=N&PAGE=fulltext&D=med18&AN=32280222)

Link to the External Link Resolver:

[SFX](https://sfx-86scu.hosted.exlibrisgroup.com.cn/86scu?sid=OVID:medline&id=pmid:32280222&id=doi:10.2147%2FIJN.S233663&issn=11769114&isbn=&volume=15&issue=&spage=2303&pages=2303-2314&date=2020&title=International+Journal+of+Nanomedicine&atitle=Filter-Membrane-Based+Ultrafiltration+Coupled+with+Surface-Enhanced+Raman+Spectroscopy+for+Potential+Differentiation+of+Benign+and+Malignant+Thyroid+Tumors+from+Blood+Plasma.&aulast=Liang&pid=<author>Liang+X%3BMiao+X%3BXiao+W%3BYe+Q%3BWang+S%3BLin+J%3BLi+C%3BHuang+Z<%2Fauthor><AN>32280222<%2FAN><DT>Journal+Article<%2FDT>)

74.

Surface-enhanced Raman spectroscopy (SERS) investigations of saliva for oral cancer diagnosis.

Falamas A, Rotaru H, Hedesiu M

Lasers in Medical Science. 35(6):1393-1401, 2020 Aug.

[Journal Article]

UI: 32170505

Saliva could be an optimal sample for non-invasive cancer detection, as it contains plenty of proteins and metabolites which can reflect the health status of an individual. Moreover, pairing it with high-sensitivity, label-free detection techniques could prove successful for early cancer diagnosis. In this study, we explore the enhancement of salivary characteristic Raman bands by using label-free, ultrasensitive surface-enhanced Raman scattering (SERS) based on gold nanoparticles. SERS maps were acquired from dry samples of saliva supernatant mixed with Au colloidal nanoparticles, which was then pipetted on clean glass slides. The SERS spectra presented a high variability of signal intensities and frequency shifts. However, several reproducible SERS spectra showing well-resolved bands were obtained at certain locations on the maps, where Au nanoparticles clustered together during the air-drying. The healthy and oral cancer saliva could be differentiated using principal components analysis based on several SERS bands assigned mainly to amino acids and proteins. Moreover, thiocyanate Raman modes were detected in saliva samples of both smoking and non-smoking volunteers and cancer patients. The analysis indicated that the cancer group displayed an overall higher level of the 2126 cm-1 band area assigned to C-N stretching vibrations of thiocyanate.

Version ID

1

Record Owner

From MEDLINE, a database of the U.S. National Library of Medicine.

Status

MEDLINE

Author NameID

Falamas, A; ORCID: <https://orcid.org/0000-0001-6449-057X> Rotaru, H; ORCID: <https://orcid.org/0000-0003-2138-3322>

Authors Full Name

Falamas, A, Rotaru, H, Hedesiu, M

Institution

Falamas, A. National Institute for Research and Development of Isotopic and Molecular Technologies, 67-103 Donat, 400293, Cluj-Napoca, Romania. afalamas@itim-cj.ro. Rotaru, H. Department of Oral and Maxillofacial Surgery, "Iuliu-Hateganu" University of Medicine and Pharmacy, Cluj-Napoca, Romania.

Hedesiu, M. Department of Oral Radiology, Faculty of Dental Medicine, "Iuliu-Hateganu" University of Medicine and Pharmacy, Cluj-Napoca, Romania.

MeSH Heading

Adult. Female. Gold/ch [Chemistry]. Humans. Male. Metal Nanoparticles/ch [Chemistry]. Middle Aged. *Mouth Neoplasms/di [Diagnosis]. Principal Component Analysis. *Saliva/ch [Chemistry]. *Spectrum Analysis, Raman. Thiocyanates/an [Analysis].

Keyword Heading

Diagnosis Oral cancer

Saliva

Surface-enhanced Raman spectroscopy.

Keyword Heading Owner

NOTNLM

Registry Number/Name of Substance

0 (Thiocyanates). 7440-57-5 (Gold). O748SU14OM (thiocyanate).

Year of Publication

2020

Link to the Ovid Full Text or citation:

[Click here for full text options](https://ovidsp.ovid.com/ovidweb.cgi?T=JS&CSC=Y&NEWS=N&PAGE=fulltext&D=med17&AN=32170505)

Link to the External Link Resolver:

[SFX](https://sfx-86scu.hosted.exlibrisgroup.com.cn/86scu?sid=OVID:medline&id=pmid:32170505&id=doi:10.1007%2Fs10103-020-02988-2&issn=02688921&isbn=&volume=35&issue=6&spage=1393&pages=1393-1401&date=2020&title=Lasers+in+Medical+Science&atitle=Surface-enhanced+Raman+spectroscopy+(SERS)+investigations+of+saliva+for+oral+cancer+diagnosis.&aulast=Falamas&pid=<author>Falamas+A%3BRotaru+H%3BHedesiu+M<%2Fauthor><AN>32170505<%2FAN><DT>Journal+Article<%2FDT>)

75.

Combined Morpho-Chemical Profiling of Individual Extracellular Vesicles and Functional Nanoparticles without Labels.

Dai Y, Bai S, Hu C, Chu K, Shen B, Smith ZJ

Analytical Chemistry. 92(7):5585-5594, 2020 04 07.

[Journal Article. Research Support, Non-U.S. Gov't]

UI: 32162516

Biological nanoparticles are important targets of study, yet their small size and tendency to aggregate makes their heterogeneity difficult to profile on a truly single-particle basis. Here we present a label-free system called 'Raman-enabled nanoparticle trapping analysis' (R-NTA) that optically traps individual nanoparticles, records Raman spectra and tracks particle motion to identify chemical composition, size, and refractive index. R-NTA has the unique capacity to characterize aggregation status and absolute chemical concentration at the single-particle level. We validate the method on NIST standards and liposomes, demonstrating that R-NTA can accurately characterize size and chemical heterogeneity, including determining combined morpho-chemical properties such as the number of lamellae in individual liposomes. Applied to extracellular vesicles (EVs), we find distinct differences between EVs from cancerous and noncancerous cells, and that knockdown of the TRPP2 ion channel, which is pathologically highly expressed in laryngeal cancer cells, leads the EVs to more closely resemble EVs from normal epithelial cells. Intriguingly, the differences in EV content are found in small subpopulations of EVs, highlighting the importance of single-particle measurements. These experiments demonstrate the power of the R-NTA system to measure and characterize the morpho-chemical heterogeneity of bionanoparticles.

Version ID

1

Record Owner

From MEDLINE, a database of the U.S. National Library of Medicine.

Status

MEDLINE

Author NameID

Dai, Yichuan; ORCID: <https://orcid.org/0000-0001-7907-2821> Smith, Zachary J; ORCID: <https://orcid.org/0000-0002-7946-7863>

Authors Full Name

Dai, Yichuan, Bai, Suwen, Hu, Chuanzhen, Chu, Kaiqin, Shen, Bing, Smith, Zachary J

Institution

Dai, Yichuan. Key Laboratory of Precision Scientific Instrumentation of Anhui Higher Education Institutes, Dept. of Precision Machinery and Precision Instrumentation, University of Science and Technology of China, Hefei, Anhui 230026, China. Bai, Suwen. Department of Physiology, School of Basic Medical Sciences, Anhui Medical University, Hefei 230026, China.

Hu, Chuanzhen. Key Laboratory of Precision Scientific Instrumentation of Anhui Higher Education Institutes, Dept. of Precision Machinery and Precision Instrumentation, University of Science and Technology of China, Hefei, Anhui 230026, China.

Chu, Kaiqin. Key Laboratory of Precision Scientific Instrumentation of Anhui Higher Education Institutes, Dept. of Precision Machinery and Precision Instrumentation, University of Science and Technology of China, Hefei, Anhui 230026, China.

Chu, Kaiqin. Hefei National Laboratory for Physical Sciences at the Microscale, University of Science and Technology of China, Hefei, Anhui 230026, China.

Shen, Bing. Department of Physiology, School of Basic Medical Sciences, Anhui Medical University, Hefei 230026, China.

Smith, Zachary J. Key Laboratory of Precision Scientific Instrumentation of Anhui Higher Education Institutes, Dept. of Precision Machinery and Precision Instrumentation, University of Science and Technology of China, Hefei, Anhui 230026, China.

MeSH Heading

Cell Line, Tumor. *Extracellular Vesicles/ch [Chemistry]. Humans. *Nanoparticles/ch [Chemistry]. Particle Size. Spectrum Analysis, Raman.

Year of Publication

2020

Link to the Ovid Full Text or citation:

[Click here for full text options](https://ovidsp.ovid.com/ovidweb.cgi?T=JS&CSC=Y&NEWS=N&PAGE=fulltext&D=med17&AN=32162516)

Link to the External Link Resolver:

[SFX](https://sfx-86scu.hosted.exlibrisgroup.com.cn/86scu?sid=OVID:medline&id=pmid:32162516&id=doi:10.1021%2Facs.analchem.0c00607&issn=00032700&isbn=&volume=92&issue=7&spage=5585&pages=5585-5594&date=2020&title=Analytical+Chemistry&atitle=Combined+Morpho-Chemical+Profiling+of+Individual+Extracellular+Vesicles+and+Functional+Nanoparticles+without+Labels.&aulast=Dai&pid=<author>Dai+Y%3BBai+S%3BHu+C%3BChu+K%3BShen+B%3BSmith+ZJ<%2Fauthor><AN>32162516<%2FAN><DT>Journal+Article<%2FDT>)

76.

Emerging Advanced Technologies Developed by IPR for Bio Medical Applications -.A Review. [Review]

Vaid A, Patil C, Sanghariyat A, Rane R, Visani A, Mukherjee S, Joseph A, Ranjan M, Augustine S, Sooraj KP, Rathore V, Nema SK, Agraj A, Garg G, Sharma A, Sharma M, Pansare K, Krishna CM, Banerjee J, Chandra S

Neurology India. 68(1):26-34, 2020 Jan-Feb.

[Journal Article. Review]

UI: 32129239

Over the last decade, research has intensified worldwide on the use of low-temperature plasmas in medicine and healthcare. Researchers have discovered many methods of applying plasmas to living tissues to deactivate pathogens; to end the flow of blood without damaging healthy tissue; to sanitize wounds and accelerate its healing; and to selectively kill malignant cancer cells. This review paper presents the latest development of advanced and plasma-based technologies used for applications in neurology in particular. Institute for Plasma Research (IPR), an aided institute of the Department of Atomic Energy (DAE), has also developed various technologies in some of these areas. One of these is an Atmospheric Pressure Plasma Jet (APPJ). This device is being studied to treat skin diseases, for coagulation of blood at faster rates and its interaction with oral, lung, and brain cancer cells. In certain cases, in-vitro studies have yielded encouraging results and limited in-vivo studies have been initiated. Plasma activated water has been produced in the laboratory for microbial disinfection, with potential applications in the health sector. Recently, plasmonic nanoparticle arrays which allow detection of very low concentrations of chemicals is studied in detail to allow early-stage detection of diseases. IPR has also been developing AI-based software called DeepCXR and AIBacilli for automated, high-speed screening and detection of footprints of tuberculosis (TB) in Chest X-ray images and for recognizing single/multiple TB bacilli in sputum smear test images, respectively. Deep Learning systems are increasingly being used around the world for analyzing electroencephalogram (EEG) signals for emotion recognition, mental workload, and seizure detection.

Version ID

1

Record Owner

From MEDLINE, a database of the U.S. National Library of Medicine.

Status

MEDLINE

Authors Full Name

Vaid, A, Patil, C, Sanghariyat, A, Rane, R, Visani, A, Mukherjee, S, Joseph, Alphonsa, Ranjan, M, Augustine, S, Sooraj, K P, Rathore, V, Nema, S K, Agraj, A, Garg, G, Sharma, A, Sharma, M, Pansare, K, Krishna, C Murali, Banerjee, Jyotirmoy, Chandra, Sarat

Institution

Vaid, A. Institute for Plasma Research, Gandhinagar, Gujarat, India. Patil, C. Institute for Plasma Research, Gandhinagar, Gujarat, India.

Sanghariyat, A. Institute for Plasma Research, Gandhinagar, Gujarat, India.

Rane, R. Institute for Plasma Research, Gandhinagar, Gujarat, India.

Visani, A. Institute for Plasma Research, Gandhinagar, Gujarat, India.

Mukherjee, S. Institute for Plasma Research, Gandhinagar, Gujarat, India.

Joseph, Alphonsa. Institute for Plasma Research, Gandhinagar, Gujarat, India.

Ranjan, M. Institute for Plasma Research, Gandhinagar, Gujarat, India.

Augustine, S. Institute for Plasma Research, Gandhinagar, Gujarat, India.

Sooraj, K P. Institute for Plasma Research, Gandhinagar, Gujarat, India.

Rathore, V. Institute for Plasma Research, Gandhinagar, Gujarat, India.

Nema, S K. Institute for Plasma Research, Gandhinagar, Gujarat, India.

Agraj, A. Institute for Plasma Research, Gandhinagar, Gujarat, India.

Garg, G. Institute for Plasma Research, Gandhinagar, Gujarat, India.

Sharma, A. Institute for Plasma Research, Gandhinagar, Gujarat, India.

Sharma, M. Institute for Plasma Research, Gandhinagar, Gujarat, India.

Pansare, K. Institute for Plasma Research, Gandhinagar, Gujarat, India.

Krishna, C Murali. Advanced Centre for Treatment, Research and Education in Cancer, TMC, Mumbai, Maharashtra, India.

Banerjee, Jyotirmoy. Department of Neurosurgery, AIIMS, New Delhi, India.

Chandra, Sarat. Advanced Centre for Treatment, Research and Education in Cancer, TMC, Mumbai, Maharashtra, India.

MeSH Heading

Academies and Institutes. Deep Learning. Humans. *Neoplasms/dg [Diagnostic Imaging]. *Neoplasms/su [Surgery]. Plasma/dg [Diagnostic Imaging]. Spectrum Analysis, Raman/mt [Methods]. *Tuberculosis/dg [Diagnostic Imaging]. *Tuberculosis/su [Surgery].

Keyword Heading

Atmospheric pressure plasma jet Surface-enhanced Raman spectroscopy

deep learning

plasma active medium.

Keyword Heading Owner

NOTNLM

Year of Publication

2020

Link to the Ovid Full Text or citation:

[Click here for full text options](https://ovidsp.ovid.com/ovidweb.cgi?T=JS&CSC=Y&NEWS=N&PAGE=fulltext&D=med17&AN=32129239)

Link to the External Link Resolver:

[SFX](https://sfx-86scu.hosted.exlibrisgroup.com.cn/86scu?sid=OVID:medline&id=pmid:32129239&id=doi:10.4103%2F0028-3886.279707&issn=00283886&isbn=&volume=68&issue=1&spage=26&pages=26-34&date=2020&title=Neurology+India&atitle=Emerging+Advanced+Technologies+Developed+by+IPR+for+Bio+Medical+Applications+-.A+Review.&aulast=Vaid&pid=<author>Vaid+A%3BPatil+C%3BSanghariyat+A%3BRane+R%3BVisani+A%3BMukherjee+S%3BJoseph+A%3BRanjan+M%3BAugustine+S%3BSooraj+KP%3BRathore+V%3BNema+SK%3BAgraj+A%3BGarg+G%3BSharma+A%3BSharma+M%3BPansare+K%3BKrishna+CM%3BBanerjee+J%3BChandra+S<%2Fauthor><AN>32129239<%2FAN><DT>Journal+Article<%2FDT>)

77.

Can ethanol affect the cell structure? A dynamic molecular and Raman spectroscopy study.

Carvalho LFCS, Dos Santos L, Bonnier F, O'Callaghan K, O'Sullivan J, Flint S, Neto LPM, Martin AA, Lyng FM, Byrne HJ

Photodiagnosis & Photodynamic Therapy. 30:101675, 2020 Jun.

[Journal Article]

UI: 31991233

The role that tobacco consumption plays in the etiology of oral cancer carcinogenesis, and of alcohol consumption acting as a co-factor, have been well established. However, in recent years, the contribution of alcohol consumption alone to oral cancer has been proposed. In fact, a high percentage of patients who develop oral cancer have both habits (tobacco and alcohol consumption), and other small patient groups only consume alcohol or do not have any other identifiable bad habits. In the present study we demonstrate, using a combination of dynamic molecular modelling and Raman spectroscopy, that ethanol has a significant effect on oral cells in vitro, mainly interacting with the lipids of the cell membrane, changing their conformation. Thus, it is possible to conclude that ethanol can affect the cell permeability, and by consequence serve as a possible trigger in oral carcinogenesis.

Copyright © 2020 Elsevier B.V. All rights reserved.

Version ID

1

Record Owner

From MEDLINE, a database of the U.S. National Library of Medicine.

Status

MEDLINE

Authors Full Name

Carvalho, Luis Felipe C S, Dos Santos, Laurita, Bonnier, Franck, O'Callaghan, Kate, O'Sullivan, Jeff, Flint, Stephen, Neto, Lazaro P M, Martin, Airton A, Lyng, Fiona M, Byrne, Hugh J

Institution

Carvalho, Luis Felipe C S. FOCAS Research Institute, Technological University Dublin, Kevin Street, Dublin 8, Ireland; Universidade de Taubate, Taubate, Brazil; Centro Universitario Braz Cubas, Mogi das Cruzes, SP, Brazil. Electronic address: luisfelipecarvalho@hotmail.com. Dos Santos, Laurita. Laboratory of Biomedical Vibrational Spectroscopy, Universidade Brasil, Sao Paulo, SP, Brazil.

Bonnier, Franck. Universite Francois-Rabelais de Tours, Faculty of Pharmacy, EA 6295 Nanomedicaments et Nanosondes, 31 Avenue Monge, 37200, Tours, France.

O'Callaghan, Kate. Dublin Dental School and Hospital, Trinity College Dublin, Dublin 2, Ireland.

O'Sullivan, Jeff. Dublin Dental School and Hospital, Trinity College Dublin, Dublin 2, Ireland.

Flint, Stephen. Dublin Dental School and Hospital, Trinity College Dublin, Dublin 2, Ireland.

Neto, Lazaro P M. Laboratory of Biomedical Vibrational Spectroscopy, Universidade Brasil, Sao Paulo, SP, Brazil.

Martin, Airton A. Laboratory of Biomedical Vibrational Spectroscopy, Universidade Brasil, Sao Paulo, SP, Brazil.

Lyng, Fiona M. Radiation and Environmental Science Centre, FOCAS Research Institute, Technological University Dublin, Kevin Street, Dublin 8, Ireland; School of Physics, Technological University Dublin, Kevin Street, Dublin 8, Ireland.

Byrne, Hugh J. FOCAS Research Institute, Technological University Dublin, Kevin Street, Dublin 8, Ireland.

MeSH Heading

Alcohol Drinking. *Ethanol. Humans. Photochemotherapy/mt [Methods]. *Photochemotherapy. Photosensitizing Agents. Spectrum Analysis, Raman.

Keyword Heading

Carcinogenesis Dynamic molecular modelling

Ethanol

Lipids

Oral cancer

Raman spectroscopy.

Keyword Heading Owner

NOTNLM

Registry Number/Name of Substance

0 (Photosensitizing Agents). 3K9958V90M (Ethanol).

Year of Publication

2020

Link to the Ovid Full Text or citation:

[Click here for full text options](https://ovidsp.ovid.com/ovidweb.cgi?T=JS&CSC=Y&NEWS=N&PAGE=fulltext&D=med17&AN=31991233)

Link to the External Link Resolver:

[SFX](https://sfx-86scu.hosted.exlibrisgroup.com.cn/86scu?sid=OVID:medline&id=pmid:31991233&id=doi:10.1016%2Fj.pdpdt.2020.101675&issn=15721000&isbn=&volume=30&issue=&spage=101675&pages=101675&date=2020&title=Photodiagnosis+%26+Photodynamic+Therapy&atitle=Can+ethanol+affect+the+cell+structure%3F+A+dynamic+molecular+and+Raman+spectroscopy+study.&aulast=Carvalho&pid=<author>Carvalho+LFCS%3BDos+Santos+L%3BBonnier+F%3BO'Callaghan+K%3BO'Sullivan+J%3BFlint+S%3BNeto+LPM%3BMartin+AA%3BLyng+FM%3BByrne+HJ<%2Fauthor><AN>31991233<%2FAN><DT>Journal+Article<%2FDT>)

78.

Promoting Active Sites in MOF-Derived Homobimetallic Hollow Nanocages as a High-Performance Multifunctional Nanozyme Catalyst for Biosensing and Organic Pollutant Degradation.

Li S, Hou Y, Chen Q, Zhang X, Cao H, Huang Y

Acs Applied Materials & Interfaces. 12(2):2581-2590, 2020 Jan 15.

[Journal Article]

UI: 31854974

Nanozymes are one of the ideal alternatives to natural enzymes for various applications. The rational design of nanozymes with improved catalytic activity stimulates increasing attention to address the low activity of current nanozymes. Here, we reported a general strategy to fabricate the Co-based homobimetallic hollow nanocages (HNCs) (C-CoM-HNC, M = Ni, Mn, Cu, and Zn) by ion-assistant solvothermal reaction and subsequent low-temperature calcination from metal-organic frameworks. The C-CoM-HNCs are featured with HNCs composed of interlaced nanosheets with homogeneous bimetallic oxide dispersion. The hierarchical structure and secondary metallic doping endow the C-CoM-HNC highly active sites. In particular, the Cu-doped C-CoCu-HNCs nanostructures exhibit superior performances over the other C-CoM-HNC as both the oxidase mimicking and peroxymonosulfate (PMS) activator. A sensitive bioassay for acetylcholinesterase (AChE) was established based on the excellent oxidase-like activity of C-CoCu-HNC, offering a linear detection range from 0.0001 to 1 mU/mL with an ultralow detection limit of 0.1 mU/L. As the PMS activator, the C-CoCu-HNC was applied for targeted organic pollutant (rhodamine B, RhB) degradation. A highly efficient RhB degradation was realized, along with good adaptability in a wide pH range and good reusability during the eight-cycle run. The results suggest that C-CoCu-HNC holds a practical potential for clinical diagnostics and pollution removal. Further density functional theory calculation reveals that Cu doping leads to a tighter connection and more negative adsorption energy for O2/PMS, as well as an upshifted d-band center in the C-CoCu-HNCs nanostructures. These changes facilitated the adsorption of O2/PMS on the C-CoCu-HNC surface for dissociation. This work not only offers a promising multifunctional nanozyme catalyst for clinical diagnostics and pollution removal but also gives some clues for the further development of novel nanozymes with high catalytic activities.

Version ID

1

Record Owner

From MEDLINE, a database of the U.S. National Library of Medicine.

Status

MEDLINE

Author NameID

Cao, Haiyan; ORCID: <http://orcid.org/0000-0002-8504-1631> Huang, Yuming; ORCID: <http://orcid.org/0000-0001-7775-6787>

Authors Full Name

Li, Siqi, Hou, Yuejie, Chen, Qiumeng, Zhang, Xiaodan, Cao, Haiyan, Huang, Yuming

Institution

Li, Siqi. The Key Laboratory of Luminescence and Real-time Analytical Chemistry, Ministry of Education, College of Chemistry and Chemical Engineering, Southwest University, Chongqing 400715, China. Hou, Yuejie. The Key Laboratory of Luminescence and Real-time Analytical Chemistry, Ministry of Education, College of Chemistry and Chemical Engineering, Southwest University, Chongqing 400715, China.

Chen, Qiumeng. The Key Laboratory of Luminescence and Real-time Analytical Chemistry, Ministry of Education, College of Chemistry and Chemical Engineering, Southwest University, Chongqing 400715, China.

Zhang, Xiaodan. The Key Laboratory of Luminescence and Real-time Analytical Chemistry, Ministry of Education, College of Chemistry and Chemical Engineering, Southwest University, Chongqing 400715, China.

Cao, Haiyan. The Key Laboratory of Chongqing Inorganic Special Functional Materials, College of Chemistry and Chemical Engineering, Yangtze Normal University, Chongqing 408100, China.

Huang, Yuming. The Key Laboratory of Luminescence and Real-time Analytical Chemistry, Ministry of Education, College of Chemistry and Chemical Engineering, Southwest University, Chongqing 400715, China.

MeSH Heading

Acetylcholinesterase/an [Analysis]. Biological Assay. *Biosensing Techniques. Catalysis. *Catalytic Domain. Cholinesterase Inhibitors/an [Analysis]. Density Functional Theory. *Metal-Organic Frameworks/ch [Chemistry]. Models, Molecular. *Nanostructures/ch [Chemistry]. Nanostructures/ul [Ultrastructure]. *Organic Chemicals/an [Analysis]. Oxidoreductases/me [Metabolism]. Photoelectron Spectroscopy. Rhodamines/an [Analysis]. Spectrophotometry, Ultraviolet. Spectrum Analysis, Raman. *Water Pollutants, Chemical/an [Analysis]. X-Ray Diffraction.

Keyword Heading

MOF derivatives RhB degradation

acetylcholinesterase

nanozyme

oxidase-mimicking

peroxymonosulfate.

Keyword Heading Owner

NOTNLM

Registry Number/Name of Substance

0 (Cholinesterase Inhibitors). 0 (Metal-Organic Frameworks). 0 (Organic Chemicals). 0 (Rhodamines). 0 (Water Pollutants, Chemical). EC 1 (Oxidoreductases). EC 3-1-1-7 (Acetylcholinesterase). K7G5SCF8IL (rhodamine B).

Year of Publication

2020

Link to the Ovid Full Text or citation:

[Click here for full text options](https://ovidsp.ovid.com/ovidweb.cgi?T=JS&CSC=Y&NEWS=N&PAGE=fulltext&D=med17&AN=31854974)

Link to the External Link Resolver:

[SFX](https://sfx-86scu.hosted.exlibrisgroup.com.cn/86scu?sid=OVID:medline&id=pmid:31854974&id=doi:10.1021%2Facsami.9b20275&issn=19448244&isbn=&volume=12&issue=2&spage=2581&pages=2581-2590&date=2020&title=Acs+Applied+Materials+%26+Interfaces&atitle=Promoting+Active+Sites+in+MOF-Derived+Homobimetallic+Hollow+Nanocages+as+a+High-Performance+Multifunctional+Nanozyme+Catalyst+for+Biosensing+and+Organic+Pollutant+Degradation.&aulast=Li&pid=<author>Li+S%3BHou+Y%3BChen+Q%3BZhang+X%3BCao+H%3BHuang+Y<%2Fauthor><AN>31854974<%2FAN><DT>Journal+Article<%2FDT>)

79.

Metabolic profile of human parathyroid adenoma.

di Masi A, Leboffe L, Sodo A, Tabacco G, Cesareo R, Sbroscia M, Giovannoni I, Taffon C, Crucitti P, Longo F, Manfrini S, Ricci MA, Ascenzi P, Crescenzi A, Palermo A

Endocrine. 67(3):699-707, 2020 03.

[Journal Article. Research Support, Non-U.S. Gov't]

UI: 31786773

PURPOSE: Recently, it has been demonstrated that Raman spectroscopy is able to differentiate between healthy parathyroid tissues and parathyroid adenoma based on the basis of a specific molecular fingerprint. However, to our knowledge, no previous studies have been performed to evaluate the metabolic profile of parathyroid adenoma. Therefore, we designed a proof of concept study aimed to investigate the glucose/fatty acid metabolisms, in addition to the mitochondrial changes, in solitary parathyroid adenoma and in healthy parathyroid glands.

METHODS: Nine females with primary hyperparathyroidism due to a solitary parathyroid adenoma and formal surgical indication for parathyroidectomy have been enrolled. At the time of surgery, the removed specimens were immediately submitted unfixed and a tissue slice of about 0.5 cm in diameter was obtained from the nodular lesion. The expression of selected metabolic enzymes and proteins has been evaluated by western blot analysis, using human parathyroid whole tissue lysates as control.

RESULTS: Data obtained highlighted an increase, compared with the healthy group, of: (i) the glucose uptake by the GLUT-1 receptor and its phosphorylation by hexokinase II (HXKII); (ii) the expression of 3-phosphoglycerate dehydrogenase (3-PGDH) and glucose-6-phosphate dehydrogenase (G6PD); (iii) lipids biosynthesis; and (iv) cytochrome c expression.

CONCLUSIONS: Our findings highlight for the first time the parathyroid adenoma metabolic hallmarks that could represent potential molecular targets usable for the development of new pharmacological treatments, allowing to reduce surgical parathyroidectomy.

Version ID

1

Record Owner

From MEDLINE, a database of the U.S. National Library of Medicine.

Status

MEDLINE

Authors Full Name

di Masi, Alessandra, Leboffe, Loris, Sodo, Armida, Tabacco, Gaia, Cesareo, Roberto, Sbroscia, Marco, Giovannoni, Isabella, Taffon, Chiara, Crucitti, Pierfilippo, Longo, Filippo, Manfrini, Silvia, Ricci, Maria Antonietta, Ascenzi, Paolo, Crescenzi, Anna, Palermo, Andrea

Institution

di Masi, Alessandra. Department of Sciences, Roma Tre University, I-00146, Roma, Italy. alessandra.dimasi@uniroma3.it. Leboffe, Loris. Department of Sciences, Roma Tre University, I-00146, Roma, Italy.

Sodo, Armida. Department of Sciences, Roma Tre University, I-00146, Roma, Italy.

Tabacco, Gaia. Unit of Endocrinology and Diabetes, Campus Bio-Medico University, Roma, Italy.

Cesareo, Roberto. Unit of Metabolic Diseases, Department of Internal Medicine, Santa Maria Goretti Hospital, Latina, Italy.

Sbroscia, Marco. Department of Sciences, Roma Tre University, I-00146, Roma, Italy.

Giovannoni, Isabella. Pathology Unit, Campus Bio-Medico University Hospital, Roma, Italy.

Taffon, Chiara. Pathology Unit, Campus Bio-Medico University Hospital, Roma, Italy.

Crucitti, Pierfilippo. Unit of Neck and Chest Surgery, Campus Bio-Medico University, Roma, Italy.

Longo, Filippo. Unit of Neck and Chest Surgery, Campus Bio-Medico University, Roma, Italy.

Manfrini, Silvia. Unit of Endocrinology and Diabetes, Campus Bio-Medico University, Roma, Italy.

Ricci, Maria Antonietta. Department of Sciences, Roma Tre University, I-00146, Roma, Italy.

Ascenzi, Paolo. Department of Sciences, Roma Tre University, I-00146, Roma, Italy.

Crescenzi, Anna. Pathology Unit, Campus Bio-Medico University Hospital, Roma, Italy.

Palermo, Andrea. Unit of Endocrinology and Diabetes, Campus Bio-Medico University, Roma, Italy.

MeSH Heading

Adenoma/su [Surgery]. *Adenoma. Female. Humans. Metabolome. Parathyroid Glands. Parathyroid Hormone. Parathyroid Neoplasms/su [Surgery]. *Parathyroid Neoplasms. Parathyroidectomy.

Keyword Heading

Cytochrome c Fatty acid

Glucose

Glycolysis

Metabolism

Parathyroid adenoma.

Keyword Heading Owner

NOTNLM

Registry Number/Name of Substance

0 (Parathyroid Hormone).

Year of Publication

2020

Link to the Ovid Full Text or citation:

[Click here for full text options](https://ovidsp.ovid.com/ovidweb.cgi?T=JS&CSC=Y&NEWS=N&PAGE=fulltext&D=med17&AN=31786773)

Link to the External Link Resolver:

[SFX](https://sfx-86scu.hosted.exlibrisgroup.com.cn/86scu?sid=OVID:medline&id=pmid:31786773&id=doi:10.1007%2Fs12020-019-02146-x&issn=1355008X&isbn=&volume=67&issue=3&spage=699&pages=699-707&date=2020&title=Endocrine&atitle=Metabolic+profile+of+human+parathyroid+adenoma.&aulast=di+Masi&pid=<author>di+Masi+A%3BLeboffe+L%3BSodo+A%3BTabacco+G%3BCesareo+R%3BSbroscia+M%3BGiovannoni+I%3BTaffon+C%3BCrucitti+P%3BLongo+F%3BManfrini+S%3BRicci+MA%3BAscenzi+P%3BCrescenzi+A%3BPalermo+A<%2Fauthor><AN>31786773<%2FAN><DT>Journal+Article<%2FDT>)

80.

Study on the biochemical mechanisms of the micro-wave ablation treatment of lung cancer by ex vivo confocal Raman microspectral imaging.

Song D, Chen T, Wang S, Chen S, Li H, Yu F, Zhang J, Zhang Z

Analyst. 145(2):626-635, 2020 Jan 20.

[Journal Article]

UI: 31782420

As a highly invasive and the most prevalent malignancy, lung cancer remains the leading cause of cancer-associated mortality worldwide, especially in China. Microwave ablation (MWA) is an effective, safe, and the least invasive ablative treatment modality, which has been increasingly used for the management of unrespectable lung tumors. However, the underlying biochemical mechanisms of MWA treatment remain to be incompletely elucidated. Therefore, to illustrate the complex biochemical responses of lung squamous cell carcinoma (LSCC) to MWA treatment, confocal Raman micro-spectral imaging (CRMI) was applied in combination with multivariate analysis. A total of twelve LSCC tissues were acquired from patients undergoing clinical treatment, and their spectral characteristics were analyzed to determine significant spectral variations following cancer progression and MWA treatment in comparison with healthy lung tissues. Point-scanned Raman datasets were acquired from sectioned tissue samples in both pre-therapy (Pre-MWA group) and post-therapy groups (Post-MWA group) and further analyzed using K-means cluster analysis (KCA) and principal component analysis (PCA) to highlight the detailed compositional variations of the biochemical constituents. The spectral variations of essential amino acids (such as phenylalanine and tryptophan), collagen, and nucleic acids in the cancerous tissues of the Post-MWA group were significantly enhanced compared to those in the Pre-MWA group. The acquired information further confirmed a remarkable increase in the content of nucleic acid, protein, and lipid in the cancerous tissue following MWA treatment and, a comparative spectral imaging investigation indicated that MWA had no noticeable adverse effects on the paracancerous tissues. Thus, the findings not only illustrated the underlying biochemical variability in lung cancer during MWA treatment but also further confirmed the feasibility of a combined analytical procedure for assessing the biochemical responses during thermal ablation, which could be applied to prominently enhance the effectiveness of MWA in lung cancer treatment in clinical settings.

Version ID

1

Record Owner

From MEDLINE, a database of the U.S. National Library of Medicine.

Status

MEDLINE

Authors Full Name

Song, Dongliang, Chen, Tianming, Wang, Shuang, Chen, Shilin, Li, Heping, Yu, Fan, Zhang, Jingyuan, Zhang, Zhe

Institution

Song, Dongliang . Institute of Photonics and Photon-Technology, Northwest University, Xi'an, Shaanxi 710069, China. swang@nwu.edu.cn.

MeSH Heading

*Carcinoma, Non-Small-Cell Lung/pa [Pathology]. Carcinoma, Non-Small-Cell Lung/th [Therapy]. Case-Control Studies. *Catheter Ablation/mt [Methods]. Cluster Analysis. Humans. *Lung Neoplasms/pa [Pathology]. Lung Neoplasms/th [Therapy]. Male. *Microwaves/tu [Therapeutic Use]. *Molecular Imaging/mt [Methods]. Principal Component Analysis. *Spectrum Analysis, Raman/mt [Methods].

Year of Publication

2020

Link to the Ovid Full Text or citation:

[Click here for full text options](https://ovidsp.ovid.com/ovidweb.cgi?T=JS&CSC=Y&NEWS=N&PAGE=fulltext&D=med17&AN=31782420)

Link to the External Link Resolver:

[SFX](https://sfx-86scu.hosted.exlibrisgroup.com.cn/86scu?sid=OVID:medline&id=pmid:31782420&id=doi:10.1039%2Fc9an01524h&issn=00032654&isbn=&volume=145&issue=2&spage=626&pages=626-635&date=2020&title=Analyst&atitle=Study+on+the+biochemical+mechanisms+of+the+micro-wave+ablation+treatment+of+lung+cancer+by+ex+vivo+confocal+Raman+microspectral+imaging.&aulast=Song&pid=<author>Song+D%3BChen+T%3BWang+S%3BChen+S%3BLi+H%3BYu+F%3BZhang+J%3BZhang+Z<%2Fauthor><AN>31782420<%2FAN><DT>Journal+Article<%2FDT>)

81.

Label-Free Raman Spectroscopy Reveals Signatures of Radiation Resistance in the Tumor Microenvironment.

Paidi SK, Diaz PM, Dadgar S, Jenkins SV, Quick CM, Griffin RJ, Dings RPM, Rajaram N, Barman I

Cancer Research. 79(8):2054-2064, 2019 04 15.

[Journal Article. Research Support, N.I.H., Extramural. Research Support, Non-U.S. Gov't]

UI: 30819665

Delay in the assessment of tumor response to radiotherapy continues to pose a major challenge to quality of life for patients with nonresponsive tumors. Here, we exploited label-free Raman spectroscopic mapping to elucidate radiation-induced biomolecular changes in tumors and uncovered latent microenvironmental differences between treatment-resistant and -sensitive tumors. We used isogenic radiation-resistant and -sensitive A549 human lung cancer cells and human head and neck squamous cell carcinoma (HNSCC) cell lines (UM-SCC-47 and UM-SCC-22B, respectively) to grow tumor xenografts in athymic nude mice and demonstrated the molecular specificity and quantitative nature of Raman spectroscopic tissue assessments. Raman spectra obtained from untreated and treated tumors were subjected to chemometric analysis using multivariate curve resolution-alternating least squares (MCR-ALS) and support vector machine (SVM) to quantify biomolecular differences in the tumor microenvironment. The Raman measurements revealed significant and reliable differences in lipid and collagen content postradiation in the tumor microenvironment, with consistently greater changes observed in the radiation-sensitive tumors. In addition to accurately evaluating tumor response to therapy, the combination of Raman spectral markers potentially offers a route to predicting response in untreated tumors prior to commencing treatment. Combined with its noninvasive nature, our findings provide a rationale for in vivo studies using Raman spectroscopy, with the ultimate goal of clinical translation for patient stratification and guiding adaptation of radiotherapy during the course of treatment. SIGNIFICANCE: These findings highlight the sensitivity of label-free Raman spectroscopy to changes induced by radiotherapy and indicate the potential to predict radiation resistance prior to commencing therapy.

Copyright ©2019 American Association for Cancer Research.

Version ID

1

Record Owner

From MEDLINE, a database of the U.S. National Library of Medicine.

Status

MEDLINE

Author NameID

Paidi, Santosh K; ORCID: <https://orcid.org/0000-0002-7034-586X>

Authors Full Name

Paidi, Santosh K, Diaz, Paola Monterroso, Dadgar, Sina, Jenkins, Samir V, Quick, Charles M, Griffin, Robert J, Dings, Ruud P M, Rajaram, Narasimhan, Barman, Ishan

Institution

Paidi, Santosh K. Department of Mechanical Engineering, Johns Hopkins University, Baltimore, Maryland. Diaz, Paola Monterroso. Department of Biomedical Engineering, University of Arkansas, Fayetteville, Arkansas.

Dadgar, Sina. Department of Biomedical Engineering, University of Arkansas, Fayetteville, Arkansas.

Jenkins, Samir V. Division of Radiation Oncology, University of Arkansas for Medical Sciences, Little Rock, Arkansas.

Quick, Charles M. Division of Pathology, University of Arkansas for Medical Sciences, Little Rock, Arkansas.

Griffin, Robert J. Division of Radiation Oncology, University of Arkansas for Medical Sciences, Little Rock, Arkansas.

Dings, Ruud P M. Division of Radiation Oncology, University of Arkansas for Medical Sciences, Little Rock, Arkansas.

Rajaram, Narasimhan. Department of Biomedical Engineering, University of Arkansas, Fayetteville, Arkansas. ibarman@jhu.edu nrajaram@uark.edu.

Barman, Ishan. Department of Mechanical Engineering, Johns Hopkins University, Baltimore, Maryland. ibarman@jhu.edu nrajaram@uark.edu.

Barman, Ishan. Department of Oncology, The Johns Hopkins University School of Medicine, Baltimore, Maryland.

Barman, Ishan. The Russell H. Morgan Department of Radiology and Radiological Science, The Johns Hopkins University School of Medicine, Baltimore, Maryland.

MeSH Heading

Animals. *Carcinoma, Squamous Cell/pa [Pathology]. Carcinoma, Squamous Cell/rt [Radiotherapy]. *Head and Neck Neoplasms/pa [Pathology]. Head and Neck Neoplasms/rt [Radiotherapy]. Humans. *Lung Neoplasms/pa [Pathology]. Lung Neoplasms/rt [Radiotherapy]. Mice. Mice, Nude. *Radiation Tolerance. *Spectrum Analysis, Raman/mt [Methods]. Tumor Cells, Cultured. *Tumor Microenvironment/re [Radiation Effects]. Xenograft Model Antitumor Assays.

Year of Publication

2019

Link to the Ovid Full Text or citation:

[Click here for full text options](https://ovidsp.ovid.com/ovidweb.cgi?T=JS&CSC=Y&NEWS=N&PAGE=fulltext&D=med16&AN=30819665)

Link to the External Link Resolver:

[SFX](https://sfx-86scu.hosted.exlibrisgroup.com.cn/86scu?sid=OVID:medline&id=pmid:30819665&id=doi:10.1158%2F0008-5472.CAN-18-2732&issn=00085472&isbn=&volume=79&issue=8&spage=2054&pages=2054-2064&date=2019&title=Cancer+Research&atitle=Label-Free+Raman+Spectroscopy+Reveals+Signatures+of+Radiation+Resistance+in+the+Tumor+Microenvironment.&aulast=Paidi&pid=<author>Paidi+SK%3BDiaz+PM%3BDadgar+S%3BJenkins+SV%3BQuick+CM%3BGriffin+RJ%3BDings+RPM%3BRajaram+N%3BBarman+I<%2Fauthor><AN>30819665<%2FAN><DT>Journal+Article<%2FDT>)

82.

Human blood test based on surface-enhanced Raman spectroscopy technology using different excitation light for nasopharyngeal cancer detection.

Lin H, Zhou J, Wu Q, Hung TM, Chen W, Yu Y, Chang JT, Pan J, Qiu S, Chen R

IET Nanobiotechnology IET. 13(9):942-945, 2019 Dec.

[Journal Article]

UI: 31811763

Nasopharyngeal carcinoma (NPC), a kind of squamous cell carcinoma, occurs in the top and the side wall of nasopharyngeal, which harms human health and life. In this study, a novel blood test (SERS) was carried out for 30 NPC patients and 30 normal ones. Using multi-variate statistical analysis for spectral data, the diagnostic sensitivities of 89.3% (50/56) and 85.7% (48/56) can be achieved for 633 and 785 nm exciting wavelength, respectively. Also corresponding specificities are 71.4% (41/56) and 78.6% (44/56), respectively. These results demonstrated that the two kinds of excitation wavelength all have the feasibility of obtaining high-quality SERS spectra to differentiate cancer from normal samples. Furthermore, the performance of the SERS test with 785 nm wavelength excitation is nearly equal to the SERS experimental effect under 633 nm wavelength excitation for NPC detection.

Version ID

1

Record Owner

From MEDLINE, a database of the U.S. National Library of Medicine.

Status

MEDLINE

Authors Full Name

Lin, Huijing, Zhou, Jiahui, Wu, Qiong, Hung, Tsung-Min, Chen, Weiwei, Yu, Yun, Chang, Joseph Tung-Chieh, Pan, Jianji, Qiu, Sufang, Chen, Rong

Institution

Lin, Huijing. Key Laboratory of OptoElectronic Science and Technology for Medicine, Ministry of Education, Fujian Provincial Key Laboratory for Photonics Technology, Fujian Normal University, Fuzhou, Fujian, People's Republic of China. Zhou, Jiahui. College of Integrated Traditional Chinese and Western Medicine, Fujian University of Traditional Chinese Medicine, Fuzhou, Fujian, People's Republic of China.

Wu, Qiong. Key Laboratory of OptoElectronic Science and Technology for Medicine, Ministry of Education, Fujian Provincial Key Laboratory for Photonics Technology, Fujian Normal University, Fuzhou, Fujian, People's Republic of China.

Hung, Tsung-Min. Department of Radiation Oncology, Chang Gung Memorial Hospital, Chang Gung University, Taoyuan, Taiwan.

Chen, Weiwei. College of Integrated Traditional Chinese and Western Medicine, Fujian University of Traditional Chinese Medicine, Fuzhou, Fujian, People's Republic of China.

Yu, Yun. College of Integrated Traditional Chinese and Western Medicine, Fujian University of Traditional Chinese Medicine, Fuzhou, Fujian, People's Republic of China.

Chang, Joseph Tung-Chieh. Department of Radiation Oncology, Chang Gung Memorial Hospital, Chang Gung University, Taoyuan, Taiwan.

Pan, Jianji. Fujian Provincial Key Laboratory of Translational Cancer Medicine, Fuzhou, Fujian, People's Republic of China.

Qiu, Sufang. Fujian Provincial Key Laboratory of Translational Cancer Medicine, Fuzhou, Fujian, People's Republic of China. sfqiu@126.com.

Chen, Rong. Key Laboratory of OptoElectronic Science and Technology for Medicine, Ministry of Education, Fujian Provincial Key Laboratory for Photonics Technology, Fujian Normal University, Fuzhou, Fujian, People's Republic of China.

MeSH Heading

Adult. Case-Control Studies. Humans. Middle Aged. Multivariate Analysis. Nasopharyngeal Neoplasms/bl [Blood]. *Nasopharyngeal Neoplasms/di [Diagnosis]. Sensitivity and Specificity. *Spectrum Analysis, Raman/mt [Methods]. Squamous Cell Carcinoma of Head and Neck/bl [Blood]. *Squamous Cell Carcinoma of Head and Neck/di [Diagnosis].

Year of Publication

2019

Link to the Ovid Full Text or citation:

[Click here for full text options](https://ovidsp.ovid.com/ovidweb.cgi?T=JS&CSC=Y&NEWS=N&PAGE=fulltext&D=med16&AN=31811763)

Link to the External Link Resolver:

[SFX](https://sfx-86scu.hosted.exlibrisgroup.com.cn/86scu?sid=OVID:medline&id=pmid:31811763&id=doi:10.1049%2Fiet-nbt.2019.0221&issn=17518741&isbn=&volume=13&issue=9&spage=942&pages=942-945&date=2019&title=IET+Nanobiotechnology+IET&atitle=Human+blood+test+based+on+surface-enhanced+Raman+spectroscopy+technology+using+different+excitation+light+for+nasopharyngeal+cancer+detection.&aulast=Lin&pid=<author>Lin+H%3BZhou+J%3BWu+Q%3BHung+TM%3BChen+W%3BYu+Y%3BChang+JT%3BPan+J%3BQiu+S%3BChen+R<%2Fauthor><AN>31811763<%2FAN><DT>Journal+Article<%2FDT>)

83.

Image-guided surgery of head and neck carcinoma in rabbit models by intra-operatively defining tumour-infiltrated margins and metastatic lymph nodes.

Sun P, Zhang Y, Li K, Wang C, Zeng F, Zhu J, Wu Y, Tao X

EBioMedicine. 50:93-102, 2019 Dec.

[Journal Article]

UI: 31734170

BACKGROUND: The infiltrative nature and lymphatic metastasis of head and neck squamous cell carcinoma (HNSCC) are the main reasons leading to its poor prognosis.

METHODS: A multimodal surface-enhanced resonance Raman spectroscopy (SERRS) and magnetic resonance (MR) nanoprobe, in which paramagnetic chelators and heptamethine cyanine-based Raman reporter molecules were functionalized on a gold nanostar (AuS) surface was developed. Preoperative MRI and intraoperative SERRS-guided surgery were performed on rabbits bearing head and neck VX2 tumours to determine feasibility of the MR/SERRS probe in defining tumour marginal infiltration and lymph nodes metastasis.

FINDINGS: Preoperative T1-weighted MRI (T1W-MRI) unambiguously delineated the orthotopic head and neck VX2 tumour xenograft and detected the metastatic lymph nodes in rabbit models after intravenous administration of the probe. With the assistance of a hand-held Raman detector, the probe not only intra-operatively demarcated invasive tumour margins but also successfully distinguished metastatic lymph nodes via a remarkable attenuated Raman signal. Importantly, the group of rabbits subjected to the SERRS-guided surgery exhibited prolonged median survival time (78 days) compared with that of the control group without surgical intervention (29 days) or the group treated with conventional white-light-guided surgery (42 days) (P < 0.0001).

INTERPRETATION: we developed a novel AuS-based multimodal MR/SERRS probe. The capability of this probe to identify both a tumour xenograft and metastatic lymph nodes preoperatively by MRI and intra-operatively by SERRS not only avoids the need for unnecessary resection of neurological structures but also provides a new opportunity to improve the surgical prognosis of head and neck carcinoma of infiltrative nature.

Copyright © 2019 The Author(s). Published by Elsevier B.V. All rights reserved.

Version ID

1

Record Owner

From MEDLINE, a database of the U.S. National Library of Medicine.

Status

MEDLINE

Authors Full Name

Sun, Pengpeng, Zhang, Yunfei, Li, Kaicheng, Wang, Cong, Zeng, Feng, Zhu, Jinyu, Wu, Yingwei, Tao, Xiaofeng

Institution

Sun, Pengpeng. Department of Radiology, School of Medicine, Shanghai Ninth People's Hospital, Shanghai Jiao Tong University, 639 Zhizao Ju Road, Shanghai 200011, China. Zhang, Yunfei. School of Pharmacy, Key Laboratory of Smart Drug Delivery, Ministry of Education, Fudan University, Shanghai 201203, China; United Imaging Healthcare, Shanghai, 201807, China.

Li, Kaicheng. Department of Radiology, School of Medicine, Shanghai Ninth People's Hospital, Shanghai Jiao Tong University, 639 Zhizao Ju Road, Shanghai 200011, China; Department of Radiology, Hainan West Central Hospital, Dan Zhou, Hai Nan, 571700, China.

Wang, Cong. School of Pharmacy, Key Laboratory of Smart Drug Delivery, Ministry of Education, Fudan University, Shanghai 201203, China.

Zeng, Feng. School of Pharmacy, Key Laboratory of Smart Drug Delivery, Ministry of Education, Fudan University, Shanghai 201203, China; Pi-Wei Institute, Guangzhou University of Chinese Medicine, Guangzhou, 510405, China.

Zhu, Jinyu. Department of Radiology, School of Medicine, Shanghai Ninth People's Hospital, Shanghai Jiao Tong University, 639 Zhizao Ju Road, Shanghai 200011, China; Department of Radiology, Shanghai Six People's Hospital, Shanghai Jiao Tong University, Shanghai 200233, China.

Wu, Yingwei. Department of Radiology, School of Medicine, Shanghai Ninth People's Hospital, Shanghai Jiao Tong University, 639 Zhizao Ju Road, Shanghai 200011, China. Electronic address: wuyw0103@hotmail.com.

Tao, Xiaofeng. Department of Radiology, School of Medicine, Shanghai Ninth People's Hospital, Shanghai Jiao Tong University, 639 Zhizao Ju Road, Shanghai 200011, China. Electronic address: cjr.taoxiaofeng@vip.163.com.

MeSH Heading

Animals. *Carcinoma/di [Diagnosis]. Carcinoma/mo [Mortality]. *Carcinoma/su [Surgery]. Cell Line, Tumor. Disease Models, Animal. *Head and Neck Neoplasms/di [Diagnosis]. Head and Neck Neoplasms/mo [Mortality]. *Head and Neck Neoplasms/su [Surgery]. Humans. Lymphatic Metastasis. Magnetic Resonance Imaging. Margins of Excision. Molecular Probes/cs [Chemical Synthesis]. Molecular Probes/ch [Chemistry]. Neoplasm Invasiveness. Neoplasm Staging. Rabbits. Spectrum Analysis, Raman. Surgery, Computer-Assisted/mt [Methods]. *Surgery, Computer-Assisted. Treatment Outcome.

Keyword Heading

Head and neck squamous cell carcinoma (HNSCC) Image-guided resection

Lymph node metastasis

Magnetic resonance imaging (MRI)

Surface-enhanced resonance Raman scattering (SERRS).

Keyword Heading Owner

NOTNLM

Registry Number/Name of Substance

0 (Molecular Probes).

Year of Publication

2019

Link to the Ovid Full Text or citation:

[Click here for full text options](https://ovidsp.ovid.com/ovidweb.cgi?T=JS&CSC=Y&NEWS=N&PAGE=fulltext&D=med16&AN=31734170)

Link to the External Link Resolver:

[SFX](https://sfx-86scu.hosted.exlibrisgroup.com.cn/86scu?sid=OVID:medline&id=pmid:31734170&id=doi:10.1016%2Fj.ebiom.2019.10.055&issn=23523964&isbn=&volume=50&issue=&spage=93&pages=93-102&date=2019&title=EBioMedicine&atitle=Image-guided+surgery+of+head+and+neck+carcinoma+in+rabbit+models+by+intra-operatively+defining+tumour-infiltrated+margins+and+metastatic+lymph+nodes.&aulast=Sun&pid=<author>Sun+P%3BZhang+Y%3BLi+K%3BWang+C%3BZeng+F%3BZhu+J%3BWu+Y%3BTao+X<%2Fauthor><AN>31734170<%2FAN><DT>Journal+Article<%2FDT>)

84.

Label-free liquid biopsy based on blood circulating DNA detection using SERS-based nanotechnology for nasopharyngeal cancer screening.

Lin D, Wu Q, Qiu S, Chen G, Feng S, Chen R, Zeng H

Nanomedicine. 22:102100, 2019 11.

[Journal Article. Research Support, Non-U.S. Gov't]

UI: 31648038

Development of a sensitive, rapid and easy-to-use liquid biopsy method is of imperative clinical value for point-of-care caner diagnostics. Here, a label-free and modification-free nanotechnology based on surface-enhanced Raman spectroscopy (SERS) was employed for DNA analysis. Using the SERS signals of phosphate backbone as internal standard, quantitative detection for nucleobases was achieved even at single base level. The method combined with principal component analysis and linear discriminant analysis was further applied for real blood circulating DNA detection for the first time, and an ideal diagnostic sensitivity of 83.3% and specificity of 82.5% could be obtained for differentiating the nasopharyngeal cancer from the normal group, demonstrating promising potential as an alternative nanotechnology for nasopharyngeal cancer screening based on liquid biopsy.

Copyright © 2019 Elsevier Inc. All rights reserved.

Version ID

1

Record Owner

From MEDLINE, a database of the U.S. National Library of Medicine.

Status

MEDLINE

Authors Full Name

Lin, Duo, Wu, Qiong, Qiu, Sufang, Chen, Guannan, Feng, Shangyuan, Chen, Rong, Zeng, Haishan

Institution

Lin, Duo. College of Integrated Traditional Chinese and Western Medicine, Fujian University of Traditional Chinese Medicine, Fuzhou, Fujian, 350122, China. Electronic address: linduo1986@163.com. Wu, Qiong. Key Laboratory of OptoElectronic Science and Technology for Medicine, Ministry of Education, Fujian Provincial Key Laboratory for Photonics Technology, Digital Fujian Internet-of-Things Laboratory of Environment Monitoring, Fujian Normal University, Fuzhou 350007, China.

Qiu, Sufang. Fujian Medical University Cancer Hospital & Fujian Cancer Hospital Radiation Oncology Department; Fujian Provincial Key Laboratory of Translational Cancer Medicine, Fuzhou, 350014, China.

Chen, Guannan. Key Laboratory of OptoElectronic Science and Technology for Medicine, Ministry of Education, Fujian Provincial Key Laboratory for Photonics Technology, Digital Fujian Internet-of-Things Laboratory of Environment Monitoring, Fujian Normal University, Fuzhou 350007, China.

Feng, Shangyuan. Key Laboratory of OptoElectronic Science and Technology for Medicine, Ministry of Education, Fujian Provincial Key Laboratory for Photonics Technology, Digital Fujian Internet-of-Things Laboratory of Environment Monitoring, Fujian Normal University, Fuzhou 350007, China. Electronic address: syfeng@fjnu.edu.cn.

Chen, Rong. Key Laboratory of OptoElectronic Science and Technology for Medicine, Ministry of Education, Fujian Provincial Key Laboratory for Photonics Technology, Digital Fujian Internet-of-Things Laboratory of Environment Monitoring, Fujian Normal University, Fuzhou 350007, China.

Zeng, Haishan. Imaging Unit - Integrative Oncology Department, BC Cancer Agency Research Centre, Vancouver, BC, V5Z 1L3, Canada. Electronic address: hzeng@bccrc.ca.

MeSH Heading

*Cell-Free Nucleic Acids/bl [Blood]. Discriminant Analysis. *Early Detection of Cancer. Humans. Liquid Biopsy. *Nanotechnology. *Nasopharyngeal Neoplasms/bl [Blood]. *Nasopharyngeal Neoplasms/pa [Pathology]. Principal Component Analysis. *Spectrum Analysis, Raman. *Staining and Labeling.

Keyword Heading

DNA Liquid biopsy

Nasopharyngeal cancer

SERS.

Keyword Heading Owner

NOTNLM

Registry Number/Name of Substance

0 (Cell-Free Nucleic Acids).

Year of Publication

2019

Link to the Ovid Full Text or citation:

[Click here for full text options](https://ovidsp.ovid.com/ovidweb.cgi?T=JS&CSC=Y&NEWS=N&PAGE=fulltext&D=med16&AN=31648038)

Link to the External Link Resolver:

[SFX](https://sfx-86scu.hosted.exlibrisgroup.com.cn/86scu?sid=OVID:medline&id=pmid:31648038&id=doi:10.1016%2Fj.nano.2019.102100&issn=15499634&isbn=&volume=22&issue=&spage=102100&pages=102100&date=2019&title=Nanomedicine&atitle=Label-free+liquid+biopsy+based+on+blood+circulating+DNA+detection+using+SERS-based+nanotechnology+for+nasopharyngeal+cancer+screening.&aulast=Lin&pid=<author>Lin+D%3BWu+Q%3BQiu+S%3BChen+G%3BFeng+S%3BChen+R%3BZeng+H<%2Fauthor><AN>31648038<%2FAN><DT>Journal+Article<%2FDT>)

85.

SERS-based differential diagnosis between multiple solid malignancies: breast, colorectal, lung, ovarian and oral cancer.

Moisoiu V, Stefancu A, Gulei D, Boitor R, Magdo L, Raduly L, Pasca S, Kubelac P, Mehterov N, Chis V, Simon M, Muresan M, Irimie AI, Baciut M, Stiufiuc R, Pavel IE, Achimas-Cadariu P, Ionescu C, Lazar V, Sarafian V, Notingher I, Leopold N, Berindan-Neagoe I

International Journal of Nanomedicine. 14:6165-6178, 2019.

[Journal Article]

UI: 31447558

PURPOSE: Surface-enhanced Raman scattering (SERS) spectroscopy on serum and other biofluids for cancer diagnosis represents an emerging field, which has shown promising preliminary results in several types of malignancies. The purpose of this study was to demonstrate that SERS spectroscopy on serum can be employed for the differential diagnosis between five of the leading malignancies, ie, breast, colorectal, lung, ovarian and oral cancer.

PATIENTS AND METHODS: Serum samples were acquired from healthy volunteers (n=39) and from patients diagnosed with breast (n=42), colorectal (n=109), lung (n=33), oral (n=17), and ovarian cancer (n=13), comprising n=253 samples in total. SERS spectra were acquired using a 532 nm laser line as excitation source, while the SERS substrates were represented by Ag nanoparticles synthesized by reduction with hydroxylamine. The classification accuracy yielded by SERS was assessed by principal component analysis-linear discriminant analysis (PCA-LDA).

RESULTS: The sensitivity and specificity in discriminating between cancer patients and controls was 98% and 91%, respectively. Cancer samples were correctly assigned to their corresponding cancer types with an accuracy of 88% for oral cancer, 86% for colorectal cancer, 80% for ovarian cancer, 76% for breast cancer and 59% for lung cancer.

CONCLUSION: SERS on serum represents a promising strategy of diagnosing cancer which can discriminate between cancer patients and controls, as well as between cancer types such as breast, colorectal, lung ovarian and oral cancer.

Version ID

1

Record Owner

From MEDLINE, a database of the U.S. National Library of Medicine.

Status

MEDLINE

Authors Full Name

Moisoiu, Vlad, Stefancu, Andrei, Gulei, Diana, Boitor, Radu, Magdo, Lorand, Raduly, Lajos, Pasca, Sergiu, Kubelac, Paul, Mehterov, Nikolay, Chis, Vasile, Simon, Marioara, Muresan, Mihai, Irimie, Alexandra Iulia, Baciut, Mihaela, Stiufiuc, Rares, Pavel, Ioana E, Achimas-Cadariu, Patriciu, Ionescu, Calin, Lazar, Vladimir, Sarafian, Victoria, Notingher, Ioan, Leopold, Nicolae, Berindan-Neagoe, Ioana

Institution

Moisoiu, Vlad. Faculty of Physics, Babes-Bolyai University, Cluj-Napoca, Romania. Moisoiu, Vlad. Faculty of Medicine, Iuliu Hatieganu University of Medicine and Pharmacy, Cluj-Napoca, Romania.

Stefancu, Andrei. Faculty of Physics, Babes-Bolyai University, Cluj-Napoca, Romania.

Stefancu, Andrei. MedFuture - Research Center for Advanced Medicine, Iuliu Hatieganu University of Medicine and Pharmacy, Cluj-Napoca, Romania.

Gulei, Diana. MedFuture - Research Center for Advanced Medicine, Iuliu Hatieganu University of Medicine and Pharmacy, Cluj-Napoca, Romania.

Boitor, Radu. School of Physics and Astronomy, University of Nottingham, Nottingham, UK.

Magdo, Lorand. Faculty of Medicine, Iuliu Hatieganu University of Medicine and Pharmacy, Cluj-Napoca, Romania.

Magdo, Lorand. Research Center for Functional Genomics, Biomedicine and Translational Medicine, Iuliu Hatieganu University of Medicine and Pharmacy, Cluj-Napoca, Romania.

Raduly, Lajos. Research Center for Functional Genomics, Biomedicine and Translational Medicine, Iuliu Hatieganu University of Medicine and Pharmacy, Cluj-Napoca, Romania.

Raduly, Lajos. Department of Pathophysiology, University of Agricultural Sciences and Veterinary Medicine, Cluj-Napoca, Romania.

Pasca, Sergiu. Faculty of Medicine, Iuliu Hatieganu University of Medicine and Pharmacy, Cluj-Napoca, Romania.

Kubelac, Paul. Faculty of Medicine, Iuliu Hatieganu University of Medicine and Pharmacy, Cluj-Napoca, Romania.

Kubelac, Paul. Department of Medical Oncology, Prof. Dr. Ion Chiricuta Clinical Cancer Center, Cluj-Napoca, Romania.

Mehterov, Nikolay. Department of Medical Biology, Faculty of Medicine, Medical University-Plovdiv, Plovdiv, Bulgaria.

Mehterov, Nikolay. Technological Center for Emergency Medicine, Plovdiv, Bulgaria.

Chis, Vasile. Faculty of Physics, Babes-Bolyai University, Cluj-Napoca, Romania.

Simon, Marioara. Department of Bronchology, Leon Daniello Pneumophysiology Clinical Hospital, Cluj-Napoca, Romania.

Muresan, Mihai. Faculty of Medicine, Iuliu Hatieganu University of Medicine and Pharmacy, Cluj-Napoca, Romania.

Muresan, Mihai. 5th Surgical Department, Cluj-Napoca Municipal Hospital, Cluj-Napoca, Romania.

Muresan, Mihai. Department of Surgical and Gynecological Oncology, Prof. Dr. Ion Chiricuta Clinical Cancer Center, Cluj-Napoca, Romania.

Irimie, Alexandra Iulia. Department of Prosthetic Dentistry and Dental Materials, Division Dental Propaedeutics, Aesthetics, Faculty of Dentistry, Iuliu Hatieganu University of Medicine and Pharmacy, Cluj-Napoca, Romania.

Baciut, Mihaela. Department of Cranio-Maxillofacial Surgery and Dental Emergencies, Iuliu Hatieganu University of Medicine and Pharmacy, Cluj-Napoca, Romania.

Stiufiuc, Rares. MedFuture - Research Center for Advanced Medicine, Iuliu Hatieganu University of Medicine and Pharmacy, Cluj-Napoca, Romania.

Stiufiuc, Rares. Department of Pharmaceutical Physics-Biophysics, Faculty of Pharmacy, Iuliu Hatieganu University of Medicine and Pharmacy, Cluj-Napoca, Romania.

Pavel, Ioana E. MedFuture - Research Center for Advanced Medicine, Iuliu Hatieganu University of Medicine and Pharmacy, Cluj-Napoca, Romania.

Pavel, Ioana E. Department of Chemistry, Wright State University, Dayton, OH, USA.

Achimas-Cadariu, Patriciu. Department of Surgery, Iuliu Hatieganu University of Medicine and Pharmacy, Cluj-Napoca, Romania.

Achimas-Cadariu, Patriciu. Department of Surgical Oncology, Prof. Dr. Ion Chiricuta Clinical Cancer Center, Cluj-Napoca, Romania.

Ionescu, Calin. Faculty of Medicine, Iuliu Hatieganu University of Medicine and Pharmacy, Cluj-Napoca, Romania.

Ionescu, Calin. 5th Surgical Department, Cluj-Napoca Municipal Hospital, Cluj-Napoca, Romania.

Lazar, Vladimir. Worldwide Innovative Network for Personalized Cancer Therapy, Villejuif, France.

Sarafian, Victoria. Department of Medical Biology, Faculty of Medicine, Medical University-Plovdiv, Plovdiv, Bulgaria.

Sarafian, Victoria. Technological Center for Emergency Medicine, Plovdiv, Bulgaria.

Notingher, Ioan. School of Physics and Astronomy, University of Nottingham, Nottingham, UK.

Leopold, Nicolae. Faculty of Physics, Babes-Bolyai University, Cluj-Napoca, Romania.

Leopold, Nicolae. MedFuture - Research Center for Advanced Medicine, Iuliu Hatieganu University of Medicine and Pharmacy, Cluj-Napoca, Romania.

Berindan-Neagoe, Ioana. MedFuture - Research Center for Advanced Medicine, Iuliu Hatieganu University of Medicine and Pharmacy, Cluj-Napoca, Romania.

Berindan-Neagoe, Ioana. Research Center for Functional Genomics, Biomedicine and Translational Medicine, Iuliu Hatieganu University of Medicine and Pharmacy, Cluj-Napoca, Romania.

Berindan-Neagoe, Ioana. Department of Functional Genomics and Experimental Pathology, Prof. Dr. Ion Chiricuta Clinical Cancer Center, Cluj-Napoca, Romania.

MeSH Heading

Aged. Breast Neoplasms/bl [Blood]. Breast Neoplasms/di [Diagnosis]. Case-Control Studies. Colorectal Neoplasms/bl [Blood]. Colorectal Neoplasms/di [Diagnosis]. Diagnosis, Differential. Discriminant Analysis. Female. Humans. Lung Neoplasms/bl [Blood]. Lung Neoplasms/di [Diagnosis]. Male. Metal Nanoparticles/ch [Chemistry]. Metal Nanoparticles/ul [Ultrastructure]. Middle Aged. Mouth Neoplasms/bl [Blood]. Mouth Neoplasms/di [Diagnosis]. Neoplasms/bl [Blood]. *Neoplasms/di [Diagnosis]. Ovarian Neoplasms/bl [Blood]. Ovarian Neoplasms/di [Diagnosis]. Principal Component Analysis. Silver/ch [Chemistry]. *Spectrum Analysis, Raman/mt [Methods].

Keyword Heading

SERS principal component analysis-linear discriminant analysis

serum

solid malignancies

surface-enhanced Raman scattering.

Keyword Heading Owner

NOTNLM

Registry Number/Name of Substance

3M4G523W1G (Silver).

Year of Publication

2019

Link to the Ovid Full Text or citation:

[Click here for full text options](https://ovidsp.ovid.com/ovidweb.cgi?T=JS&CSC=Y&NEWS=N&PAGE=fulltext&D=med16&AN=31447558)

Link to the External Link Resolver:

[SFX](https://sfx-86scu.hosted.exlibrisgroup.com.cn/86scu?sid=OVID:medline&id=pmid:31447558&id=doi:10.2147%2FIJN.S198684&issn=11769114&isbn=&volume=14&issue=&spage=6165&pages=6165-6178&date=2019&title=International+Journal+of+Nanomedicine&atitle=SERS-based+differential+diagnosis+between+multiple+solid+malignancies%3A+breast%2C+colorectal%2C+lung%2C+ovarian+and+oral+cancer.&aulast=Moisoiu&pid=<author>Moisoiu+V%3BStefancu+A%3BGulei+D%3BBoitor+R%3BMagdo+L%3BRaduly+L%3BPasca+S%3BKubelac+P%3BMehterov+N%3BChis+V%3BSimon+M%3BMuresan+M%3BIrimie+AI%3BBaciut+M%3BStiufiuc+R%3BPavel+IE%3BAchimas-Cadariu+P%3BIonescu+C%3BLazar+V%3BSarafian+V%3BNotingher+I%3BLeopold+N%3BBerindan-Neagoe+I<%2Fauthor><AN>31447558<%2FAN><DT>Journal+Article<%2FDT>)

86.

Dual-function nanostructured platform for isolation of nasopharyngeal carcinoma circulating tumor cells and EBV DNA detection.

Lee SW, Chen YW, Kuan EC, Lan MY

Biosensors & Bioelectronics. 142:111509, 2019 Oct 01.

[Journal Article]

UI: 31344600

Circulating tumor cells (CTCs) and plasma levels of Epstein-Barr virus (EBV) DNA are sensitive prognostic tools for monitoring disease status in nasopharyngeal carcinoma (NPC) patients. Herein, we introduce a novel and low-cost platform for capturing CTCs, the Si nanowires/microscale pyramids (NWs/MPs) hierarchical substrate, which could capture NPC cells in vitro and also detect EBV DNA at very low concentrations. In this study, Si NWs/MPs hierarchical substrates with varying wire length were fabricated using a metal-assisted chemical etching method. Anti-EpCAM antibodies were further conjugated on the substrate for capturing NPC CTCs in vitro. Capture efficiency was evaluated using immunofluorescence and scanning electronic microscopy (SEM) was utilized to understand cell morphology. The Si NWs/MPs substrate was also transformed into a Surface enhanced Raman scattering (SERS) substrate by coating with Ag nanoparticles (AgNPs) for detection of EBV DNA by Raman spectroscopy. The results demonstrated that Si NWs/MPs with 20min of etch time had the best capturing performance. Additionally, SEM observations revealed good contact of CTCs with Si NWs/MPs substrates. Moreover, the AgNPs-coated NWs/MPs substrate was shown to be a sensitive EBV DNA detector, by which the DNA detection limit can reach up to 10-13M. In conclusion, the Si NWs/MPs platform not only exhibits superior cell capturing ability, but also can sensitively detect EBV DNA at very low concentrations. This platform has great potential to become a promising diagnostic tool for monitoring disease status and prognostication of NPC patients.

Copyright © 2019 Elsevier B.V. All rights reserved.

Version ID

1

Record Owner

From MEDLINE, a database of the U.S. National Library of Medicine.

Status

MEDLINE

Authors Full Name

Lee, Sheng-Wei, Chen, Yi-Wei, Kuan, Edward C, Lan, Ming-Ying

Institution

Lee, Sheng-Wei. Institute of Materials Science and Engineering, National Central University, Taoyuan City 32001, Taiwan; Department of Materials Science and Engineering, University of California, Irvine, Orange, CA 92697, USA. Electronic address: swlee@g.ncu.edu.tw. Chen, Yi-Wei. Institute of Materials Science and Engineering, National Central University, Taoyuan City 32001, Taiwan.

Kuan, Edward C. Department of Otolaryngology-Head and Neck Surgery, University of California, Irvine, Orange, CA 92868, USA. Electronic address: eckuan@hs.uci.edu.

Lan, Ming-Ying. Division of Rhinology, Department of Otolaryngology Head and Neck Surgery, Taipei Veterans General Hospital, Taipei, 11217, Taiwan; School of Medicine, National Yang-Ming University, Taipei, 11221, Taiwan. Electronic address: mylan@vghtpe.gov.tw.

MeSH Heading

Antibodies, Immobilized/ch [Chemistry]. *Biosensing Techniques/mt [Methods]. Cell Line, Tumor. *DNA, Viral/an [Analysis]. Epithelial Cell Adhesion Molecule/an [Analysis]. Epstein-Barr Virus Infections/di [Diagnosis]. Epstein-Barr Virus Infections/vi [Virology]. *Herpesvirus 4, Human/ip [Isolation & Purification]. Humans. Nanostructures/ch [Chemistry]. Nanostructures/ul [Ultrastructure]. *Nasopharyngeal Carcinoma/pa [Pathology]. *Nasopharyngeal Neoplasms/pa [Pathology]. *Neoplastic Cells, Circulating/pa [Pathology]. Silicon/ch [Chemistry]. Spectrum Analysis, Raman/mt [Methods].

Keyword Heading

Circulating tumor cells EBV DNA

EpCAM

Nanowire

Nasopharyngeal carcinoma

Si.

Keyword Heading Owner

NOTNLM

Registry Number/Name of Substance

0 (Antibodies, Immobilized). 0 (DNA, Viral). 0 (Epithelial Cell Adhesion Molecule). Z4152N8IUI (Silicon).

Year of Publication

2019

Link to the Ovid Full Text or citation:

[Click here for full text options](https://ovidsp.ovid.com/ovidweb.cgi?T=JS&CSC=Y&NEWS=N&PAGE=fulltext&D=med16&AN=31344600)

Link to the External Link Resolver:

[SFX](https://sfx-86scu.hosted.exlibrisgroup.com.cn/86scu?sid=OVID:medline&id=pmid:31344600&id=doi:10.1016%2Fj.bios.2019.111509&issn=09565663&isbn=&volume=142&issue=&spage=111509&pages=111509&date=2019&title=Biosensors+%26+Bioelectronics&atitle=Dual-function+nanostructured+platform+for+isolation+of+nasopharyngeal+carcinoma+circulating+tumor+cells+and+EBV+DNA+detection.&aulast=Lee&pid=<author>Lee+SW%3BChen+YW%3BKuan+EC%3BLan+MY<%2Fauthor><AN>31344600<%2FAN><DT>Journal+Article<%2FDT>)

87.

Fiber-Optic Raman Spectroscopy with Nature-Inspired Genetic Algorithms Enhances Real-Time in Vivo Detection and Diagnosis of Nasopharyngeal Carcinoma.

Zuvela P, Lin K, Shu C, Zheng W, Lim CM, Huang Z

Analytical Chemistry. 91(13):8101-8108, 2019 07 02.

[Journal Article. Research Support, Non-U.S. Gov't]

UI: 31135136
[truncated: 659,358 more chars]
